# Efficacy and safety of Chinese herbal medicine for metabolic conditions: a systematic review and meta-analysis of randomised controlled trials

**DOI:** 10.3389/fphar.2025.1644950

**Published:** 2026-01-02

**Authors:** Yinglin Liang, Hongshen Yu, Jie Ren, Jiayi Ren, Guanlin Chen, Yikai Zhang, Yefeng Cai, Xiaojia Ni

**Affiliations:** 1 State Key Laboratory of Traditional Chinese Medicine Syndrome, The Second Affiliated Hospital of Guangzhou University of Chinese Medicine, Guangdong Provincial Hospital of Chinese Medicine, Guangdong Provincial Academy of Chinese Medical Sciences, Guangzhou, China; 2 The Second Clinical School of Guangzhou University of Chinese Medicine, Guangzhou, China

**Keywords:** Chinese herbal medicine, metabolic diseases, dampness, systematic review, meta-analysis

## Abstract

**Background and purpose:**

Observational studies indicate a high prevalence of dampness syndrome in metabolic diseases from the perspective of traditional Chinese medicine theory. This systematic review and meta-analysis aimed to assess the efficacy and safety of Chinese herbal medicine (CHM) for metabolic conditions via the therapeutic approach of eliminating dampness.

**Materials and methods:**

Six medical databases were searched up to August 2024 to identify randomised controlled trials (RCTs) involving individuals with type 2 diabetes mellitus (T2DM), hypertension, dyslipidaemia, or obesity, where the intervention included oral CHM targeting dampness. Risk of bias was assessed using the Cochrane Collaboration’s tool. Meta-analyses and forest plots were generated using Review Manager 5.3. Evidence quality was evaluated per the Grading of Recommendations Assessment, Development and Evaluation (GRADE).

**Results:**

Meta-analyses of 122 RCTs (n = 11,252 participants) showed that dampness-eliminating CHM, when combined with lifestyle interventions, improved fasting plasma glucose (FPG), diastolic blood pressure (DBP), and body mass index (BMI), but exerted limited impacts on 2-h postprandial glucose (2hPG) and systolic blood pressure (SBP). When used as an adjunct to pharmacotherapy, CHM significantly enhanced reductions in FPG, 2hPG, SBP, and DBP. The effects of CHM on lipid profiles were modest and uncertain. Although dampness-eliminating CHM as a whole conferred benefits for obesity, no outstanding formula with robust evidence was identified. Across all included RCTs, no additional adverse events were observed compared to pharmacotherapy alone. Promising CHM formulae included *Gegen Qinlian Decoction* for diabetes and *Banxia Baizhu Tianma Decoction* for hypertension. Poria, derived from the sclerotia of *Poria cocos (Schw.) Wolf.,* emerged as a key component across multiple conditions. Overall, while the meta-analysis suggested promising findings for dampness-eliminating CHM in modulating metabolic conditions, the certainty of evidence was limited due to heterogeneity and lack of blinding. Specifically, the quality of evidence for individual CHM formulae was unsatisfactory, as most studies were of small scale.

**Conclusion:**

Dampness-eliminating CHM may serve as a complementary therapy for metabolic diseases such as hypertension and diabetes. Further high-quality RCTs are required to confirm its role in dyslipidaemia and identify the most effective CHM formulae for obesity.

## 1 Introduction

Metabolic diseases, including type 2 diabetes mellitus (T2DM), hypertension, dyslipidaemia, and obesity, account for a major portion of non-communicable diseases and significantly contribute to global morbidity and mortality (Chew et al., 2023; World Health Organization, 2024). Recent data from the Global Burden of Diseases, Injuries, and Risk Factors Study estimate 43.8 million cases of T2DM and 18.5 million cases of hypertension worldwide (Chew et al., 2023). Notably, obesity was the leading cause of death in 2019, accounting for 5.0 million deaths, followed by hyperlipidaemia (4.3 million), T2DM (1.4 million), and hypertension (1.1 million) (Chew et al., 2023).

Current clinical practice guidelines universally recommend combination pharmacological therapy and lifestyle modifications for managing metabolic diseases (American Diabetes Association, 2017; American Diabetes Association, 2019; Carey et al., 2021; Gaskin et al., 2024; Hoover, 2019). Despite these standardised approaches, treatment efficacy frequently falls short of optimal targets (Ahmad et al., 2022; de la Sierra and Barrios, 2012; Kahn et al., 2006; Mancia et al., 2009). Pharmacological interventions for metabolic diseases are further complicated by considerable adverse effect profiles (Carey et al., 2022; Li S. et al., 2021; Ross, 2013; Tomaszewski et al., 2011; Ward et al., 2019). The growing need for polypharmacy in metabolic disease management further compounds adverse event potential through drug–drug interactions (Filippone et al., 2022). Patient adherence to long-term therapies also significantly affects treatment success. A previous study reported an average adherence rate of only 50% among patients with chronic diseases (De Geest and Sabaté, 2003).

Pattern Differentiation and Treatment is essential to the selection of Chinese herbal medicine (CHM) in the practice of traditional Chinese medicine (TCM). From the perspective of TCM theory, dampness is characterised by heaviness, turbidity, stickiness, stagnation, and descending nature. As a pathogenic factor, it impairs the production and transportation of body fluids, ultimately leading to the accumulation of pathological products (Wang et al., 2015). Dampness syndrome manifests as body heaviness, limb soreness, abdominal distention or diarrhoea, poor appetite and digestion, slippery tongue coating, and soggy pulse (Lu et al., 2024). From a contemporary perspective, dampness is associated with both microinflammation and dysregulation of lipid and glucose metabolism, which may increase individuals’ susceptibility to metabolic disorders (Chen et al., 2021; Li et al., 2025). Observational studies have revealed high prevalence of dampness syndrome in metabolic diseases (Lan et al., 2024; Liang et al., 2020; Ma et al., 2017; Wang et al., 2013; Zhou et al., 2024), and accumulating clinical trials have suggested benefits of Chinese herbal medicine (CHM) targeting dampness for these conditions (Chen et al., 2012; Gao et al., 2024; Tong et al., 2018; Zeng et al., 2006). For example, randomised controlled trials (RCTs) have demonstrated CHM’s effectiveness in lowering glycated haemoglobin, fasting plasma glucose (FPG), and postprandial blood glucose levels of patients with Type 2 diabetes mellitus (T2DM) (Gao et al., 2024) and in improving glycaemic control and reducing the body weight of patients with obesity (Chen et al., 2011; Ke et al., 2012).

Despite these promising preliminary findings, the evidence base remains limited by the relatively small number of high-quality RCTs and lack of comprehensive synthesis. To address this knowledge gap, we conducted a systematic review and meta-analysis to rigorously evaluate the efficacy and safety profile of CHM targeting dampness in the treatment of metabolic diseases. This study aimed to consolidate existing evidence and provide insights into potential therapeutic alternatives or adjuncts for these challenging conditions.

## 2 Materials and methods

### 2.1 Protocol registration and reporting standards

The protocol for this systematic review and meta-analysis was prospectively registered in the International Prospective Register of Systematic Reviews (PROSPERO No: CRD42024614968) (Ni et al., 2024). This study adheres to the Preferred Reporting Items for Systematic Reviews and Meta-Analyses statement and its extension for Chinese Herbal Medicines (Page et al., 2021; Zhang et al., 2020).

### 2.2 Eligibility criteria

Studies were eligible for inclusion if they met all of the following criteria:

• Study Designs: RCTs were the primary study design.
• Participants: Individuals diagnosed with T2DM, spontaneous hypertension, dyslipidaemia, or obesity. Diagnoses were established according to established clinical practice guidelines and international standards.
• Interventions: Oral CHM formulations targeting ‘dampness’ from the perspective of Chinese Materia Medica theory. Interventions were eligible if they met any of the following criteria:
   ○ Dampness was the primary Chinese medicine syndrome in study participants.
   ○ The therapeutic method specifically targeted dampness.
   ○ The prescription was recorded as a dampness-treating formula in textbooks.
   ○ The main herbs in the prescription were documented to treat dampness in the Pharmacopoeia of the People’s Republic of China.
• Comparisons: Control groups included no treatment, placebo, current pharmacological therapies recommended by clinical practice guidelines, and non-pharmacological therapies, such as lifestyle modifications.
• Primary Outcomes:
   ○ For T2DM: FPG and 2-h postprandial blood glucose (2hPG) levels.
   ○ For spontaneous hypertension: Systolic blood pressure (SBP) and diastolic blood pressure (DBP).
   ○ For dyslipidaemia: Triglyceride (TG) and low-density lipoprotein cholesterol (LDL-C) levels.
   ○ For obesity: Body mass index (BMI).
   ○ Adverse events.
• Secondary Outcomes:
   ○ For T2DM: Fasting insulin (FINS) level and homoeostasis model assessment of insulin resistance (HOMA-IR).
   ○ For hypertension: 24-h ambulatory blood pressure monitoring (24-h ABPM).
   ○ For dyslipidaemia: Total cholesterol (TC) and high-density lipoprotein cholesterol (HDL-C) levels.
   ○ For obesity: Waist circumference (WC), waist-to-hip ratio (WHR), and hip circumference (HC).
   ○ Vascular impairments and endpoint events, including mortality, cardiac infarction, stroke, and renal failure.

Studies were excluded if they focused on complications of metabolic diseases (e.g., diabetic nephropathy, hypertensive heart disease), used multiple CHM treatments together as interventions, were conference abstracts or duplicates, or contained serious methodological errors or flaws.

### 2.3 Information sources and search strategy

We systematically searched six medical databases to identify relevant literature, including China National Knowledge Infrastructure, China Biomedical database, Wanfang, PubMed, Embase, and Cochrane Library. Our search strategy incorporated both controlled vocabulary and keywords related to metabolic diseases and CHM interventions targeting dampness. To identify additional records, we also reviewed reference lists of included studies, searched for studies cited in systematic reviews of interest, and examined literature about proprietary Chinese medicines for T2DM, hypertension, dyslipidaemia, and obesity listed in the list of National Basic Medical Insurance Medicine, Employment Injury Insurance Medicine, Maternity Insurance Medicine, and Chinese Pharmacopoeia. The search was conducted from the inception of each database to August 2024. No language restrictions were applied. The detailed search strategy is provided in Supplementary Table S1.

### 2.4 Study selection and data extraction

The study was selected by removing duplicates using reference management software, screening titles/abstracts independently by two reviewers, assessing full texts for eligibility, resolving discrepancies via discussion with a senior reviewer, and documenting the process in a PRISMA flow diagram.

Data extraction was performed independently by two reviewers using a standardised electronic form designed in EpiData Software (version 3.1, EpiData Association, Odense, Denmark). A third reviewer conducted validation checks to ensure accuracy and completeness of the extracted data. Data items included bibliographic details, study designs, participant characteristics, Chinese medicine syndrome, therapeutic principle, and intervention details, such as CHM formula composition, dosage, administration route, treatment duration, and preparation method, the control, and outcomes. The therapeutic principles of TCM and the ingredients of proprietary Chinese medicines were verified through consultation of official records from the National Medical Products Administration and Chinese Pharmacopoeia. When necessary, additional clarification was sought directly from study authors or through consultation with TCM experts.

### 2.5 The assessment of risk-of-bias and evidence quality

Using the Cochrane Collaboration’s risk of bias tool for randomised trials (Higgins et al., 2011), we assessed potential bias across Seven domains, including random sequence generation, allocation concealment, blinding of participants and personnel, blinding of outcome assessment, incomplete outcome data, selective reporting, and baseline balance. For blinding assessments, we evaluated impact separately for three outcome types, including objective, clinician-reported, and patient-reported outcomes. Discrepancies between independent reviewers were resolved through structured discussion or consultation with a senior reviewer when consensus was not achieved. Each domain was categorised as low, high, or unclear risk of bias based on predefined criteria.

We also evaluated the evidence body for the primary outcomes using the Grading of Recommendations, Assessment, Development and Evaluation (GRADE) approach (Schünemann et al., 2013). The quality of evidence was graded as high, moderate, low, or very low, considering factors such as the risk of bias, imprecision, inconsistency, indirectness, and publication bias.

### 2.6 Data analysis

All meta-analyses and forest plots were generated using the Review Manager software (version 5.3, The Nordic Cochrane Centre, The Cochrane Collaboration, Copenhagen, Denmark). For dichotomous data, we calculated the relative risks (RRs) with 95% confidence intervals (CIs). For continuous outcomes, we used mean differences (MDs) with 95% CIs. All analyses followed the intention-to-treat principle when possible. Clinical and methodological diversity across included studies—key drivers of statistical heterogeneity—was identified *a priori*. A random-effects model was employed for all analyses to estimate average effect sizes of CHM interventions targeting dampness in metabolic conditions, accommodating between-study variation and ensuring conservative, robust pooled results by accounting for heterogeneity.

Pre-specified subgroup analyses were also systematically conducted to explore primary clinical sources of heterogeneity, focusing on clinically meaningful effect modifiers—including basic treatment regimens and specific types of controls. Analysing representative CHM intervention (e.g., specific decoctions or proprietary formulations), individually served as an additional strategy to address and explain heterogeneity, where fixed-effect models were used when I^2^ was <50%, and random-effects models were used when I^2^ was ≥50%. Publication bias was assessed using a funnel plot and Egger’s test if more than ten RCTs were included in the meta-analysis.

## 3 Results

### 3.1 Search results

**FIGURE 1:**
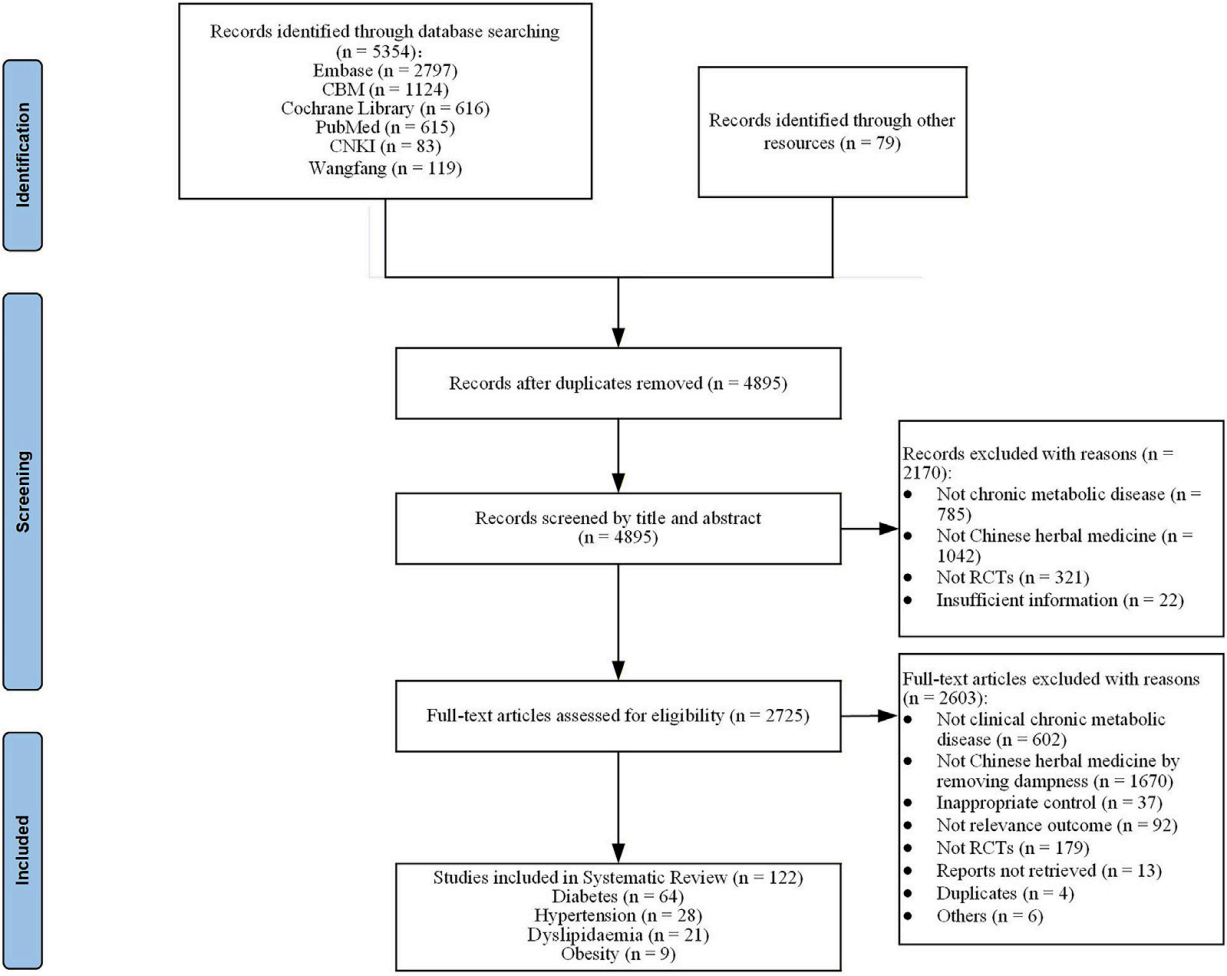
Flowchart of study selection.

The literature search identified 5,354 relevant records. After removing duplicates and study selection, 122 studies were included to the systematic review including 64 CHM RCTs for T2DM (Chen et al., 2012; Chen et al., 2022; Chen, 2022; Cheng, 2018; Dai, 2022; Deng et al., 2018; Duan et al., 2015; Fan et al., 2017; Fang, 2015; Feng et al., 2016; Fu, 2017; 2021; Ge, 2018; He et al., 2018; He and Li, 2014; Huang and Liu, 2022; Huang and Zhang, 2021; Huang et al., 2017; Jiang, 2023; Ke et al., 2019; Li, 2011; 2018; 2020; Li et al., 2020; Li X. et al., 2021; Liang and Wang, 2016; Luo et al., 2014; Ma, 2016; Ma et al., 2020; Meng et al., 2008; Ni et al., 2021; Pan et al., 2021; Song et al., 2019; Sun, 2018; Tian, 2020; Wang, 2021; Wang et al., 2018; Wang H. Y, 2022; Wang M.K. et al., 2021; Wang Q.Y. et al., 2021; Wang Y, 2022; Wu et al., 2019; Wu, 2021; Xiong, 2019; Xu et al., 2009; 2015; Yan et al., 2019; Yang, 2011; 2016; 2021; Yu et al., 2017; Yu and Chen, 2010; Zeng et al., 2006; Zhang, 2016; Zhang and Cai, 2016; Zhang et al., 2014; Zhang H.F. et al., 2019; Zhang H.J. 2019; Zhang L.N. et al., 2019; Zhang M.Q. 2019; Zheng, 2017; Zhou, 2012; 2020; Zhu, 2018), 28 for hypertension (Cheng et al., 2021; Fang, 2016; Guan and Chen, 2016; Hu, 2015; Huang, 2018; Huang and Li, 2014; Li and Wang, 2021; Lin, 2017; Liu, 2014; 2016; Liu et al., 2007; Lu, 2018; Ma et al., 2019; Mao and Li, 2022; Miao et al., 2017; Pang, 2013; Ren, 2017; 2022; Shi, 2019; Sun et al., 2021; Wang et al., 2023; Wu et al., 2020; Wu, 2019; Xiong, 2010; Yang, 2023; Yuan and Wu, 2016; Zhao et al., 2016; Zhu et al., 2019), 21 for dyslipidaemia (He et al., 2007; Hong, 2007; Hu, 2012; Kong et al., 2008; Li, 2013; Liu and Chen, 2011; Liu et al., 2008; Lu et al., 2017; Lu, 2004; Meng et al., 2004; Pan et al., 2016; Rao et al., 2015; Su et al., 2012; Tan et al., 2006; Wang and Jiao, 2012; Xiao, 2014; Xue, 2015; You, 2015; Yu et al., 2010; Zhao et al., 2021; Zheng et al., 2009) and nine for obesity (Ke et al., 2012; Lenon et al., 2012; Li et al., 2007; Liu et al., 2020; Ruan et al., 2019; Wu, 2016; Yang, 2010; Ye et al., 2016; Yu, 2016). The full process of study screening is shown in Figure 1. The list of excluded references with reasons is attached as Supplementary Table S2.

### 3.2 Study characteristics

The included 122 studies were published between 2000 and 2024, of which seven were published in English journals and 103 in Chinese journals and 12 were archived as degree thesis. The total sample size was 11,252, with an average of 92. The participants in the included RCTs were aged between 17 and 86 years, and female accounted for 45.3% of all participants. Regarding study design, all of them were parallel, controlled studies, of which seven were with multiple arms. The most common CM syndrome across four all metabolic conditions was syndrome of phlegm and dampness retained in the internal, and the most common method to assessing a CM syndrome was clinical guiding principles for new TCM drugs. The safety was monitored in 69 studies, of which 32 of them did not identify any adverse events. The details of study characteristics for individual metabolic conditions are shown in Table 1, and the full characteristics of individual studies are shown in Supplementary Tables S3–S4.

**TABLE 1:** Basic characteristics of included studies.

Basic characteristics	Number of studies (%)
T2DM	
Age (years)	
<45	5 (7.8)
≥ 45 and < 65	49 (76.6)
≥65	5 (7.8)
NR	5 (7.8)
Sex	
Male	3125 (54.7)
Female	2589 (45.3)
Disease course (years)	
<3	16 (25.0)
≥ 3 and < 10	32 (50.0)
≥10	2 (3.1)
NR	14 (21.9)
Type of comparison	
CHM vs. placebo	1 (1.6)
CHM + lifestyle intervention vs. placebo + lifestyle intervention	1 (1.6)
CHM + lifestyle intervention vs. lifestyle intervention	3 (4.7)
CHM vs. pharmacotherapy	4 (6.3)
CHM + lifestyle intervention vs. pharmacotherapy + lifestyle intervention	2 (3.1)
CHM + pharmacotherapy vs. pharmacotherapy	17 (26.6)
CHM + pharmacotherapy + lifestyle intervention vs. pharmacotherapy + lifestyle intervention	36 (56.3)
Type of intervention	
CHM decoction	56 (87.5)
Proprietary CHM	8 (12.5)
Therapeutic duration (weeks)	
<4	2 (3.1)
≥ 4 and < 12	34 (53.1)
≥12	27 (42.2)
NR	1 (1.6)
Safety assessment	
Yes	34 (53.1)
No	30 (46.9)
Spontaneous hypertension	
Age (years)	
<45	3 (10.7)
≥ 45 and < 65	21 (75.0)
≥65	4 (14.3)
Sex	
Male	1318 (54.0)
Female	1143 (46.0)
Diagnostic criteria	
WHO/International hypertension federation in 1999, which defines blood pressure >140/90 mmHg as hypertension	27 (96.4)
Hypertension was defined as blood pressure >130/80 mmHg	1 (3.6)
Disease course (years)	
<3	3 (10.7)
≥ 3 and < 10	14 (50.0)
≥10	3 (10.7)
NR	8 (28.6)
Type of comparison	
CHM + lifestyle intervention vs. placebo + lifestyle intervention	1 (3.6)
CHM vs. pharmacotherapy	3 (10.7)
CHM + pharmacotherapy vs. pharmacotherapy	16 (57.1)
CHM + pharmacotherapy + lifestyle intervention vs. pharmacotherapy + lifestyle intervention	8 (28.6)
Type of intervention	
CHM decoction	25 (89.3)
Proprietary CHM	3 (10.7)
Therapeutic duration (weeks)	
<4	1 (3.6)
≥ 4 and < 12	25 (89.3)
≥12	2 (7.1)
Safety assessment	
Yes	15 (53.6)
No	13 (46.4)
Dyslipidaemia	
Age (years)	
<45	2 (9.5)
≥ 45 and < 65	15 (71.4)
≥65	1 (4.8)
NR	3 (14.3)
Sex	
Male	1261 (61.5)
Female	791 (38.5)
Diagnostic criteria	
Dyslipidaemia was considered when at least one of the following biochemical parameters was altered: TC ≥ 6.22 mmol/L and/or TG ≥ 2.26 mmol/L and/or LDL-C ≥ 4.14 mmol/L and/or HDL-C <1.04 mmol/L	8 (38.1)
Dyslipidaemia was considered when at least one of the following biochemical parameters was altered: TC ≥ 5.72 mmol/L and/or TG ≥ 1.70 mmol/L and/or LDL-C ≥3.64 mmol/L and/or HDL-C ≤ 0.91 mmol/L	4 (19.0)
Dyslipidaemia was considered when at least one of the following biochemical parameters was altered: TC > 6.0 mmol/L and/or TG > 1.54 mmol/L and/or HDL-C ≤ 1.04 mmol/L for men and ≤1.17 mmol/L for women	2 (9.5)
Dyslipidaemia was considered when at least one of the following biochemical parameters was altered: TC > 5.72 mmol/L and/or TG > 1.70 mmol/L and/or LDL-C > 3.64 mmol/L	2 (9.5)
Dyslipidaemia was considered when at least one of the following biochemical parameters was altered: TC > 6.6 mmol/L and/or TG > 1.87 mmol/L and/or β-lipoprotein > 5.39 g/L	1 (4.8)
Dyslipidaemia was considered when at least one of the following biochemical parameters was altered: TC ≥ 6.22 mmol/L and/or TG > 5.65 mmol/L and/or LDL-C ≥ 4.1 mmol/L and/or HDL-C <0.9 mmol/L	1 (4.8)
Dyslipidaemia was considered when at least one of the following biochemical parameters was altered: TC ≥ 6.21 mmol/L and/or TG ≥ 2.26 mmol/L and/or LDL-C ≥ 4.14 mmol/L and/or HDL-C <1.03 mmol/L	1 (4.8)
Dyslipidaemia was defined as TC ≥ 6.22 mmol/L and TG ≥ 2.26 mmol/L	1 (4.8)
Dyslipidaemia was defined as TC > 5.2 mmol/L	1 (4.8)
Disease course (years)	
<3	3 (14.3)
≥3	2 (9.5)
NR	16 (76.2)
Type of comparison	
CHM vs. no treatment	1 (4.8)
CHM vs. placebo	1 (4.8)
CHM + lifestyle intervention vs. lifestyle intervention	1 (4.8)
CHM vs. pharmacotherapy	10 (47.6)
CHM + lifestyle intervention vs. pharmacotherapy + lifestyle intervention	1 (4.8)
CHM + pharmacotherapy vs. pharmacotherapy	4 (19.0)
CHM + pharmacotherapy + lifestyle intervention vs. pharmacotherapy + lifestyle intervention	3 (14.3)
Type of intervention	
CHM decoction	11 (52.4)
Proprietary CHM	10 (47.6)
Therapeutic duration (weeks)	
<12	19 (90.5)
≥12	2 (9.5)
Safety assessment	
Yes	17 (81.0)
No	4 (19.0)
Obesity	
Age (years)	
<45	8 (88.9)
≥45	1 (11.1)
Sex	
Male	241 (37.8)
Female	396 (62.2)
Diagnostic criteria	
Obesity was considered when at least two of the following three criteria were met: BMI > 26 kg/m^2^ and/or OD ≥ 20% and/or BFP ≥30%	2 (22.2)
Obesity was defined as BMI ≥28 kg/m^2^ and/or WC ≥ 85 cm for men and ≥80 cm for women	2 (22.2)
Obesity was defined as BMI ≥28 kg/m^2^ and/or WC > 90 cm for men and > 85 cm for women	1 (11.1)
Obesity was defined as BMI ≥28 kg/m^2^ and/or WC > 90 cm for men and > 80 cm for women	1 (11.1)
Obesity was defined as BMI ≥30 kg/m^2^	1 (11.1)
Obesity was defined as BMI ≥26 kg/m^2^	1 (11.1)
Obesity was defined as BMI ≥25 kg/m^2^	1 (11.1)
Disease course (years)	
<10	2 (22.2)
≥10	2 (22.2)
NR	5 (55.6)
Type of comparison	
CHM vs. placebo	2 (22.2)
CHM + lifestyle intervention vs. lifestyle intervention	7 (77.8)
Type of intervention	
CHM decoction	4 (44.2)
Proprietary CHM	5 (55.6)
Therapeutic duration (weeks)	
<12	2 (22.2)
≥12	7 (77.8)
Safety assessment	
Yes	3 (33.3)
No	6 (66.7)

BFP, body fat percentage; BMI, body mass index; CHM, chinese herbal medicine; HDL-C: high-density lipoprotein cholesterol; LDL-C, low-density lipoprotein cholesterol; NR, not reported; OD, obesity degree; TC, total cholesterol; TG, triglyceride; T2DM, type 2 diabetes mellitus; vs., versus.

Twenty-two studies assessed the independent effect of CHM for metabolic diseases, whereas 100 examined the add-on effect of CHM for the participants with lifestyle modification (16, 16%), pharmacotherapy (37, 37%), or pharmacotherapy plus lifestyle modification (47, 47%). Fifty-nine distinct formulae of CHM decoction potentially treating dampness were identified in the included RCTs, whereas six different proprietary CHM were evaluated. The therapeutic period of CHM intervention ranged from 2 weeks to 2 years.

In terms of disease diagnostic criteria, all the studies on diabetes adopted the internationally recognised standard promulgated by the World Health Organization in 1999, namely, fasting blood glucose ≥7.0 mmol/L and/or 2hPG level ≥11.0 mmol/L (World Health Organization, 1999). However, the diagnostic criteria of other metabolic diseases varied per different time periods and issuing organisation.Vascular impairments and endpoint events, including mortality, cardiac infarction, stroke, and renal failure were not reported in all the included studies.

The most frequently reported CHM formulae across the included studies were *Gegen Qinlian Decoction* (GQD) (28 studies, for T2DM), *Banxia Baizhu Tianma Decoction* (BBTD) (20 studies, for hypertension), and *Linggui Zhugan Decoction* (LZD) (3 studies, for obesity). Notably, no single CHM formula emerged as predominant in the context of dyslipidaemia, likely attributable to the significant heterogeneity of herbal compositions used in CHM decoctions for this condition. Specific to dyslipidaemia, two proprietary Chinese medicines were most extensively evaluated: Jiangzhi Tongmai Granule (5 RCTs) and berberine hydrochloride (5 RCTs). Among the individual Chinese herbs identified across the included studies, the following exhibited the highest frequency of use: Poria (Chinese pinyin: fuling), derived from the sclerotia of *Poria cocos (Schw.) Wolf*; Pinellia ternata (Chinese pinyin: banxia), obtained from the tubers of *Pinellia ternata (Thunb.) Breit.*; and Atractylodis Macrocephalae Rhizoma (Chinese pinyin: baizhu), obtained from the rhizome of *Atractylodes macrocephala Koidz*. The analysis of herbal usage patterns across different metabolic conditions revealed distinct preferences for specific Chinese herbs in their treatments, whereas Poria (Chinese pinyin: fuling), derived from the sclerotia of *P. cocos (Schw.) Wolf.*, emerged as a consistently prominent component across multiple conditions. The details on the top eight frequently used CHM formulae are presented in the Supplementary Table S5.

### 3.3 Risk-of-bias assessment in included studies

**FIGURE 2:**
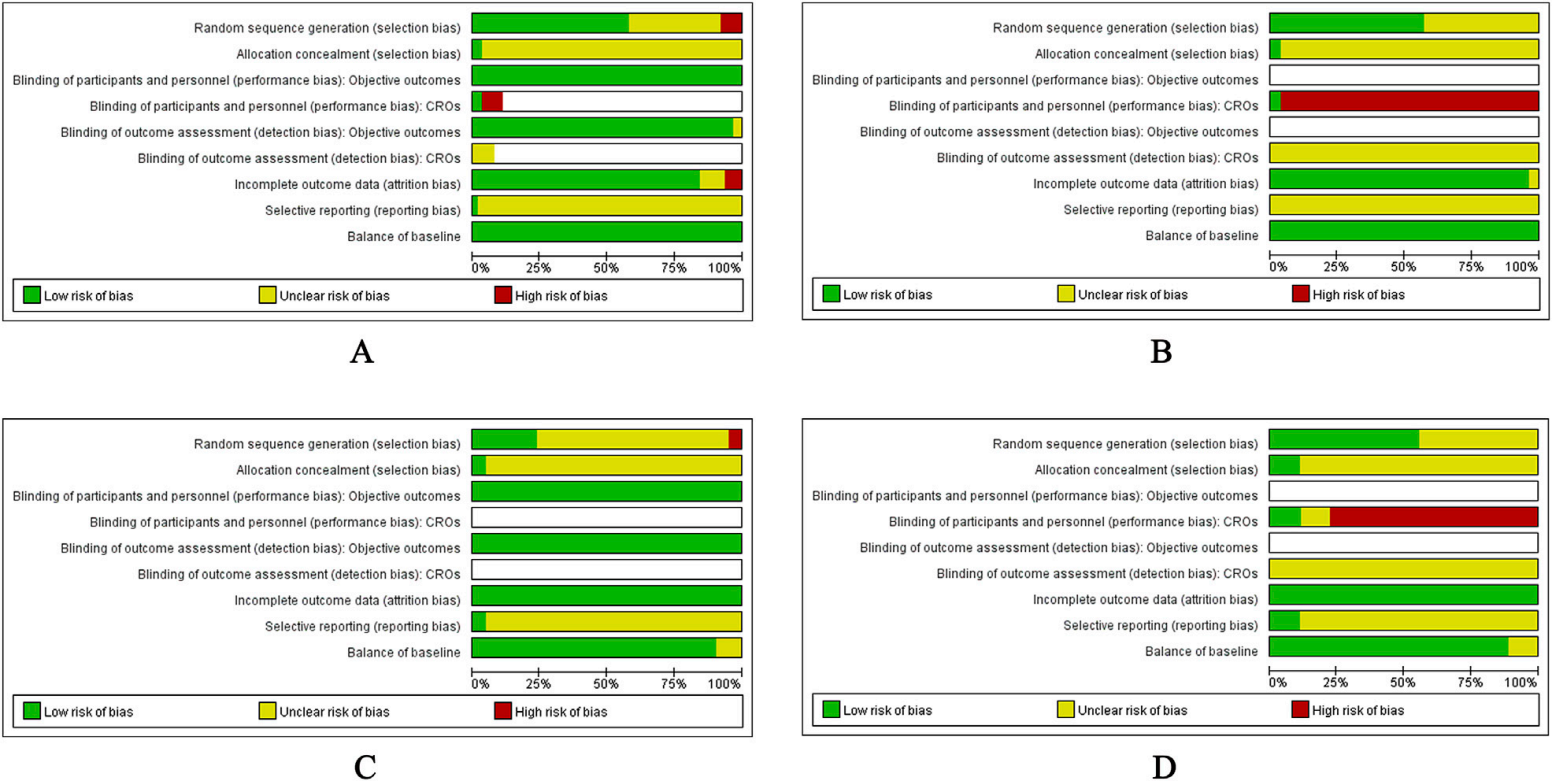
Risk of bias assessment for included studies per individual metabolic conditions. Notes: Blank space indicates that the corresponding bias risk item was not addressed or deemed not applicable in the study. Objective outcomes refer to objective data such as examination and death, including blood drawing, imaging examinations, blood glucose monitoring and all-cause mortality. CROs (clinician reporting outcomes) refer to information obtained through physical examinations or assessments by evaluators, including body mass index, blood pressure, etc. **(A)** Type 2 diabetes mellitus, **(B)** Hypertension, **(C)** Dyslipidaemia, **(D)** Obesity.

Of the 122 RCTs included in this review, only three registered their protocols in clinical trial registries (Chen et al., 2012; Lenon et al., 2012; Zhao et al., 2021). The risk of bias for the remaining 119 studies was assessed based on the published reports. Detailed results of the risk of bias assessment for individual metabolic conditions are presented in Figure 2, with full results for each study provided in Supplementary Table S6.

#### 3.3.1 Selection bias

Sixty-three (51.6%) studies had a low risk of selection bias due to random sequence generation. These studies explicitly reported methods, such as computer random number generator (1 study, 0.8%), random number table (53 studies, 43.4%), coin tossing (2 studies, 1.6%), dice rolling (3 studies, 2.5%), and drawing of lots (4 studies, 3.3%). Allocation concealment was explicitly described in 5 (4.1%) studies, achieved through central allocation systems (1 study, 0.8%), and sequentially numbered, opaque, sealed envelopes (4 studies, 3.3%). For the remaining studies, the risk of selection bias due to inadequate allocation concealment was unclear, as the sequence generation process was not reported.

#### 3.3.2 Performance and detection bias

Blinding of participants and personnel was implemented in only 5 (4.1%) studies, and none of these studies used an independent outcome assessor. Results related to objective outcomes (e.g., laboratory blood tests) in 82 (67.2%) studies were deemed unaffected by blinding status. In contrast, outcomes requiring clinical judgment (e.g., blood pressure) in 34 (27.9%) studies were considered at high risk of performance and detection bias due to the absence of blinding.

#### 3.3.3 Attrition bias

The risk of attrition bias was low in 111 (91.0%) studies owing to the following: no dropouts or lost-to-follow-up participants (101 studies, 82.8%), balanced missing data with similar reasons across groups (9 studies, 7.4%), or appropriate imputation of missing data (1 study, 0.8%).

#### 3.3.4 Reporting bias

Three (2.5%) studies were classified as low risk for reporting bias, as they fully reported all outcomes specified in their registered protocols. For the remaining studies, the risk of reporting bias was unclear owing to incomplete reporting of predefined outcomes, lack of published protocol, or no prospective registration record.

### 3.4 Results of meta-analysis

#### 3.4.1 Estimated effect of Chinese herbal medicine (CHM) for type 2 diabetes mellitus

##### 3.4.1.1 Fasting plasma glucose (FPG)

**FIGURE 3:**
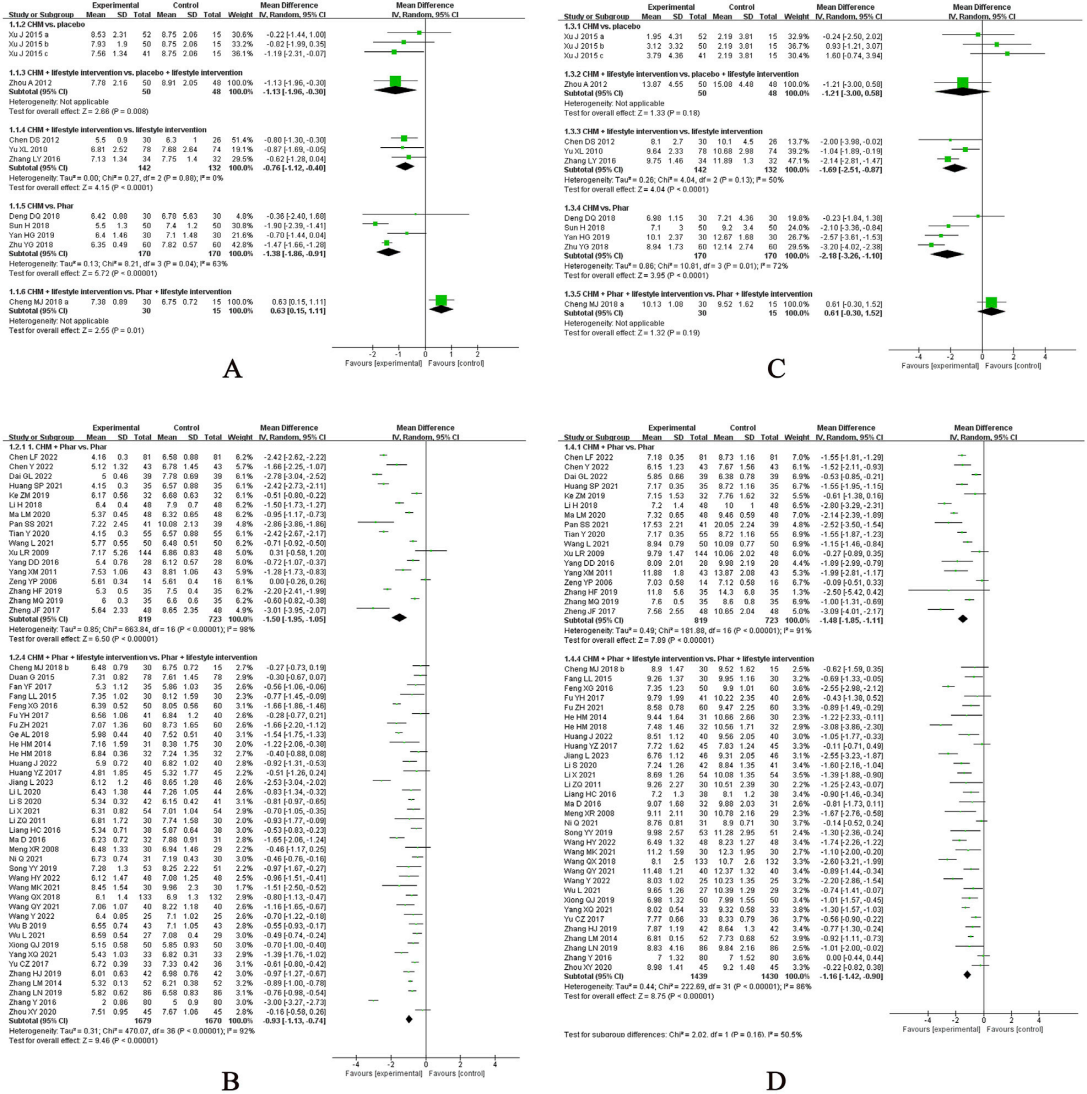
Meta-analysis of the primary outcomes for T2DM. Notes: **(A)** FPG of CHM versus control; **(B)** FPG of CHM plus conventional medicine versus control; **(C)** 2hPG of CHM versus control; **(D)** 2hPG of CHM plus conventional medicine versus control. CHM, Chinese herbal medicine; Phar, pharmacotherapy; vs., versus.

Sixty-three studies evaluated the effect of CHM on FPG levels (Chen et al., 2012; 2022; Chen, 2022; Cheng, 2018; Dai, 2022; Deng et al., 2018; Duan et al., 2015; Fan et al., 2017; Fang, 2015; Feng et al., 2016; Fu, 2017; 2021; Ge, 2018; He et al., 2018; He and Li, 2014; Huang and Liu, 2022; Huang and Zhang, 2021; Huang et al., 2017; Jiang, 2023; Ke et al., 2019; Li, 2011; 2018; 2020; Li et al., 2020; Li X. et al., 2021; Liang and Wang, 2016; Ma, 2016; Ma et al., 2020; Meng et al., 2008; Ni et al., 2021; Pan et al., 2021; Song et al., 2019; Sun, 2018; Tian, 2020; Wang, 2021; Wang et al., 2018; Wang H. Y, 2022; Wang M.K. et al., 2021; Wang Q.Y. et al., 2021; Wang Y, 2022; Wu et al., 2019; Wu, 2021; Xiong, 2019; Xu et al., 2009; 2015; Yan et al., 2019; Yang, 2011; 2016; 2021; Yu et al., 2017; Yu and Chen, 2010; Zeng et al., 2006; Zhang, 2016; Zhang and Cai, 2016; Zhang et al., 2014; Zhang H.F. et al., 2019; Zhang H.J. 2019; Zhang L.N. et al., 2019; Zhang M.Q. 2019; Zheng, 2017; Zhou, 2012; 2020; Zhu, 2018), with the meta-analysis results presented in Figures 3A,B. CHM demonstrated a statistically significant reduction in FPG compared to placebo, irrespective of whether lifestyle intervention was included as a basic therapy for both groups. Specifically, when combined with lifestyle intervention, CHM showed an MD of −1.13 mmol/L (95% CI, −1.96 to −0.30; n = 1 RCT, 98 participants). In the absence of lifestyle management, a single study comparing a CHM formula in three doses with placebo found that only the high-dose group demonstrated significant superiority of CHM over placebo in post-treatment FPG levels. The CHM formula included Puerariae lobatae radix (Chinese pinyin: gegen; dry roots of Pueraria lobata [Willd.] Ohwi), Bupleuri radix (Chinese pinyin: chaihu; dry roots of Bupleurum chinense DC.), and Dianthi herba (Chinese pinyin: qumai; dry aerial parts of Dianthus superbus L.). Among the three doses, no significant efficacy beyond placebo was observed in the low and medium doses, while the high dose alone showed statistically significant benefits. However, in a small-scale study, CHM did not outperform metformin when administered alongside lifestyle intervention (MD, 0.63 mmol/L; 95% CI, 0.15 to 1.11; n = 1 RCT, 45 participants).

In studies where pharmacotherapy was used as the baseline treatment, CHM provided additional benefits in reducing FPG levels, regardless of whether it was administered with or without structured lifestyle modification programmes. Specifically, when combined with diet and exercise, CHM yielded an MD of −0.93 mmol/L (95% CI, −1.13 to −0.74; I^2^ = 92%; n = 37 RCTs, 3349 participants). In the absence of lifestyle intervention, CHM achieved a greater reduction in FPG levels (MD, −1.50 mmol/L; 95% CI, −1.95 to −1.05; I^2^ = 98%; n = 17 RCTs, 1542 participants). Subgroup analyses were performed to evaluate the additional effects of CHM on FPG reduction when combined with different pharmacotherapies, both with and without lifestyle intervention. The results of the subgroup analyses highlight the potential of CHM as an adjunctive therapy to reduce FPG levels in patients using metformin, dipeptidyl peptidase 4 (DPP-4) inhibitors, meglitinides, α-glucosidase inhibitors, thiazolidinediones, or insulin, particularly when combined with lifestyle intervention. Notably, when CHM was combined with lifestyle intervention, it did not demonstrate additional benefits when added to glucagon-like peptide-1 (GLP-1) receptor agonists. In studies without lifestyle intervention, CHM showed significant additional benefits when combined with metformin alone, whereas the additional effect of CHM on other hypoglycaemic agents (e.g., sulfonylureas, α-glucosidase inhibitors) did not reach statistical significance. Forest plots for these subgroup analyses are provided in Supplementary Figure S1.

##### 3.4.1.2 2-h postprandial blood glucose (2h PG)

Fifty-eight studies evaluated the effect of CHM on 2h PG (Chen et al., 2012; 2022; Chen, 2022; Cheng, 2018; Dai, 2022; Deng et al., 2018; Fang, 2015; Feng et al., 2016; Fu, 2017; 2021; He et al., 2018; He and Li, 2014; Huang and Liu, 2022; Huang and Zhang, 2021; Huang et al., 2017; Jiang, 2023; Ke et al., 2019; Li, 2011; 2018; Li et al., 2020; Li X. et al., 2021; Liang and Wang, 2016; Ma, 2016; Ma et al., 2020; Meng et al., 2008; Ni et al., 2021; Pan et al., 2021; Song et al., 2019; Sun, 2018; Tian, 2020; Wang, 2021; Wang et al., 2018; Wang H. Y, 2022; Wang M.K. et al., 2021; Wang Q.Y. et al., 2021; Wang Y, 2022; Wu, 2021; Xiong, 2019; Xu et al., 2009; 2015; Yan et al., 2019; Yang, 2011; 2016; 2021; Yu et al., 2017; Yu and Chen, 2010; Zeng et al., 2006; Zhang, 2016; Zhang and Cai, 2016; Zhang et al., 2014; Zhang H.F. et al., 2019; Zhang H.J. 2019; Zhang L.N. et al., 2019; Zhang M.Q. 2019; Zheng, 2017; Zhou, 2012; 2020; Zhu, 2018), with the meta-analysis results presented in Figures 3C,D. Notably, in a small-scale study incorporating lifestyle interventions, CHM was not superior to metformin alone in reducing 2hPG levels (MD, 0.61 mmol/L; 95% CI, −0.30 to 1.52; n = 1 RCT, 45 participants).

When pharmacotherapy was used as the baseline treatment, CHM provided additional benefits in reducing 2hPG levels, regardless of whether it was combined with structured lifestyle interventions. Specifically, with lifestyle intervention, CHM yielded an MD of −1.16 mmol/L (95% CI, −1.42 to −0.90; I^2^ = 86%; n = 32 RCTs, 2869 participants). Without lifestyle intervention, CHM achieved a greater reduction in 2hPG levels (MD, −1.48 mmol/L; 95% CI, −1.85 to −1.11; I^2^ = 91%; n = 17 RCTs, 1542 participants). Subgroup analyses were performed to evaluate the additional effects of CHM on 2hPG reduction when combined with different pharmacotherapies, both with and without lifestyle intervention (Supplementary Figure S2). In studies with lifestyle intervention, subgroup analyses revealed that CHM provided additional benefits in reducing 2hPG levels when combined with specific pharmacotherapies, including metformin, DPP-4 inhibitors, meglitinides, and α-glucosidase. However, CHM did not demonstrate additional benefits in reducing 2hPG levels when added to GLP-1 receptor agonists, insulin, or thiazolidinediones. In studies without lifestyle intervention, CHM showed significant additional benefits when combined with metformin alone (MD, −1.60 mmol/L; 95% CI, −1.96 to −1.23; I^2^ = 88%; n = 13 RCTs, 1168 participants).

##### 3.4.1.3 Other outcomes

Twenty-three studies evaluated the effects of CHM on FINS levels (Chen, 2022; Fan et al., 2017; Fang, 2015; Ge, 2018; He and Li, 2014; Jiang, 2023; Li, 2020; Li et al., 2020; Li X. et al., 2021; Luo et al., 2014; Ma, 2016; Ma et al., 2020; Pan et al., 2021; Song et al., 2019; Wang, 2021; Wang et al., 2018; Wang M.K. et al., 2021; Wu et al., 2019; Wu, 2021; Xu et al., 2015; Yang, 2011; Yu et al., 2017; Yu and Chen, 2010), with the meta-analysis results presented in Supplementary Figures S3–S4. CHM administered as monotherapy alongside lifestyle modifications demonstrated superior FINS reduction compared with lifestyle management alone (MD, −2.92 mIU/L; 95% CI, −4.67 to −1.17; 1 RCT, n = 152) (Yu and Chen, 2010). In the absence of lifestyle management, a single study comparing a CHM formula in three doses with placebo found no significant superiority of CHM over placebo in post-treatment FINS levels, with no dose demonstrating efficacy beyond placebo. CHM did not outperform pioglitazone (a thiazolidinedione) in post-treatment FINS either when lifestyle interventions were controlled for (MD, −1.65 mIU/L; 95% CI, −5.22 to 1.92; 1 RCT, n = 124) (Luo et al., 2014).

With lifestyle interventions as the baseline, CHM as an adjunct to pharmacotherapy significantly reduced FINS levels, with an MD of −1.78 mIU/L (95% CI, −3.03 to −0.54; I^2^ = 96%, n = 15 RCTs, 1,345 participants). Subgroup analyses stratified based on pharmacotherapy type indicated that CHM adjunctive to metformin, α-glucosidase inhibitors, or thiazolidinediones provided additive benefits in FINS reduction when lifestyle interventions were implemented. Conversely, CHM showed no incremental advantage over insulin therapy. In trials lacking lifestyle interventions, CHM adjunctive to thiazolidinediones remained more effective than monotherapy, and small-scale trials found no superiority of CHM over metformin alone. These findings underscore context-dependent efficacy of CHM, with therapeutic benefits contingent on integration with lifestyle or pharmacological regimens.

Eighteen studies evaluated the effects of CHM on HOMA-IR (Chen et al., 2022; Fan et al., 2017; Fang, 2015; Ge, 2018; Huang and Liu, 2022; Huang and Zhang, 2021; Jiang, 2023; Li, 2020; 2011; Li et al., 2020; Ma et al., 2020; Ni et al., 2021; Pan et al., 2021; Wang, 2021; Wu, 2021; Xu et al., 2015; Yu et al., 2017; Zhang L.N. et al., 2019), with the meta-analysis results presented in Supplementary Figures S5–S6. In the absence of lifestyle management, a single study comparing three doses of the CHM formula with placebo found no significant superiority of CHM over placebo in post-treatment HOMA-IR levels, with none of the doses demonstrated superior efficacy in comparison to the placebo. However, when CHM was used as an adjunct to pharmacotherapy, it provided significant HOMA-IR reductions, regardless of lifestyle intervention status: with lifestyle intervention (MD, −1.10; 95% CI, −1.78 to −0.42; I^2^ = 98%; 12 RCTs, n = 971) and without lifestyle intervention (MD, −0.65; 95% CI, −0.81 to −0.49; I^2^ = 76%; 5 RCTs, n = 508). Subgroup analyses stratified based on pharmacotherapy type indicated that CHM adjunctive to metformin or meglitinides significantly reduced HOMA-IR in participants receiving lifestyle interventions, although no additional benefit was observed compared with insulin therapy alone. In trials lacking lifestyle intervention, CHM adjunctive to metformin or thiazolidinediones demonstrated superior HOMA-IR reductions compared with monotherapy. These findings highlight the therapeutic potential of CHM as an adjunctive treatment, particularly when combined with pharmacotherapy.

#### 3.4.2 Estimated effects of CHM for hypertension

##### 3.4.2.1 Systolic blood pressure (SBP)

**FIGURE 4:**
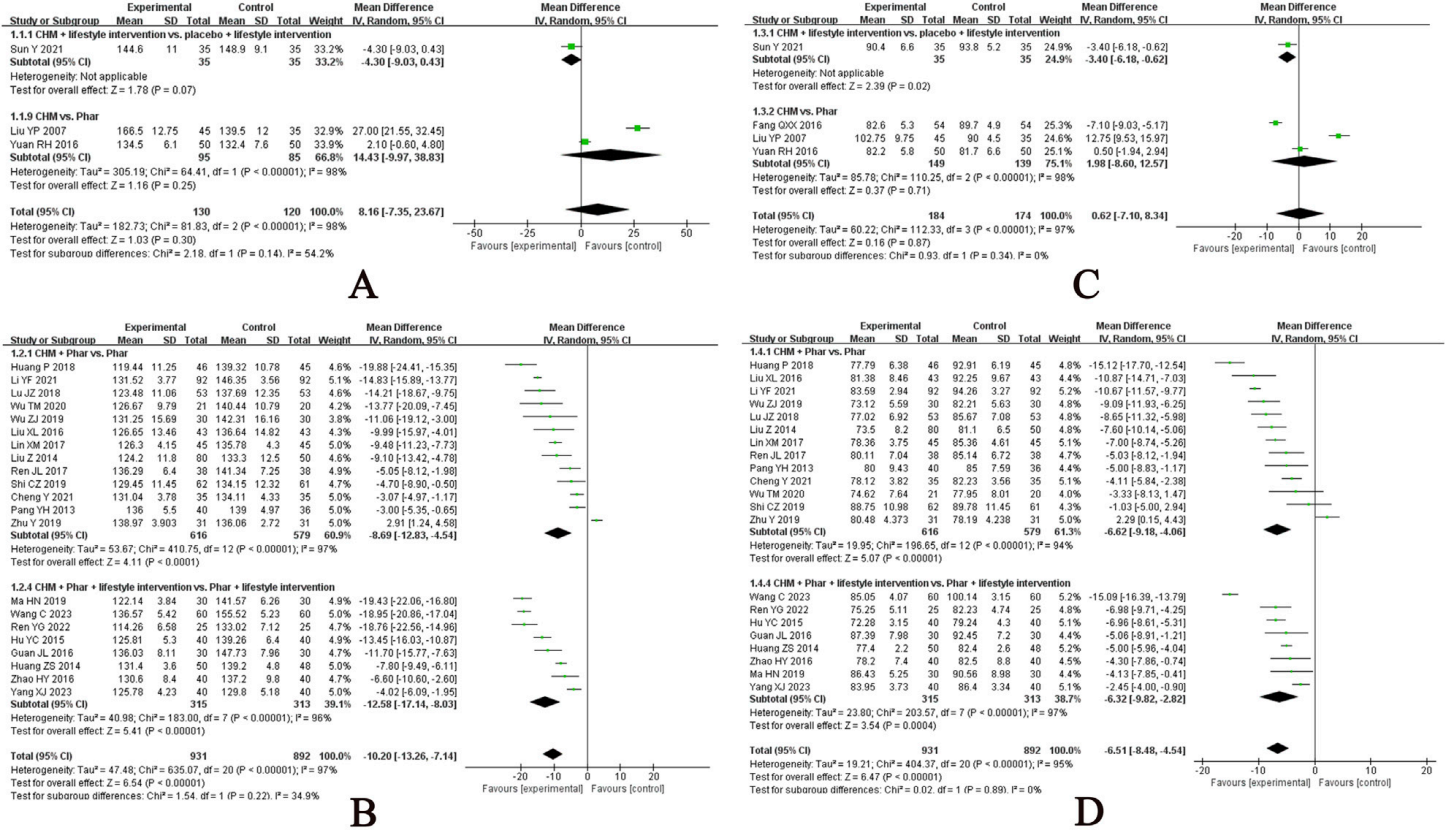
Meta-analysis of the primary outcomes for hypertension. Notes: **(A)** SBP of CHM versus control; **(B)** SBP of CHM plus conventional medicine versus control; **(C)** DBP of CHM versus control; **(D)** DBP of CHM plus conventional medicine versus control. CHM, Chinese herbal medicine; Phar, pharmacotherapy; vs., versus.

Twenty-four studies evaluated the effects of CHM on SBP (Cheng et al., 2021; Guan and Chen, 2016; Hu, 2015; Huang, 2018; Huang and Li, 2014; Li and Wang, 2021; Lin, 2017; Liu, 2016; 2014; Liu et al., 2007; Lu, 2018; Ma et al., 2019; Pang, 2013; Ren, 2017; 2022; Shi, 2019; Sun et al., 2021; Wang et al., 2023; Wu et al., 2020; Wu, 2019; Yang, 2023; Yuan and Wu, 2016; Zhao et al., 2016; Zhu et al., 2019), with the meta-analysis results presented in Figures 4A,B. A single-center, small-scale study using a self-formulated CHM regimen showed no significant benefit over placebo when combined with lifestyle intervention (MD, −4.30 mmHg; 95% CI, −9.03 to 0.43; 1 RCT, n = 70). Similarly, CHM demonstrated no significant advantage over renin–angiotensin system (RAS) inhibitors as monotherapy (MD, 14.43 mmHg; 95% CI, −9.97 to 38.83; I^2^ = 98%; 2 RCTs, n = 180). However, when used as an adjunct to pharmacotherapy, CHM provided significant additional benefits in reducing SBP compared with monotherapy alone (MD, −10.20 mmHg; 95% CI, −13.26 to −7.14; I^2^ = 97%; 21 RCTs, n = 1823). Subgroup analyses further indicated that CHM adjunctive to pharmacotherapy reduced SBP, irrespective of lifestyle intervention status: with lifestyle intervention (MD, −12.58 mmHg; 95% CI, −17.14 to −8.03; I^2^ = 96%; 8 RCTs, n = 628) and without lifestyle intervention (MD, −8.69 mmHg; 95% CI, −12.83 to −4.54; I^2^ = 97%; 13 RCTs, n = 1195). Stratification based on comparator type revealed that CHM provided additive benefits when combined with calcium channel blockers (CCBs) or RAS inhibitors, even in the absence of lifestyle intervention. However, no significant add-on effect was observed when CHM was combined with dual antihypertensive therapy (e.g., CCB + RAS inhibitor). These findings suggest that CHM may enhance SBP reduction when used as an adjunct to pharmacotherapy, particularly in combination with CCBs or RAS inhibitors, although its efficacy remains contingent on intervention context and comparator regimens. Forest plots of subgroup analyses are provided in Supplementary Figure S7.

##### 3.4.2.2 Diastolic blood pressure (DBP)

Twenty-five studies evaluated the effects of CHM on DBP (Cheng et al., 2021; Fang, 2016; Guan and Chen, 2016; Hu, 2015; Huang, 2018; Huang and Li, 2014; Li and Wang, 2021; Lin, 2017; Liu, 2016; 2014; Liu et al., 2007; Lu, 2018; Ma et al., 2019; Pang, 2013; Ren, 2017; 2022; Shi, 2019; Sun et al., 2021; Wang et al., 2023; Wu et al., 2020; Wu, 2019; Yang, 2023; Yuan and Wu, 2016; Zhao et al., 2016; Zhu et al., 2019), with the meta-analysis results presented in Figures 4C,D. A small-scale trial demonstrated that CHM, when combined with lifestyle intervention, significantly reduced DBP compared with placebo (MD, −3.40 mmHg; 95% CI, −6.18 to −0.62; 1 RCT, n = 70). However, CHM showed no significant advantage over pharmacotherapy alone (MD, 1.98 mmHg; 95% CI, −8.60 to 12.57; I^2^ = 98%; 3 RCTs, n = 288). When used as an adjunct to pharmacotherapy, CHM provided substantial additional benefits in lowering DBP compared with monotherapy or dual antihypertensive (MD, −6.51 mmHg; 95% CI, −8.48 to −4.54; I^2^ = 95%; 21 RCTs, n = 1823). Subgroup analyses indicated that CHM consistently reduced DBP, irrespective of lifestyle intervention status: with lifestyle intervention (MD, −6.32 mmHg; 95% CI, −9.82 to −2.82; I^2^ = 97%; 8 RCTs, n = 628) and without lifestyle intervention (MD, −6.62 mmHg; 95% CI, −9.18 to −4.06; I^2^ = 94%; 13 RCTs, n = 1195). Further stratification based on comparator type revealed that CHM provided additive benefits across various antihypertensive regimens, including CCBs, RAS inhibitors, and their combinations, suggesting broad applicability as an adjunctive therapy. Forest plots of subgroup analyses are provided in Supplementary Figure S8.

##### 3.4.2.3 Other outcomes

Only one study (n = 70) using ambulatory blood pressure monitoring (ABPM) evaluated the single effects of CHM on patients with hypertension with lifestyle intervention as the baseline (Sun et al., 2021), which reported that CHM significantly reduced 24-h SBP (MD, −7.20 mmHg; 95% CI, −12.50 to −1.90), daytime SBP (MD, −6.40 mmHg; 95% CI, −12.31 to −0.49), nighttime SBP (MD, −12.30 mmHg; 95% CI, −19.59 to −5.01), and nighttime DBP (MD, −7.40 mmHg; 95% CI, −12.18 to −2.62), but showed no significant effect on 24-h DBP (MD, −3.20 mmHg; 95% CI, −8.12 to 1.72) or daytime DBP (MD, −1.30 mmHg; 95% CI, −6.43 to 3.83). Meta-analysis of three additional studies (n = 208) (Mao and Li, 2022; Miao et al., 2017; Xiong, 2010) demonstrated that CHM as an adjunct to pharmacotherapy significantly lowered 24-h SBP (MD, −5.97 mmHg; 95% CI, −11.83 to −0.12; I^2^ = 75%) and nighttime SBP (MD, −7.40 mmHg; 95% CI, −11.55 to −3.25), but not 24-h DBP (MD, −5.39 mmHg; 95% CI, −12.89 to 2.11; I^2^ = 91%), daytime SBP (MD, 7.20 mmHg; 95% CI, 1.20–13.20), daytime DBP (MD, 5.90 mmHg; 95% CI, 2.11–9.69), or nighttime DBP (MD, −2.83 mmHg; 95% CI, −7.60 to 1.94) (Supplementary Figure S9). These findings suggest that CHM may enhance SBP reduction in specific contexts, although its effects on DBP remain inconsistent.

#### 3.4.3 Estimated effects of CHM for dyslipidaemia

##### 3.4.3.1 Triglycerides (TG)

**FIGURE 5:**
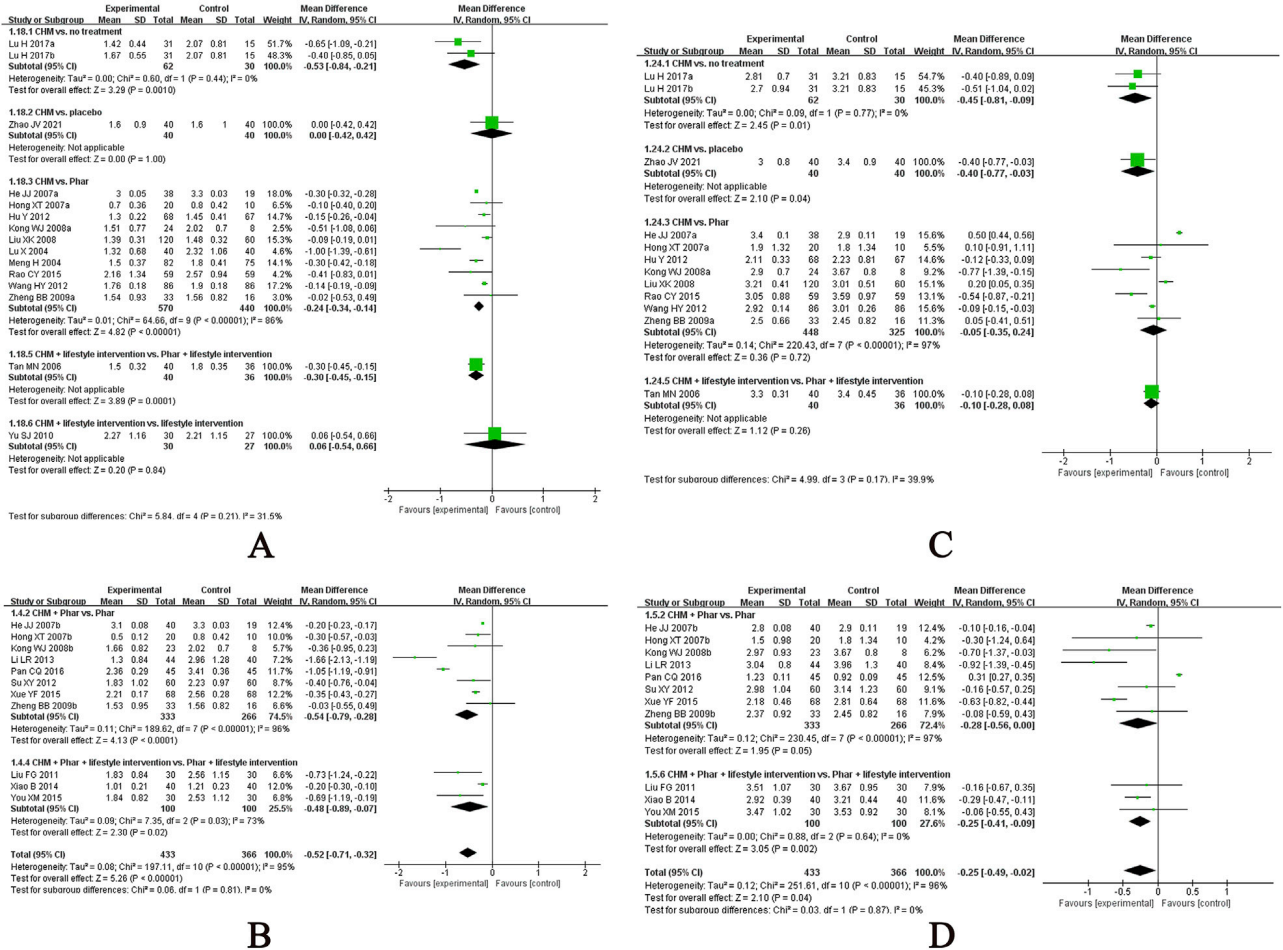
Meta-analysis of the primary outcomes for dyslipidemia. Notes: **(A)** TG of CHM versus control; **(B)** TG of CHM plus conventional medicine versus control; **(C)** LDL-C of CHM versus control; **(D)** LDL-C of CHM plus conventional medicine versus control. CHM, Chinese herbal medicine; Phar, pharmacotherapy; vs., versus.

Twenty-one studies evaluated the effects of CHM on TG levels (He et al., 2007; Hong, 2007; Hu, 2012; Kong et al., 2008; Li, 2013; Liu and Chen, 2011; Liu et al., 2008; Lu et al., 2017; Lu, 2004; Meng et al., 2004; Pan et al., 2016; Rao et al., 2015; Su et al., 2012; Tan et al., 2006; Wang and Jiao, 2012; Xiao, 2014; Xue, 2015; You, 2015; Yu et al., 2010; Zhao et al., 2021; Zheng et al., 2009), with the meta-analysis results presented in Figures 5A,B. CHM significantly reduced TG levels compared with no treatment (MD, −0.53 mmol/L; 95% CI, −0.84 to −0.21; I^2^ = 0%; 1 RCT, n = 92). CHM consistently outperformed pharmacotherapy in reducing TG levels, regardless of lifestyle intervention status: with lifestyle intervention (MD, −0.30 mmol/L; 95% CI, −0.45 to −0.15; 1 RCT, n = 76) and without lifestyle intervention (MD, −0.24 mmol/L; 95% CI, −0.34 to −0.14; I^2^ = 86%; 10 RCTs, n = 1010). Certain single CHM formulae showed no significant therapeutic effect. For instance, the self-formulated Qushi Huayu Tongluo Decoction demonstrated no advantage over lifestyle management alone (MD, 0.06 mmol/L; 95% CI, −0.54 to 0.66; 1 RCT, n = 57). Similarly, berberine, evaluated in one study (n = 80), showed no superiority over placebo (MD, 0.00 mmol/L; 95% CI, −0.42 to 0.42). These findings highlight CHM’s potential as an alternative therapy for TG reduction, although outcomes vary based on intervention context and specific formulations.

A meta-analysis of 11 RCTs (n = 799) demonstrated that CHM adjunctive to pharmacotherapy significantly reduced TG compared with pharmacotherapy alone (MD, −0.52 mmol/L; 95% CI, −0.71 to −0.32; I^2^ = 95%). Subgroup analyses indicated consistent benefits, regardless of lifestyle intervention status: with lifestyle intervention (MD, −0.48 mmol/L; 95% CI, −0.89 to −0.07; I^2^ = 73%; 3 RCTs, n = 200) and without lifestyle intervention (MD, −0.54 mmol/L; 95% CI, −0.79 to −0.28; I^2^ = 96%; 8 RCTs, n = 599). Stratification based on pharmacotherapy type revealed that CHM provided additive TG-lowering effects when combined with statins (MD, −0.41 mmol/L; 95% CI, −0.58 to −0.23; I^2^ = 88%; 7 RCTs, n = 509) or cholesterol absorption inhibitors (MD, −1.05 mmol/L; 95% CI, −1.19 to −0.91; 1 RCT, n = 90), irrespective of lifestyle intervention, with the meta-analysis results presented in Supplementary Figure S10. These findings support CHM’s role as a therapeutic adjunct for TG reduction across diverse clinical contexts.

##### 3.4.3.2 Low-density lipoprotein cholesterol (LDL-C)

Eighteen studies evaluated the effects of CHM on LDL-C levels (He et al., 2007; Hong, 2007; Hu, 2012; Kong et al., 2008; Li, 2013; Liu and Chen, 2011; Liu et al., 2008; Lu et al., 2017; Pan et al., 2016; Rao et al., 2015; Su et al., 2012; Tan et al., 2006; Wang and Jiao, 2012; Xiao, 2014; Xue, 2015; You, 2015; Zhao et al., 2021; Zheng et al., 2009), with the meta-analysis results presented in Figures 5C,D. Among participants without lifestyle intervention, CHM outperformed placebo (MD, −0.40 mmol/L; 95% CI, −0.77 to −0.03; 1 RCT, n = 80) and no treatment (MD, −0.45 mmol/L; 95% CI, −0.81 to −0.09; I^2^ = 0%; 1 RCT, n = 92). However, CHM showed no significant advantage over pharmacotherapy alone in other analyses. When used as an adjunct to pharmacotherapy, CHM significantly reduced LDL-C levels compared with monotherapy (MD, −0.25 mmol/L; 95% CI, −0.49 to −0.02; I^2^ = 96%; 11 RCTs, n = 799). Subgroup analyses indicated consistent benefits, regardless of lifestyle intervention status: with lifestyle management (MD, −0.25 mmol/L; 95% CI, −0.41 to −0.09; I^2^ = 0%; 3 RCTs, n = 200) and without lifestyle intervention (MD, −0.28 mmol/L; 95% CI, −0.56 to 0.00; I^2^ = 97%; 8 RCTs, n = 599), with the meta-analysis results presented in Supplementary Figure S11. Notably, CHM provided additive LDL-C-lowering effects when combined with statins. These findings support the potential role of CHM as a therapeutic adjunct for LDL-C reduction across diverse clinical contexts.

##### 3.4.3.3 Other outcomes

Twenty-one studies evaluated the effects of CHM on TC levels (He et al., 2007; Hong, 2007; Hu, 2012; Kong et al., 2008; Li, 2013; Liu and Chen, 2011; Liu et al., 2008; Lu et al., 2017; Lu, 2004; Meng et al., 2004; Pan et al., 2016; Rao et al., 2015; Su et al., 2012; Tan et al., 2006; Wang and Jiao, 2012; Xiao, 2014; Xue, 2015; You, 2015; Yu et al., 2010; Zhao et al., 2021; Zheng et al., 2009) (Supplementary Figures S12–S13). Among participants without lifestyle intervention, CHM outperformed placebo (MD, −0.50 mmol/L; 95% CI, −0.94 to −0.06; 1 RCT, n = 80) and no treatment (MD, −0.61 mmol/L; 95% CI, −0.95 to −0.26; I^2^ = 0%; 1 RCT, n = 92). When used as an adjunct to pharmacotherapy, CHM significantly reduced TC levels compared with monotherapy (MD, −0.44 mmol/L; 95% CI, −0.66 to −0.22; I^2^ = 90%; 11 RCTs, n = 799). Subgroup analyses indicated consistent benefits, regardless of lifestyle intervention status (MD, −0.49 mmol/L; 95% CI, −0.74 to −0.24; I^2^ = 91%; 8 RCTs, n = 599). CHM provided additive TC-lowering effects when combined with statins or cholesterol absorption inhibitors, irrespective of lifestyle intervention.

A meta-analysis of 19 studies evaluating HDL-C changes with CHM revealed significant outcomes (He et al., 2007; Hong, 2007; Hu, 2012; Kong et al., 2008; Li, 2013; Liu and Chen, 2011; Liu et al., 2008; Lu et al., 2017; Lu, 2004; Pan et al., 2016; Rao et al., 2015; Su et al., 2012; Tan et al., 2006; Wang and Jiao, 2012; Xiao, 2014; Xue, 2015; You, 2015; Zhao et al., 2021; Zheng et al., 2009) (Supplementary Figures S14–S15). CHM significantly increased HDL-C levels compared with no treatment (MD, 0.32 mmol/L; 95% CI, 0.15 to 0.48; I^2^ = 29%; 1 RCT, n = 92) and pharmacotherapy (MD, 0.17 mmol/L; 95% CI, 0.07 to 0.27; I^2^ = 88%; 9 RCTs, n = 853) in the absence of lifestyle interventions. However, a single study found no significant difference between berberine and placebo (MD, −0.11 mmol/L; 95% CI, −0.43 to 0.21; 1 RCT, n = 80). When used as an adjunct to pharmacotherapy, CHM showed no additional benefit on HDL-C levels, regardless of lifestyle intervention status: with lifestyle intervention (MD, 0.03 mmol/L; 95% CI, −0.02 to 0.08; I^2^ = 0%; 3 RCTs, n = 200) and without lifestyle intervention (MD, 0.14 mmol/L; 95% CI, −0.34 to 0.62; I^2^ = 100%; 8 RCTs, n = 599). These findings suggest that CHM may enhance HDL-C levels in specific contexts but lacks additive effects when combined with pharmacotherapy.

#### 3.4.4 Estimated effects of CHM for obesity

##### 3.4.4.1 Body mass index (BMI)

**FIGURE 6:**
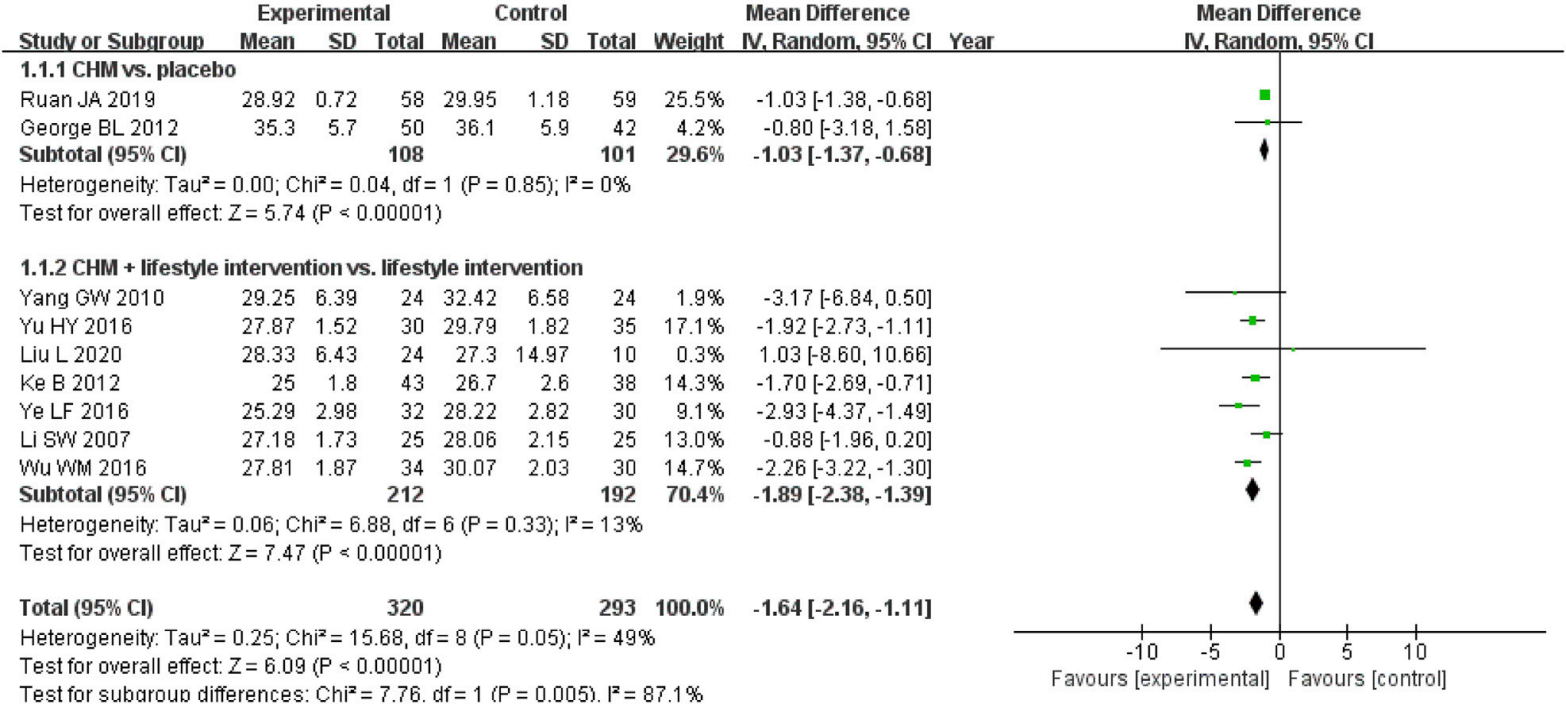
Meta-analysis of the primary outcome for obesity. Notes: CHM, Chinese herbal medicine; vs., versus.

A meta-analysis of nine studies evaluating BMI changes with CHM showed significant reductions in two contexts (Ke et al., 2012; Lenon et al., 2012; Li et al., 2007; Liu et al., 2020; Ruan et al., 2019; Wu, 2016; Yang, 2010; Ye et al., 2016; Yu, 2016) (Figure 6). Among participants without lifestyle intervention, CHM outperformed placebo (MD, −1.03 kg/m^2^; 95% CI, −1.37 to −0.68; I^2^ = 0%; 2 RCTs, n = 209). When combined with a proper diet and exercise programme, CHM demonstrated greater BMI reduction than lifestyle management alone (MD, −1.89 kg/m^2^; 95% CI, −2.38 to −1.39; I^2^ = 13%; 7 RCTs, n = 404). These findings highlight CHM’s potential for BMI reduction both as monotherapy and when integrated with lifestyle interventions.

##### 3.4.4.2 Other outcomes

Other studies evaluated the effects of CHM on anthropometric measures (Supplementary Figures S16–S18). For WC, CHM combined with lifestyle interventions showed greater reductions than lifestyle management alone (MD, −2.40 cm; 95% CI, −3.61 to −1.18; I^2^ = 0%; 3 RCTs, n = 193) (Ke et al., 2012; Li et al., 2007; Ye et al., 2016), but there was no significant difference from placebo without lifestyle intervention (MD, −2.37 cm; 95% CI, −5.44 to 0.70; I^2^ = 44%; 2 RCTs, n = 209) (Lenon et al., 2012; Ruan et al., 2019). For HC, CHM showed no superiority to placebo without lifestyle intervention (MD, −0.62 cm; 95% CI, −1.96 to 0.72; I^2^ = 0%; 2 RCTs, n = 209) (Lenon et al., 2012; Ruan et al., 2019). For WHR, CHM combined with lifestyle management was more effective than lifestyle alone (MD, −0.04; 95% CI, −0.07 to −0.01; I^2^ = 84%; 3 RCTs, n = 179) (Li et al., 2007; Wu, 2016; Yu, 2016), but not placebo without lifestyle intervention (MD, 0.02; 95% CI, −0.01 to 0.05; 1 RCT, n = 92) (Lenon et al., 2012). The results highlight CHM’s potential when integrated with lifestyle interventions for specific measures.

### 3.5 Adverse events in all included studies

**FIGURE 7:**
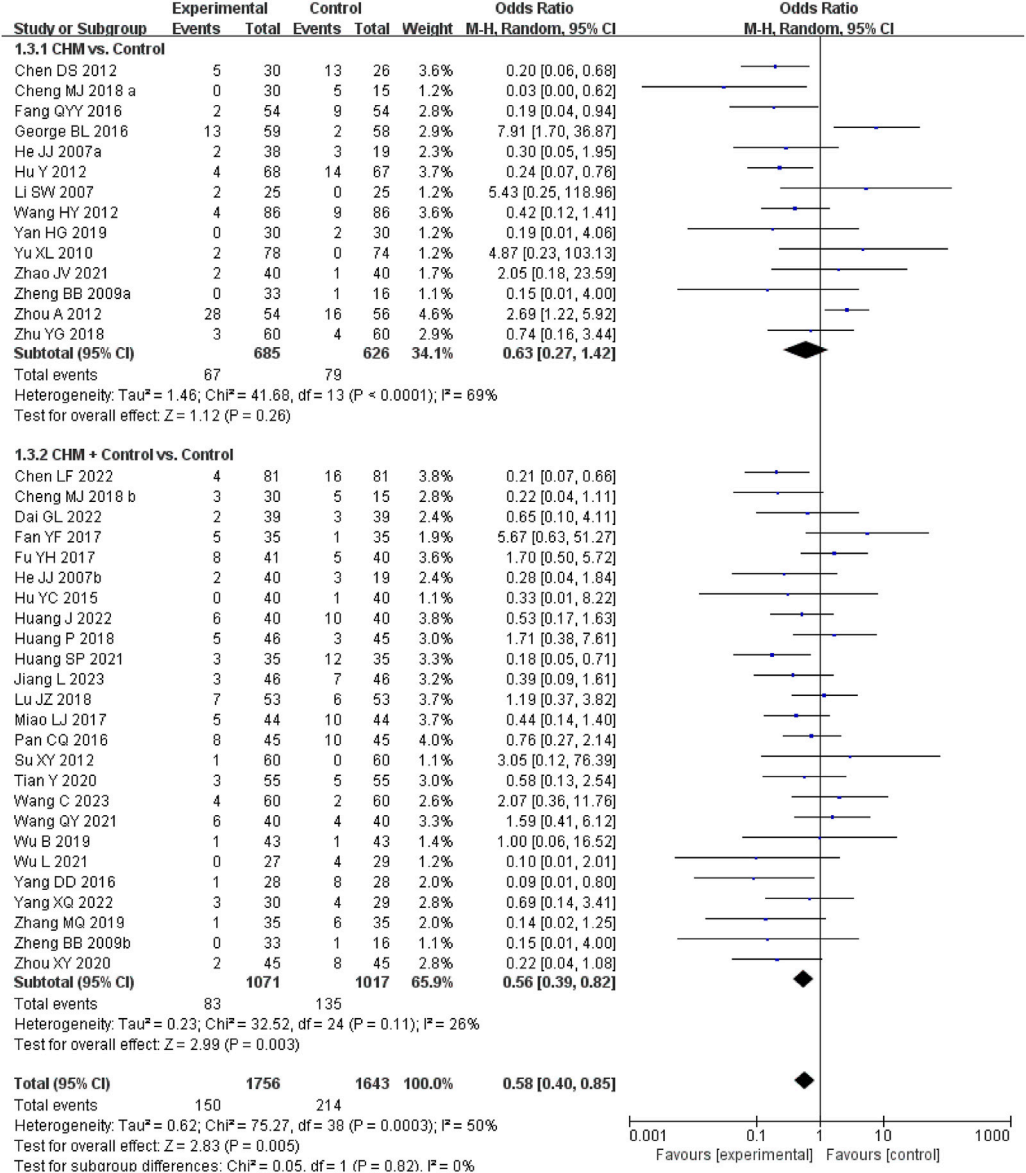
Meta-analysis of adverse effects of CHM eliminating dampness for metabolic conditions. Notes: CHM, Chinese herbal medicine; vs., versus.

A meta-analysis of 45 RCTs evaluating CHM for eliminating dampness combined with specialised drugs revealed insights into adverse events. Among these, 22 studies reported no adverse events in either the experimental or control group (Fang, 2015; Feng et al., 2016; Guan and Chen, 2016; He et al., 2018; Huang et al., 2017; Li, 2018; 2013; 2011; Liang and Wang, 2016; Lin, 2017; Liu and Chen, 2011; Ma, 2016; Ma et al., 2019; Mao and Li, 2022; Meng et al., 2008; Shi, 2019; Wu, 2019; Xiao, 2014; Xue, 2015; You, 2015; Zhang H.J., 2019; Zhu et al., 2019), whereas 23 studies documented adverse events (Chen et al., 2022; Dai, 2022; Fan et al., 2017; Fu, 2017; Hu, 2015; Huang and Liu, 2022; Huang, 2018; Huang and Zhang, 2021; Jiang, 2023; Lu, 2018; Miao et al., 2017; Pan et al., 2016; Su et al., 2012; Tian, 2020; Wang et al., 2023; Wang Q.Y. et al., 2021; Wu et al., 2019; Wu, 2021; Yang, 2016; 2023; 2021; Zhang M.Q., 2019; Zhou, 2020). A meta-analysis of these 23 studies indicated that adjunctive CHM therapy resulted in fewer adverse events compared with pharmacotherapy alone (RR = 0.56; 95% CI, 0.39–0.82). In direct comparisons between CHM for eliminating dampness and specialised drugs, 24 RCTs reported adverse events. A meta-analysis of 14 of these studies showed no significant difference in adverse event incidence between the two groups (RR = 0.63; 95% CI, 0.27–1.42). The most common adverse events in the CHM group were mild digestive issues, such as nausea, vomiting, and diarrhoea, which typically resolved with or without intervention. Importantly, no studies reported severe adverse events, indicating a favourable safety profile for CHM in this context. Figure 7 presents the forest plots on the meta-analysis of adverse events, and Supplementary Table S7 details the individual adverse events.

### 3.6 Estimated effect of promising CHM formulae

**FIGURE 8:**
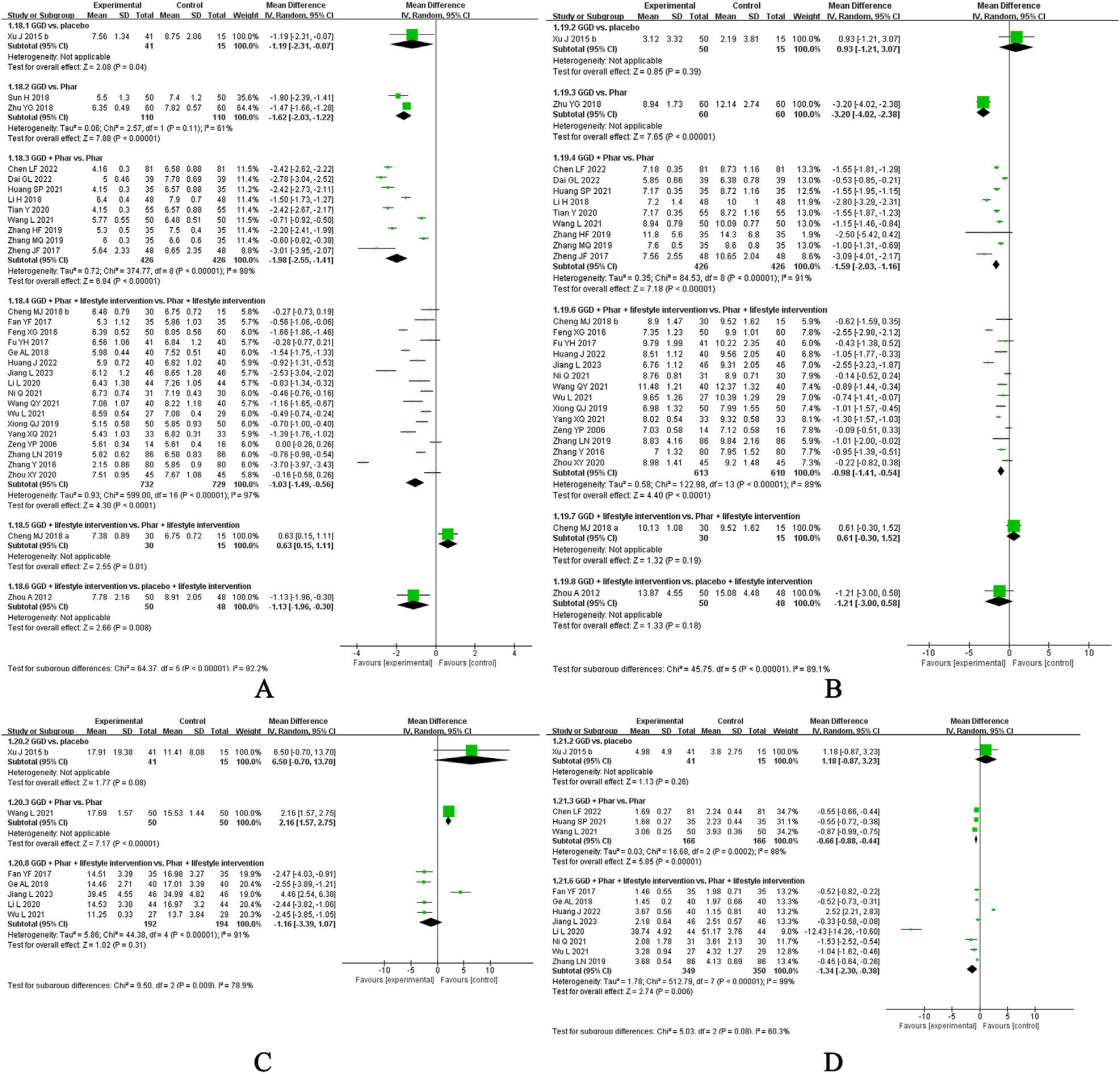
Meta-analysis of Gegen Qinlian Decoction for T2DM. Notes: **(A)** Meta-analysis of Gegen Qinlian Decoction of FPG for T2DM; **(B)** Meta-analysis of Gegen Qinlian Decoction of 2hPG for T2DM; **(C)** Meta-analysis of Gegen Qinlian Decoction of FINS for T2DM; **(D)** Meta-analysis of Gegen Qinlian Decoction of HOMA-IR for T2DM. GGD, Gegen Qinlian Decoction; Phar, pharmacotherapy; vs., versus.

**FIGURE 9:**
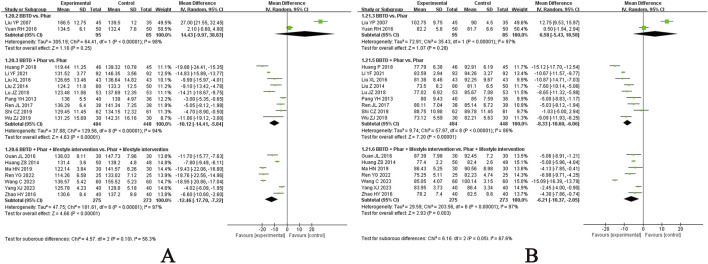
Meta-analysis of Banxia Baizhu Tianma Decoction for hypertension. Notes: **(A)** Meta-analysis of Banxia Baizhu Tianma Decoction of SBP for hypertension; **(B)** Meta-analysis of Banxia Baizhu Tianma Decoction of DBP for hypertension. BBTD, Banxia Baizhu Tianma Decoction; Phar, pharmacotherapy; vs., versus.

**FIGURE 10:**
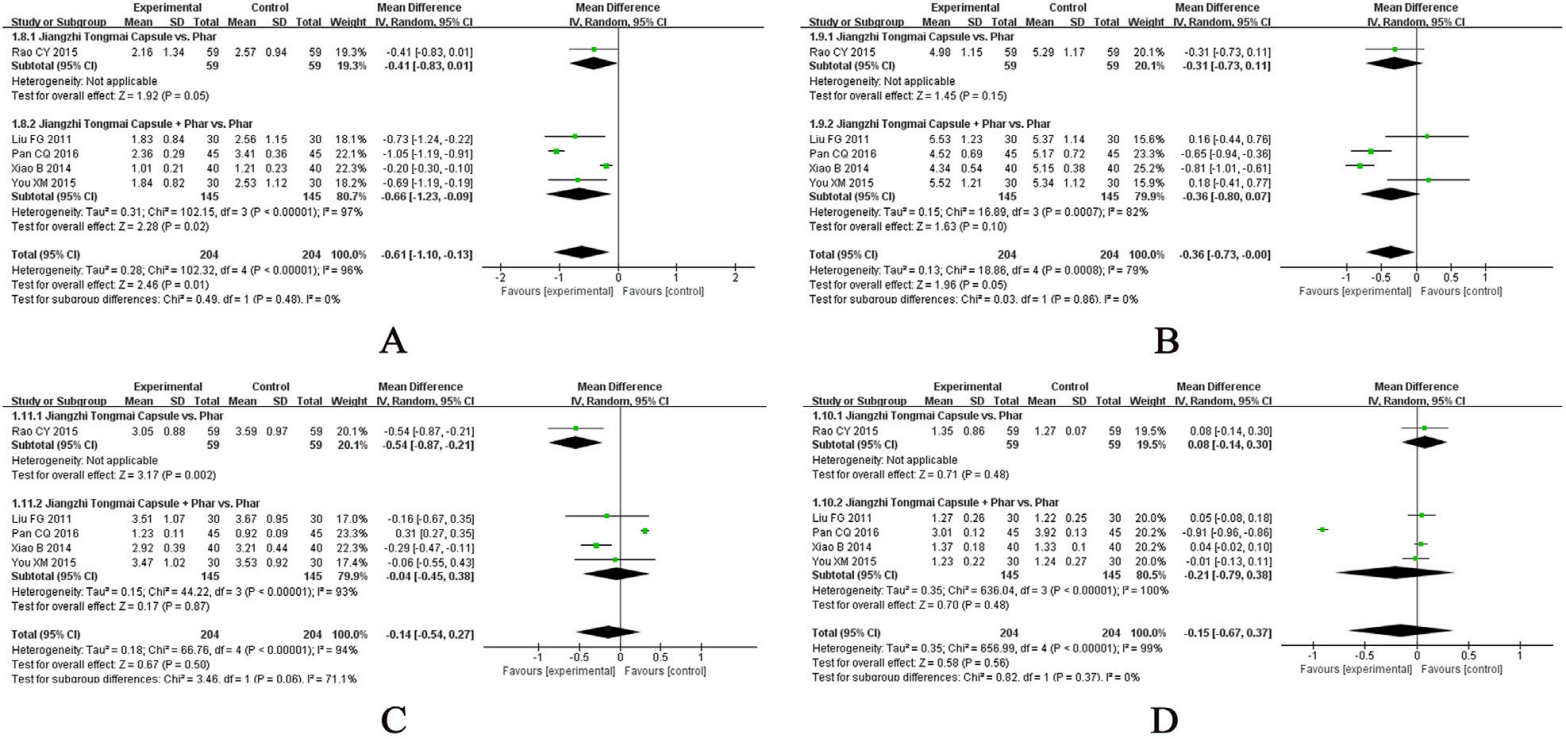
Meta-analysis of Jiangzhi Tongmai Capsule for dyslipidemia. Notes: **(A)** Meta-analysis of Jiangzhi Tongmai Capsule of TG for dyslipidemia; **(B)** Meta-analysis of Jiangzhi Tongmai Capsule of TC for dyslipidemia; **(C)** Meta-analysis of Jiangzhi Tongmai Capsule of LDL-C for dyslipidemia; **(D)** Meta-analysis of Jiangzhi Tongmai Capsule of HDL-C for dyslipidemia. Phar, pharmacotherapy; vs., versus.

**FIGURE 11:**
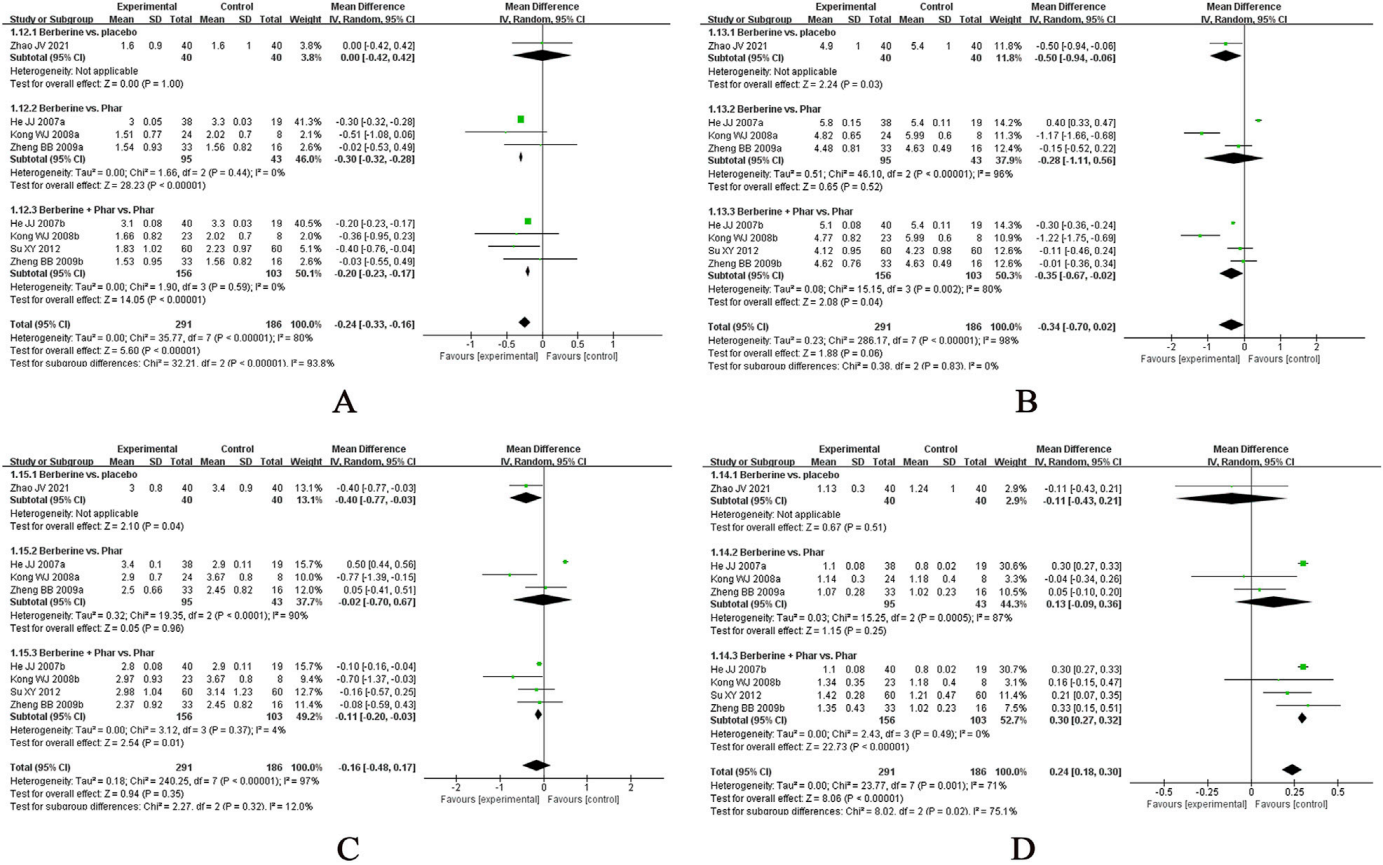
Meta-analysis of Berberine for dyslipidemia. Notes: **(A)** Meta-analysis of Berberine of TG for dyslipidemia; **(B)** Meta-analysis of Berberine of TC for dyslipidemia; **(C)** Meta-analysis of Berberine of LDL-C for dyslipidemia; **(D)** Meta-analysis of Berberine of HDL-C for dyslipidemia. Phar, pharmacotherapy; vs., versus.

**FIGURE 12:**
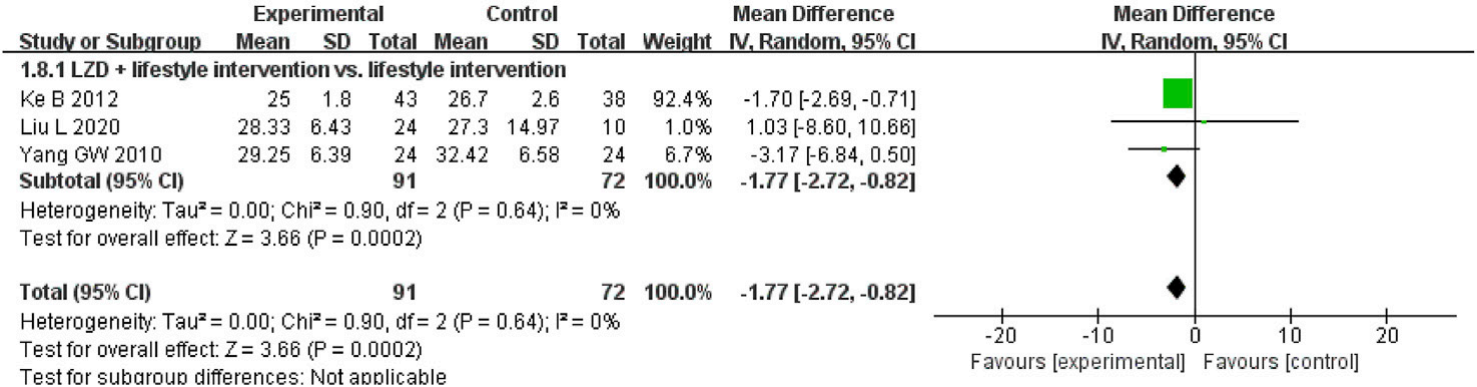
Meta-analysis of Linggui Zhugan Decoction for obesity. Notes: Meta-analysis of Linggui Zhugan Decoction of BMI for obesity. LZD, Linggui Zhugan Decoction; vs., versus.

In this systematic review, several CHM formulae exhibited notable therapeutic effects across different disease conditions:

• GQD (Chen et al., 2022; Cheng, 2018; Dai, 2022; Fan et al., 2017; Feng et al., 2016; Fu, 2017; Ge, 2018; Huang and Liu, 2022; Huang and Zhang, 2021; Jiang, 2023; Li, 2018; 2020; Ni et al., 2021; Sun, 2018; Tian, 2020; Wang, 2021; Wu, 2021; Xiong, 2019; Xu et al., 2015; Yang, 2021; Zeng et al., 2006; Zhang, 2019; Zhang and Cai, 2016; Zhang H.F. et al., 2019; Zhang L.N. et al., 2019; Zheng, 2017; Zhou, 2012; 2020; Zhu, 2018) for T2DM: Most evaluated in included RCTs, this decoction showed both alternative and add-on effects in reducing FPG levels and exhibited add-on effects on post-treatment HOMA-IR and 2hPG levels in participants using hypoglycaemic agents (Figure 8).
• BBTD (Guan and Chen, 2016; Huang, 2018; Huang and Li, 2014; Li and Wang, 2021; Liu, 2016; 2014; Liu et al., 2007; Lu, 2018; Ma et al., 2019; Pang, 2013; Ren, 2017; 2022; Shi, 2019; Wang et al., 2023; Wu, 2019; Yang, 2023; Yuan and Wu, 2016; Zhao et al., 2016) for hypertension: Most assessed in included RCTs, it demonstrated a significant add-on effect in lowering SBP and DBP in patients on antihypertensive medications but lacked alternative effects on blood pressure reduction (Figure 9).
• Jiangzhi Tongmai Capsule (Liu and Chen, 2011; Pan et al., 2016; Rao et al., 2015; Xiao, 2014; You, 2015) and Berberine (He et al., 2007; Kong et al., 2008; Su et al., 2012; Zhao et al., 2021; Zheng et al., 2009) for dyslipidaemia: Jiangzhi Tongmai Capsule showed an add-on effect on TG levels in participants treated with antihyperlipidaemic drugs (Figure 10). Berberine exhibited both alternative and add-on effects on TG levels, along with add-on effects on TC, HDL-C, and LDL-C levels (Figure 11).
• LZD (Ke et al., 2012; Liu et al., 2020; Yang, 2010) for obesity: Most assessed in included RCTs, it demonstrated an additive effect on BMI in participants with lifestyle modifications (Figure 12).

### 3.7 GRADE evidence quality

GRADE assessment of evidence quality for CHM in metabolic conditions ranged from very low to moderate. Across metabolic disorders, evidence certainty for adjunctive CHM plus conventional therapy was consistently higher than for CHM monotherapy, with obesity as the sole exception. For individual CHM formulae, evidnce certainty was very low to low, attributable to two trial profiles: small-scale, high-quality RCTs, or large-scale trials hampered by substantial methodological limitations. The full GRADE evidence profile was attached as Supplementary Table S8.

## 4 Discussion

This systematic review and meta-analysis synthesised evidence from 122 RCTs, of which 84 evaluated CHM as an adjunct therapy and 38 assessed CHM as monotherapy for chronic metabolic diseases. Unlike prior meta-analyses limited to specific interventions, this study provides the comprehensive assessment of a unified Chinese medicine principle—dampness resolution—across diverse disease contexts, including diabetes, hypertension, dyslipidaemia, and obesity. By examining frequently used herbs and formulations, we integrated fragmented evidence into a cohesive framework, revealing the therapeutic potential of dampness-resolving CHM in metabolic disease management.

### 4.1 Chinese herbal medicine for type 2 diabetes mellitus

In our study, the meta-analysis suggested that monotherapy with CHM significantly reduced FPG levels in T2DM, although heterogeneity in CHM formulations limits generalisability. For 2hPG, preliminary evidence suggests CHM monotherapy may be effective, but limited placebo-controlled trials hinder conclusive findings. Postprandial regulation is critical in T2DM, as fluctuations increase glycaemic burden and stress, causing β-cell dysfunction and complications, and impaired first-phase insulin response is an early T2DM feature (Shibib et al., 2024). Robust evidence for the role of CHM in 2hPG control requires standardised formulations and large-scale placebo-controlled trials. Overall, CHM shows promise as an adjunct therapy for glycaemic control. When combined with metformin, TCM demonstrates potential to enhance glycaemic control for both FPG and 2hPG. However, outcomes vary when CHM is paired with other antidiabetic agents, underscoring the complexity of TCM interactions in polypharmacy regimens.

The meta-analysis in our study also revealed that CHM might improve FINS levels when used in combination with lifestyle interventions, although evidence remains limited and inconclusive. CHM does not demonstrate superiority over conventional pharmacotherapy in this context. When added to existing pharmacotherapy in patients with diabetes, CHM may further enhance FINS improvement, with the strongest evidence supporting its combination with metformin. However, in patients already receiving insulin therapy, CHM appears to offer no additional benefit for FINS enhancement. The effect of CHM on FINS in the absence of lifestyle interventions remains uncertain owing to limited studies and variability in CHM formulations. Regardless of lifestyle adjustments, CHM appears to enhance the effects of pharmacotherapy on HOMA-IR. However, similar to FINS, CHM provides no additional benefit for HOMA-IR improvement in patients already treated with insulin. Elevated FINS level indicates β-cell compensation or failure, whereas elevated HOMA-IR is strongly associated with metabolic syndrome and early stages of T2DM (Muniyappa et al., 2008). The findings suggest that CHM may enhance glycaemic control by supporting β-cell function and improving insulin sensitivity, although its role in severe diabetes remains limited.

GQD, the most evaluated CHM for resolving dampness in included RCTs for diabetes, shows both alternative and add-on effects in reducing FPG, with add-on effects on post-treatment HOMA-IR and 2hPG levels in hypoglycaemic agent users, which aligned with a previous systematic review (Tan et al., 2023). Its effectiveness is dose-dependent, particularly regarding Coptidis rhizoma (Chinese pinyin: huanglian), as validated by recent RCTs (Kang et al., 2024). GQD lowers blood glucose levels by improving insulin resistance and enhancing insulin sensitivity (Gao et al., 2017; Lu et al., 2021). It modulates gut microbiota, enriching beneficial bacteria, such as Faecalibacterium, to reduce hyperglycaemia and inflammation (Gao et al., 2024). GQD contains active components, such as puerarin, baicalin, and berberine, which exhibit antioxidant, anti-inflammatory, and hypoglycaemic effects by targeting pathways, such as Nrf2 and PI3K/Akt (Lu et al., 2021; Xu et al., 2020). Additionally, GQD inhibits hepatic ferroptosis to reduce oxidative stress and improve iron metabolism and regulates the gut flora–bile acid–TGR5 axis, offering novel anti-diabetic mechanisms (Bao et al., 2022; Liu et al., 2024). These findings highlight GQD’s potential as a multifunctional agent in T2DM management, warranting further exploration of its clinical applications.

### 4.2 Chinese herbal medicine for hypertension

Extremely limited evidence from our meta-analysis suggested that CHM did not demonstrate superiority over placebo in reducing SBP when used as an adjunct to lifestyle modifications. Furthermore, CHM as a monotherapy was inferior to RAS inhibitors. However, TCM may augment the SBP-lowering effects of pharmacotherapy, particularly CCBs and RAS inhibitors, with more pronounced reductions observed when combined with lifestyle adjustments, achieving the minimal clinically important difference of 10 mmHg (Ettehad et al., 2016; Thomopoulos et al., 2014). Notably, CHM does not further reduce SBP in patients requiring dual antihypertensive therapy. These findings highlight the potential adjunctive role of CHM in SBP management but underscore its limitations as monotherapy or in combination with dual antihypertensive agents. DBP is closely tied to vascular tone and resistance in smaller arteries and arterioles (Tomiyama, 2023), and elevated DBP is more clinically relevant in younger populations, where vascular elasticity is better preserved (Bello et al., 2020). In our meta-analysis, we found that CHM demonstrated modest superiority over placebo in reducing DBP when combined with lifestyle modifications, and CHM consistently enhanced the DBP-lowering effects of pharmacotherapy, regardless of whether patients were receiving monotherapy or dual antihypertensive regimens. Notably, the additive effects of CHM on DBP highlight its potential to optimise antihypertensive management in patients with preserved vascular elasticity, or with impaired peripheral small arteries.

BBTD, the most evaluated CHM in included RCTs for hypertension, demonstrated a significant add-on effect in lowering SBP and DBP in patients on antihypertensive medications but lacked alternative effects on blood pressure reduction, being consistent with previous evidence (Mohammed et al., 2023). This can be explained by its multi-target and multi-pathway regulatory mechanisms. First, these mechanisms may be supported by its active components, such as flavonoids and triterpenoids, which act on key targets, such as AKT1, NOS3, and ACE, thereby influencing vascular tone and blood pressure regulation (Lin et al., 2022). Additionally, it promotes potassium efflux through potassium channels and inhibits calcium influx via voltage-operated calcium channels and intracellular calcium release from the sarcoplasmic reticulum (Tan et al., 2018). It also helps mitigate oxidative stress and inflammation, which are critical in hypertension pathogenesis. Specifically, BBTD enhances nitric oxide (NO) production via the NO/sGC/cGMP pathway and prostaglandin-I-2 synthesis, leading to vasodilation (Jin et al., 2024).

### 4.3 Chinese herbal medicine for dyslipidaemia

Previous evidence suggests that a 0.4 mmol/L increase in HDL-C level and a 1 mmol/L reduction in LDL-C and TG levels are associated with a lower risk of cardiovascular events (Emerging et al., 2009; Marston et al., 2019; Patel and Giugliano, 2020). In our meta-analysis, the effects of CHM for resolving dampness on TG, LDL-C, and HDL-C levels were inconclusive owing to the limited number of studies, inconsistent results, and modest effect sizes. Although CHM showed potential to enhance the effects of lipid-lowering drugs, its impact was insufficient to meaningfully alter cardiovascular risk. Notably, Jiangzhi Tongmai Capsule and Berberine, the most extensively studied proprietary CHM agents for dyslipidaemia, align with these observations. These findings highlight the need for further high-quality studies to clarify the role of CHM mainly targeting dampness syndrome in lipid management.

### 4.4 Chinese herbal medicine for obesity

Obesity measured using BMI was strongly associated with an increased risk of cardiovascular diseases (CVDs), CVD mortality, and all-cause mortality, and the risk was enhanced per 1 unit increase in BMI (Dwivedi et al., 2020). In our study, the meta-analysis suggested that CHM eliminating dampness outperformed placebo in reducing BMI while improving central obesity indicators, such as WC, requiring integration with lifestyle interventions.

LZD most assessed in included RCTs for obesity has demonstrated an additive effect on reducing BMI when combined with lifestyle modifications, such as dietary restriction and physical exercise. This synergistic effect is attributed to LZD’s multifaceted pharmacological mechanisms against obesity. LZD restores glucose homoeostasis and enhances insulin sensitivity while modulating serum lipid profiles and intestinal lipid content, particularly through significant alterations in diacylglycerol and monoacylglycerol levels (Li et al., 2024). Notably, LZD upregulates key thermogenesis-related factors, including uncoupling protein 1, PR domain containing 16, peroxisome proliferator-activated receptor gamma coactivator 1-alpha, and peroxisome proliferator-activated receptors alpha and gamma, in white adipose tissue (Li et al., 2024). This promotes adipose tissue ‘browning’ and increases energy expenditure. Animal studies further support LZD’s efficacy, showing that its combination with dietary restriction and exercise alleviates high-fat diet-induced metabolic complications, including obesity, hyperglycaemia, hyperlipidaemia, and insulin resistance, potentially through downregulation of tumor necrosis factor-alpha, leptin, and protein kinase B (Sun et al., 2022). Although these findings highlight LZD as a promising therapeutic strategy for obesity and metabolic health improvement, high-quality RCTs are required to validate its clinical efficacy and establish it as a viable adjunctive therapy.

### 4.5 Common herbs across multiple metabolic diseases

In the included RCTs, Poria (Chinese pinyin: fuling), derived from the sclerotia of *P. cocos (Schw.) Wolf.*, emerged as a consistently prominent component across multiple metabolic conditions. Overall, the multifaceted pharmacological profile of Poria cocos positions it as a valuable therapeutic agent for metabolic diseases. Its active components, including triterpenoids and polysaccharides, play pivotal roles in these therapeutic effects. Triterpenoids in Poria cocos restore glucose homeostasis and enhance insulin sensitivity, crucial for diabetes management (Guo et al., 2025). These compounds also modulate serum lipid profiles and intestinal lipid content, particularly influencing diacylglycerols and monoacylglycerols, thus aiding in dyslipidaemia treatment (Li et al., 2023). The polysaccharides in Poria cocos are notable for their role in gut microbiota modulation, which is closely associated with metabolic health benefits, including improved glucose metabolism and reduced inflammation associated with obesity (Lai et al., 2025). Additionally, Poria cocos exhibits anti-inflammatory and antioxidant properties, which help mitigate chronic inflammation, a key factor in obesity and related metabolic disorders (Li et al., 2022). Its diuretic activity contributes to hypertension management by promoting sodium and water excretion, reducing blood volume and pressure (Lee et al., 2012). Furthermore, Poria cocos may protect against obesity-induced renal damage through its antioxidant and anti-inflammatory effects (Nie et al., 2020). Although clinical evidence and pharmacological studies have demonstrated the benefits of Poria cocos for metabolic disorders, further studies are required to clarify its precise molecular mechanisms, particularly involving triterpenoids and polysaccharides. Additionally, investigating how Poria cocos modulates gut microbiota and its subsequent effects on metabolic health may reveal insights into its therapeutic potential.

### 4.6 Limitations

While most outcome measures in RCTs of metabolic diseases are objective (e.g., lipid and glucose profiles), which are unlikely to be influenced by blinding status, outcome assessors should still remain blinded for clinician-reported outcomes, such as blood pressure and BMI. This will ensures a greater precision in effect estimation. The existing evidence supports the potential benefits of dampness-eliminating CHM for metabolic diseases, but it does not exclude the possibility of supplementary effects when combined with other therapeutic principles in a multi-herbal formula. Vascular impairments and endpoint events were not reported in any included studies, thus the long-term benefits of CHM in individuals with metabolic diseases requires further investigation.

## 5 Conclusion

Dampness-eliminating CHM may serve as a complementary therapy for metabolic diseases such as hypertension and diabetes. Further high-quality RCTs are required to confirm its role in dyslipidaemia and identify the most effective CHM formulae for obesity.

## Data Availability

The original contributions presented in the study are included in the article/Supplementary Material, further inquiries can be directed to the corresponding author.

## Glossary

24-h ABPM: 24-h ambulatory blood pressure monitoring
2hPG: 2-h postprandial blood glucose
BBTD: Banxia Baizhu Tianma Decoction
BMI: Body mass index
CBM: China Biology Medicine
CCBs: Calcium channel blockers
CHM: Chinese herbal medicine
CI: Confidence intervals
CNKI: China National Knowledge Infrastructure
CVD: Cardiovascular diseases
DBP: Diastolic blood pressure
FINS: Fasting insulin
FPG: Fasting plasma glucose
GBD: Global Burden of Diseases
GQD: Gegen Qinlian Decoction
HC: Hip circumference
HDL-C: High-density lipoprotein cholesterol
HOMA-IR: Homeostasis model assessment of insulin resistance
LDL-C: low-density lipoprotein cholesterol
LZD: Linggui Zhugan Decoction
MCID: Minimal clinically important difference
MD: Mean differences
NO: Nitric oxide
PGC-1α: Peroxisome proliferator-activated receptor gamma coactivator 1-alpha
PGI2: Prostaglandin-I-2
PKB: Protein kinase B
PPARα and PPARγ: Peroxisome proliferator-activated receptors alpha and gamma
PRDM16: PR domain containing 16
PRISMA: Preferred Reporting Items for Systematic Reviews and Meta-Analyses
PROSPERO: Prospective Register of Systematic Reviews
RAS: Renin-angiotensin system
RCT: Randomized controlled trial
RR: Relative risk
SBP: Systolic blood pressure
T2DM: Type 2 diabetes mellitus
TC: Total cholesterol
TCM: Traditional Chinese medicine
TG: Triglycerides
TNF-α: Tumor necrosis factor-alpha
UCP1: Uncoupling protein 1
WC: Waist circumference
WHR: Waist-to-hip ratio
