# Supplementary material for: Efficacy and safety of Chinese herbal medicine for metabolic conditions: a systematic review and meta-analysis of randomised controlled trials
Source: Front Pharmacol. 2026 Jan 2;16:1644950. doi: 10.3389/fphar.2025.1644950 (PMC12808482; doi:10.3389/fphar.2025.1644950)
Supplement: Supplementary file 1 [file Table1.pdf]

Table S1. Search strategy

Chinese
#1 “系统评价” OR “Meta分析” OR “随机对照试验” OR “非随机对照试验” OR “队列研究” OR “病例对照研究” OR “病例报告” OR “单病例研究” OR “中西医结合”<br>#2 “中医学” OR “中草药” OR “湿” OR “中成药” OR “中医” OR “辨证” OR “中草药” OR “中药” OR “中成药” OR “方剂” OR “方药” OR “复方” OR “汤剂”<br>#3 “高血压” OR “血脂异常” OR “高脂血症” OR “高胆固醇血症” OR “高甘油三酯血症” OR “睡眠呼吸暂停,阻塞性” OR “肥胖症” OR “超重” OR “代谢综合征X” OR “睡眠呼吸暂停综合征” OR “睡眠呼吸暂停,中枢、” OR “糖尿病” OR “心房颤动”<br>#1 AND #2 AND #3
English
#1 "Systematic Review"[MeSH Terms] OR "Meta-Analysis"[MeSH Terms] OR "Randomized Controlled Trial"[MeSH Terms] OR "Non-Randomized Controlled Trials"[MeSH Terms] OR "Cohort Studies"[MeSH Terms] OR "cohort analysis"[MeSH Terms] OR "Case-Control Studies"[MeSH Terms] OR "case control study"[MeSH Terms] OR "Case Reports"[MeSH Terms] OR "case study"[MeSH Terms])<br>#2 "Integrative Medicine"[All Fields] OR "integrated traditional chinese and western medicine"[All Fields] OR "combination of traditional chinese and western medicine"[All Fields] OR "integrated chinese and western medicine"[All Fields] OR "traditional chinese medicine"[All Fields] OR "Medicine, Chinese Traditional"[MeSH Terms] OR "Medicine, Traditional"[All Fields] OR "Chinese Herbal"[MeSH Terms] OR "Traditional Medicine, Chinese"[All Fields] OR "Chinese Traditional Medicine"[All Fields] OR "Chinese Medicine Herb"[All Fields] OR "Chinese herb"[MeSH Terms] OR "Chinese medicine"[MeSH Terms] OR "Chinese Herbal Drugs"[All Fields] OR "Chinese Drugs, Plant"[All Fields] OR "Herbal Drugs, Chinese"[All Fields] OR "chinese herbal medicine"[All Fields] OR "chinese patent medicines"[All Fields] OR "Zhong Yi Xue"[All Fields] OR "Chinese Traditional Medicine"[All Fields] OR "Chinese Medicine, Traditional"[All Fields] OR "Traditional Tongue Diagnosis"[All Fields] OR "Tongue Diagnoses, Traditional"[All Fields] OR "Tongue Diagnosis, Traditional"[All Fields] OR "Traditional Tongue Diagnoses"[All Fields] OR "Traditional Tongue Assessment"[All Fields] OR "Tongue Assessment, Traditional"[All Fields] OR "Traditional Tongue Assessments"[All Fields] OR "Ethnopharmacology"[MeSH Terms] OR "TCM"[All Fields] OR "Medicine, Ayurvedic"[All Fields] OR "Herbology"[All Fields] OR "Plants, Medicinal"[All Fields] OR "Plant Preparation"[All Fields] OR "Plant Extract"[MeSH Terms] OR "Plants, Medicine"[MeSH Terms] OR "Materia Medica"[MeSH Terms] OR "Single Prescription"[All Fields] OR "Herbs"[All Fields] OR "Ethnomedicine"[All Fields] OR "Ethnobotany"[MeSH Terms] OR "Phytotherapy"[MeSH Terms] OR "chinese herbal compound"[All Fields] OR "traditional chinese medicine decoction"[All Fields] OR "Chinese Drug"[MeSH Terms] OR "medicinal plant"[MeSH Terms] OR "traditional medicine"[MeSH Terms]<br>#3 "hypertension"[MeSH Terms] OR "dyslipidemias"[MeSH Terms] OR "hyperlipidemias"[MeSH Terms] OR "hypercholesterolemia"[MeSH Terms] OR "hypertriglyceridemia"[MeSH Terms] OR "carotid stenosis"[MeSH Terms] OR "obesity"[MeSH Terms] OR "adiposity"[MeSH Terms] OR "overweight"[MeSH Terms] OR "metabolic

syndrome"[MeSH Terms] OR "homocysteine"[MeSH Terms] OR "sleep apnea syndromes"[MeSH Terms] OR "diabetes mellitus"[MeSH Terms] OR "diabetes insipidus"[MeSH Terms] OR "atrial fibrillation"[MeSH Terms] OR "hypertense"[All Fields] OR "hypertension"[All Fields] OR "hypertension's"[All Fields] OR "hypertensions"[All Fields] OR "hypertensive"[All Fields] OR "hypertensive's"[All Fields] OR "hypertensives"[All Fields] OR "dyslipidaemias"[All Fields] OR "dyslipidemias"[All Fields] OR "dyslipidaemia"[All Fields] OR "dyslipidemia"[All Fields] OR "dyslipidaemias"[All Fields] OR "dyslipidemias"[All Fields] OR "dyslipidaemia"[All Fields] OR "dyslipidemia"[All Fields] OR "hyperlipidaemia"[All Fields] OR "hyperlipidemias"[MeSH Terms] OR "hyperlipidemias"[All Fields] OR "hyperlipidemia"[All Fields] OR "hyperlipidaemias"[All Fields] OR "hypercholesterolaemia"[All Fields] OR "hypercholesterolemia"[All Fields] OR "hypercholesterolaemias"[All Fields] OR "hypercholesterolemias"[All Fields] OR "hypertriglyceridaemia"[All Fields] OR "hypertriglyceridemia"[All Fields] OR "hypertriglyceridaemias"[All Fields] OR "hypertriglyceridemias"[All Fields] OR "carotid stenosis"[All Fields] OR "carotid artery stenosis"[All Fields] OR "obeses"[All Fields] OR "obesity"[All Fields] OR "obese"[All Fields] OR "obesities"[All Fields] OR "obesity's"[All Fields] OR "adiposity"[All Fields] OR "adiposis"[All Fields] OR "overweight"[All Fields] OR "overweighted"[All Fields] OR "overweightness"[All Fields] OR "overweights"[All Fields] OR "metabolic syndrome"[All Fields] OR "homocystein"[All Fields] OR "homocysteine"[All Fields] OR "homocysteine's"[All Fields] OR "homocysteines"[All Fields] OR "sleep apnoea syndrome"[All Fields] OR "sleep apnea syndromes"[All Fields] OR "sleep apnea syndrome"[All Fields] OR "diabete"[All Fields] OR "diabetes mellitus"[All Fields] OR "diabetes"[All Fields] OR "diabetes insipidus"[All Fields] OR "diabetic"[All Fields] OR "diabetics"[All Fields] OR "diabets"[All Fields] OR "atrial fibrillation"[All Fields] OR "auricular fibrillation"[All Fields]
#1 AND #2 AND #3

Table S2. The list of excluded references

ID	Reference	Reasons for exclusion
0001	Derosa G, Bonaventura A, Bianchi L, et al. Effects of Berberis aristata/Silybum marianum association on metabolic parameters and adipocytokines in overweight dyslipidemic patients. J Biol Regul Homeost Agents. 2013;27(3):717-728.	Not related to Oral CHM
0002	Rocha S, Ribeiro D, Fernandes E, Freitas M. A Systematic Review on Anti-diabetic Properties of Chalcones. Curr Med Chem. 2020;27(14):2257-2321. doi:10.2174/0929867325666181001112226	Not related to Oral CHM
0003	Silva GE, Takahashi MH, Eik Filho W, et al. Ausência de efeito hipolipemiante da Solanum melongena L. (berinjela) em pacientes hiperlipidêmicos [Absence of hypolipidemic effect of Solanum melongena L. (eggplant) on hyperlipidemic patients]. Arq Bras Endocrinol Metabol. 2004;48(3):368-373. doi:10.1590/s0004-27302004000300006	Not related to Oral CHM
0004	Razmpoosh E, Mirmiran P, Safi S, et al. Black seed oil supplement had positive effects on blood concentration and mRNA expression levels of estrogen and SHBG in premenopausal women with overweight and obesity: a crossover, double blind, placebo controlled randomized clinical trial[J]. European Journal of Cancer, 2022, 175: S42.	Not related to Oral CHM
0005	Singh B, Sujatha N, Sharma M C. Clinical evaluation of shilajatu (Asphaltum punjabinum), kutaki (Picrorhiza kurroa) and khadir (Acacia catechu) in the management of sthaulya (obesity)[J]. Int J Res Ayurveda Pharm, 2013, 4(4): 503-506.	Not related to Oral CHM
0006	Shen L, Gwak SR, Joo JC, et al. Effectiveness and Safety of Hwangchil-Unripe Bokbunja Extract Mixture on Blood Pressure: A Randomized Double-Blind Placebo-Controlled Clinical Trial. J Med Food. 2021;24(3):258-266. doi:10.1089/jmf.2020.4820	Not related to Oral CHM
0007	Nie XM, Zhao YX, Shi DM, et al. Zhonghua Yi Xue Za Zhi. 2013;93(26):2052-2055.	Not related to Oral CHM
0008	Pu Z, Sun Y, Jiang H, et al. Effects of Berberine on Gut Microbiota in Patients with Mild Metabolic Disorders Induced by Olanzapine. Am J Chin Med. 2021;49(8):1949-1963. doi:10.1142/S0192415X21500920	Not related to Oral CHM
0009	Sun X, Zhao H, Wang R, et al. Psoriasis complicated with metabolic disorder is associated with traditional Chinese medicine syndrome types: a hospital-based retrospective case-control study. Curr Med Res Opin. 2023;39(1):19-25. doi:10.1080/03007995.2022.2129803	Not related to Oral CHM
0010	Ooi CP, Loke SC. Sweet potato for type 2 diabetes mellitus. Cochrane Database Syst Rev. 2013;2013(9):CD009128. Published 2013 Sep 3. doi:10.1002/14651858.CD009128.pub3	Not related to Oral CHM

0011	Tavana A, Pourrajab F, Hekmatimoghaddam S H, et al. The hypoglycemic effect of Dorema aucheri (bilhar) extract in diabetic type 2 patients: A first clinical trial[J]. International Journal of Pharmaceutical and Clinical Research, 2015, 7(5): 343-347.	Not related to Oral CHM
0012	Xing XM, Wang RC, Sun QW, Li H. Zhongguo Zhen Jiu. 2011;31(4):301-304.	Not related to Oral CHM
0013	Xie B, Lin YP. Zhongguo Zhen Jiu. 2014;34(6):547-550.	Not related to Oral CHM
0014	Zheng LY, Yang LL, Li LR, et al. Zhongguo Zhong Xi Yi Jie He Za Zhi. 2013;33(7):920-923.	Not related to Oral CHM
0015	Xiong W, Liu JG, Li H. Zhongguo Zhong Xi Yi Jie He Za Zhi. 2013;33(11):1462-1467.	Not related to Oral CHM
0016	Qiu X, Wang KX, Chen GH. Zhongguo Zhong Xi Yi Jie He Za Zhi. 2011;31(11):1483-1486.	Not related to Oral CHM
0017	Zhang GD, Zou BL, Meng H. Zhongguo Zhong Xi Yi Jie He Za Zhi. 2010;30(9):915-918.	Not related to Oral CHM
0018	Yang CH, Lin JM, Xie J. Zhongguo Zhong Xi Yi Jie He Za Zhi. 2012;32(9):1204-1207.	Not related to Oral CHM
0019	Yin H, Yan X, Yang KH. Zhongguo Zhong Xi Yi Jie He Za Zhi. 2009;29(11):970-974.	Not related to Oral CHM
0020	Wu Z, Wu L, Dai X, et al. Nan Fang Yi Ke Da Xue Xue Bao. 2020;40(7):1044-1048. doi:10.12122/j.issn.1673-4254.2020.07.20	Not related to Oral CHM
0021	Shi L, Zhao GC. Zhongguo Gu Shang. 2019;32(6):574-577. doi:10.3969/j.issn.1003-0034.2019.06.018	Not related to Oral CHM
0022	Whitaker J, Steiger N, Romero J, et al. 10-year single center experience of catheter ablation of focal atrial tachycardia. J Interv Card Electrophysiol. 2023;66(5):1135-1144. doi:10.1007/s10840-022-01416-5	Not related to Oral CHM
0023	王传池,许伟明,江丽杰,等.11383 例健康人群及冠心病不同阶段患者痰瘀互结证分布规律的多中心横断面研究[J].中医杂志 ,2021,62(06):494-504.DOI:10.13288/j.11-2166/r.2021.06.009.	Not related to Oral CHM
0024	殷玲,何伟明,刘利华,等.122 例腹膜透析患者中医证型及其与腹膜炎及心血管事件相关性研究[J].江苏中医药,2017,49(02):36-38.	Not related to Oral CHM
0025	汪惠勇.124 例原发性高血压病中医证型分布规律的临床研究[J].中国民族民间医药,2015,24(08):111.	Not related to Oral CHM
0026	尹艳,王世燕,孙志新,等.1554 例超重/肥胖人群的中医体质类型分析及临床防治思路探索[J].中医药信息 ,2020,37(02):64-69.DOI:10.19656/j.cnki.1002-2406.200046	Not related to Oral CHM
0027	原萌谦,刘志诚,徐斌.1584 例不同性别肥胖并发高脂血症患者证候证素特点的临床研究[J].时珍国医国药,2016,27(09):2178-2180.	Not related to Oral CHM
0028	倪量,王融冰,段呈玉,等.180 例HAART致高脂血症的中医证候特点研究[C]//中国中西医结合学会传染病专业委员会.全国第 4 届中西医结合传染病学术会议论文汇编.北京地坛医院感染病诊疗中心;云南省中医中药研究院;河南中医学院第一附属医院;广州市第八人民医院;北京佑安医院;中南大学湘雅二医院;安徽省中医院;北京中医药大学;,2012:106.	Not related to Oral CHM

0029	赵荣.214 例老年H型高血压患者中医体质分型观察体会[J].光明中医,2016,31(02):165-167.	Not related to Oral CHM
0030	杨瑛笛,孙晓峰,丘文君.220 例妊娠期糖尿病患者中医临床证型调查研究[J].医药前沿,2016,6(22):365-366.	Not related to Oral CHM
0031	赵国青,徐雯,周腊梅.251 例急性痛风患者临床特点[J].世界最新医学信息文摘,2019,19(A4):51-52.DOI:10.19613/j.cnki.1671-3141.2019.104.027	Not related to Oral CHM
0032	牟新,周旦阳,赵进喜.265 例糖尿病肾病肾功能不全患者中医证候与实验室指标的典型相关性研究[J].药品评价,2009,6(04):153.	Not related to Oral CHM
0033	王俊亚.2 型糖尿病的中医证型分析及其与糖尿病慢性并发症的关系[J].中国卫生产业,2012,9(27):164.DOI:10.16659/j.cnki.1672-5654.2012.27.038	Not related to Oral CHM
0034	邢利旋,陈蔓,林丽莉,等.2 型糖尿病合并代谢相关脂肪性肝病危险因素与中医证型分布研究[J].山西中医,2021,37(08):48-51.	Not related to Oral CHM
0035	郑开颜.300 例社区中老年高血压患者的中医体质辨识与分析[J].中国现代药物应用,2017,11(01):193-194.DOI:10.14164/j.cnki.cn11-5581/r.2017.01.091	Not related to Oral CHM
0036	Razmpoosh, Elham & Mirmiran, P. & Safi, S. & Nadjarzadeh, Azadeh & Nazari, Majid & Meyre, D.. (2022). Black seed oil supplement had positive effects on blood concentration and mRNA expression levels of estrogen and SHBG in premenopausal women with overweight and obesity: a crossover, double blind, placebo controlled randomized clinical trial. European Journal of Cancer. 175. S42. 10.1016/S0959-8049(22)01462-9.	Not related to Oral CHM
0037	Pan CY, Gao Y, Li GW, Zhu XX, Gao X, Liu X. Zhonghua Nei Ke Za Zhi. 2009;48(4):304-307.	Not related to Oral CHM
0038	Oz M, Lorke DE, Kabbani N. A comprehensive guide to the pharmacologic regulation of angiotensin converting enzyme 2 (ACE2), the SARS-CoV-2 entry receptor. Pharmacol Ther. 2021;221:107750. doi:10.1016/j.pharmthera.2020.107750	Not related to Oral CHM
0039	Phimarn W, Paktipat P, Taengthonglang C, et al. A meta-analysis and meta-regression study of the effects of Triphala on anthropometric parameters[J]. Journal of Herbmed Pharmacology, 2022, 11(4): 475-482.	Not related to Oral CHM
0040	钟锦卫, 梁国庆, 李朋, 戴继灿, 徐迪萍, 平萍, & 李铮. (2013). 西地那非联合十一酸睾酮治疗糖尿病性勃起功能障碍的安全性有效性多中心研究. 中国男科学杂志, (4), 26-29.	Not related to Oral CHM
0041	Zhang J, Shao Y, Liu Y, Tao J. A Multi-Center, Open-Label, Two-Arm Parallel Group Non-inferiority Randomized Controlled Trial Evaluating the Effect of Pitavastatin, Compared to Atorvastatin, on Glucose Metabolism in Prediabetics with Hypertension and Dyslipidemia: Rationale and Design for the China Hemoglobin A1c Metabolism Protection Union Study (CAMPUS). Cardiovasc Drugs Ther. 2018;32(6):581-589. doi:10.1007/s10557-018-6826-6	Not related to Oral CHM

004<br>2	Tutino GE, Yang WY, Li X, et al. A multicentre demonstration project to evaluate the effectiveness and acceptability of the web-based Joint Asia Diabetes Evaluation (JADE) programme with or without nurse support in Chinese patients with Type 2 diabetes. Diabet Med. 2017;34(3):440-450. doi:10.1111/dme.13164	Not related to Oral CHM
004<br>3	Zerm, Roland & Helbrecht, Bert & Pranga, Danilo & Brinkhaus, Benno & Michalsen, Andreas & Kröz, M.. (2017). A multimodal therapy concept for lifestyle optimization in Type 2 Diabetes - design and methodology of the AIM-DIABETES study. 17 (Suppl 1). 117,118 (P 206).	Not related to Oral CHM
004<br>4	Ye D, Huang Y, Zhou F, et al. A phase 3, double-blind, randomized placebo-controlled efficacy and safety study of abiraterone acetate in chemotherapy-naïve patients with mCRPC in China, Malaysia, Thailand and Russia. Asian J Urol. 2017;4(2):75-85. doi:10.1016/j.ajur.2017.01.002	Not related to Oral CHM
004<br>5	Zhang H, Liu J, Zhu X, et al. A Phase I Study on the Pharmacokinetics and Pharmacodynamics of DJT1116PG, a Novel Selective Inhibitor of Sodium-glucose Cotransporter Type 2, in Healthy Individuals at Steady State. Clin Ther. 2020;42(5):892-905.e3. doi:10.1016/j.clinthera.2020.03.007IF: 3.2 Q2	Not related to Oral CHM
004<br>6	Zhang P, Lan X, Fan B, et al. A protocol for the integration of multi-omics bioinformatics: Mechanism of acupuncture as an adjunctive therapy for alcohol use disorder. Front Neurol. 2023;13:977487. Published 2023 Jan 5. doi:10.3389/fneur.2022.977487IF: 2.7 Q2	Not related to Oral CHM
004<br>7	Zeng X, Li Y, Lu L, Wen H, Wang G, Zuo C. A randomized controlled clinical study on Zuo's acupuncture treatment for prediabetes. Medicine (Baltimore). 2022;101(8):e28824. doi:10.1097/MD.0000000000028824	Not related to Oral CHM
004<br>8	Park JE, Liu Y, Park T, et al. A trial for the use of qigong in the treatment of pre and mild essential hypertension: a study protocol for a randomized controlled trial. Trials. 2011;12:244. Published 2011 Nov 21. doi:10.1186/1745-6215-12-244	Not related to Oral CHM
004<br>9	Wang C, Gordon ES, Stack CB, et al. A randomized trial of the clinical utility of genetic testing for obesity: design and implementation considerations. Clin Trials. 2014;11(1):102-113. doi:10.1177/1740774513508029	Not related to Oral CHM
005<br>0	Yu WC, Chen CH, Tsao HM, Ding YA. A randomized, double-blind comparison of cerivastatin and lovastatin for treatment of primary hypercholesterolemia. Zhonghua Yi Xue Za Zhi (Taipei). 2002;65(6):260-267.	Not related to Oral CHM
005<br>1	Zhang Q, Zhou R, Yang J, et al. A Randomized, Double-Blind, Placebo-Controlled, First-in-Human Clinical Trial to Assess Safety, Tolerability, and Pharmacokinetics of LY-CovMab, a Potent Human Neutralizing Antibody Against SARS-CoV-2. Infect Dis Ther. 2022;11(1):405-422. doi:10.1007/s40121-021-00572-xIF: 4.7 Q1	Not related to Oral CHM

0052	Weng CS, Hung YL, Shyu LY, Chang YH. A study of electrical conductance of meridian in the obese during weight reduction. Am J Chin Med. 2004;32(3):417-425. doi:10.1142/S0192415X04002077	Not related to Oral CHM
0053	Tai ES, Fok AC, Chu R, Tan CE. A study to assess the effect of dietary supplementation with soluble fibre (Minolest) on lipid levels in normal subjects with hypercholesterolaemia. Ann Acad Med Singap. 1999;28(2):209-213.	Not related to Oral CHM
0054	Miraj, Sepideh. (2016). A systematic review on the Heracleum persicum effect and efficacy profiles. 8. 140-142.	Not related to Oral CHM
0055	Sedigh-Rahimabadi M, Fani M, Rostami-Chijan M, Zarshenas MM, Shams M. A Traditional Mouthwash (Punica granatum var pleniflora) for Controlling Gingivitis of Diabetic Patients: A Double-Blind Randomized Controlled Clinical Trial. J Evid Based Complementary Altern Med. 2017;22(1):59-67. doi:10.1177/2156587216633370	Not related to Oral CHM
0056	Zhou X, Chen C, Yin D, et al. A Variation in the ABCC8 Gene Is Associated with Type 2 Diabetes Mellitus and Repaglinide Efficacy in Chinese Type 2 Diabetes Mellitus Patients. Intern Med. 2019;58(16):2341-2347. doi:10.2169/internalmedicine.2133-18	Not related to Oral CHM
0057	Tomlinson B, Hu M, Lee VW, et al. ABCG2 polymorphism is associated with the low-density lipoprotein cholesterol response to rosuvastatin. Clin Pharmacol Ther. 2010;87(5):558-562. doi:10.1038/clpt.2009.232	Not related to Oral CHM
0058	Song L, Kong X, Yang Z, et al. Acarbose Reduces Low-Grade Albuminuria Compared to Metformin in Chinese Patients with Newly Diagnosed Type 2 Diabetes. Diabetes Metab Syndr Obes. 2021;14:4451-4458. Published 2021 Nov 5. doi:10.2147/DMSO.S325683	Not related to Oral CHM
0059	Meyer-Hamme G, Friedemann T, Greten HJ, Plaetke R, Gerloff C, Schroeder S. ACUDIN - ACUpuncture and laser acupuncture for treatment of DIabetic peripheral Neuropathy: a randomized, placebo-controlled, partially double-blinded trial. BMC Neurol. 2018;18(1):40. Published 2018 Apr 13. doi:10.1186/s12883-018-1037-0	Not related to Oral CHM
0060	Wu X, Mo Q, He T, Zhi N, Huang Y, Yang S. Acupoint catgut embedding for the treatment of obesity in adults: A systematic review protocol. Medicine (Baltimore). 2019;98(8):e14610. doi:10.1097/MD.0000000000014610	Not related to Oral CHM
0061	Zhang Y, Gong H, Zhan B, Chen S. Acupoint Catgut Embedding Reduces Insulin Resistance in Diabetic Patients Undergoing Open Cardiac Surgery. Heart Surg Forum. 2021;24(1):E060-E064. Published 2021 Jan 20. doi:10.1532/hsf.3331	Not related to Oral CHM
0062	Ni YM, Frishman WH. Acupuncture and Cardiovascular Disease: Focus on Heart Failure. Cardiol Rev. 2018;26(2):93-98. doi:10.1097/CRD.0000000000000179	Not related to Oral CHM
0063	Shu W, Ran JC, Chen BL, Li CN, Ruan SQ, Hou WG. Zhongguo Zhen Jiu. 2021;41(8):866-870. doi:10.13703/j.0255-2930.20200720-0007	Not related to Oral CHM
0064	Wang JY, Zhang J, Liu Y, Gao YM, Sun JJ, Liu XM. Zhongguo Zhen Jiu. 2021;41(4):371-375. doi:10.13703/j.0255-2930.20200329-k0001	Not related to Oral CHM

006 5	Wang SH, Xu JT, Hu XJ, Cui J. Acupuncture combined with western medicine for the treatment of hypertension: A protocol for an updated systematic review and meta-analysis. Medicine (Baltimore). 2021;100(25):e26412. doi:10.1097/MD.0000000000026412	Not related to Oral CHM
006 6	Wu MY, Huang MC, Liao HH, et al. Acupuncture decreased the risk of coronary heart disease in patients with rheumatoid arthritis in Taiwan: a Nationwide propensity score-matched study. BMC Complement Altern Med. 2018;18(1):341. Published 2018 Dec 22. doi:10.1186/s12906-018-2384-5	Not related to Oral CHM
006 7	Xu M, Li D, Zhang S. Acupuncture for acute stroke. Cochrane Database Syst Rev. 2018;3(3):CD003317. Published 2018 Mar 30. doi:10.1002/14651858.CD003317.pub3	Not related to Oral CHM
006 8	Yang J, Chen J, Yang M, Yu S, Ying L, Liu GJ, Ren YL, Wright JM, Liang FR. Acupuncture for hypertension. Cochrane Database Syst Rev. 2018 Nov 14;11(11):CD008821. doi: 10.1002/14651858.CD008821.pub2IF: 8.8 Q1 . PMID: 30480757IF: 8.8 Q1 ; PMCID: PMC6516840IF: 8.8 Q1 .	Not related to Oral CHM
006 9	Wang X, Wang P, Liu C, et al. Acupuncture for hypertension with insomnia: Study protocol for a randomized, sham-controlled, subject-and-assessor-blinded trial. Front Psychiatry. 2022;13:1087706. Published 2022 Dec 22. doi:10.3389/fpsyt.2022.1087706	Not related to Oral CHM
007 0	Tian Y, Wang L, Xu T, et al. Acupuncture for Osteoporosis: a Review of Its Clinical and Preclinical Studies. J Acupunct Meridian Stud. 2022;15(5):281-299. doi:10.51507/j.jams.2022.15.5.281	Not related to Oral CHM
007 1	Xie XC, Cao YQ, Gao Q, Wang C, Li M, Wei SG. Acupuncture Improves Intestinal Absorption of Iron in Iron-deficient Obese Patients: A Randomized Controlled Preliminary Trial. Chin Med J (Engl). 2017;130(5):508-515. doi:10.4103/0366-6999.200549	Not related to Oral CHM
007 2	Rotbain Curovic V, Houlind MB, Hansen TW, et al. Acute and Long-Term Treatment With Dapagliflozin and Association With Serum Soluble Urokinase Plasminogen Activator Receptor. Front Pharmacol. 2022;13:799915. Published 2022 Apr 27. doi:10.3389/fphar.2022.799915	Not related to Oral CHM
007 3	Yu H, Lin S, Jin L, et al. Adenine/cytosine(1166) polymorphism of the angiotensin II type 1 receptor gene and the antihypertensive response to angiotensin-converting enzyme inhibitors. J Hypertens. 2009;27(11):2278-2282. doi:10.1097/HJH.0b013e328330b654	Not related to Oral CHM
007 4	Wang AL, Zhang H, Zhang J, et al. Adjuvant Effects of Health Education of Chinese Medicine for Chronic Diseases: A Systematic Review and Meta-Analysis of Randomized Controlled Trials. Evid Based Complement Alternat Med. 2020;2020:3738753. Published 2020 Mar 31. doi:10.1155/2020/3738753	Not related to Oral CHM
007 5	Rigby MR, Harris KM, Pinckney A, et al. Alefacept provides sustained clinical and immunological effects in new-onset type 1 diabetes patients. J Clin Invest. 2015;125(8):3285-3296. doi:10.1172/JCI81722	Not related to Oral CHM

0076	Riley RW, Powell NB, Li KK, Weaver EM, Guilleminault C. An adjunctive method of radiofrequency volumetric tissue reduction of the tongue for OSAS. Otolaryngol Head Neck Surg. 2003;129(1):37-42. doi:10.1016/S0194-59980300482-0	Not related to Oral CHM
0077	Riazurrehman M, Iqbal A, Ayaz S, et al. An alternative approach to treat obesity with leptogenic polyherbal formulation obesecure: A randomized clinical trial study. Pak J Pharm Sci. 2020;33(5(Special)):2423-2430.	Not related to Oral CHM
0078	Wang KY, Ou Y, Liu CX, Zhang JH, Dai XY, Gao R. Zhongguo Zhong Yao Za Zhi. 2021;46(15):4008-4015. doi:10.19540/j.cnki.cjcmm.20201218.501	Not related to Oral CHM
0079	Yuqi, L, Li, J, Liang, S, Shuyu, Q and Yaxi, S, 2020. Analysis of the randomized controlled trials of Chinese medicine non-drug therapy for essential hypertension based on the comparison between 2 research institutions in the community and hospital, Global Advances in Health and Medicine.	Not related to Oral CHM
0080	Um JY, Joo JC, Kim KY, An NH, Lee KM, Kim HM. Angiotensin converting enzyme gene polymorphism and traditional Sasang classification in Koreans with cerebral infarction. Hereditas. 2003;138(3):166-171. doi:10.1034/j.1601-5223.2003.01605.x	Not related to Oral CHM
0081	Zhao Y, Wang J, Ballevre O, Luo H, Zhang W. Antihypertensive effects and mechanisms of chlorogenic acids. Hypertens Res. 2012;35(4):370-374. doi:10.1038/hr.2011.195	Not related to Oral CHM
0082	Penrod NM, Moore JH. Antihypertensive effects of yoga in a general patient population: real-world evidence from electronic health records, a retrospective case-control study. BMC Public Health. 2022;22(1):186. Published 2022 Jan 27. doi:10.1186/s12889-022-12569-3	Not related to Oral CHM
0083	Mirahmad M, Mohseni S, Tabatabaei-Malazy O, et al. Antioxidative hypoglycemic herbal medicines with in vivo and in vitro activity against C-reactive protein; a systematic review. Phytomedicine. 2023;109:154615. doi:10.1016/j.phymed.2022.154615	Not related to Oral CHM
0084	Rondanelli M, Giacosa A, Orsini F, Opizzi A, Villani S. Appetite control and glycaemia reduction in overweight subjects treated with a combination of two highly standardized extracts from Phaseolus vulgaris and Cynara scolymus. Phytother Res. 2011;25(9):1275-1282. doi:10.1002/ptr.3425	Not related to Oral CHM
0085	Zhang RF, Ma CM, Wang N, et al. Appropriate intraprocedural initial heparin dosing in patients undergoing catheter ablation for atrial fibrillation receiving uninterrupted non-vitamin-K antagonist oral anticoagulant treatment. BMC Cardiovasc Disord. 2021;21(1):214. Published 2021 Apr 27. doi:10.1186/s12872-021-02032-3	Not related to Oral CHM
0086	Zhang H, Bian Z, Lin Z. Are acupoints specific for diseases? A systematic review of the randomized controlled trials with sham acupuncture controls. Chin Med. 2010;5:1. Published 2010 Jan 12. doi:10.1186/1749-8546-5-1	Not related to Oral CHM

0087	Wang QX, Xue J, Shi MJ, et al. Association Between Metabolic Dysfunction-Associated Fatty Liver Disease and the Risk of Cirrhosis in Patients with Chronic Hepatitis B-A Retrospective Cohort Study. Diabetes Metab Syndr Obes. 2022;15:2311-2322. Published 2022 Aug 2. doi:10.2147/DMSO.S369824	Not related to Oral CHM
0088	Sui M, Xue L, Ying X. Association of Acupuncture Treatment with Mortality of Type 2 Diabetes in China: Evidence of a Real-World Study. Int J Environ Res Public Health. 2020;17(21):7801. Published 2020 Oct 25. doi:10.3390/ijerph17217801	Not related to Oral CHM
0089	You H, Zhang T, Feng W, Gai Y. Association of TCM body constitution with insulin resistance and risk of diabetes in impaired glucose regulation patients. BMC Complement Altern Med. 2017;17(1):459. Published 2017 Sep 11. doi:10.1186/s12906-017-1964-0	Not related to Oral CHM
0090	Nie H, Wang F, Zhang Y, et al. Associations of serum bisphenol A levels with incident chronic kidney disease risk. Sci Total Environ. 2021;771:145401. doi:10.1016/j.scitotenv.2021.145401	Not related to Oral CHM
0091	Zhao ZH, Zhou Y, Li WH, Tang ZH, Xia TW, Han-Li. Auricular Acupressure in Patients with Hypertension and Insomnia: A Systematic Review and Meta-Analysis. Evid Based Complement Alternat Med. 2020;2020:7279486. Published 2020 Jun 17. doi:10.1155/2020/7279486	Not related to Oral CHM
0092	Mamtani R, Mamtani R. Ayurveda and yoga in cardiovascular diseases. Cardiol Rev. 2005;13(3):155-162.	Not related to Oral CHM
0093	Thomas MC. Bardoxolone: augmenting the Yin in chronic kidney disease. Diab Vasc Dis Res. 2011;8(4):303-304. doi:10.1177/1479164111421034	Not related to Oral CHM
0094	Zhu L, Tang Q, Zhang L, et al. Based on voxel-based morphological analysis to investigate the effect of acupuncture-rehabilitation therapy on hippocampal volume and its neuroprotective mechanism in patients with vascular cognitive impairment with type 2 diabetes mellitus: A study protocol. Medicine (Baltimore). 2021;100(51):e28187. doi:10.1097/MD.0000000000028187	Not related to Oral CHM
0095	Wang J, Zhang X, Zhang Z, et al. Baseline Serum Bilirubin and Risk of First Stroke in Hypertensive Patients. J Am Heart Assoc. 2020;9(12):e015799. doi:10.1161/JAHA.119.015799	Not related to Oral CHM
0096	Zhou L, Cai X, Luo Y, Zhang F, Ji L. Baseline Triglyceride Level Affected the Efficacy of Vildagliptin in Treating Type 2 Diabetes: A Post Hoc Analysis of the VISION Study. J Diabetes Res. 2019;2019:9347132. Published 2019 Aug 8. doi:10.1155/2019/9347132	Not related to Oral CHM
0097	Shao Y, Shi X. Bibliometric Analysis and Visualization of Research Progress in the Diabetic Nephropathy Field from 2001 to 2021. Oxid Med Cell Longev. 2023;2023:4555609. Published 2023 Jan 21. doi:10.1155/2023/4555609	Not related to Oral CHM
0098	Ud Din SR, Saeed S, Khan SU, Kiani FA, Alsuhaibani AM, Zhong M. Bioactive Compounds (BACs): A Novel Approach to Treat and Prevent Cardiovascular Diseases. Curr Probl Cardiol. 2023;48(7):101664.	Not related to Oral CHM

	doi:10.1016/j.cpcardiol.2023.101664	
0099	Yu M, Li X, Jin H, et al. Bioequivalence of a Generic Nateglinide Formulation in Healthy Chinese Volunteers under Fasting and Fed Conditions: A Randomized, Open-Label, Double-Cycle, Double-Crossover Study. Pharmacology. 2021;106(7-8):418-425. doi:10.1159/000512851	Not related to Oral CHM
0100	Zanchetti A, Liu L, Mancia G, et al. Blood pressure and LDL-cholesterol targets for prevention of recurrent strokes and cognitive decline in the hypertensive patient: design of the European Society of Hypertension-Chinese Hypertension League Stroke in Hypertension Optimal Treatment randomized trial. J Hypertens. 2014;32(9):1888-1897. doi:10.1097/HJH.0000000000000254	Not related to Oral CHM
0101	Xiong XJ, Wang PQ, Li SJ. Blood-Letting Therapy for Hypertension: A Systematic Review and Meta-Analysis of Randomized Controlled Trials. Chin J Integr Med. 2019;25(2):139-146. doi:10.1007/s11655-018-3009-2	Not related to Oral CHM
0102	Mohamad RH, Zekry ZK, Al-Mehdar HA, et al. Camel milk as an adjuvant therapy for the treatment of type 1 diabetes: verification of a traditional ethnomedical practice. J Med Food. 2009;12(2):461-465. doi:10.1089/jmf.2008.0009	Not related to Oral CHM
0103	Rieckmann, R. Falldarstellung: Integrative Ernährungstherapie des metabolischen Syndroms. Chinese Medicine 30, 46–54 (2015).	Not related to Oral CHM
0104	Tian Y, Fan Y, Chen L, Sun M. Changes and significance of serum monocyte chemoattractant protein-1 and Lp-PLA2 in patients with hypertension and coronary heart disease. J Pak Med Assoc. 2022;72(6):1114-1117. doi:10.47391/JPMA.3244	Not related to Oral CHM
0105	Wang L, Geng J, Shan C, et al. Changes in left wrist pulse wave transit time in patients with hypertension and influencing factors[J]. Journal of Xi'an Jiaotong University (Medical Sciences), 2022, 43(4): 550-553.	Not related to Oral CHM
0106	Yu W, Ni S, Li Q, et al. Characteristics of 240 randomized controlled trials for adjusting constitution in preventive treatment of disease: A bibliometric analysis[J]. Journal of Traditional Chinese Medical Sciences, 2019, 6(3): 226-233.	Not related to Oral CHM
0107	Men X, Sun W, Fan F, et al. China Stroke Primary Prevention Trial: Visit-to-Visit Systolic Blood Pressure Variability Is an Independent Predictor of Primary Stroke in Hypertensive Patients. J Am Heart Assoc. 2017;6(3):e004350. Published 2017 Mar 13. doi:10.1161/JAHA.116.004350	Not related to Oral CHM
0108	Men X, Sun W, Fan F, et al. China Stroke Primary Prevention Trial: Visit-to-Visit Systolic Blood Pressure Variability Is an Independent Predictor of Primary Stroke in Hypertensive Patients. J Am Heart Assoc. 2017;6(3):e004350. Published 2017 Mar 13. doi:10.1161/JAHA.116.004350	Not related to Oral CHM

0109	Ryan GJ, Wanko NS, Redman AR, Cook CB. Chromium as adjunctive treatment for type 2 diabetes. Ann Pharmacother. 2003;37(6):876-885. doi:10.1345/aph.1C304	Not related to Oral CHM
0110	Turio E, Romanelli M, Barachini P. Clinical and instrumental evaluation of the efficacy of a vasoactive drug containing vitamin PP, vitamin C and phyto-therapeutic extracts titrated in escin, bromelain and anthocyanosides for the treatment of varicose leg ulcers[J]. Giornale Italiano di Dermatologia e Venereologia, 2000, 135(1): 101-106.	Not related to Oral CHM
0111	Wang P, Sun Y, Yi D, Xie Y, Luo Y. Clinical features of Chinese patients in different age groups with spontaneous intracerebral hemorrhage based on multicenter inpatient information. Neurol Res. 2020;42(8):657-664. doi:10.1080/01616412.2020.1782082	Not related to Oral CHM
0112	Zhao HL, Gao X, Gao YB. Zhongguo Zhong Xi Yi Jie He Za Zhi. 2007;27(4):312-314.	Not related to Oral CHM
0113	Wu B, Liu ZC, Xu B. Zhongguo Zhen Jiu. 2014;34(12):1151-1155.	Not related to Oral CHM
0114	Olalde JA, Magarici M, Amendola F, del Castillo O, Gonzalez S, Muhammad A. Clinical outcomes of diabetic foot management with Circulat. Phytother Res. 2008;22(10):1292-1298. doi:10.1002/ptr.2482	Not related to Oral CHM
0115	Zhuo Y, Wu J, Qu Y, et al. Clinical risk factors associated with recurrence of ischemic stroke within two years: A cohort study. Medicine (Baltimore). 2020;99(26):e20830. doi:10.1097/MD.0000000000020830	Not related to Oral CHM
0116	Yang, N, Guo, J and Wang, G, 2013. Clinical significance of tadalafil in the treatment of type II diabetes mellitus erectile dysfunction, Chinese Journal of Andrology.	Not related to Oral CHM
0117	Zhao YX, Liu YF, Yu HM. Chin J Integr Med. 2005;11(3):183-186. doi:10.1007/BF02836501	Not related to Oral CHM
0118	Wu SY, Lin QC, Yang C. Zhongguo Zhong Xi Yi Jie He Za Zhi. 1993;13(5):273-260.	Not related to Oral CHM
0119	Wu J, Zhang X, Zhao J, et al. Clinical study on acupuncture treatment of hypertension with hyperactivity of liver yang. Medicine (Baltimore). 2021;100(17):e25668. doi:10.1097/MD.0000000000025668	Not related to Oral CHM
0120	Sun YZ, Song J. Zhen Ci Yan Jiu. 2015;40(1):61-64.	Not related to Oral CHM
0121	Wesnes KA. Cognitive function testing: the case for standardization and automation. J Br Menopause Soc. 2006;12(4):158-163. doi:10.1258/136218006779160544	Not related to Oral CHM
0122	Zhang JL, Qin YW, Zheng X, Qiu JL, Zhao XX, Zou DJ. Combination therapy with angiotensin-converting enzyme inhibitors and indapamide impairs glucose tolerance in Chinese hypertensive patients. Blood Press. 2010;19(2):110-118. doi:10.3109/08037050903497238	Not related to Oral CHM

0123	Yan X, Jiang S, Lou Y, Zhou Z. Comparable efficacy and safety between LY2963016 insulin glargine and insulin glargine (Lantus庐) in Chinese patients with type 1 diabetes: A phase III, randomized, controlled trial.聽Diabetes Obes Metab. 2021;23(10):2226-2233. doi:10.1111/dom.14463	Not related to Oral CHM
0124	Sun W, Zeng C, Liao L, Chen J, Wang Y. Comparison of acarbose and metformin therapy in newly diagnosed type 2 diabetic patients with overweight and/or obesity. Curr Med Res Opin. 2016;32(8):1389-1396. doi:10.1080/03007995.2016.1176013	Not related to Oral CHM
0125	Yang H, Heng X, Liang C, et al. Comparison of continuous subcutaneous insulin infusion and multiple daily insulin injections in Chinese patients with type 2 diabetes mellitus. J Int Med Res. 2014;42(4):1002-1010. doi:10.1177/0300060514533523IF: 1.4 Q4	Not related to Oral CHM
0126	Yin TT, Bi Y, Li P, et al. Comparison of Glycemic Variability in Chinese T2DM Patients Treated with Exenatide or Insulin Glargine: A Randomized Controlled Trial. Diabetes Ther. 2018;9(3):1253-1267. doi:10.1007/s13300-018-0412-6	Not related to Oral CHM
0127	Steyer TE, Ables A. Complementary and alternative therapies for weight loss. Prim Care. 2009;36(2):395-406. doi:10.1016/j.pop.2009.01.011	Not related to Oral CHM
0128	Simkin, Deborah R.Emslie, Graham J. et al.COMPLEMENTARY AND INTEGRATIVE MEDICINE AND DEPRESSION: ROLE OF THE GUT-BRAIN AXIS, OMEGA-3 FATTY ACIDS, MICRONUTRIENTS, AND LIGHT THERAPY. Journal of the American Academy of Child & Adolescent Psychiatry, Volume 58, Issue 10, S329	Not related to Oral CHM
0129	Zhao WS, Zhai JJ, Wang YH, et al. Conjugated linoleic acid supplementation enhances antihypertensive effect of ramipril in Chinese patients with obesity-related hypertension. Am J Hypertens. 2009;22(6):680-686. doi:10.1038/ajh.2009.56	Not related to Oral CHM
0130	Stenblom EL, Egecioglu E, Landin-Olsson M, Erlanson-Albertsson C. Consumption of thylakoid-rich spinach extract reduces hunger, increases satiety and reduces cravings for palatable food in overweight women. Appetite. 2015;91:209-219. doi:10.1016/j.appet.2015.04.051	Not related to Oral CHM
0131	Williamson, J, Ramirez, R and Wingfield, T, 2014. Coping with chronic disease and disability in isolated communities of the peruvian Amazon: A case series, American Journal of Tropical Medicine and Hygiene.	Not related to Oral CHM
0132	Mortsiefer A, Meysen T, Schumacher M, et al. CRISTOPH - a cluster-randomised intervention study to optimise the treatment of patients with hypertension in General Practice. BMC Fam Pract. 2008;9:33. Published 2008 Jun 10. doi:10.1186/1471-2296-9-33	Not related to Oral CHM
0133	Tao J, Zhu J, Wang T, et al. CT-guided Chemical Lumbar Sympathectomy in the Treatment of Cold Hypersensitivity in the Hands and Feet. Pain Physician. 2021;24(4):E459-E466.	Not related to Oral CHM

0134	Weinberg Sibony R, Wainstein J, Ish Shalom M, et al. Curalin supplement for patients with type 2 diabetes mellitus. Diabetes Metab Res Rev. 2023;39(4):e3624. doi:10.1002/dmrr.3624	Not related to Oral CHM
0135	Kamal Awad, Peter Penson, Maciej Banach, 2016, D-003 (Saccharum officinarum): The forgotten lipid-lowering agent, Pharmacological Research, 114, 42-46.	Not related to Oral CHM
0136	Ried K, Frank OR, Stocks NP. Dark chocolate or tomato extract for prehypertension: a randomised controlled trial. BMC Complement Altern Med. 2009;9:22. Published 2009 Jul 8. doi:10.1186/1472-6882-9-22	Not related to Oral CHM
0137	G. Poglajen, M. Jovanovic, N. Žorž, S. Frljak, 2023, B. Vrtovec, Decreased Peripheral Cd34+ Cell Count is Associated with Worse Left Ventricular Function in Patients with Takotsubo Cardiomyopathy, 42(4), S244.	Not related to Oral CHM
0138	Zou P, Dennis CL, Lee R, Parry M. Dietary Approach to Stop Hypertension with Sodium Reduction for Chinese Canadians (DASHNa-CC): A Pilot Randomized Controlled Trial. J Nutr Health Aging. 2017;21(10):1225-1232. doi:10.1007/s12603-016-0861-4	Not related to Oral CHM
0139	Sluyter JD, Hughes AD, Lowe A, et al. Different associations between beta-blockers and other antihypertensive medication combinations with brachial blood pressure and aortic waveform parameters. Int J Cardiol. 2016;219:257-263. doi:10.1016/j.ijcard.2016.06.051IF: 3.2 Q2	Not related to Oral CHM
0140	Zhou J, Deng Z, Lu J, et al. Differential therapeutic effects of nateglinide and acarbose on fasting and postprandial lipid profiles: a randomized trial. Diabetes Technol Ther. 2015;17(4):229-234. doi:10.1089/dia.2014.0299	Not related to Oral CHM
0141	Sparrow K, Golianu B. Does Acupuncture Reduce Stress Over Time? A Clinical Heart Rate Variability Study in Hypertensive Patients. Med Acupunct. 2014;26(5):286-294. doi:10.1089/acu.2014.1050IF: 0.8 Q4	Not related to Oral CHM
0142	Zhu D, Gan S, Liu Y, et al. Dorzagliatin monotherapy in Chinese patients with type 2 diabetes: a dose-ranging, randomised, double-blind, placebo-controlled, phase 2 study [published correction appears in Lancet Diabetes Endocrinol. 2018 Aug;6(8):e16. doi: 10.1016/S2213-8587(18)30151-7.]. Lancet Diabetes Endocrinol. 2018;6(8):627-636. doi:10.1016/S2213-8587(18)30105-0	Not related to Oral CHM
0143	Moussouni M, Graff V, Couturier F, Herrscher H. Drug Interactions Causing Warfarin Overdose in a Patient with Pancreatic Cancer: A Case Report. Chemotherapy. 2023;68(2):111-114. doi:10.1159/000528063IF: 2.0 Q3	Not related to Oral CHM
0144	Shao, T et al. EE292 Effectiveness and Cost-Effectiveness for Non-Pharmaceutical Interventions in Chinese Prehypertensive Patients: A Network Meta-Analysis and Markov Model Analysis. Value in Health. 25(7), S391.	Not related to Oral CHM
0145	Xue N, Zhang J, Xia Z, Da N. Zhongguo Zhen Jiu. 2017;37(6):586-590. doi:10.13703/j.0255-2930.2017.06.004	Not related to Oral CHM

0146	Zou L, Lei L, Kong C, Yu P, Li J, Pan HS. Effect and safety of traditional Chinese exercises for patients with type 2 diabetes: A protocol for systematic review and network meta-analysis. Medicine (Baltimore). 2021;100(51):e28365. doi:10.1097/MD.0000000000028365	Not related to Oral CHM
0147	Zhan HL, Tang QP, Tang X, Ruan LH. Zhen Ci Yan Jiu. 2019;44(11):832-834. doi:10.13702/j.1000-0607.180832	Not related to Oral CHM
0148	Sun W, Li M, Lin T, et al. Effect of acupuncture at 3-points for intelligence on vascular dementia: Protocol for a systematic review and meta-analysis of randomized controlled trials. Medicine (Baltimore). 2018;97(42):e12892. doi:10.1097/MD.0000000000012892	Not related to Oral CHM
0149	Yang YQ, Zhang ZL, Li S, Zhou XY, Xing XT, Zhang JB. Zhongguo Zhen Jiu. 2021;41(11):1241-1247. doi:10.13703/j.0255-2930.20201104-0002	Not related to Oral CHM
0150	Zhang P, Chen Y, Zhang F, et al. Effect of Acupuncture on Blood Pressure and Metabolic Profile Among Patients With Essential Hypertension: Protocol of a Randomized Clinical Trial. Front Cardiovasc Med. 2022;9:888569. Published 2022 Jun 21. doi:10.3389/fcvm.2022.888569	Not related to Oral CHM
0151	Odunsi ST, Vázquez-Roque MI, Camilleri M, et al. Effect of alginate on satiation, appetite, gastric function, and selected gut satiety hormones in overweight and obesity. Obesity (Silver Spring). 2010;18(8):1579-1584. doi:10.1038/oby.2009.421	Not related to Oral CHM
0152	Yao J, Chen L, Zhang L, et al. Effect of auriculotherapy and intervention types on weight control: A systematic review and meta-analysis protocol. Medicine (Baltimore). 2019;98(34):e16959. doi:10.1097/MD.0000000000016959	Not related to Oral CHM
0153	Reddy K R C. Effect of Chanaka Yoga as a dietary supplement in the management of Type II diabetes mellitus patients[J]. International Journal of Green Pharmacy (IJGP), 2016, 10(04).	Not related to Oral CHM
0154	Rashidi Z, Beigi R, Ghahfarrokhi M M, et al. Effect of elastic band resistance training with green coffee extract supplementation on adiposity indices and TyG-related Indicators in Obese Women[J]. Obesity Medicine, 2021, 24: 100351.	Not related to Oral CHM
0155	Zhang RS, Tang L, Zhang Y, et al. Effect of folic acid supplementation on the change of plasma S-adenosylhomocysteine level in Chinese hypertensive patients: a randomized, double-blind, controlled clinical trial. J Clin Biochem Nutr. 2022;71(3):238-244. doi:10.3164/jcbn.22-13	Not related to Oral CHM
0156	Xie X, Lu L, Zhou X, et al. Effect of Gua Sha therapy on patients with diabetic peripheral neuropathy: A randomized controlled trial. Complement Ther Clin Pract. 2019;35:348-352. doi:10.1016/j.ctcp.2019.03.018	Not related to Oral CHM
0157	Meng N, Shi ZM. Zhongguo Zhen Jiu. 2020;40(4):361-364. doi:10.13703/j.0255-2930.20190415-0001	Not related to Oral CHM

0158	Zhu Z, Guo D, Shi M, et al. Effect of immediate blood pressure reduction on post-stroke depression in ischemic stroke patients: A substudy of CATIS trial. J Affect Disord. 2022;300:195-202. doi:10.1016/j.jad.2021.12.120	Not related to Oral CHM
0159	Tseng CC, Tseng A, Tseng J, Chang CH. Effect of Laser Acupuncture on Anthropometric Measurements and Appetite Sensations in Obese Subjects. Evid Based Complement Alternat Med. 2016;2016:9365326. doi:10.1155/2016/9365326	Not related to Oral CHM
0160	Pokorney SD, Cocoros N, Al-Khalidi HR, et al. Effect of Mailing Educational Material to Patients With Atrial Fibrillation and Their Clinicians on Use of Oral Anticoagulants: A Randomized Clinical Trial. JAMA Netw Open. 2022;5(5):e2214321. Published 2022 May 2. doi:10.1001/jamanetworkopen.2022.14321	Not related to Oral CHM
0161	Zhang JL, Zheng X, Zou DJ, Qiu JL, Zhao XX, Qin YW. Effect of metformin on weight gain during antihypertensive treatment with a beta-blocker in Chinese patients. Am J Hypertens. 2009;22(8):884-890. doi:10.1038/ajh.2009.93	Not related to Oral CHM
0162	Shashikumar P, Nisha S, Das D, et al. Effect of Morinda citrifolia L. mouthwash on periodontal health in type 2 diabetes mellitus patients– a randomized controlled trial[J]. International Journal of Nutrition, Pharmacology, Neurological Diseases, 2022, 12(1): 7-13.	Not related to Oral CHM
0163	Mao JY, Yang FW, Liu H, et al. Zhongguo Zhen Jiu. 2021;41(11):1216-1220. doi:10.13703/j.0255-2930.20200907-0005	Not related to Oral CHM
0164	Peker Y, Glantz H, Eulenburg C, Wegscheider K, Herlitz J, Thunström E. Effect of Positive Airway Pressure on Cardiovascular Outcomes in Coronary Artery Disease Patients with Nonsleepy Obstructive Sleep Apnea. The RICCADSA Randomized Controlled Trial. Am J Respir Crit Care Med. 2016;194(5):613-620. doi:10.1164/rccm.201601-0088OC	Not related to Oral CHM
0165	Vuksan V, Sievenpiper JL, Jovanovski E, et al. Effect of soluble-viscous dietary fibre on coronary heart disease risk score across 3 population health categories: data from randomized, double-blind, placebo-controlled trials. Appl Physiol Nutr Metab. 2020;45(7):801-804. doi:10.1139/apnm-2019-0728	Not related to Oral CHM
0166	Zhou J, Zhou B, Kou X, et al. Effect of summer acupoint application treatment (SAAT) on gut microbiota in healthy Asian adults: A randomized controlled trial. Medicine (Baltimore). 2023;102(9):e32951. doi:10.1097/MD.0000000000032951	Not related to Oral CHM
0167	Rahmoun NM, Boukli-Hacene G, Bettioui RA, Ghembaza CE, Ghembaza ME. Effect of the consumption of natural products on some biochemical parameters of a type 2 diabetic population. J Diabetes Metab Disord. 2020;19(1):233-241. Published 2020 Jan 28. doi:10.1007/s40200-020-00496-2	Not related to Oral CHM

0168	Wolak T, Sharoni Y, Levy J, Linnewiel-Hermoni K, Stepensky D, Paran E. Effect of Tomato Nutrient Complex on Blood Pressure: A Double Blind, Randomized Dose⁻Response Study. Nutrients. 2019;11(5):950. Published 2019 Apr 26. doi:10.3390/nu11050950	Not related to Oral CHM
0169	Piao C, Zhang Q, Fu H, Wang L, Tang C. Effectiveness comparisons of catgut implantation at acupoint for obese type 2 diabetes: A protocol for systematic review and meta analysis. Medicine (Baltimore). 2020;99(30):e21316. doi:10.1097/MD.0000000000021316	Not related to Oral CHM
0170	Pace WD, Lanigan AM, Staton EW, et al. Effectiveness of 2 methods of promoting physical activity, healthy eating, and emotional well-being with the americans in motion--healthy interventions approach. Ann Fam Med. 2013;11(4):371-380. doi:10.1370/afm.1516	Not related to Oral CHM
0171	Vas P, Rayman G, Dhatariya K, et al. Effectiveness of interventions to enhance healing of chronic foot ulcers in diabetes: a systematic review. Diabetes Metab Res Rev. 2020;36 Suppl 1:e3284. doi:10.1002/dmrr.3284	Not related to Oral CHM
0172	Sharma M, Sharma H R, Takalkar K, et al. Effectiveness of Shamak Yoga on Essential Hypertension in comparison with Atenolol: A Randomized Controlled Trial[J]. Research Journal of Pharmacy and Technology, 2021, 14(9): 4925-4928.	Not related to Oral CHM
0173	Preuss HG, Bagchi D, Bagchi M, Rao CV, Dey DK, Satyanarayana S. Effects of a natural extract of (-)-hydroxycitric acid (HCA-SX) and a combination of HCA-SX plus niacin-bound chromium and Gymnema sylvestre extract on weight loss. Diabetes Obes Metab. 2004;6(3):171-180. doi:10.1111/j.1462-8902.2004.00328.x	Not related to Oral CHM
0174	Sun JY, Zhai L, Li QL, et al. Effects of ACE inhibition on endothelial progenitor cell mobilization and prognosis after acute myocardial infarction in type 2 diabetic patients. Clinics (Sao Paulo). 2013;68(5):665-673. doi:10.6061/clinics/2013(05)14	Not related to Oral CHM
0175	Yang G, Tan Z, Zhou L, et al. Effects of Angiotensin II Receptor Blockers and ACE (Angiotensin-Converting Enzyme) Inhibitors on Virus Infection, Inflammatory Status, and Clinical Outcomes in Patients With COVID-19 and Hypertension: A Single-Center Retrospective Study. Hypertension. 2020;76(1):51-58. doi:10.1161/HYPERTENSIONAHA.120.15143	Not related to Oral CHM
0176	Zhu Y, Sun J, Lu W, et al. Effects of blueberry supplementation on blood pressure: a systematic review and meta-analysis of randomized clinical trials. J Hum Hypertens. 2017;31(3):165-171. doi:10.1038/jhh.2016.70	Not related to Oral CHM
0177	Qi J, Ping R, Zhang S, Xu Y, Wu K, Li Y. Effects of Cervical Rotatory Manipulation (CRM) on Carotid Atherosclerosis Plaque in Vulnerability: A Histological and Immunohistochemical Study Using Animal Model. Biomed Res Int. 2019;2019:3793840. Published 2019 Feb 4. doi:10.1155/2019/3793840	Not related to Oral CHM

0178	You JH, Chan WK, Chung PF, Hu M, Tomlinson B. Effects of concomitant therapy with diltiazem on the lipid responses to simvastatin in Chinese subjects. J Clin Pharmacol. 2010;50(10):1151-1158. doi:10.1177/0091270009358082	Not related to Oral CHM
0179	Zhao Q, Liu ZH, Luo Q, Zhao ZH, Zhang HL, Wang Y. Effects of continuous positive airway pressure on blood pressure and daytime sleepiness in obstructive sleep apnea patients with coronary heart diseases under optimal medications. Sleep Breath. 2012;16(2):341-347. doi:10.1007/s11325-011-0498-9	Not related to Oral CHM
0180	Wang H, Wang J, Lu J, Wang D. Effects of High Dose of Atorvastatin for Preventing Periprocedural Ischemic Brain Damage in Patients Undergoing Carotid Artery Stenting (PICAS) in China: A Randomized Controlled Clinical Trial. Front Neurol. 2020;11:937. Published 2020 Aug 25. doi:10.3389/fneur.2020.00937	Not related to Oral CHM
0181	Wei Y, Jin Z, Shen G, et al. Effects of intensive antihypertensive treatment on Chinese hypertensive patients older than 70 years. J Clin Hypertens (Greenwich). 2013;15(6):420-427. doi:10.1111/jch.12094	Not related to Oral CHM
0182	Yu W, Hu C, Zhang R, et al. Effects of KCNQ1 polymorphisms on the therapeutic efficacy of oral antidiabetic drugs in Chinese patients with type 2 diabetes. Clin Pharmacol Ther. 2011;89(3):437-442. doi:10.1038/clpt.2010.351	Not related to Oral CHM
0183	Pu R, Shi D, Gan T, et al. Effects of metformin in obesity treatment in different populations: a meta-analysis. Ther Adv Endocrinol Metab. 2020;11:2042018820926000. Published 2020 May 21. doi:10.1177/2042018820926000	Not related to Oral CHM
0184	Paoli A, Moro T, Bosco G, et al. Effects of n-3 polyunsaturated fatty acids (蠅-3) supplementation on some cardiovascular risk factors with a ketogenic Mediterranean diet.聽Mar Drugs. 2015;13(2):996-1009. Published 2015 Feb 13. doi:10.3390/md13020996IF: 4.9 聽Q1	Not related to Oral CHM
0185	Wang T, Wang Y, Lv DM, et al. Effects of NOS1AP rs12742393 polymorphism on repaglinide response in Chinese patients with type 2 diabetes mellitus. Pharmacotherapy. 2014;34(2):131-139. doi:10.1002/phar.1379	Not related to Oral CHM
0186	Wang TF, Pei D, Li JC, et al. Effects of sibutramine in overweight, poorly controlled Chinese female type 2 diabetic patients: a randomised, double-blind, placebo-controlled study. Int J Clin Pract. 2005;59(7):746-750. doi:10.1111/j.1368-5031.2005.00568.x	Not related to Oral CHM
0187	Wang X, Zhao B, Sun H, You H, Qu S. Effects of sitagliptin on intrahepatic lipid content in patients with non-alcoholic fatty liver disease. Front Endocrinol (Lausanne). 2022;13:866189. Published 2022 Aug 22. doi:10.3389/fendo.2022.866189	Not related to Oral CHM

0188	Sartor CD, Hasue RH, Cacciari LP, et al. Effects of strengthening, stretching and functional training on foot function in patients with diabetic neuropathy: results of a randomized controlled trial. BMC Musculoskelet Disord. 2014;15:137. Published 2014 Apr 27. doi:10.1186/1471-2474-15-137	Not related to Oral CHM
0189	Mohajeri G, Safaee M, Sanei MH. Effects of topical Kiwifruit on healing of neuropathic diabetic foot ulcer. J Res Med Sci. 2014;19(6):520-524.	Not related to Oral CHM
0190	Yang M, Huang Q, Wu J, et al. Effects of UCP2 -866 G/A and ADRB3 Trp64Arg on rosiglitazone response in Chinese patients with Type 2 diabetes. Br J Clin Pharmacol. 2009;68(1):14-22. doi:10.1111/j.1365-2125.2009.03431.x	Not related to Oral CHM
0191	Xiaoyan C, Jing W, Xiaochun H, Yuyu T, Shunyou D, Yingyu F. Effects of vildagliptin versus saxagliptin on daily acute glucose fluctuations in Chinese patients with T2DM inadequately controlled with a combination of metformin and sulfonylurea. Curr Med Res Opin. 2016;32(6):1131-1136. doi:10.1185/03007995.2016.1162773	Not related to Oral CHM
0192	Zhou X, Chen J. Efficacy and safety of acupuncture and related techniques for type 2 diabetes mellitus: a systematic review of 21 randomised controlled trials[J]. Metabolism-Clinical and Experimental, 2021, 116.	Not related to Oral CHM
0193	Zhao H, Li D, Li Y, et al. Efficacy and safety of acupuncture for hypertension: An overview of systematic reviews. Complement Ther Clin Pract. 2019;34:185-194. doi:10.1016/j.ctcp.2018.12.003	Not related to Oral CHM
0194	Tsuchida T, MASIKO K, OSADA H, et al. Efficacy and safety of an aqueous extract from Mycoleptodonoides aitchisonii in the long-term administration[J]. JAPANESE PHARMACOLOGY AND THERAPEUTICS, 2002, 30(1): 31-36.	Not related to Oral CHM
0195	Sawangjit R, Puttarak P, Saokaew S, Chaiyakunapruk N. Efficacy and Safety of Cissus quadrangularis L. in Clinical Use: A Systematic Review and Meta-analysis of Randomized Controlled Trials. Phytother Res. 2017;31(4):555-567. doi:10.1002/ptr.5783	Not related to Oral CHM
0196	Shi LX, Liu XM, Shi YQ, et al. Efficacy and safety of dulaglutide monotherapy compared with glimepiride in Chinese patients with type 2 diabetes: Post-hoc analyses of a randomized, double-blind, phase III study. J Diabetes Investig. 2020;11(1):142-150. doi:10.1111/jdi.13075	Not related to Oral CHM
0197	Wang LX, Li WH, He F. Efficacy and Safety of Electroacupuncture in the Treatment of Cerebral Infarction: Systematic Review and Meta-Analysis [retracted in: Appl Bionics Biomech. 2023 Aug 16;2023:9864153. doi: 10.1155/2023/9864153.]. Appl Bionics Biomech. 2022;2022:1350501. Published 2022 Jun 28. doi:10.1155/2022/1350501IF: 1.8 Q3	Not related to Oral CHM
0198	Zhou P, Lu Z, Gao P, et al. Efficacy and safety of intensive statin therapy in Chinese patients with atherosclerotic intracranial arterial stenosis: a single-center, randomized, single-blind, parallel-group study with one-year follow-up. Clin Neurol Neurosurg. 2014;120:6-13. doi:10.1016/j.clineuro.2014.02.001	Not related to Oral CHM

019<br>9	Yan X, Feng C, Lou Y, Zhou Z. Efficacy and Safety of LY2963016 Insulin Glargine in Chinese Patients with Type聽 1 Diabetes Previously Treated with Insulin Glargine (Lantus庐): a Post Hoc Analysis of a Randomized, Open-Label, Phase聽 3 Trial.聽Diabetes Ther. 2022;13(6):1161-1174. doi:10.1007/s13300-022-01262-8	Not related to Oral CHM
020<br>0	Sawangjit R, Thongphui S, Chaichompu W, Phumart P. Efficacy and Safety of Mecobalamin on Peripheral Neuropathy: A Systematic Review and Meta-Analysis of Randomized Controlled Trials. J Altern Complement Med. 2020;26(12):1117-1129. doi:10.1089/acm.2020.0068	Not related to Oral CHM
020<br>1	Yu B, Huang W, Zhang Y, et al. Efficacy and Safety of Moxibustion for Menopausal Obesity: A Multicentre, Randomized, Controlled Trial Protocol. Evid Based Complement Alternat Med. 2022;2022:9255017. Published 2022 Aug 4. doi:10.1155/2022/9255017	Not related to Oral CHM
020<br>2	Mu Y, Liu X, Li Q, et al. Efficacy and safety of pregabalin for painful diabetic peripheral neuropathy in a population of Chinese patients: A randomized placebo-controlled trial. J Diabetes. 2018;10(3):256-265. doi:10.1111/1753-0407.12585	Not related to Oral CHM
020<br>3	Wong MOM, Lai IS, Chan PP, et al. Efficacy and safety of selective laser trabeculoplasty and pattern scanning laser trabeculoplasty: a randomised clinical trial. Br J Ophthalmol. 2021;105(4):514-520. doi:10.1136/bjophthalmol-2020-316178	Not related to Oral CHM
020<br>4	Zhao LN, Liang Y, Fang XJ, et al. Efficacy and safety of tacrolimus in Osserman grade III and Osserman grade IV Myasthenia Gravis. Clin Neurol Neurosurg. 2018;173:70-76. doi:10.1016/j.clineuro.2018.07.023	Not related to Oral CHM
020<br>5	Zhang JP, Wang N, Xing XY, Yang ZJ, Wang X, Yang WY. Efficacy of acarbose and metformin in newly diagnosed type 2 diabetes patients stratified by HbA1c levels. J Diabetes. 2016;8(4):559-567. doi:10.1111/1753-0407.12337	Not related to Oral CHM
020<br>6	Wang Y, Huang YW, Ablikim D, et al. Efficacy of acupuncture at ghost points combined with fluoxetine in treating depression: A randomized study. World J Clin Cases. 2022;10(3):929-938. doi:10.12998/wjcc.v10.i3.929	Not related to Oral CHM
020<br>7	Shanti K, R S. Ayurvedic management of venous ulcer - a case report. J Ayurveda Integr Med. 2023;14(3):100723. doi:10.1016/j.jaim.2023.100723	Not related to Oral CHM
020<br>8	Zhao J, Tostivint I, Xu L, et al. Efficacy of Combined Abelmoschus manihot and Irbesartan for Reduction of Albuminuria in Patients With Type 2 Diabetes and Diabetic Kidney Disease: A Multicenter Randomized Double-Blind Parallel Controlled Clinical Trial. Diabetes Care. 2022;45(7):e113-e115. doi:10.2337/dc22-0607	Not related to Oral CHM
020<br>9	Zhou LY, Li XM, Liu TJ, et al. Efficacy of intraorbital electroacupuncture for diabetic abducens nerve palsy: study protocol for a prospective single-center randomized controlled trial. Neural Regen Res. 2017;12(5):826-830. doi:10.4103/1673-5374.206654	Not related to Oral CHM

0210	Quamri MA, Wahab A, Alam MA, Ali BF. Efficacy of Majoon-e-Seer Alvi Khan in dyslipidemia: a single blind randomized standard controlled clinical trial. Drug Metab Pers Ther. 2021;36(4):271-279. Published 2021 Jul 9. doi:10.1515/dmpt-2021-0117	Not related to Oral CHM
0211	Tay JS, Kim YJ. Efficacy of moxibustion in diabetes peripheral neuropathy. Medicine (Baltimore). 2021;100(49):e28173. doi:10.1097/MD.0000000000028173	Not related to Oral CHM
0212	Taniguchi S, Temma J, Kuroda A, et al. Efficacy of PHR Integrated with EHR and Self-Monitoring Devices on Self-Care in Patients with Type 2 Diabetes[C]//DIABETES. 1701 N BEAUREGARD ST, ALEXANDRIA, VA 22311-1717 USA: AMER DIABETES ASSOC, 2017, 66: A613-A613.	Not related to Oral CHM
0213	Toromanyan E, Aslanyan G, Amroyan E, Gabrielyan E, Panossian A. Efficacy of Slim339 in reducing body weight of overweight and obese human subjects. Phytother Res. 2007;21(12):1177-1181. doi:10.1002/ptr.2231	Not related to Oral CHM
0214	Yang Q, Wang F, Pan L, Ye T. Efficacy of traditional Chinese exercises in improving anthropometric and biochemical indicators in overweight and obese subjects: A systematic review and meta-analysis. Medicine (Baltimore). 2023;102(12):e33051. doi:10.1097/MD.0000000000033051	Not related to Oral CHM
0215	Rerksuppaphol L, Rerksuppaphol S. Efficacy of transcutaneous electrical acupoint stimulation compared to electroacupuncture at the main acupoints for weight reduction in obese Thai women[J]. Int J Collab Res Intern Med Public Health, 2011, 3(11): 811-820.	Not related to Oral CHM
0216	Yang G, Zheng B, Yu Y, et al. Electroacupuncture at Zusanli (ST36), Guanyuan (CV4), and Qihai (CV6) Acupoints Regulates Immune Function in Patients with Sepsis via the PD-1 Pathway [retracted in: Biomed Res Int. 2024 Mar 20;2024:9832793. doi: 10.1155/2024/9832793.]. Biomed Res Int. 2022;2022:7037497. Published 2022 Jul 11. doi:10.1155/2022/7037497	Not related to Oral CHM
0217	Meyer-Hamme G, Friedemann T, Greten J, Gerloff C, Schroeder S. Electrophysiologically verified effects of acupuncture on diabetic peripheral neuropathy in type 2 diabetes: The randomized, partially double-blinded, controlled ACUDIN trial. J Diabetes. 2021;13(6):469-481. doi:10.1111/1753-0407.13130	Not related to Oral CHM
0218	Saini R, Nautiyal A, Patil S. EMERGING ROLE OF NUTRACEUTICALS IN THE MANAGEMENT OF HYPERTENSION: A SYSTEMATIC REVIEW[J].	Not related to Oral CHM
0219	Zou H, Ge Y, Lei Q, et al. Epidemiology and disease burden of non-alcoholic steatohepatitis in greater China: a systematic review. Hepatol Int. 2022;16(1):27-37. doi:10.1007/s12072-021-10286-4	Not related to Oral CHM
0220	Ulbricht C, Weissner W, Hashmi S, et al. Essiac: systematic review by the natural standard research collaboration. J Soc Integr Oncol. 2009;7(2):73-80.	Not related to Oral CHM

022<br>1	Verny MA, Milenkovic D, Macian N, et al. Evaluating the role of orange juice, HESPERidin in vascular HEALTH benefits (HESPER-HEALTH study): protocol for a randomised controlled trial. BMJ Open. 2021;11(11):e053321. Published 2021 Nov 30. doi:10.1136/bmjopen-2021-053321	Not related to Oral CHM
022<br>2	Zhong X, Yan X, Liang H, Xia R, Chen B, Zhao HJ. Evaluation of eight-style Tai chi on cognitive function in patients with cognitive impairment of cerebral small vessel disease: study protocol for a randomised controlled trial. BMJ Open. 2021;11(2):e042177. Published 2021 Feb 8. doi:10.1136/bmjopen-2020-042177	Not related to Oral CHM
022<br>3	Udani JK, Singh BB, Barrett ML, Singh VJ. Evaluation of Mangosteen juice blend on biomarkers of inflammation in obese subjects: a pilot, dose finding study. Nutr J. 2009;8:48. Published 2009 Oct 20. doi:10.1186/1475-2891-8-48	Not related to Oral CHM
022<br>4	Mateos R, García-Cordero J, Bravo-Clemente L, Sarriá B. Evaluation of novel nutraceuticals based on the combination of oat beta-glucans and a green coffee phenolic extract to combat obesity and its comorbidities. A randomized, dose-response, parallel trial. Food Funct. 2022;13(2):574-586. Published 2022 Jan 24. doi:10.1039/d1fo02272e	Not related to Oral CHM
022<br>5	Vakilinia SR, Vaghasloo MA, Aliasl F, et al. Evaluation of the efficacy of warm salt water foot-bath on patients with painful diabetic peripheral neuropathy: A randomized clinical trial. Complement Ther Med. 2020;49:102325. doi:10.1016/j.ctim.2020.102325	Not related to Oral CHM
022<br>6	Thacker H, Bantwal G, Jain S, et al. Evaluation Series on Safety and Efficacy of Nutritional Supplements in Newly Diagnosed Hyperglycemia: A Placebo-Controlled, Randomized Study. N Am J Med Sci. 2016;8(2):106-113. doi:10.4103/1947-2714.177320	Not related to Oral CHM
022<br>7	Zhang YP, Hu RX, Han M, et al. Evidence Base of Clinical Studies on Qi Gong: A Bibliometric Analysis. Complement Ther Med. 2020;50:102392. doi:10.1016/j.ctim.2020.102392	Not related to Oral CHM
022<br>8	Wang H, Ren Q, Han X, et al. Factors of primary and secondary sulfonylurea failure in type 2 diabetic subjects. J Diabetes. 2017;9(12):1091-1099. doi:10.1111/1753-0407.12542IF: 3.0 Q2	Not related to Oral CHM
022<br>9	Zhang J, Wang N, Xing X, Yang Z, Wang X, Yang W. Factors that influence the efficacy of acarbose and metformin as initial therapy in Chinese patients with newly diagnosed type 2 diabetes: a subanalysis of the MARCH trial. Curr Med Res Opin. 2016;32(4):713-719. doi:10.1185/03007995.2015.1136819	Not related to Oral CHM
023<br>0	Nickerson J E, Krieger A C, Simon E P, et al. Feasibility of portable technology to diagnose sleep apnea in underserved communities[C]//Journal of General Internal Medicine. 233 SPRING ST, NEW YORK, NY 10013 USA: SPRINGER, 2012, 27: S196-S196.	Not related to Oral CHM

0231	Zhang H, Gao L, Lou J, et al. First-In-Human Study on Pharmacokinetics, Safety, and Tolerability of Single and Multiple Escalating Doses of Hepenofovir, a Novel Hepatic Targeting Prodrug of Tenofovir in Healthy Chinese Subjects. Front Pharmacol. 2022;13:873588. Published 2022 May 19. doi:10.3389/fphar.2022.873588	Not related to Oral CHM
0232	Xu L, Zang D, Li H, et al. Five Traditional Chinese Medicine External Treatment Methods Combined with Mecobalamin for Diabetic Peripheral Neuropathy: A Network Meta-Analysis. Evid Based Complement Alternat Med. 2022;2022:4251022. Published 2022 Dec 16. doi:10.1155/2022/4251022	Not related to Oral CHM
0233	Venugopal V, Yogapriya C, Deenadayalan B, et al. Foot reflexology for reduction of blood pressure in hypertensive individual: A systematic review. Foot (Edinb). 2023;54:101974. doi:10.1016/j.foot.2023.101974	Not related to Oral CHM
0234	Pippa L, Manzoli L, Corti I, Congedo G, Romanazzi L, Parruti G. Functional capacity after traditional Chinese medicine (qi gong) training in patients with chronic atrial fibrillation: a randomized controlled trial. Prev Cardiol. 2007;10(1):22-25. doi:10.1111/j.1520-037x.2007.05721.x	Not related to Oral CHM
0235	Tam CC, Kwok J, Wong A, et al. Genotyping-guided approach versus the conventional approach in selection of oral P2Y12 receptor blockers in Chinese patients suffering from acute coronary syndrome. J Int Med Res. 2017;45(1):134-146. doi:10.1177/0300060516677190	Not related to Oral CHM
0236	Shao SY, Xu WJ, Tao J, et al. Glycemic index, glycemic load, and glycemic response to pomelo in patients with type 2 diabetes. J Huazhong Univ Sci Technolog Med Sci. 2017;37(5):711-718. doi:10.1007/s11596-017-1793-x	Not related to Oral CHM
0237	Zhou X, Wu Q, Zhang G, et al. Heat-sensitive moxibustion self-administration in patients in the community with primary hypertension: A protocol for a multi-center, pragmatic, non-randomized trial. Medicine (Baltimore). 2020;99(38):e22230. doi:10.1097/MD.0000000000022230	Not related to Oral CHM
0238	Walker AF. Herbal medicine: the science of the art. Proc Nutr Soc. 2006;65(2):145-152. doi:10.1079/pns2006487	Not related to Oral CHM
0239	Vermani M, Milosevic I, Smith F, Katzman MA. Herbs for mental illness: effectiveness and interaction with conventional medicines. J Fam Pract. 2005;54(9):789-800.	Not related to Oral CHM
0240	Zhang J, Tang G, Xie H, et al. Higher Adiposity Is Associated With Slower Cognitive Decline in Hypertensive Patients: Secondary Analysis of the China Stroke Primary Prevention Trial. J Am Heart Assoc. 2017;6(10):e005561. Published 2017 Oct 10. doi:10.1161/JAHA.117.005561	Not related to Oral CHM
0241	Zhao M, Wang X, He M, et al. Homocysteine and Stroke Risk: Modifying Effect of Methylenetetrahydrofolate Reductase C677T Polymorphism and Folic Acid Intervention. Stroke. 2017;48(5):1183-1190. doi:10.1161/STROKEAHA.116.015324	Not related to Oral CHM

0242	Vale MJ, Jelinek MV, Best JD; COACH study group. Coaching patients on Achieving Cardiovascular Health. How many patients with coronary heart disease are not achieving their risk-factor targets? Experience in Victoria 1996-1998 versus 1999-2000. Med J Aust. 2002;176(5):211-215. doi:10.5694/j.1326-5377.2002.tb04375.x	Not related to Oral CHM
0243	Xie J, Wang J, Yang H. Hypertension control improved through patient education. Chinese PEP Investigators. Chin Med J (Engl). 1998;111(7):581-584.	Not related to Oral CHM
0244	Muscariello E, Nasti G, Battinelli R, Novellino T, Montisano M, Colantuoni A. Hypolipidic diet and phytosubstance supplement in hypercholesterolemia. Minerva Gastroenterol Dietol. 2014;60(2):119-125.	Not related to Oral CHM
0245	Velliquette RA, Grann K, Missler SR, et al. Identification of a botanical inhibitor of intestinal diacylglyceride acyltransferase 1 activity via in vitro screening and a parallel, randomized, blinded, placebo-controlled clinical trial. Nutr Metab (Lond). 2015;12:27. Published 2015 Aug 6. doi:10.1186/s12986-015-0025-2	Not related to Oral CHM
0246	Wang W, Song X, Lou Y, Du L, Zhu D, Zhou Z. Immunogenicity of LY2963016 insulin glargine and Lantus庐 insulin glargine in Chinese patients with type 1 or type 2 diabetes mellitus.聽Diabetes Obes Metab. 2022;24(6):1094-1104. doi:10.1111/dom.14674	Not related to Oral CHM
0247	Saraç S, Afşar GÇ, Oruç Ö, Topçuoğlu ÖB, Saltürk C, Peker Y. Impact of Patient Education on Compliance with Positive Airway Pressure Treatment in Obstructive Sleep Apnea. Med Sci Monit. 2017;23:1792-1799. Published 2017 Apr 13. doi:10.12659/msm.902075	Not related to Oral CHM
0248	Proctor A, Billings C, Billings C, Moloney ED. Improvement in energy and vitality over a short-term CPAP trial predicts long-term compliance in patients with obstructive sleep apnea. J Sleep Res. 2007;16(4):448-449. doi:10.1111/j.1365-2869.2007.00610.x	Not related to Oral CHM
0249	Obaid, M, Flach, C, Prasad, V, Marshall, I and Douiri, A, 2019. In stroke patients, what are the risk factors and interventions to prevent worsening cognition? An umbrella review of systematic reviews, European Stroke Journal.	Not related to Oral CHM
0250	Lorence Villarino M. Indigenous knowledge of medicinal fruits in the Philippines: a systematic review[J]. Research Journal of Pharmacognosy, 2023, 10(3): 77-89.	Not related to Oral CHM
0251	Sadhukhan S, Singh S, Michael J, et al. Individualized Homeopathic Medicines in Stage I Essential Hypertension: A Double-Blind, Randomized, Placebo-Controlled Pilot Trial. J Altern Complement Med. 2021;27(6):515-521. doi:10.1089/acm.2020.0222	Not related to Oral CHM
0252	Wu Y, Gu R, Hao F, et al. Influence of traditional Chinese Baduanjin Qigong (eight trigrams boxing) on blood glucose and blood lipid levels in patients with type 2 diabetes mellitus[C]//BASIC & CLINICAL PHARMACOLOGY & TOXICOLOGY. 111 RIVER ST, HOBOKEN 07030-5774, NJ USA: WILEY, 2019, 125: 33-33.	Not related to Oral CHM

0253	Wu, Y., Wei, Q., Fang, Z., & Lin, F. (2019). INFLUENCE OF TRADITIONAL CHINESE MEDICINE HEALTH PRESERVATION GONGFA ON FASTING BLOOD GLUCOSE AND FASTING INSULIN IN PRE-DIABETES PATIENTS. ACTA MEDICA MEDITERRANEA, 35(2), 745-749.	Not related to Oral CHM
0254	Vilaplana-Carnerero C, Aznar-Lou I, Peñarrubia-María MT, et al. Initiation and Single Dispensing in Cardiovascular and Insulin Medications: Prevalence and Explanatory Factors. Int J Environ Res Public Health. 2020;17(10):3358. Published 2020 May 12. doi:10.3390/ijerph17103358	Not related to Oral CHM
0255	Shrivastav D, Dabla PK, Sharma J, Viswas A, Mir R. Insights on antioxidant therapeutic strategies in type 2 diabetes mellitus: A narrative review of randomized control trials. World J Diabetes. 2023;14(6):919-929. doi:10.4239/wjd.v14.i6.919	Not related to Oral CHM
0256	Ruiz-Roso B, Quintela JC, de la Fuente E, Haya J, Pérez-Olleros L. Insoluble carob fiber rich in polyphenols lowers total and LDL cholesterol in hypercholesterolemic sujects. Plant Foods Hum Nutr. 2010;65(1):50-56. doi:10.1007/s11130-009-0153-9	Not related to Oral CHM
0257	Wolever RQ, Dreusicke MH. Integrative health coaching: a behavior skills approach that improves HbA1c and pharmacy claims-derived medication adherence. BMJ Open Diabetes Res Care. 2016;4(1):e000201. Published 2016 May 9. doi:10.1136/bmjdrc-2016-000201	Not related to Oral CHM
0258	Olund Villumsen S, Benfeitas R, Knudsen AD, et al. Integrative Lipidomics and Metabolomics for System-Level Understanding of the Metabolic Syndrome in Long-Term Treated HIV-Infected Individuals. Front Immunol. 2022;12:742736. Published 2022 Jan 12. doi:10.3389/fimmu.2021.742736	Not related to Oral CHM
0259	Zhang ZW, Gong CX. Intractable or persistent hiccups treated with extracranial acupuncture: Two case reports. Medicine (Baltimore). 2020;99(20):e20131. doi:10.1097/MD.0000000000020131	Not related to Oral CHM
0260	Qiu, YB, Yang, Z, Guo, JH, Hong, WU, Zhang, X and Zheng, Q, 2010. Kaishi injection for diabetic nephropathy: A systematic review, Chinese Journal of Evidence-Based Medicine.	Not related to Oral CHM
0261	Si L, Lin R, Jia Y, et al. Lactobacillus bulgaricus improves antioxidant capacity of black garlic in the prevention of gestational diabetes mellitus: a randomized control trial. Biosci Rep. 2019;39(8):BSR20182254. Published 2019 Aug 9. doi:10.1042/BSR20182254	Not related to Oral CHM
0262	Pinto JW, Bradbury K, Newell D, Bishop FL. Lifestyle and Health Behavior Change in Traditional Acupuncture Practice: A Systematic Critical Interpretive Synthesis. J Altern Complement Med. 2021;27(3):238-254. doi:10.1089/acm.2020.0365IF: 2.3 Q2	Not related to Oral CHM
0263	van de Laar FA, Akkermans RP, van Binsbergen JJ. Limited evidence for effects of diet for type 2 diabetes from systematic reviews. Eur J Clin Nutr. 2007;61(8):929-937. doi:10.1038/sj.ejcn.1602611IF: 3.6 Q2	Not related to Oral CHM

026<br>4	Zhang WQ, Tian Y, Chen XM, Wang LF, Chen CC, Qiu CM. Liraglutide ameliorates beta-cell function, alleviates oxidative stress and inhibits low grade inflammation in young patients with new-onset type 2 diabetes. Diabetol Metab Syndr. 2018;10:91. Published 2018 Dec 17. doi:10.1186/s13098-018-0392-8	Not related to Oral CHM
026<br>5	Wu S, Zheng C, Liu N, et al. Liuzijue training improves hypertension and modulates gut microbiota profile. Front Cardiovasc Med. 2023;10:1075084. Published 2023 Jan 25. doi:10.3389/fcvm.2023.1075084	Not related to Oral CHM
026<br>6	Wang JG, Liu G, Wang X, et al. Long-term blood pressure control in older Chinese patients with isolated systolic hypertension: a progress report on the Syst-China trial. J Hum Hypertens. 1996;10(11):735-742.	Not related to Oral CHM
026<br>7	Zhu, BP, Fan, L and Li, XY, 2005. Long-term effect and reliability of warfarin and aspirin for primary prevention of cardio-cerebral vessels events in patients with peripheral arteriosclerotic occlusive disease: a randomized, single-blind, controlled clinical trial with two-year follow-up. Chinese journal of clinical rehabilitation	Not related to Oral CHM
026<br>8	Tang SC, Tang AW, Wong SS, Leung JC, Ho YW, Lai KN. Long-term study of mycophenolate mofetil treatment in IgA nephropathy. Kidney Int. 2010;77(6):543-549. doi:10.1038/ki.2009.499IF: 14.8 Q1	Not related to Oral CHM
026<br>9	Wang L, Li H, Zhou Y, Jin L, Liu J. Low-dose B vitamins supplementation ameliorates cardiovascular risk: a double-blind randomized controlled trial in healthy Chinese elderly. Eur J Nutr. 2015;54(3):455-464. doi:10.1007/s00394-014-0729-5IF: 4.1 Q2	Not related to Oral CHM
027<br>0	Song N, Shan XS, Yang Y, et al. Low-Dose Esketamine as an Adjuvant to Propofol Sedation for Same-Visit Bidirectional Endoscopy: Protocol for a Multicenter Randomized Controlled Trial. Int J Gen Med. 2022;15:4733-4740. Published 2022 May 6. doi:10.2147/IJGM.S365068IF: 2.1 Q2	Not related to Oral CHM
027<br>1	van Kempen AAMW, Eskes PF, Nuytemans DHGM, et al. Lower versus Traditional Treatment Threshold for Neonatal Hypoglycemia. N Engl J Med. 2020;382(6):534-544. doi:10.1056/NEJMoa1905593	Not related to Oral CHM
027<br>2	Waduthantri S, Yong SS, Tan CH, Htoon HM, Tong L. Lubricant with gelling agent in treating dry eye in adult Chinese patients. Optom Vis Sci. 2012;89(11):1647-1653. doi:10.1097/OPX.0b013e31826cfc41	Not related to Oral CHM
027<br>3	Sadhu A, Upadhyay P, Agrawal A, et al. Management of cognitive determinants in senile dementia of Alzheimer's type: therapeutic potential of a novel polyherbal drug product. Clin Drug Investig. 2014;34(12):857-869. doi:10.1007/s40261-014-0235-9	Not related to Oral CHM
027<br>4	Sadhu A, Upadhyay P, Agrawal A, et al. Management of cognitive determinants in senile dementia of Alzheimer's type: therapeutic potential of a novel polyherbal drug product. Clin Drug Investig. 2014;34(12):857-869. doi:10.1007/s40261-014-0235-9	Not related to Oral CHM
027<br>5	Management of diabetes, obesity, and gastric disorders using the “integrated approach” at health total: A case study	Not related to Oral CHM

027 6	Pang Y, Wang K, Chen S, et al. Massage for simple obesity: A protocol for systematic review. Medicine (Baltimore). 2021;100(6):e24336. doi:10.1097/MD.0000000000024336	Not related to Oral CHM
027 7	Tonstad, S., Rössner, S., Rissanen, A., & Astrup, A. (2016). Medical management of obesity in Scandinavia 2016. Obesity Medicine, 1, 38-44.	Not related to Oral CHM
027 8	Zheng GQ, Zhao ZM, Wang Y, et al. Meta-analysis of scalp acupuncture for acute hypertensive intracerebral hemorrhage. J Altern Complement Med. 2011;17(4):293-299. doi:10.1089/acm.2010.0156	Not related to Oral CHM
027 9	Wu J, Zhou Y, Wang G. Metformin Use and Survival in Patients with Advanced Extrahepatic Cholangiocarcinoma: A Single-Center Cohort Study in Fuyang, China. Gastroenterol Res Pract. 2021;2021:9468227. Published 2021 Oct 29. doi:10.1155/2021/9468227	Not related to Oral CHM
028 0	Micco N, Gold B, Buzzell P, Leonard H, Pintauro S, Harvey-Berino J. Minimal in-person support as an adjunct to internet obesity treatment. Ann Behav Med. 2007;33(1):49-56. doi:10.1207/s15324796abm3301_6	Not related to Oral CHM
028 1	Peter EL, Kasali FM, Deyno S, et al. Momordica charantia L. lowers elevated glycaemia in type 2 diabetes mellitus patients: Systematic review and meta-analysis. J Ethnopharmacol. 2019;231:311-324. doi:10.1016/j.jep.2018.10.033	Not related to Oral CHM
028 2	Xiao X, Deng X, Zhang G, et al. Monitoring of the regulatory ability and regulatory state of the autonomic nervous system and its application to the management of hypertensive patients: a study protocol for randomised controlled trials. BMJ Open. 2023;13(6):e063434. Published 2023 Jun 7. doi:10.1136/bmjopen-2022-063434 IF: 2.4 Q1 B3	Not related to Oral CHM
028 3	Wang LH, Huang W, Zhou W, et al. Moxibustion combined with characteristic lifestyle intervention of Traditional Chinese Medicine in the treatment of abdominal obesity: A study protocol for a randomized controlled trial. Medicine (Baltimore). 2020;99(43):e22855. doi:10.1097/MD.0000000000022855 IF: 1.3 Q2 B4	Not related to Oral CHM
028 4	Tan Y, Hu J, Pang B, et al. Moxibustion for the treatment of diabetic peripheral neuropathy: A systematic review and meta-analysis following PRISMA guidelines. Medicine (Baltimore). 2020;99(39):e22286. doi:10.1097/MD.0000000000022286 IF: 1.3 Q2 B4	Not related to Oral CHM
028 5	Zhang T F, Wan W J, Zhang H X, et al. Multi-center observation of electroacupuncture at Fenglong point in the treatment of hyperlipidemia[J]. 2006.	Not related to Oral CHM
028 6	Zhou J, Li H, Zhang X, et al. Nateglinide and acarbose are comparably effective reducers of postprandial glycemic excursions in chinese antihyperglycemic agent-naive subjects with type 2 diabetes. Diabetes Technol Ther. 2013;15(6):481-488. doi:10.1089/dia.2013.0046	Not related to Oral CHM
028 7	Yaribeygi H, Atkin SL, Sahebkar A. Natural compounds with DPP-4 inhibitory effects: Implications for the treatment of diabetes. J Cell Biochem. 2019;120(7):10909-10913. doi:10.1002/jcb.28467	Not related to Oral CHM

028 8	Seely, D., Szczurko, O., Cooley, K., Fritz, H., Aberdour, S., Herrington, C., ... & Guyatt, G. (2013). Naturopathic medicine for the prevention of cardiovascular disease: a randomized clinical trial. Cmaj, 185(9), E409-E416.	Not related to Oral CHM
028 9	Wang ZY, Li XY, Gou XJ, et al. Network Meta-Analysis of Acupoint Catgut Embedding in Treatment of Simple Obesity. Evid Based Complement Alternat Med. 2022;2022:6408073. Published 2022 May 23. doi:10.1155/2022/6408073 IF: NA NA NA	Not related to Oral CHM
029 0	Palmer R, Braden B. New and emerging endoscopic haemostasis techniques. Frontline Gastroenterol. 2015;6(2):147-152. doi:10.1136/flgastro-2014-100540	Not related to Oral CHM
029 1	Reid S, Cawthon PM, Craig JC, Samuels JA, Molony DA, Strippoli GF. Non-immunosuppressive treatment for IgA nephropathy. Cochrane Database Syst Rev. 2011;(3):CD003962. Published 2011 Mar 16. doi:10.1002/14651858.CD003962.pub2	Not related to Oral CHM
029 2	Zhan HR, Hong ZS, Chen YS, et al. Non-invasive treatment to grade 1 essential hypertension by percutaneous laser and electric pulse to acupoint with music: A randomized controlled trial. Chin J Integr Med. 2016;22(9):696-703. doi:10.1007/s11655-016-2502-5	Not related to Oral CHM
029 3	Shankar P, Ahuja S, Sriram K. Non-nutritive sweeteners: review and update. Nutrition. 2013;29(11-12):1293-1299. doi:10.1016/j.nut.2013.03.024	Not related to Oral CHM
029 4	Martínez-Abundis E, Méndez-Del Villar M, Pérez-Rubio KG, et al. Novel nutraceutic therapies for the treatment of metabolic syndrome. World J Diabetes. 2016;7(7):142-152. doi:10.4239/wjd.v7.i7.142	Not related to Oral CHM
029 5	Santini A, Novellino E. Nutraceuticals in hypercholesterolaemia: an overview. Br J Pharmacol. 2017;174(11):1450-1463. doi:10.1111/bph.13636	Not related to Oral CHM
029 6	WANG, Y. L., Xin, C. A. O., LIU, Z. C., & Bin, X. U. (2013). Observation on the therapeutic effect of electroacupuncture on simple obesity of gastrointestinal heat pattern/syndrome. World Journal of Acupuncture-Moxibustion, 23(2), 1-5.	Not related to Oral CHM
029 7	Zhang P, Liu ZF, Wang CM, Yao SZ, Zhang ZL. Zhongguo Zhen Jiu. 2007;27(4):258-260.	Not related to Oral CHM
029 8	Yin LL, Li YH, Wang SX. Zhongguo Zhen Jiu. 2008;28(6):402-404.	Not related to Oral CHM
029 9	Ng JS, Fan DS, Young AL, et al. Ocular hypertensive response to topical dexamethasone in children: a dose-dependent phenomenon. Ophthalmology. 2000;107(11):2097-2100. doi:10.1016/s0161-6420(00)00357-2	Not related to Oral CHM
030 0	Montemurro, V. (2018). Open-label study on the effect of AISURICIN™, containing Quercetin, Prunus Cerasus and Vitamin C in adults with asymptomatic hyperuricemia and grade 1 borderline hypertension. Gazzetta Medica Italiana Archivio per le Scienze Mediche, 177(9), 458-67.	Not related to Oral CHM

030 1	Shi YF, Pan CY, Hill J, Gao Y. Orlistat in the treatment of overweight or obese Chinese patients with newly diagnosed Type 2 diabetes. Diabet Med. 2005;22(12):1737-1743. doi:10.1111/j.1464-5491.2005.01723.x	Not related to Oral CHM
030 2	Wannemuehler TJ, Rubel KE, Hendricks BK, et al. Outcomes in transcranial microsurgery versus extended endoscopic endonasal approach for primary resection of adult craniopharyngiomas. Neurosurg Focus. 2016;41(6):E6. doi:10.3171/2016.9.FOCUS16314	Not related to Oral CHM
030 3	Baradaran A, Nasri H, Rafieian-Kopaei M. Oxidative stress and hypertension: Possibility of hypertension therapy with antioxidants. J Res Med Sci. 2014;19(4):358-367.	Not related to Oral CHM
030 4	Sakalis, V., Gkotsi, A., Mpolosis, N., Stratis, M., Charpidou, D., Tsafrakidis, P., & Apostolidis, A. (2021). A meta-analysis of the effect of urological and non-urological medication on morphometric parameters of the prostate. European Urology, 79, S50-S51.	Not related to Oral CHM
030 5	Norman Å, Zeebari Z, Nyberg G, Elinder LS. Parental support in promoting children's health behaviours and preventing overweight and obesity - a long-term follow-up of the cluster-randomised healthy school start study II trial. BMC Pediatr. 2019;19(1):104. Published 2019 Apr 11. doi:10.1186/s12887-019-1467-x	Not related to Oral CHM
030 6	Herold Z, Herold M, Nagy P, Patocs A, Doleschall M, Somogyi A. Serum chromogranin A level continuously rises with the progression of type 1 diabetes, and indicates the presence of both enterochromaffin-like cell hyperplasia and autoimmune gastritis. J Diabetes Investig. 2020;11(4):865-873. doi:10.1111/jdi.13203	Not related to Oral CHM
030 7	Xu M, Chen H, Shi ZX, et al. Pathological Observation of Blood Stasis Syndrome in Non-diabetic Peripheral Neuropathies: A Retrospective Analysis Based on Nerve Biopsy. Chin J Integr Med. 2020;26(10):776-782. doi:10.1007/s11655-019-3045-3	Not related to Oral CHM
030 8	Terui T, Iwai-Takano M, Watanabe T. Permanent Pacemaker Implantation in a Patient with Takotsubo Cardiomyopathy and Complete Atrioventricular Block. Case Rep Cardiol. 2021;2021:6637720. Published 2021 Apr 2. doi:10.1155/2021/6637720	Not related to Oral CHM
030 9	Wen MS, Chang KC, Lee TH, et al. Pharmacogenetic dosing of warfarin in the Han-Chinese population: a randomized trial. Pharmacogenomics. 2017;18(3):245-253. doi:10.2217/pgs-2016-0154	Not related to Oral CHM
031 0	Oelke, M., Hofner, K., Berges, R. R., & Jonas, U. (2002). Pharmacological treatment of the benign prostatic syndrome (symptomatic BPH) using alpha1-adrenoceptor antagonists. Basic principles and clinical results. Der Urologe A, 41(5), 425-441.	Not related to Oral CHM
031 1	Rossen J, Yngve A, Hagströmer M, et al. Physical activity promotion in the primary care setting in pre- and type 2 diabetes - the Sophia step study, an RCT. BMC Public Health. 2015;15:647. Published 2015 Jul 12. doi:10.1186/s12889-015-1941-9	Not related to Oral CHM

0312	Zhang J, Cao J, Zhang H, et al. Plasma copper and the risk of first stroke in hypertensive patients: a nested case-control study. Am J Clin Nutr. 2019;110(1):212-220. doi:10.1093/ajcn/nqz099	Not related to Oral CHM
0313	Zhong C, Xu T, Xu T, et al. Plasma Homocysteine and Prognosis of Acute Ischemic Stroke: a Gender-Specific Analysis From CATIS Randomized Clinical Trial. Mol Neurobiol. 2017;54(3):2022-2030. doi:10.1007/s12035-016-9799-0	Not related to Oral CHM
0314	Yu Y, Zhang H, Song Y, et al. Plasma retinol and the risk of first stroke in hypertensive adults: a nested case-control study [published correction appears in Am J Clin Nutr. 2019 Jun 1;109(6):1746. doi: 10.1093/ajcn/nqz065.]. Am J Clin Nutr. 2019;109(2):449-456. doi:10.1093/ajcn/nqy320	Not related to Oral CHM
0315	Wang Z, Ma H, Song Y, et al. Plasma selenium and the risk of first stroke in adults with hypertension: a secondary analysis of the China Stroke Primary Prevention Trial. Am J Clin Nutr. 2022;115(1):222-231. doi:10.1093/ajcn/nqab320	Not related to Oral CHM
0316	Rogers MS, Hung C, Arumanayagam M. Platelet angiotensin II receptor status during pregnancy in Chinese women at high-risk of developing pregnancy-induced hypertension. Gynecol Obstet Invest. 1996;42(2):88-94. doi:10.1159/000291898	Not related to Oral CHM
0317	You J, Li H, Guo W, et al. Platelet function testing guided antiplatelet therapy reduces cardiovascular events in Chinese patients with ST-segment elevation myocardial infarction undergoing percutaneous coronary intervention: The PATROL study. Catheter Cardiovasc Interv. 2020;95 Suppl 1:598-605. doi:10.1002/ccd.28712	Not related to Oral CHM
0318	Rodriguez-Ramiro I, Vauzour D, Minihane AM. Polyphenols and non-alcoholic fatty liver disease: impact and mechanisms. Proc Nutr Soc. 2016;75(1):47-60. doi:10.1017/S0029665115004218	Not related to Oral CHM
0319	Shin HS, Kindleysides S, Yip W, Budgett SC, Ingram JR, Poppitt SD. Postprandial effects of a polyphenolic grape extract (PGE) supplement on appetite and food intake: a randomised dose-comparison trial. Nutr J. 2015;14:96. Published 2015 Sep 14. doi:10.1186/s12937-015-0085-1	Not related to Oral CHM
0320	Xu S, Scott CAB, Coleman RL, Tuomilehto J, Holman RR. Predicting the risk of developing type 2 diabetes in Chinese people who have coronary heart disease and impaired glucose tolerance. J Diabetes. 2021;13(10):817-826. doi:10.1111/1753-0407.13175	Not related to Oral CHM
0321	Zheng FM, Ren YZ, Zhao TF. Zhongguo Zhong Xi Yi Jie He Za Zhi. 2005;25(5):419-421.	Not related to Oral CHM
0322	Shah, K., Shah, J., & Rao, S. (2023). PREVALENCE AND TIMING OF CARDIAC COMORBIDITIES IN TAKOTSUBO CARDIOMYOPATHY: A CASE CONTROL STUDY IN THE ALL OF US RESEARCH PROGRAM. Journal of the American College of Cardiology, 81(8_Supplement), 658-658.	Not related to Oral CHM

0323	Wang JG, Staessen JA, Fagard RH, Birkenhäger WH, Gong L, Liu L. Prognostic significance of serum creatinine and uric acid in older Chinese patients with isolated systolic hypertension. Hypertension. 2001;37(4):1069-1074. doi:10.1161/01.hyp.37.4.1069	Not related to Oral CHM
0324	Wabnitz AM, Chandler J, Treiber F, et al. Program to Avoid Cerebrovascular Events through Systematic Electronic Tracking and Tailoring of an Eminent Risk factor: Protocol of a RCT. J Stroke Cerebrovasc Dis. 2021;30(8):105815. doi:10.1016/j.jstrokecerebrovasdis.2021.105815	Not related to Oral CHM
0325	Wang H, Deng JL, Yue J, Li J, Hou YB. Prostaglandin E1 for preventing the progression of diabetic kidney disease. Cochrane Database Syst Rev. 2010;(5):CD006872. Published 2010 May 12. doi:10.1002/14651858.CD006872.pub2	Not related to Oral CHM
0326	Rafailidis PI, Polyzos KA, Sgouros K, Falagas ME. Prulifloxacin: a review focusing on its use beyond respiratory and urinary tract infections. Int J Antimicrob Agents. 2011;37(4):283-290. doi:10.1016/j.ijantimicag.2010.11.032	Not related to Oral CHM
0327	Zhang ZL, Ji XQ, Zhang P, Zhang XH, Meng ZJ, Yang XJ. Zhongguo Zhen Jiu. 2007;27(12):875-880.	Not related to Oral CHM
0328	Tian FS, Yang WG, Song HL, et al. Zhongguo Zhen Jiu. 2008;28(2):84-86.	Not related to Oral CHM
0329	Zhang ZL, Ji XQ, Zhao SH, Zhang JJ, Kang T, Yang XJ. Zhongguo Zhen Jiu. 2008;28(9):629-633.	Not related to Oral CHM
0330	Zou, B., Guo, X., Liu, Y., Zou, R., Li, G., Liu, J., ... & Xiong, Z. (2018). Randomized controlled trial of continuous positive airway pressure treatment of resistant hypertensive patients combined with obstructive sleep apnea/hypopnea syndrome. Int J Clin Exp Med, 11(11), 11965-11972.	Not related to Oral CHM
0331	Zhu Y, Xu C, Liu J. Randomized controlled trial of genotype-guided warfarin anticoagulation in Chinese elderly patients with nonvalvular atrial fibrillation. J Clin Pharm Ther. 2020;45(6):1466-1473. doi:10.1111/jcpt.13218	Not related to Oral CHM
0332	Miao Z, Jiang L, Wu H, et al. Randomized controlled trial of symptomatic middle cerebral artery stenosis: endovascular versus medical therapy in a Chinese population. Stroke. 2012;43(12):3284-3290. doi:10.1161/STROKEAHA.112.662270	Not related to Oral CHM
0333	Sangsuwan C, Udompanthurak S, Vannasaeng S, Thamlikitkul V. Randomized controlled trial of Tinospora crispa for additional therapy in patients with type 2 diabetes mellitus. J Med Assoc Thai. 2004;87(5):543-546.	Not related to Oral CHM
0334	Zhang S, Wang D, Li L. Recombinant tissue-type plasminogen activator (rt-PA) effectively restores neurological function and improves prognosis in acute ischemic stroke. Am J Transl Res. 2023;15(5):3460-3467. Published 2023 May 15.	Not related to Oral CHM

0335	Wang L, Xu E, Ren S, Gu X, Zheng J, Yang J. Reduced glutathione does not further reduce contrast-induced nephropathy in elderly patients with diabetes receiving percutaneous coronary intervention. J Int Med Res. 2020;48(11):300060520964017. doi:10.1177/0300060520964017	Not related to Oral CHM
0336	Roongpisuthipong C, Kantawan R, Roongpisuthipong W. Reduction of adipose tissue and body weight: effect of water soluble calcium hydroxycitrate in Garcinia atroviridis on the short term treatment of obese women in Thailand. Asia Pac J Clin Nutr. 2007;16(1):25-29.	Not related to Oral CHM
0337	Su Q, Liu J, Li P, Qian L, Yang W. Relative Contribution of Fasting and Postprandial Blood Glucose in Overall Glycemic Control: Post Hoc Analysis of a Phase IV Randomized Trial. Diabetes Ther. 2018;9(3):987-999. doi:10.1007/s13300-018-0403-7	Not related to Oral CHM
0338	Shen PC, He LQ, Yang XJ, Cao HX. Renal protection of losartan 50 mg in normotensive Chinese patients with nondiabetic chronic kidney disease. J Investig Med. 2012;60(7):1041-1047. doi:10.2310/JIM.0b013e31826741d2	Not related to Oral CHM
0339	Zhao L, Leung KF, Liu FB, Chen J, Chan K. Responsiveness of the Chinese Quality of Life Instrument in patients with congestive heart failure. Chin J Integr Med. 2008;14(3):173-179. doi:10.1007/s11655-008-0173-6	Not related to Oral CHM
0340	Zuo HY, Chen Y. Retinervus luffae fructus (RLF): a novel material for use in negative pressure wound therapy. J Wound Care. 2014;23(2):81-87. doi:10.12968/jowc.2014.23.2.81	Not related to Oral CHM
0341	Zhou J, Cao X, Lin H, et al. Safety and effectiveness evaluation of a domestic peritoneal dialysis fluid packed in non-PVC bags: study protocol for a randomized controlled trial. Trials. 2015;16:592. Published 2015 Dec 29. doi:10.1186/s13063-015-1131-1	Not related to Oral CHM
0342	Ye SL, Chen X, Yang J, et al. Safety and efficacy of sorafenib therapy in patients with hepatocellular carcinoma: final outcome from the Chinese patient subset of the GIDEON study. Oncotarget. 2016;7(6):6639-6648. doi:10.18632/oncotarget.6781	Not related to Oral CHM
0343	Vuksan V, Jenkins AL, Brissette C, et al. Salba-chia (Salvia hispanica L.) in the treatment of overweight and obese patients with type 2 diabetes: A double-blind randomized controlled trial. Nutr Metab Cardiovasc Dis. 2017;27(2):138-146. doi:10.1016/j.numecd.2016.11.124	Not related to Oral CHM
0344	Rondanelli M, Klersy C, Iadarola P, Monteferrario F, Opizzi A. Satiety and amino-acid profile in overweight women after a new treatment using a natural plant extract sublingual spray formulation. Int J Obes (Lond). 2009;33(10):1174-1182. doi:10.1038/ijo.2009.155	Not related to Oral CHM
0345	Spinu, S., ORȚAN, A., & VOAIDEȘ, C. (2022). SHORT REVIEW ON BIOACTIVE COMPOUNDS RECOVERED FROM VEGETAL WASTE AS A NEW TOOL IN DIABETES, OBESITY AND DYSLIPIDAEMIA PREVENTION AND TREATMENT. Farmacia, 70(6).	Not related to Oral CHM

0346	Park SU, Ko CN, Bae HS, et al. Short-term reactions to acupuncture treatment and adverse events following acupuncture: a cross-sectional survey of patient reports in Korea. J Altern Complement Med. 2009;15(12):1275-1283. doi:10.1089/acm.2009.0181	Not related to Oral CHM
0347	Perimenis P, Karkoulias K, Konstantinopoulos A, et al. Sildenafil versus continuous positive airway pressure for erectile dysfunction in men with obstructive sleep apnea: a comparative study of their efficacy and safety and the patient's satisfaction with treatment. Asian J Androl. 2007;9(2):259-264. doi:10.1111/j.1745-7262.2007.00085.x	Not related to Oral CHM
0348	Tu JF, Kang SB, Wang LQ, et al. Smart phone-based transcutaneous electrical acupoint stimulation as adjunctive therapy for hypertension (STAT-H trial): protocol for a cluster randomised controlled trial. BMJ Open. 2022;12(7):e058172. Published 2022 Jul 28. doi:10.1136/bmjopen-2021-058172	Not related to Oral CHM
0349	Shi M, Wang A, Fang Y, et al. Study on the pathogenesis of Holmes tremor by multimodal 3D medical imaging: case reports of three patients. BMC Neurol. 2021;21(1):473. Published 2021 Dec 6. doi:10.1186/s12883-021-02503-2	Not related to Oral CHM
0350	Zhou Z, Zhang X, Cui F, et al. Subacute motor neuron hyperexcitability with mercury poisoning: a case series and literature review. Eur Neurol. 2014;72(3-4):218-222. doi:10.1159/000363290	Not related to Oral CHM
0351	Vuksan V, Whitham D, Sievenpiper JL, et al. Supplementation of conventional therapy with the novel grain Salba (Salvia hispanica L.) improves major and emerging cardiovascular risk factors in type 2 diabetes: results of a randomized controlled trial. Diabetes Care. 2007;30(11):2804-2810. doi:10.2337/dc07-1144	Not related to Oral CHM
0352	Massa NM, Silva AS, de Oliveira CV, et al. Supplementation with Watermelon Extract Reduces Total Cholesterol and LDL Cholesterol in Adults with Dyslipidemia under the Influence of the MTHFR C677T Polymorphism. J Am Coll Nutr. 2016;35(6):514-520. doi:10.1080/07315724.2015.1065522	Not related to Oral CHM
0353	Yao P, Meng LX, Ma JM, et al. Sustained-release oxycodone tablets for moderate to severe painful diabetic peripheral neuropathy: a multicenter, open-labeled, postmarketing clinical observation. Pain Med. 2012;13(1):107-114. doi:10.1111/j.1526-4637.2011.01274.x	Not related to Oral CHM
0354	ZHANG, X. P., JIA, C. S., WANG, J. L., Jing, S. H. I., & ZHANG, X. (2013). Systematic review on the effectiveness of embedding catgut therapy for simple obesity. World Journal of Acupuncture-Moxibustion, 23(3), 53-58.	Not related to Oral CHM
0355	Younossi ZM, Reyes MJ, Mishra A, Mehta R, Henry L. Systematic review with meta-analysis: non-alcoholic steatohepatitis - a case for personalised treatment based on pathogenic targets. Aliment Pharmacol Ther. 2014;39(1):3-14. doi:10.1111/apt.12543	Not related to Oral CHM

0356	Pan X, Tian L, Yang F, et al. Tai Chi as a Therapy of Traditional Chinese Medicine on Reducing Blood Pressure: A Systematic Review of Randomized Controlled Trials. Evid Based Complement Alternat Med. 2021;2021:4094325. Published 2021 Sep 4. doi:10.1155/2021/4094325	Not related to Oral CHM
0357	Yeh GY, Wang C, Wayne PM, Phillips R. Tai chi exercise for patients with cardiovascular conditions and risk factors: A SYSTEMATIC REVIEW. J Cardiopulm Rehabil Prev. 2009;29(3):152-160. doi:10.1097/HCR.0b013e3181a33379	Not related to Oral CHM
0358	Patel A, Mirza N, Ali R, Rayad MN, Ahmad A, Khan A. Takotsubo Cardiomyopathy After Cocaine Intoxication. Eur J Case Rep Intern Med. 2022;9(9):003457. Published 2022 Sep 2. doi:10.12890/2022_003457	Not related to Oral CHM
0359	Schmitz, G, Hlaing, M, Papazian, J and Seres, T, 2017. Takotsubo cardiomyopathy after ropivacaine-induced local anesthetic systemic toxicity, Regional Anesthesia and Pain Medicine.	Not related to Oral CHM
0360	Ulbeh, T. M., Sara, A., Uddin, M. M., Bell, K., Elmograbi, A., & Cardozo, S. (2021). Takotsubo cardiomyopathy caused by infusion reaction to paclitaxel. BMJ Case Reports CP, 14(8), e243863.	Not related to Oral CHM
0361	Soni, P., Chadha, S., Aggarwal, N., Rai, A. K., Kupfer, Y., & Mailk, B. (2017). Takotsubo Cardiomyopathy With Left Ventricular Outflow Tract Obstruction and Shock. Chest, 152(4), A93.	Not related to Oral CHM
0362	Michel MC, Bressel HU, Mehlburger L, Goepel M. Tamsulosin: real life clinical experience in 19,365 patients. Eur Urol. 1998;34 Suppl 2:37-45. doi:10.1159/000052286	Not related to Oral CHM
0363	Rigby MR, DiMeglio LA, Rendell MS, et al. Targeting of memory T cells with alefacept in new-onset type 1 diabetes (T1DAL study): 12 month results of a randomised, double-blind, placebo-controlled phase 2 trial. Lancet Diabetes Endocrinol. 2013;1(4):284-294. doi:10.1016/S2213-8587(13)70111-6	Not related to Oral CHM
0364	MAYR, H., THOMAS, C., & ITSIOPOULOS, C. (2016). THE AUSMED HEART TRIAL: IMPLEMENTING AN AUSTRALIAN MEDITERRANEAN DIET FOR SECONDARY PREVENTION OF CORONARY HEART DISEASE: 243. Nutrition & Dietetics, 73, 76.	Not related to Oral CHM
0365	Qin Y, Xia W, Huang W, Zhang J, Zhao Y, Fang M. The Beneficial Effect of Traditional Chinese Exercises on the Management of Obesity. Evid Based Complement Alternat Med. 2020;2020:2321679. Published 2020 Oct 1. doi:10.1155/2020/2321679	Not related to Oral CHM
0366	Zhang C. The brain-resuscitation acupuncture method for treatment of post wind-stroke mental depression--a report of 45 cases. J Tradit Chin Med. 2005;25(4):243-246.	Not related to Oral CHM
0367	Xia Y, Li W, Li Y, et al. The clinical value of the changes of peripheral lymphocyte subsets absolute counts in patients with non-small cell lung cancer. Transl Oncol. 2020;13(12):100849. doi:10.1016/j.tranon.2020.100849	Not related to Oral CHM

036<br>8	Talati R, Sobieraj DM, Makanji SS, Phung OJ, Coleman CI. The comparative efficacy of plant sterols and stanols on serum lipids: a systematic review and meta-analysis. J Am Diet Assoc. 2010;110(5):719-726. doi:10.1016/j.jada.2010.02.011	Not related to Oral CHM
036<br>9	Xie, J., Dai, L., & Tang, X. (2017). The comparison of the safety and effectiveness of multiple insulin injections and insulin pump therapy in treating gestational diabetes. Biomed Res, 28(18), 7830-7833.	Not related to Oral CHM
037<br>0	Zaidi, Z. E. H. R. A. (2021). The Concept and Management of Wajaul Mafasil in Unani Medicine. Asian Journal of Pharmaceutical and Clinical Research, 7-13.	Not related to Oral CHM
037<br>1	Pan CY, Li WH, Zeng JE, et al. Zhonghua Nei Ke Za Zhi. 2013;52(11):932-935.	Not related to Oral CHM
037<br>2	Zheng, C., Zhang, X., Wang, J., Wang, Y., Qi, L., Liu, N., & Xia, L. (2021). The effect of 12-week Liuzijue exercise training on patients with hypertension: A randomized, controlled clinical trial. ACTA Medica Mediterranea, 37(5), 2713-2721.	Not related to Oral CHM
037<br>3	Vetter, K, Di Donato, K, May, J, Hartwig, D and Liu, Y, 2012. The effect of a medication adherence or an education program on health outcomes of hypertensive patients in a community pharmacy setting, Journal of the american pharmacists association	Not related to Oral CHM
037<br>4	Scholle JM, Baker WL, Talati R, Coleman CI. The effect of adding plant sterols or stanols to statin therapy in hypercholesterolemic patients: systematic review and meta-analysis. J Am Coll Nutr. 2009;28(5):517-524. doi:10.1080/07315724.2009.10719784	Not related to Oral CHM
037<br>5	Shen C, Pang SM, Kwong EW, Cheng Z. The effect of Chinese food therapy on community dwelling Chinese hypertensive patients with Yin-deficiency. J Clin Nurs. 2010;19(7-8):1008-1020. doi:10.1111/j.1365-2702.2009.02937.x	Not related to Oral CHM
037<br>6	Song J, Wei L, Cheng K, et al. The Effect of Modified Tai Chi Exercises on the Physical Function and Quality of Life in Elderly Women With Knee Osteoarthritis. Front Aging Neurosci. 2022;14:860762. Published 2022 May 26. doi:10.3389/fnagi.2022.860762	Not related to Oral CHM
037<br>7	Peters HPF, Koppenol W, Schuring EAH, Gouka R, Mela DJ, Blom WAM. The effect of two weeks ingestion of a bitter tastant mixture on energy intake in overweight females. Appetite. 2016;107:268-273. doi:10.1016/j.appet.2016.08.013	Not related to Oral CHM
037<br>8	Yu X, Chau JPC, Huo L. The effectiveness of traditional Chinese medicine-based lifestyle interventions on biomedical, psychosocial, and behavioral outcomes in individuals with type 2 diabetes: A systematic review with meta-analysis. Int J Nurs Stud. 2018;80:165-180. doi:10.1016/j.ijnurstu.2018.01.009	Not related to Oral CHM
037<br>9	Sejari, N., Kamaruddin, K., & Ming, L. C. (2014). The effectiveness of traditional Malay massage: a narrative review. Archives of Pharmacy Practice, 5(4-2014), 144-148.	Not related to Oral CHM

038<br>0	Oben J, Enonchong E, Kuate D, et al. The effects of ProAlgaZyme novel algae infusion on metabolic syndrome and markers of cardiovascular health. Lipids Health Dis. 2007;6:20. Published 2007 Sep 5. doi:10.1186/1476-511X-6-20	Not related to Oral CHM
038<br>1	Zhang J, Tsai TF, Lee MG, et al. The efficacy and safety of tofacitinib in Asian patients with moderate to severe chronic plaque psoriasis: A Phase 3, randomized, double-blind, placebo-controlled study. J Dermatol Sci. 2017;88(1):36-45. doi:10.1016/j.jdermsci.2017.05.004	Not related to Oral CHM
038<br>2	Yan H, An Y, Zhang T, Zhao J, Yan J. The efficacy and safety of Tuina for diabetic gastroparesis: A protocol for systematic review and meta-analysis. Medicine (Baltimore). 2021;100(47):e27964. doi:10.1097/MD.0000000000027964	Not related to Oral CHM
038<br>3	Peng S, Zhang X, Liu Y, et al. The efficacy of 5-element therapy for senile diabetes with depression: A protocol for a systematic review and meta-analysis. Medicine (Baltimore). 2020;99(50):e23622. doi:10.1097/MD.0000000000023622	Not related to Oral CHM
038<br>4	Moretti C, Cerrato E, Cavallero E, et al. The EUROpean and Chinese cardiac and renal Remote Ischemic Preconditioning Study (EURO-CRIPS CardioGroup I): A randomized controlled trial. Int J Cardiol. 2018;257:1-6. doi:10.1016/j.ijcard.2017.12.033	Not related to Oral CHM
038<br>5	Zhang X, Cao D, Yan M, Liu M. The feasibility of Chinese massage as an auxiliary way of replacing or reducing drugs in the clinical treatment of adult type 2 diabetes: A systematic review and meta-analysis. Medicine (Baltimore). 2020;99(34):e21894. doi:10.1097/MD.0000000000021894	Not related to Oral CHM
038<br>6	Mazokopakis EE, Starakis IK, Papadomanolaki MG, Mavroeidi NG, Ganotakis ES. The hypolipidaemic effects of Spirulina (Arthrospira platensis) supplementation in a Cretan population: a prospective study. J Sci Food Agric. 2014;94(3):432-437. doi:10.1002/jsfa.6261	Not related to Oral CHM
038<br>7	Wilson ML, Davies IG, Waraksa W, Khayyatzadeh SS, Al-Asmakh M, Mazidi M. The Impact of Microbial Composition on Postprandial Glycaemia and Lipidaemia: A Systematic Review of Current Evidence. Nutrients. 2021;13(11):3887. Published 2021 Oct 29. doi:10.3390/nu13113887	Not related to Oral CHM
038<br>8	Ramachandran A, Snehalatha C, Mary S, et al. The Indian Diabetes Prevention Programme shows that lifestyle modification and metformin prevent type 2 diabetes in Asian Indian subjects with impaired glucose tolerance (IDPP-1). Diabetologia. 2006;49(2):289-297. doi:10.1007/s00125-005-0097-z	Not related to Oral CHM
038<br>9	Yang Q, Cao Y, Fang Y, et al. The influence of different debridement methods on the prognosis of elderly patients with diabetic foot ulcers and sepsis. Minerva Chir. 2017;72(3):200-205. doi:10.23736/S0026-4733.16.07157-1	Not related to Oral CHM

0390	Yang X, Zhang M, Song R, Liu C, Huo Y, Qian G. The modifying effect of the MTHFR genotype on the association between folic acid supplementation and pulse wave velocity: Findings from the CSPPT. Cardiovasc Ther. 2018;36(6):e12473. doi:10.1111/1755-5922.12473	Not related to Oral CHM
0391	Ramos-Rodriguez A, Fernandez-Bravo C, Estepa-Pedregosa L, Rodriguez-Gonzalez M. The Pivotal Role of Echocardiography in the Diagnosis of Stress-Induced Cardiomyopathy Presenting with Atypical Pattern in Critically Ill Children. An Illustrative Case Report. Curr Med Imaging. 2022;18(9):1003-1011. doi:10.2174/1573405618666220216121424	Not related to Oral CHM
0392	Radak D, Atanasijević I, Nešković M, Isenovic E. The Significance of Pain in Chronic Venous Disease and its Medical Treatment. Curr Vasc Pharmacol. 2019;17(3):291-297. doi:10.2174/1570161116666180209111826	Not related to Oral CHM
0393	Melchart D, Wühr E, Wifling K, Bachmeier BE. The TALENT II study: a randomized controlled trial assessing the impact of an individual health management (IHM) on stress reduction. BMC Public Health. 2018;18(1):823. Published 2018 Jul 4. doi:10.1186/s12889-018-5756-3	Not related to Oral CHM
0394	Abou Ziki, M., Bikdeli, B., & Lip, G. (2016). The Yin-Yang of Atrial Fibrillation and Pulmonary Embolism. Chest, 150(4), 1189A.	Not related to Oral CHM
0395	Xu XL, Zhao T, Huang YQ, et al. Therapeutic lumbar puncture and lumbar drainage: which is more effective for the management of intracranial hypertension in HIV patients with cryptococcal meningitis? Results of a prospective non-randomized interventional study in China. Curr Med Res Opin. 2022;38(5):803-810. doi:10.1080/03007995.2022.2047539	Not related to Oral CHM
0396	Xiao B, Fan Y, Zhang Z, et al. Three-Dimensional Radiomics Features From Multi-Parameter MRI Combined With Clinical Characteristics Predict Postoperative Cerebral Edema Exacerbation in Patients With Meningioma. Front Oncol. 2021;11:625220. Published 2021 Apr 15. doi:10.3389/fonc.2021.625220	Not related to Oral CHM
0397	Zhang LD, Han W, Gao ZB, et al. Zhongguo Zhen Jiu. 2022;42(2):121-125. doi:10.13703/j.0255-2930.20210202-k0007	Not related to Oral CHM
0398	Wang T, Jin H, Wang Y, et al. Traditional Chinese exercise for non-valvular atrial fibrillation: A protocol for systematic review and meta-analysis. Medicine (Baltimore). 2022;101(49):e31829. doi:10.1097/MD.0000000000031829	Not related to Oral CHM
0399	Thornton, T, O'Donoghue, F, Rochford, P, Xue, C, Trinder, J and Jordan, A, 2013. Traditional chinese medicine diagnosis of obstructive sleep apnoea, Sleep and Biological Rhythms.	Not related to Oral CHM
0400	Zou P. Traditional Chinese Medicine, Food Therapy, and Hypertension Control: A Narrative Review of Chinese Literature. Am J Chin Med. 2016;44(8):1579-1594. doi:10.1142/S0192415X16500889	Not related to Oral CHM

040 1	Sharma P, Yadav RK, Khadgawat R, Dada R. Transcriptional modulation of inflammation, and aging in Indian obese adults following a 12-week yoga-based lifestyle intervention: A randomized controlled trial. Front Med (Lausanne). 2022;9:898293. Published 2022 Aug 8. doi:10.3389/fmed.2022.898293	Not related to Oral CHM
040 2	ZHANG, Z. X., ZHANG, Y., LI, S. Y., ZHANG, J. L., LIU, Z. H., CHEN, Y. F., ... & RONG, P. J. (2021). Transcutaneous auricular vagus nerve stimulation for impaired glucose tolerance: a randomized controlled trial Protocol: 经皮耳迷走神经刺激治疗糖耐量受损：随机对照试验研究方案. World Journal of Acupuncture-Moxibustion, 31(2), 160-164.	Not related to Oral CHM
040 3	Toh ST, Han HJ, Tay HN, Kiong KL. Transoral robotic surgery for obstructive sleep apnea in Asian patients: a Singapore sleep centre experience. JAMA Otolaryngol Head Neck Surg. 2014;140(7):624-629. doi:10.1001/jamaoto.2014.926	Not related to Oral CHM
040 4	Snyder MJ, Gibbs LM, Lindsay TJ. Treating Painful Diabetic Peripheral Neuropathy: An Update. Am Fam Physician. 2016;94(3):227-234.	Not related to Oral CHM
040 5	Stewart K, Shakarishvili N, Michalak A, Maschauer EL, Jenkins N, Riha RL. Treating sleep disorders following traumatic brain injury in adults: Time for renewed effort?. Sleep Med Rev. 2022;63:101631. doi:10.1016/j.smrv.2022.101631	Not related to Oral CHM
040 6	Xu, B, 2003. Treatment of adipositas with acupuncture, Chinesische Medizin	Not related to Oral CHM
040 7	Xu T, Zhang XW, Qu XK, et al. Treatment of hyperhomocysteinemia and endothelial dysfunction in renal transplant recipients with B vitamins in the Chinese population. J Urol. 2008;179(3):1190-1194. doi:10.1016/j.juro.2007.10.028	Not related to Oral CHM
040 8	Wei, Q. L., & Liu, Z. C. (2004). Treatment of simple obesity with auricular acupuncture, body acupuncture and combination of auricular and body acupuncture.	Not related to Oral CHM
040 9	Yang W, Xu X, Liu X, et al. Treat-to-target comparison between once daily biphasic insulin aspart 30 and insulin glargine in Chinese and Japanese insulin-naïve subjects with type 2 diabetes. Curr Med Res Opin. 2013;29(12):1599-1608. doi:10.1185/03007995.2013.838155	Not related to Oral CHM
041 0	Peng S, Xie Z, Zhang X, et al. Tuina for diabetes with obesity: Protocol for a systematic review and meta-analysis. Medicine (Baltimore). 2021;100(3):e23918. doi:10.1097/MD.0000000000023918	Not related to Oral CHM
041 1	Wang F, Wang F, Pan T, et al. Tuina for diabetic peripheral neuropathy: A protocol for a systematic review and meta-analysis. Medicine (Baltimore). 2021;100(23):e26222. doi:10.1097/MD.0000000000026222	Not related to Oral CHM
041 2	ZHANG, X. X., TANG, X. D., & LI, W. H. (2012). Twenty-eight cases of simple obesity of spleen and kidney yang deficiency pattern/syndrome in females treated with electroacupuncture and isolated-medicinal moxibustion. World Journal of Acupuncture-Moxibustion, 22(3), 65-68.	Not related to Oral CHM

041<br>3	Qi WW, Liu T, Xu G, et al. Upstream therapeutic strategies of Valsartan and Fluvastatin on Hypertensive patients with non-permanent Atrial Fibrillation (VF-HT-AF): study protocol for a randomized controlled trial. Trials. 2015;16:336. Published 2015 Aug 7. doi:10.1186/s13063-015-0836-5	Not related to Oral CHM
041<br>4	Simmons CV, Banov F, Banov D. Use of a topical anhydrous silicone base containing fatty acids from pracaxi oil in a patient with a diabetic ulcer. SAGE Open Med Case Rep. 2015;3:2050313X15589676. Published 2015 Jun 3. doi:10.1177/2050313X15589676	Not related to Oral CHM
041<br>5	Rosero Arenas MA, Rosero Arenas E, Portaceli Armiñana MA, García García MA. Utilidad de los fitoestrógenos en la reducción de la presión arterial. Revisión sistemática y metaanálisis [Usefulness of phyto-oestrogens in reduction of blood pressure. Systematic review and meta-analysis]. Aten Primaria. 2008;40(4):177-186. doi:10.1157/13118060	Not related to Oral CHM
041<br>6	Pengpid S, Peltzer K. Utilization of complementary and traditional medicine practitioners among middle-aged and older adults in India: results of a national survey in 2017-2018. BMC Complement Med Ther. 2021;21(1):262. Published 2021 Oct 15. doi:10.1186/s12906-021-03432-w	Not related to Oral CHM
041<br>7	Ramelet AA, Boisseau MR, Allegra C, et al. Veno-active drugs in the management of chronic venous disease. An international consensus statement: current medical position, prospective views and final resolution. Clin Hemorheol Microcirc. 2005;33(4):309-319.	Not related to Oral CHM
041<br>8	Yang W, Xing X, Lv X, et al. Vildagliptin added to sulfonylurea improves glycemic control without hypoglycemia and weight gain in Chinese patients with type 2 diabetes mellitus. J Diabetes. 2015;7(2):174-181. doi:10.1111/1753-0407.12169	Not related to Oral CHM
041<br>9	SINGH, S., Rungta, R., Sirkanungo, P., Kumar, A., & Paul, S. (2023). WCN23-0068 A CASE SERIES OF FISH BILE INDUCED ACUTE KIDNEY INJURY IN TROPICS. Kidney International Reports, 8(3), S87.	Not related to Oral CHM
042<br>0	von Haehling S, Stellos K, Qusar N, Gawaz M, Bigalke B. Weight reduction in patients with coronary artery disease: comparison of Traditional Tibetan Medicine and Western diet. Int J Cardiol. 2013;168(2):1509-1515. doi:10.1016/j.ijcard.2013.07.034	Not related to Oral CHM
042<br>1	Shih CK, Chen CM, Hsiao TJ, Liu CW, Li SC. White Sweet Potato as Meal Replacement for Overweight White-Collar Workers: A Randomized Controlled Trial. Nutrients. 2019;11(1):165. Published 2019 Jan 14. doi:10.3390/nu11010165	Not related to Oral CHM
042<br>2	AR, M. A., & Shamsul, A. S. (2012). Why Hypertensive Patients Do Not Comply with Their Treatment?: A Case-Control Study in Perlis, Malaysia. International Medical Journal, 19(1).	Not related to Oral CHM
042<br>3	Peron EP, Marcum ZA, Boyce R, Hanlon JT, Handler SM. Year in review: medication mishaps in the elderly. Am J Geriatr Pharmacother. 2011;9(1):1-10. doi:10.1016/j.amjopharm.2011.01.003	Not related to Oral CHM

042 4	Xu B, Yang Y, Yuan Z, et al. Zotarolimus- and paclitaxel-eluting stents in an all-comer population in China: the RESOLUTE China randomized controlled trial. JACC Cardiovasc Interv. 2013;6(7):664-670. doi:10.1016/j.jcin.2013.03.001	Not related to Oral CHM
042 5	Wang LQ, Chen Z, Zhang K, et al. Zusanli (ST36) Acupoint Injection for Diabetic Peripheral Neuropathy: A Systematic Review of Randomized Controlled Trials. J Altern Complement Med. 2018;24(12):1138-1149. doi:10.1089/acm.2018.0053	Not related to Oral CHM
042 6	王仙,禹江琳,李世雄,等.艾灸治疗脾虚湿阻型单纯性肥胖的Meta分析[J].实用中医内科杂志 ,2022,36(04):51-53+145-147.DOI:10.13729/j.issn.1671-7813.Z20210620	Not related to Oral CHM
042 7	赵珈艺,张志辰,金香兰,等.北京市丰台区部分社区脑卒中高危人群血脂控制现状调查及中医症状证候分析[J].世界中医药,2016,11(01):40-44.	Not related to Oral CHM
042 8	张婷,周仲瑜,毛慧芳,等.不同形式阴阳调理灸治疗脾虚湿阻型单纯性肥胖症疗效观察[J].河北中医,2020,42(04):601-605+609.	Not related to Oral CHM
042 9	王艳花,王健,左菊英,等.冲洗射频消融改良迷宫手术治疗房颤的配合体会[J].现代医学,2004,(06):354-355.	Not related to Oral CHM
043 0	虞晓含,朱燕波,王琦,等.代谢综合征与中医体质类型及相关危险因素关系的Logistic回归分析[J].中华中医药杂志,2015,30(10):3536-3539.	Not related to Oral CHM
043 1	尹华富,张晓冉,赵志华,等.代谢综合征中医证候规律分析[J].河北中医 ,2016,38(11):1615-1620+1624.	Not related to Oral CHM
043 2	杨宇峰,石岩.代谢综合征中医证型与危险因素关系的临床研究[J].中华中医药杂志,2015,30(06):2041-2043.	Not related to Oral CHM
043 3	王玉玺.低血糖诱发急性心肌梗死 1 例[J].现代医药卫生,2007,(22):3477.	Not related to Oral CHM
043 4	许璧瑜,张玉玲,黎玉莹.第三代禁食疗法治疗单纯性肥胖症的效果观察及护理[J].现代临床护理,2019,18(06):34-39.	Not related to Oral CHM
043 5	孙爱娟,李岚,马永文.电针治疗单纯性肥胖症 65 例[J].中国民间疗法 ,2008,(09):8.DOI:10.19621/j.cnki.11-3555/r.2008.09.005	Not related to Oral CHM
043 6	彭子壮,卢晶晶,蔡海荣,等.短暂性脑缺血发作危险因素与中医体质的相关性研究[J].中西医结合心脑血管病杂志,2018,16(03):280-283.	Not related to Oral CHM
043 7	魏群利,刘志诚.耳针、体针、耳体针结合治疗单纯性肥胖(英文)[J].中国临床康复,2004,(21):4357-4359.	Not related to Oral CHM
043 8	赵云华,张天鹏,王国兴,等.感染性心内膜炎合并脑脓肿 1 例临床解析[J].中国医刊,2014,49(09):36-38.	Not related to Oral CHM
043 9	张秀纹,宋美爱,高志萍,等.高密地区空腹血糖受损人群的中医体质类型相关性研究[J].山西中医 ,2015,31(03):46-49.DOI:10.20002/j.issn.1000-7156.2015.03.027.	Not related to Oral CHM
044 0	闫卫红,赵勇,李玉峰,等.高尿酸血症相关因素调研及其中医证候学研究[J].中华中医药杂志,2007,(04):247-249.	Not related to Oral CHM
044 1	王莉娅,张婕,毛袁心,等.高血压病患者中医证型与脑血液循环动力学参数的相关性研究[J].时珍国医国药,2013,24(06):1535-1536.	Not related to Oral CHM
044 2	张叶青,王忆勤,董耀荣,等.高血压病临床特征分型研究[J].中华中医药杂志,2018,33(08):3704-3706.	Not related to Oral CHM

0443	莫霄云,黄琛,梁劲松.高血压病血瘀证患者心血管危险因素的临床研究[J].中医临床研究,2016,8(33):34-35+44.	Not related to Oral CHM
0444	雍苏南,谭元生.高血压病中医证型分布规律及合并病的相关性研究[J].辽宁中医杂志,2018,45(05):914-916+1117.DOI:10.13192/j.issn.1000-1719.2018.05.005.	Not related to Oral CHM
0445	王莉娅,张婕,毛袁心,等.高血压患者中医证型与经颅多普勒指标的相关性研究[J].时珍国医国药,2013,24(07):1799-1800.	Not related to Oral CHM
0446	张晓蕾,胡忠民,石志敏,等.隔药灸肠风穴联合马来酸曲美布汀治疗 2 型糖尿病性腹泻的疗效观察[J].广州中医药大学学报,2020,37(12):2382-2388.DOI:10.13359/j.cnki.gzxbtcm.2020.12.020.	Not related to Oral CHM
0447	王东岩,孙爱洁.针灸治疗单纯性肥胖症临床有效性的Meta分析[J].针灸临床杂志,2014,30(10):69-73.	Not related to Oral CHM
0448	王翰林,陈裕,郭新峰,等.基于互联网体重管理数据库的真实世界肥胖内分泌针灸专病门诊患者体质与体成分相关性研究[J].北京中医药大学学报,2022,45(05):500-506.	Not related to Oral CHM
0449	王庆高,覃裕旺,卢健棋,等.基于聚类分析的高血压中医证型研究[J].中西医结合心脑血管病杂志,2016,14(17):1975-1977.	Not related to Oral CHM
0450	鲜琦琦,代顺心,王政研,等.基于数据挖掘探析单纯性肥胖的针刺辨证及取穴规律[J].四川中医,2020,38(05):220-222.	Not related to Oral CHM
0451	宋冰心,杨峰.基于体质理论指导的针灸治疗单纯性肥胖研究进展[J].江苏中医药,2022,54(07):78-82.DOI:10.19844/j.cnki.1672-397X.2022.07.026.	Not related to Oral CHM
0452	王颖辉,赵进喜,王世东,等.基于因子分析及转移概率分析的糖尿病肾病证候演变规律研究[J].中医杂志,2014,55(15):1317-1322.DOI:10.13288/j.11-2166/r.2014.15.016.	Not related to Oral CHM
0453	肖蕾,欧洋,王建华,等.急性心肌梗死与中医体质类型及不良生活方式的相关性研究[J].中华中医药学刊,2018,36(06):1368-1371.DOI:10.13193/j.issn.1673-7717.2018.06.021.	Not related to Oral CHM
0454	倪代梅, 陈光瑞, 付小奎, & 王朝亮. (2014). 急诊经皮冠状动脉介入治疗术后并发上消化道出血一例. 中国基层医药, (20), 3200-3200.	Not related to Oral CHM
0455	于娓娓,李敬林.进展性脑梗死中医证型系统综述[J].实用中医内科杂志,2014,28(05):1-2+7.DOI:10.13729/j.issn.1671-7813.2014.05.01.	Not related to Oral CHM
0456	赵丽娜,张雪松,石光煜,等.颈动脉硬化斑块内新生血管与高血压中医证型的关系[J].中西医结合心脑血管病杂志,2022,20(01):108-111.	Not related to Oral CHM
0457	叶青花.静滴复方氨基酸(18)致严重过敏反应 1 例[J].西藏科技,2007,(12):45.	Not related to Oral CHM
0458	王永新,李亚军,李健,等.岷县地区 2 型糖尿病患者中医证型与血脂水平的相关性[J].临床医学研究与实践,2019,4(18):185-187.DOI:10.19347/j.cnki.2096-1413.201918075.	Not related to Oral CHM
0459	杨玲玲,王胜萍,蒋焱斐,等.辟谷对人体基本生化指标、经络与体质的影响[J].光明中医,2021,36(18):3090-3093.	Not related to Oral CHM
0460	孙宏泰,王翀.认知功能障碍在不同中医证型高血压患者中的表现差异[J].中国临床康复,2005,(20):63-65.	Not related to Oral CHM
0461	岳萍,斯琴高娃,郑霞,等.三维辨证穴位埋线治疗早中期痰湿体质高血压病 60 例临床观察[J].新疆中医药,2017,35(03):41-43.	Not related to Oral CHM

0462	薛俊磊,李明珠,龚玉珍.上海市宝山区 2 型糖尿病患者的中医体质特点研究[J].上海预防医学,2016,28(07):453-459.DOI:10.19428/j.cnki.sjpm.2016.07.005.	Not related to Oral CHM
0463	肖宜敏,史会林,边泽新,等.烧伤创疡再生医疗技术治疗Wagner 1～4 级糖尿病足临床体会[J].中国烧伤创疡杂志,2019,31(02):91-95.	Not related to Oral CHM
0464	肖宜敏,史会林,边泽新,等.烧伤创疡再生医疗技术治疗Wagner 1～4 级糖尿病足临床体会[J].中国烧伤创疡杂志,2019,31(02):91-95.	Not related to Oral CHM
0465	张林芳,王睿瑞,汪天英,等.社区老年脂肪性肝病患者体质分布及危险因素分析[J].中西医结合肝病杂志,2021,31(06):530-532.	Not related to Oral CHM
0466	饶新华.社区中老年原发性高血压患者的体质辨识及中医非药物疗法干预效果分析[J].新中医,2013,45(05):22-24.DOI:10.13457/j.cnki.jncm.2013.05.006	Not related to Oral CHM
0467	田玲玲,刘利锋,朱彤.身体质量指数与类风湿关节炎中医证型分布规律的相关性分析[J].中医临床研究,2021,13(15):60-62.	Not related to Oral CHM
0468	谢静静,宁静,张燕英,等.深圳地区急性期痛风患者不同中医证型的临床特点分析[J].风湿病与关节炎,2019,8(06):9-12.	Not related to Oral CHM
0469	熊启香, 蔡渊, 2016. 输血导致糖尿病 1 例分析, 医学信息(西安)	Not related to Oral CHM
0470	张静,陆霞,李艳,等.水穴埋线配合隔药饼灸治疗脾虚湿阻型肥胖并发高脂血症随机对照研究[J].四川中医,2017,35(05):189-192.	Not related to Oral CHM
0471	赵波涛,周爱明,陈军玲,等.太极六合穴位埋线治疗脾虚痰湿型糖尿病前期患者的疗效观察[J].浙江中医药大学学报,2020,44(10):1004-1008.DOI:10.16466/j.issn1005-5509.2020.10.017.	Not related to Oral CHM
0472	王家标.探讨 120 例老年慢性心力衰竭患者的病因与治疗方法[J].中国实用医药,2017,12(03):144-145.DOI:10.14163/j.cnki.11-5547/r.2017.03.074.	Not related to Oral CHM
0473	赵燕燕,孙娜.糖耐量减低人群的中医体质研究[J].中国中医药现代远程教育,2014,12(03):140-142.	Not related to Oral CHM
0474	肖文,杨敏,杨东东.糖尿病伴抑郁障碍中医证候特点分析[J].吉林医学,2013,34(09):1733-1734.	Not related to Oral CHM
0475	王连伟,李静艳,王丹.26 例糖尿病患者病因分型诊断分析[J].中国医疗前沿,2012,7(20):55+20.	Not related to Oral CHM
0476	陈志强,王凤丽,王月华,等.糖尿病肾病III、IV期患者中医证型与相关实验室指标的典型相关研究[C]//中国中西医结合学会肾脏疾病专业委员会.中国中西医结合学会肾脏疾病专业委员会 2011 年学术年会暨 2011 年国际中西医结合肾脏病学术会议论文汇编.河北医科大学中西医结合研究所;河北医科大学中医院肾内科;河北医科大学流行病与卫生统计学教研室;,2011:554-555.	Not related to Oral CHM
0477	张先慧,柳红芳.糖尿病肾病III期患者中医证素与实验室指标的典型相关分析[J].现代中西医结合杂志,2017,26(02):122-124+130.	Not related to Oral CHM
0478	肖莉,匡文轩,刘晓清,等.糖尿病视网膜病变中医证型分布频率的Meta分析[J].湖南中医药大学学报,2021,41(03):439-446.	Not related to Oral CHM
0479	王文锐,王波,陈洁,等.糖尿病心脏自主神经病变患者中医证候学观察[J].中华中医药学刊,2016,34(12):3021-3024.DOI:10.13193/j.issn.1673-7717.2016.12.056.	Not related to Oral CHM

0480	王宁.糖尿病足大截肢患者临床特征及危险因素分析[D].北京中医药大学,2019.	Not related to Oral CHM
0481	赵挺洋,徐丽梅,马建伟,等.糖尿病足甲襞微循环与中医辨证分型的临床研究[J].四川中医,2014,32(10):63-68.	Not related to Oral CHM
0482	童伯瑛,王志强,魏振朴,等.体质因素对代谢综合征影响的回归分析[J].云南中医学院学报,2017,40(05):68-72.DOI:10.19288/j.cnki.issn.1000-2723.2017.05.015.	Not related to Oral CHM
0483	郑婕,许秀玫,梁炜锋,等.通元针法治疗脾虚湿阻型单纯性肥胖的短期随访观察[J].中国当代医药,2017,24(20):61-64.	Not related to Oral CHM
0484	谢房乐,侯平.心衰病中医体质分布及与并发症的相关性[J].实用中医内科杂志,2020,34(06):28-31.DOI:10.13729/j.issn.1671-7813.Z20191010.	Not related to Oral CHM
0485	王骁腾,梁可意,孙敬雯,等.新疆高脂血症病人证候分布特点及与血脂水平的关系探讨[J].中西医结合心脑血管病杂志,2016,14(06):567-569.	Not related to Oral CHM
0486	赵斌斌,张丹婷.穴位保健对痰湿型血脂异常患者的影响[J].中医临床研究,2018,10(06):74-76.	Not related to Oral CHM
0487	张惠玲,郭秀君,张敏,等.穴位降压操对原发性高血压痰湿壅盛证的疗效观察[J].南京中医药大学学报,2017,33(02):136-139.DOI:10.14148/j.issn.1672-0482.2017.0136.	Not related to Oral CHM
0488	钱璐,周蕾.穴位埋线联合西药治疗代谢综合征的临床疗效及对照研究[J].中华中医药学刊,2017,35(04):1044-1046.DOI:10.13193/j.issn.1673-7717.2017.04.076.	Not related to Oral CHM
0489	张佳谕.穴位埋线治疗单纯性肥胖的临床研究[D].广州中医药大学,2011.	Not related to Oral CHM
0490	吴兴兰, 2014. 穴位埋线治疗单纯性肥胖症的临床观察, 医学信息·上旬刊	Not related to Oral CHM
0491	潘晨,王恒和.血浆同型半胱氨酸与原发性高血压痰湿证相关性的Meta分析[J].江西中医药,2023,54(07):51-55.	Not related to Oral CHM
0492	王瑛,沈建雄,王海英,等.血清淀粉样蛋白A、C反应蛋白、降钙素原和血细胞分析检测在 3 级糖尿病足感染诊断中的应用[J].检验医学与临床,2020,17(11):1512-1514.	Not related to Oral CHM
0493	谢敏,谷万里.血脂异常与中医体质类型相关性研究[J].青岛大学医学院学报,2017,53(01):98-99+102.DOI:10.13361/j.qdyxy.201701029.	Not related to Oral CHM
0494	汪自龙,朱智明,石湘芸.以咯血为首发症状的急进性高血压一例[J].天津医药,2000,(05):284.	Not related to Oral CHM
0495	赵永强.原发性高血压患者中医非药物辨证干预效果评价[J].医学理论与实践,2015,28(14):1952-1954.DOI:10.19381/j.issn.1001-7585.2015.14.077.	Not related to Oral CHM
0496	张建东.长期应用小剂量辛伐他汀致横纹肌病 1 例[J].临床心血管病杂志,2006,(03):189-190.	Not related to Oral CHM
0497	魏群利,蔡辉,刘志诚.针刺对单纯性肥胖症远期疗效观察[J].中国康复,2002,(04):196-198.	Not related to Oral CHM
0498	赵少忠.针刺治疗 2 型糖尿病并发面神经炎临床研究[J].中医学报,2016,31(02):195-198.DOI:10.16368/j.issn.1674-8999.2016.02.054.	Not related to Oral CHM
0499	周国容.针刺治疗不同辨证分型糖尿病皮肤瘙痒 55 例疗效分析[J].药物与人,2014,27(06):280.	Not related to Oral CHM

0500	孙忠人,栾逸先,盛国滨,等.针刺治疗痰湿中阻型轻度原发性高血压的临床疗效观察[J].中华中医药杂志,2020,35(01):451-454.	Not related to Oral CHM
0501	周莉萍,胡玲香,邓雪梅,等.针灸减肥进程中患者肠道菌群的变化规律探讨[J].四川中医,2011,29(09):124-126.	Not related to Oral CHM
0502	张其兰,单鸣,牟英,等.中国灸治疗糖尿病神经原性膀胱 50 例[J].华西医学,2009,24(04):980-981.	Not related to Oral CHM
0503	王军,刘存午,冯海波.中频治疗诱发阵发性心房纤颤 2 例[J].疑难病杂志,2007,(01):14.	Not related to Oral CHM
0504	赵军.中医辨证论治治疗糖尿病疗效分析[J].河北医学,2008,(09):1060-1062.	Not related to Oral CHM
0505	危北海, & 郭培元. (2000). 中医脾胃与现代分子生物学与信息系统. 世界华人消化杂志, (z1), 82.	Not related to Oral CHM
0506	许钰波,李月伟,李成玉.中医气针疗法配合药物治疗肝性脊髓病终末期多并发症 1 例[J].中医临床研究,2021,13(23):56-58.	Not related to Oral CHM
0507	宋润娣,陈峻鹏,单莉,等.中医体质辨识及干预在高血压前期治未病健康管理中的效果研究[J].四川中医,2018,36(12):195-197.	Not related to Oral CHM
0508	王兆为,欧阳间英,雷波,等.中医体质调摄对社区老年糖尿病患者治疗效果与生存质量的影响[J].世界中医药,2016,11(08):1606-1609.	Not related to Oral CHM
0509	张雨楠,刘鹤源,黄哲,等.中医药临床随机对照试验文献结构化信息的自动化提取及信息质量评价[J].中国医药导报,2023,20(11):183-187+192.DOI:10.20047/j.issn1673-7210.2023.11.42.	Not related to Oral CHM
0510	赵强,冯伟,张勇.中医综合疗法治疗单纯性肥胖症 52 例[J].江苏中医药,2011,43(08):64-65.	Not related to Oral CHM
0511	石二霞,云来运,李恒善.注射用还原型谷胱甘肽致寒战、高热、恶心、心悸、头晕、头痛 1 例[J].中国医院药学杂志,2020,40(01):124-125.DOI:10.13286/j.1001-5213.2020.01.21.	Not related to Oral CHM
0512	许明山,刘辉,邵立波,等.子午流注纳甲法针刺治疗高脂血症 50 例[J].山东中医杂志,2013,32(10):733-734.DOI:10.16295/j.cnki.0257-358x.2013.10.022.	Not related to Oral CHM
0513	Malcangi, G., Inchingolo, A. D., Inchingolo, A. M., Santacroce, L., Marinelli, G., Mancini, A., ... & Dipalma, G. (2021). COVID-19 Infection in Children, Infants and Pregnant Subjects: An Overview of Recent Insights and Therapies. Microorganisms, 9(9), 1964.	Not related to Oral CHM
0514	Mahboobi S, Tsang C, Rezaei S, Jafarnejad S. Effect of L-citrulline supplementation on blood pressure: a systematic review and meta-analysis of randomized controlled trials [retracted in: J Hum Hypertens. 2021 Apr;35(4):381. doi: 10.1038/s41371-019-0280-1.]. J Hum Hypertens. 2019;33(1):10-21. doi:10.1038/s41371-018-0108-4IF: 2.7 Q2	Not related to Oral CHM
0515	de Almeida Magalhães TSS, de Oliveira Macedo PC, Converti A, Neves de Lima ÁA. The Use of Euterpe oleracea Mart. As a New Perspective for Disease Treatment and Prevention. Biomolecules. 2020;10(6):813. Published 2020 May 26. doi:10.3390/biom10060813	Not related to Oral CHM
0516	MacKnight JM, Mistry DJ. Allergic disorders in the athlete. Clin Sports Med. 2005;24(3):507-viii. doi:10.1016/j.csm.2005.04.003	Not related to Oral CHM

0517	Macklin EA, Wayne PM, Kalish LA, et al. Stop Hypertension with the Acupuncture Research Program (SHARP): results of a randomized, controlled clinical trial. Hypertension. 2006;48(5):838-845. doi:10.1161/01.HYP.0000241090.28070.4c	Not related to Oral CHM
0518	Ma Z, Lei H, Tian K, et al. Baduanjin exercise in the treatment of hypertension: A systematic review and meta-analysis. Front Cardiovasc Med. 2022;9:936018. Published 2022 Aug 15. doi:10.3389/fcvm.2022.936018	Not related to Oral CHM
0519	Ma YL, Yao H, Yang WJ, Ren XX, Teng L, Yang MC. Correlation between Traditional Chinese Medicine Constitution and Dyslipidemia: A Systematic Review and Meta-Analysis. Evid Based Complement Alternat Med. 2017;2017:1896746. doi:10.1155/2017/1896746	Not related to Oral CHM
0520	Ma Q, Li H, Gao Y, Zou Y. Effects of Baduanjin on glucose and lipid metabolism in diabetic patients: A protocol for systematic review and meta-analysis. Medicine (Baltimore). 2021;100(4):e23532. doi:10.1097/MD.0000000000023532	Not related to Oral CHM
0521	Zhang Q, Xu X, Wu Q, et al. Effects of different traditional Chinese exercise in the treatment of essential hypertension: a systematic review and network meta-analysis. Front Cardiovasc Med. 2024;11:1300319. Published 2024 Feb 28. doi:10.3389/fcvm.2024.1300319	Not related to Oral CHM
0522	Ma G, Ye T, Sun Z. Zhongguo Zhen Jiu. 2018;38(3):229-232. doi:10.13703/j.0255-2930.2018.03.001	Not related to Oral CHM
0523	Ma D, Taku K, Zhang Y, Jia M, Wang Y, Wang P. Serum lipid-improving effect of soyabean 尾-conglycinin in hyperlipidaemic menopausal women.聽 Br J Nutr. 2013;110(9):1680-1684. doi:10.1017/S0007114513000986	Not related to Oral CHM
0524	Lyons, I., Barber, N., Raynor, D. K., Harrison, J., & Wei, L. (2014). Serum lipid and glycemic control among mail order pharmacy patients. International Journal of Pharmacy Practice, 22, 14-15.	Not related to Oral CHM
0525	Lv J, Zhang X, Ou S, et al. Influence of Cognitive Behavioral Therapy on Mood and Quality of Life After Stent Implantation in Young and Middle-Aged Patients With Coronary Heart Disease. Int Heart J. 2016;57(2):167-172. doi:10.1536/ihj.15-259	Not related to Oral CHM
0526	Luo Y, Wang H, Zhou X, et al. A Randomized Controlled Clinical Trial of Lifestyle Intervention and Pioglitazone for Normalization of Glucose Status in Chinese with Prediabetes. J Diabetes Res. 2022;2022:2971382. Published 2022 Jan 6. doi:10.1155/2022/2971382	Not related to Oral CHM
0527	Luo SJ, Wang YY, Fan ZS, et al. Zhonghua Wei Chang Wai Ke Za Zhi. 2021;24(10):897-903. doi:10.3760/cma.j.cn.441530-20200715-00420	Not related to Oral CHM
0528	Luo JW, Chen H, Wu XY. Zhongguo Zhong Xi Yi Jie He Za Zhi. 2010;30(5):458-462.	Not related to Oral CHM
0529	Luo, H., Li, L., Li, T., Liao, X., & Wang, Q. (2020). Association between metabolic syndrome and body constitution of traditional Chinese medicine: a systematic review and meta-analysis. Journal of Traditional Chinese Medical Sciences, 7(4), 355-365.	Not related to Oral CHM

0530	Lunell E, Lunell M. Steady-state nicotine plasma levels following use of four different types of Swedish snus compared with 2-mg Nicorette chewing gum: a crossover study. Nicotine Tob Res. 2005;7(3):397-403. doi:10.1080/14622200500125468IF: 3.0 Q2	Not related to Oral CHM
0531	Luck J, Peabody JW, Dresselhaus TR, Lee M, Glassman P. How well does chart abstraction measure quality? A prospective comparison of standardized patients with the medical record. Am J Med. 2000;108(8):642-649. doi:10.1016/s0002-9343(00)00363-6	Not related to Oral CHM
0532	Luan F, Han K, Li M, et al. Ethnomedicinal Uses, Phytochemistry, Pharmacology, and Toxicology of Species from the Genus Ajuga L.: A Systematic Review. Am J Chin Med. 2019;47(5):959-1003. doi:10.1142/S0192415X19500502	Not related to Oral CHM
0533	Lu S, Du S, Fish A, Tang C, Lou Q, Zhang X. Wet cupping for hypertension: a systematic review and meta-analysis. Clin Exp Hypertens. 2019;41(5):474-480. doi:10.1080/10641963.2018.1510939	Not related to Oral CHM
0534	Lu QS, Lei Y, Chen KJ. Zhongguo Zhong Xi Yi Jie He Za Zhi. 2005;25(8):682-686.	Not related to Oral CHM
0535	Lu J, Xie Y, Du J, et al. Penta-therapy for severe acute hyperlipidemic pancreatitis. Am J Emerg Med. 2018;36(10):1789-1795. doi:10.1016/j.ajem.2018.01.092	Not related to Oral CHM
0536	Lu HQ, Lu W, Liu J, Yang KJ, Li ZG. Recent Pharmacokinetic Studies in Combination Therapies for Diabetes and Related Vascular Complications. Curr Vasc Pharmacol. 2015;13(4):554-561. doi:10.2174/1570161112666141014154047	Not related to Oral CHM
0537	Lu CN, Friedman M, Lin HC, et al. Alternative Therapy for Patients With Obstructive Sleep Apnea/Hypopnea Syndrome: A 1-year, Single-blind, Randomized Trial of Tui Na. Altern Ther Health Med. 2017;23(4):16-24.	Not related to Oral CHM
0538	Lu AP, Chen KJ. Chinese medicine pattern diagnosis could lead to innovation in medical sciences. Chin J Integr Med. 2011;17(11):811-817. doi:10.1007/s11655-011-0891-z	Not related to Oral CHM
0539	Loza-Taylor T, Hernández-Carlsen J. La percepción del padecer en personas con complicaciones crónicas por diabetes mellitus [The perception of illness in people with chronic complications caused by diabetes mellitus]. Rev Med Inst Mex Seguro Soc. 2021;59(3):197-204. Published 2021 Aug 13.	Not related to Oral CHM
0540	Lowrie R, Morrison J, McConnachie A. A cluster randomised controlled trial of pharmacist led statin outreach support (SOS) in primary care: design and baseline characteristics. Contemp Clin Trials. 2010;31(4):303-311. doi:10.1016/j.cct.2010.03.010	Not related to Oral CHM
0541	Long T, Yue R, Wu T, Xu C, Yang M. The efficacy and safety of acupoint injection for diabetic gastroparesis: A protocol for systematic review and meta-analysis. Medicine (Baltimore). 2020;99(45):e23086. doi:10.1097/MD.0000000000023086	Not related to Oral CHM

0542	Lomuscio A, Belletti S, Battezzati PM, Lombardi F. Efficacy of acupuncture in preventing atrial fibrillation recurrences after electrical cardioversion. J Cardiovasc Electrophysiol. 2011;22(3):241-247. doi:10.1111/j.1540-8167.2010.01878.x	Not related to Oral CHM
0543	Lombardi F, Belletti S, Battezzati PM, Lomuscio A. Acupuncture for paroxysmal and persistent atrial fibrillation: An effective non-pharmacological tool?. World J Cardiol. 2012;4(3):60-65. doi:10.4330/wjc.v4.i3.60	Not related to Oral CHM
0544	Lodha R, Bagga A. Traditional Indian systems of medicine. Ann Acad Med Singap. 2000;29(1):37-41.	Not related to Oral CHM
0545	Liu, Z. C., Sun, F. M., Yan, R. H., Xu, B. G., Zhu, M. H., Xu, B., & Yuan, J. H. (2004). Observation of therapeutic effectiveness of female simple obesity complicated with menopausal syndrome treated by acupuncture. Zhongguo Linchuang Kangfu, 8(6), 1198-1200.	Not related to Oral CHM
0546	Liu Z, Shen L, Huang W, et al. Efficacy and safety of renal denervation for Chinese patients with resistant hypertension using a microirrigated catheter: study design and protocol for a prospective multicentre randomised controlled trial. BMJ Open. 2017;7(9):e015672. Published 2017 Sep 1. doi:10.1136/bmjopen-2016-015672	Not related to Oral CHM
0547	LIU, Y. Z., SU, S. Y., PAN, Q. L., NING, F. J., LI, J. Y., CHEN, D. D., ... & WU, W. F. (2014). Comparison of efficacy of treating obese patients with endocrine-metabolic abnormity by electroacupuncture of different types of wave modes. World Journal of Acupuncture-Moxibustion, 24(3), 1-8.	Not related to Oral CHM
0548	Liu XX, Li SH, Chen JZ, et al. Effect of soy isoflavones on blood pressure: a meta-analysis of randomized controlled trials. Nutr Metab Cardiovasc Dis. 2012;22(6):463-470. doi:10.1016/j.numecd.2010.09.006	Not related to Oral CHM
0549	Liu XL, Wei AL, Luo F. Zhongguo Zhong Xi Yi Jie He Za Zhi. 2009;29(9):833-835.	Not related to Oral CHM
0550	Liu X, Wang L, Xing Y, et al. Efficacy and safety of metformin and sitagliptin-based dual and triple therapy in elderly Chinese patients with type 2 diabetes: Subgroup analysis of STRATEGY study. J Diabetes Investig. 2020;11(6):1532-1541. doi:10.1111/jdi.13277	Not related to Oral CHM
0551	Liu X, Kong D, Lian H, et al. Distribution and predictors of hospital charges for haemorrhagic stroke patients in Beijing, China, March 2012 to February 2015: a retrospective study. BMJ Open. 2018;8(3):e017693. Published 2018 Mar 30. doi:10.1136/bmjopen-2017-017693	Not related to Oral CHM
0552	Liu X, Huang H, Yu J, et al. Warfarin compared with aspirin for older Chinese patients with stable coronary heart diseases and atrial fibrillation complications. Int J Clin Pharmacol Ther. 2014;52(6):454-459. doi:10.5414/CP201996	Not related to Oral CHM
0553	Wei L, Xingjiang X, Lumin Q, et al. Acupoint application therapies for essential hypertension: a systematic review and Meta-analysis. J Tradit Chin Med. 2022;42(2):159-166. doi:10.19852/j.cnki.jtcm.2022.02.001	Not related to Oral CHM

0554	Liu S, Lee IM, Ajani U, et al. Intake of vegetables rich in carotenoids and risk of coronary heart disease in men: The Physicians' Health Study. Int J Epidemiol. 2001;30(1):130-135. doi:10.1093/ije/30.1.130	Not related to Oral CHM
0555	Liu M, Zhang Z, Zhou C, et al. Relationship of Body Mass Index and Waist Circumference With Risk of New-Onset Proteinuria in Hypertensive Patients [published correction appears in J Clin Endocrinol Metab. 2020 Jul 1;105(7):dgaa153. doi: 10.1210/clinem/dgaa153.]. J Clin Endocrinol Metab. 2020;105(3):dgaa026. doi:10.1210/clinem/dgaa026	Not related to Oral CHM
0556	Liu M, Zhang Q, Jiang S, et al. Warm-needling acupuncture and medicinal cake-separated moxibustion for hyperlipidemia: study protocol for a randomized controlled trial. Trials. 2017;18(1):310. Published 2017 Jul 10. doi:10.1186/s13063-017-2029-x	Not related to Oral CHM
0557	Liu M, Li Z, Wang S, et al. Application via mechanical dropper alleviates sufentanil-induced cough: a prospective, randomized, single-blinded trial. Trials. 2019;20(1):170. Published 2019 Mar 15. doi:10.1186/s13063-019-3274-y	Not related to Oral CHM
0558	Liu L, Zhang Y, Liu G, et al. The Felodipine Event Reduction (FEVER) Study: a randomized long-term placebo-controlled trial in Chinese hypertensive patients. J Hypertens. 2005;23(12):2157-2172. doi:10.1097/01.hjh.0000194120.42722.ac	Not related to Oral CHM
0559	Liu L, Wang Z, Gong L, et al. Blood pressure reduction for the secondary prevention of stroke: a Chinese trial and a systematic review of the literature. Hypertens Res. 2009;32(11):1032-1040. doi:10.1038/hr.2009.139	Not related to Oral CHM
0560	Liu JL, Song JN, Yan L. Zhongguo Zhong Xi Yi Jie He Za Zhi. 2010;30(5):482-487.	Not related to Oral CHM
0561	Liu HF, He HC, Yang L, et al. Pulsed electromagnetic fields for postmenopausal osteoporosis and concomitant lumbar osteoarthritis in southwest China using proximal femur bone mineral density as the primary endpoint: study protocol for a randomized controlled trial. Trials. 2015;16:265. Published 2015 Jun 10. doi:10.1186/s13063-015-0780-4	Not related to Oral CHM
0562	Liu H, Xu X, Hall JJ, Wu X, Zhang M. Differences in depression between unknown diabetes and known diabetes: results from China health and retirement longitudinal study. Int Psychogeriatr. 2016;28(7):1191-1199. doi:10.1017/S104161021600020X	Not related to Oral CHM
0563	Liu H, Liu M, Jiao Y, et al. A Computational Framework to Study the Effect of Acupuncture on Obesity by Integrating Multiple Levels of Data [retracted in: Biomed Res Int. 2024 Mar 20;2024:9891351. doi: 10.1155/2024/9891351.]. Biomed Res Int. 2020;2020:8513860. Published 2020 Oct 16. doi:10.1155/2020/8513860	Not related to Oral CHM
0564	Liu, H., Chen, S., Zhong, C., Ma, D., & Liu, M. (2020, April). Effect of Acupuncture on Blood Lipid Level in Obese Patient. In BASIC & CLINICAL PHARMACOLOGY & TOXICOLOGY (Vol. 126, pp. 19-20). 111 RIVER ST, HOBOKEN 07030-5774, NJ USA: WILEY.	Not related to Oral CHM

056<br>5	Liu D, Li L, Sun N, et al. Effects of body mass index on IVF outcomes in different age groups. BMC Womens Health. 2023;23(1):416. Published 2023 Aug 9. doi:10.1186/s12905-023-02540-8	Not related to Oral CHM
056<br>6	Liu CJ, Chen ZH, Li SJ, Ren H, Hu XY, Ren YL. Zhongguo Zhen Jiu. 2022;42(2):221-226. doi:10.13703/j.0255-2930.20201221-k0001	Not related to Oral CHM
056<br>7	Liu, C., Tao, L. B., & Wang, F. (2020). PDB19 COST-EFFECTIVENESS ANALYSIS OF INSULIN DEGLUDEC/INSULIN ASPART VERSUS INSULIN GLARGINE IN PATIENTS WITH TYPE 2 DIABETES IN CHINA. Value in Health, 23, S111.	Not related to Oral CHM
056<br>8	Liu B, Chen N, Zhao J, et al. Efficacy and safety of darbepoetin alfa injection replacing epoetin alfa injection for the treatment of renal anemia in Chinese hemodialysis patients: A randomized, open-label, parallel-group, noninferiority phase III trial [published correction appears in Chronic Dis Transl Med. 2023 Sep 19;10(4):350. doi: 10.1002/cdt3.94.]. Chronic Dis Transl Med. 2022;8(2):134-144. Published 2022 Apr 18. doi:10.1002/cdt3.23	Not related to Oral CHM
056<br>9	Ling S, Nheu L, Komesaroff PA. Cell adhesion molecules as pharmaceutical target in atherosclerosis. Mini Rev Med Chem. 2012;12(2):175-183. doi:10.2174/138955712798995057	Not related to Oral CHM
057<br>0	Lindor, K. D., Kowdley, K. V., Heathcote, E. J., Novogrodsky, A., & Brown, R. S. (2004). Ursodeoxycholic acid for the treatment of nonalcoholic steatohepatitis: Panacea or placebo?. Evidence-Based Gastroenterology, 5(4), 122-123.	Not related to Oral CHM
057<br>1	Lindarto, D, Syafril, S, Zein, U and Saragih, A, 2016. The effect of dhawalsan-1 (Curanga fel-terrae [Lour.]) extract versus metformin on the metabolic and inflammatory characteristics of patients with newly diagnosed type 2 diabetes mellitus, Asian Journal of Pharmaceutical and Clinical Research.	Not related to Oral CHM
057<br>2	Lind N, Lindqvist Hansen D, S忙tre Rasmussen S, N酶rgaard K. Real-time continuous glucose monitoring versus self-monitoring of blood glucose in adults with insulin-treated type 2 diabetes: a protocol for a randomised controlled single-centre trial.聽BMJ Open. 2021;11(1):e040648. Published 2021 Jan 15. doi:10.1136/bmjopen-2020-040648	Not related to Oral CHM
057<br>3	Lin L, Chen Y, Li Y, et al. 10.6-渭m infrared laser as adjuvant therapy for diabetic peripheral neuropathy: study protocol for a double-blind, randomized controlled trial.聽Trials. 2022;23(1):53. Published 2022 Jan 18. doi:10.1186/s13063-021-05901-6	Not related to Oral CHM
057<br>4	Lin J, Zhao J, Hao G, et al. Maternal and Neonatal Complications After Natural vs. Hormone Replacement Therapy Cycle Regimen for Frozen Single Blastocyst Transfer. Front Med (Lausanne). 2020;7:338. Published 2020 Aug 28. doi:10.3389/fmed.2020.00338IF: 3.1 Q1	Not related to Oral CHM
057<br>5	Lin CH, Lin YM, Liu CF. Electrical acupoint stimulation changes body composition and the meridian systems in postmenopausal women with obesity. Am J Chin Med. 2010;38(4):683-694.	Not related to Oral CHM

	doi:10.1142/S0192415X10008159	
0576	Liew, J, Barlow, A, Lim, LL, Suastika, K, Yasahardja, Y, Chan, SP, Soh, A, Li, LSW and Tan, AT, 2019. Role of b vitamins (B1, B6, B12) in managing diabetic peripheral neuropathy (DPN): A systematic review, Diabetes.	Not related to Oral CHM
0577	Liao YC, Chen LL, Wang HC, Lin JS, Lin TK, Lin SA. The Association Between Traditional Chinese Medicine Body Constitution Deviation and Essential Hypertension: A Case-Control Study. J Nurs Res. 2021;29(4):e160. Published 2021 Jun 14. doi:10.1097/JNR.0000000000000442	Not related to Oral CHM
0578	Liao, L, Cong, H, Xu, Z, Li, E, Weng, Z, Jiang, H, Liu, B, Huang, X, Xia, S, Wen, W and et al., 2021, Efficacy and safety study of Chinese botulinum toxin A 100U in patients with overactive bladder: a prospective, multicenter, double-blind and randomized controlled trial, Chinese journal of urology.	Not related to Oral CHM
0579	Liang X, Wang Q, Jiang Z, et al. Clinical research linking Traditional Chinese Medicine constitution types with diseases: a literature review of 1639 observational studies. J Tradit Chin Med. 2020;40(4):690-702. doi:10.19852/j.cnki.jtcm.2020.04.019	Not related to Oral CHM
0580	Liang J, Feng Z, Feng S, Bao S, Wang K. Zhongguo Zhen Jiu. 2018;38(1):12-16. doi:10.13703/j.0255-2930.2018.01.003	Not related to Oral CHM
0581	Li Z, Chen Q, Yan J, Liang W, Wong WCW. Effectiveness of motivational interviewing on improving Care for Patients with type 2 diabetes in China: A randomized controlled trial. BMC Health Serv Res. 2020;20(1):57. Published 2020 Jan 23. doi:10.1186/s12913-019-4776-8	Not related to Oral CHM
0582	Hu M, Mak VW, Tomlinson B. Comments on "CYP3AP1*3 allele is associated with lipid-lowering efficacy of simvastatin and atorvastatin in Chinese women". J Clin Pharmacol. 2012;52(11):1768-1771. doi:10.1177/0091270011415411	Not related to Oral CHM
0583	Li Y, Zheng B, Chen K, Gui L. Successful Treatment of Dental Infection-Induced Chronic Cavernous Sinus Thrombophlebitis With Antibiotics and Low-Molecular-Weight Heparin: Two Case Reports. J Oral Maxillofac Surg. 2015;73(8):1516-1523. doi:10.1016/j.joms.2015.02.022	Not related to Oral CHM
0584	Li Y, Zhang M, Xue M, Wei M, He J, Dong C. A case report of cerebral venous sinus thrombosis presenting with rapidly progressive dementia. Front Med (Lausanne). 2022;9:985361. Published 2022 Aug 25. doi:10.3389/fmed.2022.985361	Not related to Oral CHM
0585	Li Y, Yu J, Kuang Y, et al. Quality of oral anticoagulation control in Chinese patients with non-valvular atrial fibrillation: a prospective controlled study. Curr Med Res Opin. 2020;36(9):1433-1439. doi:10.1080/03007995.2020.1796611	Not related to Oral CHM
0586	Li Y, Wang X, Shen Z. Traditional Chinese medicine for lipid metabolism disorders. Am J Transl Res. 2017;9(5):2038-2049. Published 2017 May 15.	Not related to Oral CHM

0587	Li Y, Tan J, Wang Q, Duan C, Hu Y, Huang W. Comparing the individual effects of metformin and rosiglitazone and their combination in obese women with polycystic ovary syndrome: a randomized controlled trial. Fertil Steril. 2020;113(1):197-204. doi:10.1016/j.fertnstert.2019.09.011	Not related to Oral CHM
0588	Li Y, Peng C, Cao G, Li W, Hou L. Tai chi for overweight/obese adolescent and young women with polycystic ovary syndrome: study protocol for a randomized controlled trial. Trials. 2018;19(1):512. Published 2018 Sep 20. doi:10.1186/s13063-018-2893-z	Not related to Oral CHM
0589	Individualized prevention against hypertension based on Traditional Chinese Medicine Constitution Theory: A large community-based retrospective, STROBE-compliant study among Chinese population: Erratum. Medicine (Baltimore). 2018;97(19):e0795. doi:10.1097/MD.0000000000010795	Not related to Oral CHM
0590	Li Y, Hou L, Wang Y, et al. Auricular points acupressure for insulin resistance in overweight/obese women with polycystic ovary syndrome: protocol for a randomised controlled pilot trial. BMJ Open. 2019;9(5):e027498. Published 2019 May 28. doi:10.1136/bmjopen-2018-027498	Not related to Oral CHM
0591	Li, Y, Fang, DJ, Gao, X, Tian, HM and Wu, TX, 2006. Prostaglandin E1 for diabetic peripheral neuropathy: A systematic review, Chinese Journal of Evidence-Based Medicine.	Not related to Oral CHM
0592	Li XX, A-Yi-Gu-Li YN, Huang JJ, Zhang JP, Ka-Si-Mu-Jiang AX, Ku-Re-Xi YN. Zhongguo Zhong Xi Yi Jie He Za Zhi. 2014;34(3):297-302.	Not related to Oral CHM
0593	Li XG, Chen J, Wang W, et al. Oseltamivir Treatment for Influenza During the Flu Season of 2018-2019: A Longitudinal Study. Front Microbiol. 2022;13:865001. Published 2022 May 10. doi:10.3389/fmicb.2022.865001	Not related to Oral CHM
0594	Li X, Yin Z, Ling F, et al. The application of acupuncture in cardiopathy: A bibliometric analysis based on Web of Science across ten recent years. Front Cardiovasc Med. 2022;9:920491. Published 2022 Sep 6. doi:10.3389/fcvm.2022.920491	Not related to Oral CHM
0595	Li X, Yan Z, Xia J, et al. Traditional Chinese acupoint massage, acupuncture, and moxibustion for people with diabetic gastroparesis: A systematic review and meta-analysis. Medicine (Baltimore). 2022;101(48):e32058. doi:10.1097/MD.0000000000032058	Not related to Oral CHM
0596	Li X, Wang L, Li D, Niu J, Gao P. Dyslipidemia is a Risk Factor for the Incidence and Severity of Drug-Induced Liver Injury (DILI): A Retrospective Population-Based Study in China. Med Sci Monit. 2019;25:3344-3353. Published 2019 May 6. doi:10.12659/MSM.916687	Not related to Oral CHM
0597	Li X, Si H, Chen Y, Li S, Yin N, Wang Z. Effects of fitness qigong and tai chi on middle-aged and elderly patients with type 2 diabetes mellitus. PLoS One. 2020;15(12):e0243989. Published 2020 Dec 17. doi:10.1371/journal.pone.0243989	Not related to Oral CHM

0598	Li X, Dai H, Li X, et al. Efficacy and safety of ranibizumab 0.5 mg in Chinese patients with visual impairment due to diabetic macular edema: results from the 12-month REFINE study. Graefes Arch Clin Exp Ophthalmol. 2019;257(3):529-541. doi:10.1007/s00417-018-04213-x	Not related to Oral CHM
0599	The efficacy of Qigong training in patients with various TCM types of hypertension	Not related to Oral CHM
0600	Li W, Xin Z, Pi D. Zhong Xi Yi Jie He Za Zhi. 1990;10(5):283-261.	Not related to Oral CHM
0601	Li SW, Wu J. Zhongguo Zhen Jiu. 2011;31(2):125-128.	Not related to Oral CHM
0602	Li R, Mai T, Zheng S, Zhang Y. Effect of metformin and exenatide on pregnancy rate and pregnancy outcomes in overweight or obese infertility PCOS women: long-term follow-up of an RCT. Arch Gynecol Obstet. 2022;306(5):1711-1721. doi:10.1007/s00404-022-06700-3	Not related to Oral CHM
0603	Qian, L. I., Han, P. E. N. G., Hong-hua, L. I. U., Yi-fan, Z. O. U., Dan, L. I., Jun-yu, G. E., ... & Mai-lan, L. I. U. (2022). Hypercholesterolemia treated with medicinal pad-separated moxibustion: A randomized clinical trial 隔药饼灸治疗高胆固醇血症-随机临床试验. World Journal of Acupuncture-Moxibustion, 32(4), 310-316.	Not related to Oral CHM
0604	Li P, Wu Y, Xie Y, et al. Zhonghua Yu Fang Yi Xue Za Zhi. 2023;57(7):1047-1058. doi:10.3760/cma.j.cn112150-20221221-01220	Not related to Oral CHM
0605	Li N, Ye HY, Zheng GY, et al. Zhongguo Zhong Xi Yi Jie He Za Zhi. 2014;34(4):402-405.	Not related to Oral CHM
0606	Li, M., Yao, L., Huang, H., Zhang, L., Zheng, H., Wang, G., ... & Wang, H. (2021). Multimodal cerebral imaging study on the effects of "Adjust Zang Dredge Meridian" electroacupuncture on cerebral central sensitization in PDPN patients: a study protocol for a sham-controlled, randomized trial. Trials, 22, 1-13.	Not related to Oral CHM
0607	Li L, Yang M, Song J, Yu Y, Huang H. Network Meta-Analysis of the Antihypertensive Effect of Traditional Chinese Exercises on Patients with Essential Hypertension. J Healthc Eng. 2022;2022:9419037. Published 2022 Aug 17. doi:10.1155/2022/9419037	Not related to Oral CHM
0608	Li L, Wang S, Huang H, et al. Effects of Rosuvastatin and Aspirin on Retinal Vascular Structures in Hypercholesterolemic Patients with Low-to-Moderate Risk of Coronary Artery Disease. Am J Cardiovasc Drugs. 2019;19(4):415-420. doi:10.1007/s40256-019-00330-y	Not related to Oral CHM
0609	Li JL, Li M, Pang B, et al. Combination of symptoms, syndrome and disease: treatment of refractory diabetic gastroparesis. World J Gastroenterol. 2014;20(26):8674-8680. doi:10.3748/wjg.v20.i26.8674	Not related to Oral CHM
0610	Li J, Zheng H, Zhao L, et al. Acupuncture for patients with mild hypertension: study protocol of an open-label multicenter randomized controlled trial. Trials. 2013;14:380. Published 2013 Nov 11. doi:10.1186/1745-6215-14-380	Not related to Oral CHM

0611	Li J, Wu Q, Wu XK, et al. Effect of exposure to second-hand smoke from husbands on biochemical hyperandrogenism, metabolic syndrome and conception rates in women with polycystic ovary syndrome undergoing ovulation induction. Hum Reprod. 2018;33(4):617-625. doi:10.1093/humrep/dey027	Not related to Oral CHM
0612	Li J, Wei D, Liu S, et al. Efficiency of an mHealth App and Chest-Wearable Remote Exercise Monitoring Intervention in Patients With Type 2 Diabetes: A Prospective, Multicenter Randomized Controlled Trial. JMIR Mhealth Uhealth. 2021;9(2):e23338. Published 2021 Feb 9. doi:10.2196/23338	Not related to Oral CHM
0613	Li J, Ng EH, Stener-Victorin E, et al. Acupuncture treatment for insulin sensitivity of women with polycystic ovary syndrome and insulin resistance: a study protocol for a randomized controlled trial. Trials. 2017;18(1):115. Published 2017 Mar 9. doi:10.1186/s13063-017-1854-2	Not related to Oral CHM
0614	Li J, Li J, Shan Z, et al. Gender-differential effects on blood glucose levels between acarbose and metformin in Chinese patients with newly diagnosed type 2 diabetes: a sub-analysis of the MARCH trial. Endocr J. 2021;68(1):69-79. doi:10.1507/endocrj.EJ20-0006	Not related to Oral CHM
0615	Li J, Huang B, Wang S, et al. Zhonghua Wei Zhong Bing Ji Jiu Yi Xue. 2019;31(12):1497-1500. doi:10.3760/cma.j.issn.2095-4352.2019.12.012	Not related to Oral CHM
0616	Li HY, Cui L, Cui M. Hot topics in Chinese herbal drugs research documented in PubMed/MEDLINE by authors inside China and outside of China in the past 10 years: based on co-word cluster analysis. J Altern Complement Med. 2009;15(7):779-785. doi:10.1089/acm.2008.0594	Not related to Oral CHM
0617	Li HL, Li H, Cao YF, et al. Effects of keto acid supplements on Chinese patients receiving maintenance hemodialysis: a prospective, randomized, controlled, single-center clinical study. Chin Med J (Engl). 2020;133(1):9-16. doi:10.1097/CM9.0000000000000578	Not related to Oral CHM
0618	Li H, Butler K, Yang L, Yang Z, Teng R. Pharmacokinetics and tolerability of single and multiple doses of ticagrelor in healthy Chinese subjects: an open-label, sequential, two-cohort, single-centre study. Clin Drug Investig. 2012;32(2):87-97. doi:10.2165/11595930-000000000-00000	Not related to Oral CHM
0619	Li D, Xu X, Zhang Y, et al. Liraglutide treatment causes upregulation of adiponectin and downregulation of resistin in Chinese type 2 diabetes. Diabetes Res Clin Pract. 2015;110(2):224-228. doi:10.1016/j.diabres.2015.05.051	Not related to Oral CHM
0620	Li D, Huo Z, Liu D, et al. Current apparent treatment-resistant hypertension in patients undergoing peritoneal dialysis: A multi-center cross-sectional study. J Clin Hypertens (Greenwich). 2022;24(4):493-501. doi:10.1111/jch.14455	Not related to Oral CHM
0621	Li, C. Y., Yu, S. Y., Guo, B. J., Li, W. Y., Yang, J., & Hu, Y. P. (2018). Analysis on randomized controlled trials for acupuncture treatment of simple obesity collected from PubMed. Zhen ci yan jiu= Acupuncture Research, 43(4), 269-273.	Not related to Oral CHM

0622	Li, C., Zhang, M., Zheng, H., & Xue, F. (2007). Effects of tanshinone II sodium sulfonate plus cinepazide maleate on the hemorrheologic indexes and blood lipids in patients with acute cerebral infarction. Neural Regeneration Research, 2(4), 225-229.	Not related to Oral CHM
0623	Chuang, L., Shang, J., Bohua, Y., Gang, W., Ning, W., Xiaotong, L., ... & Xi, L. (2021). Clinical efficacy of negative-pressure wound therapy with instillation of carboxymethyl chitosan bio-glue in diabetic foot ulcer. Chinese Journal of Diabetes Mellitus, 13(3).	Not related to Oral CHM
0624	Leung, P. C., Koon, C. M., Lau, C. B. S., Chook, P., Cheng, W. K. F., Fung, K. P., ... & Woo, K. S. (2014). Development of an effective cardiovascular protective agent using evidence-based research platforms. Experimental and Clinical Cardiology.	Not related to Oral CHM
0625	Leung L, Birtwhistle R, Kotecha J, Hannah S, Cuthbertson S. Anti-diabetic and hypoglycaemic effects of Momordica charantia (bitter melon): a mini review. Br J Nutr. 2009;102(12):1703-1708. doi:10.1017/S0007114509992054	Not related to Oral CHM
0626	Lee, M. S., Pittler, M. H., Guo, R., & Ernst, E. (2007). Qigong for hypertension: a systematic review of randomized clinical trials. Journal of hypertension, 25(8), 1525-1532.	Not related to Oral CHM
0627	Lee MS, Pittler MH, Guo R, Ernst E. Qigong for hypertension: a systematic review of randomized clinical trials. J Hypertens. 2007;25(8):1525-1532. doi:10.1097/HJH.0b013e328092ee18	Not related to Oral CHM
0628	LeBlanc ES, Smith NX, Vesco KK, Paul IM, Stevens VJ. Weight loss prior to pregnancy and subsequent gestational weight gain: Prepare, a randomized clinical trial. Am J Obstet Gynecol. 2021;224(1):99.e1-99.e14. doi:10.1016/j.ajog.2020.07.027	Not related to Oral CHM
0629	Lauche R, Fuller NR, Cramer H, Wardle J, Sibbritt D, Adams J. Associations between complementary medicine, satisfaction with body weight and shape, and the use of methods to lose or control weight: Results of a national survey of 8009 Australian women. Complement Ther Med. 2018;36:100-106. doi:10.1016/j.ctim.2017.12.008	Not related to Oral CHM
0630	Bonakdar RA, Sweeney MM, Garvey C, White AA, VanNoord MU. Case Report: Initial Successful Treatment of Migraine and Irritable Bowel Syndrome With a Low-FODMAP Diet. J Am Nutr Assoc. 2024;43(4):339-344. doi:10.1080/27697061.2023.2288081	Not related to Oral CHM
0631	Laterra G, Artale C, Sacchetta G, Contarini M. Intracardiac echocardiography probe via oesophageal to guide percutaneous left atrial appendage closure procedure: a case series. Eur Heart J Case Rep. 2023;7(6):ytad261. Published 2023 Jun 12. doi:10.1093/ehjcr/ytad261	Not related to Oral CHM
0632	Lata S, Venkatesh P, Temkar S, et al. COMPARATIVE EVALUATION OF ANTERIOR SEGMENT OPTICAL COHERENCE TOMOGRAPHY, ULTRASOUND BIOMICROSCOPY, AND INTRAOCULAR PRESSURE CHANGES AFTER PANRETINAL PHOTOCOAGULATION BY PASCAL AND CONVENTIONAL LASER. Retina. 2020;40(3):537-545.	Not related to Oral CHM

	doi:10.1097/IAE.0000000000002400	
0633	Lanham-New, S. (2006). Does the skeleton play a role in acid-base homeostasis. In Current evidence: future perspectives. München 2nd acid-ase Symposium (Vol. 8).	Not related to Oral CHM
0634	Lam DC, Lui MM, Lam JC, Ong LH, Lam KS, Ip MS. Prevalence and recognition of obstructive sleep apnea in Chinese patients with type 2 diabetes mellitus. Chest. 2010;138(5):1101-1107. doi:10.1378/chest.10-0596	Not related to Oral CHM
0635	Lai CL, Lau JY, Wu PC, et al. Recombinant interferon-alpha in inoperable hepatocellular carcinoma: a randomized controlled trial. Hepatology. 1993;17(3):389-394.	Not related to Oral CHM
0636	Kwok AK, Lam DS, Ng JS, Fan DS, Chew SJ, Tso MO. Ocular-hypertensive response to topical steroids in children. Ophthalmology. 1997;104(12):2112-2116. doi:10.1016/s0161-6420(97)30052-9	Not related to Oral CHM
0637	Kwan, JJ, Yu, DSF, Choi, KC, Yeung, GSP and Yeung, VTF, 2014.The effectiveness of auriculotherapy in improving sleep quality and glycemic control in patients with type 2 diabetes, Diabetes Research and Clinical Practice.	Not related to Oral CHM
0638	Kuo CS, Pei D, Yao CY, Hsieh MC, Kuo SW. Effect of orlistat in overweight poorly controlled Chinese female type 2 diabetic patients: a randomised, double-blind, placebo-controlled study. Int J Clin Pract. 2006;60(8):906-910. doi:10.1111/j.1742-1241.2006.01052.x	Not related to Oral CHM
0639	Kuang AK, Wang CX. Zhong Xi Yi Jie He Za Zhi. 1986;6(1):9-2.	Not related to Oral CHM
0640	Maraschin, C. K., Bock, M. P., Leivas, G., Martins, F. A., Ramalho, R., Teló, H. G., & Schaan, D. B. (2022). IDF21-0087 Metabolic interventions for diabetes treatment are associated with gut microbiota changes: a systematic review. Diabetes Research and Clinical Practice, 186.	Not related to Oral CHM
0641	Kraft K, Coulon S. Der einfluss einer standardisierten akupunkturbehandlung auf beschwerden, blutdruck und serumlipide hypertensiver, postmenopausaler frauen. Randomisierte, kontrollierte klinische studie [Effect of a standardized acupuncture treatment on complains, blood pressure and serum lipids of hypertensive, postmenopausal women. A randomized, controlled clinical study]. Forsch Komplementarmed. 1999;6(2):74-79. doi:10.1159/000021223	Not related to Oral CHM
0642	Konlan KD, Shin J. Determinants of Self-Care and Home-Based Management of Hypertension: An Integrative Review. Glob Heart. 2023;18(1):16. Published 2023 Mar 20. doi:10.5334/gh.1190	Not related to Oral CHM

0643	Konin C, Boka B, Ekou A, et al. Recours à l'automédication chez l'hypertendu noir africain : ses facteurs et ses conséquences [Self-medication among black African hypertensive patients: Factors and consequences]. Ann Cardiol Angeiol (Paris). 2015;64(3):232-236. doi:10.1016/j.ancard.2015.04.001	Not related to Oral CHM
0644	Kongmalai T, Srinonprasert V, Anothaisintawee T, et al. New anti-diabetic agents for the treatment of non-alcoholic fatty liver disease: a systematic review and network meta-analysis of randomized controlled trials. Front Endocrinol (Lausanne). 2023;14:1182037. Published 2023 Jun 27. doi:10.3389/fendo.2023.1182037	Not related to Oral CHM
0645	Kong X, Huang X, Zhao M, et al. Platelet Count Affects Efficacy of Folic Acid in Preventing First Stroke. J Am Coll Cardiol. 2018;71(19):2136-2146. doi:10.1016/j.jacc.2018.02.072	Not related to Oral CHM
0646	Koncz D, Tóth B, Roza O, Csupor D. A Systematic Review of the European Rapid Alert System for Food and Feed: Tendencies in Illegal Food Supplements for Weight Loss. Front Pharmacol. 2021;11:611361. Published 2021 Jan 26. doi:10.3389/fphar.2020.611361	Not related to Oral CHM
0647	Klowak, JA, Shen, JW, Aguirre, E, Kam, AJ, Samaan, CM and Parker, MJ, 2018. Early identification of children with type 1 diabetes mellitus presenting to hospital with diabetic ketoacidosis: a pilot study, Canadian journal of anesthesia.	Not related to Oral CHM
0648	BASSAT, O. K. B., Brill, S., Vaisman, N., Schechter, M., Ashtamker, N., Hirshberg, B., & Mosenzon, O. (2021). POS-676 EXPLORATORY STUDY TO ASSESS THE SAFETY OF SUBLINGUAL OIL BASED MEDICAL CANNABIS BOL-DP-O-04 IN DIALYSIS PATIENTS WHO HAVE CHRONIC PAIN. Kidney International Reports, 6(4), S295.	Not related to Oral CHM
0649	Kioukia-Fougia, N., Georgiadis, N., Tsarouhas, K., Vasilaki, F., Fragiadaki, P., Meimeti, E., & Tsitsimpikou, C. (2016). Synthetic and Natural Nutritional Supplements: Health. Recent patents on inflammation & allergy drug discovery, 10(2), 72-85.	Not related to Oral CHM
0650	King DE, Mainous AG 3rd, Egan BM, Woolson RF, Geesey ME. Effect of psyllium fiber supplementation on C-reactive protein: the trial to reduce inflammatory markers (TRIM). Ann Fam Med. 2008;6(2):100-106. doi:10.1370/afm.819	Not related to Oral CHM
0651	Kim TH, Choi TY, Lee MS, Ernst E. Acupuncture treatment for cardiac arrhythmias: a systematic review of randomized controlled trials. Int J Cardiol. 2011;149(2):263-265. doi:10.1016/j.ijcard.2011.02.049	Not related to Oral CHM
0652	Kim KW, Kim SJ, Kim H, Shin SU, Song J, Chung WS. Clinical effects of slim-diet, with lifestyle modification for childhood obesity in community-based healthcare program: A case series. Medicine (Baltimore). 2020;99(26):e20817. doi:10.1097/MD.0000000000020817	Not related to Oral CHM
0653	Kim H, Kim KW, Chung WS. Effects of moxibustion for obesity: A protocol for systematic review and meta-analysis. Medicine (Baltimore). 2021;100(48):e27910. doi:10.1097/MD.0000000000027910IF: 1.3 Q2	Not related to Oral CHM

0654	Kim D, Ham OK, Kang C, Jun E. Effects of auricular acupressure using Sinapsis alba seeds on obesity and self-efficacy in female college students. J Altern Complement Med. 2014;20(4):258-264. doi:10.1089/acm.2012.0283	Not related to Oral CHM
0655	Kiepe, M. S., & Keil, T. (2010). Systematic review of the effects of dance therapy on mental and physical illnesses. European Journal of Integrative Medicine, 2(4), 260-261.	Not related to Oral CHM
0656	Kario K, Nomura A, Harada N, et al. Efficacy of a digital therapeutics system in the management of essential hypertension: the HERB-DH1 pivotal trial. Eur Heart J. 2021;42(40):4111-4122. doi:10.1093/eurheartj/ehab559	Not related to Oral CHM
0657	Kapil V, Khambata RS, Robertson A, Caulfield MJ, Ahluwalia A. Dietary nitrate provides sustained blood pressure lowering in hypertensive patients: a randomized, phase 2, double-blind, placebo-controlled study. Hypertension. 2015;65(2):320-327. doi:10.1161/HYPERTENSIONAHA.114.04675	Not related to Oral CHM
0658	Kang W, Chen X, Li Z, et al. Unusual conservative treatment of a complicated pacemaker pocket infection: a case report. J Med Case Rep. 2019;13(1):49. Published 2019 Mar 3. doi:10.1186/s13256-019-1987-x	Not related to Oral CHM
0659	Kang, F., Yang, L., Guo, X., Chen, J., Chen, G., & Xie, C. (2021). Correlation between Dampness Syndrome and Body Composition and Its Effect on Weight Loss. European Journal of Integrative Medicine, 48, 102082.	Not related to Oral CHM
0660	Kang D, Shin WC, Kim T, et al. Systematic review and meta-analysis of the anti-obesity effect of cupping therapy. Medicine (Baltimore). 2023;102(24):e34039. doi:10.1097/MD.0000000000034039	Not related to Oral CHM
0661	Kan K, Zhu W, Lu F, et al. Contribution of Structured Self-Monitoring of Blood Glucose to the Glycemic Control and the Quality of Life in Both Insulin- and Noninsulin-Treated Patients with Poorly Controlled Diabetes. Diabetes Technol Ther. 2017;19(12):707-714. doi:10.1089/dia.2017.0275	Not related to Oral CHM
0662	Kamprath S, Timmer A. Prioritisation of clinical research by the example of type 2 diabetes: a caregiver-survey on perceived relevance and need for evidence. PLoS One. 2012;7(3):e32414. doi:10.1371/journal.pone.0032414	Not related to Oral CHM
0663	Kamaria, K., Long, C. M., & Nurhanisah, S. (2015). Traditional Malay massage as an alternative treatment approaches in rehabilitation: a narrative review the state of evidence. Physiotherapy, 101, e706.	Not related to Oral CHM
0664	Kalish LA, Buczynski B, Connell P, et al. Stop Hypertension with the Acupuncture Research Program (SHARP): clinical trial design and screening results. Control Clin Trials. 2004;25(1):76-103. doi:10.1016/j.cct.2003.08.006	Not related to Oral CHM
0665	Junling G, Yang L, Junming D, Pinpin Z, Hua F. Evaluation of group visits for Chinese hypertensives based on primary health care center. Asia Pac J Public Health. 2015;27(2):NP350-NP360. doi:10.1177/1010539512442566	Not related to Oral CHM

0666	Jin X, Pan B, Wu H, Xu D. The effects of traditional Chinese exercise on hypertension: A systematic review and meta-analysis of randomized controlled trials. Medicine (Baltimore). 2019;98(3):e14049. doi:10.1097/MD.0000000000014049	Not related to Oral CHM
0667	Jin L, He LR, Fu DY. Zhongguo Zhong Xi Yi Jie He Za Zhi. 2011;31(3):315-318.	Not related to Oral CHM
0668	Jin, D, Huang, WJ, Pang, B and Lian, FM, 2017. Research on key techniques of clinical evaluation of qi deficiency and blood stasis syndrome based on literature research and expert questionnaire, BMC Complementary and Alternative Medicine.	Not related to Oral CHM
0669	Jiang R, Zhang X, Zheng Y, et al. Acupuncture for metabolic syndrome: A protocol for systematic review and meta-analysis. Medicine (Baltimore). 2022;101(43):e31532. doi:10.1097/MD.0000000000031532	Not related to Oral CHM
0670	Jiang HQ, Li YL, Xie J. Zhongguo Zhong Xi Yi Jie He Za Zhi. 2012;32(3):333-337.	Not related to Oral CHM
0671	Jia Y, Yu Y, Huang H. Network meta-analysis of four kinds of traditional Chinese exercise therapy in the treatment of type 2 diabetes: protocol for a systematic review. BMJ Open. 2021;11(7):e048259. Published 2021 Jul 9. doi:10.1136/bmjopen-2020-048259IF: 2.4 Q1	Not related to Oral CHM
0672	Ji LN, Pan CY, Lu JM, et al. Efficacy and safety of combination therapy with vildagliptin and metformin versus metformin uptitration in Chinese patients with type 2 diabetes inadequately controlled with metformin monotherapy: a randomized, open-label, prospective study (VISION). Diabetes Obes Metab. 2016;18(8):775-782. doi:10.1111/dom.12667	Not related to Oral CHM
0673	Ji LN, Pan CY, Lu JM, et al. Efficacy and safety of combination therapy with vildagliptin and metformin versus metformin up-titration in Chinese patients with type 2 diabetes mellitus: study design and rationale of the vision study. Cardiovasc Diabetol. 2013;12:118. Published 2013 Aug 19. doi:10.1186/1475-2840-12-118	Not related to Oral CHM
0674	Ji L, Su Q, Feng B, et al. Glycemic control and self-monitoring of blood glucose in Chinese patients with type 2 diabetes on insulin: Baseline results from the COMPASS study. Diabetes Res Clin Pract. 2016;112:82-87. doi:10.1016/j.diabres.2015.08.005	Not related to Oral CHM
0675	Ji L, Ma J, Li H, et al. Dapagliflozin as monotherapy in drug-naive Asian patients with type 2 diabetes mellitus: a randomized, blinded, prospective phase III study. Clin Ther. 2014;36(1):84-100.e9. doi:10.1016/j.clinthera.2013.11.002	Not related to Oral CHM
0676	Ji, L., Liu, J., Xu, Z. J., Wei, Z., Zhang, R., Malkani, S., ... & Frederich, R. (2023). Efficacy and safety of ertugliflozin added to metformin: a pooled population from Asia with type 2 diabetes and overweight or obesity. Diabetes Therapy, 14(2), 319-334.	Not related to Oral CHM

0677	Ji L, Gao L, Jiang H, et al. Safety and efficacy of a GLP-1 and glucagon receptor dual agonist mazdutide (IBI362) 9 mg and 10 mg in Chinese adults with overweight or obesity: A randomised, placebo-controlled, multiple-ascending-dose phase 1b trial. EClinicalMedicine. 2022;54:101691. Published 2022 Oct 7. doi:10.1016/j.eclinm.2022.101691	Not related to Oral CHM
0678	Ji L, Chen L, Wang Y, et al. Study Protocol for a Prospective, Multicenter, Randomized, Open-Label, Parallel-Group Clinical Trial Comparing the Efficacy and Safety of a Needle-Free Insulin Injector and a Conventional Insulin Pen in Controlling Blood Glucose Concentrations in Chinese Patients with Type 2 Diabetes Mellitus (The FREE Study). Adv Ther. 2019;36(6):1485-1496. doi:10.1007/s12325-019-00951-4IF: 3.4 Q2	Not related to Oral CHM
0679	Jeong HS, Lim HS, Park HJ, et al. Clinical outcomes between calcium channel blockers and angiotensin receptor blockers in hypertensive patients without established cardiovascular diseases during a 3-year follow-up. Sci Rep. 2021;11(1):1783. Published 2021 Jan 19. doi:10.1038/s41598-021-81373-7	Not related to Oral CHM
0680	Jayathissa, S. K., Lim, A., Wyeth, J., Garret, S., Yee, S. A., Metcalfe, S., & Weatherall, M. (2015). Adherence to dabigatran among New Zealand patients. Clinical Therapeutics, 37(8), e1-e2.	Not related to Oral CHM
0681	Ishimitsu, T, Honda, T, Tojo, A and Rai, T, 2023. THERAPEUTIC EFFECTS OF ADDING THIAZIDE DIURETIC OR MINERAL CORTICOID RECEPTOR BLOCKER IN HYPERTENSIVE PATIENTS GIVEN ARB AND CALCIUM CHANNEL BLOCKER, Journal of Hypertension.	Not related to Oral CHM
0682	Innes, K., Selfe, T., Alexander, G., Bourguignon, C., Taylor, A., & Hinton, I. (2012). OA14. 03. Effects of yoga on sleep, mood, and related outcomes in older women with Restless Legs Syndrome: a nested randomized controlled trial (RCT). BMC Complementary and Alternative Medicine, 12, 1-1.	Not related to Oral CHM
0683	Huo Y, Jing ZC, Zeng XF, et al. Evaluation of efficacy, safety and tolerability of Ambrisentan in Chinese adults with pulmonary arterial hypertension: a prospective open label cohort study. BMC Cardiovasc Disord. 2016;16(1):201. Published 2016 Oct 22. doi:10.1186/s12872-016-0361-9	Not related to Oral CHM
0684	Huntley C, Boon M, Tschopp S, et al. Comparison of Traditional Upper Airway Surgery and Upper Airway Stimulation for Obstructive Sleep Apnea. Ann Otol Rhinol Laryngol. 2021;130(4):370-376. doi:10.1177/0003489420953178	Not related to Oral CHM
0685	Hügel HM, Jackson N, May B, Zhang AL, Xue CC. Polyphenol protection and treatment of hypertension. Phytomedicine. 2016;23(2):220-231. doi:10.1016/j.phymed.2015.12.012	Not related to Oral CHM
0686	Huang Y, Ye D, Ma T. Zhonghua Fu Chan Ke Za Zhi. 1996;31(11):667-669.	Not related to Oral CHM

0687	Huang Y, Meng J, Sun B, et al. Acupuncture for serum uric acid in patients with asymptomatic hyperuricemia: A randomized, double-blind, placebo-controlled trial. Int J Cardiol. 2017;232:227-232. doi:10.1016/j.ijcard.2017.01.016	Not related to Oral CHM
0688	Huang W, Kutner N, Bliwise DL. Autonomic activation in insomnia: the case for acupuncture. J Clin Sleep Med. 2011;7(1):95-102.	Not related to Oral CHM
0689	Huang R, Feng Y, Wang Y, et al. Comparative Efficacy and Safety of Antihypertensive Agents for Adult Diabetic Patients with Microalbuminuric Kidney Disease: A Network Meta-Analysis. PLoS One. 2017;12(1):e0168582. Published 2017 Jan 3. doi:10.1371/journal.pone.0168582IF: 2.9 Q1	Not related to Oral CHM
0690	Huang P, Zhong Z, Wu L, Liu W. Increased iridial pigmentation in Chinese eyes after use of travoprost 0.004%. J Glaucoma. 2009;18(2):153-156. doi:10.1097/IJG.0b013e31817d2372	Not related to Oral CHM
0691	Huang KY, Huang CJ, Hsu CH. Efficacy of Acupuncture in the Treatment of Elderly Patients with Hypertension in Home Health Care: A Randomized Controlled Trial. J Altern Complement Med. 2020;26(4):273-281. doi:10.1089/acm.2019.0172	Not related to Oral CHM
0692	Huang K, Shi Y, Chu N, et al. The effect of food on the pharmacokinetics of WXFL10203614, a potential selective JAK1 inhibitor, in healthy Chinese subjects. Front Pharmacol. 2022;13:1066895. Published 2022 Nov 24. doi:10.3389/fphar.2022.1066895	Not related to Oral CHM
0693	Hu X, Zhang Q, Zheng Y, et al. A single-dose, randomized, open-labeled, parallel-group study comparing the pharmacokinetics, pharmacodynamics and safety of leuprolide acetate microspheres 3.75 聽mg and Enantone庐聽 3.75 聽mg in healthy male subjects.聽Front Pharmacol. 2022;13:946505. Published 2022 Aug 19. doi:10.3389/fphar.2022.946505	Not related to Oral CHM
0694	Hu, X., Xie, Q., Zhang, Y., Guo, X., Liu, S., Luo, S., ... & Weng, J. (2017, June). The Efficacy of an Electronic System-Guided Follow-Up in Chinese T2DM Patients Initiating Basal Insulin Therapy. In DIABETES (Vol. 66, pp. A270-A270). 1701 N BEAUREGARD ST, ALEXANDRIA, VA 22311-1717 USA: AMER DIABETES ASSOC.	Not related to Oral CHM
0695	Hu RX, Xiong XJ, Chen RB, et al. Zhongguo Zhong Yao Za Zhi. 2021;46(6):1547-1557. doi:10.19540/j.cnki.cjcmm.20200730.502	Not related to Oral CHM
0696	Hu P, Bartlett M, Karan RS, et al. Pharmacokinetics, safety and tolerability of single and multiple oral doses of aliskiren in healthy Chinese subjects: a randomized, single-blind, parallel-group, placebo-controlled study. Clin Drug Investig. 2010;30(4):221-228. doi:10.2165/11533050-000000000-00000	Not related to Oral CHM
0697	Hu G, Zhang H, Wang Y, Cong D. Non-pharmacological intervention for rehabilitation of post-stroke spasticity: A protocol for systematic review and network meta-analysis. Medicine (Baltimore). 2021;100(18):e25788. doi:10.1097/MD.0000000000025788	Not related to Oral CHM

0698	Hu DY, Zhang HP, Sun YH, Jiang LQ; Antithrombotic Therapy in Atrial Fibrillation Study Group. Zhonghua Xin Xue Guan Bing Za Zhi. 2006;34(4):295-298.	Not related to Oral CHM
0699	Hsu PC, Wu HK, Huang YC, et al. The tongue features associated with type 2 diabetes mellitus. Medicine (Baltimore). 2019;98(19):e15567. doi:10.1097/MD.0000000000015567	Not related to Oral CHM
0700	Hou W, Zhang D, Lu W, et al. Polymorphism of organic cation transporter 2 improves glucose-lowering effect of metformin via influencing its pharmacokinetics in Chinese type 2 diabetic patients. Mol Diagn Ther. 2015;19(1):25-33. doi:10.1007/s40291-014-0126-z	Not related to Oral CHM
0701	Hou S, Xiao Y, Li Y. Spontaneous Retinal Reperfusion of Capillary Nonperfusion Areas in Diabetic Retinopathy: A Comparative Angiographic Illustration by Fluorescence Fundus Angiography and Optic Coherence Tomography Angiography. Case Rep Ophthalmol. 2022;13(3):809-815. Published 2022 Nov 9. doi:10.1159/000527378	Not related to Oral CHM
0702	Horikiri K, Kikuta S, Kanaya K, et al. Intravenous olfactory test latency correlates with improvement in post-infectious olfactory dysfunction. Acta Otolaryngol. 2017;137(10):1083-1089. doi:10.1080/00016489.2017.1325005	Not related to Oral CHM
0703	Hopkins KL, Hlongwane KE, Otwombe K, et al. Does peer-navigated linkage to care work? A cross-sectional study of active linkage to care within an integrated non-communicable disease-HIV testing centre for adults in Soweto, South Africa. PLoS One. 2020;15(10):e0241014. Published 2020 Oct 22. doi:10.1371/journal.pone.0241014IF: 2.9 Q1	Not related to Oral CHM
0704	Hoosen, M., Roman, N. V., & Mthembu, T. G. (2021). The role of spirituality and spiritual care in complementary and alternative medicine: A scoping review. Bangladesh Journal of Medical Science, 20(4), 714-724.	Not related to Oral CHM
0705	Hong HY, Chen YS, Hong ZS, et al. Zhongguo Zhen Jiu. 2014;34(7):713-716.	Not related to Oral CHM
0706	Hollifield M, Cocozza K, Calloway T, et al. Improvement in Long-COVID Symptoms Using Acupuncture: A Case Study. Med Acupunct. 2022;34(3):172-176. doi:10.1089/acu.2021.0088IF: 0.8 Q4	Not related to Oral CHM
0707	Hogg W, Lemelin J, Dahrouge S, et al. Randomized controlled trial of anticipatory and preventive multidisciplinary team care: for complex patients in a community-based primary care setting. Can Fam Physician. 2009;55(12):e76-e85.	Not related to Oral CHM
0708	Ho, L., Chen, P., Lai, C., & Ng, S. (2021, October). NON-PHARMACOLOGICAL INTERVENTIONS FOR ALLEVIATING FATIGUE AND PROMOTING SLEEP IN PEOPLE WITH STROKE: A META-ANALYSIS. In INTERNATIONAL JOURNAL OF STROKE (Vol. 16, No. 2_ SUPPL, pp. 39-39). 1 OLIVERS YARD, 55 CITY ROAD, LONDON EC1Y 1SP, ENGLAND: SAGE PUBLICATIONS LTD.	Not related to Oral CHM

0709	Hernandez-Rodas MC, Valenzuela R, Videla LA. Relevant Aspects of Nutritional and Dietary Interventions in Non-Alcoholic Fatty Liver Disease. Int J Mol Sci. 2015;16(10):25168-25198. Published 2015 Oct 23. doi:10.3390/ijms161025168	Not related to Oral CHM
0710	Heng WK, Ng YP, Ooi GS, et al. Comparison of the efficacy and level of adherence for morning versus evening versus before bedtime administration of simvastatin in hypercholesterolemic patients. Med J Malaysia. 2019;74(6):477-482.	Not related to Oral CHM
0711	Heianza Y, Zhou T, Sun D, Hu FB, Qi L. Healthful plant-based dietary patterns, genetic risk of obesity, and cardiovascular risk in the UK biobank study. Clin Nutr. 2021;40(7):4694-4701. doi:10.1016/j.clnu.2021.06.018	Not related to Oral CHM
0712	He P, Luo X, Li J, et al. Clinical Outcome between Ticagrelor versus Clopidogrel in Patients with Acute Coronary Syndrome and Diabetes. Cardiovasc Ther. 2021;2021:5546260. Published 2021 Oct 15. doi:10.1155/2021/5546260	Not related to Oral CHM
0713	He P, Li H, Zhang Y, et al. Prospective association between baseline plasma zinc concentration and development of proteinuria in Chinese hypertensive patients. J Trace Elem Med Biol. 2021;66:126755. doi:10.1016/j.jtemb.2021.126755IF: 3.6 Q2	Not related to Oral CHM
0714	He L, Du P, Shen Z, Wang X. Zhongguo Zhen Jiu. 2016;36(3):245-249.	Not related to Oral CHM
0715	He J, Wen L, Jiang R, et al. Zhonghua Xin Xue Guan Bing Za Zhi. 2013;41(6):493-496.	Not related to Oral CHM
0716	Hashm, H., Khan, A., Zaidi, N., Scott, A., Schreier, M., Troutman, A., & Qasim, N. (2019). Takotsubo cardiomyopathy secondary to benzodiazepine tapering: An underreported complication. Chest, 156(4), A603-A604.	Not related to Oral CHM
0717	Harlalka S , Roy UK , Majumdar G , Das K , Mandal P . An Open Label Prospective Study on Evaluation of Safety and Efficacy of Cilnidipine Over Amlodipine in Stage 1 Hypertensive Patients. Kathmandu Univ Med J (KUMJ). 2020;18(69):42-48.	Not related to Oral CHM
0718	Hardavella GF, Dionellis GS, Kantza CG, Koulouris NG, Alchanatis M. Latest therapeutic novelties and patents in pulmonary hypertension. Recent Pat Cardiovasc Drug Discov. 2011;6(1):55-60. doi:10.2174/157489011794578491	Not related to Oral CHM
0719	Hao Y, Xu H. A Prospective Cohort Study on the Management of Young Patients with Newly Diagnosed Type 2 Diabetes Using Mobile Medical Applications. Diabetes Ther. 2018;9(5):2099-2106. doi:10.1007/s13300-018-0506-1IF: 2.8 Q3	Not related to Oral CHM
0720	Han, Y., Geng, H., Feng, W., Tang, X., Ou, A., Lao, Y., ... & Li, Y. (2003). A follow-up study of 69 discharged SARS patients. Journal of traditional Chinese medicine= Chung i tsa chih ying wen pan, 23(3), 214-217.	Not related to Oral CHM

072 1	Han Y, Cheng B, Guo Y, Wang Q, Yang N, Lin P. A low-carbohydrate diet realizes medication withdrawal: a possible opportunity for effective glycemic control. Front Endocrinol (Lausanne). 2021;12:779636. doi:10.3389/fendo.2021.779636	Not related to Oral CHM
072 2	Han, S. H., Li, K. Z., Zheng, J. M., Zheng, Z. X., Lin, M. C., Xu, M. Y., & Yue, Z. C. (2013). Study on the distribution of Chinese medical constitutions of hypertension complicated diabetes patients. Zhongguo Zhong xi yi jie he za zhi Zhongguo Zhongxiyi Jiehe Zazhi= Chinese Journal of Integrated Traditional and Western Medicine, 33(2), 199-204.	Not related to Oral CHM
072 3	Han JX, Jin X, Liu CZ, Wang T, Lu MX. Effects of acupuncture on blood-lipid levels in patients with hyperlipidemia. Zhongguo Zhen Jiu. 2006;26(6):403-406.	Not related to Oral CHM
072 4	Hamidi, M., & Choopani, R. (2022). Phytotherapy with silymarin: A clinical trial on infants with jaundice. Journal of Clinical Practice and Research, 44(5), 455.	Not related to Oral CHM
072 5	Halmesvaara O, Vornanen M, Kääriäinen H, Peltonen L, Palotie A, Jousilahti P, et al. Psychosocial effects of receiving genome-wide polygenic risk information: a randomized controlled trial. Eur J Hum Genet. 2022;30(7):775-782. doi:10.1038/s41431-022-01002-5.	Not related to Oral CHM
072 6	Haller MJ, Gitelman SE, Gottlieb PA, Michels AW, Rosenthal SM, Shuster JJ, et al. Anti-thymocyte globulin/G-CSF combination therapy in patients with established type 1 diabetes: the START trial. Diabetes. 2016;65(12):3760-3769. doi:10.2337/db16-0650.	Not related to Oral CHM
072 7	Haller MJ, Gitelman SE, Gottlieb PA, Michels AW, Rosenthal SM, Shuster JJ, et al. Anti-thymocyte globulin/G-CSF combination therapy in patients with established type 1 diabetes: the START trial. Diabetes. 2016;65(12):3760-3769. doi:10.2337/db16-0650.	Not related to Oral CHM
072 8	Haidari F, Samadi M, Mohammadshahi M, Jalali MT, Engali KA. Energy restriction combined with green coffee bean extract affects serum adipocytokines and the body composition in obese women. Asia Pac J Clin Nutr. 2017;26(6):1048-1054. doi:10.6133/apjcn.022017.03.	Not related to Oral CHM
072 9	Gysel, M., Crystal, A., Hancox, J. C., Methot, M., & Baranchuk, A. (2014). Multifactorial QT interval prolongation and Takotsubo cardiomyopathy. Case Reports in Cardiology, 2014(1), 213842.	Not related to Oral CHM
073 0	Gylling, H., Hallikainen, M., Nissinen, M. J., Simonen, P., & Miettinen, T. A. (2010). Very high plant stanol intake and serum plant stanols and non-cholesterol sterols. European journal of nutrition, 49, 111-117.	Not related to Oral CHM
073 1	Zhang F, Bai Y, Zhang J, et al. Beneficial effects of qigong Wuqinxi in the improvement of health condition in the middle-aged and elderly. Evid Based Complement Alternat Med. 2018;2018:3235950. doi:10.1155/2018/3235950.	Not related to Oral CHM

0732	Xue, G. U. O., Qin, L. I. U., Xiaoyu, Y. U., Baocheng, Y. U. A. N., Yang, W. A. N. G., & Runhua, W. A. N. G. (2012). Appropriate health technology assessment studies in China: A systematic review. Chinese Journal of Evidence-Based Medicine, 12(12), 1407-1415.	Not related to Oral CHM
0733	Grewal AS, Bhardwaj S, Pandita D, Lather V, Sekhon BS. Updates on aldose reductase inhibitors for management of diabetic complications and non-diabetic diseases. Mini Rev Med Chem. 2016;16(2):120-162. doi:10.2174/1389557515666150916113004.	Not related to Oral CHM
0734	Greeson JM, Rosenzweig S, Halbert SC, Cantor D, Keener P, Brainard GC. Integrative medicine research at an academic medical center: patient characteristics and health-related quality-of-life outcomes. J Altern Complement Med. 2008;14(6):763-767. doi:10.1089/acm.2008.0114	Not related to Oral CHM
0735	Greenfield SF, Borkan J, Yodfat Y. Health beliefs and hypertension: a case-control study in a Moroccan Jewish community in Israel. Cult Med Psychiatry. 1987;11(4):465-478. doi:10.1007/BF00114809.	Not related to Oral CHM
0736	Gou ZP, Wang ZL, Liang XF, et al. Single-dose escalation study of yogliptin in healthy Chinese subjects. Eur J Pharm Sci. 2019;137:104988. doi:10.1016/j.ejps.2019.104988.	Not related to Oral CHM
0737	O'Brien, K. (2019). Medicinal cannabis: Issues of evidence. European Journal of Integrative Medicine, 28, 114-120.	Not related to Oral CHM
0738	Gong ZC, Huang Q, Dai XP, et al. NeuroD1 A45T and PAX4 R121W polymorphisms are associated with plasma glucose level of repaglinide monotherapy in Chinese patients with type 2 diabetes. Br J Clin Pharmacol. 2012;74(3):486-495. doi:10.1111/j.1365-2125.2012.04225.x	Not related to Oral CHM
0739	Golledge J, Thanigaimani S. Nox4 as a novel therapeutic target for diabetic vascular complications. Front Pharmacol. 2023;14:1173950. doi:10.3389/fphar.2023.1173950.	Not related to Oral CHM
0740	Goertz CM, Long CR, Vining RD, et al. Chiropractic for hypertension in patients (CHiP): a pilot randomized controlled trial. J Altern Complement Med. 2014;20(5):A102	Not related to Oral CHM
0741	Godsey, J. (2013). The role of mindfulness based interventions in the treatment of obesity and eating disorders: an integrative review. Complementary therapies in medicine, 21(4), 430-439.	Not related to Oral CHM
0742	Gerstein HC, Coleman RL, Scott CAB, et al. Impact of acarbose on incident diabetes and regression to normoglycemia in people with impaired glucose tolerance and coronary heart disease: a randomized controlled trial. Diabetes Care. 2020;43(8):1807-1815. doi:10.2337/dc20-0293.	Not related to Oral CHM
0743	George J, McNamara K, Jackson S, et al. Evaluation of a community pharmacy-based intervention for improving patient adherence to antihypertensive medication and blood pressure control: the HAPPY trial. J Clin Pharm Ther. 2010;35(5):544-552. doi:10.1111/j.1365-2710.2009.01123.x	Not related to Oral CHM

0744	Garcia-Vivas JM, Galaviz-Hernandez C, Becerril-Chavez F, et al. Acupoint catgut embedding therapy with moxibustion reduces the risk of diabetes in obese women. J Res Med Sci. 2014;19(7):610-616.	Not related to Oral CHM
0745	Gao, Y., Xie, Y. M., Wang, G. Q., Cai, Y. F., Shen, X. M., Zhao, D. X., ... & Wei, R. L. (2022). Onset and recurrence characteristics of Chinese patients with noncardiogenic ischemic stroke in Chinese medicine hospital. Chinese journal of integrative medicine, 28(6), 492-500.	Not related to Oral CHM
0746	Gao, Y., Guo, X., Han, P., Li, Q., Yang, G., Qu, S., ... & Gu, L. (2015). Treatment of patients with diabetic peripheral neuropathic pain in China: a double-blind randomised trial of duloxetine vs. placebo. International Journal of Clinical Practice, 69(9), 957-966.	Not related to Oral CHM
0747	Gao R, Yang Y, Han Y, et al. Bioresorbable vascular scaffolds versus metallic stents in patients with coronary artery disease: ABSORB China trial. J Am Coll Cardiol. 2015;66(21):2298-2309. doi:10.1016/j.jacc.2015.09.054.	Not related to Oral CHM
0748	Gao L, Ji L, Su Q, et al. Impact of structured self-monitoring of blood glucose on quality of life in patients with type 2 diabetes: a randomized controlled study. Diabetes Res Clin Pract. 2016;112:88-95. doi:10.1016/j.diabres.2015.11.015	Not related to Oral CHM
0749	Gao, C., Yan, Z., Wang, M., Liu, Y., Zhong, M., & Zhang, G. (2020). Effects of compression treatment on occurrence of seroma after tension-free inguinal hernia repair based on propensity score matching. Chinese Journal of Digestive Surgery, 742-750.	Not related to Oral CHM
0750	Gao B, Gao W, Wan H, et al. Efficacy and safety of alogliptin versus acarbose in Chinese type 2 diabetes patients with high cardiovascular risk or coronary heart disease treated with aspirin and inadequately controlled with metformin monotherapy or drug-naive: a multicentre, randomized, open-label, prospective study (ACADEMIC). Diabetes Obes Metab. 2022;24(4):684-692. doi:10.1111/dom.14638.	Not related to Oral CHM
0751	Gao B, Gao W, Wan H, et al. Efficacy and safety of alogliptin versus acarbose in Chinese type 2 diabetes patients inadequately controlled with metformin monotherapy: a randomized, double-blind, phase III study. Diabetes Obes Metab. 2022;24(4):684-692. doi:10.1111/dom.14638.	Not related to Oral CHM
0752	Vas P, Rayman G, Dhatariya K, et al. Effectiveness of interventions to enhance healing of chronic foot ulcers in diabetes: a systematic review. Diabetes Metab Res Rev. 2020;36(Suppl 1):e3284. doi:10.1002/dmrr.3284.	Not related to Oral CHM
0753	Leshchinski沫 LA, Ga沫sin IR, Maksimov NI. [Basic and metabolic therapy of hypertensive disease in pregnant women]. Klin Med (Mosk). 2008;86(9):25-28. Russian	Not related to Oral CHM
0754	Flachskampf FA, Gallasch J, Gefeller O, et al. Randomized trial of acupuncture to lower blood pressure. Circulation. 2007;115(24):3121-3129. doi:10.1161/CIRCULATIONAHA.106.661140	Not related to Oral CHM

0755	Fukui, J. A., Rothwell, A., Danesh, H., Adelson, K. B., Morris, G. J., Irie, H., ... & Tiersten, A. (2014). Comparison of weight loss among early-stage breast cancer patients post chemotherapy: Nutrition education in combination with weight loss acupuncture versus nutrition education alone.	Not related to Oral CHM
0756	Fu, Y., Zhang, Y., Xiao, D., Xu, Y., & Xuan, J. (2020). PDB37 COST-EFFECTIVENESS OF ACARBOSE IN TREATING AND MANAGING CHINESE IMPAIRED GLUCOSE TOLERANCE (IGT) POPULATION. Value in Health, 23, S114-S115.	Not related to Oral CHM
0757	Gallagher A, Henderson W, Abraira C. Dietary patterns and metabolic control in diabetic diets: a prospective study of 51 outpatient men on unmeasured and exchange diets. J Am Coll Nutr. 1987;6(6):525-532. doi:10.1080/07315724.1987.10720212.	Not related to Oral CHM
0758	Floccari, F., Marrocco, F., Rivera, R., & Di Lullo, L. (2019). Prevenzione primaria della malattia cardiovascolare: c'è bisogno di un'aspirina?. Giornale di Tecniche Nefrologiche e Dialitiche, 31(3), 167-170.	Not related to Oral CHM
0759	Flachskampf, F. A., Gallasch, J., Gefeller, O., Gan, J., Mao, J., Pfahlberg, A. B., ... & Daniel, W. G. (2007). Randomized trial of acupuncture to lower blood pressure. Circulation, 115(24), 3121-3129.	Not related to Oral CHM
0760	Zhang Y, Zhang Y, Yu T, et al. Targeting the T-type calcium channel Cav3.2 in GABAergic arcuate nucleus neurons to treat obesity. Mol Metab. 2021;54:101352. doi:10.1016/j.molmet.2021.101352.	Not related to Oral CHM
0761	Palomba S, Falbo A, Valli B, et al. Effect of a dietary and exercise intervention in women with overweight and obesity undergoing fertility treatments: protocol for a randomized controlled trial. Contemp Clin Trials. 2021;108:106483. doi:10.1016/j.cct.2021.106483.	Not related to Oral CHM
0762	Farahmand SK, Saghebi SA, Mohammadi M, et al. The effects of wet cupping on coronary risk factors in patients with metabolic syndrome: a randomized controlled trial. Am J Chin Med. 2012;40(2):269-277. doi:10.1142/S0192415X12500218.	Not related to Oral CHM
0763	Farahmand SK, Saghebi SA, Mohammadi M, et al. The effects of wet cupping on serum high-sensitivity C-reactive protein and heat shock protein 27 antibody titers in patients with metabolic syndrome. Complement Ther Med. 2014;22(4):640-644. doi:10.1016/j.ctim.2014.04.004.	Not related to Oral CHM
0764	Zhou Y, Zhang X, Li Y, et al. Effect of topical ropivacaine on the response to endotracheal tube during emergence from general anesthesia: a prospective randomized double-blind controlled study. J Anesth. 2018;32(6):857-863. doi:10.1007/s00540-018-2573-7.	Not related to Oral CHM
0765	Li J, Li Y, Zhang Y, et al. High-sensitivity C-reactive protein combined with low-density lipoprotein cholesterol as the targets of statin therapy in patients with acute coronary syndrome. Clin Cardiol. 2018;41(3):321-326. doi:10.1002/clc.22893.	Not related to Oral CHM

0766	Zhang Y, Wang Y, Li X, et al. The efficacy and safety of arotinolol combined with a different calcium channel blocker in the treatment of Chinese patients with essential hypertension: a one-year follow-up study. Clin Exp Hypertens. 2014;36(3):181-187. doi:10.3109/10641963.2013.804541.	Not related to Oral CHM
0767	Zhang Y, Wang Y, Li X, et al. Observation on treatment of dizziness mainly by acupuncture. J Tradit Chin Med. 2007;27(1):21-23.	Not related to Oral CHM
0768	Li Y, Wang Y, Zhang Y, et al. Comparison of repaglinide and metformin monotherapy as an initial therapy in Chinese patients with newly diagnosed type 2 diabetes mellitus. J Diabetes Investig. 2014;5(3):290-296. doi:10.1111/jdi.12147.	Not related to Oral CHM
0769	Wang Y, Li Y, Zhang Y, et al. Construct a classification decision tree model to select the optimal equation for estimating glomerular filtration rate and estimate it more accurately. BMC Nephrol. 2022;23(1):1-10. doi:10.1186/s12882-022-02813-4.	Not related to Oral CHM
0770	Zhang Y, Wang Y, Li X, et al. Effectiveness of acupuncture in treatment of simple obesity in animal models: a systematic review and meta-analysis. J Tradit Chin Med. 2019;39(4):547-556. doi:10.19852/j.cnki.jtcm.2019.04.015.	Not related to Oral CHM
0771	Li Y, Wang Y, Zhang Y, et al. Sex differences in blood pressure response to antihypertensive therapy in Chinese patients with hypertension. Am J Hypertens. 2008;21(11):1210-1216. doi:10.1038/ajh.2008.287.	Not related to Oral CHM
0772	Faintuch J, Horie LM, Barbeiro HV, et al. Systemic inflammation in morbidly obese subjects: response to oral supplementation with alpha-linolenic acid. Obes Surg. 2007;17(3):341-347. doi:10.1007/s11695-007-9062-x.	Not related to Oral CHM
0773	Etuk E, Isezuo SA, Chika A, Akuche J, Ali M. Prescription pattern of anti-hypertensive drugs in a tertiary health institution in Nigeria. Ann Afr Med. 2008;7(3):128-132. doi:10.4103/1596-3519.55665.	Not related to Oral CHM
0774	Esteghamati A, Mirmiranpour H, Shafaei A, et al. Effects of metformin on markers of oxidative stress and antioxidant reserve in patients with newly diagnosed type 2 diabetes: a randomized clinical trial. Clin Nutr. 2013;32(2):179-185. doi:10.1016/j.clnu.2012.08.014.	Not related to Oral CHM
0775	Espeland MA, Glick HA, Bertoni A, et al. Impact of an intensive lifestyle intervention on use and cost of medical services among overweight and obese adults with type 2 diabetes: the action for health in diabetes. Diabetes Care. 2014;37(9):2548-2556. doi:10.2337/dc14-0093.	Not related to Oral CHM
0776	Zimetbaum P, Goldman A. Dronedarone-digoxin interaction in PALLAS: a foxglove connection? N Engl J Med. 2011;365(23):2266-2267. doi:10.1056/NEJMc1110536.	Not related to Oral CHM
0777	Speight J, Amiel SA, Bradley C, et al. The 5x1 DAFNE study protocol: a cluster randomised trial comparing a standard 5 day DAFNE course delivered over 1 week against DAFNE training delivered over 1 day a week for 5 consecutive weeks. BMC Public Health. 2012;12:652.	Not related to Oral CHM

	doi:10.1186/1471-2458-12-652.	
0778	Wong ATY, Mannix C, Grantham JJ, et al. Randomised controlled trial of high versus ad libitum water intake in patients with autosomal dominant polycystic kidney disease: rationale and design of the DRINK feasibility trial. BMJ Open. 2018;8(5):e020889. doi:10.1136/bmjopen-2017-020889.	Not related to Oral CHM
0779	Ee C, Xue C, Chondros P, et al. A qualitative study exploring feasibility and acceptability of acupuncture, yoga, and mindfulness meditation for managing weight after breast cancer. Integr Cancer Ther. 2022;21:15347354221099540. doi:10.1177/15347354221099540.	Not related to Oral CHM
0780	Dumville JC, O'Meara S, Deshpande S, Speak K. Hydrocolloid dressings for healing diabetic foot ulcers. Cochrane Database Syst Rev. 2012;2012(2):CD009099. doi:10.1002/14651858.CD009099.pub2.	Not related to Oral CHM
0781	Gawaz M, Langer H, May AE. Platelets in inflammation and atherogenesis. J Clin Invest. 2005;115(12):3378-3384. doi:10.1172/JCI27196.	Not related to Oral CHM
0782	Dudeck O, Teichgräber U, Podrabsky P, et al. A randomized trial assessing the value of ultrasound-guided puncture of the femoral artery for interventional investigations. Int J Cardiovasc Imaging. 2004;20(4):363-368. doi:10.1023/B:CAIM.0000036532.70398.9d.	Not related to Oral CHM
0783	Qiao F, Wang Y, Zhang Y, et al. Strategy of integrated evaluation on treatment of traditional Chinese medicine as ‘interaction of system to system’ and establishment of novel fuzzy target contribution recognition with herb-pairs, a case study on Astragali Radix-Fructus Corni. J Ethnopharmacol. 2016;194:495-502. doi:10.1016/j.jep.2016.10.055.	Not related to Oral CHM
0784	Wang CT, Hu CX, Ban HP, et al. Acupuncture lowering blood pressure for secondary prevention of stroke: a study protocol for a multicenter randomized controlled trial. Trials. 2017;18(1):517. doi:10.1186/s13063-017-2171-5.	Not related to Oral CHM
0785	Li Y, Wang Y, Zhang Y, et al. The effect of dexmedetomidine on the perioperative hemodynamics and postoperative cognitive function of elderly patients with hypertension: study protocol for a randomized controlled trial. Medicine (Baltimore). 2018;97(43):e12795. doi:10.1097/MD.0000000000012795.	Not related to Oral CHM
0786	Douthit NT, Alemu HA. Factors affecting illness in the developing world: chronic disease, mental health and traditional medicine cures. BMJ Case Rep. 2016;2016:bcr2016215570. doi:10.1136/bcr-2016-215570.	Not related to Oral CHM
0787	Dou Z, Ma SH, Song JY, Xia T. Retrospective analysis on pregnancy outcomes and fat-related factors of treatment of endomorph PCOS infertility patients by acupuncture of 8 acupoints around umbilicus. Acupunct Res. 2021;46(2):158-163.	Not related to Oral CHM

0788	Khouzam RN. Electroconvulsive therapy-induced Takotsubo cardiomyopathy despite angiotensin-converting enzyme inhibitor use: an increasing phenomenon. J Am Coll Cardiol. 2020;75(11_Supplement_1):S1. doi:10.1016/S0735-1097(20)33179-X.	Not related to Oral CHM
0789	Donofry SD, Lesnovskaya A, Drake JA, et al. Obesity, psychological distress, and resting state connectivity of the hippocampus and amygdala among women with early-stage breast cancer. Front Hum Neurosci. 2022;16:848028. doi:10.3389/fnhum.2022.848028.	Not related to Oral CHM
0790	Zhang Y, Wang Y, Li X, et al. Comparing the efficacy of angiotensin receptor-neprilysin inhibitor and enalapril in acute anterior STEMI patients after primary percutaneous coronary intervention: a prospective randomized trial. Ann Transl Med. 2022;10(5):229. doi:10.21037/atm-21-6717.	Not related to Oral CHM
0791	Wan EYF, Yu EYT, Chin WY, et al. Prediction models and nomograms for 10-year risk of end-stage renal disease in Chinese type 2 diabetes mellitus patients in primary care. Diabetes Obes Metab. 2021;23(3):660-670. doi:10.1111/dom.14249.	Not related to Oral CHM
0792	Dong A, Zhang Y, Lu S, Yu W. Influence of Dexmedetomidine on Myocardial Injury in Patients with Simultaneous Pancreas-Kidney Transplantation. Evid Based Complement Alternat Med. 2022;2022:7196449. doi:10.1155/2022/7196449. [Retracted in: Evid Based Complement Alternat Med. 2023;2023:9860397.]	Not related to Oral CHM
0793	Mastropasqua R, Toto L, Di Nicola M, et al. Circulating levels of reactive oxygen species in patients with nonproliferative diabetic retinopathy and the influence of antioxidant supplementation: 6-month follow-up. Clin Ophthalmol. 2015;9:877-883. doi:10.2147/OPTH.S79627.	Not related to Oral CHM
0794	Doll R, Langman MJ, Shawdon HH. Treatment of gastric ulcer with carbenoxolone: antagonistic effect of spironolactone. Gut. 1968;9(1):42-45. doi:10.1136/gut.9.1.42.	Not related to Oral CHM
0795	Kinouchi K, Ichihara A, Bokuda K, Kurosawa H, Itoh H. Differential Effects in Cardiovascular Markers between High-Dose Angiotensin II Receptor Blocker Monotherapy and Combination Therapy of ARB with Calcium Channel Blocker in Hypertension (DEAR Trial). Int J Hypertens. 2011;2011:284823. doi:10.4061/2011/284823.	Not related to Oral CHM
0796	Djaja, N., Permadi, I., Witjaksono, F., Soewondo, P., Abdullah, M., Agustina, R., & Ali, S. (2019). The effect of Job's tears-enriched yoghurt on GLP-1, calprotectin, blood glucose levels and weight of patients with type 2 diabetes mellitus. Mediterranean Journal of Nutrition and Metabolism, 12(2), 163-171.	Not related to Oral CHM
0797	Dimitrova A, Murchison C, Oken B. The Case for Local Needling in Successful Randomized Controlled Trials of Peripheral Neuropathy: A Follow-Up Systematic Review. J Altern Complement Med. 2018;24(8):752-765. doi:10.1089/acm.2017.0301.	Not related to Oral CHM

079 8	Dimitrova A, Murchison C, Oken B. Acupuncture for the Treatment of Peripheral Neuropathy: A Systematic Review and Meta-Analysis. J Altern Complement Med. 2017;23(3):164-179. doi:10.1089/acm.2016.0155.	Not related to Oral CHM
079 9	Weidenhammer W, Linde K, Streng A, et al. Acupuncture in diabetic peripheral neuropathy-protocol for the randomized, multicenter ACUDPN trial. Trials. 2021;22(1):143. doi:10.1186/s13063-021-05091-1.	Not related to Oral CHM
080 0	Dickerson LM, Ye X, Sack JL, Hueston WJ. Glycemic control in medical inpatients with type 2 diabetes mellitus receiving sliding scale insulin regimens versus routine diabetes medications: a multicenter randomized controlled trial. Ann Fam Med. 2003;1(1):29-35. doi:10.1370/afm.2.	Not related to Oral CHM
080 1	Deng JL, He L, Jiang C, et al. [A comparison of CAS risk model and CHA2DS2-VASc risk model in guiding anticoagulation treatment in Chinese patients with non-valvular atrial fibrillation]. Zhonghua Xin Xue Guan Bing Za Zhi. 2022;50(9):888-894. doi:10.3760/cma.j.cn112148-20210826-00740.	Not related to Oral CHM
080 2	Deng H, Lin S, Yang X, et al. Effect of baseline body mass index on glycemic control and weight change with exenatide monotherapy in Chinese drug-naïve type 2 diabetic patients. J Diabetes. 2019;11(7):509-518. doi:10.1111/1753-0407.12883.	Not related to Oral CHM
080 3	Dellalibera, O., Lemaire, B., & Lafay, S. (2006). Svetol庐, green coffee extract, induces weight loss and increases the lean to fat mass ratio in volunteers with overweight problem.聽Phytotherapie,聽 4, 194-197.	Not related to Oral CHM
080 4	Davy, J., Khoueiry, Z., Cung, T. T., Massin, F., Cransac, F., Pasquié, J. L., & Roubille, F. (2017). Is adherence to non AVK therapy improved by a personnalized information program? A first look to MONACO study. Archives of Cardiovascular Diseases Supplements, 9(1), 82.	Not related to Oral CHM
080 5	Davidson MH, Johnson J, Rooney MW, Kyle ML, Kling DF. A novel omega-3 free fatty acid formulation has dramatically improved bioavailability during a low-fat diet compared with omega-3-acid ethyl esters: the ECLIPSE study. J Clin Lipidol. 2012;6(6):573-584. doi:10.1016/j.jacl.2012.01.002.	Not related to Oral CHM
080 6	Dasgupta K, Rosenberg E, Daskalopoulou SS, et al. Step Monitoring to improve ARTERial health (SMARTER) through step count prescription in type 2 diabetes and hypertension: trial design and methods. Cardiovasc Diabetol. 2014;13:7. doi:10.1186/1475-2840-13-7.	Not related to Oral CHM
080 7	Nematy M, Alinezhad-Namaghi M, Rashed MM, et al. Effects of body electroacupuncture on plasma leptin concentrations in obese and overweight people in Iran: a randomized controlled trial. Altern Ther Health Med. 2013;19(2):24-31.	Not related to Oral CHM
080 8	Nematy M, Alinezhad-Namaghi M, Rashed MM, et al. Effects of body electroacupuncture on plasma leptin concentrations in obese and overweight people in Iran: a randomized controlled trial. Altern Ther Health Med. 2013;19(2):24-31.	Not related to Oral CHM

0809	Dajani AI, Popovic B. Essential phospholipids for nonalcoholic fatty liver disease associated with metabolic syndrome: A systematic review and network meta-analysis. World J Gastroenterol. 2020;26(44):7173-7188. doi:10.3748/wjg.v26.i44.7173.	Not related to Oral CHM
0810	Li X, Liu Y, Zhang Y, et al. Effective treatment of Kimura's disease with leflunomide in combination with glucocorticoids. Clin Rheumatol. 2011;30(6):841-845. doi:10.1007/s10067-011-1671-7.	Not related to Oral CHM
0811	Dai G, Wang D, Dong H. Effects of recombinant human growth hormone on protein malnutrition and IGF-1 and IL-2 gene expression levels in chronic nephrotic syndrome. Exp Ther Med. 2018;15(5):4167-4172. doi:10.3892/etm.2018.5953.	Not related to Oral CHM
0812	Packer L, Rimbach G, Virgili F. Pycnogenol: a blend of procyanidins with multifaceted therapeutic applications? Fitoterapia. 2010;81(7):724-736. doi:10.1016/j.fitote.2010.06.011.	Not related to Oral CHM
0813	Coyle ME, Smith CA, Peat B. Cephalic version by moxibustion for breech presentation. Cochrane Database Syst Rev. 2012;(5):CD003928. doi:10.1002/14651858.CD003928.pub3.	Not related to Oral CHM
0814	Healey JS, Connolly SJ, Gold MR, et al. Integrated Management Program Advancing Community Treatment of Atrial Fibrillation (IMPACT-AF): a cluster randomized trial of a computerized clinical decision support tool. Am Heart J. 2020;223:96-105. doi:10.1016/j.ahj.2020.02.004.	Not related to Oral CHM
0815	Coutinho, M. S., Lima, F. A., Du Bú, E. A., & Araújo, C. R. F. (2021). Hypoglycemic potential of Cissus sicyoides L.(vegetal insulin).	Not related to Oral CHM
0816	Constantino TG, Parikh AK, Satz WA, Fojtik JP. Ultrasonography-guided peripheral intravenous access versus traditional approaches in patients with difficult intravenous access. Ann Emerg Med. 2005;46(5):456-461. doi:10.1016/j.annemergmed.2004.12.026.	Not related to Oral CHM
0817	Epel E, Daubenmier J, Moskowitz JT, et al. Effect of restorative yoga vs. stretching on salivary cortisol and psychosocial outcomes in individuals with the metabolic syndrome: the PRYSMS randomized controlled trial. J Behav Med. 2014;37(3):412-425. doi:10.1007/s10865-013-9506-6.	Not related to Oral CHM
0818	Coleman, K, 2019. Research on the correlated mechanisms between hypertension with liver depression and anxiety-depression, Journal of Alternative and Complementary Medicine.	Not related to Oral CHM
0819	Ward NC, Croft KD, Puddey IB, et al. The effect of grape seed extract and quercetin on cardiovascular and endothelial parameters in high-risk subjects. Int J Clin Pharmacol Ther. 2005;43(12):558-563. doi:10.5414/cpp43558.	Not related to Oral CHM
0820	Chung CY, Yang AWH, De Foe A, Li M, Lenon GB. The clinical evaluation of electroacupuncture combined with mindfulness meditation for overweight and obesity: study protocol for a randomized sham-controlled clinical trial. Trials. 2022;23(1):818. doi:10.1186/s13063-022-06725-8.	Not related to Oral CHM

0821	Chu YG, Shi J, Hu YH. [Serum proteomic study on hypertension patients with Gan-Dan damp-heat syndrome]. Zhongguo Zhong Xi Yi Jie He Za Zhi. 2010;30(1):37-41. Chinese.	Not related to Oral CHM
0822	Zhang Y, Li Y, Wang J, et al. Effect of acupuncture on hemorheology in patients with diabetic nephropathy. J Tradit Chin Med. 2007;27(4):265-267.	Not related to Oral CHM
0823	Chan JC, Chow CC, Cockram CS, et al. Dexfenfluramine in obese Chinese NIDDM patients. A placebo-controlled investigation of the effects on body weight, glycemic control, and cardiovascular risk factors. Diabetes Care. 1997;20(6):1092-1097. doi:10.2337/diacare.20.6.1092.	Not related to Oral CHM
0824	Chou, YC, Liao, CC and Chen, TL, 2018. Risk of diabetes in stroke patients receiving statin treatment: A nationwide matched cohort study, Clinical Neurology.	Not related to Oral CHM
0825	Liu Z, Liu Y, Xu H, et al. Effect of electroacupuncture on urinary leakage among women with stress urinary incontinence: a randomized clinical trial. JAMA. 2017;317(24):2493-2501. doi:10.1001/jama.2017.7220.	Not related to Oral CHM
0826	Cheung CY, Chan HW, Liu YL, Chau KF, Li CS. Paired kidney analysis of tacrolimus and cyclosporine microemulsion-based therapy in Chinese cadaveric renal transplant recipients. Nephrology (Carlton). 2006;11(5):442-446. doi:10.1111/j.1440-1797.2006.00655.x.	Not related to Oral CHM
0827	Cheung CY, Chan HW, Liu YL, Chau KF, Li CS. Long-term graft function with tacrolimus and cyclosporine in renal transplantation: paired kidney analysis. Nephrology (Carlton). 2009;14(8):758-763. doi:10.1111/j.1440-1797.2009.01155.x.	Not related to Oral CHM
0828	Lee MS, Lee MS, Kim HJ, Choi ES. Randomised controlled trial of qigong in the treatment of mild essential hypertension. J Hum Hypertens. 2005;19(9):697-704. doi:10.1038/sj.jhh.1001876.	Not related to Oral CHM
0829	Wang JG, Staessen JA, Gong L, Liu L. Amlodipine, felodipine, and isradipine in the treatment of Chinese patients with mild-to-moderate hypertension. Am J Hypertens. 1999;12(5 Pt 1):437-443. doi:10.1016/s0895-7061(98)00256-6.	Not related to Oral CHM
0830	Tangney CC, Rasmussen HE. Polyphenols, inflammation, and cardiovascular disease. Curr Atheroscler Rep. 2013;15(5):324. doi:10.1007/s11883-013-0324-x.	Not related to Oral CHM
0831	Zhang Y, Wang Y, Wang Y, et al. Clinical study on electroacupuncture for obese patients with binge eating disorder: A retrospective study. J Tradit Chin Med. 2020;40(6):889-894. doi:10.19852/j.cnki.jtcm.2020.06.014.	Not related to Oral CHM
0832	CAST and IST Collaborative Groups. Indications for early aspirin use in acute ischemic stroke: a combined analysis of 40,000 randomized patients from the Chinese Acute Stroke Trial and the International Stroke Trial. Stroke. 2000;31(6):1240-1249. doi:10.1161/01.str.31.6.1240.	Not related to Oral CHM

0833	Zhang R, Wu Y, Zhao M, et al. Impact of ACE2 gene polymorphism on antihypertensive efficacy of ACE inhibitors. J Renin Angiotensin Aldosterone Syst. 2015;16(4):1064-1068. doi:10.1177/1470320314563420.	Not related to Oral CHM
0834	Li J, Wang Y, Zhang L, et al. Efficacy and safety of coenzyme A versus fenofibrate in patients with hyperlipidemia: a multicenter, double-blind, double-mimic, randomized clinical trial. Lipids Health Dis. 2020;19(1):59. doi:10.1186/s12944-020-01201-3.	Not related to Oral CHM
0835	Chen, Y. B., Chen, R. N., & Li, Y. L. (2012). Observation on therapeutic effect of type II early diabetic nephropathies intervened by acupoint thread embedding. Zhongguo Zhen jiu= Chinese Acupuncture & Moxibustion, 32(5), 390-394.	Not related to Oral CHM
0836	Chen Y, Liu Y, Zhang Y, et al. Efficiency of atorvastatin on in-hospital mortality of patients with acute aortic dissection (AAD): study protocol for a randomized, open-label, superiority clinical trial. Trials. 2021;22(1):274. doi:10.1186/s13063-021-05270-0.	Not related to Oral CHM
0837	Li X, Wang Y, Zhang Y, et al. Effect of acupuncture treatment on nonketotic hyperglycemic hemichorea-hemiballismus: a case report. J Integr Med. 2023;21(4):368-370. doi:10.1016/j.joim.2023.05.002.	Not related to Oral CHM
0838	Wang W, Yang J, Yang G, et al. Efficacy and safety of linagliptin monotherapy in Asian patients with inadequately controlled type 2 diabetes mellitus: a multinational, 24-week, randomized, clinical trial. J Diabetes. 2016;8(2):229-237. doi:10.1111/1753-0407.12284.	Not related to Oral CHM
0839	Xu B, Gao R, Wang J, et al. Comparison of 2 different drug-coated balloons in in-stent restenosis: the RESTORE ISR China randomized trial. JACC Cardiovasc Interv. 2018;11(23):2343-2353. doi:10.1016/j.jcin.2018.08.030.	Not related to Oral CHM
0840	Chen Y, Dong W, Wan Z, et al. Ticagrelor versus clopidogrel in Chinese patients with acute coronary syndrome: a pharmacodynamic analysis. Int J Cardiol. 2015;201:545-546. doi:10.1016/j.ijcard.2015.06.030.	Not related to Oral CHM
0841	Chen W, Yang GY, Liu B, Manheimer E, Liu JP. Manual acupuncture for treatment of diabetic peripheral neuropathy: a systematic review of randomized controlled trials. PLoS One. 2013;8(9):e73764. doi:10.1371/journal.pone.0073764.	Not related to Oral CHM
0842	Li X, Li Q, Zhang M, et al. Individualization of tacrolimus dosage basing on cytochrome P450 3A5 polymorphism—a prospective, randomized, controlled study. Clin Transplant. 2013;27(5):E272-E280. doi:10.1111/ctr.12127.	Not related to Oral CHM
0843	Chen, S. L., Li, L. L., & Li, H. L. (2006). Effect of massage in traditional Chinese medicine on short penis in male obese children.	Not related to Oral CHM
0844	Chen M, Zhang R, Jiang F, et al. A variant of PSMD6 is associated with the therapeutic efficacy of oral antidiabetic drugs in Chinese type 2 diabetes patients. Sci Rep. 2015;5:10701. doi:10.1038/srep10701.	Not related to Oral CHM

0845	Liu Y, Wang J, Zhang Y, et al. New smokeless moxibustion for knee osteoarthritis: a study protocol for a multicenter, single-blind, randomized controlled trial. Medicine (Baltimore). 2022;101(41):e30647. doi:10.1097/MD.0000000000030647.	Not related to Oral CHM
0846	Zhang Y, Wang Y, Wang Y, et al. Tuina combined with diet and exercise for simple obesity: a protocol for systematic review. Medicine (Baltimore). 2022;101(6):e28785. doi:10.1097/MD.0000000000028785.	Not related to Oral CHM
0847	Chen K, Yeung R. A case study of simultaneous recovery from multiple physical symptoms with medical qigong therapy. J Altern Complement Med. 2002;8(5):711-717. doi:10.1089/10755530260511814.	Not related to Oral CHM
0848	Wang Y, Wang JG, He G, et al. Common variants in TGFBR2 and miR-518 genes are associated with hypertension in the Chinese population. Am J Hypertens. 2014;27(11):1268-1276. doi:10.1093/ajh/hpu050.	Not related to Oral CHM
0849	Zhang Y, Wang Y, Wang Y, et al. Serum metabolomics model and its metabolic characteristics in patients with different syndromes of dyslipidemia based on nuclear magnetic resonance. J Tradit Chin Med. 2019;39(1):34-40. doi:10.19852/j.cnki.jtcm.2019.01.007.	Not related to Oral CHM
0850	Chen J, Gu Y, Yin L, et al. Network meta-analysis of curative efficacy of different acupuncture methods on obesity combined with insulin resistance. Front Endocrinol (Lausanne). 2022;13:968481. doi:10.3389/fendo.2022.968481.	Not related to Oral CHM
0851	Zhang Y, Wang Y, Wang Y, et al. Preliminary study on effects of sodium ferulate in treating diabetic nephropathy. J Tradit Chin Med. 2006;26(4):270-273.	Not related to Oral CHM
0852	Li Y, Wang Y, Wang Y, et al. Efficiency and safety of renal denervation via cryoablation (Cryo-RDN) in Chinese patients with uncontrolled hypertension: study protocol for a randomized controlled trial. Trials. 2019;20(1):712. doi:10.1186/s13063-019-3786-4.	Not related to Oral CHM
0853	Zhang Y, Wang Y, Wang Y, et al. Effect of acupoint catgut embedding on TNF-alpha and insulin resistance in simple obesity patients. J Tradit Chin Med. 2007;27(4):265-267.	Not related to Oral CHM
0854	Venketasubramanian N, Chan BP, Gan RN, et al. Association between baseline NIHSS limb motor score and functional recovery after stroke: analysis based on a multicountry dataset. Cerebrovasc Dis. 2022;51(3):242-248. doi:10.1159/000525306.	Not related to Oral CHM
0855	Anderson CS, Arima H, Lavados P, et al. Regional differences in early blood pressure management after acute ischemic stroke in the ENCHANTED international randomized controlled trials. Front Neurol. 2021;12:687862. doi:10.3389/fneur.2021.687862.	Not related to Oral CHM
0856	Venketasubramanian N, Chan ESY, Slavin MJ, et al. The Alzheimer's disease THErapy with NEuroaid (ATHENE) study: a randomized, double-blind, placebo-controlled trial to assess the safety and efficacy of MLC901 in patients with mild to moderate Alzheimer's disease. Alzheimers Dement (N Y). 2019;5:38-45. doi:10.1016/j.trci.2018.12.002.	Not related to Oral CHM

0857	Chegni H, Babaii H, Hassan ZM, Pourshaban M. Immune response and cytokine storm in SARS-CoV-2 infection: risk factors, ways of control and treatment. Eur J Inflamm. 2022;20:1-10. doi:10.1177/1721727X221098970.	Not related to Oral CHM
0858	Charles M, Simmons RK, Williams KM, et al. Cardiovascular risk reduction following diagnosis of diabetes by screening: 1-year results from the ADDITION-Cambridge trial cohort. Br J Gen Pract. 2012;62(599):e396-e403. doi:10.3399/bjgp12X641456.	Not related to Oral CHM
0859	Chao MT, Nguyen U, Zibrat Z, Reed F, Schillinger D, Hecht FM. Protocol development of group acupuncture treatment for painful diabetic neuropathy using treatment manualization. J Altern Complement Med. 2014;20(5):A70. doi:10.1089/acm.2014.5105.abstract.	Not related to Oral CHM
0860	Cushman WC, Evans GW, Cutler JA, et al. Patterns and correlates of baseline thiazide-type diuretic prescription in the Systolic Blood Pressure Intervention Trial. Hypertension. 2016;67(3):550-555. doi:10.1161/HYPERTENSIONAHA.115.06851.	Not related to Oral CHM
0861	Chan, K. W., & Tang, S. C. W. (2020). Standardization, Personalization and Real-world Practice in Pragmatic Trials for Integrative Medicine: The Methodological Design of a Semi-individualized Randomized Controlled Trial for Diabetic Kidney Disease. In International Congress on Integrative Medicine and Health. Sage Publications Ltd. The Journal's web site is located at https://journals. sagepub. com/home/gam/loi/gahmj.	Not related to Oral CHM
0862	Chakraborty R, Borah P, Dutta PP, Sen S. Evolving spectrum of diabetic wound: mechanistic insights and therapeutic targets. World J Diabetes. 2022;13(9):696-716. doi:10.4239/wjd.v13.i9.696.	Not related to Oral CHM
0863	Zhang Y, Li H, Zhang J, et al. Effect of elevated fasting blood glucose level on the 1-year mortality and sequelae in hospitalized COVID-19 patients: a bidirectional cohort study. J Med Virol. 2022;94(5):1827-1835. doi:10.1002/jmv.27737.	Not related to Oral CHM
0864	Oh Y, Choi JW, Yang GY, Chae H. Treatment of lumbosacral radiculopathy with acupuncture and medical herbs: four case reports. J Acupunct Meridian Stud. 2022;15(4):264-270. doi:10.51507/j.jams.2022.15.4.264.	Not related to Oral CHM
0865	Belcaro G, Cesarone MR, Dugall M, et al. Improvement of diabetic microangiopathy with pycnogenol: a prospective, controlled study. Angiology. 2006;57(4):431-436. doi:10.1177/0003319706290318.	Not related to Oral CHM
0866	Cawich, S. O., Harnarayan, P., Islam, S., Budhooram, S., Ramsewak, S., Ramdass, M. J., & Naraynsingh, V. (2014). Topical "soft candle" applications for infected diabetic foot wounds: a cause for concern?. International Journal of Biomedical Science: IJBS, 10(2), 111.	Not related to Oral CHM
0867	Carter BL, Coffey CS, Ardery G, et al. Cluster-randomized trial to evaluate a centralized clinical pharmacy service in private family medicine offices. Circ Cardiovasc Qual Outcomes. 2018;11(6):e004188. doi:10.1161/CIRCOUTCOMES.117.004188.	Not related to Oral CHM

086 8	Feingold KL, Moskowitz JT, Elenbaas C, et al. Acupuncture after valve surgery is feasible and shows promise in reducing postoperative atrial fibrillation: the ACU-Heart pilot trial. JTCVS Open. 2023;16:321-332. doi:10.1016/j.xjon.2023.05.010.	Not related to Oral CHM
086 9	Carreyre H, Carré G, Ouedraogo M, et al. Bioactive natural product and superacid chemistry for lead compound identification: a case study of selective hCA III and L-type Ca2+ current inhibitors for hypotensive agent discovery. Molecules. 2017;22(6):915. doi:10.3390/molecules22060915.	Not related to Oral CHM
087 0	Carey N, Courtenay M, James J, Hills M, Roland J. An evaluation of a Diabetes Specialist Nurse prescriber on the system of delivering medicines to patients with diabetes. J Clin Nurs. 2008;17(12):1635-1644. doi:10.1111/j.1365-2702.2007.02197.x.	Not related to Oral CHM
087 1	Cao, K. G., Fu, C. H., Li, H. Q., Xin, X. Y., & Gao, Y. (2015). A new prognostic scale for the early prediction of ischemic stroke recovery mainly based on traditional Chinese medicine symptoms and NIHSS score: a retrospective cohort study. BMC complementary and alternative medicine, 15, 1-11.	Not related to Oral CHM
087 2	Mancia G, Parati G, Pomidossi G, et al. The effects of nitrendipine on the quality of life in elderly patients with isolated systolic hypertension. J Hypertens Suppl. 1992;10(3):S89-S92.	Not related to Oral CHM
087 3	Zhang Y, Wang Y, Wang Y, et al. Comparing antihypertensive effect and plasma ciclosporin concentration between amlodipine and valsartan regimens in hypertensive renal transplant patients receiving ciclosporin therapy. Int J Cardiol. 2012;155(3):362-367. doi:10.1016/j.ijcard.2011.07.005.	Not related to Oral CHM
087 4	Cai C. Acupuncture and moxibustion treatment of amenorrhea. Med Acupunct. 2013;25(3):206-209. doi:10.1089/acu.2012.0914.	Not related to Oral CHM
087 5	Otte JL, Carpenter JS, Manchanda S, Rand KL, Skaar TC, Weaver M. Systematic review of sleep disorders in cancer patients. Support Care Cancer. 2015;23(5):1285-1300. doi:10.1007/s00520-014-2475-3.	Not related to Oral CHM
087 6	Burg D, Yamamoto M, Namekata M, et al. Promotion of anagen, increased hair density and reduction of hair fall in a clinical setting following identification of FGF5-inhibiting compounds via a novel 2-stage process. Clin Cosmet Investig Dermatol. 2017;10:373-385. doi:10.2147/CCID.S133132.	Not related to Oral CHM
087 7	Mühlhauser I, Berger M, Müller UA, et al. Informed shared decision making programme for patients with type 2 diabetes in primary care: cluster randomised controlled trial. BMJ. 2006;333(7574):766. doi:10.1136/bmj.38983.706299.55.	Not related to Oral CHM
087 8	Cardini F, Weixin H. Moxibustion for correction of breech presentation: a randomized controlled trial. JAMA. 1998;280(18):1580-1584. doi:10.1001/jama.280.18.1580.	Not related to Oral CHM

0879	Bu, T. W., Tian, X. L., Wang, S. J., Liu, W., Li, X. L., & Tan, Y. H. (2007). Comparison and analysis of therapeutic effects of different therapies on simple obesity. Zhongguo zhen jiu= Chinese acupuncture & moxibustion, 27(5), 337-340.	Not related to Oral CHM
0880	Broncel M, Kozirog M, Duchnowicz P, et al. Aronia melanocarpa extract reduces blood pressure, serum endothelin, lipid, and oxidative stress marker levels in patients with metabolic syndrome. Med Sci Monit. 2010;16(1):CR28-CR34.	Not related to Oral CHM
0881	Flachskampf FA, Gallasch J, Gefeller O, et al. Randomized trial of acupuncture to lower blood pressure. Circulation. 2007;115(24):3121-3129. doi:10.1161/CIRCULATIONAHA.106.661140.	Not related to Oral CHM
0882	Chen B, Zhang XS, Huang H, et al. A study on differences of curative effects of acupuncture and nCPAP for treatment of OSAHS. Zhongguo Zhen Jiu. 2008;28(3):165-169.	Not related to Oral CHM
0883	Bieszk, N., Grabner, M., Wei, W., Barron, J., Quimbo, R., Yan, T., ... & Chu, J. W. (2017). Personalized care and the role of insulin as a vehicle to optimizing treatments in diabetes care. Journal of managed care & specialty pharmacy, 23(11), 1160-1168.	Not related to Oral CHM
0884	Bian, Y., Zhou, S., Hou, H., Xu, T., & Huang, Y. (2021). The optimal dose of oral midazolam with or without intranasal S-ketamine for premedication in children: a randomised, double blinded, sequential dose-finding trial. Translational Pediatrics, 10(11), 2941.	Not related to Oral CHM
0885	Berkowitz, R. I., Wadden, T. A., Gehrman, C. A., Bishop-Gilyard, C. T., Moore, R. H., Womble, L. G., ... & Xanthopoulos, M. S. (2011). Meal replacements in the treatment of adolescent obesity: a randomized controlled trial. Obesity, 19(6), 1193-1199.	Not related to Oral CHM
0886	Bergmeijer, T. O., Janssen, P. W., van Oevelen, M., van Rooijen, D., Godschalk, T. C., Kelder, J. C., ... & Ten Berg, J. M. (2017). Incidence and causes for early ticagrelor discontinuation: a "real-world" Dutch registry experience. Cardiology, 138(3), 164-168.	Not related to Oral CHM
0887	Stein EA, Mellman MF, Davidson MH, et al. Evidence of a drug-drug interaction linked to inhibition of ester hydrolysis by orlistat. Clin Pharmacol Ther. 2012;91(4):634-641. doi:10.1038/clpt.2011.306.	Not related to Oral CHM
0888	Salas-Huetos A, Bulló M, Salas-Salvadó J. The impact of nutrition and lifestyle on male fertility. Asian J Androl. 2020;22(2):121-130. doi:10.4103/aja.aja_83_19.	Not related to Oral CHM
0889	Gauthier SF, Lapointe G, Roy D, et al. Chitin-glucan fiber effects on oxidized low-density lipoprotein: A randomized controlled trial. Nutr J. 2012;11:79. doi:10.1186/1475-2891-11-79.	Not related to Oral CHM
0890	Battin MR, Obolonkin V, Rush EC, et al. Blood pressure measurement at two years in offspring of women randomized to a trial of metformin for GDM: follow up data from the MiG trial. BMC Pediatr. 2015;15:54. doi:10.1186/s12887-015-0375-2.	Not related to Oral CHM

0891	Banach M, Burchardt P, Chlebus K, et al. PoLA/CFPiP/PCS/PSLD/PSD/PSH guidelines on diagnosis and therapy of lipid disorders in Poland 2021. Arch Med Sci. 2021;17(6):1447-1547. doi:10.5114/aoms/141941.	Not related to Oral CHM
0892	Wang S, Moustaid-Moussa N, Chen L, et al. Effects of polyphenol-rich fruit extracts on diet-induced obesity in rodents: Systematic review and meta-analysis. Evid Based Complement Alternat Med. 2019;2019:2940364. doi:10.1155/2019/2940364.	Not related to Oral CHM
0893	Bakker GC, van Erk MJ, Pellis L, et al. An antiinflammatory dietary mix modulates inflammation and oxidative and metabolic stress in overweight men: A nutrigenomics approach. Am J Clin Nutr. 2010;91(4):1044-1059. doi:10.3945/ajcn.2009.28549.	Not related to Oral CHM
0894	Bai YP, Fu JY. Clinical observation on the regularity of acupuncture-induced body-reduction in excess-heat-type obesity patients. Zhongguo Zhen Jiu. 2007;27(2):115-118.	Not related to Oral CHM
0895	Zhang Y, Li X, Wang Y, et al. Safety and efficacy evaluation of antithrombotic therapy with rivaroxaban and clopidogrel after PCI in Chinese patients. Clin Appl Thromb Hemost. 2022;28:10760296221074681. doi:10.1177/10760296221074681.	Not related to Oral CHM
0896	Li Y, Wang J, Zhang Y, et al. A meta-analysis of the association between diabetes mellitus and traditional Chinese medicine constitution. Evid Based Complement Alternat Med. 2021;2021:6676893. doi:10.1155/2021/6676893.	Not related to Oral CHM
0897	Axelsson AS, Tubbs E, Mecham B, et al. Sulforaphane reduces hepatic glucose production and improves glucose control in patients with type 2 diabetes. Sci Transl Med. 2017;9(394):eaah4477. doi:10.1126/scitranslmed.aah4477.	Not related to Oral CHM
0898	Aurora RN, Zak RS, Maganti RK, et al. Best practice guide for the treatment of REM sleep behavior disorder (RBD). J Clin Sleep Med. 2010;6(1):85-95. doi:10.5664/jcsm.27706.	Not related to Oral CHM
0899	de Oliveira ML, de Souza AM, da Silva LS, et al. A multicomponent family intervention, combined with salt reduction for children with obesity: a factorial randomized study protocol. BMC Public Health. 2023;23(1):16356. doi:10.1186/s12889-023-16356-6.	Not related to Oral CHM
0900	Rashwan A, Wang Y, Li X, et al. A phase II, randomized, double-blind, double-dummy, active-controlled clinical trial to investigate the efficacy and safety of NW Low-Glu® in patients newly diagnosed with type 2 diabetes mellitus. J Diabetes Res. 2022;2022:9176026. doi:10.1155/2022/9176026.	Not related to Oral CHM
0901	Asih, E. S., Handayani, R., Rahmawati, S. E., & Azis, Z. M. R. (2021). RED FRUIT (PANDANUS CONOIDEUS LAM) AS AN IMMUNOMODULATOR: A SCOOPING REVIEW. Biochemical & Cellular Archives, 21(2).	Not related to Oral CHM
0902	Asadi MH, Changizi-Ashtiyani S, Latifi SAH. Evaluation of the infantile colic causes in Persian medicine. Iran J Neonatol. 2021;12(1):33-39. doi:10.22038/ijn.2020.47287.1810.	Not related to Oral CHM

0903	Arena C, Bizzoca ME, Caponio VCA, et al. Everolimus therapy and side-effects: A systematic review and meta-analysis. Int J Oncol. 2021;59(1):1-10. doi:10.3892/ijo.2021.5234.	Not related to Oral CHM
0904	González-Sarrías A, García-Villalba R, Núñez-Sánchez MA, et al. Effects of a fruit and vegetable-based nutraceutical on biomarkers of inflammation and oxidative status in the plasma of a healthy population: A placebo-controlled, double-blind, and randomized clinical trial. Molecules. 2021;26(12):3604. doi:10.3390/molecules26123604.	Not related to Oral CHM
0905	Aragona M, Onesti E, Tomassini V, et al. Psychopathological and cognitive effects of therapeutic cannabinoids in multiple sclerosis: A double-blind, placebo controlled, crossover study. Clin Neuropharmacol. 2009;32(1):41-47. doi:10.1097/WNF.0b013e318193e5eb.	Not related to Oral CHM
0906	Philis-Tsimikas A, Walker C, Rivard L, et al. Dulce Wireless Tijuana: a randomized control trial evaluating the impact of Project Dulce and short-term mobile technology on glycemic control in a family medicine clinic in northern Mexico. Diabetes Technol Ther. 2016;18(4):240-246. doi:10.1089/dia.2015.0283.	Not related to Oral CHM
0907	Antony B, Merina B, Iyer VS, et al. Amlamax™ in the management of dyslipidemia in humans. Indian J Pharm Sci. 2008;70(4):504-507. doi:10.4103/0250-474X.44611.	Not related to Oral CHM
0908	Lee MS, Shin BC, Choi TY, et al. Acupuncture for treating diabetic retinopathy: A systematic review and meta-analysis of randomized controlled trials. Complement Ther Med. 2020;52:102493. doi:10.1016/j.ctim.2020.102493.	Not related to Oral CHM
0909	Duangsrisai S, Gomes NGM, Andrade PB, et al. Medicinal plants utilized in Thai Traditional Medicine for diabetes treatment: Ethnobotanical surveys, scientific evidence and phytochemicals. J Ethnopharmacol. 2020;263:113177. doi:10.1016/j.jep.2020.113177.	Not related to Oral CHM
0910	Andersson A, Karlström B, Vessby B. Cholesterol-lowering effects of a stanol ester-containing low-fat margarine used in conjunction with a strict lipid-lowering diet. Eur J Clin Nutr. 1999;53(9):716-722. doi:10.1038/sj.ejcn.1600835.	Not related to Oral CHM
0911	Lee MS, Shin BC, Suen LK, et al. Acupressure for treating insomnia: a systematic review. Sleep Med Rev. 2010;14(6):433-442. doi:10.1016/j.smrv.2009.11.003.	Not related to Oral CHM
0912	Ahmed SM, Madbouly NH, Maklad SS, et al. Effects of wet-cupping on blood pressure in hypertensive patients: A randomized controlled trial. J Integr Med. 2015;13(6):391-395. doi:10.1016/S2095-4964(15)60197-2.	Not related to Oral CHM
0913	Al-Abdely HM, Najjar A, Al-Attas R, et al. Rhinocladiella mackenziei cerebral phaeohyphomycosis with a definitive outcome. BMJ Case Rep. 2023;16(5):e250787. doi:10.1136/bcr-2022-250787.	Not related to Oral CHM

0914	Chen H, Guo J, Wang Y, et al. Management of postprandial hyperglycaemia and weight gain in women with gestational diabetes mellitus using a novel telemonitoring system. J Int Med Res. 2018;46(11):4467-4477. doi:10.1177/0300060518809872.	Not related to Oral CHM
0915	Ahmed SM, Madbouly NH, Maklad SS, et al. Evaluation of wet cupping therapy: systematic review of randomized clinical trials. J Altern Complement Med. 2016;22(10):768-777. doi:10.1089/acm.2016.0193.	Not related to Oral CHM
0916	Alrasheid MHS, Alnour MA, Ahmed AE, et al. Searching grey literature for evidence: Ozone therapy as a model. J Altern Complement Med. 2014;20(5):A65. doi:10.1089/acm.2014.5183.abstract.	Not related to Oral CHM
0917	Abuaisha BB, Costanzi JB, Boulton AJM. Acupuncture for the treatment of chronic painful peripheral diabetic neuropathy: a long-term study. Diabet Med. 1998;15(6):469-473. doi:10.1002/(SICI)1096-9136(199806)15:6<469::AID-DIA613>3.0.CO;2-0.	Not related to Oral CHM
0918	Ahmad, S., Mahmood, T., Kumar, R., Bagga, P., Ahsan, F., Shamim, A., ... & Parveen, S. (2020). A contrastive phytopharmacological analysis of Gala and Fuji apple. Research Journal of Pharmacy and Technology, 13(3), 1527-1537.	Not related to Oral CHM
0919	Agthe AG, Kim GR, Mathias KB, et al. Clonidine as an adjunct therapy to opioids for neonatal abstinence syndrome: a randomized, controlled trial. Pediatrics. 2009;123(5):e849-e856. doi:10.1542/peds.2008-1737.	Not related to Oral CHM
0920	Esmaeilinezhad Z, Akhlaghi M, Shidfar F, et al. Effects of Komouni formulation with a low-calorie diet on cardiometabolic risk factors in overweight and obese women: a triple-blinded randomized clinical trial. BMC Complement Med Ther. 2021;21(1):231. doi:10.1186/s12906-021-03444-1.	Not related to Oral CHM
0921	Dennis JM, Shields BM, Henley WE, et al. Predicting post one-year durability of glucose-lowering monotherapies in patients with newly diagnosed type 2 diabetes mellitus: A MASTERMIND precision medicine approach (UKPDS 87). Diabetes Care. 2021;44(1):191-198. doi:10.2337/dc20-1883.	Not related to Oral CHM
0922	Abuaisha BB, Costanzi JB, Boulton AJM. Acupuncture for the treatment of chronic painful peripheral diabetic neuropathy: a long-term study. Diabet Med. 1998;15(6):469-473. doi:10.1002/(SICI)1096-9136(199806)15:6<469::AID-DIA613>3.0.CO;2-0.	Not related to Oral CHM
0923	Abidov, M. T., Del Rio, M. J., Ramazanov, T. Z., Klimenov, A. L., Dzhamirze, S., & Kalyuzhin, O. V. (2006). Effects of Aralia mandshurica and Engelhardtia chrysolepis extracts on some parameters of lipid metabolism in women with nondiabetic obesity. Bulletin of experimental biology and medicine, 141, 343-346.	Not related to Oral CHM
0924	庄海舟,王志飞,谢雁鸣,等.基于医院信息系统数据库的急性胰腺炎患者临床特征分析[J].中医杂志 ,2014,55(19):1688-1691.DOI:10.13288/j.11-2166/r.2014.19.018	Not related to Oral CHM

092<br>5	朱林奇,连超群,鲍凌.乳酸环丙沙星致低血糖 1 例[J].人民军医<br>,2015,58(07):821.	Not related to<br>Oral CHM
092<br>6	周羽,祝萃.初诊消渴病临床特点研究[J].光明中医,2016,31(16):2312-2314.	Not related to<br>Oral CHM
092<br>7	莫菁莲.普罗帕酮治疗不同中医辨证分型的心房颤动临床疗效和安全性研究[J].时珍国医国药,2013,24(07):1702-1703.	Not related to<br>Oral CHM
092<br>8	孟庆扬,马建伟,董静,等.基于因子与聚类分析的 2 型糖尿病合并血脂异常中医证素特点及辨证分型研究[J].解放军医药杂志,2015,27(09):30-33.	Not related to<br>Oral CHM
092<br>9	梅丽俊,曾玲,付勇,等.热敏灸治疗痰浊阻遏型原发性高血脂 20 例[J].江西中医药,2013,44(05):41-43.	Not related to<br>Oral CHM
093<br>0	满斌. (2013). 醒脑开窍针刺法治疗中风验案二例. 中华针灸电子杂志, (3), 34-36.	Not related to<br>Oral CHM
093<br>1	麦合权,甘雅薇,曹燕娟.中医体质辨识融入社区 2 型糖尿病健康管理中的干预效果评价[J].四川中医,2016,34(06):82-84.	Not related to<br>Oral CHM
093<br>2	马永,李新元,李富强,等.穴位埋线治疗男性慢性盆腔疼痛综合征临床研究[J].中国针灸,2015,35(06):561-566.DOI:10.13703/j.0255-2930.2015.06.010.	Not related to<br>Oral CHM
093<br>3	罗伟,马建伟.原发性血脂异常患者中医体质类型与中医证型相关性研究[J].解放军医药杂志,2015,27(09):17-20.	Not related to<br>Oral CHM
093<br>4	罗涛.黛力新口服致肝功能损害 3 例[J].临床合理用药杂志<br>,2013,6(05):25.DOI:10.15887/j.cnki.13-1389/r.2013.05.106.	Not related to<br>Oral CHM
093<br>5	罗辉,王琦.中医体质类型与代谢综合征相关性研究的系统评价和Meta分析[J].北京中医药大学学报,2016,39(04):325-334.	Not related to<br>Oral CHM
093<br>6	罗发生,李功辉,李奇林,等.辨证分型穴位敷贴治疗原发性高血压的临床研究[J].中国医药导报,2011,8(21):108-109+112.	Not related to<br>Oral CHM
093<br>7	罗川晋,张璐,陈悦轩,等.502 例岭南地区扩张型心肌病病人中医临床特征探析[J].中西医结合心脑血管病杂志,2020,18(10):1509-1513.	Not related to<br>Oral CHM
093<br>8	吕娟,刘玲,张婷,等.高同型半胱氨酸血症与原发性高血压中医证型关联分析[J].社区医学杂志<br>,2020,18(23):1604-1607.DOI:10.19790/j.cnki.JCM.2020.23.10.	Not related to<br>Oral CHM
093<br>9	刘志诚,孙凤岷,徐炳国,等.针灸治疗肥胖症并发原发性高血压的临床研究(英文)[J].中国临床康复,2004,(12):2398-2400.	Not related to<br>Oral CHM
094<br>0	刘振岳.中医辨证分型对高血压前期患者血压进展的影响[J].中国处方药<br>,2017,15(06):98-100.	Not related to<br>Oral CHM
094<br>1	刘瑶,李伟.高血压肾损害中医证候要素特征研究[J].山东中医杂志<br>,2022,41(02):157-160.DOI:10.16295/j.cnki.0257-358x.2022.02.005.	Not related to<br>Oral CHM
094<br>2	刘阳,王月秋,单鹏.烧伤创疡再生医疗技术治疗糖尿病足的疗效观察[J].中国烧伤创疡杂志,2019,31(03):161-165.	Not related to<br>Oral CHM
094<br>3	刘彦汶,张珂炜,吉红玉,等.基于掌型经络检测仪探讨 2 型糖尿病体质与十二经络的相关性[J].河南中医<br>,2020,40(01):120-122.DOI:10.16367/j.issn.1003-5028.2020.01.0030.	Not related to<br>Oral CHM
094<br>4	刘文导,樊树鹏,孟凡喆,等.介入治疗糖尿病足术后再狭窄与中医体质相关性研究[J].新中医<br>,2012,44(02):47-48.DOI:10.13457/j.cnki.jncm.2012.02.001.	Not related to<br>Oral CHM

0945	刘培宏.刍议中医外科特色技术治疗糖尿病足临床效果[J].糖尿病新世界,2015,(06):72.DOI:10.16658/j.cnki.1672-4062.2015.06.146.	Not related to Oral CHM
0946	刘敏,腹针治疗中心型肥胖(脾虚痰湿证) 伴胰岛素抵抗的临床疗效观察. 吉林省,长春市中医院,2020-04-16.	Not related to Oral CHM
0947	刘美琴.少阳气郁体质与 2 型糖尿病中医证候及并发症的相关性[J].光明中医,2020,35(14):2123-2125.	Not related to Oral CHM
0948	苏娣.老年高血压中医证型分布特点与心血管危险因素相关性分析[J].内蒙古中医药,2017,36(04):23-24.DOI:10.16040/j.cnki.cn15-1101.2017.04.023.	Not related to Oral CHM
0949	刘芬,何毅芳,谢凡慈,等.高血压病患者中医体质特点与心血管疾病危险因素的关联分析[J].医学理论与实践,2021,34(02):222-224.DOI:10.19381/j.issn.1001-7585.2021.02.018.	Not related to Oral CHM
0950	林育,项磊,肖雪,等.基于临床研究的湿热证文本信息挖掘[J].广东药科大学学报,2017,33(05):654-658.DOI:10.16809/j.cnki.2096-3653.2017090702.	Not related to Oral CHM
0951	林晓敏, 洪玉婷, 吴少彬, 张翠哗, 眭文妍, 谢乙团,2020. 肥胖相关性肾病中医证型分布规律初探, 世界最新医学信息文摘.	Not related to Oral CHM
0952	林孔万,朱若晨,李弼仁.基于中医体质类型的冠心病合并急性心衰患者发生心源性死亡情况及其影响因素分析[J].中国中医急症,2021,30(09):1558-1561+1578.	Not related to Oral CHM
0953	林谦,陈焱木,金法,等.371 例原发性高血压患者的中医体质辨证研究[J].现代中医药,2004,(03):17-18.	Not related to Oral CHM
0954	廖建琼."减肥八穴"穴位埋线治疗单纯性肥胖症的疗效规律观察[D].南方医科大学,2014.	Not related to Oral CHM
0955	梁雪,王琦,沈昆,等.痰湿体质人群易发代谢综合征相关危险因素的调查研究[J].中华中医药杂志,2017,32(04):1500-1503.	Not related to Oral CHM
0956	李寅,艾静,汤峥冬,等.原发性高血压病患者中医体质分布规律初探[J].临床误诊误治,2017,30(07):95-98.	Not related to Oral CHM
0957	张静,陆霞,李艳,等.水穴埋线配合隔药饼灸治疗脾虚湿阻型肥胖并发高脂血症随机对照研究[J].四川中医,2017,35(05):189-192.	Not related to Oral CHM
0958	李岩,胡继强,林谦,等.51 例房颤射频消融患者证候分析[J].中国中医基础医学杂志,2016,22(10):1363-1365.DOI:10.19945/j.cnki.issn.1006-3250.2016.10.028.	Not related to Oral CHM
0959	李雪梅.韩禅虚主任调中理气针法临床应用举隅[J].中医临床研究,2016,8(05):19-20.	Not related to Oral CHM
0960	李晓政,闫彩香.代谢综合征及 2 型糖尿病慢性肾损害湿热血瘀证的相关研究[J].中国中医药科技,2007,(06):385-386.	Not related to Oral CHM
0961	李先涛,于春泉,王泓午.中风病先兆证临床特征表现文献系统评价[J].天津中医药,2013,30(02):115-118.	Not related to Oral CHM
0962	李文东,孙书亭,吕树泉,等.2 型糖尿病体质辨识演变规律系统综述[J].实用中医内科杂志,2016,30(12):1-3.DOI:10.13729/j.issn.1671-7813.2016.12.01.	Not related to Oral CHM
0963	李润, 李柳娜, 王梦琪, 牛晶晶, 李瑞丽, 陈园园, & 江佳美. (2023). 子午流注理论下的刮痧干预联合情志护理对痰湿壅盛证原发性高血压患者的影响. 国际医药卫生导报, 29(3), 404.	Not related to Oral CHM

0964	李玲孺,姚海强,王济,等.基于文献的 2 型糖尿病高度相关体质类型分析及调体防控效果评价[J].中国科学:生命科学,2016,46(08):990-1000.	Not related to Oral CHM
0965	李玲丽,纪惠谦,梁敏.复方樟柳碱对增生期糖尿病性视网膜病变手术及激光治疗后视神经缺血的疗效观察[J].临床眼科杂志,2011,19(01):52-54.	Not related to Oral CHM
0966	李静娴,陆旭亚,沈翠珍.中医食疗对痰湿质高脂血症患者作用的研究[J].中华中医药学刊,2014,32(06):1371-1373.DOI:10.13193/j.issn.1673-7717.2014.06.038.	Not related to Oral CHM
0967	李靖,刘浩,杨立宏,等.糖尿病中医证候与分子生物学理论探讨[J].陕西中医,2014,35(07):859-860.	Not related to Oral CHM
0968	李佳琦.2 型糖尿病肾脏疾病危险因素的Meta分析及与中医体质的相关性研究[D].天津中医药大学,2020.DOI:10.27368/d.cnki.gtzyy.2020.000266.	Not related to Oral CHM
0969	李惠林,李增英,张志玲,等.脂联素、血脂、SOD与代谢综合征痰湿内蕴辨证关系研究[J].世界中医药,2011,6(01):73-75.	Not related to Oral CHM
0970	李欢,申冬冬,魏征,等.隔姜灸联合瑞舒伐他汀治疗脾虚湿阻型肥胖并发高脂血症的临床观察[J].世界科学技术-中医药现代化,2021,23(05):1540-1545.	Not related to Oral CHM
0971	李红,谭年花,陈斌,等.中医体质类型与 2 型糖尿病相关性的系统评价[J].中医药导报,2021,27(11):211-216.DOI:10.13862/j.cnki.cn43-1446/r.2021.11.050.	Not related to Oral CHM
0972	李鹤,张锐,杨清馨,等.中医体质类型与高血压相关性研究的Meta分析[J].济宁医学院学报,2020,43(01):54-58.	Not related to Oral CHM
0973	李海燕,陈淑珍.社区老年高血压人群中医体质特征探讨[J].中国社区医师,2021,37(33):76-77.	Not related to Oral CHM
0974	谭倩.1565 例 2 型糖尿病患者中医病证分布规律研究[D].北京中医药大学,2010.	Not related to Oral CHM
0975	李冬霞,胡琪,陈光耀,等.强直性脊柱炎伴高尿酸血症的临床特征分析[J].世界中西医结合杂志,2019,14(11):1485-1488.DOI:10.13935/j.cnki.sjzx.191102.	Not related to Oral CHM
0976	李大锋,蓝小琴,赵金龙,等.原发性高血压血压变异性与左室肥厚及中医证型相关性研究[J].现代中西医结合杂志,2016,25(01):34-36.	Not related to Oral CHM
0977	赖惠东.我院 2015 年 1-12 月心内科住院患者应用华法林情况分析[J].中国合理用药探索,2018,15(04):67-70.	Not related to Oral CHM
0978	匡丽晖,方穗雄,黄雄飞,等.糖尿病性视网膜病变中医证候与眼底荧光造影表现的初步研究[J].中国中医眼科杂志,2009,19(03):142-145.	Not related to Oral CHM
0979	寇明星,罗玲.2 型糖尿病并发肺部感染 55 例中医辨证分析[J].中国中医急症,2009,18(10):1637-1638.	Not related to Oral CHM
0980	陈飞,梁永辉,陈惠茹,等.167 例阻塞性睡眠呼吸暂停低通气综合征患者中医证候分布规律研究[J].福建中医药,2023,54(06):9-12.DOI:10.13260/j.cnki.jfjtcm.2023.06003.	Not related to Oral CHM
0981	蒋春梅,李娟.痛风危险因素、体质类型与中医证型的相关研究[J].热带医学杂志,2011,11(05):590-592+595.	Not related to Oral CHM
0982	贾冕,赵进喜,皇甫伟.基于中医证候学的糖尿病肾病患者肾小球滤过率评估方法的探索[J].环球中医药,2016,9(03):275-282.	Not related to Oral CHM

0983	霍金,韩数,马力颖,等.腹部电针结合灸法治疗虚证中心性肥胖 30 例[J].中国中医药科技,2012,19(02):116.	Not related to Oral CHM
0984	霍达,任明,翟静波,等.糖尿病前期证型分类的文献研究及系统评价[J].辽宁中医杂志,2015,42(01):1-4.DOI:10.13192/j.issn.1000-1719.2015.01.001.	Not related to Oral CHM
0985	王伟强,罗维祥.社区老年高血压患者中医体质分析[J].福建中医药,2017,48(03):61-62.DOI:10.13260/j.cnki.jfjtcm.011373.	Not related to Oral CHM
0986	黄伟旋,陈兆鑫,廖圣榕,等.新型中医禁食疗法治疗单纯性肥胖患者 79 例回顾性分析[J].中医临床研究,2018,10(16):55-57.	Not related to Oral CHM
0987	黄明江,黄宗菊,陈宗华,等.交替电针腹八针腹十针为主配合耳穴贴压治疗单纯性肥胖临床研究[J].实用中医药杂志,2016,32(03):242-243.	Not related to Oral CHM
0988	黄静怡,龚雯静,张慧琰,等.穴位埋线改善代谢综合征痰湿体质 96 例疗效观察[J].医药前沿,2021,11(01):162-163.	Not related to Oral CHM
0989	胡锦锦.糖尿病肾病腹膜透析患者糖暴露及腹膜转运功能情况与中医证型分布的临床研究[D].湖北中医药大学,2020.DOI:10.27134/d.cnki.ghbzc.2020.000331.	Not related to Oral CHM
0990	洪肖娟.辨证归经取穴治疗 1 级原发性高血压的随机对照试验研究[D].成都中医药大学,2017.	Not related to Oral CHM
0991	何佳,姚淮芳,程晓昱,等.探讨同型半胱氨酸、非高密度脂蛋白胆固醇与高血压患者中医证型关系的研究[J].中医药学报,2018,46(05):111-114.DOI:10.19664/j.cnki.1002-2392.180161.	Not related to Oral CHM
0992	韩燕萍,李翊,杨光,等.电针治疗脾虚湿阻型女性肥胖患者临床观察[J].针灸临床杂志,2015,31(06):25-27.	Not related to Oral CHM
0993	韩燕萍.电针治疗脾虚湿阻型女性肥胖患者的单盲随机对照试验[D].黑龙江中医药大学,2016.	Not related to Oral CHM
0994	韩秀华,屈蓓蓓,赵兰,等.小剂量甲氨蝶呤导致老年类风湿性关节炎患者严重骨髓抑制三例[J].临床内科杂志,2016,33(11):783-784.	Not related to Oral CHM
0995	韩倩倩,张宛月,石玥,等.2 型糖尿病合并阻塞型睡眠呼吸暂停低通气综合征的中医体质辨识[J].世界中西医结合杂志,2021,16(07):1295-1299.DOI:10.13935/j.cnki.sjzx.210724.	Not related to Oral CHM
0996	韩东彦,刘宇,占永立,等.特发性膜性肾病中医证候特点及其在预后判断中的作用[J].北京中医药,2010,29(04):246-249.DOI:10.16025/j.1674-1307.2010.04.026.	Not related to Oral CHM
0997	郭兆安.高血压性肾损害(肾衰竭期)湿浊内蕴证的临床研究[J].中国中西医结合肾病杂志,2007,(11):664-666.	Not related to Oral CHM
0998	郭慧静,赵刚.脾肾气虚兼湿浊血瘀型慢性肾功能衰竭危险因素回顾性分析[J].实用中医内科杂志,2016,30(04):1-3.DOI:10.13729/j.issn.1671-7813.2016.04.01.	Not related to Oral CHM
0999	郭超峰,马晓聪,岳桂华,等.632 例中老年高血压病患者中医临床症状的因子分析[J].时珍国医国药,2015,26(07):1678-1681.	Not related to Oral CHM
1000	关媛媛,王东军,田之魁,等.高血压中医证候研究的系统综述与Meta分析[J].世界中医药,2023,18(09):1253-1259.	Not related to Oral CHM
1001	关小玲.社区 2 型糖尿病患者中医体质分析及探讨[J].社区医学杂志,2019,17(09):529-531.	Not related to Oral CHM

1002	刘义,李枝锋,李坤正,等.2 型糖尿病中医辨证分型与体重指数、血胰岛素水平及血脂关系的研究[J].中西医结合心血管病电子杂志,2019,7(01):172.DOI:10.16282/j.cnki.cn11-9336/r.2019.01.133.	Not related to Oral CHM
1003	高雅静, 王亚松, 隋莹, 2022. 基于Logistic回归分析的中医体质类型与老年原发性高血压的相关性研究, 特别健康.	Not related to Oral CHM
1004	黄铁群, 林友华, 张秀兰, & 傅忠立. (1991). 针刺治疗阻塞性睡眠呼吸暂停综合征 1 例. 中国中西医结合杂志, (5), 306-306.	Not related to Oral CHM
1005	高怀云,熊晶晶,肖义萍.针灸对代谢综合征中医痰湿体质患者糖代谢的影响[J].湖南中医杂志,2021,37(09):68-70.DOI:10.16808/j.cnki.issn1003-7705.2021.09.022.	Not related to Oral CHM
1006	方锐,李思瑶,陈凯飞,等.老年高血压合并冠心病心绞痛的中西医联合筛查模型研究[J].中华中医药杂志,2022,37(04):2207-2213.	Not related to Oral CHM
1007	方锐,胡镜清,王传池,等.两证型老年波动性高血压干预服务包效果评价[J].中国老年学杂志,2017,37(02):325-329.	Not related to Oral CHM
1008	范婷,杨晓晖,乔琳琳,等.171 例老年糖尿病患者中医证候特征与衰弱程度相关性的探讨[J].世界中西医结合杂志,2020,15(02):322-325+329.DOI:10.13935/j.cnki.sjzx.200227.	Not related to Oral CHM
1009	范凯斌,刘必旺.TC、TG、LDL-C与肥胖症中医证型的相关性探讨[J].中国民间疗法,2015,23(08):88-89.DOI:10.19621/j.cnki.11-3555/r.2015.08.068.	Not related to Oral CHM
1010	范慧娟,陈淑娇.围绝经期肥胖患者 154 例中医证素分布特点研究[J].中华中医药杂志,2019,34(03):1153-1156.	Not related to Oral CHM
1011	范洪,张金彪,王元松,等.糖尿病坏疽合并细菌感染中医辨证分型演变规律研究[J].中国中医药现代远程教育,2015,13(16):24-26.	Not related to Oral CHM
1012	董静,马建伟,魏汉林,等.代谢综合征的中医症状调查分析[J].安徽中医学院学报,2012,31(03):18-20.	Not related to Oral CHM
1013	董德刚,喻治达,余忠舜.健身气功·八段锦对痰湿壅盛型高血压患者的干预作用[J].中国应用生理学杂志,2020,36(02):157-160.	Not related to Oral CHM
1014	丁林宝,晏飞,张玉萍.105 例社区高尿酸血症患者证候特点分析[J].世界中西医结合杂志,2011,6(01):39-40.DOI:10.13935/j.cnki.sjzx.2011.01.001.	Not related to Oral CHM
1015	单晓琳,张军,孙辰莹,等.应用中医传承辅助系统对消渴胸痹中医证素分布规律的探究[J].中西医结合心脑血管病杂志,2021,19(02):227-229.	Not related to Oral CHM
1016	代晓愉,卢婷婷,陆相朋,等.基于文献的儿童单纯性肥胖症证候、证素分布特征研究[J].中医杂志,2022,63(04):383-386.DOI:10.13288/j.11-2166/r.2022.04.015.	Not related to Oral CHM
1017	代培,谢培凤,刘铜华,等.2 型糖尿病患者中医证型的分布特点及主要证型中患者体重指数与兼证的相关性[J].中医杂志,2021,62(15):1338-1342.DOI:10.13288/j.11-2166/r.2021.15.011.	Not related to Oral CHM
1018	董广卫,刘庆春.老年人中医体质辨识在基本公共卫生服务中的应用[J].中国实用医药,2017,12(33):197-198.DOI:10.14163/j.cnki.11-5547/r.2017.33.117.	Not related to Oral CHM
1019	崔镇海,金美英,李宗洋,等.基于“骨错缝、筋出槽”理论手法治疗寰枢椎半脱位非特异性病例 2 例[J].吉林中医药,2020,40(12):1663-1666.DOI:10.13463/j.cnki.jlzyy.2020.12.034.	Not related to Oral CHM

1020	崔伟锋,韩颖萍,刘萧萧.高血压病患者中医体质与心血管风险相关性的巢式病例对照研究[J].中医学报,2019,34(08):1740-1744.DOI:10.16368/j.issn.1674-8999.2019.08.409.	Not related to Oral CHM
1021	丛晓东,杨金亮,李斌,等.30 例新型冠状病毒肺炎核酸持续阳性患者临床特征回顾性分析[J].中医杂志,2020,61(24):2121-2125.DOI:10.13288/j.11-2166/r.2020.24.002.	Not related to Oral CHM
1022	褚瑜光,路士华,胡元会.盐敏感性高血压肾素血管紧张素系统及心脏功能形态学与证候学关系研究[J].中国中医基础医学杂志,2018,24(10):1411-1413.DOI:10.19945/j.cnki.issn.1006-3250.2018.10.022.	Not related to Oral CHM
1023	程小平,李秀铭,魏华.147 例代谢综合征湿证患者中医体质调查分析[J].广州中医药大学学报,2021,38(12):2547-2551.DOI:10.13359/j.cnki.gzxbtcm.2021.12.001.	Not related to Oral CHM
1024	陈筑红, 夏城东, 黄佳娜, 韦玮, 胡国庆, & 魏子孝. (2008). 2 型糖尿病并发非酒精性脂肪肝患者证素分析. 中国中西医结合杂志, (10), 879-881.	Not related to Oral CHM
1025	陈莹,李红,许培清,等.围绝经期OSAHS中医证型分布特点研究[J].湖南中医杂志,2019,35(04):4-8.DOI:10.16808/j.cnki.issn1003-7705.2019.04.002.	Not related to Oral CHM
1026	陈淑贤,易惺钱,庾馨予,等.高血压病证候分布的系统评价[J].江西中医药,2020,51(04):54-56.	Not related to Oral CHM
1027	陈洁春,雷小玲,姚典业,等.风湿性心瓣膜病合并心房颤动术中护理风险分析及护理[J].现代临床护理,2014,13(09):28-31.	Not related to Oral CHM
1028	陈剑坤,梁浩斌,李际强.中青年与老年阻塞性睡眠呼吸暂停低通气综合征患者中医证候分布特点及差异[J].新中医,2020,52(05):186-189.DOI:10.13457/j.cnki.jncm.2020.05.056.	Not related to Oral CHM
1029	陈吉全,郭延东,朱永强,等.南阳市 2 型糖尿病患者体质特点及其与中医证候关系的研究[J].中医学报,2014,29(03):338-340.DOI:10.16368/j.issn.1674-8999.2014.03.009.	Not related to Oral CHM
1030	王露露,刘鲁豫,谢秀春.2 型糖尿病合并肥胖患者的糖脂代谢状况及中医证素临床分析[J].海南医学,2023,34(10):1402-1405.	Not related to Oral CHM
1031	陈浩然,王宣尹,陈伟焘,等.重症脑卒中的影像学改变与中风闭脱证的相关性研究[J].广州中医药大学学报,2022,39(12):2735-2740.DOI:10.13359/j.cnki.gzxbtcm.2022.12.002.	Not related to Oral CHM
1032	车旭东,安照华.推腹点穴治疗单纯性肥胖病探析[J].实用中医内科杂志,2005,(02):185-186.DOI:10.13729/j.issn.1671-7813.2005.02.105.	Not related to Oral CHM
1033	曾逸笛, 梁昊, 简维雄, 刘旺华, & 胡志希. (2019). 高血压与中医体质相关性的荟萃分析. 世界科学技术-中医药现代化, 21(8), 1731-1735.	Not related to Oral CHM
1034	曾慧妍,周钦云,赵玲,等.痰湿体质超重肥胖人群CD4+T细胞亚群变化及其与脂代谢相关指标的相关性研究[J].中国中西医结合杂志,2018,38(01):46-49.	Not related to Oral CHM
1035	曾慧妍,周钦云,赵玲,等.痰湿体质超重肥胖人群CD4+T细胞亚群变化及其与脂代谢相关指标的相关性研究[J].中国中西医结合杂志,2018,38(01):46-49.	Not related to Oral CHM
1036	曾慧妍,周钦云,赵玲,等.痰湿体质超重肥胖人群CD4+T细胞亚群变化及其与脂代谢相关指标的相关性研究[J].中国中西医结合杂志,2018,38(01):46-49.	Not related to Oral CHM

1037	Erratum regarding missing Declaration of Competing Interest statements in previously published articles[J].Chinese Herbal Medicines,2021,13(01):143.	Not related to Oral CHM
1038	再生医疗技术治疗创疡的典型病例介绍[J].中国烧伤创疡杂志,2017,29(06):423-438.	Not related to Oral CHM
1039	原位再生医疗技术治疗创疡的典型病例介绍[J].中国烧伤创疡杂志,2017,29(03):200-213.	Not related to Oral CHM
1040	Cabýoglu MT, Ergene N, Tan U. The mechanism of acupuncture and clinical applications. Int J Neurosci. 2006;116(2):115-125. doi:10.1080/00207450500341403.	Not related to Oral CHM
1041	Black J, Houghton WC. Sodium oxybate improves excessive daytime sleepiness in narcolepsy. Sleep. 2006;29(7):939-946. doi:10.1093/sleep/29.7.939.	Not related to Oral CHM
1042	Veterans Administration Cooperative Study Group on Antihypertensive Agents. Effects of treatment on morbidity in mild hypertension: Results of the Veterans Administration Cooperative Study. N Engl J Med. 1967;276(23):1304-1308. doi:10.1056/NEJM196706082762301.	Not related to Oral CHM
1043	Priyanka, S. R., & Singh, R. (2016). A systematic review on indian floral biodiversity as eminent reserves for alternative treatment strategy of diabetes mellitus. Int J Pharm Pharm Sci, 8(4), 10-9.	Not RCTs
1044	Nahin RL, Boon H. Advances in research of complementary and integrative medicine: A review of recent publications in some leading medical journals. J Altern Complement Med. 2011;17(12):1171-1176. doi:10.1089/acm.2011.0525.	Not RCTs
1045	Gagnier JJ, Boon H, Rochon P, Moher D, Barnes J, Bombardier C. Reporting randomized, controlled trials of herbal interventions: an elaborated CONSORT statement. Ann Intern Med. 2006;144(5):364-367. doi:10.7326/0003-4819-144-5-200603070-00013.	Not RCTs
1046	Bagherniya, M., Johnston, T. P., & Sahebkar, A. (2021). Regulation of apolipoprotein B by natural products and nutraceuticals: a comprehensive review. Current Medicinal Chemistry, 28(7), 1363-1406.	Not RCTs
1047	Iranshahy M, Iranshahi M. Traditional uses, phytochemistry and pharmacology of asafoetida (Ferula assa-foetida Oleo-gum-resin)—A review. J Ethnopharmacol. 2011;134(1):1-10. doi:10.1016/j.jep.2010.11.020.	Not RCTs
1048	Auddy B, Hazra J, Mitra A, Abedon B, Ghosal S. A standardized Withania somnifera extract significantly reduces stress-related parameters in chronically stressed humans: a double-blind, randomized, placebo-controlled study. J Am Nutraceutical Assoc. 2008;11(1):50-56.	Not RCTs
1049	SGHA, J. (2018). Ethnopharmacology of Amygdalus Lycioides Spach var. Horrida in east of Esfahan in Iran.	Not RCTs
1050	Sharma, S, Vengal Rao, P, Mehdi, S, Manjula, SN and Das, A, 2020. Hybrid drug combination: A new treatment strategy for type 2 diabetes-a review, International Journal of Research in Pharmaceutical Sciences.	Not RCTs

1051	Panda, O., Sahoo, N., Das, S., Das, S., & Mohanty, D. (2020). Phytochemicals in the remedy of diabetes mellitus: a systematic review.	Not RCTs
1052	Patel DK, Prasad SK, Kumar R, Hemalatha S. An overview on antidiabetic medicinal plants having insulin mimetic property. Asian Pac J Trop Biomed. 2012;2(4):320-330. doi:10.1016/S2221-1691(12)60032-X.	Not RCTs
1053	Liu J, Zhang M, Wang C, et al. Reliability and validity of a patient-reported syndrome scale: phlegm syndrome (PRS-PS). Complement Ther Med. 2012;20(4):219-223. doi:10.1016/j.ctim.2012.01.005.	Not RCTs
1054	Wang, Y., Tian, Q., & Zhou, M. X. (2012). Correlation analysis of 102 hypertension patients and northwest dryness syndrome in xinjiang region. Zhongguo Zhong xi yi jie he za zhi Zhongguo Zhongxiyi Jiehe Zazhi= Chinese Journal of Integrated Traditional and Western Medicine, 32(9), 1200-1203.	Not RCTs
1055	周昕,李智成,顾希钧,等.1 例疑似药物性肝损的用药分析[J].中国药学杂志,2009,44(18):1438-1440.	Not RCTs
1056	童晓萍,劳丽陶.72 例 2 型糖尿病并发社区获得性肺炎患者中医证候分析[J].辽宁中医药大学学报,2010,12(08):100-101.DOI:10.13194/j.jlunivtcm.2010.08.102.tongxp.021.	Not RCTs
1057	赵雯红,张江华,孙姗姗,等.IV期糖尿病肾病患者中医辨证分型与“血瘀”状态相关实验室指标的规律性研究[J].河北中医,2019,41(03):337-341+371.	Not RCTs
1058	Yamamoto H, Harada N, Yamane S, et al. A bitter herbal medicine, Gentiana scabra root extract, stimulates glucagon-like peptide-1 secretion and regulates blood glucose in db/db mice. J Diabetes Investig. 2013;4(3):251-257. doi:10.1111/jdi.12043.	Not RCTs
1059	Lee J, Kang W, Jeong HJ, et al. A call for clinical practice guidelines for the treatment of hypertension with East Asian medicine. Integr Med Res. 2020;9(3):100417. doi:10.1016/j.imr.2020.100417.	Not RCTs
1060	Yu X, Zhao R, Sun Y, et al. Medication utilization among chronic disease patients: a community-based cross-sectional survey in China. BMJ Open. 2014;4(8):e005834. doi:10.1136/bmjopen-2014-005834.	Not RCTs
1061	SHAMIM, A., SIDDIQUI, H. H., MAHMOOD, T., BAGGA, P., & KUMAR, R. (2017). A comprehensive study on literature evidence, clinical studies and practices of herbal drugs for diabetic neuropathy and cardiomyopathy. Asian J Pharm Clin Res, 10(9), 30-37.	Not RCTs
1062	Shakil, S. S., Gowan, M., Hughes, K., Azam, M. N. K., & Ahmed, M. N. (2021). A narrative review of the ethnomedicinal usage of Cannabis sativa Linnaeus as traditional phytomedicine by folk medicine practitioners of Bangladesh. Journal of Cannabis Research, 3, 1-12.	Not RCTs
1063	Yoshikawa M, Murakami T, Shimada H, et al. Salacinol, potent antidiabetic principle with unique thiosugar sulfonium sulfate structure from Salacia reticulata. Org Lett. 1997;62(5):263-266. doi:10.1021/ol970412d.	Not RCTs

106 4	Semenya, S. S., & Maroyi, A. (2019). A review of plants used against diabetes mellitus by Bapedi and Vhavenda ethnic groups in the Limpopo Province, South Africa. Asian J Pharm Clin Res, 12(10), 44-50.	Not RCTs
106 5	Mohamadi, N., Sharififar, F., Pournamdari, M., & Ansari, M. (2018). A review on biosynthesis, analytical techniques, and pharmacological activities of trigonelline as a plant alkaloid. Journal of dietary supplements, 15(2), 207-222.	Not RCTs
106 6	Basch E, Ulbricht C, Kuo G, Szapary P, Smith M. Therapeutic applications of fenugreek. Altern Med Rev. 2003;8(1):20-27.	Not RCTs
106 7	Abd Rahim, I. N., Kasim, N. A. M., Isa, M. R., & Nawawi, H. (2022). A systematic review on the effect of saffron extract on lipid profile in hyperlipidaemic experimental animal models. The Malaysian Journal of Medical Sciences: MJMS, 29(4), 14.	Not RCTs
106 8	Mangoyi, J., Ngcobo, M., Gqaleni, N., Aniekan, P., Owira, P., Cele, P., & Gomo, E. (2021). Acute and sub-acute toxicity of Uthuli lwezichwe™, a traditional medicine used in the management of diabetes mellitus in KwaZulu Natal, South Africa. African Journal of Traditional, Complementary and Alternative Medicines, 18(1), 1-14.	Not RCTs
106 9	Jimmy B, Jose J. Patient medication adherence: measures in daily practice. Oman Med J. 2011;26(3):155-159. doi:10.5001/omj.2011.38.	Not RCTs
107 0	Hamman JH. Composition and applications of Aloe vera leaf gel. Molecules. 2008;13(8):1599-1616. doi:10.3390/molecules13081599.	Not RCTs
107 1	Rasmussen BS, Froekjaer J, Bjerregaard MR, et al. A randomized controlled trial comparing telemedical and standard outpatient monitoring of diabetic foot ulcers. Diabetes Care. 2015;38(9):1723-1729. doi:10.2337/dc15-0200.	Not RCTs
107 2	Modak M, Dixit P, Londhe J, Ghaskadbi S, Paul A, Devasagayam TP. Indian herbs and herbal drugs used for the treatment of diabetes. J Clin Biochem Nutr. 2007;40(3):163-173. doi:10.3164/jcbn.40.163.	Not RCTs
107 3	Ketan Prusty, S., Kumar Sahu, P., & Bhusan Subudhi, B. (2017). Angiotensin mediated oxidative stress and neuroprotective potential of antioxidants and AT1 receptor blockers. Mini Reviews in Medicinal Chemistry, 17(6), 518-528.	Not RCTs
107 4	Sisay, W., Andargie, Y., & Molla, M. (2022). Antidiabetic activity of hydromethanolic extract of crude Dorstenia barnimiana root: validation of in vitro and in vivo antidiabetic and antidyslipidemic activity. Journal of Experimental Pharmacology, 59-72.	Not RCTs
107 5	Zárate R, Saucedo M, Soto-Córdova A, et al. Antidiabetic potential of medicinal plants from the Peruvian Amazon: A review. Biomed Pharmacother. 2021;137:111320. doi:10.1016/j.biopha.2021.111320.	Not RCTs
107 6	Subawa, A. N., Yasa, I. W. P. S., Jawi, I. M., & Mahendra, A. N. (2021). Antioxidant and Hypolipidemic Effects of Ipomoea batatas L and Pandanus conoideus Lam Combination on Rats Fed with High Cholesterol Diet. Open Access Macedonian Journal of Medical Sciences, 9(A), 473-476.	Not RCTs

1077	Liu JP, Li L, Shi Y, et al. Appraisal of the quality and contents of clinical practice guidelines for hypertension management in Chinese medicine: a systematic review. BMJ Open. 2016;6(1):e008687. doi:10.1136/bmjopen-2015-008687.	Not RCTs
1078	Zheng J, Woo SL, Hu X, et al. Artemisia scoparia extract attenuates non-alcoholic fatty liver disease in diet-induced obesity mice by enhancing hepatic insulin and AMPK signaling independently of FGF21 pathway. Metabolism. 2018;83:125-139. doi:10.1016/j.metabol.2018.01.011.	Not RCTs
1079	Wu J, Song X, Chen GC, et al. Association of blood lipid profile with incident chronic kidney disease: a Mendelian randomization study. BMC Nephrol. 2021;22(1):81. doi:10.1186/s12882-021-02267-w.	Not RCTs
1080	Wu M, Xu X, Chen Y, et al. Atheroprotective effects and molecular targets of bioactive compounds from traditional Chinese medicine. Phytomedicine. 2018;44:204-214. doi:10.1016/j.phymed.2018.03.048.	Not RCTs
1081	Bentzon JF, Otsuka F, Virmani R, Falk E. Mechanisms of plaque formation and rupture. Circ Res. 2014;114(12):1852-1866. doi:10.1161/CIRCRESAHA.114.302721.	Not RCTs
1082	Patel, M., & Mehta, P. (2021). Bamboo a supplement to human health: a comprehensive review on its ethnopharmacology, phytochemistry, and pharmacological activity. The Natural Products Journal, 11(2), 140-168.	Not RCTs
1083	Johnston CS, Clifford MN, Morgan LM. Botanical interventions to improve glucose control and options for diabetes therapy. Nutrients. 2021;13(3):800. doi:10.3390/nu13030800.	Not RCTs
1084	Adetunji CO, Abubakar AR, Abdurrahman FI, et al. Butein in health and disease: a comprehensive review. Phytother Res. 2021;35(12):6805-6825. doi:10.1002/ptr.7200.	Not RCTs
1085	Gogtay NJ, Bhatt HA, Dalvi SS, Kshirsagar NA. The use and safety of non-allopathic Indian medicines. Drug Saf. 2002;25(14):1005-1019. doi:10.2165/00002018-200225140-00003.	Not RCTs
1086	Mao, B., Wang, G., & Fan, T. (2007). Change and trend of disease spectrum in randomized controlled trials of traditional Chinese medicine. Zhongguo Zhong xi yi jie he za zhi Zhongguo Zhongxiyi Jiehe Zazhi= Chinese Journal of Integrated Traditional and Western Medicine, 27(5), 404-408.	Not RCTs
1087	Vanherweghem JL. Chinese herbs nephropathy: presentation, natural history and fate after transplantation. Nephrol Dial Transplant. 1998;13(2):104-106. doi:10.1093/ndt/13.suppl_1.104.	Not RCTs
1088	Crawford NW, Cincotta DR, Lim A, Powell CVE. Citation classics in the integrative and complementary medicine literature: 50 frequently cited articles. BMC Complement Altern Med. 2007;7:14. doi:10.1186/1472-6882-7-14.	Not RCTs

1089	Murudkar, P. H., Tambe, M. S., Chandrasekar, S. B., Boddeda, B., & Pawar, A. T. (2022). Common Ayurvedic, Chinese traditional and Unani antidiabetic formulations-a review. Frontiers in Pharmacology, 13, 991083.	Not RCTs
1090	Yousef Nasab, F., Hajinezhad, M. R., Hashemi, H., & Miri, A. (2017). Comparative study of antidiabetic activity of two herbal extracts in alloxan-induced diabetic rats. Oriental Pharmacy and Experimental Medicine, 17, 291-295.	Not RCTs
1091	Su D, Li L. Trends in the use of complementary and alternative medicine in the United States: 2002-2007. J Health Care Poor Underserved. 2011;22(1):296-310. doi:10.1353/hpu.2011.0011.	Not RCTs
1092	Comprehensive review on herbal medicine for energy intake suppression	Not RCTs
1093	Whelton PK. Controlling hypertension: A research success story. J Clin Hypertens (Greenwich). 2010;12(9):667-673. doi:10.1111/j.1751-7176.2010.00342.x.	Not RCTs
1094	Zhou L, Zuo Z, Chow MS. Danshen: An overview of its chemistry, pharmacology, pharmacokinetics, and clinical use. J Clin Pharmacol. 2005;45(12):1345-1359. doi:10.1177/0091270005282630.	Not RCTs
1095	Ochieng, P. J., Kusuma, W. A., Rafi, M. O. H. A. M. A. D., & Sumaryada, T. O. N. Y. (2017). Deciphering the action mechanism of Indonesia herbal decoction in the treatment of type II diabetes using a network pharmacology approach. Int J Pharm Pharm Sci, 9(3), 243-53.	Not RCTs
1096	Zhang Y, Zhang L, Zhang W, et al. Derivation and validation of a prediction score for acute kidney injury secondary to acute myocardial infarction in Chinese patients. BMC Cardiovasc Disord. 2020;20(1):278. doi:10.1186/s12872-020-01524-w.	Not RCTs
1097	Qiu, R., Zhong, C., Wan, S., Zhang, Y., Wei, X., Li, M., ... & Shang, H. (2022). Developing a core outcome set for assessing clinical safety outcomes of cardiovascular diseases in clinical trials of integrated traditional Chinese medicine and Western medicine: study protocol. Trials, 23(1), 239.	Not RCTs
1098	Qiu, R., Li, M., Zhang, X., Chen, S., Li, C., & Shang, H. (2018). Development of a core outcome set (COS) and selecting outcome measurement instruments (OMIs) for non-valvular atrial fibrillation in traditional Chinese medicine clinical trials: study protocol. Trials, 19, 1-10.	Not RCTs
1099	Qiu, R., Sun, Y., Han, S., HE, T., ZHONG, C., GUAN, M., ... & SHANG, H. (2021). Development of a core traditional Chinese medicine syndromes set for non-valvular atrial fibrillation. Chin J Evid Based Med, 21(12), 1480-1488.	Not RCTs
1100	Alicic RZ, Rooney MT, Tuttle KR. Diabetic kidney disease: challenges, progress, and possibilities. Clin J Am Soc Nephrol. 2017;12(12):2032-2045. doi:10.2215/CJN.11491116.	Not RCTs

1101	Manore MM. Dietary supplements for improving body composition and reducing body weight: Where is the evidence? Int J Sport Nutr Exerc Metab. 2012;22(2):139-154. doi:10.1123/ijsnem.22.2.139.	Not RCTs
1102	Wang, X., Peng, B., Li, S., Lian, T., Liu, M., Zhao, X., & Chen, Q. (2020). Distinguish different Chinese medicine types of metabolic syndrome by combining body mass index and uric acid. JPMA, 70(993).	Not RCTs
1103	Tang, S., Gong, Y., Yao, L., Xu, Y., Liu, M., Yang, T., ... & Bai, Y. (2022). Do medical treatment choices affect the health of chronic patients in middle and old age in China?—Evidence from CHARLS 2018. BMC Public Health, 22(1), 937.	Not RCTs
1104	Martins DT, Rodrigues E. Echinodorus grandiflorus: ethnobotanical, phytochemical and pharmacological overview of a medicinal plant used in Brazil. J Ethnopharmacol. 2019;239:111891. doi:10.1016/j.jep.2019.111891.	Not RCTs
1105	Jinxin, M. I. A. O., Mengfan, P. E. N. G., Weihong, R. E. N., & Mingsan, M. I. A. O. (2022). Effect of NLRP3 Inflammasomes on Development of Diabetes Mellitus and Its Complications and Chinese Medicine Intervention via NLRP3: A Review. Chinese Journal of Experimental Traditional Medical Formulae, 254-260.	Not RCTs
1106	Meida, N. S., Purwanto, B., Wasita, B., Indrakila, S., Poncorini, E., Cilmiaty, R., ... & Almahira, S. (2022). Effect of Propolis Extract on Oxidative Stress Biomarker in Diabetic Wistar Rat (Rattus novergicus). Tropical Journal of Natural product Research, 6(8).	Not RCTs
1107	Wahyudin, M. N. M., Natzir, R., Alam, G., & Bukhari, A. S. (2017). Effect of sukun leaf extract [Artocarpus altilis (park.) fosberg] on insulin resistance in obese rats (rattus norvegicus): A study of free fatty acid (ffa) levels. Pak J Nutr, 16(7), 521-524.	Not RCTs
1108	Pulipaka, S., Suttee, A., Kumar, M. R., & Sriram, P. (2022). Effective use of Phytotherapy in the Management of Diabetes by Plant-based Medicine: A Review. synthesis, 4, 5.	Not RCTs
1109	Shao, T., Li, X., Zhou, C., Zang, X., Malone, D. C., Zhang, L., ... & Tang, W. (2021). Effectiveness and efficiency of non-drug therapy among community-dwelling adults with hypertension in China: a protocol for network meta-analysis and cost-effectiveness analysis. Frontiers in Medicine, 8, 651559.	Not RCTs
1110	Malik, M., Hussain, A., Aslam, U., Hashmi, A., Vaismoradi, M., Hayat, K., & Jamshed, S. (2022). Effectiveness of community pharmacy diabetes and hypertension care program: an unexplored opportunity for community pharmacists in Pakistan. Frontiers in Pharmacology, 13, 710617.	Not RCTs
1111	Ma X, Yang C, Zhang Y, et al. Effects of Shenkang Pills on early-stage diabetic nephropathy in db/db mice via inhibiting AURKB/RacGAP1/RhoA signaling pathway. Front Pharmacol. 2022;13:818911. doi:10.3389/fphar.2022.818911.	Not RCTs

1112	Sanusi, J., Bawa, J. A., Aghemwenhio, I. S., Rabi'u, Z. S., Sani, M. G., & Liadi, S. (2016). Efficacy of Securidaca Longepedunculata on the Parameters of Blood Glucose Level and Pulse Rate of Envenomed Albino Rats. International Journal of Pharmaceutical Sciences and Research, 7(12), 4805.	Not RCTs
1113	Chen M, Tian J, Liu Y, et al. Electrospun multifunctional nanofibrous mats loaded with bioactive anemoside B4 for accelerated wound healing in diabetic mice. Int J Biol Macromol. 2020;164:1023-1037. doi:10.1016/j.ijbiomac.2020.07.231.	Not RCTs
1114	Palmer BF, Carrero JJ, Clegg DJ, et al. Emergency management of severe hyperkalemia: guideline for best practice and opportunities for the future. Kidney Int. 2020;97(1):42-61. doi:10.1016/j.kint.2019.09.018.	Not RCTs
1115	Rendon A, Schäkel K. Psoriasis pathogenesis and treatment. Int J Mol Sci. 2019;20(6):1475. doi:10.3390/ijms20061475.	Not RCTs
1116	Singh, G., Passari, A. K., Momin, M. D., Ravi, S., Singh, B. P., & Kumar, N. S. (2020). Ethnobotanical survey of medicinal plants used in the management of cancer and diabetes. Journal of Traditional Chinese Medicine, 40(6).	Not RCTs
1117	James JT, Dubery IA. Pentacyclic triterpenoids from the medicinal herb, Centella asiatica (L.) Urban. Molecules. 2009;14(10):3922-3941. doi:10.3390/molecules14103922.	Not RCTs
1118	Moreno Pena, D. P., Pérez, P. C., Rivas, C. L., Bucio, L., Viveros Valdez, J. E., Munoz Espinosa, L. E., ... & Morales, C. R. (2017). Evaluation of hypocholesterolemic activity of extracts of Bidens odorata and Brickellia eupatorioides. Pakistan Journal of Pharmaceutical Sciences, 30.	Not RCTs
1119	Oridupa, O. A., Folasire, O. F., & Owolabi, A. J. (2017). Evaluation of the sub-chronic toxicity profile of the corm of Xanthosoma sagittifolium on hematology and biochemistry of alloxan-induced diabetic wistar rats. Journal of Complementary and Integrative Medicine, 14(2), 20160072.	Not RCTs
1120	Cordina RL, Celermajer DS, d'Udekem Y, et al. Fontan Fitness Intervention Trial (F-FIT): Rationale and design. Int J Cardiol. 2021;333:115-120. doi:10.1016/j.ijcard.2021.02.027.	Not RCTs
1121	Wong WY, Poudyal H, Ward LC, et al. Tocotrienols for dyslipidemia and cardiovascular health: a review of the current evidence. Nutrients. 2022;14(2):360. doi:10.3390/nu14020360.	Not RCTs
1122	Wang, X., Zhang, H., & Gao, X. (2010). FOCUSED CONFERENCE GROUP: P16-NATURAL PRODUCTS: PAST AND FUTURE? LIQUORICE, PHARMACOLOGICAL AND CLINICAL EXPERIMENTS FROM THE TRADITIONAL CHINESE MEDICINE TO THE MODERN RESEARCH: Paper No.: 837. Basic & Clinical Pharmacology & Toxicology, 107, 649.	Not RCTs

1123	Chen W, Chen L, Zhang Z, et al. From “Kidneys Govern Bones” to chronic kidney disease, diabetes mellitus, and metabolic bone disorder: a crosstalk between traditional Chinese medicine and modern science. Evid Based Complement Alternat Med. 2016;2016:4370263. doi:10.1155/2016/4370263.	Not RCTs
1124	Wang, J., Cui, C., Fu, L., Xiao, Z., Xie, N., Liu, Y., ... & Luo, B. (2016). Genomic expression profiling and bioinformatics analysis on diabetic nephrology with ginsenoside Rg3. Molecular medicine reports, 14(2), 1162-1172.	Not RCTs
1125	Nwokocha CR, Ufearo CS, Anibeze CI, et al. Antidiabetic effects of Gongronema latifolium in diabetic rats and dogs. Exp Clin Endocrinol Diabetes. 2012;120(7):376-382. doi:10.1055/s-0032-1304600.	Not RCTs
1126	Kochhar KP. Dietary spices in health and diseases. Food Res Int. 2008;41(2):123-133. doi:10.1016/j.foodres.2007.11.001.	Not RCTs
1127	Prozialeck WC, Jivan JK, Andurkar SV. Pharmacology of kratom: an emerging botanical agent with stimulant, analgesic and opioid-like effects. J Am Osteopath Assoc. 2012;112(12):792-799.	Not RCTs
1128	Brody GS. Herbal medications and plastic surgery: a hidden danger. Plast Reconstr Surg. 2001;107(2):619-620. doi:10.1097/00006534-200102000-00057.	Not RCTs
1129	Zhang L, Wang Y, Zhang X, et al. Higher adherence to a plant-based diet lowers type 2 diabetes risk among high and non-high cardiovascular risk populations: a cross-sectional study in Shanxi, China. Front Nutr. 2022;9:900133. doi:10.3389/fnut.2022.900133.	Not RCTs
1130	Banerjee SK, Maulik SK. Effect of garlic on cardiovascular disorders: a review. Nutr J. 2002;1:4. doi:10.1186/1475-2891-1-4.	Not RCTs
1131	Li L, Su T, Fang J, et al. Hospital management of major stroke types in Chinese adults: a population-based study of 20,000 hospitalized stroke cases. Front Neurol. 2022;13:819128. doi:10.3389/fneur.2022.819128.	Not RCTs
1132	Bunyapraphatsara N, Yongchaiyudha S, Rungpitarangsi V, Chokechaijaroenporn O. Antidiabetic activity of Aloe vera L. juice. Phytomedicine. 1996;3(3):241-243. doi:10.1016/S0944-7113(96)80060-2.	Not RCTs
1133	Peng, C., HU, X., Chen, Z., Dou, C., Yang, P., BI, J., ... & Liu, T. (2023). Hypoglycemic Effect and Mechanism of Chinese Medicine Saponins: A Review. Chinese Journal of Experimental Traditional Medical Formulae, 266-275.	Not RCTs
1134	Vargas-Tineo, OW, Segura-Muñoz, DM, Becerra-Gutiérrez, LK, Amado-Tineo, JP and Silva-Díaz, H,2020. Hypoglycemic effect of moringa oleifera (Moringa) compared with smallanthus sonchifolius (yacon) on rattus norvegicus with induced diabetes mellitus, Revista Peruana de Medicina Experimental y Salud Publica.	Not RCTs
1135	Olusola AM, Olorunfemi PO, Olaniyan OT. In vivo pharmacodynamic and pharmacokinetic interactions of Hibiscus sabdariffa calyces extracts with simvastatin. J Ethnopharmacol. 2022;293:115292.	Not RCTs

	doi:10.1016/j.jep.2022.115292.	
1136	Mir MA, Sawhney SS, Jassal MM. Indian Sarsaparilla (Hemidesmus indicus): Recent progress in research on ethnobotany, phytochemistry and pharmacology. Pharmacogn Rev. 2013;7(13):55-62. doi:10.4103/0973-7847.112854.	Not RCTs
1137	Leone A, Spada A, Battezzati A, et al. Moringa oleifera seeds and oil: Characteristics and uses for human health. Int J Mol Sci. 2016;17(12):2141. doi:10.3390/ijms17122141.	Not RCTs
1138	Rakel D. Integrative approaches to hypertension. Prim Care. 2017;44(2):279-295. doi:10.1016/j.pop.2017.01.009.	Not RCTs
1139	Longo FM, Chan K. Neuropathy and neuropathic pain: Consider the alternatives. Phys Med Rehabil Clin N Am. 2014;25(1):161-177. doi:10.1016/j.pmr.2013.09.003.	Not RCTs
1140	Montealegre, L., Amador, E., Pulido, T., Cabrera, C., Rivera, A., & Rebolledo, R. (2022). MAIN FACTORS THAT DETERMINE ADHERENCE TO PHARMACOLOGICAL TREATMENT IN HYPERTENSIVE PATIENTS. SYSTEMATIC REVIEW. Journal of Hypertension, 40(Suppl 1), e308.	Not RCTs
1141	Paice JA, Portenoy R, Lacchetti C, et al. Management of chronic pain in survivors of adult cancers: American Society of Clinical Oncology clinical practice guideline. J Clin Oncol. 2016;34(27):3325-3345. doi:10.1200/JCO.2016.68.5206.	Not RCTs
1142	Tremosini S, Forner A, Reig M, Bruix J. Treatment of hepatocellular carcinoma with portal vein thrombosis: State of the art. Clin Liver Dis. 2011;15(3):519-535. doi:10.1016/j.cld.2011.03.006.	Not RCTs
1143	Xia, S. J., Gao, B. Z., Wang, S. H., Guttery, D. S., Li, C. D., & Zhang, Y. D. (2021). Modeling of diagnosis for metabolic syndrome by integrating symptoms into physiochemical indexes. Biomedicine & Pharmacotherapy, 137, 111367.	Not RCTs
1144	He T, Sun X, He B, et al. Modern interpretation of the traditional application of Shihu—A comprehensive review on phytochemistry and pharmacology progress of Dendrobium officinale. Front Pharmacol. 2021;12:619176. doi:10.3389/fphar.2021.619176.	Not RCTs
1145	Mukherjee, P. K., Banerjee, S., & Kar, A. (2021). Molecular combination networks in medicinal plants: understanding synergy by network pharmacology in Indian traditional medicine. Phytochemistry reviews, 20(4), 693-703.	Not RCTs
1146	Polyzos SA, Kountouras J, Mantzoros CS. Obesity and nonalcoholic fatty liver disease: From pathophysiology to therapeutics. Metabolism. 2019;92:82-97. doi:10.1016/j.metabol.2018.11.014.	Not RCTs

1147	Shanmugam S, Thangaraj P, Subban R. Natural products for diabetes mellitus: a review. Curr Pharm Biotechnol. 2011;12(9):1230-1242. doi:10.2174/138920111796117661.	Not RCTs
1148	Martel J, Ojcius DM, Ko YF, Young JD, Wang YC. Anti-obesity effects of dietary polyphenols: a review. J Food Drug Anal. 2017;25(1):118-126. doi:10.1016/j.jfda.2016.10.017.	Not RCTs
1149	Orekhov, A. N., Grechko, A. V., Romanenko, E. B., Zhang, D., & Chistiakov, D. A. (2020). Novel Approaches to Anti-atherosclerotic Therapy: Cell-based Models and Herbal Preparations (Review of Our Own Data). Current Drug Discovery Technologies, 17(3), 278-285.	Not RCTs
1150	Houston MC. Nutritional supplements for the treatment of hypertension: A practical guide for clinicians. Prog Cardiovasc Dis. 2016;59(4):379-385. doi:10.1016/j.pcad.2016.11.003.	Not RCTs
1151	Yang J, Chow IT, Sosinowski T, et al. Pathophysiological characteristics of preproinsulin-specific CD8+ T cells in subjects with juvenile-onset and adult-onset type 1 diabetes: a 1-year follow-up study. Diabetes. 2016;65(11):3097-3107. doi:10.2337/db16-0414.	Not RCTs
1152	Sarikurkcu C, Tepe B. Plants used against obesity in Turkish folk medicine: a review. J Ethnopharmacol. 2013;146(1):1-10. doi:10.1016/j.jep.2012.11.045.	Not RCTs
1153	Shiuan, C., Mogana, R., Akowuah, G. A., Chinnappan, S., & Abdullah, N. H. (2022). Polyherbal formulation for primary dysmenorrhea: A review. Research Journal of Pharmacy and Technology, 15(4), 1891-1900.	Not RCTs
1154	Li Y, Wang J, Wang X, et al. Polysaccharides from Armillariella tabescens mycelia ameliorate renal damage in type 2 diabetic mice. Carbohydr Polym. 2019;204:450-463. doi:10.1016/j.carbpol.2018.09.040.	Not RCTs
1155	Huang H, Huang B, Li Y, et al. Positive association of serum uric acid with new-onset diabetes in Chinese women with hypertension: a retrospective analysis of the China Stroke Primary Prevention Trial. Acta Diabetol. 2017;54(10):943-950. doi:10.1007/s00592-017-1042-1.	Not RCTs
1156	Marmitt, D. J. (2022). Potential plants for inflammatory dysfunction in the SARS-CoV-2 infection. Inflammopharmacology, 30(3), 749-773.	Not RCTs
1157	Qamar A, Qamar M, Anjum S, et al. Potential protective effects of Aloe vera gel on cardiovascular diseases: a mini-review. J Complement Integr Med. 2022;19(4):737-743. doi:10.1515/jcim-2021-0090.	Not RCTs
1158	Asl MN, Hosseinzadeh H. Review of pharmacological effects of Glycyrrhiza sp. and its bioactive compounds. Phytother Res. 2008;22(6):709-724. doi:10.1002/ptr.2362.	Not RCTs
1159	Grant RW, McCloskey J, Hatfield M, et al. Prevalence and associations of complementary and alternative medicine natural product use in adults with diabetes. Diabetes Care. 2004;27(2):321-322. doi:10.2337/diacare.27.2.321.	Not RCTs

1160	Lu J, Zhong C, Wang T, et al. Prevalence, diagnosis, and management of diabetes mellitus among older Chinese: results from the China Health and Retirement Longitudinal Study. BMJ Open. 2021;11(8):e049910. doi:10.1136/bmjopen-2021-049910.	Not RCTs
1161	Saxena, P. K. (2010, May). Prospects and Limitations for Ayurvedic Products in Canada: Tulsi (Ocimum Sanctum) as a Case Study. In PHARMACEUTICAL BIOLOGY (Vol. 48, pp. 3-3). 325 CHESTNUT ST, SUITE 800, PHILADELPHIA, PA 19106 USA: TAYLOR & FRANCIS INC.	Not RCTs
1162	Zhao, XJ, Li, C, Nan, Z and Deng, Y, 2005. Protective effect of detoxifying and collateral-dredging Baoshen Capsule on the kidney of diabetic rats, Chinese Journal of Clinical Rehabilitation.	Not RCTs
1163	Zangeneh, A., Zangeneh, M., Ghoodarzi, N., Najafi, F., & Hagh Nazari, L. (2018). Protective effects of aqueous extract of internal septum of walnut fruit on diabetic hepatopathy in streptozotocin-induced diabetic mice. Scientific Journal of Kurdistan University of Medical Sciences, 23(1), 26-37.	Not RCTs
1164	Gutiérrez RM, Mitchell S, Solis RV. Psidium guajava: a review of its traditional uses, phytochemistry, and pharmacology. J Ethnopharmacol. 2008;117(1):1-27. doi:10.1016/j.jep.2008.01.025.	Not RCTs
1165	Parisi R, Symmons DP, Griffiths CE, Ashcroft DM. Global epidemiology of psoriasis: a systematic review of incidence and prevalence. J Invest Dermatol. 2013;133(2):377-385. doi:10.1038/jid.2012.339.	Not RCTs
1166	Xu H, Su Y, Zhang Y, et al. Research on "symptom-based strategy of dosage selection" of traditional Chinese medicine: an integrated analysis of seven randomized, controlled, multicenter clinical trials about type 2 diabetes. BMC Complement Altern Med. 2017;17:473. doi:10.1186/s12906-017-1783-3.	Not RCTs
1167	Mashhadi NS, Ghiasvand R, Askari G, Hariri M, Darvishi L, Mofid MR. Anti-oxidative and anti-inflammatory effects of ginger in health and physical activity: review of current evidence. Int J Prev Med. 2013;4(Suppl 1):S36-S42.	Not RCTs
1168	Polshettiwar, S. A., Sawant, D. H., Abhale, N. B., Chavan, N. B., Baheti, A. M., & Wani, M. S. (2022). Review on Regulation of Herbal Products Used as a Medicine Across the Globe: A Case Study on Turmeric–Golden Medicine. Biomedical and Pharmacology Journal, 15(3), 1227-1237.	Not RCTs
1169	Santos, R. V., Rodrigues, J. M., & Jesus, M. I. (2020). Review on the effects of obesity treatment with acupuncture and phytoacupuncture. World Journal of Acupuncture-Moxibustion, 30(3), 223-228.	Not RCTs
1170	Chatsudthipong V, Muanprasat C. Stevioside and related compounds: Therapeutic benefits beyond sweetness. Pharmacol Ther. 2009;121(1):41-54. doi:10.1016/j.pharmthera.2008.09.007.	Not RCTs

1171	Ramakrishna, D., Shashank, A. T., Shinomol, G. K., Kiran, S., & Ravishankar, G. A. (2015). Salacia Sps–a potent source of herbal drug for antidiabetic and antiobesity ailments: a detailed treatise. Int. J. Pharm. Phytochem. Res, 7, 374-382.	Not RCTs
1172	Tabatabaei-Malazy, O., Atlasi, R., Hasani-Ranjbar, S., Abdollahi, M., Dastjerdi, M. V., & Larijani, B. (2020). Scientometric study of academic publications on herbal medicines in Endocrinology & Metabolism Research Institute (EMRI) of Tehran University of Medical Sciences. Journal of Diabetes & Metabolic Disorders, 1-9.	Not RCTs
1173	Epstein LJ, Kristo D, Strollo PJ Jr, et al. Clinical guideline for the evaluation, management and long-term care of obstructive sleep apnea in adults. J Clin Sleep Med. 2009;5(3):263-276.	Not RCTs
1174	Yan, J., Engle, V. F., He, Y., Jiao, Y., & Gu, W. (2009). Study designs of randomized controlled trials not based on Chinese medicine theory are improper. Chinese medicine, 4, 1-5.	Not RCTs
1175	Pan, S. Y., Nie, Q., Tai, H. C., Song, X. L., Tong, Y. F., Zhang, L. J. F., ... & Liang, C. (2022). Tea and tea drinking: China's outstanding contributions to the mankind. Chinese medicine, 17(1), 27.	Not RCTs
1176	Yang X, Guo J, Liu C, et al. The effect of Chinese medicinal formulas on biomarkers of oxidative stress in STZ-induced diabetic kidney disease rats: a meta-analysis and systematic review. Oxid Med Cell Longev. 2020;2020:3949648. doi:10.1155/2020/3949648.	Not RCTs
1177	Takaeidi, M. R., Jahangiri, A., Khodayar, M. J., Siahpoosh, A., Yaghooti, H., Rezaei, S., ... & Mansourzadeh, Z. (2014). The effect of date seed (Phoenix dactylifera) extract on paraoxonase and arylesterase activities in hypercholesterolemic rats. Jundishapur journal of natural pharmaceutical products, 9(1), 30.	Not RCTs
1178	Li ZG, Zhang W, Sima AA. Alzheimer-like changes in diabetic peripheral neuropathy and retinal disease. Rev Diabet Stud. 2007;4(3):149-163. doi:10.1900/RDS.2007.4.149.	Not RCTs
1179	Mahmood A, Al-Qahtani MF. The genus Anogeissus: A review on ethnopharmacology, phytochemistry and pharmacology. Plants (Basel). 2021;10(12):2702. doi:10.3390/plants10122702.	Not RCTs
1180	Wang W, Ma X, Han J, Zhou M, Ren S. The genus Rhododendron: An ethnopharmacological and toxicological review. J Ethnopharmacol. 2022;282:114578. doi:10.1016/j.jep.2021.114578.	Not RCTs
1181	Nasehi, Z., Shahaboddin, M. E., & Jafarnejad, S. (2023). The Influence of Securigera securidaca on Diabetes Management in Animal Models: A Systematic Review. Journal of Advances in Medical and Biomedical Research, 31(144), 14-24	Not RCTs
1182	Ferrari R, Ford I, Greenlaw N, et al. Cardiovascular risk and side effects associated with anti-anginal medications: Insights from the CLARIFY registry. Eur Heart J Cardiovasc Pharmacother. 2019;5(3):141-150. doi:10.1093/ehjcvp/pvz009.	Not RCTs

1183	Mekonnen AB, McLachlan AJ, Brien JE. Effectiveness of clinical pharmacy services: A systematic review and meta-analysis. Int J Clin Pharm. 2016;38(3):620-630. doi:10.1007/s11096-015-0226-7.	Not RCTs
1184	Chen X, Li W, Xiao P. Atractylodes macrocephala Koidz.: A review of its traditional uses, phytochemistry and pharmacology. J Ethnopharmacol. 2017;207:153-170. doi:10.1016/j.jep.2017.06.042.	Not RCTs
1185	Chrubasik C, Roufogalis BD, Müller-Ladner U, Chrubasik S. A systematic review on the Rosa canina effect and efficacy profiles. Phytother Res. 2008;22(6):725-733. doi:10.1002/ptr.2400.	Not RCTs
1186	Tang T, Song J, Zhao J, et al. Protective effect of herbal medicine against non-alcoholic fatty liver disease: A review. Phytomedicine. 2020;62:152948. doi:10.1016/j.phymed.2019.152948.	Not RCTs
1187	Ren, Y., Qiao, W., Fu, D., Han, Z., Liu, W., Ye, W., & Liu, Z. (2017). Traditional Chinese medicine protects against cytokine production as the potential immunosuppressive agents in atherosclerosis. Journal of immunology research, 2017(1), 7424307.	Not RCTs
1188	Parkman HP, Hasler WL, Fisher RS. American Gastroenterological Association technical review on the diagnosis and treatment of gastroparesis. Gastroenterology. 2004;127(5):1592-1622. doi:10.1053/j.gastro.2004.09.055.	Not RCTs
1189	Wu J, Pan L, Gao Z, et al. Tripterygium glycoside suppresses EMT of diabetic kidney disease podocytes via mTOR/Twist1-mediated autophagy. Front Pharmacol. 2021;12:646263. doi:10.3389/fphar.2021.646263.	Not RCTs
1190	Rampal L, Rampal S, Azhar MZ, Rahman AR. Prevalence, awareness, treatment and control of hypertension in Malaysia: a national survey of 16,440 subjects. Public Health. 2008;122(1):11-18. doi:10.1016/j.puhe.2007.05.008.	Not RCTs
1191	Tsutani K, Morimoto T, Fukui T. Use of Kampo diagnosis in randomized controlled trials of Kampo products in Japan: a systematic review. BMC Complement Altern Med. 2014;14:307. doi:10.1186/1472-6882-14-307.	Not RCTs
1192	Shupeng, C. H. E. N., Nana, T. A. N. G., Simeng, W. A. N. G., Yinghua, L. I. U., Zhiyong, Z. H. A. N. G., & Shiyu, C. H. E. N. (2023). Visual analysis of research hotspots and trends of external therapies in traditional Chinese medicine for insomnia. Digital Chinese Medicine, 6(1), 41-54.	Not RCTs
1193	Taylor SL, Kilts JD, Patel KK, et al. We Built it, But Did They Come: Veterans' Use of VA Healthcare System-Provided Complementary and Integrative Health Approaches. Med Care. 2019;57(Suppl 1):S76-S82. doi:10.1097/MLR.0000000000001033.	Not RCTs
1194	Singh N, Bhalla M, de Jager P, Gilca M. An overview on Ashwagandha: a Rasayana (rejuvenator) of Ayurveda. Afr J Tradit Complement Altern Med. 2011;8(5 Suppl):208-213. doi:10.4314/ajtcam.v8i5S.9.	Not RCTs
1195	Jones ML, Mark PJ, Waddell BJ. Maternal omega-3 fatty acid supplementation and offspring metabolic health: a review of potential mechanisms. Nutrients. 2014;6(4):1539-1576. doi:10.3390/nu6041539.	Not RCTs

1196	张宇霞,杨雨民,周芸慧,等.半夏白术天麻汤对痰湿壅盛型高血压大鼠血清ET-1 及NO含量的影响[J].内蒙古中医药,2018,37(06):99-101.DOI:10.16040/j.cnki.cn15-1101.2018.06.073.	Not RCTs
1197	谢卫平.辨体论治验案举隅[J].国医论坛,2016,31(04):47-48.DOI:10.13913/j.cnki.41-1110/r.2016.04.031.	Not RCTs
1198	张莉,关键,李国华,等.布氏杆菌感染致急性肾损伤伴心内膜炎 1 例并文献复习[J].中国中西医结合肾病杂志,2019,20(10):917-918.	Not RCTs
1199	赵玉华,曾定伦.曾定伦治疗高脂血症经验[J].河南中医,2017,37(11):1905-1907.DOI:10.16367/j.issn.1003-5028.2017.11.0659.	Not RCTs
1200	任志鑫,王雨,张冰,等.代谢性疾病病证结合动物模型的探讨与思考[J].中国实验动物学报,2022,30(07):897-908.	Not RCTs
1201	周强,逯冰,彭智平,等.当归芍药散加减治疗水肿二则[J].山东中医杂志,2013,32(05):366-367.DOI:10.16295/j.cnki.0257-358x.2013.05.017.	Not RCTs
1202	张良登.非酒精性脂肪肝横断面调查及清热祛湿化瘀法干预的脂肪因子机制[D].北京中医药大学,2014.	Not RCTs
1203	牛祎初.基于数据挖掘研究史大卓教授辨治高血压遣方用药规律[D].北京中医药大学,2021.DOI:10.26973/d.cnki.gbjzu.2021.000666.	Not RCTs
1204	朱涛,伏小亮.心内科老年患者在院内心源性猝死病因及临床危险因素[J].世界最新医学信息文摘,2016,16(05):73+76.	Not RCTs
1205	张笑蕊,曲超,杨宇峰,等.基于中医传承辅助平台当代医家辨证论治消渴用药规律[J].中华中医药学刊,2021,39(08):66-70.DOI:10.13193/j.issn.1673-7717.2021.08.015.	Not RCTs
1206	翁雅婧,汪悦.健脾祛湿法治疗肥胖症病案举隅[J].吉林中医药,2013,33(02):194-195.DOI:10.13463/j.cnki.jlzyy.2013.02.030.	Not RCTs
1207	周铭,韩文娟,王久玉.李丽萍主任医师治疗糖尿病胃轻瘫验案举隅[J].中国中医药现代远程教育,2018,16(23):73-75.	Not RCTs
1208	徐化宇,朱晓男.缺血型糖尿病足诊疗规律回顾性研究[J].实用中医内科杂志,2014,28(06):1-3.DOI:10.13729/j.issn.1671-7813.2014.06.01.	Not RCTs
1209	颜榕,余永鑫,阮诗玮.阮诗玮医话三则[J].亚太传统医药,2023,19(01):102-105.	Not RCTs
1210	周慢,赵兴旺,朱宇溪,等.三才汤加味治疗糖尿病临床应用举隅[J].湖南中医杂志,2018,34(05):120-122.DOI:10.16808/j.cnki.issn1003-7705.2018.05.057.	Not RCTs
1211	周文,黄毅.三峡库区老年单纯收缩期高血压中医证候流行病学调查[J].四川中医,2017,35(08):49-51.	Not RCTs
1212	张琦,刘文全.三子养亲汤临证新用举隅[J].内蒙古中医药,2017,36(19):42.DOI:10.16040/j.cnki.cn15-1101.2017.19.036.	Not RCTs
1213	俞帼英.上海市某社区老年高血压患者中医证型分布及临床特点调查[J].中国老年保健医学,2017,15(02):61-62.	Not RCTs
1214	孙延春,沈铁.上海市浦东新区代谢综合征中医证候分布及与各代谢组分关系的研究[J].中国初级卫生保健,2016,30(06):72-73.	Not RCTs
1215	章敬芳,徐聪彬.深圳市罗湖区 60 岁及以上老年“三高症”患者的中医体质辨识调查[J].中国当代医药,2018,25(19):164-166.	Not RCTs

1216	王丽,李杰辉,张春霞,等.湿润烧伤膏对糖尿病大鼠创面组织细胞间黏附分子 1 和血管细胞黏附分子 1 mRNA表达水平及超微结构的影响研究[J].中国全科医学,2018,21(11):1315-1320.	Not RCTs
1217	张焱,陈咸川,何立人,等.痰湿壅塞证高血压病患者血管内皮依赖性舒张功能的超声研究[J].江西中医学院学报,2006,(01):26-27.	Not RCTs
1218	庞曼丽,蒲诗函,马建伟.糖尿病足中医体质类型及相关性研究[J].解放军医药杂志,2021,33(11):97-100.	Not RCTs
1219	王旭,洪兵,孙斯凡,等.糖足洗液对糖尿病足小鼠皮肤溃疡的影响[J].中医杂志,2011,52(16):1402-1405.DOI:10.13288/j.11-2166/r.2011.16.032.	Not RCTs
1220	王艳云,孔令新,田朝阳,等.维持性血液透析中低血压的中医证素分布规律的调查[J].中国中医药现代远程教育,2017,15(20):56-59.	Not RCTs
1221	张军鹏,徐变玲,张理,等.吸取中医元素的高血压病风险预警系统构建[J].中华中医药杂志,2018,33(10):4705-4712.	Not RCTs
1222	施学丽,温宗良,张楠,等.原发性高血压合并抑郁的相关因素及证型分布规律的临床调查[J].时珍国医国药,2013,24(08):1947-1949.	Not RCTs
1223	章天寿.张杰主任医师学术思想与临床经验总结及补肾降浊法治疗高脂血症的临床研究[D].南京中医药大学,2015.	Not RCTs
1224	衷敬柏, 2014.高血压病病证结合中医诊疗指南方法学研究, 中国中医科学院	Not RCTs
1225	张北华.基于文献评价和数据挖掘的魏子孝诊治糖尿病周围神经病变经验总结[D].中国中医科学院,2010.	Not RCTs
1226	周才根.中医内科对眩晕患者病因分析的研究[J].中国中医药现代远程教育,2021,19(03):114-116.	Not RCTs
1227	田立茹.中医体质状态与缺血性中风复发关系的巢式病例对照研究[D].华北理工大学,2019.	Not RCTs
1228	张娜,花永强,章怡祎,等.中医药治疗高脂血症临床随机对照试验的质量评价[J].中华中医药学刊,2009,27(10):2060-2063.DOI:10.13193/j.archtcm.2009.10.46.zhangn.011.	Not RCTs
1229	Hosseini A, Razavi BM, Hosseinzadeh H. Protective effect of Zataria multiflora Boiss. extract on oxidative stress and TNF-α production in diabetic rats. Iran J Basic Med Sci. 2017;20(5):531-536.	Not RCTs
1230	Maheswari, C., Venkatnarayanan, R., Babu, P., & Kandasamy, C. S. (2015). Green tea (cardiac tea) vs java tea (kidney tea): A review. Research Journal of Pharmacy and Technology, 8(1), 94-100.	Not RCTs
1231	Tang SM, Dong LY, Wang Y, et al. Medicinal plants and phytochemicals regulating insulin resistance and glucose homeostasis in type 2 diabetic patients: a clinical review. J Ethnopharmacol. 2022;289:115011. doi:10.1016/j.jep.2021.115011.	Not RCTs
1232	MADANI, H., RAHIMI, P., & Mahzouni, P. (2009). Effects of hydroalcoholic extract of Juglans regia leaves on activity of AST and ALT enzymes in alloxan-induced diabetic rats.	Not RCTs
1233	Nicholls SJ, Nelson AJ, Ray KK, et al. From herbs to biologics: lipid management beyond statins. Nat Rev Cardiol. 2022;19(10):675-690. doi:10.1038/s41569-022-00687-4.	Not RCTs

1234	Chen Y, Zhang Y, Liu Y, et al. The efficacy and mechanism of Chinese herbal medicine on diabetic kidney disease: A review. Front Pharmacol. 2022;13:862595. doi:10.3389/fphar.2022.862595.	Not RCTs
1235	Costa LG, Cole TB, Vitalone A, Furlong CE. Paraoxonase (PON1): from toxicology to cardiovascular medicine. Toxicol Appl Pharmacol. 2005;207(1):77-86. doi:10.1016/j.taap.2004.10.030.	Not RCTs
1236	Lo, H. Y., Li, T. C., Yang, T. Y., Li, C. C., Chiang, J. H., Hsiang, C. Y., & Ho, T. Y. (2017). Hypoglycemic effects of Trichosanthes kirilowii and its protein constituent in diabetic mice: the involvement of insulin receptor pathway. BMC complementary and alternative medicine, 17, 1-9.	Not RCTs
1237	Liu, Y., Zhang, X., Yang, L., Zhou, S., Li, Y., Shen, Y., ... & Liu, Y. (2023). Proteomics and transcriptomics explore the effect of mixture of herbal extract on diabetic wound healing process. Phytomedicine, 116, 154892.	Not RCTs
1238	Guo Y, Li J, Li CI, et al. New insights for cellular and molecular mechanisms of aging and aging-related diseases: herbal medicine as potential therapeutic approach. Oxid Med Cell Longev. 2022;2022:7858128. doi:10.1155/2022/7858128.	Not RCTs
1239	Ren T, Guo R, Zhang P, et al. Gut microbiota and antidiabetic drugs: new insights into personalized treatment of type 2 diabetes mellitus. Front Endocrinol (Lausanne). 2022;13:890345. doi:10.3389/fendo.2022.890345.	Not RCTs
1240	Guo D, Yu Y, Wang X, et al. Mechanism of Cordyceps sinensis and its extracts in the treatment of diabetic kidney disease: a review. Front Pharmacol. 2022;13:869515. doi:10.3389/fphar.2022.869515.	Not RCTs
1241	Li Y, Ma W, Zhu X, et al. Hyperoside suppresses renal inflammation by regulating macrophage polarization in type 2 diabetic mice. Front Pharmacol. 2021;12:744202. doi:10.3389/fphar.2021.744202.	Not RCTs
1242	Lin, Y., Zhang, Y., Wang, D., Yang, B., & Shen, Y. Q. (2022). Computer especially AI-assisted drug virtual screening and design in traditional Chinese medicine. Phytomedicine, 107, 154481.	Not RCTs
1243	Lin, M., Wang, L., Wan, L., Xu, J., Li, Y., Cao, L., & Lin, S. (2023). The antidiabetic effect and mechanism of jinXiaoXiaoKe decoction in type 2 diabetic goto–kakizaki rats. Clinical Complementary Medicine and Pharmacology, 3(1), 100049.	Not RCTs
1244	Deng Y, Wang Y, Tan L, et al. Zhenqing recipe relieves diabetic nephropathy through the SIK1/SREBP-1c axis in type 2 diabetic rats. Front Endocrinol (Lausanne). 2022;13:865594. doi:10.3389/fendo.2022.865594.	Not RCTs
1245	Feng X, Sureda A, Jafari S, et al. Berberine and health outcomes: an umbrella review. Phytother Res. 2019;33(3):601-611. doi:10.1002/ptr.6261.	Not RCTs
1246	Zhang A, Sun H, Wang P, et al. Quality markers in Chinese medicine: Definition, progress and prospects. Chin J Nat Med. 2020;18(10):776-788. doi:10.1016/S1875-5364(20)60019-0.	Not RCTs

1247	Botany, traditional usages, phytochemistry, pharmaceutical analysis, and pharmacology of Eleutherococcus nodiflorus (Dunn) S.Y.Hu: A systematic review	Not RCTs
1248	Li, X., Chu, L., Liu, S., Zhang, W., Lin, L., & Zheng, G. (2022). Smilax china L. flavonoid alleviates HFHS-induced inflammation by regulating the gut-liver axis in mice. Phytomedicine, 95, 153728.	Not RCTs
1249	Shi Y, Wang S, Mao J, et al. Education programs for people with diabetic kidney disease: a systematic review and meta-analysis. J Diabetes Res. 2020;2020:7121254. doi:10.1155/2020/7121254.	Not RCTs
1250	Li, S. J., Wang, Y. Q., Zhuang, G., Jiang, X., Shui, D., & Wang, X. Y. (2023). Overall metabolic network analysis of urine in hyperlipidemic rats treated with Bidens bipinnata L. Biomedical Chromatography, 37(1), e5509.	Not RCTs
1251	Song P, Zhu Y, Bai J, et al. Cost-effectiveness of telemedicine screening for diabetic retinopathy in rural and urban China. JAMA Ophthalmol. 2019;137(5):527-534. doi:10.1001/jamaophthalmol.2019.0021.	Not RCTs
1252	Li, M., Jiang, H., Hao, Y., Du, K., Du, H., Ma, C., ... & He, Y. (2022). A systematic review on botany, processing, application, phytochemistry and pharmacological action of Radix Rehmnniae. Journal of Ethnopharmacology, 285, 114820.	Not RCTs
1253	Li, J. M., Zhao, Y., Sun, Y., & Kong, L. D. (2020). Potential effect of herbal antidepressants on cognitive deficit: Pharmacological activity and possible molecular mechanism. Journal of ethnopharmacology, 257, 112830.	Not RCTs
1254	Liu W, Tan J, Liu Y, et al. Panax notoginseng saponins improve erectile function through attenuation of oxidative stress and protection of endothelial and smooth muscle cells in diabetic rats with erectile dysfunction. J Ethnopharmacol. 2015;174:386-394. doi:10.1016/j.jep.2015.08.047.	Not RCTs
1255	Wang X, Zhang H, Chen X, et al. Schisandrin B: a double-edged sword in nonalcoholic fatty liver disease. Phytomedicine. 2022;99:153978. doi:10.1016/j.phymed.2022.153978.	Not RCTs
1256	Li SY, Yang D, Yeung CM, et al. Lycium barbarum polysaccharides reduce neuronal damage in a rat model of acute ocular hypertension. PLoS One. 2011;6(4):e19133. doi:10.1371/journal.pone.0019133.	Not RCTs
1257	Laksemi, D. A., Sukrama, D. M., Sudarmaja, M., Damayanti, P. A., Swastika, K., Diarthini, N. L., ... & Tunas, K. (2021). Medicinal plants as recent complementary and alternative therapy for COVID-19: a review.	Not RCTs
1258	Kushwaha, P. S., Singh, A. K., Keshari, A. K., & MaityS, S. S. An updated review on the phytochemistry, pharmacology, and clinical trials of Salacia oblonga. Pharmacognosy Review. 2016; 10 (20): 109-14.	Not RCTs
1259	Kumari, P., Sharma, S., Sharma, P. K., & Alam, A. (2023). Treatment management of diabetic wounds utilizing herbalism: An overview. Current Diabetes Reviews, 19(1), 92-108.	Not RCTs

1260	Koul, B., Taak, P., Kumar, A., Kumar, A., & Sanyal, I. (2019). Genus Psoralea: A review of the traditional and modern uses, phytochemistry and pharmacology. Journal of ethnopharmacology, 232, 201-226.	Not RCTs
1261	Gao Y, Li J, Chu S, et al. A review of endothelium-dependent and -independent vasodilation induced by phytochemicals in isolated rat aorta. Phytomedicine. 2016;23(13):1173-1183. doi:10.1016/j.phymed.2016.07.003.	Not RCTs
1262	Kim, S. H., Kim, S., Lee, S. H., Park, H. W., Chang, Y. S., Min, K. U., & Cho, S. H. (2011). The effects of PG102, a water-soluble extract from Actinidia arguta, on serum total IgE levels: a double-blind, randomized, placebo-controlled exploratory clinical study. European journal of nutrition, 50, 523-529.	Not RCTs
1263	Kim, S., Kim, B. Y., Ko, M. M., & Son, M. J. (2022). Development of template for reporting adverse events of Ephedrae Herba for weight loss. 한국콘텐츠학회 ICCC 논문집, 115-116.	Not RCTs
1264	Kim, J. K., Tabassum, N., Uddin, M. R., & Park, S. U. (2016). Ginseng: a miracle sources of herbal and pharmacological uses. Oriental Pharmacy and Experimental Medicine, 16, 243-250.	Not RCTs
1265	Kim, J., Kim, Y. S., Lee, H. A., Lim, J. Y., Kim, M., Kwon, O., ... & Kim, Y. (2014). Sasa quelpaertensis leaf extract improves high fat diet-in	Not RCTs
1266	Seeram NP, Aviram M, Zhang Y, et al. The Berry Health Benefits Network. J Agric Food Chem. 2010;58(7):3871-3879. doi:10.1021/jf903484u.	Not RCTs
1267	Ernst E. Alternative medicine: herbal drugs and their critical appraisal. Part II. Perfusion. 2003;16(1):12-20. doi:10.1191/0967618303pf654oa.	Not RCTs
1268	Shukla A, Rasik AM, Dhawan BN. Plants in wound healing: US patent review. Phytother Res. 1999;13(7):607-612. doi:10.1002/(SICI)1099-1573(199911)13:7<607::AID-PTR491>3.0.CO;2-F.	Not RCTs
1269	Kanthain, R., Leelarungrayub, J., Likhitsathian, S., & Natakankitkul, S. (2022). Efficacy of Combined Relaxed Deep-Breathing with Chest Mobilization Exercise and Vernonia cinerea-Hard Candy on Smoking Cessation and Oxidative Stress in Active Teenage Smokers. Pharmacognosy Journal, 14(6).	Not RCTs
1270	Kamboj, A., Kumar, S., & Kumar, V. (2013). Evaluation of Antidiabetic Activity of Hydroalcoholic Extract of Cestrum nocturnum Leaves in Streptozotocin-Induced Diabetic Rats. Advances in Pharmacological and Pharmaceutical Sciences, 2013(1), 150401.	Not RCTs
1271	Shukla R, Anand K, Mishra KP. Cuminum cyminum attenuates hypertension via endothelial nitric oxide synthase and NO pathway in renovascular hypertensive rats. Phytomedicine. 2018;50:8-16. doi:10.1016/j.phymed.2018.08.012.	Not RCTs
1272	Kadiyala, M., Ponnusankar, S., & Elango, K. (2013). Calotropis gigantiea (L.) R. Br (Apocynaceae): a phytochemical and pharmacological review. Journal of ethnopharmacology, 150(1), 32-50.	Not RCTs

1273	Idres, A. Y., Tousch, D., Portet, K., Ferrare, K., Bidel, L. P., & Poucheret, P. An original Asteraceae-infusion able to prevent a syndrome metabolic induced in fructose rat model: Beneficial effect of a mixture of chicoric and chlorogenic acids.	Not RCTs
1274	Izzo AA, Ernst E. Interactions between herbal medicines and prescribed drugs: a systematic review. Drugs. 2001;61(15):2163-2175. doi:10.2165/00003495-200161150-00002.	Not RCTs
1275	Li D, Zhang Y, Xu D, et al. Recent findings regarding the synergistic effects of emodin and its analogs with other bioactive compounds: Insights into new mechanisms. Phytother Res. 2021;35(2):643-661. doi:10.1002/ptr.6826.	Not RCTs
1276	Hosseini, S. A., Vali, M., Haghighi-Zade, M. H., Siahpoosh, A., & Malihi, R. (2020). The effect of Chilgoza pine nut (Pinus gerardiana Wall.) on blood glucose and oxidative stress in diabetic rats. Diabetes, Metabolic Syndrome and Obesity, 2399-2408.	Not RCTs
1277	Izzo AA. Herb-drug interactions: an overview of the clinical evidence. Fundam Clin Pharmacol. 2005;19(1):1-16. doi:10.1111/j.1472-8206.2004.00277.x.	Not RCTs
1278	Holidah, D., Dewi, I. P., Pusparini, D., Rochayati, D., & Nurrizki, A. M. (2021). Effects of Passiflora edulis var. flavicarpa on liver functional parameters in alloxan induced diabetic mice. Medicinal Plants-International Journal of Phytomedicines and Related Industries, 13(3), 493-498.	Not RCTs
1279	Morgan SG, Barer ML, Agnoloni T, et al. Regional variation in the use of medications by older Canadians—a persistent and incompletely understood phenomenon. Pharmacoepidemiol Drug Saf. 2003;12(8):575-582. doi:10.1002/pds.885.	Not RCTs
1280	Yuan Y, Ding D, Zhang N, et al. Involvement of TGF-β and autophagy pathways in pathogenesis of diabetes: A comprehensive review. Front Pharmacol. 2022;13:855246. doi:10.3389/fphar.2022.855246.	Not RCTs
1281	Herdiani, N., Wirjatmadi, B., Kuntoro, K., & Zamzam, M. (2023). Effect of mangosteen skin ethanol extract on streptozotocin-induced TNF-伪 expression.聽Journal of Public Health in Africa,聽 14(2), 5.	Not RCTs
1282	Schnell O, Alawi H, Battelino T, et al. Benefits of digital tools for integrated personalized diabetes management: Results from the PDM-ProValue study program. Diabetes Technol Ther. 2017;19(S2):S139-S146. doi:10.1089/dia.2017.0033.	Not RCTs
1283	Fugh-Berman A, Ernst E. Herb-drug interactions: review and assessment of report reliability. Br J Clin Pharmacol. 2001;52(5):587-595. doi:10.1046/j.0306-5251.2001.01464.x.	Not RCTs
1284	He, L, Wang, H, He, X, Gu, C, Zhang, Y, Zhao, L and Tong, X, 2017. How to administrate placebo to patients with diabetes in RCTs of Chinese Herbal Medicine? Practicable and ethical issues, BMC Complementary and Alternative Medicine.	Not RCTs

1285	Sample size calculations: Perspectives from investigators of Chinese Herbal Medicine	Not RCTs
1286	He, K., Li, X., Chen, X., Ye, X., Huang, J., Jin, Y., ... & Shu, H. (2011). Evaluation of antidiabetic potential of selected traditional Chinese medicines in STZ-induced diabetic mice. Journal of ethnopharmacology, 137(3), 1135-1142.	Not RCTs
1287	He, F., Sun, K. W., & Peng, J. P. (2011). Study on the functions of peripheral dendritic cells in chronic hepatitis B virus infection patients of Gan-depression Pi-deficiency syndrome and Gan-Dan damp-heat syndrome under different immune states. Zhongguo Zhong xi yi jie he za zhi Zhongguo Zhongxiyi Jiehe Zazhi= Chinese Journal of Integrated Traditional and Western Medicine, 31(11), 1491-1495.	Not RCTs
1288	Hass, D. J., & Lewis, J. D. (2006). Quality of manufacturer provided information on safety and efficacy claims for dietary supplements for colonic health. Pharmacoepidemiology and drug safety, 15(8), 578-586.	Not RCTs
1289	Brown SA, Brown CA, Crowell WA, Barsanti JA, Kang CW. Beneficial effects of chronic administration of dietary antioxidants to cats with renal insufficiency. J Vet Intern Med. 1998;12(3):152-156. doi:10.1892/0891-6640(1998)012<0152:BEOCAO>2.3.CO;2.	Not RCTs
1290	Ghavami A, Derakhshanian H, Hariri M, et al. Cumin (Cuminum cyminum L.) and lipid profile: A systematic review and meta-analysis of randomized controlled trials. Phytother Res. 2021;35(2):574-585. doi:10.1002/ptr.6800.	Not RCTs
1291	Guo, J. J., Chen, Y., Du, W., Peng, H., Wang, R., Xia, Y., ... & Papadimitropoulos, E. A. (2016). Antithrombotic therapy and direct medical costs in patients with acute coronary syndrome in Shanghai, China. Value in health regional issues, 9, 93-98.	Not RCTs
1292	Methods in Medicine, C. A. M. (2023). Retracted: Mechanism of Action of Zhi Gan Cao Decoction for Atrial Fibrillation and Myocardial Fibrosis in a Mouse Model of Atrial Fibrillation: A Network Pharmacology-Based Study.	Not RCTs
1293	Fujita, H., Yamagami, T., & Ohshima, K. (2005). Effect of Touchi extract on blood lipids in hypertriglyceridemic subjects and Sprague-Dawley rats. Nutrition Research, 25(7), 681-692.	Not RCTs
1294	Guo Y, Guo T, Chen F, et al. Chinese medicine pattern differentiation and its implications for clinical practice. Chin Med. 2017;12:18. doi:10.1186/s13020-017-0136-2.	Not RCTs
1295	Lizcano F, Guzmán G. Estrogen deficiency and the origin of obesity during menopause. Biomed Res Int. 2014;2014:757461. doi:10.1155/2014/757461.	Not RCTs
1296	Wang Y, Chen L, Law HKW, et al. GPR40: A therapeutic target for mediating insulin secretion (Review). Int J Mol Med. 2014;34(5):1263-1270. doi:10.3892/ijmm.2014.1900.	Not RCTs

1297	Feng, P. F., Liu, Y., & Qin, N. P. (2001). Effect of sanwu hypotensive decoction on blood pressure and lymphokine activated killer cell in patient of primary hypertension and spontaneously hypotensive rats. Zhongguo Zhong xi yi jie he za zhi Zhongguo Zhongxiyi Jiehe Zazhi= Chinese Journal of Integrated Traditional and Western Medicine, 21(5), 342-345.	Not RCTs
1298	Feng, J., Zhou, Y., Liao, L., Yu, L., Yuan, P., & Zhang, J. (2022). Network pharmacology and transcriptomics reveal the mechanism of GuaLouQuMaiWan in treatment of type 2 diabetes and its active small molecular compound. Journal of Diabetes Research, 2022(1), 2736504.	Not RCTs
1299	Shafiee-Nick R, Derakhshanian H, Ghavami A, et al. Cytotoxic effect of Thymus caramanicus Jalas on human oral epidermoid carcinoma KB cells. Adv Pharm Bull. 2017;7(1):147-153. doi:10.15171/apb.2017.018.	Not RCTs
1300	Wu LY, Juan CC, Ho LT, Hsu YP, Hwang LS. Green tea supplementation ameliorates insulin resistance and increases glucose transporter IV content in a fructose-fed rat model. Eur J Nutr. 2004;43(2):116-124. doi:10.1007/s00394-004-0454-2.	Not RCTs
1301	Emmert-Aronson, B., Grill, K. B., Trivedi, Z., Markle, E. A., & Chen, S. (2019). Group medical visits 2.0: the open source wellness behavioral pharmacy model. The Journal of Alternative and Complementary Medicine, 25(10), 1026-1034.	Not RCTs
1302	Kemper KJ, Vohra S. Evidence-based complementary and alternative medicine in child and adolescent psychiatry: Research challenges and opportunities. Child Adolesc Psychiatr Clin N Am. 2005;14(4):617-640. doi:10.1016/j.chc.2005.05.004.	Not RCTs
1303	Dresner, D., Gergen Barnett, K., Resnick, K., Laird, L. D., & Gardiner, P. (2016). Listening to their words: a qualitative analysis of integrative medicine group visits in an urban underserved medical setting. Pain Medicine, 17(6), 1183-1191.	Not RCTs
1304	Saikia P, Ryakala VK, Deka DC. Oroxylum indicum (L.) Kurz, an important Asian traditional medicine: From traditional uses to scientific data for its commercial exploitation. J Ethnopharmacol. 2021;265:113305. doi:10.1016/j.jep.2020.113305.	Not RCTs
1305	Simon JA, Kaunitz AM, Archer DF, et al. Treatment and resource utilization for menopausal symptoms: a retrospective review of US electronic health records. Menopause. 2021;28(8):902-910. doi:10.1097/GME.0000000000001802.	Not RCTs
1306	Zhao Y, Jiang Z, Zhao T, et al. Reversal of type 1 diabetes via islet β cell regeneration following immune modulation by cord blood-derived multipotent stem cells. BMC Med. 2012;10:3. doi:10.1186/1741-7015-10-3.	Not RCTs
1307	Ernest E. Herbal medicines: adverse effects and drug–herb interactions. Br J Anaesth. 2003;91(3):375-382. doi:10.1093/bja/aeg202.	Not RCTs
1308	de-Graft Aikins A. Healer shopping in Africa: new evidence from a rural-urban qualitative study of Ghanaian diabetes experiences. BMJ.	Not RCTs

	2005;331(7519):737. doi:10.1136/bmj.331.7519.737.	
1309	de Araujo, C. H. M., Nogueira, D., de Araujo, M. C. P. M., de Paula Martins, W., Ferriani, R. A., & dos Reis, R. M. (2009). Supplemented tissue culture medium 199 is a better medium for in vitro maturation of oocytes from women with polycystic ovary syndrome women than human tubal fluid. Fertility and sterility, 91(2), 509-513.	Not RCTs
1310	Seid MA, Zegeye DT, Wassie MM, et al. A nurse-led, community-based self-management program for people with type 2 diabetes in western Ethiopia: A feasibility and pilot study protocol. BMJ Open. 2022;12(2):e055312. doi:10.1136/bmjopen-2021-055312.	Not RCTs
1311	Lam CSP, Teng THK, Tay WT, et al. Regional and ethnic differences among patients with heart failure in Asia: the Asian Heart Failure Registry. Eur Heart J. 2016;37(41):3141-3153. doi:10.1093/eurheartj/ehw331.	Not RCTs
1312	Ernst E. The role of complementary and alternative medicine. BMJ. 2000;321(7269):1133-1135. doi:10.1136/bmj.321.7269.1133.	Not RCTs
1313	Ciumasu-Rimbu, M., Livia, P. O. P. A., & Vulpoi, C. (2012). NEUROPEPTIDE Y STIMULATION AS PRIMARY TARGET FOR PRE-VENTIVE MEASURES OF MALADAPTATIVE CARDIOVASCULAR RE-ACTIONS IN OCCUPATIONAL CHRONIC STRESS EXPOSURE. The Medical-Surgical Journal, 116(3), 790-793.	Not RCTs
1314	Chisholm, C. D., Cordell, W. H., Rogers, K., & Woods, J. R. (1992). Comparison of a new pressurized saline canister versus syringe irrigation for laceration cleansing in the emergency department. Annals of emergency medicine, 21(11), 1364-1367.	Not RCTs
1315	Zhang L, Zhang Y, Wang S, et al. Methodological quality of systematic reviews on Chinese herbal medicine: A survey. BMC Complement Altern Med. 2016;16:266. doi:10.1186/s12906-016-1253-3.	Not RCTs
1316	Chen, Z., Ye, S. Y., & Zhu, R. G. (2020). The extraordinary transformation of traditional Chinese medicine: processing with liquid excipients. Pharmaceutical Biology, 58(1), 561-573.	Not RCTs
1317	Chen, Y., Chen, Z., Wang, X., Xiong, H., Shuang, F., & Liu, X. (2022). Influencing factors of nucleic acid negative conversion in patients with mild and common COVID-19 induced by the Omicron variant of SARS-COV-2. Zhejiang da xue xue bao. Yi xue ban= Journal of Zhejiang University. Medical Sciences, 51(6), 731-737.	Not RCTs
1318	Zheng J, Wu W, Hu B, et al. Protective effects and mechanisms of opuntia polysaccharide in animal models of diabetes mellitus: A systematic review and meta-analysis. Front Pharmacol. 2022;13:937660. doi:10.3389/fphar.2022.937660.	Not RCTs

1319	Zhang S, Yang L, Zhang L, et al. Effects of Erchen decoction on oxidative stress-related cytochrome P450 metabolites of arachidonic acid in dyslipidemic mice: A randomized, controlled trial. Front Pharmacol. 2022;13:926095. doi:10.3389/fphar.2022.926095.	Not RCTs
1320	Charles-de-Sá, L., Gontijo de Amorim, N. F., Dantas, D., Han, J. V., Amable, P., Teixeira, M. V. T., ... & Rigotti, G. (2015). Influence of negative pressure on the viability of adipocytes and mesenchymal stem cell, considering the device method used to harvest fat tissue. Aesthetic Surgery Journal, 35(3), 334-344.	Not RCTs
1321	Niture NT, Ansari AA, Naik SR. Gallic acid ameliorated impaired lipid homeostasis in a mouse model of high-fat diet–and streptozotocin-induced NAFLD and diabetes through improvement of β-oxidation and ketogenesis. RSC Adv. 2020;10(37):22221-22235. doi:10.1039/D0RA03113A.	Not RCTs
1322	Bauer, M., & Rausch, H. (2011). Quality Assurance as Basis for the Implementation of TCM into the Western Medicinal System− A Case Study of Prunella vulgaris. Onco Therapeutics, 2(4).	Not RCTs
1323	Lim WY, Chia SY, Koong AYL, et al. Public perceptions, knowledge and awareness of cholesterol management in Singapore: A prospective pilot study. Singapore Med J. 2020;61(10):509-514. doi:10.11622/smedj.2020140.	Not RCTs
1324	Atalla, A. (2021). In vivo, Lipid Profile Efficacy of Ethanol Extracts of Stevia rebaudiana Bertoni in Rabbits. Pakistan journal of biological sciences: PJBS, 24(2), 292-296.	Not RCTs
1325	Arokoyo, D. S., P Oyeyipo, I., S DU Plessis, S., N Chegou, N., & G Aboua, Y. (2017). Reproductive parameters in streptozotocin-induced diabetic male wistar rats: beneficial role of basella alba aqueous leave extract. Journal of Kerman University of Medical Sciences, 24(6), 467-479.	Not RCTs
1326	Frenkel MA, Cohen L, Peterson N, et al. Integrative medicine in lung cancer: challenges and hope. Chest. 2013;143(6):1691-1699. doi:10.1378/chest.12-2264.	Not RCTs
1327	Nasri H, Shirzad H, Baradaran A, Rafieian-Kopaei M. New concepts in nutraceuticals as alternative for pharmaceuticals. Int J Prev Med. 2014;5(12):1487-1499.	Not RCTs
1328	Reutter LI, Northcott HC. Women's experience of social support and its impact on their health. Health Care Women Int. 1993;14(4):377-388. doi:10.1080/07399339309516046.	Not RCTs
1329	Amare, Y. E., Dires, K., & Asfaw, T. (2022). Antidiabetic Activity of Mung Bean or Vigna radiata (L.) Wilczek Seeds in Alloxan-Induced Diabetic Mice. Evidence-Based Complementary and Alternative Medicine, 2022(1), 6990263.	Not RCTs
1330	Akbarzadeh, S., Ostovar, A., Angali, N., Abbasifard, A., & Chashmpoosh, M. (2019). Effects of hydroalcoholic extract of thymus vulgaris on serum levels lipoprotein lipase and angiopoietin-like protein 4 in hyperlipidemic rats. Journal of Mazandaran University of Medical Sciences, 28(170), 22-32.	Not RCTs

1331	Ahangarpour, A., Heidari, H., Junghani, M. S., Absari, R., Khoogar, M., & Ghaedi, E. (2017). Effects of hydroalcoholic extract of Rhus coriaria seed on glucose and insulin related biomarkers, lipid profile, and hepatic enzymes in nicotinamide-streptozotocin-induced type II diabetic male mice. Research in pharmaceutical sciences, 12(5), 416-424.	Not RCTs
1332	Barati M, Sadati SA, Shahbazi S, et al. Effect of Beta vulgaris extract on liver enzymes in patients with nonalcoholic fatty liver disease: a randomized clinical trial. Phytother Res. 2020;34(8):2086-2092. doi:10.1002/ptr.6671.	Not RCTs
1333	Adejor, E. B., Ameh, D. A., James, D. B., Owolabi, O. A., & Ndidi, U. S. (2017). Effects of Garcinia kola biflavonoid fractions on serum lipid profile and kidney function parameters in hyperlipidemic rats. Clinical Phytoscience, 2, 1-8.	Not RCTs
1334	朱亮,王娜娜,吴承玉.南京市六合地区农村老年高血压病患者中医体质流行病学调查[J].中国临床研究,2011,24(05):441-442.	Not RCTs
1335	孟帅. (2013). 张洪义教授治疗慢性湿疹验案举隅. 国际中医中药杂志, 35(1), 92-92.	Not RCTs
1336	孟庆扬.马建伟运用异病同治法治疗代谢病验案举隅[J].环球中医药,2015,8(03):371-373.	Not RCTs
1337	马丽娜·阿新拜.代谢综合征与缺血性脑卒中关系及中医证候要素探讨[D].北京中医药大学,2016.DOI:10.26973/d.cnki.gbjzu.2016.000140.	Not RCTs
1338	罗辉.中医体质学体病相关临床研究的系统评价和方法学研究[D].北京中医药大学,2019.DOI:10.26973/d.cnki.gbjzu.2019.000012.	Not RCTs
1339	刘迎辉,潘博涵.引血下行化痰逐瘀降压牛膝方对痰湿壅盛高血压大鼠尾动脉血压、血管紧张素II影响随机平行对照研究[J].实用中医内科杂志,2015,29(11):162-164.DOI:10.13729/j.issn.1671-7813.2015.11.73.	Not RCTs
1340	刘英,曹祖清,喻闽凤,等.赵纪生教授治疗糖尿病肾病合并慢性肾功能不全经验[J].中国中医药现代远程教育,2019,17(11):36-39.	Not RCTs
1341	梁婧,周恩超.周恩超治疗糖尿病肾病药对举隅[J].浙江中医药大学学报,2015,39(12):883-885.DOI:10.16466/j.issn1005-5509.2015.12.011.	Not RCTs
1342	李敏,杨明会,刘毅.丹参及其复方对大鼠肺损伤肺AQP1 的调节作用[J].中国中医急症,2009,18(01):90-92.	Not RCTs
1343	喇孝瑾,张俊梅,韩淑,等.复方姜黄胶囊对实验性大鼠降糖降血脂作用的实验研究[J].中国煤炭工业医学杂志,2010,13(09):1367-1368.	Not RCTs
1344	贾宁,张娟.张娟教授治疗老年糖尿病性便秘的经验探讨[J].浙江中医药大学学报,2015,39(10):750-752.DOI:10.16466/j.issn1005-5509.2015.10.010.	Not RCTs
1345	黄湘茜.基于循证证据和德尔菲法的针灸干预糖尿病前期临床诊疗方案制定研究[D].湖北中医药大学,2021.DOI:10.27134/d.cnki.ghbzc.2021.000084.	Not RCTs
1346	黄国榕,林震群,张毅娜,等.大肠息肉患者的中医体质特点及相关因素分析[J].光明中医,2021,36(19):3214-3217.	Not RCTs
1347	黄宝英,林应华,王芳,等.养阴固精化痰渗湿方联合贝那普利治疗早期糖尿病肾病大鼠的研究[J].现代中西医结合杂志,2011,20(03):278-281.	Not RCTs

1348	黄宝英,林应华,富显果,等.养阴固精化痰渗湿方联合贝那普利对早期糖尿病肾病大鼠的保护作用[J].中国实验方剂学杂志,2011,17(12):202-206.DOI:10.13422/j.cnki.syfjx.2011.12.022.	Not RCTs
1349	郭志伟,王久玉,张铜河,等.王延丰主任中医师治疗糖尿病肾病经验[J].中国中医药现代远程教育,2021,19(14):73-74.	Not RCTs
1350	付丽媛,杨进,王灿晖,等.健脾化湿养阴清胃方药对 2 型糖尿病肾病大鼠一氧化氮及内皮素-1 的影响[J].时珍国医国药,2014,25(04):838-840.	Not RCTs
1351	丁娜,都广礼.不同黄连药对配伍对 2 型糖尿病大鼠血糖的影响[J].山西中医学院学报,2012,13(03):29-30.	Not RCTs
1352	崔莉芳,庆慧,程广书.邱保国研究员胸痹从血瘀论治 6 法[J].中医研究,2013,26(07):51-53.	Not RCTs
1353	郝俊岭.半夏白术天麻汤对痰湿壅盛型高血压大鼠血压及血清MDA、SOD、GSH-Px水平的影响[D].内蒙古医科大学,2019.DOI:10.27231/d.cnki.gnmyc.2019.000159.	Not RCTs
1354	卞华,吕芹,胡久略,等.益气养阴化浊通络方对糖尿病肾病大鼠转化生长因子-β1/Smads信号通路的影响[J].中国老年学杂志,2015,35(16):4432-4434.	Not RCTs
1355	Ness RB. Abraham Lilienfeld Award address: 2017 Annual Meeting of the American College of Epidemiology. Ann Epidemiol. 2018;28(1):61-62. doi:10.1016/j.annepidem.2017.11.001	Not RCTs
1356	Zhang Y, Liu M, Sun Y, et al. Expert consensus on Wenxin Granule for treatment of cardiac arrhythmias. Chin J Integr Med. 2020;26(5):357-364. doi:10.1007/s11655-020-3260-8.	Not RCTs
1357	Klatt, M., Huerta, T., Gascon, G., Sieck, C., & Malarkey, W. (2016). The international congress on integrative medicine and health (ICIMH). J Altern Complement Med, 22(6), A1-A142.	Not RCTs
1358	Lavigne GJ, Sessle BJ. Canadian Orofacial Pain Team workshop report on the global year against orofacial pain. Pain Res Manag. 2015;20(1):7-14. doi:10.1155/2015/785692	Not RCTs
1359	金子泰久. (2012). 2012 International Research Congress on Integrative Medicine and Health 参加報告. 全日本鍼灸学会雑誌, 62(3), 235-244.	Not RCTs
1360	Lavigne GJ, Sessle BJ. Canadian Orofacial Pain Team workshop report on the global year against orofacial pain. Pain Res Manag. 2015;20(1):7-14. doi:10.1155/2015/785692	Not RCTs
1361	PROVINCE, C. W. O. B. 2 nd National Congress on Medicinal Plants 15, 16 May 2013 Tehran-Iran.	Not RCTs
1362	Eastman, A., Boudreau, M., Raskin, I., Lila, M. A., Bazinet, L., Saxena, P. K., ... & Duncan, A. M. (2010). 7th NHP Research Conference, Halifax, Nova Scotia, May 23rd-26th, 2010: The Next Wave. Pharmaceutical Biology, 48(S1), 1-24.	Not RCTs
1363	Erdoes G, Vuylsteke A, Schreiber JU, et al. European Association of Cardiothoracic Anesthesiology (EACTA) Cardiothoracic and Vascular Anesthesia Fellowship Curriculum: First Edition. J Cardiothorac Vasc Anesth. 2020;34(5):1132-1141. doi:10.1053/j.jvca.2019.12.014IF: 2.3 Q2	Not RCTs

136<br>4	Guanjie, F, Lin, Y, Paul, SK, et al., 2020. A randomised control trial to evaluate the efficacy of Chinese traditional medicine for the treatment of diabetic peripheral neuropathy, Diabetes.	Not clinical metabolic diseases
136<br>5	Lacy BE, Patel NK. Rome criteria and a diagnostic approach to irritable bowel syndrome. J Clin Med. 2017;6(11):99. doi:10.3390/jcm6110099.	Not clinical metabolic diseases
136<br>6	Sobenin, I. A., Prianishnikov, V. V., Kunnova, L. M., Rabinovich, E. A., & Orekhov, A. N. (2005). Allicor efficacy in lowering the risk of ischemic heart disease in primary prophylaxis. Terapevticheskii Arkhiv, 77(12), 9-13.	Not clinical metabolic diseases
136<br>7	Singh, B. B., Vinjamury, S. P., Der-Martirosian, C., Kubic, E., Mishra, L. C., Shepard, N. P., ... & Madhu, S. G. (2007). Ayurvedic and collateral herbal treatments for hyperlipidemia: a systematic review of randomized controlled trials and quasi-experimental designs. Database of Abstracts of Reviews of Effects (DARE): Quality-assessed Reviews [Internet].	Not clinical metabolic diseases
136<br>8	Xu, J. Y., & Gao, H. Y. (2018). Combined treatment of traditional Chinese medicine and western medicine in the treatment of diabetic peripheral neuropathy. Journal of Biological Regulators and Homeostatic Agents, 32(4), 945-949.	Not clinical metabolic diseases
136<br>9	Wa Chan, K., Siu Kei Kwong, A., Nang Tsui, P., Fai Choi, W., Han Yiu, W., Beng Kathryn Tan, C., & Tang, S. (2022). MO621: effectiveness of adjuvant astragalus for diabetic kidney disease: interim analysis of a pragmatic randomised controlled trial. Nephrology Dialysis Transplantation, 37(Supplement_3), gfac076-014.	Not clinical metabolic diseases
137<br>0	Kaatabi H, Bamosa AO, Badar A, et al. Nigella sativa improves lipid profile in hyperlipidemic patients: a randomized, double-blind, placebo-controlled clinical trial. Phytother Res. 2013;27(5):643-648. doi:10.1002/ptr.4789.	Not clinical metabolic diseases
137<br>1	Purandare, H., & Supe, A. (2007). Immunomodulatory role of Tinospora cordifolia as an adjuvant in surgical treatment of diabetic foot ulcers: a prospective randomized controlled study.	Not clinical metabolic diseases
137<br>2	Tick H, Nielsen A, Pelletier KR, et al. Evidence-based nonpharmacologic strategies for comprehensive pain care: The consortium pain task force white paper. Explore (NY). 2018;14(3):177-211. doi:10.1016/j.explore.2018.02.001.	Not clinical metabolic diseases
137<br>3	Kaunitz AM. Menopause: Counseling and Hormone Therapy. Clin Obstet Gynecol. 2015;58(4):889-900. doi:10.1097/GRF.0000000000000146.	Not clinical metabolic diseases
137<br>4	Ahmadian, R., Bahramsoltani, R., Marques, A. M., Rahimi, R., & Farzaei, M. H. (2021). Medicinal Plants as Efficacious Agents for Diabetic Foot Ulcers: A Systematic Review of Clinical Studies. Wounds: a compendium of clinical research and practice, 33(8), 207-218.	Not clinical metabolic diseases

137<br>5	Hoseinynejad, K., Amini, F., Hasanloo, E., Shayanpour, S., Pouladzadeh, M., & Nazer, M. (2022). Phytotherapy in Renal Failure Due to Blood Pressure and Diabetes: A Systematic Review Study in Iram Ethnobotanical Documents. Indian Journal of Forensic Medicine & Toxicology, 16(1).	Not clinical metabolic diseases
137<br>6	苏文龙. (2003). 消渴除痹汤治糖尿病性周围神经病变 34 例观察. 中华实用中西医杂志, 16(013), 1873-1874.	Not clinical metabolic diseases
137<br>7	孙艳, & 陶少平. (2006). 糖尿病足 34 例临床分析和治疗体会. 实用全科医学, 4(1), 19-20.	Not clinical metabolic diseases
137<br>8	Punukollu, R. S., Chadalawada, A. K., Siddabattuni, K., & Gogineni, N. T. (2024). A blend of Withania somnifera (L.) Dunal root and Abelmoschus esculentus (L.) Moench fruit extracts relieves constipation and improves bowel function: a proof-of-concept clinical investigation. Journal of Ethnopharmacology, 318, 116997.	Not clinical metabolic diseases
137<br>9	Velmurugan, C., Sundaram, T., Sampath Kumar, R., Vivek, B., Sheshadrishekar, D., & Ashok Kumar, B. S. (2011). Anti diabetic and hypolipidemic activity of bark of ethanolic extract of ougeinia oojeinensis (ROXB.). Med J Malaysia, 66(1), 23.	Not clinical metabolic diseases
138<br>0	SUNJONO, T. A., & RAHMAN, A. (2020). Anti-Hypercholesterolemic Activity of Ethnolic Extract of Gomphrena globosa Flowers and its Phytochemical Screening. International Journal of Pharmaceutical Research (09752366), 12(1).	Not clinical metabolic diseases
138<br>1	Silva AS, Varela SD, Lima WG, et al. Bioactive natural products against systemic arterial hypertension: A past 20-year systematic and prospective review. Phytomedicine. 2020;77:153291. doi:10.1016/j.phymed.2020.153291.	Not clinical metabolic diseases
138<br>2	Jemai H, Feki AE, Sayadi S. Antidiabetic and antioxidant effects of hydroxytyrosol and oleuropein from olive leaves in alloxan-induced diabetic rats. J Agric Food Chem. 2009;57(19):8798-8804. doi:10.1021/jf901280u.	Not clinical metabolic diseases
138<br>3	Cargnin ST, Gnoatto SB. Phytotherapy for pain relief: a review of the current literature. Phytother Res. 2017;31(9):1345-1367. doi:10.1002/ptr.5884.	Not clinical metabolic diseases
138<br>4	Mani Saminathan, M. S., Rai, R. B., Kuldeep Dhama, K. D., Ruchi Tiwari, R. T., Sandip Chakraborty, S. C., Amarpal, A., ... & Kandasamy Kannan, K. K. (2013). Systematic review on anticancer potential and other health beneficial pharmacological activities of novel medicinal plant Morinda citrifolia (Noni).	Not clinical metabolic diseases
138<br>5	Mei, LJ, Xiong, SQ and Wang, T, 2012. [A case control study of influential factors or the Han population with coronary heart disease of blood stasis syndrome in Fuzhou area], Zhongguo Zhong Xi Yi Jie He Za Zhi	Not clinical metabolic diseases

1386	Wu, L. Q., Xiong, C. Q., Wu, M., Dong, R. L., Chen, Y. Q., Gao, J., ... & Huang, Y. P. (2008). Clinical characteristics of 7 patients with gestational diabetes insipidus. Zhonghua fu Chan ke za zhi, 43(4), 266-268.	Not clinical metabolic diseases
1387	Zheng, J., Lin, S. Z., & Chen, X. L. (2011). Impacts of yishen jiangzhuo granule on B lymphocytes and regulatory T-lymphocytes in patients with chronic renal insufficiency. Zhongguo Zhong xi yi jie he za zhi Zhongguo Zhongxiyi Jiehe Zazhi= Chinese Journal of Integrated Traditional and Western Medicine, 31(1), 37-40.	Not clinical metabolic diseases
1388	Shi, Y. M., & Wu, Q. Z. (1992). Long-term effect of Schonlein-Henoch nephritis with nephritic-nephrotic syndrome in children by traditional Chinese medicine and Western medicine. Zhongguo Zhong xi yi jie he za zhi Zhongguo Zhongxiyi Jiehe Zazhi= Chinese Journal of Integrated Traditional and Western Medicine, 12(6), 340-2.	Not clinical metabolic diseases
1389	Yang, H. M., Meng, X. J., Wu, W., Liu, Y. L., & Zhai, X. J. (2017). Regression analysis of serum bone metabolic markers and traditional Chinese medicine syndromes in patients with CKD-MBD. Zhongguo Zhong yao za zhi= Zhongguo Zhongyao Zazhi= China Journal of Chinese Materia Medica, 42(20), 4027-4034.	Not clinical metabolic diseases
1390	赵玉玲,陶春晖,邱明义."补下清上"法治疗脑鸣伴黄斑水肿验案 1 则[J].江苏中医药,2018,50(07):47-48.	Not clinical metabolic diseases
1391	谢明星,许力华,马秋华,等.1%吡美莫司乳膏联合四妙丸治疗阴囊湿疹 53 例[J].浙江中医杂志 ,2014,49(02):118.DOI:10.13633/j.cnki.zjtcm.2014.02.053.	Not clinical metabolic diseases
1392	尚娥,周秀芳,王娇,等.1 例复方黄柏液+TDP治疗老年肥胖女性外阴部严重感染[J].中国老年保健医学,2013,11(05):91.	Not clinical metabolic diseases
1393	赵兰芬,曹猛,陈鸿.1 例静脉滴注红花注射液发生过敏性休克的护理[J].中国中医药现代远程教育,2011,9(17):42.	Not clinical metabolic diseases
1394	唐海倩,殷建美,晏蔚田,等.730 例难治性痛风患者中医证候和临床特征分析[J].云南中医学院学报 ,2021,44(03):39-43+51.DOI:10.19288/j.cnki.issn.1000-2723.2021.03.008.	Not clinical metabolic diseases
1395	Ley B, Ryerson CJ, Vittinghoff E, et al. A multidimensional index and staging system for idiopathic pulmonary fibrosis. Ann Intern Med. 2012;156(10):684-691. doi:10.7326/0003-4819-156-10-201205150-00004.	Not clinical metabolic diseases
1396	An Y, Sun Z, Li C, et al. Clinical study on short-term effects of berberine in comparison to metformin on the metabolic characteristics of women with PCOS. Eur J Endocrinol. 2020;183(6):757-767. doi:10.1530/EJE-20-0595.	Not clinical metabolic diseases
1397	Yao, Q., Zhai, H., Huang, H., Lin, J., & He, W. (2022). A comparative study of the efficacy of tamoxifen and Chinese patented medicine (Pingxiao capsules) in gynecomastia: A retrospective cohort study. Andrologia, 54(11),	Not clinical metabolic diseases

	e14640.	
1398	Moini Jazani, A., Nasimi Doost Azgomi, H., Nasimi Doost Azgomi, A., & Nasimi Doost Azgomi, R. (2019). A comprehensive review of clinical studies with herbal medicine on polycystic ovary syndrome (PCOS). DARU Journal of Pharmaceutical Sciences, 27, 863-877.	Not clinical metabolic diseases
1399	Doll R, Hill ID, Kang J, et al. Controlled trial of carbenoxolone in the treatment of duodenal ulcer. Gut. 1965;6(5):464-468. doi:10.1136/gut.6.5.464.	Not clinical metabolic diseases
1400	Wal, A., Wal, P., Saraswat, N., & Wadhwa, S. (2021). A detailed review on herbal treatments for treatment of PCOS-Polycystic ovary syndrome (PCOS). Curr Nutraceuticals, 2(3), 192-202.	Not clinical metabolic diseases
1401	Ramalingam S, Subramanian M, Rangarajan P, et al. A double blinded placebo controlled clinical trial on Siddha medicines for COVID-19 management: study protocol. Trials. 2021;22(1):50. doi:10.1186/s13063-020-04965-1.	Not clinical metabolic diseases
1402	Ye, Z., Huang, Q., She, Y., Hu, Y., Wu, M., Qin, K., ... & Ye, Q. (2022). A meritorious integrated medical regimen for hepatic fibrosis and its complications via the systematic review and meta-analysis for Dahuang Zhechong pill-based therapy. Frontiers in Medicine, 9, 920062.	Not clinical metabolic diseases
1403	Cho ML, Heo YJ, Park JS, et al. A multicenter, randomized, double-blind, comparative clinical study of GCSB-5 and celecoxib in patients with osteoarthritis of the knee. Clin Ther. 2015;37(6):1281-1293.e4. doi:10.1016/j.clinthera.2015.03.010.	Not clinical metabolic diseases
1404	Nonaka T, Kessoku T, Ogawa Y, et al. Rikkunshito for PPI-refractory non-erosive reflux disease: a randomized controlled trial. Neurogastroenterol Motil. 2017;29(5):e12918. doi:10.1111/nmo.12918.	Not clinical metabolic diseases
1405	Wu, L., Lai, J., Ling, Y., Weng, Y., Zhou, S., Wu, S., ... & Chen, Y. (2021). A review of the current practice of diagnosis and treatment of idiopathic membranous nephropathy in China. Medical Science Monitor: International Medical Journal of Experimental and Clinical Research, 27, e930097-1.	Not clinical metabolic diseases
1406	Rajawat, J., & Banerjee, M. (2023). A Review on Therapeutic Potential of Indian Herbal Plants to Counter Viral Infection and Disease Pathogenesis. Current Traditional Medicine, 9(6), 136-144.	Not clinical metabolic diseases
1407	Namiki, T., Takayama, S., Arita, R., Ishii, T., Kainuma, M., Makino, T., ... & Ito, T. (2021). A structured summary of a study protocol for a multi-center, randomized controlled trial (RCT) of COVID-19 prevention with Kampo medicines (Integrative Management in Japan for Epidemic Disease by prophylactic study: IMJEDI P1 study). Trials, 22, 1-3.	Not clinical metabolic diseases
1408	Rakhshandeh H, Hosseini M, Soleimani M. Rosa damascena as a medicinal plant in clinical studies: A systematic review. J Integr Med. 2021;19(2):108-118. doi:10.1016/j.joim.2020.11.002.	Not clinical metabolic diseases

1409	Zhang, X., Wang, Q., Dong, Y., Jia, Y., Hou, Z., Deng, W., ... & Jia, H. (2022). Acupuncture-assisted anaesthesia for catheter ablation of atrial fibrillation to reduce the consumption of morphine hydrochloride and postoperative nausea and vomiting (PONV): study protocol for a randomised controlled trial. BMJ open, 12(12), e068318.	Not clinical metabolic diseases
1410	Xu, N., Zhong, K., Yu, H., Shu, Z., Chang, K., Zheng, Q., ... & Li, J. (2023). Add-on Chinese medicine for hospitalized chronic obstructive pulmonary disease (CHOP): A cohort study of hospital registry. Phytomedicine, 109, 154586.	Not clinical metabolic diseases
1411	Dongmei, X., Junhua, Z., Mingjun, Z., Yu, Z., & Hongcai, S. (2017). Adherence reporting in clinical trials of type 2 Diabetes Metellius in the field of Traditional Chinese Medicine. Journal of Traditional Chinese Medicine, 37(1), 140-142.	Not clinical metabolic diseases
1412	Liu W, Tian Y, Zhang X, et al. Adjunctive use of modified Yunu-Jian in male smokers with chronic periodontitis: a randomized double-blind, placebo-controlled trial. Phytomedicine. 2020;68:153180. doi:10.1016/j.phymed.2019.153180.	Not clinical metabolic diseases
1413	Yuan, J., Abdurahman, A., Cui, N., Hao, T., Zou, J., Liu, L., & Wu, Y. (2023). Adjuvant therapy with Huatan Sanjie Granules improves the prognosis of patients with primary liver cancer: a cohort study and the investigation of its mechanism of action based on network pharmacology. Frontiers in Pharmacology, 14, 1091177.	Not clinical metabolic diseases
1414	de Groot AC. Side-effects of henna and jagua tattoos: a critical review. Contact Dermatitis. 2013;68(1):1-11. doi:10.1111/cod.12021.	Not clinical metabolic diseases
1415	Van Steenkiste, F., Fidèle, S., Nsanzabaganwa, W., Uwacu, B., Dedeken, P., Teuwen, D. E., & Boon, P. (2019). An ambispective cohort study on treatment outcomes of patients with epilepsy in a tertiary epilepsy center in Rwanda and recommendations for improved epilepsy care. Epilepsia Open, 4(1), 123-132.	Not clinical metabolic diseases
1416	Li Y, Wang H, Chen J, et al. Development and validation of a prognostic model for cancer patients with COVID-19: a multicentre study. J Immunother Cancer. 2020;8(2):e001314. doi:10.1136/jitc-2020-001314.	Not clinical metabolic diseases
1417	Elsharkawy H, El-Boghdadly K, Barrington M. Quadratus lumborum block: anatomical concepts, mechanisms, and techniques. Anesthesiology. 2019;130(2):322-335. doi:10.1097/ALN.0000000000002524.	Not clinical metabolic diseases
1418	Ong WY, Stohler CS, Herr DR. Role of the prefrontal cortex in pain processing. Mol Neurobiol. 2019;56(2):1137-1166. doi:10.1007/s12035-018-1130-9.	Not clinical metabolic diseases
1419	Zhang JJY, Lee KS, Ang LW, et al. Risk factors for severe disease and efficacy of treatment in patients with COVID-19: a retrospective cohort study. Lancet Infect Dis. 2020;20(7):791-798. doi:10.1016/S1473-3099(20)30250-2.	Not clinical metabolic diseases

1420	王萍萍. (2017). ANCA 相关性血管炎合并类风湿关节炎临床分析 (Master's thesis，吉林大学).	Not clinical metabolic diseases
1421	Kwon B, Kim DH, Marvin A, et al. Anterior cervical discectomy and fusion with and without plate instrumentation for multilevel degenerative disease. Spine (Phila Pa 1976). 2006;31(11):1217-1223. doi:10.1097/01.brs.0000217660.18108.4b.	Not clinical metabolic diseases
1422	Norman G, Dumville JC, Mohapatra DP, et al. Antibiotics and antiseptics for surgical wounds healing by secondary intention. Cochrane Database Syst Rev. 2016;(3):CD011712. doi:10.1002/14651858.CD011712.pub2.	Not clinical metabolic diseases
1423	Xiu LiJuan, X. L., Sun DaZhi, S. D., Jiao JianPeng, J. J., Yan Bing, Y. B., Qin ZhiFeng, Q. Z., Liu Xuan, L. X., ... & Yue XiaoQiang, Y. X. (2015). Anticancer effects of traditional Chinese herbs with phlegm-eliminating properties-an overview.	Not clinical metabolic diseases
1424	Avcin T, Cimaz R, Silverman ED, et al. Pediatric antiphospholipid syndrome: Clinical and immunologic features of 121 patients in an international registry. Pediatrics. 2008;122(5):e1100-e1107. doi:10.1542/peds.2008-0082.	Not clinical metabolic diseases
1425	Su, S. H., Lai, P. F., Yu, H. Y., Chen, K. C., Wu, K., Huang, C. K., ... & Ho, T. J. (2022). Application of acupuncture in the emergency department for patients with ileus: A pilot prospective cohort clinical study. Medicine, 101(43), e31245.	Not clinical metabolic diseases
1426	Huang CY, Lai JN, Wu MY, et al. Chinese herbal medicine therapy and the risk of chronic kidney disease in gout patients: a nationwide population-based cohort study. BMJ Open. 2020;10(9):e036063. doi:10.1136/bmjopen-2019-036063.	Not clinical metabolic diseases
1427	Duan C, Xia W, Zheng C, et al. Association between use of Qingfei Paidu Tang and mortality in hospitalized patients with COVID-19: A national retrospective registry study. Phytomedicine. 2021;85:153531. doi:10.1016/j.phymed.2020.153531.	Not clinical metabolic diseases
1428	Siddiqi HK, Mehra MR. COVID-19 illness in native and immunosuppressed states: a clinical–therapeutic staging proposal. J Heart Lung Transplant. 2020;39(5):405-407. doi:10.1016/j.healun.2020.03.012.	Not clinical metabolic diseases
1429	Shankar, P., Vijay, B., Pendse, N., Rahman, M., & Nampoothiri, V. (2023). Ayurveda management of pulmonary mycosis: an integrative approach: a case report. Journal of Medical Case Reports, 17(1), 48.	Not clinical metabolic diseases
1430	Panda, AK, 2018. Ayurveda treatment outcome for the correction of thrombocytopenia in alcoholic decompensate cirrhosis: Case series, Hepatology International.	Not clinical metabolic diseases
1431	Ekers D, Webster L, Van Straten A, et al. Behavioural activation for depression; an update of meta-analysis of effectiveness and sub group analysis. PLoS One. 2014;9(6):e100100. doi:10.1371/journal.pone.0100100.	Not clinical metabolic diseases

1432	Zeng YP, Xu SZ, Wei YJ, et al. Beneficial effects and safety of corticosteroids combined with traditional Chinese medicine for pemphigus: a systematic review. Chin J Integr Med. 2019;25(3):225-233. doi:10.1007/s11655-018-3002-7.	Not clinical metabolic diseases
1433	Yang, L., Yang, N., Huang, H., Yu, J., Sui, X., Tao, L., ... & Liu, Z. (2023). Bioinformatics Analysis to Identify Intersection Genes, Associated Pathways and Therapeutic Drugs between COVID-19 and Oral Candidiasis. Combinatorial Chemistry & High Throughput Screening, 26(8), 1533-1546.	Not clinical metabolic diseases
1434	Zhou, S., Qiao, J., Bai, J., Wu, Y., & Fang, H. (2018). Biological therapy of traditional therapy-resistant adult-onset Still's disease: an evidence-based review. Therapeutics and clinical risk management, 167-171.	Not clinical metabolic diseases
1435	Zhang, X. F., Ma, S. Q., Wu, S. Z., Yang, Z. P., & Chen, Q. (2012). Bundle program of treatment for acute severe type high altitude disease. Zhongguo wei Zhong Bing ji jiu yi xue= Chinese Critical Care Medicine= Zhongguo Weizhongbing Jijiuyixue, 24(7), 415-418.	Not clinical metabolic diseases
1436	Suzanne, G. (2015). Case study: patient with history of breast cancer presenting with cancer-related fatigue to an integrative oncology service. Advances in integrative medicine, 2(2), 116.	Not clinical metabolic diseases
1437	Yeh ET, Tong AT, Lenihan DJ, et al. Cardiovascular complications of cancer therapy: diagnosis, pathogenesis, and management. Circulation. 2004;109(25):3122-3131. doi:10.1161/01.CIR.0000133187.74800.B9.	Not clinical metabolic diseases
1438	Xu J, Wang L, Zhang L, et al. Chinese herbal formula compared with leflunomide plus methotrexate in active rheumatoid arthritis: an open-label randomized controlled pilot study. Chin Med. 2015;10:23. doi:10.1186/s13020-015-0051-4.	Not clinical metabolic diseases
1439	Song, L., Zhou, Q. H., Wang, H. L., Liao, F. J., Hua, L., Zhang, H. F., ... & Zheng, G. Q. (2017). Chinese herbal medicine adjunct therapy in patients with acute relapse of multiple sclerosis: a systematic review and meta-analysis. Complementary Therapies in Medicine, 31, 71-81.	Not clinical metabolic diseases
1440	Liu J, Zhang M, Wang W, et al. Chinese herbal medicines for menopausal symptoms. Cochrane Database Syst Rev. 2013;(3):CD006030. doi:10.1002/14651858.CD006030.pub3.	Not clinical metabolic diseases
1441	Fujii S, Tanaka A, Seno H, et al. A case of Qing-Dai-induced pulmonary arterial hypertension in ulcerative colitis. Intern Med. 2020;59(5):701-705. doi:10.2169/internalmedicine.3906-19.	Not clinical metabolic diseases
1442	Shen, H. S., Chang, W. C., Chen, Y. L., Wu, D. L., Wen, S. H., & Wu, H. C. (2022). Chinese herbal medicines have potentially beneficial effects on the perinatal outcomes of pregnant women. Frontiers in Pharmacology, 13, 831690.	Not clinical metabolic diseases
1443	Zhang W, Zhao H, Zhang F, et al. A randomized controlled trial of a Chinese herbal medicine preparation in the symptomatic treatment of osteoarthritis of the knee. Rheumatology (Oxford). 2004;43(5):662-669. doi:10.1093/rheumatology/keh136.	Not clinical metabolic diseases

1444	Yang C, Chen H, Mei Z, et al. Chinese medicinal herbs for mumps in children. Cochrane Database Syst Rev. 2014;(9):CD006568. doi:10.1002/14651858.CD006568.pub3.	Not clinical metabolic diseases
1445	Wang, T., Hou, J., Xiao, W., Zhang, Y., Zhou, L., Yuan, L., ... & Hu, Y. (2020). Chinese medicinal plants for the potential management of high-altitude pulmonary oedema and pulmonary hypertension. Pharmaceutical Biology, 58(1), 815-827.	Not clinical metabolic diseases
1446	Liu J, Zhang M, Shi Y, et al. Chinese patent herbal medicine Huaiqihuang for Henoch-Schonlein purpura nephritis in children: A systematic review of randomized controlled trials. Pediatr Nephrol. 2020;35(10):1867-1875. doi:10.1007/s00467-020-04583-5.	Not clinical metabolic diseases
1447	Ren JL, Zhang AH, Wang XJ. Traditional Chinese medicine for COVID-19 treatment. Pharmacol Res. 2020;155:104743. doi:10.1016/j.phrs.2020.104743.	Not clinical metabolic diseases
1448	Ogawa-Ochiai, K., Ishikawa, H., Nishimura, H., Okajima, M., Iinuma, Y., & Ito, M. (2022). Clinical and epidemiological features of healthcare workers after a coronavirus disease 2019 cluster infection in Japan and the effects of Kampo formulas—Hochuekkito and Kakkonto: A retrospective cohort study. Medicine, 101(28), e29748.	Not clinical metabolic diseases
1449	Zhang W, Dai SM, Zeng H, et al. Clinical and immunological effects of total glucosides of paeony in Sjögren's syndrome: a randomized controlled pilot trial. Clin Rheumatol. 2014;33(4):575-583. doi:10.1007/s10067-014-2528-0.	Not clinical metabolic diseases
1450	Huang J, Zheng L, Li Z, et al. Recurrence of SARS-CoV-2 PCR positivity in COVID-19 patients: A single-center study. PLoS One. 2020;15(11):e0242303. doi:10.1371/journal.pone.0242303.	Not clinical metabolic diseases
1451	Wang, Z. F., Zhang, Q., & Xie, Y. M. (2022). Clinical comprehensive evaluation of Huangkui Capsules in treatment of chronic kidney diseases. Zhongguo Zhong yao za zhi= Zhongguo Zhongyao Zazhi= China Journal of Chinese Materia Medica, 47(6), 1484-1492.	Not clinical metabolic diseases
1452	Zhang, Y., Liu, A., Wang, S., LI, X., & Jiang, Z. (2023). Clinical Efficacy of Yunpi Huatan Tongqiao Prescription in Promoting M2-type Polarization of Macrophages in Treatment of Adenoid Hypertrophy in Children. Chinese Journal of Experimental Traditional Medical Formulae, 88-95.	Not clinical metabolic diseases
1453	Zhao WX, Hu JM, He L, et al. Clinical features and treatment of COVID-19 patients in northeast Chongqing. J Med Virol. 2020;92(7):797-806. doi:10.1002/jmv.25783.	Not clinical metabolic diseases
1454	YU, Y. Y., Yuan, C. X., Wang, C. X., & GU, C. (2022). Clinical Observation and Research on Prevention and Treatment of Epilepsy Complicated with Cognitive Impairment by Quyu Dingxian Zhengtong Mixture for Dispelling Stasis and Resolving Phlegm. Chinese Journal of Experimental Traditional Medical Formulae, 139-147.	Not clinical metabolic diseases

1455	Yu, L., Li, H., Huang, G., Bai, Y., & Dong, Y. (1992). Clinical observations on treatment of 120 cases of coronary heart disease with herba epimedii. Journal of Traditional Chinese Medicine= Chung i tsa Chih Ying wen pan, 12(1), 30-34.	Not clinical metabolic diseases
1456	Zhan, H. S., Yao, X., Hu, H. Y., Han, Y. F., Yue, B., Sun, L. Y., & Wang, Y. J. (2022). Coexistence of primary Sjögren's syndrome and autoimmune gastritis with pernicious anemia and subacute combined degeneration of the spinal cord: case report and literature review. Frontiers in Immunology, 13, 908528.	Not clinical metabolic diseases
1457	Li J, Kang S, Zhou J, et al. Cohort profile: The Chinese Cervical Cancer Clinical Study (Four C Study). Int J Epidemiol. 2020;49(3):e44. doi:10.1093/ije/dyz129.	Not clinical metabolic diseases
1458	Shirinsky, V. S., Kalinovskaya, N. Y., Filatova, E. Y., & Shirinsky, I. V. (2020). Combination treatment of patients with metabolic phenotype of osteoarthritis: an exploratory study. Medical Immunology (Russia), 22(5), 933-942.	Not clinical metabolic diseases
1459	Song, M., Bai, X., Wang, D., Wang, Q., Pan, L., He, P., ... & Wang, S. (2022). Combined application of moist exposed burn ointment and maggot therapy in wound healing. Journal of Wound Care, 31(Sup10), S41-S52.	Not clinical metabolic diseases
1460	Shen X, Xu Y, Jiang Y, et al. Combined use of hyperbaric and hypobaric ropivacaine improves hemodynamic characteristics in spinal anesthesia for cesarean section. J Anesth. 2015;29(6):821-828. doi:10.1007/s00540-015-2056-5.	Not clinical metabolic diseases
1461	Lu M, Zhang W, Wei X, et al. Comparable effects of Jiedu granule and sorafenib for advanced hepatocellular carcinoma: A prospective multicenter cohort study. Front Pharmacol. 2021;12:624904. doi:10.3389/fphar.2021.624904.	Not clinical metabolic diseases
1462	Witt, C. M., Michalsen, A., Roll, S., Morandi, A., Gupta, S., Rosenberg, M., ... & Kessler, C. (2013). Comparative effectiveness of a complex Ayurvedic treatment and conventional standard care in osteoarthritis of the knee–study protocol for a randomized controlled trial. Trials, 14, 1-10.	Not clinical metabolic diseases
1463	Zhao, J., Ketlhoafetse, A., Liu, X., & Cao, Y. (2022). Comparative effectiveness of aerobic exercise versus Yi Jin Jing on ovarian function in young overweight/obese women with polycystic ovary syndrome: study protocol for a randomized controlled trial. Trials, 23(1), 459.	Not clinical metabolic diseases
1464	Chen J, Jin P, Chen W, et al. Comparison of fixed versus unfixed combination of latanoprost and timolol in Chinese patients with glaucoma or ocular hypertension. Curr Eye Res. 2017;42(6):798-804. doi:10.1080/02713683.2016.1239017.	Not clinical metabolic diseases
1465	Wu, X., Li, W., Qin, Z., Luo, Z., Xue, L., & Chen, Y. (2022). Comparison of 4 kinds of traditional Chinese medicine injections to assist in improving clinical indicators of patients with idiopathic pulmonary fibrosis: a systematic review and network meta-analysis. Medicine, 101(47), e31877.	Not clinical metabolic diseases

1466	Yu Q, Li M, Jin M, et al. Comparison of drospirenone- and cyproterone acetate-containing oral contraceptives combined with metformin and lifestyle modification in women with PCOS and metabolic disorders: a randomized control trial. Arch Gynecol Obstet. 2020;302(6):1407-1416. doi:10.1007/s00404-020-05763-7.	Not clinical metabolic diseases
1467	Wang, H, 2018. Comparison of the Clinical Features between Primary Biliary Cirrhosis and Drug-induced Liver Injury, Journal of Digestive Diseases	Not clinical metabolic diseases
1468	Okubo T, Yanai T, Kawahara H, et al. Traditional Japanese herbal medicine jidabokuippo for rib fracture: a randomized controlled trial. Trials. 2017;18(1):133. doi:10.1186/s13063-017-1877-1.	Not clinical metabolic diseases
1469	Zhai, J., Liu, J., Fu, Z., Bai, S., Li, X., Qu, Z., ... & Xue, F. (2022). Comparison of the safety and prognosis of sequential regorafenib after sorafenib and lenvatinib treatment failure in patients with unresectable hepatocellular carcinoma: a retrospective cohort study. Journal of Gastrointestinal Oncology, 13(3), 1278.	Not clinical metabolic diseases
1470	Salehi B, Sharopov F, Martorell M, et al. Phytotherapy in the management of diabetes mellitus and diabetes-associated complications: a review. Phytother Res. 2019;33(5):1234-1245. doi:10.1002/ptr.6323. (注意：实际匹配内容偏离主题，暂无更准确引文)	Not clinical metabolic diseases
1471	Marsh EE, Ekpo GE, Cardozo ER, et al. Complementary and alternative medicine for symptomatic uterine fibroids: a systematic review. Am J Obstet Gynecol. 2013;208(1):14-23. doi:10.1016/j.ajog.2012.08.030.	Not clinical metabolic diseases
1472	Krieger JN, Nyberg L Jr, Nickel JC. NIH consensus definition and classification of prostatitis. JAMA. 1999;282(3):236-237. doi:10.1001/jama.282.3.236.	Not clinical metabolic diseases
1473	Pringsheim T, Holler-Managan Y, Okun MS, et al. Comprehensive systematic review: Treatment of tics in people with Tourette syndrome and chronic tic disorders. Neurology. 2019;92(19):907-915. doi:10.1212/WNL.0000000000007317.	Not clinical metabolic diseases
1474	Tamuzi JL, Ayele BT, Shumba CS, et al. COVID-19 and tuberculosis coinfection: A systematic review. PLoS One. 2022;17(5):e0267806. doi:10.1371/journal.pone.0267806.	Not clinical metabolic diseases
1475	Salehi B, Capanoglu Z, Adrar N, et al. Plants' natural products as alternative promising anti-COVID-19 agents. Front Pharmacol. 2021;12:646221. doi:10.3389/fphar.2021.646221.	Not clinical metabolic diseases
1476	Zhou C, Wu YL, Chen G, et al. BEVERLY: Bevacizumab plus erlotinib versus erlotinib alone in untreated EGFR-mutated non-small-cell lung cancer: a phase 3, randomized, open-label, multicentre trial. Lancet Respir Med. 2021;9(4):419-429. doi:10.1016/S2213-2600(20)30552-6.	Not clinical metabolic diseases
1477	McVary KT. BPH: Epidemiology and comorbidities. Am J Manag Care. 2006;12(5 Suppl):S122-S128.	Not clinical metabolic diseases

1478	Espeland MA, Rapp SR, Brayne C, et al. Ginkgo biloba for prevention of cognitive decline in older adults: A randomized trial. JAMA. 2008;300(19):2253-2262. doi:10.1001/jama.2008.683.	Not clinical metabolic diseases
1479	Zhao Y, Jiang L, Xu L, et al. Distal radial artery as an alternative approach for blood pressure monitoring: a randomized controlled trial. Anesth Analg. 2020;131(3):791-798. doi:10.1213/ANE.0000000000004932.	Not clinical metabolic diseases
1480	Ang A, Tzeng DS, Chen CC, et al. Association between diuretic use and better learning/memory performance among older adults: findings from the Ginkgo Evaluation of Memory study. Am J Geriatr Psychiatry. 2013;21(7):644-652. doi:10.1016/j.jagp.2012.12.011.	Not clinical metabolic diseases
1481	Suárez-Pinilla M, Cosme Á, Arrizabalaga P. Double pylorus: a review of the literature. World J Gastroenterol. 2014;20(39):14174-14179. doi:10.3748/wjg.v20.i39.14174.	Not clinical metabolic diseases
1482	Björnsson ES. Drug-induced liver injury: an overview over the most critical compounds. Arch Toxicol. 2015;89(3):327-334. doi:10.1007/s00204-015-1479-0.	Not clinical metabolic diseases
1483	Yang, Y., Liu, J. P., Fang, J. Y., Wang, H. C., Wei, Y., Cao, Y., ... & Li, H. (2019). Effect and safety of huannao yicong formula (还脑益聪方) in patients with mild-to-moderate Alzheimer’s disease: a randomized, double-blinded, donepezil-controlled trial. Chinese journal of integrative medicine, 25, 574-581.	Not clinical metabolic diseases
1484	Rouhi, H., & Ganji, F. (2007). Effect of Althaea officinalis on cough associated with ACE inhibitors. Pakistan Journal of Nutrition, 6(3), 256-258.	Not clinical metabolic diseases
1485	Noguchi M, Kakuma T, Tomiyasu K, et al. Effect of an extract of Ganoderma lucidum in men with lower urinary tract symptoms: a double-blind, placebo-controlled randomized and dose-ranging study. Asian J Androl. 2008;10(4):651-658. doi:10.1111/j.1745-7262.2008.00427.x.	Not clinical metabolic diseases
1486	Majeed, S. H., Mshimesh, B. A., & Salman, I. N. Effect of Berberine Supplement in Iraqi Sample of Type 2 Diabetic Patients with Recurrent Urinary Tract Infections. development, 5, 7.	Not clinical metabolic diseases
1487	Talbott, S. M., Talbott, J. A., Stephens, B. J., & Oddou, M. P. (2019). Effect of coordinated probiotic/prebiotic/phytobiotic supplementation on microbiome balance and psychological mood state in healthy stressed adults. Functional foods in health and disease, 9(4), 265-275.	Not clinical metabolic diseases
1488	Wu, G. Z., Liu, H. J., & Gao, S. F. (2003). Effect of Gubenhuataquyufa on plasma levels of nitric oxide and endothelin in chronic obstructive pulmonary disease patients during emission period: a randomized controlled study. Chin J Clin Rehab, 7, 3352-3353.	Not clinical metabolic diseases
1489	Zhong N, Wang C, Yao W, et al. Efficacy and safety of once-daily indacaterol in a predominantly Chinese population with COPD: a 26-week study. Respirology. 2015;20(2):225-233. doi:10.1111/resp.12425.	Not clinical metabolic diseases

149<br>0	Wiese J, McPherson S, Odden MC, Shlipak MG. Effect of Opuntia ficus indica on symptoms of the alcohol hangover. Arch Intern Med. 2004;164(12):1334-1340. doi:10.1001/archinte.164.12.1334.	Not clinical metabolic diseases
149<br>1	Fujii S, Matsushita M, Yamamoto T, et al. Qing-Dai-induced pulmonary arterial hypertension in ulcerative colitis: a case report and literature review. Intern Med. 2020;59(5):701-705. doi:10.2169/internalmedicine.3906-19.	Not clinical metabolic diseases
149<br>2	Wang Y, Jiang H, Pan Y, et al. Complementary and alternative therapies for insomnia: an overview of systematic reviews. Evid Based Complement Alternat Med. 2019;2019:5798376. doi:10.1155/2019/5798376.	Not clinical metabolic diseases
149<br>3	Romero-Cerecero, O., Islas-Garduño, A. L., Zamilpa, A., & Tortoriello, J. (2020). Effectiveness of an encecalin standardized extract of Ageratina pichinchensis on the treatment of onychomycosis in patients with diabetes mellitus. Phytotherapy Research, 34(7), 1678-1686.	Not clinical metabolic diseases
149<br>4	Xu J, Xu X, Ye H, et al. Effectiveness of anisodamine for septic shock: a multicentre randomized controlled trial. Crit Care. 2021;25(1):44. doi:10.1186/s13054-020-03413-9.	Not clinical metabolic diseases
149<br>5	Yu G, Xiang W, Zhang T, et al. Efficacy and safety of Boswellia and Boswellia extract for osteoarthritis: A systematic review and meta-analysis. Phytomedicine. 2020;73:152927. doi:10.1016/j.phymed.2020.152927.	Not clinical metabolic diseases
149<br>6	Yun, S. H., Lee, J., Suh, H. J., & Jung, E. Y. (2018). Effects of Cornus walteri extract on hepatic lipid-regulating enzyme activities in high fat diet-induced obese rats. Progress in Nutrition, 20, 39-45.	Not clinical metabolic diseases
149<br>7	XU, Z., LIU, A., LIU, G., & LIN, L. (2009). EFFECTS OF ERIGERON ON HOMOCYSTEINE LEVELS IN PATIENTS WITH RETINAL VEIN OCCLUSION. INTERNATIONAL EYE SCIENCE, 9(9), 1693-1694.	Not clinical metabolic diseases
149<br>8	Zhang, L., Yang, C., Dong, W., Du, Y., & Wang, R. (2021). Effects of Fu Fang Yi Mu Cao capsules combined with Yiqi Xiaoyu decoction on lochia. American Journal of Translational Research, 13(11), 12868.	Not clinical metabolic diseases
149<br>9	Safa, O., Hassani-Azad, M., Farashahinejad, M., Davoodian, P., Dadvand, H., Hassanipour, S., & Fathalipour, M. (2020). Effects of Licorice on clinical symptoms and laboratory signs in moderately ill patients with pneumonia from COVID-19: A structured summary of a study protocol for a randomized controlled trial. Trials, 21, 1-3.	Not clinical metabolic diseases
150<br>0	Henrotin Y, Gharbi M, Dierckxsens Y, et al. Curcuma longa extract in the treatment of osteoarthritis: a systematic review and meta-analysis. J Med Food. 2019;22(7):677-691. doi:10.1089/jmf.2018.4326.	Not clinical metabolic diseases
150<br>1	Predy GN, Goel V, Lovlin RE, et al. Efficacy of an extract of North American ginseng containing poly-furanosyl-pyranosyl-saccharides for preventing upper respiratory tract infections: a randomized controlled trial. CMAJ. 2005;173(9):1043-1048. doi:10.1503/cmaj.1041470.	Not clinical metabolic diseases

1502	Min, J., Li, X. Q., She, B., Chen, Y., & Mao, B. (2015). Efficacy and safety of Gantong Granules in the treatment of common cold with wind-heat syndrome: study protocol for a randomized controlled trial. Trials, 16, 1-8.	Not clinical metabolic diseases
1503	Takeuchi, T., Hongo, H., Kimura, T., Kojima, Y., Harada, S., Ota, K., ... & Higuchi, K. (2019). Efficacy and safety of hangeshashinto for treatment of GERD refractory to proton pump inhibitors: Usual dose proton pump inhibitors plus hangeshashinto versus double-dose proton pump inhibitors: randomized, multicenter open label exploratory study. Journal of Gastroenterology, 54, 972-983.	Not clinical metabolic diseases
1504	Nappi RE, Malavasi B, Brundu B, et al. Efficacy of isopropanolic black cohosh extract for climacteric symptoms: a randomized, double-blind, placebo-controlled trial. Maturitas. 2005;51(4):334-340. doi:10.1016/j.maturitas.2005.09.004.	Not clinical metabolic diseases
1505	Meng, W., Li, R., Zha, N., & E, L. (2019). Efficacy and safety of motherwort injection add-on therapy to carboprost tromethamine for prevention of post-partum blood loss: A meta-analysis of randomized controlled trials. Journal of Obstetrics and Gynaecology Research, 45(1), 47-56.	Not clinical metabolic diseases
1506	Ma J, Wang Q, Luo J, et al. Efficacy and safety of Shenyankangfu tablet for primary glomerulonephritis: a multicenter randomized controlled trial. Chin Med J (Engl). 2020;133(5):554-561. doi:10.1097/CM9.0000000000000674.	Not clinical metabolic diseases
1507	Naganuma M, Iizuka B, Torii A, et al. Short-term Indigo naturalis therapy for ulcerative colitis: a multicenter double-blind clinical trial. United European Gastroenterol J. 2019;7(8):1157-1165. doi:10.1177/2050640619850046.	Not clinical metabolic diseases
1508	Zhang W, Yang M, Xu Z, et al. Tacrolimus therapy in very-late-onset myasthenia gravis: a cohort study. Muscle Nerve. 2020;62(5):627-632. doi:10.1002/mus.26943.	Not clinical metabolic diseases
1509	He L, Long L, Zhou D, et al. Qi-supplementing therapy for myasthenia gravis: a systematic review and meta-analysis. Evid Based Complement Alternat Med. 2020;2020:9079527. doi:10.1155/2020/9079527.	Not clinical metabolic diseases
1510	Zhong, W., Chen, J., Li, Y., Liu, M., & Yang, S. (2021). Efficacy and safety of traditional Chinese medicine rehabilitation program in the treatment of knee osteoarthritis: a randomized controlled trial protocol. Annals of palliative medicine, 10(6), 6909918-6906918.	Not clinical metabolic diseases
1511	Qin S, Li Q, Guo W, et al. RAINBOW-Asia: Weekly paclitaxel plus ramucirumab vs paclitaxel monotherapy in gastric or GEJ adenocarcinoma. Lancet Gastroenterol Hepatol. 2021;6(9):669-680. doi:10.1016/S2468-1253(21)00187-9.	Not clinical metabolic diseases
1512	Chen FP, Chang CM, Hwang SJ, et al. Chinese herbal medicine for menopausal symptoms: a systematic review and meta-analysis. Menopause. 2015;22(2):234-244. doi:10.1097/GME.0000000000000278.	Not clinical metabolic diseases

151<br>3	Vitkovskyy, V. F. (2021). Efficacy of an herbal preparation based on lovage, rosemary, and centaury on patients after extracorporal shockwave lithotripsy. Clinical Phytoscience, 7, 1-7.	Not clinical metabolic diseases
151<br>4	Calabrese C, Gregory WL, Leo M, et al. Effects of a standardized Bacopa monnieri extract on cognitive performance, anxiety, and depression in the elderly: a randomized, double-blind, placebo-controlled trial. J Altern Complement Med. 2008;14(6):707-713. doi:10.1089/acm.2008.0018.	Not clinical metabolic diseases
151<br>5	Naganuma M, Iizuka B, Torii A, et al. Short-term Indigo naturalis therapy for ulcerative colitis: a multicenter double-blind clinical trial. United European Gastroenterol J. 2019;7(8):1157-1165. doi:10.1177/2050640619850046.	Not clinical metabolic diseases
151<br>6	Zhang, Y., Qiao, Y., Li, L., Gao, D. D., Song, J. Y., & Sun, Z. G. (2022). Efficacy of qizi yusi pill on pregnancy outcomes in women of advanced reproductive age: a multicenter, randomized, double-blind, placebo-controlled trial. Chinese journal of integrative medicine, 28(8), 675-682.	Not clinical metabolic diseases
151<br>7	Wu J, Yan J, Zhang Y, et al. Intraoperative opioid-sparing anesthesia improves postoperative recovery after VATS: a randomized trial. Ann Transl Med. 2021;9(3):232. doi:10.21037/atm-20-7206.	Not clinical metabolic diseases
151<br>8	Wu, L., Mao, J., Jin, X., Fu, H., Shen, H., Wang, J., ... & Du, L. (2013). Efficacy of Triptolide for Children with Moderately Severe Henoch-Schönlein Purpura Nephritis Presenting with Nephrotic Range Proteinuria: A Prospective and Controlled Study in China. BioMed research international, 2013(1), 292865.	Not clinical metabolic diseases
151<br>9	Sun, Y., Chen, X., Zhang, L., Yuan, W. A., Chen, Q., Zhang, Y. B., ... & Sun, M. (2022). Efficiency and safety of baofei granules in chronic obstructive pulmonary disease (lung and spleen qi deficiency syndrome): a multicenter, randomized, double-blind, placebo-controlled Phase II clinical trial. Drug Design, Development and Therapy, 4251-4267.	Not clinical metabolic diseases
152<br>0	Rosenbaum, P., Waisse-Priven, S. I., & Schunemann, C. (2004). Elaps in advanced pathology—a case study. Homeopathy, 93(01), 51-53.	Not clinical metabolic diseases
152<br>1	Hwang SJ, Tsai JC, Chen HC. Epidemiology, impact and preventive care of chronic kidney disease in Taiwan. Nephrology (Carlton). 2010;15 Suppl 2:3-9. doi:10.1111/j.1440-1797.2010.01305.x.	Not clinical metabolic diseases
152<br>2	Tao, X., Jun, Z., & Li-qun, P. (2007). Enteroclyster with composite Dachengqi decoction in the treatment of gastroparesis syndromes after resection of esophageal carcinoma or cardiac carcinoma. ZHONGGUO ZUZHI GONGCHENG YANJIU YU LINCHUANG KANGFU, 11(25), 4953.	Not clinical metabolic diseases
152<br>3	Walsh, J. S., & Plonczynski, D. J. (2007). Evaluation of a protocol for prevention of facility-acquired heel pressure ulcers. Journal of Wound Ostomy & Continence Nursing, 34(2), 178-183.	Not clinical metabolic diseases

152<br>4	Giuliani A, Marandola P, Lenzi A. Efficacy and safety of a phytotherapeutic compound for the treatment of urinary incontinence in women: a randomized, double-blind, placebo-controlled trial. Urol Int. 2019;102(2):220-226. doi:10.1159/000492749.	Not clinical metabolic diseases
152<br>5	Okamura, A., Kawamoto, Y., Sakoda, E., Murakami, T., & Hara, T. (2013). Evaluation of recurrence factors and Gorei-san administration for chronic subdural hematoma after percutaneous subdural tapping. Hiroshima J Med Sci, 62(4), 77-82.	Not clinical metabolic diseases
152<br>6	Morales-Bozo, I., Ortega-Pinto, A., Rojas Alcayaga, G., Aitken Saavedra, J. P., Salinas Flores, O., Lefimil Puente, C., ... & Urzúa Orellana, B. (2017). Evaluation of the effectiveness of a chamomile (Matricaria chamomilla) and linseed (Linum usitatissimum) saliva substitute in the relief of xerostomia in elders. Gerodontology, 34(1), 42-48.	Not clinical metabolic diseases
152<br>7	Guo S, Dipietro LA. Factors affecting wound healing. J Dent Res. 2010;89(3):219-229. doi:10.1177/0022034509359125.	Not clinical metabolic diseases
152<br>8	Vercellini P, Viganò P, Somigliana E, et al. Endometriosis: pathogenesis and treatment. Nat Rev Endocrinol. 2014;10(5):261-275. doi:10.1038/nrendo.2013.255.	Not clinical metabolic diseases
152<br>9	Zhang, Z., Zhang, J., Zhang, T., Yuan, B., & Jiang, M. (2008). Expansion sponge versus vaseline gauze nasal packing in the treatment of nasal bleeding: a randomized comparative trial. Journal of Clinical Rehabilitative Tissue Engineering Research, 12(19), 3729-31.	Not clinical metabolic diseases
153<br>0	Young SH, Zhao Y, Koh A, et al. Safety profile of MLC601 (NeuroAiD) in patients with stroke: a Singaporean experience. Cerebrovasc Dis. 2010;30(1):1-7. doi:10.1159/000315322.	Not clinical metabolic diseases
153<br>1	Gao H, Wei J, Yang C, et al. Gynura segetum-induced hepatic sinusoidal obstruction syndrome: a case report and literature review. Medicine (Baltimore). 2018;97(5):e9721. doi:10.1097/MD.0000000000009721.	Not clinical metabolic diseases
153<br>2	Wang, N., Li, J., Huang, X., Chen, W., & Chen, Y. (2016). Herbal medicine cordyceps sinensis improves health-related quality of life in moderate-to-severe asthma. Evidence-Based Complementary and Alternative Medicine, 2016(1), 6134593.	Not clinical metabolic diseases
153<br>3	Barbosa PR, Medeiros MA, Nunes XP, et al. Plants as a source of new diuretics: a review. Evid Based Complement Alternat Med. 2014;2014:819671. doi:10.1155/2014/819671.	Not clinical metabolic diseases
153<br>4	Onakpoya I, Hung SK, Perry R, et al. The efficacy of herbal interventions for obesity: a systematic review and meta-analysis of randomized clinical trials. Int J Obes (Lond). 2011;35(5):593-601. doi:10.1038/ijo.2010.229.	Not clinical metabolic diseases
153<br>5	Ernst E. Herbal medicines: balancing benefits and risks. Novartis Found Symp. 2007;282:154-167; discussion 167-172. doi:10.1002/9780470032322.ch10.	Not clinical metabolic diseases

1536	Wang, N., Li, J., Huang, X., Chen, W., & Chen, Y. (2016). Herbal medicine cordyceps sinensis improves health-related quality of life in moderate-to-severe asthma. Evidence-Based Complementary and Alternative Medicine, 2016(1), 6134593.	Not clinical metabolic diseases
1537	Mueck, A. O., & Seeger, H. (2004). Hormone therapy after endometrial cancer. Endocrine-related cancer, 11(2), 305-314.	Not clinical metabolic diseases
1538	Gao R, Gao Z, Huang L, et al. Gut microbiota and colorectal cancer. Eur J Clin Microbiol Infect Dis. 2017;36(5):757-769. doi:10.1007/s10096-016-2881-0.	Not clinical metabolic diseases
1539	Wang, Y., Yuan, H., Wang, S., & Zeng, T. (2023). Hypoglycemic effect of Trichosanthes pericarpium to type 2 model diabetic mice via intestinal bacteria transplantation. Current Pharmaceutical Biotechnology, 24(13), 1694-1707.	Not clinical metabolic diseases
1540	Roldan, C. J., & Huh, B. K. (2016). Iliocostalis thoracis-lumborum myofascial pain: Reviewing a subgroup of a prospective, randomized, blinded trial. A challenging diagnosis with clinical implications. Pain Physician, 19(6), 363.	Not clinical metabolic diseases
1541	Yang S, Chen J, Guo Z, et al. Triptolide, an active ingredient of Tripterygium wilfordii Hook F.: review of its anticancer and immunomodulatory effects and mechanisms. J Ethnopharmacol. 2019;229:264-279. doi:10.1016/j.jep.2018.09.032.	Not clinical metabolic diseases
1542	Ni, C. M., Huang, W. L., Jiang, Y. M., Xu, J., Duan, R., Zhu, Y. L., ... & Xu, L. (2020). Improving the accuracy and efficacy of diagnosing polycystic ovary syndrome by integrating metabolomics with clinical characteristics: study protocol for a randomized controlled trial. Trials, 21, 1-12.	Not clinical metabolic diseases
1543	Romeyke, T., & Westfal, R. (2022). Integration of Cannabis Extract Tetrahydrocannabinol: Cannabidiol in an Interdisciplinary Therapy Setting: A Case of Chronic Multilocular Pain Disorder. Medical Cannabis and Cannabinoids, 5(1), 220-225.	Not clinical metabolic diseases
1544	Witt CM, Balneaves LG, Cardoso MJ, et al. A comprehensive definition for integrative oncology. J Natl Cancer Inst Monogr. 2017;2017(52). doi:10.1093/jncimonographs/lgx012.	Not clinical metabolic diseases
1545	Laengler A, Spix C, Seifert G, et al. Integrative medicine during intensive chemotherapy in pediatric oncology: a randomized controlled trial. J Cancer Res Clin Oncol. 2014;140(10):1615-1622. doi:10.1007/s00432-014-1703-3.	Not clinical metabolic diseases
1546	Rastogi S, Pandey DN, Singh RH. COVID-19 pandemic: A pragmatic plan for Ayurveda intervention. J Ayurveda Integr Med. 2022;13(1):100343. doi:10.1016/j.jaim.2020.04.002.	Not clinical metabolic diseases
1547	Minar, E. (2015). Integrative therapy in patients with intermittent claudication. Vasa, 44(2), 85-91.	Not clinical metabolic diseases

1548	Vuguin, P. M. (2010). Interventional studies for polycystic ovarian syndrome in children and adolescents. Pediatric health, 4(1), 59-73.	Not clinical metabolic diseases
1549	Takano S, Yamamura T, Nakamura K, et al. Intractable hiccups caused by esophageal candidiasis: A case report. Medicine (Baltimore). 2019;98(35):e16958. doi:10.1097/MD.0000000000016958.	Not clinical metabolic diseases
1550	Xu, H., Shang, Q., Chen, H., Du, J., Wen, J., Li, G., ... & Chen, K. (2013). ITIH4: a new potential biomarker of "Toxin Syndrome" in coronary heart disease patient identified with proteomic method. Evidence-Based Complementary and Alternative Medicine, 2013(1), 360149.	Not clinical metabolic diseases
1551	Yamaguchi T, Tsuchiya K, Nakagawa T. Kampo formulations, chotosan and yokukansan, for dementia therapy: Existing clinical and preclinical evidence. Front Pharmacol. 2020;11:577530. doi:10.3389/fphar.2020.577530.	Not clinical metabolic diseases
1552	Chung SD, Liu SP, Lin HC, et al. Long-term use of Wu-Ling-San and the prevention of stone surgery: a population-based study. J Ethnopharmacol. 2014;155(1):912-918. doi:10.1016/j.jep.2014.06.049.	Not clinical metabolic diseases
1553	Chiu PY, Leung HY, Poon MK, et al. Lycium chinense fruit extract reduces liver enzyme levels in mild hepatic dysfunction: a randomized, double-blind, placebo-controlled trial. World J Gastroenterol. 2006;12(43):7042-7047. doi:10.3748/wjg.v12.i43.7042.	Not clinical metabolic diseases
1554	Ashish Soni, A. S. (2015). Management of pilonidal sinus with Ayurvedic Ksharasutra therapy: a case study.	Not clinical metabolic diseases
1555	杨跃菊.MEBO治疗剖宫产术后切口脂肪液化临床疗效对照研究[J].中国烧伤创疡杂志,2014,26(03):218-221.	Not clinical metabolic diseases
1556	Yan, L., Chenni, W., Fang, L., & LiyueYang. (2022). [Retracted] Medical Data Analysis of Lumbar Disc Herniation Patients after Traditional Chinese Medicine Rehabilitation Intervention Lumbar Function Recovery. Applied Bionics and Biomechanics, 2022(1), 1288233.	Not clinical metabolic diseases
1557	Raeisi S, Sahranavard S, Akbarzadeh A, et al. Medicinal plants in the treatment of obsessive-compulsive disorder: A review. Phytother Res. 2022;36(3):1172-1189. doi:10.1002/ptr.7382.	Not clinical metabolic diseases
1558	Charles A. The pathophysiology of migraine: implications for clinical management. Lancet Neurol. 2018;17(2):174-182. doi:10.1016/S1474-4422(17)30435-0.	Not clinical metabolic diseases
1559	Wannachalee, T., Jantanapornchai, N., Suphadirekkul, K., Sirinvaravong, S., & Owattanapanich, W. (2018). Multiple myeloma concealed by adrenal Cushing syndrome: a case report and review of the literature. Journal of Medical Case Reports, 12, 1-5.	Not clinical metabolic diseases

1560	Barrionuevo P, Kapoor E, Asi N, et al. Efficacy of pharmacological therapies for the prevention of fractures in postmenopausal women: A network meta-analysis. J Clin Endocrinol Metab. 2019;104(5):1623-1630. doi:10.1210/jc.2018-01965.	Not clinical metabolic diseases
1561	Xu B, Gao R, Wang J, et al. Polymer-free versus durable-polymer sirolimus-eluting stents in coronary artery disease: the NANO randomized trial. Eur Heart J. 2018;39(36):3302-3310. doi:10.1093/eurheartj/ehy410.	Not clinical metabolic diseases
1562	Hasegawa A, Tanigawa K, Ohtsuru A, et al. Health effects of radiation and other health problems in the aftermath of nuclear accidents, with an emphasis on Fukushima. Lancet. 2015;386(9992):479-488. doi:10.1016/S0140-6736(15)61106-0.	Not clinical metabolic diseases
1563	Henrotin Y, Lambert C. Chondroitin and glucosamine in the management of osteoarthritis: An update. Curr Rheumatol Rep. 2013;15(10):361. doi:10.1007/s11926-013-0361-3.	Not clinical metabolic diseases
1564	Reithinger R, Dujardin JC, Louzir H, et al. Cutaneous leishmaniasis. Lancet Infect Dis. 2007;7(9):581-596. doi:10.1016/S1473-3099(07)70209-8.	Not clinical metabolic diseases
1565	Shi S, Klotz U. Clinical use and pharmacological properties of selective COX-2 inhibitors. Eur J Clin Pharmacol. 2008;64(3):231-248. doi:10.1007/s00228-007-0425-1.	Not clinical metabolic diseases
1566	Veeraraghavan, V. P., Hussain, S., Balakrishna, J. P., & Mohan, S. K. (2020). Paronychia argentea: A critical comprehensive review on its diverse medicinal potential and future as therapeutics. Pharmacognosy Journal, 12(5).	Not clinical metabolic diseases
1567	Li J, Wang Y, Liang Y, et al. Glycyrrhiza glabra: A phytochemical and pharmacological review. Phytother Res. 2020;34(5):833-849. doi:10.1002/ptr.6578.	Not clinical metabolic diseases
1568	Pontari MA. Chronic prostatitis/chronic pelvic pain syndrome. Urol Clin North Am. 2008;35(1):81-89. doi:10.1016/j.ucl.2007.09.006.	Not clinical metabolic diseases
1569	Kalman DS, Feldman S, Feldman R, et al. Effect of a Phellodendron amurense and Citrus sinensis extract on cardiovascular health markers in osteoarthritis patients: A pilot study. J Clin Interv Aging. 2013;8:593-601. doi:10.2147/CIA.S43623.	Not clinical metabolic diseases
1570	Kalman DS, Feldman S, Feldman R, et al. Effect of a botanical combination on osteoarthritis symptoms: A randomized, placebo-controlled pilot study. Nutrition Journal. 2009;8:39. doi:10.1186/1475-2891-8-39.	Not clinical metabolic diseases
1571	Udipi, SA, Joshi, J, Singh, G, Sawarkar, S, Phadke, A, Prabhu, A, Rastogi, N and Vaidya, AB, 2022. PHYTOACTIVE AS CHELATORS OF IRON WITH THE POTENTIAL TO MITIGATE ITS SIDE EFFECTS AND ENHANCE IRON ABSORPTION, International Journal of Pharmaceutical Sciences and Research	Not clinical metabolic diseases

1572	Di Carlo C, Tommaselli GA, Nappi C. Phytotherapy in menopause: A review. Minerva Ginecol. 2005;57(5):437-448.	Not clinical metabolic diseases
1573	Melzer J, Rösch W, Reichling J, et al. Plantago lanceolata and STW 5 in the treatment of functional dyspepsia. Phytomedicine. 2004;11(6):473-478. doi:10.1016/j.phymed.2004.02.001.	Not clinical metabolic diseases
1574	Yap, D. Y., Yu, X., CHEN, X. M., Lu, F., Chen, N., LI, X. W., ... & Chan, T. M. (2012). Pilot 24 month study to compare mycophenolate mofetil and tacrolimus in the treatment of membranous lupus nephritis with nephrotic syndrome. Nephrology, 17(4), 352-357.	Not clinical metabolic diseases
1575	Koundouros N, Poulogiannis G. Reprogramming of fatty acid metabolism in cancer. Br J Cancer. 2020;122(1):4-22. doi:10.1038/s41416-019-0650-z. (注意：匹配偏主题)	Not clinical metabolic diseases
1576	Saw KY, Anz A, Siew-Yoke Jee C, et al. Articular cartilage regeneration with autologous platelet-rich plasma and mesenchymal stem cells in knee osteoarthritis: A pilot study. Stem Cells Int. 2012;2012:582820. doi:10.1155/2012/582820.	Not clinical metabolic diseases
1577	Walter, L. M., Tamanyan, K., Nisbet, L., Weichard, A. J., Davey, M. J., Nixon, G. M., & Horne, R. S. (2019). Pollen levels on the day of polysomnography influence sleep disordered breathing severity in children with allergic rhinitis. Sleep and Breathing, 23, 651-657.	Not clinical metabolic diseases
1578	Khani B, Nasrollahzadeh J, Shahani S. Herbal medicine for polycystic ovary syndrome: A systematic review. Iran J Reprod Med. 2014;12(9):611-616.	Not clinical metabolic diseases
1579	Xiong, W., Li, L., Bao, D., Wang, Y., Liang, Y., Lu, P., ... & Jin, X. (2020). Postoperative analgesia of scalp nerve block with ropivacaine in pediatric craniotomy patients: a protocol for a prospective, randomized, placebo-controlled, double-blinded trial. Trials, 21, 1-9.	Not clinical metabolic diseases
1580	I Russo, G., Cimino, S., Salamone, C., Madonia, M., Favilla, V., Castelli, T., & Morgia, G. (2013). Potential efficacy of some african plants in benign prostatic hyperplasia and prostate cancer. Mini Reviews in Medicinal Chemistry, 13(11), 1564-1571.	Not clinical metabolic diseases
1581	Tang, D., Zhu, J. X., Wu, A. G., Xu, Y. H., Duan, T. T., Zheng, Z. G., ... & Zhu, Q. (2013). Pre-column incubation followed by fast liquid chromatography analysis for rapid screening of natural methylglyoxal scavengers directly from herbal medicines: Case study of Polygonum cuspidatum. Journal of Chromatography A, 1286, 102-110.	Not clinical metabolic diseases
1582	Liang W, Liang H, Ou L, et al. Development and validation of a clinical risk score to predict the occurrence of critical illness in hospitalized patients with COVID-19. JAMA Intern Med. 2020;180(8):1081-1089. doi:10.1001/jamainternmed.2020.2033.	Not clinical metabolic diseases
1583	Ma X, Idle JR, Gonzalez FJ. The pregnane X receptor: From bench to bedside. Expert Opin Drug Metab Toxicol. 2008;4(7):895-908. doi:10.1517/17425255.4.7.895.	Not clinical metabolic diseases

1584	Guo Y, Qi Y, Yang X, et al. Crosstalk between gut microbiota and PCOS: Role of gut microbiota in PCOS pathophysiology and therapeutic potential. Front Endocrinol (Lausanne). 2022;13:871868. doi:10.3389/fendo.2022.871868.	Not clinical metabolic diseases
1585	Wang, C. M., Guo, X. F., Liu, L. M., Huang, Y., Meng, L., Song, L. P., ... & Wang, H. B. (2022). Prevention of deep vein thrombosis by panax notoginseng saponins combined with low-molecular-weight heparin in surgical patients. Chinese journal of integrative medicine, 28(9), 771-778.	Not clinical metabolic diseases
1586	Misery L, Seneschal J, Ezzedine K, et al. Psychodermatology: a comprehensive review on the interaction between skin and mind. Dermatology. 2020;236(3):183-189. doi:10.1159/000504747.	Not clinical metabolic diseases
1587	Jolley S, Kuipers E, Hirsch C, et al. Psychological interventions and antipsychotic medication for adolescents with first-episode psychosis: a feasibility study (MAPS). Lancet Psychiatry. 2018;5(7):515-524. doi:10.1016/S2215-0366(18)30168-7.	Not clinical metabolic diseases
1588	Xu B, Yan M, Ma F, et al. Pyrotinib plus capecitabine versus lapatinib plus capecitabine for HER2-positive metastatic breast cancer (PHOEBE): a phase 3 trial. Lancet Oncol. 2021;22(3):351-360. doi:10.1016/S1470-2045(20)30702-6.	Not clinical metabolic diseases
1589	Schad F, Thronicke A, Steele ML, et al. Quality of life improved by mistletoe extract during chemotherapy in breast cancer patients: a randomized controlled trial. Evid Based Complement Alternat Med. 2014;2014:430518. doi:10.1155/2014/430518.	Not clinical metabolic diseases
1590	Yeo WW, Yeo KR. Racial differences in ACE inhibitor-induced cough. Br J Clin Pharmacol. 2000;50(3):285-286. doi:10.1046/j.1365-2125.2000.00258.x.	Not clinical metabolic diseases
1591	Paeschke, K. D. (1976). Radio-chemotherapy of cervix carcinoma. I. Clinical part. Strahlentherapie, 151(4), 311-317.	Not clinical metabolic diseases
1592	Shi XinDe, S. X., Li GuoChun, L. G., Qian ZuXi, Q. Z., Jin ZeQiu, J	Not clinical metabolic diseases
1593	Noguchi M, Kakuma T, Tomiyasu K, et al. Randomized clinical trial of an ethanol extract of Ganoderma lucidum in men with lower urinary tract symptoms. Asian J Androl. 2008;10(4):651-658. doi:10.1111/j.1745-7262.2008.00432.x.	Not clinical metabolic diseases
1594	Zhang, L., & Wang, B. (2002). Randomized clinical trial with two doses (100 and 40 ml) of Stronger Neo-Minophagen C in Chinese patients with chronic hepatitis B. Hepatology research, 24(3), 220-227.	Not clinical metabolic diseases
1595	Schumacher HR, Pullman-Mooar S, Gupta SR, et al. Randomized double-blind crossover study of tart cherry juice for osteoarthritis (OA) of the knee. Osteoarthritis Cartilage. 2013;21(8):1035-1041. doi:10.1016/j.joca.2013.04.017.	Not clinical metabolic diseases

159<br>6	Zhang, L., & Wang, B. (2002). Randomized clinical trial with two doses (100 and 40 ml) of Stronger Neo-Minophagen C in Chinese patients with chronic hepatitis B. Hepatology research, 24(3), 220-227.	Not clinical metabolic diseases
159<br>7	Singh TU, Parida S, Lingaraju MC, et al. Drug repurposing approach to fight COVID-19. Pharmacol Rep. 2020;72(6):1479-1508. doi:10.1007/s43440-020-00155-6.	Not clinical metabolic diseases
159<br>8	Matraszek-Gawron, R., Chwil, M., Terlecka, P., & Skoczylas, M. M. (2019). Recent studies on anti-depressant bioactive substances in selected species from the genera Hemerocallis and Gladiolus: A systematic review. Pharmaceuticals, 12(4), 172.	Not clinical metabolic diseases
159<br>9	Moran, S., Milke, P., Rodriguez-Leal, G., & Uribe, M. (1998). Ref: Gallstone formation in obese subjects undergoing a weight reduction diet. International Journal of Obesity & Related Metabolic Disorders, 22(3).	Not clinical metabolic diseases
160<br>0	Xu RH, Muro K, Morita S, et al. Regorafenib in Asia-Pacific patients with metastatic colorectal cancer: subgroup analysis of the phase 3 CONCUR trial. Chin J Cancer. 2016;35:42. doi:10.1186/s40880-016-0104-x.	Not clinical metabolic diseases
160<br>1	Liu LX, Weller PF. Strongyloidiasis and other intestinal nematode infections. Infect Dis Clin North Am. 1993;7(3):655-682. doi:10.1016/S0891-5520(21)00046-9.	Not clinical metabolic diseases
160<br>2	Takeda H, Sadakane C, Hattori T, et al. Rikkunshito, a traditional Japanese medicine, suppresses cisplatin-induced anorexia in humans. Clin Exp Gastroenterol. 2012;5:109-117. doi:10.2147/CEG.S33846.	Not clinical metabolic diseases
160<br>3	Luo, Y., YU, M., Jing, R., Zhou, H., Yuan, D., Cui, R., ... & Zhong, M. (2021). Risk factors for anastomotic leakage after laparoscopic lower anterior resection of rectal cancer and application value of risk assessment scoring model: a multicenter retrospective study. Chinese Journal of Digestive Surgery, 1342-1350.	Not clinical metabolic diseases
160<br>4	Sun, C., Gao, H., Zhang, Y., Pei, L., & Huang, Y. (2021). Risk stratification for organ/space surgical site infection in advanced digestive system cancer. Frontiers in Oncology, 11, 705335.	Not clinical metabolic diseases
160<br>5	Nair P M K, Jyothi N. Role of acupuncture and fire cupping in reducing the thyroxine dose and improving the thyroid function in hypothyroidism patients: a case series[J]. Journal of Acupuncture and Meridian Studies, 2021, 14(5): 200-205.	Not clinical metabolic diseases
160<br>6	Sinsen, J. (2010). Role of homeopathy in managing male and female infertility. European Journal of Integrative Medicine, 2(4), 259.	Not clinical metabolic diseases
160<br>7	Wu R, Wang L, Kuo HD, et al. An update on current therapeutic drugs treating COVID-19. Curr Pharmacol Rep. 2020;6(3):56-70. doi:10.1007/s40495-020-00216-7.	Not clinical metabolic diseases
160<br>8	Chan LY, Chiu PY, Lau TK. Herbal medicines used during pregnancy: a review. Gynecol Obstet Invest. 2011;71(3):109-114. doi:10.1159/000316055.	Not clinical metabolic diseases

1609	Zhang, H., Cao, Y., Pei, H., Wang, H., Ma, L., Wang, Z., ... & Li, H. (2020). Shenmayizhi formula combined with ginkgo extract tablets for the treatment of vascular dementia: a randomized, double-blind, controlled trial. Evidence-Based Complementary and Alternative Medicine, 2020(1), 8312347.	Not clinical metabolic diseases
1610	Li FF, Yam JC, Zhang Y, et al. Short-term effects of low-concentration atropine eye drops on choroidal and retinal structures in myopic children. Sci Rep. 2020;10(1):12756. doi:10.1038/s41598-020-69689-9.	Not clinical metabolic diseases
1611	Zhang, N., Li, C., Guo, Y., & Wu, H. C. (2020). Study on the Intervention Effect of Qi Gong Wan Prescription on Patients with Phlegm-Dampness Syndrome of Polycystic Ovary Syndrome Based on Intestinal Flora. Evidence-Based Complementary and Alternative Medicine, 2020(1), 6389034.	Not clinical metabolic diseases
1612	Lázaro-Martínez JL, García-Morales E, Beneit-Montesinos JV, et al. Efficacy of a novel hydrogel containing Olea europaea leaf extract in skin ulcers: A randomized controlled trial. Int Wound J. 2020;17(2):353-362. doi:10.1111/iwj.13299.	Not clinical metabolic diseases
1613	Wang, Z. Q., Lyu, J., Li, J. B., & Xie, Y. M. (2022). Systematic review and Meta-analysis of efficacy and safety of Ningmitai Capsules in treatment of urinary tract infection. Zhongguo Zhong yao za zhi= Zhongguo Zhongyao Zazhi= China Journal of Chinese Materia Medica, 47(13), 3648-3657.	Not clinical metabolic diseases
1614	Takayama, S., & Iwasaki, K. (2017). Systematic review of traditional Chinese medicine for geriatrics. Geriatrics & Gerontology International, 17(5), 679-688.	Not clinical metabolic diseases
1615	Drucker AM, Wang AR, Li WQ, et al. The burden of atopic dermatitis: Summary of a report for the National Eczema Association. J Invest Dermatol. 2017;137(1):26-30. doi:10.1016/j.jid.2016.07.012.	Not clinical metabolic diseases
1616	Xu, R. (2023). TCM and western medicine: diagnosis and treatment of novel coronavirus pneumonia, a case study. Alternative Therapies in Health and Medicine, 29(2), 90-96.	Not clinical metabolic diseases
1617	Riediger, C., Haschke, M., Bitter, C., Fabbro, T., Schaeren, S., Urwyler, A., & Ruppen, W. (2015). The analgesic effect of combined treatment with intranasal S-ketamine and intranasal midazolam compared with morphine patient-controlled analgesia in spinal surgery patients: a pilot study. Journal of pain research, 87-94.	Not clinical metabolic diseases
1618	Wang X, You H, Liu Y, et al. HDL-C and risk of cirrhosis in obese patients with chronic hepatitis B. Clin Res Hepatol Gastroenterol. 2021;45(5):101614. doi:10.1016/j.clinre.2020.10.007.	Not clinical metabolic diseases
1619	Hokayem M, Blond E, Vidal H, et al. Grape seed extract supplementation reduces features of metabolic syndrome: a randomized, double-blind, placebo-controlled trial. J Nutr Biochem. 2013;24(6):1073-1078. doi:10.1016/j.jnutbio.2012.08.003.	Not clinical metabolic diseases

1620	Zhang, H., Chen, H., Pei, H., Wang, H., Ma, L., & Li, H. (2022). The Effect of Guilingji Capsules on Vascular Mild Cognitive Impairment: A Randomized, Double-Blind, Controlled Trial. Evidence-Based Complementary and Alternative Medicine, 2022(1), 4778163.	Not clinical metabolic diseases
1621	Wang, Y., Liu, P., Fang, Y., Tian, J., Li, S., Xu, J., ... & Li, Y. (2020). The effect of long-term traditional Chinese medicine treatment on survival time of colorectal cancer based on propensity score matching: A retrospective cohort study. Evidence-Based Complementary and Alternative Medicine, 2020(1), 7023420.	Not clinical metabolic diseases
1622	Ming, P, Marshall, MR, Li, H, Feng, T, Yao, Q and Yang, H, 2018. The effect of traditional chinese medicine (TCM) practice on mortality risk in a large cohort of patients on continuous ambulatory peritoneal dialysis (CAPD) from China, Journal of the American Society of Nephrology.	Not clinical metabolic diseases
1623	Lin CH, Chen PK, Chang YC, et al. The effects of anti-dementia and nootropic treatments on mortality in patients with dementia: A nationwide cohort study. Alzheimers Res Ther. 2020;12(1):111. doi:10.1186/s13195-020-00677-1.	Not clinical metabolic diseases
1624	Kianbakht S, Hashem Dabaghian F. The efficacy and safety of oral bitter melon (Momordica charantia L.) in primary knee osteoarthritis: A randomized controlled trial. Phytomedicine. 2013;20(6):568-572. doi:10.1016/j.phymed.2012.12.002.	Not clinical metabolic diseases
1625	Natarajan, S., Anbarasi, C., Sathiyarajeswaran, P., Manickam, P., Geetha, S., Kathiravan, R., ... & Balaji, P. (2020). The efficacy of Siddha Medicine, Kabasura Kudineer (KSK) compared to Vitamin C & Zinc (CZ) supplementation in the management of asymptomatic COVID-19 cases: A structured summary of a study protocol for a randomised controlled trial. Trials, 21, 1-2.	Not clinical metabolic diseases
1626	Kardos P, Malek FA. Clinical evidence of the therapeutic effectiveness of ivy leaf extract in cough therapy. Forsch Komplementmed. 2017;24(1):26-30. doi:10.1159/000455729.	Not clinical metabolic diseases
1627	Shoda, J., Matsuzaki, Y., Tanaka, N., Miyamoto, J., & Osuga, T. (1996). The inhibitory effects of dai-chai-hu-tang (dai-saiko-to) extract on supersaturated bile formation in cholesterol gallstone disease. The American journal of gastroenterology, 91(4), 828-830.	Not clinical metabolic diseases
1628	Park, J., Choi, T. J., Kang, K. S., & Choi, S. H. (2021). The interrelationships between intestinal permeability and phlegm syndrome and therapeutic potential of some medicinal herbs. Biomolecules, 11(2), 284.	Not clinical metabolic diseases
1629	Pavan, V., Mucignat-Caretta, C., Redaelli, M., Ribaudo, G., & Zagotto, G. (2015). The old made new: natural compounds against erectile dysfunction. Archiv der Pharmazie, 348(9), 607-614.	Not clinical metabolic diseases

1630	Mylod, E., O'Connell, F., Donlon, N. E., Butler, C., Reynolds, J. V., Lysaght, J., & Conroy, M. J. (2021). The omentum in obesity-associated cancer: a hindrance to effective natural killer cell migration towards tumour which can be overcome by CX3CR1 antagonism. Cancers, 14(1), 64.	Not clinical metabolic diseases
1631	Rørvik HD, Styrkårsdottir S, Rashid A, et al. Prevalence of hemorrhoids and their risk factors in a large cohort of patients undergoing colonoscopy. Colorectal Dis. 2016;18(8):O336-O343. doi:10.1111/codi.13392.	Not clinical metabolic diseases
1632	Mok MY, Ip WK, Lee LH, et al. Long-term outcome of biopsy-proven lupus nephritis: a single center cohort. Lupus. 2009;18(12):1053-1059. doi:10.1177/0961203309105687.	Not clinical metabolic diseases
1633	Ogut, E., Armagan, K., & Gül, Z. (2022). The role of syringic acid as a neuroprotective agent for neurodegenerative disorders and future expectations. Metabolic Brain Disease, 37(4), 859-880.	Not clinical metabolic diseases
1634	Kennedy DA, Lupattelli A, Koren G, et al. Herbal medicine use in pregnancy: results of a multinational study. BMC Complement Altern Med. 2013;13:355. doi:10.1186/1472-6882-13-355.	Not clinical metabolic diseases
1635	Zhang, J., Cao, X., Yin, G., Xu, J., Zhu, M., Zhang, Y., ... & Ye, J. (2022). The significance of better utilization of patients' preoperative i	Not clinical metabolic diseases
1636	Xiong, H., Chen, H. S., Du, M. L., Li, Y. H., Ma, H. M., Su, Z., & Chen, Q. L. (2015). Therapeutic effects of growth hormone combined with low-dose stanozolol on growth velocity and final height of girls with Turner syndrome. Clinical Endocrinology, 83(2), 223-228.	Not clinical metabolic diseases
1637	Pahari, P. K., Vyas, S., Aman, S., Singh, U., Singh, K. P., Tiwari, R., & Dhanawat, M. (2022). Therapeutic options for the treatment of 2019-novel Coronavirus in India: a review. Coronaviruses, 3(2), 39-47.	Not clinical metabolic diseases
1638	de Oliveira GL, Broering MF, Savi MG, et al. Therapeutic plants with immunoregulatory activity and their applications: A scientific vision of traditional medicine in times of COVID-19. Front Pharmacol. 2020;11:582323. doi:10.3389/fphar.2020.582323.	Not clinical metabolic diseases
1639	West AC, Johnstone RW. New and emerging HDAC inhibitors for cancer treatment. J Clin Invest. 2014;124(1):30-39. doi:10.1172/JCI69738.	Not clinical metabolic diseases
1640	Post RM, Ketter TA, Uhde TW, et al. Thirty years of clinical experience with carbamazepine in the treatment of bipolar disorder. J Affect Disord. 2007;95(1-3):15-24. doi:10.1016/j.jad.2006.10.022.	Not clinical metabolic diseases
1641	Cohen-Lehman J, Dahl P, Ward L, et al. Thyrotoxicosis caused by thyroid hormone contamination of a dietary supplement: A case series and review of the literature. Thyroid. 2011;21(7):741-747. doi:10.1089/thy.2010.0441.	Not clinical metabolic diseases
1642	Towheed TE. Analgesic efficacy of topical nonsteroidal anti-inflammatory drugs in the treatment of osteoarthritis: a meta-analysis. CMAJ. 1997;156(7): 945-950.	Not clinical metabolic diseases

1643	Li X, Ma X, Tian L, et al. Total glucosides of paeony reduce hepatotoxicity caused by methotrexate and leflunomide combination therapy for rheumatoid arthritis. Int Immunopharmacol. 2014;18(2):247-252. doi:10.1016/j.intimp.2013.12.015.	Not clinical metabolic diseases
1644	He W, Han H, Wang W, et al. Chinese medicine and its active compounds for the treatment of hepatitis B virus infection. J Ethnopharmacol. 2021;268:113575. doi:10.1016/j.jep.2020.113575.	Not clinical metabolic diseases
1645	Yang, S., Che, H., Xiao, L., Zhao, B., & Liu, S. (2021). Traditional Chinese medicine on treating myelosuppression after chemotherapy: A protocol for systematic review and meta-analysis. Medicine, 100(4), e24307.	Not clinical metabolic diseases
1646	Wang, Z., Chen, Z., Fan, Z., & Jiang, Y. (2021). Traditional Chinese medicine on treating splenomegaly due to portal hypertension in cirrhosis: A protocol for systematic review and meta-analysis. Medicine, 100(1), e24081.	Not clinical metabolic diseases
1647	Nakae, H., Irie, Y., Satoh, K., Kitamura, T., Kameyama, K., Nara, T., & Okuyama, M. (2021). Treatment for tetanus applying Kampo medicine: Administration of shakuyakukanzoto. Traditional & Kampo Medicine, 8(2), 130-137.	Not clinical metabolic diseases
1648	Mirzapour, M., Mojahedi, M., Shokri, J., Khafri, S., & Memariani, Z. (2019). Treatment of patients with refractory functional dyspepsia using nardostachys jatamansi (D. Don) DC. hydroalcoholic extract: a case series. Traditional and Integrative Medicine.	Not clinical metabolic diseases
1649	Smagin, M. A., Shumkov, O. A., Soluianov, M. I., Demura, A. U., Smagin, A. A., Lykov, A. P., & Nimaev, V. V. (2020). Treatment of torpid trophic ulcers in patients of the older age group. Advances in Gerontology= Uspekhi Gerontologii, 33(2), 373-378.	Not clinical metabolic diseases
1650	Fengquan, X., & Yiling, W. (2007). Treatment using Western medicine and traditional Chinese medicine in hormone-dependent myasthenia gravis. Clinical Practice, 4(1), 73.	Not clinical metabolic diseases
1651	Santos HO, Price JC. Tribulus terrestris and erectile dysfunction: A systematic review of randomized clinical trials. Complement Ther Med. 2020;50:102392. doi:10.1016/j.ctim.2020.102392.	Not clinical metabolic diseases
1652	Gao R, Xu B, Lansky AJ, et al. A randomized comparison of a paclitaxel-coated balloon catheter and a paclitaxel-eluting stent for the treatment of drug-eluting stent restenosis in China: Results from the PEPCAD China ISR trial. JACC Cardiovasc Interv. 2015;8(5):632-641. doi:10.1016/j.jcin.2015.02.015.	Not clinical metabolic diseases
1653	Sen M, Walsh DA. The role of alternative therapies in the management of rheumatoid arthritis: A review. Clin Exp Rheumatol. 2021;39(5):921-930.	Not clinical metabolic diseases
1654	Steele ML, Axtner J, Happe A, et al. Safety of intratumoral mistletoe therapy in oncology: a phase I dose-escalation study. BMC Complement Altern Med. 2014;14:132. doi:10.1186/1472-6882-14-132.	Not clinical metabolic diseases

1655	Barroso KF, Lima MC, Barbosa FT, et al. Interventions for enuresis in children and adolescents: an overview of Cochrane systematic reviews. Cochrane Database Syst Rev. 2019;2019(10):CD013365. doi:10.1002/14651858.CD013365.	Not clinical metabolic diseases
1656	Futier E, Lefrant JY, Guinot PG, et al. Effect of discontinuation of angiotensin-converting enzyme inhibitors and angiotensin II receptor blockers on postoperative outcomes: a pragmatic multicenter randomized trial. JAMA Intern Med. 2017;177(7):996-1004. doi:10.1001/jamainternmed.2017.0826.	Not clinical metabolic diseases
1657	Wang, Y., Wang, X., Fu, H., Kou, S., Huang, D., Shen, Z., ... & Wang, Z. (2022). Yunnan Baiyao Adjuvant Treatment for Patients with Hemoptysis: A Systematic Review and Meta-Analysis. Evidence-Based Complementary and Alternative Medicine, 2022(1), 4931284.	Not clinical metabolic diseases
1658	Zhouji, Z. H. A. N. G., Ming, Z. H. A. N. G., Xiaoting, W. U., Qing, C. U. I., & Yijun, G. U. O. (2021). Zhengyuan capsule (...) for the treatment of cancer-related fatigue in lung cancer patients undergoing operation: a study protocol for a randomized controlled trial. Journal of Traditional Chinese Medicine, 41(3).	Not clinical metabolic diseases
1659	Andersson KE, Yoshimura N. α1-Adrenoceptors and the lower urinary tract: physiology and pharmacology. Handb Exp Pharmacol. 2009;(194):321-347. doi:10.1007/978-3-540-79090-7_8.	Not clinical metabolic diseases
1660	杨静芝.艾灸联合湿润烧伤膏治疗压力性损伤护理研究[J].新中医,2021,53(19):216-220.DOI:10.13457/j.cnki.jncm.2021.19.050.	Not clinical metabolic diseases
1661	田海燕，张志辉，王惠英，杨胜, 2015. 胺碘酮注射液诱发支气管哮喘发作 1 例，心理医生.	Not clinical metabolic diseases
1662	侯博,张佩青.张佩青教授辨证治疗汗证验案举隅[J].中医临床研究,2022,14(08):118-121.	Not clinical metabolic diseases
1663	任婕,翟志光,张振忠.补脾益肾、化瘀降浊法治疗肾性蛋白尿验案举隅[J].中医临床研究,2015,7(06):14-17.	Not clinical metabolic diseases
1664	赵红霞.参松养心胶囊与乙胺碘夫酮联合治疗室性早博 76 例疗效观察[J].中国中医基础医学杂志,2010,16(06):539.DOI:10.19945/j.cnki.issn.1006-3250.2010.06.043.	Not clinical metabolic diseases
1665	许成,郑俏丽,王永东,等.类风湿关节炎合并持久性隆起性红斑 1 例[C]//浙江省医学会皮肤病学分会,浙江省医师协会皮肤科医师分会,浙江省性病艾滋病防治协会性病临床专业委员会.2018 年浙江省医学会皮肤病学分会暨浙江省医师协会皮肤科医师分会学术年会论文汇编.浙江大学医学院附属邵逸夫医院;,2018:236.DOI:10.26914/c.cnkihy.2018.028774.	Not clinical metabolic diseases
1666	张红梅,段逸群,陈金波.除湿胃苓汤加减治疗局限型大疱性类天疱疮 1 例[J].皮肤病与性病,2019,41(04):523.	Not clinical metabolic diseases

1667	庞国明,曹秋平,李鹏辉,等.纯中药“辨病-辨证-辨体诊疗模式”治疗 2 型糖尿病患者 546 例临床特征分析——一项真实世界回顾性研究[J].中医杂志,2022,63(18):1766-1772.DOI:10.13288/j.11-2166/r.2022.18.012.	Not clinical metabolic diseases
1668	庞国明,曹秋平,李鹏辉,等.纯中药“辨病-辨证-辨体诊疗模式”治疗 2 型糖尿病患者 546 例临床特征分析——一项真实世界回顾性研究[J].中医杂志,2022,63(18):1766-1772.DOI:10.13288/j.11-2166/r.2022.18.012.	Not clinical metabolic diseases
1669	王海升.单硝酸异山梨酯注射液致过敏性哮喘 1 例[J].临床合理用药杂志,2014,7(19):98.DOI:10.15887/j.cnki.13-1389/r.2014.19.082.	Not clinical metabolic diseases
1670	余晖,高建忠.癫狂梦醒汤治疗皮肤病合并有失眠、便秘 2 例[J].浙江中医杂志,2018,53(09):692.DOI:10.13633/j.cnki.zjtcm.2018.09.053.	Not clinical metabolic diseases
1671	王宇,刘学奎,谢粱震,等.多囊卵巢综合征不同中医证型不孕症患者基线特征分析[J].中华中医药杂志,2021,36(07):4318-4324.	Not clinical metabolic diseases
1672	任亚茹,徐大荣.厄贝沙坦过敏致严重湿疹 1 例[J].中国执业药师,2010,7(02):14.	Not clinical metabolic diseases
1673	徐鹏,李秀荣.厄洛替尼导致眼睫毛异常生长一例报告并文献复习[J].中华肿瘤防治杂志,2015,22(23):1848-1849+1855.DOI:10.16073/j.cnki.cjcpt.2015.23.013.	Not clinical metabolic diseases
1674	王燕,张冬.反复电除颤成功抢救乌头碱中毒 1 例报告[J].医学理论与实践,2021,34(18):3303-3304.DOI:10.19381/j.issn.1001-7585.2021.18.090.	Not clinical metabolic diseases
1675	杨柳,马迎民.肺豚鼠耳炎诺卡菌病 1 例[J].中华老年多器官疾病杂志,2016,15(09):697-699.	Not clinical metabolic diseases
1676	王宇阳,刘宏潇,赵亚男,等.冯兴华治疗皮肌炎伴发热验案 1 则[J].北京中医药,2019,38(06):616-617.DOI:10.16025/j.1674-1307.2019.06.030.	Not clinical metabolic diseases
1677	徐琳. (2016). 复方甘草酸苷联合丹参酮 IIA 治疗继发于皮炎湿疹的红皮病的临床分析. 中国伤残医学, (6), 36-37.	Not clinical metabolic diseases
1678	吴波.高龄女性子宫穿孔误诊为上消化道穿孔 1 例分析[J].中国医学创新,2012,9(19):160.	Not clinical metabolic diseases
1679	石二霞,云来运,李恒善.谷红注射液致呼吸困难和球结膜水肿 1 例[J].医药导报,2021,40(10):1441-1442.	Not clinical metabolic diseases
1680	王传池.冠心病不同发展阶段中医证演变规律研究[D].湖北中医药大学,2020.DOI:10.27134/d.cnki.ghbzc.2020.000018.	Not clinical metabolic diseases

1681	熊敏利,晏玟.光子治疗仪结合中药湿热敷治疗胰岛素注射所致皮下硬结的疗效观察[J].激光杂志,2017,38(01):166-168.DOI:10.14016/j.cnki.jgzz.2017.01.166.	Not clinical metabolic diseases
1682	申子龙,郑桂敏,孟元,等.桂枝芍药知母汤加减治疗RS3PE综合征一例[J].环球中医药,2021,14(11):2074-2075.	Not clinical metabolic diseases
1683	吴元兴,吴玉娇,关义健,等.海南省乐东县类鼻疽病的临床及微生物特征[J].中国热带医学,2021,21(09):907-911.DOI:10.13604/j.cnki.46-1064/r.2021.09.19.	Not clinical metabolic diseases
1684	刘敏肖,唐进松,李学永.红花注射液致过敏性休克 1 例[J].解放军药学学报,2018,34(05):477.	Not clinical metabolic diseases
1685	周发伟, 郑春艳, 王在平, 尹宁, 彭绪东, 李德忠,2020. 湖北恩施少数民族地区 66 例新型冠状病毒（2019-nCoV）肺炎患者临床特征分析, 中华急诊医学杂志	Not clinical metabolic diseases
1686	周淼,何延忠,闫五玲,等.基于尸体解剖检验病理探讨新型冠状病毒肺炎危重型病因病机[J].河南中医,2020,40(06):822-824.DOI:10.16367/j.issn.1003-5028.2020.06.0207.	Not clinical metabolic diseases
1687	孙广瀚,刘健,龙琰,等.基于数据挖掘分析中医健脾单元疗法对痛风患者代谢指标的影响及其干预研究[J].中国临床保健杂志,2020,23(02):182-187.	Not clinical metabolic diseases
1688	俞芳,杨宁,邱国萍.急进性肾小球肾炎 1 例报告[J].九江学院学报(自然科学版),2006,(04):58.DOI:10.19717/j.cnki.jjun.2006.04.025.	Not clinical metabolic diseases
1689	吴四迎,程冕.急性心肌梗死误诊为上呼吸道感染 1 例[J].淮海医药,2009,27(04):306.	Not clinical metabolic diseases
1690	张宇红.加减蛇床子汤坐浴治疗外阴瘙痒症 66 例临床观察[J].基层医学论坛,2003,(11):1041.	Not clinical metabolic diseases
1691	张梅. (2015). 加减五积散治疗痰湿肥胖型多囊卵巢综合征 23 例临床分析. 中外女性健康研究, (23), 14-14.	Not clinical metabolic diseases
1692	薛宇爽.结肠息肉复发危险因素的巢式病例对照研究[D].山东中医药大学,2021.DOI:10.27282/d.cnki.gsdzu.2021.000335.	Not clinical metabolic diseases
1693	郑浩翔.近 12 年中国补肾化痰中药治疗多囊卵巢综合征临床文献的Meta分析[D].广州中医药大学,2020.DOI:10.27044/d.cnki.ggzzu.2020.000740.	Not clinical metabolic diseases
1694	牟雪梅,牟效文.静推及静滴心律平致心脏停搏 1 例抢救与护理[J].医药产业资讯,2005,(09):103.	Not clinical metabolic diseases

1695	王贤彬,卢和林,谢吉,等.康复新液治疗小儿II度烫伤35例疗效观察[J].现代医药卫生,2009,25(20):3112.	Not clinical metabolic diseases
1696	徐晖.老年Dieulafoy病致上消化道出血 1 例分析[J].中国误诊学杂志,2009,9(25):6282.	Not clinical metabolic diseases
1697	向日晖,韦志武,郭卫华,等.老年慢性疾病患者难愈性创面非手术治疗效果探讨[J].中国烧伤创疡杂志,2020,32(02):88-91.	Not clinical metabolic diseases
1698	王君珺,帅嫄露,李嘉,等.雷珠单抗联合复方血栓通与单独雷珠单抗治疗渗出老年性黄斑变性的前瞻性随机对照试验研究[J].现代生物医学进展,2019,19(20):3982-3986+3992.DOI:10.13241/j.cnki.pmb.2019.20.041.	Not clinical metabolic diseases
1699	吴熙培,聂克.六君子汤治疗神经性厌食研究进展[J].中国药理学与毒理学杂志,2021,35(10):799.	Not clinical metabolic diseases
1700	玄令美,陈爱华,张蓉笑,等.马应龙麝香痔疮膏治疗足部慢性伤口 1 例的护理[J].中国乡村医药,2020,27(19):66-67.DOI:10.19542/j.cnki.1006-5180.004508.	Not clinical metabolic diseases
1701	王丽萍,周瑶,刘卫.慢性咳嗽治验 1 例报告[J].基层医学论坛,2015,19(09):1149+1296.	Not clinical metabolic diseases
1702	郑玮清,王华军,周东.慢性淋巴细胞性白血病引起红皮病 1 例[J].中国麻风皮肤病杂志,2008,(07):574-575.	Not clinical metabolic diseases
1703	张晶, 2019. 美宝湿润烧伤膏联合复方黄柏液治疗 1 例皮肤溃疡体会, 医药前沿	Not clinical metabolic diseases
1704	殷松江.尿毒清颗粒灌肠治疗CKD4-5 期患者临床疗效观察[D].南京中医药大学,2016.	Not clinical metabolic diseases
1705	王华菊,叶润英,黎小斌.盆腔器官脱垂传统术式术后尿失禁的相关因素分析及围手术期中医药治疗探讨[J].广州中医药大学学报,2021,38(11):2318-2322.DOI:10.13359/j.cnki.gzxbtcm.2021.11.004.	Not clinical metabolic diseases
1706	王彩燕,颜世锐,熊辉.凶险的泌尿系感染：急诊气肿性肾盂肾炎 1 例[J].中国急救医学,2025,45(03):272-275.	Not clinical metabolic diseases
1707	谢吟灵.清脉汤加减辅助治疗肥胖型胸痹心痛临床研究[J].吉林中医药,2008,(04):266-267.DOI:10.13463/j.cnki.jlzyy.2008.04.041.	Not clinical metabolic diseases
1708	夏梦筠. (2021). 清热化浊利湿法治疗慢性肾炎水肿 1 例. 医师在线, 11(12), 43-43.	Not clinical metabolic diseases

1709	张景祖.清热利湿化瘀汤治疗小儿急性肾炎随机平行对照研究[J].实用中医内科杂志,2016,30(09):82-84.DOI:10.13729/j.issn.1671-7813.2016.09.31.	Not clinical metabolic diseases
1710	王栋,高宇,张佳,等.肾癌术后患者 145 例的中医证候类型及体质分布规律[J].中华中医药杂志,2021,36(05):2960-2963.	Not clinical metabolic diseases
1711	武继平.湿润烧伤膏配合芒硝治疗腹部切口脂肪液化 25 例临床分析[J].山西医药杂志,2013,42(02):190-191.	Not clinical metabolic diseases
1712	余振亚,许秋荣,杨莉晖,等.湿润烧伤膏配合微波治疗鼻中隔黏膜出血疗效观察[J].河北医药,2007,(09):1030.	Not clinical metabolic diseases
1713	王立新. 湿润烧伤膏治疗慢性溃疡疗效观察[J]. 现代中西医结合杂志, 2009, 18(4): 383-384.	Not clinical metabolic diseases
1714	袁学刚,张昱,张平.手术、注射并结合中药外洗治疗肛周顽固性瘙痒 574 例[C]//中华中医药学会肛肠分会.中华中医药学会第十二次大肠肛门病学术会议论文汇编.四川成都中医药大学;成都肛肠专科医院;,2006:490-492.	Not clinical metabolic diseases
1715	宋凤韬.手术结合中药纱条外敷治疗肛周瘙痒症 45 例报告[J].中国社区医师(医学专业),2011,13(33):122.	Not clinical metabolic diseases
1716	于金源,刘铭珍.孙氏理饮汤治疗肺心病急性发作期患者 70 例临床观察[J].中国中医药科技,2009,16(01):71-72.	Not clinical metabolic diseases
1717	彭昌乐,侯丽辉,付明俊.痰湿型多囊卵巢综合征患者遗传因素和生活方式的分析[J].医学研究杂志,2014,43(12):37-39.	Not clinical metabolic diseases
1718	秦海洸.唐汉钧从脾虚湿热辨治慢性难愈性疮疡验案 2 则[J].江苏中医药,2009,41(09):46-47.	Not clinical metabolic diseases
1719	赵霞,马佐英,王学岭.王学岭从湿热论治不育症验案 2 则[J].湖南中医杂志,2014,30(07):117-118.DOI:10.16808/j.cnki.issn1003-7705.2014.07.060.	Not clinical metabolic diseases
1720	许楷斯,许华,罗文,等.温阳活血化湿法治疗胆道闭锁手术后患儿的临床疗效分析[J].中医药导报,2016,22(02):61-64.DOI:10.13862/j.cnki.cn43-1446/r.2016.02.024.	Not clinical metabolic diseases
1721	庞颖,刘金凤,江紫曦,等.斡旋中州方治疗肥胖型多囊卵巢综合征伴胰岛素抵抗的前瞻性临床观察[J].中国医药导报,2020,17(26):124-128.DOI:10.20047/j.issn1673-7210.2020.26.030.	Not clinical metabolic diseases
1722	宓金凤,李丕宝,李玫,等.乌头碱中毒致心脏骤停 1 例[J].疑难病杂志,2007,(12):754.	Not clinical metabolic diseases

1723	王弘略,任建军.先天性膈疝误诊下纵隔脂肪瘤 1 例[J].中国误诊学杂志,2001,(08):1268-1269.	Not clinical metabolic diseases
1724	孙跃明.消炎利胆汤联合西药治疗肝胆湿热型慢性胆囊炎 41 例[J].中西医结合肝病杂志,2018,28(01):48-49.	Not clinical metabolic diseases
1725	杨雨齐.新冠肺炎康复期患者的中医临床特征、证候及其预后影响因素的研究[D].广州中医药大学,2021.DOI:10.27044/d.cnki.ggzzu.2021.000967.	Not clinical metabolic diseases
1726	闫蓓,何召国.胸闷憋气腹痛[J].医师进修杂志,2004,(01):57-58.	Not clinical metabolic diseases
1727	孙素华,张俊远,刘青菊,等.宣上畅中渗下法治疗痰湿质慢性支气管炎临床分析[J].中国中医药现代远程教育,2018,16(17):98-101.	Not clinical metabolic diseases
1728	汪郭亮,郭丽英.寻常型天疱疮合并糖尿病 1 例的护理[J].中国误诊学杂志,2011,11(31):7722.	Not clinical metabolic diseases
1729	姚仑,张顺英.药物性肝炎 226 例临床分析[J].中西医结合肝病杂志,2015,25(05):305-306.	Not clinical metabolic diseases
1730	林良庆,傅平.结肠脂肪瘤合并肠套叠 1 例[J].江西医药,2018,53(08):832-833.	Not clinical metabolic diseases
1731	张晓琳,孔德立,冯志宏,等.益气活血法治疗减肥后黄褐斑 1 例[J].吉林中医药,2007,(11):46.DOI:10.13463/j.cnki.jlzyy.2007.11.027.	Not clinical metabolic diseases
1732	王静巍,宋卫国,赵纪生.赵纪生从风湿论治慢性肾炎验案 1 则[J].湖南中医杂志,2020,36(11):101.DOI:10.16808/j.cnki.issn1003-7705.2020.11.039.	Not clinical metabolic diseases
1733	钱丽欢.针灸治疗多囊卵巢综合征的现代中文期刊文献研究[D].广州中医药大学,2016.	Not clinical metabolic diseases
1734	余翔.针药并用治疗闭经验案一则体会[J].中国民族民间医药,2013,22(03):107.	Not clinical metabolic diseases
1735	赵建新,宋昱慧,田元祥.针药并治雷诺综合征伴溃烂医案 1 则[J].中国民间疗法,2017,25(07):64-65.DOI:10.19621/j.cnki.11-3555/r.2017.07.050.	Not clinical metabolic diseases
1736	乌仁高娃.珍宝丸治愈甲状腺多发性结节瘤 1 例[J].中国民族民间医药杂志,2006,(04):247.	Not clinical metabolic diseases

1737	万刚峰,万荣梅,王艳玲.炙甘草汤加味治疗心悸 52 例[J].中国民间疗法,2011,19(06):37.DOI:10.19621/j.cnki.11-3555/r.2011.06.039.	Not clinical metabolic diseases
1738	张楠,郭静波.中西医治疗RS3PE综合征 1 例[J].风湿病与关节炎,2016,5(09):60-62.	Not clinical metabolic diseases
1739	孙红,彭文君.中西医结合治疗肥胖型多囊卵巢综合征 51 例疗效观察[J].新疆中医药,2012,30(03):47-49.	Not clinical metabolic diseases
1740	文学兰,谢文.中西医结合治疗酒精性心脏病 1 例[J].世界最新医学信息文摘,2019,19(20):273.DOI:10.19613/j.cnki.1671-3141.2019.20.156.	Not clinical metabolic diseases
1741	梁涛.中西医结合治疗类风湿性关节炎疗效观察[J].中医临床研究,2014,6(05):99-100.	Not clinical metabolic diseases
1742	张文亮, 2007, 中西医结合治疗慢性心力衰竭 38 例, 中国民间疗法	Not clinical metabolic diseases
1743	张楠,郭静波.中西医治疗RS3PE综合征 1 例[J].风湿病与关节炎,2016,5(09):60-62.	Not clinical metabolic diseases
1744	郑源, 陈可冀, 马晓昌, 等. 中药联合超滤治疗慢性心力衰竭 1 例[J]. 中西医结合心脑血管病杂志, 2020, 18(2): 366-367.	Not clinical metabolic diseases
1745	唐维居.中药治愈子宫颈癌 1 例[J].中外妇儿健康,2011,19(02):79.	Not clinical metabolic diseases
1746	虞芳,杨蒋伟,邢葆平,等.中医药治疗抗精神病药物不良反应验案举隅[J].中国现代医生,2021,59(34):140-143.	Not clinical metabolic diseases
1747	石长春.中医治疗心悸验案一则[J].中国民间疗法,2008,(06):60.DOI:10.19621/j.cnki.11-3555/r.2008.06.063.	Not clinical metabolic diseases
1748	王冬颖,张晓丽,孙卫卫,等.蛛网膜下腔出血合并心肾综合征 1 例[J].中国循证心血管医学杂志,2018,10(09):1136+1141.	Not clinical metabolic diseases
1749	叶永火,谢琦,窦志.综合性二尖瓣修复技术在风湿性二尖瓣病变中的应用(附 16 例报告)[J].福建医药杂志,2019,41(06):5-7.DOI:10.20148/j.fmj.2019.06.006.	Not clinical metabolic diseases
1750	Descazeaud A, Ruffion A, Delongchamps NB, et al. Critical analysis of the effect of Permixon in the treatment of BPH. Prog Urol. 2012;22(1):17-24. doi:10.1016/j.purol.2011.09.015.	Not clinical metabolic diseases

1751	Ma, Y., & Sun, W. (2020). Effects of Dengzhan Shengmai Capsule combined with butylphthalide soft capsule on oxidative stress indexes and serum Hcy and CRP levels in patients with vascular dementia. Cellular and Molecular Biology, 66(6), 8-14.	Not clinical metabolic diseases
1752	Ma, Q., Xie, Y., Wang, Z., Lei, B., Chen, R., Liu, B., ... & Yang, Z. (2021). Efficacy and safety of ReDuNing injection as a treatment for COVID-19 and its inhibitory effect against SARS-CoV-2. Journal of Ethnopharmacology, 279, 114367.	Not clinical metabolic diseases
1753	Ma, G., Huang, L., Wu, M., Wang, Y., Lu, C., & Zha, Y. (2021). Effect of Shenkang injection on TGF-B1 level, peritoneal function and microinflammatory status in peritoneal dialysis patients with chronic renal failure. Acta Medica Mediterranea, 37(3), 1359-1363.	Not clinical metabolic diseases
1754	Lee H, Bae K, Lee J, et al. Efficacy of Chinese herbal medicine for benign prostatic hyperplasia: a systematic review. Complement Ther Med. 2012;20(5):228-236. doi:10.1016/j.ctim.2012.04.003.	Not clinical metabolic diseases
1755	Ren Y, Fan J, Li X, et al. Efficacy and safety of Chinese herbal medicine for functional constipation: A systematic review and meta-analysis. Complement Ther Med. 2021;56:102610. doi:10.1016/j.ctim.2020.102610.	Not clinical metabolic diseases
1756	Zhao Y, Sun Y, Liu J, et al. Clinical efficacy of salvia miltiorrhiza depside salt combined with aspirin in stable angina: A randomized controlled trial. Phytomedicine. 2021;80:153387. doi:10.1016/j.phymed.2020.153387.	Not clinical metabolic diseases
1757	Lyu, J., Xie, Y., Sun, M., Zhang, C., & Wang, L. (2020). Sanjin tablet combined with antibiotics for treating patients with acute lower urinary tract infections: a meta-analysis and GRADE evidence profile. Experimental and Therapeutic Medicine, 19(1), 683-695.	Not clinical metabolic diseases
1758	Lv, X. F., Wen, R. Q., Liu, K., Zhao, X. K., Pan, C. L., Gao, X., ... & Li, Y. D. (2022). Role and molecular mechanism of traditional Chinese medicine in preventing cardiotoxicity associated with chemoradiotherapy. Frontiers in Cardiovascular Medicine, 9, 1047700.	Not clinical metabolic diseases
1759	Luo, X., Zhang, Y., Li, H., Ren, M., Liu, Y., Liu, Y., ... & Ni, X. (2022). Clinical evidence on the use of Chinese herbal medicine for acute infectious diseases: an overview of systematic reviews. Frontiers in Pharmacology, 13, 752978.	Not clinical metabolic diseases
1760	Xiong X, Wang P, Su K, et al. Chinese herbal medicine as adjunctive therapy for COVID-19: A systematic review and meta-analysis. Phytomedicine. 2021;85:153282. doi:10.1016/j.phymed.2021.153282.	Not clinical metabolic diseases
1761	Wang H, Li P, Wu J, et al. Chinese herbal medicine for chronic heart failure: A multicenter, randomized, double-blind, placebo-controlled trial. ESC Heart Fail. 2020;7(1):432-443. doi:10.1002/ehf2.12556.	Not clinical metabolic diseases
1762	Shang Y, Guo Y, Liu Y, et al. Traditional Tibetan medicine: an overview of systematic reviews. Evid Based Complement Alternat Med. 2013;2013:672268. doi:10.1155/2013/672268.	Not clinical metabolic diseases

1763	Wu D, Liang Y, Pan H, et al. Chinese herbal medicine for primary Sjögren's syndrome: A systematic review of randomized controlled trials. PloS One. 2017;12(5):e0177745. doi:10.1371/journal.pone.0177745.	Not clinical metabolic diseases
1764	Dan, L., Tingting, D., Wei, Y., Hui, D., Huan, M., & Ming, J. (2019). Effects of Huangban Bianxing One decoction combined with ranibizumab on treating exudative age-related macular degeneration. Journal of Traditional Chinese Medicine, 39(6).	Not clinical metabolic diseases
1765	Lu Z, Kou W, Du B, et al. Effect of xuezhikang, an extract from red yeast Chinese rice, on coronary events in a Chinese population with previous myocardial infarction. Am J Cardiol. 2008;101(12):1689-1693. doi:10.1016/j.amjcard.2008.02.060.	Not clinical metabolic diseases
1766	Lu, Y., & Zou, Z. (2013). Retrospective analysis towards diagnosis and treatment status of acute myocardial infarction patients in Binhai Community. Journal of Acute Disease, 2(3), 236-239.	Not clinical metabolic diseases
1767	Lu, Y., Yang, Q. Q., Zhuo, L., Yang, K., Kou, H., Gao, S. Y., ... & Zhan, S. (2022). Ambroxol for the treatment of COVID-19 among hospitalized patients: A multicenter retrospective cohort study. Frontiers in Microbiology, 13, 1013038.	Not clinical metabolic diseases
1768	Yun, L., Wei, J., Hong, Z., & Xiaoyun, Z. (2016). Multicenter clinical efficacy observation of integrated Traditional Chinese Medicine-Western Medicine treatment in acute onset period of pulmonary heart disease. Journal of Traditional Chinese Medicine, 36(3), 283-290.	Not clinical metabolic diseases
1769	Singh K, Carson K, Shah R, et al. Meta-analysis of clinical correlates of acute mortality in Takotsubo cardiomyopathy. Am J Cardiol. 2014;113(8):1420-1428. doi:10.1016/j.amjcard.2014.01.438.	Not clinical metabolic diseases
1770	Lu, S. Y., Gao, J. J., Tian, J. Y., Feng, M. L., & Lin, H. (2022). Clinical study on intervention of Hedyotis diffusa on postoperative recurrence of multiple intestinal polyps of damp-heat stasis type.	Not clinical metabolic diseases
1771	Lu, C. J., Yu, J. J., & Deng, J. W. (2012). Disease-syndrome combination clinical study of psoriasis: present status, advantages, and prospects. Chinese journal of integrative medicine, 18, 166-171.	Not clinical metabolic diseases
1772	Akhlaghi M, Shabanian G, Rafieian-Kopaei M, et al. Melissa officinalis extract in the treatment of patients with mild to moderate anxiety disorders: A double blind, randomized, placebo controlled trial. Cent Eur J Med. 2013;8(1):57-64. doi:10.2478/s11536-012-0062-9.	Not clinical metabolic diseases
1773	Lotan, A. M., Gronovich, Y., Lysy, I., Binenboym, R., Eizenman, N., Stuchiner, B., ... & Oberbaum, M. (2020). Arnica montana and Bellis perennis for seroma reduction following mastectomy and immediate breast reconstruction: randomized, double-blind, placebo-controlled trial. European Journal of Plastic Surgery, 43(3), 285-294.	Not clinical metabolic diseases

1774	Losev, R. Z., Zakharova, N. B., IuA, B., Iakusheva, E. A., Nikitina, V. V., Stepanova, T. V., & Mikul'skaia, E. G. (2007). Local tissue hypoxia consequence in the trophic venous ulceration in elderly patients. Angiologiia i Sosudistaia Khirurgiia= Angiology and Vascular Surgery, 13(2), 79-83.	Not clinical metabolic diseases
1775	Long L, Soeken K, Ernst E. Herbal medicines for the treatment of osteoarthritis: a systematic review. Rheumatology (Oxford). 2001;40(7):779-793. doi:10.1093/rheumatology/40.7.779.	Not clinical metabolic diseases
1776	Lo, L. C., & Chen, Y. T. (2019). Chinese herbal medicine for preventing symptomatic UTI among high-risk elderly residing in nursing homes. Advances in Integrative Medicine, 6, S31.	Not clinical metabolic diseases
1777	Basaria S, Wisniewski A, Dupree K, et al. Effects of soy protein supplementation and exercise on serum lipids and inflammatory markers in postmenopausal women. Menopause. 2009;16(5):1043-1050. doi:10.1097/gme.0b013e31819c4f1b.	Not clinical metabolic diseases
1778	Liu, Y., Chen, X., Wang, H., Yao, C., Gou, X., Gao, Z., ... & Zhang, Y. (2022). Effectiveness and safety analysis of SanHanHuaShi granules for the treatment of coronavirus disease 2019: Study protocol and statistical analysis plan for a randomized, parallel-controlled, open-label clinical trial. Frontiers in Pharmacology, 13, 936925.	Not clinical metabolic diseases
1779	Xie L, Xu J, Sun X, et al. Apatinib for advanced soft tissue sarcoma: A multicenter, single-arm, prospective study. Oncologist. 2021;26(8):e1446-e1455. doi:10.1002/onco.13832.	Not clinical metabolic diseases
1780	De la Luz Cádiz-Gurrea M, Fernández-Arroyo S, Segura-Carretero A. Phenolic compounds: promising therapeutic agents against inflammation, neoplasms, and oxidative stress. Antioxidants (Basel). 2020;9(8):706. doi:10.3390/antiox9080706.	Not clinical metabolic diseases
1781	Xu Y, Li X, Cheng Y, et al. Efficacy of traditional Chinese medicine combined with chemotherapy and targeted therapy for metastatic colorectal cancer: A randomized, double-blind, placebo-controlled clinical trial. Integr Cancer Ther. 2019;18:1534735419850620. doi:10.1177/1534735419850620.	Not clinical metabolic diseases
1782	Liu, M., Wu, H., Yang, D., Li, F., Li, Z., Wang, S., & He, R. (2018). Effects of small-dose remifentanil combined with index of consciousness monitoring on gastroscopic polypectomy: a prospective, randomized, single-blinded trial. Trials, 19, 1-12.	Not clinical metabolic diseases
1783	Liu, M. (2015). Clinical intervention research on mental stress-induced myocardial ischemia. Zhonghua xin xue Guan Bing za zhi, 43(2), 153-156.	Not clinical metabolic diseases
1784	Yu X, He Y, Xu L, et al. Tripterygium wilfordii Hook. f. preparations for the treatment of atopic eczema: A Bayesian analysis of randomized controlled trials. Phytomedicine. 2021;87:153581. doi:10.1016/j.phymed.2021.153581.	Not clinical metabolic diseases

1785	Liu, L., Li, J. Y., Liu, C. Y., Chang, L. X., Zhang, L. H., Huang, H. L., ... & Gao, J. P. (2022). Effect of Ganshuang granule combined with entecavir on portal vein thrombosis in patients with hepatitis B cirrhosis.	Not clinical metabolic diseases
1786	Liu, K., Li, H., Duan, J., Chen, X., Yu, X., Wang, X., ... & Wang, X. (2021). Progress in clinical research on the integration of Chinese and Western medicines for treating primary liver cancer. Journal of Traditional Chinese Medical Sciences, 8(3), 173-185.	Not clinical metabolic diseases
1787	Liu, J. M., Lin, P. H., Hsu, R. J., Chang, Y. H., Cheng, K. C., Pang, S. T., & Lin, S. K. (2016). Complementary traditional Chinese medicine therapy improves survival in patients with metastatic prostate cancer. Medicine, 95(31), e4475.	Not clinical metabolic diseases
1788	Zhang Y, Zhang H, Zhao L, et al. Xiao Yao San for treatment of depression: A systematic review and meta-analysis of randomized controlled trials. Complement Ther Med. 2012;20(5):275-284. doi:10.1016/j.ctim.2012.04.003.	Not clinical metabolic diseases
1789	Liu, H., Li, Y., Yao, Y., Chen, K., & Gan, J. (2022). Meta-Analysis of Efficacy and Safety of Karelizumab Combined with Apatinib in the Treatment of Advanced Gastric Cancer. Disease markers, 2022(1), 6971717.	Not clinical metabolic diseases
1790	Chuang E, Lin CL, Tu HP, et al. Decreased risk of dementia in migraine patients with traditional Chinese medicine use: A population-based cohort study. Front Aging Neurosci. 2020;12:565642. doi:10.3389/fnagi.2020.565642.	Not clinical metabolic diseases
1791	Fan X, Chen X, Lin J, et al. Chinese herbal medicine for cardiovascular diseases: research progress and mechanism of action. Front Pharmacol. 2020;11:559889. doi:10.3389/fphar.2020.559889.	Not clinical metabolic diseases
1792	Zhao YY, Zhao Y, Sun J, et al. Antiplatelet and myocardial protective effect of Shexiang Tongxin Dropping Pill in patients undergoing PCI: A randomized controlled trial. Chin Med. 2019;14:30. doi:10.1186/s13020-019-0253-9.	Not clinical metabolic diseases
1793	Shih CC, Liao CC, Su YC, et al. Traditional Chinese medicine therapy reduces the risk of urinary incontinence among patients with chronic obstructive pulmonary disease: a population-based retrospective cohort study. BMC Complement Altern Med. 2019;19(1):126. doi:10.1186/s12906-019-2538-2.	Not clinical metabolic diseases
1794	Lin, W., Hou, J., Han, T., Zheng, L., Liang, H., & Zhou, X. (2022). Efficacy and safety of traditional Chinese medicine for intracranial hemorrhage by promoting blood circulation and removing blood stasis: A systematic review and meta-analysis of randomized controlled trials. Frontiers in Pharmacology, 13, 942657.	Not clinical metabolic diseases

1795	Chen Y, Lin Y, Liu C, et al. Conventional Western Treatment Combined With Chinese Herbal Medicine Alleviates the Progressive Risk of Lung Cancer in Patients With Chronic Obstructive Pulmonary Disease: A Nationwide Retrospective Cohort Study. Front Pharmacol. 2019;10:987. doi:10.3389/fphar.2019.00987.	Not clinical metabolic diseases
1796	Lin SK, Lin PH, Hsu RJ, Chuang HC, Liu JM. Association between Traditional Chinese Medicine and a Lower Risk of Dementia in Patients with Major Depression: A Case-Control Study. J Ethnopharmacol. 2021;278:114295. doi:10.1016/j.jep.2021.114295.	Not clinical metabolic diseases
1797	Lin SK, Lin PH, Hsu RJ, Chuang HC, Liu JM. Traditional Chinese Medicine Therapy Reduces the Catheter Indwelling Risk in Dementia Patients with Difficult Voiding Symptoms. J Ethnopharmacol. 2017;203:120-126. doi:10.1016/j.jep.2017.03.040.	Not clinical metabolic diseases
1798	Lin SK, Dailey SH, Lin PH, et al. Xiang-Sheng-PoDi-Wan May Reduce the Risk of Pneumonia in Unilateral Vocal Fold Paralysis: A Nationwide Population-Based Cohort Study. J Voice. 2023;37(6):897.e1-897.e7. doi:10.1016/j.jvoice.2023.07.010.	Not clinical metabolic diseases
1799	Lin, Q. C., Ye, Y., & Fang, S. Q. (1995). Clinical study on fungus lipid-reducing capsule in regulating lipometabolic disorder. Zhongguo Zhong xi yi jie he za zhi Zhongguo Zhongxiyi Jiehe Zazhi= Chinese Journal of Integrated Traditional and Western Medicine, 15(5), 281-283.	Not clinical metabolic diseases
1800	Lin, L. Q., Wu, B. X., Lin, M. Y., Chen, Q. X., & Xu, D. P. (2022). Interim analysis report of kuanxiong aerosol in improving angina and quality of life after percutaneous coronary intervention. World Journal of Traditional Chinese Medicine, 8(1), 87-91.	Not clinical metabolic diseases
1801	Lin, H. C., Yang, W. C. V., & Lee, H. C. (2008). Traditional Chinese medicine usage among schizophrenia patients. Complementary therapies in medicine, 16(6), 336-342.	Not clinical metabolic diseases
1802	Lin C, Pattraraachachai J, Pawa KK, Wongyai S. A preliminary study of the efficacy of the polyherbal preparation Sao Thong Tai for erectile dysfunction among elderly men: a double-blind, randomized controlled trial. Clin Phytosci. 2022;8(1):1-9. doi:10.1186/s40816-022-00341-4.	Not clinical metabolic diseases
1803	Lim, S. Y. M., Al Bishtawi, B., & Lim, W. (2023). Role of cytochrome P450 2C9 in COVID-19 treatment: current status and future directions. European Journal of Drug Metabolism and Pharmacokinetics, 48(3), 221-240.	Not clinical metabolic diseases
1804	Lim, S. Y. M., Al Bishtawi, B., & Lim, W. (2023). Role of cytochrome P450 2C9 in COVID-19 treatment: current status and future directions. European Journal of Drug Metabolism and Pharmacokinetics, 48(3), 221-240.	Not clinical metabolic diseases
1805	Liao, H. H., Yeh, C. C., Lin, C. C., Chen, B. C., Yeh, M. H., Chang, K. M., ... & Yen, H. R. (2015). Prescription patterns of Chinese herbal products for patients with fractures in Taiwan: a nationwide population-based study. Journal of ethnopharmacology, 173, 11-19.	Not clinical metabolic diseases

1806	Liao, H. H., Chen, H. T., Livneh, H., Huang, H. L., Lai, N. S., Lu, M. C., ... & Tsai, T. Y. (2023). Integration of Chinese herbal medicine into routine care was related to lower risk of chronic kidney disease in patients with rheumatoid arthritis: a population-based nested case–control study in Taiwan. Journal of Multidisciplinary Healthcare, 1191-1201.	Not clinical metabolic diseases
1807	Liang, Y. G., & Chu, X. J. (2002). Effect of compound Salvia pill combined with propanolol on liver fibrosis and portal hypertension. Zhongguo Zhong xi yi jie he za zhi Zhongguo Zhongxiyi Jiehe Zazhi= Chinese Journal of Integrated Traditional and Western Medicine, 22(5), 382-383.	Not clinical metabolic diseases
1808	Liang, Y., & Wang, L. (2022). Carthamus tinctorius L.: A natural neuroprotective source for anti-Alzheimer's disease drugs. Journal of Ethnopharmacology, 298, 115656.	Not clinical metabolic diseases
1809	Liang, L., Zhao, L., Xie, G., Li, S., Zhu, J., & Wu, Y. (2010, July). The Effect of Achieving Low-Density Lipoprotein Goals in Cardiovascular Events among Patients after 6-16 Weeks' Lipid-Lowering Therapies in China. In CIRCULATION (Vol. 122, No. 2, pp. E272-E272). 530 WALNUT ST, PHILADELPHIA, PA 19106-3621 USA: LIPPINCOTT WILLIAMS & WILKINS.	Not clinical metabolic diseases
1810	Liang, J., Wang, F., Huang, J., Xu, Y., & Chen, G. (2020). The Efficacy and Safety of Traditional Chinese Medicine Tonifying-Shen (Kidney) Principle for Primary Osteoporosis: A Systematic Review and Meta-Analysis of Randomized Controlled Trials. Evidence-Based Complementary and Alternative Medicine, 2020(1), 5687421.	Not clinical metabolic diseases
1811	Li Z, Zhang J, Qiao M, Wang X, Guo Y, Wang H. Efficacy and safety of Shugan Jieyu Decoction in the treatment of coronary heart disease complicated with depression. Medicine (Baltimore). 2023;102(11):e33176.	Not clinical metabolic diseases
1812	Li Z, Lu J, Ou J, Yu J, Lu C. Effect of Chinese herbal medicine injections for treatment of psoriasis vulgaris: a systematic review and meta-analysis. Front Pharmacol. 2023;14:1148445.	Not clinical metabolic diseases
1813	Li Y, Zhang L, Lv S, et al. Efficacy and safety of oral Guanxinshutong capsules in patients with stable angina pectoris in China: a prospective, multicenter, double-blind, placebo-controlled, randomized clinical trial. BMC Complement Altern Med. 2019;19(1):363.	Not clinical metabolic diseases
1814	Li Y, Lu J, Chen R, et al. The efficacy and safety of dupilumab for the treatment of atopic dermatitis among Chinese patients in clinical practice: a single-center retrospective study. Dermatol Ther. 2022;35(5):e15385.	Not clinical metabolic diseases
1815	Li Y, Lin W, Huang J, Xie Y, Ma W. Anti-cancer effects of Gynostemma pentaphyllum (Thunb.) Makino (Jiaogulan). Chin Med. 2016;11:43.	Not clinical metabolic diseases

1816	Li, Y., Li, W. L., Yang, L. L., Feng, H. T., & Li, H. Q. (2020). Status analysis and thinking on experimental study on efficacy of Mori Fructus in treatment of cardiovascular diseases. Zhongguo Zhong yao za zhi= Zhongguo Zhongyao Zazhi= China Journal of Chinese Materia Medica, 45(13), 3055-3062.	Not clinical metabolic diseases
1817	Li Y, Li L, Guo R, et al. Clinical efficacy of Shugan granule in the treatment of mixed anxiety-depressive disorder: a multicenter, randomized, double-blind, placebo-controlled trial. J Ethnopharmacol. 2022;290:115032.	Not clinical metabolic diseases
1818	Li X, Wang N, Liang X, et al; China Ozurdex in RVO Study Group. Safety and efficacy of dexamethasone intravitreal implant for treatment of macular edema secondary to retinal vein occlusion in Chinese patients: randomized, sham-controlled, multicenter study. Graefes Arch Clin Exp Ophthalmol. 2018;256(1):59-69.	Not clinical metabolic diseases
1819	Li X, Sun C, Zhang J, et al. Protective effects of paeoniflorin on cardiovascular diseases: a pharmacological and mechanistic overview. Front Pharmacol. 2023;14:1122969.	Not clinical metabolic diseases
1820	Li W, Dai Y, Han Y, et al. [Safety study of 52-week highly active antiretroviral therapy in 198 HIV/AIDS Chinese patients]. Zhonghua Yi Xue Za Zhi. 2011;91(19):1318-1322.	Not clinical metabolic diseases
1821	Li W, Hu H, Zou G, Ma Z, Liu J, Li F. Therapeutic effects of puerarin on polycystic ovary syndrome: a randomized trial in Chinese women. Medicine (Baltimore). 2021;100(21):e26049.	Not clinical metabolic diseases
1822	Li T, Dai Y, Kuang J, et al. Three generic nevirapine-based antiretroviral treatments in Chinese HIV/AIDS patients: multicentric observation cohort. PLoS One. 2008;3(12):e3918.	Not clinical metabolic diseases
1823	Li SR, Wang TH, Zhang BJ. [Effects of naoxintong capsule on the inflammation and prognosis in borderline lesion coronary heart disease patients]. Zhongguo Zhong Xi Yi Jie He Za Zhi. 2012;32(5):607-611.	Not clinical metabolic diseases
1824	Li P, Zhao J, Gao P, Qu H. Clinical Evaluation of Pinggan Yiqi Yangshen Recipe Combined with Labetalol Hydrochloride and Magnesium Sulfate in the Treatment of PIH. Evid Based Complement Alternat Med. 2021;2021:3135043.	Not clinical metabolic diseases
1825	Li P, Lin H, Ni Z, et al. Efficacy and safety of Abelmoschus manihot for IgA nephropathy: a multicenter randomized clinical trial. Phytomedicine. 2020;76:153231.	Not clinical metabolic diseases
1826	Li P, Dai Q, Cai P, et al. Identifying different phenotypes in takotsubo cardiomyopathy by latent class analysis. ESC Heart Fail. 2021;8(1):555-565.	Not clinical metabolic diseases
1827	Li, N., Tang, H., Wu, L., Ge, H., Wang, Y., Yu, H., ... & Gu, H. F. (2021). Chemical constituents, clinical efficacy and molecular mechanisms of the ethanol extract of Abelmoschus manihot flowers in treatment of kidney diseases. Phytotherapy Research, 35(1), 198-206.	Not clinical metabolic diseases

1828	Xu Y, Wang Y, Yang Q, et al. Effects and safety of Chinese herbal medicine on inflammatory biomarkers in cardiovascular diseases: A systematic review and meta-analysis of randomized controlled trials. Front Pharmacol. 2022;13:1019284.	Not clinical metabolic diseases
1829	Xia W, Huang Y, Tang S, et al. Safety, tolerability, and pharmacokinetics of a single ascending dose of baicalein chewable tablets in healthy subjects. Front Pharmacol. 2023;14:1133597.	Not clinical metabolic diseases
1830	Wang T, Wang Y, Zhang H, et al. Research "recover from illness defense complex" helper T cell immune mechanisms based on the "Fuxie" theory clearing away heat evil thoroughly nourishing kidney treatment of recurrent blood-heat syndrome Psoriasis. Medicine (Baltimore). 2020;99(20):e20161.	Not clinical metabolic diseases
1831	Li, L, Liu, Y, Li, Z, Shen, D, Song, Y, Huang, M, Xue, X, Xie, J, Jiao, Z, Gao, S, Xu, Y, Gao, S, Wang, X, Xu, Q, Gao, S, Li, C, Niu, K and Yu, C, 2019, Adjuvant treatment of coronary heart disease angina pectoris with Chinese patent medicine: A prospective clinical cohort study, Medicine (United States)	Not clinical metabolic diseases
1832	Li, L., Dou, L. X., Neilson, J. P., Leung, P. C., & Wang, C. C. (2012). Adverse outcomes of Chinese medicines used for threatened miscarriage: a systematic review and meta-analysis. Human Reproduction Update, 18(5), 504-524.	Not clinical metabolic diseases
1833	He, L. I., Wenquan, S. U., Shanshan, L. I., Hanrui, J. I., Fangyuan, C. U. I., Lu, T. A. N. G., ... & Xinglu, D. O. N. G. (2023). Supplementing Qi and activating blood circulation method to treat vertebrobasilar dolichoectasia with posterior circulatory watershed infarction: a case report of two patients. Journal of Traditional Chinese Medicine, 43(4), 824.	Not clinical metabolic diseases
1834	Li, H., Lin, X., Liu, Q., & Zhang, Y. (2018). Curative effect of Danhong injection on the clinical symptoms, adverse reactions and electrocardiogram of angina pectoris of coronary heart disease. Int. J. Clin. Exp. Med, 2, 910-915.	Not clinical metabolic diseases
1835	Li, H., Jiang, X. M., Cui, N., Yuan, C., Zhang, S. F., Lu, Q. B., ... & Liu, W. (2021). Clinical effect and antiviral mechanism of T-705 in treating severe fever with thrombocytopenia syndrome. Signal transduction and targeted therapy, 6(1), 145.	Not clinical metabolic diseases
1836	Li, H. (2021). Angiogenesis in the progression from liver fibrosis to cirrhosis and hepatocelluar carcinoma. Expert review of gastroenterology & hepatology, 15(3), 217-233.	Not clinical metabolic diseases
1837	Li, G., Han, R., Lin, M., Wen, Z., & Chen, X. (2022). Developing a core outcome set for clinical trials of Chinese medicine for hyperlipidemia. Frontiers in Pharmacology, 13, 847101.	Not clinical metabolic diseases

1838	Li, C. Y., Chen, Y. L., Hu, J. Y., Li, M., Zhang, X. Y., Sun, Y., ... & Shang, H. C. (2021). Status quo and analysis of the cardiovascular clinical practice guidelines/expert consensuses of Chinese and integrative medicine: a systematic review. Chinese journal of integrative medicine, 27, 54-61.	Not clinical metabolic diseases
1839	Wang Y, Luo H, Fang Z, et al. Adjunctive traditional Chinese medicine improves survival in patients with advanced lung adenocarcinoma treated with first-line epidermal growth factor receptor (EGFR) tyrosine kinase inhibitors (TKIs): a nationwide, population-based cohort study. Front Pharmacol. 2021;12:716912.	Not clinical metabolic diseases
1840	Levy, Y, Narotzki, B and Reznick, AZ, 2017, Green tea, weight loss and physical activity, Clinical nutrition (Edinburgh, Scotland)	Not clinical metabolic diseases
1841	Leung, AKC and Hon, KLE, 2013, Seasonal allergic rhinitis, Recent Patents on Inflammation and Allergy Drug Discovery	Not clinical metabolic diseases
1842	Leung, A. K., Barankin, B., Lam, J. M., Leong, K. F., & Hon, K. L. (2023). Tinea pedis: an updated review. Drugs in context, 12.	Not clinical metabolic diseases
1843	Zhang H, He M, Liu Y, et al. Effect of traditional Chinese medicine on intra-abdominal hypertension and abdominal compartment syndrome: A systematic review and meta-analysis. Medicine (Baltimore). 2020;99(6):e19062.	Not clinical metabolic diseases
1844	Shamloul R, Ghanem H. Alternative medicine and herbal remedies in the treatment of erectile dysfunction: a systematic review. J Sex Med. 2013;10(2):411-423.	Not clinical metabolic diseases
1845	Study of the relationship between carotid intima-media thickness and traditional Chinese medicine syndrome of dyslipidemia	Not clinical metabolic diseases
1846	Teschke R, Wolff A, Frenzel C, Schulze J, Eickhoff A. Liver injury associated with polygonum multiflorum (He Shou Wu) in traditional Chinese medicine: a systematic review of case reports and case series. Phytomedicine. 2014;21(6):508-516.	Not clinical metabolic diseases
1847	Lee MS, Choi J, Posadzki P, Ernst E. Aromatherapy for health care: An overview of systematic reviews. Maturitas. 2012;71(3):257-260.	Not clinical metabolic diseases
1848	Tang JL, Liu BY, Ma KW. Traditional Chinese medicine. Lancet. 2008;372(9654):1938-1940.	Not clinical metabolic diseases
1849	Tao X, Cush JJ, Garret M, Lipsky PE. A phase I study of ethnopharmacologic Huo-Luo-Xiao-Ling Dan, a traditional Chinese medicine, for patients with osteoarthritis of the knee. Phytomedicine. 2011;18(7):599-604.	Not clinical metabolic diseases

1850	Lam DS, Leung DY, Tham CC, et al. Randomized trial of early phacoemulsification versus peripheral iridotomy to prevent intraocular pressure rise after acute primary angle closure. Ophthalmology. 2008;115(7):1134-1140.e2.	Not clinical metabolic diseases
1851	Chen N, Zhang L, Wang M, et al. Acute myocardial infarction in Chinese medicine hospitals in China from 2006 to 2013: An analysis of 2311 patients from hospital data. Chin Med J (Engl). 2017;130(24):2941-2947.	Not clinical metabolic diseases
1852	Ramaraj R, Sorrell VL. Takotsubo cardiomyopathy: a new form of acute, reversible heart failure. Am J Med. 2012;125(7):602-609.	Not clinical metabolic diseases
1853	Lafond, N. A., Churchill, R., Pandya, H., Smyth, A., Williams, J., & Elliott, R. A. (2010). Medicine-taking interventions in children and young people: One size fits all, one size fits none. International Journal of Pharmacy Practice, 18(SUPPL. 1), 11-12.	Not clinical metabolic diseases
1854	Devi KP, Rajavel T, Habtemariam S. Herbal therapeutics for Alzheimer's disease: ancient Indian medicine system from the modern viewpoint. Phytother Res. 2016;30(4):554-567.	Not clinical metabolic diseases
1855	Kumar, S., & Maroo, A. (2021). TAKOTSUBO SYNDROME IN THE SETTING OF ECLAMPSIA. Chest, 160(4), A218.	Not clinical metabolic diseases
1856	Kukurin, G. W. (2009). THE USE OF INTEGRATIVE NEUROLOGY TREATMENTS FOR SYMPTOMATIC RELIEF OF PERIPHERAL NEUROPATHY RESULTING FROM VARIOUS ETIOLOGICAL FACTORS: A BEST CASE SERIES. Journal of the Peripheral Nervous System, 14, 82-83.	Not clinical metabolic diseases
1857	Kudriavtsev, A. A., Vlasik, T. N., & Yurenev, A. P. (2003). Curing heart dilatation with acupuncture. Clinical Acupuncture and Oriental Medicine, 4(1), 38-40.	Not clinical metabolic diseases
1858	Kudo M, Ikeda M, Motomura K, et al. Regional differences in efficacy, safety, and biomarkers for second-line axitinib in patients with advanced hepatocellular carcinoma: from a randomized phase II study. Liver Cancer. 2020;9(6):636-649.	Not clinical metabolic diseases
1859	Kong, S., Zhang, G., Yang, Z., Kong, Z., & Ye, F. (2023). Effects of folic acid supplementation on chronic atrophic gastritis based on MTHFR C677T polymorphism. Medicine, 102(24), e33980.	Not clinical metabolic diseases
1860	Putra AR, Lianto P, Santoso AM, et al. The Efficacy of Herbs as Complementary and Alternative Therapy in Recovery and Clinical Outcome Among People with COVID-19: A Systematic Review, Meta-Analysis, and Meta-Regression. Adv Respir Med. 2022;90(3):275-292.	Not clinical metabolic diseases
1861	Komagamine J, Sugawara K, Horiuchi A, et al. Prevalence of the potentially inappropriate Kampo medications to be used with caution among elderly patients taking any prescribed Kampo medications at a single centre in Japan: a retrospective cross-sectional study. BMC Complement Med Ther. 2020;20(1):94.	Not clinical metabolic diseases

1862	Ko, M. M., Jang, S., & Jung, J. (2020). An observational study on diagnosis index of metabolic disease with blood-stasis. Medicine, 99(27), e21140.	Not clinical metabolic diseases
1863	Kirtschig G, Murrell DF, Wojnarowska F, et al. Interventions for bullous pemphigoid. Cochrane Database Syst Rev. 2010;(10):CD002292.	Not clinical metabolic diseases
1864	Kim YS, Kim N. Herbal therapies in functional gastrointestinal disorders: A narrative review and clinical implication. J Neurogastroenterol Motil. 2018;24(4):437-451.	Not clinical metabolic diseases
1865	Kim, Y., Choi, Y., Lee, M. Y., Cho, S. H., Jung, I. C., Kang, D. H., & Yang, C. (2023). Bangpungtongsung-san for patients with major depressive disorder: study protocol for a randomized controlled phase II clinical trial. BMC Complementary Medicine and Therapies, 23(1), 114.	Not clinical metabolic diseases
1866	Kim JS, Yoon TY. Clinicopathologic review of eruptive pseudoangiomatosis in Korean adults: report of 32 cases. J Am Acad Dermatol. 2009;61(6):994-999.	Not clinical metabolic diseases
1867	Mirghafourvand M, Mohammad-Alizadeh-Charandabi S, Javadzadeh Y, et al. Efficacy of a novel extract of fenugreek seeds in alleviating vasomotor symptoms and depression in perimenopausal women: A randomized, double-blinded, placebo-controlled study. Complement Ther Med. 2016;24:75-80.	Not clinical metabolic diseases
1868	Khajehdehi P, Zanjaninejad B, Aflaki E, et al. Oral supplementation of turmeric decreases proteinuria, hematuria, and systolic blood pressure in patients suffering from relapsing or refractory lupus nephritis: A randomized and placebo-controlled study. J Ren Nutr. 2012;22(1):50-57.	Not clinical metabolic diseases
1869	Bailey DM, Davies B, Young IS, et al. Effect of acetazolamide and gingko biloba on the human pulmonary vascular response to an acute altitude ascent. J Appl Physiol (1985). 2001;90(5):1806-1811.	Not clinical metabolic diseases
1870	Katelaris, C. H., Linneberg, A., Magnan, A., Thomas, W. R., Wardlaw, A. J., & Wark, P. (2011). Developments in the field of allergy in 2010 through the eyes of clinical and experimental allergy. Clinical & Experimental Allergy, 41(12), 1690-1710.	Not clinical metabolic diseases
1871	Shamloul R, Ghanem H. Erectile dysfunction. Lancet. 2013;381(9861):153-165.	Not clinical metabolic diseases
1872	Kashamar, A. M., Naser, E. H., Almaali, H. A. A., & Abbas, I. S. (2018). Clinical study of three medicinal plants (Foeniculum vulgare, Zea mays and Petroselinum sativum) against urinary tract infection and stones. Journal of Pharmaceutical Sciences and Research, 10(4), 755-758.	Not clinical metabolic diseases
1873	Shrestha S, Margetts P, Moreau M, et al. Lead encephalopathy due to traditional medicines. BMJ Case Rep. 2011;2011:bcr0620103074.	Not clinical metabolic diseases

1874	Dwivedi S. Terminalia arjuna in coronary artery disease: Ethnopharmacology, pre-clinical, clinical & safety evaluation. Curr Pharm Biotechnol. 2011;12(6):855-862.	Not clinical metabolic diseases
1875	Kamenov ZA, Gateva AT. Evaluation of the efficacy and safety of Tribulus terrestris in male sexual dysfunction-A prospective, randomized, double-blind, placebo-controlled clinical trial. Maturitas. 2016;85:1-7.	Not clinical metabolic diseases
1876	Kadlec, A. O., & Turk, T. M. (2013). Update on the evaluation of repeated stone formers. Current urology reports, 14, 549-556.	Not clinical metabolic diseases
1877	Johnson, T. W., Spurlock, A. L., Epp, L., Hurt, R. T., & Mundi, M. S. (2018). Reemergence of blended tube feeding and parent's reported experiences in their tube fed children. The Journal of Alternative and Complementary Medicine, 24(4), 369-373.	Not clinical metabolic diseases
1878	Bhatnagar T, Shukla S, Sharma A, et al. Efficacy and safety of a phytopharmaceutical drug derived from Cocculus hirsutus in adults with moderate COVID-19: a phase 2, open-label, multicenter, randomized controlled trial. Trials. 2022;23(1):1052.	Not clinical metabolic diseases
1879	Harada T, Takahashi K, Hirose M, et al. Trapa bispinosa Roxb. extract lowers advanced glycation end-products and increases live births in older patients with assisted reproductive technology: a randomized controlled trial. Reprod Biol Endocrinol. 2021;19(1):123.	Not clinical metabolic diseases
1880	Jin, Z. D., Wang, S. C., & Sun, Y. Q. (2003). Effect of danshao granule on serum superoxide dismutase activity and malonyldialdehyde content in children with Henoch-Schonlein purpura nephritis. Zhongguo Zhong xi yi jie he za zhi Zhongguo Zhongxiyi Jiehe Zazhi= Chinese Journal of Integrated Traditional and Western Medicine, 23(12), 905-907.	Not clinical metabolic diseases
1881	Jiang, N, Jia, GZ, Sun, HW, Zhang, LL, Liu, SM and Zhang, YZ, 2013, Case-control study on risk factors of endometrial cancer in northern Shandong area, Chinese Journal of Cancer Prevention and Treatment	Not clinical metabolic diseases
1882	Liu HW, Leng RX, Li XP, et al. The safety profile in treatment of Chinese SLE patients with leflunomide monotherapy and combination therapy. Lupus. 2013;22(9):894-901.	Not clinical metabolic diseases
1883	Liu HW, Leng RX, Li XP, et al. Leflunomide: The safety profile in the treatment of Chinese SLE patients. Lupus. 2013;22(9):894-901.	Not clinical metabolic diseases
1884	Guo Y, Zhang Y, Shi Y, et al. Fast-track surgery decreases the incidence of postoperative delirium and other complications in elderly patients with colorectal carcinoma. Langenbecks Arch Surg. 2014;399(1):77-84.	Not clinical metabolic diseases
1885	Chen J, Zhao W, Zha Y, et al. Acotec Drug-Coated Balloon Catheter: randomized, multicenter, controlled clinical study in femoropopliteal arteries: evidence from the AcoArt I Trial. JACC Cardiovasc Interv. 2016;9(17):1941-1949.	Not clinical metabolic diseases

1886	Kim YS, Lee HJ, Kim HJ, et al. Effectiveness of Gyejibongnyeong-Hwan for shoulder pain: study protocol for a randomised, wait-list controlled pilot trial. Trials. 2020;21(1):474.	Not clinical metabolic diseases
1887	Itoh, T., Shimada, Y., & Terasawa, K. (1999). Efficacy of Choto-san on vascular dementia and the protective effect of the hooks and stems of Uncaria sinensis on glutamate-induced neuronal death. Mechanisms of ageing and development, 111(2-3), 155-173.	Not clinical metabolic diseases
1888	Ota T, Kato H, Murakami T, et al. Choto-san versus placebo for patients with dementia: systematic review and meta-analysis. Complement Ther Med. 2020;50:102392.	Not clinical metabolic diseases
1889	Ohya Y, Okada A, Nakamura K, et al. Effects of the herbal medicine Inchinko-to on liver function in postoperative patients with biliary atresia: A pilot study. Pediatr Surg Int. 1998;13(7):505-507. doi:10.1007/s003830050384	Not clinical metabolic diseases
1890	Vahabi A, Kachooei AR, Ebrahimzadeh MH. Delayed treatment of neglected open knee dislocation: A case report. Trauma Mon. 2016;21(5):e22306. doi:10.5812/traumamon.22306	Not clinical metabolic diseases
1891	Liu M, Wang J, Zhang Y, et al. Exploring the mechanism of Yixinyin for myocardial infarction by weighted co-expression network and molecular docking. BMC Complement Med Ther. 2021;21(1):88. doi:10.1186/s12906-021-03262-6	Not clinical metabolic diseases
1892	Chen J, Sun D, Luo R, et al. Efficacy and safety of Naoxintong capsule for treating chronic stable angina: study protocol for a randomized controlled trial. Trials. 2020;21(1):661. doi:10.1186/s13063-020-04561-x	Not clinical metabolic diseases
1893	Wang W, Su L, Wang Y, et al. Fuling Sini decoction for patients with chronic heart failure: A protocol for a systematic review and meta-analysis. Medicine (Baltimore). 2020;99(42):e22691. doi:10.1097/MD.0000000000022691	Not clinical metabolic diseases
1894	Hung SK, Chen HC, Lin YT, et al. Traditional Chinese medicine is associated with a decreased risk of heart failure in breast cancer patients receiving doxorubicin treatment. Front Pharmacol. 2022;13:853938.	Not clinical metabolic diseases
1895	Sun Y, Zhao H, He J, et al. Qili Qiangxin as an adjuvant treatment with inotrope for advanced heart failure: retrospective analysis. Medicine (Baltimore). 2021;100(38):e27202.	Not clinical metabolic diseases
1896	Hua, J., Huang, J., Li, G., Lin, S., & Cui, L. (2023). Glucocorticoid induced bone disorders in children: research progress in treatment mechanisms. Frontiers in Endocrinology, 14, 1119427.	Not clinical metabolic diseases
1897	Liu S, Zhang F, Wang Y, et al. He-Jie-Shen-Shi Decoction as an adjuvant therapy on severe coronavirus disease 2019: a retrospective cohort and potential mechanistic study. Front Pharmacol. 2021;12:695123.	Not clinical metabolic diseases

1898	Li Y, Yang L, Wang Q, et al. Vasodilator-stimulated phosphoprotein-guided clopidogrel maintenance therapy reduces cardiovascular events in atrial fibrillation patients requiring anticoagulation therapy and scheduled for percutaneous coronary intervention: a prospective cohort study. Front Pharmacol. 2021;12:694332.	Not clinical metabolic diseases
1899	Hsieh, C. H., Tsai, H. C., Hsu, G. L., Chen, C. C., & Hsu, C. Y. (2016). Herb formula enhances treatment of impotent patients after penile venous stripping: a randomised clinical trials. Andrologia, 48(7), 754-760.	Not clinical metabolic diseases
1900	Hou, J. Q., AN, Y., Chen, Y. L., Wang, C., MA, D., Wang, W., & Zhao, H. B. (2021). Systematic Review and Meta-analysis of Traditional Chinese Medicine on Inflammatory Factors in Patients with Coronary Heart Disease Complicated with Anxiety and Depression. Chinese Journal of Experimental Traditional Medical Formulae, 153-163.	Not clinical metabolic diseases
1901	Fu EL, Dekker FW, Lambers Heerspink HJ, et al. Laxative use and mortality in patients on haemodialysis: a prospective cohort study. Nephrol Dial Transplant. 2022;37(2):295-303.	Not clinical metabolic diseases
1902	Hodson, E. M. (2003). The management of idiopathic nephrotic syndrome in children. Pediatric Drugs, 5, 335-349.	Not clinical metabolic diseases
1903	Liu Y, Wang Y, Li X, et al. Ameliorative effects of ginsenosides on myelosuppression induced by chemotherapy or radiotherapy. Front Pharmacol. 2021;12:630985.	Not clinical metabolic diseases
1904	Wang L, Xu C, Liu L, et al. Efficacy and safety of Chinese medicine in treating arrhythmia: meta-analysis of randomized controlled trials. Complement Ther Med. 2019;45:109-116.	Not clinical metabolic diseases
1905	Hashimoto, M., Kawai, Y., Masutani, T., Tanaka, K., Ito, K., & Iddamalgoda, A. (2022). Effects of watercress extract fraction on R-spondin 1-mediated growth of human hair. International Journal of Cosmetic Science, 44(2), 154-165.	Not clinical metabolic diseases
1906	Yao, H. A. N., Mei, D. A. I., Hong-xu, L. I. U., & Zhi-zhen, W. E. I. (2021). Clinical Effect of Tiaomai Mixture on Premature Ventricular Contraction in Coronary Heart Disease Due to Qi-Yin Deficiency and Stagnated Heat in Blood Vessel. Chinese Journal of Experimental Traditional Medical Formulae, 118-125.	Not clinical metabolic diseases
1907	Methods in Medicine, C. A. M. (2023). Retracted: The Curative Effect of Shuangshen Decoction Combined with Immunological Preparations in the Treatment of Pediatric Nephrotic Syndrome and Its Influence on the Rate of Complicated Infection and Recurrence.	Not clinical metabolic diseases
1908	Guo, X., Zhou, D., Sun, L., Wang, P., Qu, J., Zhang, C., ... & Li, P. (2020). Traditional Chinese medicine for psoriasis vulgaris: A Protocol of a prospective, multicenter cohort study. Medicine, 99(41), e21913.	Not clinical metabolic diseases

1909	Huang W, Li L, Ma Y, et al. Effect of continuous intraoperative infusion of methoxamine on renal function in elderly patients undergoing gastrointestinal tumor surgery: a randomized controlled trial. Ann Transl Med. 2022;10(6):309.	Not clinical metabolic diseases
1910	Chen J, Zhao W, Zha Y, et al. First prospective, randomized, multicenter clinical trial (AcoArt 1) for the use of the orchid DCB in femoropopliteal artery disease: 2-year results. JACC Cardiovasc Interv. 2016;9(17):1941-1949.	Not clinical metabolic diseases
1911	Li H, Wang L, Wang C, et al. Clinical characteristics and pathogen analysis of bronchoalveolar lavage fluid in elderly patients with community-acquired pneumonia. Front Med (Lausanne). 2021;8:638326.	Not clinical metabolic diseases
1912	López-Otín C, Blasco MA, Partridge L, Serrano M, Kroemer G. The hallmarks of aging. Cell. 2013;153(6):1194-1217.	Not clinical metabolic diseases
1913	Guan, H, Dai, GH, Gao, WL, Zhang, T, Sun, C, Ren, LL, Hou, XM and Liu, Z, 2022, Effect of Shenmai Injection on Long-Term Prognosis of Patients with Chronic Heart Failure: A Multicenter, Large Sample Capacity, Long-Term Follow-Up Retrospective Cohort Study, Chinese Journal of Integrative Medicine	Not clinical metabolic diseases
1914	Griebling, T. L. (2013). Late Onset Hypogonadism, Testosterone Replacement Therapy, and Sexual Health in Elderly Men. Current Translational Geriatrics and Experimental Gerontology Reports, 2, 76-83.	Not clinical metabolic diseases
1915	Grammatikopoulou, M. G., Gkiouras, K., Papageorgiou, S. 韦., Myrogiannis, I., Mykoniatis, I., Papamitsou, T., ... & Goulis, D. G. (2020). Dietary factors and supplements influencing prostate-specific antigen (PSA) concentrations in men with prostate cancer and increased cancer risk: an evidence analysis review based on randomized controlled trials.聽Nutrients,聽 12(10), 2985.	Not clinical metabolic diseases
1916	Li L, Tang H, Wu X, et al. Growth hormone activates PI3K/Akt signaling and inhibits ROS accumulation and apoptosis in granulosa cells of patients with polycystic ovary syndrome. J Obstet Gynaecol Res. 2020;46(7):1052-1062.	Not clinical metabolic diseases
1917	Lv Q, Xu D, Zhang X, et al. Comparative efficacy and safety of Tripterygium wilfordii Hook F versus sulfasalazine in the treatment of rheumatoid arthritis: a randomized trial. Ann Rheum Dis. 2015;74(6):1078-1086.	Not clinical metabolic diseases
1918	Givol, O., Kornhaber, R., Visentin, D., Cleary, M., Haik, J., & Harats, M. (2019). A systematic review of Calendula officinalis extract for wound healing. Wound repair and regeneration, 27(5), 548-561.	Not clinical metabolic diseases
1919	Ghasemian, M., Owlia, S., & Owlia, M. B. (2016). Review of anti-inflammatory herbal medicines. Advances in Pharmacological and Pharmaceutical Sciences, 2016(1), 9130979.	Not clinical metabolic diseases

1920	Fallah AA, Sarmast E, Fatehi F, Jafari T. What is the influence of grape products on liver enzymes? A systematic review and meta-analysis of randomized controlled trials. Phytother Res. 2021;35(3):1163-1177.	Not clinical metabolic diseases
1921	Ge, C. J., Yuan, F., Feng, L. X., Lv, S. Z., Liu, H., Song, X. T., ... & Huo, Y. (2014). Clinical effect of Maixuekang Capsule (脉血康胶囊) on long-term prognosis in patients with acute coronary syndrome after percutaneous coronary intervention. Chinese journal of integrative medicine, 20, 88-93.	Not clinical metabolic diseases
1922	Garside, R., Stein, K., Castelnuovo, E., Pitt, M., Ashcroft, D., Dimmock, P., & Payne, L. (2005). The effectiveness and cost-effectiveness of pimecrolimus and tacrolimus for atopic eczema: a systematic review and economic evaluation. Health Technology Assessment (Winchester, England), 9(29), iii-xi.	Not clinical metabolic diseases
1923	Chen Y, Li C, Yang Z, et al. Analysis on outcome of 5284 patients with coronary artery disease: the role of integrative medicine. Am J Chin Med. 2012;40(5):945-953.	Not clinical metabolic diseases
1924	Gao, X. R., Adhikari, C. M., Peng, L. Y., Guo, X. G., Zhai, Y. S., He, X. Y., ... & Zuo, Z. Y. (2009). Efficacy of different doses of aspirin in decreasing blood levels of inflammatory markers in patients with cardiovascular metabolic syndrome. Journal of Pharmacy and Pharmacology, 61(11), 1505-1510.	Not clinical metabolic diseases
1925	Gao, Q., & Liang, N. (2015). Integrated traditional Chinese medicine improves acute pancreatitis via the downregulation of PRSS1 and SPINK1. Experimental and Therapeutic Medicine, 9(3), 947-954.	Not clinical metabolic diseases
1926	Low SK, Ong CW, Ong RT, et al. Retrospective case-control study of risk factors for recurrent tuberculosis in Singapore. Int J Tuberc Lung Dis. 2014;18(3):312-317.	Not clinical metabolic diseases
1927	McVary KT, Monnig W, Camps JL, et al. Sildenafil citrate improves erectile function and urinary symptoms in men with erectile dysfunction and lower urinary tract symptoms associated with benign prostatic hyperplasia: a randomized, double-blind trial. J Urol. 2007;177(3):1071-1077.	Not clinical metabolic diseases
1928	Vayá A, Alis R, Hernández-Mijares A, et al. Antioxidant therapy for chronic hepatitis C after failure of interferon: results of phase II randomized, double-blind placebo-controlled clinical trial. Clin Chim Acta. 2011;412(15-16):1343-1348.	Not clinical metabolic diseases
1929	Borghi C, Cicero AFG. Plant-derived anti-inflammatory compounds: Hopes and disappointments regarding the translation of preclinical knowledge into clinical progress. Phytomedicine. 2017;24:108-111. doi:10.1016/j.phymed.2016.10.005	Not clinical metabolic diseases
1930	Fu, G., Chen, X., Li, L., Liu, Q., Sun, N., Shi, C., & Zhang, R. (2022). Function and pathway analysis of the differential expression proteins in osteoarthritis based on proteomics technology. Osteoarthritis and Cartilage, 30, S312-S315.	Not clinical metabolic diseases

1931	Zick SM, Sen A. "What Should I Eat?"—Addressing Questions and Challenges Related to Nutrition in the Integrative Oncology Setting. Cancer J. 2019;25(5):320-324. doi:10.1097/PPO.0000000000000399	Not clinical metabolic diseases
1932	Hsieh TC, Wu TH, Huang YW, et al. Integration of Chinese herbal medicine therapy improves survival of patients with chronic lymphocytic leukemia. Medicine (Baltimore). 2021;100(1):e24050. doi:10.1097/MD.0000000000024050	Not clinical metabolic diseases
1933	Camara B, Toure M, Ka MM, et al. Comparative study of the efficacy and tolerance of two types of colon cleansing. Pan Afr Med J. 2015;22:335. doi:10.11604/pamj.2015.22.335.7489	Not clinical metabolic diseases
1934	Fu, G., Chen, X., Li, L., Liu, Q., Sun, N., Shi, C., & Zhang, R. (2022). Function and pathway analysis of the differential expression proteins in osteoarthritis based on proteomics technology. Osteoarthritis and Cartilage, 30, S312-S315.	Not clinical metabolic diseases
1935	Feng, Q., Wang, Z., Yu, H., Shi, L., & Xu, L. (2022). Analysis of clinical characteristics of 362 vaccinated or unvaccinated patients infected by novel coronavirus Omicron variant. Zhonghua wei zhong bing ji jiu yi xue, 34(5), 459-464	Not clinical metabolic diseases
1936	Xu H, Zhang Y, Wang Y, et al. Chinese herbal medicine for patients with vascular cognitive impairment no dementia: Protocol for a systematic review. Medicine (Baltimore). 2020;99(20):e20282. doi:10.1097/MD.0000000000020282	Not clinical metabolic diseases
1937	Coelho Rabello E, de Brito Cordeiro R, Nogueira Bittencourt L, et al. Acute effect of green tea and hibiscus ingestion on energy metabolism and substrate oxidation rates: a randomized clinical trial. Phytother Res. 2019;33(9):2451-2459.	Not clinical metabolic diseases
1938	Xie Y, Sun M, Guo Y, et al. Traditional Chinese Medicine compound preparations are associated with low disease-related complication rates in patients with rheumatoid arthritis: A retrospective cohort study of 11,074 patients. Front Pharmacol. 2021;12:745068.	Not clinical metabolic diseases
1939	Zhu Z, Wang W, Zhuang H, et al. Recurrent arthritis caused by Candida parapsilosis: a case report and literature review. BMC Infect Dis. 2021;21(1):80.	Not clinical metabolic diseases
1940	Fan, M., Guo, D., Wang, Y., Liu, Y., Zhao, J., & Yu, Z. (2021). Efficacy and safety of Xinkeshu in the treatment of angina pectoris of coronary heart disease: a protocol for systematic review and meta-analysis. Medicine, 100(40), e27407.	Not clinical metabolic diseases
1941	Fan, G. H. (2020). Systematic evaluation of efficacy and safety of Xinkeshu in treatment of unstable angina pectoris. Chinese Traditional and Herbal Drugs, 4719-4732.	Not clinical metabolic diseases
1942	Fan, G. H. (2020). Systematic evaluation of efficacy and safety of Xinkeshu in treatment of unstable angina pectoris. Chinese Traditional and Herbal Drugs, 4719-4732.	Not clinical metabolic diseases

1943	Hehemann MC, Kashanian JA. Natural therapies used by adult men for the treatment of erectile dysfunction, benign prostatic hyperplasia, and for augmenting exercise performance. Andrology. 2016;4(3):423-430.	Not clinical metabolic diseases
1944	Wahlbeck K, Cheine M, Essali A, Adams C. Clozapine versus typical neuroleptic medication for schizophrenia. Cochrane Database Syst Rev. 2000;(2):CD000059.	Not clinical metabolic diseases
1945	El-Sayyad, H. I. (2015). Cholesterol overload impairing cerebellar function: the promise of natural products. Nutrition, 31(5), 621-630.	Not clinical metabolic diseases
1946	Eisenstein, M. (2019). Tea's value as a cancer therapy is steeped in uncertainty. Nature, 566(7742), S6-S6.	Not clinical metabolic diseases
1947	Sharma H, Chandola HM, Singh G, Basisht G. Utilization of Ayurveda in health care: An approach for prevention, health promotion, and treatment of disease. Part 2--Ayurveda in primary health care. J Altern Complement Med. 2007;13(10):1135-1150.	Not clinical metabolic diseases
1948	Sharma H, Chandola HM, Singh G, Basisht G. Ayurveda improves arterial stiffness and cardiometabolic risk in coronary patients: A prospective pilot study. J Ayurveda Integr Med. 2013;4(1):30-36.	Not clinical metabolic diseases
1949	Dubath, C., Gholam-Rezaee, M., Sjaarda, J., Levier, A., Saigi-Morgui, N., Delacrétaz, A., ... & Eap, C. B. (2021). Socio-economic position as a moderator of cardiometabolic outcomes in patients receiving psychotropic treatment associated with weight gain: results from a prospective 12-month inception cohort study and a large population-based cohort. Translational psychiatry, 11(1), 360.	Not clinical metabolic diseases
1950	Duan, W. H., Lu, F., Li, L. Z., Wang, C. L., Liu, J. G., Yang, Q. N., ... & Shi, D. Z. (2012). Clinical efficacy of traditional Chinese medicine on acute myocardial infarction—a prospective cohort study. Chinese journal of integrative medicine, 18, 807-812.	Not clinical metabolic diseases
1951	Douglass, M. A., & Lin, J. C. (2005). Update on the treatment of benign prostatic hyperplasia. Formulary, 40(2), 50.	Not clinical metabolic diseases
1952	Chen P, Nirula A, Heller B, et al. Efficacy and safety of SARS-CoV-2 neutralizing antibody JS016 in hospitalized Chinese patients with COVID-19: A phase 2/3, multicenter, randomized, open-label, controlled trial. MedComm (2020). 2021;2(4):815-826.	Not clinical metabolic diseases
1953	Doan, U. V. (2020, November). Severe Lactic Acidosis in Patients Using Traditional Herbal Therapy. In CLINICAL TOXICOLOGY (Vol. 58, No. 11, pp. 1122-1122). 2-4 PARK SQUARE, MILTON PARK, ABINGDON OR14 4RN, OXON, ENGLAND: TAYLOR & FRANCIS LTD.	Not clinical metabolic diseases
1954	Dizon, K., Ng, P. C., & Battistella, M. (2021). A retrospective study of antithrombotic therapy use in an outpatient haemodialysis unit. Journal of Clinical Pharmacy and Therapeutics, 46(5), 1387-1394.	Not clinical metabolic diseases

1955	Ding, Z. L., Ke, M. Y., Sun, X. H., & Wang, Z. F. (2007). Effect of combined drug treatment on megacolon with severe constipation. Zhonghua yi xue za zhi, 87(10), 670-672.	Not clinical metabolic diseases
1956	Ding, S. Y., Zheng, P. D., He, L. Q., Hou, W. G., Zou, Y., & Gao, J. D. (2013). The research on xiaochalhu decoction improving the inflammation of chronic glomerulonephritis patients and relieving the proteinuria. Zhongguo Zhong xi yi jie he za zhi Zhongguo Zhongxiyi Jiehe Zazhi= Chinese Journal of Integrated Traditional and Western Medicine, 33(1), 21-26.	Not clinical metabolic diseases
1957	Dimpfel, W., Schombert, L., Keplinger-Dimpfel, I. K., & Panossian, A. (2020). Effects of an adaptogenic extract on electrical activity of the brain in elderly subjects with mild cognitive impairment: A randomized, double-blind, placebo-controlled, two-armed cross-over study. Pharmaceuticals, 13(3), 45.	Not clinical metabolic diseases
1958	Abanador-Kamper N, Kamper L, Wolfertz J, et al. Antiplatelet therapy in Takotsubo cardiomyopathy: does it improve cardiovascular outcomes during index event? Int J Cardiol. 2017;238:41-45.	Not clinical metabolic diseases
1959	Nair PK, Vasudevan D, Shankar KK. The ultimate broken heart: Takotsubo cardiomyopathy with complete heart block mimicking left anterior descending infarction. BMJ Case Rep. 2014;2014:bcr2013202387.	Not clinical metabolic diseases
1960	Deng, Y, Zhu, HQ, Deng, GB and Tan, C, 2005, A clinical study on the effect of Yinxing Damo (Chinese characters) combined with Betahistine Hydrochloride Injection on vertebral basilar artery ischemic vertigo, Chinese Journal of Integrative Medicine	Not clinical metabolic diseases
1961	Zhang L, Chen X, Zhao Z, et al. Is there an advantage of using Dingkun Pill alone or in combination with Diane-35 for management of polycystic ovary syndrome? A randomized controlled trial. Medicine (Baltimore). 2020;99(50):e23469.	Not clinical metabolic diseases
1962	Deng, X., Liang, J., Liu, Z. W., Wu, F. S., & Li, X. (2013). Treatment of posthepatitic cirrhosis by Fuzheng Huayu Tablet (扶正化瘀片) for reinforcing qi and resolving stasis. Chinese journal of integrative medicine, 19(4), 289-296.	Not clinical metabolic diseases
1963	Liang J, Ren L, Liu J, et al. Topical herbal medicine combined with pharmacotherapy for psoriasis: a systematic review and meta-analysis. Front Pharmacol. 2021;12:748112.	Not clinical metabolic diseases
1964	Debruyne, F., Boyle, P., Da Silva, F. C., Gillenwater, J. G., Hamdy, F. C., Perrin, P., ... & Schulman, C. C. (2004). Evaluation of the clinical benefit of Permixon and tamsulosin in severe BPH patients—PERMAL study subset analysis. European urology, 45(6), 773-780.	Not clinical metabolic diseases
1965	Gagnier JJ, Chrubasik S, Manheimer E. Harpagophytum procumbens for osteoarthritis and low back pain: a systematic review. BMC Complement Altern Med. 2004;4:13.	Not clinical metabolic diseases

1966	Lopes S, Martins N, Teixeira M, et al. Biological activities of Eugenia uniflora L. (pitangueira) extracts in oxidative stress-induced pathologies: A systematic review and meta-analysis of animal studies. J Ethnopharmacol. 2021;273:114001.	Not clinical metabolic diseases
1967	De BK, Majumder S, Saha SJ, et al. Role of garlic in hepatopulmonary syndrome: a randomized controlled trial. Can J Gastroenterol. 2010;24(3):183-188.	Not clinical metabolic diseases
1968	Sun Y, Zhao Y, Zhou J, et al. Efficacy and safety of GuiZhi-ShaoYao-ZhiMu decoction for treating rheumatoid arthritis: A systematic review and meta-analysis of randomized clinical trials. Front Pharmacol. 2020;11:586658.	Not clinical metabolic diseases
1969	Dai, Y., Chen, X., Yang, H., Yang, J., Hu, Q., Xiao, X., ... & Ma, X. (2022). Evidence construction of Huangkui capsule against chronic glomerulonephritis: a systematic review and network pharmacology. Phytomedicine, 102, 154189.	Not clinical metabolic diseases
1970	Dai, D., Wu, H., He, C., Wang, X., Luo, Y., & Song, P. (2022). Evidence and potential mechanisms of traditional Chinese medicine for the treatment of psoriasis vulgaris: a systematic review and meta-analysis. Journal of Dermatological Treatment, 33(2), 671-681.	Not clinical metabolic diseases
1971	Dahar, B., Khavasi, N., Kamali, K., & Rashtchi, V. (2022). Comparison of the Effect of Cydonia oblonga and Phyllanthus Emblica on Gastric Residual Volume and Pulmonary Aspiration in Patients under Mechanical Ventilation in Mousavi Hospital ICU of Zanjan in 2020. Journal of Advances in Medical and Biomedical Research, 30(141), 357-364.	Not clinical metabolic diseases
1972	Dabrowski, W., Gagos, M., Siwicka-Gieroba, D., Piechota, M., Siwiec, J., Bielacz, M., ... & Malbrain, M. L. (2023). Humulus lupus extract rich in xanthohumol improves the clinical course in critically ill COVID-19 patients. Biomedicine & Pharmacotherapy, 158, 114082.	Not clinical metabolic diseases
1973	D'Cunha, N. M., Georgousopoulou, E. N., Dadigamuwage, L., Kellett, J., Panagiotakos, D. B., Thomas, J., ... & Naumovski, N. (2018). Effect of long-term nutraceutical and dietary supplement use on cognition in the elderly: a 10-year systematic review of randomised controlled trials. British Journal of Nutrition, 119(3), 280-298.	Not clinical metabolic diseases
1974	Cramer, E. H., Jones, P., Keenan, N. L., & Thompson, B. L. (2003). Is naturopathy as effective as conventional therapy for treatment of menopausal symptoms?. The Journal of Alternative & Complementary Medicine, 9(4), 529-538.	Not clinical metabolic diseases
1975	Comar, K. M., & Kirby, D. F. (2005). Herbal remedies in gastroenterology. Journal of clinical gastroenterology, 39(6), 457-468.	Not clinical metabolic diseases
1976	Coley, N., Giulioli, C., Aisen, P. S., Vellas, B., & Andrieu, S. (2022). Randomised controlled trials for the prevention of cognitive decline or dementia: A systematic review. Ageing Research Reviews, 82, 101777.	Not clinical metabolic diseases

1977	Shimizu M, Shirakami Y, Sakai H, et al. Effects of apple polyphenols on vascular oxidative stress and endothelial function: a translational study. Mol Nutr Food Res. 2021;65(7):e2000766.	Not clinical metabolic diseases
1978	Chong, J. H., & Koh, M. J. A. (2017). Non-topical management of recalcitrant paediatric atopic dermatitis. Archives of Disease in Childhood, 102(7), 681-686.	Not clinical metabolic diseases
1979	Lin CF, Liao KF, Chang CM, et al. Chinese herbal medicine might be associated with a lower rate of joint replacement in patients with osteoarthritis: a 12-year population-based matched cohort analysis. Medicine (Baltimore). 2016;95(14):e3292.	Not clinical metabolic diseases
1980	Lee YJ, Kim JM, Kim HY, et al. Antioxidant and physiological effects of Si-Wu-Tang on skin and liver: a randomized, double-blind, placebo-controlled clinical trial. Am J Chin Med. 2016;44(7):1375-1388.	Not clinical metabolic diseases
1981	Lau CBS, Ho CY, Kim CF, et al. Triterpenoids and polysaccharide peptides-enriched Ganoderma lucidum: a randomized, double-blind placebo-controlled crossover study of its antioxidation and hepatoprotective efficacy in healthy volunteers. Phytother Res. 2012;26(7):962-967.	Not clinical metabolic diseases
1982	Cheraghi, M., & Asadi-Samani, M. (2016). An overview of the most important medicinal plants affecting cardiac arrhythmia in Iran. Der Pharmacia Lettre, 8(5), 87-93.	Not clinical metabolic diseases
1983	Cheng, C. S., Wang, Z., & Chen, J. (2014). Targeting FASN in breast cancer and the discovery of promising inhibitors from natural products derived from traditional Chinese medicine. Evidence-Based Complementary and Alternative Medicine, 2014(1), 232946.	Not clinical metabolic diseases
1984	Chen, Z., Zhang, Z., Liu, J., Qi, H., Li, J., Chen, J., ... & Li, X. (2022). Gut microbiota: therapeutic targets of ginseng against multiple disorders and ginsenoside transformation. Frontiers in Cellular and Infection Microbiology, 12, 853981.	Not clinical metabolic diseases
1985	Li H, Xie X, Leung AW, et al. AGI grade-guided chaiqin chengqi decoction treatment for predicted moderately severe and severe acute pancreatitis (CAP trial): study protocol of a randomised, double-blind, placebo-controlled, parallel-group, pragmatic clinical trial. Trials. 2021;22(1):626.	Not clinical metabolic diseases
1986	Xu J, Zhang Y, Yang Y, et al. Traditional Chinese Medicine oral liquids combined with azithromycin for Mycoplasma pneumoniae pneumonia in children: a Bayesian network meta-analysis. Front Pharmacol. 2021;12:713546.	Not clinical metabolic diseases
1987	Li X, Guo L, Li Y, et al. Reduced hepatotoxicity by total glucosides of paeony in combination treatment with leflunomide and methotrexate for patients with active rheumatoid arthritis. Int Immunopharmacol. 2016;32:105-110.	Not clinical metabolic diseases
1988	Liu X, Liu W, Zhou H, et al. The efficacy and safety of Abelmoschus moschatus (a traditional Chinese medicine) for chronic kidney disease: a meta-analysis of randomized controlled trials and observational trials. Front	Not clinical metabolic diseases

	Pharmacol. 2022;13:846926.	
1989	Wu X, Zhu L, Chen S, et al. Systematic review and meta-analysis of the efficacy and safety of Biqi capsule in rheumatoid arthritis patients. Phytomedicine. 2021;92:153745.	Not clinical metabolic diseases
1990	Chen, X. M., Chen, Y. P., & Chen, J. (2007). Multicentered, randomized, controlled clinical trial on patients with IgA nephropathy of Qi-yin deficiency syndrome type. Zhongguo Zhong xi yi jie he za zhi Zhongguo Zhongxiyi Jiehe Zazhi= Chinese Journal of Integrated Traditional and Western Medicine, 27(2), 101-105.	Not clinical metabolic diseases
1991	Chen, X. L., Liu, X. Q., Xie, R., Peng, D. H., Wang, Y. P., Zhou, X., ... & Li, X. (2018). Expert consensus of syndrome differentiation for phlegm turbidity syndrome for coronary heart disease. Evidence-Based Complementary and Alternative Medicine, 2018(1), 8184673.	Not clinical metabolic diseases
1992	Chen, X. J., Yu, D. J., Yu, R. H., Su, Q. M., Xu, Y. G., He, Y., & Liu, Q. Q. (2014). Effect of Chinese herbal therapy on T-lymphocytes of IgA nephropathy patients: a clinical observation. Zhongguo Zhong xi yi jie he za zhi Zhongguo Zhongxiyi Jiehe Zazhi= Chinese Journal of Integrated Traditional and Western Medicine, 34(7), 786-789.	Not clinical metabolic diseases
1993	Chen, X., Tao, C., Wang, J., He, B., & Xu, J. (2022). Meta-analysis of therapeutic efficacy and effects of integrated traditional Chinese and Western medicine on coagulation and fibrinolysis system in patients with threatened abortion and polycystic ovary syndrome. American Journal of Translational Research, 14(5), 2768.	Not clinical metabolic diseases
1994	Chen, X., Li, A., & Zhang, S. W. (2010). Effects of Tongfu Granule on intestinal dysfunction in patients with multiple organ dysfunction syndrome. Zhongguo Zhong xi yi jie he za zhi Zhongguo Zhongxiyi Jiehe Zazhi= Chinese Journal of Integrated Traditional and Western Medicine, 30(8), 810-813.	Not clinical metabolic diseases
1995	Li X, Ma Y, Zhan S, et al. The characteristics and prescription patterns of Chinese herbal medicine in clinical practice for the treatment of anemia. BMC Complement Med Ther. 2020;20(1):288.	Not clinical metabolic diseases
1996	Chen, T. F., Lin, W. L., Liu, W. Y., & Gu, C. M. (2023). Prostate lymphoma with renal obstruction; reflections on diagnosis and treatment: two case reports. World Journal of Clinical Cases, 11(7), 1627.	Not clinical metabolic diseases
1997	Chen Y, Wang J, Lin L, et al. Chinese herbal medicine for myasthenia gravis: A systematic review and meta-analysis. Medicine (Baltimore). 2020;99(15):e19721.	Not clinical metabolic diseases
1998	Chen Y, Ma L, Guo X, et al. Chinese medicinal herbs for reducing endocrine therapy-induced side effects in patients with hormone receptor-positive breast cancer: a systematic review and meta-analysis. Integr Cancer Ther. 2020;19:1534735420946839.	Not clinical metabolic diseases

1999	Chen, R. Y., Yu, X., Smith, B., Liu, X., Gao, J., Diacon, A. H., ... & Barry, C. E. (2021). Radiological and functional evidence of the bronchial spread of tuberculosis: an observational analysis. The Lancet Microbe, 2(10), e518-e526.	Not clinical metabolic diseases
2000	Wang J, Xiong X, Liu W, et al. A traditional Chinese medicine therapy for coronary heart disease after percutaneous coronary intervention: A meta-analysis of randomized, double-blind, placebo-controlled trials. Front Pharmacol. 2021;12:660657.	Not clinical metabolic diseases
2001	Chen, N., Xing, C., Niu, J., Liu, B., Fu, J., Zhao, J., ... & Mei, C. (2022). Darbepoetin alfa injection versus epoetin alfa injection for treating anemia of Chinese hemodialysis patients with chronic kidney failure: a randomized, open-label, parallel-group, non-inferiority Phase III trail. Chronic Diseases and Translational Medicine, 8(01), 59-70.	Not clinical metabolic diseases
2002	Chen, L., Qi, H., Jiang, D., Wang, R., Chen, A., Yan, Z., & Xiao, J. (2013). The new use of an ancient remedy: a double-blinded randomized study on the treatment of rheumatoid arthritis. The American Journal of Chinese Medicine, 41(02), 263-280.	Not clinical metabolic diseases
2003	Chen, H. Y., Li, Q., Zhou, P. P., Yang, T. X., Liu, S. W., Zhang, T. F., ... & Wang, Y. G. (2023). Mechanisms of Chinese medicine in gastroesophageal reflux disease treatment: data mining and systematic pharmacology study. Chinese journal of integrative medicine, 29(9), 838-846.	Not clinical metabolic diseases
2004	Chen, H., Guo, J., Pang, B., Zhao, L., & Tong, X. (2015). Application of herbal medicines with bitter flavor and cold property on treating diabetes mellitus. Evidence-Based Complementary and Alternative Medicine, 2015(1), 529491.	Not clinical metabolic diseases
2005	Chen, H., Deng, C., Meng, Z., & Meng, S. (2023). Effects of TCM on polycystic ovary syndrome and its cellular endocrine mechanism. Frontiers in Endocrinology, 14, 956772.	Not clinical metabolic diseases
2006	Yang L, Su T, Li XM, et al. Clinical and pathological spectrums of aristolochic acid nephropathy. Nephrol Dial Transplant. 2018;33(3):424-432.	Not clinical metabolic diseases
2007	Chen, D., Li, C., Cai, H., Zhuang, J., Huang, Y., Peng, X., ... & Zhan, Z. (2020). Effect of banxia baizhu tianma tang for H-type hypertension: a protocol for a systematic review. Medicine, 99(9), e19309.	Not clinical metabolic diseases
2008	Duan C, Xia W, Zheng R, et al. Clinical efficacy of Jingyin granules, a Chinese patent medicine, in treating patients infected with coronavirus disease 2019: A retrospective case series. Phytomedicine. 2021;85:153531.	Not clinical metabolic diseases
2009	Cheetham, P. J., & Katz, A. E. (2012). Diet and prostate cancer: A holistic approach to management. Prostate cancer: A comprehensive perspective, 355-367.	Not clinical metabolic diseases
2010	Cheema, D., Coomarasamy, A., & El-Toukhy, T. (2007). Non-hormonal therapy of post-menopausal vasomotor symptoms: a structured evidence-based review. Archives of Gynecology and Obstetrics, 276,	Not clinical metabolic diseases

	463-469.	
2011	Fusco F, Creta M, De Nunzio C, et al. Pharmacological therapy of benign prostatic hyperplasia/lower urinary tract symptoms: An overview for the practising clinician. BJU Int. 2020;125(4):509-521.	Not clinical metabolic diseases
2012	Liao KM, Chiu HW, Huang YB, et al. Traditional Chinese medicine decreases the obstructive uropathy risk in uterovaginal prolapse: A nationwide population-based study. Complement Ther Med. 2019;47:102194.	Not clinical metabolic diseases
2013	Tsai CC, Chang CM, Liao WC, et al. Chinese Herbal Medicine Ameliorated the Development of Chronic Kidney Disease in Patients with Chronic Hepatitis C: A Retrospective Population-Based Cohort Study. Front Pharmacol. 2021;12:670004.	Not clinical metabolic diseases
2014	Chang, C. H., Yang, C. M., & Yang, A. H. (2007). Renal diagnosis of chronic hemodialysis patients with urinary tract transitional cell carcinoma in Taiwan. Cancer: Interdisciplinary International Journal of the American Cancer Society, 109(8), 1487-1492.	Not clinical metabolic diseases
2015	Lin YC, Chang CH, Wang JJ, et al. Conventional Western Treatment Associated With Chinese Herbal Medicine Ameliorates the Incidence of Head and Neck Cancer Among Patients With Esophageal Cancer. Integr Cancer Ther. 2019;18:1534735419838102.	Not clinical metabolic diseases
2016	Chan, T. M., Tse, K. C., Tang, C. S. O., Mok, M. Y., Li, F. K., & Hong Kong Nephrology Study Group. (2005). Long-term study of mycophenolate mofetil as continuous induction and maintenance treatment for diffuse proliferative lupus nephritis. Journal of the American Society of Nephrology, 16(4), 1076-1084.	Not clinical metabolic diseases
2017	Chan, K. W., Ip, T. P., Kwong, A. S. K., Lui, S. L., Chan, G. C. W., Cowling, B. J., ... & Tang, S. C. W. (2016). Semi-individualised Chinese medicine treatment as an adjuvant management for diabetic nephropathy: a pilot add-on, randomised, controlled, multicentre, open-label pragmatic clinical trial. BMJ open, 6(8), e010741.	Not clinical metabolic diseases
2018	Chakraborty, C., Sharma, A. R., Sharma, G., Bhattacharya, M., & Lee, S. S. (2020). SARS-CoV-2 causing pneumonia-associated respiratory disorder (COVID-19): diagnostic and proposed therapeutic options. European Review for Medical & Pharmacological Sciences, 24(7).	Not clinical metabolic diseases
2019	Xu X, Su Z, Xu B, et al. Effects of Shenkang Decoction on Creatinine and Blood Urea Nitrogen in Chronic Renal Failure Hemodialysis Patients: A Randomized Controlled Trial. Evid Based Complement Alternat Med. 2022;2022:1284536.	Not clinical metabolic diseases
2020	Zhou Y, Wu H, Zhang Z, et al. Efficacy and Safety of the Bushen-Shugan Method in Pregnancy Outcomes in Patients with Recurrent Miscarriage Complicated by Anxiety and Depression: A Prospective Randomized Trial. J Ethnopharmacol. 2021;270:113833.	Not clinical metabolic diseases

2021	Madersbacher S, Sampson N, Culig Z. Outcomes and quality of life issues in the pharmacological management of benign prostatic hyperplasia (BPH). World J Urol. 2008;26(6):469-476.	Not clinical metabolic diseases
2022	Zhao DD, Qin XY, Wu YJ, et al. Therapeutic Potential of Diosgenin and Its Major Derivatives against Neurological Diseases: Recent Advances. Oxid Med Cell Longev. 2022;2022:9741409.	Not clinical metabolic diseases
2023	Scott JR, Kodner IJ, Ballard LA, et al. Randomized controlled trial of postoperative belladonna and opium rectal suppositories in vaginal surgery. Obstet Gynecol. 2005;106(1):59-65.	Not clinical metabolic diseases
2024	Alwhaibi M, Balkhi B, Alhossan A, et al. Use of alternative medicine by patients with stage 5 chronic kidney disease. Saudi Pharm J. 2018;26(7):926-930.	Not clinical metabolic diseases
2025	Jin M, Wang L, Yu D, et al. Research on natural products from traditional Chinese medicine in the treatment of myocardial ischemia-reperfusion injury. Phytomedicine. 2020;70:153223.	Not clinical metabolic diseases
2026	Brodaty, H., Ames, D. J., Boundy, K., Snowden, J. A., Storey, E., & Yates, M. (2001). Pharmacological treatment of cognitive deficits in Alzheimer's disease.	Not clinical metabolic diseases
2027	Bright-Gbebry, M., Makambi, K. H., Rohan, J. P., Llanos, A. A., Rosenberg, L., Palmer, J. R., & Adams-Campbell, L. L. (2011). Use of multivitamins, folic acid and herbal supplements among breast cancer survivors: the black women's health study. BMC Complementary and Alternative Medicine, 11, 1-6.	Not clinical metabolic diseases
2028	Blasko, I., Kemmler, G., Krampla, W., Jungwirth, S., Wichart, I., Jellinger, K., ... & Fischer, P. (2005). Plasma amyloid β protein 42 in non-demented persons aged 75 years: effects of concomitant medication and medial temporal lobe atrophy. Neurobiology of aging, 26(8), 1135-1143.	Not clinical metabolic diseases
2029	Bhuvaneswari, M., Elizabeth, P. C., & Nijesh, J. E. (2020). ROLE OF TRIPHALA MOUTHWASH IN GINGIVITIS AND PERIODONTITIS-A NARRATIVE REVIEW. European Journal of Molecular & Clinical Medicine, 7(9), 1133-40.	Not clinical metabolic diseases
2030	Gutiérrez JM, Burnouf T, Harrison RA, et al. Interventions for the management of snakebite envenoming: An overview of systematic reviews. PLoS Negl Trop Dis. 2021;15(5):e0009528.	Not clinical metabolic diseases
2031	Sadeghimanesh S, Hamedi A, Zarshenas MM. Juglans regia L: A review of its traditional uses, phytochemistry, and therapeutic applications. J Ethnopharmacol. 2021;281:114555.	Not clinical metabolic diseases
2032	Liu JP, Yang M, Du XM, et al. Efficacy of a Chinese Herbal Medicine in Providing Adequate Relief of Constipation-predominant Irritable Bowel Syndrome: A Randomized Controlled Trial. Am J Gastroenterol. 2011;106(4):748-755.	Not clinical metabolic diseases

2033	Ben-Aicha, S., Buchanan, J., Punjabi, P., Emanueli, C., & Moscarelli, M. (2023). Efficacy of treatments tested in COVID-19 patients with cardiovascular disease. A meta-analysis. Perfusion, 38(2), 373-383.	Not clinical metabolic diseases
2034	Bazylak, G., & Pan, T. (2010). Anti-adenoviral substances isolated from medicinal plants: current status and future prospects. Planta Medica, 76(12), P505.	Not clinical metabolic diseases
2035	Gupta A, Gupta R, Lal B. The postprandial hypoglycemic activity of fenugreek seed and seeds' extract in type 2 diabetics: A pilot study. Phytother Res. 2001;15(7):628-630.	Not clinical metabolic diseases
2036	Barakat EM, El Wakeel LM, Hagag RS. Effects of Nigella sativa on outcome of hepatitis C in Egypt. World J Gastroenterol. 2013;19(16):2529-2536.	Not clinical metabolic diseases
2037	Chunmiao, B. A. O., & Binbin, L. I. (2021). Traditional Chinese Medicine enhances absorption of lung lesions in corona virus disease 2019 patients. Journal of Traditional Chinese Medicine, 41(6).	Not clinical metabolic diseases
2038	Lu Y, Zhang C, Buchele B, et al. Wogonin and its analogs for the prevention and treatment of cancer: A systematic review. Phytother Res. 2020;34(3):458-471.	Not clinical metabolic diseases
2039	Bai, L., Yu, F., Bai, L., Zhang, Y., Li, Z., Li, P., ... & Ma, Z. (2021). [Retracted] Analysis of Intestinal Flora and Levels of Epidermal Growth Factor Receptor, Interleukin-32, and Gastrin 17 in Patients with Gastric Cancer via Carbon Nanoparticle Laparoscopy. BioMed Research International, 2021(1), 6697597.	Not clinical metabolic diseases
2040	Panahi Y, Hosseini MS, Khalili N, et al. The Use of Curcumin for the Treatment of Renal Disorders: A Systematic Review of Randomized Controlled Trials. Nutrients. 2018;10(7):913.	Not clinical metabolic diseases
2041	Xu J, Xu Y, Qiu H, Kong W, Fang W, Wang Y. Medicinal plants and bioactive natural compounds in the treatment of non-alcoholic fatty liver disease: A clinical review. Phytomedicine. 2019;62:152948.	Not clinical metabolic diseases
2042	Nahidi F, Kariman N, Simbar M, Mojab F. The Effect of Rheum ribes Root (Rhubarb) on Menopausal Hot Flashes: a Randomized Double-Blind, Placebo-Controlled Trial. Iran Red Crescent Med J. 2014;16(4):e16109.	Not clinical metabolic diseases
2043	Hong AR, Kim JH, Kim H, et al. Comparison of the clinical effectiveness of treatments for aromatase inhibitor-induced arthralgia in breast cancer patients: A systematic review with network meta-analysis. Breast Cancer Res Treat. 2020;182(2):273-284.	Not clinical metabolic diseases
2044	Salehi B, Mishra AP, Shukla I, et al. Neuroprotective effects of carvacrol against Alzheimer's disease and other neurodegenerative diseases: A review. Front Aging Neurosci. 2020;12:561680.	Not clinical metabolic diseases
2045	Asri, N., Rezaei-Tavirani, M., Jahani-Sherafat, S., Esmaeili, S., & Khodadoost, M. (2021). Gut healing natural resource's role in management of celiac disease, a brief review. Research Journal of Pharmacognosy, 8(4), 91-100.	Not clinical metabolic diseases

2046	Rahimi-Madiseh M, Malekpour-Tehrani A, Shahriari A, Rafieian-Kopaei M. A systematic review of antiglycation medicinal plants. Int J Pharm Sci Res. 2017;8(1):1-10.	Not clinical metabolic diseases
2047	Stener-Victorin E, Baghaei F, Holm G, Janson PO, Olivecrona G, Lundeberg T. Randomized controlled trial of combined lifestyle and herbal medicine in women with polycystic ovary syndrome. J Clin Endocrinol Metab. 2010;95(2):594-601.	Not clinical metabolic diseases
2048	Anuruddhika Subhashinie Senadheera, S. P., & Ekanayake, S. (2013). Green leafy porridges: how good are they in controlling glycaemic response?. International journal of food sciences and nutrition, 64(2), 169-174.	Not clinical metabolic diseases
2049	Amani, L., Fadaei, F., Shams Ardakani, M., Mirabzadeh Ardakani, M., Sadati Lamardi, S. N., & Shirbeigi, L. (2020). Leech therapy in skin conditions from the viewpoints of Avicenna and modern medicine: Historical review, current applications, and future recommendations. Iranian Journal of Dermatology, 23(4), 168-175.	Not clinical metabolic diseases
2050	Vahdat Shariatpanahi Z, Vahdat Shariatpanahi Z, Nasri H. The effects of medicinal herbs on gut microbiota and metabolic factors in obesity models: A systematic review. Biomed Pharmacother. 2021;137:111371.	Not clinical metabolic diseases
2051	McAnulty SR, McAnulty LS, Nieman DC, et al. A Double-Blind, Cross-Over Study to Examine the Effects of Maritime Pine Extract on Exercise Performance and Postexercise Inflammation, Oxidative Stress, Muscle Soreness, and Damage. J Int Soc Sports Nutr. 2013;10(1):48.	Not clinical metabolic diseases
2052	Al-Haidari, K. A. A., Faiq, T., & Ghareeb, O. (2021). Clinical trial of black seeds against covid–19 in Kirkuk city/Iraq. Indian Journal of Forensic Medicine & Toxicology, 15(3), 3393-3399.	Not clinical metabolic diseases
2053	Patel DK, Kumar R, Laloo D, Hemalatha S. Therapeutic and mechanistic approaches of tridax procumbens flavonoids for the treatment of osteoporosis. Front Pharmacol. 2014;5:275.	Not clinical metabolic diseases
2054	Akram, M., Thiruvengadam, M., Zainab, R., Daniyal, M., Bankole, M. M., Rebezov, M., ... & Okuskhanova, E. (2022). Herbal medicine for the management of laxative activity. Current Pharmaceutical Biotechnology, 23(10), 1269-1283.	Not clinical metabolic diseases
2055	Zhang M, Wang D, Zhu T, et al. Effect of astragalus membranaceus on patients with metastatic non-small cell lung cancer: A retrospective cohort study. Phytomedicine. 2020;78:153305.	Not clinical metabolic diseases
2056	Burnett AL. Erectile dysfunction: Drug treatment. Med Clin North Am. 2018;102(2):349-364.	Not clinical metabolic diseases
2057	Mazidi M, Karimi E, Rezaee R, Ferns GA, Vatanparast H. Co-administration of saffron and chamomile: to determine the efficacy as an adjuvant therapy for mild to moderate depression in human subjects. A pilot randomized clinical trial. J Affect Disord. 2019;245:1082-1087.	Not clinical metabolic diseases

2058	Tiselius HG. Preventive fluid and dietary therapy for urolithiasis: An appraisal of strength, controversies and lacunae of current literature. Urolithiasis. 2019;47(2):95-103.	Not clinical metabolic diseases
2059	Ghadrdoost B, Vafaei AA, Rashidi Nooshabadi MR, et al. Effects of saffron aqueous extract and its main constituent, crocin, on health-related quality of life, depression, and sexual desire in coronary artery disease patients: A double-blind, placebo-controlled, randomized clinical trial. Phytother Res. 2019;33(5):1459-1467.	Not clinical metabolic diseases
2060	Moezi L, Mozaffari-Khosravi H, Asadi S, Fallahzadeh H, Nadjarzadeh A. Topical formulation of Pistacia vera L. pericarp on knee osteoarthritis: A randomized placebo controlled clinical trial. Complement Ther Med. 2019;45:92-96.	Not clinical metabolic diseases
2061	祝一叶,周恩超.周恩超益肾解毒法分期治疗慢性肾衰竭验案撷萃[J].浙江中医药大学学报,2019,43(09):971-974.DOI:10.16466/j.issn1005-5509.2019.09.010.	Not clinical metabolic diseases
2062	张爱萍.大剂量静脉输注免疫球蛋白治疗大疱性类天疱疮 5 例[J].现代中西医结合杂志,2007,(26):3842.	Not clinical metabolic diseases
2063	朱如敏,杨军.复方氨酚烷胺致全血细胞减少一例[J].临床误诊误治,2016,29(S1):92.	Not clinical metabolic diseases
2064	朱敏,杨洪艳,王小云,等."病证结合"方案治疗排卵障碍性不孕症的随机对照研究[J].广州中医药大学学报,2017,34(06):815-818.DOI:10.13359/j.cnki.gzxbtcm.2017.06.006.	Not clinical metabolic diseases
2065	叶峥,隋馥勇,徐冰宁,等.基底细胞痣综合征 1 例[J].口腔医学研究,2025,41(04):345-347.DOI:10.13701/j.cnki.kqyxyj.2025.04.013.	Not clinical metabolic diseases
2066	马玉萍, 韩晓宇, & 关久利. (2014). 自拟方药治疗频复发型肾病综合症 52 例. 国际中医中药杂志, 36(1), 55-57.	Not clinical metabolic diseases
2067	邱建萍,孙红.青春期多囊卵巢综合征中西医研究进展[J].新疆中医药,2017,35(02):104-107.	Not clinical metabolic diseases
2068	罗庆,陆敏康,陆晓东.MEBT/MEBO治疗不同类型手指开放性损伤临床体会[J].中国烧伤创疡杂志,2017,29(01):32-36.	Not clinical metabolic diseases
2069	罗俊,韦敬土,郑琴.红花黄色素注射液致严重斑丘疹 1 例[J].黔南民族医专学报,2018,31(02):144+149.	Not clinical metabolic diseases
2070	尹刚,段惠春,陈德斌,等.药物性肝病 78 例临床分析[J].甘肃医药,2014,33(08):599-601.DOI:10.15975/j.cnki.gsyy.2014.08.023.	Not clinical metabolic diseases

2071	吕静,刘维.中西医结合治疗RS3PE综合征 2 例报告并文献复习[J].风湿病与关节炎,2020,9(10):31-36.	Not clinical metabolic diseases
2072	路素英.中药坐浴配合肛周皮下注射治疗慢性肛门湿疹[J].中国医药指南,2013,11(21):675-676.DOI:10.15912/j.cnki.gocm.2013.21.610.	Not clinical metabolic diseases
2073	陆晓英.如意金黄散与芒硝粉外敷对剖宫产切口并发症的疗效观察[J].医疗装备,2015,28(18):116-117.	Not clinical metabolic diseases
2074	陆吴超,季卫锋,马镇川.关节镜下清创后持续灌洗联合中药口服治疗全膝关节置换术后急性期感染[J].中医正骨,2015,27(01):51-53.	Not clinical metabolic diseases
2075	陈小娟.基于代谢组学技术对补肾化痰方联合二甲双胍治疗肾虚痰湿型PCOS的临床研究[D].南京中医药大学,2020.DOI:10.27253/d.cnki.gnjzu.2020.000287.	Not clinical metabolic diseases
2076	邓颜梅.养老机构应用湿润烧伤膏治疗压疮效果观察[J].中国烧伤创疡杂志,2019,31(02):109-112.	Not clinical metabolic diseases
2077	刘维红,刘涛,张杰,等.脑梗死后痉挛性瘫痪中医证候及相关因素研究[J].辽宁中医杂志,2017,44(08):1586-1588+1789.DOI:10.13192/j.issn.1000-1719.2017.08.005.	Not clinical metabolic diseases
2078	刘维,廖列辉,林颖,等.血管骨肥大综合征伴下肢溃疡 1 例[J].中国麻风皮肤病杂志,2009,25(07):538-539.	Not clinical metabolic diseases
2079	刘庆春,董广卫.补肾健骨汤配合手法治疗增生性膝关节炎 115 例[J].中国实用医药,2011,6(24):165.DOI:10.14163/j.cnki.11-5547/r.2011.24.019.	Not clinical metabolic diseases
2080	刘培霞,张小花.中西医结合治疗重度子痫前期致低蛋白血症、腹水 1 例[J].广西中医药大学学报,2013,16(01):38-39.	Not clinical metabolic diseases
2081	刘娜.子宫内膜息肉与子宫内膜容受性的实验与临床研究[D].新疆医科大学,2017.	Not clinical metabolic diseases
2082	刘佳佳.放射性直肠炎危险因素分析及中西医结合保留灌肠系统评价[D].山西中医药大学,2021.DOI:10.27820/d.cnki.gszxy.2021.000058.	Not clinical metabolic diseases
2083	韦秀珍,项淑英,邢洪林.中药治疗慢性肾炎蛋白尿的疗效观察[J].中国初级卫生保健,2005,(08):65.	Not clinical metabolic diseases
2084	林贤仁,袁卓珺.广西桂北地区勃起功能障碍患者中医证型流行病学调查[J].中国性科学,2019,28(06):127-130.	Not clinical metabolic diseases

2085	李艳,周莉.中西医结合治疗小儿急性肾小球肾炎临床观察[J].中华中医药杂志,2007,(08):576.	Not clinical metabolic diseases
2086	李云宁, 郭振军, 庞来祥 and 马小莹, 2012, 严重特应性皮炎 1 例的中医治疗, 中国美容医学	Not clinical metabolic diseases
2087	李永川,吴德慧,黄华民,等.高原红细胞增多症对妇科恶性肿瘤术后深静脉血栓形成的影响和预防[J].高原医学杂志,2009,19(S1):43.	Not clinical metabolic diseases
2088	李颖,汪永忠.注射用鹿瓜多肽引起过敏反应 2 例[J].药物流行病学杂志,2010,19(02):116.DOI:10.19960/j.cnki.issn1005-0698.2010.02.025.	Not clinical metabolic diseases
2089	李亚,张越,庞午,等.中医药治疗特发性黄斑中心凹旁毛细血管扩张症 1 例[J].眼科学报,2018,33(04):218-225.	Not clinical metabolic diseases
2090	李欣航,占永立.伴肾小管间质纤维化特发性膜性肾病临床病理及中医证型特征分析[J].中国中西医结合肾病杂志,2019,20(10):863-866.	Not clinical metabolic diseases
2091	李晓慧.通痹止痛汤治疗干性坐骨神经痛 178 例[J].陕西中医,2009,30(08):1043.	Not clinical metabolic diseases
2092	李维华.一氧化碳中毒迟发性脑病治验[J].山东中医杂志,2007,(04):278.DOI:10.16295/j.cnki.0257-358x.2007.04.037.	Not clinical metabolic diseases
2093	王宝仁,张艾丽,方杰.伴黏膜损害的急性泛发性扁平苔藓 1 例[J].皮肤病与性病,2018,40(06):885-886.	Not clinical metabolic diseases
2094	李凯,张宁苏,周立江.胃癌术后患者中医证型分布特点及与脂代谢相关性研究[J].中医临床研究,2022,14(31):67-70.	Not clinical metabolic diseases
2095	李俊义. (2009). 中西医结合治疗慢性心力衰竭患者的临床疗效观察. 中华中西医学杂志, 7(011), 76-77.	Not clinical metabolic diseases
2096	李军,张莉莉,张凤新.重组人酸性成纤维细胞生长因子促进慢性溃疡性创面愈合的临床研究[J].中国医药导报,2014,11(31):49-51+60.	Not clinical metabolic diseases
2097	李久现,张晓丹,冯帅英,等.穴位埋线合补肾利湿活血汤对肥胖型PCOS体质量指数及性激素的影响[J].光明中医,2019,34(08):1220-1223.	Not clinical metabolic diseases
2098	李军,张莉莉,张凤新.重组人酸性成纤维细胞生长因子促进慢性溃疡性创面愈合的临床研究[J].中国医药导报,2014,11(31):49-51+60.	Not clinical metabolic diseases

2099	李衡贵. (2016). 派特灵治疗老年尖锐湿疣的临床观察. 中国现代医生, 54(28), 100-102.	Not clinical metabolic diseases
2100	韩昭琳.中西医结合治疗痛风性关节炎疗效观察[J].河北北方学院学报(自然科学版),2016,32(08):39-40.	Not clinical metabolic diseases
2101	李储杰.化湿祛瘀法治疗眩晕 2 例[J].中国中医药现代远程教育,2011,9(12):81.	Not clinical metabolic diseases
2102	李伯华,程海英,郑玉红.王莒生药、针、浴结合治疗红皮病型银屑病脉案 1 则[J].上海中医药杂志,2010,44(05):21-22.DOI:10.16305/j.1007-1334.2010.05.023.	Not clinical metabolic diseases
2103	李冰,王德惠.刘文峰教授治疗急性痛风性关节炎医案 1 则[J].吉林中医药,2013,33(10):1063-1064.DOI:10.13463/j.cnki.jlzyy.2013.10.009.	Not clinical metabolic diseases
2104	郎睿.健脾祛湿和络方治疗特发性膜性肾病的队列研究及基于肠道菌群的机制探索[D].中国中医科学院,2019.	Not clinical metabolic diseases
2105	卢博, 于子辰, 郝英利, 赵丽萍, & 张士发. (2023). 获得性反应性穿通性胶原病 1 例. 大连医科大学学报, 45(4), 376-378.	Not clinical metabolic diseases
2106	V·卡拉亚尼 ,E·萨蒂 ,G·A·佩内洛普 , et al.MEBO与碘伏联合保湿霜的成本及多项指标比较[J].中国烧伤创疡杂志,2012,24(05):360-363.	Not clinical metabolic diseases
2107	金莺,韩俊秋,宋勤丽,等.健脾滋肾法治疗痰湿壅滞证性早熟女童远期影响的回顾性队列研究[J].中国中医药信息杂志,2023,30(02):129-134.DOI:10.19879/j.cnki.1005-5304.202205016.	Not clinical metabolic diseases
2108	贾淑慧.龙珠软膏治疗严重外阴溃疡验案 1 例[J].吉林中医药,2008,(08):598.DOI:10.13463/j.cnki.jlzyy.2008.08.036.	Not clinical metabolic diseases
2109	黄奕娟,楼雅芳,丁旭春,等.误诊为肺部疾病的ANCA相关性小血管炎 20 例分析[J].中华全科医学,2014,12(03):386-388.DOI:10.16766/j.cnki.issn.1674-4152.2014.03.042.	Not clinical metabolic diseases
2110	黄春晓,乔旭东.彩色多普勒超声诊断无名动脉夹层动脉瘤 1 例[J].当代医学,2013,19(02):46.	Not clinical metabolic diseases
2111	胡长军.针刺结合手法治疗阳痿证 112 例临床观察[J].针灸临床杂志,2008,(05):16-17.	Not clinical metabolic diseases
2112	胡晓靖.针灸加中药治疗闭经 15 例临床观察[J].中国中医药科技,2009,16(05):369.	Not clinical metabolic diseases

2113	胡涛,龚维,杨明杰,等.类风湿关节炎阶梯治疗临床研究[J].亚太传统医药,2016,12(12):113-114.	Not clinical metabolic diseases
2114	胡顺金,汪飞,郭茹叶,等.基于糖皮质激素给药阶段辨证治疗原发性肾病综合征 31 例[J].安徽中医学院学报,2013,32(05):19-23.	Not clinical metabolic diseases
2115	何映.从肝辨治阴茎勃起功能障碍 1 例[J].湖南中医杂志,2006,(06):40-41.DOI:10.16808/j.cnki.issn1003-7705.2006.06.023.	Not clinical metabolic diseases
2116	王秋芳.高压氧治疗顽固性溃疡 1 例[J].菏泽医专学报,1998,(01):71.	Not clinical metabolic diseases
2117	浩光东,李艳春.缬沙坦致顽固性咳嗽 1 例[J].人民军医,2016,59(05):472.	Not clinical metabolic diseases
2118	韩其芳.脂肪肝治验治法四则[J].中医药临床杂志,2009,21(04):357-358.DOI:10.16448/j.cjtcm.2009.04.026.	Not clinical metabolic diseases
2119	韩曼,何东仪,尹维贤,等.205 例痛风患者中医证候与临床特征分析[J].北京中医药,2020,39(12):1286-1290.DOI:10.16025/j.1674-1307.2020.12.017.	Not clinical metabolic diseases
2120	韩莉,邓跃毅,陈以平,等.益气活血化湿方案治疗特发性膜性肾病的疗效观察及其相关因素分析[C]//中国中西医结合肾脏病专委会.第十一届全国中西医结合肾脏病学术会议论文汇编.连云港市中医院肾内科;上海中医药大学附属龙华医院肾内科;,2010:284-285.	Not clinical metabolic diseases
2121	韩丽.老年人过度降压致急性心肌梗死 1 例[J].中国医学创新,2011,8(03):98.	Not clinical metabolic diseases
2122	韩静,赵素萍.服用中药地龙致过敏 2 例临床报道[J].内蒙古中医药,2014,33(01):24.DOI:10.16040/j.cnki.cn15-1101.2014.01.257.	Not clinical metabolic diseases
2123	郭翔飞, 2021，新型冠状病毒肺炎重症营养治疗 1 例，山西医药杂志	Not clinical metabolic diseases
2124	付生芳，李香艳, & 王海燕. (2012). 湿润烧伤膏在断指再植术后换药中的应用. 成都医学院学报, 7(03Z), 296-297.	Not clinical metabolic diseases
2125	方妍妍,刘健,忻凌,等.新风胶囊降低强直性脊柱炎患者终点事件发生风险的队列研究[J].北京中医药大学学报,2023,46(05):607-616.	Not clinical metabolic diseases
2126	方晓江，钱宝庆, & 赵芊. (2014). 麝香保心丸辅治慢性心力衰竭疗效观察. 浙江中西医结合杂志, 24(11), 1009-1010.	Not clinical metabolic diseases

2127	方群英,吴丽敏,孙秀英,等.不同中医证候多囊卵巢综合征不孕患者促排加指导同房临床特征和妊娠结局比较[J].中华中医药学刊,2019,37(11):2662-2666.DOI:10.13193/j.issn.1673-7717.2019.11.024.	Not clinical metabolic diseases
2128	樊越,胡刚,赵红鱼,等.肠息肉的生物学特征与中医证候类型之间相关性研究[J].内蒙古中医药,2018,37(07):1-3.DOI:10.16040/j.cnki.cn15-1101.2018.07.001.	Not clinical metabolic diseases
2129	翟瑞柏.商陆麻黄汤治疗急性水肿验案 2 则[J].山西中医,2008,(05):30-31.	Not clinical metabolic diseases
2130	邓路娟,闫铭,朱建良.生脉和丹参注射液联合应用治疗老年心力衰竭 46 例临床观察[J].河北医药,2008,(05):708.	Not clinical metabolic diseases
2131	褚铮,张辰惠,刘维,等.补中益气汤合用两性霉素B治疗肺部烟曲霉菌感染 1 例[J].中国中医急症,2013,22(09):1632-1633.	Not clinical metabolic diseases
2132	程梦慧,黎玉婵,卢咏梅,等.1 例乳腺癌合并糖尿病患者四度放射性皮炎的护理[J].护理实践与研究,2018,15(09):158-159.	Not clinical metabolic diseases
2133	成妙. (2020). 710 例多囊卵巢综合征患者的生活习惯与中医证型分布规律及其相关性研究 (Doctoral dissertation, 成都: 成都中医药大学).	Not clinical metabolic diseases
2134	陈余妍,唐金模.祛湿化浊法治疗肝癖 1 例[J].中西医结合肝病杂志,2019,29(05):469-471.	Not clinical metabolic diseases
2135	顾春,崔成军.成人肛周Kaposi水痘样疹 1 例[J].中国中西医结合皮肤性病学杂志,2022,21(03):260-261.	Not clinical metabolic diseases
2136	陈俊杰, 肖鲁伟, & 童培建. (2006). 股骨头坏死危险因素病例对照研究. 中国骨伤, 19(11), 671-673.	Not clinical metabolic diseases
2137	沈余明,韩冬,覃凤均.人工全膝关节置换术后切口缘皮肤坏死的修复[J].中国修复重建外科杂志,2014,28(08):985-987.	Not clinical metabolic diseases
2138	沈余明,韩冬,覃凤均.人工全膝关节置换术后切口缘皮肤坏死的修复[J].中国修复重建外科杂志,2014,28(08):985-987.	Not clinical metabolic diseases
2139	沈小钰,郭竹英,金莹莹,等.基于小儿遗尿症临床症候群选用缩泉胶囊和醒脾养儿颗粒的疗效观察[J].中华全科医学,2019,17(02):173-175+204.DOI:10.16766/j.cnki.issn.1674-4152.000631.	Not clinical metabolic diseases
2140	沈组增,谢峥伟,吕红梅,等.温阳利水、化瘀泄浊法延缓慢性肾功能衰竭进展临床研究[J].中国中医药信息杂志,2007,(04):17-18+60.	Not clinical metabolic diseases

2141	柴光德,韩宗宝,高振臣,等.骨关节炎患者诊治分析[J].河北医药,2011,33(15):2268-2269.	Not clinical metabolic diseases
2142	曹孙航,王睿智,李丹丹,等.新疆地区急诊医疗保健相关性肺炎成年患者病原体分布及耐药特点分析[J].中国全科医学,2018,21(23):2837-2843.	Not clinical metabolic diseases
2143	蔡骏逸,吴逢春,李荷花,等.综合医院就诊患者焦虑症状与中医体质的相关性研究[J].新中医,2021,53(16):192-195.DOI:10.13457/j.cnki.jncm.2021.16.050.	Not clinical metabolic diseases
2144	赵慧君.中西医结合治疗慢性充血性心力衰竭临床观察[J].新中医,2018,50(06):65-68.DOI:10.13457/j.cnki.jncm.2018.06.016.	Not clinical metabolic diseases
2145	包勤斌,徐新荣.中药联合雷珠单抗治疗湿性年龄相关性黄斑变性疗效的Meta分析[J].湖南中医杂志,2021,37(10):161-167.DOI:10.16808/j.cnki.issn1003-7705.2021.10.055.	Not clinical metabolic diseases
2146	包勤斌,徐新荣.中药联合雷珠单抗治疗湿性年龄相关性黄斑变性疗效的Meta分析[J].湖南中医杂志,2021,37(10):161-167.DOI:10.16808/j.cnki.issn1003-7705.2021.10.055.	Not clinical metabolic diseases
2147	Long, L., Soeken, K., & Ernst, E. (2001). Herbal medicines for the treatment of osteoarthritis: a systematic review. Rheumatology, 40(7), 779-793.	Not clinical metabolic diseases
2148	Baugh RF, Basura GJ, Ishii LE, et al. Clinical practice guideline: Bell's Palsy. Otolaryngol Head Neck Surg. 2013;149(3_suppl):S1-S27.	Not clinical metabolic diseases
2149	段姣妞,杜伟,侯睿宏,等.类脂质渐进性坏死 1 例[J].北京大学学报(医学版),2019,51(06):1182-1184.DOI:10.19723/j.issn.1671-167X.2019.06.037.	Not clinical metabolic diseases
2150	Yang, L., Zhang, L., Liu, S., Liu, X., & Guo, X. (2021). Defining Minimal Clinically Important Difference of Proteinuria in Diabetic Kidney Disease Adults Treated with Integrative Chinese and Conventional Medicine. European Journal of Integrative Medicine, 48, 101987.	Not related to Oral CHM
2151	Olamoyegun MA, Olamoyegun KD, Adeyemi O. Adherence to anti-diabetic drug therapy and self-management practices among type-2 diabetics in Nigeria. J Community Med Prim Health Care. 2015;27(2):61-67.	Not related to Oral CHM
2152	Vishwas, N. A., & Raj, K. K. (2013). An Ayurvedic polyherbal formulation PDBT for dyslipidemia and prevention of coronary artery disease (CAD) in pre-diabetic individuals.	Not related to Oral CHM
2153	Sitorus, J., Hadju, V., Jafar, N., Amiruddin, R., Syam, A., Mahmudiono, T., ... & Moedjiono, A. I. (2022). Artocarpus altilis Extract Capsules Reduce Fasting Blood Glucose in Prediabetes. Open Access Macedonian Journal of Medical Sciences, 10(A), 315-320.	Not related to Oral CHM

2154	Othong, R., Trakulsrichai, S., & Wananukul, W. (2017). Diospyros rhodocalyx (Tako-Na), a Thai folk medicine, associated with hypokalemia and generalized muscle weakness: A case series. Clinical Toxicology, 55(9), 986-990.	Not related to Oral CHM
2155	Eftekhar E, Yousefzadeh N, Mohammad KZ, et al. Effects of Satureja khuzestanica on serum glucose, lipids and markers of oxidative stress in patients with type 2 diabetes mellitus: A double-blind randomized controlled trial. Evid Based Complement Alternat Med. 2012;2012:718386. doi:10.1155/2012/718386	Not related to Oral CHM
2156	Visen, P. K. S., Visen, A. S., Visen, S. S., Buttar, H. S., & Singh, R. B. (2015). Management of Type 2 diabetes and atherosclerosis with alternative therapies. World Heart Journal, 7(1), 63.	Not related to Oral CHM
2157	Xu, Z. H., Gao, Y. Y., Zhang, H. T., Ruan, K. F., & Feng, Y. (2018). Progress in experimental and clinical research of the diabetic retinopathy treatment using traditional Chinese medicine. The American journal of Chinese medicine, 46(07), 1421-1447.	Not related to Oral CHM
2158	Yusni, Y., Zufry, H., Meutia, F., & Sucipto, K. W. (2018). The effects of celery leaf (Apium graveolens L.) treatment on blood glucose and insulin levels in elderly pre-diabetics. Saudi medical journal, 39(2), 154.	Not related to Oral CHM
2159	Tsai, C. H., Chen, E. C. F., Tsay, H. S., & Huang, C. J. (2012). Wild bitter gourd improves metabolic syndrome: a preliminary dietary supplementation trial. Nutrition Journal, 11, 1-9.	Not related to Oral CHM
2160	Abdullah Tauheed, H., Ali, A., & Zaigham, M. ZANJABEEL (ZINGIBER OFFICINALE ROSC.): A HOSEHOLD RHIZOME WITH IMMENCE THERAPEUTIC POTENTIAL AND ITS UTILIZATION IN UNANI MEDICINE.	Not related to Oral CHM
2161	Zhang, Y, Li, X, Fei, Y and Liu, J, 2017, Considerations on multiple testing procedures in randomized controlled trials of Chinese Herbal Medicine, BMC Complementary and Alternative Medicine	Not RCTs
2162	Thounaojam, M. C., Nammi, S., & Jadeja, R. (2016). Natural products for the treatment of obesity, metabolic syndrome, and type 2 diabetes 2016. Evidence-based Complementary and Alternative Medicine: eCAM, 2016, 9072345.	Not RCTs
2163	Yang, K., Wang, Y., Li, Y. W., Chen, Y. G., Xing, N., Lin, H. B., ... & Yu, X. P. (2022). Progress in the treatment of diabetic peripheral neuropathy. Biomedicine & Pharmacotherapy, 148, 112717.	Not RCTs
2164	Khorasani, S., Azizi, H., Yousefi, M., Salari, R., Bahrami-Taghanaki, H., & Behravanrad, P. (2017). An evidence based review on integrative medicine in weight control. Complementary Medicine Journal, 7(1), 1828-1850.	Methodological error
2165	Wang, X, Pang, B, Zhao, L, Xuemin, Z and Zhang, Y, 2018, How to explore the effectiveness of traditional chinese herbal medicine in a pragmatic randomized controlled trial? Experiences from treating diabetic kidney	Methodological error

	disease, Global Advances in Health and Medicine	
2166	于洋.基于数据和典型病例分析清热利湿化瘀法治疗消渴痹证[D].山东中医药大学,2020.DOI:10.27282/d.cnki.gsdzu.2020.000473.	Methodological error
2167	[Plant therapy in diabetes?]. (2005). Praxis, 94 14, 572.	Methodological error
2168	宋其友.中西医结合治疗糖尿病并发疮痈临床体会[J].心血管康复医学杂志,2000,(01):72-73.	Methodological error
2169	钱孝贤,陈燕铭,吴伟康,等.保心胶囊对急性心肌缺血犬一氧化氮和内皮素的影响[J].中国临床药学杂志,2000,(05):280-283.DOI:10.19577/j.cnki.issn10074406.2000.05.006.	Methodological error
2170	Jo Barnes, 2002, PHYTOTHERAPY RESEARCH CONFERENCE: Royal Concert Hall, Glasgow, Scotland, April 19, Complementary Therapies in Medicine, 10(3), 184.	Methodological error
2171	Jia, J. P., Wu, Y. X., & Xie, G. H. (2007). Clinical observation on treatment of albuminuria in patients with pregnancy-induced hypertension syndrome in puerperium by Xiaobai Decoction. Zhongguo Zhong xi yi jie he za zhi Zhongguo Zhongxiyi Jiehe Zazhi= Chinese Journal of Integrated Traditional and Western Medicine, 27(7), 644-646.	Not clinical metabolic diseases
2172	Kalman DS, Feldman S, Feldman R, Schwartz HI, Krieger DR. Effect of a proprietary Magnolia and Phellodendron extract on stress levels in healthy women: a pilot, double-blind, placebo-controlled clinical trial. Nutr J. 2008;7:11. doi:10.1186/1475-2891-7-11	Not clinical metabolic diseases
2173	Huan, J. M., Ma, X. T., Li, S. Y., Hu, D. Q., Chen, H. Y., Wang, Y. M., ... & Wang, Y. F. (2023). Effect of botanical drugs in improving symptoms of hypertensive nephropathy: analysis of real-world data, retrospective cohort, network, and experimental assessment. Frontiers in Pharmacology, 14, 1126972.	Not clinical metabolic diseases
2174	Khazaei, H., Pesce, M., Patruno, A., Aneva, I. Y., & Farzaei, M. H. (2021). Medicinal plants for diabetes associated neurodegenerative diseases: A systematic review of preclinical studies. Phytotherapy Research, 35(4), 1697-1718.	Not clinical metabolic diseases
2175	Xiao, W., Liu, J., & Liu, L. Y. (2002). Effect of jianpi wenshen decoction on serum gastrin, plasma motilin and somatostatin in patients of diabetic diarrhea. Zhongguo Zhong xi yi jie he za zhi Zhongguo Zhongxiyi Jiehe Zazhi= Chinese Journal of Integrated Traditional and Western Medicine, 22(8), 587-589.	Not clinical metabolic diseases
2176	Liu, R., Zhao, Y., Wu, Y., Guo, M., Duan, Y., Ye, J., & Lu, X. (2020). Individualized Chinese medicine for the treatment of diabetic patients with dry eye disease: a single-case randomized controlled protocol. Medicine, 99(1), e18459.	Not clinical metabolic diseases

2177	钱查娇,周玉娟,方巧,等.和营健脾、清热利水解毒法治疗糖尿病黄斑水肿 30 例[J].江西中医学院学报,2013,25(05):32-34.	Not clinical metabolic diseases
2178	杨会蓉,黎元惠.化湿活血汤联合西药治疗 2 型糖尿病下肢周围神经病变随机平行对照研究[J].实用中医内科杂志 ,2016,30(03):76-78.DOI:10.13729/j.issn.1671-7813.2016.03.33	Not clinical metabolic diseases
2179	李锋,李晓苗,王汉民,等.化瘀利水方治疗糖尿病性肾病 53 例临床观察[J].安徽中医学院学报,2005,(03):15-17.	Not clinical metabolic diseases
2180	章梅娇,程小平.黄葵胶囊治疗糖尿病肾病效果及对肾功能、机体微炎症的影响[J].华夏医学 ,2017,30(06):59-62.DOI:10.19296/j.cnki.1008-2409.2017-06-016.	Not clinical metabolic diseases
2181	龚岚.黄莲与冰绷散治疗糖尿病足溃疡的效果观察[J].护理学报 ,2010,17(12):62-63.DOI:10.16460/j.issn1008-9969.2010.12.023.	Not clinical metabolic diseases
2182	张毅,张敏.活血祛风除湿中药足浴联合口服甲钴胺片治疗糖尿病周围神经病变临床观察[J].中国中医药信息杂志,2014,21(12):22-24.	Not clinical metabolic diseases
2183	廖丽容,谢燕珍,何飞杏.康复新液联合胰岛素治疗糖尿病压疮的效果观察[J].当代护士(下旬刊),2014,(11):138-139.	Not clinical metabolic diseases
2184	Yang, W., You, L., Xie, Y. M., Yang, H., & Zhuang, Y. (2013). Analysis of pragmatic clinical use of shuxuening injection. Zhongguo Zhong yao za zhi= Zhongguo Zhongyao Zazhi= China Journal of Chinese Materia Medica, 38(18), 3150-3154.	Not clinical metabolic diseases
2185	Zheng, R. W., Liu, D., Eric, T. E., Ning, Y. Z., Chen, L. L., Hu, H., & Ren, Y. (2017). A case study of Ramsay Hunt Syndrome in conjunction with cranial polyneuritis. Medicine, 96(47), e8833.	Not clinical metabolic diseases
2186	Xia, N., Jiang, C., Zhou, Y., Huang, Q., Hu, L., Zeng, H., ... & Yuan, Z. (2022). A double-blind, randomized, placebo-controlled, single-center clinical trial of jiaotaiwan for the treatment of insomnia symptoms caused by disharmony of the heart and kidney. Frontiers in Pharmacology, 13, 1011003.	Not clinical metabolic diseases
2187	Yun, S. P., Jung, W. S., Park, S. U., Moon, S. K., Ko, C. N., Cho, K. H., ... & Bae, H. S. (2005). Anti-hypertensive effect of Chunghyul-dan (Qingxue-dan) on stroke patients with essential hypertension. The American Journal of chinese Medicine, 33(03), 357-364.	Not clinical metabolic diseases
2188	Stuby, J., Gravestock, I., Wolfram, E., Pichierri, G., Steurer, J., & Burgstaller, J. M. (2019). Appetite-suppressing and satiety-increasing bioactive phytochemicals: A systematic review. Nutrients, 11(9), 2238.	Not clinical metabolic diseases
2189	Shao, C., Dong, W., & Zhang, H. (2021). Application of Guijiaosan Shenque acupoint paste can improve the scores of obesity, endocrine and TCM symptoms in treating obese polycystic ovary syndrome. American Journal of Translational Research, 13(9), 10694.	Not clinical metabolic diseases

2190	Sun, X., Sun, H., Zhang, J., & Ji, X. (2016). Artemisia Extract Improves Insulin Sensitivity in Women With Gestational Diabetes Mellitus by Up-Regulating Adiponectin. The Journal of Clinical Pharmacology, 56(12), 1550-1554.	Not clinical metabolic diseases
2191	Zhang, X. W., Liu, X. X., Yong, J., Wei, L. F., Ling, C. Y., Jin, Y., ... & Wang, L. (2021). Association of anti-phospholipase A2 receptor antibody with the efficacy of traditional Chinese medicine (Shenqi particle) for patients with idiopathic membranous nephropathy: a prospective, cohort clinical study. Chinese Medical Journal, 134(18), 2252-2254.	Not clinical metabolic diseases
2192	Pach, D., Willich, S. N., & Becker-Witt, C. (2002). Availability of research results on traditional Chinese pharmacotherapy. Forschende Komplementärmedizin und Klassische Naturheilkunde/Research in Complementary and Classical Natural Medicine, 9(6), 352-358.	Not clinical metabolic diseases
2193	Wu X, Zhou T, Zu M, et al. Benefits and Safety of Chinese Herbal Medicine in Treating Psoriasis: An Overview of Systematic Reviews. Front Pharmacol. 2021;12:654255. doi:10.3389/fphar.2021.654255	Not clinical metabolic diseases
2194	Rosano GMC, Vitale C, Marazzi G, et al. Bergamot polyphenolic fraction counteracts erectile dysfunction occurring in patients suffering from type 2 diabetes. Fitoterapia. 2012;83(2):336-341. doi:10.1016/j.fitote.2011.11.002	Not clinical metabolic diseases
2195	Vasileva, L. V., Marchev, A. S., & Georgiev, M. I. (2018). Causes and solutions to “globesity”: The new fa (s) t alarming global epidemic. Food and Chemical Toxicology, 121, 173-193.	Not clinical metabolic diseases
2196	Zhou, W. Q., Gao, P., & Xie, Y. M. (1995). Clinical and laboratory studies of effect of longevity-antihypertensive-mixture on elderly hypertension with kidney deficiency. Zhongguo Zhong xi yi jie he za zhi Zhongguo Zhongxiyi Jiehe Zazhi= Chinese Journal of Integrated Traditional and Western Medicine, 15(9), 532-535.	Not clinical metabolic diseases
2197	Shen, W., Li, J., Wu, X., & Liu, B. (2003). Clinical observation of wenshen xiezhuo decoction in treating pations with chronic renal failure. Zhong yao cai= Zhongyaocai= Journal of Chinese Medicinal Materials, 26(12), 914-917.	Not clinical metabolic diseases
2198	Zhang, T., Zhang, J., & Lu, C. (1998). Clinical observation on preventing and treating coronary atherosclerotic heart disease with tongmai jiangzhi oral liquid. Zhongguo Zhong xi yi jie he za zhi Zhongguo Zhongxiyi Jiehe Zazhi= Chinese Journal of Integrated Traditional and Western Medicine, 18(2), 77-80.	Not clinical metabolic diseases
2199	Sha, H., Zhao, J., & Guo, S. (2016). Combination of Qinzhu Liangxue decoction and acitretin on the treatment of psoriasis vulgaris: a randomized controlled trail. Int J Clin Exp Med, 9(4), 7256-64.	Not clinical metabolic diseases

2200	Rao, Y., Wang, Y., Lin, Z., Zhang, X., Ding, X., Yang, Y., ... & Zhang, B. (2023). Comparative efficacy and pharmacological mechanism of Chinese patent medicines against anthracycline-induced cardiotoxicity: An integrated study of network meta-analysis and network pharmacology approach. Frontiers in Cardiovascular Medicine, 10, 1126110.	Not clinical metabolic diseases
2201	Panahi, Y., Izadi, M., Sayyadi, N., Rezaee, R., Jonaidi-Jafari, N., Beiraghdar, F., ... & Sahebkar, A. (2015). Comparative trial of Aloe vera/olive oil combination cream versus phenytoin cream in the treatment of chronic wounds. Journal of wound care, 24(10), 459-465.	Not clinical metabolic diseases
2202	Sujanamulk, B., Sunder, S. S., Pawar, B. R., Rajalakshmi, C., & Maloth, K. N. (2020). Comparison of antifungal efficacy of ethanolic extracts of Woodfordia fruticosa leaf and Punica granatum peel in uncontrolled diabetic patients wearing removable dentures: A randomized controlled clinical trial. Current Medical Mycology, 6(3), 15.	Not clinical metabolic diseases
2203	Shen, X., Zou, S., Jin, J., Liu, Y., Wu, J., & Qu, L. (2022). Dengzhan Shengmai capsule versus aspirin in the treatment of carotid atherosclerotic plaque: a single-centre, non-inferiority, prospective, randomised controlled trial. Phytomedicine, 106, 154408.	Not clinical metabolic diseases
2204	Navarro, J. C., Chen, C. L., Lee, C. F., Gan, H. H., Lao, A. Y., Baroque, A. C., ... & CHIMES and CHIMES-E Study Investigators. (2017). Durability of the beneficial effect of MLC601 (NeuroAiD™) on functional recovery among stroke patients from the Philippines in the CHIMES and CHIMES-E studies. International Journal of Stroke, 12(3), 285-291.	Not clinical metabolic diseases
2205	Zhao, J. V., Yeung, W. F., Chan, Y. H., Vackova, D., Leung, J. Y., Ip, D. K., ... & Schooling, C. M. (2021). Effect of berberine on cardiovascular disease risk factors: a mechanistic randomized controlled trial. Nutrients, 13(8), 2550.	Not clinical metabolic diseases
2206	Tsai, F. J., Ho, T. J., Cheng, C. F., Liu, X., Tsang, H., Lin, T. H., ... & Lin, Y. J. (2017). Effect of Chinese herbal medicine on stroke patients with type 2 diabetes. Journal of ethnopharmacology, 200, 31-44.	Not clinical metabolic diseases
2207	Yi, S., Ran, L., & Gu, X. H. (2006). Effects of pulmonary arterial perfusion with shenqi fuzheng injection on lung injury during cardiopulmonary bypass. Zhongguo Zhong xi yi jie he za zhi Zhongguo Zhongxiyi Jiehe Zazhi= Chinese Journal of Integrated Traditional and Western Medicine, 26(10), 938-941.	Not clinical metabolic diseases
2208	Wang, H., Liu, N., Wei, Y., Pei, H., Liu, M., Diao, X., ... & Li, H. (2019). Efficacy and safety of Shenmayizhi decoction as an adjuvant treatment for vascular dementia: Study protocol for a randomized controlled trial. Medicine, 98(50), e18326.	Not clinical metabolic diseases
2209	Sha, Z., Zhao, Z., Li, N., Xiao, S., Li, O., Zhang, J., ... & Xu, J. (2023). Efficacy and safety of Yi Shen Fang granules in elderly people with MCI: study protocol for a multicentre, randomized, double-blind, parallel-group, controlled trial. BMC Complementary Medicine and Therapies, 23(1), 101.	Not clinical metabolic diseases

2210	Lu S, Xu X, Yuan J, et al. Efficacy of "Pinggan Formula" in Controlling Acute Type B Aortic Dissection Perioperative Blood Pressure: A Randomized Controlled Clinical Trial. Evid Based Complement Alternat Med. 2019;2019:6432953. doi:10.1155/2019/6432953	Not clinical metabolic diseases
2211	Qian, H., Xu, W., Cui, L., Wang, R., Wang, J., Tang, M., ... & Wang, L. (2021). Efficacy of Bushen Huatan Decoction combined with Baduanjin in the treatment of polycystic ovary syndrome with insulin resistance (IR-PCOS), kidney deficiency and phlegm dampness: study protocol for a randomized controlled trial. Trials, 22, 1-10.	Not clinical metabolic diseases
2212	Peng, W., Lauche, R., Ferguson, C., Frawley, J., Adams, J., & Sibbritt, D. (2017). Efficacy of Chinese herbal medicine for stroke modifiable risk factors: a systematic review. Chinese medicine, 12, 1-29.	Not clinical metabolic diseases
2213	Zhou, Y, Xia, Z, Zhang, J, Wang, Z, Liu, Y and Shang, H, 2021. Efficacy of Chinese medicine injection for treating heart failure: A network meta-analysis, Chinese Journal of Evidence-Based Medicine.	Not clinical metabolic diseases
2214	Zhou, H., Shi, H. J., Yang, J., Chen, W. G., Xia, L., Song, H. B., ... & Ma, W. (2017). Efficacy of oxymatrine for treatment and relapse suppression of severe plaque psoriasis: results from a single-blinded randomized controlled clinical trial. British Journal of Dermatology, 176(6), 1446-1455.	Not clinical metabolic diseases
2215	Tan, Y., Li, R., Zhou, P., Li, N., Xu, W., Zhou, X., ... & Yu, J. (2023). Huobahuagen tablet improves renal function in diabetic kidney disease: A real-world retrospective cohort study. Frontiers in Endocrinology, 14, 1166880.	Not clinical metabolic diseases
2216	Mody, F. V. (2014). Integrative and complementary medicine in cardiology: New hope or just “hip” and “hype”. Cardiology, 128, 94-94.	Not clinical metabolic diseases
2217	Ledda, A., Belcaro, G., Cesarone, M. R., Dugall, M., & Schönlau, F. (2010). Investigation of a complex plant extract for mild to moderate erectile dysfunction in a randomized, double-blind, placebo-controlled, parallel-arm study. BJU international, 106(7), 1030-1033.	Not clinical metabolic diseases
2218	Shen, H. S., Hsu, C. Y., Yip, H. T., & Lin, I. H. (2022). Lower risk of ischemic stroke among patients with chronic kidney disease using chinese herbal medicine as add-on therapy: A real-world nationwide cohort study. Frontiers in Pharmacology, 13, 883148.	Not clinical metabolic diseases
2219	肖宜敏.MEBO治疗慢性难愈性皮肤溃疡的临床疗效观察[J].中国烧伤创疡杂志,2015,27(06):410-414.	Not clinical metabolic diseases
2220	王安林.MEBO治疗糖尿病下肢溃疡临床分析[J].中国烧伤创疡杂志,2010,22(01):54-56.	Not clinical metabolic diseases

2221	Su, S., Duan, J., Wang, P., Liu, P., Guo, J., Shang, E., ... & Tang, Z. (2013). Metabolomic study of biochemical changes in the plasma and urine of primary dysmenorrhea patients using UPLC–MS coupled with a pattern recognition approach. Journal of proteome research, 12(2), 852-865.	Not clinical metabolic diseases
2222	Zhao, X. F., Su, S. J., GUO, Y. H., & Shu, W. (2012). Mortality and recurrence of vascular disease among stroke patients treated with combined TCM therapy. Journal of Traditional Chinese Medicine, 32(2), 173-178.	Not clinical metabolic diseases
2223	Quintans, J. S., Antoniolli, A. R., Almeida, J. R., Santana-Filho, V. J., & Quintans-Júnior, L. J. (2014). Natural products evaluated in neuropathic pain models-a systematic review. Basic & clinical pharmacology & toxicology, 114(6), 442-450.	Not clinical metabolic diseases
2224	Yao, D. N., Lu, C. J., Wen, Z. H., Yan, Y. H., Xuan, M. L., Li, X. Y., ... & Ou, A. H. (2016). Oral PSORI-CM01, a Chinese herbal formula, plus topical sequential therapy for moderate-to-severe psoriasis vulgaris: pilot study for a double-blind, randomized, placebo-controlled trial. Trials, 17, 1-10.	Not clinical metabolic diseases
2225	Shergis JL, Zhang AL, Zhou W, Xue CC. Panax ginseng in randomized controlled trials: A systematic review. Phytother Res. 2013;27(7):949-965. doi:10.1002/ptr.4847	Not clinical metabolic diseases
2226	Zhao, H., Wang, L., Zhang, L., & Zhao, H. (2023). Phytochemicals targeting lncRNAs: a novel direction for neuroprotection in neurological disorders. Biomedicine & Pharmacotherapy, 162, 114692.	Not clinical metabolic diseases
2227	Anderson JW, Allgood LD, Turner J, Oeltgen PR, Daggy BP. Psyllium decreased serum glucose and glycosylated hemoglobin significantly in diabetic outpatients. J Am Coll Nutr. 1999;18(4):283-290. doi:10.1080/07315724.1999.10718861	Not clinical metabolic diseases
2228	Song, J., Chen, X., Lyu, Y., Zhuang, W., Zhang, J., Gao, L., & Tong, X. (2019). Sanhuang Xiexin decoction promotes good functional outcome in acute ischemic stroke. Brain and Behavior, 9(1), e01185.	Not clinical metabolic diseases
2229	Li Y, Gong M, Zhang Y, et al. Shenmai Injection Improves Energy Metabolism in Patients With Heart Failure: a Randomized Controlled Trial. Evid Based Complement Alternat Med. 2019;2019:6206917. doi:10.1155/2019/6206917	Not clinical metabolic diseases
2230	Riego, M. A., Kim, P., & Salonia, J. (2022). Supraventricular Arrhythmias Induced by Aconite Poisoning. In B48. OCCUPATIONAL AND EXPOSURE-RELATED CASE REPORTS (pp. A2987-A2987). American Thoracic Society.	Not clinical metabolic diseases
2231	Luo TT, Lu Y, Yan SK, et al. Systems pharmacology analysis identifies molecular mechanisms of herbal medicines for stroke treatment and prevention. Front Pharmacol. 2019;10:746. doi:10.3389/fphar.2019.00746	Not clinical metabolic diseases

2232	Qi, R., Zhang, H., Li, D., Gao, F., Miao, Q., Chen, S., ... & Chen, Z. (2022). The Efficacy and Safety of Xinjia Xuanbai Chengqi Granules in Acute Exacerbation of COPD: A Multicentre, Randomised, Double-Blind, Controlled Trial. Evidence-Based Complementary and Alternative Medicine, 2022(1), 7366320.	Not clinical metabolic diseases
2233	Sun, K., Fu, C., Nie, S., & You, Y. (2014). The index and improvement effect of using Danhong injection to patients with atherosclerosis symptoms of coronary heart disease (CHD). Pakistan journal of pharmaceutical sciences, 27(5).	Not clinical metabolic diseases
2234	Tripathi S, Pandey S, Srivastava S, et al. Therapeutic and delivery strategies of phytoconstituents for renal fibrosis: Current status and future directions. Biomed Pharmacother. 2023;162:114585. doi:10.1016/j.biopha.2023.114585	Not clinical metabolic diseases
2235	Wang, Z., Wu, J., Li, D., Tang, X., Zhao, Y., Cai, X., ... & Huang, R. (2020). Traditional Chinese medicine Biqi capsule compared with leflunomide in combination with methotrexate in patients with rheumatoid arthritis: a randomized controlled trial. Chinese Medicine, 15, 1-15.	Not clinical metabolic diseases
2236	Wang, J., Zhao, H., Shi, K., & Wang, M. (2023). Treatment of insomnia based on the mechanism of pathophysiology by acupuncture combined with herbal medicine: a review. Medicine, 102(11), e33213.	Not clinical metabolic diseases
2237	Zhao Y, Wang L, Zhang T, et al. Visualizing Research Trends and Identifying Hotspots of Traditional Chinese Medicine (TCM) Nursing Technology for Insomnia: A 18-Years Bibliometric Analysis of Web of Science Core Collection. Front Public Health. 2022;10:926541. doi:10.3389/fpubh.2022.926541	Not clinical metabolic diseases
2238	Xu, J. H., Huang, Y. M., Ling, W., Li, Y., Wang, M., Chen, X. Y., ... & Zhao, H. L. (2015). Wen Dan Decoction for hemorrhagic stroke and ischemic stroke. Complementary Therapies in Medicine, 23(2), 298-308.	Not clinical metabolic diseases
2239	Sun J, Zhou M, Lv G, et al. Xinkeshu Improves Endothelial Function and Augments Reendothelialization Capacity in Coronary Artery Disease with Anxiety/Depression. Oxid Med Cell Longev. 2021;2021:5561272. Published 2021 Jul 18. doi:10.1155/2021/5561272	Not clinical metabolic diseases
2240	Wu, M. N., Zhou, L. J. M., & Zhou, D. M. (2022). Xiyanping injection combined with acitretin for psoriasis vulgaris: a systematic review and meta-analysis. Frontiers in Pharmacology, 13, 971715.	Not clinical metabolic diseases
2241	祁丽霞.半夏白术天麻汤临床运用验案四则[J].亚太传统医药,2019,15(03):112-113.	Not clinical metabolic diseases
2242	王庆高,潘朝锌,张振千,等.补阳还五汤对不稳定性心绞痛患者血管内皮功能的影响[J].中国社区医师,2009,25(07):42.	Not clinical metabolic diseases
2243	王甸红,王世琤,林越,等.参苓白术散对服二甲双胍后胃肠道反应的疗效观察[J].结直肠肛门外科,2015,21(S1):64-65.	Not clinical metabolic diseases

2244	孙国军.独活寄生汤加减治疗慢性风湿性关节炎 90 例[J].内蒙古中医药,2014,33(17):32.DOI:10.16040/j.cnki.cn15-1101.2014.17.026.	Not clinical metabolic diseases
2245	王改仙,周铭,高颜华.读经典治呕吐一则[J].中国中医药现代远程教育,2010,8(04):71.	Not clinical metabolic diseases
2246	王玲,刘芳,岳莹.复方黄柏液治疗糖尿病下肢慢性溃疡疗效观察[J].天津药学,2017,29(04):45-47.	Not clinical metabolic diseases
2247	吴忧,白倩,葛星峰,等.血必净注射液对感染性多器官功能障碍综合征患者肝肾功能及炎性因子的影响[J].黑龙江医学,2025,49(01):58-60.	Not clinical metabolic diseases
2248	徐莎莎,胡顺金.胡顺金分期辨治糖尿病肾脏疾病验案举隅[J].陕西中医药大学学报,2020,43(03):69-72.DOI:10.13424/j.cnki.jsctcm.2020.03.018.	Not clinical metabolic diseases
2249	张明顺.化腐生肌散治疗糖尿病足坏疽的效果分析[J].当代医药论丛,2017,15(16):22-23.	Not clinical metabolic diseases
2250	周小爱,王丽群.化湿活血汤联合甲钴胺治疗 2 型糖尿病下肢周围神经病变 52 例[J].浙江中医杂志,2016,51(04):250.DOI:10.13633/j.cnki.zjtcm.2016.04.009.	Not clinical metabolic diseases
2251	周静,孟静,王聪,等.化浊解毒加减方治疗 2 型糖尿病合并轻中度抑郁症(气郁化火证)的临床研究[J].天津中医药,2019,36(05):449-452.	Not clinical metabolic diseases
2252	王秀颖,袁晓庆,谢建军.加味八正散治疗湿热下注型糖尿病神经性膀胱的效果[J].医学信息,2020,33(02):163-164.	Not clinical metabolic diseases
2253	王秀颖,袁晓庆,谢建军.加味八正散治疗湿热下注型糖尿病神经性膀胱的效果[J].医学信息,2020,33(02):163-164.	Not clinical metabolic diseases
2254	杨娟,李雪梅.加味升降散对T2DM并血脂异常患者调脂作用临床研究[J].云南中医中药杂志,2012,33(09):12-15+1.DOI:10.16254/j.cnki.53-1120/r.2012.09.008.	Not clinical metabolic diseases
2255	袁思成,黄肖玲,华胜毅,等.健脾补气法治疗急诊及ICU低蛋白血症的真实世界研究[J].时珍国医国药,2021,32(06):1407-1412.	Not clinical metabolic diseases
2256	薛菲.健脾化湿类中药配伍治疗高脂血症的疗效及对血脂水平的影响[J].中国医药指南,2018,16(17):181.DOI:10.15912/j.cnki.gocm.2018.17.137.	Not clinical metabolic diseases
2257	任丽曼, 2018. 健脾利湿法治疗重度单纯性肥胖 1 例, 中国药业.	Not clinical metabolic diseases

2258	王全顺, 付瑞萍, 李伟, 卫华, 张甜甜, 刘洁, 2019. 健脾利湿解毒汤治疗糖尿病合并泌尿系统感染的临床效果观察, 国际医药卫生导报.	Not clinical metabolic diseases
2259	缪亚香,肖爱萍.健脾渗湿法联合激光治疗糖尿病黄斑水肿[J].山东中医杂志,2015,34(10):748-749.DOI:10.16295/j.cnki.0257-358x.2015.10.006.	Not clinical metabolic diseases
2260	杨贵亮.健脾化痰治疗高血脂症疗效观察[J].中西医结合心血管病电子杂志,2018,6(21):137-138.DOI:10.16282/j.cnki.cn11-9336/r.2018.21.104.	Not clinical metabolic diseases
2261	于学康.苓桂术甘汤治验 2 则[J].陕西中医,2006,(12):1583.	Not clinical metabolic diseases
2262	周敏华.刘良徛运用李氏清暑益气汤治疗疑难发热 1 例[J].江西中医药,2014,45(05):57-58.	Not clinical metabolic diseases
2263	杨超平.龙胆泻肝汤证治举隅[J].河北中医,2008,(03):271.	Not clinical metabolic diseases
2264	吴婉君,刘祖发.龙胆泻肝汤治疗内科杂病验案 4 则[J].中国民间疗法,2020,28(19):95-96.DOI:10.19621/j.cnki.11-3555/r.2020.1939.	Not clinical metabolic diseases
2265	石颢.清肝泄浊汤治疗高脂血症[J].医药论坛杂志,2006,(04):62-63.	Not clinical metabolic diseases
2266	魏瑞丽,谢雁鸣,王连心,等.清开灵注射液治疗缺血性脑血管病患者真实世界临床特征及联合用药分析[J].中南药学,2016,14(12):1287-1292.	Not clinical metabolic diseases
2267	田振兴,高珊,张娟.清热利湿法治疗糖尿病合并尿路感染验案一则[J].亚太传统医药,2017,13(18):112-113.	Not clinical metabolic diseases
2268	汪远娇,魏明照.社区中医护理联合常规药物干预老年高血压随机平行对照研究[J].实用中医内科杂志,2014,28(01):164-166.	Not clinical metabolic diseases
2269	许大明,皮健,王晓娟.湿润烧伤膏对糖尿病病人烧伤创面的疗效[J].中国烧伤创疡杂志,2004,(02):121-123.	Not clinical metabolic diseases
2270	谢乾.湿润烧伤膏合胰岛素外用治疗糖尿病患者烧伤 27 例体会[J].中医药导报,2008,(02):48-49.DOI:10.13862/j.cnki.cn43-1446/r.2008.02.053.	Not clinical metabolic diseases
2271	任海萍,刘洪年.湿润烧伤膏及胰岛素治疗糖尿病足 35 例[J].青岛医药卫生,2007,(02):114.	Not clinical metabolic diseases

2272	闫大志,张可勇,初惠亨.湿润烧伤膏加高压氧治疗糖尿病足溃疡 1 例[J].中国现代医药杂志,2009,11(06):113.	Not clinical metabolic diseases
2273	杨惠珍,马淑琴,吕娟琴,等.湿润烧伤膏联合电磁波治疗糖尿病患者皮肤感染的效果观察[J].护理学报,2011,18(06):60-62.DOI:10.16460/j.issn1008-9969.2011.06.021	Not clinical metabolic diseases
2274	史会林.湿润烧伤膏与创疡贴治疗糖尿病足 89 例临床体会[J].中国烧伤创疡杂志,2012,24(02):124-125.	Not clinical metabolic diseases
2275	徐刚,黄金华,刘亦峰,等.湿润烧伤膏与美宝创疡贴联合治疗糖尿病足溃疡[J].中国烧伤创疡杂志,2010,22(05):382-384.	Not clinical metabolic diseases
2276	F.Sakr M ,M.Hamed H ,陈永翀 , et al.湿润烧伤膏治疗慢性糖尿病足溃疡的多中心对照研究[J].中国烧伤创疡杂志,2012,24(02):102-118.	Not clinical metabolic diseases
2277	徐礼笑子,李民.湿润烧伤膏治疗糖尿病合并足部II度烫伤疗效观察[J].中国烧伤创疡杂志,2019,31(02):113-116.	Not clinical metabolic diseases
2278	钟晓光.湿润烧伤膏治疗糖尿病患者足部低温烫伤疗效观察[J].中国烧伤创疡杂志,2017,29(03):165-168.	Not clinical metabolic diseases
2279	王德银, 2020. 探讨痰湿体质高血压患者采用半夏白术天麻汤合温胆汤治疗的临床价值, 世界最新医学信息文摘.	Not clinical metabolic diseases
2280	王尚勇, & 周玉兰. (1997). 温病治验三则. 北京中医, 16(2), 32-33.	Not clinical metabolic diseases
2281	辛小红,姚蓝.五积散临证举隅[J].辽宁中医杂志,2014,41(10):2098-2099.DOI:10.13192/j.issn.1000-1719.2014.10.029	Not clinical metabolic diseases
2282	陈志昌,陈茵.消渴丸致低血糖性昏迷 1 例报告[J].广州医药,1987,(01):31.	Not clinical metabolic diseases
2283	赵巍, 张隆基, 2005. 新癀片治疗痛风性关节炎临床观察, 中华现代中西医杂志.	Not clinical metabolic diseases
2284	张正媚,申子龙,王梅杰,等.益气活血清利法联合西药治疗特发性膜性肾病的meta分析[J].海南医学院学报,2022,28(10):756-765.DOI:10.13210/j.cnki.jhmu.20211224.002.	Not clinical metabolic diseases
2285	彭俏颖,许进雄,卢松钊.益肾化湿颗粒联合贝那普利治疗糖尿病肾病的效果评价[J].海峡药学,2020,32(04):151-153.	Not clinical metabolic diseases

2286	邱葵,董珍宇.银杏达莫注射液致肝功能异常升高 1 例[J].解放军药学学报,2018,34(01):100.	Not clinical metabolic diseases
2287	邱葵,董珍宇.银杏达莫注射液致肝功能异常升高 1 例[J].解放军药学学报,2018,34(01):100.	Not clinical metabolic diseases
2288	周瑞群.运用经方治疗肠炎病案三则[J].中国民族民间医药,2012,21(19):94.	Not clinical metabolic diseases
2289	姚国召,黄莺,张宁.张宁教授应用四妙丸加味验案举隅[J].中医临床研究,2021,13(31):94-97.	Not clinical metabolic diseases
2290	隋杨,孔庆辉,刘春芳.针药结合治疗重度非酒精性脂肪性肝炎 1 例[J].中国现代药物应用,2015,9(03):232-233.DOI:10.14164/j.cnki.cn11-5581/r.2015.03.171.	Not clinical metabolic diseases
2291	张永红,彭敏.脂肪肝中西医结合治疗的护理体会[J].中国民间疗法,2008,(02):58-59.DOI:10.19621/j.cnki.11-3555/r.2008.02.057.	Not clinical metabolic diseases
2292	杨建兵,袁惠芬,郑雪华.中西药联合血液净化治疗顽固性心力衰竭 32 例[J].浙江中医杂志,2014,49(11):804.DOI:10.13633/j.cnki.zjtcm.2014.11.017.	Not clinical metabolic diseases
2293	张新志,邓瑞,叶文静,等.中西医结合治疗湿热下注 2 型糖尿病周围神经病变[J].甘肃医药,2014,33(04):246-248.DOI:10.15975/j.cnki.gsyy.2014.04.007.	Not clinical metabolic diseases
2294	邢伯威.中西医结合治疗糖尿病手指坏疽 1 例[J].中国中西医结合外科杂志,2014,20(06):661.	Not clinical metabolic diseases
2295	孙树君.中西医结合治疗眩晕症 144 例[J].中国医疗前沿,2011,6(10):21+4.	Not clinical metabolic diseases
2296	林振华,李杏.中西医结合治疗阵发性房颤 30 例[J].浙江中医杂志,2018,53(07):526.DOI:10.13633/j.cnki.zjtcm.2018.07.042.	Not clinical metabolic diseases
2297	詹育和,王学锋.中药对糖尿病性黄斑水肿治疗作用的观察[J].中医药临床杂志,2008,(01):45-46.DOI:10.16448/j.cjtcm.2008.01.039.	Not clinical metabolic diseases
2298	吴熹.中药泡足联合紫花烧伤膏外敷治疗糖尿病足[J].护理学杂志,2010,25(21):48-49.	Not clinical metabolic diseases
2299	舒祝明,施政,章建军,等.中医药治疗高尿酸血症临床疗效荟萃分析[J].浙江中西医结合杂志,2017,27(05):439-442.	Not clinical metabolic diseases

2300	闫丽丽,盛梅笑.中医药治疗银屑病合并肾脏损害 3 例[J].中国中医药信息杂志,2010,17(06):84-85.	Not clinical metabolic diseases
2301	王会杰.自拟经验方中药湿热敷治疗 2 型糖尿病下肢周围神经疼痛临床观察[J].光明中医,2016,31(01):77-78.	Not clinical metabolic diseases
2302	王国荣,李颖.自拟温心补肾汤治疗病窦综合征 23 例临床观察[J].中国中医药科技,2008,(04):286.	Not clinical metabolic diseases
2303	王徐龙. (2012). 综合治疗糖尿病足 15 例. 中国中医药咨讯, 4(2), 379-379.	Not clinical metabolic diseases
2304	张霜梅. (2020). 卒中后失眠的临床研究及其中医药治疗的 Meta 分析 (Doctoral dissertation, 广州中医药大学).	Not clinical metabolic diseases
2305	Wang Y, Zhang Y, Li X, et al. Integrating Chinese and Western medicines reduced the incidence of hepatocellular carcinoma in patients with diabetes mellitus: A Taiwanese population-based cohort study. J Ethnopharmacol. 2021;279:114747. doi:10.1016/j.jep.2021.114747	Not clinical metabolic diseases
2306	Garcia M, Lopez R, Hernandez J, et al. Alternative Medicine Methods Used for Weight Loss and Diabetes Control by Overweight and Obese Hispanic Immigrant Women. J Altern Complement Med. 2019;25(1):69-75. doi:10.1089/acm.2018.0421	Not clinical metabolic diseases
2307	Chen L, Xu W, Zhang Y, et al. TCM as adjunctive therapy improves risks of respiratory hospitalizations in persons with type 2 diabetes: A retrospective cohort study. Complement Ther Med. 2020;51:102377. doi:10.1016/j.ctim.2020.102377	Not clinical metabolic diseases
2308	Li J, Wang H, Liu Z, et al. Effect of jiangzhi zhongyao pian on serum lipid and antioxidation of hyperlipemic patients. Chin J Integr Med. 2018;24(10):762-768. doi:10.1007/s11655-018-2963-2	Not clinical metabolic diseases
2309	Zhang X, Liu Y, Wang Q, et al. The curative effect of rhubarb extract on severe periodontitis in patients with diabetes mellitus. J Ethnopharmacol. 2017;198:241-248. doi:10.1016/j.jep.2017.05.003	Not clinical metabolic diseases
2310	Kim S, Park J, Lee H, et al. Effect of Yanggyuksanhwa-tang on non-insulin-dependent diabetes mellitus unresponsive to oral hypoglycemic agents: A case report. Integr Med Res. 2016;5(3):202-206. doi:10.1016/j.imr.2016.04.002	Not clinical metabolic diseases
2311	Zhao Y, Li X, Wang Y, et al. Effect of Xuebijing injection on myocardium during cardiopulmonary bypass: A prospective, randomized, double blind trial. Chin J Integr Med. 2015;21(9):683-688. doi:10.1007/s11655-015-2115-1	Not clinical metabolic diseases

2312	Liu Y, Zhang H, Wang L, et al. Efficacy and safety of external application of Traditional Chinese Medicine for the treatment of acute gouty arthritis: A systematic review and Meta-analysis. J Ethnopharmacol. 2022;289:115123. doi:10.1016/j.jep.2022.115123	Not clinical metabolic diseases
2313	Huang Y, Lin H, Wang X, et al. Chinese Herbal Medicine Decreases Incidence of Cirrhosis in Patients with Non-Alcoholic Fatty Liver Disease in Taiwan: A Propensity Score-Matched Cohort Study. Front Pharmacol. 2021;12:654321. doi:10.3389/fphar.2021.654321	Not clinical metabolic diseases
2314	Chen Y, Wang Z, Liu H, et al. Increased risk of chronic kidney disease among users of non-prescribed Chinese herbal medicine in Taiwan. Nephrology (Carlton). 2019;24(7):713-721. doi:10.1111/nep.13456	Not clinical metabolic diseases
2315	Nakamura M, Nishida K, Kato M, et al. Goshajinkigan for reducing chemotherapy-induced peripheral neuropathy: Protocol for a systematic review and meta-analysis. BMJ Open. 2018;8(8):e021915. doi:10.1136/bmjopen-2018-021915	Not clinical metabolic diseases
2316	Li F, Zhang Y, Wang Y, et al. The effects of dietary supplements and natural products targeting glucose levels: An overview. Curr Diab Rep. 2020;20(12):64. doi:10.1007/s11892-020-01313-9	Not clinical metabolic diseases
2317	Wang J, Liu Y, Chen X, et al. Current Status and Evaluation of Randomized Clinical Trials of Traditional Chinese Medicine in the Treatment of Cardiovascular Diseases. Evid Based Complement Alternat Med. 2016;2016:1234567. doi:10.1155/2016/1234567	Not clinical metabolic diseases
2318	Zhang L, Li X, Wang Y, et al. Diabetic kidney disease treated with a modified Shenzhuo formula derived from Traditional Chinese Medicine: A case report. J Integr Med. 2017;15(3):229-232. doi:10.1016/S2095-4964(17)60314-6	Not clinical metabolic diseases
2319	Gang, X., Gao, T., Han, Y., Tai, Y., Zhong, C., Chen, S., ... & Liu, M. (2022). Effectiveness and safety of different academic schools of traditional Chinese medicine in the treatment of obesity: A protocol for systematic review and meta-analysis. Medicine, 101(49), e31960.	Not clinical metabolic diseases
2320	Fujimoto M, Tsuneyama K, Kinoshita H, et al. The traditional Japanese formula keishibukuryogan reduces liver injury and inflammation in patients with nonalcoholic fatty liver disease. Ann N Y Acad Sci. 2010;1190:151-158. doi:10.1111/j.1749-6632.2009.05265.x	Not clinical metabolic diseases
2321	Fu, B., Shang, Z., Song, S., Xu, Y., Wei, L., Li, G., & Yang, H. (2023). Adverse reactions of Niaoduqing granules: A systematic review and meta-analysis. Phytomedicine, 109, 154535.	Not clinical metabolic diseases
2322	Ferrara A, Hedderson MM, Zhu Y, et al. Association of SARS-CoV-2 Infection With Pregnancy Outcomes in a Large Health System in California. JAMA Intern Med. 2021;181(5):714-722. doi:10.1001/jamainternmed.2021.2326.	Not clinical metabolic diseases

2323	Chen YH, Lin CH, Lin YH, et al. Risk of developing coronary artery disease in patients with type 2 diabetes receiving traditional Chinese medicine therapy: A nationwide retrospective cohort study. J Altern Complement Med. 2015;21(2):96-102. doi:10.1089/acm.2014.0079.	Not clinical metabolic diseases
2324	du Toit A, van der Kooy F. Artemisia afra, a controversial herbal remedy or a treasure trove of new drugs? J Ethnopharmacol. 2019;244:112127. doi:10.1016/j.jep.2019.112127.	Not clinical metabolic diseases
2325	Cesarone MR, Belcaro G, Nicolaides AN, et al. Treatment of edema and increased capillary filtration in venous hypertension with total triterpenic fraction of Centella asiatica: a clinical, prospective, placebo-controlled, randomized, dose-ranging trial. Angiology. 2001;52(Suppl 2):S37-S41. doi:10.1177/000331970105202S11.	Not clinical metabolic diseases
2326	Zhang Y, Zhang L, Zhang Y, et al. Efficacy and safety of Yirui capsule in patients with hyperlipidemia: Study protocol for a multicenter, randomized, double-blind, placebo-controlled trial. Trials. 2016;17:289. doi:10.1186/s13063-016-1419-9.	Not clinical metabolic diseases
2327	Coon JT, Ernst E. Complementary and alternative therapies in the treatment of chronic hepatitis C: A systematic review. J Hepatol. 2004;40(3):491-500. doi:10.1016/j.jhep.2003.11.014.	Not clinical metabolic diseases
2328	Yeh YH, Hsieh IC, Lin YH, et al. Reduced risk of stroke in patients with cardiac arrhythmia receiving traditional Chinese medicine: A nationwide matched retrospective cohort study. Eur J Integr Med. 2016;8(1):54-59. doi:10.1016/j.eujim.2015.09.002.	Not clinical metabolic diseases
2329	Chous AP, Richer SP, Gerson JD, et al. The Diabetes Visual Function Supplement Study (DiVFuSS). Br J Ophthalmol. 2016;100(2):227-234. doi:10.1136/bjophthalmol-2014-306534.	Not clinical metabolic diseases
2330	Chiao YW, Chen YJ, Kuo YH, Lu CY. Traditional Chinese Medical Care and Incidence of Stroke in Elderly Patients Treated with Antidiabetic Medications. Int J Environ Res Public Health. 2018;15(6):1267. doi:10.3390/ijerph15061267.	Not clinical metabolic diseases
2331	Chen, Q., Huang, W., & Tan, C. (2021). RESEARCH ON THE TREATMENT OF DIABETIC NEPHROPATHY WITH MEDICINE COMBINATION. ACTA MEDICA MEDITERRANEA, 37(4), 2251-2254.	Not clinical metabolic diseases
2332	Cha J, Lee J, Lee YJ, et al. Weight Control Registry Using Korean Medicine: A Protocol for a Prospective Registry Study. Int J Environ Res Public Health. 2022;19(21):13903. doi:10.3390/ijerph192113903.	Not clinical metabolic diseases
2333	Belcaro G, Cesarone MR, Errichi BM, et al. Evaluation of treatment of diabetic microangiopathy with total triterpenic fraction of Centella asiatica: a clinical prospective randomized trial with a microcirculatory model. Angiology. 2001;52(Suppl 2):S45-S48. doi:10.1177/000331970105202S10.	Not clinical metabolic diseases

2334	Belcaro G, Cesarone MR, Errichi BM, et al. Microcirculatory effects of total triterpenic fraction of Centella asiatica in chronic venous hypertension: measurement by laser Doppler, TcPO2-CO2, and leg volumetry. Angiology. 2001;52(Suppl 2):S49-S52. doi:10.1177/000331970105202S09.	Not clinical metabolic diseases
2335	Belcaro G, Cesarone MR, Errichi BM, et al. Increase in echogenicity of echolucent carotid plaques after treatment with total triterpenic fraction of Centella asiatica: a prospective, placebo-controlled, randomized trial. Angiology. 2001;52(Suppl 2):S53-S56. doi:10.1177/000331970105202S08.	Not clinical metabolic diseases
2336	Belcaro G, Cesarone MR, Errichi BM, et al. Effects of the total triterpenic fraction of Centella asiatica in venous hypertensive microangiopathy: a prospective, placebo-controlled, randomized trial. Angiology. 2001;52(Suppl 2):S57-S60. doi:10.1177/000331970105202S07.	Not clinical metabolic diseases
2337	Cao H, Ren M, Guo L, et al. JinQi-Jiangtang tablet, a Chinese patent medicine, for pre-diabetes: a randomized controlled trial. Trials. 2010;11:27. doi:10.1186/1745-6215-11-27.	Not clinical metabolic diseases
2338	Lin H. Clinical effect of the principle of tonifying-kidney, invigorating-spleen, nourishing and activating-blood on patients with vascular dementia. Int J Clin Exp Med. 2018;11(4):3816-3822.	Not clinical metabolic diseases
2339	Bega D. Complementary and Integrative Interventions for Chronic Neurologic Conditions Encountered in the Primary Care Office. Prim Care. 2017;44(2):305-322. doi:10.1016/j.pop.2017.02.004.	Not clinical metabolic diseases
2340	Banerjee A, Sriramulu S, Catanzaro R, et al. Natural Compounds as Integrative Therapy for Liver Protection against Inflammatory and Carcinogenic Mechanisms: From Induction to Molecular Biology Advancement. Curr Mol Med. 2022;22(3):216-231. doi:10.2174/1566524022666220316102310.	Not clinical metabolic diseases
2341	Azushima K, Tamura K, Haku S, et al. Effects of the oriental herbal medicine Bofu-tsusho-san in obesity hypertension: a multicenter, randomized, parallel-group controlled trial. Atherosclerosis. 2015;240(1):297-304. doi:10.1016/j.atherosclerosis.2015.01.025.	Not clinical metabolic diseases
2342	Khosravi-Boroujeni H, Nikbakht-Jam I, Natanelov E, et al. Effect of cinnamon, cardamom, saffron and ginger consumption on blood pressure and a marker of endothelial function in patients with type 2 diabetes mellitus: A randomized controlled clinical trial. J Renin Angiotensin Aldosterone Syst. 2015;16(3):567-572. doi:10.1177/1470320314563422.	Not clinical metabolic diseases
2343	Jing A, Li-Mei Z, Yan-Jie L, et al. A randomized, multicentre, open-label, parallel-group trial to compare the efficacy and safety profile of Daming capsule in patients with hypercholesterolemia. Phytother Res. 2009;23(7):1039-1042. doi:10.1002/ptr.2654.	Not clinical metabolic diseases
2344	邹红,任建萍,缪晚虹.行气活血健脾利水方联合雷珠单抗治疗糖尿病性黄斑水肿的临床研究[J].中国中医眼科杂志,2016,26(02):71-74.DOI:10.13444/j.cnki.zgzyykzz.2016.02.001.	Not clinical metabolic diseases

2345	朱晓燕.睡眠呼吸暂停综合征的辨证与治疗对策探究[J].世界睡眠医学杂志,2019,6(06):742-743.	Not clinical metabolic diseases
2346	朱晓冬.经方验案 2 则[J].光明中医,2013,28(03):594.	Not clinical metabolic diseases
2347	朱杰,郝有孝.化湿通络方治疗代谢综合征 35 例[J].陕西中医,2010,31(12):1607-1608.	Not clinical metabolic diseases
2348	茅立明.中医辨证治疗原发性高血压 181 例观察[J].中国乡村医药,2014,21(01):29-30.DOI:10.19542/j.cnki.1006-5180.2014.01.017.	Not clinical metabolic diseases
2349	马超,金明.AMD与全身性疾病及其它眼底疾病的相关因素分析[J].中国中医眼科杂志,2014,24(04):266-270.DOI:10.13444/j.cnki.zgzyykzz.003311.	Not clinical metabolic diseases
2350	吕锦涛,张冰,林志健,等.基于文献计量和知识图谱分析中西药合用的国内研究进展[J].中国医院药学杂志,2020,40(21):2269-2275.DOI:10.13286/j.1001-5213.2020.21.12.	Not clinical metabolic diseases
2351	陆源源.中西医结合治疗糖尿病合并慢性湿疹 35 例[J].河北中医,2010,32(11):1677-1678.	Not clinical metabolic diseases
2352	陆西宛,陆曙.陆曙教授治疗代谢综合征验案 2 则[J].现代中医药,2011,31(06):1-2.DOI:10.13424/j.cnki.mtcm.2011.06.002.	Not clinical metabolic diseases
2353	陆磊,韦莉莉,冯琴梅,等.拔毒生肌膏配合食品包装保鲜膜治疗褥疮等皮肤溃疡 24 例[J].现代中西医结合杂志,2008,(14):2175-2176.	Not clinical metabolic diseases
2354	张素云.健脾化湿类中药配伍治疗高脂血症的疗效观察[J].基层医学论坛,2014,18(29):3978-3979.	Not clinical metabolic diseases
2355	卢亚萍,彭素娥.苓桂术甘汤临证新用三则[J].实用中西医结合临床,2012,12(05):75-76.	Not clinical metabolic diseases
2356	程红梅,李素娟.参附注射液致过敏性休克 1 例[J].药物流行病学杂志,2013,22(10):575-576.DOI:10.19960/j.cnki.issn1005-0698.2013.10.021.	Not clinical metabolic diseases
2357	刘愚.化浊降脂汤治疗痰浊中阻型高脂血症 23 例临床研究[J].内蒙古中医药,2013,32(20):9.DOI:10.16040/j.cnki.cn15-1101.2013.20.022.	Not clinical metabolic diseases
2358	刘英猛.雷根平用大剂量黄芪治疗糖尿病肾病验案 1 则[J].湖南中医杂志,2016,32(05):131-132.DOI:10.16808/j.cnki.issn1003-7705.2016.05.068.	Not clinical metabolic diseases

2359	刘延陵,闵娜,马拴全.中医内外并治下肢溃疡 34 例[J].现代中西医结合杂志,2007,(32):4808.	Not clinical metabolic diseases
2360	刘桐伊,吴凡,田春雨,等.王淑玲治疗 2 型糖尿病验案 1 则[J].临床合理用药杂志,2019,12(25):168-169.DOI:10.15887/j.cnki.13-1389/r.2019.25.102.	Not clinical metabolic diseases
2361	李艳彬.探讨益气利湿降浊汤联合中药灌肠治疗早期糖尿病肾病临床效果[J].世界最新医学信息文摘,2018,18(91):143-144.DOI:10.19613/j.cnki.1671-3141.2018.91.120.	Not clinical metabolic diseases
2362	刘冬梅.原发性高血压中西医结合治疗临床观察[J].深圳中西医结合杂志,2016,26(20):55-56.DOI:10.16458/j.cnki.1007-0893.2016.20.027.	Not clinical metabolic diseases
2363	刘柳洪,张勇,李际涛.湿润烧伤膏联合蚕食清创治疗Wagner 2～4 级缺血性糖尿病足疗效分析[J].中国烧伤创疡杂志,2019,31(05):320-324.	Not clinical metabolic diseases
2364	刘繁荣,郭新建,李君平.降脂理肝汤治疗非酒精性脂肪性肝炎 34 例[J].陕西中医,2015,36(01):8-9.	Not clinical metabolic diseases
2365	刘朝华.从肝脾论治高血压病眩晕验案 5 则[J].广西中医药,2011,34(06):36-37.	Not clinical metabolic diseases
2366	刘波,陈晓奇.加减柴平汤临证新用举隅[J].上海中医药杂志,2011,45(10):66-67.DOI:10.16305/j.1007-1334.2011.10.025.	Not clinical metabolic diseases
2367	凌云,崔德强.大柴胡汤加味治疗代谢综合征验案 2 则[J].中国中医药现代远程教育,2017,15(24):140-142.	Not clinical metabolic diseases
2368	林丽萍, & 涂玉宏. (2011). 湿润烧伤膏治疗糖尿病足的临床体会. 中国烧伤创疡杂志, 23(1), 89-90.	Not clinical metabolic diseases
2369	林立业,张俊明,汲泓.痛风性关节炎合并高血压病用药规律研究[J].中医临床研究,2023,15(04):49-54.	Not clinical metabolic diseases
2370	林进生.金糖宁胶囊治疗 2 型糖尿病III期临床试验观察[J].海峡药学,2008,(08):106-109.	Not clinical metabolic diseases
2371	林海勇,刘珊,李乃民.除痹利湿活络饮治疗糖尿病足 34 例[J].临床荟萃,2008,23(23):1721-1722.	Not clinical metabolic diseases
2372	林达秋.补气行气活血利湿法治疗单纯性肥胖 25 例临床疗效观察[J].深圳中西医结合杂志,2017,27(11):41-42.DOI:10.16458/j.cnki.1007-0893.2017.11.018.	Not clinical metabolic diseases

2373	梁群, 张时浩, 谢小玉, & 朱嘉敏. (2021). 真武汤合五苓散加减联合西医常规方法治疗 1 例梗阻性肾病合并心力衰竭患者报告. 中国中西医结合急救杂志, 28(5), 621-623.	Not clinical metabolic diseases
2374	廉洁.桂枝芍药知母汤治疗糖尿病合并干燥综合征 1 则[J].河南中医,2014,34(10):1884-1885.DOI:10.16367/j.issn.1003-5028.2014.10.015.	Not clinical metabolic diseases
2375	李志峰,李焕才,平慧,等.湿润烧伤膏治疗咽瘘 24 例[J].武警医学院学报,2008,(05):451-452.	Not clinical metabolic diseases
2376	王晓璐, 曲世超, 石礼静,等.中西医结合治疗糖尿病周围神经病变临床观察[J].国际临床医学,2024,6(4):	Not clinical metabolic diseases
2377	李艳秋.活血通络除湿法治疗高脂血症 102 例[J].河北中医,2005,(06):430.	Not clinical metabolic diseases
2378	李艳芬,赵亚楠,王瑞华,等.湿浊相关糖尿病合并症辨治举隅[J].国医论坛,2019,34(04):59-61.DOI:10.13913/j.cnki.41-1110/r.2019.04.027.	Not clinical metabolic diseases
2379	李逊.俞氏养生御病方治疗小儿肥胖症验案 1 则[J].中国民间疗法,2020,28(17):95-96.DOI:10.19621/j.cnki.11-3555/r.2020.1745.	Not clinical metabolic diseases
2380	李逊.俞氏养生御病方治疗小儿肥胖症验案 1 则[J].中国民间疗法,2020,28(17):95-96.DOI:10.19621/j.cnki.11-3555/r.2020.1745.	Not clinical metabolic diseases
2381	李晓佳,王瑜,王小莹.中医治疗眩晕验案 1 则[J].长春中医药大学学报,2011,27(04):627-628.DOI:10.13463/j.cnki.cczyy.2011.04.067.	Not clinical metabolic diseases
2382	李晓辉,梁苹茂.梁苹茂从湿论治糖尿病验案举隅[J].山西中医,2017,33(04):45.	Not clinical metabolic diseases
2383	李爽.通脉利湿汤治疗糖尿病下肢血管病变导致水肿 1 例[J].河南中医,2013,33(07):1168-1169.DOI:10.16367/j.issn.1003-5028.2013.07.071.	Not clinical metabolic diseases
2384	李舒彬,梁苹茂.梁苹茂治疗糖尿病并发症验案 3 则[J].湖南中医杂志,2015,31(08):100-101.DOI:10.16808/j.cnki.issn1003-7705.2015.08.053.	Not clinical metabolic diseases
2385	李如英,陆燕.王顺贤诊治疑难杂症验案 3 则[J].江苏中医药,2012,44(10):53-54.	Not clinical metabolic diseases
2386	李敏州.糖尿病肾病合并肾性贫血的中医药治疗[J].中国中西医结合肾病杂志,2017,18(02):187-188.	Not clinical metabolic diseases

2387	李丽花.庞国明教授运用攻下法治疗消渴病验案 4 则[J].中医研究,2013,26(03):49-51.	Not clinical metabolic diseases
2388	李景华.中医为主治疗糖尿病并发周围神经炎 33 例[J].吉林中医药,2007,(11):21.DOI:10.13463/j.cnki.jlzyy.2007.11.011.	Not clinical metabolic diseases
2389	李进,史载祥.风湿性心脏病房颤转复为窦性心律 1 例报告[J].中国中西医结合杂志,2020,40(04):502-503.	Not clinical metabolic diseases
2390	李杰,黄淑田.血脂康致高龄病人横纹肌溶解症 1 例[J].中西医结合心脑血管病杂志,2017,15(07):890-891.	Not clinical metabolic diseases
2391	高志赟.湿润烧伤膏治疗糖尿病足临床疗效观察[J].中国烧伤创疡杂志,2018,30(03):157-161.	Not clinical metabolic diseases
2392	李红梅.湿润烧伤膏配合中医辨证治疗糖尿病足溃疡临床观察[J].深圳中西医结合杂志,2018,28(18):41-43.DOI:10.16458/j.cnki.1007-0893.2018.18.020.	Not clinical metabolic diseases
2393	李红.足三里穴位注射配合祛湿活血通络汤对改善糖尿病引起周围神经病变的疗效观察[J].海峡药学,2017,29(09):106-107.	Not clinical metabolic diseases
2394	范强,薛燕芳,王涛涛,等.靳锋主任医师治疗糖尿病肾病常用药对举隅[J].中医研究,2017,30(12):47-50.	Not clinical metabolic diseases
2395	李海松,梁苹茂.梁苹茂运用中药降糖验案 3 则[J].上海中医药杂志,2011,45(08):57-58.DOI:10.16305/j.1007-1334.2011.08.026.	Not clinical metabolic diseases
2396	李福,王兴,张艳珠,等.经方临证验案三则[J].浙江中医杂志,2021,56(05):384.DOI:10.13633/j.cnki.zjtcm.2021.05.054.	Not clinical metabolic diseases
2397	李春桂,曹柏龙,苗桂珍,等.健脾祛湿化痰降浊方为主治疗肥胖型糖尿病前期的临床观察[J].陕西中医,2016,37(08):1021-1022.	Not clinical metabolic diseases
2398	黎鹏程,卢丽丽.程丑夫教授从瘀论治疑难病验案 3 则[J].中医药导报,2015,21(19):79-81.DOI:10.13862/j.cnki.cn43-1446/r.2015.19.031.	Not clinical metabolic diseases
2399	黎鹏程,卢丽丽.程丑夫教授从痰论治疑难病验案三则[J].湖南中医药大学学报,2014,34(09):43-45.	Not clinical metabolic diseases
2400	郎睿,王新慧,张涛,等.健脾祛湿和络方治疗特发性膜性肾病的队列研究[J].空军医学杂志,2021,37(06):510-514.	Not clinical metabolic diseases

2401	荆志斌, 王永刚, 徐全壹, & 马维骐. (2008). 中医治疗 1 例顽固性糖尿病合并湿疹的经验. 中国临床医药研究杂志, (022), 46-47.	Not clinical metabolic diseases
2402	金小琴.袁占盈教授治疗糖尿病合并自汗盗汗验案 2 则[J].光明中医,2017,32(20):2922-2923.	Not clinical metabolic diseases
2403	姜国贤,喻国华,余传友.调肝降脂法治疗非酒精性脂肪肝 32 例[J].陕西中医,2010,31(05):528-530.	Not clinical metabolic diseases
2404	贾晓颖,李逸潇,刘琴,等.从“湿”论治糖尿病周围神经病变 1 例[J].中国医药导报,2019,16(35):116-118+130.	Not clinical metabolic diseases
2405	贾晓颖,李逸潇,刘琴,等.从“湿”论治糖尿病周围神经病变 1 例[J].中国医药导报,2019,16(35):116-118+130.	Not clinical metabolic diseases
2406	贾海涛. (2010). 中西医结合治疗糖尿病合并褥疮感染的 1 例分析. 中医临床研究, 2(9), 69-69.	Not clinical metabolic diseases
2407	黄小清,陈小莉.湿润烧伤膏外敷加红外线照射治疗老年糖尿病褥疮[J].现代医药卫生,2006,(14):2141.	Not clinical metabolic diseases
2408	黄德慧,凌军.黄芪桂枝五物汤验案举隅[J].浙江中医杂志,2014,49(10):768.DOI:10.13633/j.cnki.zjtcm.2014.10.099.	Not clinical metabolic diseases
2409	黄达,郑亚琳,李光善,等.中西医结合治疗Felty综合征合并甲状腺功能亢进、糖尿病 1 例[J].中医杂志,2014,55(02):179-180.DOI:10.13288/j.11-2166/r.2014.02.026.	Not clinical metabolic diseases
2410	华旭霞.中西医结合联合食疗辨治糖尿病合并泌尿系感染的临床研究[J].辽宁中医杂志,2015,42(04):799-800.DOI:10.13192/j.issn.1000-1719.2015.04.054.	Not clinical metabolic diseases
2411	华琼,任永朋,刘彦妍.李培旭教授辨治糖尿病肾病临证经验与验案举隅[J].中医研究,2018,31(06):48-50.	Not clinical metabolic diseases
2412	胡智芬.五苓散加味联合苯磺酸氨氯地平治疗慢性肾炎高血压下肢水肿医案一则[J].实用中医内科杂志,2012,26(10):65-66.	Not clinical metabolic diseases
2413	胡智芬.五苓散加味联合苯磺酸氨氯地平治疗慢性肾炎高血压下肢水肿医案一则[J].实用中医内科杂志,2012,26(10):65-66.	Not clinical metabolic diseases
2414	胡智芬.五苓散加味联合苯磺酸氨氯地平治疗慢性肾炎高血压下肢水肿医案一则[J].实用中医内科杂志,2012,26(10):65-66.	Not clinical metabolic diseases

2415	贺俭.补肾化湿汤治疗高脂血症 64 例[J].山东中医杂志,2003,(08):462-463.DOI:10.16295/j.cnki.0257-358x.2003.08.008.	Not clinical metabolic diseases
2416	韩芸,黄俭仪.温胆汤临床应用举隅[J].名医,2021,(22):30-31.	Not clinical metabolic diseases
2417	何成莲.标本兼顾治疗糖尿病肾病验案一则[J].成都中医药大学学报,2008,(02):20.	Not clinical metabolic diseases
2418	郝秀英.内科疾病临床不合理用药情况分析[J].临床合理用药杂志,2018,11(36):39-40.DOI:10.15887/j.cnki.13-1389/r.2018.36.019.	Not clinical metabolic diseases
2419	韩馨悦,刘健,孙艳秋,等.痛风性关节炎患者脂代谢变化及中医药干预数据挖掘研究[J].江西中医药大学学报,2021,33(02):33-37.	Not clinical metabolic diseases
2420	郭良清,文世芳.当归六黄汤加减治疗糖尿病并反应性穿通性胶原病 1 例[J].山东中医杂志,2014,33(03):235.DOI:10.16295/j.cnki.0257-358x.2014.03.027.	Not clinical metabolic diseases
2421	郭锋斌.瘦身饮治疗单纯性肥胖病 42 例[J].陕西中医,2010,31(07):857-858.	Not clinical metabolic diseases
2422	管子函.中医药治疗糖尿病坏疽 1 例[J].河南中医,2012,32(11):1547-1548.DOI:10.16367/j.issn.1003-5028.2012.11.068.	Not clinical metabolic diseases
2423	关婷婷,杨晓晖.从"顽痰"辨治糖尿病腹泻验案一则[J].环球中医药,2021,14(09):1691-1693.	Not clinical metabolic diseases
2424	顾艳芳,李俊毅,冯慧静.自拟降脂汤治疗高脂血症 52 例[J].中国民间疗法,2009,17(04):28.DOI:10.19621/j.cnki.11-3555/r.2009.04.027.	Not clinical metabolic diseases
2425	顾培芳,褚小燕,步丽佳.祛瘀燥湿方足浴联合中药口服干预早期糖尿病足 30 例[J].浙江中医杂志,2018,53(08):582.DOI:10.13633/j.cnki.zjtcm.2018.08.019.	Not clinical metabolic diseases
2426	施暕.中成药治疗高血压病用药探析[J].湖南中医药大学学报,2013,33(12):25-26.	Not clinical metabolic diseases
2427	高志豪.温胆汤治疗高血压病 32 例体会[J].中国社区医师(医学专业),2012,14(28):185.	Not clinical metabolic diseases
2428	高巍.新诊断 2 型糖尿病中医分型护理疗效观察[J].辽宁中医杂志,2010,37(12):2442-2443.DOI:10.13192/j.ljtcm.2010.12.175.gaow.011.	Not clinical metabolic diseases

2429	高舒春.从痰湿论治消渴病 85 例[J].中国中医药现代远程教育,2013,11(14):15.	Not clinical metabolic diseases
2430	高惠玲.祛湿法在儿科杂病中的运用举隅[J].中国中医药信息杂志,2009,16(12):84-85.	Not clinical metabolic diseases
2431	冯宇,李军祥.肝脂消对非酒精性脂肪肝患者降脂作用的研究[J].中西医结合肝病杂志,2005,(03):134-135+138.	Not clinical metabolic diseases
2432	冯宇,李军祥.肝脂消治疗非酒精性脂肪肝临床疗效观察[J].中国中医药信息杂志,2005,(02):16-18.	Not clinical metabolic diseases
2433	范志刚,贾高锁,梁培福,等.柴苓降脂颗粒治疗脂肪肝合并高脂血症 104 例临床观察[J].中国医院药学杂志,2014,34(07):558-561.DOI:10.13286/j.cnki.chinhosppharmacyj.2014.07.16.	Not clinical metabolic diseases
2434	范志刚,范志强,贾高锁,等.柴苓降脂颗粒与柴苓降脂汤治疗脂肪肝痰湿瘀阻证的临床疗效[J].江苏医药,2014,40(12):1411-1414.DOI:10.19460/j.cnki.0253-3685.2014.12.017.	Not clinical metabolic diseases
2435	段阳泉.二陈汤加味治疗肥胖验案 2 则[J].江苏中医药,2009,41(02):41-42.	Not clinical metabolic diseases
2436	丁祎.湿润烧伤膏辅助治疗糖尿病足溃疡22例临床分析[J].南通大学学报(医学版),2012,32(04):322-323.	Not clinical metabolic diseases
2437	丁明华,李贺海,邱红刚,等.原位再生复原技术与负压引流治疗糖尿病溃疡疗效分析[J].中国烧伤创疡杂志,2012,24(02):119-123.	Not clinical metabolic diseases
2438	刁爱玲.湿润烧伤膏治疗慢性皮肤溃疡 53 例临床观察[J].中国当代医药,2010,17(09):46+49.	Not clinical metabolic diseases
2439	邓元龙.半夏白术天麻汤治疗顽症举隅[J].中医研究,2012,25(05):49-51.	Not clinical metabolic diseases
2440	邓德强,谷培恒.健脾化湿法治疗 2 型糖尿病 33 例[J].陕西中医,2004,(06):484-485.	Not clinical metabolic diseases
2441	单留峰,郭丽芳.解郁化浊汤治疗 2 型糖尿病前期 25 例的疗效观察[J].中国实用医药,2016,11(12):197-198.DOI:10.14163/j.cnki.11-5547/r.2016.12.145.	Not clinical metabolic diseases
2442	崔爽. (2013). 辨证治疗糖尿病合并高血压 50 例. 中国中医药现代远程教育, 11(22), 47-48.	Not clinical metabolic diseases

2443	崔莉芳,庆慧,李志毅.邱保国研究员辨证论治不同时期高血压病验案举隅[J].中医研究,2013,26(11):42-44.	Not clinical metabolic diseases
2444	崔吉英.运用燥湿健脾法治疗糖尿病验案 2 则[J].辽宁中医杂志,2003,(06):504.DOI:10.13192/j.ljtcm.2003.06.80.cuijy.064.	Not clinical metabolic diseases
2445	崔吉英.运用燥湿健脾法治疗糖尿病验案 2 则[J].辽宁中医杂志,2003,(06):504.DOI:10.13192/j.ljtcm.2003.06.80.cuijy.064.	Not clinical metabolic diseases
2446	崔惠兰,玄雄.复方甘草酸苷引起血压升高 1 例药物分析[J].中国民康医学,2013,25(19):126-127.	Not clinical metabolic diseases
2447	程婷,简小兵.简小兵论治糖尿病肾病验案举隅[J].山东中医药大学学报,2018,42(06):529-531.DOI:10.16294/j.cnki.1007-659x.2018.06.015.	Not clinical metabolic diseases
2448	程少民.刘效家教授诊治RS3PE综合征 2 例[J].风湿病与关节炎,2017,6(01):46-48+55.	Not clinical metabolic diseases
2449	程秋霞,王梅,翟新梅.湿润烧伤膏联合莫匹罗星治疗糖尿病足 23 例效果研究[J].山西医药杂志,2015,44(11):1311-1313.	Not clinical metabolic diseases
2450	程鹏,何仁亮,向红辉.MEBO联合微粒皮种植术治愈 1 例糖尿病足截肢后残端创面[J].中国烧伤创疡杂志,2013,25(02):118-121.	Not clinical metabolic diseases
2451	陈仲英.自拟化痰活血汤治疗高脂血症 56 例报道[J].甘肃中医,2004,(12):21-22.	Not clinical metabolic diseases
2452	陈铮,邓红姝,凌韶军.Wagner 3～4 级糖尿病足保足治疗体会[J].中国烧伤创疡杂志,2018,30(04):241-245.	Not clinical metabolic diseases
2453	陈泽冰,周晖,莫伟,等.三仁汤治疗糖尿病及其并发症的临床应用举隅[J].环球中医药,2018,11(08):1291-1293.	Not clinical metabolic diseases
2454	吴钧俊.加味甘露消毒丹治疗湿热内蕴型初诊 2 型糖尿病临床研究[J].吉林中医药,2009,29(11):959-960.DOI:10.13463/j.cnki.jlzyy.2009.11.015.	Not clinical metabolic diseases
2455	陈燕斌,许钻杰.辨证施治膝骨关节炎合并糖尿病的疗效及中医体质特征研究[J].内蒙古中医药,2017,36(Z2):33-34.DOI:10.16040/j.cnki.cn15-1101.2017.z2.026.	Not clinical metabolic diseases
2456	陈燕斌,许钻杰.辨证施治膝骨关节炎合并糖尿病的疗效及中医体质特征研究[J].内蒙古中医药,2017,36(Z2):33-34.DOI:10.16040/j.cnki.cn15-1101.2017.z2.026.	Not clinical metabolic diseases

2457	陈宁,陈永翀,袁媛,等.烧伤创疡再生医学与疗法治疗糖尿病皮肤溃疡的护理体会[J].中国烧伤创疡杂志,2015,27(03):192-196.	Not clinical metabolic diseases
2458	陈南官, & 周智文. (2009). 细辛治疗心血管病临证应用举隅. 实用中医内科杂志, 23(6), 51-52.	Not clinical metabolic diseases
2459	陈丽英,张曼韵,钱立明.疏肝利湿降脂方对脂肪肝患者血生化指标的影响[J].辽宁中医杂志,2003,(11):935-936.DOI:10.13192/j.ljtcm.2003.11.72.chenly.050.	Not clinical metabolic diseases
2460	陈宏灿,庞国明.庞国明教授从湿热论治 2 型糖尿病验案举隅[J].中国民族民间医药,2019,28(16):78-80.	Not clinical metabolic diseases
2461	陈桂香.稳心颗粒联合胺碘酮治疗风心病心房颤动临床观察[J].中国中医急症,2012,21(06):977-978.	Not clinical metabolic diseases
2462	沈志新,高恩芳,翁文庆.活血利水方联合曲安奈德后Tenon囊下注射治疗糖尿病性黄斑水肿 31 例[J].浙江中医杂志,2015,50(09):681-682.DOI:10.13633/j.cnki.zjtcm.2015.09.048.	Not clinical metabolic diseases
2463	常文华,陆斌.腰痛宁胶囊致血压升高 4 例[J].新疆中医药,2008,(04):35-36.	Not clinical metabolic diseases
2464	曾亭亭,刘道利,周伟青,等.流行性出血热合并 2 型糖尿病 1 例[J].实用检验医师杂志,2021,13(02):117-119.	Not clinical metabolic diseases
2465	曾玲玉,胡陵静,李航.胡陵静主任少阳论治甲状腺癌术后高血压验案举隅[J].中国民族民间医药,2021,30(05):113-114.	Not clinical metabolic diseases
2466	曹爱梅.和解法治疗糖尿病性顽固性腹泻中医辨治举隅[J].中国医药指南,2014,12(13):312.DOI:10.15912/j.cnki.gocm.2014.13.025.	Not clinical metabolic diseases
2467	蔡雯婷,车树强.补肾活血利湿降浊法治疗高尿酸血症验案举隅[J].实用中医药杂志,2016,32(03):270-271.	Not clinical metabolic diseases
2468	卜平元,罗成群,阳萍,等.1 例高龄糖尿病足患者全程治疗及护理体会[J].中国烧伤创疡杂志,2021,33(02):106-108.	Not clinical metabolic diseases
2469	卞红霞.跖疣性溃疡合并糖尿病 1 例[J].中国实用医药,2013,8(19):208-209.DOI:10.14163/j.cnki.11-5547/r.2013.19.101.	Not clinical metabolic diseases
2470	周丽萍.自拟化湿活血汤联合甲钴胺治疗 2 型糖尿病下肢周围神经病变临床疗效分析[J].双足与保健,2018,27(18):117-118.DOI:10.19589/j.cnki.issn1004-6569.2018.18.117.	Not clinical metabolic diseases

2471	艾莉蓉.中药湿热敷结合红外线照射治疗胰岛素注射所致皮下硬结的疗效及护理[J].中医药导报,2013,19(04):115-116.DOI:10.13862/j.cnki.cn43-1446/r.2013.04.058.	Not clinical metabolic diseases
2472	An, W., Huang, Y., Chen, S., Teng, T., Liu, J., & Xu, Y. (2021). Efficacy and safety of Huangkui capsule for diabetic nephropathy: a protocol for systematic review and meta-analysis. Medicine, 100(42), e27569.	Not clinical metabolic diseases
2473	Rahman MM, Islam MR, Islam MA, et al. Diabetic neuropathy: An insight on the transition from synthetic drugs to herbal therapies. Phytomedicine. 2020;76:153278. doi:10.1016/j.phymed.2020.153278	Not clinical metabolic diseases
2474	Cao, M. Z., Wei, C. H., Wen, M. C., Song, Y., Srivastava, K., Yang, N., ... & Li, X. M. (2023). Clinical efficacy of weight loss herbal intervention therapy and lifestyle modifications on obesity and its association with distinct gut microbiome: A randomized double-blind phase 2 study. Frontiers in Endocrinology, 14, 1054674.	Not clinical metabolic diseases
2475	Su X, Zhang Y, Yan L, et al. Semi-individualised Chinese medicine treatment as an adjuvant management for diabetic nephropathy: A pilot add-on, randomised, controlled, multicentre, open-label pragmatic clinical trial. BMJ Open. 2019;9(11):e031094. doi:10.1136/bmjopen-2019-031094	Not clinical metabolic diseases
2476	Hung YJ, Fang WH, Chang CM, et al. Outcomes after stroke in patients receiving adjuvant therapy with traditional Chinese medicine: A nationwide matched interventional cohort study. BMC Complement Med Ther. 2021;21(1):180. doi:10.1186/s12906-021-03318-x	Not clinical metabolic diseases
2477	Liao Z, Zhao Z, Wu X, et al. Is oral administration of Chinese herbal medicine effective and safe as an adjunctive therapy for managing diabetic foot ulcers? A systematic review and meta-analysis. Phytomedicine. 2021;92:153727. doi:10.1016/j.phymed.2021.153727	Not clinical metabolic diseases
2478	Shuo, C., Jianwei, M., Limei, X., Tianhui, N., Jing, D., Wenjun, L., & Qi, H. (2017). Safety and effectiveness of Traditional Chinese Medicinal herbs for diabetic foot: a systematic review and Meta-analysis. Journal of Traditional Chinese Medicine, 37(6), 735-745.	Not clinical metabolic diseases
2479	Xiao X, Liu X, He M, et al. Topical herbal medicine for treatment of diabetic peripheral neuropathy: A systematic review of randomized controlled trials. Complement Ther Med. 2018;41:107-117. doi:10.1016/j.ctim.2018.09.010	Not clinical metabolic diseases
2480	Guohua, D., Wulin, G., Dongxue, B., Chunhua, L., Yuhan, L., Ning, W., & Chen, Z. (2018). Efficacy of Traditional Chinese Medicine in patients with acute myocardial infarction suffering from diabetes mellitus. Journal of Traditional Chinese Medicine, 38(3), 412-418.	Not clinical metabolic diseases
2481	Liu Y, Wang Y, Xie Y, et al. Chinese herbal medicine for hyperlipidaemia: A review based on data mining from 1990 to 2016. Complement Ther Med. 2019;42:116-123. doi:10.1016/j.ctim.2018.10.018	Not clinical metabolic diseases

248<br>2	Dou, Z., Xia, Y., Zhang, J., Li, Y., Zhang, Y., Zhao, L., ... & Liu, Y. (2021). Syndrome differentiation and treatment regularity in traditional Chinese medicine for type 2 diabetes: a text mining analysis. Frontiers in endocrinology, 12, 728032.	Not clinical metabolic diseases
248<br>3	Shahmohammadi A, Movahedian A, Ghaffari S. The efficacy of Rheum ribes L. stalk extract on lipid profile in hypercholesterolemic type II diabetic patients: A randomized, double-blind, placebo-controlled, clinical trial. Iran J Pharm Res. 2015;14(4):1235-1241.	Not clinical metabolic diseases
248<br>4	Forte, R., Cennamo, G., Bonavolonta, P., Pascotto, A., de Crecchio, G., & Cennamo, G. (2013). Long-term follow-up of oral administration of flavonoids, Centella asiatica and Melilotus, for diabetic cystoid macular edema without macular thickening. Journal of ocular pharmacology and therapeutics, 29(8), 733-737.	Not clinical metabolic diseases
248<br>5	FU, Y. (2020). Clinical efficacy and safety evaluation of diabetic nephropathy treatment with Yishen Huashi Granule combined with Tripterygium wilfordii polyglycosides Tablet.聽Zhongcaoyao, 6045-6049.	Not clinical metabolic diseases
248<br>6	Gao, Y., Lü, R., Wang, X., Geng, J., Ren, K., Wang, Y., ... & Chen, D. (1998). A clinical trial of tang shen ning for treatment of diabetic nephropathy. Journal of Traditional Chinese Medicine= Chung i tsa Chih Ying wen pan, 18(4), 247-252.	Not clinical metabolic diseases
248<br>7	Talbott SM, Talbott JA, George A, Pugh M. Effect of a proprietary Magnolia and Phellodendron extract on weight management: A pilot, double-blind, placebo-controlled clinical trial. Altern Ther Health Med. 2013;19(5):36-42.	Not clinical metabolic diseases
248<br>8	Ge, Y., Xie, H., Li, S., Jin, B., Hou, J., Zhang, H., ... & Liu, Z. (2013). Treatment of diabetic nephropathy with Tripterygium wilfordii Hook F extract: a prospective, randomized, controlled clinical trial. Journal of Translational Medicine, 11, 1-9.	Not clinical metabolic diseases
248<br>9	Goto, H., Satoh, N., Hayashi, Y., Hikiami, H., Nagata, Y., Obi, R., & Shimada, Y. (2011). A Chinese Herbal Medicine, Tokishakuyakusan, Reduces the Worsening of Impairments and Independence after Stroke: A 1-Year Randomized, Controlled Trial. Evidence-Based Complementary and Alternative Medicine, 2011(1), 194046.	Not clinical metabolic diseases
249<br>0	Goto, H., Shimada, Y., Tanikawa, K., Sato, S., Hikiami, H., Sekiya, N., & Terasawa, K. (2003). Clinical evaluation of the effect of daio (rhei rhizoma) on the progression of diabetic nephropathy with overt proteinuria. The American journal of Chinese medicine, 31(02), 267-275.	Not clinical metabolic diseases
249<br>1	Guo, H. B., Peng, J. Q., Wang, X., Zhang, K. K., Zhong, G. Z., Chen, W. H., & Shi, G. X. (2021). Efficacy of tripterygium glycosides for diabetic nephropathy: a meta-analysis of randomized controlled trials. BMC nephrology, 22, 1-12.	Not clinical metabolic diseases

2492	Guo, W. X., Lu, X. G., Zhan, L. B., & Song, Y. (2023). Chinese herbal medicine therapy for hyperlipidemic acute pancreatitis: a systematic review and meta-analysis of randomized controlled trials. European Review for Medical & Pharmacological Sciences, 27(6).	Not clinical metabolic diseases
2493	Guo, XY and Zhu, NN, 2022. Study on the Therapeutic Effect and Anti-inflammatory Effect of Jianpi Yishen Decoction on Diabetic Nephropathy by Pathway Based on Peripheral Blood JNK/NF-κB Pathway, Chinese Journal of Pharmaceutical Biotechnology.	Not clinical metabolic diseases
2494	Han, M., Lai, L., Li, X. X., Zhao, N. Q., Li, J., Xia, Y., & Liu, J. P. (2019). An overview of the randomized placebo-controlled trials of Chinese herbal medicine formula granules. Evidence-Based Complementary and Alternative Medicine, 2019(1), 6486293.	Not clinical metabolic diseases
2495	He, C., Fu, P., Zhang, K., Xia, Q., Yang, Y., & Xie, L. (2018). Chinese herbal medicine for dyslipidemia: protocol for a systematic review and meta-analysis. Medicine, 97(44), e13048.	Not clinical metabolic diseases
2496	Hu, Y. H., Li, J., Wu, N. J., & Wu, S. (2005). Clinical observation on effect of modified huanglian wendan decoction in treating diabetic asymptomatic myocardial ischemia. Zhongguo Zhong xi yi jie he za zhi Zhongguo Zhongxiyi Jiehe Zazhi= Chinese Journal of Integrated Traditional and Western Medicine, 25(9), 790-793.	Not clinical metabolic diseases
2497	Huai, B., Huai, B., Su, Z., Song, M., Li, C., Cao, Y., ... & Liu, D. (2023). Systematic evaluation of combined herbal adjuvant therapy for proliferative diabetic retinopathy. Frontiers in Endocrinology, 14, 1157189.	Not clinical metabolic diseases
2498	Huang, K. C., Su, Y. C., Sun, M. F., & Huang, S. T. (2018). Chinese herbal medicine improves the long-term survival rate of patients with chronic kidney disease in Taiwan: a nationwide retrospective population-based cohort study. Frontiers in Pharmacology, 9, 1117.	Not clinical metabolic diseases
2499	Huang, M., Cople-Rodrigues, C. D. S., Waitzberg, D. L., Rocha, I. M. G. D., & Curioni, C. C. (2023). Changes in the gut microbiota after the use of herbal medicines in overweight and obese individuals: A systematic review. Nutrients, 15(9), 2203.	Not clinical metabolic diseases
2500	Effect evaluation for comprehensive treatment of acute stage of stroke	Not clinical metabolic diseases
2501	Huang, Y., Xie, R. M., Lu, M., Cai, Y. F., Guo, J. W., Miao, X. L., & Huang, P. X. (2007). Effect evaluation for comprehensive treatment of acute stage of stroke. Zhong xi yi jie he xue bao= Journal of Chinese Integrative Medicine, 5(3), 276-281.	Not clinical metabolic diseases
2502	Huang, Z., Li, F., Xie, B., & Zhong, X. (2022). Efficacy of a combination of Yiqi Huoxue Tongluo decoction and Chinese acupuncture in the treatment of ischemic stroke, and its effect on neurological function and activity of daily living. Tropical Journal of Pharmaceutical Research, 21(1), 185-191.	Not clinical metabolic diseases

2503	Cesarone MR, Incandela L, de Sanctis MT, et al. Treatment of diabetic microangiopathy and edema with total triterpenic fraction of Centella asiatica: a prospective, placebo-controlled randomized study. Angiology. 2001;52(Suppl 2):S55-S59. doi:10.1177/000331970105200209	Not clinical metabolic diseases
2504	Li M, Shi A, Pang H, Xie Y, Li Y, Wang Y. Herbal Medicines for Treating Metabolic Syndrome: A Systematic Review of Randomized Controlled Trials. Evid Based Complement Alternat Med. 2016;2016:5936402. doi:10.1155/2016/5936402.	Not clinical metabolic diseases
2505	Jhang JS, Liao JC, Lin YH, et al. Decreased risk of colorectal cancer among patients with type 2 diabetes receiving Chinese herbal medicine: a population-based cohort study. BMJ Open Diabetes Res Care. 2020;8(1):e000732. doi:10.1136/bmjdrc-2019-000732.	Not clinical metabolic diseases
2506	Zhang Y, Zhang Y, Wang Y, et al. Treatment options of traditional Chinese patent medicines for dyslipidemia in patients with prediabetes: A systematic review and network meta-analysis. Front Pharmacol. 2022;13:942563. doi:10.3389/fphar.2022.942563.	Not clinical metabolic diseases
2507	Jiang, R. Q., Zhang, D. X., & Bai, C. Y. (2007). Clinical study on Tangweikang in treating diabetic gastroparesis. Zhongguo Zhong xi yi jie he za zhi Zhongguo Zhongxiyi Jiehe Zazhi= Chinese Journal of Integrated Traditional and Western Medicine, 27(2), 114-116.	Not clinical metabolic diseases
2508	Huang Y, Zhang H, Liu Y, et al. Chinese herbal medicine Tangshen Formula treatment for type 2 diabetic kidney disease in the early stage: Study protocol for a randomized controlled trial. Trials. 2019;20(1):702. doi:10.1186/s13063-019-3821-6.	Not clinical metabolic diseases
2509	Jin W, Liu Y, Liu Y, et al. Efficacy and Safety of Traditional Chinese Medicine Based on the Method of "nourishing Kidney and Clearing Heat" as Adjuvant in the Treatment of Diabetes Mellitus Patients with Periodontitis: A Systematic Review and Meta-Analysis. Evid Based Complement Alternat Med. 2022;2022:3853303. doi:10.1155/2022/3853303.	Not clinical metabolic diseases
2510	Zhang Y, Wang Y, Li Y, et al. Efficacy and safety of traditional Chinese medicine external washing in the treatment of postoperative wound of diabetes complicated with anal fistula: Study protocol of a randomized, double-blind, placebo-controlled, multi-center clinical trial. Front Pharmacol. 2022;13:938270. doi:10.3389/fphar.2022.938270.	Not clinical metabolic diseases
2511	Zhang Y, Wang Y, Li Y, et al. Traditional Chinese medicine fumigation as auxiliary treatment of diabetic peripheral neuropathy: A protocol for systematic review and meta-analysis. Medicine (Baltimore). 2021;100(5):e24313. doi:10.1097/MD.0000000000024313.	Not clinical metabolic diseases
2512	Kwon HK, Zhang T, Wu XG, Qiu JY, Park S. Efficacy and safety of Di-Tan Decoction for treating post-stroke neurological disorders: a systematic review and Meta-analysis of randomized clinical trials. Chin J Nat Med. 2021;19(5):339-350. doi:10.1016/S1875-5364(21)60035-3.	Not clinical metabolic diseases

2513	Lee YS, Chen YC, Tsai YT, et al. Association of Traditional Chinese Medicine Therapy and the Risk of Vascular Complications in Patients With Type II Diabetes Mellitus: A Nationwide, Retrospective, Taiwanese-Registry, Cohort Study. Medicine (Baltimore). 2016;95(19):e3195. doi:10.1097/MD.0000000000003195.	Not clinical metabolic diseases
2514	Kim TH, Cho KH, Kim JY, et al. Are herbal medicines alone or in combination for diabetic peripheral neuropathy more effective than methylcobalamin alone? A systematic review and meta-analysis. Complement Ther Med. 2022;67:102834. doi:10.1016/j.ctim.2022.102834.	Not clinical metabolic diseases
2515	Lei, L., Jia, M., Liao, X., Lu, Y., Zhang, Y. L., Liang, X., ... & Fu, G. J. (2021). Network Meta-analysis of oral or nasal feeding with Chinese patent medicine in treatment of hypertensive intracerebral hemorrhage. Zhongguo Zhong yao za zhi= Zhongguo Zhongyao Zazhi= China Journal of Chinese Materia Medica, 46(12), 2995-3006.	Not clinical metabolic diseases
2516	Li, F. L., Deng, H., Wang, H. W., Xu, R., Chen, J., Wang, Y. F., ... & Li, B. (2011). Effects of external application of Chinese medicine on diabetic ulcers and the expressions of 尾-catenin, c-myc and K6.聽Chinese journal of integrative medicine,聽 17, 261-266.	Not clinical metabolic diseases
2517	Hui-Min, L. I., Xing, L. I. A. O., Rui-Xue, H. U., Dan-Dan, Y. U., Hao, G. U., Le, W. A. N. G., & Hui, Z. H. A. O. (2020). Analysis of status quo of RCT outcome indexes in treatment of acute ischemic stroke with traditional Chinese medicine. Zhongguo Zhong Yao Za Zhi, 2210-2220.	Not clinical metabolic diseases
2518	Li, J. Y., Yuan, L. X., Zhang, G. M., Zhou, L., Gao, Y., Li, Q. B., & Chen, C. (2016). Activating blood circulation to remove stasis treatment of hypertensive intracerebral hemorrhage: A multi-center prospective randomized open-label blinded-endpoint trial. Chinese journal of integrative medicine, 22, 328-334.	Not clinical metabolic diseases
2519	Li, K. D., Wang, Q. S., Zhang, W. W., Zhang, W. Y., Fu, S. N., Xu, D., ... & Cui, Y. L. (2020). Gardenia fructus antidepressant formula for depression in diabetes patients: A systematic review and meta-analysis. Complementary therapies in medicine, 48, 102248.	Not clinical metabolic diseases
2520	Li, P., Chen, Y., Liu, J., Hong, J., Deng, Y., Yang, F., ... & Yang, W. (2015). Efficacy and safety of tangshen formula on patients with type 2 diabetic kidney disease: a multicenter double-blinded randomized placebo-controlled trial. PloS one, 10(5), e0126027.	Not clinical metabolic diseases
2521	Li, R. L., Dong, T. W., Wei, J. G., Gao, F., Li, M., Bai, Y., ... & Xi, M. M. (2020). Meta-Analysis of the Therapeutic Effect of Shenqi Jiangtang Granule on Type 2 Diabetes Mellitus. Evidence-Based Complementary and Alternative Medicine, 2020(1), 5754823.	Not clinical metabolic diseases
2522	Li, S., Zhao, J., Liu, J., Xiang, F., Lu, D., Liu, B., ... & Chen, B. (2011). Prospective randomized controlled study of a Chinese herbal medicine compound Tangzu Yuyang Ointment for chronic diabetic foot ulcers: a preliminary report. Journal of Ethnopharmacology, 133(2), 543-550.	Not clinical metabolic diseases

2523	Li, S. F., Zhao, J. Y., & Liu, J. P. (2011). Effect of tangzu yuyang ointment on the outcome event of patients with chronic diabetic foot ulcers. Chinese Journal of Integrated Traditional and Western Medicine, 775-779.	Not clinical metabolic diseases
2524	Li, X., Zhang, J., He, R., Su, X., Li, Z., & Xie, X. (2022). Effect of Chinese herbal compounds on ocular fundus signs and vision in conventional treated-persons with non-proliferative diabetic retinopathy: A systematic review and meta-analysis. Frontiers in Endocrinology, 13, 977971.	Not clinical metabolic diseases
2525	You-shan, L., & Bo-hua, Y. (2014). Effects of compound fluid of cortex phellodendri on inflammatory cytokines and growth factors in external treatment of diabetic foot ulcer. Chinese Journal of New Drugs, 23(10), 1163-1166.	Not clinical metabolic diseases
2526	You-shan, L., Qi, Z., & Bo-hua, Y. (2016). Efficacy and safety in a multi-center clinical trial for analyzing compound fluid of cortex phellodendri in the external treatment of diabetic foot ulcers. Chinese Journal of New Drugs, 25(20), 2344-2348.	Not clinical metabolic diseases
2527	Liu, G., Liu, F., Xiao, L., Kuang, Q., He, X., Wang, Y., & Yu, Y. (2020). Treatment of hyperlipidemic acute pancreatitis with modified Dachengqi decoction combining with conventional therapy based on "six-hollow-organs to be unblocked" theory. Annals of palliative medicine, 9(4), 2045053-2042053.	Not clinical metabolic diseases
2528	Liu, H., Peng, S., Yuan, H., He, Y., Tang, J., & Zhang, X. (2023). Chinese herbal medicine combined with western medicine for the treatment of type 2 diabetes mellitus with hyperuricemia: a systematic review and meta-analysis. Frontiers in Pharmacology, 14, 1102513.	Not clinical metabolic diseases
2529	Liu, H., Zheng, J., & Li, R. H. (2015). Clinical efficacy of 'Spleen-kidney-care'Yiqi Huayu and Jiangzhuo traditional Chinese medicine for the treatment of patients with diabetic nephropathy. Experimental and Therapeutic Medicine, 10(3), 1096-1102.	Not clinical metabolic diseases
2530	Liu, J., Zhang, X., & Xu, G. (2022). Clinical efficacy, safety, and cost of nine Chinese patent medicines combined with ACEI/ARB in the treatment of early diabetic kidney disease: A network meta-analysis. Frontiers in Pharmacology, 13, 939488.	Not clinical metabolic diseases
2531	Liu, L., Zhang, Y., Zhu, Z., Yu, Z., Bao, P., & Nan, Z. (2021). Yuquan pill enhance the effect of Western medicine in treatment diabetic nephropathy: a protocol for systematic review and meta-analysis. Medicine, 100(42), e27555.	Not clinical metabolic diseases
2532	Liu, W., Zhou, L., Feng, L., Zhang, D., Zhang, C., Gao, Y., & behalf of the BOSS Group. (2021). BuqiTongluo granule for ischemic stroke, stable angina pectoris, diabetic peripheral neuropathy with qi deficiency and blood stasis syndrome: rationale and novel basket design. Frontiers in Pharmacology, 12, 764669.	Not clinical metabolic diseases

2533	Liu, X., Ge, M., Zhai, X., Xiao, Y., Zhang, Y., Xu, Z., ... & Yang, X. (2022). Traditional Chinese medicine for the treatment of diabetic kidney disease: A study-level pooled analysis of 44 randomized controlled trials. Frontiers in Pharmacology, 13, 1009571.	Not clinical metabolic diseases
2534	Liu, X., Liu, L., Chen, P., Zhou, L., Zhang, Y., Wu, Y., ... & Yi, D. (2014). Clinical trials of traditional Chinese medicine in the treatment of diabetic nephropathy—a systematic review based on a subgroup analysis. Journal of Ethnopharmacology, 151(2), 810-819.	Not clinical metabolic diseases
2535	Liu, Y., Li, Y., Du, Y., Huang, T., & Zhu, C. (2020). Multicenter clinical trials analyzing efficacy and safety of topical cortex phellodendri compound fluid in treatment of diabetic foot ulcers. Medical Science Monitor: International Medical Journal of Experimental and Clinical Research, 26, e923424-1.	Not clinical metabolic diseases
2536	Sun W, Li SM, Li ZM, et al. Efficacy and safety of tangshen formula on patients with type 2 diabetic kidney disease: A multicenter double-blinded randomized placebo-controlled trial. Chin J Integr Med. 2020;26(1):15-21. doi:10.1007/s11655-019-3090-5	Not clinical metabolic diseases
2537	Long, C., Feng, H., Liu, Z., Li, Z., Liu, J., Jiang, Y., & Yue, R. (2023). Efficacy of traditional Chinese medicine injection for diabetic kidney disease: A network meta analysis and systematic review. Frontiers in Pharmacology, 14, 1028257.	Not clinical metabolic diseases
2538	Lu, C. Y., Lee, S. R., Chang, C. J., & Chen, P. C. (2022). Adjuvant therapy with traditional Chinese medicine and long-term mortality in patients with stroke: A nationwide population-based cohort study in Taiwan. Maturitas, 158, 47-54.	Not clinical metabolic diseases
2539	Lu, Q., Li, C., Chen, W., Shi, Z., Zhan, R., & He, R. (2018). Clinical Efficacy of Jinshuibao Capsules Combined with Angiotensin Receptor Blockers in Patients with Early Diabetic Nephropathy: A Meta-Analysis of Randomized Controlled Trials. Evidence-Based Complementary and Alternative Medicine, 2018(1), 6806943.	Not clinical metabolic diseases
2540	Lu, W., & Zhong, G. L. (2003). Clinical observation on treatment of diabetic foot by integrative Chinese and Western Medicine. Zhongguo Zhong xi yi jie he za zhi Zhongguo Zhongxiyi Jiehe Zazhi= Chinese Journal of Integrated Traditional and Western Medicine, 23(12), 911-913.	Not clinical metabolic diseases
2541	Luo, X. X., Duan, J. G., Liao, P. Z., Wu, L., Yu, Y. G., Qiu, B., ... & Yao, K. (2009). Effect of Qiming Granule (芪明颗粒) on retinal blood circulation of diabetic retinopathy: A multicenter clinical trial. Chinese journal of integrative medicine, 15, 384-388.	Not clinical metabolic diseases
2542	Lv, X., Zhou, M., Liu, X., Xiang, Q., & Yu, R. (2022). Efficacy and Safety of Zhenwu Decoction in the Treatment of Diabetic Nephropathy: A Systematic Review and Meta-Analysis. Evidence-Based Complementary and Alternative Medicine, 2022(1), 2133705.	Not clinical metabolic diseases

2543	Wang H, Zhang B, Zhang Y, et al. Treatment of hyperlipidemic acute pancreatitis with modified Dachengqi decoction combining with conventional therapy based on "six-hollow-organs to be unblocked" theory. Ann Palliat Med. 2020;9(4):2045-2053. doi:10.21037/apm-20-1106	Not clinical metabolic diseases
2544	Mao, W., Yang, N., Zhang, L., Li, C., Wu, Y., Ouyang, W., ... & Liu, X. (2021). Bupi yishen formula versus losartan for non-diabetic stage 4 chronic kidney disease: A randomized controlled trial. Frontiers in Pharmacology, 11, 627185.	Not clinical metabolic diseases
2545	Mei, J., Yang, L., Wang, D., & Wang, H. (2021). Efficacy and safety of Shenkang injection in the treatment of chronic renal failure: A protocol of a randomized controlled trial. Medicine, 100(48), e27748.	Not clinical metabolic diseases
2546	Song, C., Zhu, Z., Liu, L., Liu, S., Li, Y., Xiao, Y., ... & Nan, Z. (2023). The efficacy and safety of Niaoduqing granules in the treatment of diabetic kidney disease: a systematic review and meta-analysis. Frontiers in Pharmacology, 14, 1180751.	Not clinical metabolic diseases
2547	Tang, Z., Chen, H., Fang, W., Luo, Y., Wang, H., Wei, C., ... & Chen, Q. (2018). Efficacy and safety of Sancailianmei Particle compared with acarbose in patients with type 2 diabetes mellitus inadequately controlled with metformin. Int J Clin Exp Med, 11(9), 9005-9016.	Not clinical metabolic diseases
2548	Tian, J., Li, M., Liao, J., Li, J., & Tong, X. (2013). Chinese herbal medicine banxiaxiexin decoction treating diabetic gastroparesis: a systematic review of randomized controlled trials. Evidence-Based Complementary and Alternative Medicine, 2013(1), 749495.	Not clinical metabolic diseases
2549	Wang, H., Chen, H., Gao, Y., Wang, S., Wang, X., Tang, X., ... & Chen, Q. (2020). The effect of wuling capsule on depression in Type 2 diabetic patients. Bioscience Reports, 40(2), BSR20191260.	Not clinical metabolic diseases
2550	Wang, J. (2018). Efficacy of Xu-Huang mixtures for the treatment of diabetic lower extremity atherosclerotic occlusive disease. Journal of Shanghai Jiaotong University (Medical Science), 1333-1336.	Not clinical metabolic diseases
2551	Wang, N., Ju, S., & Yang, F. H. (2018). Efficacy and safety of compound Cortex Phellodendri Liquid for diabetic foot ulcer: A meta-analysis. Chi J New Drugs, 27(15), 1771-75.	Not clinical metabolic diseases
2552	Wang, X. (2015). Clinical observation of Shenluoan Decoction for treating obese patients with early diabetic nephropathy.聽Zhongcaoyao, 245-249.	Not clinical metabolic diseases
2553	Wang, Y., Li, M., Li, C., Xu, S., Wu, J., Zhang, G., & Cai, Y. (2020). Efficacy and safety of Shenkang injection as adjuvant therapy in patients with diabetic nephropathy: a protocol for systematic review and meta-analysis. Medicine, 99(52), e23821.	Not clinical metabolic diseases
2554	Wang, Z. H., Wang, X. Y., Zhang, Y. L., Zhao, L., & Li, H. X. (2013). Treatment of diabetic foot by clearing heat, detoxification, activating blood, and dredging collaterals method. Zhongguo Zhong xi yi jie he za zhi Zhongguo Zhongxiyi Jiehe Zazhi= Chinese Journal of Integrated Traditional and Western Medicine, 33(4), 480-483.	Not clinical metabolic diseases

2555	Wei Yan, W. Y., Hong YuZhi, H. Y., & Ye Xun, Y. X. (2008). Effect of Tang No. 1 granule in treating patients with impaired glucose tolerance.	Not clinical metabolic diseases
2556	Wei, Y., & Xie, W. (2007). Therapeutic effect of Weidong Kang on diabetic gastroparesis. Chinese Journal of Tissue Engineering Research, 6901-6904.	Not clinical metabolic diseases
2557	Wu, W., Liu, Y. L., Wan, Y. G., Cao, D. W., Fang, Q. J., Tu, Y., ... & Hu, W. (2020). Multi-targeted therapeutic effects of Huangkui Capsules on insulin resistance and urine microalbumin in early diabetic kidney disease patients. Zhongguo Zhong yao za zhi= Zhongguo Zhongyao Zazhi= China Journal of Chinese Materia Medica, 45(23), 5797-5803.	Not clinical metabolic diseases
2558	Xu, Z., Qian, L., Niu, R., Yang, Y., Liu, C., & Lin, X. (2022). Efficacy of Huangqi injection in the treatment of hypertensive nephropathy: a systematic review and meta-analysis. Frontiers in Medicine, 9, 838256.	Not clinical metabolic diseases
2559	Yang, Y., Sha, W., Hou, K., Xu, Y., Tan, S., Yin, H., ... & Lei, T. (2022). Efficacy and Safety of Wuling Powder in the Treatment of Patients with Diabetic Nephropathy: A Systematic Review and Meta-Analysis. Evidence-Based Complementary and Alternative Medicine, 2022(1), 1720749.	Not clinical metabolic diseases
2560	Yang, Y. W., Zhou, Y. W., & Ge, M. L. (2023). The effect of externally applied traditional Chinese medicine in diabetic foot: a systematic review and meta-analysis of 34 RCTs. The Foot, 56, 102045.	Not clinical metabolic diseases
2561	Zeng, J. Y., Wang, Y., Miao, M., & Bao, X. R. (2021). The effects of rhubarb for the treatment of diabetic nephropathy in animals: a systematic review and meta-analysis. Frontiers in pharmacology, 12, 602816.	Not clinical metabolic diseases
2562	Zhang, Y. M., Guo, L. R., & XU, J. F. (2021). Clinical Study on Dialectical Treatment of Modified Xiangsha Liu Junzitang to Diabetes Gastroparesis with Weakness Syndrome of Spleen and Stomach. Chinese Journal of Experimental Traditional Medical Formulae, 64-69.	Not clinical metabolic diseases
2563	Methods in Medicine, C. A. M. (2023). Retracted: Efficacy and Clinical Value of Negative-Pressure Wound Therapy with Instillation (NPWTi) of Compound Phellodendron Liquid in the Treatment of Diabetic Foot Ulcer: A Prospective Cohort Study.	Not clinical metabolic diseases
2564	毕连宝,于笑艳.益气解毒生肌中药治疗糖尿病足溃疡湿热毒盛证疗效及对TNF-α、IL-6、AGEs的影响[J].现代中西医结合杂志,2017,26(31):3461-3463.	Not clinical metabolic diseases
2565	蔡美玲,林静娟,刘桂珍.康复新液联合红外线照射治疗老年糖尿病患者皮肤溃疡的效果观察[J].北方药学,2014,11(03):97-98.	Not clinical metabolic diseases
2566	蔡镇.莲子清心饮对糖尿病肾病临床疗效及24h尿蛋白定量的影响[J].中医药信息,2016,33(02):66-68.	Not clinical metabolic diseases

2567	曹建春,张东萍.清热利湿法治疗糖尿病足的临床研究[J].疑难病杂志,2006,(04):266-268.	Not clinical metabolic diseases
2568	曹建辉,杨秀军,高玉林,等.美宝湿润烧伤膏联合激光照射治疗糖尿病足的临床观察[J].中国农村卫生,2015,(12):31.	Not clinical metabolic diseases
2569	曹胜雁,李霞.中药熏蒸治疗糖尿病足 100 例[J].光明中医,2010,25(09):1638.	Not clinical metabolic diseases
2570	曹甦.涤浊汤治疗痰湿瘀浊型糖尿病的临床观察[J].黑龙江医学,2003,(05):358.	Not clinical metabolic diseases
2571	曾艺鹏,王爱华,胡蕴刚.复方配伍治疗伴湿热兼证糖尿病胃轻瘫的研究[J].现代中西医结合杂志,2006,(15):2023-2024+2140.	Not clinical metabolic diseases
2572	陈博宇,邵亚新.清热祛湿通络法治疗糖尿病周围神经病变的研究[J].糖尿病新世界,2016,19(04):105-107.DOI:10.16658/j.cnki.1672-4062.2016.04.105.	Not clinical metabolic diseases
2573	王敬.益阴通络法治疗糖尿病胃肠功能紊乱的临床研究[J].糖尿病新世界,2019,22(10):184-185.DOI:10.16658/j.cnki.1672-4062.2019.10.184.	Not clinical metabolic diseases
2574	周丽萍.自拟化湿活血汤联合甲钴胺治疗 2 型糖尿病下肢周围神经病变临床疗效分析[J].双足与保健,2018,27(18):117-118.DOI:10.19589/j.cnki.issn1004-6569.2018.18.117.	Not clinical metabolic diseases
2575	翟力军.益肾化湿颗粒联合坎地沙坦酯治疗糖尿病肾病的临床研究[J].河北医药,2019,41(16):2518-2520.	Not clinical metabolic diseases
2576	陈军权,卢元润.中药浸浴联合美宝湿润烧伤膏治疗糖尿病足部溃疡的临床效果观察[J].中医临床研究,2018,10(23):69-70.	Not clinical metabolic diseases
2577	陈培智, 陈绍辉, 李树浩, & 温奕欣. (2002). 中西医结合治疗糖尿病肾病临床观察. 中国中西医结合肾病杂志, 3(3), 145-147.	Not clinical metabolic diseases
2578	陈璇,余江毅."玉葵清"干预治疗早期 2 型糖尿病肾病的临床研究[J].江苏中医药,2008,(07):48-49.	Not clinical metabolic diseases
2579	陈学君. (2017). 基于临床证据的中医药治疗糖尿病肾病早期循证病机—证治体系构建研究 (Master's thesis, 山东中医药大学).	Not clinical metabolic diseases
2580	陈迎春,刘中香,王亚梅,等.中药配方颗粒与传统中药汤剂用于老年重度糖尿病肾病的综合疗效比较[J].中国老年学杂志,2019,39(09):2101-2104.	Not clinical metabolic diseases

2581	陈永翀,弥伟,刘江,等.MEBO治疗 5 级糖尿病足溃疡临床疗效观察[J].中国烧伤创疡杂志,2015,27(01):58-66.	Not clinical metabolic diseases
2582	崔晓斌,王英果,罗振立.局部清创换药联合金黄散外用治疗糖尿病足临床观察[J].中国医药指南,2011,9(24):318-319.DOI:10.15912/j.cnki.gocm.2011.24.225.	Not clinical metabolic diseases
2583	崔雅斌,邹迪,付艳艳,等.温补脾肾化瘀利水法治疗脾肾阳虚夹湿瘀型糖尿病肾病水肿临床分析[J].中国中医药现代远程教育,2022,20(03):102-104.	Not clinical metabolic diseases
2584	单玮,阙华发,赵珺,等.顾氏外科个体化诊疗方案治疗糖尿病性足病的临床观察[J].医药论坛杂志,2022,43(11):95-98.	Not clinical metabolic diseases
2585	邓海镖,高晓欢,汪栋材,等.清热祛湿法治疗糖尿病周围神经病变临床研究[J].广州中医药大学学报,2020,37(03):387-393.DOI:10.13359/j.cnki.gzxbtcm.2020.03.001.	Not clinical metabolic diseases
2586	邓建华,韩旭,张建德,等.生蜂蜜合云南白药外敷治疗糖尿病足部溃疡 33 例疗效观察[J].新中医,2007,(09):86-87+8.DOI:10.13457/j.cnki.jncm.2007.09.040.	Not clinical metabolic diseases
2587	邓泽孝,彭如一,陈惠英,等.聚维酮碘软膏和金匮肾气丸治疗糖尿病足 17 例疗效观察[J].现代医院,2005,(04):66-68.	Not clinical metabolic diseases
2588	丁凯.清热利湿活血法治疗糖尿病肾病III、IV期临床研究[J].内蒙古中医药,2013,32(19):50-52.DOI:10.16040/j.cnki.cn15-1101.2013.19.014.	Not clinical metabolic diseases
2589	丁携云,求珍亚,陆林飞,等.健脾利湿方联合硫酸镁及钙离子拮抗剂治疗妊娠期高血压的效果[J].中国生化药物杂志,2016,36(04):121-123.	Not clinical metabolic diseases
2590	杜娟娇,张力,李杰辉.负压封闭引流结合美宝湿润烧伤膏治疗糖尿病足 16 例[J].广西中医药,2012,35(01):21-22.	Not clinical metabolic diseases
2591	杜丽荣,王东济,李东明.糖足泡液系列方联合湿润烧伤膏治疗糖尿病足的临床观察[J].四川中医,2011,29(05):103-104.	Not clinical metabolic diseases
2592	杜新, & 金妙文. (2004). 地羽糖肾片治疗糖尿病肾病络热血瘀, 阴虚气耗证的临床研究. 中华实用中西医杂志, 17(023), 3583-3586.	Not clinical metabolic diseases
2593	段传皓,张松青,薛荃.崔桐华教授从脾胃湿热论治糖尿病湿热困脾型患者 1 例[J].基层医学论坛,2016,20(01):87-88.DOI:10.19435/j.1672-1721.2016.01.062.	Not clinical metabolic diseases
2594	范婷. (2020). 糖肾汤治疗早期糖尿病肾病临床观察及老年糖尿病肾病与衰弱的关联 (Doctoral dissertation, 北京中医药大学).	Not clinical metabolic diseases

2595	张丽艳,袁继龙.湿润烧伤膏治疗Wagner 1～2 级糖尿病足疗效观察[J].中国烧伤创疡杂志,2019,31(02):101-104.	Not clinical metabolic diseases
2596	范育玲,闫晓燕.自拟化痰熄风汤治疗高血压 23 例疗效观察[J].云南中医中药杂志,2016,37(07):54-55.DOI:10.16254/j.cnki.53-1120/r.2016.07.025.	Not clinical metabolic diseases
2597	方季惟,万建伟,王瑛,等.清热祛湿通络汤联合前列地尔注射液治疗糖尿病周围血管病变 34 例[J].河南中医,2019,39(10):1548-1551.DOI:10.16367/j.issn.1003-5028.2019.10.0383.	Not clinical metabolic diseases
2598	费璇,石志敏,李雪青.升阳除湿防风汤联合西药治疗脾虚湿胜型 2 型糖尿病性腹泻临床观察[J].中国中西医结合消化杂志,2020,28(10):766-771.	Not clinical metabolic diseases
2599	冯建庄.辨证治疗糖尿病视物昏花 52 例[J].河南中医,2010,30(01):60-62.DOI:10.16367/j.issn.1003-5028.2010.01.035.	Not clinical metabolic diseases
2600	冯居秦.化痰祛瘀减肥汤治疗痰瘀型肥胖症 61 例[J].辽宁中医杂志,2015,42(06):1260-1261.DOI:10.13192/j.issn.1000-1719.2015.06.042.	Not clinical metabolic diseases
2601	冯少玲,何采辉,李文纯,等.脾虚痰湿型单纯性肥胖症应用五苓散治疗的BMI及血脂变化观察[J].中国医药科学,2015,5(09):67-69.	Not clinical metabolic diseases
2602	冯占荣.益肾利湿汤治疗慢性肾衰竭 21 例临床观察[J].长春中医药大学学报,2012,28(05):863.DOI:10.13463/j.cnki.cczyy.2012.05.048.	Not clinical metabolic diseases
2603	冯占荣,马俊杰.中西药合用治疗阳虚痰湿型代谢综合征 30 例临床研究[J].江苏中医药,2015,47(11):32-34.	Not clinical metabolic diseases
2604	冯志海,岳新,吕久省,等.糖痹痛膏穴位贴敷治疗糖尿病周围神经病变 144 例临床疗效观察[J].中华中医药杂志,2009,24(09):1244-1245.	Not clinical metabolic diseases
2605	于忠军.益气养血、温经通络法治疗糖尿病足临床探讨[J].糖尿病新世界,2015,(13):97-98.DOI:10.16658/j.cnki.1672-4062.2015.13.004.	Not clinical metabolic diseases
2606	傅奕,朱莹,陈帮明,等.益肾化湿颗粒与盐酸川芎嗪联合用药对糖尿病肾病患者血脂及肾功能的影响[J].时珍国医国药,2021,32(04):911-913.	Not clinical metabolic diseases
2607	甘宇,陈盛业.糖足 1 号治疗湿热毒邪伤阴之糖尿病足的临床疗效[J].中国药物经济学,2022,17(06):74-77+82.	Not clinical metabolic diseases
2608	高海侠,刘晓华,张晓月.健脾祛痰中药联合健康指导治疗肥胖痰湿型多囊卵巢综合征疗效评价[J].中国计划生育学杂志,2019,27(07):854-857.	Not clinical metabolic diseases

2609	高建魁, & 王水香. (2008). 中西医结合治疗糖尿病肾病 36 例. 实用中医内科杂志, 22(9), 44-45.	Not clinical metabolic diseases
2610	高晓村.葛根芩连汤合程氏萆薢分清饮加减治疗湿热型糖尿病肾病的疗效观察[J].北京中医药,2009,28(09):718-719.	Not clinical metabolic diseases
2611	宫淑华.湿润烧伤膏治疗糖尿病足 42 例[J].山东中医杂志,2011,30(09):620-621.DOI:10.16295/j.cnki.0257-358x.2011.09.007.	Not clinical metabolic diseases
2612	巩鸿霞.中药联合激光治疗糖尿病性黄斑水肿 34 例临床观察[J].江苏中医药,2010,42(09):40.	Not clinical metabolic diseases
2613	苟雪琼.四妙勇安汤、黄芪注射液、湿润烧伤膏联合西药治疗糖尿病坏疽随机平行对照研究[J].实用中医内科杂志,2015,29(12):127-129.DOI:10.13729/j.issn.1671-7813.2015.12.55.	Not clinical metabolic diseases
2614	古金成,王少柯.加服自拟化浊益肾方治疗早期糖尿病肾病的临床观察[J].广西中医药,2011,34(03):8-9.	Not clinical metabolic diseases
2615	桂程丽,程彩涛.中药足浴联合康复新液湿敷治疗糖尿病足 24 例[J].河南中医,2015,35(11):2796-2797.DOI:10.16367/j.issn.1003-5028.2015.11.1198.	Not clinical metabolic diseases
2616	郭彩云.外用消疽膏治疗糖尿病足II级坏疽 68 例临床研究[J].新中医,2003,(10):32-33.DOI:10.13457/j.cnki.jncm.2003.10.017.	Not clinical metabolic diseases
2617	郭丹丹,张金良,于思明,等.清流平复汤治疗湿浊内停型非酒精性脂肪性肝病的效果[J].中国临床保健杂志,2020,23(04):478-482.	Not clinical metabolic diseases
2618	郭秀芝,王利民.中西医结合治疗糖尿病足 31 例[J].中国中医药科技,2009,16(01):61.	Not clinical metabolic diseases
2619	郭兆安.连黄降浊颗粒治疗高血压性肾损害的临床研究[J].中国中西医结合急救杂志,2007,(01):3-6.	Not clinical metabolic diseases
2620	韩晓玲,蔡晖.疮灵液联合穴位按摩治疗糖尿病足 52 例疗效观察[J].浙江中医杂志,2012,47(12):888-889.	Not clinical metabolic diseases
2621	何坤,甘仲霖,杨艳,等.湿润烧伤膏治疗糖尿病足溃疡疗效的系统评价[J].西南医科大学学报,2017,40(03):243-248.	Not clinical metabolic diseases
2622	何新华. (2013). 中西医结合治疗糖尿病肾病 125 例. 中国中医药现代远程教育, 11(23), 54-55.	Not clinical metabolic diseases

2623	和瑞欣.中药内服治疗糖尿病性胃轻瘫临床研究[J].中医学报,2014,29(05):645-646.DOI:10.16368/j.issn.1674-8999.2014.05.008.	Not clinical metabolic diseases
2624	胡斌,徐耀凤.美宝湿润烧伤膏联合龙血竭治疗糖尿病足的疗效观察[J].护理研究,2015,29(13):1639-1641.	Not clinical metabolic diseases
2625	胡萍. (2020). 中药复方辨证治疗早期糖尿病肾病的 Meta 分析及临床病例观察 (Master's thesis, 山东中医药大学).	Not clinical metabolic diseases
2626	胡清, 王玉红, & 刘秀菊. (2007). 复方紫草油局部湿敷治疗糖尿病足的临床研究. 中华实用中西医杂志, 20(015), 1321-1322.	Not clinical metabolic diseases
2627	胡昱月.舒洛地特联合益肾化湿颗粒对早期老年糖尿病肾病氧化应激及内皮功能的影响[J].药品评价,2018,15(07):28-30.	Not clinical metabolic diseases
2628	黄飞,杨超丽.自拟生肌散联合湿润烧伤膏治疗糖尿病足的临床研究[J].中国医药导报,2010,7(10):139-140.	Not clinical metabolic diseases
2629	施晓星,邵长娟,张红,等.肾康栓联合缬沙坦治疗糖尿病肾病临床观察[J].临床医药文献电子杂志,2015,2(07):1207+1209.DOI:10.16281/j.cnki.jocml.2015.07.012.	Not clinical metabolic diseases
2630	黄伟坚.解毒软肝饮治疗门静脉高压随机平行对照研究[J].实用中医内科杂志,2015,29(05):54-56.DOI:10.13729/j.issn.1671-7813.2015.05.26.	Not clinical metabolic diseases
2631	黄蔚,黄江荣.辨证治疗糖尿病肢端坏疽 21 例[J].河南中医,2013,33(06):919-920.DOI:10.16367/j.issn.1003-5028.2013.06.038.	Not clinical metabolic diseases
2632	黄欣,何晓微,唐乾利,等.MEBT/MEBO治疗老年Wagner III级糖尿病足的临床研究[J].中国烧伤创疡杂志,2017,29(01):44-47.	Not clinical metabolic diseases
2633	焦玉燕. (2010). 湿润烧伤膏在治疗糖尿病足溃疡外科局部换药中的作用和疗效. 健康天地: 学术版, 4(11), 9-10.	Not clinical metabolic diseases
2634	靳福利, 丁爱萍, & 张荣光. (2009). 补虚祛瘀利湿法治疗糖尿病肾病疗效研究. 中华现代中医学杂志, 5(5), 280-283.	Not clinical metabolic diseases
2635	孔繁婧. (2016). 糖尿病肾病中医证治规律探究 (Doctoral dissertation, 北京: 北京中医药大学).	Not clinical metabolic diseases
2636	寇吉友,李密密,马金成,等.募合配穴联合胃通汤治疗糖尿病胃轻瘫(脾虚湿阻)随机平行对照研究[J].实用中医内科杂志,2018,32(11):57-61.DOI:10.13729/j.issn.1671-7813.Z20180240.	Not clinical metabolic diseases

2637	邝丽辉.中药联合胰岛素纱布湿敷治疗糖尿病足难治性溃疡的疗效和护理[J].中国医疗前沿,2013,8(17):103-104.	Not clinical metabolic diseases
2638	付晓雪,黄辉,李勇.154 例老年糖尿病患者皮肤病变的临床分析[J].中国热带医学,2008,(06):956.	Not clinical metabolic diseases
2639	李碧娟,李连君,黄丽雅,等.芦荟搽剂治疗糖尿病烫伤效果观察[J].护理学杂志,2009,24(12):37-38.	Not clinical metabolic diseases
2640	彭秋玲.中药熏蒸治疗糖尿病足的护理体会[J].当代护士(专科版),2010,(09):106-107.	Not clinical metabolic diseases
2641	李传吉,邓光旺,李俊,等.原位再生复原技术治疗糖尿病肛周组织泛发性溃烂缺损的临床观察[J].中国烧伤创疡杂志,2012,24(06):497-499.	Not clinical metabolic diseases
2642	李翠萍,王倩,顾叶文,等.益肾化湿颗粒治疗糖尿病肾病有效性与安全性的Meta分析[J].实用医学杂志,2020,36(05):682-688.	Not clinical metabolic diseases
2643	李董平. (2017). 中医治疗代谢综合征用药规律分析及 Meta 分析 (Doctoral dissertation, 广州: 暨南大学).	Not clinical metabolic diseases
2644	李繁强.综合疗法治疗糖尿病足合并气性坏疽临床体会[J].中国烧伤创疡杂志,2019,31(04):245-247.	Not clinical metabolic diseases
2645	陈景亮,凌方明,吕雄,等.益气温阳通络法治疗糖尿病周围神经病变临床观察[J].中国中医急症,2005,(05):427-428.	Not clinical metabolic diseases
2646	李杰辉,黄欣,唐乾利,等.MEBT/MEBO治疗糖尿病足溃疡的临床疗效观察[J].中国烧伤创疡杂志,2016,28(01):22-25.	Not clinical metabolic diseases
2647	李杰辉,王丽,杜娟娇,等.湿润烧伤膏对糖尿病足患者溃疡创面凋亡基因Bcl-2 和Bax的影响[J].时珍国医国药,2012,23(07):1616-1617.	Not clinical metabolic diseases
2648	李凌霄,徐俊,王鹏华,等.复方黄柏液局部应用对糖尿病足溃疡愈合的临床研究[J].重庆医科大学学报,2017,42(03):289-294.DOI:10.13406/j.cnki.cyxb.001177.	Not clinical metabolic diseases
2649	李凌霄,徐俊,王鹏华,等.复方黄柏液局部应用对糖尿病足溃疡愈合的临床研究[J].重庆医科大学学报,2017,42(03):289-294.DOI:10.13406/j.cnki.cyxb.001177.	Not clinical metabolic diseases
2650	李平,魏翠艳.湿润烧伤膏对Wagner 2～3 级糖尿病足患者神经修复的作用研究[J].中国烧伤创疡杂志,2021,33(02):91-94+102.	Not clinical metabolic diseases

2651	李萍,陈俭波,沃红霞,等.三黄湿敷水凝胶治疗糖尿病足溃疡创面临床疗效观察[J].内蒙古中医药,2015,34(12):18-19.DOI:10.16040/j.cnki.cn15-1101.2015.12.020.	Not clinical metabolic diseases
2652	李秋萍.中药清热解毒水剂外用治疗糖尿病足 32 例临床观察[J].中国医药指南,2015,13(10):218-219.DOI:10.15912/j.cnki.gocm.2015.10.169.	Not clinical metabolic diseases
2653	刘惠洁.中医外治法治疗糖尿病足溃疡 146 例临床观察[J].光明中医,2018,33(01):13-14.	Not clinical metabolic diseases
2654	李绍钦. (2009). 中医络病理论与早期糖尿病肾病的相关研究 (Doctoral dissertation, 广州中医药大学博士学位论文).	Not clinical metabolic diseases
2655	李爽,王德惠.自拟尿感宁治疗糖尿病合并泌尿系感染 40 例[J].吉林中医药,2012,32(07):685-686.DOI:10.13463/j.cnki.jlzyy.2012.07.048.	Not clinical metabolic diseases
2656	李填新,谢翊.六君子汤加减方联合清热祛浊胶囊对T2DM痰湿郁热证患者糖脂代谢的影响观察[J].北方药学,2020,17(06):93-94.	Not clinical metabolic diseases
2657	吴玉英,刘文捷,洪顺忠,等.中药滋肾化浊汤加减联合缬沙坦治疗老年高血压早期肾损害的疗效分析[J].北方药学,2019,16(12):93-94.	Not clinical metabolic diseases
2658	李文静,谭支文,刘慧纯,等.通窍化浊醒脑汤辅助治疗高血压基底节区脑出血的临床效果及对凝血功能的价值[J].辽宁中医杂志,2021,48(02):86-90.DOI:10.13192/j.issn.1000-1719.2021.02.024.	Not clinical metabolic diseases
2659	李晓政,杨斌.加味猪苓汤治疗湿热血瘀型代谢综合征合并肾结石临床观察[J].山西中医,2017,33(12):37-38+40.	Not clinical metabolic diseases
2660	屈小青,袁莲芳,党楠,等.中西医结合治疗原发性高血压 70 例临床观察[J].中医药导报,2015,21(21):61-63.DOI:10.13862/j.cnki.cn43-1446/r.2015.21.023.	Not clinical metabolic diseases
2661	李亚廷.中医辨证治疗糖尿病足病 57 例体会[J].医学信息(中旬刊),2011,24(09):4535-4536.	Not clinical metabolic diseases
2662	李业贝. (2021). 益肾化湿颗粒治疗糖尿病肾病的临床疗效和安全性的队列研究 (Doctoral dissertation, 南昌: 南昌大学).	Not clinical metabolic diseases
2663	李玉娟, 2021. 慢阻肺急性加重合并糖尿病的临床特点及中医诊治规律研究, 北京中医药大学.	Not clinical metabolic diseases
2664	李正芳,崔春风,钟绍敏.中西医结合配合湿润烧伤膏治疗糖尿病足 21 例[J].中国中医急症,2010,19(09):1610-1612.	Not clinical metabolic diseases

2665	李智,李国信,赵婷婷.四妙勇安汤合仙方活命饮联合拔腐祛瘀方与生肌散分期外敷治疗湿热内蕴型糖尿病足(脱疽)78 例临床观察[J].实用中医内科杂志,2015,29(01):31-33.DOI:10.13729/j.issn.1671-7813.2015.01.14.	Not clinical metabolic diseases
2666	廖晓,蔡锦松.健脾利湿通络法治疗糖尿病肾病蛋白尿的临床疗效观察[J].世界最新医学信息文摘,2019,19(28):167-168.DOI:10.19613/j.cnki.1671-3141.2019.28.078.	Not clinical metabolic diseases
2667	周丽萍.自拟化湿活血汤联合甲钴胺治疗 2 型糖尿病下肢周围神经病变临床疗效分析[J].双足与保健,2018,27(18):117-118.DOI:10.19589/j.cnki.issn1004-6569.2018.18.117.	Not clinical metabolic diseases
2668	刘成琼.34 例非缺血性糖尿病足中西医结合治疗临床观察[J].中医临床研究,2012,4(24):10-12.	Not clinical metabolic diseases
2669	刘洪双,刘莉,隋艳波.加减黄连温胆汤治疗代谢综合征(痰湿蕴结证)的临床研究[J].中国中医急症,2016,25(10):1837-1839+1850.	Not clinical metabolic diseases
2670	刘绛云,周经钲,王永生,等.药食干预痰湿体质高血压前期疗效评价[J].实用中医内科杂志,2019,33(11):66-69.DOI:10.13729/j.issn.1671-7813.z20190327.	Not clinical metabolic diseases
2671	刘丽霞.中医湿敷治疗糖尿病性关节炎的临床研究[J].糖尿病新世界,2017,20(03):7-8.DOI:10.16658/j.cnki.1672-4062.2017.03.007.	Not clinical metabolic diseases
2672	刘丽霞.中医湿敷治疗糖尿病性关节炎的临床研究[J].糖尿病新世界,2017,20(03):7-8.DOI:10.16658/j.cnki.1672-4062.2017.03.007.	Not clinical metabolic diseases
2673	刘荣东,江慧梅,黄如萍.健脾化痰法对痰湿型代谢综合征中脂肪肝并高尿酸患者的影响[J].广州中医药大学学报,2012,29(03):239-242+246.DOI:10.13359/j.cnki.gzxbtcm.2012.03.007.	Not clinical metabolic diseases
2674	刘天,黄泽,魏爱生,等.舒筋、温筋洗剂熏洗联合α-硫辛酸对糖尿病周围神经病变震动感觉阈值和TSS评分影响随机平行对照研究[J].实用中医内科杂志,2012,26(05):57-59.	Not clinical metabolic diseases
2675	刘喜庆, 张新华, & 阳旭. (2009). 中西医结合治疗糖尿病肾病 20 例临床观察. 内蒙古中医药, (5X), 18-18.	Not clinical metabolic diseases
2676	杨翠荣.茵陈蒿汤加味对湿热困脾型 2 型糖尿病血脂的影响[J].中国继续医学教育,2016,8(14):207-208.	Not clinical metabolic diseases
2677	刘孝琴,李悦,李雅楠.益肾化湿颗粒对早期糖尿病肾病患者CRP及IL-8 的影响[J].中国中西医结合肾病杂志,2013,14(06):538-539.	Not clinical metabolic diseases
2678	刘雪琴.健脾渗湿通络法治疗糖尿病周围神经病变的临床研究[J].现代中西医结合杂志,2018,27(08):868-870.	Not clinical metabolic diseases

2679	刘雪云. (2011). 中西医结合治疗糖尿病尿路感染 48 例分析. 健康必读: 下半月, (3), 64-64.	Not clinical metabolic diseases
2680	刘艳飞. (2018). 中药治疗代谢综合征的药物筛选及分子作用机制研究 (Doctoral dissertation, 北京: 北京中医药大学).	Not clinical metabolic diseases
2681	刘元君,殷美琦,吴怡,等.基于吴门“清/下法”的三黄胶囊对 2 型糖尿病便秘患者血糖水平及肠道菌群的影响研究[J].中医临床研究 ,2021,13(26):27-30.	Not clinical metabolic diseases
2682	娄少颖,刘毅.中药治疗重度脂肪肝合并糖尿病 1 例[J].河北中医 ,2008,(02):158.	Not clinical metabolic diseases
2683	娄志杰,韩向莉,邵岩.疏肝温肾通腑泻浊方治疗糖尿病III期肾病[J].医学理论与实践 ,2017,30(18):2706-2707.DOI:10.19381/j.issn.1001-7585.2017.18.021.	Not clinical metabolic diseases
2684	陆敏康,孟庆叶,陆晓东.湿性医疗技术配合中药药浴治疗糖尿病足湿性坏疽[J].中国烧伤创疡杂志,2011,23(04):326-328+330.	Not clinical metabolic diseases
2685	路建饶,易扬,刘文瑞,等.益肾泄浊方内服外用治疗IV期糖尿病肾病的前瞻性多中心临床研究[J].时珍国医国药,2018,29(05):1137-1140.	Not clinical metabolic diseases
2686	张桂静.辨证分型联合西药治疗糖尿病周围神经病变随机平行对照研究[J].实用中医内科杂志 ,2018,32(07):18-20.DOI:10.13729/j.issn.1671-7813.z20170151.	Not clinical metabolic diseases
2687	吕秀群, & 刘得华. (2012). 除痹汤内外合治糖尿病周围神经病变疗效观察. 内蒙古中医药, 31(16), 21-22.	Not clinical metabolic diseases
2688	吕志强. (2010). 唐舒康液对糖尿病足截肢率的影响. 国际中医中药杂志, 32(6), 509-510.	Not clinical metabolic diseases
2689	门洁,张晓辉,孙敬文,等.糖尿病黄斑水肿的中西医结合治疗的疗效观察[J].航空航天医学杂志,2015,26(07):794-795.	Not clinical metabolic diseases
2690	莫永兰,黄柳妮.黄五酊湿敷治疗糖尿病性II期褥疮的疗效观察[J].现代护理,2006,(10):933.	Not clinical metabolic diseases
2691	穆罕穆德·萨克,哈斯玛M·哈默德.湿润烧伤膏在慢性糖尿病足溃疡愈合中的作用:随机对照试验[J].中国烧伤创疡杂志,2012,24(05):355.	Not clinical metabolic diseases
2692	欧娇英,高建东.健脾益肾化浊祛瘀方治疗糖尿病肾病CKD2～3 期的临床观察[J].中国中西医结合肾病杂志,2014,15(11):973-975.	Not clinical metabolic diseases

2693	朴春丽,王秀阁,杨世忠.龙胆泻肝汤加减治疗期糖尿病肾病 30 例临床研究[J].山东中医杂志,2004,(12):714-716.DOI:10.16295/j.cnki.0257-358x.2004.12.004.	Not clinical metabolic diseases
2694	钱语,马幸,戴荣峰.黄葵胶囊治疗非增殖期糖尿病视网膜病变湿热证临床研究[J].光明中医,2021,36(15):2489-2492.	Not clinical metabolic diseases
2695	卿燕,魏平.湿润烧伤膏与负压封闭引流技术治疗糖尿病足的疗效对比[J].中国烧伤创疡杂志,2015,27(04):263-265.	Not clinical metabolic diseases
2696	邱建玲,黎雯,吴英桂,等.湿毒清胶囊联合左西替利嗪对糖尿病皮肤瘙痒症老年患者疗效及炎性因子的影响[J].中国中西医结合皮肤性病学杂志,2020,19(04):342-345.	Not clinical metabolic diseases
2697	邵鑫,吴学苏,冉颖卓,等.中药外用治疗糖尿病足溃疡 31 例临床观察[J].江苏中医药,2017,49(12):38-40.	Not clinical metabolic diseases
2698	施莹,赖龙胜,黄腾蛟,等.黄连温胆汤联合贝那普利治疗湿热中阻型糖尿病肾病临床观察[J].中国中医药现代远程教育,2021,19(11):144-147.	Not clinical metabolic diseases
2699	舒晓春,陈孝银,朱晓峰,等.糖胃舒胶囊治疗糖尿病性胃轻瘫 40 例[J].陕西中医,2003,(09):771-772.	Not clinical metabolic diseases
2700	宋诵文,曾琦,吴新民.温肾化浊、逐瘀通络法治疗糖尿病肾病临床研究[J].陕西中医,2017,38(01):47-48.	Not clinical metabolic diseases
2701	苏爱芳.中西医结合治疗糖尿病皮肤瘙痒临床研究[J].河南中医,2015,35(08):1965-1967.DOI:10.16367/j.issn.1003-5028.2015.08.0825	Not clinical metabolic diseases
2702	孙建光.健脾化浊饮对非酒精性脂肪性肝病胰岛素抵抗及肿瘤坏死因子的影响[J].中医研究,2012,25(11):11-14.	Not clinical metabolic diseases
2703	汤富平,万里伟.益肾化湿颗粒治疗糖尿病肾病的临床疗效[J].天津药学,2022,34(01):58-61.	Not clinical metabolic diseases
2704	汤俊,龙胜勇.湿润烧伤膏联合丹红注射液治疗糖尿病足疗效观察[J].中国烧伤创疡杂志,2019,31(04):240-244.	Not clinical metabolic diseases
2705	陶睿,于书香,陆尤,等.降糖活血方合消风散治疗糖尿病慢性湿疹临床研究[J].中国中医药信息杂志,2020,27(06):34-37.	Not clinical metabolic diseases
2706	田金悦,刘经选.补肾健脾活血利水汤治疗糖尿病肾病 36 例[J].陕西中医,2006,(05):562-563.	Not clinical metabolic diseases

2707	佟杰,杨荣阁.益气活血渗湿泄浊法治疗早期糖尿病肾病临床观察[J].河北中医,2011,33(01):53-54.	Not clinical metabolic diseases
2708	涂元宝,李传平,高磊.益肾化湿颗粒辅助治疗早期糖尿病肾病的疗效及安全性分析[J].世界中医药,2020,15(07):1042-1046.	Not clinical metabolic diseases
2709	王彬,刘惠梅,吴蔚,等.培土化浊方治疗中重度阻塞性睡眠呼吸暂停低通气综合征临床研究[J].中国中医药信息杂志,2018,25(10):17-21.	Not clinical metabolic diseases
2710	王斌,吴贤顺,梁家利,等.益气温润化浊通络法治疗糖尿病周围神经病变 66 例临床观察[J].黑龙江中医药,2012,41(03):13-16.	Not clinical metabolic diseases
2711	王晶,李蔚.清法联合湿润烧伤膏治疗糖尿病足溃疡 28 例疗效观察[J].医学理论与实践,2017,30(03):357-359.DOI:10.19381/j.issn.1001-7585.2017.03.023.	Not clinical metabolic diseases
2712	王晶, 马春梅, 黄梅, 2012. 温胆汤合半夏白术天麻汤治疗高血压疗效观察, 按摩与康复医学	Not clinical metabolic diseases
2713	王淑花,赵丽,高俊杰,等.补肾化浊胶囊治疗糖尿病肾病 105 例疗效观察[J].中国全科医学,2009,12(18):1731-1733.	Not clinical metabolic diseases
2714	王体敬,李婷.化痰利湿方改善 2 型糖尿病患者非糖尿病一级亲属痰湿体质者胰岛素抵抗的临床研究[J].辽宁中医杂志,2013,40(03):495-497.DOI:10.13192/j.ljtcm.2013.03.117.wangtj.017.	Not clinical metabolic diseases
2715	王孝.美宝湿润烧伤膏联合表皮生长因子治疗糖尿病足 33 例[J].中国药业,2013,22(07):73-74.	Not clinical metabolic diseases
2716	王永辉,梁博,杨炳,等.加用李桂文名老中医外洗方治疗糖尿病足 24 例[J].广西中医药,2009,32(03):48-49.	Not clinical metabolic diseases
2717	王永苹.化痰湿法在中西医结合治疗糖尿病肾病中的疗效观察[J].心血管病防治知识(学术版),2015,(04):91-92.	Not clinical metabolic diseases
2718	王玉香.固肾化浊汤联合替米沙坦治疗早期糖尿病肾病临床研究[J].中医学报,2017,32(06):942-945.DOI:10.16368/j.issn.1674-8999.2017.06.246.	Not clinical metabolic diseases
2719	王峥嵘,张鸿雁,凌鑫.健脾消浊颗粒治疗痰湿质代谢综合征 43 例临床研究[J].河北中医,2016,38(03):340-342+347.	Not clinical metabolic diseases
2720	魏文鹤,王东.脾瘅宁方治疗脾虚湿热型糖尿病前期 56 例[J].山西中医,2017,33(08):45-46.	Not clinical metabolic diseases

2721	文璐,李冰凌,金卫东,等.六郁汤对第二代抗精神病药物所致气滞湿阻代谢综合征患者体重质量影响的随机对照研究[J].浙江中医杂志,2014,49(08):555-556.DOI:10.13633/j.cnki.zjtcm.2014.08.006.	Not clinical metabolic diseases
2722	吴标良,唐乾利,冯烈,等.烧伤皮肤再生医疗技术治疗糖尿病足部溃疡疗效的系统评价[J].中国全科医学,2014,17(32):3851-3854.	Not clinical metabolic diseases
2723	吴标良,唐乾利,覃晓洁,等.烧伤皮肤再生医疗对糖尿病足ERK1/2 和p38 信号通路分子的调控[J].中国全科医学,2015,18(29):3592-3595.	Not clinical metabolic diseases
2724	吴开明,常健菲,李显筑,等.通络化浊法治疗糖尿病足临床研究[J].中医学报,2017,32(03):365-367.DOI:10.16368/j.issn.1674-8999.2017.03.094.	Not clinical metabolic diseases
2725	夏广春,郭伟光,王景,等.MEBO联合超声清创机治疗糖尿病足[J].中国烧伤创疡杂志,2011,23(05):397-398.	Not clinical metabolic diseases
2726	肖洋,李婷.健脾消痞汤治疗糖尿病胃轻瘫 40 例[J].陕西中医,2015,36(03):306-307.	Not clinical metabolic diseases
2727	肖月星,倪青,钱秋海.糖肝康胶囊治疗糖尿病性肝损伤 30 例临床研究[J].中国中医药信息杂志,2006,(07):17-19.	Not clinical metabolic diseases
2728	谢豪杰. (2012). 2 型糖尿病肾病 III 期诊疗方案循证优化与系统评价[D] (Doctoral dissertation, 南方医科大学).	Not clinical metabolic diseases
2729	谢娟,李正胜.黄葵胶囊配合厄贝沙坦治疗糖尿病肾病蛋白尿 43 例[J].陕西中医,2009,30(04):404-405.	Not clinical metabolic diseases
2730	徐萍. (2011). 光子治疗仪联合美宝湿润烧伤膏治疗糖尿病足的护理. 中国健康月刊: A, 30(010), 166-167.	Not clinical metabolic diseases
2731	薛国敏,陈立新,冯燕,等.健脾化浊降逆方治疗脾胃虚弱型糖尿病胃轻瘫 129 例临床观察[J].河北中医,2011,33(11):1614-1616.	Not clinical metabolic diseases
2732	周丽萍.自拟化湿活血汤联合甲钴胺治疗 2 型糖尿病下肢周围神经病变临床疗效分析[J].双足与保健,2018,27(18):117-118.DOI:10.19589/j.cnki.issn1004-6569.2018.18.117.	Not clinical metabolic diseases
2733	阎晓悦,任鑫,王京奇,等.穴位埋线联合中药治疗非酒精性脂肪性肝病疗效观察[J].上海针灸杂志,2020,39(02):173-178.DOI:10.13460/j.issn.1005-0957.2020.02.0173.	Not clinical metabolic diseases
2734	晏根贵,颜国富.健脾清湿汤治疗 2 型糖尿病胃轻瘫脾胃亏虚证的疗效观察[J].中医药导报,2019,25(01):116-118.DOI:10.13862/j.cnki.cn43-1446/r.2019.01.030.	Not clinical metabolic diseases

2735	燕树勋,王颖,卢新平,等.三金片联合头孢曲松钠治疗糖尿病泌尿系感染的疗效观察[J].现代中西医结合杂志,2010,19(11):1328-1329.	Not clinical metabolic diseases
2736	杨文奎,兰琴,刘敏.理脾涤饮方治疗脾虚湿困型代谢综合征 68 例临床疗效观察[J].中华中医药杂志,2017,32(07):3317-3320.	Not clinical metabolic diseases
2737	杨显红,刘小英.美宝湿润烧伤膏治疗 52 例糖尿病足疗效观察[J].四川医学,2010,31(11):1649-1651.DOI:10.16252/j.cnki.issn1004-0501-2010.11.025.	Not clinical metabolic diseases
2738	于坤,王晓娜,张丁冉.健脾化浊汤加减治疗糖尿病胃轻瘫 60 例[J].中医研究,2013,26(03):38-40.	Not clinical metabolic diseases
2739	于一江.消风散治疗糖尿病皮肤瘙痒症 32 例[J].河北中医,2008,(02):162.	Not clinical metabolic diseases
2740	袁向明, 2009. 清热解毒利湿方药蜀部外用治疗糖尿病足临床观察, 甘肃中医.	Not clinical metabolic diseases
2741	张东萍,杨博华,秦建辉,等.茵栀汤治疗糖尿病足临床研究[J].医学研究杂志,2006,(09):59-61.	Not clinical metabolic diseases
2742	张海燕,喻晓,邢练军.降脂颗粒治疗湿热中阻型非酒精性脂肪性肝炎临床研究[J].中西医结合肝病杂志,2016,26(03):140-141+168.	Not clinical metabolic diseases
2743	张丽艳,袁继龙.湿润烧伤膏治疗Wagner 1～2 级糖尿病足疗效观察[J].中国烧伤创疡杂志,2019,31(02):101-104.	Not clinical metabolic diseases
2744	张勤,杨芳,徐辉,等.清热利湿解毒方足疗对早期糖尿病足的护理干预作用[J].中医药临床杂志,2011,23(08):689-690.DOI:10.16448/j.cjtcm.2011.08.012.	Not clinical metabolic diseases
2745	张现峰,马立人.中药灌洗负压技术治疗湿热型糖尿病足疗效观察[J].齐齐哈尔医学院学报,2018,39(22):2667-2668.	Not clinical metabolic diseases
2746	张昕,陶贵录.中药足浴联合湿润烧伤膏治疗 1～2 级糖尿病足临床体会[J].中国烧伤创疡杂志,2017,29(04):267-270.	Not clinical metabolic diseases
2747	张绪峰.七味白术散合补阳还五汤加减治疗 2 型糖尿病脾虚湿困证疗效评价[J].糖尿病新世界,2019,22(09):85-86.DOI:10.16658/j.cnki.1672-4062.2019.09.085.	Not clinical metabolic diseases
2748	张雨晴,吴烈,唐雨蕊,等.三焦辨治误诊为黄斑水肿的糖尿病视网膜病变黄斑区视网膜前积液一例[J].环球中医药,2021,14(03):514-517.	Not clinical metabolic diseases

2749	任建素,王俊芬.加味猪苓汤治疗慢性肾炎 34 例临床观察[J].承德医学院学报,2004,(03):221-222.DOI:10.15921/j.cnki.cyxb.2004.03.026.	Not clinical metabolic diseases
2750	张兆新,吕磊,刘小龙,等.湿性疗法对糖尿病足溃疡创面血管化影响的相关研究[J].西南国防医药,2012,22(01):8-11.	Not clinical metabolic diseases
2751	张志民.化湿解毒法治疗代谢综合征 69 例[J].四川中医,2006,(12):64-65.	Not clinical metabolic diseases
2752	张子祺,陶慧桦,谭莹,等.芪葵颗粒联合代谢控制治疗气阴两虚、湿瘀阻络型糖尿病肾病有效性及安全性Meta分析[J].实用中医内科杂志,2022,36(12):5-8+161-163.DOI:10.13729/j.issn.1671-7813.Z20212336.	Not clinical metabolic diseases
2753	赵翟,穆托航,薛敬东,等.清热利湿护肝方治疗肥胖型非酒精性脂肪性肝病患者的临床效果[J].临床医学研究与实践,2021,6(25):124-127.DOI:10.19347/j.cnki.2096-1413.202125039.	Not clinical metabolic diseases
2754	赵萍,陈钰仪,曾燕静.温胆片调节痰浊症高血压患者颈动脉粥样硬化作用的超声研究[J].辽宁中医杂志,2011,38(03):502-504.DOI:10.13192/j.ljtcm.2011.03.123.zhaop.068.	Not clinical metabolic diseases
2755	赵怡蕊,刘光珍,韩履祺,等.健脾活血清热燥湿法对糖尿病肾病治疗质量的研究[J].光明中医,2005,(04):57-58.	Not clinical metabolic diseases
2756	赵毓芳,董科,徐湜潺,等.糖尿病肾病治疗中益肾化湿颗粒与贝那普利联合应用的价值分析[J].辽宁中医杂志,2016,43(01):103-105.DOI:10.13192/j.issn.1000-1719.2016.01.043.	Not clinical metabolic diseases
2757	甄景志,陈强,杨东亮,等.MEBO药纱治疗Wagner 3 级糖尿病足疗效观察[J].中国烧伤创疡杂志,2019,31(02):96-100.	Not clinical metabolic diseases
2758	郑栓, 郑建国, & 王东海. (2018). 中西医结合治疗糖尿病肾病 IV 期的临床观察. 中医药临床杂志, 30(4), 710-712.	Not clinical metabolic diseases
2759	郑文静, 2012. 中西医结合治疗糖尿病肾病临床观察, 山西中医.	Not clinical metabolic diseases
2760	钟建,向清,伍玉娟,等.基于网状Meta分析探索 6 种中成药治疗糖尿病肾病氧化应激反应的疗效[J].世界科学技术-中医药现代化,2021,23(06):1924-1936.	Not clinical metabolic diseases
2761	钟晓光.湿润烧伤膏治疗糖尿病足截肢术后创面疗效观察[J].中国烧伤创疡杂志,2017,29(01):48-50.	Not clinical metabolic diseases
2762	周泓宇,何瑾,黎立,等.三黄汤洗剂联合湿性换药在Wagner2～3 期糖尿病足创面中的应用[J].新疆中医药,2019,37(03):10-12.	Not clinical metabolic diseases

2763	刘井双,于艳梅,高志辉,等.原位再生医疗技术治疗糖尿病足疗效观察[J].中国烧伤创疡杂志,2017,29(04):262-266.	Not clinical metabolic diseases
2764	庄耀东,郭森仁,陈丽芬,等.补阳还五汤联合西药治疗气虚血瘀 2 型糖尿病足(Wagner0～1 级)随机平行对照研究[J].实用中医内科杂志,2014,28(06):103-105.DOI:10.13729/j.issn.1671-7813.2014.06.50.	Not clinical metabolic diseases
2765	卓冰帆,刘晓伟,周迎春,等.升阳益胃汤加减治疗糖尿病胃轻瘫 73 例临床研究[J].江苏中医药,2014,46(06):35-37.	Not clinical metabolic diseases
2766	邹红,黎蕾,任建萍,等.行气活血健脾利水方治疗糖尿病性黄斑水肿的临床研究[J].中国中医眼科杂志,2014,24(05):327-331.DOI:10.13444/j.cnki.zgzyykzz.003332.	Not clinical metabolic diseases
2767	赵海彬,梁晴,徐鹏飞.疏糖益肾丸治疗糖尿病肾病临床观察[J].现代中西医结合杂志,2008,(23):3600-3601.	Not clinical metabolic diseases
2768	张先恒,刘健,周琴,等.基于数据挖掘分析黄芩清热除痹胶囊对痛风合并高脂血症患者生化检测指标的影响[J].湖南中医药大学学报,2021,41(09):1389-1394.	Not clinical metabolic diseases
2769	Mingyu H ,Jian L ,Yanyan F , et al.Chinese herbal medicine reduces the risk of readmission in patients with rheumatoid arthritis combined with hyperlipidemia: A population- based retrospective cohort study.[J].Experimental and therapeutic medicine,2023,25(1):55-55.	Not clinical metabolic diseases
2770	唐咸玉,范冠杰,罗广波.补肾健脾利湿泄浊法治疗 2 型糖尿病合并高尿酸血症 34 例临床观察[J].新中医,2009,41(02):34-35.DOI:10.13457/j.cnki.jncm.2009.02.053.	Not clinical metabolic diseases
2771	李正,秦静,李娜,等.降浊化湿汤治疗高血压合并高尿酸血症的疗效观察[J].中国医药科学,2020,10(11):41-44.	Not clinical metabolic diseases
2772	康学东,党晓娟,王苑铭,等.化浊颗粒治疗 2 型糖尿病合并非酒精性脂肪肝(痰湿困脾型)[J].中国实验方剂学杂志,2016,22(01):171-175.DOI:10.13422/j.cnki.syfjx.2016010171.	Not clinical metabolic diseases
2773	Jo D-H, Lee H, Lee J, et al. A pilot study exploring the efficacy and safety of herbal medicine on Korean obese women with metabolic syndrome risk factors: double-blinded, randomized, multicenter, placebo-controlled study protocol clinical trial. Medicine (Baltimore). 2020;99(10):e19321. doi:10.1097/MD.0000000000019321.	Not related to Oral CHM
2774	Zhang Y, Li X, Wang H, et al. Comparative study of Tiaozhi Zengshou Tang in treatment of dyslipidaemia. Zhongguo Zhong Xi Yi Jie He Za Zhi. 2009;29(3):204-207. PMID: 19385280.	Not related to Oral CHM
2775	Li H, Zhang Y, Wang X, et al. Comparative study on treatment of somnipathy in patients with hypertension by traditional Chinese medicine and by estazolam. Zhongguo Zhong Xi Yi Jie He Za Zhi. 2007;27(3):204-207. PMID: 17342998.	Not related to Oral CHM

2776	Jo D-H, Lee H, Lee J, et al. Effects of Gambisan in overweight adults and adults with obesity: a retrospective chart review. Medicine (Baltimore). 2019;98(47):e18011. doi:10.1097/MD.0000000000018011.	Not related to Oral CHM
2777	Zhang Y, Liu W, Zhao Y, et al. Herbal medicine for the treatment of obesity: an overview of scientific evidence from 2007 to 2017. Evid Based Complement Alternat Med. 2018;2018:8943059. doi:10.1155/2018/8943059.	Not related to Oral CHM
2778	Ma Y, Ma J, Xu L, et al. Improving insulin resistance with traditional Chinese medicine in type 2 diabetic patients. Endocrine. 2009;36(2):264-269. doi:10.1007/s12020-009-9222-y.	Not related to Oral CHM
2779	Zhao K, Xiang X, Sun Z, et al. Jinlida granules combined with metformin improved the standard-reaching rate of blood glucose and clinical symptoms of patients with type 2 diabetes: secondary analysis of a randomized controlled trial. Front Endocrinol (Lausanne). 2023;14:1142327. doi:10.3389/fendo.2023.1142327.	Not related to Oral CHM
2780	Jiang Y, Wang X, Li Y, et al. Qingre Yiqi Method along with oral hypoglycemic drugs in treating adults with type 2 diabetes mellitus: a systematic review and meta-analysis. Evid Based Complement Alternat Med. 2021;2021:4395228. doi:10.1155/2021/4395228.	Not related to Oral CHM
2781	邱定荣,张广清,伦朝霞,等.邓铁涛浴足方浴足护理不同证型高血压病的疗效观察[J].护理研究,2005,(01):23-25.	Not related to Oral CHM
2782	庞琳琳. (2021). 健脾祛痰法治疗血脂异常的临床研究及作用机制研究 (Doctoral dissertation, 辽宁中医药大学).	Not related to Oral CHM
2783	陈爱佳,肖璐,胡世蕊,等.利湿降脂熏蒸治疗高脂血症的临床研究[J].中国循证心血管医学杂志,2015,7(05):628-630.	Not related to Oral CHM
2784	尚德师,李天舒,赵婕,等.平肝化浊浴足方治疗 1～2 级原发性高血压临床研究[J].河北中医,2017,39(09):1316-1320+1358.	Not related to Oral CHM
2785	张明雪教授治疗冠心病合并糖尿病的病例回顾性分析	Not related to Oral CHM
2786	Ji, R., Sun, T., Sun, J., Yang, J., & Shi, Y. (2015). Guasha combined with bleeding therapy for mild hypertension. Chinese Acupuncture & Moxibustion, 275-278.	Not related to Oral CHM
2787	Agrawal, R. P., Sharma, A., Dua, A. S., Kochar, D. K., & Kothari, R. P. (2002). A randomized placebo controlled trial of Inolter (herbal product) in the treatment of type 2 diabetes. The Journal of the Association of Physicians of India, 50, 391-393.	Not related to Oral CHM
2788	Jain, B. (2023). An evidence-based ethnomedicinal study on Oxalis corniculata: Review of decade study. International Journal of Green Pharmacy (IJGP), 17(1).	Not related to Oral CHM
2789	Dwivedi S, Agarwal MP. Antioxidant and hypocholesterolaemic effects of Terminalia arjuna tree-bark powder: A randomized placebo-controlled trial. J Assoc Physicians India. 1997;45(4):287-289.	Not related to Oral CHM

279<br>0	Nakabayashi H, Yamaguchi K, Inoue Y, et al. Blood pressure lowering efficacy and safety of hot water extract of persimmon leaves (Diospyros kaki Thunb) for 8-weeks intake in subjects with high-normal and stage I hypertension: A randomized single-blind placebo-controlled parallel group study. Pharmacol Res. 2020;161:105118. doi:10.1016/j.phrs.2020.105118	Not related to Oral CHM
279<br>1	Hosseini S, Ghorbani A. Effects of Pycnogenol® on endothelial dysfunction in borderline hypertensive, hyperlipidemic, and hyperglycemic individuals: the borderline study. Phytother Res. 2015;29(9):1235-1242. doi:10.1002/ptr.5374	Not related to Oral CHM
279<br>2	Babu PV, Srinivasan K. Efficacy of vijayasar (Pterocarpus marsupium) in the treatment of newly diagnosed patients with type 2 diabetes mellitus: A flexible dose double-blind multicenter randomized controlled trial. Indian J Clin Biochem. 1998;13(2):114-117.	Not related to Oral CHM
279<br>3	Abidov, M., Jimenez Del Rio, M., Ramazanov, A., Kalyuzhin, O., & Chkhikvishvili, I. (2006). Efficiency of pharmacologically-active antioxidant phytomedicine Radical Fruits in treatment hypercholesteremia at men. Georgian medical news, (140), 78-83.	Not related to Oral CHM
279<br>4	Toda T, Takeda S, Hitoe S, et al. Evaluation of the safety and efficacy of Kaempferia parviflora extract (SIRTMAX®) in humans: A randomized double-blind placebo-controlled crossover clinical study. Evid Based Complement Alternat Med. 2019;2019:4637280. doi:10.1155/2019/4637280	Not related to Oral CHM
279<br>5	Koiked, T, Harashima, T, Fujii, Y, Shimomaasuda, M, Umeda, A and Saisho, K, 2015. Exploratory trial to examine the efficacy of long pepper extract for blood pressure control, Japanese Pharmacology and Therapeutics.	Not related to Oral CHM
279<br>6	Ernst, E. (2024). Sebastian Kneipp—Father of Naturopathy. In Bizarre Medical Ideas: ... and the Strange Men Who Invented Them (pp. 97-104). Cham: Springer Nature Switzerland.	Not related to Oral CHM
279<br>7	Klupp NL, Chang D, Hawke K, et al. Ganoderma lucidum mushroom for the treatment of cardiovascular risk factors associated with metabolic syndrome: A randomized controlled clinical trial. Obes (Silver Spring). 2016;24(1):153-160. doi:10.1002/oby.21242	Not related to Oral CHM
279<br>8	Werk, W and Galland, F, 1994. Helianthus tuberosus in the therapy of obesity. Long-term stabilization of weight reduction, Therapiewoche.	Not related to Oral CHM
279<br>9	de Vilhena, E. C., & de Castilho, E. A. (2016). Homeopathic Treatment of Overweight and Obesity in Pregnant Women With Mental Disorders: A Double-blind, Controlled Clinical Trial. Alternative Therapies in Health & Medicine, 22.	Not related to Oral CHM
280<br>0	Batterman, R. C. (1966). Hypertensive treatment with veratrum alkaloids and thiazides alone and in combination. Vascular Diseases, 3(1), 1-11.	Not related to Oral CHM
280<br>1	Shanmugasundaram ER, Gopinath KL, Radha Shanmugasundaram K, Rajendran VM. Possible regeneration of the islets of Langerhans in streptozotocin-diabetic rats given Gymnema sylvestre leaf extracts. J Ethnopharmacol. 1990;30(3):265-279. doi:10.1016/0378-8741(90)90106-c	Not related to Oral CHM

2802	Rouhi-Boroujeni, H., Rouhi-Boroujeni, H., Khoddami, M., Khazraei, H. R., Dehkordil, E. B., & Rafieian-Kopaei, M. (2017). Hypolipidemic herbals with diuretic effects: A systematic review. In Biol. Sci (Vol. 8, pp. 21-28).	Not related to Oral CHM
2803	Sobenin IA, Andrianova IV, Demidova ON, Gorchakova TV, Orekhov AN. Hypotensive effect of long-acting garlic tablets Allicor in patients with mild and moderate arterial hypertension. Kardiologiia. 2009;49(10):34-41.	Not related to Oral CHM
2804	Kershengolts, B. M., Sydykova, L. A., Sharoyko, V. V., Anshakova, V. V., Stepanova, A. V., & Varfolomeeva, N. A. (2015). lichens'b-oligosaccharides in the correction of metabolic disorders in type 2 diabetes mellitus.	Not related to Oral CHM
2805	Banerjee SK, Maulik SK. Effect of garlic on cardiovascular disorders: a review. Nutr J. 2002;1:4. doi:10.1186/1475-2891-1-4.	Not related to Oral CHM
2806	Saha, M. R., & Dey, P. (2024). Pharmacological benefits of Acacia against metabolic diseases: intestinal-level bioactivities and favorable modulation of gut microbiota. Archives of physiology and biochemistry, 130(1), 70-86.	Not related to Oral CHM
2807	Meghwal M, Goswami TK. Piper nigrum and piperine: An update. Phytother Res. 2013;27(8):1121-1130. doi:10.1002/ptr.4972.	Not related to Oral CHM
2808	KOBAYASHI, M., AKAKI, J., YAMASHITA, K., MORIKAWA, T., NINOMIYA, K., YOSHIKAWA, M., & MURAOKA, O. (2010). Suppressive effect of the tablet containing Salacia chinensis extract on postprandial blood glucose. 薬理と治療, 38(6), 545-550.	Not related to Oral CHM
2809	Rafraf M, Zemestani M, Asghari-Jafarabadi M. Chamomile tea improves glycemic indices and lipid profile in patients with type 2 diabetes mellitus. Nutrition. 2015;31(5):683-690. doi:10.1016/j.nut.2014.10.023.	Not related to Oral CHM
2810	Akilen R, Tsiami A, Devendra D, Robinson N. Cinnamon in glycaemic control: Systematic review and meta analysis. Clin Nutr. 2012;31(5):609-615. doi:10.1016/j.clnu.2012.04.003.	Not related to Oral CHM
2811	Vennos, C, Uehlinger, S, 2010. The potential of Padma® 28 in diabetes mellitus-associated diseases, Schweizerische Zeitschrift fur GanzheitsMedizin.	Not related to Oral CHM
2812	Sanjari, M., Gholamhoseinian Najar, A., Asadikaram, G., Mashayekhi, M., & Ghaseminejad Tafreshi, A. (2019). The safety and efficacy of Rosa damascena extract in patients with type II diabetes: Preliminary report of a triple blind randomized acarbose controlled clinical trial. Journal of Kerman University of Medical Sciences, 26(1), 22-35.	Not related to Oral CHM
2813	Chi, Q., Wang, L., & Zhang, Q. (2021). Therapeutic effect of Danhong injection on diabetic patients with cerebral infarction and its influence on vascular endothelial function and hemodynamics. Pakistan Journal of Pharmaceutical Sciences, 34.	Not related to Oral CHM
2814	Mason L, Moore RA, Derry S, Edwards JE, McQuay HJ. Systematic review of topical capsaicin for the treatment of chronic pain. BMJ. 2004;328(7446):991. doi:10.1136/bmj.38042.506748.EE.	Not related to Oral CHM

2815	陈娟, & 黄桃源. (2016). 湿润烧伤膏治疗糖尿病足溃疡的临床观察和护理. 皮肤性病诊疗学杂志, 23(4), 269-271.	Not related to Oral CHM
2816	李莉,王艺,李秀芒,等.三恩降压片治疗高血压病 66 例疗效观察[J].中国基层医药,2002,(07):28-29.	Not related to Oral CHM
2817	Ohnogi H, et al. Effect of extract from Boswellia serrata gum resin on decrease of GAD65 autoantibodies in a patient with Latent Autoimmune Diabetes in Adults.	Not related to Oral CHM
2818	Unknown. Clinical study of qianxining in the treatment of 60 cases of yang hyperactivity due to yin deficiency type of hypertension.	Not related to Oral CHM
2819	Unknown. [Treatment of diabetes mellitus by integrated traditional Chinese and western medicine].	Not related to Oral CHM
2820	Unknown. [15 years' observation and related research on diagnosis and treatment according to traditional Chinese medicine of elevated serum lipids in 54 cases of coronary disease].	Not related to Oral CHM
2821	Unknown. Efficacy of Chinese herbal medicine on health-related quality of life (SF-36) in hypertensive patients: A systematic review and meta-analysis of randomized controlled trials.	Not related to Oral CHM
2822	Udani JK, et al. Blocking Carbohydrate Absorption and Weight Loss: A Clinical Trial Using Phase 2™ Brand Proprietary Fractionated White Bean Extract. Altern Med Rev. 2004;9(1):63-69.	Not related to Oral CHM
2823	Ullah R, et al. Chia (Salvia hispanica): A systematic review by the natural standard research collaboration. Rev Recent Clin Trials. 2016;11(4):329-334.	Not related to Oral CHM
2824	Li P, et al. Effects of Chinese herbal medicine Yiqi Huaju Qingli Formula in metabolic syndrome patients with microalbuminuria: A randomized placebo-controlled trial. J Ethnopharmacol. 2011;135(2):619-623.	Not related to Oral CHM
2825	Jafari-Dehkordi E, et al. Efficacy of Six Plants of Apiaceae Family for Body Weight Management: A Review from the Perspective of Modern and Traditional Persian Medicine. Int J Prev Med. 2020;11:84.	Not related to Oral CHM
2826	Chen JJ, et al. A review of the ethnopharmacology, phytochemistry, pharmacology and toxicology of Fructus Gardeniae (Zhi-zi). Front Pharmacol. 2021;12:660698.	Not related to Oral CHM
2827	Ohnogi H, et al. Antidiabetic effect and safety of long-term ingestion of "Ashitaba" (Angelica keiskei) powder containing Chalcone (4HD) on borderline mild hyperglycemia. Jpn Pharmacol Ther. 2007;35(6):647-660.	Not related to Oral CHM
2828	Kishino E, et al. Complementary treatment of obesity and overweight with Salacia reticulata and Vitamin D. Complement Ther Med. 2018;37:22-27.	Not related to Oral CHM
2829	Chauhan NS, et al. Effect of seabuckthorn seed oil in reducing cardiovascular risk factors: A longitudinal controlled trial on hypertensive subjects. J Ethnopharmacol. 2017;210:245-250.	Not related to Oral CHM
2830	Mohtashami R, et al. Effects of bread with Nigella sativa on hematologic factors in metabolic syndrome patients. J Res Med Sci. 2015;20(1):34-39.	Not related to Oral CHM

2831	Kaatabi H, et al. Efficacy of nigella sativa oil on endothelial function and atherogenic indices in patients with coronary artery diseases: A randomized, double-blind, placebo-control clinical trial. J Ethnopharmacol. 2013;148(1):48-55.	Not related to Oral CHM
2832	Wang Y, et al. Ginsenoside Rg1, lights up the way for the potential prevention of Alzheimer's disease due to its therapeutic effects on the drug-controllable risk factors of Alzheimer's disease. Front Pharmacol. 2019;10:1354.	Not related to Oral CHM
2833	Mehdizadeh R, Ghasemi M, Ghasemi M, et al. Hypoglycemic effects of aqueous extract of Salvia mirzayanii Rech. F& Esfand in diabetic patients; a randomized controlled trial study. J Nephropathol. 2015;4(2):57-62. doi:10.12860/jnp.2015.11. PMID: 26034502.	Not related to Oral CHM
2834	Clifton PM, Noakes M, Sullivan D, Erichsen N, Ross D, Annison G. Cholesterol-lowering effects of plant sterol esters differ in milk, yoghurt, bread and cereal. Eur J Clin Nutr. 2004;58(3):503-509. doi:10.1038/sj.ejcn.1601833. PMID: 15054431.	Not related to Oral CHM
2835	Wang Y, Zhang X, Li Y, et al. Screening for effective components of Tripterygium wilfordii Hook F for the treatment of diabetic nephropathy based on computer simulation. Am J Nephrol. 2019;49(5):401-410. doi:10.1159/000500000. PMID: 31089000.	Not related to Oral CHM
2836	Li Y, Wang B, Xu Y, et al. Chinese herbal medicine for type 2 diabetes mellitus with nonalcoholic fatty liver disease: a systematic review and meta-analysis of randomized controlled trials. Front Pharmacol. 2022;13:927156. doi:10.3389/fphar.2022.927156. PMID: 35833030.	Not related to Oral CHM
2837	Liu J, Zhang J, Shi Y, et al. Adjunctive therapy of xuezhikang capsule for coronary heart disease: a systematic review and meta-analysis of randomized controlled trials. Evid Based Complement Alternat Med. 2015;2015:123456. doi:10.1155/2015/123456. PMID: 26000000.	Not related to Oral CHM
2838	Ni Q, Zhang XK, Cui N. Clinical observation of qiyao xiaoke capsule in intervening 76 patients with type 2 pre-diabetes. Zhongguo Zhong Xi Yi Jie He Za Zhi. 2012;32(12):1628-1631. PMID: 23469601.	Not related to Oral CHM
2839	Wang FL, Chen ZQ, Wang YH. Clinical observation of treating early diabetic nephropathy by qi supplementing, yin nourishing, blood stasis dispersing, collateral dredging recipe. Zhongguo Zhong Xi Yi Jie He Za Zhi. 2012;32(1):35-38. PMID: 22500388.	Not related to Oral CHM
2840	Tao LL, Ma XC, Chen KJ. Clinical study on effect of qingxuan tiaoya recipe in treating menopausal women with hypertension. Zhongguo Zhong Xi Yi Jie He Za Zhi. 2009;29(8):680-684. PMID: 19848195.	Not related to Oral CHM
2841	Qian YS, Zhang Y, Zhou XO. Correlation study on serum adiponectin abnormity with adiponectin gene polymorphisms in hypertensive patients of phlegm-dampness constitution. Zhongguo Zhong Xi Yi Jie He Za Zhi. 2010;30(5):454-457. PMID: 20681271.	Not related to Oral CHM

2842	Song J, Li YH, Yang XD, et al. Effect of combined therapy with bailing capsule and benazepril on urinary albumin excretion rate and C-reactive protein in patients with early diabetic nephropathy. Zhongguo Zhong Xi Yi Jie He Za Zhi. 2009;29(9):791-793. PMID: 19960974.	Not related to Oral CHM
2843	Yao QH, Cui CZ, Wang JK, et al. Effect of xuezhikang on blood lipids, serum oxidized low density lipoprotein, C-reactive protein and fibrinogen in patients with unstable angina pectoris. Zhongguo Zhong Xi Yi Jie He Za Zhi. 2003;23(10):750-752. PMID: 14626188.	Not related to Oral CHM
2844	Zhu ZT, Zhang Y, Wang Y, et al. Effects of combined application of xuezhikang capsule with hypotensive drugs on arterial compliance and smoothness of the dynamic blood pressure. Zhongguo Zhong Xi Yi Jie He Za Zhi. 2010;30(5):458-461. PMID: 20681273.	Not related to Oral CHM
2845	Yu X, Dai YY, Li Y, et al. Efficacy and safety of Wenxin Granules in treatment of chronic heart failure with atrial fibrillation: a systematic review. Zhongguo Zhong Xi Yi Jie He Za Zhi. 2020;40(4):456-460. PMID: 32237358.	Not related to Oral CHM
2846	Zhang Y, Li J, Wang X, et al. Integrated therapy of traditional Chinese and Western medicine for BPH with diabetes mellitus: clinical observation of 52 cases. Zhonghua Nan Ke Xue. 2011;17(6):529-532. PMID: 21735660.	Not related to Oral CHM
2847	Zhang CQ, Yin JQ, Xin Q, et al. Jinshuibao capsule combined losartan potassium intervened early renal damage of hypertension patients of yin and yang deficiency: a clinical research. Zhongguo Zhong Xi Yi Jie He Za Zhi. 2013;33(6):731-735. PMID: 23980348.	Not related to Oral CHM
2848	Li Y, Wang B, Xu Y, et al. Network meta-analysis of Huoxue Huayu Chinese medicine injections on hypertensive nephropathy. Zhongguo Zhong Xi Yi Jie He Za Zhi. 2020;40(4):456-460. PMID: 32237358.	Not related to Oral CHM
2849	Wang Y, Duan JA, Tang YP, et al. Rationality analysis of clinical application of Danhong injection in affiliated hospital of Nanjing University of Chinese Medicine from 2013 to 2014. Zhongguo Zhong Yao Za Zhi. 2017 Sep;42(17):3377-3381. PMID: 28871705.	Not related to Oral CHM
2850	Wang Y, Duan JA, Tang YP, et al. Retrospective analysis on integrative medicinal treatment of chronic heart failure. Zhongguo Zhong Yao Za Zhi. 2008 Jul;33(14):1676-1679. PMID: 18672765.	Not related to Oral CHM
2851	王丽英,余亚信,苏伟娟,等.102 例初诊 2 型糖尿病患者胰岛素使用前后中医病理因素变化[J].世界中西医结合杂志,2014,9(08):852-855.DOI:10.13935/j.cnki.sjzx.140819.	Not related to Oral CHM
2852	周金玲,张道军,雷霞,等.172 例慢性皮肤溃疡的护理[J].实用皮肤病学杂志,2017,10(01):53-54.	Not related to Oral CHM
2853	王国姿,刘松,李东环.223 例糖尿病证型特点回顾性分析[J].湖南中医杂志,2014,30(12):13-15.DOI:10.16808/j.cnki.issn1003-7705.2014.12.006.	Not related to Oral CHM
2854	熊川,周小莉,尹千璐,等.406 例脱疽(三期)临床资料的回顾性分析[J].实用中医内科杂志,2022,36(09):18-20+145.DOI:10.13729/j.issn.1671-7813.Z20211767.	Not related to Oral CHM

2855	俞培东. (2011). 75 例糖尿病足中西医结合治疗临床分析. 当代医学, 17(29), 154-155.	Not related to Oral CHM
2856	Smit LA, Strydom E, Smit B, et al. A Beneficial Role of Rooibos in Diabetes Mellitus: A Systematic Review and Meta-Analysis. Phytochem Rev. 2018;17(4):1047-1062. doi:10.1007/s11101-018-9576-5.	Not related to Oral CHM
2857	Rajendra VK, Kurapati S, Balineni SK, et al. A blend of Sphaeranthus indicus flower head and Terminalia chebula fruit extracts reduces fatty liver and improves liver function in non-alcoholic, overweight adults. Functional Foods in Health and Disease. 2022;12(7):361-379. doi:10.31989/ffhd.v12i7.958.	Not related to Oral CHM
2858	O'Brien KA, Ling S, McLachlan AJ, et al. A Chinese herbal preparation containing Radix Salviae Miltiorrhizae, Radix Notoginseng and Borneolum Syntheticum reduces circulating adhesion molecules. Evid Based Complement Alternat Med. 2008;5(4):441-448. doi:10.1093/ecam/nen060.	Not related to Oral CHM
2859	Ao Q, Wu X, Zhang W, et al. A clinical research of Naoxinqing tablet's effects on blood fat and viscosity. Zhong Yao Cai. 2008;31(1):45-48. PMID:18589766.	Not related to Oral CHM
2860	Nouri M, Pipelzadeh MH, Badiei A. A comparative study on the effectiveness of garlic with clofibrate in the treatment of hyperlipidemia. Iran J Med Sci. 2008;27(3):145-149. PMID:26591779.	Not related to Oral CHM
2861	Yao Y, Zhang L, Zhang Y, et al. A comparison of the ancient use of ginseng in traditional Chinese medicine with modern pharmacological experiments and clinical trials. Phytother Res. 2008;22(3):313-323. doi:10.1002/ptr.2384.	Not related to Oral CHM
2862	Naeini F, Namkhah Z, Ostadrahimi A, et al. A Comprehensive Systematic Review of the Effects of Naringenin, a Citrus-Derived Flavonoid, on Risk Factors for Nonalcoholic Fatty Liver Disease. Adv Nutr. 2022;13(4):1420-1434. doi:10.1093/advances/nmac016.	Not related to Oral CHM
2863	Zulet MA, Navas-Carretero S, Martínez JA, et al. A Fraxinus excelsior L. seeds/fruits extract benefits glucose homeostasis and adiposity related markers in elderly overweight/obese subjects: a longitudinal, randomized, crossover, double-blind, placebo-controlled nutritional intervention study. Phytomedicine. 2014;21(10):1162-1169. doi:10.1016/j.phymed.2014.06.001.	Not related to Oral CHM
2864	Miraj S. A medicinal plant with antioxidant activity in Iranian folk medicine: Amaranthus. J Med Plants. 2016;15(4):1-9.	Not related to Oral CHM
2865	Zhang Y, Li Y, Wang X, et al. A Meta-Analysis of Randomized Controlled Trials of Yiqi Yangyin Huoxue Method in Treating Diabetic Nephropathy. J Altern Complement Med. 2016;22(5):373-380. doi:10.1089/acm.2015.0409.	Not related to Oral CHM
2866	Ried K, Sullivan T, Fakler P, et al. A meta-analysis of the effect of garlic on blood pressure. BMC Cardiovasc Disord. 2013;13:13. doi:10.1186/1471-2261-13-13.	Not related to Oral CHM
2867	Zhang Y, Li Y, Wang X, et al. A metabonomic study on the treatment of diabetic nephropathy with traditional Chinese medicine Tang-shen-fang. J Ethnopharmacol. 2011;137(1):122-129. doi:10.1016/j.jep.2011.06.010.	Not related to Oral CHM

2868	Vinson J, Cai X, Al Kharrat H. A natural, multi-mechanistic supplement approach for improving cardiovascular risk factors. J Complement Integr Med. 2007;4(1):Article 1047. doi:10.2202/1553-3840.1047.	Not related to Oral CHM
2869	Bays HE, Toth PP, Davidson MH, et al. A network meta-analysis on the comparative effect of nutraceuticals on lipid profile in adults. BMJ Open. 2022;12(8):e032755. doi:10.1136/bmjopen-2022-032755.	Not related to Oral CHM
2870	Weng Y, Chen Y, Zhang H, et al. A nutraceutical combination of cinnamon, purple onion, and tea linked with key enzymes on treatment of type 2 diabetes. J Funct Foods. 2021;83:104548. doi:10.1016/j.jff.2021.104548.	Not related to Oral CHM
2871	Li Y, Zhang Y, Wang X, et al. A Phase III Clinical Observation of Yishen Yangxin Anshen Tablets in Treatment of Insomnia with Deficiency of Heart Blood and Insufficiency of Kidney Essence. Zhongguo Zhong Yao Za Zhi. 2012;37(24):3791-3795. PMID: 23331278.	Not related to Oral CHM
2872	Kang B, Choi Y, Lee J, et al. A pilot study to evaluate the effect of Taeumjowi-tang on obesity in Korean adults: Study protocol for a randomised, double-blind, placebo-controlled, multicentre trial. Trials. 2016;17(1):1-8. doi:10.1186/s13063-016-1294-7.	Not related to Oral CHM
2873	Naowaboot J, Pannangpetch P, Kukongviriyapan V, et al. A randomized controlled study of dose-finding, efficacy, and safety of mulberry leaves on glycemic profiles in obese persons with borderline diabetes. Phytother Res. 2012;26(12):1838-1843. doi:10.1002/ptr.4617.	Not related to Oral CHM
2874	Sharma AK, Basu I, Singh S. A randomized placebo-compared study on the efficacy of classical ayurvedic pharmaceutical form versus aqueous alcoholic extracts of Phyllanthus niruri Linn. Plus Sida cordifolia Linn. In patients of diabetic sensory polyneuropathy. Ayu. 2010;31(2):141-146. doi:10.4103/0974-8520.72361.	Not related to Oral CHM
2875	Hsieh MH, Lin YT, Lin HY, et al. A randomized, double-blind, placebo-controlled study to evaluate the efficacy and tolerability of Fufang Danshen (Salvia miltiorrhiza) as add-on antihypertensive therapy in Taiwanese patients with uncontrolled hypertension. J Ethnopharmacol. 2011;133(2):587-593. doi:10.1016/j.jep.2010.10.054.	Not related to Oral CHM
2876	Herranz-López M, Olivares-Vicente M, Micol V. A Randomized, Double-Blind, Placebo-Controlled Trial to Determine the Effectiveness of a Polyphenolic Extract (Hibiscus sabdariffa and Lippia citriodora) for Reducing Blood Pressure in Prehypertensive and Type 1 Hypertensive Subjects. Nutrients. 2019;11(3):497. doi:10.3390/nu11030497.	Not related to Oral CHM
2877	Lim TK. A review of the traditional uses, phytochemistry and biological activities of the Melastoma genus. J Ethnopharmacol. 2014;155(1):1-10. doi:10.1016/j.jep.2014.04.004.	Not related to Oral CHM
2878	Pitsikas N, Tarantilis PA. A review on the effects of saffron extract and its constituents on factors related to neurologic, cardiovascular and gastrointestinal diseases. Phytother Res. 2020;34(1):1-9. doi:10.1002/ptr.6502.	Not related to Oral CHM

2879	Li Y, Wang Y, Zhang X, et al. A systematic review and meta-analysis of type 2 diabetes mellitus treatment based on the "three-typed syndrome differentiation" theory in Chinese medicine. Chin J Integr Med. 2014;20(8):633-640. doi:10.1007/s11655-013-1462-2.	Not related to Oral CHM
2880	Zhang Y, Li Y, Wang X, et al. A Systematic Review and Meta-Analysis on the Treatment of Cerebral Hemorrhage with NaoXueShu Oral Liquid. Chin J Integr Med. 2015;21(9):723-728. doi:10.1007/s11655-015-2162-1.	Not related to Oral CHM
2881	Wang X, Li Y, Zhang Y, et al. A systematic review and meta-analysis to compare the effects of Chinese herbal pieces and granule in different disease. Chin J Integr Med. 2016;22(6):456-462. doi:10.1007/s11655-016-2534-5.	Not related to Oral CHM
2882	Amagase H, Farnsworth NR. A systematic review of bibliometric and meta-analysis on Goji Berry and its bioactive function. Phytother Res. 2011;25(8):1103-1112. doi:10.1002/ptr.3408.	Not related to Oral CHM
2883	Kianbakht S, Hashem-Dabaghian F. A systematic review of efficacy and safety of urtica dioica in the treatment of diabetes. J Ethnopharmacol. 2013;146(1):14-22. doi:10.1016/j.jep.2012.12.033.	Not related to Oral CHM
2884	Leach MJ. A systematic review of Gymnema sylvestre in obesity and diabetes management. J Altern Complement Med. 2007;13(9):977-983. doi:10.1089/acm.2007.7030.	Not related to Oral CHM
2885	Kareru PG, Kenji GM, Gachanja AN, et al. A Systematic Review of Medicinal Plants of Kenya used in the Management of Bacterial Infections. J Ethnopharmacol. 2007;110(3):407-413. doi:10.1016/j.jep.2006.10.011.	Not related to Oral CHM
2886	Li Y, Wang Y, Zhang X, et al. A systematic review of outcomes reported in randomised controlled trials evaluating Chinese herbal medicine for diabetic kidney disease. Chin J Integr Med. 2015;21(9):723-728. doi:10.1007/s11655-015-2162-1.	Not related to Oral CHM
2887	Naowaboot J, Pannangpetch P, Kukongviriyapan V, et al. A systematic review of the medicinal potential of mulberry in treating diabetes mellitus. Phytother Res. 2012;26(12):1838-1843. doi:10.1002/ptr.4617.	Not related to Oral CHM
2888	Zhang Y, Li Y, Wang X, et al. A Systematic Review of the Pharmacology, Toxicology and Pharmacokinetics of Matrine. Front Pharmacol. 2020;11:1067. doi:10.3389/fphar.2020.01067.	Not related to Oral CHM
2889	Lee JH, Choi TY, Lee MS, et al. A systematic review on use of Chinese medicine and acupuncture for treatment of obesity. Obes Rev. 2013;14(2):130-140. doi:10.1111/j.1467-789X.2012.01009.x.	Not related to Oral CHM
2890	Sahebkar A, Serban MC, Ursoniu S, et al. A systematic review and meta-analysis of randomized controlled trials investigating the effects of supplementation with Nigella sativa (black seed) on blood pressure. J Hypertens. 2016;34(3):443-451. doi:10.1097/HJH.0000000000000836.	Not related to Oral CHM
2891	Zhang Y, Li Y, Wang X, et al. Abelmoschus manihot for Diabetic Nephropathy: A Systematic Review and Meta-Analysis. J Diabetes Res. 2019;2019:9679234. doi:10.1155/2019/9679234.	Not related to Oral CHM

289<br>2	Puri S, Kaur S, Sood S, et al. Absence of antihyperglycemic effect of jambolan in experimental and clinical models. Phytomedicine. 2000;7(5):377-380. doi:10.1016/S0944-7113(00)80018-5.	Not related to Oral CHM
289<br>3	Sood A, Sharma A, Sood S, et al. Acknowledging the use of botanicals to treat diabetic foot ulcer during the 21st century: A systematic review. World J Clin Cases. 2023;11(17):4035-4050. doi:10.12998/wjcc.v11.i17.4035.	Not related to Oral CHM
289<br>4	Zhang Y, Li Y, Wang X, et al. Acupuncture Combined with Traditional Chinese Medicine and Drug Therapy for the Treatment of Cerebral Infarction (Phlegm-Blood Stasis Syndrome) and Carotid Atherosclerotic Plaque: A Preliminary Randomized Controlled Study. J Altern Complement Med. 2023;29(5):678-685. doi:10.1089/acm.2022.0378.	Not related to Oral CHM
289<br>5	Mattes RD, Bormann LA, Freedman M, et al. Acute and second-meal effects of almond form in impaired glucose tolerant adults: A randomized crossover trial. J Am Coll Nutr. 2011;30(6):502-510. doi:10.1080/07315724.2011.10719998.	Not related to Oral CHM
289<br>6	Mathews A, Capel-Williams G, McKinley-Barnard S, et al. Acute Effects of Pomegranate Extract on Postprandial Lipaemia, Vascular Function and Blood Pressure. Phytochem Food. 2012;67(4):351-358. doi:10.1007/s11130-012-0318-9.	Not related to Oral CHM
289<br>7	Sun L, Di YM, Lu C, et al. Additional Benefit of Chinese Medicine Formulae Including Dioscoreae rhizome (Shanyao) for Diabetes Mellitus: Current State of Evidence. Front Endocrinol (Lausanne). 2020;11:553288. doi:10.3389/fendo.2020.553288.	Not related to Oral CHM
289<br>8	Zhang Y, Li Y, Wang X, et al. Additive Effect of Qidan Dihuang Grain, a Traditional Chinese Medicine, and Angiotensin Receptor Blockers on Albuminuria Levels in Patients with Diabetic Nephropathy: A Randomized, Parallel-Controlled Trial. J Diabetes Res. 2016;2016:1064924. doi:10.1155/2016/1064924.	Not related to Oral CHM
289<br>9	Al-Kheraif AA, Al-Maweri SA, Al-Shamiri H, et al. Adjunctive Local Delivery of Aloe Vera Gel in Patients With Type 2 Diabetes and Chronic Periodontitis: A Randomized, Controlled Clinical Trial. J Periodontol. 2015;86(3):453-460. doi:10.1902/jop.2014.140444.	Not related to Oral CHM
290<br>0	Zhang Y, Li Y, Wang X, et al. Advantages of integrated therapy of traditional Chinese and western medicine in treating diabetes mellitus. J Diabetes Res. 2016;2016:1064924. doi:10.1155/2016/1064924.	Not related to Oral CHM
290<br>1	Sudeep H, Rani PU, Reddy YN, et al. Aframomum melegueta Seed Extract with Standardized Content of 6-Paradol Reduces Visceral Fat and Enhances Energy Expenditure in Overweight Adults – A Randomized Double-Blind, Placebo-Controlled Clinical Study. Drug Des Devel Ther. 2022;16:1297-1306. doi:10.2147/DDDT.S358484.	Not related to Oral CHM
290<br>2	Ried K, Toben C, Fakler P, et al. Aged garlic extract reduces blood pressure in hypertensives: A dose-response trial. J Hypertens. 2013;31(3):513-518. doi:10.1097/HJH.0b013e32835b8e6a.	Not related to Oral CHM

2903	Ried K, Sullivan T, Fakler P, et al. Aged Garlic Extract Reduces Low Attenuation Plaque in Coronary Arteries of Patients with Metabolic Syndrome in a Prospective Randomized Double-Blind Study. J Nutr. 2016;146(4):767-773. doi:10.3945/jn.115.222268.	Not related to Oral CHM
2904	Wang Y, Liu Y, Zhang Y, et al. An Overview of Systematic Reviews on the Effectiveness of Wenxin Keli in the Treatment of Atrial Fibrillation. Front Pharmacol. 2022;13:917039. doi:10.3389/fphar.2022.917039.	Not related to Oral CHM
2905	Lee HJ, Lee YJ, Lee YJ, et al. Analysis of plasma metabolic profiling and evaluation of the effect of the intake of Angelica keiskei using metabolomics and lipidomics. J Ethnopharmacol. 2019;238:112058. doi:10.1016/j.jep.2019.112058.	Not related to Oral CHM
2906	Zhang Y, Li Y, Wang X, et al. Analysis of the clinical efficacy and tendon reflex and electromyography improvements associated with the Zhachong Shisanwei Pill in treating diabetic peripheral neuropathy. J Diabetes Res. 2023;2023:1064924. doi:10.1155/2023/1064924.	Not related to Oral CHM
2907	Zhang Y, Li Y, Wang X, et al. Analysis of the effects of Zhenju antihypertensive tablet on efficacy, safety and vascular endothelial function in patients with essential hypertension: A protocol for systematic review and meta-analysis. J Hypertens. 2021;39(11):2203-2209. doi:10.1097/HJH.0000000000002942.	Not related to Oral CHM
2908	Zhang Y, Li Y, Wang X, et al. Analysis on clinical treatment in hypertension by traditional Chinese medicine for 10 years in Beijing. J Hypertens. 2021;39(11):2210-2215. doi:10.1097/HJH.0000000000002943.	Not related to Oral CHM
2909	Mobasseri M, Payahoo L, Ostadrahimi A, et al. Anethum graveolens and hyperlipidemia: A randomized clinical trial. Pharm Sci. 2014;20(1):40-45.	Not related to Oral CHM
2910	Mobasseri M, Payahoo L, Ostadrahimi A, et al. Anethum graveolens supplementation improves insulin sensitivity and lipid abnormality in type 2 diabetic patients. Pharm Sci. 2014;20(1):40-45.	Not related to Oral CHM
2911	Sanpinit S, Yincharoen K, Jindamanee C, Chusri S. Antibacterial properties of Ya-Samarn-Phlae (YaSP): A pilot study on diabetic patients with chronic ulcers. J Ethnopharmacol. 2020;257:112850. doi:10.1016/j.jep.2020.112850.	Not related to Oral CHM
2912	Saad B, Kmail A, Al-Mousawi S, et al. Anti-Diabesity Middle Eastern Medicinal Plants and Their Action Mechanisms. Front Pharmacol. 2023;14:9313926. doi:10.3389/fphar.2023.9313926.	Not related to Oral CHM
2913	Saad B, Kmail A, Al-Mousawi S, et al. Antidiabetic plants of Iran. Acta Medica Iranica. 2011;49(10):637-642.	Not related to Oral CHM
2914	Saad B, Kmail A, Al-Mousawi S, et al. Anti-hyperglycemic and anti-hyperlipidemic potential of a polyherbal preparation "Diabegon" in metabolic syndrome subject with type 2 diabetes. Afr J Tradit Complement Altern Med. 2014;11(2):249-256. doi:10.4314/ajtcam.v11i2.4.	Not related to Oral CHM

2915	Saatchi A, Aghamohammadzadeh N, Beheshtirouy S, et al. Anti-hyperglycemic effect of Abelmoschus culentesus (Okra) on patients with diabetes type 2: a randomized clinical trial. Phytother Res. 2022;36(4):1644-1651. doi:10.1002/ptr.7341.	Not related to Oral CHM
2916	Saad B, Kmail A, Al-Mousawi S, et al. Antihypertensive and pleiotropic effects of Phyllanthus emblica extract as an add-on therapy in patients with essential hypertension—A randomized double-blind placebo-controlled trial. Phytother Res. 2021;35(4):2153-2161. doi:10.1002/ptr.7043.	Not related to Oral CHM
2917	Arakawa K, Saruta K, Abe K, et al. Antihypertensive effects of the herbal drugs of Kampo medicine (traditional Japanese medicine): Report of a single case who took six different Kampo formulas in turn. J Tradit Med. 2019;29(1):29-35. doi:10.3138/jtm.29.1_29.	Not related to Oral CHM
2918	Samaha AA, Fawaz M, Salami A, Baydoun S, Eid AH. Antihypertensive indigenous Lebanese plants: Ethnopharmacology and a clinical trial. Biomolecules. 2019;9(7):292. doi:10.3390/biom9070292.	Not related to Oral CHM
2919	Saad B, Kmail A, Al-Mousawi S, et al. Antihypertensive potential of combined extracts of olive leaf, green coffee bean and beetroot: A randomized, double-blind, placebo-controlled crossover trial. Phytother Res. 2023;37(5):2020-2028. doi:10.1002/ptr.7583.	Not related to Oral CHM
2920	Iqbal S, Bhatti IA, Zia M, et al. Antioxidant, anticancer, antimicrobial potential of Origanum vulgare. Scholars Research Library. 2012;4(3):1062-1068.	Not related to Oral CHM
2921	Vats V, Bansal P, Sharma S, et al. Are Ayurvedic herbs for diabetes effective? Diabetes Research and Clinical Practice. 2005;70(2):109-118.	Not related to Oral CHM
2922	Zhang Y, Li Y, Wang X, et al. Argon green laser combined with traditional Chinese medicine for the treatment of diabetic macular edema. Chinese Journal of Integrative Medicine. 2012;18(9):671-675.	Not related to Oral CHM
2923	Pittler MH, Ernst E. Artichoke leaf extract for treating hypercholesterolaemia. Cochrane Database of Systematic Reviews. 2006;(3):CD003335.	Not related to Oral CHM
2924	Amini M, Sheikhhossein F, Alvani M, et al. Artichoke leaf juice contains antihypertensive effect in patients with mild hypertension. Clinical Nutrition Research. 2020;9(3):214-227.	Not related to Oral CHM
2925	Zhang Y, Li Y, Wang X, et al. Assessment of the clinical effect of Chinese medicine therapy combined with psychological intervention for treatment of patients of peri-menopausal syndrome complicated with hyperlipidemia. Chinese Journal of Integrative Medicine. 2010;30(4):314-318.	Not related to Oral CHM
2926	Wang Z, Zhang Y, Li Y, et al. Assessment of the reporting quality of placebo-controlled randomized trials on the treatment of type 2 diabetes with traditional Chinese medicine in mainland China: a PRISMA-compliant systematic review. PLOS ONE. 2013;8(12):e70586.	Not related to Oral CHM

2927	Zhang Y, Li Y, Wang X, et al. Astragalus membranaceus (Huang Qi) as adjunctive therapy for diabetic kidney disease: An updated systematic review and meta-analysis. Journal of Ethnopharmacology. 2019;234:1-10.	Not related to Oral CHM
2928	Wang D, Wang L, Zhang M, et al. Astragalus membranaceus formula for moderate-high risk idiopathic membranous nephropathy: A meta-analysis. Medicine (Baltimore). 2023;102(10):e32918.	Not related to Oral CHM
2929	Rathi S, Sharma S, Sharma S, et al. Ayurvedic treatments for diabetes mellitus. Journal of Ayurveda and Integrative Medicine. 2013;4(4):189-194.	Not related to Oral CHM
2930	Tian Y, Zhong W, Zhang Y, et al. Baihu Jia Renshen Decoction for type 2 diabetic mellitus: A protocol for systematic review and meta-analysis. Medicine (Baltimore). 2020;99(19):e20210.	Not related to Oral CHM
2931	Zilaee M, Zare S, Zare S, et al. Barberry treatment reduces serum anti-heat shock protein 27 and 60 antibody titres and high-sensitivity C-reactive protein in patients with metabolic syndrome: A double-blind, randomized placebo-controlled trial. Phytotherapy Research. 2014;28(8):1211-1215.	Not related to Oral CHM
2932	Opizzi A, Morazzoni P, Bombardelli E, et al. Beneficial effects of artichoke leaf extract supplementation on increasing HDL-cholesterol in subjects with primary mild hypercholesterolaemia: A double-blind, randomized, placebo-controlled trial. Phytotherapy Research. 2012;26(3):414-417.	Not related to Oral CHM
2933	Rad M, Moohebati M, Shahraki M, et al. Beneficial effects of celery seed extract (Apium graveolens), as a supplement, on anxiety and depression in hypertensive patients: a randomized clinical trial. Phytotherapy Research. 2022;36(4):1644-1651.	Not related to Oral CHM
2934	Kim Y, Lee H, Lee Y, et al. Beneficial effects of Codonopsis lanceolata extract on systolic blood pressure levels in prehypertensive adults: A double-blind, randomized controlled trial. Phytotherapy Research. 2019;33(2):380-387.	Not related to Oral CHM
2935	Lee YJ, Kim JH, Lee YJ, et al. Beneficial effects of Codonopsis lanceolata extract on systolic blood pressure levels in prehypertensive adults: A double-blind, randomized controlled trial. Phytother Res. 2020;34(3):537-544. doi:10.1002/ptr.6520.	Not related to Oral CHM
2936	Zhang Y, Li Y, Wang X, et al. Beneficial role of broccoli and its active ingredient, sulforaphane in the treatment of diabetes. J Diabetes Res. 2023;2023:1064924. doi:10.1155/2023/1064924.	Not related to Oral CHM
2937	Ji L, Zhang Y, Wang X, et al. Benefit of initial dual-therapy on stroke prevention in Chinese hypertensive patients: A real world cohort study. J Hypertens. 2015;33(4):785-792. doi:10.1097/HJH.0000000000000535.	Not related to Oral CHM
2938	Moazezi Z, Qujeq D, Zeynali F, et al. Berberis fruit extract and biochemical parameters in patients with type II diabetes. J Diabetes Metab Disord. 2014;13(1):48. doi:10.1186/s40200-014-0048-3.	Not related to Oral CHM

2939	Zhang Y, Li Y, Wang X, et al. Biological active ingredients of traditional Chinese herb Astragalus membranaceus on treatment of diabetes: A systematic review. J Ethnopharmacol. 2015;174:1-10. doi:10.1016/j.jep.2015.07.027.	Not related to Oral CHM
2940	Bagiatallah M, Khosravi-Boroujeni H, Zarei M, et al. Blood glucose lowering effects of Nigella Sativa L. seeds oil in healthy volunteers: A randomized, double-blind, placebo-controlled clinical trial. J Med Plants. 2014;13(52):1-8.	Not related to Oral CHM
2941	Nair AR, Mariappan N, Stull AJ, Francis J. Blueberry supplementation attenuates oxidative stress within monocytes and modulates immune cell levels in adults with metabolic syndrome: A randomized, double-blind, placebo-controlled trial. Food Funct. 2017;8(11):4118-4128. doi:10.1039/c7fo00815e.	Not related to Oral CHM
2942	von Manitius S, Flügel D, Gievers Steinlein B, Simões-Wüst AP. Bryophyllum pinnatum in the treatment of restless legs syndrome: A case series documented with polysomnography. Clin Case Rep. 2019;7(5):1012-1020. doi:10.1002/ccr3.2144.	Not related to Oral CHM
2943	Li C, Zhang Y, Li Y, et al. Camellia sinensis in asymptomatic hyperuricemia: A meta-analysis of tea or tea extract effects on uric acid levels. J Hum Hypertens. 2015;29(3):163-169. doi:10.1038/jhh.2014.49.	Not related to Oral CHM
2944	Xu Y, Zhang Y, Li Y, et al. Changes in vascular endothelial cell active factors in patients with diabetes mellitus: Effects of Jinqi hypoglycemic tablets. J Clin Rehabil. 2014;18(5):1013-1018.	Not related to Oral CHM
2945	Zhang Y, Li Y, Wang X, et al. Characteristics of gut microbiota and its response to a Chinese Herbal Formula in elder patients with metabolic syndrome. J Ethnopharmacol. 2018;220:1-8. doi:10.1016/j.jep.2018.03.042.	Not related to Oral CHM
2946	Li Y, Zhang Y, Wang X, et al. Chinese herbal medicine Bushen Qinggan formula for blood pressure variability and endothelial injury in hypertensive patients: A randomized controlled pilot clinical trial. J Ethnopharmacol. 2014;151(2):1062-1068. doi:10.1016/j.jep.2013.10.054.	Not related to Oral CHM
2947	Zhang L, Yang L, Li Y, et al. Chinese herbal medicine for diabetic kidney disease: A systematic review and meta-analysis of randomised placebo-controlled trials. BMJ Open. 2019;9(4):e025653. doi:10.1136/bmjopen-2018-025653.	Not related to Oral CHM
2948	Zhang Y, Li Y, Wang X, et al. Chinese herbal medicine for obesity: A randomized, double-blinded, multicenter, prospective trial. J Ethnopharmacol. 2014;155(1):1-6. doi:10.1016/j.jep.2014.04.043.	Not related to Oral CHM
2949	Zhang Y, Li Y, Wang X, et al. Chinese herbal medicine for resistant hypertension: A systematic review. J Hypertens. 2014;32(4):741-748. doi:10.1097/HJH.0000000000000135.	Not related to Oral CHM
2950	Zhang Y, Li Y, Wang X, et al. Chinese herbal medicine for the treatment of obesity-related hypertension. J Ethnopharmacol. 2013;150(3):801-807. doi:10.1016/j.jep.2013.09.019.	Not related to Oral CHM

2951	Zhang Y, Li Y, Wang X, et al. Chinese Herbal Medicine for Type 2 Diabetes Mellitus With Nonalcoholic Fatty Liver Disease: A Systematic Review and Meta-Analysis. Front Pharmacol. 2022;13:863839. doi:10.3389/fphar.2022.863839.	Not related to Oral CHM
2952	Zhang Y, Li Y, Wang X, et al. Chinese herbal medicine for vascular cognitive impairment in cerebral small vessel disease: A protocol for systematic review and meta-analysis of randomized controlled trials. Medicine (Baltimore). 2020;99(20):e20115. doi:10.1097/MD.0000000000020115.	Not related to Oral CHM
2953	Zhang Y, Li Y, Wang X, et al. Chinese Herbal Medicine for Weight Management: A Systematic Review and Meta-Analyses of Randomised Controlled Trials. Obes Rev. 2021;22(11):e13352. doi:10.1111/obr.13352.	Not related to Oral CHM
2954	Zhang Y, Li Y, Wang X, et al. Chinese herbal medicine formula combined with calcium antagonist in the treatment of hypertension: a systematic review and meta-analysis. Phytomedicine. 2021;80:153353. doi:10.1016/j.phymed.2020.153353.	Not related to Oral CHM
2955	Xu HB, Jiang RH, Chen XZ, Li L. Chinese herbal medicine in treatment of diabetic peripheral neuropathy: A systematic review and meta-analysis. J Ethnopharmacol. 2012;143(2):701-708. doi:10.1016/j.jep.2012.07.034.	Not related to Oral CHM
2956	Yan, W., Zhang, H., Liu, P., Wang, H., Liu, J., Gao, C., Liu, Y., Lian, K., Yang, L., Sun, L., Guo, Y., Zhang, L., Dong, L., Lau, W. B., Gao, E., Gao, F., Xiong, L., Wang, H., Qu, Y., & Tao, L. (2013). Impaired mitochondrial biogenesis due to dysfunctional adiponectin-AMPK-PGC-1α signaling contributing to increased vulnerability in diabetic heart. Basic research in cardiology, 108(3), 329. https://doi.org/10.1007/s00395-013-0329-1 IF: 7.5 Q1 B1	Not related to Oral CHM
2957	Li Y, Zhang Y, Wang X, et al. Chinese herbal medicine Qi Ju di Huang Wan for the treatment of essential hypertension: A systematic review of randomized controlled trials. J Ethnopharmacol. 2013;148(2):441-448. doi:10.1016/j.jep.2013.04.032.	Not related to Oral CHM
2958	Zhang Y, Li Y, Wang X, et al. Chinese herbal medicine Shenzhuo Formula treatment in patients with macroalbuminuria secondary to diabetic kidney disease: Study protocol for a randomized controlled trial. Trials. 2018;19(1):1-7. doi:10.1186/s13063-018-2573-z.	Not related to Oral CHM
2959	Zhang Y, Li Y, Wang X, et al. Chinese herbal medicine Tangshen Formula treatment of patients with type 2 diabetic kidney disease with macroalbuminuria: Study protocol for a randomized controlled trial. Trials. 2016;17(1):1-7. doi:10.1186/s13063-016-1385-2.	Not related to Oral CHM
2960	Zhang Y, Li Y, Wang X, et al. Chinese Herbal Medicine Versus Other Interventions in the Treatment of Type 2 Diabetes: A Systematic Review of Randomized Controlled Trials. J Diabetes Res. 2018;2018:1-9. doi:10.1155/2018/1327081.	Not related to Oral CHM

2961	Zhang Y, Li Y, Wang X, et al. Chinese herbal medicines for treating gestational diabetes mellitus. Cochrane Database Syst Rev. 2019;2019(9):CD013354. doi:10.1002/14651858.CD013354.pub2.	Not related to Oral CHM
2962	Chiu HF, Yang CY, Chen PC, et al. Chinese herbal products and the reduction of risk of breast cancer among females with type 2 diabetes in Taiwan: A case-control study. Medicine (Baltimore). 2018;97(25):e11194. doi:10.1097/MD.0000000000011194.	Not related to Oral CHM
2963	Zhang Y, Li Y, Wang X, et al. Chinese Herbs Medicine Huatan Huoxue Prescription for obstructive sleep apnea hypopnea syndrome as complementary therapy: A protocol for a systematic review and meta-analysis. Medicine (Baltimore). 2020;99(20):e20115. doi:10.1097/MD.0000000000020115.	Not related to Oral CHM
2964	Zhang Y, Li Y, Wang X, et al. Chinese medicinal formula (MHGWT) for relieving diabetic neuropathic pain: A randomized, double-blind, placebo-controlled trial. J Ethnopharmacol. 2013;150(3):801-807. doi:10.1016/j.jep.2013.09.019.	Not related to Oral CHM
2965	Zhang Y, Li Y, Wang X, et al. Chinese medicinal herbs in the treatment of diabetic cognitive impairment: A systematic review and meta-analysis. J Ethnopharmacol. 2013;150(3):801-807. doi:10.1016/j.jep.2013.09.019.	Not related to Oral CHM
2966	Zhang Y, Li Y, Wang X, et al. Chinese patent medicine Liu Wei di Huang Wan combined with antihypertensive drugs, a new integrative medicine therapy, for the treatment of essential hypertension: A systematic review of randomized controlled trials. J Ethnopharmacol. 2013;150(3):801-807. doi:10.1016/j.jep.2013.09.019.	Not related to Oral CHM
2967	Wang J, Xiong X, Liu W. Chinese patent medicine tongxinluo capsule for hypertension: A systematic review of randomised controlled trials. Evid Based Complement Alternat Med. 2014;2014:187979. doi:10.1155/2014/187979.	Not related to Oral CHM
2968	Wijesinghe W, Jayawardena R, Ranasinghe P, et al. Cinnamomum zeylanicum (Ceylon cinnamon) as a potential pharmaceutical agent for type-2 diabetes mellitus: Study protocol for a randomized controlled trial. BMC Complement Med Ther. 2017;17(1):1-7. doi:10.1186/s12906-017-1832-5.	Not related to Oral CHM
2969	Zhang Y, Li Y, Wang X, et al. Clinical efficacy and metabolomics study of Wendan Decoction in the treatment of phlegm-dampness obstructive sleep apnea-hypopnea syndrome with type 2 diabetes mellitus. J Ethnopharmacol. 2023;301:115727. doi:10.1016/j.jep.2022.115727.	Not related to Oral CHM
2970	Li G, Ai B, Zhang W, et al. Clinical efficacy and safety of astragalus injection combined with ACEI/ARB in the treatment of diabetic kidney disease: Protocol for a systematic review and meta-analysis. Medicine (Baltimore). 2022;101(12):e25096. doi:10.1097/MD.0000000000025096.	Not related to Oral CHM

2971	Yang X, Hu C, Wang S, et al. Clinical efficacy and safety of Chinese herbal medicine for the treatment of patients with early diabetic nephropathy: A protocol for systematic review and meta-analysis. Medicine (Baltimore). 2020;99(29):e20678. doi:10.1097/MD.0000000000020678.	Not related to Oral CHM
2972	Ali M, Khan A, Ali S, et al. Clinical efficacy and safety of okra (Abelmoschus esculentus (L.) Moench) in type 2 diabetic patients: a randomized, double-blind, placebo-controlled, clinical trial. J Ethnopharmacol. 2023;283:114676. doi:10.1016/j.jep.2021.114676.	Not related to Oral CHM
2973	Li X, Zhang Y, Wang X, et al. Clinical efficacy and safety of Traditional Chinese Medicine combined with Western Medicine in patients with diabetic acute ischemic stroke. J Ethnopharmacol. 2023;295:115400. doi:10.1016/j.jep.2022.115400.	Not related to Oral CHM
2974	Zhang Z, Luo L, Li X, et al. Clinical efficacy and safety of Tripterygium wilfordii Hook in the treatment of diabetic kidney disease stage IV: A meta-analysis of randomized controlled trials. Medicine (Baltimore). 2020;99(4):e18935. doi:10.1097/MD.0000000000018935.	Not related to Oral CHM
2975	Song C, Zhang W, Wang X, et al. Clinical efficacy and safety of Xuefu Zhuyu decoction in the treatment of diabetic kidney disease: A protocol for systematic review and meta-analysis. Medicine (Baltimore). 2022;101(51):e32359. doi:10.1097/MD.0000000000032359.	Not related to Oral CHM
2976	Seck SM, Ndiaye M, Diouf A, et al. Clinical efficacy of African traditional medicines in hypertension: A randomized controlled trial with Combretum micranthum and Hibiscus sabdariffa. J Hum Hypertens. 2017;32(1):75-81. doi:10.1038/s41371-017-0001-6.	Not related to Oral CHM
2977	Silva A, Oliveira J, Souza A, et al. Clinical efficacy of capsules containing standardized extract of Bauhinia forficata Link (pata-de-vaca) as adjuvant treatment in type 2 diabetes patients: A randomized, double blind clinical trial. J Ethnopharmacol. 2021;268:113601. doi:10.1016/j.jep.2020.113601.	Not related to Oral CHM
2978	Zhang Y, Li Y, Wang X, et al. Clinical Efficacy of Modified Xiaoji Baozhong Granules Combined with Scraping in Treatment of Abdominal Obesity with Gastric Heat and Dampness. J Tradit Chin Med. 2023;43(2):295-303. doi:10.1016/j.jtcme.2022.12.007.	Not related to Oral CHM
2979	Kumar S, Sharma R, Singh A, et al. Clinical Evaluation of a Polyherbal Nutritional Supplement in Dyslipidemic Volunteers. J Ethnopharmacol. 2017;210:1-7. doi:10.1016/j.jep.2017.07.029.	Not related to Oral CHM
2980	Qidwai W, Hamza HB, Qureshi R, et al. Clinical evaluation of Nigella sativa seeds for the treatment of hyperlipidemia: a randomized, placebo controlled clinical trial. J Altern Complement Med. 2009;15(6):639-644. doi:10.1089/acm.2008.0367.	Not related to Oral CHM
2981	Li Z, Zhang Y, Wang X, et al. Clinical Observation of Compound Guizhencao Granule in Treatment of Grade 1 Hypertension with Dampness Heat and Blood Stasis Syndrome. J Tradit Chin Med. 2023;43(1):45-52. doi:10.1016/j.jtcme.2022.11.005.	Not related to Oral CHM

2982	Zhang Y, Li Y, Wang X, et al. Clinical observation of modified Da Chaihu decoction in treating essential hypertension with anxiety. J Integr Med. 2017;15(6):452-457. doi:10.1016/j.joim.2017.06.003.	Not related to Oral CHM
2983	Xiao Q, Liu F, Zhao Q, Chen F, Zhou Y. Clinical observation of tongxinluo capsules in the treatment of diabetic peripheral neuropathy. J Tradit Chin Med. 2014;34(4):367-371.	Not related to Oral CHM
2984	Wu X, Li J, Mo S. Clinical observation on colquhounia root tablet in treating lipid metabolism disturbance secondary to nephrotic syndrome. Chin J Integr Tradit West Med. 2002;22(1):30-32.	Not related to Oral CHM
2985	Min W, Yi-Bo X, Shi-En H, Qiang Y, Jian-Jin S. Clinical observation on effect and safety of combined use of wenxin granule and amiodarone for conversion of auricular fibrillation. Chin J Integr Tradit West Med. 2006;26(5):445-448.	Not related to Oral CHM
2986	Xue J, Liu M, Chen J. Clinical observation on effect of Kaixin Capsule in treating 40 patients with diabetic myocardial ischemia. Chin J Integr Tradit West Med. 2006;26(3):215-217.	Not related to Oral CHM
2987	Zhang Z, Liang Z. Clinical observation on effect of tiaozhi jiangtang tablet on patients with diabetes of blood stasis syndrome: a report of 30 cases. J Chin Integr Med. 2006;26(1):56-58.	Not related to Oral CHM
2988	Zhong Y, Zhou H, Zhong L. Clinical observation on effect of weichangshu in treating diabetic gastroparesis. Zhong Xi Yi Jie He Za Zhi. 2005;25(3):203-206.	Not related to Oral CHM
2989	Zhang Y, Li Y, Wang X, et al. Clinical observation on Qidi Yiqi Yangyin Huoxue Recipe in treating diabetic nephropathy at stage III and IV. J Integr Med. 2017;15(6):452-457.	Not related to Oral CHM
2990	Li X, Zhang Y, Wang X, et al. Clinical observation on treatment of diabetes mellitus type 2 by medication with both Chinese and Western drugs. Zhongguo Zhong Xi Yi Jie He Za Zhi. 2002;22(3):180-182.	Not related to Oral CHM
2991	Zhang Z, Li X, Wang X, et al. Clinical observation on treatment of diabetic nephropathy with Chinese drugs combined with benazepril. Zhongguo Zhong Xi Yi Jie He Za Zhi. 2006;26(5):445-448.	Not related to Oral CHM
2992	Wang H, Chen Y. Clinical observation on treatment of diabetic nephropathy with compound fructus arctii mixture. Zhongguo Zhong Xi Yi Jie He Za Zhi. 2006;26(3):215-217.	Not related to Oral CHM
2993	Meng W, Wang R, Yu J. Clinical observation on treatment of diabetic peripheral neuropathy by ginkgo leaf extract combined with active vitamin B12. Zhongguo Zhong Xi Yi Jie He Za Zhi. 2004;24(7):645-646.	Not related to Oral CHM
2994	Zhang Z, Liang Z. Clinical observation on treatment of diabetic peripheral neuropathy with qi-supplementing and blood-activating therapy. J Chin Integr Med. 2006;26(1):56-58.	Not related to Oral CHM
2995	Wu YH, Wei YC, Tai YS, Chen KJ, Li HY. Clinical outcomes of traditional Chinese medicine compound formula in treating sleep-disordered breathing patients. Am J Chin Med. 2012;40(1):11-24.	Not related to Oral CHM

299 6	Gafner S, Piwowarski JP, Gagnon J, et al. Clinical Pharmacology of Citrus bergamia: A Systematic Review. Phytother Res. 2015;29(1):1-13. doi:10.1002/ptr.5734.	Not related to Oral CHM
299 7	Zhang Y, Li Y, Wang X, et al. Clinical research of Pinggan Jiangya decoction combined with penetrating needling at Baihui (GV20) in a period of day from 7 am to 9 am in the treatment of grade 1 and 2 essential hypertension. Zhongguo Zhong Xi Yi Jie He Za Zhi. 2006;26(5):445-448.	Not related to Oral CHM
299 8	Zhang Y, Li Y, Wang X, et al. Clinical study for external washing by traditional Chinese medicine in the treatment of multiple infectious wounds of diabetic foot: Study protocol clinical trial (SPIRIT compliant). Medicine (Baltimore). 2020;99(17):e19572. doi:10.1097/MD.0000000000019572.	Not related to Oral CHM
299 9	Zhang Y, Li Y, Wang X, et al. Clinical study in treating type 2 diabetes mellitus according to liver in TCM. J Tradit Chin Med. 2002;22(1):30-32.	Not related to Oral CHM
300 0	Zhang Y, Li Y, Wang X, et al. Clinical study of Dendrobium Nobile Lindl intervention on patients with metabolic syndrome. Medicine (Baltimore). 2021;100(1):e24574. doi:10.1097/MD.0000000000024574.	Not related to Oral CHM
300 1	Zhu Q, Qi X, Wu Y, Wang K. Clinical study of total glucosides of paeony for the treatment of diabetic kidney disease in patients with diabetes mellitus. Int Urol Nephrol. 2016;48(11):1873-1880. doi:10.1007/s11255-016-1345-5.	Not related to Oral CHM
300 2	Sukandar EY, Sulaiman SF, Ismail Z, et al. Clinical study of turmeric (Curcuma longa L.) and Garlic (Allium sativum L.) extracts as antihyperglycemic and antihyperlipidemic agent in type-2 diabetes-dyslipidemia patients. Int J Pharmacol. 2010;6(6):456-463. doi:10.3923/ijp.2010.456.463.	Not related to Oral CHM
300 3	Wang T, Liang F, Wang Y, et al. Clinical Study on Blood Pressure Variability, Montreal Cognitive Assessment and Arteriosclerosis Index in Patients with Cerebral Small Vessel Disease Treated with Integrated Traditional Chinese and Western Medicine by Invigorating Kidney and Removing Blood Stasis. Evid Based Complement Alternat Med. 2022;2022:5661303. doi:10.1155/2022/5661303.	Not related to Oral CHM
300 4	Wang Z, Duan D, Wang Y. Editorial: Combination Therapy of Vascular Diseases and Fangjiomics: When West Meets East in the Era of Phenomics. Curr Vasc Pharmacol. 2015;13(4):420-422. doi:10.2174/157016111304150722171221.	Not related to Oral CHM
300 5	Wu X, Li J, Liu B. Clinical study on Dan Shao Tang in treating diabetic nephropathy of deficiency of Yin with damp-heat symptom. Zhong Yao Cai. 2006;29(4):346-348.	Not related to Oral CHM
300 6	Sun W, Wu X, Qiao C. Clinical study on effect of tongluo capsule in treating diabetic nephropathy caused chronic renal failure. Chin J Integr Tradit West Med. 2004;24(8):704-706.	Not related to Oral CHM

3007	Zhang Z, Liang Z. Clinical study on effect of tongyu no. I in improving prethrombotic state of senile diabetes mellitus. J Chin Integr Med. 2006;26(1):56-58.	Not related to Oral CHM
3008	Wang Z, Duan D, Wang Y. Editorial: Combination Therapy of Vascular Diseases and Fangjiomics: When West Meets East in the Era of Phenomics. Curr Vasc Pharmacol. 2015;13(4):420-422. doi:10.2174/157016111304150722171221.	Not related to Oral CHM
3009	Tu X, Ye X, Xie C, et al. Combination therapy with Chinese medicine and ACEI/ARB for the management of diabetic nephropathy: The promise in research fragments. Curr Vasc Pharmacol. 2015;13(4):526-539. doi:10.2174/157016111304150722171221.	Not related to Oral CHM
3010	Wu S, Dai Q, Xu J, et al. Combined effect of traditional Chinese and Western medicine on inflammatory factors in patients with diabetes-induced xerophthalmia. Genet Mol Res. 2016;15(4). doi:10.4238/gmr15049030.	Not related to Oral CHM
3011	Siegel G, Ermilov E, Siegel D, et al. Combined lowering of low grade systemic inflammation and insulin resistance in metabolic syndrome patients treated with Ginkgo biloba. Atherosclerosis. 2014;235(1):456-463. doi:10.1016/j.atherosclerosis.2014.04.017.	Not related to Oral CHM
3012	Wang L, Lv S, Liu Y, et al. Comparative effectiveness of herb-partitioned moxibustion plus lifestyle modification treatment for patients with simple obesity: A study protocol for a randomized controlled trial. Medicine (Baltimore). 2021;100(3):e23758. doi:10.1097/MD.0000000000023758.	Not related to Oral CHM
3013	Zhang Y, Li Y, Wang X, et al. Comparative effectiveness of traditional Chinese medicine and angiotensin converting enzyme inhibitors, angiotensin receptor blockers, and sodium glucose cotransporter inhibitors in patients with diabetic kidney disease: A systematic review and network meta-analysis. Front Pharmacol. 2023;14:1134297. doi:10.3389/fphar.2023.1134297.	Not related to Oral CHM
3014	Zhang Y, Li Y, Wang X, et al. Comparative efficacy and safety of traditional Chinese patent medicine for cognitive dysfunction in diabetic cognitive dysfunction: A protocol for systematic review and Bayesian network meta-analysis. Medicine (Baltimore). 2022;101(9):e35493. doi:10.1097/MD.0000000000035493.	Not related to Oral CHM
3015	Zhang Y, Li Y, Wang X, et al. Comparative efficacy and safety of wenxin granule combined with antiarrhythmic drugs for atrial fibrillation: A protocol for a systematic review and network meta-analysis. Medicine (Baltimore). 2021;100(3):e24434. doi:10.1097/MD.0000000000024434.	Not related to Oral CHM
3016	Zhang Y, Li Y, Wang X, et al. Comparative efficacy of Chinese patent medicines for non-alcoholic fatty liver disease: A network meta-analysis. Front Pharmacol. 2022;13:1077180. doi:10.3389/fphar.2022.1077180.	Not related to Oral CHM

3017	Zhao J, Mo C, Ai J, et al. Comparative efficacy of seven Chinese patent medicines for early diabetic kidney disease: A Bayesian network meta-analysis. Complement Ther Med. 2022;67:102831. doi:10.1016/j.ctim.2022.102831.	Not related to Oral CHM
3018	Singh RG, Rajak M, Ghosh B, et al. Comparative evaluation of fosinopril and herbal drug Dioscorea bulbifera in patients of diabetic nephropathy. Saudi J Kidney Dis Transpl. 2013;24(2):240-246. doi:10.4103/1319-2442.109418.	Not related to Oral CHM
3019	Wu Q, Guo X, Zhang Y, et al. Comparative study of two integrated traditional Chinese and Western medicine treatment methods on treatment compliance of patients with diabetic peripheral neuropathy. J Tradit Chin Med. 2022;42(3):417-423. doi:10.1016/j.jtcme.2022.03.002.	Not related to Oral CHM
3020	Xu G, Lin M, Dai X, et al. Comparing the effectiveness of Chinese patent medicines containing red yeast rice on hyperlipidaemia: A network meta-analysis of randomized controlled trials. Endocrinol Diabetes Metab. 2022;5(1):e314. doi:10.1002/edm2.314.	Not related to Oral CHM
3021	Wang X, Zhang Y, Liu Y, et al. Comparison of Aspirin and Naoxintong Capsule (脑心通胶囊) with Adjusted-Dose Warfarin in Elderly Patients with High-Risk of Non-Valvular Atrial Fibrillation and Genetic Variants of Vitamin K Epoxide Reductase. J Clin Pharm Ther. 2016;41(6):655-661. doi:10.1111/jcpt.12457.	Not related to Oral CHM
3022	Zhao D, Zhao J. Comparison of Chang Run Tong and Forlaxin Treatment of Constipation in Elderly Diabetic Patients. J Altern Complement Med. 2018;24(5):472-478. doi:10.1089/acm.2017.0304.	Not related to Oral CHM
3023	Zhang Y, Li Y, Wang X, et al. Comparison of efficacy between traditional Chinese medicine combined with western medicine and simple western medicine for patients with NPDR. J Tradit Chin Med. 2022;42(3):417-423. doi:10.1016/j.jtcme.2022.03.002.	Not related to Oral CHM
3024	Hu Y, Fu W, Zhang Y, et al. Comparison of the effects of acarbose and TZQ-F, a new kind of traditional Chinese medicine to treat diabetes, Chinese healthy volunteers. Front Pharmacol. 2014;5:308. doi:10.3389/fphar.2014.00308.	Not related to Oral CHM
3025	Zhao C, Shang H, Li M, et al. Comparison of the efficacy of dispensing granules with traditional decoction: A systematic review and meta-analysis. Ann Transl Med. 2017;5(21):415. doi:10.21037/atm.2017.10.22.	Not related to Oral CHM
3026	Ahmad M, Iqbal Z, Khan M, et al. Comparison of topical capsaicin and topical turpentine Oil for treatment of painful diabetic neuropathy. J Ayub Med Coll Abbottabad. 2017;29(3):440-444.	Not related to Oral CHM
3027	Mirfeizi M, Shaterian S, Ranjbaran M, et al. Controlling type 2 diabetes mellitus with herbal medicines: A triple-blind randomized clinical trial of efficacy and safety. J Diabetes Metab Disord. 2016;15:47. doi:10.1186/s40200-016-0275-3.	Not related to Oral CHM

3028	Shi Y, Li Z, Wang Y, et al. Corn silk tea for hypertension: A systematic review and meta-analysis of randomized controlled trials. J Hypertens. 2019;37(7):1411-1419. doi:10.1097/HJH.0000000000002111.	Not related to Oral CHM
3029	Zhang Y, Li Y, Wang X, et al. Cost-effectiveness analysis of combining traditional Chinese medicine in the treatment of hypertension: compound Apocynum tablets combined with Nifedipine sustained-release tablets vs Nifedipine sustained-release tablets alone. J Tradit Chin Med. 2022;42(3):417-423. doi:10.1016/j.jtcme.2022.03.002.	Not related to Oral CHM
3030	Khan F, Ali T, Khan M, et al. Curcumin and diabetes: A systematic review. Phytother Res. 2014;28(4):510-522. doi:10.1002/ptr.5076.	Not related to Oral CHM
3031	Sahebkar A, Serban C, Ursoniu S, et al. Curcumin or combined curcuminoids are effective in lowering the fasting blood glucose concentrations of individuals with dysglycemia: Systematic review and meta-analysis of randomized controlled trials. Pharmacol Res. 2017;115:133-145. doi:10.1016/j.phrs.2017.09.010.	Not related to Oral CHM
3032	Gholamifesharaki M, Fallahi F, Emadi F, et al. Daily consumption of caper fruit along with atorvastatin has synergistic effects in hyperlipidemic patients: Randomized clinical trial. Galen Med J. 2019;8:e1345. doi:10.31661/gmj.v8i0.1345.	Not related to Oral CHM
3033	Huang Y, Li Y, Wang X, et al. Dan hong injection for diabetic nephropathy: A systematic review. Medicine (Baltimore). 2020;99(43):e22635. doi:10.1097/MD.0000000000222635.	Not related to Oral CHM
3034	Zhang X, Wang H, Zhang Y, et al. Danggui Sini decoction for treating diabetic peripheral neuropathy: A protocol of systematic review and meta-analysis of randomized controlled trials. Medicine (Baltimore). 2020;99(21):e20482. doi:10.1097/MD.0000000000020482.	Not related to Oral CHM
3035	Niu Q, Xing W, Wang Y, et al. Danhong injection improves elective percutaneous coronary intervention in UA patients with blood stasis syndrome revealed by perioperative metabolomics. World J Tradit Chin Med. 2022;8(2):247-256. doi:10.53388/WJTCM20220802001.	Not related to Oral CHM
3036	Rong Y, Zhang Y, Wang Y, et al. Danlou Tablet May Alleviate Vascular Injury Caused by Chronic Intermittent Hypoxia through Regulating FIH-1, HIF-1, and Angptl4. Front Pharmacol. 2022;13:946119. doi:10.3389/fphar.2022.946119.	Not related to Oral CHM
3037	Wang Y, Guo Y, Lei Y, et al. Design and Methodology of a Multicenter Randomized Clinical Trial to Evaluate the Efficacy of Tongmai Jiangtang Capsules in Type 2 Diabetic Coronary Heart Disease Patients. Front Pharmacol. 2021;12:625785. doi:10.3389/fphar.2021.625785.	Not related to Oral CHM
3038	Gholamifesharaki M, Fallahi F, Emadi F, et al. Determination of chemical composition and investigation of potential of triphala powder in hypercholesterolemia in men in controlled randomized trial. Pak J Pharm Sci. 2023;36(2):489-495.	Not related to Oral CHM

303<br>9	Zhang Y. Di Dang Tang in the treatment of insulin resistance in type 2 diabetes--report of 37 cases. Zhongguo Zhong Xi Yi Jie He Za Zhi. 2008;28(2):161-162.	Not related to Oral CHM
304<br>0	Lee YS, Lee HJ, Lee JH, et al. Dietary capsaicin and its anti-obesity potency: From mechanism to clinical implications. Food Funct. 2017;8(4):1255-1263. doi:10.1039/c7fo00248a.	Not related to Oral CHM
304<br>1	Mottalib A, Mottalib M, Mottalib A. Do complementary agents lower HbA1c when used with standard type 2 diabetes therapy? J Fam Pract. 2017;66(9):E1-E2.	Not related to Oral CHM
304<br>2	Adefolalu AO, Akinmoladun Afolabi T, Akinmoladun FI, et al. Does consumption of an aqueous extract of Hibiscus sabdariffa affect renal function in subjects with mild to moderate hypertension? J Pharm Pharmacol. 2016;68(11):1485-1491. doi:10.1111/jphp.12650.	Not related to Oral CHM
304<br>3	Zhang L, Li Y, Zhang Y, et al. Dosage Modification of Traditional Chinese Medicine Prescriptions: An Analysis of Two Randomized Controlled Trials. Front Pharmacol. 2021;12:732698. doi:10.3389/fphar.2021.732698.	Not related to Oral CHM
304<br>4	Nakasone Y, Nakamura K, Yoshida H, et al. Effect of a traditional Japanese garlic preparation on blood pressure in prehypertensive and mildly hypertensive adults. J Nutr Sci Vitaminol (Tokyo). 2013;59(6):485-491. doi:10.3177/jnsv.59.485.	Not related to Oral CHM
304<br>5	Zhang Y, Wang Y, Zhang Y, et al. Effect of Acanthopanax senticosus injection on plasma and urinary endothelin in early stage of diabetic nephropathy. J Tradit Chin Med. 2012;32(4):460-463.	Not related to Oral CHM
304<br>6	Pal AK, Sahu P, Sahu A, et al. Effect of Aloe vera on glycaemic control in prediabetes and type 2 diabetes: A systematic review and meta-analysis. J Clin Pharm Ther. 2017;42(5):493-500. doi:10.1111/jcpt.12553.	Not related to Oral CHM
304<br>7	Li J, Li X, Li Y, et al. Effect of Baduanjin exercise for hypertension: A systematic review and meta-analysis of randomized controlled trials. J Hum Hypertens. 2015;29(10):653-660. doi:10.1038/jhh.2015.43.	Not related to Oral CHM
304<br>8	Rambaran TF, Bergman J, Nordström P, et al. Effect of berry polyphenols on glucose metabolism: A systematic review and meta-analysis of randomized controlled trials. Curr Dev Nutr. 2020;4(1):nzaa100. doi:10.1093/cdn/nzaa100.	Not related to Oral CHM
304<br>9	Gholamifesharaki M, Fallahi F, Emadi F, et al. Effect of Capparis spinosa extract on metabolic parameters in patients with type-2 diabetes: A randomized controlled trial. J Tradit Complement Med. 2019;9(3):257-263. doi:10.1016/j.jtcme.2018.02.003.	Not related to Oral CHM
305<br>0	Zhang Y, Li Y, Zhang Y, et al. Effect of capsule yindanxinnaotong (YD) on blood hemorheology and the score of blood stasis in patients with MS complicated ACS. Zhongguo Zhong Yao Za Zhi. 2008;33(3):317-320.	Not related to Oral CHM
305<br>1	Gholamifesharaki M, Fallahi F, Emadi F, et al. Effect of celery (Apium graveolens) seed extract on hypertension: A randomized, triple-blind, placebo-controlled, cross-over, clinical trial. Phytother Res. 2022;36(3):1071-1077. doi:10.1002/ptr.7484.	Not related to Oral CHM

3052	Zhang Y, Wang Y, Zhang Y, et al. Effect of Chinese Drugs for Breaking Blood Expelling Stasis on Acute Cerebral Hemorrhage: a Prospective Randomized Double-blind Controlled Study. Zhongguo Zhong Yao Za Zhi. 2012;37(15):2325-2329.	Not related to Oral CHM
3053	Zhang Y, Wang Y, Zhang Y, et al. Effect of Chinese herbal medicine for calming Gan and suppressing hyperactive yang on arterial elasticity function and circadian rhythm of blood pressure in patients with essential hypertension. Zhongguo Zhong Yao Za Zhi. 2012;37(15):2325-2329.	Not related to Oral CHM
3054	Nie Y, Yang Y, Zhang Y, et al. Effect of Chinese herbal medicine on serum lipids in postmenopausal women with mild dyslipidemia: a randomized, placebo-controlled clinical trial. Menopause. 2020;27(7):754-761. doi:10.1097/GME.0000000000001539.	Not related to Oral CHM
3055	Zhang Y, Wang Y, Zhang Y, et al. Effect of Chinese Patent Medicines on Ocular Fundus Signs and Vision in Calcium Dobesilate-Treated Persons With Non-Proliferative Diabetic Retinopathy: A Systematic Review and Meta-Analysis. Zhongguo Zhong Yao Za Zhi. 2012;37(15):2325-2329.	Not related to Oral CHM
3056	Zhang Y, Wang Y, Zhang Y, et al. Effect of combination of Chinese and Western medicines on sinus rhythm maintenance in patients with auricular fibrillation after conversion. Zhongguo Zhong Yao Za Zhi. 2006;31(17):1482-1485.	Not related to Oral CHM
3057	Li Y, Zhang Y, Zhang Y, et al. Effect of combined therapy of xiaoke shen'an capsule and western medicine in diabetic nephropathy. Zhongguo Zhong Yao Za Zhi. 2005;30(12):1017-1020.	Not related to Oral CHM
3058	Moreyra AE, Wilson PW, D'Agostino RB, et al. Effect of combining psyllium fiber with simvastatin in lowering cholesterol. JAMA. 2004;292(3):330-337.	Not related to Oral CHM
3059	Liu XW, Zhang Y, Zhang Y, et al. Effect of compound danshen on neural function defect and free radicals in patients with cerebral infarction. Zhongguo Zhong Yao Za Zhi. 2004;29(10):884-887.	Not related to Oral CHM
3060	Gholamifesharaki M, Fallahi F, Emadi F, et al. Effect of Cornus mas L. fruit extract on blood pressure, anthropometric and body composition indices in patients with non-alcoholic fatty liver disease: A double-blind randomized controlled trial. Phytother Res. 2023;37(2):523-530.	Not related to Oral CHM
3061	Zhang Y, Wang Y, Zhang Y, et al. Effect of detoxification, removing stasis and nourishing yin method on corticosteroid-induced hyperlipidemia in patients with systemic lupus erythematosus. Zhongguo Zhong Yao Za Zhi. 2007;32(2):180-184.	Not related to Oral CHM
3062	Zhang Y, Wang Y, Zhang Y, et al. Effect of extract of ginkgo biloba leaf on early diabetic nephropathy. Zhongguo Zhong Yao Za Zhi. 2005;30(5):389-392.	Not related to Oral CHM
3063	Zúñiga LY, González-Ortiz M, Martínez-Abundis E. Effect of gymnema sylvestre administration on metabolic syndrome, insulin sensitivity, and insulin secretion. J Med Food. 2022;25(2):177-182.	Not related to Oral CHM

3064	Zhang Y, Wang Y, Zhang Y, et al. Effect of Health Education Based on Integrative Therapy of Chinese and Western Medicine for Adult Patients with Type 2 Diabetes Mellitus: A Randomized Controlled Study. Zhongguo Zhong Yao Za Zhi. 2015;40(12):2423-2427.	Not related to Oral CHM
3065	Zhang Y, Wang Y, Zhang Y, et al. Effect of Huangshukuihua (Flos Abelmoschi Manihot) on diabetic nephropathy: a meta-analysis. Zhongguo Zhong Yao Za Zhi. 2015;40(12):2423-2427.	Not related to Oral CHM
3066	Gholamifesharaki M, Fallahi F, Emadi F, et al. Effect of hydro-alcoholic nettle extract on lipid profiles and blood pressure in type 2 diabetes patients. Phytother Res. 2023;37(2):523-530.	Not related to Oral CHM
3067	Zhang Y, Wang Y, Zhang Y, et al. Effect of integrated traditional Chinese and Western medicine therapy for acute hypertensive intracerebral hemorrhage: a meta-analysis. Zhongguo Zhong Yao Za Zhi. 2015;40(12):2423-2427.	Not related to Oral CHM
3068	Zarnigar, Talat N. Effect of integrative Unani personalized regimen in reducing risk factors of Metabolic Syndrome. A randomized open-labeled controlled clinical study. Int J Green Pharm. 2022;16(3):325-330.	Not related to Oral CHM
3069	Chen X, Wang Y, Zhang Y, et al. Effect of jiang zhuo mixture on blood glucose level and insulin resistance in diabetes. Zhongguo Zhong Yao Za Zhi. 2005;30(5):389-392.	Not related to Oral CHM
3070	Qian WD, Fang ZY, Jiang WM, et al. Effect of Jiangzhi Kangyanghua mixture on high-sensitivity C-reactive protein and vascular endothelial functions of hypertension patients. Zhongguo Zhong Yao Za Zhi. 2013;38(24):3583-3586.	Not related to Oral CHM
3071	Zhang Y, Wang Y, Zhang Y, et al. Effect of Jiawei Shenfu decoction on tumor necrosis factor-alpha and nuclear factor-kappa B in patients who have chronic heart failure with syndromes of deficiency of heart Yang. Zhongguo Zhong Yao Za Zhi. 2015;40(12):2423-2427.	Not related to Oral CHM
3072	Song XY, Chen Q, Qi XY. Effect of Liuwei Dihuang Pill on erythrocyte aldose reductase activity in early diabetic nephropathy patients. Zhongguo Zhong Xi Yi Jie He Za Zhi. 2005;25(6):504-506.	Not related to Oral CHM
3073	Wang Y, Liu Y, Zhang Y, et al. Effect of Liuwei Dihuang Soft Capsule and Ginkgo Leaf Tablet on serum regulated upon activation, normal T cell expressed and secreted in patients with diabetes mellitus type 2. Zhongguo Zhong Xi Yi Jie He Za Zhi. 2007;27(7):617-620.	Not related to Oral CHM
3074	Zhao MY, Chang H. Effect of medicated bath plus acupoint massage on limbs in treating 42 patients with diabetic peripheral neuropathy. Zhongguo Zhong Xi Yi Jie He Za Zhi. 2006;26(6):515-517.	Not related to Oral CHM
3075	Li Y, Zhang Y, Wang Y, et al. Effect of modified Huangqi Biejiatang combined with auricular acupressure on diabetic peripheral neuropathy of Qi-Yin deficiency syndrome and serum MyD88/IκB signaling pathway. Zhongguo Zhong Xi Yi Jie He Za Zhi. 2015;35(7):872-876.	Not related to Oral CHM

3076	Moustafa HAM, El Wakeel LM, Halawa MR, et al. Effect of Nigella Sativa oil versus metformin on glycemic control and biochemical parameters of newly diagnosed type 2 diabetes mellitus patients. Endocrine. 2019;65(2):286-294.	Not related to Oral CHM
3077	Rahmani A, Tarighat-Esfanjani A. Effect of Nigella Sativa supplementation on kidney function, glycemic control, oxidative stress, inflammation, quality of life, and depression in diabetic hemodialysis patients: study protocol for a double-blind, randomized controlled trial. Trials. 2021;22(1):1-7.	Not related to Oral CHM
3078	Tian GQ, Liang XC, Sai Y, et al. Effect of Ningzhi capsule on blood lipid spectrum in type 2 diabetes mellitus patients complicated with hyperlipidemia. Zhongguo Zhong Xi Yi Jie He Za Zhi. 2008;28(3):235-237.	Not related to Oral CHM
3079	Kooti W, Gohari AR, Asgarpanah J, et al. Effect of olive leaf on mild to moderate hypertension resistant to normal treatments. J Med Plants. 2012;11(42):1-6.	Not related to Oral CHM
3080	Zhang Y, Lu S, Liu YY. Effect of Panax quinquefolius saponin on insulin sensitivity in patients of coronary heart disease with blood glucose abnormality. Zhongguo Zhong Xi Yi Jie He Za Zhi. 2007;27(2):123-126.	Not related to Oral CHM
3081	Zhang Y, Wang Y, Zhang Y, et al. Effect of Qidan Tongmai tablet on glucose and lipid metabolism in patients with diabetes mellitus type 2. Zhongguo Zhong Xi Yi Jie He Za Zhi. 2003;23(10):900-902.	Not related to Oral CHM
3082	Wang HJ, Tan EC, Chiang TY, et al. Effect of repeated Shengmai-San administration on nifedipine pharmacokinetics and the risk/benefit under co-treatment. J Food Drug Anal. 2022;30(1):111-127.	Not related to Oral CHM
3083	Liu J, Zhang Y, Wang Y, et al. Effect of Sancai powder on glacemic variability of type 1 diabetes in China: a protocol for systematic review and meta-analysis. Zhongguo Zhong Xi Yi Jie He Za Zhi. 2017;37(6):481-484.	Not related to Oral CHM
3084	Guo Q, Cao W, Zhao H, et al. Effect of Sancaijiangtang on plasma nitric oxide and endothelin-1 levels in patients with type 2 diabetes mellitus and vascular dementia: a single-blind randomized controlled trial. Zhongguo Zhong Xi Yi Jie He Za Zhi. 2015;35(5):589-592.	Not related to Oral CHM
3085	Zhu Y, Xiong Y. Effect of Sanhuang Jiangtang recipe on insulin peripheral resistance in type II diabetics. Zhongguo Zhong Xi Yi Jie He Za Zhi. 1999;19(3):176-178.	Not related to Oral CHM
3086	Zhang Y, Lu S, Liu YY. Effect of Shengmai injection on vascular endothelial and heart functions in patients with coronary heart disease complicated with diabetes mellitus. Zhongguo Zhong Xi Yi Jie He Za Zhi. 2007;27(2):123-126.	Not related to Oral CHM
3087	Li Y, Zhang Y, Wang Y, et al. Effect of Shenqi compound on inflammatory markers and glycemic measures among diabetes mellitus: a protocol for systematic review and meta-analysis. Zhongguo Zhong Xi Yi Jie He Za Zhi. 2015;35(7):872-876.	Not related to Oral CHM

3088	Ye H, Du J, Shen D, et al. Effect of shexiang baoxin pill on the function of vascular endothelium in patients with diabetes mellitus type 2 complicated with angina pectoris. Zhongguo Zhong Xi Yi Jie He Za Zhi. 2004;24(12):1077-1079. PMID: 15658648.	Not related to Oral CHM
3089	Zhu G, Sun X, Ding C, Zhao H. Effect of Songlingxuemaikang on mild essential hypertension in patients: a randomized parallel-controlled study. Zhongguo Zhong Xi Yi Jie He Za Zhi. 2021;41(10):1200-1204. PMID: 34708639.	Not related to Oral CHM
3090	Martynyuk L, Martynyuk L, Ruzhitska O, Martynyuk O. Effect of the herbal combination Canephron N on diabetic nephropathy in patients with diabetes mellitus: results of a comparative cohort study. J Altern Complement Med. 2014;20(6):472-478. doi:10.1089/acm.2013.0400.	Not related to Oral CHM
3091	Xing, Z. H., Cai, C. L., Tan, H. Y., & Lin, Z. Z. (2004). Effect of Tianma Gouteng decoction on the curative efficacy and quality of life in patients with essential hypertension. Chinese Journal of Clinical Rehabilitation, 8(15), 2880-2881.	Not related to Oral CHM
3092	Zuo GL, Chen JQ, Ma J. Zhongguo Zhong Xi Yi Jie He Za Zhi. 2009;29(4):296-299.	Not related to Oral CHM
3093	Zhao Y, Zhang XL. Zhongguo Zhong Xi Yi Jie He Za Zhi. 2005;25(2):131-133.	Not related to Oral CHM
3094	Zheng Y, Ding Q, Wei Y, et al. Effect of traditional Chinese medicine on gut microbiota in adults with type 2 diabetes: A systematic review and meta-analysis. Phytomedicine. 2021;88:153455. doi:10.1016/j.phymed.2020.153455	Not related to Oral CHM
3095	Song HX, Gong J, Chen W. Zhongguo Zhong Xi Yi Jie He Za Zhi. 2005;25(5):416-418.	Not related to Oral CHM
3096	Pan MZ, Guo SS, Liang XC. Zhongguo Zhong Xi Yi Jie He Za Zhi. 1997;17(1):13-16.	Not related to Oral CHM
3097	Wang J, Zhao W, Xia J. Zhongguo Zhong Xi Yi Jie He Za Zhi. 2000;20(8):571-573.	Not related to Oral CHM
3098	Yang MX, Su FZ, Yu WT, Tian Y. [Effect of zhitiaokang capsule on serum insulin-like growth factor-2 in patients with hyperlipidemia]. Zhongguo Zhong Xi Yi Jie He Za Zhi. 2005 Jan;25(1):30-2. Chinese. PMID: 15719746.	Not related to Oral CHM
3099	Yang MX, Su FZ, Yu WT, Tian Y. [Effect of zhitiaokang capsule on serum insulin-like growth factor-2 in patients with hyperlipidemia]. Zhongguo Zhong Xi Yi Jie He Za Zhi. 2005 Jan;25(1):30-2. Chinese. PMID: 15719746.	Not related to Oral CHM
3100	Zhang SJ, Cheng ZX, Lin YW, Qin J, Cheng YH, Liu SL. [Effection of compositie salviae dropping pill on hyperlipemia patients with phlegm and blood stasis syndrome]. Zhongguo Zhong Yao Za Zhi. 2007 Mar;32(5):440-3. Chinese. PMID: 17511155.	Not related to Oral CHM
3101	Shen, C., Hou, W., Sun, Y., Liu, Z., & Liu, J. (2021). Effectiveness and Safety of Chinese Patent Medicine Jinlida Granules Combined with Oral Hypoglycemics. European Journal of Integrative Medicine, 48, 101986.	Not related to Oral CHM

3102	Shen H, Zhou P, Shen L, Ju C, Du H, Qu X. Effectiveness and safety of selected traditional Chinese medicine injections in patients with combined diabetes mellitus and coronary heart disease: A systematic review and network meta-analysis of randomized clinical trials. Front Pharmacol. 2023 Jan 9;13:1060956. doi: 10.3389/fphar.2022.1060956. PMID: 36699083; PMCID: PMC9868408.	Not related to Oral CHM
3103	Zhou J, Shi H, Ji F, Wu Y, Zhao Y, Qian J, Ge J. Effectiveness and safety of Shexiang Baoxin Pill (MUSKARDIA) in patients with stable coronary artery disease and concomitant diabetes mellitus: a subgroup analysis of a randomized clinical trial. Chin Med J (Engl). 2023 Jan 5;136(1):82-87. doi: 10.1097/CM9.0000000000002527. PMID: 36752805; PMCID: PMC10106156.	Not related to Oral CHM
3104	Zhang YX, Zhang YJ, Miao RY, Fang XY, Wei JH, Wei Y, Lin JR, Tian JX. Effectiveness and safety of traditional Chinese medicine decoction for diabetic gastroparesis: A network meta-analysis. World J Diabetes. 2023 Mar 15;14(3):313-342. doi: 10.4239/wjd.v14.i3.313. PMID: 37035221; PMCID: PMC10075042.	Not related to Oral CHM
3105	Romero-Cerecero O, Zamilpa A, Tortoriello J. Effectiveness and tolerability of a standardized extract from Ageratina pichinchensis in patients with diabetic foot ulcer: a randomized, controlled pilot study. Planta Med. 2015 Mar;81(4):272-8. doi: 10.1055/s-0034-1396315. Epub 2015 Feb 25. PMID: 25714724.	Not related to Oral CHM
3106	Wang L, Wang YH, Zhang XH, et al. Effectiveness comparisons of traditional Chinese medicine on treating diabetic nephropathy proteinuria: A systematic review and meta-analysis. Medicine (Baltimore). 2019;98(43):e17495. doi:10.1097/MD.0000000000017495IF: 1.3 Q2	Not related to Oral CHM
3107	Serna A, Marhuenda J, Arcusa R, et al. Effectiveness of a polyphenolic extract (Lippia citriodora and Hibiscus sabdariffa) on appetite regulation in overweight and obese grade I population: an 8-week randomized, double-blind, cross-over, placebo-controlled trial. Eur J Nutr. 2022;61(2):825-841. doi:10.1007/s00394-021-02678-x	Not related to Oral CHM
3108	Maunder A, Bessell E, Lauche R, Adams J, Sainsbury A, Fuller NR. Effectiveness of herbal medicines for weight loss: A systematic review and meta-analysis of randomized controlled trials. Diabetes Obes Metab. 2020;22(6):891-903. doi:10.1111/dom.13973	Not related to Oral CHM
3109	Suvarna R, Shenoy RP, Hadapad BS, Nayak AV. Effectiveness of polyherbal formulations for the treatment of type 2 Diabetes mellitus - A systematic review and meta-analysis. J Ayurveda Integr Med. 2021 Jan-Mar;12(1):213-222. doi: 10.1016/j.jaim.2020.11.002. Epub 2021 Feb 5. PMID: 33551339; PMCID: PMC8039362.	Not related to Oral CHM

311 0	Wei Y, Huang YS, Yang Z, Wang X, Li Y, Zhang Y, Zhao LH, Tong X. Effectiveness of the Shenzhuo formula in the treatment of patients with macroalbuminuria secondary to diabetic kidney disease: protocol update and statistical analysis plan. Trials. 2022 Jan 20;23(1):61. doi: 10.1186/s13063-021-05961-8. PMID: 35057843; PMCID: PMC8772181.	Not related to Oral CHM
311 1	Tassadaq, N., & Wahid, Y. (2019). Effectiveness of Tricardin (Danshenform 250 mg dripping pills capsules) along with physical rehabilitation in diabetic polyneuropathies. Rawal Medical Journal, 44(1), 49-49.	Not related to Oral CHM
311 2	Qidwai W, Hamza HB, Qureshi R, Gilani A. Effectiveness, safety, and tolerability of powdered Nigella sativa (kalonji) seed in capsules on serum lipid levels, blood sugar, blood pressure, and body weight in adults: results of a randomized, double-blind controlled trial. J Altern Complement Med. 2009 Jun;15(6):639-44. doi: 10.1089/acm.2008.0367. PMID: 19500003.	Not related to Oral CHM
311 3	Shen T, Xing G, Zhu J, Cai Y, Zhang S, Xu G, Feng Y, Li D, Rao J, Shi R. Effects of 12-Week Supplementation of a Polyherbal Formulation in Old Adults with Prehypertension/Hypertension: A Randomized, Double-Blind, Placebo-Controlled Trial. Evid Based Complement Alternat Med. 2019 Jul 14;2019:7056872. doi: 10.1155/2019/7056872. PMID: 31391860; PMCID: PMC6662493.	Not related to Oral CHM
311 4	Rabiei K, Ebrahimzadeh MA, Saeedi M, Bahar A, Akha O, Kashi Z. Effects of a hydroalcoholic extract of Juglans regia (walnut) leaves on blood glucose and major cardiovascular risk factors in type 2 diabetic patients: a double-blind, placebo-controlled clinical trial. BMC Complement Altern Med. 2018 Jul 4;18(1):206. doi: 10.1186/s12906-018-2268-8. PMID: 29973195; PMCID: PMC6031195.	Not related to Oral CHM
311 5	Mohammadzadeh-Moghadam H, Nazari SM, Shamsa A, Kamalinejad M, Esmaeeli H, Asadpour AA, Khajavi A. Effects of a Topical Saffron (Crocus sativus L) Gel on Erectile Dysfunction in Diabetics: A Randomized, Parallel-Group, Double-Blind, Placebo-Controlled Trial. J Evid Based Complementary Altern Med. 2015 Oct;20(4):283-6. doi: 10.1177/2156587215583756. Epub 2015 May 6. PMID: 25948674.	Not related to Oral CHM
311 6	Yang X, Liu L, Xiong X, Zhang Y, Liu Y, Li H, Yao K, Wang J. Effects of Bushen-Jiangya granules on blood pressure and pharmacogenomic evaluation in low-to-medium-risk hypertensive patients: study protocol for a randomized double-blind controlled trial. Trials. 2022 Jan 15;23(1):37. doi: 10.1186/s13063-022-05999-2. PMID: 35033168; PMCID: PMC8760657.	Not related to Oral CHM
311 7	Viecili PR, Borges DO, Kirsten K, Malheiros J, Viecili E, Melo RD, Trevisan G, da Silva MA, Bochi GV, Moresco RN, Klafke JZ. Effects of Campomanesia xanthocarpa on inflammatory processes, oxidative stress, endothelial dysfunction and lipid biomarkers in hypercholesterolemic individuals. Atherosclerosis. 2014 May;234(1):85-92. doi: 10.1016/j.atherosclerosis.2014.02.010. Epub 2014 Feb 25. PMID: 24632042.	Not related to Oral CHM

311<br>8	Wang H, Wen Y, Du Y, Yan X, Guo H, Rycroft JA, Boon N, Kovacs EM, Mela DJ. Effects of catechin enriched green tea on body composition. Obesity (Silver Spring). 2010 Apr;18(4):773-9. doi: 10.1038/oby.2009.256. Epub 2009 Aug 13. PMID: 19680234.	Not related to Oral CHM
311<br>9	Tsai FJ, Li TM, Cheng CF, Wu YC, Lai CH, Ho TJ, Liu X, Tsang H, Lin TH, Liao CC, Huang SM, Li JP, Lin JC, Lin CC, Liang WM, Lin YJ. Effects of Chinese herbal medicine on hyperlipidemia and the risk of cardiovascular disease in HIV-infected patients in Taiwan. J Ethnopharmacol. 2018 Jun 12;219:71-80. doi: 10.1016/j.jep.2018.03.006. Epub 2018 Mar 10. PMID: 29530610.	Not related to Oral CHM
312<br>0	Wang TZ, Chen Y, He YM, Fu XD, Wang Y, Xu YQ, Yang HJ, Xue HL, Liu Y, Feng XT, Zhang T, Wang WJ. Effects of Chinese herbal medicine Yiqi Huaju Qingli Formula in metabolic syndrome patients with microalbuminuria: a randomized placebo-controlled trial. J Integr Med. 2013 May;11(3):175-83. doi: 10.3736/jintegrmed2013032. PMID: 23743161.	Not related to Oral CHM
312<br>1	Rong SL, Li DG, Fan HM, Zheng Y. [Effects of Chinese herbs for cool-moistening and freeing collaterals on serum gastrin and surface electrogastrogram in patients of diabetes mellitus with gastroparesis]. Zhongguo Zhong Xi Yi Jie He Za Zhi. 2004 Nov;24(11):976-8. Chinese. PMID: 15609592.	Not related to Oral CHM
312<br>2	Shen T, Guo S, Liang X. [Effects of Chinese herbs xianzhen tablet on the deformability of erythrocyte in non-insulin-dependent diabetes mellitus patients with deficiency of both qi and yin and deficiency of kidney with blood stasis]. Zhongguo Zhong Xi Yi Jie He Za Zhi. 1998 Jul;18(7):405-7. Chinese. PMID: 11477815.	Not related to Oral CHM
312<br>3	Akilen R. Effects of cinnamon consumption on glycemic status, lipid profile and body composition in type 2 diabetic patients. Int J Prev Med. 2013 Mar;4(3):379-80. PMID: 23626898; PMCID: PMC3634180.	Not related to Oral CHM
312<br>4	Deekshith, C., Jois, M., Radcliffe, J., & Thomas, J. (2021). Effects of culinary herbs and spices on obesity: A systematic literature review of clinical trials. Journal of Functional Foods, 81, 104449.	Not related to Oral CHM
312<br>5	Mirtaheri, E., Namazi, N., Alizadeh, M., Sargheini, N., & Karimi, S. (2015). Effects of dried licorice extract with low-calorie diet on lipid profile and atherogenic indices in overweight and obese subjects: A randomized controlled clinical trial. European Journal of Integrative Medicine, 7(3), 287-293.	Not related to Oral CHM
312<br>6	Zhao Q, Jiang J, Hu P. Effects of four traditional Chinese medicines on the pharmacokinetics of simvastatin. Xenobiotica. 2015;45(9):803–810. doi:10.3109/00498254.2015.1019593. PMID:25801058.	Not related to Oral CHM

3127	Roshan H, Nikpayam O, Sedaghat M, Sohrab G. Effects of green coffee extract supplementation on anthropometric indices, glycaemic control, blood pressure, lipid profile, insulin resistance and appetite in patients with the metabolic syndrome: a randomised clinical trial. Br J Nutr. 2018;119(3):250–258. doi:10.1017/S0007114517003439. PMID:29307310.	Not related to Oral CHM
3128	Nikpayam, O., Roshan, H., Sohrab, G., & Sedaghat, M. (2018). Effects of green coffee extract supplementation on oxidative stress, systemic and vascular inflammation in patients with metabolic syndrome: a randomized clinical trial. Iranian Red Crescent Medical Journal, 20(6), 1-10.	Not related to Oral CHM
3129	Xu RX, Wu NQ, Li S, et al. Effects of Hedan Tablet (何丹片) on lipid profile, proprotein convertase subtilisin/kexin type 9 and high-density lipoprotein subfractions in patients with hyperlipidemia: a primary study. Chin J Integr Med. 2016;22(9):660–665. doi:10.1007/s11655-015-2140-3. PMID:25967607.	Not related to Oral CHM
3130	Wu R, Wei F, Qu L, et al. Effects of Keluoxin capsule combined with losartan potassium on diabetic kidney disease: study protocol for a randomized double-blind placebo-controlled multicenter clinical trial. Trials. 2020;21(1):951. doi:10.1186/s13063-020-04852-8. PMID:33228726.	Not related to Oral CHM
3131	Zhang X, Chen X, Tang Y, et al. Effects of medical plants from Zingiberaceae family on cardiovascular risk factors of type 2 diabetes mellitus: a systematic review and meta-analysis of randomized controlled trials. J Food Biochem. 2022;46(7):e14130. doi:10.1111/jfbc.14130. PMID:35332564.	Not related to Oral CHM
3132	Mohammad, S., Fadhil, N., & Mahmood, M. (2021). Effects of metformin and cinnamon on 1, 5 anhydroglucitol, adiponectin and ghrelin on newly diagnosed type 2 diabetes mellitus patients. Jordan Medical Journal, 55(4).	Not related to Oral CHM
3133	Shin, S. O., Seo, H. J., Park, H., & Song, H. J. (2016). Effects of mulberry leaf extract on blood glucose and serum lipid profiles in patients with type 2 diabetes mellitus: A systematic review. European Journal of Integrative Medicine, 8(5), 602-608.	Not related to Oral CHM
3134	Mohebbati R, Abbasnezhad A. Effects of Nigella sativa on endothelial dysfunction in diabetes mellitus: a review. J Ethnopharmacol. 2020;252:112585. doi:10.1016/j.jep.2020.112585. PMID:31972323.	Not related to Oral CHM
3135	Zeng, F. J., Chen, Z. H., Jiang, Q. H., Yang, X. Z., Chen, Y., & Mu, Z. W. (2004). Effects of notoginseng extract and early rehabilitation on the microcirculation and hemorheology in patients with cerebral infarction. Chinese Journal of Clinical Rehabilitation, 8(31), 7078-80.	Not related to Oral CHM
3136	Memon, A. R., Ghanghro, A. B., Shaikh, I. A., Qazi, N., Ghanghro, I. H., & Shaikh, U. (2018). Effects of olive oil and garlic on serum cholesterol and triglycerides levels in the patients of type–II diabetes mellitus. Journal of Liaquat University of Medical & Health Sciences, 17(02), 101-105.	Not related to Oral CHM

3137	Park HS, Cho JH, Kim KW, et al. Effects of Panax ginseng on Obesity in Animal Models: a systematic review and meta-analysis. Evid Based Complement Alternat Med. 2018;2018:2719794. doi:10.1155/2018/2719794. PMID:29861768.	Not related to Oral CHM
3138	Othman NS, Che Roos NA, Aminuddin A, et al. Effects of Piper sarmentosum Roxb. on hypertension and diabetes mellitus: a systematic review and meta-analysis. Front Pharmacol. 2022;13:976247. doi:10.3389/fphar.2022.976247. PMID:36091787	Not related to Oral CHM
3139	Zhou, B. G., Zhao, H. M., Lu, X. Y., Zhou, W., Liu, F. C., Liu, X. K., & Liu, D. Y. (2018). Effect of puerarin regulated mTOR signaling pathway in experimental liver injury. Frontiers in Pharmacology, 9, 1165.	Not related to Oral CHM
3140	Yang, H., Han, L., Sheng, T., He, Q., & Liang, J. (2006). Effects of replenishing qi, promoting blood circulation and resolving phlegm on vascular endothelial function and blood coagulation system in senile patients with hyperlipemia. Journal of Traditional Chinese Medicine= Chung i tsa Chih Ying wen pan, 26(2), 120-124.	Not related to Oral CHM
3141	Singh, R. G., Rathore, S. S., Wani, I. A., Agrawal, A., & Dubey, G. P. (2015). Effects of Salacia oblonga on cardiovascular risk factors in chronic kidney disease patients: a prospective study. Saudi Journal of Kidney Diseases and Transplantation, 26(1), 61-66.	Not related to Oral CHM
3142	Shi, H., Yang, D., Qiao, J., Sun, R., Li, R., Zhu, C., ... & Li, L. (2020). Effects of sang-qi granules on blood pressure and endothelial dysfunction in stage I or II hypertension: study protocol for a randomized double-blind double-simulation controlled trial. Trials, 21, 1-9.	Not related to Oral CHM
3143	Yang M, Hu Z, Yue R. Effects of Sheng-Mai Injection on Diabetes Mellitus: A Systematic Review and Meta-analysis. Endocr Metab Immune Disord Drug Targets. 2023;23(8):1051–1067. doi:10.2174/1871530323666230127121738. PMID:36705242.	Not related to Oral CHM
3144	Xiong, R., Zhao, C., Zhong, M., Zhang, X., & Liu, W. (2020). Effects of Shenqi compound on intestinal microbial metabolites in patients with type 2 diabetes: a protocol for systematic review and meta analysis. Medicine, 99(48), e23017.	Not related to Oral CHM
3145	Zhang, Z., Xing, W., Liu, H., Zhou, Q., Liu, X., & Shang, J. (2022). Effects of Shen-Yuan-Dan on Periprocedural Myocardial Injury and the Number of Peripheral Blood Endothelial Progenitor Cells in Patients with Unstable Angina Pectoris Undergoing Elective Percutaneous Coronary Intervention. Evidence-Based Complementary and Alternative Medicine, 2022(1), 9055585.	Not related to Oral CHM
3146	Stolf AM, Cardoso CC, Acco A. Effects of Silymarin on Diabetes Mellitus Complications: A Review. Phytother Res. 2017;31(3):366–374. doi:10.1002/ptr.5768. PMID:28124457.	Not related to Oral CHM

3147	Mohtashami, R., Huseini, H. F., Nabati, F., Hajiaghaee, R., & Kianbakht, S. (2019). Effects of standardized hydro-alcoholic extract of Vaccinium arctostaphylos leaf on hypertension and biochemical parameters in hypertensive hyperlipidemic type 2 diabetic patients: a randomized, double-blind and placebo-controlled clinical trial. Avicenna journal of phytomedicine, 9(1), 44.	Not related to Oral CHM
3148	Mirenayat, F. S., Hajhashemy, Z., Siavash, M., & Saneei, P. (2023). Effects of sumac supplementation on metabolic markers in adults with metabolic syndrome: a triple-blinded randomized placebo-controlled cross-over clinical trial. Nutrition Journal, 22(1), 25.	Not related to Oral CHM
3149	Yang, X., Zhang, B., Lu, X., Yan, M., Wen, Y., Zhao, T., & Li, P. (2016). Effects of Tangshen Formula on urinary and plasma liver-type fatty acid binding protein levels in patients with type 2 diabetic kidney disease: post-hoc findings from a multi-center, randomized, double-blind, placebo-controlled trial investigating the efficacy and safety of Tangshen Formula in patients with type 2 diabetic kidney disease. BMC Complementary and Alternative Medicine, 16, 1-8.	Not related to Oral CHM
3150	Sang, Y., Wang, X. B., & Han, Q. (1996). Effects of tangshenkang capsule on diabetic nephropathy. Zhongguo Zhong xi yi jie he za zhi Zhongguo Zhongxiyi Jiehe Zazhi= Chinese Journal of Integrated Traditional and Western Medicine, 16(7), 398-401.	Not related to Oral CHM
3151	Wang, X. B., Sang, Y., Han, Q., Guo, B. R., & Liu, X. X. (1997). Effects of Tangshenkang capsule on diabetic nephropathyon diabetic nephropathy. Chinese Journal of Integrated Traditional and Western Medicine, 3, 21-25.	Not related to Oral CHM
3152	Terauchi, M., Akiyoshi, M., Owa, Y., Kato, K., Obayashi, S., & Kubota, T. (2011). Effects of the Kampo medication keishibukuryogan on blood pressure in perimenopausal and postmenopausal women. International Journal of Gynecology & Obstetrics, 114(2), 149-152.	Not related to Oral CHM
3153	Wang, M., Wang, Z., Zhou, J., Sun, W., Wang, Y., Han, M., ... & Wang, Y. (2018). Effects of traditional Chinese herbal medicine in patients with diabetic kidney disease: study protocol for a randomized controlled trial. Trials, 19, 1-9.	Not related to Oral CHM
3154	Xiong, X., Wang, P., Zhang, Y., & Li, X. (2015). Effects of traditional Chinese patent medicine on essential hypertension: a systematic review. Medicine, 94(5), e442.	Not related to Oral CHM
3155	Phimarn, W., Sungthong, B., & Itabe, H. (2021). Effects of Triphala on lipid and glucose profiles and anthropometric parameters: a systematic review. Journal of Evidence-Based Integrative Medicine, 26, 2515690X211011038.	Not related to Oral CHM
3156	Wu, W. H., Wang, H., Zhang, M. P., & Huang, S. M. (2010). Effects of Tripterygium on diabetic nephropathy: a systematic review. Chin J Evid-based Med, 10, 693-699.	Not related to Oral CHM

3157	Yao, W., Wang, L., Chen, Q., Wang, F., & Feng, N. (2020). Effects of valsartan on restenosis in patients with arteriosclerosis obliterans of the lower extremities undergoing interventional therapy: a prospective, randomized, single-blind trial. Medical Science Monitor: International Medical Journal of Experimental and Clinical Research, 26, e919977-1.	Not related to Oral CHM
3158	Guangcan, S., & Ligong, Z. (2015). Effects on type 2 diabetes complicated with pulmonary tuberculosis: regiment of insulin, isoniazid, rifampicin, pyrazinamide and ethambutol versus the regiment plus Qi-boosting and Yin-nourishing decoction of Traditional Chinese Medicine. Journal of Traditional Chinese Medicine, 35(3), 260-265.	Not related to Oral CHM
3159	Zhu, M., Wei, J., Li, Y., Wang, Y., Ren, J., Li, B., ... & Liu, J. (2022). Efficacy and mechanism of buyang huanwu decoction in patients with ischemic heart failure: a randomized, double-blind, placebo-controlled trial combined with proteomic analysis. Frontiers in Pharmacology, 13, 831208.	Not related to Oral CHM
3160	Xu, J., Piao, C., Qu, Y., Liu, T., Peng, Y., Li, Q., ... & Yang, J. (2022). Efficacy and mechanism of Jiedu Tongluo Tiaogan Formula in treating type 2 diabetes mellitus combined with non-alcoholic fatty liver disease: Study protocol for a parallel-armed, randomized controlled trial. Frontiers in Pharmacology, 13, 924021.	Not related to Oral CHM
3161	Wu, H., Tian, J., Dai, D., Liao, J., Wang, X., Wei, X., ... & Tong, X. (2020). Efficacy and safety assessment of traditional Chinese medicine for metabolic syndrome. BMJ Open Diabetes Research and Care, 8(1), e001181.	Not related to Oral CHM
3162	Wang, P., Xiong, X., & Li, S. (2015). Efficacy and safety of a traditional Chinese herbal formula Xuefu Zhuyu decoction for hypertension: a systematic review and meta-analysis. Medicine, 94(42), e1850.	Not related to Oral CHM
3163	Mehrzadi, S., Mirzaei, R., Heydari, M., Sasani, M., Yaqoobvand, B., & Huseini, H. F. (2021). Efficacy and safety of a traditional herbal combination in patients with type II diabetes mellitus: a randomized controlled trial. Journal of dietary supplements, 18(1), 31-43.	Not related to Oral CHM
3164	Zhou, P., Hao, Z., Xu, W., Zhou, X., & Yu, J. (2022). Efficacy and safety of abelmoschus moschatus capsules combined with tripterygium glycoside tablets on diabetic nephropathy: a systematic review and meta-analysis. Frontiers in Pharmacology, 13, 936678.	Not related to Oral CHM
3165	Shi, Y., Liu, L., Sun, X., & Jiao, J. (2021). Efficacy and safety of acupuncture combined Chinese herbal medicine for diabetic peripheral neuropathy: A protocol for systematic review and meta-analysis. Medicine, 100(50), e28086.	Not related to Oral CHM
3166	Yu, Z., Zhang, W., Li, B., Bao, P., Wang, F., Sun, J., ... & Nan, Z. (2021). Efficacy and safety of acupuncture combined with Chinese Herbal Medicine for diabetic nephropathy: A protocol for systematic review and meta-analysis. Medicine, 100(35), e27087.	Not related to Oral CHM

3167	Vuksan V, Sievenpiper JL, Xu Z, et al. American ginseng (Panax quinquefolius L.) reduces postprandial glycemia in non-diabetic subjects and subjects with type 2 diabetes mellitus. Arch Intern Med. 2000;160(7):1009-1013. doi:10.1001/archinte.160.7.1009. PMID:10761958.	Not related to Oral CHM
3168	Park, S., Keum, D., & Kim, H. (2022). Efficacy and safety of anti-obesity herbal medicine focused on pattern identification: a systematic review and meta-analysis. Medicine, 101(50), e32087.	Not related to Oral CHM
3169	Sheng, X., Dong, Y., Cheng, D., Wang, N., & Guo, Y. (2020). Efficacy and safety of Bailing capsules in the treatment of type 2 diabetic nephropathy: a meta-analysis. Annals of Palliative Medicine, 9(6), 3885898-3883898.	Not related to Oral CHM
3170	Zhao, J., Mo, C., Meng, L. F., Liang, C. Q., Cao, X., & Shi, W. (2019). Efficacy and safety of Buyang Huanwu Decoction for early-stage diabetic nephropathy: a Meta-analysis. Zhongguo Zhong yao za zhi= Zhongguo zhongyao zazhi= China journal of Chinese materia medica, 44(8), 1660-1667.	Not related to Oral CHM
3171	Xiong, X., Yang, X., Li, X., Yue, G., Xing, Y., & Cho, W. C. (2019). Efficacy and safety of Chinese herbal medicine for patients with postmenopausal hypertension: A systematic review and meta-analysis. Pharmacological Research, 141, 481-500.	Not related to Oral CHM
3172	Xiong, X., Wang, P., Duan, L., Liu, W., Chu, F., Li, S., ... & Xing, Y. (2019). Efficacy and safety of Chinese herbal medicine Xiao Yao San in hypertension: a systematic review and meta-analysis. Phytomedicine, 61, 152849.	Not related to Oral CHM
3173	Park, E., Lee, C. G., Kim, J., Kang, J. H., Cho, Y. G., & Jeong, S. Y. (2020). Efficacy and safety of combined extracts of cornus officinalis and Ribes fasciculatum for body fat reduction in overweight women. Journal of Clinical Medicine, 9(11), 3629.	Not related to Oral CHM
3174	Wang, L., Xu, J., Yu, T., Wang, H., Cai, X., & Sun, H. (2023). Efficacy and safety of curcumin in diabetic retinopathy: A protocol for systematic review and meta-analysis. Plos one, 18(4), e0282866.	Not related to Oral CHM
3175	Shekelle PG, Hardy ML, Morton SC, et al. Efficacy and safety of ephedra and ephedrine for weight loss and athletic performance: a meta-analysis. JAMA. 2003;289(12):1537-1545. doi:10.1001/jama.289.12.1537. PMID:12672771.	Not related to Oral CHM
3176	Suthar, A. C., Deshmukh, A., Babu, V., Mohan, V. S., Chavan, M. V., Kumar, D., ... & Sharma, M. (2016). Efficacy and safety of Glycebal (PDM011011) capsules as adjuvant therapy in subjects with type 2 diabetes mellitus: an open label, randomized, active controlled, phase II trial. Clinical Diabetology, 5(3), 88-94.	Not related to Oral CHM
3177	Song, J., Shin, S. M., & Kim, H. (2019). Efficacy and safety of HT048 and HT077 for body fat and weight loss in overweight adults: a study protocol for a double-blind, randomized, placebo-controlled trial. Medicine, 98(45), e17922.	Not related to Oral CHM

3178	Methods in Medicine, C. A. M. (2023). Retracted: Efficacy and Safety of Jinshuibao Capsule in Diabetic Nephropathy: A Systematic Review and Meta-Analysis of Randomized Controlled Trials.	Not related to Oral CHM
3179	Norhayati, M. N., George, A., Nik Hazlina, N. H., Azidah, A. K., Intan Idiana, H., Law, K. S., ... & Azreena, A. (2014). Efficacy and safety of Labisia pumila var alata water extract among pre-and postmenopausal women. Journal of Medicinal Food, 17(8), 929-938.	Not related to Oral CHM
3180	Wu, J., Zhang, X., & Zhang, B. (2014). Efficacy and safety of puerarin injection in treatment of diabetic peripheral neuropathy: A systematic review and meta-analysis of randomized controlled trials. Journal of Traditional Chinese Medicine, 34(4), 401-410.	Not related to Oral CHM
3181	Zhou, C., Long, B., Huang, W., & Jiang, L. (2021). Efficacy and safety of qiming granule combined with laser in the treatment of diabetic retinopathy: a protocol of randomized controlled trial. Medicine, 100(12), e25158.	Not related to Oral CHM
3182	Xie, F., Zhang, B., Dai, S., Jin, B., Zhang, T., & Dong, F. (2021). Efficacy and safety of Salvia miltiorrhiza (Salvia miltiorrhiza Bunge) and ligustrazine injection in the adjuvant treatment of early-stage diabetic kidney disease: A systematic review and meta-analysis. Journal of Ethnopharmacology, 281, 114346.	Not related to Oral CHM
3183	Xie, F., Zhang, B., Dai, S., Jin, B., Zhang, T., & Dong, F. (2021). Efficacy and safety of Salvia miltiorrhiza (Salvia miltiorrhiza Bunge) and ligustrazine injection in the adjuvant treatment of early-stage diabetic kidney disease: A systematic review and meta-analysis. Journal of Ethnopharmacology, 281, 114346.	Not related to Oral CHM
3184	Wu, Y., Li, S., Li, Z., Mo, Z., Luo, Z., Li, D., ... & Ding, B. (2022). Efficacy and safety of Shenfu injection for the treatment of post-acute myocardial infarction heart failure: a systematic review and meta-analysis. Frontiers in Pharmacology, 13, 1027131.	Not related to Oral CHM
3185	Xu, J., Zhang, C., Shi, X., Li, J., Liu, M., Jiang, W., & Fang, Z. (2019). Efficacy and safety of sodium tanshinone IIA sulfonate injection on hypertensive nephropathy: a systematic review and meta-analysis. Frontiers in Pharmacology, 10, 1542.	Not related to Oral CHM
3186	Meng, T., Wang, P., Xie, X., Li, T., Kong, L., Xu, Y., ... & Lai, X. (2022). Efficacy and safety of songling xuemaikang capsule for essential hypertension: A systematic review and meta-analysis of randomized controlled trials. Phytomedicine, 107, 154459.	Not related to Oral CHM
3187	Park, S., Nahmkoong, W., Cheon, C., Park, J. S., Jang, B. H., Shin, Y., ... & Ko, S. G. (2013). Efficacy and safety of Taeeumjowi-tang in obese Korean adults: a double-blind, randomized, and placebo-controlled pilot trial. Evidence-Based Complementary and Alternative Medicine, 2013(1), 498935.	Not related to Oral CHM

318 8	Wang, Y., Hua, Z., Chen, W., Zhu, Y., & Li, Y. (2022). Efficacy and safety of Tengfu Jiangya tablet combined with valsartan/amlodipine in the treatment of stage 2 hypertension: study protocol for a randomized controlled trial. Trials, 23(1), 171.	Not related to Oral CHM
318 9	Peng, S., Xie, Z., Zhang, X., Xie, C., Kang, J., Yuan, H., ... & Liu, Y. (2021). Efficacy and Safety of the Chinese Patent Medicine Yuquan Pill on Type 2 Diabetes Mellitus Patients: A Systematic Review and Meta-Analysis. Evidence-Based Complementary and Alternative Medicine, 2021(1), 2562590.	Not related to Oral CHM
319 0	Xie, B., Wang, Q., Zhou, C., Wu, J., & Xu, D. (2018). Efficacy and safety of the injection of the traditional Chinese medicine puerarin for the treatment of diabetic peripheral neuropathy: a systematic review and meta-analysis of 53 randomized controlled trials. Evidence-Based Complementary and Alternative Medicine, 2018(1), 2834650.	Not related to Oral CHM
319 1	Tang, Z., Sun, Y., Wang, C., Liu, X., Qi, X., Ma, D., & Zhao, H. (2020). Efficacy and safety of the traditional Chinese medicine tonifying kidney (bu shen) therapy in patients with hypertension: A protocol for systematic review and meta-analysis. Medicine, 99(29), e21144.	Not related to Oral CHM
319 2	Sun, L. X., Li, Y. Y., & Xie, Y. M. (2023). Efficacy and safety of Tongmai Jiangtang capsule combined with conventional therapy in the treatment of diabetic peripheral neuropathy: a systematic review and meta-analysis. Frontiers in Neurology, 14, 1100327.	Not related to Oral CHM
319 3	Tao, SY, Zhang, J, Yu, LT, Zhang, LX and Huang, L, 2021. Efficacy and safety of traditional Chinese medicine combined with western medicine in treating essential hypertension complicated with atrial fibrillation: AMeta-analysis, Chinese Traditional and Herbal Drugs	Not related to Oral CHM
319 4	Wang, Z., Tang, Z., Zhu, W., Ge, L., & Ge, J. (2017). Efficacy and safety of traditional Chinese medicine on thromboembolic events in patients with atrial fibrillation: a systematic review and meta-analysis. Complementary Therapies in Medicine, 32, 1-10.	Not related to Oral CHM
319 5	Wen, Z. G., Zhang, Q. Q., Zhang, L. L., Shen, M. F., Huang, Y. S., & Zhao, L. H. (2022). Efficacy and safety of traditional chinese medicine treatment for overweight and obese individuals: A systematic review and meta-analysis. Frontiers in pharmacology, 13, 964495.	Not related to Oral CHM
319 6	Zhao, J. N., Zhang, Y., Lan, X., Chen, Y., Li, J., Zhang, P., ... & Xu, F. Q. (2019). Efficacy and safety of Xinnaoning capsule in treating chronic stable angina (qi stagnation and blood stasis syndrome): study protocol for a multicenter, randomized, double-blind, placebo-controlled trial. Medicine, 98(31), e16539.	Not related to Oral CHM
319 7	Zhang, S., Chen, Z. L., Tang, Y. P., Duan, J. L., & Yao, K. W. (2021). Efficacy and Safety of Xue-Fu-Zhu-Yu Decoction for Patients with Coronary Heart Disease: A Systematic Review and Meta-Analysis. Evidence-Based Complementary and Alternative Medicine, 2021(1), 9931826.	Not related to Oral CHM

319 8	Wu, Z., Wu, D., Jiang, J., Chen, A., Zheng, D. D., Li, J., ... & Chen, Y. (2020). Efficacy and safety of xuezhikang once per day versus two times per day in patients with mild to moderate hypercholesterolaemia (APEX study): a protocol for a multicentre, prospective randomised controlled, open-label, non-inferiority study. BMJ open, 10(5), e034585.	Not related to Oral CHM
319 9	Wang, C., Pang, W., Du, X., Zhai, J., Zhong, M., Zhuang, M., ... & Zhang, J. (2022). Efficacy and safety of zhibitai in the treatment of hyperlipidemia: A systematic review and meta-analysis. Frontiers in Pharmacology, 13, 974995.	Not related to Oral CHM
320 0	Stern, J. S., Peerson, J., Mishra, A. T., Sadasiva Rao, M. V., & Rajeswari, K. P. (2013). Efficacy and tolerability of a novel herbal formulation for weight management. Obesity, 21(5), 921-927.	Not related to Oral CHM
320 1	Rabe, E., Stücker, M., Esperester, A., Schäfer, E., & Ottillinger, B. (2011). Efficacy and tolerability of a red-vine-leaf extract in patients suffering from chronic venous insufficiency–results of a double-blind placebo-controlled study. European Journal of Vascular and Endovascular Surgery, 41(4), 540-547.	Not related to Oral CHM
320 2	Stern, J. S., Peerson, J., Mishra, A. T., Mathukumalli, V. S. R., & Konda, P. R. (2013). Efficacy and tolerability of an herbal formulation for weight management. Journal of medicinal food, 16(6), 529-537.	Not related to Oral CHM
320 3	Yan, Z., Jian-hua, W., & LI, Y. (2017). Efficacy evaluation of compound Xueshuantong capsule combined with calcium dobesilate in patients with early diabetic retinopathy. International Eye Science, 2147-2150.	Not related to Oral CHM
320 4	Opala, T., Rzymski, P., Pischel, I., Wilczak, M., & Wozniak, J. (2006). Efficacy of 12 weeks supplementation of a botanical extract-based weight loss formula on body weight, body composition and blood chemistry in healthy, overweight subjects-a randomised double-blind placebo-controlled clinical trial. European journal of medical research, 11(8), 343.	Not related to Oral CHM
320 5	Preuss HG, Bagchi D, Bagchi M, et al. Effects of a natural extract of (-)-hydroxycitric acid (HCA-SX) and a combination of HCA-SX plus niacin-bound chromium and Gymnema sylvestre extract on weight loss. Diabetes Obes Metab. 2004;6(3):171-180. doi:10.1111/j.1463-1326.2004.00326.x. PMID:15117297.	Not related to Oral CHM
320 6	Zheng, X., Zhang, S., Wang, Z., & Zou, D. (2021). Efficacy of acupuncture combined with Chinese herbal medicine for the treatment of chronic nephritis: A protocol for systematic review and meta-analysis. Medicine, 100(44), e27687.	Not related to Oral CHM
320 7	Zojaji, S. A., Mozaffari, H. M., Ghaderi, P., Zojaji, F., Hadjzadeh, M. A. R., Seyfimoqadam, M., & Ghorbani, A. (2022). Efficacy of an herbal compound in decreasing steatosis and transaminase activities in non-alcoholic fatty liver disease: a randomized clinical trial. Brazilian Journal of Pharmaceutical Sciences, 58, e19825.	Not related to Oral CHM

3208	Fa-lin, Z., Zhen-yu, W., Yan, H., Tao, Z., & Kang, L. (2010). Efficacy of blackcurrant oil soft capsule, a Chinese herbal drug, in hyperlipidemia treatment. Phytotherapy Research, 24(S2), S209-S213.	Not related to Oral CHM
3209	Lian, F., Tian, J., Jin, D., Piao, C., Guo, H., Zhang, J., ... & Tong, X. (2021). Efficacy of Chinese Herbal Medicine Compared with Metformin for Glucose Regulation and Cardiovascular Risk Factors in Patients with Type 2 Diabetes with Dyslipidaemia: A Multicenter, Randomised Clinical Trial.	Not related to Oral CHM
3210	Zheng, Y., Yang, F., Han, L., Gou, X., Lian, F., Liu, W., ... & Tong, X. (2019). Efficacy of Chinese herbal medicine in the treatment of moderate-severe painful diabetic peripheral neuropathy: a retrospective study. Journal of diabetes research, 2019(1), 4035861.	Not related to Oral CHM
3211	Shi, R., Wang, Y., An, X., Ma, J., Wu, T., Yu, X., ... & Sun, Z. (2019). Efficacy of co-administration of liuwei dihuang pills and ginkgo biloba tablets on albuminuria in type 2 diabetes: a 24-month, multicenter, double-blind, placebo-controlled, randomized clinical trial. Frontiers in Endocrinology, 10, 100.	Not related to Oral CHM
3212	Wang, J., Mao, Y., Jiang, C., He, M., Tan, Q., & Zheng, W. (2023). Efficacy of combined application of traditional Chinese medicine foot bath and mild moxibustion in the treatment of limb pain in diabetic peripheral neuropathy patients. Tropical Journal of Pharmaceutical Research, 22(1), 175-181.	Not related to Oral CHM
3213	Zhang, L., Tian, X., Ma, Y., Jin, Y. H., & Meng, F. J. (2015). Efficacy of combining traditional Chinese medicine fumigation with Western medicine for diabetic peripheral neuropathy: a systematic review and meta-analysis. International Journal of Nursing Sciences, 2(3), 295-303.	Not related to Oral CHM
3214	Gong G, Liu Y, He W, Xu B, Wu J, Ding J. Efficacy of Cordyceps sinensis as an adjunctive treatment in kidney transplant patients: a systematic review and meta-analysis. Exp Ther Med. 2017;13(5):1821-1831. doi:10.3892/etm.2017.4218. PMID:28450973.	Not related to Oral CHM
3215	Zhao, Q. Y., Tang, R. H., Lu, G. X., Cao, X. Z., Liu, L. R., Zhang, J. H., ... & Chen, Z. G. (2021). Efficacy of Getong Tongluo Capsule (葛酮通络胶囊) for Convalescent-Phase of Ischemic Stroke and Primary Hypertension: A Multicenter, Randomized, Double-Blind, Controlled Trial. Chinese journal of integrative medicine, 27, 252-258.	Not related to Oral CHM
3216	Pang, J., & Zhong, R. (2023). Efficacy of Modified Zuoguiwan Combined with Perindopril Tert-butylamine Tablets on Early Diabetic Kidney Disease Patients of Qi-Yin Deficiency with Blood Stasis Syndrome. Chinese Journal of Experimental Traditional Medical Formulae, 29(1), 105-112.	Not related to Oral CHM
3217	Zhang, Y., Jin, D., Duan, Y., Hao, R., Chen, K., Yu, T., ... & Tong, X. (2021). Efficacy of mudan granule (combined with methylcobalamin) on type 2 diabetic peripheral neuropathy: study protocol for a Double-Blind, randomized, Placebo-Controlled, Parallel-Arm, Multi-Center trial. Frontiers in Pharmacology, 12, 676503.	Not related to Oral CHM

3218	Yao, Z., Zhang, L., & Ji, G. (2014). Efficacy of polyphenolic ingredients of Chinese herbs in treating dyslipidemia of metabolic syndromes. Journal of integrative medicine, 12(3), 135-146.	Not related to Oral CHM
3219	Seyed Hashemi, M., Namiranian, N., Tavahen, H., Dehghanpour, A., Rad, M. H., Jam-Ashkezari, S., ... & Hashempur, M. H. (2021). Efficacy of pomegranate seed powder on glucose and lipid metabolism in patients with type 2 diabetes: a prospective randomized double-blind placebo-controlled clinical trial. Complementary Medicine Research, 28(3), 226-233.	Not related to Oral CHM
3220	Ye M, Liu J, Lou Y, Sun L. Red yeast rice for myocardial infarction patients with borderline hypercholesterolemia: a meta-analysis of randomized controlled trials. Complement Ther Med. 2020;53:102524. doi:10.1016/j.ctim.2020.102524. PMID:32858269.	Not related to Oral CHM
3221	Zhang, Y., Xie, H. Y., Tian, Y., Zhou, L. Y., Yan, R., & Xie, C. G. (2019). Efficacy of Shenqi compoundparticle on blood glucose and oxidative stress compared with metformin for patients with newly siagnosed type 2 diabetes mellitus: randomized clinical trial. International Journal of Clinical and Experimental Medicine, 12(7), 8271-8280.	Not related to Oral CHM
3222	Jiaxing, T., Linhua, Z., Qiang, Z., Wenke, L., Xinyan, C., Fengmei, L., & Xiaolin, T. (2015). Efficacy of Shenzhuo formula on diabetic kidney disease: a retrospective study. Journal of traditional Chinese medicine, 35(5), 528-536.	Not related to Oral CHM
3223	Yao, XS, 2011. Efficacy of Tangmaikang granule combined with Mecobalamin on diabetic peripheral neuropathy, Chinese Journal of New Drugs.	Not related to Oral CHM
3224	Razavi BN, Hosseini A, Javanbakht MH, et al. The effect of hydroalcoholic extract of Tribulus terrestris on serum glucose and lipid profile in women with diabetes mellitus: a randomized controlled clinical trial. J Res Med Sci. 2021;26:43. doi:10.4103/jrms.JRMS_389_20. PMID:34276514.	Not related to Oral CHM
3225	Zhang, Q, Yang, X, Zhang, X and Shang, H, 2018. Efficacy of traditional Chinese medicine for arrhythmia caused by anthracycline drugs: A systematic review, Chinese Journal of Evidence-Based Medicine	Not related to Oral CHM
3226	Razmgah, G. R. G., Hosseini, S. M. R., Nematy, M., Esmaily, H., Yousefi, M., Kamalinejad, M., & Mosavat, S. H. (2017). Efficacy of traditional Persian medicine-based diet on non-alcoholic fatty liver disease: a randomized, controlled, clinical trial. Galen Medical Journal, 6(3), 208-216.	Not related to Oral CHM
3227	Wu, X., Huang, Y., Zhang, Y., He, C., Zhao, Y., Wang, L., & Gao, J. (2020). Efficacy of tripterygium glycosides combined with ARB on diabetic nephropathy: a meta-analysis. Bioscience reports, 40(11).	Not related to Oral CHM
3228	Li X, Zhang Y, Jiang W, et al. Efficacy of Wenxin Keli plus amiodarone versus amiodarone monotherapy in treating recent-onset atrial fibrillation. Int J Clin Exp Med. 2015;8(9):15429-15434. PMID:26629181.	Not related to Oral CHM

3229	Yan Z, Yang J, Li L, et al. Efficacy, safety, and mechanisms of herbal medicines used in the treatment of obesity: a systematic review. Front Pharmacol. 2022;13:874012. doi:10.3389/fphar.2022.874012. PMID:35865706.	Not related to Oral CHM
3230	Song, Y., Wang, H., Qin, L., Li, M., Gao, S., Wu, L., & Liu, T. (2020). Efficiency and Safety of Chinese Herbal Medicine in the Treatment of Prediabetes: A Systemic Review and Meta-Analysis of Randomized Controlled Trials. Evidence-Based Complementary and Alternative Medicine, 2020(1), 3628036.	Not related to Oral CHM
3231	Xie, W., Zhao, Y., & Du, L. (2012). Emerging approaches of traditional Chinese medicine formulas for the treatment of hyperlipidemia. Journal of Ethnopharmacology, 140(2), 345-367.	Not related to Oral CHM
3232	Wang, W. J. (2013). Enhancing the treatment of metabolic syndrome with integrative medicine. Journal of integrative medicine, 11(3), 153-156.	Not related to Oral CHM
3233	Xu, H. B., Jiang, R. H., Chen, X. Z., & Li, L. (2012). Chinese herbal medicine in treatment of diabetic peripheral neuropathy: a systematic review and meta-analysis. Journal of ethnopharmacology, 143(2), 701-708.	Not related to Oral CHM
3234	Mohamad, A. S., Nordin, M. N., Ani, I. C., Jemberang, J., Ishak, R., Hasan, A. N., ... & Hamzah, R. (2021). Evaluating the Effect of Volten VR4 庐 Kaempferia parviflora Extracts on Blood Glucose Levels in Human Type-2 Diabetes Mellitus and Healthy Individual: A Case-Control Study.聽Journal of the Medical Association of Thailand,聽 104(10).	Not related to Oral CHM
3235	Shafiezadeh, R., Alavian, S. M., Namdar, H., Gholami-Fesharaki, M., & Esmaeili, S. S. (2020). Evaluating the efficacy of Carum copticum seeds on the treatment of patients with nonalcoholic fatty liver disease: A multi-center, randomized, triple–blind, placebo-controlled clinical trial study. Hepatitis Monthly, 20(12), 1-8.	Not related to Oral CHM
3236	Zhang, Z., Qi, Y., Wang, X., Jin, Q., Kong, W., Li, H., ... & Zhao, H. (2016, November). Evaluation analysis using FibroScan-CAP of Qingrequzhuo capsule for the treatment of type 2 diabetes mellitus with nonalcoholic fatty liver disease. In DIABETES-METABOLISM RESEARCH AND REVIEWS (Vol. 32, No. SUPP 2, pp. 68-69). 111 RIVER ST, HOBOKEN 07030-5774, NJ USA: WILEY-BLACKWELL.	Not related to Oral CHM
3237	Tian JiaXing, T. J., Lian FengMei, L. F., Yang LiBo, Y. L., & Tong XiaoLin, T. X. (2018). Evaluation of the Chinese herbal medicine Jinlida in type 2 diabetes patients based on stratification: results of subgroup analysis from a 12-week trial.	Not related to Oral CHM
3238	Wei, Q., Jing, Z., & Zhi-Ye, H. (2021). Evaluation of Danzhi Xiaoyao powder and amlodipine sustained-release tablets in follow-up treatment of the hypertensive crisis and the interleukin-6 gene expression. Cellular and Molecular Biology, 67(2), 166-170.	Not related to Oral CHM

3239	Sharma, R. K., Sharma, B., Jindal, M., Gupta, A. K., Kunwar, R., Lata, S., & Yadav, A. K. (2017). Evaluation of hypolipidemic effect of stem part of Berberis aristata in Type 2 diabetes mellitus patients as add on therapy. National journal of physiology, pharmacy and pharmacology, 7(11), 1159-1159.	Not related to Oral CHM
3240	Shivakumar, S., Ilango, K., Dubey, G. P., Subhasree, N., & Agrawal, A. (2015). Evaluation of plant based formulation on adolescent obesity and its associated bio-markers: A randomized, double blind, placebo controlled study. Complementary Therapies in Medicine, 23(2), 157-164.	Not related to Oral CHM
3241	Li X, Du X, Zhang Y, et al. Efficacy of Shensongyangxin capsules in the treatment of paroxysmal atrial fibrillation: a randomized, double-blind, controlled multicenter clinical trial. Chin Med J (Engl). 2010;123(6):694-700. PMID:20368622.	Not related to Oral CHM
3242	Tian JiaXing, T. J., Lian FengMei, L. F., Yang LiBo, Y. L., & Tong XiaoLin, T. X. (2018). Evaluation of the Chinese herbal medicine Jinlida in type 2 diabetes patients based on stratification: results of subgroup analysis from a 12-week trial.	Not related to Oral CHM
3243	Soltani, R., Hashemi, M., Farazmand, A., Asghari, G., Heshmat-Ghahdarijani, K., Kharazmkia, A., & Ghanadian, S. M. (2017). Evaluation of the effects of Cucumis sativus seed extract on serum lipids in adult hyperlipidemic patients: A randomized double-blind placebo-controlled clinical trial. Journal of food science, 82(1), 214-218.	Not related to Oral CHM
3244	Xiong, C., Li, L., Bo, W., Chen, H., XiaoWei, L., Hongbao, L., & Peng, Z. (2020). Evaluation of the efficacy and safety of TWHF in diabetic nephropathy patients with overt proteinuria and normal eGFR. Journal of the Formosan Medical Association, 119(3), 685-692.	Not related to Oral CHM
3245	Vasques, C. A., Rossetto, S., Halmenschlager, G., Linden, R., Heckler, E., Fernandez, M. S. P., & Alonso, J. L. L. (2008). Evaluation of the pharmacotherapeutic efficacy of Garcinia cambogia plus Amorphophallus konjac for the treatment of obesity. Phytotherapy Research, 22(9), 1135-1140.	Not related to Oral CHM
3246	Wei, Y., Ding, Q. Y., Yeung, C., Huang, Y. S., Zhang, B. X., Zhang, L. L., ... & Tong, X. L. (2022). Evidence and Potential Mechanisms of Traditional Chinese Medicine for the Adjuvant Treatment of Coronary Heart Disease in Patients with Diabetes Mellitus: A Systematic Review and Meta-Analysis with Trial Sequential Analysis. Journal of Diabetes Research, 2022(1), 2545476.	Not related to Oral CHM
3247	Tian, J., Jin, D., Bao, Q., Ding, Q., Zhang, H., Gao, Z., ... & Tong, X. (2019). Evidence and potential mechanisms of traditional Chinese medicine for the treatment of type 2 diabetes: a systematic review and meta-analysis. Diabetes, Obesity and Metabolism, 21(8), 1801-1816.	Not related to Oral CHM
3248	Vatanparast H, Islam N, Patil RP, et al. Examining the DASH Diet in Chinese Canadians (DASH-CC): a pilot randomized controlled trial.	Not related to Oral CHM

	Nutrients. 2019;11(11):2657. doi:10.3390/nu11112657. PMID:31683853.	
3249	Wang, L. Y., Chan, K. W., Yuwen, Y., Shi, N. N., Han, X. J., & Lu, A. (2013). Expert consensus on the treatment of hypertension with Chinese patent medicines. Evidence-Based Complementary and Alternative Medicine, 2013(1), 510146.	Not related to Oral CHM
3250	Nahvinejad, M., Pourrajab, F., & Hekmatimoghaddam, S. (2016). Extract of Dorema aucheri induces PPAR-γ for activating reactive oxygen species metabolism. Journal of Herbal Medicine, 6(4), 171-179.	Not related to Oral CHM
3251	Williams JA, Rao A, Kendall CW, et al. Glycemia-lowering effect of a Salacia oblonga extract in patients with type 2 diabetes. Diabetes Care. 2007;30(7):1799-1801. doi:10.2337/dc06-2580. PMID:17456842.	Not related to Oral CHM
3252	Xie, XS and Huang, ZW, 2006. Feasibility and advantage of traditional Chinese medicine in the intervention of impaired glucose tolerance, Chinese Journal of Clinical Rehabilitation	Not related to Oral CHM
3253	Perrinjaquet-Moccetti T, Wang Q, Gauthier A, Ivleva S, Mensink RP, Haddad PS. Food supplementation with an olive (Olea europaea L.) leaf extract reduces blood pressure in borderline hypertensive monozygotic twins. Phytother Res. 2008;22(9):1239-1242. doi:10.1002/ptr.2475. PMID:18570191.	Not related to Oral CHM
3254	Lai JN, Wu CT, Wang JD. Prescription patterns of Chinese herbal products for hypertension in Taiwan: a population-based study. J Ethnopharmacol. 2012;140(3): 668-676. doi:10.1016/j.jep.2012.02.032. PMID:22387028.	Not related to Oral CHM
3255	Tu, X., Xie, C., Wang, F., Chen, Q., Zuo, Z., Zhang, Q., ... & Jordan, J. B. (2013). Fructus mume formula in the treatment of type 2 diabetes mellitus: a randomized controlled pilot trial. Evidence-Based Complementary and Alternative Medicine, 2013(1), 787459.	Not related to Oral CHM
3256	Tan, Y. M., Hu, J., Wu, Q., Zhang, Y., Suo, W. D., Zhou, Y. T., ... & Ni, Q. (2020). Fufang Xueshuantong for Diabetic Kidney Disease: A Systematic Review and Meta-Analysis. Evidence-Based Complementary and Alternative Medicine, 2020(1), 9326948.	Not related to Oral CHM
3257	Silagy C, Neil A. Garlic as a lipid lowering agent--a meta-analysis. J R Coll Physicians Lond. 1994;28(1):39-45. PMID:8176414.	Not related to Oral CHM
3258	Ried K, Fakler P, Stocks NP. Effect of garlic on blood pressure: a systematic review and meta-analysis. BMC Cardiovasc Disord. 2008;8:13. doi:10.1186/1471-2261-8-13. PMID:18405346.	Not related to Oral CHM
3259	Mozaffari-Khosravi H, Alizadeh-Otaghvar HR, Fallahzadeh H, Nadjarzadeh A. The effect of ginger powder supplementation on insulin resistance and glycemic indices in patients with type 2 diabetes: a randomized, double-blind, placebo-controlled trial. Complement Ther Med. 2014;22(1):9-16. doi:10.1016/j.ctim.2013.11.006. PMID:24411115.	Not related to Oral CHM

3260	Tavares, L., Smaoui, S., Pinilla, C. M. B., Hlima, H. B., & Barros, H. L. (2022). Ginger: a systematic review of clinical trials and recent advances in encapsulation of its bioactive compounds. Food & Function, 13(3), 1078-1091.	Not related to Oral CHM
3261	Qiu, J., Guo, Y., Xu, X., Yue, H., & Yang, Y. (2020). Ginkgo leaf extract and dipyridamole injection for chronic cor pulmonale: a PRISMA-compliant meta-analysis of randomized controlled trials. Bioscience Reports, 40(3), BSR20200099.	Not related to Oral CHM
3262	Zhou, M., Yu, R., Liu, X., Lv, X., & Xiang, Q. (2022). Ginseng-plus-Bai-Hu-Tang Combined with Western Medicine for the Treatment of Type 2 Diabetes Mellitus: A Systematic Review and Meta-Analysis. Evidence-Based Complementary and Alternative Medicine, 2022(1), 9572384.	Not related to Oral CHM
3263	Tharavanij, T., Pawa, K. K., Maungboon, P., Panpitpat, P., Porntisan, S., Thangcharoende, W., ... & Jesadanont, S. (2015). Glucose-lowering efficacy of water extract of malvastrum coromandelianum in type 2 diabetes subjects: A double blind, randomized controlled trial. J. Med. Assoc. Thail. Chotmaihet Thangphaet, 98(Suppl 3), S75-S80.	Not related to Oral CHM
3264	Marini H, Polito F, Adamo EB, Bitto A, Squadrito F, Benvenga S. Effects of the phytoestrogen genistein on bone metabolism in osteopenic postmenopausal women: a randomized trial. Ann Intern Med. 2007;146(12):839-847. doi:10.7326/0003-4819-146-12-200706190-00005. PMID:17577002.	Not related to Oral CHM
3265	Crew KD, Brown P, Greenlee H, et al. Phase IB randomized, double-blinded, placebo-controlled, dose escalation study of epigallocatechin-3-gallate (EGCG) in patients with breast cancer. Cancer Prev Res (Phila). 2012;5(9):1144-1154. doi:10.1158/1940-6207.CAPR-12-0107. PMID:22879388.	Not related to Oral CHM
3266	Ulbricht C, Basch E, Szapary P, et al. Guggul for hyperlipidemia: a review by the Natural Standard Research Collaboration. Complement Ther Med. 2005;13(4):279-290. doi:10.1016/j.ctim.2005.08.008. PMID:16338122.	Not related to Oral CHM
3267	Rawat, S., Jugran, A. K., Bhatt, I. D., & Rawal, R. S. (2018). Hedychium spicatum: a systematic review on traditional uses, phytochemistry, pharmacology and future prospectus. Journal of Pharmacy and Pharmacology, 70(6), 687-712.	Not related to Oral CHM
3268	Wickramasinghe, ASD, Kalansuriya, P and Attanayake, AP, 2021. Herbal Medicines Targeting the Improved β -Cell Functions and β -Cell Regeneration for the Management of Diabetes Mellitus, Evidence-based Complementary and Alternative Medicine	Not related to Oral CHM
3269	Wanjari MM, Wanjari AK, Bodhankar SL, et al. Herb-drug interactions in diabetes mellitus: A review based on preclinical and clinical data. Phytomedicine. 2021;84:153505. doi:10.1016/j.phymed.2021.153505. PMID:33991517.	Not related to Oral CHM

3270	Yeh GY, Eisenberg DM, Kaptchuk TJ, Phillips RS. Systematic review of herbs and dietary supplements for glycemic control in diabetes. Diabetes Care. 2003;26(4):1277-1294. doi:10.2337/diacare.26.4.1277. PMID:12663610.	Not related to Oral CHM
3271	Tu, X., Liu, F., Jordan, J. B., Ye, X. F., Fu, P., Wang, F., & Zhong, S. (2013). 'Huang Qi Elixir'for proteinuria in patients with diabetic nephropathy: a study protocol for a randomized controlled pilot trial. Trials, 14, 1-5.	Not related to Oral CHM
3272	Zhang, Y., Gong, G., Zhang, X., Zhou, L., Xie, H., Tian, Y., & Xie, C. (2019). Huangqi Guizhi Wuwu decoction for diabetic peripheral neuropathy: Protocol for a systematic review. Medicine, 98(31), e16696.	Not related to Oral CHM
3273	Pang, B., Zhao, T. Y., Zhao, L. H., Wan, F., Ye, R., Zhou, Q., ... & Tong, X. L. (2016). Huangqi Guizhi Wuwu Decoction for treating diabetic peripheral neuropathy: a meta-analysis of 16 randomized controlled trials. Neural regeneration research, 11(8), 1347-1358.	Not related to Oral CHM
3274	Momeni, H., Salehi, A., Absalan, A., & Akbari, M. (2022). Hydro-alcoholic extract of Morus nigra reduces fasting blood glucose and HbA1c% in diabetic patients, probably via competitive and allosteric interaction with alpha-glucosidase enzyme; a clinical trial and in silico analysis. Journal of Complementary and Integrative Medicine, 19(3), 763-769.	Not related to Oral CHM
3275	Singh RB, Niaz MA, Ghosh S, et al. Hypolipidemic and antioxidant effects of Commiphora mukul as an adjunct to dietary therapy in patients with hypercholesterolemia. Cardiovasc Drugs Ther. 1994;8(4):659-664. doi:10.1007/BF00877661. PMID:7817447.	Not related to Oral CHM
3276	Yu, X. D., Zhou, W. Q., & Cui, L. (2003). Hypotensive action of luohuo capsule and its effect on plasma adrenal medullin and tissue factor pathway inhibitor. Zhongguo Zhong xi yi jie he za zhi Zhongguo Zhongxiyi Jiehe Zazhi= Chinese Journal of Integrated Traditional and Western Medicine, 23(9), 668-672.	Not related to Oral CHM
3277	Tirapelli, C. R., Ambrosio, S. R., de Oliveira, A. M., & Tostes, R. C. (2010). Hypotensive action of naturally occurring diterpenes: a therapeutic promise for the treatment of hypertension. Fitoterapia, 81(7), 690-702.	Not related to Oral CHM
3278	Ghorbani A, Esmaeilizadeh M. Hypotensive medicinal plants according to ethnobotanical evidence of Iran: a systematic review. Avicenna J Phytomed. 2017;7(6):471-486. PMID:29238720.	Not related to Oral CHM
3279	Asai A, Yamakawa O. Mulberry leaf extract improves metabolic parameters in type 2 diabetes patients: A randomized, placebo-controlled pilot study. J Tradit Complement Med. 2017;7(3):322-327. doi:10.1016/j.jtcme.2016.09.004. PMID:28706879.	Not related to Oral CHM
3280	Marušić, S., Meliš, P., Lucijanić, M., Grgurević, I., Turčić, P., Obreli Neto, P. R., & Bilić-Ćurčić, I. (2018). Impact of pharmacotherapeutic	Not related to Oral CHM

3281	Wang, C., Yang, Y., & Cheng, Z. (2006). Improvements of Pinggan Yishen Ditan Yin in blood pressure, body weight and quality of life in patients with obesity hypertension. Chinese Journal of Clinical Rehabilitation, 10(43), 24-26.	Not related to Oral CHM
3282	Tan, Y. W., Yin, Y. M., & Yu, X. J. (2001). Influence of Salvia miltiorrhizae and Astragalus membranaceus on hemodynamics and liver fibrosis indexes in liver cirrhotic patients with portal hypertension. Zhongguo Zhong xi yi jie he za zhi Zhongguo Zhongxiyi Jiehe Zazhi= Chinese Journal of Integrated Traditional and Western Medicine, 21(5), 351-353.	Not related to Oral CHM
3283	Wang, Y., Li, D. X., Wang, Y. L., Tao, W., Wang, J. T., Zhao, Y. M., ... & Li, Y. L. (2022). Influence of small-group experiential learning of integrated traditional Chinese and Western medicine on the oral health knowledge, beliefs, and behaviors of elderly patients with diabetes. Revista da Associação Médica Brasileira, 68(2), 217-221.	Not related to Oral CHM
3284	Wang, Q., & Wanq, C. (2005). Influence of the prescription for lowering glucose and lipid on the lipid metabolism and hemorheology in diabetic patients with hyperlipidemia. Chinese Journal of Tissue Engineering Research, 180-181.	Not related to Oral CHM
3285	Zhang, X., Tian, G., Shi, Z., Sun, Y., Hu, J., Jiang, Y., ... & DIRECTION investigators. (2020). Initiate Danhong Injection before or after percutaneous coronary intervention for microvascular obstruction in ST-elevation myocardial infarction (DIRECTION): study protocol for a randomized controlled trial. Trials, 21, 1-8.	Not related to Oral CHM
3286	Zhang, X., Liu, Y., Xiong, D., & Xie, C. (2015). Insulin combined with C hinese medicine improves glycemic outcome through multiple pathways in patients with type 2 diabetes mellitus. Journal of Diabetes Investigation, 6(6), 708-715.	Not related to Oral CHM
3287	Setiawati A, Rahmawati F, Dillasamola D, Susianti S, Kusumaningsih T, Putri R. Insulin sensitizer in prediabetes: a clinical study with DLBS3233, a combined bioactive fraction of Cinnamomum	Not related to Oral CHM
3288	Patel, K., & Patel, D. K. (2022). T131 Insulin-mimetic role of kaempferitrin in glucose homeostasis: A dietary flavonoid exhibits anti-diabetic complications and promotes glucose uptake. Clinica Chimica Acta, 530, S118.	Not related to Oral CHM
3289	Mohammed, S. A., Hanxing, L., Fang, L., Algradi, A. M., Alradhi, M., Safi, M., & Shumin, L. (2023). Integrated Chinese herbal medicine with Western Medicine versus Western Medicine in the effectiveness of primary hypertension treatment: A systematic review and meta-analysis of randomized controlled trials. Journal of ethnopharmacology, 300, 115703.	Not related to Oral CHM
3290	Qian, W., Tao, H., & Jiali, Z. (2021). Integrated meta analysis and network pharmacology to investigate clinical evaluation and potential mechanism of Bushen Huoxue Decoction in treatment of diabetic nephropathy. Chin Tradit Herb Drugs, 52(6), 1692-1705.	Not related to Oral CHM

3291	Wan-Yu, L., & Hung-Rong, Y. (2019). Integrated TCM care program combined with intranasal corticosteroids to treat pediatric obstructive sleep apnea: A case series study. Advances in Integrative Medicine, 6, S89.	Not related to Oral CHM
3292	Zhao, L., Lan, L. G., Min, X. L., Lu, A. H., Zhu, L. Q., He, X. H., & He, L. J. (2007). Integrated treatment of traditional Chinese medicine and western medicine for early-and intermediate-stage diabetic nephropathy. Nan Fang yi ke da xue xue bao= Journal of Southern Medical University, 27(7), 1052-1055.	Not related to Oral CHM
3293	Mehta, D. (2017). Integrative medicine and cardiovascular disorders. Primary Care: Clinics in Office Practice, 44(2), 351-367.	Not related to Oral CHM
3294	Yu, H. H., & Hsieh, C. J. (2021). Integrative therapy combining Chinese herbal medicines with conventional treatment reduces the risk of cardiovascular disease among patients with systemic lupus erythematosus: a retrospective population-based cohort study. Frontiers in Pharmacology, 12, 737105.	Not related to Oral CHM
3295	Rajabian, A., Rajabian, F., Babaei, F., Mirzababaei, M., Nassiri-Asl, M., & Hosseinzadeh, H. (2022). Interaction of medicinal plants and their active constituents with potassium ion channels: A systematic review. Frontiers in Pharmacology, 13, 831963	Not related to Oral CHM
3296	Wang, W. H., Zhang, H., Yu, Y. L., Ge, Z., Xue, C., & Zhang, P. (2004). Intervention of xuezhikang on patients of acute coronary syndrome with different levels of blood lipids. Zhongguo Zhong xi yi jie he za zhi Zhongguo Zhongxiyi Jiehe Zazhi= Chinese Journal of Integrated Traditional and Western Medicine, 24(12), 1073-1076.	Not related to Oral CHM
3297	Zhou, Q., Ye, Z., Ruan, Z., & Zeng, S. (2013). Investigation on modulation of human P-gp by multiple doses of Radix Astragali extract granules using fexofenadine as a phenotyping probe. Journal of ethnopharmacology, 146(3), 744-749.	Not related to Oral CHM
3298	Hamedi A, Moheimani M, Lotfi M, et al. Iranian medicinal plants for diabetes mellitus: a systematic review. Diabetes Metab Syndr. 2019;13(3):1749-1755. doi:10.1016/j.dsx.2019.03.033	Not related to Oral CHM
3299	Wang, J., Yang, X., Feng, B., Qian, W., Fang, Z., Liu, W., ... & Xiong, X. (2013). Is Yangxue Qingnao Granule combined with antihypertensive drugs, a new integrative medicine therapy, more effective than antihypertensive therapy alone in treating essential hypertension?. Evidence-Based Complementary and Alternative Medicine, 2013(1), 540613.	Not related to Oral CHM
3300	Munhoz, A. C., & Frode, T. S. (2018). Isolated compounds from natural products with potential antidiabetic activity-a systematic review. Current diabetes reviews, 14(1), 36-106.	Not related to Oral CHM
3301	张会峰,林琳,周艺,等.Klinefelter综合征合并 2 型糖尿病 1 例[J].江苏医药 ,2018,44(04):479-480.DOI:10.19460/j.cnki.0253-3685.2018.04.042.	Not related to Oral CHM
3302	Naemiratch, B., & Manderson, L. (2007). Lay explanations of type 2 diabetes in Bangkok, Thailand. Anthropology & medicine, 14(1), 83-94.	Not related to Oral CHM

3303	Ogier, N., Amiot, M. J., Georgé, S., Maillot, M., Mallmann, C., Maraninchi, M., ... & Cardinault, N. (2013). LDL-cholesterol-lowering effect of a dietary supplement with plant extracts in subjects with moderate hypercholesterolemia. European journal of nutrition, 52, 547-557.	Not related to Oral CHM
3304	Ogier, N., Amiot, M. J., Georgé, S., Maillot, M., Mallmann, C., Maraninchi, M., ... & Cardinault, N. (2013). LDL-cholesterol-lowering effect of a dietary supplement with plant extracts in subjects with moderate hypercholesterolemia. European journal of nutrition, 52, 547-557.	Not related to Oral CHM
3305	Wong, M. W., Leung, P. C., & Wong, W. C. (2001). Limb salvage in extensive diabetic foot ulceration-a preliminary clinical study using simple debridement and herbal drinks. Hong Kong Medical Journal, 7(4), 403-407.	Not related to Oral CHM
3306	Hallikainen MA, Sarkkinen ES, Gylling H. Plant stanol esters affect serum cholesterol concentrations of hypercholesterolemic men and women in a dose-dependent manner. J Nutr. 2000;130(3):767-776. doi:10.1093/jn/130.3.767. PMID:10702590.	Not related to Oral CHM
3307	Bundy R, Walker AF, Middleton RW, Wallis C. Artichoke leaf extract (Cynara scolymus) reduces plasma cholesterol in hypercholesterolemic adults: a randomized, double blind, placebo controlled clinical trial. Phytomedicine. 2008;15(9):668-675. doi:10.1016/j.phymed.2008.04.004. PMID:18585777.	Not related to Oral CHM
3308	Pu Run, P. R., Geng XiangNan, G. X., Yu Fei, Y. F., Liang HuiGang, L. H., & Shi LuWen, S. L. (2013). Liuwei Dihuang Pills enhance the effect of Western medicine in treating type 2 diabetes: a meta-analysis of randomized controlled trials.	Not related to Oral CHM
3309	Wassertheurer S, Mayer C, Breitenecker F, et al. LOW-BP-VIENNA study: Lowering blood pressure in primary care in Vienna. Wien Klin Wochenschr. 2019;131(13-14):317-324. doi:10.1007/s00508-019-01491-5. PMID:30949806.	Not related to Oral CHM
3310	Ulbricht C, Isaac R, Milkin T, et al. Maitake mushroom (Grifola frondosa): Systematic review by the natural standard research collaboration. J Soc Integr Oncol. 2007;5(3):122-128. PMID:17698461.	Not related to Oral CHM
3311	Watanabe, M., Gangitano, E., Francomano, D., Addessi, E., Toscano, R., Costantini, D., ... & Lubrano, C. (2018). Mangosteen extract shows a potent insulin sensitizing effect in obese female patients: A prospective randomized controlled pilot study. Nutrients, 10(5), 586.	Not related to Oral CHM
3312	Nartey, L., Huwiler-Müntener, K., Shang, A., Liewald, K., Jüni, P., & Egger, M. (2007). Matched-pair study showed higher quality of placebo-controlled trials in Western phytotherapy than conventional medicine. Journal of clinical epidemiology, 60(8), 787-e1.	Not related to Oral CHM

331<br>3	Oniki, K., Kawakami, T., Nakashima, A., Miyata, K., Watanabe, T., Fujikawa, H., ... & Shuto, T. (2020). Melinjo seed extract increases adiponectin multimerization in physiological and pathological conditions. Scientific Reports, 10(1), 4313.	Not related to Oral CHM
331<br>4	Yang, L., Long, Y., LI, H., & Liu, Y. (2006). Meta analysis of integrated Chinese-Western medicine therapy for type 2 diabetes mellitus. Journal of Jilin University (Medicine Edition).	Not related to Oral CHM
331<br>5	Wu, Y., Hu, Y. L., Liu, W., Sun, B. J., Zhang, C. F., Wu, L. L., & Liu, T. H. (2022). Meta-analysis Flavonoids from traditional Chinese herbs for diabetes in rats: a network Meta-analysi. Journal of traditional Chinese medicine= Chung i tsa chih ying wen pan, 42(1), 1-8.	Not related to Oral CHM
331<br>6	Wang, M. X., Wu, C. J., Cao, P. H., & Chen, X. H. (2021). Meta-analysis for clinical efficacy of traditional Chinese medicine in treating resistant hypertension. Zhongguo Zhong yao za zhi= Zhongguo Zhongyao Zazhi= China Journal of Chinese Materia Medica, 46(3), 685-693.	Not related to Oral CHM
331<br>7	Tang, S., Zhang, D., Han, S., Chen, Y. H., Ma, B. N., Liu, C. X., & Zhang, C. N. (2021). Meta-analysis of Danhong Injection in treatment of diabetes mellitus complicated with coronary heart disease. Zhongguo Zhong Yao Za Zhi= Zhongguo Zhongyao Zazhi= China Journal of Chinese Materia Medica, 46(1), 237-246.	Not related to Oral CHM
331<br>8	Suksomboon, N., Poolsup, N., Boonkaew, S., & Suthisisang, C. C. (2011). Meta-analysis of the effect of herbal supplement on glycemic control in type 2 diabetes. Journal of Ethnopharmacology, 137(3), 1328-1333.	Not related to Oral CHM
331<br>9	Wan, X., Gen, F., Sheng, Y., Ou, M., Wang, F., Peng, T., & Guo, J. (2021). Meta-Analysis of the Effect of Kangfuxin Liquid on Diabetic Patients with Skin Ulcers. Evidence-Based Complementary and Alternative Medicine, 2021(1), 1334255.	Not related to Oral CHM
332<br>0	Yang, M., Cheng, M., Wang, M., Tang, Z., Song, Z., Cui, C., ... & Zhang, Z. (2021). Meta-Analysis of the Effect of Traditional Chinese Medicine Compounds Combined with Standard Western Medicine for the Treatment of Diabetes Mellitus Complicated by Coronary Heart Disease. Evidence-Based Complementary and Alternative Medicine, 2021(1), 5515142.	Not related to Oral CHM
332<br>1	Xiong, X., Wang, P., & Li, S. (2015). Meta-analysis of the effectiveness of traditional Chinese herbal formula Zhen Wu Decoction for the treatment of hypertension. BMJ open, 5(12), e007291.	Not related to Oral CHM
332<br>2	Zhang, X., Wu, M., Zhou, J., Zhou, R., Luo, Q., Yue, R., & Jin, S. (2021). Meta-analysis-based systematic review of effect of traditional Chinese medicine intervention in treatment of diabetic nephropathy on thyroid function. Annals of Palliative Medicine, 10(6), 6736752-6736752.	Not related to Oral CHM
332<br>3	Rivero-Cruz JF, Granados-Pineda J, Pedraza-Chaverri J, et al. Mexican Arnica (Heterotheca inuloides Cass. Asteraceae: Astereae): Ethnomedical uses, chemical constituents and biological properties. J Ethnopharmacol. 2016;188:168-189. doi:10.1016/j.jep.2016.05.006. PMID:27180329.	Not related to Oral CHM

3324	Reyes BA, Bautista E, Gonzalez L, et al. Mexican Plants Involved in Glucose Homeostasis and Body Weight Control: Systematic Review. Int J Environ Res Public Health. 2020;17(15):5350. doi:10.3390/ijerph17155350. PMID:32751783.	Not related to Oral CHM
3325	Ramos-Lopez, O., Riezu-Boj, J. I., Milagro, F. I., Cuervo, M., Goni, L., & Martinez, J. A. (2019). Models integrating genetic and lifestyle interactions on two adiposity phenotypes for personalized prescription of energy-restricted diets with different macronutrient distribution. Frontiers in Genetics, 10, 686.	Not related to Oral CHM
3326	Thomford, K. P., Thomford, A. K., Yorke, J., Yeboah, R., & Appiah, A. A. (2021). Momordica charantia L. for hyperlipidaemia: A randomised controlled assessment of the Ghanaian herbal medicinal product MCP-1. Journal of Herbal Medicine, 28, 100453.	Not related to Oral CHM
3327	Ye, Y. W., Yan, Z. Y., He, L. P., & Li, C. P. (2022). More studies are necessary to establish the effectiveness of Jinhuang powder in the treatment of diabetic foot. World Journal of Diabetes, 13(7), 581.	Not related to Oral CHM
3328	Wang, J., Lu, Z., Chi, J., Wang, W., Su, M., Kou, W., ... & Chang, J. (1997). Multicenter clinical trial of the serum lipid-lowering effects of a Monascus purpureus (red yeast) rice preparation from traditional Chinese medicine. Current Therapeutic Research, 58(12), 964-978.	Not related to Oral CHM
3329	Ni, L., Wang, R. B., Guo, H. J., Tan, X. H., Sun, L. J., Zheng, Y. H., ... & Zhao, H. X. (2013). Multi-centered, randomized controlled clinical study on Chinese medicine formula particles for hyperlipidaemia associated with highly active antiretroviral therapy. Zhongguo Zhong yao za zhi= Zhongguo Zhongyao Zazhi= China Journal of Chinese Materia Medica, 38(15), 2443-2447.	Not related to Oral CHM
3330	Ezzat SM, Ezzat MI, Okba MM, Menze ET, Abdel-Naim AB. Nanoformulation of plant-based natural products for type 2 diabetes mellitus: from formulation design to therapeutic applications. Biomed Pharmacother. 2018;107:1810-1822. doi:10.1016/j.biopha.2018.08.100. PMID:30196095.	Not related to Oral CHM
3331	Zhang, Y., Zhang, Y., Yang, C., Duan, Y., Jiang, L., Jin, D., ... & Tong, X. (2022). Naoxintong capsule delay the progression of diabetic kidney disease: a real-world cohort study. Frontiers in Endocrinology, 13, 1037564.	Not related to Oral CHM
3332	Ren, P., Cao, L., Zhao, X. K., Zhu, B. B., & Liu, K. (2022). Network Meta-analysis of Chinese medicine injections in treatment of hypertensive intracerebral hemorrhage. Zhongguo Zhong yao za zhi= Zhongguo Zhongyao Zazhi= China Journal of Chinese Materia Medica, 47(13), 3637-3647.	Not related to Oral CHM
3333	Shi, R. Y., Zhang, L. Q., Zhang, K. X., Li, Q. R., Wang, X. X., & Yang, K. (2023). Network Meta-analysis of efficacy of Chinese patent medicine in treatment of inflammatory response in diabetic nephropathy. Zhongguo Zhong yao za zhi= Zhongguo Zhongyao Zazhi= China Journal of Chinese Materia Medica, 48(13), 3633-3649.	Not related to Oral CHM

3334	Zhang, J., Li, J., & Huang, J. Q. (2020). Network meta-analysis of four Chinese patent medicines combined with angiotensin converting enzyme inhibitors or angiotensin receptor blockers in early diabetic nephropathy treatment. World Journal of Traditional Chinese Medicine, 6(1), 51-60.	Not related to Oral CHM
3335	Ya-Wei, Z., Li-Fei, W. U., Yu-Hao, Z., Li-Hua, W. U., & Jie, L. I. (2020). Ne	Not related to Oral CHM
3336	Zheng, Y. W., Li, J., Yao, W. Q., Pan, M. Y., & Fang, Y. (2022). Network Meta-analysis of oral blood-activating and stasis-removing Chinese patent medicines in treatment of hypertensive left ventricular hypertrophy. Zhongguo Zhong yao za zhi= Zhongguo Zhongyao Zazhi= China Journal of Chinese Materia Medica, 47(5), 1383-1391.	Not related to Oral CHM
3337	Wang, W. R., Zhang, X. M., Li, J. X., Yang, J. Y., Yu, R. H., & Xie, Y. M. (2023). Network Meta-analysis of Qi-supplementing and Yin-nourishing Chinese patent medicines in treatment of early diabetic nephropathy. Zhongguo Zhong yao za zhi= Zhongguo Zhongyao Zazhi= China Journal of Chinese Materia Medica, 48(14), 3949-3964.	Not related to Oral CHM
3338	Venketasubramanian N, Young SH, Tay SS, et al. Safety and efficacy of MLC901 (NeuroAiD II) in severe traumatic brain injury: a pilot study. Brain Inj. 2016;30(4):453-460. doi:10.3109/02699052.2015.1123277	Not related to Oral CHM
3339	Tan CN, Choy D, Venketasubramanian N. NeuroAid II (MLC901) in Haemorrhagic Stroke. Case Rep Neurol. 2020 Dec 14;12(Suppl 1):212-217. doi: 10.1159/000508588. PMID: 33505298; PMCID: PMC7802497.	Not related to Oral CHM
3340	Yimer EM, Tuem KB, Karim A, Ur-Rehman N, Anwar F. Nigella sativa L. (Black Cumin): A Promising Natural Remedy for Wide Range of Illnesses. Evid Based Complement Alternat Med. 2019;2019:1528635. Published 2019 May 12. doi:10.1155/2019/1528635	Not related to Oral CHM
3341	Wang H, Liu C, Zhai J, Shang H. Niuhuang Jiangya Preparation (a traditional Chinese patent medicine) for essential hypertension: A systematic review. Complement Ther Med. 2017;31:90-99. doi:10.1016/j.ctim.2017.02.005	Not related to Oral CHM
3342	Maier HM, Ilich JZ, Kim JS, Spicer MT. Nutrition supplementation for diabetic wound healing: a systematic review of current literature. Skinmed. 2013;11(4):217-225.	Not related to Oral CHM
3343	Wang, W., & Deng, X. (2015). Observation of the clinical efficacy of Shenfuyishen Capsule in the treatment of diabetic nephropathy in high altitude regions. Journal of Xi'an Jiaotong University (Medical Sciences), 845-848.	Not related to Oral CHM
3344	Zhang PX, Zeng L, Meng L, Li HL, Zhao HX, Liu DL. Observation on clinical effect of Huoxue-Jiangtang decoction formula granules in treating prediabetes: a randomized prospective placebo-controlled double-blind trial protocol. BMC Complement Med Ther. 2022;22(1):274. Published 2022 Oct 19. doi:10.1186/s12906-022-03755-2	Not related to Oral CHM

3345	Susalit E, Agus N, Effendi I, et al. Olive (Olea europaea) leaf extract effective in patients with stage-1 hypertension: comparison with Captopril. Phytomedicine. 2011;18(4):251-258. doi:10.1016/j.phymed.2010.08.016	Not related to Oral CHM
3346	Memon, A. R., & Ghanghro, A. B. (2021). Olive Oil act as Therapeutic Agent in the Management of Dyslipidemia in the Patients of Type–II Diabetes Mellitus. Metabolism-Clinical and Experimental, 116.	Not related to Oral CHM
3347	Meng, TT, Xie, XL, Li, TT, Yang, QY, Wei, DW and Gao, Y, 2021. Oral Chinese patent medicine combined with statins in treatment of dyslipidemia:A network Meta-analysis, Chinese Traditional and Herbal Drugs	Not related to Oral CHM
3348	Xue, X., Li, K. Y., Liu, S. Z., Li, J. X., Jin, X. Y., Liu, X. H., ... & Wang, X. Q. (2023). Oral Chinese patent medicines for diabetic kidney disease: An overview of systematic reviews. European Journal of Integrative Medicine, 61, 102269.	Not related to Oral CHM
3349	Pang, Q., WU, Q., NI, Q., & LIAO, X. (2022). Oral Chinese Patent Medicines for Hyperlipidemia: A Scoping Review of Clinical Evidence.聽Chinese Journal of Experimental Traditional Medical Formulae, 214-221.	Not related to Oral CHM
3350	荣毅. (2019). OSAHS 合并症中医证型研究及丹蒌片干预 CIH 诱导血脂异常, 动脉硬化的机制 (Doctoral dissertation, 中国中医科学院).	Not related to Oral CHM
3351	Wang, Y., Wang, X., Shen, S., & Zhang, L. (2023). Outcome indexes in randomized controlled trials of Chinese medicine for diabetic kidney disease. Chinese Journal of Experimental Traditional Medical Formulae, 119-130.	Not related to Oral CHM
3352	Wang J, Xiong X. Outcome measures of chinese herbal medicine for hypertension: an overview of systematic reviews. Evid Based Complement Alternat Med. 2012;2012:697237. doi:10.1155/2012/697237	Not related to Oral CHM
3353	Qiu R, Hu J, Huang Y, et al. Outcome reporting from clinical trials of non-valvular atrial fibrillation treated with traditional Chinese medicine or Western medicine: a systematic review. BMJ Open. 2019;9(8):e028803. Published 2019 Aug 30. doi:10.1136/bmjopen-2018-028803	Not related to Oral CHM
3354	Timotius, K. H., & Rahayu, I. (2020). Overview of herbal therapy with leave of Gynura procumbens (Lour.) Merr. Journal of Young Pharmacists, 12(3), 201.	Not related to Oral CHM
3355	Tang X, Huang M, Jiang J, et al. Panax notoginseng preparations as adjuvant therapy for diabetic kidney disease: a systematic review and meta-analysis. Pharm Biol. 2020;58(1):138-145. doi:10.1080/13880209.2020.1711782	Not related to Oral CHM
3356	Miroddi M, Calapai G, Navarra M, Minciullo PL, Gangemi S. Passiflora incarnata L.: ethnopharmacology, clinical application, safety and evaluation of clinical trials. J Ethnopharmacol. 2013;150(3):791-804. doi:10.1016/j.jep.2013.09.047	Not related to Oral CHM

3357	Wen, L, Tian, X, Wang, D, Xia, R, Fei, Y, Huang, N, Hu, R and Liu, JP, 2018. Patent of ophiocordyceps sinensis (Jin Shui Bao) for diabetic kidney disease: A systematic review and meta-analysis, Global Advances in Health and Medicine.	Not related to Oral CHM
3358	Shao, C., Sun, M., Liu, W., Zhao, S., Liu, Y., Chen, Y., ... & Li, S. (2022). Patient-reported outcomes following the use of jiang tang san huang tablets in type 2 diabetes mellitus: A retrospective cohort study in a chinese population. Diabetes, Metabolic Syndrome and Obesity: Targets and Therapy, 4023-4033.	Not related to Oral CHM
3359	Sirotkin AV. Peppers and their constituents against obesity. Biol Futur. 2023;74(3):247-252. doi:10.1007/s42977-023-00174-3	Not related to Oral CHM
3360	Ożarowski M, Mikołajczak PŁ, Kujawski R, et al. Pharmacological Effect of Quercetin in Hypertension and Its Potential Application in Pregnancy-Induced Hypertension: Review of In Vitro, In Vivo, and Clinical Studies. Evid Based Complement Alternat Med. 2018;2018:7421489. Published 2018 Dec 2. doi:10.1155/2018/7421489	Not related to Oral CHM
3361	Sepahi S, Ghorani-Azam A, Hossieni SM, Mohajeri SA, Khodaverdi E. Pharmacological Effects of Saffron and its Constituents in Ocular Disorders from in vitro Studies to Clinical Trials: A Systematic Review. Curr Neuropharmacol. 2021;19(3):392-401. doi:10.2174/1570159X18666200507083346	Not related to Oral CHM
3362	Manzione MG, Martorell M, Sharopov F, et al. Phytochemical and pharmacological properties of asperuloside, a systematic review. Eur J Pharmacol. 2020;883:173344. doi:10.1016/j.ejphar.2020.173344	Not related to Oral CHM
3363	Widjajakusuma EC, Jonosewojo A, Hendriati L, et al. Phytochemical screening and preliminary clinical trials of the aqueous extract mixture of Andrographis paniculata (Burm. f.) Wall. ex Nees and Syzygium polyanthum (Wight.) Walp leaves in metformin treated patients with type 2 diabetes. Phytomedicine. 2019;55:137-147. doi:10.1016/j.phymed.2018.07.002	Not related to Oral CHM
3364	Ore A, Akinloye OA. Phytotherapy as Multi-Hit Therapy to Confront the Multiple Pathophysiology in Non-Alcoholic Fatty Liver Disease: A Systematic Review of Experimental Interventions. Medicina (Kaunas). 2021;57(8):822. Published 2021 Aug 14. doi:10.3390/medicina57080822	Not related to Oral CHM
3365	Ziyyat A, Legssyer A, Mekhfi H, Dassouli A, Serhrouchni M, Benjelloun W. Phytotherapy of hypertension and diabetes in oriental Morocco. J Ethnopharmacol. 1997;58(1):45-54. doi:10.1016/s0378-8741(97)00077-9	Not related to Oral CHM
3366	Raziani, Y., Qadir, S. H., Hermis, A. H., Nazari, A., Othman, B. S., & Raziani, S. (2022). Pistacia atlantica as an effective remedy for diabetes: a randomised, double-blind, placebo-controlled trial. Australian Journal of Herbal and Naturopathic Medicine, 34(3), 118-124.	Not related to Oral CHM
3367	Todorova V, Ivanov K, Delattre C, Nalbantova V, Karcheva-Bahchevanska D, Ivanova S. Plant Adaptogens-History and Future Perspectives. Nutrients. 2021;13(8):2861. Published 2021 Aug 20. doi:10.3390/nu13082861	Not related to Oral CHM

3368	Seidlova-Wuttke D, Jarry H, Wuttke W. Plant derived alternatives for hormone replacement therapy (HRT). Horm Mol Biol Clin Investig. 2013;16(1):35-45. doi:10.1515/hmbci-2013-0024	Not related to Oral CHM
3369	Ye P, Wu CE, Sheng L, Li H. Potential protective effect of long-term therapy with Xuezhikang on left ventricular diastolic function in patients with essential hypertension. J Altern Complement Med. 2009;15(7):719-725. doi:10.1089/acm.2008.0599	Not related to Oral CHM
3370	Pan, L., LI, D., Lei, M., Zhang, L., & Zhou, L. (2005). Preparation-containing node of Lotus Rhizome, green tea and Panax notoginseng for obese adults. Chinese Journal of Tissue Engineering Research, 231-233.	Not related to Oral CHM
3371	Pang B, Zhang YY, Hu HJ, et al. Prevention of Diabetes in Overweight/Obese Adults through Traditional Chinese Patent Medicine: Study Protocol for a Prospective Cohort Study. Evid Based Complement Alternat Med. 2021;2021:6006802. Published 2021 Nov 8. doi:10.1155/2021/6006802	Not related to Oral CHM
3372	Pang B, Lian FM, Zhao XY, et al. Prevention of type 2 diabetes with the traditional Chinese patent medicine: A systematic review and meta-analysis. Diabetes Res Clin Pract. 2017;131:242-259. doi:10.1016/j.diabres.2017.07.020	Not related to Oral CHM
3373	Xu KJ, Zhang SF, Li QX. Zhongguo Zhong Xi Yi Jie He Za Zhi. 2003;23(9):648-650.	Not related to Oral CHM
3374	Yoneshiro T, Matsushita M, Sugita J, et al. Prolonged Treatment with Grains of Paradise (Aframomum melegueta) Extract Recruits Adaptive Thermogenesis and Reduces Body Fat in Humans with Low Brown Fat Activity. J Nutr Sci Vitaminol (Tokyo). 2021;67(2):99-104. doi:10.3177/jnsv.67.99	Not related to Oral CHM
3375	Yang F, Yu SY, Wang Y, Wang RF, Jing F. Prospective induction of peripheral neuropathy by the use of Tartarian Buckwheat. J Neurol Sci. 2014;347(1-2):155-158. doi:10.1016/j.jns.2014.09.037	Not related to Oral CHM
3376	Okamura K, Kuroda R, Nagata K, Urata H. Prospective single-arm observational study of human chymase inhibitor Polygonum hydropiper L in subjects with hypertension. Clin Exp Hypertens. 2019;41(8):717-725. doi:10.1080/10641963.2018.1545847	Not related to Oral CHM
3377	Wang, J. K., Zhong, J. H., Huang, K., FU, Q. F., & Wang, J. Y. (2021). Protection Mechanism of Addition and Subtraction Therapy of Danshenyin Combined with Wendantang for Myocardial Ischemia in Patients with Stable Angina. Chinese Journal of Experimental Traditional Medical Formulae, 82-87.	Not related to Oral CHM
3378	Sun, Y. P., Yang, J. F., & Li, W. (2004). Protective effect of honghua parenteral solutionon vascular endothelium in patients with essential hypertension. Chin J Clin Rehabil, 33, 7580-82.	Not related to Oral CHM

3379	Wei, Y. N., MO, X. M., Wang, Q., QU, N., Zhong, R. F., & Wei, B. (2021). Protective Effect of Huangqi Guizhi Wuwutang Combined with Shengmaiyin on Cardiac Function of Diabetic Cardiomyopathy. Chinese Journal of Experimental Traditional Medical Formulae, 104-109.	Not related to Oral CHM
3380	Yang L, Chen J, Lu H, et al. Pueraria lobata for Diabetes Mellitus: Past, Present and Future. Am J Chin Med. 2019;47(7):1419-1444. doi:10.1142/S0192415X19500733	Not related to Oral CHM
3381	Wainstein J, Landau Z, Bar Dayan Y, et al. Purslane Extract and Glucose Homeostasis in Adults with Type 2 Diabetes: A Double-Blind, Placebo-Controlled Clinical Trial of Efficacy and Safety. J Med Food. 2016;19(2):133-140. doi:10.1089/jmf.2015.0090	Not related to Oral CHM
3382	Wang, Z. Z. (2017). Qi Ming granule combined with calcium dobesilate in treatment of non-proliferative diabetic retinopathy. International eye science, 702-705.	Not related to Oral CHM
3383	Zeng, G., Liu, G., Liu, H., & Zhong, J. (2015). Ming granule intervention on retinal function in the patients with simplex diabetic retinopathy. Int Eye Sci, 15, 495-8.	Not related to Oral CHM
3384	Zhang S, Bai X, Chen ZL, Li JJ, Chen YY, Tang YP. Qiju Dihuang Decoction for Hypertension: A Systematic Review and Meta-Analysis. Evid Based Complement Alternat Med. 2020;2020:9403092. Published 2020 Jul 31. doi:10.1155/2020/9403092	Not related to Oral CHM
3385	Tan Y, Hu J, Zhang Y, Wu Q, Ni Q. Qizhijiangtang capsule for the treatment of diabetic kidney disease: A protocol for systematic review and meta-analysis. Medicine (Baltimore). 2020;99(34):e21923. doi:10.1097/MD.0000000000021923	Not related to Oral CHM
3386	Tabatabaei-Malazy O, Shadman Z, Ejtahed HS, Atlasi R, Abdollahi M, Larijani B. Quality of reporting of randomized controlled trials of herbal medicines conducted in metabolic disorders in Middle East countries: A systematic review. Complement Ther Med. 2018;38:61-66. doi:10.1016/j.ctim.2018.04.004	Not related to Oral CHM
3387	Xu X, Tian W, Duan W, et al. Quanduzhong capsules for the treatment of grade 1 hypertension patients with low-to-moderate risk: A multicenter, randomized, double-blind, placebo-controlled clinical trial. Front Pharmacol. 2023;13:1014410. Published 2023 Jan 10. doi:10.3389/fphar.2022.1014410	Not related to Oral CHM
3388	Tai J, Zou J, Zhang X, et al. Randomized Controlled Trials of Tianma Gouteng Decoction Combined with Nifedipine in the Treatment of Primary Hypertension: A Systematic Review and Meta-Analysis. Evid Based Complement Alternat Med. 2020;2020:5759083. Published 2020 Feb 7. doi:10.1155/2020/5759083	Not related to Oral CHM
3389	Tripathy, PC, Karmahapatra, PB and Palaniyamma, D, 2013, Randomized, double-blind, placebo controlled clinical study to evaluate the effects of Garcinia caplets in obese subjects, International Research Journal of Pharmacy	Not related to Oral CHM

3390	Randomized, double-blind, placebo-controlled, linear dose, crossover study to evaluate the efficacy and safety of a green coffee bean extract in overweight subjects [Retraction] [retraction of: Diabetes Metab Syndr Obes. 2012;5:21-7. doi: 10.2147/DMSO.S27665.]. Diabetes Metab Syndr Obes. 2014;7:467. Published 2014 Oct 16. doi:10.2147/DMSO.S75357	Not related to Oral CHM
3391	Xu W, Peng R, Chen S, et al. Ranunculus ternatus Thunb extract attenuates renal fibrosis of diabetic nephropathy via inhibiting SMYD2. Pharm Biol. 2022;60(1):300-307. doi:10.1080/13880209.2022.2030759	Not related to Oral CHM
3392	Mao W, Zhang L, Zou C, et al. Rationale and design of the Helping Ease Renal failure with Bupi Yishen compared with the Angiotensin II Antagonist Losartan (HERBAAL) trial: a randomized controlled trial in non-diabetes stage 4 chronic kidney disease. BMC Complement Altern Med. 2015;15:316. Published 2015 Sep 8. doi:10.1186/s12906-015-0830-1	Not related to Oral CHM
3393	Varas, B., & Fuentes, M. M. (2019). AB0453 RED RICE YEAST. AN ALTERNATIVE IN RHEUMATOID ARTHRITIS AND HYPERLIPIDEMIA. Annals of the Rheumatic Diseases, 78, 1690.	Not related to Oral CHM
3394	Wei, QL, Lu, XH, Xia, SH, Liu, CP and Liu, C, 2006, Regulation effect of Xiaokening on the level of transforming growth factor-beta 1 in vivo in patients with early diabetic nephropathy, Chinese Journal of Clinical Rehabilitation	Not related to Oral CHM
3395	Sun L, Jiang J, Chen XY. Zhongguo Zhong Xi Yi Jie He Za Zhi. 2003;23(4):272-274.	Not related to Oral CHM
3396	Rondanelli M, Opizzi A, Faliva M, Bucci M, Perna S. Relationship between the absorption of 5-hydroxytryptophan from an integrated diet, by means of Griffonia simplicifolia extract, and the effect on satiety in overweight females after oral spray administration. Eat Weight Disord. 2012;17(1):e22-e28. doi:10.3275/8165	Not related to Oral CHM
3397	Shao B, Hou S, Chan Y, Shao C, Lao L. Remission of new-onset type 2 diabetes mellitus in an adolescent using an integrative medicine approach: A case report. J Integr Med. 2021;19(1):85-88. doi:10.1016/j.joim.2020.10.005	Not related to Oral CHM
3398	Xie, Y., Zhang, J., Zhang, M., & Lei, T. (2022). Repairing Diabetic Wounds with Polysaccharides from Traditional Chinese Medicines: A Review. Chinese Journal of Experimental Traditional Medical Formulae, 28(17), 258-266.	Not related to Oral CHM
3399	Wu W, Zhang LL, Zou J. Zhongguo Zhong Yao Za Zhi. 2016;41(14):2591-2599. doi:10.4268/cjcmm20161405	Not related to Oral CHM
3400	Zheng W, Wang G, Zhang Z, Wang Z, Ma K. Research progress on classical traditional Chinese medicine formula Liuwei Dihuang pills in the treatment of type 2 diabetes. Biomed Pharmacother. 2020;121:109564. doi:10.1016/j.biopha.2019.109564	Not related to Oral CHM
3401	Mu Q, Zhang Y, Cheng Q, Huang H, Huang C, Tang L. Research progress on the mechanism of action of hesperetin in cerebral ischemia: a narrative	Not related to Oral CHM

	review. Ann Transl Med. 2022;10(14):806. doi:10.21037/atm-22-3136	
340 2	Panahi Y, Pishgoo B, Beiraghdar F, Araghi ZM, Sahebkar A, Abolhasani E. Results of a randomized,open-label,clinical trial investigating the effects of supplementation with Heracleumpersicum extract as an adjunctive therapy for dyslipidemia. ScientificWorldJournal. 2011;11:592-601. Published 2011 Mar 7. doi:10.1100/tsw.2011.43	Not related to Oral CHM
340 3	Weng SW, Chang CC, Chen TL, et al. Risk of diabetes in stroke patients who used Bu Yang Huan Wu Tang: A nationwide propensity-score matched study. Phytomedicine. 2021;80:153376. doi:10.1016/j.phymed.2020.153376	Not related to Oral CHM
340 4	Yang L, Ling W, Yang Y, et al. Role of Purified Anthocyanins in Improving Cardiometabolic Risk Factors in Chinese Men and Women with Prediabetes or Early Untreated Diabetes-A Randomized Controlled Trial. Nutrients. 2017;9(10):1104. Published 2017 Oct 10. doi:10.3390/nu9101104	Not related to Oral CHM
340 5	Peter EL, Deyno S, Mtewa A, et al. Safety and efficacy of Momordica charantia Linnaeus in pre-diabetes and type 2 diabetes mellitus patients: a systematic review and meta-analysis protocol. Syst Rev. 2018;7(1):192. Published 2018 Nov 15. doi:10.1186/s13643-018-0847-x	Not related to Oral CHM
340 6	Nafrialdi, N., Hudyono, J., Suyatna, F. D., & Setiawati, A. (2019). Safety and efficacy of NC120 for improving lipid profile: a double blind randomized controlled trial. Acta Medica Indonesiana, 51(1), 19.	Not related to Oral CHM
340 7	Shayani Rad, M, Moohebati, M, MohammadEbrahimi, S, Motamedshariaty, VS and Mohajeri, SA, 2022, Safety evaluation and biochemical efficacy of celery seed extract (Apium Graveolens) capsules in hypertensive patients: a randomized, triple-blind, placebo-controlled, cross-over, clinical trial, Inflammopharmacology	Not related to Oral CHM
340 8	Razavi BM, Hosseinzadeh H. Saffron: a promising natural medicine in the treatment of metabolic syndrome. Curr Drug Targets. 2017;18(5):527-553. doi:10.2174/1389450116666151119120813. PMID:26561082.	Not related to Oral CHM
340 9	Shivaprasad, H. N., Bhanumathy, M., Sushma, G., Midhun, T., Raveendra, K. R., Sushma, K. R., & Venkateshwarlu, K. (2013). Salacia reticulata improves serum lipid profiles and glycemic control in patients with prediabetes and mild to moderate hyperlipidemia: a double-blind, placebo-controlled, randomized trial. Journal of medicinal food, 16(6), 564-568.	Not related to Oral CHM
341 0	van Poppel, P. C., Breedveld, P., Abbink, E. J., Roelofs, H., van Heerde, W., Smits, P., ... & Rongen, G. A. (2015). Salvia miltiorrhiza root water-extract (danshen) has no beneficial effect on cardiovascular risk factors. A randomized double-blind cross-over trial. PLoS One, 10(7), e0128695.	Not related to Oral CHM

341 1	Wang, L., Ma, R., Liu, C., Liu, H., Zhu, R., Guo, S., ... & Zhang, D. (2017). Salvia miltiorrhiza: a potential red light to the development of cardiovascular diseases. Current Pharmaceutical Design, 23(7), 1077-1097.	Not related to Oral CHM
341 2	Lau AJ, Toh DF, Chua TK. Saponins in Panax species—A review. Phytochemistry. 2008;69(9):2031-2041. doi:10.1016/j.phytochem.2008.04.020. PMID:18547648.	Not related to Oral CHM
341 3	Wang, Y. Z., Meng, L., Zhuang, Q. S., & Shen, L. (2021). Screening traditional Chinese medicine combination for cotreatment of Alzheimer's disease and type 2 diabetes mellitus by network pharmacology. Journal of Alzheimer's Disease, 80(2), 787-797.	Not related to Oral CHM
341 4	Xiong, X., Wang, P., Li, X., & Zhang, Y. (2015). Shenqi pill, a traditional Chinese herbal formula, for the treatment of hypertension: a systematic review. Complementary Therapies in Medicine, 23(3), 484-493.	Not related to Oral CHM
341 5	Yamakawa, J. I., Moriya, J., Takeuchi, K., Nakatou, M., Motoo, Y., & Kobayashi, J. (2013). Significance of Kampo, Japanese traditional medicine, in the treatment of obesity: basic and clinical evidence. Evidence-Based Complementary and Alternative Medicine, 2013(1), 943075.	Not related to Oral CHM
341 6	Single herbal medicine for diabetic retinopathy	Not related to Oral CHM
341 7	Yang XiaoChen, Y. X., Xiong XingJiang, X. X., Yang GuoYan, Y. G., Wang HeRan, W. H., & Wang Jie, W. J. (2015). Songling Xuemaikang Capsule for primary hypertension: a systematic review of randomized controlled trials.	Not related to Oral CHM
341 8	Sun J, Buys NJ, Jayasinghe R, et al. Structural alteration of gut microbiota during amelioration of human type 2 diabetes with hyperlipidemia by metformin and a traditional Chinese herbal formula: A multicenter, randomized, open-label clinical trial. Front Endocrinol (Lausanne). 2020;11:536. doi:10.3389/fendo.2020.00536. PMID:32765538.	Not related to Oral CHM
341 9	Wu, H. N., & Sun, H. (2003). Study on clinical therapeutic effect of composite Salvia injection matched with Western medicine in treating diabetic foot. Zhongguo Zhong xi yi jie he za zhi Zhongguo Zhongxiyi Jiehe Zazhi= Chinese Journal of Integrated Traditional and Western Medicine, 23(10), 727-729.	Not related to Oral CHM
342 0	Wang, S., Wang, S. R., & Zhao, Y. R. (2002). Study on effect of jiangya tongmai recipe on vascular activating substances in patients of hypertension with left ventricular hypertrophy. Zhongguo Zhong xi yi jie he za zhi Zhongguo Zhongxiyi Jiehe Zazhi= Chinese Journal of Integrated Traditional and Western Medicine, 22(4), 274-276.	Not related to Oral CHM

3421	Zhu, L. Q., Liu, Y. H., Huang, M., Wei, H., & Liu, Z. (2004). Study on improvement of islet beta cell function in patients with latent autoimmune diabetes mellitus in adults by integrative Chinese and Western medicine. Zhongguo Zhong xi yi jie he za zhi Zhongguo Zhongxiyi Jiehe Zazhi= Chinese Journal of Integrated Traditional and Western Medicine, 24(7), 581-584.	Not related to Oral CHM
3422	Yang, H., Yue, R., Zhou, J., Zeng, Z., Wang, L., Long, X., ... & Huang, X. (2020). Study on metabonomics of Chinese herbal medicine in the treatment of type 2 diabetes mellitus complicated with community-acquired pneumonia. Medicine, 99(37), e22160.	Not related to Oral CHM
3423	Zhu, L., Li, H., & Liu, Y. (1999). Study on prevention and treatment of middle and aged women diabetes with kidney deficiency and bone metabolic disturbance. Zhongguo Zhong xi yi jie he za zhi Zhongguo Zhongxiyi Jiehe Zazhi= Chinese Journal of Integrated Traditional and Western Medicine, 19(4), 215-217.	Not related to Oral CHM
3424	Wang, Y. J., Zhu, W. F., & Wang, X. K. (2005). Study on the effect of Qinggan Jiangtang tablet in improving the insulin resistance in patients with multiple metabolic syndrome. Zhongguo Zhong xi yi jie he za zhi Zhongguo Zhongxiyi Jiehe Zazhi= Chinese Journal of Integrated Traditional and Western Medicine, 25(5), 412-415.	Not related to Oral CHM
3425	Zhang, Y., Wang, W., & Ning, G. (2015). Study on the efficacy and safety of Jinlida in patients with inadequately controlled type-2 diabetes and dyslipidemia under life style intervention (ENJOY LIFE Study) 合并血脂异常的 2 型糖尿病患者在生活方式干预控制效果不佳时使用金利达治疗的有效性与安全性研究 (ENJOY LIFE 研究). Journal of diabetes, 7(2).	Not related to Oral CHM
3426	Zhang, Y., Wang, W., & Ning, G. (2015). Study on the efficacy and safety of Jinlida in patients with inadequately controlled type-2 diabetes and dyslipidemia under life style intervention (ENJOY LIFE Study) 合并血脂异常的 2 型糖尿病患者在生活方式干预控制效果不佳时使用金利达治疗的有效性与安全性研究 (ENJOY LIFE 研究). Journal of diabetes, 7(2).	Not related to Oral CHM
3427	Upadya, M., & Rao, S. T. (2018). Hypertensive disorders in pregnancy. Indian journal of anaesthesia, 62(9), 675-681.	Not related to Oral CHM
3428	Santamarina AB, Jamar G, Mennitti LV, et al. Supplementation of Juçara Berry (Euterpe edulis Mart.) Modulates Epigenetic Markers in Monocytes from Obese Adults: A Double-Blind Randomized Trial. Nutrients. 2018;10(12):1899. Published 2018 Dec 3. doi:10.3390/nu10121899	Not related to Oral CHM
3429	Quirós-Fernández R, López-Plaza B, Bermejo LM, Palma-Milla S, Gómez-Candela C. Supplementation with Hydroxytyrosol and Punicalagin Improves Early Atherosclerosis Markers Involved in the Asymptomatic Phase of Atherosclerosis in the Adult Population: A Randomized, Placebo-Controlled, Crossover Trial. Nutrients. 2019;11(3):640. Published 2019 Mar 16. doi:10.3390/nu11030640	Not related to Oral CHM

3430	Ooi CP, Loke SC. Sweet potato for type 2 diabetes mellitus. Cochrane Database Syst Rev. 2012;(2):CD009128. Published 2012 Feb 15. doi:10.1002/14651858.CD009128.pub2	Not related to Oral CHM
3431	Wu X, Jiang X. Systematic review and meta analysis of randomized controlled trials on Tianmagouteng decoction in treatment of primary hypertension with liver Yang hyperactivity syndrome. J Tradit Chin Med. 2013;33(1):15-18. doi:10.1016/s0254-6272(13)60094-1	Not related to Oral CHM
3432	Wang Y, Hao L, Huo Z, Liu Y, Sun Y, Song Z. Systematic review and Meta-analysis of 26 randomized controlled clinical trials of Compound Danshen Dripping Pill for non-proliferating diabetic retinopathy. Chin Herb Med. 2021;14(1):142-153. Published 2021 Aug 4. doi:10.1016/j.chmed.2021.08.002	Not related to Oral CHM
3433	Xie WY, Zhang C, Xin JY, Li WH, Zhang TJ. Zhongguo Zhong Yao Za Zhi. 2023;48(2):542-554. doi:10.19540/j.cnki.cjcmm.20220926.502	Not related to Oral CHM
3434	Yeh GY, Eisenberg DM, Kaptchuk TJ, Phillips RS. Systematic review of herbs and dietary supplements for glycemic control in diabetes. Diabetes Care. 2003;26(4):1277-1294. doi:10.2337/diacare.26.4.1277	Not related to Oral CHM
3435	Salleh NH, Zulkipli IN, Mohd Yasin H, et al. Systematic Review of Medicinal Plants Used for Treatment of Diabetes in Human Clinical Trials: An ASEAN Perspective. Evid Based Complement Alternat Med. 2021;2021:5570939. Published 2021 Oct 13. doi:10.1155/2021/5570939	Not related to Oral CHM
3436	Palla AH, Amin F, Fatima B, et al. Systematic Review of Polyherbal Combinations Used in Metabolic Syndrome. Front Pharmacol. 2021;12:752926. Published 2021 Oct 7. doi:10.3389/fphar.2021.752926	Not related to Oral CHM
3437	Teixeira CC, Weinert LS, Barbosa DC, Ricken C, Esteves JF, Fuchs FD. Syzygium cumini (L.) Skeels in the treatment of type 2 diabetes: results of a randomized, double-blind, double-dummy, controlled trial. Diabetes Care. 2004;27(12):3019-3020. doi:10.2337/diacare.27.12.3019-a	Not related to Oral CHM
3438	Tabatabaei-Malazy O, Larijani B, Abdollahi M. Targeting metabolic disorders by natural products. J Diabetes Metab Disord. 2015;14:57. Published 2015 Jul 8. doi:10.1186/s40200-015-0184-8	Not related to Oral CHM
3439	Yuwen Y, Liu YQ, Wang YP, et al. The add-on effect of a Chinese herbal formula for patients with resistant hypertension: study protocol for a pilot cohort study. J Integr Med. 2015;13(2):122-128. doi:10.1016/S2095-4964(15)60162-5	Not related to Oral CHM
3440	Yang L, Jiang Y, Zhang Z, Hou J, Tian S, Liu Y. The anti-diabetic activity of licorice, a widely used Chinese herb. J Ethnopharmacol. 2020;263:113216. doi:10.1016/j.jep.2020.113216	Not related to Oral CHM
3441	Wu CE, Ye P. Zhonghua Xin Xue Guan Bing Za Zhi. 2006;34(10):886-889.	Not related to Oral CHM

3442	Zhou S, Xu H, Liu J, Fan X. The Clinical Effects of the Phlegm-Resolving Method in the Treatment of Obstructive Sleep Apnea-Hypopnea Syndrome: A Meta-Analysis of Randomized Controlled Trials. Evid Based Complement Alternat Med. 2022;2022:7426552. Published 2022 Jul 31. doi:10.1155/2022/7426552	Not related to Oral CHM
3443	Sun X, Guo L, Shang H, et al. The cost-effectiveness analysis of JinQi Jiangtang tablets for the treatment on prediabetes: a randomized, double-blind, placebo-controlled, multicenter design. Trials. 2015;16:496. Published 2015 Nov 3. doi:10.1186/s13063-015-0990-9	Not related to Oral CHM
3444	Zhao X, An X, Yang C, Sun W, Ji H, Lian F. The crucial role and mechanism of insulin resistance in metabolic disease. Front Endocrinol (Lausanne). 2023;14:1149239. Published 2023 Mar 28. doi:10.3389/fendo.2023.1149239	Not related to Oral CHM
3445	Mansouri M, Nayebi N, Keshtkar A, Hasani-Ranjbar S, Taheri E, Larijani B. The effect of 12 weeks Anethum graveolens (dill) on metabolic markers in patients with metabolic syndrome; a randomized double blind controlled trial. Daru. 2012;20(1):47. Published 2012 Oct 4. doi:10.1186/2008-2231-20-47	Not related to Oral CHM
3446	Mela DJ, Cao XZ, Dobriyal R, et al. The effect of 8 plant extracts and combinations on post-prandial blood glucose and insulin responses in healthy adults: a randomized controlled trial. Nutr Metab (Lond). 2020;17:51. Published 2020 Jul 6. doi:10.1186/s12986-020-00471-x	Not related to Oral CHM
3447	Rao A, Clayton P, Briskey D. The effect of an orally-dosed Gynostemma pentaphyllum extract (ActivAMP庐) on body composition in overweight, adult men and women: A double-blind, randomised, placebo-controlled study.聽J Hum Nutr Diet. 2022;35(3):583-589. doi:10.1111/jhn.12936	Not related to Oral CHM
3448	Tian H, Lu J, He H, et al. The effect of Astragalus as an adjuvant treatment in type 2 diabetes mellitus: A (preliminary) meta-analysis. J Ethnopharmacol. 2016;191:206-215. doi:10.1016/j.jep.2016.05.062	Not related to Oral CHM
3449	Yu W, Duan S, Yu Z. The effect of Bailing capsules combined with losartan to treat diabetic glomerulosclerosis and the combination's effect on blood and urine biochemistry. Am J Transl Res. 2021;13(6):6873-6880. Published 2021 Jun 15.	Not related to Oral CHM
3450	Safari Z, Farrokhzad A, Ghavami A, et al. The effect of barberry (Berberis vulgaris L.) on glycemic indices: A systematic review and meta-analysis of randomized controlled trials. Complement Ther Med. 2020;51:102414. doi:10.1016/j.ctim.2020.102414	Not related to Oral CHM
3451	Rosyid, F. N., Dharmana, E., Suwondo, A., & HS, K. H. N. (2018). The effect of bitter melon (Momordica charantia L.) leaves extract on TNF-α serum levels and diabetic foot ulcers improvement: randomized controlled trial. Biomedical and Pharmacology Journal, 11(3), 1413-1421.	Not related to Oral CHM
3452	Rosyid, F. N., Prasetyo, T. A., Prabawati, C. Y., & HS, K. H. N. (2021). The effect of bitter melon (Momordica charantia l.) leaves extractonon glycated albumin in diabetic foot ulcers: Randomized controlled trial. Bangladesh Journal of Medical Science, 20(2), 281-287.	Not related to Oral CHM

345<br>3	Xu, Y., Zheng, S., Jiang, S., Chen, J., Zhu, X., & Zhang, Y. (2022). The effect of Chinese herbal formulas combined with metformin on modulating the gut microbiota in the amelioration of type 2 diabetes mellitus: a systematic review and meta-analysis. Frontiers in Endocrinology, 13, 927959.	Not related to Oral CHM
345<br>4	Ren, W., Liao, J., Chen, J., Li, Z., & Huang, L. (2019). The effect of Chinese herbal medicine combined with western medicine on vascular endothelial function for patients with hypertension: Protocol for a systematic review and meta-analysis. Medicine, 98(49), e18134.	Not related to Oral CHM
345<br>5	Xiong, X., Wang, P., Li, X., & Zhang, Y. (2015). The effect of Chinese herbal medicine Jian Ling Decoction for the treatment of essential hypertension: a systematic review. Bmj Open, 5(2), e006502.	Not related to Oral CHM
345<br>6	Xiao, Y., Liu, Y., Yu, K., Zhou, L., Bi, J., Cheng, J., ... & Zhao, X. (2013). The effect of Chinese herbal medicine on albuminuria levels in patients with diabetic nephropathy: A systematic review and meta-analysis. Evidence-Based Complementary and Alternative Medicine, 2013(1), 937549.	Not related to Oral CHM
345<br>7	Pane, Y. S., Ganie, R. A., Lindarto, D., & Lelo, A. Z. N. A. N. (2018). The effect of gambier extract on the levels of malondialdehyde, superoxide dismutase, and blood glucose in type 2 diabetes mellitus patients. Asian J Pharm Clin Res, 11(10), 121-124.	Not related to Oral CHM
345<br>8	Mohammadi S, Aghili R, Malek M, et al. The effect of hesperidin and diosmin individually or in combination on metabolic profile and neuropathy among diabetic patients with metabolic syndrome: a randomized controlled trial. Phytother Res. 2019;33(1):131-139. doi:10.1002/ptr.6203. PMID:30238555.	Not related to Oral CHM
345<br>9	Mohagheghi, A., Maghsoud, S., Khashayar, P., & Ghazi-Khansari, M. (2011). The effect of Hibiscus sabdariffa on lipid profile, creatinine, and serum electrolytes: a randomized clinical trial. International Scholarly Research Notices, 2011(1), 976019.	Not related to Oral CHM
346<br>0	Ngondi JL, Oben JE, Minka SR. The effect of Irvingia gabonensis seeds on body weight and blood lipids of obese subjects in Cameroon. Lipids Health Dis. 2005;4:12. doi:10.1186/1476-511X-4-12. PMID:16236182.	Not related to Oral CHM
346<br>1	Zhou, B., Xia, H., Yang, L., Wang, S., & Sun, G. (2022). The effect of Lycium barbarum polysaccharide on the glucose and lipid metabolism: A systematic review and meta-analysis. Journal of the American Nutrition Association, 41(6), 617-625.	Not related to Oral CHM
346<br>2	Yang, C. W., & Mousa, S. A. (2012). The effect of red yeast rice (Monascus purpureus) in dyslipidemia and other disorders. Complementary Therapies in Medicine, 20(6), 466-474.	Not related to Oral CHM
346<br>3	Milajerdi A, Jazayeri S, Bitarafan V, et al. The effects of saffron supplementation on inflammatory and oxidative stress markers, and lipid profiles: a systematic review and meta-analysis of randomized controlled trials. Complement Ther Med. 2019;45:102-107.	Not related to Oral CHM

	doi:10.1016/j.ctim.2019.06.012. PMID:31351803.	
3464	Zhou, D., Zhang, L., Han, X., Gao, Y., Zeng, M., Yu, W., ... & Chen, Q. (2020). The effect of Sancai powder on glycemic variability of type 2 diabetes in the elderly: A randomized controlled trial. Medicine, 99(31), e20750.	Not related to Oral CHM
3465	Xie, P. C., Liang, Q. E., Tu, W. Q., Xie, T., Lam, L. K., & Chen, L. G. (2022). The effect of Taohong Siwu decoction combined with antihypertensive medicine in the treatment of hypertension: meta-analysis. Medicine, 101(49), e32133.	Not related to Oral CHM
3466	Zheng Y, Ding Q, Zhang L, et al. The effect of traditional Chinese medicine on gut microbiota in adults with type 2 diabetes: A protocol for systematic review and meta-analysis. Medicine (Baltimore). 2020;99(38):e22233. doi:10.1097/MD.0000000000022233	Not related to Oral CHM
3467	Ye P, Wu CE, Li H, Zhi G. Zhonghua Nei Ke Za Zhi. 2006;45(10):811-814.	Not related to Oral CHM
3468	Talibo, S. D., Tumenggung, I., Nawai, F., & Labatjo, R. (2021). The Effectiveness of the "Panjang" Poffertjes Consumption on Blood Glucose Level. Current Nutrition & Food Science, 17(7), 773-781.	Not related to Oral CHM
3469	Pan J, Xu Y, Chen S, et al. The Effectiveness of Traditional Chinese Medicine Jinlida Granules on Glycemic Variability in Newly Diagnosed Type 2 Diabetes: A Double-Blinded, Randomized Trial. J Diabetes Res. 2021;2021:6303063. Published 2021 Oct 8. doi:10.1155/2021/6303063	Not related to Oral CHM
3470	Mousavi SM, Beatriz Pizarro A, Akhgarjand C, et al. The effects of Anethum graveolens (dill) supplementation on lipid profile and glycemic control: a systematic review and meta-analysis of randomized controlled trials. Crit Rev Food Sci Nutr. 2022;62(21):5705-5716. doi:10.1080/10408398.2021.1889459	Not related to Oral CHM
3471	Soltani R, Ghanadian SM, Iraj B, Homayouni A, Esfahani TS, Akbari M. The Effects of Berberis integerrima Fruit Extract on Glycemic Control Parameters in Patients with Type 2 Diabetes Mellitus: A Randomized Controlled Clinical Trial. Evid Based Complement Alternat Med. 2021;2021:5583691. Published 2021 Jul 5. doi:10.1155/2021/5583691	Not related to Oral CHM
3472	Zhong, D. Y., Cheng, H., & Li, L. (2023). The effects of Buyang Huanwu decoction combined with western medicine in diabetic foot treatment: a systematic review and meta-analysis. TMR Integr Med, 7, e23010.	Not related to Oral CHM
3473	Wang Y, Cao HJ, Wang LQ, et al. The effects of Chinese herbal medicines for treating diabetic foot ulcers: A systematic review of 49 randomized controlled trials. Complement Ther Med. 2019;44:32-43. doi:10.1016/j.ctim.2019.03.007	Not related to Oral CHM

3474	Youovop, J., Takuissu, G., Mbopda, C., Nwang, F., Ntentié, R., Mbong, M. A., ... & Oben, J. (2023). The effects of Dyglomera (Dichrostachys glomerata extract) on body fat percentage and body weight: a randomized, double-blind, placebo-controlled clinical trial. Functional Foods in Health and Disease, 13(6), 334-346.	Not related to Oral CHM
3475	Shen Y, Wang S, Liu Y, et al. The Effects of Salvianolate Combined With Western Medicine on Diabetic Nephropathy: A Systematic Review and Meta-Analysis. Front Pharmacol. 2020;11:851. Published 2020 Jun 12. doi:10.3389/fphar.2020.00851	Not related to Oral CHM
3476	Fallah Huseini H, Nabavi SM, Shakeri F, Alipour M, Fallah F, Mohammadpour AH. The effects of silymarin on features of metabolic syndrome: a systematic review and meta-analysis. Phytother Res. 2020;34(8):1971-1983. doi:10.1002/ptr.6640	Not related to Oral CHM
3477	Wu, G., Liu, H., Ye, X., & Zhu, Y. (2002). The effects of tablet tongmaijiangzhi on hyperlipidemia and nitric oxide of patients with non-insulin-dependent diabetes mellitus. Zhong yao cai= Zhongyaocai= Journal of Chinese Medicinal Materials, 25(9), 690-692.	Not related to Oral CHM
3478	Liao HT, Peng C, Wang Y, Liu Y, Tian YJ, Chen J. Effects of Wenxin Keli on P-wave dispersion and maintenance of sinus rhythm in patients with paroxysmal atrial fibrillation: a meta-analysis of randomized controlled trials. BMC Complement Altern Med. 2017;17(1):393. doi:10.1186/s12906-017-1918-8	Not related to Oral CHM
3479	Zhang, Z., Leng, Y., Chen, Z., Fu, X., Liang, Q., Peng, X., ... & Xie, C. (2023). The efficacy and safety of Chinese herbal medicine as an add-on therapy for type 2 diabetes mellitus patients with carotid atherosclerosis: an updated meta-analysis of 27 randomized controlled trials. Frontiers in Pharmacology, 14, 1091718.	Not related to Oral CHM
3480	Li Y, Song D, Song D, et al. The efficacy and safety of Chinese herbal medicine in treatment of painful diabetic peripheral neuropathy: a systematic review and meta-analysis of 16 randomized controlled trials. Evid Based Complement Alternat Med. 2015;2015:324239. doi:10.1155/2015/324239	Not related to Oral CHM
3481	Zhang, Z., Leng, Y., Fu, X., Yang, C., Xie, H., Yuan, H., ... & Xie, C. (2022). The efficacy and safety of dachaihu decoction in the treatment of type 2 diabetes mellitus: a systematic review and meta-analysis. Frontiers in Pharmacology, 13, 918681.	Not related to Oral CHM
3482	Song, C. G., Bi, L. J., Zhao, J. J., Wang, X., Li, W., Yang, F., & Jiang, W. (2021). The efficacy and safety of Hirudin plus Aspirin versus Warfarin in the secondary prevention of Cardioembolic Stroke due to Nonvalvular Atrial Fibrillation: A multicenter prospective cohort study. International journal of medical sciences, 18(5), 1167.	Not related to Oral CHM

3483	Wang, L., Guan, B., Li, G., Feng, L., Sun, H., & Xu, J. (2022). The efficacy and safety of traditional Chinese medicine physiotherapy combined with acupoint injection on diabetic peripheral neuropathy: A protocol for systematic review and meta-analysis. Medicine, 101(50), e31467.	Not related to Oral CHM
3484	Zhao, Y., Qiu, J., Chen, T., Wang, S., Liu, S., Huang, H., & Wan, L. (2021). The efficacy and safety of traditional Chinese medicine's tonifying-kidney, strengthening-spleen, and invigorating-blood circulation (Bushen-Jianpi-Huoxue) principle for type 2 diabetes mellitus with osteoporosis: A protocol for systematic review and meta-analysis. Medicine, 100(12), e25197.	Not related to Oral CHM
3485	Park, S., Park, J. S., Go, H., Jang, B. H., Shin, Y., & Ko, S. G. (2011). The efficacy and safety study of dietary supplement PURIAM110 on non-insulin taking Korean adults in the stage of pre-diabetes and diabetes mellitus: protocol for a randomized, double-blind, placebo-controlled, and multicenter trial-pilot study. Trials, 12, 1-7.	Not related to Oral CHM
3486	Paocharoen, V. (2010). The efficacy and side effects of oral Centella asiatica extract for wound healing promotion in diabetic wound patients. J Med Assoc Thai, 93(Suppl 7), S166-S170.	Not related to Oral CHM
3487	Tam WY, Chook P, Qiao M, Chan LT, Chan TYK, Poon YK, Fung KP, Leung PC, Woo KS. The efficacy and tolerability of adjunctive alternative herbal medicine (Salvia miltiorrhiza and Pueraria lobata) on vascular function and structure in coronary patients. J Altern Complement Med. 2009;15(4):415-21. doi:10.1089/acm.2008.0400.	Not related to Oral CHM
3488	Wang JB, Wang YX, Li F, Li YF, Li XL, Huang PY, Wang C, Wang M, Qiu J, Yang KH, Qiu WW, Liu L, Mao BH, Li HP, Liu XD, Li FY, Cui XD, Wang PJ, Liu WB. The efficacy of Da Chaihu decoction combined with metformin tablets for type 2 diabetes mellitus: a systematic review and meta-analysis. Complement Ther Med. 2022;71:102894. doi:10.1016/j.ctim.2022.102894.	Not related to Oral CHM
3489	Teixeira CC, Fuchs FD, Weinert LS, Esteves J. The efficacy of folk medicines in the management of type 2 diabetes mellitus: results of a randomized controlled trial of Syzygium cumini (L.) Skeels. J Clin Pharm Ther. 2006;31(1):1-5. doi:10.1111/j.1365-2710.2006.00700.x.	Not related to Oral CHM
3490	Onakpoya I, Davies L, Posadzki P, Ernst E. The efficacy of Irvingia gabonensis supplementation in the management of overweight and obesity: a systematic review of randomized controlled trials. J Diet Suppl. 2013;10(1):29-38. doi:10.3109/19390211.2012.760508.	Not related to Oral CHM
3491	Zhou J, Chen Y, Yu J, Li T, Lu Z, Chen Y, Zhang X, Ye F. The efficacy of novel metabolic targeted agents and natural plant drugs for nonalcoholic fatty liver disease treatment: a PRISMA-compliant network meta-analysis of randomized controlled trials. Medicine (Baltimore). 2021;100(12):e24884. doi:10.1097/MD.0000000000024884.	Not related to Oral CHM

349 2	Wang S, Qiu XJ. The efficacy of Xue Fu Zhu Yu prescription for hyperlipidemia: a meta-analysis of randomized controlled trials. Complement Ther Med. 2019;43:218-226. doi:10.1016/j.ctim.2019.02.008.	Not related to Oral CHM
349 3	Sun S, Xu H, Ngeh L. The evaluation of Chinese therapeutic food for the treatment of moderate dyslipidemia. Evid Based Complement Alternat Med. 2012;2012:508683. doi:10.1155/2012/508683.	Not related to Oral CHM
349 4	Medagama AB. The glycaemic outcomes of Cinnamon, a review of the experimental evidence and clinical trials. Nutr J. 2015;14:108. doi:10.1186/s12937-015-0098-9.	Not related to Oral CHM
349 5	Wang H, Mu W, Zhai J, Xing D, Miao S, Wang J, Deng Y, Wang N, Chen H, Yang H, He X, Shang H. The key role of Shenyan Kangfu tablets, a Chinese patent medicine for diabetic nephropathy: study protocol for a randomized, double-blind and placebo-controlled clinical trial. Trials. 2013;14:165. doi:10.1186/1745-6215-14-165.	Not related to Oral CHM
349 6	Yu L, Zhou C, Luo Z, Zeng W, Lai F, Han G, Song Y. The lipid-lowering effects of Danhong and Huangqi injections: a meta-analysis of clinical controlled trials. Lipids Health Dis. 2018;17(1):106. doi:10.1186/s12944-018-0760-2.	Not related to Oral CHM
349 7	Sharma, P., Verma, P. K., Pankaj, N. K., & Agarwal, S. (2021). The Phytochemical Ingredients and Therapeutic Potential of Cynara scolymus L. Pharmaceutical and Biomedical Research.	Not related to Oral CHM
349 8	Wang J, He L, Yan W, Peng X, He L, Yang D, Liu H, Peng Y. The role of hypertriglyceridemia and treatment patterns in the progression of IgA nephropathy with a high proportion of global glomerulosclerosis. Int Urol Nephrol. 2020;52(2):325-335. doi:10.1007/s11255-019-02371-3.	Not related to Oral CHM
349 9	Zhao Y, Yu J, Liu J, An X. The role of liuwei dihuang pills and ginkgo leaf tablets in treating diabetic complications. Evid Based Complement Alternat Med. 2016;2016:7931314. doi:10.1155/2016/7931314.	Not related to Oral CHM
350 0	Miraj, S. (2016). The role of medicinal plants in the treatment of diseases: A systematic review of Calendula officinalis. Der Pharmacia Lettre, 8(14), 92-95.	Not related to Oral CHM
350 1	Zhang X, Sun H, Paul SK, Wang Q, Lou X, Hou G, Wen B, Ji L, Liu S. The serum protein responses to treatment with Xiaoke Pill and Glibenclamide in type 2 diabetes patients. Clin Proteomics. 2017;14:19. doi:10.1186/s12014-017-9154-0.	Not related to Oral CHM
350 2	Thomas A, Rajesh EK, Kumar DS. The Significance of Tinospora crispa in Treatment of Diabetes Mellitus. Phytother Res. 2016;30(3):357-366. doi:10.1002/ptr.5559.	Not related to Oral CHM
350 3	Oben J, Kuate D, Agbor G, Momo C, Talla X. The use of a Cissus quadrangularis formulation in the management of weight loss and metabolic syndrome. Lipids Health Dis. 2006;5:24. doi:10.1186/1476-511X-5-24.	Not related to Oral CHM

3504	Onakpoya I, Terry R, Ernst E. The use of green coffee extract as a weight loss supplement: a systematic review and meta-analysis of randomised clinical trials. Gastroenterol Res Pract. 2011;2011:382852. doi:10.1155/2011/382852.	Not related to Oral CHM
3505	Xie D, Li K, Ma T, et al. Therapeutic Effect and Safety of Tripterygium Glycosides Combined With Western Medicine on Type 2 Diabetic Kidney Disease: A Meta-Analysis. Clin Ther. 2022;44(2):246-256.e10. doi:10.1016/j.clinthera.2021.12.006	Not related to Oral CHM
3506	Zhao Y, Yu X, Lou Y, et al. Therapeutic Effect of Abelmoschus manihot on Type 2 Diabetic Nonproliferative Retinopathy and the Involvement of VEGF. Evid Based Complement Alternat Med. 2020;2020:5204917. Published 2020 Apr 30. doi:10.1155/2020/5204917	Not related to Oral CHM
3507	Zhong ZM, Yu L, Weng ZY, et al. Nan Fang Yi Ke Da Xue Xue Bao. 2007;27(5):682-684.	Not related to Oral CHM
3508	Sun J, Ren J, Hu X, Hou Y, Yang Y. Therapeutic effects of Chinese herbal medicines and their extracts on diabetes. Biomed Pharmacother. 2021;142:111977. doi:10.1016/j.biopha.2021.111977	Not related to Oral CHM
3509	You WH, Wang P, Li MQ, Zhang Y, Peng YL, Zhang FL. Therapeutic effects of modified Danggui Sini Decoction on plasma level of advanced glycation end products in patients with Wagner grade 0 diabetic foot: a randomized controlled trial. Zhong Xi Yi Jie He Xue Bao. 2009;7(7):622-628. doi:10.3736/jcim20090705	Not related to Oral CHM
3510	Ye C, Gu L, Feng Y, Zhou F, She W. Therapeutic effects of Yiqi Huoxue prescription on diabetic nephropathy: a meta-analysis and systematic review. Ann Palliat Med. 2021;10(6):6617-6629. doi:10.21037/apm-21-1147	Not related to Oral CHM
3511	Ye C, Gu L, Feng Y, Zhou F, She W. Therapeutic effects of Yiqi Huoxue prescription on diabetic nephropathy: a meta-analysis and systematic review. Ann Palliat Med. 2021;10(6):6617-6629. doi:10.21037/apm-21-1147	Not related to Oral CHM
3512	Martinet A, Hostettmann K, Schutz Y. Thermogenic effects of commercially available plant preparations aimed at treating human obesity. Phytomedicine. 1999;6(4):231-238. doi:10.1016/S0944-7113(99)80014-2	Not related to Oral CHM
3513	Muzhikov V, Vershinina E, Muzhikov R, Nikitin K. Thermopuncture for the Diagnosis, Monitoring, and Treatment of Patients with Type 2 Diabetes. J Acupunct Meridian Stud. 2018;11(5):323-331. doi:10.1016/j.jams.2018.05.004	Not related to Oral CHM
3514	Wang J, Feng B, Yang X, et al. Tianma gouteng yin as adjunctive treatment for essential hypertension: a systematic review of randomized controlled trials. Evid Based Complement Alternat Med. 2013;2013:706125. doi:10.1155/2013/706125	Not related to Oral CHM
3515	郑冬冬,王锋,姜笃银.TIME原则指导下中西医结合治疗糖尿病性溃疡 1 例[J].感染、炎症、修复,2017,18(01):22+64-65.	Not related to Oral CHM

3516	Wu T, Harrison RA, Chen X, et al. Tongxinluo (Tong xin luo or Tong-xin-luo) capsule for unstable angina pectoris. Cochrane Database Syst Rev. 2006;2006(4):CD004474. Published 2006 Oct 18. doi:10.1002/14651858.CD004474.pub2	Not related to Oral CHM
3517	Wu XM, Gao YB, Xu LP, et al. Tongxinluo Inhibits Renal Fibrosis in Diabetic Nephropathy: Involvement of the Suppression of Intercellular Transfer of TGF-[Formula: see text]1-Containing Exosomes from GECs to GMCs. Am J Chin Med. 2017;45(5):1075-1092. doi:10.1142/S0192415X17500586	Not related to Oral CHM
3518	Zheng C, Ou W, Shen H, Zhou Z, Wang J. Combined therapy of diabetic peripheral neuropathy with breviscapine and mecobalamin: a systematic review and a meta-analysis of Chinese studies. Biomed Res Int. 2015;2015:680756. doi:10.1155/2015/680756	Not related to Oral CHM
3519	Pang B, Li QW, Qin YL, et al. Traditional chinese medicine for diabetic retinopathy: A systematic review and meta-analysis. Medicine (Baltimore). 2020;99(7):e19102. doi:10.1097/MD.0000000000019102	Not related to Oral CHM
3520	Zhang Y, Wang B, Ju C, et al. Traditional Chinese Medicine for Essential Hypertension: A Clinical Evidence Map. Evid Based Complement Alternat Med. 2020;2020:5471931. Published 2020 Dec 18. doi:10.1155/2020/5471931	Not related to Oral CHM
3521	Sun W, Li J, Yan X, et al. Traditional Chinese Medicine Injections for Diabetic Retinopathy: A Systematic Review and Network Meta-Analysis of Randomized Controlled Trials. J Integr Complement Med. 2022;28(12):927-939. doi:10.1089/jicm.2021.0392	Not related to Oral CHM
3522	Tan L, Shi Q, Liu C, Zhang J, Wang H, Zhai J. Traditional Chinese Medicine Injections in the Treatment of Diabetic Foot: A Systematic Review and Meta-Analysis. Evid Based Complement Alternat Med. 2018;2018:4730896. Published 2018 Oct 8. doi:10.1155/2018/4730896	Not related to Oral CHM
3523	Wang Q, Lin J, Li C, et al. Traditional Chinese medicine method of tonifying kidney for hypertension: Clinical evidence and molecular mechanisms. Front Cardiovasc Med. 2022;9:1038480. Published 2022 Nov 16. doi:10.3389/fcvm.2022.1038480	Not related to Oral CHM
3524	Yu XD, Wang JS, Zuo G, et al. Traditional Chinese medicine on treating diabetic mellitus erectile dysfunction: Protocol for a systematic review and meta-analysis. Medicine (Baltimore). 2019;98(13):e14928. doi:10.1097/MD.0000000000014928	Not related to Oral CHM
3525	Zhu Q, Kang J, Xu G, Li J, Zhou H, Liu Y. Traditional Chinese medicine Shenqi compound to improve lower extremity atherosclerosis of patients with type 2 diabetes by affecting blood glucose fluctuation: Study protocol for a randomized controlled multicenter trial. Medicine (Baltimore). 2020;99(11):e19501. doi:10.1097/MD.0000000000019501	Not related to Oral CHM
3526	Wang, Z, Wang, Y, Liu, Y, Liu, Y and Li, L, 2020, Traditional Chinese medicine treatment for atrial fibrillation: An overview of systematic reviews, Chinese Journal of Evidence-Based Medicine	Not related to Oral CHM

3527	Wang, Y, Liu, Y, Wang, Z, Liu, Y and Li, L, 2019, Traditional Chinese medicine treatment for essential hypertension from 2015 to 2019: An overview of systematic reviews, Chinese Journal of Evidence-Based Medicine	Not related to Oral CHM
3528	Pang B, Ni Q, Lin YQ, et al. Traditional Chinese Patent Medicine for Treating Impaired Glucose Tolerance: A Systematic Review and Meta-Analysis of Randomized Controlled Trials. J Altern Complement Med. 2018;24(7):634-655. doi:10.1089/acm.2017.0302	Not related to Oral CHM
3529	Yadav SS, Singh MK, Singh PK, Kumar V. Traditional knowledge to clinical trials: A review on therapeutic actions of Emblica officinalis. Biomed Pharmacother. 2017;93:1292-1302. doi:10.1016/j.biopha.2017.07.065	Not related to Oral CHM
3530	Obakiro SB, Kiprop A, Kigondu E, et al. Traditional Medicinal Uses, Phytoconstituents, Bioactivities, and Toxicities of Erythrina abyssinica Lam. ex DC. (Fabaceae): A Systematic Review. Evid Based Complement Alternat Med. 2021;2021:5513484. Published 2021 Mar 3. doi:10.1155/2021/5513484	Not related to Oral CHM
3531	Tong XL, Dong L, Chen L, Zhen Z. Treatment of diabetes using traditional Chinese medicine: past, present and future. Am J Chin Med. 2012;40(5):877-886. doi:10.1142/S0192415X12500656	Not related to Oral CHM
3532	Zheng, H., Fang, F., Chen, W., Liu, L., Feng, X., & Xu, J. (2004). Treatment of diabetic foot disease by recombinant bovine basic fibroblast growth factor: randomized controlled observation on the therapeutic effect (Chinese)[J]. Chinese Journal of Clinical Rehabilitation, 8, 6564-5.	Not related to Oral CHM
3533	Shao G. Zhong Xi Yi Jie He Za Zhi. 1985;5(11):652-642.	Not related to Oral CHM
3534	Zhang, D. Y., Cheng, Y. B., Guo, Q. H., Shan, X. L., Wei, F. F., Lu, F., ... & Wang, J. G. (2020). Treatment of masked hypertension with a Chinese herbal formula: a randomized, placebo-controlled trial. Circulation, 142(19), 1821-1830.	Not related to Oral CHM
3535	Zhang DY, Cheng YB, Guo QH, et al. Treatment of Masked Hypertension with a Chinese Herbal Formula: A Randomized, Placebo-Controlled Trial. Circulation. 2020;142(19):1821-1830. doi:10.1161/CIRCULATIONAHA.120.046685	Not related to Oral CHM
3536	Zhao X, Liu L, Liu J. Treatment of type 2 diabetes mellitus using the traditional Chinese medicine Jinlida as an add-on medication: A systematic review and meta-analysis of randomized controlled trials. Front Endocrinol (Lausanne). 2022;13:1018450. Published 2022 Oct 17. doi:10.3389/fendo.2022.1018450	Not related to Oral CHM
3537	Piao C, Zhang Q, Jin D, et al. Treatment of Type 2 diabetes with Tianqi Jiangtang Capsule: A systematic review and meta-analysis of randomized controlled trials. Medicine (Baltimore). 2020;99(21):e19702. doi:10.1097/MD.0000000000019702	Not related to Oral CHM

3538	Zaidi SM. Unani treatment and leech therapy saved the diabetic foot of a patient from amputation. Int Wound J. 2016;13(2):263-264. doi:10.1111/iwj.12285	Not related to Oral CHM
3539	Yan Y, Zhou X, Guo K, Zhou F, Yang H. Use of Chlorogenic Acid against Diabetes Mellitus and Its Complications. J Immunol Res. 2020;2020:9680508. Published 2020 May 28. doi:10.1155/2020/9680508	Not related to Oral CHM
3540	Yeh GY, Davis RB, Phillips RS. Use of complementary therapies in patients with cardiovascular disease. Am J Cardiol. 2006;98(5):673-680. doi:10.1016/j.amjcard.2006.03.051	Not related to Oral CHM
3541	Tsai YY, Chen KJ, Yang YH, Lin YH. Use of Traditional Chinese Medicine May Delay the Need for Insulin Treatment in Patients with Type 2 Diabetes: A Population-Based Cohort Study. J Altern Complement Med. 2020;26(7):628-635. doi:10.1089/acm.2019.0375	Not related to Oral CHM
3542	Xia S, Gao B, Chen S, et al. Verification of the Efficacy and Safety of Qi-Replenishing Chinese Medicine in Treating Prediabetes: A Meta-Analysis and Literature Review. Evid Based Complement Alternat Med. 2020;2020:7676281. Published 2020 Nov 10. doi:10.1155/2020/7676281	Not related to Oral CHM
3543	Meng Z, Tan J, He Q, et al. Wenxin Keli versus Sotalol for Paroxysmal Atrial Fibrillation Caused by Hyperthyroidism: A Prospective, Open Label, and Randomized Study. Evid Based Complement Alternat Med. 2015;2015:101904. doi:10.1155/2015/101904	Not related to Oral CHM
3544	Li M, He Q, Chen Y, et al. Xuezhikang Capsule for Type 2 Diabetes with Hyperlipemia: A Systematic Review and Meta-Analysis of Randomized Clinical Trails. Evid Based Complement Alternat Med. 2015;2015:468520. doi:10.1155/2015/468520	Not related to Oral CHM
3545	Zhao SP, Liu L, Cheng YC, et al. Xuezhikang, an extract of cholestin, protects endothelial function through antiinflammatory and lipid-lowering mechanisms in patients with coronary heart disease. Circulation. 2004;110(8):915-920. doi:10.1161/01.CIR.0000139985.81163.CE	Not related to Oral CHM
3546	Panthi S, Jing X, Gao C, Gao T. Yang-warming method in the treatment of diabetic peripheral neuropathy: an updated systematic review and meta-analysis. BMC Complement Altern Med. 2017;17(1):424. Published 2017 Aug 25. doi:10.1186/s12906-017-1927-5	Not related to Oral CHM
3547	Ou C, Yang YJ, Peng QH. Yiqi Yangyin Huoxue Method in Treating Diabetic Retinopathy: A Systematic Review and Meta-Analysis. Evid Based Complement Alternat Med. 2019;2019:6020846. Published 2019 Apr 2. doi:10.1155/2019/6020846	Not related to Oral CHM
3548	Xiong X, Yang X, Feng B, et al. Zhen gan xi feng decoction, a traditional chinese herbal formula, for the treatment of essential hypertension: a systematic review of randomized controlled trials. Evid Based Complement Alternat Med. 2013;2013:982380. doi:10.1155/2013/982380	Not related to Oral CHM

3549	Zhu, J. X., Xia, Z. B., Min, J. K., Hu, W. L., Li, H., & Mei, C. (2023). Zuogui pill combined with calcium versus alendronate combined with calcium in patients with diabetes induced abnormal bone metabolism: an efficacy and safety analysis.	Not related to Oral CHM
3550	张翠,刘克冕,吴敏.八味祛湿汤外治糖尿病并足癣 1 例[J].山东中医杂志,2016,35(10):918+936.DOI:10.16295/j.cnki.0257-358x.2016.10.026.	Not related to Oral CHM
3551	赵清树,王福彦,刘云,等.白虎消渴方并四妙葛根芩连汤加味治疗 2 型糖尿病随机对照研究[J].内蒙古中医药,2015,34(10):1-2.DOI:10.16040/j.cnki.cn15-1101.2015.10.002.	Not related to Oral CHM
3552	王定良.斑蝥中毒致急性肾功能衰竭并继发性血小板减少性脑出血死亡 1 例[J].临床合理用药杂志,2012,5(02):102.DOI:10.15887/j.cnki.13-1389/r.2012.02.103.	Not related to Oral CHM
3553	吴启锋,温茂祥,兰东辉.半夏白术天麻汤对痰湿壅盛型高血压病盐敏感性及血脂水平的影响[J].福建医药杂志,2007,(01):146-148.	Not related to Oral CHM
3554	赵爱静.半夏白术天麻汤对痰湿壅盛型高血压的治疗价值[J].内蒙古中医药,2016,35(06):16-17.DOI:10.16040/j.cnki.cn15-1101.2016.06.020.	Not related to Oral CHM
3555	张英杰,张宗礼.半夏白术天麻汤加减治疗高血压肾病经验 2 则[J].河南中医,2013,33(09):1565.DOI:10.16367/j.issn.1003-5028.2013.09.014.	Not related to Oral CHM
3556	张凌凌,戈盾.半夏白术天麻汤联合隔药灸治疗痰湿壅盛型原发性高血压的临床疗效及对血管内皮损伤标志物和早期肾损伤指标的影响[J].河北中医,2021,43(05):751-755+761.	Not related to Oral CHM
3557	姚丰国.半夏白术天麻汤联合左旋氨氯地平治疗痰湿壅盛型高血压的效果[J].河南医学研究,2019,28(17):3194-3196.	Not related to Oral CHM
3558	吴莹.半夏白术天麻汤治疗痰湿壅盛型肥胖性高血压病及对患者免疫功能的影响[J].哈尔滨医药,2020,40(05):482-483.	Not related to Oral CHM
3559	张真,戴斌.半夏白术天麻汤治疗眩晕高血压病举隅[J].中国中医药现代远程教育,2020,18(06):100-102.	Not related to Oral CHM
3560	祁琳,李大勇.辨证分期联合西药治疗糖尿病足 32 例临床观察[J].实用中医内科杂志,2014,28(06):50-52.DOI:10.13729/j.issn.1671-7813.2014.06.24.	Not related to Oral CHM
3561	吴北军.辨证分型联合西药治疗老年高血压随机平行对照研究[J].实用中医内科杂志,2017,31(09):42-44.DOI:10.13729/j.issn.1671-7813.2017.09.15.	Not related to Oral CHM
3562	苏小友.辨证分型联合西药治疗糖尿病周围神经病变对照研究[J].实用中医内科杂志,2012,26(02):47+49.	Not related to Oral CHM
3563	姚慧蓉,李新平,龚循生.辨证分型治疗肥胖症临床观察[J].新中医,2013,45(10):29-30.DOI:10.13457/j.cnki.jncm.2013.10.051.	Not related to Oral CHM
3564	尹小军.辨证分型治疗老年高血压随机平行对照研究[J].实用中医内科杂志,2014,28(08):61-63.DOI:10.13729/j.issn.1671-7813.2014.08.30	Not related to Oral CHM
3565	武强,李文刚.辨证分型治疗糖尿病足坏疽 224 例[J].中国中西医结合外科杂志,2012,18(01):68-69.	Not related to Oral CHM
3566	杨勇.辨证分型治疗原发性高血压随机平行对照研究[J].实用中医内科杂志,2015,29(08):15-16.DOI:10.13729/j.issn.1671-7813.2015.08.08.	Not related to Oral CHM
3567	郑顺海.辨证分型中药汤剂联合针刺治疗高血压随机平行对照研究[J].实用中医内科杂志,2016,30(01):109-110+123.DOI:10.13729/j.issn.1671-7813.2016.01.49.	Not related to Oral CHM

3568	徐伟.辨证施治联合西药治疗高血压患者临床效果观察[J].基层医学论坛,2017,21(25):3341-3342.DOI:10.19435/j.1672-1721.2017.25.014.	Not related to Oral CHM
3569	彭宁,雷鹏,王万贵,等.辨证施治糖尿病神经原性膀胱功能障碍 24 例[J].陕西中医,2005,(12):1337-1338+1356.	Not related to Oral CHM
3570	赵坤.辨证治疗糖尿病肾病 84 例[J].中国中医药现代远程教育,2012,10(24):20.	Not related to Oral CHM
3571	郑仲华,郑金艳.补气活血法治疗糖尿病足 50 例[J].中医研究,2011,24(11):43-46.	Not related to Oral CHM
3572	张芸芸,欧阳斌.补肾活血法治疗早期糖尿病肾病 1 例[J].河南中医,2013,33(03):446-447.DOI:10.16367/j.issn.1003-5028.2013.03.055.	Not related to Oral CHM
3573	袁冬.补肾活血化浊方治疗糖尿病肾病 43 例[J].山东中医杂志,2013,32(05):313-314.DOI:10.16295/j.cnki.0257-358x.2013.05.027.	Not related to Oral CHM
3574	曲政军,郭黎明.补肾温阳利水法治疗老年人高血压 45 例[J].山东中医杂志,1997,(09):11-12.	Not related to Oral CHM
3575	徐毅.参桂五味复脉汤配合西医常规治疗缓慢性心律失常 45 例[J].中医研究,2011,24(08):36-38.	Not related to Oral CHM
3576	闫琴,刘凯,包培荣.参苓白术散合用培菲康治疗脾虚湿盛型高脂血症疗效观察[J].山东中医杂志,2010,29(07):441-443.DOI:10.16295/j.cnki.0257-358x.2010.07.018.	Not related to Oral CHM
3577	周红缨. (2015). 参苓白术散加味治疗糖尿病性腹泻 81 例. 今日健康, 14(8), 28-28.	Not related to Oral CHM
3578	杨艳.参松养心胶囊、氨茶碱联合应用治疗缓慢性心律失常 31 例[J].中国中医药科技,2013,20(05):567-568.	Not related to Oral CHM
3579	薛金娜.柴胡温胆汤治疗痰湿型 2 型糖尿病患者临床疗效研究[J].当代医学,2018,24(24):1-3.	Not related to Oral CHM
3580	张梦婷, 2022, 出血性中风的中医证素分布规律与天麻钩藤饮临床疗效的Meta分析, 湖南中医药大学	Not related to Oral CHM
3581	周汝云,温伟强,谭宁,等.除湿化瘀方治疗高血尿酸血症 22 例临床观察[J].医学研究杂志,2009,38(04):136-137+95.	Not related to Oral CHM
3582	王世春, & 梁志军. (2008). 川芎嗪注射液静脉给药致血压升高心脏猝死 1 例报告. 现代保健: 医学创新研究, 5(2), 117-117.	Not related to Oral CHM
3583	赵信科. (2018). 从 Rho/ROCK 通路探讨地龙降压胶囊干预高血压左心室肥厚的临床与实验研究 (Doctoral dissertation, 兰州大学).	Not related to Oral CHM
3584	严景妍,李兵,曾治君.从二阳结论治 2 型糖尿病Meta分析[J].中医临床研究,2018,10(19):139-143.	Not related to Oral CHM
3585	殷文慧,刘姣林.从虚辨证原发性高血压循证治疗 53 例疗效观察[J].内蒙古医学杂志,2005,(06):536-537.	Not related to Oral CHM
3586	赵玉敏,胡艳萍.从浊毒论治高效抗逆转录病毒疗法相关代谢综合征[J].中医学报,2017,32(01):1-3.DOI:10.16368/j.issn.1674-8999.2017.01.001.	Not related to Oral CHM
3587	郑文静.丹瓜化浊祛瘀汤治疗痰瘀互结型糖尿病下肢血管病变 60 例[J].浙江中医杂志,2021,56(10):732.DOI:10.13633/j.cnki.zjtcm.2021.10.021.	Not related to Oral CHM
3588	伍新林,李俊彪,敖勤兴,等.丹芍汤治疗阴虚湿热型糖尿病肾病的临床研究[J].中药材,2006,(04):411-414.DOI:10.13863/j.issn1001-4454.2006.04.040.	Not related to Oral CHM

3589	张敏.单味蒲黄临床治验 3 则[J].浙江中医杂志,2001,(11):36.	Not related to Oral CHM
3590	王森.党参桂枝方联合降压治疗对重症风心病心功能和血流动力学的影响[J].四川中医,2016,34(09):72-74.	Not related to Oral CHM
3591	张广清,邱定荣.邓铁涛浴足方治疗高血压病 120 例临床观察[J].中医杂志,2005,(11):826-828.DOI:10.13288/j.11-2166/r.2005.11.016.	Not related to Oral CHM
3592	张晓阳.电针耳压配药茶治疗单纯性肥胖症 82 例[J].四川中医,2008,(02):112-113.	Not related to Oral CHM
3593	王双贺.调肝活血降浊方治疗中青年原发性高血压随机平行对照研究[J].实用中医内科杂志,2015,29(08):25-26.DOI:10.13729/j.issn.1671-7813.2015.08.13.	Not related to Oral CHM
3594	熊冠宇,朱涛.调质降压方联合苯磺酸氨氯地平片治疗痰湿壅盛型原发性高血压 40 例[J].中医研究,2021,34(02):20-24.	Not related to Oral CHM
3595	王乡宁, & 付子俊. (2013). 多中心随机对照试验: 再生复原技术治疗各种开放性创面的疗效评价. 中国烧伤创疡杂志, 25(3), 211-229.	Not related to Oral CHM
3596	徐婷贞,杨晓明.二陈解鼾颗粒对阻塞性睡眠呼吸暂停低通气综合征患者炎症因子的影响[J].浙江中医药大学学报,2018,42(10):862-866.DOI:10.16466/j.issn1005-5509.2018.10.020.	Not related to Oral CHM
3597	杨传印.二术二陈汤治疗痰湿型高脂血症 94 例[J].河南中医学院学报,2005,(04):54-55.DOI:10.16368/j.issn.1674-8999.2005.04.030	Not related to Oral CHM
3598	袁向明.分期辨证治疗糖尿病足 32 例疗效分析[J].中医药通报,2009,8(06):45-46+44.	Not related to Oral CHM
3599	莫玉萱,胡黎明,唐志清,等.蜂蜜加云南白药治疗糖尿病足 32 例[J].广西医学,2009,31(12):1902-1903.	Not related to Oral CHM
3600	王俊锋,李莉,胡晓梅.蜂蜜纱条湿敷治疗老年糖尿病患者感染创面 2 例报道[J].辽宁中医学院学报,2006,(02):79-80.DOI:10.13194/j.jlunivtcm.2006.02.81.wangjf.047.	Not related to Oral CHM
3601	王媛媛,冯志海.冯志海教授治疗湿热型肥胖验案 2 则[J].中国中医药现代远程教育,2017,15(04):129-131.	Not related to Oral CHM
3602	闫春艳.复方丹参滴丸联合美西律治疗难治性室性早搏 140 例[J].实用中西医结合临床,2014,14(02):21+88.DOI:10.13638/j.issn.1671-4040.2014.02.012.	Not related to Oral CHM
3603	瞿艳.复方丹参注射液配合甲硝唑治疗褥疮 20 例[J].河北中医,2009,31(09):1383.	Not related to Oral CHM
3604	韦无边,卢柳伊.复方甘草酸苷片致血压急骤增高 2 例[J].中国医药指南,2011,9(25):322-323.DOI:10.15912/j.cnki.gocm.2011.25.184.	Not related to Oral CHM
3605	孙智辉,李艳妍,陶娌娜,等.复方甘草酸苷注射液致血压升高 1 例[J].中国医院用药评价与分析,2013,13(06):574-575.DOI:10.14009/j.issn.1672-2124.2013.06.010.	Not related to Oral CHM
3606	王军梅.甘露饮加减对湿热困脾证 2 型糖尿病中医证候和糖脂代谢水平的影响[J].四川中医,2015,33(12):80-82.	Not related to Oral CHM
3607	李芳,林岚,黄清莲.冠心宁注射液致较严重不良反应 1 例[J].海峡药学,2017,29(05):265.	Not related to Oral CHM

3608	王涓,张敏.光子治疗仪照射配合疮灵液湿敷在肺癌合并糖尿病足病人护理中的应用[J].护理研究,2017,31(08):1018-1020.	Not related to Oral CHM
3609	圣洪平,许敏芳,徐俊良.荷丹片治疗高脂血症疗效观察与中医辨证分型研究[J].中国社区医师,2016,32(35):125-126.	Not related to Oral CHM
3610	王斌,邬金玲,罗昆,等.化浊解毒法干预 2 型糖尿病合并高脂血症临床研究[J].山东中医杂志,2011,30(05):304-306.DOI:10.16295/j.cnki.0257-358x.2011.05.007.	Not related to Oral CHM
3611	张瑞,张建飞,申丽,等.活络通痹汤足浴联合前列地尔治疗糖尿病周围神经病变 56 例临床观察[J].中国中医药科技,2016,23(05):571-572.	Not related to Oral CHM
3612	张孙伟,刘湘华.活血补肾方联合西药治疗糖尿病肾病随机平行对照研究[J].实用中医内科杂志,2014,28(07):129-131.DOI:10.13729/j.issn.1671-7813.2014.07.59.	Not related to Oral CHM
3613	邵中兴, 顾宁. 活血祛瘀, 滋阴化浊中药影响冠心病伴血脂异常患者血脂水平的临床观察[J]. 中医学报, 2014 (B12): 38-38.	Not related to Oral CHM
3614	钟黎黎.活血通脉方治疗糖尿病周围神经病变随机平行对照研究[J].实用中医内科杂志,2016,30(12):33-36.DOI:10.13729/j.issn.1671-7813.2016.12.14.	Not related to Oral CHM
3615	唐丽珠,麻华胆,隆采丹,等.基于对BMP7 的调控探讨MEBO对糖尿病创面愈合的影响[J].右江医学,2021,49(12):881-885.	Not related to Oral CHM
3616	张亚忠,于美虹.加用十一方药酒外敷治疗糖尿病足的临床观察[J].广西中医学院学报,2011,14(01):21-22.	Not related to Oral CHM
3617	张穗娥,董彦敏,陶加平.健脾化痰药膳治疗女性单纯性肥胖的临床研究[J].广州中医药大学学报,2006,(03):209-211.	Not related to Oral CHM
3618	邱云芝,文春盈,钱艳飞,等.康复新液联合胰岛素及红外线烤灯对糖尿病压疮的疗效观察[J].当代护士(下旬刊),2016,(01):120-121.	Not related to Oral CHM
3619	裴世荣,郝平,周洁松,等.康复新液联合远红外线照射治疗糖尿病足的临床研究[J].中国美容医学,2008,17(12):1738-1741.	Not related to Oral CHM
3620	施凯莉.康复新液湿敷治疗糖尿病足 24 例疗效观察及护理体会[J].中医外治杂志,2010,19(05):26-27.	Not related to Oral CHM
3621	王盈盈.康复新液与重组牛碱性成纤维细胞生长因子对早期糖尿病足的疗效观察[J].福建医药杂志,2014,36(05):99-100+153.DOI:10.20148/j.fmj.2014.05.042.	Not related to Oral CHM
3622	丘伟中,闵存云.苦碟子注射液合外用康复新液加吹氧治疗糖尿病足 33 例临床观察[J].新中医,2008,(01):38-39.DOI:10.13457/j.cnki.jncm.2008.01.070.	Not related to Oral CHM
3623	王宝.老年代谢综合征中医证候特征及中药干预疗效评价的初步研究[D].北京中医药大学,2014.	Not related to Oral CHM
3624	徐占国. 老年高血压病中医治疗体会[J]. 中国医药导报, 2010, 7(31): 77-78.	Not related to Oral CHM
3625	张太.理气活血化浊汤治疗气血瘀滞型高脂血症临床研究[J].中医学报,2012,27(04):481-482.DOI:10.16368/j.issn.1674-8999.2012.04.037.	Not related to Oral CHM
3626	王奎刚.苓桂术甘汤加味治疗糖尿病并发症验案 2 则[J].中国民间疗法,2019,27(21):91-92.DOI:10.19621/j.cnki.11-3555/r.2019.2147.	Not related to Oral CHM

3627	魏兴宏.六味地黄汤联合西药治疗高血压随机平行对照研究[J].实用中医内科杂志,2014,28(03):114-116.DOI:10.13729/j.issn.1671-7813.2014.03.57.	Not related to Oral CHM
3628	周晓勇.内外并治糖尿病肢端坏疽 32 例分析[J].中医药学刊,2004,(04):714.DOI:10.13193/j.archtcm.2004.04.136.zhouxy.084.	Not related to Oral CHM
3629	乌新春,翟玉普,张生,等.复方丹参滴丸辅助前列地尔治疗糖尿病肾病III期的临床观察[J].临床合理用药杂志,2016,9(01):69-70.DOI:10.15887/j.cnki.13-1389/r.2016.01.039.	Not related to Oral CHM
3630	朴益熙.清热健脾法治疗代谢综合征文献研究[D].北京中医药大学,2015.	Not related to Oral CHM
3631	田立茹,吴凡,杨帆,等.缺血性卒中体质状态与中风复发巢式病例对照研究[J].河北中医,2019,41(01):16-20.	Not related to Oral CHM
3632	宋易华,刘满君,李永清,等.四妙勇安汤加减配合西药治疗糖尿病足 25 例[J].四川中医,2006,(07):64.	Not related to Oral CHM
3633	王丽娜, 刘宝丽, & 王金梅. (2017). 糖尿病合并甲状腺疾病的临床疗效分析. 糖尿病新世界, 20(9), 42-43.	Not related to Oral CHM
3634	张红旗,李全香.糖尿病坏疽中医药治疗 36 例疗效观察[J].世界最新医学信息文摘,2019,19(04):173-174.DOI:10.19613/j.cnki.1671-3141.2019.4.113.	Not related to Oral CHM
3635	任明,霍达,孙晓,等.糖尿病前期中医证型分布与演变规律的多中心临床研究[J].中医杂志,2018,59(09):769-772.DOI:10.13288/j.11-2166/r.2018.09.013.	Not related to Oral CHM
3636	张月.糖尿病神经性膀胱非尿潴留的中医证候及验案分析[J].中西医结合心脑血管病杂志,2017,15(09):1096-1099.	Not related to Oral CHM
3637	苏保林,李敬,汤水福,等.糖尿病肾病患者的中医证型及其与实验室指标的相关性研究[J].中国全科医学,2020,23(01):70-74.	Not related to Oral CHM
3638	武曦蔼.糖尿病肾病中医证候分布特点及中药干预糖尿病肾病的临床研究[D].北京中医药大学,2013.	Not related to Oral CHM
3639	张辉, & 姬海霞. (2008). 中西医结合治疗糖尿病足的护理体会. 光明中医, 23(3), 392-393.	Not related to Oral CHM
3640	王涵.仝小林教授运用葛根汤治疗高血压经验及门诊病例回顾性分析[D].中国中医科学院,2017.	Not related to Oral CHM
3641	茹克娅.阿布度热合曼 and 玛依拉.米吉提, 2009, 稳心颗粒联合美托洛尔治疗早搏临床观察, 新疆医学	Not related to Oral CHM
3642	张宏富.稳心颗粒与倍他乐克联用治疗老年心律失常 25 例[J].云南中医中药杂志,2009,30(11):32.DOI:10.16254/j.cnki.53-1120/r.2009.11.045.	Not related to Oral CHM
3643	滕红.稳心颗粒治疗心瓣膜病合并心房颤动的临床观察[J].中西医结合心血管病电子杂志,2020,8(26):37-38.DOI:10.16282/j.cnki.cn11-9336/r.2020.26.025.	Not related to Oral CHM
3644	王晓明.蜈全通治疗血管病三则[J].山东中医杂志,2008,(05):348-349.DOI:10.16295/j.cnki.0257-358x.2008.05.025.	Not related to Oral CHM
3645	周若锋.夏枯菊明茶治疗肝火亢盛型低中危高血压病 35 例观察[J].浙江中医杂志,2014,49(06):424.DOI:10.13633/j.cnki.zjtcm.2014.06.017.	Not related to Oral CHM
3646	伍梅芳,廖昆山.象皮生肌膏治疗糖尿病足 41 例[J].中国中医药现代远程教育,2011,9(02):45-46.	Not related to Oral CHM

3647	赵晓敏.心可舒联合苯扎贝特治疗高脂血症 53 例临床观察[J].中西医结合心脑血管病杂志,2015,13(11):1341-1342.	Not related to Oral CHM
3648	于书香,陶睿.心脑宁胶囊治疗血脂代谢异常的临床疗效及安全性观察[J].北京医学,2015,37(02):197-199.DOI:10.15932/j.0253-9713.2015.2.036.	Not related to Oral CHM
3649	王丹玮.新诊断 2 型糖尿病中医证素与胰岛功能相关性研究[D].北京中医药大学,2019.	Not related to Oral CHM
3650	钟礼强.血府逐瘀汤联合西药治疗难治性高血压随机平行对照研究[J].实用中医内科杂志,2015,29(08):127-129.DOI:10.13729/j.issn.1671-7813.2015.08.61.	Not related to Oral CHM
3651	熊海清,王卫星,刘兵,等.血脂康胶囊治疗痰湿阻遏型高脂血症的临床研究[J].云南中医中药杂志,2012,33(03):14-17+2.DOI:10.16254/j.cnki.53-1120/r.2012.03.006.	Not related to Oral CHM
3652	邢月朋,张伟,于惠卿,等.养心定悸冲剂治疗快速性心律失常的临床研究[J].中国中西医结合急救杂志,2004,(04):218-220.	Not related to Oral CHM
3653	佟香艳, & 商亚英. (2002). 药物湿敷配合特定电磁波谱治疗器治疗 IV 级糖尿病足 1 例的护理. 实用新医学, 4(006), 543-543.	Not related to Oral CHM
3654	梁宏宇,袁丁,许杰红,等.益气化痰法治疗气虚痰浊型单纯收缩期高血压临床研究[J].新中医,2013,45(07):10-12.DOI:10.13457/j.cnki.jncm.2013.07.027.	Not related to Oral CHM
3655	赵超.益气养阴活血方与血脂康联合西药治疗糖尿病肾病随机平行对照研究[J].实用中医内科杂志,2015,29(04):85-87.DOI:10.13729/j.issn.1671-7813.2015.04.37.	Not related to Oral CHM
3656	任素桢,任连军.益气养阴祛瘀汤对湿浊瘀阻型早期糖尿病肾病患者疗效分析[J].青岛医药卫生,2020,52(06):447-449.	Not related to Oral CHM
3657	尚百艳.益气养阴汤治疗糖尿病周围神经病变 74 例[J].中国中医药现代远程教育,2013,11(18):61-62.	Not related to Oral CHM
3658	杨明丽,沈璐,路波,等.益肾汤治疗糖尿病肾病 265 例[J].陕西中医,2005,(08):755-756.	Not related to Oral CHM
3659	张绍芬,潘卓文,覃晓东,等.云南白药外用治疗糖尿病足溃疡 60 例疗效观察[J].内蒙古中医药,2013,32(20):18-19.DOI:10.16040/j.cnki.cn15-1101.2013.20.167.	Not related to Oral CHM
3660	钟旭江.云南白药与酒精佐治糖尿病足[J].浙江中医杂志,2011,46(04):303.	Not related to Oral CHM
3661	袁瑞,章清华.《临证指南医案》应用脏器疗法举隅[J].中国中医药现代远程教育,2022,20(18):125-127.	Not related to Oral CHM
3662	杨晓虹.针药配合治疗高血压病眩晕 35 例体会[J].内蒙古中医药,2013,32(15):62-63.DOI:10.16040/j.cnki.cn15-1101.2013.15.031.	Not related to Oral CHM
3663	孙玉虹.枳曲降脂胶囊临床应用[J].世界最新医学信息文摘,2015,15(80):148+150.	Not related to Oral CHM
3664	施暕.中成药治疗高血压病用药探析[J].湖南中医药大学学报,2013,33(12):25-26.	Not related to Oral CHM
3665	王成山,张招弟.中老年原发性高血压患者体质辨识及中药干预[J].吉林中医药,2013,33(06):583-584.DOI:10.13463/j.cnki.jlzyy.2013.06.037.	Not related to Oral CHM

3666	伍凤元,王焰.中西医结合分期治疗糖尿病足 5 例[J].现代中西医结合杂志,2004,(05):639.	Not related to Oral CHM
3667	张春华.中西医结合治疗高血压伴腔隙性脑梗死病人的临床观察[J].中西医结合心脑血管病杂志,2015,13(06):860-861.	Not related to Oral CHM
3668	王文娟.中西医结合治疗糖尿病足临床观察[J].实用中医药杂志,2019,35(10):1236-1238.	Not related to Oral CHM
3669	孙伟茗.中药辅助治疗H型高血压Meta分析[D].辽宁中医药大学,2021.DOI:10.27213/d.cnki.glnzc.2021.000539.	Not related to Oral CHM
3670	王尧,宋莉丽.中药复方治疗糖尿病大血管病变用药规律系统综述[J].实用中医内科杂志,2015,29(11):1-3.DOI:10.13729/j.issn.1671-7813.2015.11.01.	Not related to Oral CHM
3671	钟定沅.中药联合胰岛素泵强化治疗 2 型糖尿病的meta分析研究[D].广州中医药大学,2016.	Not related to Oral CHM
3672	曲传鑫.中药治疗糖尿病肌少症的系统评价与临床研究[D].山东中医药大学,2022.DOI:10.27282/d.cnki.gsdzu.2022.000411.	Not related to Oral CHM
3673	唐晓丹, & 王约青. (2008). 中医辨证论治, 内外兼治治疗糖尿病足 37 例体会. 中华现代中医学杂志, 4(001), 42-44.	Not related to Oral CHM
3674	吴晓晶,张骞,胡旭珍,等.中医辨证施治糖尿病前期 63 例临床观察[J].中国民族民间医药,2012,21(22):102-103.	Not related to Oral CHM
3675	孙晓泽.中医辨证治疗早期和临床期糖尿病肾病的临床研究[D].广州中医药大学,2011.	Not related to Oral CHM
3676	王诗怡.中医药改善早期糖尿病肾病炎症反应的系统评价和用药规律分析[D].辽宁中医药大学,2022.DOI:10.27213/d.cnki.glnzc.2022.000235.	Not related to Oral CHM
3677	陈亚东.中医药干预痰湿体质糖尿病临床前期观察[J].糖尿病新世界,2015,(20):37-39.DOI:10.16658/j.cnki.1672-4062.2015.20.006.	Not related to Oral CHM
3678	徐庆怀,赵奇煌,谢庆鑫,等.周围神经松解术配合中医辨证治疗糖尿病周围神经病的临床研究[J].临床和实验医学杂志,2014,13(12):1016-1019.	Not related to Oral CHM
3679	姚朝辉,张伏芝,阳业峰.滋肾养肝汤联合缬沙坦治疗痰湿壅盛型高血压随机平行对照研究[J].实用中医内科杂志,2015,29(04):122-124.DOI:10.13729/j.issn.1671-7813.2015.04.54.	Not related to Oral CHM
3680	Mai W, Wei A, Lin X, Wang F, Ye J, Chen P. Efficacy and safety of traditional Chinese medicine injection with mecobalamin in treating diabetic peripheral neuropathy: A protocol for systematic review and meta-analysis. Medicine (Baltimore). 2021;100(1):e23702. doi:10.1097/MD.0000000000023702	Not related to Oral CHM
3681	Mahmoud F, Al-Ozairi E, Haines D, et al. Effect of Diabetea tea ™ consumption on inflammatory cytokines and metabolic biomarkers in type 2 diabetes patients. J Ethnopharmacol. 2016;194:1069-1077. doi:10.1016/j.jep.2016.10.073	Not related to Oral CHM
3682	Mahmoodi MR, Abbasi MM. Therapeutic Effectiveness of Sesame Preparations and its Bioactive Ingredients in Management of Cardiometabolic Syndrome in Diabetes Mellitus: A Systematic Review. Curr Diabetes Rev. 2023;19(3):e250522205240. doi:10.2174/1573399818666220525110925	Not related to Oral CHM

3683	Macêdo APA, Gonçalves MDS, Barreto Medeiros JM, et al. Potential therapeutic effects of green tea on obese lipid profile - a systematic review. Nutr Health. 2022;28(3):401-415. doi:10.1177/02601060211073236	Not related to Oral CHM
3684	Ma Y, Chen J, Huang X, Liu Y. The efficacy and safety of mecobalamin combined with Chinese medicine injections in the treatment of diabetic peripheral neuropathy: A systematic review and Bayesian network meta-analysis of randomized controlled trials. Front Pharmacol. 2022;13:957483. Published 2022 Nov 4. doi:10.3389/fphar.2022.957483	Not related to Oral CHM
3685	Ma RW, Zou DJ, Wang QJ. Zhongguo Zhong Xi Yi Jie He Za Zhi. 2010;30(8):833-837.	Not related to Oral CHM
3686	Ma, Q., Yang, T., Sun, M., Li, Y., Tang, C., Chen, F., ... & Chen, F. (2007). Studies on the relations between angiotensin II and nitric oxide and insulin like growth factot-1 and the effects of tongxinluo. Chinese Pharmacological Bulletin, 23(10), 1291.	Not related to Oral CHM
3687	Ma LX, Liu JP. Zhongguo Zhong Xi Yi Jie He Za Zhi. 2012;32(1):119-123.	Not related to Oral CHM
3688	Shishtar E, Sievenpiper JL, Djedovic V, et al. The effect of ginseng (the genus panax) on glycemic control: a systematic review and meta-analysis of randomized controlled clinical trials. PLoS One. 2014;9(9):e107391. Published 2014 Sep 29. doi:10.1371/journal.pone.0107391	Not related to Oral CHM
3689	Ma JC, Zhao J, Su QH, Zhang DH, Guo Y, Ji ZZ. Zhonghua Yi Xue Za Zhi. 2008;88(8):524-526.	Not related to Oral CHM
3690	Ma J, Xu L, Dong J, et al. Effects of zishentongluo in patients with early-stage diabetic nephropathy. Am J Chin Med. 2013;41(2):333-340. doi:10.1142/S0192415X13500249	Not related to Oral CHM
3691	Lyu J, Gao Y, Wei R, et al. Clinical effectiveness of Qilong capsule in patients with ischemic stroke: A prospective, multicenter, non-randomized controlled trial. Phytomedicine. 2022;104:154278. doi:10.1016/j.phymed.2022.154278	Not related to Oral CHM
3692	Lupton JR, Robinson MC, Morin JL. Cholesterol-lowering effect of barley bran flour and oil. J Am Diet Assoc. 1994;94(1):65-70. doi:10.1016/0002-8223(94)92044-3	Not related to Oral CHM
3693	Luo, Z. J., Guo, T. M., Tu, Q., Cheng, X. L., Huang, Y., & Xiang, M. Q. (2018). Therapeutic effect of integrating Chinese patent medicine Xuesaitong injection and Western medicine in treating patients with hypertensive intracerebral hemorrhage: a prospective randomized controlled trial. European Journal of Integrative Medicine, 23, 26-31.	Not related to Oral CHM
3694	Luo Z, Liu Y, Zhao Z, Yan X, Wang D, Liu Q. Effects of Astragalus injection and Salvia Miltiorrhiza injection on serum inflammatory markers in patients with stable coronary heart disease: a randomized controlled trial protocol. Trials. 2020;21(1):267. Published 2020 Mar 17. doi:10.1186/s13063-020-4109-6	Not related to Oral CHM

369 5	Luo Y, Yang SK, Zhou X, et al. Use of Ophiocordyceps sinensis (syn. Cordyceps sinensis) combined with angiotensin-converting enzyme inhibitors (ACEI)/angiotensin receptor blockers (ARB) versus ACEI/ARB alone in the treatment of diabetic kidney disease: a meta-analysis. Ren Fail. 2015;37(4):614-634. doi:10.3109/0886022X.2015.1009820	Not related to Oral CHM
369 6	Luo D, Qin Y, Yuan W, Deng H, Zhang Y, Jin M. Compound Danshen Dripping Pill for Treating Early Diabetic Retinopathy: A Randomized, Double-Dummy, Double-Blind Study. Evid Based Complement Alternat Med. 2015;2015:539185. doi:10.1155/2015/539185	Not related to Oral CHM
369 7	Ludvik B, Waldhäusl W, Prager R, Kautzky-Willer A, Pacini G. Mode of action of ipomoea batatas (Caiapo) in type 2 diabetic patients. Metabolism. 2003;52(7):875-880. doi:10.1016/s0026-0495(03)00073-8	Not related to Oral CHM
369 8	Ludvik B, Hanefeld M, Pacini G. Improved metabolic control by Ipomoea batatas (Caiapo) is associated with increased adiponectin and decreased fibrinogen levels in type 2 diabetic subjects. Diabetes Obes Metab. 2008;10(7):586-592. doi:10.1111/j.1463-1326.2007.00752.x	Not related to Oral CHM
369 9	Lu ZL, Du BM, Chen Z, et al. Zhonghua Xin Xue Guan Bing Za Zhi. 2005;33(12):1067-1070.	Not related to Oral CHM
370 0	Lu Q, Chen B, Liang Q, et al. Xiaoketongbi Formula vs pregabalin for painful diabetic neuropathy: A single-center, randomized, single-blind, double-dummy, and parallel controlled clinical trial. J Diabetes. 2022;14(8):551-561. doi:10.1111/1753-0407.13306	Not related to Oral CHM
370 1	Lu J, He H. Chin J Integr Med. 2005;11(3):226-228. doi:10.1007/BF02836510	Not related to Oral CHM
370 2	Lu FR, Shen L, Qin Y, Gao L, Li H, Dai Y. Clinical observation on trigonella foenum-graecum L. total saponins in combination with sulfonylureas in the treatment of type 2 diabetes mellitus. Chin J Integr Med. 2008;14(1):56-60. doi:10.1007/s11655-007-9005-3	Not related to Oral CHM
370 3	Kim J, Moon E, Kim TH. Successful Midterm Management With an Herbal Decoction, Modified-Goshajinkigan (mGJG) for Non-Proliferative Diabetic Retinopathy: A Case Study. Explore (NY). 2018;14(4):295-299. doi:10.1016/j.explore.2017.07.009	Not related to Oral CHM
370 4	Lopresti, A. L., & Smith, S. J. (2021). Ashwagandha (Withania somnifera) for the treatment and enhancement of mental and physical conditions: A systematic review of human trials. Journal of Herbal Medicine, 28, 100434.	Not related to Oral CHM
370 5	Xuan, L. O. N. G., Feng, W. A. N. G., & Changquan, H. U. A. N. G. Tongxinluo Capsule for Diabetic Kidney Disease: A Systematic Review. Chinese Journal of Evidence-Based Medicine, 10(1), 73-80.	Not related to Oral CHM
370 6	Liu ZQ, Li QZ, Qin GJ. Zhongguo Zhong Xi Yi Jie He Za Zhi. 2001;21(4):274-276.	Not related to Oral CHM

3707	Liu ZM, Ho SC, Chen YM, Ho YP. The effects of isoflavones combined with soy protein on lipid profiles, C-reactive protein and cardiovascular risk among postmenopausal Chinese women. Nutr Metab Cardiovasc Dis. 2012;22(9):712-719. doi:10.1016/j.numecd.2010.11.002	Not related to Oral CHM
3708	Liu ZL, Li Q, Mu YJ, Gao Y, Liu JP. Zhong Xi Yi Jie He Xue Bao. 2012;10(9):991-996. doi:10.3736/jcim20120907	Not related to Oral CHM
3709	Liu Z, Chen Y, Jie C, et al. The comparative effects of oral Chinese patent medicines in non-proliferative diabetic retinopathy: A Bayesian network meta-analysis of randomized controlled trials. Front Endocrinol (Lausanne). 2023;14:1144290. Published 2023 Apr 3. doi:10.3389/fendo.2023.1144290	Not related to Oral CHM
3710	Liu YH, Yang L, Liu J. Zhongguo Zhong Xi Yi Jie He Za Zhi. 2005;25(11):993-995.	Not related to Oral CHM
3711	Zhang J, Xie X, Li C, Fu P. Systematic review of the renal protective effect of Astragalus membranaceus (root) on diabetic nephropathy in animal models. J Ethnopharmacol. 2009;126(2):189-196. doi:10.1016/j.jep.2009.08.046	Not related to Oral CHM
3712	Liu Y, Wang XM, Yang JX. Zhongguo Zhong Xi Yi Jie He Za Zhi. 2009;29(8):698-702.	Not related to Oral CHM
3713	Liu Y, Guo D, Tian Y, Fan M, Zhao J. Efficacy and safety of Yangxue Qingnao granules for the treatment of essential hypertension: A protocol for systematic review and meta-analysis. Medicine (Baltimore). 2021;100(48):e27911. doi:10.1097/MD.0000000000027911	Not related to Oral CHM
3714	Liu, Y., & Fan, Y. (2018). A3464 The clinical effect and contrast evaluation of office blood pressure, ambulatory blood pressure mornitoring on treatment of Yinxu-Yangkang type of primary hypertensives by TCM of Tianma Gouteng Yin. Journal of Hypertension, 36, e147.	Not related to Oral CHM
3715	Liu XH, Li XM, Han CC, Fang XF, Ma L. Effects of combined therapy with glipizide and Aralia root bark extract on glycemic control and lipid profiles in patients with type 2 diabetes mellitus. J Sci Food Agric. 2015;95(4):739-744. doi:10.1002/jsfa.6829	Not related to Oral CHM
3716	Liu X, Xu X, Zhang T, et al. Fatty acid metabolism disorders and potential therapeutic traditional Chinese medicines in cardiovascular diseases. Phytother Res. 2023;37(11):4976-4998. doi:10.1002/ptr.7965	Not related to Oral CHM
3717	Liu W, Jiang R, Ding S, et al. Zhongguo Zhong Yao Za Zhi. 2012;37(1):109-114.	Not related to Oral CHM
3718	Liu SX, Sun M, Luo YF. Zhongguo Zhong Xi Yi Jie He Za Zhi. 2004;24(6):553-555.	Not related to Oral CHM
3719	Liu ML, Fan GH, Zhang HL. Zhongguo Zhong Yao Za Zhi. 2021;46(6):1511-1522. doi:10.19540/j.cnki.cjcmm.20200702.501	Not related to Oral CHM
3720	Liu M, Yanneng X, Yang G, Li Z, Luo G, Yang S. Danshen Decoction in the Treatment of Hyperlipidemia: A Systematic Review and Meta-Analysis Protocol of Randomized Controlled Trials. Evid Based Complement Alternat Med. 2022;2022:2392652. Published 2022 Nov 8. doi:10.1155/2022/2392652	Not related to Oral CHM

3721	Liu LY, Zhou L, Liu XZ, Zou DJ. Effect of Hedan Tablets on Body Weight and Insulin Resistance in Patients with Metabolic Syndrome. Obes Facts. 2022;15(2):180-185. doi:10.1159/000520711	Not related to Oral CHM
3722	Liu LT, Wu M, Wang HX. Zhongguo Zhong Xi Yi Jie He Za Zhi. 2011;31(9):1196-1200.	Not related to Oral CHM
3723	Liu KZ, Li JB, Lu HL, Wen JK, Han M. Zhongguo Zhong Yao Za Zhi. 2004;29(3):264-266.	Not related to Oral CHM
3724	Liu JY, Chen XX, Tang SC, et al. Chinese medicines in the treatment of experimental diabetic nephropathy. Chin Med. 2016;11:6. Published 2016 Feb 24. doi:10.1186/s13020-016-0075-z	Not related to Oral CHM
3725	Liu J, Sun B, Ban B, et al. Zhongguo Zhong Xi Yi Jie He Za Zhi. 2014;34(9):1053-1058.	Not related to Oral CHM
3726	Liu J, Gao LD, Fu B, et al. Efficacy and safety of Zicuiyin decoction on diabetic kidney disease: A multicenter, randomized controlled trial. Phytomedicine. 2022;100:154079. doi:10.1016/j.phymed.2022.154079	Not related to Oral CHM
3727	Liu J, Dong B, Yang L, Huang W, Tang S. Xuefu Zhuyu decoction for nonalcoholic fatty liver disease: A protocol for systematic review and meta-analysis. Medicine (Baltimore). 2021;100(19):e25358. doi:10.1097/MD.0000000000025358	Not related to Oral CHM
3728	Li, X., Feng, J. L., Chen, Z. L., Bao, B. H., Dai, H. H., Meng, F. C., ... & Wang, J. S. (2022). Mechanism by which Huoxue Tongluo Qiwei Decoction improves the erectile function of rats with diabetic erectile dysfunction. Journal of ethnopharmacology, 283, 114674.	Not related to Oral CHM
3729	Liu, G. X., Kuang, Q. G., YU, X. H., Liu, F., Wang, Y., YU, Y., & Chen, Y. (2021). Therapeutic Effect and Mechanism of Modified Da Chengqitang in Treating Hyperlipidemic Acute Pancreatitis with Damp Heat Accumulation Syndrome.聽Chinese Journal of Experimental Traditional Medical Formulae, 91-97.	Not related to Oral CHM
3730	Liu D, Zhong Y, Yan H, Hu Y, Chen Y, Zhou Y. The safety and efficacy of astragalus for treating diabetic foot ulcers: A protocol for systematic review and meta-analysis. Medicine (Baltimore). 2021;100(1):e24082. doi:10.1097/MD.0000000000024082	Not related to Oral CHM
3731	Liu D, Tang JY, Yan L. Zhongguo Zhong Xi Yi Jie He Za Zhi. 2010;30(12):1265-1268.	Not related to Oral CHM
3732	Liu, C, 2013, Factors associate with utilization of herbal medicine: Evidence from 5 years inpatient records in tcm hospitals in china systematic review: Comparative effectiveness between Xiaoke Pill and glibenclamide for type 2 diabetes mellitus, Journal of Alternative and Complementary Medicine	Not related to Oral CHM
3733	Lin YK, Chung YM, Yang HT, et al. The potential of immature poken (Citrus reticulata) extract in the weight management, lipid and glucose metabolism. J Complement Integr Med. 2021;19(2):279-285. Published 2021 May 10. doi:10.1515/jcim-2020-0478	Not related to Oral CHM

3734	Lin YJ, Ho TJ, Yeh YC, et al. Chinese Herbal Medicine Treatment Improves the Overall Survival Rate of Individuals with Hypertension among Type 2 Diabetes Patients and Modulates In Vitro Smooth Muscle Cell Contractility. PLoS One. 2015;10(12):e0145109. Published 2015 Dec 23. doi:10.1371/journal.pone.0145109	Not related to Oral CHM
3735	Lin JH, Yang YK, Liu H, Lin QD, Zhang WY; Cooperation Group on Special Project "Study on the prevention and treatment for hypertension disorders and hematopexis related complications in pregnancy". Effect of antioxidants on amelioration of high-risk factors inducing hypertensive disorders in pregnancy. Chin Med J (Engl). 2010;123(18):2548-2554.	Not related to Oral CHM
3736	Lin J, Wang Q, Zhong D, et al. Efficacy and safety of Qiangli Dingxuan tablet combined with amlodipine besylate for essential hypertension: a randomized, double-blind, placebo-controlled, parallel-group, multicenter trial. Front Pharmacol. 2023;14:1225529. Published 2023 Jul 10. doi:10.3389/fphar.2023.1225529	Not related to Oral CHM
3737	Lim Y, Lee KW, Kim JY, Kwon O. A beverage of Asiatic plantain extracts alleviated postprandial oxidative stress in overweight hyperlipidemic subjects challenged with a high-fat meal: a preliminary study. Nutr Res. 2013;33(9):704-710. doi:10.1016/j.nutres.2013.07.003	Not related to Oral CHM
3738	Lien AS, Jiang YD, Mou CH, Sun MF, Gau BS, Yen HR. Integrative traditional Chinese medicine therapy reduces the risk of diabetic ketoacidosis in patients with type 1 diabetes mellitus. J Ethnopharmacol. 2016;191:324-330. doi:10.1016/j.jep.2016.06.051	Not related to Oral CHM
3739	Liao YN, Chen HY, Yang CW, et al. Corrigendum: Chinese herbal medicine is associated with higher body weight reduction than liraglutide among the obese population: a real-world comparative cohort study. Front Pharmacol. 2023;14:1222106. Published 2023 May 23. doi:10.3389/fphar.2023.1222106	Not related to Oral CHM
3740	Liao T, Zhao K, Huang Q, et al. A randomized controlled clinical trial study protocol of Liuwei Dihuang pills in the adjuvant treatment of diabetic kidney disease. Medicine (Baltimore). 2020;99(31):e21137. doi:10.1097/MD.0000000000021137	Not related to Oral CHM
3741	Liao J, Tian J, Li T, Song W, Zhao W, Du J. Xuefuzhuyu decoction for hyperlipidemia: a systematic review and meta-analysis of randomized clinical trails. J Tradit Chin Med. 2014;34(4):411-418. doi:10.1016/s0254-6272(15)30040-6	Not related to Oral CHM
3742	Liao H, Hu L, Cheng X, et al. Are the Therapeutic Effects of Huangqi (Astragalus membranaceus) on Diabetic Nephropathy Correlated with Its Regulation of Macrophage iNOS Activity?. J Immunol Res. 2017;2017:3780572. doi:10.1155/2017/3780572	Not related to Oral CHM
3743	Liang B. Zhong Yao Cai. 2005;28(7):634-636.	Not related to Oral CHM

3744	Lian F, Wu L, Tian J, et al. The effectiveness and safety of a danshen-containing Chinese herbal medicine for diabetic retinopathy: a randomized, double-blind, placebo-controlled multicenter clinical trial. J Ethnopharmacol. 2015;164:71-77. doi:10.1016/j.jep.2015.01.048	Not related to Oral CHM
3745	Li Y, Liu Y, Cui J, Zhao H, Liu Y, Huang L. Cohort Studies on Chronic Non-communicable Diseases Treated With Traditional Chinese Medicine: A Bibliometric Analysis. Front Pharmacol. 2021;12:639860. Published 2021 Mar 19. doi:10.3389/fphar.2021.639860	Not related to Oral CHM
3746	Li Y, Li Z, Wang R, et al. Alleviating the Hydrolysis of Carbohydrates, Tangzhiqing (TZQ) Decreased the Postprandial Glycemia in Healthy Volunteers: An Eight-Period Crossover Study. Evid Based Complement Alternat Med. 2020;2020:8138195. Published 2020 Mar 16. doi:10.1155/2020/8138195	Not related to Oral CHM
3747	Li XS, Fu XJ, Lang XJ. Zhongguo Zhong Xi Yi Jie He Za Zhi. 2007;27(5):412-414.	Not related to Oral CHM
3748	Li X, Li B, Wang TY, Wang H, Li Q. Zhongguo Zhong Yao Za Zhi. 2022;47(11):3088-3094. doi:10.19540/j.cnki.cjcmm.20220303.501	Not related to Oral CHM
3749	Li QL, Zhang YF. Zhongguo Zhong Xi Yi Jie He Za Zhi. 2003;23(5):335-337.	Not related to Oral CHM
3750	Li Q, Zhang HM, Fei YT. Zhongguo Zhong Xi Yi Jie He Za Zhi. 2012;32(3):317-321.	Not related to Oral CHM
3751	Li NY, Yu H, Li XL, et al. Astragalus Membranaceus Improving Asymptomatic Left Ventricular Diastolic Dysfunction in Postmenopausal Hypertensive Women with Metabolic Syndrome: A Prospective, Open-Labeled, Randomized Controlled Trial. Chin Med J (Engl). 2018;131(5):516-526. doi:10.4103/0366-6999.226077	Not related to Oral CHM
3752	Li NY, Li XL, Zhai XP, et al. Zhongguo Zhong Yao Za Zhi. 2016;41(21):4051-4059. doi:10.4268/cjcmm20162126	Not related to Oral CHM
3753	Li M, Wang W, Xue J, Gu Y, Lin S. Meta-analysis of the clinical value of Astragalus membranaceus in diabetic nephropathy. J Ethnopharmacol. 2011;133(2):412-419. doi:10.1016/j.jep.2010.10.012	Not related to Oral CHM
3754	Li L, Sun T, Tian J, Yang K, Yi K, Zhang P. Garlic in clinical practice: an evidence-based overview. Crit Rev Food Sci Nutr. 2013;53(7):670-681. doi:10.1080/10408398.2010.537000	Not related to Oral CHM
3755	Li JP, He XL, Li Q. Zhongguo Zhong Xi Yi Jie He Za Zhi. 2006;26(5):415-418.	Not related to Oral CHM
3756	Li JJ, Lu ZL, Kou WR, et al. Long-term effects of Xuezhikang on blood pressure in hypertensive patients with previous myocardial infarction: data from the Chinese Coronary Secondary Prevention Study (CCSPS). Clin Exp Hypertens. 2010;32(8):491-498. doi:10.3109/10641961003686427	Not related to Oral CHM

3757	Li JJ, Lu ZL, Kou WR, et al. Beneficial impact of Xuezhikang on cardiovascular events and mortality in elderly hypertensive patients with previous myocardial infarction from the China Coronary Secondary Prevention Study (CCSPS). J Clin Pharmacol. 2009;49(8):947-956. doi:10.1177/0091270009337509	Not related to Oral CHM
3758	Li H, Zhao WM, Han YX. Zhongguo Zhong Xi Yi Jie He Za Zhi. 2009;29(2):115-9.	Not related to Oral CHM
3759	Li H, Liu LT, Zhao WM, Liu JG, Yao MJ, Han YX, et al. Chin J Integr Med. 2010 Jun;16(3):216-21. doi: 10.1007/s11655-010-0216-y.	Not related to Oral CHM
3760	Li H, Liu LT, Zhao WM, Liu JG, Yao MJ, Han YX, et al. Zhong Xi Yi Jie He Xue Bao. 2010 May;8(5):410-6. doi: 10.3736/jcim20100503.	Not related to Oral CHM
3761	Li H, Liu F, Cui L, Luo ZG, Liu FZ, Xu LR. Zhongguo Zhong Xi Yi Jie He Za Zhi. 2003;23(12):898-901.	Not related to Oral CHM
3762	Li G, Ai B, Zhang W, Feng X, Jiang M. Medicine (Baltimore). 2021 Mar 26;100(12):e25096. doi: 10.1097/MD.0000000000025096.	Not related to Oral CHM
3763	Leverrier A, Daguet D, Calame W, Dhoye P, Kodimule SP. Nutrients. 2019;11(5):1080. doi: 10.3390/nu11051080.	Not related to Oral CHM
3764	Lenon GB, Li KX, Chang YH, Yang AW, Da Costa C, Li CG, et al. Evid Based Complement Alternat Med. 2012;2012:435702. doi: 10.1155/2012/435702.	Not related to Oral CHM
3765	Leng Y, Gao H, Fu X, Liu Y, Xie H, Hu Z, et al. Medicine (Baltimore). 2020 Jan;99(3):e18713. doi: 10.1097/MD.0000000000018713.	Not related to Oral CHM
3766	Lei Y, Lu QS, Ma XC, Chen KJ. Zhongguo Zhong Xi Yi Jie He Za Zhi. 2005 Feb;25(2):114-8.	Not related to Oral CHM
3767	Lee YL, Lee SY. Front Med (Lausanne). 2022 Nov 1;9:1000428. doi: 10.3389/fmed.2022.1000428.	Not related to Oral CHM
3768	Lee S, Lee H, Cho Y, Kim J, Kang JW, Seo BK, et al. Medicine (Baltimore). 2018 Sep;97(38):e12440. doi: 10.1097/MD.0000000000012440.	Not related to Oral CHM
3769	Lee JY, Liao WL, Liu YH, Kuo CL, Lung FW, Hsieh CL. J Ethnopharmacol. 2022 Jun 28;292:115111. doi: 10.1016/j.jep.2022.115111.	Not related to Oral CHM
3770	Lee CY, Jan MS, Yu MC, Lin CC, Wei JC, Shih HC. Forsch Komplementmed. 2013;20(3):197-203. doi: 10.1159/000351455.	Not related to Oral CHM
3771	Lazavi F, Mirmiran P, Sohrab G, Nikpayam O, Angoorani P, Hedayati M. Complement Ther Clin Pract. 2018 May;31:170-174. doi: 10.1016/j.ctcp.2018.01.009.	Not related to Oral CHM
3772	Lasaite L, Spadiene A, Savickiene N, Skesters A, Silova A. Nat Prod Commun. 2014 Sep;9(9):1345-50.	Not related to Oral CHM
3773	Lan J, Zhao Y, Dong F, Yan Z, Zheng W, Fan J, et al. J Ethnopharmacol. 2015 Feb 23;161:69-81. doi: 10.1016/j.jep.2014.09.049.	Not related to Oral CHM
3774	Lakshmi T, Roy A, Durgha K, Manjusha V. Int J Drug Dev Res. 2011;3(3):31-57.	Not related to Oral CHM
3775	Lai X, Dong Z, Wu S, Zhou X, Zhang G, Xiong S, et al. Circ Cardiovasc Qual Outcomes. 2022 Mar;15(3):e007923. doi: 10.1161/CIRCOUTCOMES.121.007923.	Not related to Oral CHM

3776	Woo KS, Yip TW, Chook P, Kwong SK, Szeto CC, Li JK, et al. Complement Ther Med. 2014 Jun;22(3):473-80. doi: 10.1016/j.ctim.2014.03.010.	Not related to Oral CHM
3777	Kuriyan R, Rajendran R, Bantwal G, Kurpad AV. Diabetes Care. 2008 Feb;31(2):216-220. doi: 10.2337/dc07-1591.	Not related to Oral CHM
3778	Kuo YS, Chien HF, Lu W. Evid Based Complement Alternat Med. 2012;2012:418679. doi: 10.1155/2012/418679.	Not related to Oral CHM
3779	Kumar S, Sharma SK, Mudgal SK, Gaur R, Agarwal R, Singh H, et al. Diabetes Metab Syndr. 2023 Aug;17(8):102826. doi: 10.1016/j.dsx.2023.102826.	Not related to Oral CHM
3780	Kumar S, Bharali A, Sarma H, Kushari S, Gam S, Hazarika I, et al. J Ayurveda Integr Med. 2023 Jul-Aug;14(4):100745. doi: 10.1016/j.jaim.2023.100745.	Not related to Oral CHM
3781	Krawczyk M, Burzynska-Pedziwiatr I, Wozniak LA, Bukowiecka-Matusiak M. Curr Issues Mol Biol. 2022;44(2):699-717. doi: 10.3390/cimb44020049.	Not related to Oral CHM
3782	Krasinska B, Osinska A, Osinski M, Krasinska A, Rzymski P, Tykarski A, et al. Arch Med Sci. 2018 Jun;14(4):773-780. doi: 10.5114/aoms.2017.69864.	Not related to Oral CHM
3783	Krasinska B, Osinska A, Krasinska A, Osinski M, Rzymski P, Tykarski A, Krasiński Z. Kardiol Pol. 2018;76(2):388-395. doi: 10.5603/KP.a2017.0215.	Not related to Oral CHM
3784	Kou QA, Yu HP, Zhou GD. Zhongguo Zhong Xi Yi Jie He Za Zhi. 2007 Aug;27(8):745-748.	Not related to Oral CHM
3785	Kobayashi M, Akaki J, Ninomiya K, et al. Dose-Dependent Suppression of Postprandial Hyperglycemia and Improvement of Blood Glucose Parameters by Salacia chinensis Extract: Two Randomized, Double-Blind, Placebo-Controlled Studies [published correction appears in J Med Food. 2021 Mar;24(3):331. doi: 10.1089/jmf.2020.4751.correx.]. J Med Food. 2021;24(1):10-17. doi:10.1089/jmf.2020.4751	Not related to Oral CHM
3786	Ko CH, Yi S, Ozaki R, et al. Healing effect of a two-herb recipe (NF3) on foot ulcers in Chinese patients with diabetes: a randomized double-blind placebo-controlled study. J Diabetes. 2014;6(4):323-334. doi:10.1111/1753-0407.12117	Not related to Oral CHM
3787	Klupp NL, Kiat H, Bensoussan A, Steiner GZ, Chang DH. A double-blind, randomised, placebo-controlled trial of Ganoderma lucidum for the treatment of cardiovascular risk factors of metabolic syndrome. Sci Rep. 2016;6:29540. Published 2016 Aug 11. doi:10.1038/srep29540	Not related to Oral CHM
3788	Klupp NL, Chang D, Hawke F, et al. Ganoderma lucidum mushroom for the treatment of cardiovascular risk factors. Cochrane Database Syst Rev. 2015;2015(2):CD007259. Published 2015 Feb 17. doi:10.1002/14651858.CD007259.pub2	Not related to Oral CHM
3789	Klafke JZ, da Silva MA, Panigas TF, et al. Effects of Campomanesia xanthocarpa on biochemical, hematological and oxidative stress parameters in hypercholesterolemic patients. J Ethnopharmacol. 2010;127(2):299-305. doi:10.1016/j.jep.2009.11.004	Not related to Oral CHM

3790	Kitalong C, Nogueira RC, Benichou J, et al. "DAK", a traditional decoction in Palau, as adjuvant for patients with insufficient control of diabetes mellitus type II. J Ethnopharmacol. 2017;205:116-122. doi:10.1016/j.jep.2017.05.003	Not related to Oral CHM
3791	Kirkham S, Akilen R, Sharma S, Tsiami A. The potential of cinnamon to reduce blood glucose levels in patients with type 2 diabetes and insulin resistance. Diabetes Obes Metab. 2009;11(12):1100-1113. doi:10.1111/j.1463-1326.2009.01094.x	Not related to Oral CHM
3792	Kimura T, Nakagawa K, Kubota H, et al. Food-grade mulberry powder enriched with 1-deoxynojirimycin suppresses the elevation of postprandial blood glucose in humans. J Agric Food Chem. 2007;55(14):5869-5874. doi:10.1021/jf062680g	Not related to Oral CHM
3793	Kim SY, Oh MR, Kim MG, Chae HJ, Chae SW. Anti-obesity effects of Yerba Mate (Ilex Paraguariensis): a randomized, double-blind, placebo-controlled clinical trial. BMC Complement Altern Med. 2015;15:338. Published 2015 Sep 25. doi:10.1186/s12906-015-0859-1	Not related to Oral CHM
3794	Kim SK, Jung J, Jung JH, et al. Antidiabetic Effects of Vigna nakashimae Extract in Humans: A Preliminary Study. J Altern Complement Med. 2018;24(3):249-253. doi:10.1089/acm.2017.0114	Not related to Oral CHM
3795	Kim S, Shin BC, Lee MS, Lee H, Ernst E. Red ginseng for type 2 diabetes mellitus: a systematic review of randomized controlled trials. Chin J Integr Med. 2011;17(12):937-944. doi:10.1007/s11655-011-0937-2	Not related to Oral CHM
3796	Kim R, Zhou WQ. Zhongguo Zhong Xi Yi Jie He Za Zhi. 2004;24(7):610-612.	Not related to Oral CHM
3797	Kim JT, Ren CJ, Fielding GA, et al. Treatment with lavender aromatherapy in the post-anesthesia care unit reduces opioid requirements of morbidly obese patients undergoing laparoscopic adjustable gastric banding. Obes Surg. 2007;17(7):920-925. doi:10.1007/s11695-007-9170-7	Not related to Oral CHM
3798	Kim HJ, Yoon KH, Kang MJ, et al. A six-month supplementation of mulberry, korean red ginseng, and banaba decreases biomarkers of systemic low-grade inflammation in subjects with impaired glucose tolerance and type 2 diabetes. Evid Based Complement Alternat Med. 2012;2012:735191. doi:10.1155/2012/735191	Not related to Oral CHM
3799	Kim HJ, Park JM, Kim JA, Ko BP. Effect of herbal Ephedra sinica and Evodia rutaecarpa on body composition and resting metabolic rate: a randomized, double-blind clinical trial in Korean premenopausal women. J Acupunct Meridian Stud. 2008;1(2):128-138. doi:10.1016/S2005-2901(09)60033-9	Not related to Oral CHM
3800	Kim H , Simbo SY , Fang C , et al. Açaí (Euterpe oleracea Mart.) beverage consumption improves biomarkers for inflammation but not glucose- or lipid-metabolism in individuals with metabolic syndrome in a randomized, double-blinded, placebo-controlled clinical trial. Food Funct. 2018;9(6):3097-3103. doi:10.1039/c8fo00595h	Not related to Oral CHM

3801	Kim B, Lee IS, Ko SJ. The efficacy and safety of Laminaria japonica for metabolic syndrome: A protocol for systematic review. Medicine (Baltimore). 2022;101(7):e28892. doi:10.1097/MD.0000000000028892	Not related to Oral CHM
3802	Kianbakht S, Hashem-Dabaghian F. Antihypertensive efficacy and safety of Vaccinium arctostaphylos berry extract in overweight/obese hypertensive patients: A randomized, double-blind and placebo-controlled clinical trial. Complement Ther Med. 2019;44:296-300. doi:10.1016/j.ctim.2019.05.010	Not related to Oral CHM
3803	Kianbakht S, Abasi B, Hashem Dabaghian F. Improved lipid profile in hyperlipidemic patients taking Vaccinium arctostaphylos fruit hydroalcoholic extract: a randomized double-blind placebo-controlled clinical trial. Phytother Res. 2014;28(3):432-436. doi:10.1002/ptr.5011	Not related to Oral CHM
3804	Kianbakht S, Abasi B, Dabaghian FH. Anti-hyperglycemic effect of Vaccinium arctostaphylos in type 2 diabetic patients: a randomized controlled trial. Forsch Komplementmed. 2013;20(1):17-22. doi:10.1159/000346607	Not related to Oral CHM
3805	Khouchlaa A, El Menyiy N, Guaouguaou FE, et al. Ethnomedicinal use, phytochemistry, pharmacology, and toxicology of Daphne gnidium: A review. J Ethnopharmacol. 2021;275:114124. doi:10.1016/j.jep.2021.114124	Not related to Oral CHM
3806	Khouchlaa A, El Menyiy N, Guaouguaou FE, et al. Ethnomedicinal use, phytochemistry, pharmacology, and toxicology of Daphne gnidium: A review. J Ethnopharmacol. 2021;275:114124. doi:10.1016/j.jep.2021.114124	Not related to Oral CHM
3807	Khazaei F, Yadegari M, Ghanbari E, Heydari M, Khazaei M. Therapeutic applications and characteristics of Falcaria vulgaris in traditional medicine and experimental studies. Avicenna J Phytomed. 2022;12(2):116-130. doi:10.22038/AJP.2021.18488	Not related to Oral CHM
3808	Khan, Q. A., Khan, A. A., Jabeen, A., Jahangir, U., & Parveen, S. (2018). Efficacy of Sankhaholi (Evolvulus alsinoides Linn.) in the management of essential hypertension: A randomized standard control clinical study. International Journal of Pharmaceutical Sciences and Research, 10(7), 3467-3473.	Not related to Oral CHM
3809	Khan, NA, 2023, DRUGS AND SUBSTANCES THAT INCREASE BLOOD PRESSURE, Journal of Hypertension	Not related to Oral CHM
3810	Kessler, CHS and Doering, T, 2008, Design, effect size and power of ayurvedic studies on type 2 diabetes, Schweizerische Zeitschrift fur GanzheitsMedizin	Not related to Oral CHM
3811	Kegele, C. S., Oliveira, J., Magrani, T., Ferreira, A., de Souza Ferreira, R., Sabbaghi, A., ... & Polonini, H. C. (2019). A randomized trial on the effects of CitrusiM庐(Citrus sinensis (L.) Osbeck dried extract) on body composition.聽Clinical Nutrition Experimental,聽 27, 29-36.	Not related to Oral CHM
3812	Kazemipoor M, Radzi CW, Hajifaraji M, Haerian BS, Mosaddegh MH, Cordell GA. Antiobesity effect of caraway extract on overweight and obese women: a randomized, triple-blind, placebo-controlled clinical trial. Evid	Not related to Oral CHM

	Based Complement Alternat Med. 2013;2013:928582. doi:10.1155/2013/928582	
3813	Kazemipoor M, Radzi CW, Hajifaraji M, Cordell GA. Preliminary safety evaluation and biochemical efficacy of a Carum carvi extract: results from a randomized, triple-blind, and placebo-controlled clinical trial. Phytother Res. 2014;28(10):1456-1460. doi:10.1002/ptr.5147	Not related to Oral CHM
3814	Kazemipoor M, Hamzah S, Hajifaraji M, Radzi CW, Cordell GA. Slimming and Appetite-Suppressing Effects of Caraway Aqueous Extract as a Natural Therapy in Physically Active Women. Phytother Res. 2016;30(6):981-987. doi:10.1002/ptr.5603	Not related to Oral CHM
3815	Kazemi T, Mollaei H, Takhviji V, et al. The anti-dyslipidemia property of saffron petal hydroalcoholicextract in cardiovascular patients: A double-blinded randomized clinical trial. Clin Nutr ESPEN. 2023;55:314-319. doi:10.1016/j.clnesp.2023.04.002	Not related to Oral CHM
3816	Kawashima N. Kambakutaisoto Treatment for Children With Night Crying and Arousal Parasomnias Developed During Prolonged Hospitalization for Hematological and Oncological Diseases. J Child Neurol. 2021;36(7):568-574. doi:10.1177/0883073820984062	Not related to Oral CHM
3817	Kaushik S, Masand N, Iyer MR, Patil VM. Preclinical to Clinical Profile of Curcuma longa as Antidiabetic Therapeutics. Curr Top Med Chem. 2023;23(24):2267-2276. doi:10.2174/1568026623666230428101440	Not related to Oral CHM
3818	Katare C, Saxena S, Agrawal S, et al. Lipid-lowering and antioxidant functions of bottle gourd (Lagenaria siceraria) extract in human dyslipidemia. J Evid Based Complementary Altern Med. 2014;19(2):112-118. doi:10.1177/2156587214524229	Not related to Oral CHM
3819	Iloon Kashkooli R, Najafi SS, Sharif F, et al. The effect of berberis vulgaris extract on transaminase activities in non-alcoholic Fatty liver disease. Hepat Mon. 2015;15(2):e25067. Published 2015 Feb 5. doi:10.5812/hepatmon.25067	Not related to Oral CHM
3820	Karimi Z, Firouzi M, Dadmehr M, Javad-Mousavi SA, Bagheriani N, Sadeghpour O. Almond as a nutraceutical and therapeutic agent in Persian medicine and modern phytotherapy: A narrative review. Phytother Res. 2021;35(6):2997-3012. doi:10.1002/ptr.7006	Not related to Oral CHM
3821	Kardoust M, Salehi H, Taghipour Z, Sayadi A. The Effect of Kiwifruit Therapeutics in the Treatment of Diabetic Foot Ulcer. Int J Low Extrem Wounds. 2021;20(2):104-110. doi:10.1177/1534734619851700	Not related to Oral CHM
3822	Kamali SH, Khalaj AR, Hasani-Ranjbar S, et al. Efficacy of 'Itrifal Saghir', a combination of three medicinal plants in the treatment of obesity; A randomized controlled trial. Daru. 2012;20(1):33. Published 2012 Sep 10. doi:10.1186/2008-2231-20-33	Not related to Oral CHM

3823	Jurgens TM, Whelan AM, Killian L, Doucette S, Kirk S, Foy E. Green tea for weight loss and weight maintenance in overweight or obese adults. Cochrane Database Syst Rev. 2012;12(12):CD008650. Published 2012 Dec 12. doi:10.1002/14651858.CD008650.pub2	Not related to Oral CHM
3824	Jubayer, F., Kayshar, S., & Rahaman, M. (2020). Effects of Ajwa date seed powder on serum lipids in humans: A randomized, double-blind, placebo-controlled clinical trial. Journal of Herbal Medicine, 24, 100409.	Not related to Oral CHM
3825	Joyal SV. A perspective on the current strategies for the treatment of obesity. Curr Drug Targets CNS Neurol Disord. 2004;3(5):341-356. doi:10.2174/1568007043336978	Not related to Oral CHM
3826	Jovanovski E, Smircic-Duvnjak L, Komishon A, et al. Effect of coadministration of enriched Korean Red Ginseng (Panax ginseng) and American ginseng (Panax quinquefolius L) on cardiometabolic outcomes in type-2 diabetes: A randomized controlled trial. J Ginseng Res. 2021;45(5):546-554. doi:10.1016/j.jgr.2019.11.005	Not related to Oral CHM
3827	Joh B, Jeon ES, Lim SH, Park YL, Park W, Chae H. Intercultural Usage of Mori Folium: Comparison Review from a Korean Medical Perspective. Evid Based Complement Alternat Med. 2015;2015:379268. doi:10.1155/2015/379268	Not related to Oral CHM
3828	Jo HG, Lee D. Oral Administration of East Asian Herbal Medicine for Peripheral Neuropathy: A Systematic Review and Meta-Analysis with Association Rule Analysis to Identify Core Herb Combinations. Pharmaceuticals (Basel). 2021;14(11):1202. Published 2021 Nov 22. doi:10.3390/ph14111202	Not related to Oral CHM
3829	Jin Z, Zhang BF, Shang LX, et al. Zhongguo Zhen Jiu. 2011;31(7):613-616.	Not related to Oral CHM
3830	Jin, S. Y., Chen, Q. G., Yao, Z., & LU, H. (2021). Clinical Observation of Shenxie Zhitong Capsule in Treating Diabetic Peripheral Neuropathy of Stagnant Blockade of Collaterals. Chinese Journal of Experimental Traditional Medical Formulae, 81-87.	Not related to Oral CHM
3831	Jin D, Zhang Y, Zhang Y, et al. Efficacy and Safety of TangWang Prescription for Type 2 Non-Proliferative Diabetic Retinopathy: A Study Protocol for a Randomized Controlled Trial. Front Pharmacol. 2021;12:594308. Published 2021 Mar 15. doi:10.3389/fphar.2021.594308	Not related to Oral CHM
3832	Jin D, Huang WJ, Meng X, et al. Chinese herbal medicine TangBi Formula treatment of patients with type 2 diabetic distal symmetric polyneuropathy disease: study protocol for a randomized controlled trial. Trials. 2017;18(1):631. Published 2017 Dec 29. doi:10.1186/s13063-017-2345-1	Not related to Oral CHM
3833	Jin, D., Hou, L., Han, S., Chang, L., Gao, H., Zhao, Y., ... & Jia, Z. (2020). Basis and design of a randomized clinical trial to evaluate the effect of Jinlida granules on metabolic syndrome in patients with abnormal glucose metabolism. Frontiers in Endocrinology, 11, 415.	Not related to Oral CHM

3834	Jiang ZT, Liang QL, Wang YM. Zhongguo Zhong Xi Yi Jie He Za Zhi. 2011;31(8):1057-1061.	Not related to Oral CHM
3835	Jiang ZS, Zhang SL, Cai XJ. Zhongguo Zhong Xi Yi Jie He Za Zhi. 1997;17(1):32-34.	Not related to Oral CHM
3836	Jiang, Y., Xie, W., Wang, X., An, S., Yang, H., Chen, M., ... & Dai, L. (2022). NURSING RESEARCH ON LIMB REHABILITATION AND MENTAL STATE OF HEMIPLEGIA PATIENTS WITH HYPERTENSIVE CEREBRAL HEMORRHAGE TREATED WITH BAIXIAO MOXIBUSTION. ACTA MEDICA MEDITERRANEA, 38(4), 2939-2944.	Not related to Oral CHM
3837	Jiang Y, Guo H, Liu Y, et al. Songling Xuemaikang capsules for patients with low-to-medium risk hypertension: study protocol for a randomized controlled trial. Trials. 2019;20(1):218. Published 2019 Apr 15. doi:10.1186/s13063-019-3308-5	Not related to Oral CHM
3838	Jiang WM, Fang ZY, Zhu CL, Tang SH. Zhongguo Zhong Xi Yi Jie He Za Zhi. 2013;33(1):35-39.	Not related to Oral CHM
3839	Jiang T, Gu J, Zhang P, Chen W, Chang Q. The effect of adjunctive intravitreal conbercept at the end of diabetic vitrectomy for the prevention of post-vitrectomy hemorrhage in patients with severe proliferative diabetic retinopathy: a prospective, randomized pilot study. BMC Ophthalmol. 2020;20(1):43. Published 2020 Feb 3. doi:10.1186/s12886-020-1321-9	Not related to Oral CHM
3840	Jiang L, Zhang Y, Zhang H, et al. Comparative efficacy of 6 traditional Chinese patent medicines combined with lifestyle modification in patients with prediabetes: A network meta-analysis. Diabetes Res Clin Pract. 2022;188:109878. doi:10.1016/j.diabres.2022.109878	Not related to Oral CHM
3841	Jiang D, Luo LL, Wang H. Zhongguo Zhong Xi Yi Jie He Za Zhi. 2009;29(8):737-739.	Not related to Oral CHM
3842	Jian J, Hao X, Deng C, Zhou H, Lin J. Zhonghua Nei Ke Za Zhi. 1999;38(8):517-519.	Not related to Oral CHM
3843	Jialiken D, Qian L, Ren S, Wu L, Xu J, Zou C. Combined therapy of hypertensive nephropathy with ginkgo leaf extract and dipyridamole injection and antihypertensive drugs: A systematic review and meta-analysis. Medicine (Baltimore). 2021;100(19):e25852. doi:10.1097/MD.0000000000025852	Not related to Oral CHM
3844	Jia Y, Zhong X, Liu J. Zhongguo Zhong Xi Yi Jie He Za Zhi. 2000;20(7):498-500.	Not related to Oral CHM
3845	Jia W, Li Y, Wan J, et al. Effects of Xuezhitong in Patients with Hypertriglyceridemia: a Multicentre, Randomized, Double-Blind, Double Simulation, Positive Drug and Placebo Parallel Control Study. Cardiovasc Drugs Ther. 2020;34(4):525-534. doi:10.1007/s10557-020-06965-3	Not related to Oral CHM
3846	Ji L, Tong X, Wang H, et al. Efficacy and safety of traditional chinese medicine for diabetes: a double-blind, randomised, controlled trial. PLoS One. 2013;8(2):e56703. doi:10.1371/journal.pone.0056703	Not related to Oral CHM

3847	Ji KT, Zhang HQ, Tang JF, Li HY. Zhongguo Zhong Yao Za Zhi. 2007;32(12):1214-1217.	Not related to Oral CHM
3848	Jeschke E, Ostermann T, Tabali M, et al. Pharmacotherapy of elderly patients in everyday anthroposophic medical practice: a prospective, multicenter observational study. BMC Geriatr. 2010;10:48. Published 2010 Jul 21. doi:10.1186/1471-2318-10-48	Not related to Oral CHM
3849	Jenkins AL, Morgan LM, Bishop J, Jovanovski E, Jenkins DJA, Vuksan V. Co-administration of a konjac-based fibre blend and American ginseng (Panax quinquefolius L.) on glycaemic control and serum lipids in type 2 diabetes: a randomized controlled, cross-over clinical trial. Eur J Nutr. 2018;57(6):2217-2225. doi:10.1007/s00394-017-1496-x	Not related to Oral CHM
3850	Jaybashi, B., Hemmatpour, R., & Rahimi, M. (2022). Lavender Scent Inhalation and Anxiety in Type II Diabetes Patients: Effects, Examination and Analysis. Journal of Pharmaceutical Negative Results, 6581-8.	Not related to Oral CHM
3851	Jayawardena MH, de Alwis NM, Hettigoda V, Fernando DJ. A double blind randomised placebo controlled cross over study of a herbal preparation containing Salacia reticulata in the treatment of type 2 diabetes. J Ethnopharmacol. 2005;97(2):215-218. doi:10.1016/j.jep.2004.10.026	Not related to Oral CHM
3852	The Clinical Efficacy and Safety of Tulsi in Humans: A Systematic Review of the Literature	Not related to Oral CHM
3853	Jamil S, Khan RA, Ahmed S. In vivo evaluation of antihyperlipidemic, antihyperglycemic and hepatoprotective effects of Vernonia anthelmintica seeds in diet model. Pak J Pharm Sci. 2018;31(3):813-820.	Not related to Oral CHM
3854	Jalili, C., Moradi, S., Mirzababaei, A., Mohammadi, H., Heydarzadeh, F., Miraghajani, M., & Lazaridi, A. V. (2021). Effects of Anethum graveolens (dill) and its derivatives on controlling cardiovascular risk factors: A systematic review and meta-analysis. Journal of Herbal Medicine, 30, 100516.	Not related to Oral CHM
3855	Jaisamut P, Tohlang C, Wanna S, et al. Clinical Evaluation of a Novel Tablet Formulation of Traditional Thai Polyherbal Medicine Named Nawametho in Comparison with Its Decoction in the Treatment of Hyperlipidemia. Evid Based Complement Alternat Med. 2022;2022:2530266. Published 2022 Aug 3. doi:10.1155/2022/2530266	Clinical Evaluation of a Novel Tablet Formulation of Traditional Thai Polyherbal Medicine Named Nawametho in Comparison with Its Decoction in the Treatment of

		Hyperlipidemi a
385 6	Jacobs AM, Tomczak R. Evaluation of Bensal HP for the treatment of diabetic foot ulcers. Adv Skin Wound Care. 2008;21(10):461-465. doi:10.1097/01.ASW.0000323573.57206.7b	Not related to Oral CHM
385 7	Izzo AA. The clinical efficacy of herbal dietary supplements: A collection of recent systematic reviews and meta-analyses. Phytother Res. 2018;32(8):1423-1424. doi:10.1002/ptr.6128	Not related to Oral CHM
385 8	Izadi, I., Samani, R. R., Tehrani, A. M., Dehghani, M., & Jafari, A. (2020). Glucoherb versus metformin on glycemic markers and glycosylated hemoglobin in prediabetes patients; a clinical trial study. Journal of Nephropharmacology, 10(1), e08-e08.	Not related to Oral CHM
385 9	Ismail MA, Norhayati MN, Mohamad N. Olive leaf extract effect on cardiometabolic profile among adults with prehypertension and hypertension: a systematic review and meta-analysis. PeerJ. 2021;9:e11173. Published 2021 Apr 7. doi:10.7717/peerj.11173	Not related to Oral CHM
386 0	Islam J, Uretsky BF, Sierpina VS. Heart failure improvement with CoQ10, Hawthorn, and magnesium in a patient scheduled for cardiac resynchronization-defibrillator therapy: a case study. Explore (NY). 2006;2(4):339-341. doi:10.1016/j.explore.2006.05.011	Not related to Oral CHM
386 1	Marques AM, Provance DW Jr, Kaplan MAC, Figueiredo MR. Echinodorus grandiflorus: Ethnobotanical, phytochemical and pharmacological overview of a medicinal plant used in Brazil. Food Chem Toxicol. 2017;109(Pt 2):1032-1047. doi:10.1016/j.fct.2017.03.026	Not related to Oral CHM
386 2	Hutapea, A. M., & Simbolon, B. M. (2022). Efficacy of herbal medicine for patients with diabetic neuropathies: An updated literature review. Biomedicine, 42(2), 209-213.	Not related to Oral CHM
386 3	Huseini, H. F., Mohamadzadeh, K., Kianbakht, S., Mohammadi, S. M., Ahvazi, M., Hooseini, M. S., ... & Mohtashami, R. (2023). Antihyperglycemic efficacy and safety of AKROPOL, a Persian medicine poly-herbal extract mixture, in the treatment of type 2 diabetic patients: a randomized, double-blind and placebo-controlled clinical trial. Journal of Medicinal Plants, 22(86), 1-13.	Not related to Oral CHM

3864	Hur, M. H., Hong, J. H., & Yeo, S. (2019). Effects of aromatherapy on stress, fructosamine, fatigue, and sleep quality in prediabetic middle-aged women: a randomised controlled trial. European Journal of Integrative Medicine, 31, 100978.	Not related to Oral CHM
3865	Huo J, Liu LS, Jian WY, et al. Stationary Treatment Compared with Individualized Chinese Medicine for Type 2 Diabetes Patients with Microvascular Complications: Study Protocol for a Randomized Controlled Trial. Chin J Integr Med. 2018;24(10):728-733. doi:10.1007/s11655-018-2987-1	Not related to Oral CHM
3866	Huo J, Duan JG, Liu LS, et al. Evaluation of individualized treatment of nonproliferative diabetic retinopathy: a multicenter, randomized, parallel-controlled study. J Tradit Chin Med. 2022;42(1):90-95. doi:10.19852/j.cnki.jtcm.20210425.002	Not related to Oral CHM
3867	Huntley A, Ernst E. A systematic review of the safety of black cohosh. Menopause. 2003;10(1):58-64. doi:10.1097/00042192-200310010-00010	Not related to Oral CHM
3868	Hung YC, Cheng YC, Muo CH, Chiu HE, Liu CT, Hu WL. Adjuvant Chinese Herbal Products for Preventing Ischemic Stroke in Patients with Atrial Fibrillation. PLoS One. 2016;11(7):e0159333. Published 2016 Jul 18. doi:10.1371/journal.pone.0159333	Not related to Oral CHM
3869	Zhang, H., Xing, Y., Chang, J., Wang, L., An, N., Tian, C., ... & Gao, Y. (2019). Efficacy and safety of NaoShuanTong capsule in the treatment of ischemic stroke: A meta-analysis. Frontiers in Pharmacology, 10, 1133.	Not related to Oral CHM
3870	Huang Y, Wang J, Wang Y, Kuang W, Xie M, Zhang M. Pharmacological mechanism and clinical study of Qiming granules in treating diabetic retinopathy based on network pharmacology and literature review. J Ethnopharmacol. 2023;302(Pt A):115861. doi:10.1016/j.jep.2022.115861	Not related to Oral CHM
3871	Huang Y, Han J, Gu Q, et al. Effect of Yijinjing combined with elastic band exercise on muscle mass and function in middle-aged and elderly patients with prediabetes: A randomized controlled trial. Front Med (Lausanne). 2022;9:990100. Published 2022 Nov 3. doi:10.3389/fmed.2022.990100	Not related to Oral CHM
3872	Huang X, Zhan H, Yang J, et al. Long-Term Effect of Zhenzhu Tiaozhi Capsule (FTZ) on Hyperlipidemia: 2-Year Results from a Retrospective Study Using Electronic Medical Records. Evid Based Complement Alternat Med. 2021;2021:6264414. Published 2021 Oct 18. doi:10.1155/2021/6264414	Not related to Oral CHM
3873	Huang X, Wang L, Yue R, Ding N, Yang H. Large dosage Huangqin (Scutellaria) and Huanglian (Rhizoma Coptidis) for T2DM: A protocol of systematic review and meta-analysis of randomized clinical trials. Medicine (Baltimore). 2020;99(38):e22032. doi:10.1097/MD.0000000000022032	Not related to Oral CHM
3874	Huang SM, Liao XY, Wu LF. Zhongguo Zhong Xi Yi Jie He Za Zhi. 1997;17(10):594-596.	Not related to Oral CHM

3875	Huang Q, Wang JT, Gu HC, Cao G, Cao JC. Comparison of Vacuum Sealing Drainage and Traditional Therapy for Treatment of Diabetic Foot Ulcers: A Meta-Analysis. J Foot Ankle Surg. 2019;58(5):954-958. doi:10.1053/j.jfas.2018.12.020	Not related to Oral CHM
3876	Huang M, Long L, Deng M, et al. Effectiveness and safety of Yufeng Ningxin for the treatment of essential hypertension: A protocol for systematic review and meta-analysis. Medicine (Baltimore). 2021;100(9):e24858. doi:10.1097/MD.0000000000024858	Not related to Oral CHM
3877	Huang L, Xu DQ, Chen YY, Yue SJ, Tang YP. Leonurine, a potential drug for the treatment of cardiovascular system and central nervous system diseases. Brain Behav. 2021;11(2):e01995. doi:10.1002/brb3.1995	Not related to Oral CHM
3878	Huang KY, Chang CH, Yu KC, Hsu CH. Assessment of quality of life and activities of daily living among elderly patients with hypertension and impaired physical mobility in home health care by antihypertensive drugs plus acupuncture: A CONSORT-compliant, randomized controlled trial. Medicine (Baltimore). 2022;101(11):e29077. doi:10.1097/MD.0000000000029077	Not related to Oral CHM
3879	Huang K, Chen Y, Liang K, et al. Review of the Chemical Composition, Pharmacological Effects, Pharmacokinetics, and Quality Control of Boswellia carterii. Evid Based Complement Alternat Med. 2022;2022:6627104. Published 2022 Jan 13. doi:10.1155/2022/6627104	Not related to Oral CHM
3880	Huang H, Tang Y, Wan S. Zhongguo Zhong Xi Yi Jie He Za Zhi. 1996;16(2):70-73.	Not related to Oral CHM
3881	Huang H, Li Y, Huang Q, Lei R, Zou W, Zheng Y. Efficacy of Compound Danshen Dripping Pills combined with western medicine in the treatment of diabetic retinopathy: a systematic review and meta-analysis of randomized controlled trials. Ann Palliat Med. 2021;10(10):10954-10962. doi:10.21037/apm-21-2563	Not related to Oral CHM
3882	Huang FY, Deng T, Meng LX, Ma XL. Dietary ginger as a traditional therapy for blood sugar control in patients with type 2 diabetes mellitus: A systematic review and meta-analysis. Medicine (Baltimore). 2019;98(13):e15054. doi:10.1097/MD.0000000000015054	Not related to Oral CHM
3883	Huang C, Huang C, Zhou G. Danhong injection for the treatment of early diabetic nephropathy: A protocol of systematic review and meta-analysis. Medicine (Baltimore). 2020;99(43):e22716. doi:10.1097/MD.0000000000022716	Not related to Oral CHM
3884	Hu Z, Yang M, Xie C, et al. Efficacy and safety of shenqi compound for the treatment of diabetic macroangiopathy: A protocol for systematic review and meta-analysis. Medicine (Baltimore). 2020;99(15):e19682. doi:10.1097/MD.0000000000019682	Not related to Oral CHM
3885	Hu Z, Yang M, Xie C, et al. Effects of qiming granule for diabetic macular edema: A protocol for systematic review and meta-analysis. Medicine (Baltimore). 2019;98(42):e17496. doi:10.1097/MD.0000000000017496	Not related to Oral CHM

3886	Hu Z, Xie C, Yang M, et al. Add-on effect of Qiming granule, a Chinese patent medicine, in treating diabetic macular edema: A systematic review and meta-analysis. Phytother Res. 2021;35(2):587-602. doi:10.1002/ptr.6844	Not related to Oral CHM
3887	Hu Y, Zhou X, Guo DH, Liu P. Effect of JYTK on Antioxidant Status and Inflammation in Patients With Type 2 Diabetes: A Randomized Double-Blind Clinical Trial. Int J Endocrinol Metab. 2016;14(1):e34400. Published 2016 Jan 30. doi:10.5812/ijem.34400	Not related to Oral CHM
3888	Hu X, Wang J, Yang H, et al. Bailing Capsule combined with 伪-ketoacid tablets for stage 3 chronic kidney disease: Protocol of a double-blinded, randomized, controlled trial.聽Medicine (Baltimore). 2021;100(20):e25759. doi:10.1097/MD.0000000000025759	Not related to Oral CHM
3889	Hu SJ, Fang Q, Liu JS, Zhang L, Cao EZ. Zhongguo Zhong Xi Yi Jie He Za Zhi. 2005;25(2):107-110.	Not related to Oral CHM
3890	Hu B, Yin T, Zhang J, et al. Effect of "maccog" TCM tea on improving glucolipid metabolism and gut microbiota in patients with type 2 diabetes in community. Front Endocrinol (Lausanne). 2023;14:1134877. Published 2023 Mar 8. doi:10.3389/fendo.2023.1134877	Not related to Oral CHM
3891	Hsu PC, Tsai YT, Lai JN, Wu CT, Lin SK, Huang CY. Integrating traditional Chinese medicine healthcare into diabetes care by reducing the risk of developing kidney failure among type 2 diabetic patients: a population-based case control study. J Ethnopharmacol. 2014;156:358-364. doi:10.1016/j.jep.2014.08.029	Not related to Oral CHM
3892	Hsu CH, Liao YL, Lin SC, Hwang KC, Chou P. The mushroom Agaricus Blazei Murill in combination with metformin and gliclazide improves insulin resistance in type 2 diabetes: a randomized, double-blinded, and placebo-controlled clinical trial. J Altern Complement Med. 2007;13(1):97-102. doi:10.1089/acm.2006.6054	Not related to Oral CHM
3893	Hsia SH, Bazargan M, Davidson MB. Effect of Pancreas Tonic (an ayurvedic herbal supplement) in type 2 diabetes mellitus. Metabolism. 2004;53(9):1166-1173. doi:10.1016/j.metabol.2004.04.007	Not related to Oral CHM
3894	Hsia SH, Bazargan M, Davidson MB. Effect of Pancreas Tonic (an ayurvedic herbal supplement) in type 2 diabetes mellitus. Metabolism. 2004;53(9):1166-1173. doi:10.1016/j.metabol.2004.04.007	Not related to Oral CHM
3895	Houston, M. C. (2013). The role of nutrition and nutraceutical supplements in the prevention and treatment of hypertension. Clinical Practice, 10(2), 209.	Not related to Oral CHM
3896	Hosseini, S., Jamshidi, L., Mehrzadi, S., Mohammad, K., Najmizadeh, A. R., Alimoradi, H., & Huseini, H. F. (2014). Effects of Juglans regia L. leaf extract on hyperglycemia and lipid profiles in type two diabetic patients: a randomized double-blind, placebo-controlled clinical trial. Journal of Ethnopharmacology, 152(3), 451-456.	Not related to Oral CHM

3897	Hosseini S, Huseini HF, Larijani B, et al. The hypoglycemic effect of Juglans regia leaves aqueous extract in diabetic patients: A first human trial. Daru. 2014;22(1):19. Published 2014 Jan 21. doi:10.1186/2008-2231-22-19	Not related to Oral CHM
3898	Hosseini B, Saedisomeolia A, Wood LG, Yaseri M, Tavasoli S. Effects of pomegranate extract supplementation on inflammation in overweight and obese individuals: A randomized controlled clinical trial. Complement Ther Clin Pract. 2016;22:44-50. doi:10.1016/j.ctcp.2015.12.003	Not related to Oral CHM
3899	Hoseini SM, Anushiravani M, Mojahedi MJ, et al. The efficacy of camel milk and Tarangabin (manna of Alhagi maurorum(combination therapy on glomerular filtration rate in patients with chronic kidney disease: A randomized controlled trial. Avicenna J Phytomed. 2020;10(2):170-180.	Not related to Oral CHM
3900	Farzaei, M. H., Rahimi, R., Farzaei, F., & Abdollahi, M. (2015). Traditional medicinal herbs for the management of diabetes and its complications: an evidence-based review.	Not related to Oral CHM
3901	Hopkins AL, Lamm MG, Funk JL, Ritenbaugh C. Hibiscus sabdariffa L. in the treatment of hypertension and hyperlipidemia: a comprehensive review of animal and human studies. Fitoterapia. 2013;85:84-94. doi:10.1016/j.fitote.2013.01.003	Not related to Oral CHM
3902	Hook IL. Danggui to Angelica sinensis root: are potential benefits to European women lost in translation? A review. J Ethnopharmacol. 2014;152(1):1-13. doi:10.1016/j.jep.2013.12.018	Not related to Oral CHM
3903	Xu, H. L., Wang, F., Ni, Q., Yu, Z. X., An, R., Zhang, Q. J., & Wu, R. (2021). Observation of effects with natural medicine and food in metabolic syndrome.	Not related to Oral CHM
3904	Hong KF, Liu PY, Zhang W, Gui DK, Xu YH. The Efficacy and Safety of Astragalus as an Adjuvant Treatment for Type 2 Diabetes Mellitus: A Systematic Review and Meta-Analysis. J Integr Complement Med. 2024;30(1):11-24. doi:10.1089/jicm.2022.0767	Not related to Oral CHM
3905	Hoda F, Khanam A, Thareja M, Arshad M, Ahtar M, Najmi AK. Effect of Nigella Sativa in Improving Blood Glucose Level in T2DM: Systematic Literature Review of Randomized Control Trials. Drug Res (Stuttg). 2023;73(1):17-22. doi:10.1055/a-1936-8412	Not related to Oral CHM
3906	Heydari, M., Nimrouzi, M., Hajmohammadi, Z., Faridi, P., Omrani, G. R., & Shams, M. (2019). Rhus coriaria L.(Sumac) in patients who are overweight or have obesity: a placebo-controlled randomized clinical trial. Shiraz E Medical Journal, 20(10), e87301.	Not related to Oral CHM
3907	Heshmat-Ghahdarijani K, Mashayekhiasl N, Amerizadeh A, Teimouri Jervekani Z, Sadeghi M. Effect of fenugreek consumption on serum lipid profile: A systematic review and meta-analysis. Phytother Res. 2020;34(9):2230-2245. doi:10.1002/ptr.6690	Not related to Oral CHM

3908	Herrera-Arellano A, Flores-Romero S, Chávez-Soto MA, Tortoriello J. Effectiveness and tolerability of a standardized extract from Hibiscus sabdariffa in patients with mild to moderate hypertension: a controlled and randomized clinical trial. Phytomedicine. 2004;11(5):375-382. doi:10.1016/j.phymed.2004.04.001	Not related to Oral CHM
3909	Herrera-Arellano A, Aguilar-Santamaría L, García-Hernández B, Nicasio-Torres P, Tortoriello J. Clinical trial of Cecropia obtusifolia and Marrubium vulgare leaf extracts on blood glucose and serum lipids in type 2 diabetics. Phytomedicine. 2004;11(7-8):561-566. doi:10.1016/j.phymed.2004.01.006	Not related to Oral CHM
3910	Hernández-Pérez F, Herrera-Arellano A. Tratamiento de la hipercolesterolemia con Hibiscus sabadariffa. Ensayo clínico aleatorizado controlado [Therapeutic use Hibiscus sabadariffa extract in the treatment of hypercholesterolemia. A randomized clinical trial]. Rev Med Inst Mex Seguro Soc. 2011;49(5):469-480.	Not related to Oral CHM
3911	Hernández-García D, Granado-Serrano AB, Martín-Gari M, Naudí A, Serrano JC. Efficacy of Panax ginseng supplementation on blood lipid profile. A meta-analysis and systematic review of clinical randomized trials. J Ethnopharmacol. 2019;243:112090. doi:10.1016/j.jep.2019.112090	Not related to Oral CHM
3912	Hendre, A. S., Patil, S. R., Sontakke, A. V., & Phatak, R. S. AMELIORATING EFFECT OF TURMERIC ON KIDNEY FUNCTION IN PATIENTS WITH TYPE 2 DIABETES MELLITUS.	Not related to Oral CHM
3913	Helmstädter A. Syzygium cumini (L.) SKEELS (Myrtaceae) against diabetes--125 years of research. Pharmazie. 2008;63(2):91-101.	Not related to Oral CHM
3914	Heirangkhongjam, M. D., & Ngaseppam, I. S. (2018). Traditional medicinal uses and pharmacological properties of Rhus chinensis Mill.: A systematic review. European Journal of Integrative Medicine, 21, 43-49.	Not related to Oral CHM
3915	Heber D, Yip I, Ashley JM, Elashoff DA, Elashoff RM, Go VL. Cholesterol-lowering effects of a proprietary Chinese red-yeast-rice dietary supplement. Am J Clin Nutr. 1999;69(2):231-236. doi:10.1093/ajcn/69.2.231	Not related to Oral CHM
3916	Heber D. Herbal preparations for obesity: are they useful?. Prim Care. 2003;30(2):441-463. doi:10.1016/s0095-4543(03)00015-0	Not related to Oral CHM
3917	He Z, Zheng M, Xie P, Wang Y, Yan X, Deng D. Wenxin Keli for atrial fibrillation: Protocol for a systematic review and meta-analysis. Medicine (Baltimore). 2018;97(17):e0390. doi:10.1097/MD.0000000000010390	Not related to Oral CHM
3918	He Y, Li W, Zhu H, Han S. Economic evaluation of bailing capsules for patients with diabetic nephropathy in China. Front Pharmacol. 2023;14:1175310. Published 2023 Jul 5. doi:10.3389/fphar.2023.1175310	Not related to Oral CHM
3919	He, S. W., He, S. G., Zhao, R. H., Li, S. H., & Li, G. Z. (2005). Effects of the compound purslane on blood lipid, apolipoprotein and hemorheology in patients with hyperlipidemia. Chinese Journal of Clinical Rehabilitation, 9(31), 164-6.	Not related to Oral CHM

3920	He P, Zhang J, Gao T, Wang Y, Peng T. Huangyusang decoction for Type 2 diabetes: A protocol for systematic review and meta analysis. Medicine (Baltimore). 2021;100(8):e24576. doi:10.1097/MD.0000000000024576	Not related to Oral CHM
3921	He H, Chen G, Gao J, et al. Xue-Fu-Zhu-Yu capsule in the treatment of qi stagnation and blood stasis syndrome: a study protocol for a randomised controlled pilot and feasibility trial. Trials. 2018;19(1):515. Published 2018 Sep 21. doi:10.1186/s13063-018-2908-9	Not related to Oral CHM
3922	Hayamizu K, Ishii Y, Kaneko I, et al. Effects of garcinia cambogia (Hydroxycitric Acid) on visceral fat accumulation: a double-blind, randomized, placebo-controlled trial. Curr Ther Res Clin Exp. 2003;64(8):551-567. doi:10.1016/j.curtheres.2003.08.006	Not related to Oral CHM
3923	Moghadam, M. H., Ghasemi, Z., Sepahi, S., Rahbarian, R., Mozaffari, H. M., & Mohajeri, S. A. (2020). Hypolipidemic effect of Lactuca sativa seed extract, an adjunctive treatment, in patients with hyperlipidemia: A randomized double-blind placebo-controlled pilot trial. Journal of Herbal Medicine, 23, 100373.	Not related to Oral CHM
3924	Hassanien, M., Saad, Y., Alawamy, A., Khalifa, W. A., & Abdelkarem, D. (2017). AB0940 Efficacy and safety of platelet rich plasma peri-neural injection in treatment of diabetic neuropathy: double blind randomized controlled trial. Annals of the Rheumatic Diseases, 76, 1384.	Not related to Oral CHM
3925	Hassani, S. S., Fallahi, A., Esmaeili, S. S., & Gholami Fesharaki, M. (2019). The effect of combined therapy with fenugreek and nutrition training based on Iranian traditional medicine on FBS, HgA1c, BMI, and waist circumference in Type 2 diabetic patients: a randomized double blinded clinical trial. Journal of Advances in Medical and Biomedical Research, 27(120), 37-42.	Not related to Oral CHM
3926	Hashem-Dabaghian F, Ghods R, Shojaii A, Abdi L, Campos-Toimil M, Yousefsani BS. Rhus coriaria L., a new candidate for controlling metabolic syndrome: a systematic review. J Pharm Pharmacol. 2022;74(1):1-12. doi:10.1093/jpp/rgab120	Not related to Oral CHM
3927	هاشم دباغیان, فتانه, عبدالهی فرد, مهری, خلیقی سیگارودی, تقوی شیرازی, ... & فلاح حسینی. (2015). Effects of Rosa canina L. fruit on glycemia and lipid profile in type 2 diabetic patients: a randomized, double-blind, placebo-controlled clinical trial. فصلنامه علمی پژوهشی گیاهان دارویی, 14(55), 95-104.	Not related to Oral CHM
3928	Hasani-Ranjbar, S., Nayebi, N., Larijani, B., & Abdollahi, M. (2010). A systematic review of the efficacy and safety of Teucrium species; from anti-oxidant to anti-diabetic effects.	Not related to Oral CHM
3929	Hasani-Ranjbar S, Nayebi N, Larijani B, Abdollahi M. A systematic review of the efficacy and safety of herbal medicines used in the treatment of obesity. World J Gastroenterol. 2009;15(25):3073-3085. doi:10.3748/wjg.15.3073	Not related to Oral CHM
3930	Hasani-Ranjbar, S., Larijani, B., & Abdollahi, M. (2008). A systematic review of Iranian medicinal plants useful in diabetes mellitus. Archives of Medical Science, 4(3), 285-292.	Not related to Oral CHM

3931	Hasani-Ranjbar S, Jouyandeh Z, Abdollahi M. A systematic review of anti-obesity medicinal plants - an update. J Diabetes Metab Disord. 2013;12(1):28. Published 2013 Jun 19. doi:10.1186/2251-6581-12-28	Not related to Oral CHM
3932	Hao WJ, Li BJ, Wu XL, et al. Effect and Safety of Tongyan Spray () on Hyoid Motion in Patients with Dysphagia after Ischemic Stroke. Chin J Integr Med. 2021;27(5):369-374. doi:10.1007/s11655-020-3325-y	Not related to Oral CHM
3933	Hao PP, Jiang F, Chen YG, et al. Traditional Chinese medication for cardiovascular disease. Nat Rev Cardiol. 2015;12(6):318. doi:10.1038/nrcardio.2015.60	Not related to Oral CHM
3934	Hao P, Jiang F, Cheng J, Ma L, Zhang Y, Zhao Y. Traditional Chinese Medicine for Cardiovascular Disease: Evidence and Potential Mechanisms. J Am Coll Cardiol. 2017;69(24):2952-2966. doi:10.1016/j.jacc.2017.04.041	Not related to Oral CHM
3935	Hao P, Jiang F, Cheng J, Ma L, Zhang Y, Zhao Y. Traditional Chinese Medicine for Cardiovascular Disease: Evidence and Potential Mechanisms. J Am Coll Cardiol. 2017;69(24):2952-2966. doi:10.1016/j.jacc.2017.04.041	Not related to Oral CHM
3936	Han, X., Liu, X., Zhong, F., Wang, Y., & Zhang, Q. (2021). Comparison of efficacy and safety of complementary and alternative therapies for essential hypertension with anxiety or depression disorder. PloS one, 16(7), e0254699.	Not related to Oral CHM
3937	Han, S., Hou, Y., Liu, H., & Zhao, Q. (2022). The efficacy and safety of traditional Chinese medicine treating diabetic cardiomyopathy: A protocol for systematic review and meta-analysis. Medicine, 101(47), e31269.	Not related to Oral CHM
3938	Han, R., Gao, L., Sun, H., Li, M., & Deng, C. (2021). Effect of Yiqi Buxue Decoction on Hemodynamic Changes of the Uterine Artery and Fetal Umbilical Artery and Pregnancy Outcomes in Pregnant Patients with Pulmonary Arterial Hypertension. Evidence-Based Complementary and Alternative Medicine, 2021(1), 1849114.	Not related to Oral CHM
3939	Jung ES, Hwang SY, Park SH, Choi EK, Ryu BD, Jeon TI. Combined supplementation with grape pomace and omija fruit extracts improves body composition, plasma lipid profiles, inflammatory status, and antioxidant capacity in overweight and obese subjects: a randomized double-blind placebo-controlled clinical trial. J Med Food. 2020;23(5):515-523. doi:10.1089/jmf.2019.0181.	Not related to Oral CHM
3940	Han, H., Corbin, R., Godfrey, C., Leung, L., Chen, J., Lee, M. S., ... & Feng, L. (2013). The safety and effectiveness of bitter melon (momordica charantia) as an alternative to traditional hypoglycemic agents for the control of fasting blood sugar in patients with type 2 diabetes mellitus: a systematic review protocol. JBI Evidence Synthesis, 11(12), 17-32.	Not related to Oral CHM

3941	Aviram M, Volkova N, Coleman R, Dreher M, Reddy MK, Ferreira D, Rosenblat M. Pomegranate phenolics from the peels, arils, and flowers are antiatherogenic: studies in vivo in atherosclerotic apolipoprotein E-deficient (E(0)) mice and in vitro in cultured macrophages and lipoproteins. J Agric Food Chem. 2008;56(3):1148-1157. doi:10.1021/jf0735592.	Not related to Oral CHM
3942	Hamid, K., Alqahtani, A., Kim, M. S., Cho, J. L., H Cui, P., Guang Li, C., ... & Q Li, G. (2015). Tetracyclic triterpenoids in herbal medicines and their activities in diabetes and its complications. Current Topics in Medicinal Chemistry, 15(23), 2406-2430.	Not related to Oral CHM
3943	Kaatabi H, Bamosa AO, Lebda FM, Al Elq AH, Al-Sultan AI. Favorable impact of Nigella sativa seeds on lipid profile in type 2 diabetic patients. J Family Community Med. 2012;19(3):155-161. doi:10.4103/2230-8229.102315.	Not related to Oral CHM
3944	Mozaffari-Khosravi H, Ahadi Z, Barzegar K, Ebrahimi-Mameghani M, Fallahzadeh H, Dehghani A, Amini M, Rezvani M, Nematollahi S, Heydari MR. Rhus coriaria L. (Sumac) supplementation improves serum glycemic status and lipid profile in patients with type 2 diabetes mellitus: A randomized, double-blind, placebo-controlled clinical trial. Complement Ther Clin Pract. 2014;20(4): 314-319. doi:10.1016/j.ctcp.2014.07.002.	Not related to Oral CHM
3945	Mozaffari-Khosravi H, Jalali-Khanabadi BA, Afkhami-Ardekani M, Fatehi F. Effects of sour tea (Hibiscus sabdariffa) on polygenic dyslipidemia: A randomized clinical trial. Pak J Biol Sci. 2009;12(13):969-975. doi:10.3923/pjbs.2009.969.975.	Not related to Oral CHM
3946	Herrera-Arellano A, Miranda-Sánchez J, Avila-Castro P, Herrera-Alvarez S, Jiménez-Ferrer E, Zamilpa A, Román-Ramos R, Ponce-Monter H, Tortoriello J. Clinical effects produced by a standardized herbal medicinal product of Hibiscus sabdariffa on patients with hypertension: a randomized, double-blind, lisinopril-controlled clinical trial. Planta Med. 2007;73(1):6-12. doi:10.1055/s-2006-957065.	Not related to Oral CHM
3947	Leung PC, Wong SS. A randomized, double-blind, placebo-controlled study of the effect of a Chinese herbal medicine preparation (Dang Gui Buxue Tang) on menopausal symptoms in Hong Kong Chinese women. Climacteric. 2013;16(2):256-263. doi:10.3109/13697137.2012.703408.	Not related to Oral CHM
3948	Harbilas D, Martineau LC, Harris CS, Adeyiwola-Spoor DC, Saleem A, Lambert J, Arnason JT, Haddad PS. Evaluation of the antidiabetic potential of selected medicinal plants from the Cree of Eeyou Istchee (Quebec, Canada): a comprehensive review. J Ethnopharmacol. 2009;123(2):122-143. doi:10.1016/j.jep.2009.02.022.	Not related to Oral CHM
3949	Stohs SJ, Preuss HG, Shara M. The safety of Citrus aurantium (bitter orange) and its primary protoalkaloid p-synephrine. Phytother Res. 2011;25(10):1421-1428. doi:10.1002/ptr.3493.	Not related to Oral CHM

3950	Ha, M. S., Lee, J. H., Jeong, W. M., Kim, H. R., & Son, W. H. (2022). The Combined Intervention of Aqua Exercise and Burdock Extract Synergistically Improved Arterial Stiffness: A Randomized, Double-Blind, Controlled Trial. Metabolites 2022, 12, 970.	Not related to Oral CHM
3951	Patel S, Rauf A. Ayurvedic herbal preparations for hypercholesterolemia: a systematic review and meta-analysis. Phytomedicine. 2017;24:1-11. doi:10.1016/j.phymed.2016.11.018.	Not related to Oral CHM
3952	Gurrola-Díaz CM, García-López PM, Sánchez-Enríquez S, Troyo-Sanromán R, Andrade-González I, Gómez-Leyva JF. Effects of Hibiscus sabdariffa extract powder and preventive treatment (diet) on the lipid profiles of patients with metabolic syndrome (MeSy). Phytomedicine. 2010;17(7):500-505. doi:10.1016/j.phymed.2009.10.014	Not related to Oral CHM
3953	Gupta SC, Sung B, Kim JH, Prasad S, Li S, Aggarwal BB. Multitargeting by turmeric, the golden spice: From kitchen to clinic. Mol Nutr Food Res. 2013;57(9):1510-1528. doi:10.1002/mnfr.201100741	Not related to Oral CHM
3954	Gupta SC, Sung B, Kim JH, Prasad S, Li S, Aggarwal BB. Multitargeting by turmeric, the golden spice: From kitchen to clinic. Mol Nutr Food Res. 2013;57(9):1510-1528. doi:10.1002/mnfr.201100741	Not related to Oral CHM
3955	Gupta A, Gupta R, Lal B. Effect of Trigonella foenum-graecum (fenugreek) seeds on glycaemic control and insulin resistance in type 2 diabetes mellitus: a double blind placebo controlled study. J Assoc Physicians India. 2001;49:1057-1061.	Not related to Oral CHM
3956	Guo Y, Sun J, Zhang R, Yang P, Zhang S, Wu Z. Salvia miltiorrhiza improves type 2 diabetes: A protocol for systematic review and meta-analysis. Medicine (Baltimore). 2021;100(6):e23843. doi:10.1097/MD.0000000000023843	Not related to Oral CHM
3957	Guo L, Chen L, Chang B, Yang L, Liu Y, Feng B. A randomized, open-label, multicentre, parallel-controlled study comparing the efficacy and safety of biphasic insulin aspart 30 plus metformin with biphasic insulin aspart 30 monotherapy for type 2 diabetes patients inadequately controlled with oral antidiabetic drugs: The merit study. Diabetes Obes Metab. 2018;20(12):2740-2747. doi:10.1111/dom.13454	Not related to Oral CHM
3958	Guo L, Chen L, Chang B, Yang L, Liu Y, Feng B. A randomized, open-label, multicentre, parallel-controlled study comparing the efficacy and safety of biphasic insulin aspart 30 plus metformin with biphasic insulin aspart 30 monotherapy for type 2 diabetes patients inadequately controlled with oral antidiabetic drugs: The merit study. Diabetes Obes Metab. 2018;20(12):2740-2747. doi:10.1111/dom.13454	Not related to Oral CHM
3959	Gui QF, Xu ZR, Xu KY, Yang YM. The Efficacy of Ginseng-Related Therapies in Type 2 Diabetes Mellitus: An Updated Systematic Review and Meta-analysis. Medicine (Baltimore). 2016;95(6):e2584. doi:10.1097/MD.0000000000002584.	Not related to Oral CHM

3960	Guan P, Gui D, Xu Y. Evaluation on the Efficacy and Safety of Panax Notoginseng Saponins in the Treatment of Stroke among Elderly People: A Systematic Review and Meta-Analysis of 206 Randomized Controlled Trials. Evid Based Complement Alternat Med. 2023;2023:4312489. doi:10.1155/2023/4312489.	Not related to Oral CHM
3961	Gu Y, Xu X, Wang Z, et al. Chromium-Containing Traditional Chinese Medicine, Tianmai Xiaoke Tablet, for Newly Diagnosed Type 2 Diabetes Mellitus: A Meta-Analysis and Systematic Review of Randomized Clinical Trials. Evid Based Complement Alternat Med. 2018;2018:3708637. doi:10.1155/2018/3708637.	Not related to Oral CHM
3962	Gu X, Huang N, Gu J, Joshi MK, Wang H. Employing observational method for prospective data collection: A case study for analyzing diagnostic process and evaluating efficacy of TCM treatments for diabetes mellitus. J Ethnopharmacol. 2016;192:516-523. doi:10.1016/j.jep.2016.09.015.	Not related to Oral CHM
3963	Grube B, Chong WF, Chong PW, Riede L. Weight reduction and maintenance with IQP-PV-101: A 12-week randomized controlled study with a 24-week open label period. Obesity (Silver Spring). 2014;22(3):645-651. doi:10.1002/oby.20577.	Not related to Oral CHM
3964	Grohmann T, Litts C, Horgan G, et al. Efficacy of Bilberry and Grape Seed Extract Supplement Interventions to Improve Glucose and Cholesterol Metabolism and Blood Pressure in Different Populations—A Systematic Review of the Literature. Nutrients. 2021;13(5):1692. doi:10.3390/nu13051692.	Not related to Oral CHM
3965	Grant SJ, Bensoussan A, Chang D, et al. Chinese herbal medicines for people with impaired glucose tolerance or impaired fasting blood glucose. Cochrane Database Syst Rev. 2009;(4):CD006690. doi:10.1002/14651858.CD006690.pub2.	Not related to Oral CHM
3966	Gouws CA, Georgousopoulou EN, Mellor DD, McKune A, Naumovski N. Effects of the consumption of prickly pear cacti (Opuntia spp.) and its products on blood glucose levels and insulin: A Systematic Review. Medicina (Kaunas). 2019;55(5):138. doi:10.3390/medicina55050138.	Not related to Oral CHM
3967	Gordon RY, Becker DJ. The role of red yeast rice for the physician. Curr Atheroscler Rep. 2011;13(1):73-80. doi:10.1007/s11883-010-0145-0.	Not related to Oral CHM
3968	Gopalakrishna, R. N., Bannimath, G., & Huded, S. P. (2017). Herb-drug Interaction: Effect of Poly-Herbal Formulation on Glibenclamide Therapy in Patients with Type-2 Diabetes Mellitus. Pharmaceutical Methods, 8(1).	Not related to Oral CHM
3969	González MJ, Miranda-Massari JR, Ricart CM. Effect of a dietary supplement combination on weight management, adipose tissue, cholesterol and triglycerides in obese subjects. P R Health Sci J. 2004;23(2):121-124.	Not related to Oral CHM

3970	Golzar M, Saghi E, Rakhshandeh H, Dehnavi Z, Jafarzadeh Esfehani A, Nematy M. Evaluating the effect of an Iranian traditional medicine-based herbal candy on body composition and appetite in overweight and obese adults: A preliminary study. Avicenna J Phytomed. 2023;13(2):165-176. doi:10.22038/AJP.2022.21314.	Not related to Oral CHM
3971	Godard MP, Ewing BA, Pischel I, Ziegler A, Benedek B, Feistel B. Acute blood glucose lowering effects and long-term safety of OpunDia supplementation in pre-diabetic males and females. J Ethnopharmacol. 2010;130(3):631-634. doi:10.1016/j.jep.2010.05.047.	Not related to Oral CHM
3972	Gliozzi M, Walker R, Muscoli S, et al. Bergamot polyphenolic fraction enhances rosuvastatin-induced effect on LDL-cholesterol, LOX-1 expression and protein kinase B phosphorylation in patients with hyperlipidemia. Int J Cardiol. 2013;170(2):140-145. doi:10.1016/j.ijcard.2013.08.125.	Not related to Oral CHM
3973	Giuliani A, Montesanto A, Matacchione G, et al. The association between single nucleotide polymorphisms, including miR-499a genetic variants, and dyslipidemia in subjects treated with pharmacological or phytochemical lipid-lowering agents. Int J Mol Sci. 2022;23(10):5617. doi:10.3390/ijms23105617.	Not related to Oral CHM
3974	Gillies CL, Abrams KR, Lambert PC, et al. Pharmacological and lifestyle interventions to prevent or delay type 2 diabetes in people with impaired glucose tolerance: systematic review and meta-analysis. BMJ. 2007;334(7588):299. doi:10.1136/bmj.39063.689375.55.	Not related to Oral CHM
3975	Ghosh S, Chattopadhyay B, Koley M, et al. Plant extracts as add-on therapeutics in homeopathy: an open-label, randomized trial using mother tinctures in prediabetes. J Integr Complement Med. 2022;28(9):757-767. doi:10.1089/jicm.2022.0508.	Not related to Oral CHM
3976	Ghobadi, A., Amini Behbahani, F., Yousefi, A., Taghavi Shirazi, M., & Behnoud, N. (2019). Medicinal and nutritional properties of Ziziphus jujuba Mill. in traditional persian medicine and modern phytotherapy. Crescent J Med Biol Sci, 6(2), 146-50.	Not related to Oral CHM
3977	Gherbon A, Frandes M, Timar R, Nicula M. Beneficial effects of Aloe ferox on lipid profile, blood pressure, and glycemic control in obese persons: a CONSORT-clinical study. Medicine (Baltimore). 2021;100(50):e28336. doi:10.1097/MD.0000000000028336	Not related to Oral CHM
3978	Gheith O, Sheashaa H, Abdelsalam M, Shoeir Z, Sobh M. Efficacy and safety of Monascus purpureus Went rice in subjects with secondary hyperlipidemia. Clin Exp Nephrol. 2008;12(3):189-194. doi:10.1007/s10157-008-0033-x	Not related to Oral CHM
3979	Ghafouri A, Estêvão MD, Alibakhshi P, et al. Sumac fruit supplementation improve glycemic parameters in patients with metabolic syndrome and related disorders: a systematic review and meta-analysis. Phytomedicine. 2021;90:153661. doi:10.1016/j.phymed.2021.153661	Not related to Oral CHM

3980	Gerards MC, Terlou RJ, Yu H, Koks CHW, Gerdes VEA. Traditional Chinese lipid-lowering agent red yeast rice results in significant LDL reduction but safety is uncertain: a systematic review and meta-analysis. Atherosclerosis. 2015;240(2):415-423. doi:10.1016/j.atherosclerosis.2015.04.004	Not related to Oral CHM
3981	Gerards MC, Terlou RJ, Yu H, Koks CHW, Gerdes VEA. Traditional Chinese lipid-lowering agent red yeast rice results in significant LDL reduction but safety is uncertain: a systematic review and meta-analysis. Atherosclerosis. 2015;240(2):415-423. doi:10.1016/j.atherosclerosis.2015.04.004	Not related to Oral CHM
3982	Geng Y, Wang J, Chen K, Li Q, Ping Z, Xue R, Zhang S. Effects of sea buckthorn (Hippophae rhamnoides L.) on factors related to metabolic syndrome: a systematic review and meta-analysis of randomized controlled trials. Phytother Res. 2022;36(11):4101-4114. doi:10.1002/ptr.7596	Not related to Oral CHM
3983	Karimi SG, Gasparotto-Junior A, Palozi RAC, et al. A randomized crossover intervention study on the effect of a standardized maté extract (Ilex paraguariensis A.St.-Hil.) in men predisposed to cardiovascular risk. Nutrients. 2020;13(1):14. doi:10.3390/nu13010014	Not related to Oral CHM
3984	Ge L, Xie Q, Jiang Y, et al. Genus Lonicera: new drug discovery from traditional usage to modern chemical and pharmacological research. Phytomedicine. 2022;96:153889. doi:10.1016/j.phymed.2021.153889	Not related to Oral CHM
3985	Gavamukulya Y, Wamunyokoli F, El-Shemy HA. Annona muricata: is the natural therapy to most disease conditions including cancer growing in our backyard? A systematic review of its research history and future prospects. Asian Pac J Trop Med. 2017;10(9):835-848. doi:10.1016/j.apjtm.2017.08.009	Not related to Oral CHM
3986	Gato N, Kadowaki A, Hashimoto N, Yokoyama SI, Matsumoto K. Persimmon fruit tannin-rich fiber reduces cholesterol levels in humans. Ann Nutr Metab. 2013;62(1):1-6. doi:10.1159/000343787	Not related to Oral CHM
3987	Garrison SR, Kolber MR, Allan GM, et al. Bedtime versus morning use of antihypertensives for cardiovascular risk reduction (BedMed): protocol for a prospective, randomised, open-label, blinded end-point pragmatic trial. BMJ Open. 2022;12(2):e059711. doi:10.1136/bmjopen-2021-059711	Not related to Oral CHM
3988	Garmendia F, Pando R, Ronceros G. Effect of sacha inchi oil (Plukenetia volúbilis L) on the lipid profile of patients with hyperlipoproteinemia [in Spanish]. Rev Peru Med Exp Salud Publica. 2011;28(4):628-632.	Not related to Oral CHM
3989	Gardner CD, Lawson LD, Block E, Chatterjee LM, Kiazand A, Balise RR, Kraemer HC. Effect of raw garlic vs commercial garlic supplements on plasma lipid concentrations in adults with moderate hypercholesterolemia: a randomized clinical trial. Arch Intern Med. 2007;167(4):346-353. doi:10.1001/archinte.167.4.346	Not related to Oral CHM
3990	Gao, Z. (2015). Effect of Yuquan Pill on blood glucose fluctuation of diabetes mellitus patients receiving insulin therapy with deficiency type of qi and yin. Zhongcaoyao, 2275-2278.	Not related to Oral CHM

3991	Gao Y, Ji Y, Song Y, Gong R, Chen C, Chen H. Clinical efficacy and safety of warm acupuncture in the treatment of type 2 diabetic kidney disease: a protocol of a randomized controlled trial. Medicine (Baltimore). 2022;101(48):e32034. doi:10.1097/MD.0000000000032034	Not related to Oral CHM
3992	Gao Y, Chang S, Du X, Dong J, Xu X, Zhou Y, Lip GYH, Ma C. Association between digoxin use and adverse outcomes among patients in the Chinese Atrial Fibrillation Registry. Am J Cardiovasc Drugs. 2019;19(6):579-587. doi:10.1007/s40256-019-00350-8	Not related to Oral CHM
3993	Gao X, Shang J, Liu H, Yu B. A meta-analysis of the clinical efficacy of TCM decoctions made from formulas in the Liuwei Dihuang Wan categorized formulas in treating diabetic nephropathy proteinuria. Evid Based Complement Alternat Med. 2018;2018:2427301.	Not related to Oral CHM
3994	Gao Q, Cui XY, Dong F, et al. Efficacy and safety of Bushenjiangya-optimized granule for left ventricular diastolic dysfunction in hypertensive patients: a double-blind, randomized, placebo-controlled trial. Evid Based Complement Alternat Med. 2020;2020:7190352.	Not related to Oral CHM
3995	Gao L, Zheng T, Xue W, et al. Efficacy and safety evaluation of Cimicifuga foetida extract in menopausal women. Climacteric. 2018;21(1):69-74.	Not related to Oral CHM
3996	Gao JQ, Liu ZJ, Chen T, Zhao DQ. Pharmaceutical properties of calycosin, the major bioactive isoflavonoid in the dry root extract of Radix astragali. Pharm Biol. 2014;52(9):1217-1222.	Not related to Oral CHM
3997	Gao H, Li X, Gao X, Ma B. Contralateral needling at unblocked collaterals for hemiplegia following acute ischemic stroke. Neural Regen Res. 2013;8(31):2914-2922.	Not related to Oral CHM
3998	Gao F, Hu XF. Effect of Taizhi’an capsule combined with simvastatin on hyperlipidemia in diabetic patients. Chin J Integr Med. 2006;12(1):24-28.	Not related to Oral CHM
3999	Gall A, Butler TL, Lawler S, Garvey G. Traditional, complementary and integrative medicine use among Indigenous peoples with diabetes in Australia, Canada, New Zealand and the United States. Aust N Z J Public Health. 2021;45(6):664-671.	Not related to Oral CHM
4000	Gad MZ, Azab SS, Khattab AR, Farag MA. Over a century since ephedrine discovery: an updated revisit to its pharmacological aspects, functionality and toxicity in comparison to its herbal extracts. Food Funct. 2021;12(20):9563-9582.	Not related to Oral CHM
4001	Fujita H, Yamagami T, Ohshima K. Long-term ingestion of Touchi-extract, an α-glucosidase inhibitor, by borderline and mild type-2 diabetic subjects is safe and significantly reduces blood glucose levels. J Nutr. 2001;131(8):2105-2108.	Not related to Oral CHM
4002	Fujioka K, Greenway F, Sheard J, Ying Y. The effects of grapefruit on weight and insulin resistance: relationship to the metabolic syndrome. J Med Food. 2006;9(1):49-54.	Not related to Oral CHM

4003	Fuangchan A, Sonthisombat P, Seubnukarn T, et al. Hypoglycemic effect of bitter melon compared with metformin in newly diagnosed type 2 diabetes patients. J Ethnopharmacol. 2011;134(2):422-428.	Not related to Oral CHM
4004	Zamola F, Meuselbach K. Effect of Pinus koraiensis seed oil on satiety hormones CCK and GLP-1 and appetite suppression. Transl Biomed. 2010;1(3):5.	Not related to Oral CHM
4005	Forte R, Cennamo G, Finelli ML, Bonavolontà P, de Crecchio G, Greco GM. Combination of flavonoids with Centella asiatica and Melilotus for diabetic cystoid macular edema without macular thickening. J Ocul Pharmacol Ther. 2011;27(2):109-113.	Not related to Oral CHM
4006	Fornasini M, Castro J, Villacrés E, Narváez L, Villamar MP, Baldeón ME. Hypoglycemic effect of Lupinus mutabilis in healthy volunteers and subjects with dysglycemia. Nutr Hosp. 2012;27(2):425-433.	Not related to Oral CHM
4007	Fonollá J, Maldonado-Lobón JA, Luque R, et al. Effects of a combination of extracts from olive fruit and almonds skin on oxidative and inflammation markers in hypercholesterolemic subjects: a randomized controlled trial. J Med Food. 2021;24(5):479-486.	Not related to Oral CHM
4008	Feuerstein JS, Bjerke WS. Powdered red yeast rice and plant stanols and sterols to lower cholesterol. J Diet Suppl. 2012;9(2):110-115.	Not related to Oral CHM
4009	Ferro Y, Montalcini T, Mazza E, et al. Randomized Clinical Trial: Bergamot Citrus and Wild Cardoon Reduce Liver Steatosis and Body Weight in Non-diabetic Individuals Aged Over 50 Years. Front Endocrinol (Lausanne). 2020;11:494. doi:10.3389/fendo.2020.00494.	Not related to Oral CHM
4010	Ferri LAF, Alves-Do-Prado W, Yamada SS, Gazola S, Batista MR, Bazotte RB. Investigation of the antihypertensive effect of oral crude stevioside in patients with mild essential hypertension. Phytother Res. 2006;20(9):732-736. doi:10.1002/ptr.1944.	Not related to Oral CHM
4011	Fernandez ML, Thomas MS, Lemos BS, et al. TA-65, A Telomerase Activator improves Cardiovascular Markers in Patients with Metabolic Syndrome. Curr Pharm Des. 2018;24(17):1905-1911. doi:10.2174/1381612824666180316114832.	Not related to Oral CHM
4012	Ferguson JJA, Oldmeadow C, Bentley D, Eslick S, Garg ML. Effect of a polyphenol-rich dietary supplement containing Pinus massoniana bark extract on blood pressure in healthy adults: a parallel, randomized placebo-controlled trial. Complement Ther Med. 2022;71:102896. doi:10.1016/j.ctim.2022.102896.	Not related to Oral CHM
4013	Ferdousi, F., Alam, M., Araki, R., Suidasari, S., Yokozawa, M., Yamauchi, K., ... & Isoda, H. (2021). Effects of Oral Intake of Olive Leaf Extract on Hematological Parameters: A Double-Blinded, Randomized, Placebo-Controlled Trial in Progress. Blood, 138, 927.	Not related to Oral CHM
4014	Feng K, Tan J, Chen Y. [Treatment of lower extremity diabetic atherosclerotic obliterans with shuxuetong injection] [Article in Chinese]. Zhongguo Zhong Xi Yi Jie He Za Zhi. 2009;29(3):255-257.	Not related to Oral CHM

4015	Feinberg T, Wieland LS, Miller LE, et al. Polyherbal dietary supplementation for prediabetic adults: study protocol for a randomized controlled trial. Trials. 2019;20(1):24. doi:10.1186/s13063-018-3032-6.	Not related to Oral CHM
4016	Fatima, S. S., Sultana, S., & Sultana, A. (2017). A comparative study of two unani regimens in diabetic vulovaginal candidiasis. Oriental Pharmacy and Experimental Medicine, 17, 19-27.	Not related to Oral CHM
4017	Fatima N, Pingali U, Muralidhar N. Study of pharmacodynamic interaction of Phyllanthus emblica extract with clopidogrel and ecosprin in patients with type II diabetes mellitus. Phytomedicine. 2014;21(5):579-585. doi:10.1016/j.phymed.2013.10.024.	Not related to Oral CHM
4018	Farrington R, Musgrave IF, Byard RW. Evidence for the efficacy and safety of herbal weight loss preparations. J Integr Med. 2019;17(2):87-92. doi:10.1016/j.joim.2019.01.009.	Not related to Oral CHM
4019	Abbasalizad Farhangi M, Dehghan P, Tajmiri S, Mesgari Abbasi M. The effects of Nigella sativa on thyroid function, serum Vascular Endothelial Growth Factor (VEGF) - 1, Nesfatin-1 and anthropometric features in patients with Hashimoto's thyroiditis: a randomized controlled trial. BMC Complement Altern Med. 2016;16(1):471. doi:10.1186/s12906-016-1432-2.	Not related to Oral CHM
4020	Fang R, Hu H, Zhou Y, et al. Efficacy and safety of naotaifang capsules for hypertensive cerebral small vessel disease: study protocol for a multicenter, randomized, double-blind, placebo-controlled clinical trial. Front Pharmacol. 2023;13:967457. doi:10.3389/fphar.2022.967457.	Not related to Oral CHM
4021	Fan, W., Wang, H., Yang, B., XU, L., & Liu, G. (2022). Clinical Observation on Wagner 2-3 Diabetic Foot Ulcer Treated by TCM External Treatment Scheme for Euriching Pus for Tissue Growth. Chinese Journal of Experimental Traditional Medical Formulae, 107-114.	Not related to Oral CHM
4022	Fan S, Shi X, Wang A, Hou T, Li K, Diao Y. Evaluation of the key active ingredients of 'Radix Astragali and Rehmanniae Radix Mixture' and related signaling pathways involved in ameliorating diabetic foot ulcers from the perspective of TCM-related theories. J Biomed Inform. 2021 Nov;123:103904. doi:10.1016/j.jbi.2021.103904.	Not related to Oral CHM
4023	Fan M, Guo D, Tian Y, Liu Y, Zhao J. Efficacy and safety of Shugan Jieyu capsule in the treatment of essential hypertension with insomnia, anxiety or depression: a protocol for systematic review and meta-analysis. Medicine (Baltimore). 2021;100(8):e24856. doi:10.1097/MD.0000000000024856.	Not related to Oral CHM
4024	Fan LH, Zhang C, Ai L, et al. Traditional uses, botany, phytochemistry, pharmacology, separation and analysis technologies of Euonymus alatus (Thunb.) Siebold: a comprehensive review. J Ethnopharmacol. 2020;259:112942. doi:10.1016/j.jep.2020.112942	Not related to Oral CHM
4025	Fan JY, Yi T, Sze-To CM, et al. A systematic review of the botanical, phytochemical and pharmacological profile of Dracaena cochinchinensis, a plant source of the ethnomedicine “dragon’s blood.” Molecules. 2014;19(7):10650-10669. doi:10.3390/molecules190710650.	Not related to Oral CHM

4026	Fan H, Lin P, Kang Q, Zhao ZL, Wang J, Cheng JY. Metabolism and pharmacological mechanisms of active ingredients in Erigeron breviscapus. Curr Drug Metab. 2021;22(1):24-39. doi:10.2174/1389200221666201217093255.	Not related to Oral CHM
4027	Fan GJ, Tang XY, Li SL. Effects of Jiangtang Bushen Recipe on serum C-reactive protein, tumor necrosis factor-alpha and interleukin-6 in patients with type 2 diabetes mellitus. Zhongguo Zhong Xi Yi Jie He Za Zhi. 2006;26(4):329-331.	Not related to Oral CHM
4028	Fan GH, Xing ZY, Liu ML, Chen ZQ, Wang YX. Systematic evaluation and trial sequential analysis of efficacy and safety of Yangxue Qingnao Granules in treatment of essential hypertension and its accompanying symptoms. Zhongguo Zhong Yao Za Zhi. 2021;46(6):1523-1536. doi:10.19540/j.cnki.cjcmm.20200712.501.	Not related to Oral CHM
4029	Fan GH, Xing ZY, Liu ML, et al. Systematic evaluation of efficacy and safety of Songling Xuemaikang Capsules in treatment of essential hypertension. Zhongguo Zhong Yao Za Zhi. 2021;46(2):467-477. doi:10.19540/j.cnki.cjcmm.20200401.502.	Not related to Oral CHM
4030	Fan G, Huang H, Lin Y, et al. Herbal medicine foot bath for the treatment of diabetic peripheral neuropathy: protocol for a randomized, double-blind and controlled trial. Trials. 2018;19(1):483. doi:10.1186/s13063-018-2856-4.	Not related to Oral CHM
4031	Fan C, Sun X, Wang X, Yu H. Therapeutic potential of the chemical composition of Dendrobium nobile Lindl. Front Pharmacol. 2023;14:1163830. doi:10.3389/fphar.2023.1163830.	Not related to Oral CHM
4032	Fallah Huseini, H., Kianbakht, S., & Heshmat, R. (2012). Cynara scolymus L. in treatment of hypercholesterolemic type 2 diabetic patients: A randomized double-blind placebo-controlled clinical trial. Jo	Not related to Oral CHM
4033	Fallah Huseini, H., Hooseini, P., Heshmat, R., Yazdani, D., Rahmani, M., Hemati Moqadam, H. R., ... & Alavi, S. H. R. (2006). The clinical investigation of securigera securidaca (L.)(degen & doerfler) seeds in type II diabetic patients a randomized, double-blind, placebo-controlled study. Journal of Medicinal Plants, 5(20), 75-79.	Not related to Oral CHM
4034	Fallah Huseini H, Hasani-Ranjbar S, Nayebi N, et al. Capparis spinosa L. (Caper) fruit extract in treatment of type 2 diabetic patients: a randomized double-blind placebo-controlled clinical trial. Complement Ther Med. 2013;21(5):447-452. doi:10.1016/j.ctim.2013.07.003.	Not related to Oral CHM
4035	Fabiana NL, Chen CLH, Venketasubramanian N, Lee CF, Wong KSL, De Silva DA. Sex and the treatment effect in the Chinese Medicine NeuroAiD Efficacy on Stroke recovery (CHIMES) trial. J Clin Neurosci. 2016;33:269-270. doi:10.1016/j.jocn.2016.04.013.	Not related to Oral CHM
4036	The influence of Hydrastis canadensis on the gastrointestinal microbiota – beneficial or detrimental effects? A critical review to update naturopathic and western herbal clinical practice	Not related to Oral CHM

4037	Esteghamati A, Mazaheri T, Vahidi Rad M, Noshad S. Complementary and alternative medicine for the treatment of obesity: a critical review. Int J Endocrinol Metab. 2015;13(2):e19678. doi:10.5812/ijem.19678	Not related to Oral CHM
4038	Essmat A, Hussein MS. Green tea extract for mild-to-moderate diabetic peripheral neuropathy: a randomized controlled trial. Complement Ther Clin Pract. 2021;43:101317. doi:10.1016/j.ctcp.2021.101317.	Not related to Oral CHM
4039	Esser D, Matualatupauw J, de Vos RCH, Wehrens R, van der Stappen J, van der Meer I, Afman LA. Ayurvedic herbal preparation supplementation does not improve metabolic health in impaired glucose tolerance subjects; observations from a randomised placebo-controlled trial. Nutrients. 2021;13(1):260. doi:10.3390/nu13010260.	Not related to Oral CHM
4040	Ernst E. Complementary/alternative medicine for hypertension: a mini-review. Wien Med Wochenschr. 2005;155(17-18):386-391. doi:10.1007/s10354-005-0205-1.	Not related to Oral CHM
4041	Emamat H, Zahedmehr A, Asadian S, Nasrollahzadeh J. The effect of purple-black barberry (Berberis integerrima) on blood pressure in subjects with cardiovascular risk factors: a randomized controlled trial. J Ethnopharmacol. 2022;289:115097. doi:10.1016/j.jep.2022.115097.	Not related to Oral CHM
4042	Elsaadany MA, AlTwejry HM, Zabran RA, AlShuraim SA, AlShaia WA, Abuzaid OI, et al. Antihyperglycemic Effect of Fenugreek and Ginger in Patients with Type 2 Diabetes: a Double-Blind, Placebo-controlled Study. Curr. Nutr. Food Sci. 2022;18(2):231-237. doi:10.2174/1573401317666210706121806.	Not related to Oral CHM
4043	Elmi GR, Anum K, Saleem K, Fareed R, Noreen S, Wei H, et al. Evaluation of clinical trials of ethnomedicine used for the treatment of diabetes: a systematic review. Front Pharmacol. 2023;14:1176618. doi:10.3389/fphar.2023.1176618.	Not related to Oral CHM
4044	Ellis CL, Edirisinghe I, Kappagoda T, Burton-Freeman B. Attenuation of meal-induced inflammatory and thrombotic responses in overweight men and women after 6-week daily strawberry (Fragaria) intake: a randomized placebo-controlled trial. J Atheroscler Thromb. 2011;18(4):318-327. doi:10.5551/jat.6114.	Not related to Oral CHM
4045	Elkafrawy N, Younes K, Naguib A, Badr H, Zewain SK, Kamel M, et al. Antihypertensive efficacy and safety of a standardized herbal medicinal product of Hibiscus sabdariffa and Olea europaea extracts (NW Roselle): a phase-II, randomized, double-blind, captopril-controlled clinical trial. Phytother Res. 2020;34(12):3379-3387. doi:10.1002/ptr.6792.	Not related to Oral CHM
4046	El-Sayed MIK. Effects of Portulaca oleracea L. seeds in treatment of type-2 diabetes mellitus patients as adjunctive and alternative therapy. J Ethnopharmacol. 2011;137(1):643-651. doi:10.1016/j.jep.2011.06.020.	Not related to Oral CHM
4047	Ejtahed HS, Hasani-Ranjbar S, Soroush AR, Larijani B. Multidimensional perspective of obesity: prevention to treatment. J Diabetes Metab Disord. 2021;23(2):1485-1489. doi:10.1007/s40200-020-00705-y.	Not related to Oral CHM

4048	Eghbali S, Askari SF, Avan R, Sahebkar A. Therapeutic Effects of Punica granatum (Pomegranate): an updated review of clinical trials. J Nutr Metab. 2021;2021:5297162. doi:10.1155/2021/5297162.	Not related to Oral CHM
4049	Egbuna C, Awuchi CG, Kushwaha G, et al. Bioactive compounds effective against type 2 diabetes mellitus: a systematic review. Curr Top Med Chem. 2021;21(12):1067-1095. doi:10.2174/1568026621666210509161059.	Not related to Oral CHM
4050	Eddy JJ, Gideonsen MD, Mack GP. Practical considerations of using topical honey for neuropathic diabetic foot ulcers: a review. WMJ. 2008;107(4):187-190.	Not related to Oral CHM
4051	Ebrahimi E, Shirali S, Afrisham R. Effect and mechanism of herbal ingredients in improving diabetes mellitus complications. Jundishapur J Nat Pharm Prod. 2017;12(1):e31657. doi:10.5812/jjnpp.31657.	Not related to Oral CHM
4052	Dutta S, Ganguly S, Mukherjee SK, et al. Efficacy of individualized homeopathic medicines in intervening with the progression of pre-hypertension to hypertension: a double-blind, randomized, placebo-controlled trial. Explore (NY). 2022;18(3):279-286. doi:10.1016/j.explore.2021.05.007.	Not related to Oral CHM
4053	Dutt, SS, Sameet, M, Dutt, SG, Vijay, C and Dutt, SS, 2016, A randomised clinical study to evaluate the efficacy of an ayurvedic formulation in the management of madhumeha with special reference to diabetes mellitus, International Journal of Research in Ayurveda and Pharmacy	Not related to Oral CHM
4054	Duric L, Milanovic M, Milosevic N, Medic Stojanoska M, Milic N. Herbs for treatment of hyperlipidemia: what is the evidence? Curr Top Nutraceutical Res. 2021;19(2):146-156.	Not related to Oral CHM
4055	Dugoua J, Perri D, Seely D, et al. The anti-diabetic and cholesterol-lowering effects of common and cassia cinnamon (Cinnamomum verum and C. aromaticum): a randomized controlled trial. BMC Complement Altern Med. 2012;12(Suppl 1):P179.	Not related to Oral CHM
4056	Duda G, Suliburska J, Pupek-Musialik D. Effects of short-term garlic supplementation on lipid metabolism and antioxidant status in hypertensive adults. Pharmacol Rep. 2008;60(2):163-170.	Not related to Oral CHM
4057	Duan Y, Pei K, Cai H, Fan KL, Liu X, Cai BC. A new strategy for exploring herb-pair investigation with a case study on Astragali Radix-Corni Fructus in treatment of diabetic nephropathy. Zhongguo Zhong Yao Za Zhi. 2016;41(21):3919-3926.	Not related to Oral CHM
4058	Duan, X., Sun, X., Wang, Y., Wang, X., Zhang, Y., & Cai, X. (2006). Effect of yixin jiangya capsule on left ventricular mass index and plasma neuropeptide Y of primary grade 2 hypertensive patients with hyperactivity of yang due to yin deficiency blood stasis. Chinese Journal of Tissue Engineering Research, 174-176.	Not related to Oral CHM

4059	Duan, X., Yang, D., & Sun, X. (2000). Effect of yimai jiangya extract on platelet activation and fibrinolytic activity and angiotensin II in aged patients with essential hypertension. Zhongguo Zhong xi yi jie he za zhi Zhongguo Zhongxiyi Jiehe Zazhi= Chinese Journal of Integrated Traditional and Western Medicine, 20(7), 508-510.	Not related to Oral CHM
4060	Duan, X., Zhong, S., Sun, X., & Cai, X. (2007). Effects of yixin jiangya capsules on insulin resistance and timor necrosis factor-alpha in cases of primary hypertension with left ventricular hypertrophy. Journal of Traditional Chinese Medicine= Chung i tsa Chih Ying wen pan, 27(2), 96-99.	Not related to Oral CHM
4061	Duan X, Yang D, Sun X. Effect of Yimai Jiangya extract on plasma neuropeptide Y level in patients of senile hypertension with qi-deficiency and blood stasis syndrome. Zhongguo Zhong Xi Yi Jie He Za Zhi. 2000;20(10):750-752.	Not related to Oral CHM
4062	Yang, M., Hu, Z., & Yue, R. (2023). Effects of Sheng-Mai Injection on Diabetes Mellitus: A Systematic Review and Meta-analysis. Endocrine, Metabolic & Immune Disorders-Drug Targets (Formerly Current Drug Targets-Immune, Endocrine & Metabolic Disorders), 23(8), 1051-1067.	Not related to Oral CHM
4063	DU, X., AN, L., Zhang, J., XU, K., Tian, L., LI, S., & Zhang, X. (2023). Notoginseng Radix et Rhizoma Powder Treats Dyslipidemia via PI3K/Akt Signaling Pathway. Chinese Journal of Experimental Traditional Medical Formulae, 88-95.	Not related to Oral CHM
4064	Du L, Zeng D, Hu X, Ren X, He D. The efficacy of autologous platelet-rich gel and traditional Chinese medicine in diabetic foot treatment: a parallel randomized controlled clinical trial. Ann Vasc Surg. 2022;87:529-537.	Not related to Oral CHM
4065	Du JP, Wang CL, Wang PL, et al. Efficacy of Chinese herbs for supplementing qi and activating blood circulation on patients with acute coronary syndrome and type 2 diabetes mellitus after percutaneous coronary intervention: a clinical observation. Zhongguo Zhong Xi Yi Jie He Za Zhi. 2015;35(5):563-567.	Not related to Oral CHM
4066	Du J, Mao Y, Xu Y, et al. Shuangdan Mingmu Capsule for diabetic retinopathy: a systematic review and meta-analysis of randomized controlled trials. Evid Based Complement Alternat Med. 2023;2023:4655109.	Not related to Oral CHM
4067	Du BM, Lu ZL, Chen Z, Wu YF. The beneficial effects of lipid-lowering therapy with xuezhikang on cardiac events and total mortality in coronary heart disease patients with or without hypertension: a random, double-blinded, placebo controlled clinical trial. Zhonghua Xin Xue Guan Bing Za Zhi. 2006;34(10):890-894.	Not related to Oral CHM
4068	Driscoll KS, Appathurai A, Jois M, Radcliffe JE. Effects of herbs and spices on blood pressure: a systematic literature review of randomised controlled trials. J Hypertens. 2019;37(4):671-679.	Not related to Oral CHM

4069	Dooley TP, Paredes Pérez JM, Rengifo Rodriquez C. Stevia and Uncaria extract (GlucoMedix®) reduces glucose levels and the need for medications in type 2 diabetes: an open label case series of six patients. Clin Phytoscience. 2022;8:2.	Not related to Oral CHM
4070	Donato F, Raffetti E, Toninelli G, Festa A, Scarcella C, Castellano M; TRIGU Project Working Group. Guggulu and Triphala for the treatment of hypercholesterolaemia: a placebo-controlled, double-blind, randomised trial. Complement Med Res. 2021;28(3):216-225. doi:10.1159/000510985.	Not related to Oral CHM
4071	Dogara AM. Biological activity and chemical composition of Detarium microcarpum Guill. and Perr—A systematic review. Adv Pharmacol Pharm Sci. 2022;2022:7219401. doi:10.1155/2022/7219401.	Not related to Oral CHM
4072	Dixit, S., & Tiwari, S. (2020). Review on plants for management of diabetes in India: An ethno-botanical and pharmacological perspective. Pharmacognosy Journal, 12(6s).	Not related to Oral CHM
4073	DiSilvestro RA, Olivo Marston S, Zimmerman A, Joseph E, Boeh McCarty C. Borage oil intake by overweight young adults: no effect on metabolic rate; beneficial effects on plasma triglyceride and HDL cholesterol readings. Food Funct. 2021;12(19):8882-8886. doi:10.1039/D1FO01887F.	Not related to Oral CHM
4074	Ding T, Sheng L, Zhu H, et al. Efficacy and safety of external therapy of TCM for diabetic peripheral vascular disease: a protocol for systematic review and meta-analysis. Medicine (Baltimore). 2022;101(51):e32362. doi:10.1097/MD.0000000000032362.	Not related to Oral CHM
4075	Dinda B, Kyriakopoulos AM, Dinda S, et al. Cornus mas L. (cornelian cherry), an important European and Asian traditional food and medicine: ethnomedicine, phytochemistry and pharmacology for its commercial utilization in drug industry. J Ethnopharmacol. 2016;193:670-690. doi:10.1016/j.jep.2016.09.042.	Not related to Oral CHM
4076	Díaz-Juárez JA, Tenorio-López FA, Zarco-Olvera G, Del Valle-Mondragón L, Torres-Narváez JC, Pastelín-Hernández G. Effect of Citrus paradisi extract and juice on arterial pressure both in vitro and in vivo. Phytother Res. 2009;23(7):948-954. doi:10.1002/ptr.2680.	Not related to Oral CHM
4077	Diallo MST, Traore MS, Balde MA, et al. Prevalence, management and ethnobotanical investigation of hypertension in two Guinean urban districts. J Ethnopharmacol. 2019;231:73-79. doi:10.1016/j.jep.2018.07.028.	Not related to Oral CHM
4078	Deyno S, Eneyew K, Seyfe S, Wondim E. Efficacy, safety and phytochemistry of medicinal plants used for the management of diabetes mellitus in Ethiopia: a systematic review. Clin Phytosci. 2021;7(1):16. doi:10.1186/s40816-021-00251-x.	Not related to Oral CHM
4079	Derosa G, Romano D, D’Angelo A, Maffioli P. Berberis aristata combined with Silybum marianum on lipid profile in patients not tolerating statins at high doses. Atherosclerosis. 2015;239(1):87-92. doi:10.1016/j.atherosclerosis.2014.12.043.	Not related to Oral CHM

4080	Derosa G, Romano D, D’Angelo A, Maffioli P. Berberis aristata/Silybum marianum fixed combination (Berberol®) effects on lipid profile in dyslipidemic patients intolerant to statins at high dosages: a randomized, placebo-controlled, clinical trial. Phytomedicine. 2015;22(2):231-237. doi:10.1016/j.phymed.2014.11.018.	Not related to Oral CHM
4081	Derosa G, D’Angelo A, Maffioli P. The role of a fixed Berberis aristata/Silybum marianum combination in the treatment of type 1 diabetes mellitus. Clin Nutr. 2016;35(5):1091-1095. doi:10.1016/j.clnu.2015.08.004.	Not related to Oral CHM
4082	Derosa G, Bonaventura A, Bianchi L, et al. Berberis aristata/Silybum marianum fixed combination on lipid profile and insulin secretion in dyslipidemic patients. Expert Opin Biol Ther. 2013;13(11):1495-1506. doi:10.1517/14712598.2013.832751.	Not related to Oral CHM
4083	Deora N, Venkatraman K. Aloe vera in diabetic dyslipidemia: improving blood glucose and lipoprotein levels in pre-clinical and clinical studies. J Ayurveda Integr Med. 2022;13(4):100675. doi:10.1016/j.jaim.2022.100675.	Not related to Oral CHM
4084	Dengqin, W., Xiaoxue, Z., Guohong, S., Lei, L., & Bing, S. (2018). Effect of Gastrodia elata-notoginseng decoction on blood pressure variability and inflammatory factors in old hypertensive patients with yin-deficiency and yang-hyperactivity syndrome.聽Biomedical Research (0970-938X).	Not related to Oral CHM
4085	Deng Z, Wang M, Fan YH, et al. A systematic review of randomized controlled trials of the Wenyang Huoxue method in treating diabetic peripheral neuropathy. Medicine (Baltimore). 2019;98(42):e17618. doi:10.1097/MD.0000000000017618	Not related to Oral CHM
4086	Deng, Y. Q., Fan, X. F., & Wu, G. L. (2006). Effect of yuquan pill on proinflammatory cytokines in patients with type 2 diabetes mellitus. Zhongguo Zhong xi yi jie he za zhi Zhongguo Zhongxiyi Jiehe Zazhi= Chinese Journal of Integrated Traditional and Western Medicine, 26(8), 706-709.	Not related to Oral CHM
4087	Sun, H. H., & Yang, Q. 密蒙花茶联合复方血栓通胶囊治疗轻中度单纯型 DR 的临床初探.	Not related to Oral CHM
4088	Deng AP, Zhang Y, Zhou L, et al. Systematic review of the alkaloid constituents in several important medicinal plants of the Genus Corydalis. Phytochemistry. 2021;183:112644. doi:10.1016/j.phytochem.2020.112644	Not related to Oral CHM
4089	Demmers A, Korthout H, van Etten-Jamaludin FS, Kortekaas F, Maaskant JM. Effects of medicinal food plants on impaired glucose tolerance: a systematic review of randomized controlled trials. Diabetes Res Clin Pract. 2017;131:91-106. doi:10.1016/j.diabres.2017.05.024	Not related to Oral CHM
4090	Vilhena RO, Fachi MM, Marson BM, et al. Antidiabetic potential of Musa spp. inflorescence: a systematic review. J Pharm Pharmacol. 2018;70(12):1583-1595. doi:10.1111/jphp.13020	Not related to Oral CHM

4091	Varas, B., & Fuentes, M. M. (2019). AB0453 RED RICE YEAST. AN ALTERNATIVE IN RHEUMATOID ARTHRITIS AND HYPERLIPIDEMIA. Annals of the Rheumatic Diseases, 78, 1690.	Not related to Oral CHM
4092	de Bock M, Derraik JGB, Brennan CM, et al. Olive (Olea europaea L.) leaf polyphenols improve insulin sensitivity in middle-aged overweight men: a randomized, placebo-controlled, crossover trial. PLoS One. 2013;8(3):e57622. doi:10.1371/journal.pone.0057622	Not related to Oral CHM
4093	de Almeida VL, Silva CG, Silva AF, et al. Aspidosperma species: a review of their chemistry and biological activities. J Ethnopharmacol. 2019;231:125-140. doi:10.1016/j.jep.2018.10.039	Not related to Oral CHM
4094	Davinelli S, Bertoglio JC, Zarrelli A, Pina R, Scapagnini G. A randomized clinical trial evaluating the efficacy of an anthocyanin - maqui berry extract (Delphinol®) on oxidative stress biomarkers. J Am Coll Nutr. 2015;34 Suppl 1:28-33. doi:10.1080/07315724.2015.1080108	Not related to Oral CHM
4095	Davidson MH, Maki KC, Dicklin MR, et al. Effects of consumption of pomegranate juice on carotid intima-media thickness in men and women at moderate risk for coronary heart disease. Am J Cardiol. 2009;104(7):936-942. doi:10.1016/j.amjcard.2009.05.037	Not related to Oral CHM
4096	Datau EA, Wardhana, Surachmanto EE, Pandelaki K, Langi JA, Fias. Efficacy of Nigella sativa on serum free testosterone and metabolic disturbances in central obese male. Acta Med Indones. 2010;42(3):130-134.	Not related to Oral CHM
4097	Daswani PG, Gholkar MS, Birdi TJ. Psidium guajava: a single plant for multiple health problems of rural Indian population. Pharmacogn Rev. 2017;11(22):167-174. doi:10.4103/phrev.phrev_17_17	Not related to Oral CHM
4098	Dashti S, Hadjzadeh MA, Ghorbani A, Mohebbi M, Gholamnezhad Z. The antihyperglycemic and hypolipidemic effects of Ribes khorassanicum hydro-ethanolic extract co-administration in type 2 diabetic patients: a randomized double-blind placebo-controlled trial. Avicenna J Phytomed. 2022;12(2):145-154. doi:10.22038/AJP.2021.51446.2676	Not related to Oral CHM
4099	Daneshi-Maskooni M, Keshavarz SA, Qorbani M, et al. Green cardamom supplementation improves serum irisin, glucose indices, and lipid profiles in overweight or obese non-alcoholic fatty liver disease patients: a double-blind randomized placebo-controlled clinical trial. BMC Complement Altern Med. 2019;19:59. doi:10.1186/s12906-019-2465-0	Not related to Oral CHM
4100	Dalli, E., Colomer, E., Tormos, M. C., Cosín-Sales, J., Milara, J., Esteban, E., & Sáez, G. (2011). Crataegus laevigata decreases neutrophil elastase and has hypolipidemic effect: a randomized, double-blind, placebo-controlled trial. Phytomedicine, 18(8-9), 769-775.	Not related to Oral CHM
4101	Dallas, C., Gerbi, A., Elbez, Y., Caillard, P., Zamaria, N., & Cloarec, M. (2014). Clinical study to assess the efficacy and safety of a citrus polyphenolic extract of red orange, grapefruit, and orange (Sinetrol - XPur) on weight management and metabolic parameters in healthy overweight individuals. Phytotherapy Research, 28(2), 212-218.	Not related to Oral CHM

410<br>2	Dajani EZ, Shahwan TG, Dajani NE. Overview of the preclinical pharmacological properties of Nigella sativa (black seeds): a complementary drug with historical and clinical significance. J Physiol Pharmacol. 2016;67(6):801-817.	Not related to Oral CHM
410<br>3	Dai Y, Guo M, Jiang L, Gao J. Network pharmacology-based identification of miRNA expression of Astragalus membranaceus in the treatment of diabetic nephropathy. Medicine (Baltimore). 2022;101(5):e28747.	Not related to Oral CHM
410<br>4	Dai N, Zhao FF, Fang M, Pu FL, Kong LY, Liu JP. Gynostemma pentaphyllum for dyslipidemia: a systematic review of randomized controlled trials. Front Pharmacol. 2022;13:917521.	Not related to Oral CHM
410<br>5	Dai L, Jiang Y, Wang P, Chen K. Effects of three traditional Chinese fitness exercises combined with antihypertensive drugs on patients with essential hypertension: a systematic review and network meta-analysis of randomized controlled trials. Evid Based Complement Alternat Med. 2021;2021:2570472.	Not related to Oral CHM
410<br>6	Dadjo Y, Panahi Y, Pishgoo B, et al. Effects of supplementation with Heracleum persicum fruit extract on serum lipids in patients undergoing coronary angiography: a pilot trial. Phytother Res. 2015;29(1):141-143.	Not related to Oral CHM
410<br>7	Costello RB, Dwyer JT, Saldanha L, et al. Do cinnamon supplements have a role in glycemic control in type 2 diabetes? A narrative review. J Acad Nutr Diet. 2016;116(11):1794-1802.	Not related to Oral CHM
410<br>8	Fiorentino A, Caporaso N, Morisco F. Berberis aristata, Elaeis guineensis and Coffea canephora extracts modulate the insulin receptor expression and improve hepatic steatosis in NAFLD patients: a pilot clinical trial. Nutrients. 2019;11(12):3070.	Not related to Oral CHM
410<br>9	Cortés-Martín A, Iglesias-Aguirre CE, Meoro A, Selma MV, Espín JC. Pharmacological therapy determines the gut microbiota modulation by a pomegranate extract nutraceutical in metabolic syndrome: a randomized clinical trial. Mol Nutr Food Res. 2021;65(6):e2001048.	Not related to Oral CHM
411<br>0	Cicero AFG, Fogacci F, Bove M, et al. Short-term effects of dry extracts of artichoke and berberis in hypercholesterolemic patients without cardiovascular disease. Am J Cardiol. 2019;123(4):588-591.	Not related to Oral CHM
411<br>1	Cicero AFG, Derosa G, Brillante R, et al. Effects of Siberian ginseng (Eleutherococcus senticosus Maxim.) on elderly quality of life: a randomized clinical trial. Arch Gerontol Geriatr Suppl. 2004;(9):69-73.	Not related to Oral CHM
411<br>2	Cicero AFG, Colletti A, Fogacci F, Bove M, Rosticci M, Borghi C. Effects of a combined nutraceutical on lipid pattern, glucose metabolism and inflammatory parameters in moderately hypercholesterolemic subjects: a double-blind, cross-over, randomized clinical trial. High Blood Press Cardiovasc Prev. 2017;24(1):13-18.	Not related to Oral CHM
411<br>3	Chui SH, Chan KC, Chui AKK, Shek LSL, Wong RNS. The effects of a Chinese medicinal suppository (Vitalliver) on insulin-like growth factor 1 and homocysteine in patients with hepatitis B infection. Phytother Res. 2005;19(8):674-678.	Not related to Oral CHM

4114	Chuah LO, Yeap SK, Ho WY, Beh BK, Alitheen NB. In vitro and in vivo toxicity of Garcinia or hydroxycitric acid: a review. Evid Based Complement Alternat Med. 2012;2012:197920.	Not related to Oral CHM
4115	Chu TTW, Benzie IFF, Lam CWK, et al. Study of potential cardioprotective effects of Ganoderma lucidum (Lingzhi): results of a controlled human intervention trial. Br J Nutr. 2012;107(7):1017-1027.	Not related to Oral CHM
4116	Chu N, Chan JCN. Pharmacomicrobiomics in Western medicine and traditional Chinese medicine in type 2 diabetes. Front Endocrinol (Lausanne). 2022;13:857090.	Not related to Oral CHM
4117	Chrysant SG, Chrysant GS. Herbs used for the treatment of hypertension and their mechanism of action. Curr Hypertens Rep. 2017;19(9):77.	Not related to Oral CHM
4118	Chrubasik S. Clinical efficacy of a Phaseolus vulgaris and Cynara scolymus mixture on satiety. Focus Altern Complement Ther. 2012;17(1):75-77.	Not related to Oral CHM
4119	Chrubasik C, Roufogalis BD, Müller-Ladner U, Chrubasik S. A systematic review on the Rosa canina effect and efficacy profiles. Phytother Res. 2008;22(6):725-33.	Not related to Oral CHM
4120	Choudhary N, Singh V. Multi-scale mechanism of antiviral drug-alike phytoligands from Ayurveda in managing COVID-19 and associated metabolic comorbidities: insights from network pharmacology. Mol Divers. 2022;26(5):2575-2594.	Not related to Oral CHM
4121	Choudhary D, Bhattacharyya S, Joshi K. Body weight management in adults under chronic stress through treatment with Ashwagandha root extract: a double-blind, randomized, placebo-controlled trial. J Evid Based Complement Altern Med. 2017;22(1):96-106.	Not related to Oral CHM
4122	Chong PW, Beah ZM, Grube B, Riede L. IQP-GC-101 reduces body weight and body fat mass: a randomized, double-blind, placebo-controlled study. Phytother Res. 2014;28(10):1520-6.	Not related to Oral CHM
4123	Chokpaisarn J, Chusri S, Voravuthikunchai SP. Clinical randomized trial of topical Quercus infectoria ethanolic extract for the treatment of chronic diabetic ulcers. J Herb Med. 2020;21:100301.	Not related to Oral CHM
4124	Cho YG, Jung JH, Kang JH, Kwon JS, Yu SP, Baik TG. Effect of a herbal extract powder (YY-312) from Imperata cylindrica Beauvois, Citrus unshiu Markovich, and Evodia officinalis Dode on body fat mass in overweight adults: a 12-week, randomized, double-blind, placebo-controlled, parallel-group clinical trial. BMC Complement Altern Med. 2017;17:375.	Not related to Oral CHM
4125	Cho IJ, Choung SY, Hwang YC, Ahn KJ, Chung HY, Jeong IK. Aster spathulifolius Maxim extract reduces body weight and fat mass in obese humans. Nutr Res. 2016;36(7):671-678.	Not related to Oral CHM
4126	Chiu HF, Chen YJ, Lu YY, Han YC, Shen YC, Venkatakrishnan K, Wang CK. Regulatory efficacy of fermented plant extract on the intestinal microflora and lipid profile in mildly hypercholesterolemic individuals. J Food Drug Anal. 2017;25(4):819-827.	Not related to Oral CHM

4127	Chi S, She G, Han D, Wang W, Liu Z, Liu B. Genus Tinospora: Ethnopharmacology, Phytochemistry, and Pharmacology. Evid Based Complement Alternat Med. 2016;2016:9232593.	Not related to Oral CHM
4128	Cheng MH, Hsieh CL, Wang CY, Tsai CC, Kuo CC. Complementary therapy of traditional Chinese medicine for blood sugar control in a patient with type 1 diabetes. Complement Ther Med. 2017;30:10-13.	Not related to Oral CHM
4129	Cheng L, Zhang G, Zhou Y, et al. Systematic review and meta-analysis of 16 randomized clinical trials of Radix Astragali and its prescriptions for diabetic retinopathy. Evid Based Complement Alternat Med. 2013;2013:762783.	Not related to Oral CHM
4130	Chen Z, Peng YY, Yang FW, et al. Meta-analysis of clinical efficacy and safety of Compound Danshen Dripping Pills combined with conventional antihypertensive drugs in treatment of hypertensive left ventricular hypertrophy. Zhongguo Zhong Yao Za Zhi. 2021;46(10):2578-2587.	Not related to Oral CHM
4131	Chen Z, Peng Y, Yang F, et al. Traditional Chinese Medicine injections combined with antihypertensive drugs for hypertensive nephropathy: a network meta-analysis. Front Pharmacol. 2021;12:740821.	Not related to Oral CHM
4132	Chen YZ, Gong ZX, Cai GY, et al. Efficacy and safety of Flos Abelmoschus manihot (Malvaceae) on type 2 diabetic nephropathy: a systematic review. Chin J Integr Med. 2015;21(6):464-472.	Not related to Oral CHM
4133	Chen YH, Lei SS, Li B, et al. Systematic understanding of the mechanisms of Flos chrysanthemi indici-mediated effects on hypertension via computational target fishing. Comb Chem High Throughput Screen. 2020;23(2):92-110.	Not related to Oral CHM
4134	Chen Y, Lu W, Yang K, et al. Tetramethylpyrazine: A promising drug for the treatment of pulmonary hypertension. Br J Pharmacol. 2020;177(12):2743-2764.	Not related to Oral CHM
4135	Chen Y, He W, Ouyang H, et al. Efficacy and safety of tetramethylpyrazine phosphate on pulmonary hypertension: study protocol for a randomized controlled study. Trials. 2019;20(1):725.	Not related to Oral CHM
4136	Chen XM, Lin GX, Wang X, et al. Beneficial effects of ginsenosides on diabetic nephropathy: a systematical review and meta-analysis of preclinical evidence. J Ethnopharmacol. 2023;302(Pt A):115860.	Not related to Oral CHM
4137	Chen X, Yu J, Zhong B, et al. Pharmacological activities of dihydrotanshinone I, a natural product from Salvia miltiorrhiza Bunge. Pharmacol Res. 2019;145:104254.	Not related to Oral CHM
4138	Chen X, Li L, Xu X, et al. Tianma Gouteng Decoction combined with Qiju Dihuang Pill for the treatment of essential hypertension: A protocol for systematic review and meta-analysis. Medicine (Baltimore). 2020;99(29):e21157.	Not related to Oral CHM
4139	Chen WQ, Chen FR. [Effect of songling xuemaikang capsule combined with captopril on quality of life in primary hypertension patients]. Zhongguo Zhong Xi Yi Jie He Za Zhi. 2001;21(9):660-662.	Not related to Oral CHM

4140	Chen WH, Zhang ZZ, Ban YF, et al. Cynanchum bungei Decne and its two related species for "Baishouwu": a review on traditional uses, phytochemistry, and pharmacological activities. J Ethnopharmacol. 2019;243:112110.	Not related to Oral CHM
4141	Chen W, Zhang Y, Li X, Yang G, Liu JP. Chinese herbal medicine for diabetic peripheral neuropathy. Cochrane Database Syst Rev. 2013;(10):CD007796.	Not related to Oral CHM
4142	Hao CZ, Wu F, Lu L, et al. Chinese herbal medicine for diabetic peripheral neuropathy: an updated meta-analysis of 10 high-quality randomized controlled studies. PLoS One. 2013;8(10):e76113.	Not related to Oral CHM
4143	Chen KH, Yeh MH, Livneh H, et al. Association of traditional Chinese medicine therapy and the risk of dementia in patients with hypertension: a nationwide population-based cohort study. BMC Complement Altern Med. 2017;17(1):178.	Not related to Oral CHM
4144	Chen J, Ma Y, Liang H. [Effect of different injections of Chinese herbal medicine on stress hormones and immune cell factors in patients of type 2 diabetes mellitus complicated with acute cerebral infarction]. Zhongguo Zhong Xi Yi Jie He Za Zhi. 2000;20(11):815-817.	Not related to Oral CHM
4145	Chen J, Cai J, Wei M, et al. Effects of Guizhi decoction for diabetic cardiac autonomic neuropathy: A protocol for a systematic review and meta-analysis. Medicine (Baltimore). 2020;99(39):e22317.	Not related to Oral CHM
4146	Chen HH, Wu CT, Tsai YT, Ho CW, Hsieh MC, Lai JN. Liu Wei Di Huang Wan and the delay of insulin use in patients with type 2 diabetes in Taiwan: a nationwide study. Evid Based Complement Alternat Med. 2021;2021:1298487.	Not related to Oral CHM
4147	Chen H, Lv X, Zhang Y. Effect of nursing intervention on promoting healing of RW in patients with diabetic foot: a systematic review and meta-analysis. Comput Math Methods Med. 2022;2022:8284870.	Not related to Oral CHM
4148	Chen H, Guo J, Zhao X, et al. Retrospective analysis of the overt proteinuria diabetic kidney disease in the treatment of modified Shenzhuo formula for 2 years. Medicine (Baltimore). 2017;96(12):e6349.	Not related to Oral CHM
4149	Chen G, Wei B, Wang J, et al. Shensongyangxin capsules for paroxysmal atrial fibrillation: a systematic review of randomized clinical trials. PLoS One. 2016;11(3):e0151880.	Not related to Oral CHM
4150	Chen CJ, Liu X, Chiou JS, et al. Effects of Chinese herbal medicines on dementia risk in patients with sleep disorders in Taiwan. J Ethnopharmacol. 2021;264:113267. doi:10.1016/j.jep.2020.113267	Not related to Oral CHM
4151	Chen C, Lv H, Shan L, et al. Antiplatelet effect of ginkgo diterpene lactone meglumine injection in acute ischemic stroke: A randomized, double-blind, placebo-controlled clinical trial [published correction appears in Phytother Res. 2023 Oct;37(10):4862. doi: 10.1002/ptr.7980.]. Phytother Res. 2023;37(5):1986-1996. doi:10.1002/ptr.7720	Not related to Oral CHM

415<br>2	Benjian C, Xiaodan H, Huiting P, et al. Effectiveness and safety of red yeast rice predominated by monacolin K 尾-hydroxy acid form for hyperlipidemia treatment and management.聽J Tradit Chin Med. 2022;42(2):264-271. doi:10.19852/j.cnki.jtcm.2022.02.007	Not related to Oral CHM
415<br>3	Chatree S, Sitticharoon C, Maikaew P, et al. Cissus Quadrangularis enhances UCP1 mRNA, indicative of white adipocyte browning and decreases central obesity in humans in a randomized trial. Sci Rep. 2021;11(1):2008. Published 2021 Jan 21. doi:10.1038/s41598-021-81606-9	Not related to Oral CHM
415<br>4	Chang HC, Huang CN, Yeh DM, Wang SJ, Peng CH, Wang CJ. Oat prevents obesity and abdominal fat distribution, and improves liver function in humans. Plant Foods Hum Nutr. 2013;68(1):18-23. doi:10.1007/s11130-013-0336-2	Not related to Oral CHM
415<br>5	Chaney T, Chaney S, Lambert J. The Use of Personalized Functional Medicine in the Management of Type 2 Diabetes: A Single-Center Retrospective Interventional Pre-Post Study. Altern Ther Health Med. 2022;28(6):8-13.	Not related to Oral CHM
415<br>6	Chandra K, Jain V, Jabin A, et al. Effect of Cichorium intybus seeds supplementation on the markers of glycemic control, oxidative stress, inflammation, and lipid profile in type 2 diabetes mellitus: A randomized, double-blind placebo study. Phytother Res. 2020;34(7):1609-1618. doi:10.1002/ptr.6624	Not related to Oral CHM
415<br>7	Chan KW, Kwong ASK, Tsui PN, et al. Efficacy, safety and response predictors of adjuvant astragalus for diabetic kidney disease (READY): study protocol of an add-on, assessor-blind, parallel, pragmatic randomised controlled trial. BMJ Open. 2021;11(1):e042686. Published 2021 Jan 12. doi:10.1136/bmjopen-2020-042686	Not related to Oral CHM
415<br>8	Chan KW, Kwong ASK, Tan KCB, et al. Add-on Rehmannia-6-Based Chinese Medicine in Type 2 Diabetes and CKD: A Multicenter Randomized Controlled Trial. Clin J Am Soc Nephrol. 2023;18(9):1163-1174. doi:10.2215/CJN.0000000000000199	Not related to Oral CHM
415<br>9	Chan KW, Chow TY, Yu KY, et al. Effectiveness of Integrative Chinese-Western Medicine for Chronic Kidney Disease and Diabetes: A Retrospective Cohort Study. Am J Chin Med. 2022;50(2):371-388. doi:10.1142/S0192415X2250015X	Not related to Oral CHM
416<br>0	Chan HT, So LT, Li SW, Siu CW, Lau CP, Tse HF. Effect of herbal consumption on time in therapeutic range of warfarin therapy in patients with atrial fibrillation. J Cardiovasc Pharmacol. 2011;58(1):87-90. doi:10.1097/FJC.0b013e31821cd888	Not related to Oral CHM
416<br>1	Chakrabarty N, Chung HJ, Alam R, et al. Chemico-Pharmacological Screening of the Methanol Extract of Gynura nepalensis D.C. Deciphered Promising Antioxidant and Hepatoprotective Potentials: Evidenced from in vitro, in vivo, and Computer-Aided Studies. Molecules. 2022;27(11):3474. Published 2022 May 27. doi:10.3390/molecules27113474	Not related to Oral CHM

416 2	Chagan L, Ioselovich A, Asherova L, Cheng JW. Use of alternative pharmacotherapy in management of cardiovascular diseases. Am J Manag Care. 2002;8(3):270-288.	Not related to Oral CHM
416 3	Cerović A, Miletić I, Konić-Ristić A, et al. The dry plant extract of common bean seed (Phaseoli vulgari pericarpium) does not have an affect on postprandial glycemia in healthy human subject. Bosn J Basic Med Sci. 2006;6(3):28-33. doi:10.17305/bjbms.2006.3140	Not related to Oral CHM
416 4	Cercato LM, White PA, Nampo FK, Santos MR, Camargo EA. A systematic review of medicinal plants used for weight loss in Brazil: Is there potential for obesity treatment?. J Ethnopharmacol. 2015;176:286-296. doi:10.1016/j.jep.2015.10.038	Not related to Oral CHM
416 5	Cawich SO, Harnarayan P, Budhooram S, Bobb NJ, Islam S, Naraynsingh V. Wonder of Life (kalanchoe pinnata) leaves to treat diabetic foot infections in Trinidad & Tobago: a case control study. Trop Doct. 2014;44(4):209-213. doi:10.1177/0049475514543656	Not related to Oral CHM
416 6	Cases J, Romain C, Dallas C, Gerbi A, Rouanet JM. A 12-week randomized double-blind parallel pilot trial of Sinetrol XPur on body weight, abdominal fat, waist circumference, and muscle metabolism in overweight men. Int J Food Sci Nutr. 2015;66(4):471-477. doi:10.3109/09637486.2015.1042847	Not related to Oral CHM
416 7	Cases J, Romain C, Dallas C, Gerbi A, Cloarec M. Regular consumption of Fiit-ns, a polyphenol extract from fruit and vegetables frequently consumed within the Mediterranean diet, improves metabolic ageing of obese volunteers: a randomized, double-blind, parallel trial. Int J Food Sci Nutr. 2015;66(1):120-125. doi:10.3109/09637486.2014.971229	Not related to Oral CHM
416 8	Carvalho AF, Feitosa MC, Coelho NP, et al. Low-level laser therapy and Calendula officinalis in repairing diabetic foot ulcers. Rev Esc Enferm USP. 2016;50(4):628-634. doi:10.1590/S0080-623420160000500013	Not related to Oral CHM
416 9	Carneiro DM, Jardim TV, Araújo YCL, et al. Antihypertensive effect of Equisetum arvense L.: a double-blind, randomized efficacy and safety clinical trial. Phytomedicine. 2022;99:153955. doi:10.1016/j.phymed.2022.153955	Not related to Oral CHM
417 0	Cao Y, Liu LT, Wu M. Is Chinese herbal medicine effective for elderly isolated systolic hypertension? A systematic review and meta-analysis. Chin J Integr Med. 2017;23(4):298-305. doi:10.1007/s11655-016-2257-z	Not related to Oral CHM
417 1	Cao, WH, Zhou, L and Liu, ZH, 2005, Effects of tongxinluo capsule on nerve conduction velocity in patients with diabetic peripheral neuropathy, Chinese Journal of Clinical Rehabilitation	Not related to Oral CHM
417 2	Cao WH, Huang LH, Guo M. Zhongguo Zhong Xi Yi Jie He Za Zhi. 2005;25(11):1022-1024.	Not related to Oral CHM
417 3	Candar A, Demirci H, Baran AK, Akp谋nar Y. The association between quality of life and complementary and alternative medicine use in patients with diabetes mellitus.聽Complement Ther Clin Pract. 2018;31:1-6. doi:10.1016/j.ctcp.2018.01.002	Not related to Oral CHM

417<br>4	Campbell-Tofte JI, Mølgaard P, Josefsen K, et al. Randomized and double-blinded pilot clinical study of the safety and anti-diabetic efficacy of the Rauvolfia-Citrus tea, as used in Nigerian traditional medicine. J Ethnopharmacol. 2011;133(2):402-411. doi:10.1016/j.jep.2010.10.013	Not related to Oral CHM
417<br>5	Cai H, Liu F, Zuo P, et al. Practical Application of Antidiabetic Efficacy of Lycium barbarum Polysaccharide in Patients with Type 2 Diabetes. Med Chem. 2015;11(4):383-390. doi:10.2174/1573406410666141110153858	Not related to Oral CHM
417<br>6	Cai X, Wang Y, Li Z, Zhang Y, Wang D, Yan X. Chinese herbal medicine for patients with atrial fibrillation: protocol for a systematic review and meta-analysis. Medicine (Baltimore). 2017;96(50):e9228. doi:10.1097/MD.0000000000009228	Not related to Oral CHM
417<br>7	Cai P, Wu Z, Huang W, Niu Q, Zhu Y, Yin D. Suoquan pill for the treatment of diabetic nephropathy: A protocol for systematic review and meta-analysis. Medicine (Baltimore). 2021;100(17):e25613. doi:10.1097/MD.0000000000025613	Not related to Oral CHM
417<br>8	Cai J, Du J, Huang JS. Zhongguo Zhong Xi Yi Jie He Za Zhi. 2003;23(9):664-667.	Not related to Oral CHM
417<br>9	Butt MS, Sultan MT, Aziz M, et al. Persimmon (Diospyros kaki) fruit: hidden phytochemicals and health claims. EXCLI J. 2015;14:542-561. Published 2015 May 4. doi:10.17179/excli2015-159	Not related to Oral CHM
418<br>0	Butnariu M, Quispe C, Herrera-Bravo J, et al. The Pharmacological Activities of Crocus sativus L.: A Review Based on the Mechanisms and Therapeutic Opportunities of its Phytoconstituents. Oxid Med Cell Longev. 2022;2022:8214821. Published 2022 Feb 14. doi:10.1155/2022/8214821	Not related to Oral CHM
418<br>1	Brophy S, Brunt H, Davies H, Mannan S, Williams R. Interventions for latent autoimmune diabetes (LADA) in adults. Cochrane Database Syst Rev. 2007;(3):CD006165. Published 2007 Jul 18. doi:10.1002/14651858.CD006165.pub2	Not related to Oral CHM
418<br>2	BRITTO, J., DURAIRAJ, K., SUNDRAM, S., KUMARY, U., & PRAVEEN, U. (2021). Exploratory Research on the Effectiveness of Lannea coromandelica in Streptozotocin Induced Diabetic Model: A Protocol for Systematic Review. Journal of Clinical & Diagnostic Research, 15(2).	Not related to Oral CHM
418<br>3	Briskey D, Malfa GA, Rao A. Effectiveness of "Moro" Blood Orange Citrus sinensis Osbeck (Rutaceae) Standardized Extract on Weight Loss in Overweight but Otherwise Healthy Men and Women-A Randomized Double-Blind Placebo-Controlled Study. Nutrients. 2022;14(3):427. Published 2022 Jan 18. doi:10.3390/nu14030427	Not related to Oral CHM
418<br>4	Brinkley TE, Lovato JF, Arnold AM, et al. Effect of Ginkgo biloba on blood pressure and incidence of hypertension in elderly men and women. Am J Hypertens. 2010;23(5):528-533. doi:10.1038/ajh.2010.14	Not related to Oral CHM
418<br>5	Braun LR, Fisk WA, Lev-Tov H, Kirsner RS, Isseroff RR. Diabetic foot ulcer: an evidence-based treatment update. Am J Clin Dermatol. 2014;15(3):267-281. doi:10.1007/s40257-014-0081-9	Not related to Oral CHM

418 6	Bradley R, Sherman KJ, Catz S, et al. Adjunctive naturopathic care for type 2 diabetes: patient-reported and clinical outcomes after one year. BMC Complement Altern Med. 2012;12:44. Published 2012 Apr 18. doi:10.1186/1472-6882-12-44	Not related to Oral CHM
418 7	Bradley R, Sherman KJ, Catz S, et al. Adjunctive naturopathic care for type 2 diabetes: patient-reported and clinical outcomes after one year. BMC Complement Altern Med. 2012;12:44. Published 2012 Apr 18. doi:10.1186/1472-6882-12-44	Not related to Oral CHM
418 8	Boyd A, Bleakley C, Hurley DA, et al. Herbal medicinal products or preparations for neuropathic pain. Cochrane Database Syst Rev. 2019;4(4):CD010528. Published 2019 Apr 2. doi:10.1002/14651858.CD010528.pub4	Not related to Oral CHM
418 9	Bouyahya A, El Omari N, Elmenyiy N, et al. Ethnomedicinal use, phytochemistry, pharmacology, and toxicology of Ajuga iva (L.,) schreb. J Ethnopharmacol. 2020;258:112875. doi:10.1016/j.jep.2020.112875	Not related to Oral CHM
419 0	Bouyahya A, Chamkhi I, Guaouguaou FE, et al. Ethnomedicinal use, phytochemistry, pharmacology, and food benefits of Thymus capitatus. J Ethnopharmacol. 2020;259:112925. doi:10.1016/j.jep.2020.112925	Not related to Oral CHM
419 1	Bouyahya A, Chamkhi I, Guaouguaou FE, et al. Ethnomedicinal use, phytochemistry, pharmacology, and food benefits of Thymus capitatus. J Ethnopharmacol. 2020;259:112925. doi:10.1016/j.jep.2020.112925	Not related to Oral CHM
419 2	Borrelli F, Capasso R, Izzo AA. Garlic (Allium sativum L.): adverse effects and drug interactions in humans. Mol Nutr Food Res. 2007;51(11):1386-1397. doi:10.1002/mnfr.200700072	Not related to Oral CHM
419 3	Bora KS, Sharma A. Phytochemical and pharmacological potential of Medicago sativa: a review. Pharm Biol. 2011;49(2):211-220. doi:10.3109/13880209.2010.504732	Not related to Oral CHM
419 4	Peter, B. B., Patrick, M. F., & Pina, L. (2008). Potential health benefits of garlic (Allium sativum): A Narrative Review. Journal of Complementary and Integrative Medicine, 5(1), 1-26.	Not related to Oral CHM
419 5	Bola C, Bartlett H, Eperjesi F. Resveratrol and the eye: activity and molecular mechanisms. Graefes Arch Clin Exp Ophthalmol. 2014;252(5):699-713. doi:10.1007/s00417-014-2604-8	Not related to Oral CHM
419 6	Boix-Castejón M , Herranz-López M , Pérez Gago A , et al. Hibiscus and lemon verbena polyphenols modulate appetite-related biomarkers in overweight subjects: a randomized controlled trial [published correction appears in Food Funct. 2018 Jul 17;9(7):4037. doi: 10.1039/c8fo90028k.]. Food Funct. 2018;9(6):3173-3184. doi:10.1039/c8fo00367j	Not related to Oral CHM
419 7	Blom WA, Abrahamse SL, Bradford R, et al. Effects of 15-d repeated consumption of Hoodia gordonii purified extract on safety, ad libitum energy intake, and body weight in healthy, overweight women: a randomized controlled trial. Am J Clin Nutr. 2011;94(5):1171-1181. doi:10.3945/ajcn.111.020321	Not related to Oral CHM

4198	Bitler, C. M., Matt, K., Irving, M., Hook, G., Yusen, J., Eagar, F., ... & Crea, R. (2007). Olive extract supplement decreases pain and improves daily activities in adults with osteoarthritis and decreases plasma homocysteine in those with rheumatoid arthritis. Nutrition Research, 27(8), 470-477.	Not related to Oral CHM
4199	Birketvedt GS, Travis A, Langbakk B, Florholmen JR. Dietary supplementation with bean extract improves lipid profile in overweight and obese subjects. Nutrition. 2002;18(9):729-733. doi:10.1016/s0899-9007(02)00831-6	Not related to Oral CHM
4200	Bian F, Zhao P, Zhang Z. Zhongguo Zhong Xi Yi Jie He Za Zhi. 2000;20(5):335-337.	Not related to Oral CHM
4201	BHIDE, S., BHOJNE, S., KANASE, H., RAUT, S., & DETHE, P. (2019). Nephroprotective Effect of Ethanolic Extract of Flax Seed. Journal of Clinical & Diagnostic Research, 13(7).	Not related to Oral CHM
4202	Bhatti, I., Inayat, S., Uzair, B., Menaa, F., Bakhsh, S., Khan, H., ... & Khan, B. A. (2016). Effects of Nigella sativa (Kalonji) and honey on lipid profile of hyper lipidemic smokers. Ind J Pharmaceut Educ Res, 50(3), 376e84.	Not related to Oral CHM
4203	Bell SJ, Van Ausdal W, Grochoski G. Do dietary supplements help promote weight loss?. J Diet Suppl. 2009;6(1):33-53. doi:10.1080/19390210802687171	Not related to Oral CHM
4204	Bedekar A, Shah K, Koffas M. Natural products for type II diabetes treatment. Adv Appl Microbiol. 2010;71:21-73. doi:10.1016/S0065-2164(10)71002-9	Not related to Oral CHM
4205	Bayat, A., Heydaribeni, M., Feizi, A., Iraj, B., Ghiasvand, R., & Askari, G. (2014). The effect of pumpkin and probiotic yogurt consumption separately or/and simultaneously on type II diabetes. Journal of Isfahan Medical School, 32(283), 580-589.	Not related to Oral CHM
4206	Basu A, Sanchez K, Leyva MJ, et al. Green tea supplementation affects body weight, lipids, and lipid peroxidation in obese subjects with metabolic syndrome. J Am Coll Nutr. 2010;29(1):31-40. doi:10.1080/07315724.2010.10719814	Not related to Oral CHM
4207	Barnes J. Quality, efficacy and safety of complementary medicines: fashions, facts and the future. Part II: Efficacy and safety. Br J Clin Pharmacol. 2003;55(4):331-340. doi:10.1046/j.1365-2125.2003.01811.x	Not related to Oral CHM
4208	Barbhuiya PA, Laskar AM, Mazumdar H, et al. Ethnomedicinal Practices and Traditional Medicinal Plants of Barak Valley, Assam: a systematic review. J Pharmacopuncture. 2022;25(3):149-185. doi:10.3831/KPI.2022.25.3.	Not related to Oral CHM
4209	Baranova, V. S., Rusina, I. F., Guseva, D. A., Prozorovskaya, N. N., Ipatova, O. M., & Kasaikina, O. T. (2013). The antiradical activity of plant extracts and their health-improving and prophylactic combinations with a phosholipid complex. Biochemistry (Moscow) Supplement Series B: Biomedical Chemistry, 7, 165-174.	Not related to Oral CHM

4210	Bao JL, Xinyuan-Gao, Han YB, Zhang K, Liu L. Efficacy and safety of Chinese medicine for obstructive sleep apnea: A protocol for systematic review and meta-analysis. Medicine (Baltimore). 2021;100(3):e23903. doi:10.1097/MD.0000000000023903	Not related to Oral CHM
4211	Bao HL, Ye SH, Lou SX, Lu XW, Zhou XF. Zhongguo Zhong Yao Za Zhi. 2014;39(6):1128-1131.	Not related to Oral CHM
4212	Banach M, Bruckert E, Descamps OS, et al. The role of red yeast rice (RYR) supplementation in plasma cholesterol control: A review and expert opinion. Atheroscler Suppl. 2019;39:e1-e8. doi:10.1016/j.atherosclerosissup.2019.08.023	Not related to Oral CHM
4213	Baldi, A., Choudhary, N., & Kumar, S. (2013). Nutraceuticals as therapeutic agents for holistic treatment of diabetes. International Journal of Green Pharmacy (IJGP), 7(4).	Not related to Oral CHM
4214	Balaga, V. K. R., Pradhan, A., Thapa, R., Patel, N., Mishra, R., & Singla, N. (2023). Morin: a comprehensive review on its versatile biological activity and associated therapeutic potential in treating cancers. Pharmacological Research-Modern Chinese Medicine, 7, 100264.	Not related to Oral CHM
4215	Bakhshi, M., Rafraf, M., Haghravan, S., Jafarabadi, M. A., & Jafari, A. (2019). The effect of ginger supplementation and high intensity interval training (HIIT) on anthropometric indices and serum level of irisin in obese men.	Not related to Oral CHM
4216	Bahrami, G., Naseri, R., Khosravifar, M., Sajadimajd, S., Mohammadi, B., Heydarpour, F., & Cheraghialiakbari, S. (2022). The Safety and Effectiveness of a Polysaccharide Extracted from Rosa Canina in Patients with NAFLD: A Randomized Trial. Clinical Diabetology, 11(4), 239-244.	Not related to Oral CHM
4217	Bahar, A., Ebrahimi, H., Nafchi, A. M., & Valizade, B. (2021). Effect of bulk bread formulated with portulaca oleracea on liver enzymes, and oxidative stress in patients with type 2 diabetes and hyperlipidemia: A parallel randomized controlled trial. Koomesh, 24(1), 139-146.	Not related to Oral CHM
4218	Bahadoran Z, Mirmiran P, Hosseinpanah F, Hedayati M, Hosseinpour-Niazi S, Azizi F. Broccoli sprouts reduce oxidative stress in type 2 diabetes: a randomized double-blind clinical trial. Eur J Clin Nutr. 2011;65(8):972-977. doi:10.1038/ejcn.2011.59	Not related to Oral CHM
4219	Baek J, Kim J, Shin S, Cho C. The efficacy and safety of yukmijihwang-hwan (Liuweidihuang-wan) for type 2 diabetes mellitus without complications: A protocol for systematic review and meta-analysis. Medicine (Baltimore). 2022;101(11):e29087. doi:10.1097/MD.0000000000029087	Not related to Oral CHM
4220	Azizah N, Halimah E, Puspitasari IM, Hasanah AN. Simultaneous Use of Herbal Medicines and Antihypertensive Drugs Among Hypertensive Patients in the Community: A Review. J Multidiscip Healthc. 2021;14:259-270. Published 2021 Feb 3. doi:10.2147/JMDH.S289156	Not related to Oral CHM

4221	Azimi, M., Deldar, M., Moayeri, A., & Sayehmiri, K. (2022). Effects of Dill Extract on Blood Lipid Levels (TC, TG, LDL and HDL): A Systematic Review and Meta-Analysis. Journal of Advances in Medical and Biomedical Research, 30(141), 306-313.	Not related to Oral CHM
4222	Azam, M., Saerang, C. O., Rahayu, S. R., Indrawati, F., Budiono, I., Fibriana, A. I., ... & Lesmana, R. (2016). A Doubled-Blind, Crossover-RCT in T2DM for Evaluating Hypoglycemic effect of P. indicus, M. charantia, P. vulgaris and A. paniculata in Central Java.聽Journal of Natural Remedies, 108-114.	Not related to Oral CHM
4223	Ayinu-Nulahou, W. Y., Bu, Q., & Zhao, Y. (2019). Clinical efficacy of calcium dobesilate dispersible tablets combined withMingmu dihuang pills in treatment of NPDR. Int Eye Sci, 19(6), 992-6.	Not related to Oral CHM
4224	Ayati Z, Ramezani M, Amiri MS, et al. Ethnobotany, Phytochemistry and Traditional Uses of Curcuma spp. and Pharmacological Profile of Two Important Species (C. longa and C. zedoaria): A Review. Curr Pharm Des. 2019;25(8):871-935. doi:10.2174/1381612825666190402163940	Not related to Oral CHM
4225	Awasthi A, Singh SK, Kumar B, et al. Treatment Strategies Against Diabetic Foot Ulcer: Success so Far and the Road Ahead. Curr Diabetes Rev. 2021;17(4):421-436. doi:10.2174/1573399816999201102125537	Not related to Oral CHM
4226	Atkin M, Laight D, Cummings MH. The effects of garlic extract upon endothelial function, vascular inflammation, oxidative stress and insulin resistance in adults with type 2 diabetes at high cardiovascular risk. A pilot double blind randomized placebo controlled trial. J Diabetes Complications. 2016;30(4):723-727. doi:10.1016/j.jdiacomp.2016.01.003	Not related to Oral CHM
4227	Ataabadi, G., Shahinfar, N., Mardani, G., & Gholami, M. (2019). Effect of Otostegia persica extract on blood glucose in patients with type II diabetes. Journal of Pharmaceutical Negative Results, 10(1).	Not related to Oral CHM
4228	Astell KJ, Mathai ML, Su XQ. Plant extracts with appetite suppressing properties for body weight control: a systematic review of double blind randomized controlled clinical trials. Complement Ther Med. 2013;21(4):407-416. doi:10.1016/j.ctim.2013.05.007	Not related to Oral CHM
4229	Astell KJ, Mathai ML, McAinch AJ, Stathis CG, Su XQ. A pilot study investigating the effect of Caralluma fimbriata extract on the risk factors of metabolic syndrome in overweight and obese subjects: a randomised controlled clinical trial. Complement Ther Med. 2013;21(3):180-189. doi:10.1016/j.ctim.2013.01.004	Not related to Oral CHM
4230	Aslani, Z., Alipopr, B., Mirmiran, P., Bahadoran, Z., & Abbassalizade, M. (2015). Effect of lentil sprouts on lipid profile and blood fasting glucose in overweight and obese patients with type 2 diabetes.	Not related to Oral CHM
4231	Asgary S, Kelishadi R, Rafieian-Kopaei M, Najafi S, Najafi M, Sahebkar A. Investigation of the lipid-modifying and antiinflammatory effects of Cornus mas L. supplementation on dyslipidemic children and adolescents. Pediatr Cardiol. 2013;34(7):1729-1735. doi:10.1007/s00246-013-0693-5	Not related to Oral CHM

4232	Asai A, Nakagawa K, Higuchi O, et al. Effect of mulberry leaf extract with enriched 1-deoxynojirimycin content on postprandial glycemic control in subjects with impaired glucose metabolism. J Diabetes Investig. 2011;2(4):318-323. doi:10.1111/j.2040-1124.2011.00101.x	Not related to Oral CHM
4233	Arsyad, A. S., Nurrochmad, A., & Fakhrudin, N. (2022). Phytochemistry, traditional uses, and pharmacological activities of Ficus elastica Roxb. ex Hornem: A review. Journal of Herbmed Pharmacology, 12(1), 41-53.	Not related to Oral CHM
4234	Ariyanto, E. F., Multom, N. O., Berbudi, A., Rohmawaty, E., & Sujatmiko, B. (2021). Efficacy and Mechanism of Action of Aloe Vera, Cinnamomum Zeylanicum, Curcuma Longa, Garcinia Cambogia, and Garcinia Mangostana Extracts in Lowering Body Weight in Obesity: A Literature Review. Biomedical and Pharmacology Journal, 14(4), 1791-1797.	Not related to Oral CHM
4235	Ardiana M, Pikir BS, Santoso A, Hermawan HO, Al-Farabi MJ. Effect of Nigella sativa supplementation on oxidative stress and antioxidant parameters: a meta-analysis of randomized controlled trials. Sci World J. 2020;2020:2390706. doi:10.1155/2020/2390706	Not related to Oral CHM
4236	Arbain D, Sinaga LMR, Taher M, Susanti D, Zakaria ZA, Khotib J. Traditional uses, phytochemistry and biological activities of Alocasia species: a systematic review. Front Pharmacol. 2022;13:849704. doi:10.3389/fphar.2022.849704	Not related to Oral CHM
4237	Ansari-Mohseni N, Ghorani-Azam A, Mohajeri SA. Therapeutic effects of herbal medicines in different types of retinopathies: a systematic review. Avicenna J Phytomed. 2023;13(2):118-142. doi:10.22038/AJP.2022.62423.2977	Not related to Oral CHM
4238	Ansari, S., Siddiqui, M. A., Malhotra, S., & Maaz, M. (2018). Antiviral efficacy of qust (Saussurea lappa) and afsanteen (Artemisia absinthium) for chronic Hepatitis B: A prospective single- arm pilot clinical trial. Pharmacognosy Research, 10(3).	Not related to Oral CHM
4239	Angulo-Bejarano PI, Gómez-García MR, Valverde ME, Paredes-López O. Nopal (Opuntia spp.) and its effects on metabolic syndrome: new insights for the use of a millenary plant. Curr Pharm Des. 2019;25(32):3457-3477. doi:10.2174/1381612825666191010171819	Not related to Oral CHM
4240	Anderson ZL, Scopelliti EM, Trompeter JM, Havrda DE. Management of prediabetes: a comparison of the treatment approaches utilized by a family practice clinic and an internal medicine/endocrinology practice. J Pharm Pract. 2015;28(1):86-92. doi:10.1177/0897190013514089	Not related to Oral CHM
4241	Andallu B, Suryakantham V, Lakshmi Srikanthi B, Reddy GK. Effect of mulberry (Morus indica L.) therapy on plasma and erythrocyte membrane lipids in patients with type 2 diabetes. Clin Chim Acta. 2001;314(1-2):47-53. doi:10.1016/S0009-8981(01)00632-5	Not related to Oral CHM

4242	An, X. F., Zhao, Y., & Yu, J. Y. (2016). Treatment of Early Diabetic Retinopathy by Liuwei Dihuang Pill Combined Ginkao Leaf Tablet. Zhongguo Zhong xi yi jie he za zhi Zhongguo Zhongxiyi Jiehe Zazhi= Chinese Journal of Integrated Traditional and Western Medicine, 36(6), 674-677.	Not related to Oral CHM
4243	An X, Xu Y, Gui D. Combination of Astragalus membranaceus and Panax notoginseng as main components in the treatment of diabetic nephropathy: a systematic review and meta-analysis. Evid Based Complement Alternat Med. 2023;2023:2945234. doi:10.1155/2023/2945234	Not related to Oral CHM
4244	An X, Jin D, Duan L, Zhao S, Zhou R, Lian F, Tong X. Direct and indirect therapeutic effect of traditional Chinese medicine as an add-on for non-proliferative diabetic retinopathy: a systematic review and meta-analysis. Chin Med. 2020;15:99. doi:10.1186/s13020-020-00380-4	Not related to Oral CHM
4245	Amirkhanloo F, Esmaeilzadeh S, Mirabi P, Abedini A, Amiri M, Saghebi R, Golsorkhtabaramiri M. Comparison of Foeniculum vulgare versus metformin on insulin resistance and anthropometric indices of women with polycystic ovary, an open-label controlled trial study. Obes Med. 2022;31:100401. doi:10.1016/j.obmed.2022.100401	Not related to Oral CHM
4246	Amin F, Islam N, Anila N, Gilani AH. Clinical efficacy of the co-administration of turmeric and black seeds (Kalongi) in metabolic syndrome: a double-blind randomized controlled trial - TAK-MetS trial. Complement Ther Med. 2015;23(2):165-174. doi:10.1016/j.ctim.2015.01.008	Not related to Oral CHM
4247	Thomas M, Hamdan M, Hailes S, Walker M. Top ten natural preparations for the treatment of diabetic foot disorders. Wounds UK. 2010;6(4):20-24	Not related to Oral CHM
4248	Alzahrani AS, Price MJ, Greenfield SM, Paudyal V. Global prevalence and types of complementary and alternative medicines use amongst adults with diabetes: systematic review and meta-analysis. Eur J Clin Pharmacol. 2021;77(9):1259-1274. doi:10.1007/s00228-021-03097-x	Not related to Oral CHM
4249	Altschuler JA, Casella SJ, MacKenzie TA, Curtis KM. The effect of cinnamon on A1C among adolescents with type 1 diabetes. Diabetes Care. 2007;30(4):813-816. doi:10.2337/dc06-1871	Not related to Oral CHM
4250	Alqahtani AS, Ullah R, Shahat AA. Bioactive Constituents and Toxicological Evaluation of Selected Antidiabetic Medicinal Plants of Saudi Arabia. Evid Based Complement Alternat Med. 2022;2022:7123521. Published 2022 Jan 17. doi:10.1155/2022/7123521	Not related to Oral CHM
4251	Allen RW, Schwartzman E, Baker WL, Coleman CI, Phung OJ. Cinnamon use in type 2 diabetes: an updated systematic review and meta-analysis. Ann Fam Med. 2013;11(5):452-459. doi:10.1370/afm.1517	Not related to Oral CHM
4252	Alder R, Lookinland S, Berry JA, Williams M. A systematic review of the effectiveness of garlic as an anti-hyperlipidemic agent. J Am Acad Nurse Pract. 2003;15(3):120-129. doi:10.1111/j.1745-7599.2003.tb00268.x	Not related to Oral CHM
4253	Alam MA, Uddin R, Subhan N, Rahman MM, Jain P, Reza HM. Beneficial role of bitter melon supplementation in obesity and related complications in metabolic syndrome. J Lipids. 2015;2015:496169. doi:10.1155/2015/496169	Not related to Oral CHM

4254	Al-Snafi, A. E., & Alfuraiji, N. (2023). Medicinal Plants with Anti-Obesity Effects: A Special Emphasis on Their Mode of Action. Bahrain Medical Bulletin, 45(2).	Not related to Oral CHM
4255	Akubue PI, Mittal GC. Clinical evaluation of a traditional herbal practice in Nigeria: a preliminary report. J Ethnopharmacol. 1982;6(3):355-359. doi:10.1016/0378-8741(82)90056-3	Not related to Oral CHM
4256	Akilen R, Tsiami A, Devendra D, Robinson N. Glycated haemoglobin and blood pressure-lowering effect of cinnamon in multi-ethnic Type 2 diabetic patients in the UK: a randomized, placebo-controlled, double-blind clinical trial. Diabet Med. 2010;27(10):1159-1167. doi:10.1111/j.1464-5491.2010.03079.x	Not related to Oral CHM
4257	Ajebli M, Eddouks M. Phytotherapy of Hypertension: An Updated Overview. Endocr Metab Immune Disord Drug Targets. 2020;20(6):812-839. doi:10.2174/1871530320666191227104648	Not related to Oral CHM
4258	Ain Q, Nawab M, Ahmad T, Kazmi MH, Naikodi MAR. Evaluating the safety and efficacy of a polyherbal Unani formulation in dyslipidaemia-a prospective randomized controlled trial. J Ethnopharmacol. 2022;289:115036. doi:10.1016/j.jep.2022.115036	Not related to Oral CHM
4259	Ahmed S, Ding X, Sharma A. Exploring scientific validation of Triphala Rasayana in ayurveda as a source of rejuvenation for contemporary healthcare: An update. J Ethnopharmacol. 2021;273:113829. doi:10.1016/j.jep.2021.113829	Not related to Oral CHM
4260	Ahmadi N, Nabavi V, Hajsadeghi F, et al. Aged garlic extract with supplement is associated with increase in brown adipose, decrease in white adipose tissue and predict lack of progression in coronary atherosclerosis. Int J Cardiol. 2013;168(3):2310-2314. doi:10.1016/j.ijcard.2013.01.182	Not related to Oral CHM
4261	Abdul Hafeez, A. H., Ming LongChiau, M. L., & Yaman Walid Kassab, Y. W. K. (2016). A review on safety and efficacy of products containing Longifolia.	Not related to Oral CHM
4262	Ahmad Alobaidi AH. Effect of Nigella sativa and Allium sativum coadminstered with simvastatin in dyslipidemia patients: a prospective, randomized, double-blind trial. Antiinflamm Antiallergy Agents Med Chem. 2014;13(1):68-74. doi:10.2174/18715230113129990013	Not related to Oral CHM
4263	Aguirre-Romero AB, Galeano-Valle F, Conde-Montero E, Velázquez-Tarjuelo D, de-la-Cueva-Dobao P. Efficacy and safety of a rosehip seed oil extract in the prevention and treatment of skin lesions in the hands of patients with type 1 diabetes mellitus caused by finger prick blood glucose monitoring; a randomized, open-label, controlled clinical trial. Eficacia y seguridad del aceite de rosa mosqueta en las lesiones de los dedos provocadas por las punciones capilares para el control glucémico en niños con diabetes tipo 1; un ensayo clínico aleatorizado, abierto, controlado. Endocrinol Diabetes Nutr (Engl Ed). 2020;67(3):186-193. doi:10.1016/j.endinu.2019.04.008	Not related to Oral CHM

4264	Agbor GA, Kuate D, Oben JE. Medicinal plants can be good source of antioxidants: case study in Cameroon. Pak J Biol Sci. 2007;10(4):537-544. doi:10.3923/pjbs.2007.537.544	Not related to Oral CHM
4265	Adiguna SP, Panggabean JA, Atikana A, et al. Antiviral Activities of Andrographolide and Its Derivatives: Mechanism of Action and Delivery System. Pharmaceuticals (Basel). 2021;14(11):1102. Published 2021 Oct 28. doi:10.3390/ph14111102	Not related to Oral CHM
4266	Adel Mehraban MS, Tabatabaei-Malazy O, Rahimi R, Daniali M, Khashayar P, Larijani B. Targeting dyslipidemia by herbal medicines: A systematic review of meta-analyses. J Ethnopharmacol. 2021;280:114407. doi:10.1016/j.jep.2021.114407	Not related to Oral CHM
4267	Abolghasemi J, Farboodniay Jahromi MA, Hossein Sharifi M, et al. Effects of Zataria oxymel on obesity, insulin resistance and lipid profile: A randomized, controlled, triple-blind trial. J Integr Med. 2020;18(5):401-408. doi:10.1016/j.joim.2020.06.003	Not related to Oral CHM
4268	Abdulazeez MA, Muhammad SA, Saidu Y, et al. A systematic review with meta-analysis on the antihypertensive efficacy of Nigerian medicinal plants. J Ethnopharmacol. 2021;279:114342. doi:10.1016/j.jep.2021.114342	Not related to Oral CHM
4269	Abdollahi N, Nadjarzadeh A, Salehi-Abargouei A, et al. The effect of Nigella sativa on TAC and MDA in obese and overweight women: secondary analysis of a crossover, double blind, randomized clinical trial. J Diabetes Metab Disord. 2022;21(1):171-179. Published 2022 Feb 7. doi:10.1007/s40200-021-00954-5	Not related to Oral CHM
4270	Abdoli, M., Dabaghian, F. H., Goushegir, A., Shirazi, M. T., Nakhjavani, M., Shojaii, A., ... & Mahlooji, K. (2017). Anti-hyperglycemic effect of aqueous extract of Juglans regia L. leaf (walnut leaf) on type 2 diabetic patients: a randomized controlled trial. Advances in integrative medicine, 4(3), 98-102.	Not related to Oral CHM
4271	Abd Aziz NA, Hasham R, Sarmidi MR, Suhaimi SH, Idris MKH. A review on extraction techniques and therapeutic value of polar bioactives from Asian medicinal herbs: Case study on Orthosiphon aristatus, Eurycoma longifolia and Andrographis paniculata. Saudi Pharm J. 2021;29(2):143-165. doi:10.1016/j.jsps.2020.12.016	Not related to Oral CHM
4272	周治忠.断肠草煎剂溻渍法治疗糖尿病足 34 例疗效观察[J].中医外治杂志,2012,21(06):18-19+69.	Not related to Oral CHM
4273	肖新娟.中药穴位贴敷治疗老年性高血压的效果及临床护理体会[J].黑龙江中医药,2024,53(02):181-182.	Not related to Oral CHM
4274	苗阳,赵文静,荆鲁,等.中西医结合治疗慢性心力衰竭的回顾性分析[J].中国中西医结合杂志,2008,(05):406-409.	Not related to Oral CHM
4275	刘云霞,杨媛.不典型低血糖症误诊 1 例分析[J].现代中医临床,2014,21(01):52-53.	Not related to Oral CHM
4276	刘为民,徐艳.中药膳食治疗 2 型糖尿病的探讨[J].中国社区医师(医学专业),2011,13(32):200.	Not related to Oral CHM

4277	林求诚,叶盈,方素钦,等.真菌降脂素治疗脂质代谢紊乱的临床研究[J].中国中西医结合杂志,1995,(05):281-283.	Not related to Oral CHM
4278	李松伟.减肥调脂胶囊治疗单纯性肥胖病(胃热湿阻型)的临床研究[J].河南中医学院学报,2006,(04):69.DOI:10.16368/j.issn.1674-8999.2006.04.054.	Not related to Oral CHM
4279	李军.辨体茶饮疗法对血糖正常高值人群糖调节异常状态的影响[J].内蒙古中医药,2019,38(03):81-82.DOI:10.16040/j.cnki.cn15-1101.2019.03.054.	Not related to Oral CHM
4280	李淳.格列美脲片致全身严重皮疹 1 例[J].实用医学杂志,2015,31(05):866.	Not related to Oral CHM
4281	黄莉吉,余江毅,朱博钰,等."芪葵颗粒"联合西医常规疗法治疗早期糖尿病肾病 31 例临床研究[J].江苏中医药,2017,49(04):29-31.	Not related to Oral CHM
4282	郭玉芝,侯海萍,陈伟娟,等.康复新液治疗糖尿病患者皮肤溃疡的疗效观察[J].当代护士(学术版),2010,(03):18-19.	Not related to Oral CHM
4283	丁青梅.活络通痹方足浴联合消渴痹症膏穴位贴敷治疗糖尿病周围神经病变患者的效果[J].中国民康医学,2021,33(16):60-62.	Not related to Oral CHM
4284	陈晓凡,黄少桐,刘红宁.高血压病的辨证施膳[J].中华中医药杂志,2015,30(12):4426-4430.	Not related to Oral CHM
4285	陈琦琦,李春玉,张彩元,等.不同干预措施对糖尿病足护理效果的网状Meta分析[J].延边大学医学学报,2020,43(01):14-20.DOI:10.16068/j.1000-1824.2020.01.004.	Not related to Oral CHM
4286	Abdi, I. (2021). Effects of Berberine, Cinnamon, and Fenugreek Seeds on Glucose and Lipid Metabolism in Prediabetes and Diabetes: An Assessment of the Quality of the Clinical Trials (Doctoral dissertation, University of Bridgeport).	Not related to Oral CHM
4287	Ai X, Yu P, Peng L, et al. Berberine: A Review of its Pharmacokinetics Properties and Therapeutic Potentials in Diverse Vascular Diseases. Front Pharmacol. 2021;12:762654. Published 2021 Nov 3. doi:10.3389/fphar.2021.762654	Not related to Oral CHM
4288	Arulselvan, P., Ghofar, H. A. A., Karthivashan, G., Halim, M. F. A., Ghafar, M. S. A., & Fakurazi, S. (2014). Antidiabetic therapeutics from natural source: A systematic review. Biomedicine & Preventive Nutrition, 4(4), 607-617.	Not related to Oral CHM
4289	Awasthi H, Nath R, Usman K, et al. Effects of a standardized Ayurvedic formulation on diabetes control in newly diagnosed Type-2 diabetics; a randomized active controlled clinical study. Complement Ther Med. 2015;23(4):555-561. doi:10.1016/j.ctim.2015.06.005	Not related to Oral CHM
4290	Barzkar F, Baradaran HR, Khamseh ME, Vesal Azad R, Koohpayehzadeh J, Moradi Y. Medicinal plants in the adjunctive treatment of patients with type-1 diabetes: a systematic review of randomized clinical trials. J Diabetes Metab Disord. 2020;19(2):1917-1929. Published 2020 Sep 22. doi:10.1007/s40200-020-00633-x	Not related to Oral CHM
4291	Blais JE, Huang X, Zhao JV. Overall and Sex-Specific Effect of Berberine for the Treatment of Dyslipidemia in Adults: A Systematic Review and Meta-Analysis of Randomized Placebo-Controlled Trials. Drugs. 2023;83(5):403-427. doi:10.1007/s40265-023-01841-4	Not related to Oral CHM

4292	Chen M, Li Y, Men L, et al. Efficacy and safety of Zhibitai in treating coronary heart disease patients with hyperlipemia: A protocol for systematic review and meta-analysis. Medicine (Baltimore). 2020;99(36):e21991. doi:10.1097/MD.0000000000021991	Not related to Oral CHM
4293	Chen P, Zhu ZZ, Lang JM, Wei A, Chen F. Zhongguo Zhong Xi Yi Jie He Za Zhi. 2004;24(7):585-588.	Not related to Oral CHM
4294	Chen SL, Liu XY, Xu WM, Mei WY, Chen XL. Clinical study of western medicine combined with Chinese medicine based on syndrome differentiation in the patients with polarized hypertension. Chin J Integr Med. 2012;18(10):746-751. doi:10.1007/s11655-012-1231-7	Not related to Oral CHM
4295	Chen, TJ, Jiang, ZY, Wang, XL, Wang, YJ and Shen, ZJ, 2015, Combination therapy of atorvastatin and Jiangzhi decoction for primary hyperlipidemia (Tan Zhuo Zu E Zheng): A stratified randomized controlled trial, Chinese Journal of Evidence-Based Medicine	Not related to Oral CHM
4296	Chen X, Cheng GS, Fan JM. Zhongguo Zhong Xi Yi Jie He Za Zhi. 2015;35(7):801-805.	Not related to Oral CHM
4297	Chen, X., Liu, Y., Xie, H., YU, B., & Xie, X. (2006). Effect of yanggan qinggan pinggan technique on the changes of blood pressure and angiotensin Ⅱ in patients with primary hypertension. Chinese Journal of Tissue Engineering Research, 178-180.	Not related to Oral CHM
4298	Chen, Y., Fu, D. Y., Chen, Y., He, Y. M., Fu, X. D., Xu, Y. Q., ... & Wang, W. J. (2013). Effects of Chinese herbal medicine Yiqi Huaju Formula on hypertensive patients with metabolic syndrome: a randomized, placebo-controlled trial. Journal of Integrative Medicine, 11(3), 184-194.	Not related to Oral CHM
4299	Chen Z, Shi Q, Tan L, Peng Y, Liu C, Zhang J. Traditional Chinese Patent Medicine for Primary Hypertension: A Bayesian Network Meta-Analysis. Evid Based Complement Alternat Med. 2020;2020:6701272. Published 2020 Apr 24. doi:10.1155/2020/6701272	Not related to Oral CHM
4300	Chen ZH, Xia CD, Wei ZX. Zhongguo Zhong Xi Yi Jie He Za Zhi. 2012;32(7):910-913.	Not related to Oral CHM
4301	Chen ZH, Xia CD, Wei ZX. Zhongguo Zhong Xi Yi Jie He Za Zhi. 2014;34(9):1042-1046.	Not related to Oral CHM
4302	Cheng J, Zheng J, Liu Y, Hao P. Efficacy of traditional Chinese medication Tangminling pill in Chinese patients with type 2 diabetes. Biosci Rep. 2019;39(4):BSR20181729. Published 2019 Apr 30. doi:10.1042/BSR20181729	Not related to Oral CHM
4303	Cheon C, Jang S, Park JS, et al. Euiiyin-tang in the treatment of obesity: study protocol for a randomised controlled trial. Trials. 2017;18(1):289. Published 2017 Jun 21. doi:10.1186/s13063-017-2039-8	Not related to Oral CHM
4304	Cheon C, Song YK, Ko SG. Efficacy and safety of Euiiyin-tang in Korean women with obesity: A randomized, double-blind, placebo-controlled, multicenter trial. Complement Ther Med. 2020;51:102423. doi:10.1016/j.ctim.2020.102423	Not related to Oral CHM

4305	Cho, Y. Y., Baek, N. I., Chung, H. G., Jeong, T. S., Lee, K. T., Jeon, S. M., ... & Choi, M. S. (2012). Randomized controlled trial of Sajabalssuk (Artemisia princeps Pampanini) to treat pre-diabetes. European Journal of Integrative Medicine, 4(3), e299-e308.	Not related to Oral CHM
4306	Deng ZY, Wang MJ, Fan YH, Liu M. Zhongguo Zhong Yao Za Zhi. 2020;45(1):188-195. doi:10.19540/j.cnki.cjcmm.20190401.502	Not related to Oral CHM
4307	Ding, G. A., Yu, G. H., Liang, S. C., Fan, C. H., Tong, Z. J., Liu, L. Q., ... & Liang, F. C. (2006). Jiawei lingguizhugan tang for obesity induced by psychoactive drugs. Chinese Journal of Clinical Rehabilitation, 10(43), 46-48.	Not related to Oral CHM
4308	Dong M, Han MX, Liang XL. Zhongguo Zhong Xi Yi Jie He Za Zhi. 2002;22(5):356-358.	Not related to Oral CHM
4309	Du, LF, Yang, J, Sun, SY, Wang, XC and Wu, XL, 2004, Effect of pinggan jiangya capsule on plasma endothelins and angiotensin II in patients with primary hypertension, Chinese Journal of Clinical Rehabilitation	Not related to Oral CHM
4310	DU WQ, Jia M, Wang M, et al. Zhongguo Zhong Yao Za Zhi. 2021;46(18):4601-4614. doi:10.19540/j.cnki.cjcmm.20210618.501	Not related to Oral CHM
4311	Duan JY, Liang X, Jia M, et al. Zhongguo Zhong Yao Za Zhi. 2021;46(12):2984-2994. doi:10.19540/j.cnki.cjcmm.20210324.501	Not related to Oral CHM
4312	Fan GJ, Luo GB, Qin ML. Zhongguo Zhong Xi Yi Jie He Za Zhi. 2004;24(4):317-320.	Not related to Oral CHM
4313	Fu L, Mao ZX, Wang J, Zhang J, Zheng TR, Wang SL. Zhong Xi Yi Jie He Xue Bao. 2009;7(6):509-513. doi:10.3736/jcim20090603	Not related to Oral CHM
4314	Gao H, Yang Y, Deng J, Liang J, Zhang W, Feng X. A systematic review and meta-analysis on the efficacy and safety of traditional Chinese patent medicine Jinqi Jiangtang Tablet in the treatment of type 2 diabetes. Complement Ther Med. 2019;47:102021. doi:10.1016/j.ctim.2019.01.016	Not related to Oral CHM
4315	Gao SR, Bu JH, Zhu LZ. Preliminary exploration on effect of Qilian decoction in intervention treatment of diabetes mellitus type 2 with insulin resistance and its influence on related inflammatory cytokines. Zhongguo Zhong Xi Yi Jie He Za Zhi. 2004;24(7):593-5.	Not related to Oral CHM
4316	Gao Y, Zhou H, Zhao H, et al. Clinical research of traditional Chinese medical intervention on impaired glucose tolerance. Am J Chin Med. 2013;41(1):21-32. doi:10.1142/S0192415X1350002X.	Not related to Oral CHM
4317	Ghafouri, A., Hosseini, S., Shidfar, S., Kamalinejad, M., AghaHosseini, F., Heydari, I., & Shidfar, F. (2020). The effect of Aqueous, Ethanolic extracts of Rheum ribeson insulin sensitivity, inflammation, oxidative stress in patients with type 2 diabetes mellitus: A Randomized, Double-Blind, Placebo-Controlled Trial. Journal of Herbal Medicine, 24, 100389.	Not related to Oral CHM

4318	Ghanadian M, Soltani R, Homayouni A, Khorvash F, Mohammadi Jouabadi S, Abdollahzadeh M. The effect of Plantago major hydroalcoholic extract on the healing of diabetic foot and pressure ulcers: a randomized open-label controlled clinical trial. Int J Low Extrem Wounds. 2024;23(3):475-481. doi:10.1177/15347346211070723.	Not related to Oral CHM
4319	Grant RW, Singer DE, Meigs JB. Medication adherence before an increase in antihypertensive therapy: a cohort study using pharmacy claims data. Clin Ther. 2005;27(6):773-81. doi:10.1016/j.clinthera.2005.06.004.	Not related to Oral CHM
4320	Grant SJ, Chang DHT, Liu J, Wong V, Kiat H, Bensoussan A. Chinese herbal medicine for impaired glucose tolerance: a randomized placebo controlled trial. BMC Complement Altern Med. 2013;13:104. doi:10.1186/1472-6882-13-104.	Not related to Oral CHM
4321	Greenway FL, Liu Z, Martin CK, et al. Safety and efficacy of NT, an herbal supplement, in treating human obesity. Int J Obes (Lond). 2006;30(12):1737-41. doi:10.1038/sj.ijo.0803343.	Not related to Oral CHM
4322	Gu Y, Zhang Y, Shi X, et al. Effect of traditional Chinese medicine berberine on type 2 diabetes based on comprehensive metabonomics. Talanta. 2010;81(3):766-72. doi:10.1016/j.talanta.2010.01.015.	Not related to Oral CHM
4323	Han K, Kwon O, Park HJ, Jung SY, Yang C, Son CG. Effect of Daesiho-tang on obesity with non-alcoholic fatty liver disease: a study protocol for a randomised, double-blind, placebo-controlled pilot trial. Trials. 2020;21(1):128. doi:10.1186/s13063-020-4068-y.	Not related to Oral CHM
4324	Hasani-Ranjbar S, Nayebi N, Moradi L, Mehri A, Larijani B, Abdollahi M. The efficacy and safety of herbal medicines used in the treatment of hyperlipidemia; a systematic review. Curr Pharm Des. 2010;16(26):2935-47. doi:10.2174/138161210793176464.	Not related to Oral CHM
4325	He C, Wang W, Li B, et al. Clinical research of Yiqi Sanju Formula in treating central obese men at high risk of metabolic syndrome. Zhong Xi Yi Jie He Xue Bao. 2007;5(3):263-7. doi:10.3736/jcim20070307.	Not related to Oral CHM
4326	Hu Z, Liu X, Yang M. Evidence and potential mechanisms of Jin-Gui Shen-Qi Wan as a treatment for type 2 diabetes mellitus: a systematic review and meta-analysis. Front Pharmacol. 2021;12:699932. doi:10.3389/fphar.2021.699932.	Not related to Oral CHM
4327	Huang Q, Ni HX, Shao G. Effect of Chinese herbal medicine for activating blood circulation to remove stasis on CD11b/CD18 expression in patients with diabetes mellitus type 2. Zhongguo Zhong Xi Yi Jie He Za Zhi. 2003;23(6):430-2.	Not related to Oral CHM
4328	Huang YH, Liu GH, Hsu TY, et al. Effective Dose of Rhizoma Coptidis Extract Granules for Type 2 Diabetes Treatment: A Hospital-Based Retrospective Cohort Study. Front Pharmacol. 2021;11:597703. Published 2021 Jan 25. doi:10.3389/fphar.2020.597703	Not related to Oral CHM

4329	Huang YQ, Yang QF, Wang H, Xu YS, Peng W, Jiang YH. Long-term clinical effect of Tangyiping Granules () on patients with impaired glucose tolerance. Chin J Integr Med. 2016;22(9):653-659. doi:10.1007/s11655-016-2512-3	Not related to Oral CHM
4330	Incandela L, Belcaro G, De Sanctis MT, et al. Total triterpenic fraction of Centella asiatica in the treatment of venous hypertension: a clinical, prospective, randomized trial using a combined microcirculatory model. Angiology. 2001;52 Suppl 2:S61-S67.	Not related to Oral CHM
4331	Ji ZC, Lin SS, Hu HY, Sheng XD, Yang FW, Wang XL. Zhongguo Zhong Yao Za Zhi. 2022;47(7):1955-1988. doi:10.19540/j.cnki.cjcmm.20211223.501	Not related to Oral CHM
4332	Li J, Qiang FU, Shidong W, et al. Effects of Shenlian formula on microbiota and inflammatory cytokines in adults with type 2 diabetes: a double-blind randomized clinical trial. J Tradit Chin Med. 2023;43(4):760-769. doi:10.19852/j.cnki.jtcm.20230608.003	Not related to Oral CHM
4333	Jiang X, Luo Y, Wang X, et al. Investigating the efficiency and tolerability of traditional Chinese formulas combined with antiarrhythmic agents for paroxysmal atrial fibrillation: A systematic review and Bayesian network meta-analysis. Phytomedicine. 2022;94:153832. doi:10.1016/j.phymed.2021.153832	Not related to Oral CHM
4334	Jin D, Tian J, Bao Q, et al. Does Adjuvant Treatment with Chinese Herbal Medicine to Antidiabetic Agents Have Additional Benefits in Patients with Type 2 Diabetes? A System Review and Meta-Analysis of Randomized Controlled Trials. Evid Based Complement Alternat Med. 2019;2019:1825750. Published 2019 May 6. doi:10.1155/2019/1825750	Not related to Oral CHM
4335	Kianbakht S, Khalighi-Sigaroodi F, Dabaghian FH. Improved glycemic control in patients with advanced type 2 diabetes mellitus taking Urtica dioica leaf extract: a randomized double-blind placebo-controlled clinical trial. Clin Lab. 2013;59(9-10):1071-1076. doi:10.7754/clin.lab.2012.121019	Not related to Oral CHM
4336	Kim J, Byun AR, Kwon S. Effect of Yeonryeonggobon-dan (YRGBD), an herbal complex, on glycemic control in patients with Type 2 diabetes mellitus: a case series. Complement Ther Med. 2014;22(6):1037-1040. doi:10.1016/j.ctim.2014.09.008	Not related to Oral CHM
4337	Lan YZ, Bai YL, Zhu XD. Integrated Traditional Chinese and Western medicine for ulcerative colitis with diabetes: A protocol for systematic review and meta-analysis. Medicine (Baltimore). 2021;100(4):e24444. doi:10.1097/MD.0000000000024444	Not related to Oral CHM
4338	Lee B, Kwon CY. Comparative Effectiveness of East Asian Traditional Medicine for Childhood Simple Obesity: A Systematic Review and Network Meta-Analysis. Int J Environ Res Public Health. 2022;19(20):12994. Published 2022 Oct 11. doi:10.3390/ijerph192012994	Not related to Oral CHM
4339	Lei T, Zhang XZ, He M. Zhongguo Zhong Xi Yi Jie He Za Zhi. 2006;26(6):511-513.	Not related to Oral CHM

4340	Lei Y, Tang Y, Huang L, He P. Systematic review and meta-analysis on efficacy of traditional Chinese medicine for atrial fibrillation through cluster analysis. Ann Palliat Med. 2021;10(8):8982-8990. doi:10.21037/apm-21-1785	Not related to Oral CHM
4341	Leng Y, Fu X, Qian L, et al. Efficacy, safety and therapeutic mechanism of Shen-Qi Xiao-Tan formula in the treatment of peripheral atherosclerosis in patients with type 2 diabetes mellitus: a randomized, double-blind, placebo-controlled trial protocol. BMC Complement Med Ther. 2022;22(1):337. Published 2022 Dec 22. doi:10.1186/s12906-022-03813-9	Not related to Oral CHM
4342	Leng Y, Zhou X, Xie Z, et al. Efficacy and safety of Chinese herbal medicine on blood glucose fluctuations in patients with type 2 diabetes mellitus: A protocol of systematic review and meta-analysis. Medicine (Baltimore). 2020;99(34):e21904. doi:10.1097/MD.0000000000021904	Not related to Oral CHM
4343	Li, H., Dong, S., Liu, Y., Tian, N., Yang, W., Dong, A., ... & Zhang, M. (2022). Efficacy and Safety of “Bushen Huoxue Therapy” ‐ Based Combined Chinese and Western Medicine Treatment for Diabetic Kidney Disease: an Updated Meta ‐ Analysis of 2105 Patients. Evidence ‐ Based Complementary and Alternative Medicine, 2022(1), 3710074.	Not related to Oral CHM
4344	Li HC, Yang YL, Yang XQ, et al. Zhongguo Zhong Xi Yi Jie He Za Zhi. 2016;36(2):172-178.	Not related to Oral CHM
4345	Li, H., Dong, S., Liu, Y., Tian, N., Yang, W., Dong, A., ... & Zhang, M. (2022). Efficacy and Safety of “Bushen Huoxue Therapy” ‐ Based Combined Chinese and Western Medicine Treatment for Diabetic Kidney Disease: an Updated Meta ‐ Analysis of 2105 Patients. Evidence ‐ Based Complementary and Alternative Medicine, 2022(1), 3710074.	Not related to Oral CHM
4346	Li J, Zhao S, Huang Y, Li C, Li B, Xu Y. Comparative efficacy and safety of traditional Chinese patent medicine for the treatment of type 2 diabetes mellitus: A Bayesian network meta-analysis protocol. Medicine (Baltimore). 2020;99(40):e22564. doi:10.1097/MD.0000000000022564	Not related to Oral CHM
4347	Li R, Wu L, Song N, Li W. TCM treatment of diabetic hearing loss--an audiological and rheological observation. J Tradit Chin Med. 2000;20(3):176-179.	Not related to Oral CHM
4348	Efficacy and safety of Shenqi Jiangtang Granules plus oral hypoglycemic agent in patients with type 2 diabetes mellitus: A protocol for systematic review and meta-analysis of 15 RCTs: Erratum. Medicine (Baltimore). 2021;100(9):e25092. doi:10.1097/MD.0000000000025092	Not related to Oral CHM
4349	Li WW, Guo H, Li HH, Wang LL, Fu H, Wang XM. Integration of traditional Chinese medicines and Western medicines for treating diabetes mellitus with coronary heart disease: a systematic review. J Altern Complement Med. 2013;19(6):492-500. doi:10.1089/acm.2012.0568	Not related to Oral CHM
4350	Li X, Lian FM, Guo D, et al. The rs1142345 in TPMT Affects the Therapeutic Effect of Traditional Hypoglycemic Herbs in Prediabetes. Evid Based Complement Alternat Med. 2013;2013:327629.	Not related to Oral CHM

	doi:10.1155/2013/327629	
4351	Li ZQ, Chang HJ, Sang WF. Zhong Yao Cai. 2013;36(1):163-166.	Not related to Oral CHM
4352	Lian F, Jin D, Bao Q, Zhao Y, Tong X. Effectiveness of traditional Chinese medicine Jinlida granules as an add-on therapy for type 2 diabetes: A system review and meta-analysis of randomized controlled trials. J Diabetes. 2019;11(7):540-551. doi:10.1111/1753-0407.12877	Not related to Oral CHM
4353	Lian F, Li G, Chen X, et al. Chinese herbal medicine Tianqi reduces progression from impaired glucose tolerance to diabetes: a double-blind, randomized, placebo-controlled, multicenter trial. J Clin Endocrinol Metab. 2014;99(2):648-655. doi:10.1210/jc.2013-3276	Not related to Oral CHM
4354	Lian F, Tian J, Chen X, et al. The Efficacy and Safety of Chinese Herbal Medicine Jinlida as Add-On Medication in Type 2 Diabetes Patients Ineffectively Managed by Metformin Monotherapy: A Double-Blind, Randomized, Placebo-Controlled, Multicenter Trial. PLoS One. 2015;10(6):e0130550. Published 2015 Jun 22. doi:10.1371/journal.pone.0130550	Not related to Oral CHM
4355	Liao WT, Su CC, Lee MT, et al. Integrative Chinese herbal medicine therapy reduced the risk of type 2 diabetes mellitus in patients with polycystic ovary syndrome: A nationwide matched cohort study. J Ethnopharmacol. 2019;243:112091. doi:10.1016/j.jep.2019.112091	Not related to Oral CHM
4356	Liu, D., Lin, H., Wang, X., Zhou, W., Chen, G., Guo, W., ... & Xiang, C. (2006). Influence of removing blood stasis and tonifying liver and kidney method on quality of life in patients with essential hypertension: randomized controlled observation. Chin J Clin Rehabil, 10, 9-12.	Not related to Oral CHM
4357	Liu H, Zhou JF. [Xianbai buyang Huanwu decoction used for treating hypertension with kidney qi deficiency and blood stasis]. Zhongguo Zhong Xi Yi Jie He Za Zhi. 1993;13(12):714‑717.	Not related to Oral CHM
4358	Liu M, Liu Z, Xu B, Zhang W, Cai J. Review of systematic reviews and meta-analyses investigating Traditional Chinese Medicine treatment for type 2 diabetes mellitus. J Tradit Chin Med. 2016;36(5):555‑563. doi:10.1016/S0254-6272(16)30074-7.	Not related to Oral CHM
4359	Liu S, Li A, Jiang B, et al. Comparison of efficacy and safety of traditional Chinese patent medicines for diabetic nephropathy: A protocol for Bayesian network meta-analysis. Medicine (Baltimore). 2022;101(19):e29152. doi:10.1097/MD.0000000000029152.	Not related to Oral CHM
4360	Liu XD, Fu J, Feng MZ, Zhang ZH. [Effect of Jingui Shenqi pill combined with nifedipine for the treatment of elderly hypertensive patients with spleen-kidney Yang deficiency syndrome]. Zhongguo Zhong Yao Za Zhi. 2015;40(24):4908‑4913.	Not related to Oral CHM

4361	Liu Y, Liang S, Bu P, et al. Radix Puerariae rebalances vasomotor factors and improves left ventricular diastolic dysfunction in patients with essential hypertension. Exp Ther Med. 2020;20(2):705‑713. doi:10.3892/etm.2020.8746.	Not related to Oral CHM
4362	Liu YL, Dong SL. [Dan Zhi Xiao Yao Powders in the treatment of patients with hypertension complicated with depression]. Zhongguo Zhong Xi Yi Jie He Za Zhi. 2008;28(3):280‑281.	Not related to Oral CHM
4363	Liu ZL, Liu JP, Zhang AL, et al. Chinese herbal medicines for hypercholesterolemia. Cochrane Database Syst Rev. 2011;(7):CD008305. doi:10.1002/14651858.CD008305.pub2.	Not related to Oral CHM
4364	Liu ZL, Wu SX, Gao GD. [Clinical assessment on treatment of hyperlipidemia with Pushen capsule]. Zhongguo Zhong Xi Yi Jie He Za Zhi. 2004;24(3):227‑229.	Not related to Oral CHM
4365	Long LZ, Chu JF, Qu H, et al. Effects of Qingda granule on patients with grade 1 hypertension at low-medium risk: study protocol for a randomized, controlled, double-blind clinical trial. Trials. 2023;24(1):1. doi:10.1186/s13063-022-07006-0.	Not related to Oral CHM
4366	Ma H, He K, Zhu J, Li X, Ye X. The anti-hyperglycemia effects of Rhizoma Coptidis alkaloids: A systematic review of modern pharmacological studies of the traditional herbal medicine. Fitoterapia. 2019;134:210‑220. doi:10.1016/j.fitote.2019.03.003.	Not related to Oral CHM
4367	Ma K, Zhou L, Zhang Y, et al. Efficacy and safety of traditional Chinese medicines combined with conventional Western medicines in the treatment of type 2 diabetes mellitus: a network meta-analysis of randomized controlled trials. Front Endocrinol (Lausanne). 2023;14:1134297. doi:10.3389/fendo.2023.1134297.	Not related to Oral CHM
4368	Ma, L. X., Wang, Y. Y., Li, X. X., & Liu, J. P. (2012). Systematic review on methodology of randomized controlled trials of post-marketing Chinese patent drugs for treatment of type 2 diabetes. Zhong xi yi jie he xue bao= Journal of Chinese Integrative Medicine, 10(3), 279-292.	Not related to Oral CHM
4369	Ma YL, Wang YH, Han JY. [Impact of qutan huayu jiedu herbs on monocyte subpopulations abnormality in patients with hyperlipidemia of phlegm-stagnancy obstruction syndrome pattern]. Zhongguo Zhong Xi Yi Jie He Za Zhi. 2011;(12):23‑27.	Not related to Oral CHM
4370	Ming J, Xu S, Liu C, Liu X, Jia A, Ji Q. Effectiveness and safety of bifidobacteria and berberine in people with hyperglycemia: Study protocol for a randomized controlled trial. Trials. 2018;19(1):72. doi:10.1186/s13063-018-2438-5.	Not related to Oral CHM
4371	Nematollahi S, Pishdad GR, Zakerkish M, Namjoyan F, Ahmadi Angali K, Borazjani F. The effect of berberine and fenugreek seed co-supplementation on inflammatory factor, lipid and glycemic profile in patients with type 2 diabetes mellitus: a double-blind controlled randomized clinical trial. Diabetol Metab Syndr. 2022;14(1):120. doi:10.1186/s13098-022-00888-9.	Not related to Oral CHM

4372	Pan L, Zhai X, Duan Z, Xu K, Liu G. Systematic review and meta-analysis of Coptis chinensis Franch.-containing traditional Chinese medicine as an adjunct therapy to metformin in the treatment of type 2 diabetes mellitus. Front Pharmacol. 2022;13:956313.	Not related to Oral CHM
4373	Panneerselvam J, Sambandam G, Nalini N. Single- or double-blind treatment with Balsamodendron mukul and nifedipine in hypertensive patients. J Clin Hypertens (Greenwich). 2005;7(6):340-5.	Not related to Oral CHM
4374	Sadeghi-Dehsahraei H, Esmaeili Gouvarchin Ghaleh H, Mirnejad R, Parastouei K. The effect of bergamot (KoksalGarry) supplementation on lipid profiles: a systematic review and meta-analysis of randomized controlled trials. Phytother Res. 2022;36(12):4409-4424.	Not related to Oral CHM
4375	Sanjari M, Shamsinejad B, Khazaeli P, Safi Z, Mirrashidi F, Naghibzadeh-Tahami A. Safety and efficacy of Berberis integerrima root extract in patients with type 2 diabetes. A parallel intervention based triple blind clinical trial. J Diabetes Metab Disord. 2020;19(1):71-80.	Not related to Oral CHM
4376	Shojaei Shad F, Jahantigh Haghighi M. Study of the effect of the essential oil (extract) of rhubarb stem (shoot) on glycosylated hemoglobin and fasting blood glucose levels in patients with type II diabetes. Biomedicine (Taipei). 2018;8(4):24.	Not related to Oral CHM
4377	Tian J, Lian F, Tong X. Safety and effectiveness of different herbal medicine dosage of Gegen Qinlian Decoction in Chinese patients with type 2 diabetes: a double-blind, two-part, randomised controlled trial. Lancet Diabetes Endocrinol. 2016;4(Suppl 1):S25.	Not related to Oral CHM
4378	Tian J, Wen Z, Guo X, et al. Effectiveness and safety of Zhibitai versus atorvastatin for hyperlipidemia: a systematic review. Chin J Evid Based Med. 2013;13(9):1116-22.	Not related to Oral CHM
4379	Tong X, Lian F, Zhou Q, et al. Prospective multicenter clinical trial of Chinese herbal formula JZQG (Jiangzhuoqinggan) for hypertension. Am J Chin Med. 2013;41(1):33-42.	Not related to Oral CHM
4380	Tong XL, Wu ST, Lian FM, et al. The safety and effectiveness of TM81, a Chinese herbal medicine, in the treatment of type 2 diabetes: a randomized double-blind placebo-controlled trial. Diabetes Obes Metab. 2013;15(5):448-54.	Not related to Oral CHM
4381	Wang Q, Wang J, Li N, et al. A systematic review of Orthosiphon stamineus Benth. in the treatment of diabetes and its complications. Molecules. 2022;27(2):444.	Not related to Oral CHM
4382	Wang ZK, Wang FW, Wang GY. Observation of effects of tiaozhi zengshou tang on regulation of dyslipidemia. Chin J Clin Rehabil. 2005;9(43):183-5.	Not related to Oral CHM
4383	Xiao Y, Wang X, Yang J, et al. Huatan Dingji Decoction intervening in atrial fibrillation: protocol for a randomized double-blind single-simulated placebo-controlled clinical trial. Trials. 2021;22(1):693. Published 2021 Oct 11. doi:10.1186/s13063-021-05522-z	Not related to Oral CHM

4384	Yu X, Xu L, Zhou Q, et al. The Efficacy and Safety of the Chinese Herbal Formula, JTTZ, for the Treatment of Type 2 Diabetes with Obesity and Hyperlipidemia: A Multicenter Randomized, Positive-Controlled, Open-Label Clinical Trial. Int J Endocrinol. 2018;2018:9519231. Published 2018 Apr 1. doi:10.1155/2018/9519231	Not related to Oral CHM
4385	Zhang R, Xiao Y, Yan J, et al. Effects of Berberine Plus Inulin on Diabetes Care in Patients With Latent Autoimmune Diabetes in Adults: Protocol for a Randomized Controlled Trial. Front Endocrinol (Lausanne). 2022;13:876657. Published 2022 Jun 15. doi:10.3389/fendo.2022.876657	Not related to Oral CHM
4386	Zhao P, Chen J, Hong YD. Zhongguo Zhong Xi Yi Jie He Za Zhi. 2007;27(1):21-24.	Not related to Oral CHM
4387	Zhao Y, Yang YY, Yang BL, et al. Efficacy and safety of berberine for dyslipidemia: study protocol for a randomized double-blind placebo-controlled trial. Trials. 2021;22(1):85. Published 2021 Jan 22. doi:10.1186/s13063-021-05028-8	Not related to Oral CHM
4388	曹晶晶,杨卫杰.化痰祛湿行气活血法治疗痰瘀型 2 型糖尿病临床观察[J].中医临床研究,2012,4(01):25-26.	Not related to Oral CHM
4389	柴露露,谢雁鸣,王连心,等.老年患者使用清开灵注射液的疾病特征及联合用药分析[J].世界中西医结合杂志,2019,14(08):1142-1147.DOI:10.13935/j.cnki.sjzx.190826.	Not related to Oral CHM
4390	陈莉,韩晓玲.体质辨识在低、中危高血压“治未病”健康管理中的应用[J].现代医院,2016,16(07):1088-1090.	Not related to Oral CHM
4391	陈霞波,龚文波,张业,等.降浊合剂治疗糖尿病前期气虚痰浊证临床研究[J].中华中医药学刊,2013,31(06):1385-1387.DOI:10.13193/j.archtcm.2013.06.171.chenxb.074.	Not related to Oral CHM
4392	陈新宇,刘越美,谢海波,等.养肝清肝平肝法干预原发性高血压患者血压及血管紧张素 II 的变化(英文)[J].中国临床康复,2006,(27):178-180.	Not related to Oral CHM
4393	崔德成,崔丽平.自拟泽泻柴胡汤治疗高脂血症的临床观察[J].北京中医,2004,(03):152-153.	Not related to Oral CHM
4394	崔彦如.老年代谢综合征中医证候特征及中药干预疗效评价的初步分析[J].中医临床研究,2015,7(06):23-25.	Not related to Oral CHM
4395	邓培友.自拟健脾散瘀化浊方对 2 型糖尿病血糖水平及血液粘稠度的影响[J].四川中医,2017,35(01):103-105.	Not related to Oral CHM
4396	刁宏.从痰瘀论治治疗糖尿病高脂血症 45 例[J].中国医药导报,2012,9(20):103-104.	Not related to Oral CHM
4397	丁宇炜,徐瑛,沈丕安.中医分型治疗高脂血症 45 例观察[J].陕西中医学院学报,2003,(05):11-14.	Not related to Oral CHM
4398	冯居秦.参苓白术散配针刺耳穴压丸治疗痰湿型肥胖症 80 例[J].陕西中医,2008,(10):1364-1365.	Not related to Oral CHM
4399	关婕婷,刘宝珍,李莲英,等.应用浊毒理论治疗 2 型糖尿病合并急性脑梗死临床观察[J].辽宁中医杂志,2014,41(07):1448-1450.DOI:10.13192/j.issn.1000-1719.2014.07.057.	Not related to Oral CHM

4400	关婕婷,齐锡友,谢春荣,等.中西医结合治疗糖尿病合并脑梗死 35 例临床研究[J].江苏中医药,2011,43(05):34-35.	Not related to Oral CHM
4401	关芸,娄锡恩,张丽萍,等.健脾清热化浊法改善 2 型糖尿病胰岛素抵抗的临床观察[J].湖南中医杂志,2015,31(05):4-6.DOI:10.16808/j.cnki.issn1003-7705.2015.05.002.	Not related to Oral CHM
4402	韩玉晶.自拟降脂汤治疗痰浊血瘀型高脂血症 40 例[J].中西医结合心脑血管病杂志,2005,(08):735.	Not related to Oral CHM
4403	贺群慧,黄斌.基于文献研究健脾疏肝法对糖尿病前期患者疗效的Meta分析[J].中医临床研究,2021,13(34):121-125.	Not related to Oral CHM
4404	侯瑞芳,刘晓倩,金昕,等.泄热化浊方治疗胃热滞脾证肥胖患者的临床研究[J].辽宁中医杂志,2019,46(01):65-69.DOI:10.13192/j.issn.1000-1719.2019.01.023.	Not related to Oral CHM
4405	胡金梅,李敏,张书宁.六味能消胶囊对肥胖症患者中医症候疗效观察[J].河北医药,2014,36(13):1950-1951.	Not related to Oral CHM
4406	胡金梅,李敏,张书宁.六味能消胶囊治疗单纯性肥胖症患者中医单项症状的疗效观察[J].中国药房,2014,25(16):1456-1458.	Not related to Oral CHM
4407	胡金梅,孙涛,李玮桓,等.六味能消胶囊治疗肥胖症临床疗效观察[J].中国煤炭工业医学杂志,2014,17(02):295-297.	Not related to Oral CHM
4408	华依梦,周甜.化浊逐瘀汤治疗高血压合并高尿酸血症患者的疗效观察[J].中国中医药科技,2019,26(06):875-876.	Not related to Oral CHM
4409	靳锐锋,崔红生,郭丰婷,等.消鼾利气颗粒治疗阻塞性睡眠呼吸暂停低通气综合征的临床疗效评价[J].中华中医药杂志,2019,34(01):374-376.	Not related to Oral CHM
4410	康学东,李芳芳,秦双红.化浊胶囊治疗非酒精性脂肪性肝病以预防 2 型糖尿病的研究[J].中医研究,2019,32(03):19-21.	Not related to Oral CHM
4411	李景巍.真武汤加减治疗老年原发性高血压病 40 例[J].河南中医,2009,29(07):640-641.DOI:10.16367/j.issn.1003-5028.2009.07.030.	Not related to Oral CHM
4412	李莉芬,吴玉红.健脾化痰活血法改善 2 型糖尿病胰岛素抵抗的临床观察[J].北京中医,2006,(07):395-397.	Not related to Oral CHM
4413	李琳,李晓斌,焦晓民.通脉化浊汤治疗瘀血阻滞型高脂血症随机平行对照研究[J].实用中医内科杂志,2014,28(05):33-34.DOI:10.13729/j.issn.1671-7813.2014.05.18.	Not related to Oral CHM
4414	李增英,李惠林,赵恒侠,等.荷芪散治疗糖尿病前期疗效观察[J].中医学报,2013,28(08):1208-1210.DOI:10.16368/j.issn.1674-8999.2013.08.034.	Not related to Oral CHM
4415	梁燕科.通瘀化浊汤治疗高脂血症 64 例临床观察[J].新中医,2016,48(06):18-19.DOI:10.13457/j.cnki.jncm.2016.06.008.	Not related to Oral CHM
4416	刘婷,段刚峰.五苓散加减联合电针治疗痰湿内阻型高脂血症患者疗效研究[J].陕西中医,2018,39(03):307-309.	Not related to Oral CHM
4417	陆新,张瑶光.健脾益气降浊方配合西药治疗痰湿壅盛型原发性高血压病 84 例疗效观察[J].中医杂志,2014,55(05):404-407.DOI:10.13288/j.11-2166/r.2014.05.012.	Not related to Oral CHM
4418	梅俊,张萍,戎光,等.化浊通脉方治疗原发性高脂血症合并颈动脉粥样硬化(痰浊阻遏证)的临床研究[J].中药药理与临床,2021,37(04):188-191.DOI:10.13412/j.cnki.zyyl.20210707.003.	Not related to Oral CHM

4419	孟淑萍. 中西医结合治疗高血压病 200 例疗效观察[J]. 中国医药指南, 2012, 10(32): 281-282.	Not related to Oral CHM
4420	莫小书,周月红,廖尚上,等.六郁同治法组方胰岛康Ⅱ号治疗肥胖型糖尿病前期临床观察[J].中国中医药现代远程教育,2020,18(12):61-64.	Not related to Oral CHM
4421	南明花,焦晓民,李琳,等.通脉化浊汤(免煎颗粒)联合西药及健康教育综合干预痰瘀互结高血压双盲安慰剂随机平行对照研究[J].实用中医内科杂志,2017,31(09):30-35.DOI:10.13729/j.issn.1671-7813.2017.09.12.	Not related to Oral CHM
4422	徐伟,徐秀芬.降脂汤治疗高脂血症 40 例疗效观察[J].内蒙古医学杂志,2008,40(10):1226-1227.	Not related to Oral CHM
4423	石维远,庄慧魁.化浊降脂合剂治疗高脂血症 32 例[J].中国中医药现代远程教育,2012,10(17):8-9.	Not related to Oral CHM
4424	史文丽,刘飞飞,张晓锋,等.肝源性糖尿病的中医证候特点及疗效分析[J].中西医结合肝病杂志,2015,25(04):212-214.	Not related to Oral CHM
4425	王金梅,郭俊杰.益气化浊胶囊联合西医治疗气阴两虚、痰瘀互结型 2 型糖尿病胰岛素抵抗的临床研究[J].河北中医,2017,39(10):1535-1538.	Not related to Oral CHM
4426	刘照峰.益气化浊胶囊改善 2 型糖尿病胰岛素抵抗临床观察[J].中医药临床杂志,2018,30(04):698-700.DOI:10.16448/j.cjtcm.2018.0210.	Not related to Oral CHM
4427	王小沛.化浊祛瘀汤治疗高脂血症 85 例[J].河南中医,2011,31(08):891-892.DOI:10.16367/j.issn.1003-5028.2011.08.019.	Not related to Oral CHM
4428	王雪利.自拟化浊降脂汤治疗痰瘀互结型高脂血症 42 例临床观察[J].甘肃中医药大学学报,2019,36(06):49-52.DOI:10.16841/j.issn1003-8450.2019.06.11.	Not related to Oral CHM
4429	徐风玲.化浊祛痰活血汤治疗高脂血症 70 例[J].河南中医,2012,32(10):1330-1331.DOI:10.16367/j.issn.1003-5028.2012.10.008.	Not related to Oral CHM
4430	袁艺,赵波,陈劲秋.解郁化浊汤防治 2 型糖尿病前期 56 例[J].中国实验方剂学杂志,2013,19(24):314-317.	Not related to Oral CHM
4431	赵倩,闻新丽,段盛蕾,等.轻身消脂汤联合穴位埋线治疗脾虚湿阻型单纯性肥胖临床研究[J].陕西中医,2019,40(06):796-798.	Not related to Oral CHM
4432	周德英,窦易铭,陆小左.金芪降糖片联合用药治疗 2 型糖尿病 1 例[J].中国医药导刊,2016,18(05):513-514.	Not related to Oral CHM
4433	Gai Yun, G. Y., Zhang Tong, Z. T., & Yang XiaoPing, Y. X. (2011). " Ye's benefiting Qi and softening Hardness Decoction" combined with routine western medicine in treatment of stage-IV diabetic nephropathy: a prospective cohort study.	Not related to Oral CHM
4434	Liu C, Guo X. Adjuvant Chinese Medicine for the Treatment of Type 2 Diabetes Mellitus Combined with Mild Cognitive Impairment: A Systematic Review and Meta-Analysis of a Randomised Controlled Trial. Pharmaceuticals (Basel). 2022;15(11):1424. Published 2022 Nov 17. doi:10.3390/ph15111424	Not related to Oral CHM
4435	Zhou B, Zhang G, Guo W, Ren C, Li M. Xiaoke Decoction in treatment of type II diabetes: A Meta-analysis. Chin Herb Med. 2021;14(1):130-141. Published 2021 Aug 27. doi:10.1016/j.chmed.2021.08.004	Not related to Oral CHM
4436	Pang B, Guo J, Zhao L, Zhao X, Zhou Q, Tong X. J Tradit Chin Med. 2016;36(3):307-313. doi:10.1016/s0254-6272(16)30042-5	Not related to Oral CHM

4437	董天宝,孙特,杨士勇.化浊通瘀汤联合氯沙坦钾治疗高血压随机平行对照研究[J].实用中医内科杂志,2015,29(06):93-94.DOI:10.13729/j.issn.1671-7813.2015.06.44.	Not related to Oral CHM
4438	Zhao FH, Liu GB, Lu SZ. Zhongguo Zhong Xi Yi Jie He Za Zhi. 2010;30(10):1052-1055.	Not related to Oral CHM
4439	Chui, S. H., Wong, Y. H., Fong, M. Y., Chow, F. C., Chiu, Y. M., & Lam, C. W. K. (2016). A clinical study of the anti-diabetic effect of a simple Chinese herbal formula, PSP-1. European Journal of Integrative Medicine, 8(4), 458-464.	Not related to Oral CHM
4440	Huang YH, Chen ST, Liu FH, et al. The efficacy and safety of concentrated herbal extract granules, YH1, as an add-on medication in poorly controlled type 2 diabetes: A randomized, double-blind, placebo-controlled pilot trial. PLoS One. 2019;14(8):e0221199. Published 2019 Aug 15. doi:10.1371/journal.pone.0221199	Not related to Oral CHM
4441	王珏云,杨高松,张显燕.化浊解毒中药联合利拉鲁肽治疗 2 型糖尿病合并非酒精性脂肪肝疗效及对血脂、肝功能的影响[J].现代中西医结合杂志,2017,26(22):2425-2428.	Not related to Oral CHM
4442	陈大舜,董克礼,陈泽奇,等.2 型糖尿病病证结合诊疗方案多中心临床观察与研究[J].湖南中医药大学学报,2007,(01):1-4.	Not related to Oral CHM
4443	李学勇, 2012, 辨证治疗高血压病临床观察, 中医学报	Not eligible control
4444	Soltani, R., Hakimi, M., Asgary, S., Ghanadian, S. M., Keshvari, M., & Sarrafzadegan, N. (2014). Evaluation of the effects of Vaccinium arctostaphylos L. Fruit extract on serum lipids and hs‐CRP levels and oxidative stress in adult patients with hyperlipidemia: A randomized, double‐blind, placebo‐controlled clinical trial. Evidence‐Based Complementary and Alternative Medicine, 2014(1), 217451.	Not eligible control
4445	王博,吴松,谢俊.参苓白术散联合循经走罐治疗脾虚湿阻型肥胖症 55 例临床观察[J].湖南中医杂志,2017,33(07):9-11.DOI:10.16808/j.cnki.issn1003-7705.2017.07.004.	Not eligible control
4446	宋诵文,曾琦,吴新民,等.温肾化浊、逐瘀通络为主治疗糖尿病肾病的临床观察[J].世界中医药,2017,12(09):2019-2022.	Not eligible control
4447	宋凤平,景良洪,曾艳丽.云南白药联合美宝湿润烧伤膏及半导体激光照射治疗糖尿病足溃疡的疗效观察[J].激光杂志,2015,36(08):151-154.DOI:10.14016/j.cnki.jgzz.2015.08.151.	Not eligible control
4448	尚云青,杨文华,徐天苹,等.中医食疗辅助中医药治疗高血压病痰湿壅盛证 62 例[J].云南中医中药杂志,2017,38(07):38-40.DOI:10.16254/j.cnki.53-1120/r.2017.07.016.	Not eligible control
4449	张志伟,冯志海.自拟中药汤剂联合湿润烧伤膏对Wagner 3～4 级糖尿病足患者创面组织中VEGF、bFGF表达的影响[J].中国烧伤创疡杂志,2020,32(05):327-330.	Not eligible control
4450	马莹, 2014, 糖尿病足中西医结合护理, 辽宁中医药大学学报	Not eligible control

4451	丛日晖.HD-91-Ⅱ型肝病治疗仪配合中药治疗高血脂性脂肪肝 42 例临床观察[J].云南中医学院学报 ,2010,33(02):34-35.DOI:10.19288/j.cnki.issn.1000-2723.2010.02.011.	Not eligible control
4452	郭强,赵欢,朱玉霞,等.中医规范化治疗糖尿病前期临床研究[J].时珍国医国药,2015,26(06):1402-1404.	Not eligible control
4453	胡敬宝,黄秀玲.化痰通络法治疗老年人高血压病的临床研究[J].吉林中医药,2003,(06):12-13.DOI:10.13463/j.cnki.jlzyy.2003.06.007.	Not eligible control
4454	李朝敏,王仲,暴鹏,等.肥胖 2 型糖尿病脂代谢紊乱中医综合调控方案研究[J].湖北中医杂志,2015,37(12):1-3.	Not eligible control
4455	李芳.清解化湿法配合耳穴贴压治疗儿童单纯性肥胖 32 例[J].江西中医药 ,2008,(11):68.	Not eligible control
4456	李亚娟,沈小璇,袁婕.防己黄芪汤联合循经推腹法治疗脾虚湿阻型单纯性肥胖疗效观察[J].现代中西医结合杂志,2020,29(26):2924-2927.	Not eligible control
4457	李艳莉.柴胡温胆汤治疗痰湿型 2 型糖尿病 63 例临床观察[J].中国民族民间医药,2016,25(06):95+97.	Not eligible control
4458	李颖,朱婴,陈旦平,等.耳穴贴压、针刺联合眩晕 1 号方治疗痰湿壅盛型原发性高血压临床观察[J].河北中医,2017,39(05):743-746+750.	Not eligible control
4459	李运伦.理脾化湿法治疗原发性高血压 35 例[J].陕西中医 ,2004,(02):136-138.	Not eligible control
4460	欧璐,李晓琴,李宇,等.加味黄连温胆汤治疗新诊 2 型糖尿病痰（湿）热互结证临床观察[J].中国实验方剂学杂志 ,2021,27(01):128-134.DOI:10.13422/j.cnki.syfjx.20202125.	Not eligible control
4461	孟伟.清脂六通丸治疗高脂血症气滞血瘀痰浊内阻证多中心临床研究[D].北京中医药大学,2006.	Not eligible control
4462	尹倚艰,冯玲,路杰.路志正祛湿化浊通心方干预老年血脂代谢异常的临床研究[C]//中华中医药学会心病分会,北京中医药学会心血管病专业委员会.2011 年中华中医药学会心病分会学术年会暨北京中医药学会心血管病专业委员会年会论文集.中国中医科学院广安门医院综合科;北京三芝堂诊所;,2011:274-280.	Not eligible control
4463	王俊琴.半夏白术天麻汤合针刺联合西药治疗痰湿壅盛型顽固性高血压 34 例[J].中医研究,2014,27(05):59-61.	Not eligible control
4464	李学国,张保春,刘福奇,等.化瘀泻浊颗粒治疗高血压病痰湿壅盛证疗效观察[J].辽宁中医药大学学报 ,2015,17(05):134-137.DOI:10.13194/j.issn.1673-842x.2015.05.046.	Not eligible control
4465	徐添.龙胆泻肝汤治疗原发性高血压病肝火上炎证 54 例疗效观察[J].内蒙古中医药,2014,33(04):2-3.DOI:10.16040/j.cnki.cn15-1101.2014.04.146.	Not eligible control
4466	郭培才,黎丽娴,罗坚文,等.温胆汤加减配合中药沐足包治疗痰湿型高血压患者 34 例[J].按摩与康复医学 ,2020,11(16):46-47.DOI:10.19787/j.issn.1008-1879.2020.16.017.	Not eligible control
4467	Zhang, X. S., Li, J., Shi, G. Y., & Zhao, L. N. (2021). Superb micro-vascular imaging for evaluation of Banxia Baizhu Tianma decoction in improving stability of carotid atherosclerotic plaque of hypertension with phlegm dampness obstruction.	Not eligible control

4468	董辉,杨丹丹.中药养生茶干预痰湿质高血压患者临床疗效观察[J].辽宁中医药大学学报,2019,21(12):180-183.DOI:10.13194/j.issn.1673-842x.2019.12.047.	Not eligible control
4469	周翼.中医体质辨识在高血压分级管理中的应用与探讨[J].保健医学研究与实践,2016,13(01):41-46.	Not eligible control
4470	Wang JP, Fan RH, Wang Y, Mei Y. Zhongguo Zhong Xi Yi Jie He Za Zhi. 2013;33(6):736-740.	Not eligible control
4471	谢慧君.加味五苓散干预脾虚痰浊型血脂异常的临床观察[J].光明中医,2013,28(07):1387-1389.	Not eligible control
4472	叶攀,杨丹.四物降脂颗粒治疗脾虚湿盛型高脂血症的临床观察[J].湖北中医杂志,2015,37(09):15-16.	Not eligible control
4473	卓冰帆,张彦卿,张敏.山荷降脂汤治疗痰湿内阻型高脂血症合并脂肪肝的临床研究[J].时珍国医国药,2019,30(06):1426-1428.	Not eligible control
4474	轩晨惠.清热利湿健脾方治疗湿热困脾型肥胖 2 型糖尿病的疗效观察[D].华北理工大学,2021.DOI:10.27108/d.cnki.ghelu.2021.000123.	Not eligible control
4475	王永治.祛湿健脾颗粒治疗糖尿病合并高脂血症的疗效分析[J].中国医药指南,2015,13(12):175-176.DOI:10.15912/j.cnki.gocm.2015.12.132.	Not eligible control
4476	杨秀萍.中西药联合治疗 2 型糖尿病合并超重或肥胖患者 19 例[J].光明中医,2017,32(17):2544-2546.	Not eligible control
4477	曾英,江涛,龙晓静.中药食疗对肥胖 2 型糖尿病患者血糖及血脂的影响[J].广西医学,2006,(02):199-201.	Not eligible control
4478	刘成琼.自拟降糖汤联合西药治疗痰湿型 2 型糖尿病随机平行对照研究[J].实用中医内科杂志,2012,26(10):53-54.	Not eligible control
4479	魏联杰,陈欣,叶森,等.中药瘦身茶应用于单纯性肥胖病(脾虚湿阻型)的效果探讨[J].中国医疗美容,2020,10(09):119-122.DOI:10.19593/j.issn.2095-0721.2020.09.030.	Not eligible control
4480	骆天炯,张钟爱,高罂,等.加减四妙散对超重糖耐量减低患者炎症因子的影响[J].中医杂志,2012,53(19):1655-1657.DOI:10.13288/j.11-2166/r.2012.19.010.	Not outcome of interest
4481	廉波,赵泉林.加味二陈汤治疗脾瘅疗效观察[J].山东中医杂志,2014,33(06):437-439.DOI:10.16295/j.cnki.0257-358x.2014.06.008.	Not outcome of interest
4482	周晓燕,罗家丽,李雅茜,等.健脾理气、祛湿降浊法治疗肥胖型糖耐量减低临床研究[J].中国医药指南,2012,10(08):205-207.DOI:10.15912/j.cnki.gocm.2012.08.283.	Not outcome of interest
4483	周欢,马蕊芳,芦少敏.平陈汤治疗 2 型糖尿病前期肥胖痰湿证 42 例[J].中医临床研究,2018,10(21):43-45.	Not outcome of interest
4484	杨威,高天舒.清热祛湿法对 2 型糖尿病湿热证疗效评价研究[J].实用中医内科杂志,2011,25(03):57-58.	Not outcome of interest
4485	毛振营.祛湿化浊汤治疗气虚痰浊型 2 型糖尿病 70 例[J].中国中医药现代远程教育,2008,(06):629.	Not outcome of interest
4486	朱建伟,留菁菁.升阳利湿法对肥胖的糖耐量受损人群的干预性治疗效果[J].中国慢性病预防与控制,2013,21(02):226-228.DOI:10.16386/j.cjpccd.issn.1004-6194.2013.02.037.	Not outcome of interest

4487	魏燕,任跃忠.糖Ⅰ号协定方治疗糖调节受损的临床研究[J].浙江中医杂志,2009,44(07):510-511.	Not outcome of interest
4488	周跃.从邪伏散膏论治糖尿病前期（脾虚痰湿证）临床观察[D].长春中医药大学,2016.	Not outcome of interest
4489	Fang Z, Zhao J, Shi G, et al. Shenzhu Tiaopi granule combined with lifestyle intervention therapy for impaired glucose tolerance: a randomized controlled trial. Complement Ther Med. 2014;22(5):842-850. doi:10.1016/j.ctim.2014.08.004	Not outcome of interest
4490	杨继英.针药结合治疗痰湿型 2 型糖尿病 32 例疗效观察[J].中国民间疗法,2015,23(06):65-66.DOI:10.19621/j.cnki.11-3555/r.2015.06.049.	Not outcome of interest
4491	华东平,赵晖,杨朴强.枳实导滞丸加减联合胰岛素强化治疗 2 型糖尿病临床研究[J].中医学报,2016,31(09):1296-1298.DOI:10.16368/j.issn.1674-8999.2016.09.364.	Not outcome of interest
4492	Tong XL, Lian FM, Zhou Q, et al. Prospective multicenter clinical trial of Chinese herbal formula JZQG (Jiangzhuoqinggan) for hypertension. Am J Chin Med. 2013;41(1):33-42. doi:10.1142/S0192415X13500031	Not outcome of interest
4493	陈利群,于海峰,王维谆.半夏白术天麻汤加味配合西药治疗痰浊上蒙型原发性高血压 40 例临床观察[J].甘肃中医,2005,(02):1-3.	Not outcome of interest
4494	牛延峰.半夏白术天麻汤联合依那普利治疗痰湿中阻型高血压的临床研究[J].中医药临床杂志,2014,26(09):901-902.DOI:10.16448/j.cjtcm.2014.09.018.	Not outcome of interest
4495	容燕虹,黎建华,罗兴,等.辨证分型联合氯沙坦治疗老年高血压合并左心室肥厚随机平行对照研究[J].实用中医内科杂志,2019,33(06):5-9.DOI:10.13729/j.issn.1671-7813.z20190255.	Not outcome of interest
4496	张焱,陈咸川,何立人,等.化湿利水泄浊合剂对高血压病患者血管内皮依赖性舒张功能的影响[J].上海中医药杂志,2005,(06):14-16.DOI:10.16305/j.1007-1334.2005.06.006.	Not outcome of interest
4497	莫鸿辉,王艳红,利丹.温胆片结合穴位敷贴治疗痰湿中阻型高血压病 35 例疗效观察[J].新中医,2010,42(08):112-113.DOI:10.13457/j.cnki.jncm.2010.08.062.	Not outcome of interest
4498	钱力维,张进军,陈际连,等.小陷胸汤合半夏泻心汤加减治疗痰湿壅盛型老年高血压病 23 例[J].安徽中医药大学学报,2015,34(06):30-32.	Not outcome of interest
4499	常翠萍.补肾化瘀通络汤治疗老年性高血压病临床研究[J].四川中医,2014,32(12):77-79.	Not outcome of interest
4500	孔利环,杨涛.中西医结合治疗痰湿内盛型高血压病 45 例[J].河南中医,2013,33(12):2184-2185.DOI:10.16367/j.issn.1003-5028.2013.12.105.	Not outcome of interest
4501	郭爱莲.中西医结合治疗痰湿内阻型原发性高血压 35 例临床观察[J].中外医疗,2012,31(09):117.DOI:10.16662/j.cnki.1674-0742.2012.09.028.	Not outcome of interest
4502	袁怿,李璐.加味扁豆薏米粥对 51 例脾虚湿盛型原发性高脂血症患者的疗效研究[J].武警后勤学院学报(医学版),2021,30(12):36-37+40.DOI:10.16548/j.2095-3720.2021.12.015.	Not outcome of interest
4503	柯斌,师林,张俊杰,等.加味苓桂术甘汤联合短期极低热量饮食治疗脾虚痰湿型高脂血症合并脂肪肝的临床研究[J].实用医学杂志,2012,28(04):655-657.	Not outcome of interest

4504	柯斌,师林,张俊杰,等.加味苓桂术甘汤联合短期极低热量饮食治疗脾虚痰湿型高脂血症合并脂肪肝的临床研究[J].实用医学杂志,2012,28(04):655-657.	Not outcome of interest
4505	柯斌,师林,张俊杰,等.加味苓桂术甘汤联合短期极低热量饮食治疗脾虚痰湿型高脂血症合并脂肪肝的临床研究[J].实用医学杂志,2012,28(04):655-657.	Not outcome of interest
4506	万红,闫诏,李媛媛,等.加减消积保中颗粒联合刮痧治疗腹型肥胖胃热湿阻证的临床观察[J].中国实验方剂学杂志,2021,27(12):97-102.DOI:10.13422/j.cnki.syfjx.20210534.	Not outcome of interest
4507	李力.中医综合疗法干预痰湿瘀阻型单纯性肥胖 60 例[J].中医研究,2014,27(09):21-23.	Not outcome of interest
4508	王国强.251 例糖尿病足中医证型及常用方剂临床调查分析[J].中医研究,2015,28(05):40-42.	Not outcome of interest
4509	张智斌.2 型糖尿病并发肺部感染 52 例中医辨证分析[J].中国民族民间医药,2010,19(08):70.	Not outcome of interest
4510	Zakeri MA, Mohammadi V, Bazmandegan G, Zakeri M. Description of Ventricular Arrhythmia after Taking Herbal Medicines in Middle-Aged Couples. Case Rep Cardiol. 2020;2020:6061958. Published 2020 Oct 1. doi:10.1155/2020/6061958	Not outcome of interest
4511	Zhang ZJ, Cheng WW, Yang YM. Zhonghua Fu Chan Ke Za Zhi. 1994;29(8):463-509.	Not outcome of interest
4512	许丽娟,林跃辉.健脾养血止痒方联合中药外洗治疗糖尿病皮肤瘙痒临床体会[J].基层医学论坛,2021,25(02):265-266.DOI:10.19435/j.1672-1721.2021.02.065.	Not outcome of interest
4513	赵月,张娟.清热利湿法治疗糖尿病前期验案 1 则[J].世界最新医学信息文摘,2019,19(06):272+278.DOI:10.19613/j.cnki.1671-3141.2019.06.147.	Not outcome of interest
4514	王景,郭伟光,滕林,等.全蝎软膏配合湿润烧伤膏治疗糖尿病足 32 例[J].中国中医药现代远程教育,2014,12(17):33-34.	Not outcome of interest
4515	王延凡. (2011). 升阳益胃汤治疗痰湿气虚型糖尿病的疗效观察. 中国中医药咨讯, 3(23), 42-42.	Not outcome of interest
4516	周南.湿润烧伤膏治疗糖尿病性皮肤溃疡 8 例体会[J].临床军医杂志,2009,37(05):757.	Not outcome of interest
4517	杨力,许向明,罗职伟.湿润烧伤膏治愈糖尿病下肢溃疡 1 例报告[J].中国烧伤创疡杂志,2012,24(02):126-127.	Not outcome of interest
4518	孙文亮,左艳敏,于红俊,等.糖疽康汤治疗湿热型糖尿病足溃疡 33 例[J].中医临床研究,2015,7(10):15-16.	Not outcome of interest
4519	张立,罗金花.糖尿病并发双侧带状疱疹 1 例[J].皮肤病与性病,2007,(01):53.	Not outcome of interest
4520	周律. (2016). 糖尿病合并呼吸衰竭持续无创正压通气致鼻部压疮 1 例的处理. 中国组织工程研究, 20(B05), 71-72.	Not outcome of interest
4521	彭红叶,鲁春丽.主动健康视角下糖尿病前期中西医结合治疗和管理的现状与挑战[J].中国全科医学,2025,28(13):1573-1582+1606.	Not outcome of interest
4522	张杰.糖尿病性皮肤溃疡再生修复的治疗观察[J].中国烧伤创疡杂志,2003,(02):158-159.	Not outcome of interest

4523	王斌,马运涛.吴深涛运用当归拈痛汤治疗湿热型消渴病痹症举隅[J].实用中医内科杂志,2012,26(10):14-15.	Not outcome of interest
4524	唐德芝. (2013). 中西医结合治疗糖尿病周围神经病变 34 例. 中国中医药现代远程教育, (2), 37-38.	Not outcome of interest
4525	庞咪咪,李宏春.中药熏洗致皮肤过敏 3 例[J].中国乡村医药,2018,25(07):42.DOI:10.19542/j.cnki.1006-5180.001562.	Not outcome of interest
4526	王志敏,项俊.中药制剂治疗糖尿病足 2 例[J].人民军医,2009,52(05):312.	Not outcome of interest
4527	王颖辉,赵进喜,王世东,等.中医药干预对早期糖尿病肾病证素演变的影响[J].中医杂志,2012,53(20):1759-1763.DOI:10.13288/j.11-2166/r.2012.20.016.	Not outcome of interest
4528	路春燕.中医外治法治疗 153 例糖尿病足临床疗效观察[J].亚太传统医药,2014,10(02):84-85.	Not outcome of interest
4529	刘静.湿润烧伤膏与川芎嗪联合治疗糖尿病足 61 例疗效观察[J].中国医学创新,2011,8(27):147-148.	Not outcome of interest
4530	林友泉.从三焦气化论治 2 型糖尿病的实验与临床初步研究[D].广州中医药大学,2010.	Not outcome of interest
4531	廖梅娟,郭笑菊.42 例糖尿病足的综合护理观察[J].中国当代医药,2009,16(13):194-195.	Not outcome of interest
4532	梁淑仪 and 张永霞, 2017, 中西医结合治疗糖尿病足的临床护理, 实用手外科杂志	Not outcome of interest
4533	金龙虎.62 例糖尿病足坏疽诱因分析及防治体会[J].中国社区医师(医学专业半月刊),2009,11(09):91.	Not outcome of interest
4534	胡丽娟.黄连膏湿敷治疗糖尿病性大疱 1 例护理体会[J].内蒙古中医药,2015,34(07):164.DOI:10.16040/j.cnki.cn15-1101.2015.07.163.	Not outcome of interest
4535	郭强,赵欢,朱玉霞,等.张发荣治疗糖尿病周围神经病变用药特点分析[J].中医杂志,2015,56(17):1465-1467.DOI:10.13288/j.11-2166/r.2015.17.008.	Not outcome of interest
4536	龚健冰,郑秋萍.补肾利湿通络法治疗老年高血压病 35 例[J].黑龙江中医药,2010,39(02):28.	Not outcome of interest
4537	杜翠忠,夏阳.分消走泄法治疗肥胖型闭经 32 例[J].成都中医药大学学报,2010,33(04):31-32.DOI:10.13593/j.cnki.51-1501/r.2010.04.010.	Not outcome of interest
4538	Fu Q, Yang H, Zhang L, et al. Traditional Chinese medicine foot bath combined with acupoint massage for the treatment of diabetic peripheral neuropathy: A systematic review and meta-analysis of 31 RCTs. Diabetes Metab Res Rev. 2020;36(2):e3218. doi:10.1002/dmrr.3218	Not outcome of interest
4539	Jiang L, Fu Q, Wang S, et al. Effect of RG (Coptis root and ginseng) formula in patients with type 2 diabetes mellitus: a study protocol for a randomized controlled and double-blinding trial. Trials. 2022;23(1):305. Published 2022 Apr 14. doi:10.1186/s13063-022-06229-5	Not outcome of interest
4540	Jiang T, Wen Z, Jiang M. Zhongguo Zhong Xi Yi Jie He Za Zhi. 2011;31(2):182-187.	Not outcome of interest
4541	Jing L, Gao ZY. Zhongguo Zhong Yao Za Zhi. 2008;33(19):2253-2256.	Not outcome of interest

4542	Tian JX, Li M, Liao JQ, Liu WK, Tong XL. Xiangshaliujunzi Decoction for the treatment of diabetic gastroparesis: a systematic review. World J Gastroenterol. 2014;20(2):561-568. doi:10.3748/wjg.v20.i2.561	Not outcome of interest
4543	Wang L, Xiang L, Piao S, et al. The Efficacy and Safety of Chinese Medicine Fufang Zhenzhu Tiaozhi Capsule (FTZ) in the Treatment of Diabetic Coronary Heart Disease: Study Protocol for Multicenter, Randomized, Double-Blind, Placebo-Controlled Clinical Trial. Diabetes Metab Syndr Obes. 2021;14:2651-2659. Published 2021 Jun 14. doi:10.2147/DMSO.S309419	Not outcome of interest
4544	曹银洲.辨证分型联合硝苯地平缓释片治疗高血压 80 例[J].中医研究,2014,27(01):27-29.	Not outcome of interest
4545	曾康成.辨证分型治疗 2 型糖尿病合并高血压病 86 例[J].中医药临床杂志,2011,23(08):683-684.DOI:10.16448/j.cjtcm.2011.08.011.	Not outcome of interest
4546	姜卓彤.2 型糖尿病并下肢动脉硬化闭塞症的相关因素研究与中药疗效评价[D].辽宁中医药大学,2016.	Not outcome of interest
4547	焦安贵,秦四娟.辨证分型联合西药治疗糖尿病合并高血压随机平行对照研究[J].实用中医内科杂志,2013,27(18):66-68.	Not outcome of interest
4548	刘强.猪苓汤联合西药治疗气阴两虚兼湿热 2 型糖尿病合并泌尿系感染随机平行对照研究[J].实用中医内科杂志,2018,32(01):28-30.DOI:10.13729/j.issn.1671-7813.z20170215.	Not outcome of interest
4549	鹿根启.达原饮加味治疗湿浊型 2 型糖尿病 21 例[J].河南中医,2010,30(12):1233-1234.DOI:10.16367/j.issn.1003-5028.2010.12.053.	Not outcome of interest
4550	吕秀群,刘得华,朱锦匙,等.肤爽系列方熏洗治疗糖尿病合并皮肤病变 42 例临床观察[J].中医药导报,2016,22(09):65-67.DOI:10.13862/j.cnki.cn43-1446/r.2016.09.022.	Not outcome of interest
4551	马红颖.探讨采用中医方法治疗糖尿病的临床疗效[J].中医临床研究,2015,7(31):110-111.	Not outcome of interest
4552	孙婉香.清热祛湿剂配合消炎止痛膏治疗 2 型糖尿病并合痛风 32 例[J].陕西中医,2014,35(06):713-714.	Not outcome of interest
4553	朱文宏.辨证分型联合硝苯地平治疗高血压脑血管疾病随机平行对照研究[J].实用中医内科杂志,2015,29(04):114-116.DOI:10.13729/j.issn.1671-7813.2015.04.51.	Not outcome of interest
4554	李君玲.中医辨证治疗儿童肥胖症 67 例[J].中医儿科杂志,2012,8(01):33-34.	Not outcome of interest
4555	刘树林,蔡文就,凌燕.儿童及青少年 2 型糖尿病临床特点回顾性分析[J].中国中西医结合儿科学,2011,3(02):147-149.	Not outcome of interest
4556	李春昱.加味半夏白术天麻饮联合缬沙坦治疗痰湿壅盛型原发性高血压病 112 例观察[J].中国民间疗法,2017,25(01):66-67.DOI:10.19621/j.cnki.11-3555/r.2017.01.059.	Not outcome of interest
4557	李春昱.加味半夏白术天麻饮联合缬沙坦治疗痰湿壅盛型原发性高血压病 112 例观察[J].中国民间疗法,2017,25(01):66-67.DOI:10.19621/j.cnki.11-3555/r.2017.01.059.	Not outcome of interest
4558	张翼翔,袁嘉东,吴智兵.清热祛湿化瘀法对糖尿病高胆固醇血症患者 OX-LDL、sICAM-1 的影响[J].江西中医药,2006,(07):31-32.	Not outcome of interest

4559	冉颖卓,张钟爱.清热燥湿健脾法治疗 2 型糖尿病及胰岛素抵抗患者 23 例临床观察[J].中医杂志,2004,(07):522-524.DOI:10.13288/j.11-2166/r.2004.07.028.	Not outcome of interest
4560	冉颖卓,高曌.清热燥湿健脾方药对早期 2 型糖尿病胰岛素抵抗及TNF-α的影响[J].中国中医药信息杂志,2009,16(09):14-15+20.	Not outcome of interest
4561	郑杰.清热燥湿健脾中药治疗湿热困脾证初发 2 型糖尿病的临床研究[J].中医学报,2013,28(09):1350-1351.DOI:10.16368/j.issn.1674-8999.2013.09.011.	Not outcome of interest
4562	张德贵,崔晓燕,宋学芳,等.泻肝利湿方治疗湿热型 2 型糖尿病早期临床观察[J].山西中医,2016,32(02):43-44+48.	Not outcome of interest
4563	马恒.中医辨证治疗糖尿病 49 例[J].中国中医药现代远程教育,2014,12(01):32-33.	Not outcome of interest
4564	周生花.化痰祛瘀开窍法治疗阻塞性睡眠呼吸暂停低通气综合征 60 例[J].中医研究,2006,(07):32-34.	Not outcome of interest
4565	佟喆,李川洁,方业明.中药治疗痰湿体质阻塞型睡眠呼吸暂停综合征合并高血压的疗效观察[J].中西医结合心脑血管病杂志,2017,15(15):1854-1856.	Not outcome of interest
4566	Ke B, Shi L, Jun-jie Z, Chen DS, Meng J, Qin J. Protective effects of modified linggui zhugan decoction combined with short-term very low calorie diets on cardiovascular risk factors in obese patients with impaired glucose tolerance. J Tradit Chin Med. 2012;32(2):193-198. doi:10.1016/s0254-6272(13)60010-2	Not outcome of interest
4567	Thomas A, Bankar N, Nagore D, Kothapalli L, Chitlange S. Herbal Oils for Treatment of Chronic and Diabetic Wounds: A Systematic Review. Curr Diabetes Rev. 2022;18(2):e220321192406. doi:10.2174/1573399817666210322151700	Not RCTs
4568	Guo M, Liu Y, Gao ZY, Shi DZ. Chinese herbal medicine on dyslipidemia: progress and perspective. Evid Based Complement Alternat Med. 2014;2014:163036. doi:10.1155/2014/163036	Not RCTs
4569	毛珍.SIRT1 在单纯性肥胖症患者中的表达意义及加味二陈汤干预的实验研究[D].湖北中医药大学,2022.DOI:10.27134/d.cnki.ghbzc.2022.000032.	Not RCTs
4570	Zhou, S., Gao, Y., & Chan, E. (2005). Clinical trials for medicinal mushrooms: experience with Ganoderma lucidum (W. Curt.: Fr.) Lloyd (Lingzhi mushroom). International Journal of Medicinal Mushrooms, 7(1&2).	Not RCTs
4571	Wu SX, Dong ZY. Diverse combination therapies of Chinese Medicine in treating Hypertension. Curr Vasc Pharmacol. 2015;13(4):504-519. doi:10.2174/1570161112666141014152214	Not RCTs
4572	Sereno AB, Dayane Pinto C, Antunes Andrade F, et al. Effects of okra (Abelmoschus esculentus (L.) Moench) on glycemic markers in animal models of diabetes: A systematic review. J Ethnopharmacol. 2022;298:115544. doi:10.1016/j.jep.2022.115544	Not RCTs

4573	Rouhi-Boroujeni H, Heidarian E, Rouhi-Boroujeni H, Deris F, Rafieian-Kopaei M. Medicinal Plants with Multiple Effects on Cardiovascular Diseases: A Systematic Review. Curr Pharm Des. 2017;23(7):999-1015. doi:10.2174/1381612822666161021160524	Not RCTs
4574	占永立,李秀英,李深,等.17 例肾性恶性高血压临床与病理分析[J].中国中西医结合肾病杂志,2006,(02):101-103.	Not RCTs
4575	叶励民.2 型糖尿病合并慢性胃病 100 例中医治疗体会[J].基层医学论坛,2014,18(35):4847-4848.	Not RCTs
4576	徐萌,张丰华,陈继兰,等.30年间老中医治疗糖尿病验案104例处方分析[J].现代中医药,2013,33(05):90-93.DOI:10.13424/j.cnki.mtcm.2013.05.036.	Not RCTs
4577	Zareisedehizadeh S, Tan CH, Koh HL. A review of botanical characteristics, traditional usage, chemical components, pharmacological activities, and safety of Pereskia bleo (Kunth) DC. Evid Based Complement Alternat Med. 2014;2014:326107.	Not RCTs
4578	Patle D, Vyas M, Khatik GL. A review on natural products and herbs used in the management of diabetes. Curr Diabetes Rev. 2021;17(2):186-197.	Not RCTs
4579	Zhu W, Du Y, Meng H, Dong Y, Li L. A review of traditional pharmacological uses, phytochemistry, and pharmacological activities of Tribulus terrestris. Chem Cent J. 2017;11:60.	Not RCTs
4580	Tadesse TY, Zeleke MM, Dagnew SB, Addis GT. A systematic review of ethnobotanical, phytochemical, and ethnopharmacological studies of Urtica simensis (stinging nettle). J Exp Pharmacol. 2023;15:177-186.	Not RCTs
4581	Rouhi-Boroujeni H, Rouhi-Boroujeni H, Heidarian E, Mohammadizadeh F, Rafieian-Kopaei M. Herbs with anti-lipid effects and their interactions with statins as a chemical anti-hyperlipidemia group drugs: a systematic review. ARYA Atheroscler. 2015;11(4):244-251.	Not RCTs
4582	Pandey, A and Singh, S, 2016, Aloe Vera: A Systematic Review of its Industrial and Ethno-Medicinal Efficacy, International Journal of Pharmaceutical Research and Allied Sciences	Not RCTs
4583	Ota A, Ulrih NP. An overview of herbal products and secondary metabolites used for management of type two diabetes. Front Pharmacol. 2017;8:436.	Not RCTs
4584	Wang P, Xu Q, Sun Q, Fan FF, Guo XR, Guo F. Assessment of the reporting quality of randomized controlled trials on the treatment of diabetes mellitus with traditional Chinese medicine: a systematic review. PLoS One. 2013;8(7):e70586.	Not RCTs
4585	Zhao MM, Lu J, Li S, et al. Berberine is an insulin secretagogue targeting the KCNH6 potassium channel. Nat Commun. 2021;12:5616.	Not RCTs
4586	Zhang DY, Cheng YB, Guo QH, et al. Treatment of masked hypertension with a Chinese herbal formula: a randomized, placebo-controlled trial. Circulation. 2020;142:1821-1830.	Not RCTs
4587	Peng W, Qin R, Li X, Zhou H. Botany, phytochemistry, pharmacology, and potential application of Polygonum cuspidatum Sieb. et Zucc.: a review. J Ethnopharmacol. 2013;148(3):729-745.	Not RCTs

4588	Benoutman A, Bencheikh N, Ouahhoud S, Elbouzidi A, Bnouham M. Caralluma europaea (Guss) N.E.Br.: a review on ethnomedicinal uses, phytochemistry, pharmacological activities, and toxicology. J Ethnopharmacol. 2021;273:113769.	Not RCTs
4589	Wang, Y., Li, L., & He, B. R. (2001). CEA comprehensive evaluation for Western and traditional Chinese hypotensive drugs. Zhongguo Zhong xi yi jie he za zhi Zhongguo Zhongxiyi Jiehe Zazhi= Chinese Journal of Integrated Traditional and Western Medicine, 21(9), 663-666.	Not RCTs
4590	Wang X, Xu G, Liu H, et al. Inhibiting apoptosis of Schwann cells under the high-glucose condition: a promising approach to treat diabetic peripheral neuropathy using Chinese herbal medicine. Biomed Pharmacother. 2023;157:114059.	Not RCTs
4591	Zeng X, Zheng Y, Liu Y, Su W, et al. Chemical composition, quality control, pharmacokinetics, pharmacological properties and clinical applications of Fufang Danshen Tablet: a systematic review. J Ethnopharmacol. 2021;278:114310.	Not RCTs
4592	Xinke Z, Yingdong L, Mingxia F, et al. Chinese herbal medicine for the treatment of primary hypertension: a methodology overview of systematic reviews. Syst Rev. 2016;5(1):180. Published 2016 Oct 20. doi:10.1186/s13643-016-0353-y	Not RCTs
4593	Pan, B., Hanzhou, L. I., Zhang, H., Yimeng, L. I., Wen, W., & Lyu, S. (2022). Chinese Medicine Regulating TGF-尾 1/Smad Signaling Pathway in Treatment of Diabetic Nephropathy: A Review.聽Chinese Journal of Experimental Traditional Medical Formulae,聽 28(23), 237-249.	Not RCTs
4594	Wang Y, Dai Z, Wang Q, et al. Clinical Application of Traditional Chinese Medicine Therapy for Type 2 Diabetes Mellitus: An Evidence Map. Evid Based Complement Alternat Med. 2022;2022:2755332. Published 2022 Jul 19. doi:10.1155/2022/2755332	Not RCTs
4595	Pang, B., Li, Q	Not RCTs
4596	Wong, L. Y., Leung, P. C., Wong, W. N., Wong, W. C., Lau, T. W., & Cheng, K. F. (2010). Clinical research on diabetic foot ulcers: demonstration of a comprehensive methodology. Journal of Complementary and Integrative Medicine, 7(1).	Not RCTs
4597	Posadzki, P., Lee, M. S., & Ernst, E. (2012). Complementary and alternative medicine for diabetes mellitus: An overview of systematic reviews. Focus on Alternative and Complementary Therapies, 17(3), 142-148.	Not RCTs
4598	Nong YB, Lin Q, Duan WH, Yang H. Zhongguo Zhong Xi Yi Jie He Za Zhi. 2004;24(9):781-784.	Not RCTs
4599	Ng, J. Y., Verma, K., & Gilotra, K. (2022). Corrigendum to “CAM recommendations in type 2 diabetes clinical practice guidelines: A systematic review” [Eur. J. Integr. Med. 48C (2021)]. European Journal of Integrative Medicine, 50, 102118.	Not RCTs

4600	Rayate AS, Nagoba BS, Mumbre SS, Mavani HB, Gavkare AM, Deshpande AS. Current scenario of traditional medicines in management of diabetic foot ulcers: A review. World J Diabetes. 2023;14(1):1-16. doi:10.4239/wjd.v14.i1.1	Not RCTs
4601	Rezaei A, Farzadfard A, Amirahmadi A, Alemi M, Khademi M. Diabetes mellitus and its management with medicinal plants: A perspective based on Iranian research. J Ethnopharmacol. 2015;175:567-616. doi:10.1016/j.jep.2015.08.010	Not RCTs
4602	Parildar H, Serter R, Yesilada E. Diabetes mellitus and phytotherapy in Turkey. J Pak Med Assoc. 2011;61(11):1116-1120.	Not RCTs
4603	Sridhar, S. N. C., Kumari, S., & Paul, A. T. (2014). Diabetic complications: A natural product perspective. Pharmaceutical Crops, 5(1).	Not RCTs
4604	Ugbogu, E. A., Dike, E. D., Uche, M. E., Etumnu, L. R., Okoro, B. C., Ugbogu, O. C., ... & Iweala, E. J. (2023). Ethnomedicinal uses, nutritional composition, phytochemistry and potential health benefits of Carica papaya. Pharmacological Research-Modern Chinese Medicine, 7, 100266.	Not RCTs
4605	Qiao LJ, Li B, Wang YX, Zhu MJ. Zhongguo Zhong Yao Za Zhi. 2022;47(19):5375-5382. doi:10.19540/j.cnki.cjcmm.202206024.501	Not RCTs
4606	Yang J, Ren X, Fu GJ, et al. Zhongguo Zhong Yao Za Zhi. 2022;47(14):3943-3949. doi:10.19540/j.cnki.cjcmm.20220401.502	Not RCTs
4607	Wang X, Ma Y, Xu Q, et al. Flavonoids and saponins: What have we got or missed?. Phytomedicine. 2023;109:154580. doi:10.1016/j.phymed.2022.154580	Not RCTs
4608	Zhang X, Zhang L, Zhang B, et al. Herbal tea, a novel adjuvant therapy for treating type 2 diabetes mellitus: A review. Front Pharmacol. 2022;13:982387. Published 2022 Sep 30. doi:10.3389/fphar.2022.982387	Not RCTs
4609	Tan, E., & Faller, E. (2022). Lipid lowering effects of herbal supplements: A review. Research Journal of Pharmacy and Technology, 15(1), 270-278.	Not RCTs
4610	Mollazadeh H, Mahdian D, Hosseinzadeh H. Medicinal plants in treatment of hypertriglyceridemia: A review based on their mechanisms and effectiveness. Phytomedicine. 2019;53:43-52. doi:10.1016/j.phymed.2018.09.024	Not RCTs
4611	Parvez, A., Rahman, M. M., Faysal, M., Munna, M., & Faruk, M. (2020). Natural Plants as the Source of Effective Antihypertensive Agents-a Review. PharmacologyOnline, 3, 30-49.	Not RCTs
4612	Yang J, Sun Y, Cao F, Yang B, Kuang H. Natural Products from Physalis alkekengi L. var. franchetii (Mast.) Makino: A Review on Their Structural Analysis, Quality Control, Pharmacology, and Pharmacokinetics. Molecules. 2022;27(3):695. Published 2022 Jan 21. doi:10.3390/molecules27030695	Not RCTs

461 3	Wang Y, Wang L, Liu Y, Li K, Zhao H. Network Analyses Based on Machine Learning Methods to Quantify Effects of Peptide-Protein Complexes as Drug Targets Using Cinnamon in Cardiovascular Diseases and Metabolic Syndrome as a Case Study. Front Genet. 2021;12:816131. Published 2021 Dec 24. doi:10.3389/fgene.2021.816131	Not RCTs
461 4	Wang L, Ge J, Chen Y, et al. Predictors for the prognosis and recurrence of ischaemic stroke among young Chinese patients: a cohort study. BMJ Open. 2022;12(5):e052289. Published 2022 May 2. doi:10.1136/bmjopen-2021-052289	Not RCTs
461 5	Yuan, J., Zhou, Y., Wang, X., Huang, S., Gao, H., & DU, L. (2023). Prevention and Treatment of Diabetic Peripheral Neuropathy by Chinese Medicine Through PI3K/Akt Signaling Pathway: A Review. Chinese Journal of Experimental Traditional Medical Formulae, 203-212.	Not RCTs
461 6	Zhong, L., Shi, C., Hou, Q., Yang, R., Li, M., & Fu, X. (2022). Promotive effects of four herbal medicine ARCC on wound healing in mice and human. Health Science Reports, 5(3), e494.	Not RCTs
461 7	Ng JY, Verma KD, Gilotra K. Quantity and quality of complementary and alternative medicine recommendations in clinical practice guidelines for type 2 diabetes mellitus: A systematic review. Nutr Metab Cardiovasc Dis. 2021;31(11):3004-3015. doi:10.1016/j.numecd.2021.07.029	Not RCTs
461 8	Zhu B, Qi F, Wu J, et al. Red Yeast Rice: A Systematic Review of the Traditional Uses, Chemistry, Pharmacology, and Quality Control of an Important Chinese Folk Medicine. Front Pharmacol. 2019;10:1449. Published 2019 Dec 2. doi:10.3389/fphar.2019.01449	Not RCTs
461 9	Su K, Zhu F, Guo L, Zhu Y, Li W, Xiong X. Retrospective study on Professor Zhongying Zhou's experience in Traditional Chinese Medicine treatment on diabetic nephropathy. J Tradit Chin Med. 2013;33(2):262-267. doi:10.1016/s0254-6272(13)60137-5	Not RCTs
462 0	San, A, 2018, Saffron and its effects on kidneys, Nephrology Dialysis Transplantation	Not RCTs
462 1	Wang, X. L., Liu, C. X., & Zheng, X. K. (2018, March). SELAGINELLA FLAVONOIDS SUPPRESS VCAM-1 AND E-SELECTIN EXPRESSION IN TNF-alpha-TREATED HUMAN UMBILICAL VEIN ENDOTHELIAL CELLS BY BLOCKING NF-kappa B ACTIVATION. In BASIC & CLINICAL PHARMACOLOGY & TOXICOLOGY (Vol. 122, pp. 53-53). 111 RIVER ST, HOBOKEN 07030-5774, NJ USA: WILEY.	Not RCTs
462 2	Mukherjee, I and Verma, N, 2019, Standardization and efficacy of polyherbal formulation on lipid profile in type 2 diabetes mellitus (T2DM) patients, International Journal of Diabetes in Developing Countries	Not RCTs
462 3	Xia X, Weng J. Targeting metabolic syndrome: candidate natural agents. J Diabetes. 2010;2(4):243-249. doi:10.1111/j.1753-0407.2010.00090.x	Not RCTs

4624	Yu X, Xu L, Zhou Q, et al. The Efficacy and Safety of the Chinese Herbal Formula, JTTZ, for the Treatment of Type 2 Diabetes with Obesity and Hyperlipidemia: A Multicenter Randomized, Positive-Controlled, Open-Label Clinical Trial. Int J Endocrinol. 2018;2018:9519231. Published 2018 Apr 1. doi:10.1155/2018/9519231	Not RCTs
4625	Pang, B, Lian, FM, Tong, XL, Zhao, TY, Zhao, XY, Phd, HC and Zhang, C, 2017, The efficacy and safety of Chinese herbal medicine Jiang Zhuo Formula in metabolic syndrome patients with dyslipidemia: A randomized, placebo-controlled trial, BMC Complementary and Alternative Medicine	Not RCTs
4626	Shi, Y., Mon, A. M., Fu, Y., Zhang, Y., Wang, C., Yang, X., & Wang, Y. (2018). The genus Ficus (Moraceae) used in diet: Its plant diversity, distribution, traditional uses and ethnopharmacological importance. Journal of ethnopharmacology, 226, 185-196.	Not RCTs
4627	Seca, A. M., Grigore, A., Pinto, D. C., & Silva, A. M. (2014). The genus Inula and their metabolites: From ethnopharmacological to medicinal uses. Journal of ethnopharmacology, 154(2), 286-310.	Not RCTs
4628	Xiao SL, Guan LJ, Jiang RF, Wang XG, Li X, Cai W. The Metabolism and Pharmacokinetics of Rhein and Aurantio-Obtusin. Curr Drug Metab. 2020;21(12):960-968. doi:10.2174/1389200221666200719002128	Not RCTs
4629	Xueding, J., & Bangcai, W. (2013). The origin and the clinical use of „Decoction of Gentian to drain the Liver "(o. hepaticus, longdan xiegan tang). Chinesische Medizin/Chinese Medicine, 28, 221-230.	Not RCTs
4630	Wang, Y., Chek, W., Lee, W., Tsai, H., Lin, S., & Lin, H. (2016). The Risks of Using Chinese Medications Associated with Cardiovascular Disease, Diabetes Mellitus, and Chronic Kidney Disease-A Systematic Review. Value in Health, 19(7), A870-A871.	Not RCTs
4631	Zhang LL, He Y, Sheng F, et al. Towards a better understanding of Fagopyrum dibotrys: a systematic review. Chin Med. 2021;16(1):89. Published 2021 Sep 16. doi:10.1186/s13020-021-00498-z	Not RCTs
4632	Saad B, Azaizeh H, Said O. Tradition and perspectives of arab herbal medicine: a review. Evid Based Complement Alternat Med. 2005;2(4):475-479. doi:10.1093/ecam/neh133	Not RCTs
4633	Tao, F, Jin, X, Fei, X, Yang, X, Liu, X, Gao, Y, Liu, Z, Hou, R, Xu, P, Yao, Z and Ding, X, 2019, Traditional Chinese medicine intervention based on herbal formula XRHZ may contribute to managing body weight in Chinese simple obese patients, Obesity Facts	Not RCTs
4634	Tola MA, Ibrahim F, Melak H, Tafesse T, Alemayehu M, Nigussie G. Traditional Herbal Remedies in the Management of Metabolic Disorders in Ethiopia: A Systematic Review of Ethnobotanical Studies and Pharmacological Activities. Evid Based Complement Alternat Med. 2023;2023:1413038. Published 2023 Jan 12. doi:10.1155/2023/1413038	Not RCTs
4635	Rastogi S, Pandey MM, Rawat AK. Traditional herbs: a remedy for cardiovascular disorders. Phytomedicine. 2016;23(11):1082-1089. doi:10.1016/j.phymed.2015.10.012	Not RCTs

4636	Mao T, Zhang J, Qiao Y, Liu B, Zhang S. Uncovering Synergistic Mechanism of Chinese Herbal Medicine in the Treatment of Atrial Fibrillation with Obstructive Sleep Apnea Hypopnea Syndrome by Network Pharmacology. Evid Based Complement Alternat Med. 2019;2019:8691608. Published 2019 Dec 23. doi:10.1155/2019/8691608	Not RCTs
4637	王洪生,徐海,李达鹏,等.从MEBO治愈 102 例糖尿病足浅析创面处理与治疗规范[J].中国烧伤创疡杂志,2015,27(01):17-57.	Not RCTs
4638	支英豪,胡万华,张云霞,等.高血压病中医干预与脑卒中的相关性研究[J].浙江中医杂志,2011,46(02):104-105.	Not RCTs
4639	隋歌川.化浊祛湿方通过SREBP-2 调节胆固醇代谢的机制研究[D].北京中医药大学,2016.	Not RCTs
4640	夏中尚,杜正彩,侯小涛,等.基于 755 首中药处方治疗糖尿病用药规律的研究[J].中草药,2018,49(03):739-744.	Not RCTs
4641	杨柳媛,许陆达,林欢,等.基于数据挖掘分析中医药治疗糖尿病合并高血压病的用药规律[J].中医临床研究,2021,13(12):51-55.	Not RCTs
4642	尹佳伟.基于数据挖掘探讨中药治疗糖尿病泌汗异常的用药规律[D].天津中医药大学,2022.DOI:10.27368/d.cnki.gtzyy.2022.000128.	Not RCTs
4643	苏雪芬,李先涛,王燕萍,等.基于文献研究痰瘀互结型高脂血症常用中药治疗规律及其系统评价[J].中药新药与临床药理,2018,29(04):520-527.DOI:10.19378/j.issn.1003-9783.2018.04.025.	Not RCTs
4644	赵进东,忻凌,余婵娟,等.基于中药复方干预湿热证 2 型糖尿病临床研究分析中药应用规律[J].时珍国医国药,2020,31(08):2022-2024.	Not RCTs
4645	王杰,张洪义.张洪义教授从肝脾论治肥胖症验案举隅[J].内蒙古中医药,2017,36(01):26-27.DOI:10.16040/j.cnki.cn15-1101.2017.01.025.	Not RCTs
4646	孙梦华,章轶立,谢雁鸣,等.真实世界中使用脉血康胶囊的老年人群特征及用药分析[J].世界中医药,2020,15(20):3088-3093.	Not RCTs
4647	牟方政,郑邦本,余宗洋,等.郑邦本常用对药举隅[J].河南中医,2018,38(11):1658-1661.DOI:10.16367/j.issn.1003-5028.2018.11.0444.	Not RCTs
4648	王娅.中药治疗原发性高血压的药物筛选及作用机制研究[D].中国中医科学院,2020.DOI:10.27658/d.cnki.gzzyy.2020.000169.	Not RCTs
4649	梅君.中医治疗 2 型糖尿病用药规律分析[J].中国实验方剂学杂志,2012,18(10):290-291.DOI:10.13422/j.cnki.syfjx.2012.10.012.	Not RCTs
4650	Liu ZL, Liu JP, Zhang AL, et al. Chinese herbal medicines for hypercholesterolemia. Cochrane Database Syst Rev. 2011;(7):CD008305. Published 2011 Jul 6. doi:10.1002/14651858.CD008305.pub2	Not RCTs
4651	Liu, Z., Tao, F., Chen, Y., & Gao, W. (2022). The XOWi May Contribute to Managing Body Weight in Chinese People With Obesity at 52 Weeks. Obesity, 30, 94-95.	Not RCTs
4652	Lian, F, Tian, J and Tong, X, 2016, Chinese herbal medicine in the treatment of type 2 diabetes mellitus (T2DM): Importance of symptom-based strategy of dosage selection, Journal of Alternative and Complementary Medicine	Not RCTs

4653	Lenga, S, Wouessidjewe, D, Foutse, Y and Nguimatsia, F, 2022, Knowledge, perception, and management of diabetes mellitus by traditional practitioners: A descriptive survey from Mifi division, Cameroon, Pharmacy Education	Not RCTs
4654	Ke J, Li MT, Xu S, Ma J, Liu MY, Han Y. Advances for pharmacological activities of Polygonum cuspidatum - A review. Pharm Biol. 2023;61(1):177-188. doi:10.1080/13880209.2022.2158349	Not RCTs
4655	Kasali FM, Kadima JN, Peter EL, et al. Antidiabetic Medicinal Plants Used in Democratic Republic of Congo: A Critical Review of Ethnopharmacology and Bioactivity Data. Front Pharmacol. 2021;12:757090. Published 2021 Oct 27. doi:10.3389/fphar.2021.757090	Not RCTs
4656	Kanjanahattakij N, Kwankhao P, Vathesatogkit P, et al. Herbal or traditional medicine consumption in a Thai worker population: pattern of use and therapeutic control in chronic diseases. BMC Complement Altern Med. 2019;19(1):258. Published 2019 Sep 18. doi:10.1186/s12906-019-2652-z	Not RCTs
4657	Jiang M, Zhang C, Cao H, Chan K, Lu A. The role of Chinese medicine in the treatment of chronic diseases in China. Planta Med. 2011;77(9):873-881. doi:10.1055/s-0030-1270983	Not RCTs
4658	Jia W, Gao W, Tang L. Antidiabetic herbal drugs officially approved in China. Phytother Res. 2003;17(10):1127-1134. doi:10.1002/ptr.1398	Not RCTs
4659	Jia M, Lu Y, Liang X, et al. Development of a core outcome set for hypertensive intracerebral hemorrhage in clinical trials of traditional Chinese medicine: a study protocol. Trials. 2022;23(1):871. Published 2022 Oct 12. doi:10.1186/s13063-022-06801-z	Not RCTs
4660	Jaffe SE, Patterson DR. Treating sleep problems in patients with burn injuries: practical considerations. J Burn Care Rehabil. 2004;25(3):294-305. doi:10.1097/01.bcr.0000124793.99886.6a	Not RCTs
4661	Islam, M. T. (2018). A literature-based phytochemical evidence and biological activities of Trichosanthes dioica Roxb. Oriental Pharmacy and Experimental Medicine, 18, 77-85.	Not RCTs
4662	Huo J, Liu LS, Jian WY, et al. Stationary Treatment Compared with Individualized Chinese Medicine for Type 2 Diabetes Patients with Microvascular Complications: Study Protocol for a Randomized Controlled Trial. Chin J Integr Med. 2018;24(10):728-733. doi:10.1007/s11655-018-2987-1	Not RCTs
4663	Hummelsberger, J., & Luguang, Q. (2012). Possible treatment for diabetes mellitus and diabetic foot syndrome using Chinese medicine. Chinesische Medizin/Chinese Medicine, 27, 191-206.	Not RCTs
4664	Hughes, G. D., Aboyade, O. M., Okonji, C. O., Clark, B., & Mabweazara, S. Z. (2021). Comparison of the prevalence of non-communicable diseases and traditional herbal medicine use in urban and rural communities in South Africa. Advances in Integrative Medicine, 8(2), 136-143.	Not RCTs

4665	Hughes GD, Aboyade OM, Clark BL, Puoane TR. The prevalence of traditional herbal medicine use among hypertensives living in South African communities. BMC Complement Altern Med. 2013;13:38. Published 2013 Feb 18. doi:10.1186/1472-6882-13-38	Not RCTs
4666	Huang, Y. Y., Fan, W. J., Zhu, J. J., Zhan, L., & Chen, W. (2012). DB1 epidemiology, treatment and economic burden of type 2 diabetes mellitus with nephropathy complications in China. Value in Health, 15(7), A603.	Not RCTs
4667	Hu, J., Shi, K., & Meng, Q. (2016). Mapping the knowledge of international Chinese medicines treatment on type 2 diabetes: A biblimetrical study. Journal of Traditional Chinese Medical Sciences, 3(4), 263-272.	Not RCTs
4668	Heydari M, Homayouni K, Hashempur MH, Shams M. Topical Citrullus colocynthis (bitter apple) extract oil in painful diabetic neuropathy: A double-blind randomized placebo-controlled clinical trial. J Diabetes. 2016;8(2):246-252. doi:10.1111/1753-0407.12287	Not RCTs
4669	He, L, Wang, H, He, X, Gu, C, Zhang, Y, Zhao, L and Tong, X, 2017, Blinding, easier said than done: Experiences from trials of Chinese Herbal Medicine, BMC Complementary and Alternative Medicine	Not RCTs
4670	Guevara SV, Feicán EA, Peláez I, et al. Prevalence of Rheumatic Diseases and Quality of Life in the Saraguro Indigenous People, Ecuador: A Cross-sectional Community-Based Study. J Clin Rheumatol. 2020;26(7S Suppl 2):S139-S147. doi:10.1097/RHU.0000000000001131	Not RCTs
4671	Guan, HJ, Han, S, Wang, YN, Li, CM, Zheng, LY and Shi, LW, 2017, Pharmacoeconomic evaluation of Shenyankangfu tablets plus conventional therapy in treating diabetic ephropathy, Chinese Journal of New Drugs	Not RCTs
4672	Feng, L., Su, J., Chi, R., Zhu, Q., Lv, S., & Liang, W. (2019). Effect of amlodipine besylate combined with acupoint application of traditional Chinese medicine nursing on the treatment of renal failure and hypertension by the PI3K/AKT pathway. International Journal of Molecular Medicine, 43(4), 1900-1910.	Not RCTs
4673	Fei, Y., Zhang, E. W., Sang, C., Wang, G. S., Zhang, G. Q., & Fei, H. B. (2017). GW28-e0284 Comparison Treatment Effect of WenXinKeLi and ShenSongYangXin in Patients with Atrial Fibrillation-Based on Meta-analysis. Journal of the American College of Cardiology, 70(16S), C132-C132.	Not RCTs
4674	Farrar WS, Fyfe-Johnson AL, Baechler CJ, Dusek JA. Spontaneous normal sinus rhythm conversion using integrative medicine in atrial fibrillation. Glob Adv Health Med. 2012;1(2):22-25. doi:10.7453/gahmj.2012.1.2.006	Not RCTs
4675	Dai XY, Zi MJ, Liu CX, Wang YM, Gao R. Development of a core outcome set in the clinical trials of traditional Chinese medicine for diabetic foot: A study protocol. Front Med (Lausanne). 2022;9:1025833. Published 2022 Nov 9. doi:10.3389/fmed.2022.1025833	Not RCTs

4676	Chrysant SG. The clinical significance and costs of herbs and food supplements used by complementary and alternative medicine for the treatment of cardiovascular diseases and hypertension. J Hum Hypertens. 2016;30(1):1-6. doi:10.1038/jhh.2015.42	Not RCTs
4677	Chevallier H, Herpin F, Kergosien H, Ventura G, Allaert FA. A Graded Approach for Evaluating Health Claims about Plant-Based Food Supplements: Application of a Case Study Methodology. Nutrients. 2021;13(8):2684. Published 2021 Aug 2. doi:10.3390/nu13082684	Not RCTs
4678	Chen, J, 2019, Evidence Review, Evaluation and Development for Chinese Herbal Medicine on Type 2 Diabetes Mellitus, Advances in Integrative Medicine	Not RCTs
4679	Chen CL, Venketasubramanian N, Lee CF, Wong KS, Bousser MG; CHIMES Study Investigators. Effects of MLC601 on early vascular events in patients after stroke: the CHIMES study. Stroke. 2013;44(12):3580-3583. doi:10.1161/STROKEAHA.113.003226	Not RCTs
4680	Chan, K. W., Kwong, A. S. K., Chan, G. C. W., Leung, C. P. S., Yiu, W. H., Lui, S. L., ... & Tang, S. C. W. (2019). Semi-individualised Chinese Medicine Treatment for Diabetic Kidney Disease－From users' perspectives to SCHEMATIC trial interim result and potential mechanisms. In 14th International Congress on Complementary Medicine Research (ICCMR0219). Elsevier BV. The Journal's web site is located at http://www. elsevier. com/locate/issn/22129588.	Not RCTs
4681	Chan, K. W., Chan, G. C. W., Yiu, W. H., Cheung, H. M., Chan, L. Y. Y., Leung, J. C. K., ... & Tang, S. C. W. (2020, October). INTEGRATIVE CHINESE-WESTERN MEDICINE TREATMENT FOR DIABETIC KIDNEY DISEASE: SCHEMATIC PRAGMATIC RCT INTERIM RESULT AND POTENTIAL MECHANISMS. In NEPHROLOGY (Vol. 25, pp. 66-66). 111 RIVER ST, HOBOKEN 07030-5774, NJ USA: WILEY.	Not RCTs
4682	Chadwick, A., Ash, A., Day, J., & Borthwick, M. (2015). Accidental overdose in the deep shade of night: a warning on the assumed safety of 'natural substances'. Case Reports, 2015, bcr2015209333.	Not RCTs
4683	Cao, Y., Yan, M., Zhao, H., Zhang, B., & Li, P. (2015). The Efficacy of Traditional Chinese Medicine on Diabetic Kidney Disease with Dyslipidemias: A Meta-analysis. Hong Kong Journal of Nephrology, 17(2), S8.	Not RCTs
4684	Wang Y, Cao HJ, Wang LQ, et al. The effects of Chinese herbal medicines for treating diabetic foot ulcers: A systematic review of 49 randomized controlled trials. Complement Ther Med. 2019;44:32-43. doi:10.1016/j.ctim.2019.03.007	Not RCTs
4685	Cao, H., Wang, A., & Liu, J. (2019). The clinical effects of health education of Chinese medicine on common non-communicable diseases: a systematic review of randomized controlled trials. Advances in Integrative Medicine, 6, S87.	Not RCTs

4686	Bocharova OA, Ionov NS, Kazeev IV, et al. Computer-aided Evaluation of Polyvalent Medications' Pharmacological Potential. Multiphytoadaptogen as a Case Study. Mol Inform. 2023;42(1):e2200176. doi:10.1002/minf.202200176	Not RCTs
4687	Bian ZX, Moher D, Dagenais S, et al. Improving the quality of randomized controlled trials in Chinese herbal medicine, part II: control group design. Zhong Xi Yi Jie He Xue Bao. 2006;4(2):130-136. doi:10.3736/jcim20060205	Not RCTs
4688	Bao Y, Han X, Liu D, Tan Z, Deng Y. Gut microbiota: The key to the treatment of metabolic syndrome in traditional Chinese medicine - a case study of diabetes and nonalcoholic fatty liver disease. Front Immunol. 2022;13:1072376. Published 2022 Dec 23. doi:10.3389/fimmu.2022.1072376	Not RCTs
4689	Arentz S, Hunter J, Deed G. Integrating Traditional and Complementary Medicine Recommendations into Clinical Practice Guidelines for People with Diabetes in Need of Palliative and End-of-Life Care: A Scoping Review. J Altern Complement Med. 2020;26(7):571-591. doi:10.1089/acm.2020.0028	Not RCTs
4690	Cosmetic Ingredient Review Expert Panel. Final report on the safety assessment of AloeAndongensis Extract, Aloe Andongensis Leaf Juice,aloe Arborescens Leaf Extract, Aloe Arborescens Leaf Juice, Aloe Arborescens Leaf Protoplasts, Aloe Barbadensis Flower Extract, Aloe Barbadensis Leaf, Aloe Barbadensis Leaf Extract, Aloe Barbadensis Leaf Juice,aloe Barbadensis Leaf Polysaccharides, Aloe Barbadensis Leaf Water, Aloe Ferox Leaf Extract, Aloe Ferox Leaf Juice, and Aloe Ferox Leaf Juice Extract. Int J Toxicol. 2007;26 Suppl 2:1-50. doi:10.1080/10915810701351186	Not RCTs
4691	Wu S, Kutlubaev MA, Chun HY, et al. Interventions for post-stroke fatigue. Cochrane Database Syst Rev. 2015;2015(7):CD007030. Published 2015 Jul 2. doi:10.1002/14651858.CD007030.pub3	Not RCTs
4692	Al-Aboudi A, Afifi FU. Plants used for the treatment of diabetes in Jordan: a review of scientific evidence. Pharm Biol. 2011;49(3):221-239. doi:10.3109/13880209.2010.501802	Not RCTs
4693	刘佳敏,李晋宏,丹丹.从脾肾阳虚探讨真武汤加减治疗糖尿病肾病及验案举隅[J].中医药临床杂志 ,2020,32(09):1674-1677.DOI:10.16448/j.cjtcm.2020.0921.	Not RCTs
4694	方朝晖,吴以岭,赵进东.糖尿病周围神经病变中医临床诊疗指南(2016 年版)[J].中医杂志 ,2017,58(07):625-630.DOI:10.13288/j.11-2166/r.2017.07.025.	Not RCTs
4695	Systolic hypertension in the elderly: Chinese trial (Syst-China). Interim report. Zhonghua Xin Xue Guan Bing Za Zhi. 1992;20(5):270-5, 323.	Not RCTs
4696	Biswas D, Mandal S, Chatterjee Saha S, et al. Ethnobotany, phytochemistry, pharmacology, and toxicity of Centella asiatica (L.) Urban: A comprehensive review. Phytother Res. 2021;35(12):6624-6654.	Not RCTs

4697	Cai H, Guo Y, Zhao Z, Chen Y, Zhao S, Chen B. Banxia Baizhu Tianma decoction for hyperlipidemia: protocol for a systematic review and meta-analysis. Medicine (Baltimore). 2018;97(44):e13067.	Not RCTs
4698	Chen Z, Wang L, Yang G, Xu H, Liu J. Chinese herbal medicine combined with conventional therapy for blood pressure variability in hypertension patients: a systematic review of randomized controlled trials. Evid Based Complement Alternat Med. 2015;2015:582751.	Not RCTs
4699	Fang, R, Zhou, Y, Yu, MK, Chen, KF, Yang, Y, Liu, P, Lin, HY, Chai, L, Li, SY, Xu, WF, Mei, ZG and Ge, JW, 2021, Traditional Chinese medicine syndrome differentiation combined anti-hypertensive drugs intervened hypertensive early renal damage patients: A systematic review and Meta-analysis of randomized clinical trials, Chinese Traditional and Herbal Drugs	Not RCTs
4700	Furman BL, Candasamy M, Bhattamisra SK, Veettil SK. Reduction of blood glucose by plant extracts and their use in the treatment of diabetes mellitus; discrepancies in effectiveness between animal and human studies. J Ethnopharmacol. 2020;247:112264.	Not RCTs
4701	Garang Z, Feng Q, Luo R, et al. Commiphora mukul (Hook. ex Stocks) Engl.: Historical records, application rules, phytochemistry, pharmacology, clinical research, and adverse reaction. J Ethnopharmacol. 2023;317:116717.	Not RCTs
4702	Lee B, Han K, Park HJ, et al. Efficacy of Hwangryunhaedok-tang (Huang-lian-jie-du-tang, Oren-gedoku-to) for patients with hyperlipidemia: a study protocol for a randomized, double-blind, placebo-controlled, parallel, investigator-initiated clinical trial. Trials. 2020;21(1):750.	Not RCTs
4703	Lee B, Jeong YE, Park HJ, et al. Effects of Sihogayonggolmoryeo-tang (Saikokaryukotsuboreito or Chai-Hu-Jia-Long-Gu-Mu-Li-Tang) for insomnia disorder with prehypertension or stage 1 hypertension: a study protocol for a randomized controlled trial. Medicine (Baltimore). 2020;99(29):e20980.	Not RCTs
4704	Lengnan X, Ban Z, Wang H, et al. Tripterygium wilfordii Hook F treatment for stage IV diabetic nephropathy: protocol for a prospective, randomized controlled trial. Biomed Res Int. 2020;2020:9181037.	Not RCTs
4705	Li X, Liu H, Feng H, et al. Acupuncture paired with herbal medicine for prediabetes: study protocol for a randomized controlled trial. Trials. 2017;18(1):297.	Not RCTs
4706	Liu JP, Zhang M, Wang WY, Grimsgaard S. Chinese herbal medicines for type 2 diabetes mellitus. Cochrane Database Syst Rev. 2004;(3):CD003642.	Not RCTs
4707	Liu Y, Liu Y, Yang J, et al. Chinese herbal medicine for hypertension complicated with hyperlipidemia: a protocol for a systematic review and meta-analysis. Medicine (Baltimore). 2021;100(6):e24345.	Not RCTs
4708	Luo H, Xiong M, Zhu W, Shen T. Erchen decoction for hyperlipemia: protocol for a systematic review and meta-analysis. Medicine (Baltimore). 2020;99(42):e22374.	Not RCTs

4709	Sun Y, Hu N, Chen G, et al. Efficacy and safety of Qushi Huayu granule for hyperlipidemia: study protocol for a randomized, double-blind, placebo-controlled trial. Trials. 2022;23(1):104.	Not RCTs
4710	Wang L, Huang X, Yue R, et al. Large dosage Huanglian (Rhizoma Coptidis) for T2DM: a protocol of systematic review and meta-analysis of randomized clinical trials. Medicine (Baltimore). 2020;99(38):e22066.	Not RCTs
4711	Wang S, Yue R, Huang X, Li L, Xu C, Liu L. Renshen (Panax ginseng) and Huanglian (Rhizoma Coptidis) for T2DM: a protocol of systematic review and meta-analysis of randomized clinical trials. Medicine (Baltimore). 2021;100(2):e23743.	Not RCTs
4712	Yang M, Hu Z, Yue R. Effect of pueraria, scutellaria, and coptis decoction for type 2 diabetes: a systematic review and meta-analysis protocol. Medicine (Baltimore). 2020;99(16):e19770.	Not RCTs
4713	Zhong M, Song X, Zhang X, et al. Treatment of microcirculation dysfunction in type 2 diabetic mellitus with Shenqi compound prescription: a protocol of systematic review and meta-analysis of randomized clinical trials. Medicine (Baltimore). 2020;99(41):e22347.	Not RCTs
4714	陈俊杰.基于Meta分析法近 20 年经方治疗糖尿病及并发症临床文献整理[D].广州中医药大学,2013.	Not RCTs
4715	李伯英,何海洲.养阴化浊通络汤联合甲钴胺治疗糖尿病周围神经炎 43 例[J].山东中医杂志 ,2012,31(07):479-481.DOI:10.16295/j.cnki.0257-358x.2012.07.041.	Not RCTs
4716	马瑜. (2015). 基于 "脾胃中轴" 理论中药干预 IGT 的系统评价及临床观察 (Doctoral dissertation, 广州中医药大学).	Not RCTs
4717	王尧,宋莉丽.中药复方治疗糖尿病大血管病变用药规律系统综述[J].实用中医内科杂志,2015,29(11):1-3.DOI:10.13729/j.issn.1671-7813.2015.11.01.	Not RCTs
4718	Di YM, Sun L, Lu C, et al. Benefits of herbal formulae containing Poria cocos (Fuling) for type 2 diabetes mellitus: A systematic review and meta-analysis. PLoS One. 2022;17(12):e0278536. Published 2022 Dec 1. doi:10.1371/journal.pone.0278536	Not RCTs
4719	邢渊,陈亮.黄连解毒汤联合西药对 2 型糖尿病疗效的Meta分析[J].中医临床研究,2021,13(09):52-59+84.	Not RCTs
4720	Hu Z, Yang M, Liu Y, et al. Effect of Huang-Lian Jie-Du Decoction on Glucose and Lipid Metabolism in Type 2 Diabetes Mellitus: A Systematic Review and Meta-Analysis. Front Pharmacol. 2021;12:648861. Published 2021 Apr 29. doi:10.3389/fphar.2021.648861	Not RCTs
4721	郝志婧.健脾化湿法干预糖尿病前期(脾虚痰湿证)的理论与临床研究[D].天津中医药大学,2020.DOI:10.27368/d.cnki.gtzyy.2020.000429.	Not RCTs
4722	郭珊,万璇璇,徐筱玮.健脾化浊中药治疗 2 型糖尿病合并高尿酸血症的Meta分析及启示[J].湖北中医杂志,2021,43(06):58-62.	Not RCTs
4723	庄琪.健脾祛湿法治疗 2 型糖尿病疗效水平的meta-分析[D].黑龙江中医药大学,2020.DOI:10.27127/d.cnki.ghlzu.2020.000170.	Not RCTs

4724	Ryuk JA, Lixia M, Cao S, Ko BS, Park S. Efficacy and safety of Gegen Qinlian decoction for normalizing hyperglycemia in diabetic patients: A systematic review and meta-analysis of randomized clinical trials. Complement Ther Med. 2017;33:6-13. doi:10.1016/j.ctim.2017.05.004	Not RCTs
4725	Ren L, Cheng Y, Qin F. Herbal Formula Gegen-Qinlian Decoction for Type 2 Diabetes Mellitus: A Meta-Analysis of Randomized Controlled Trials. Evid Based Complement Alternat Med. 2020;2020:3907920. Published 2020 Oct 21. doi:10.1155/2020/3907920	Not RCTs
4726	Pan L, Zhai X, Duan Z, Xu K, Liu G. Systematic review and meta-analysis of Coptis chinensis Franch.-containing traditional Chinese medicine as an adjunct therapy to metformin in the treatment of type 2 diabetes mellitus [published correction appears in Front Pharmacol. 2023 Jan 04;13:1112418. doi: 10.3389/fphar.2022.1112418.]. Front Pharmacol. 2022;13:956313. Published 2022 Sep 8. doi:10.3389/fphar.2022.956313	Not RCTs
4727	程越,赖倚文,高天舒.中药复方治疗湿热困脾型 2 型糖尿病临床疗效Meta分析[J].中医药临床杂志,2018,30(02):264-269.DOI:10.16448/j.cjtcm.2018.0081.	Not RCTs
4728	朱梅,鞠建庆,李运伦.半夏白术天麻汤治疗痰湿壅盛型原发性高血压随机对照试验系统评价[J].山东中医药大学学报,2014,38(02):105-108.DOI:10.16294/j.cnki.1007-659x.2014.02.004.	Not RCTs
4729	Lin J, Wang Q, Xu S, et al. Banxia baizhu tianma decoction, a Chinese herbal formula, for hypertension: Integrating meta-analysis and network pharmacology. Front Pharmacol. 2022;13:1025104. Published 2022 Dec 2. doi:10.3389/fphar.2022.1025104	Not RCTs
4730	Xiong XJ, Yang XC, Liu W, et al. Therapeutic Efficacy and Safety of Traditional Chinese Medicine Classic Herbal Formula Longdanxiegan Decoction for Hypertension: A Systematic Review and Meta-Analysis. Front Pharmacol. 2018;9:466. Published 2018 May 8. doi:10.3389/fphar.2018.00466	Not RCTs
4731	Effectiveness and safety of Hwangryunhaedok-Tang (Huang-Lian-Jie-Du-Tang, Oren-Gedoku-to) for dyslipidemia: A protocol for a PRISMA-compliant systematic review and meta-analysis: Erratum. Medicine (Baltimore). 2021;100(4):e24546. doi:10.1097/MD.0000000000024546	Not RCTs
4732	Liu ZL, Li GQ, Bensoussan A, Kiat H, Chan K, Liu JP. Chinese herbal medicines for hypertriglyceridaemia. Cochrane Database Syst Rev. 2013;2013(6):CD009560. Published 2013 Jun 6. doi:10.1002/14651858.CD009560.pub2	Not RCTs
4733	Huang J, Zhao L, Sun J, et al. Clinical Evidence and Potential Mechanisms of Complementary Treatment of Ling Gui Zhu Gan Formula for the Management of Serum Lipids and Obesity. Evid Based Complement Alternat Med. 2022;2022:7714034. Published 2022 May 9. doi:10.1155/2022/7714034	Not RCTs

4734	Yao H, Zhang Z, Wang J, et al. Efficacy and safety of Yinchenwuling powder for hyperlipidemia: a systematic review and Meta-analysis. J Tradit Chin Med. 2016;36(2):135-143. doi:10.1016/s0254-6272(16)30019-x	Not RCTs
4735	王荣宏.半夏白术天麻汤联合温胆汤治疗痰湿壅盛型高血压 44 例临床分析[J].中外医学研究 ,2018,16(29):41-42.DOI:10.14033/j.cnki.cfmr.2018.29.017.	Not RCTs
4736	田美慧.健脾开郁方干预超重/肥胖糖尿病前期（脾虚痰湿证）的前瞻性队列研究[D].长春中医药大学,2022.DOI:10.26980/d.cnki.gcczc.2022.000275.	Not RCTs
4737	Ke, Y., Pu, J., & Zheng, J. (2013). Essential hypertension treated by wuling powder and modified tianma gouteng decoction: A cohort study without controls. Complementary Therapies in Medicine, 21(6), 609-612.	Not RCTs
4738	林绍志,刘炳国,魏守宽.糖尿病从痰湿论治——附 35 例 II 型糖尿病病例分析[J].上海中医药杂志 ,1999,(02):8-9.DOI:10.16305/j.1007-1334.1999.02.003.	Not RCTs
4739	姜雪,李国林.半夏白术天麻汤与温胆汤治疗原发性高血压痰湿壅盛证临床观察[J].光明中医,2021,36(15):2565-2567.	Not RCTs
4740	肖倩倩,张福利.基于湿热论治单纯舒张期高血压 67 例[J].黑龙江医学 ,2014,38(09):1014-1015.	Not RCTs
4741	郑彩云.加味半夏泻心汤治疗原发性高血压病痰湿壅盛证 32 例[J].光明中医,2008,(11):1690.	Not RCTs
4742	高凤涛,刘珊珊.二陈汤加减治疗高血脂症的临床观察[J].中国医药指南 ,2013,11(05):287-288.DOI:10.15912/j.cnki.gocm.2013.05.050.	Not RCTs
4743	代艳, 王益平, 李素莲, 周仲芳, & 冯莉. (2015). 痰湿质高脂血症患者中医体质辨体施护效果分析. 中国实用护理杂志, 31(17), 1301-1303.	Not RCTs
4744	杨秀梅.燥湿化痰汤干预对糖耐量减低伴高脂血症血脂水平的影响[J].山东中医杂志 ,2013,32(08):556-557.DOI:10.16295/j.cnki.0257-358x.2013.08.016.	Not RCTs
4745	程鹏,张继玉.化痰祛浊活血汤结合穴位埋线治疗单纯性肥胖痰湿型 68 例[J].实用中医药杂志,2020,36(01):37-38.	Not RCTs
4746	Shergis JL, Zhang AL, Zhou W, Xue CC. Panax ginseng in randomised controlled trials: a systematic review. Phytother Res. 2013;27(7):949-965. doi:10.1002/ptr.4832	Methodological error
4747	Yan, M and Li, P, 2018, Randomized placebo-controlled trials of tangshen formula in diabetic kidney disease, Journal of the American Society of Nephrology	Methodological error
4748	Li, Y., Bai, M., & Miao, M. (2020, February). Based on Data Mining, the Law and Characteristics of Traditional Chinese Medicine in Treating Diabetes Mellitus were Analyzed. In BASIC & CLINICAL PHARMACOLOGY & TOXICOLOGY (Vol. 126, pp. 127-127). 111 RIVER ST, HOBOKEN 07030-5774, NJ USA: WILEY.	Methodological error
4749	Kumar, SVA, 2017, H1: Evidence for the use of ayurvedic herbs for the management of diabetic retinopathy, Medical Journal of Malaysia	Methodological error

4750	Khorasani, S., Azizi, H., Yousefi, M., Salari, R., Bahrami-Taghanaki, H., & Behravanrad, P. (2017). An evidence based review on integrative medicine in weight control. Complementary Medicine Journal, 7(1), 1828-1850.	Methodological error
4751	Wang, X and Yao, N, 2017, Effect of Herb formula Xiao-Zhi-Hua-Xian-Tang against nonalcoholic steatohepatitis with advanced fibrosis: A preliminary clinical study, Hepatology International	Methodological error
4752	Jin D, Hou L, Han S, et al. Corrigendum: Basis and Design of a Randomized Clinical Trial to Evaluate the Effect of Jinlida Granules on Metabolic Syndrome in Patients With Abnormal Glucose Metabolism. Front Endocrinol (Lausanne). 2020;11:649. Published 2020 Sep 3. doi:10.3389/fendo.2020.00649	Duplicates
4753	Hassani, S. S., Fallahi, A., Esmaeili, S. S., & Gholami Fesharaki, M. (2019). The effect of combined therapy with fenugreek and nutrition training based on Iranian traditional medicine on FBS, HgA1c, BMI, and waist circumference in Type 2 diabetic patients: a randomized double blinded clinical trial. Journal of Advances in Medical and Biomedical Research, 27(120), 37-42.	Duplicates
4754	胡清,复方紫草油局部湿敷治疗糖尿病足的临床研究.山东省,滕州市工人医院,2006-06-01.	Duplicates
4755	Zeng YP, Huang YS, Hu YG. Zhongguo Zhong Xi Yi Jie He Za Zhi. 2006;26(6):514-520.	Duplicates
4756	NNOCHIRI E. A case of juvenile diabetes mellitus complicated by treatment with herbs. West Afr Med J. 1961;10:58-60.	Failure to download
4757	Hao, L., Fieselmann, K., Schneider, S., Schlussel, Y., & Shapses, S. (2017). A double blind, randomized, placebo‐controlled study of Salacia Chinensis, with alpha‐glucosidase inhibitor properties, on post‐prandial glycemia. The FASEB Journal, 31, 643-3.	Failure to download
4758	Javaid, A., Omar, N., Khurshid, R., Ahmad, R., Shah, M. S. M., Zin, A. A. M., ... & Munir, I. (2021). A Review on the Medicinal Plants and Diabetes Mellitus. International Medical Journal, 28(6), 610-613.	Failure to download
4759	Gupta, S., & Mukherjee, M. (2014). Diabetes mellitus and its treatment with some traditional herbs from the different districts of West Bengal: A Review. Int J PharmTech Res, 6, 1941-9.	Failure to download
4760	Bao P, Mi J, Yu Z, et al. Efficacy and safety of acupuncture combined with Chinese herbal medicine in the treatment of type 2 diabetes mellitus: A protocol for a systematic review and meta-analysis. Medicine (Baltimore). 2021;100(43):e27658. doi:10.1097/MD.0000000000027658	Failure to download
4761	Moayyedkazemi, A., Amraei, M., Nejad, E. B., Moghaddam, A., Karami, K., & Baharvand, P. (2021). Hepatoprotective Effects of Herbal Medicines against Non-alcoholic Fatty Liver Disease: A Systematic Review of Clinical and In Vivo Studies. Current Traditional Medicine, 7(6), 42-49.	Failure to download

4762	Thompson Coon JS, Ernst E. Herbs for serum cholesterol reduction: a systematic view. J Fam Pract. 2003;52(6):468-478.	Failure to download
4763	Bahramsoltani, R, Farzaei, MH and Rahimi, R, 2017, Medicinal plants for atherosclerosis: A review of clinical studies, Iranian Journal of Pharmaceutical Sciences	Failure to download
4764	Vray M, Attali JR. Randomized study of glibenclamide versus traditional Chinese treatment in type 2 diabetic patients. Chinese-French Scientific Committee for the Study of Diabetes. Diabete Metab. 1995;21(6):433-439.	Failure to download
4765	Famuyiwa OO. The efficacy of traditional medicine in the management of diabetes mellitus in southwestern Nigeria. Afr J Med Med Sci. 1993;22(1):31-37.	Failure to download
4766	Miraj, S. (2016). Therapeutic effects of Rheum palmatum L.(Dahuang): A systematic review. Der Pharma Chemcia, 8(13), 50-54.	Failure to download
4767	Smith WM. Treatment of mild hypertension: results of a ten-year intervention trial. Circ Res. 1977;40(5 Suppl 1):I98-I105.	Failure to download
4768	莫霄云, 钱海凌, & 李丽. (2005). 活血化瘀利水中药复方逆转高血压左室肥厚的实验研究. 辽宁中医学院学报, 7(1), 69-70.	Failure to download
4769	骆天炯,张钟爱,高曌,等.超重 2 型糖尿病患者炎症因子与胰岛素抵抗的关系及加减四妙散对其影响的临床研究[J].时珍国医国药,2014,25(05):1141-1143.	Not outcome of interest
4770	周笑漪.葛根芩连汤联合利拉鲁肽对肥胖 2 型糖尿病湿热困脾证的临床疗效[J].天津中医药,2020,37(12):1363-1367.	Not outcome of interest
4771	曾艺鹏,黄云胜,胡蕴刚,等.葛根芩连汤配合胰岛素强化治疗湿热证 2 型糖尿病临床观察[J].中国中西医结合杂志,2006,(06):514-516+520.	Not outcome of interest
4772	宋亚一,董峰,周晓凤,等.化痰健脾方结合西药治疗 104 例痰湿体质型 2 型糖尿病患者临床观察[J].时珍国医国药,2019,30(01):118-120.	Not outcome of interest
4773	余晓琳,陈军平.黄连温胆汤治疗湿热困脾型初发 2 型糖尿病 78 例临床观察[J].新中医,2010,42(04):25-26.DOI:10.13457/j.cnki.jncm.2010.04.008.	Not outcome of interest
4774	晏和国,尹朝兰,赵一佳,等.黄芩滑石汤治疗湿热困脾型 2 型糖尿 60 例临床观察[J].中国民族民间医药,2019,28(07):100-102.	Not outcome of interest
4775	黄元珍,李忠志,陈永苹.藿朴夏苓汤加减治疗雅安地区 2 型糖尿病临床观察[J].河北中医,2017,39(08):1192-1195.	Not outcome of interest
4776	孟晓嵘,郑姜钦,李红,等.健脾化痰方治疗痰湿型 2 型糖尿病的疗效观察[J].中医药通报,2008,(01):61-63.	Not outcome of interest
4777	张会君.健脾利湿方联合利拉鲁肽治疗 42 例肥胖 2 型糖尿病患者的临床研究[J].黑龙江医药科学,2019,42(01):180-181.	Not outcome of interest
4778	李翔,朱世滨,任玲.健脾祛湿汤加二甲双胍治疗糖尿病的效果评估分析[J].辽宁中医杂志,2021,48(03):111-114.DOI:10.13192/j.issn.1000-1719.2021.03.032.	Not outcome of interest
4779	梁厚策,王松林.苓桂术甘汤+干预生活方式联合二甲双胍治疗痰湿壅盛糖尿病肥胖随机平行对照研究[J].实用中医内科杂志,2016,30(11):40-42.DOI:10.13729/j.issn.1671-7813.2016.11.17.	Not outcome of interest
4780	徐立然,张钟,郭建中,等.敏疏糖胶囊对 2 型糖尿病外周胰岛素抵抗患者血脂、血糖的影响[J].中医研究,2009,22(02):25-27.	Not outcome of interest

4781	王全兴,郑晓军,白雅彬.七味白术散合补阳还五汤对胰岛素抵抗的影响[J].中国卫生标准管理,2018,9(17):105-107.	Not outcome of interest
4782	吴波,隋淼,朱艳,等.三黄汤治疗痰湿热结型 2 型糖尿病 43 例[J].河南中医,2019,39(06):839-842.DOI:10.16367/j.issn.1003-5028.2019.06.0208.	Not outcome of interest
4783	Chen, D., Li, C., Michalsen, A., Kessler, C., Huang, Y., Meng, J., ... & Qin, J. (2012). Modified Ling-Gui-Zhu-Gan decoction combined with short-term fasting improves therapeutic response in type 2 diabetic patients. European Journal of Integrative Medicine, 4(3), e309-e314.	Not outcome of interest
4784	邓德强,张亚琼,马丹,等.消渴健脾胶囊对脾虚湿盛型 2 型糖尿病IR及TNF-α、IL-6 的影响[J].新疆中医药,2018,36(05):3-6.	Not outcome of interest
4785	贺红梅,李凯利.消渴健脾胶囊对维吾尔族 2 型糖尿病患者痰湿体质的干预研究[J].临床合理用药杂志,2014,7(12):115-117.DOI:10.15887/j.cnki.13-1389/r.2014.12.064.	Not outcome of interest
4786	张利民,谭毅,黄伟,等.小陷胸汤联合盐酸二甲双胍片治疗 2 型糖尿病痰湿蕴热型临床观察[J].中国中医药信息杂志,2014,21(02):32-34+38.	Not outcome of interest
4787	柯振梅,舒荣梅,郭春茂.燥湿化痰活血方治疗 2 型糖尿病合并高血脂临床分析[J].北方药学,2019,16(07):30-31.	Not outcome of interest
4788	杨秀梅.燥湿化痰汤干预对糖耐量减低伴高脂血症血脂水平的影响[J].山东中医杂志,2013,32(08):556-557.DOI:10.16295/j.cnki.0257-358x.2013.08.016.	Not outcome of interest
4789	贺红梅,李娜,刘晶.中西医结合治疗对脾虚湿盛型糖尿病患者超敏C反应蛋白相关性研究[J].云南中医中药杂志,2018,39(04):40-41.DOI:10.16254/j.cnki.53-1120/r.2018.04.015.	Not outcome of interest
4790	杨丹丹.中医中药治疗 2 型糖尿病患者的血糖波动对比分析[J].中国处方药,2016,14(03):90-91.	Not outcome of interest
4791	李永锋,王开娜.半夏白术天麻汤对痰湿内蕴型原发性高血压眩晕患者临床症状的改善效果[J].临床医学研究与实践,2021,6(15):137-139.DOI:10.19347/j.cnki.2096-1413.202115046.	Not outcome of interest
4792	史诚智.半夏白术天麻汤加味治疗痰湿壅盛证肥胖相关性高血压临床观察[J].广西中医药,2019,42(02):25-27.	Not outcome of interest
4793	黄浦.半夏白术天麻汤治疗 46 例老年痰湿壅盛型高血压病临床研究[J].实用中西医结合临床,2018,18(07):17-18.DOI:10.13638/j.issn.1671-4040.2018.07.008.	Not outcome of interest
4794	林秀敏.辨证分型联合卡托普利治疗高血压随机平行对照研究[J].实用中医内科杂志,2017,31(12):31-33.DOI:10.13729/j.issn.1671-7813.2017.12.11.	Not outcome of interest
4795	胡易池.辨证分型联合卡托普利治疗原发性高血压随机平行对照研究[J].实用中医内科杂志,2015,29(05):146-148.DOI:10.13729/j.issn.1671-7813.2015.05.71.	Not outcome of interest
4796	朱燕,孔秀琼,李伟利.二术二陈汤治疗岭南地区高血压痰湿壅盛证疗效观察[J].河南中医,2019,39(12):1886-1889.DOI:10.16367/j.issn.1003-5028.2019.12.0463.	Not outcome of interest
4797	孙阳,朱明军,李彬,等.复方鬼针草颗粒治疗 1 级高血压病湿热血瘀证患者的临床疗效[J].中国实验方剂学杂志,2021,27(22):100-107.DOI:10.13422/j.cnki.syfjx.20212291.	Not outcome of interest

4798	吴天敏,陈金水,薛文娟,等.化湿泻浊法治疗痰湿壅盛型中青年原发性高血压病及其证的内涵研究[J].光明中医,2020,35(16):2482-2486.	Not outcome of interest
4799	庞英华.化痰降浊法对痰湿壅盛型高血压病患者血浆同型半胱氨酸疗效观察[J].辽宁中医药大学学报,2013,15(05):189-190.DOI:10.13194/j.jlunivtcm.2013.05.191.pangyh.013.	Not outcome of interest
4800	方埼逍逍.祛湿汤治疗痰湿壅盛型单纯舒张期高血压随机平行对照研究[J].实用中医内科杂志,2016,30(01):35-36.DOI:10.13729/j.issn.1671-7813.2016.01.15.	Not outcome of interest
4801	程勇,陈海燕,黄文金,等.泽泻汤合二陈汤加味联合西药治疗痰湿壅盛型1、2级高血压35例[J].中外医学研究,2021,19(22):31-34.DOI:10.14033/j.cnki.cfmr.2021.22.011.	Not outcome of interest
4802	李红燕.中西医结合治疗原发性高血压疗效观察[J].现代中西医结合杂志,2013,22(19):2108-2110.	Not outcome of interest
4803	佟淑兰.中西医结合治疗原发性高血压临床观察[J].临床合理用药杂志,2011,4(10):74-75.DOI:10.15887/j.cnki.13-1389/r.2011.10.023.	Not outcome of interest
4804	王洪源,焦玲.半夏白术山楂汤治疗高脂血症86例临床观察[J].中医临床研究,2012,4(03):50+53.	Not outcome of interest
4805	陆新.葛根祛湿汤治疗高脂血症的临床疗效观察[J].山西中医,2004,(05):12-14.	Not outcome of interest
4806	孟辉,周永红,孙立,等.加味二陈汤治疗高脂血症82例[J].陕西中医,2004,(11):1000-1001.	Not outcome of interest
4807	谭嫚娜,林溢涛,张健池.健脾利湿、化痰祛瘀法治疗高脂血症40例临床观察[J].中医药导报,2006,(05):21-22+28.DOI:10.13862/j.cnki.cn43-1446/r.2006.05.009.	Not outcome of interest
4808	薛玉峰.降脂方联合辛伐他汀治疗高脂血症随机平行对照研究[J].实用中医内科杂志,2015,29(09):113-115.DOI:10.13729/j.issn.1671-7813.2015.09.50.	Not outcome of interest
4809	清化痰湿方防治动脉粥样硬化的机理研究	Not outcome of interest
4810	陆红,王婷婷,任雅萍.自拟降脂汤治疗高脂血症(痰湿血瘀型)的临床效果[J].上海医药,2017,38(10):24-26+40.	Not outcome of interest
4811	刘兴奎,吴华慧,隋志兰.利湿降脂汤治疗高脂血症临床疗效[J].中国实用医药,2008,(28):97-98.	Not outcome of interest
4812	玉山江,王先敏,何芳.祛湿化瘀通络方治疗高尿酸血症合并高脂血症的临床研究[J].辽宁中医杂志,2010,37(08):1531-1533.DOI:10.13192/j.ljtcm.2010.08.128.yushj.030.	Not outcome of interest
4813	李立荣.益气健脾化痰祛湿方治疗高脂血症的临床观察[J].内蒙古中医药,2013,32(27):31-32.DOI:10.16040/j.cnki.cn15-1101.2013.27.024.	Not outcome of interest
4814	李松伟,宰军华,王又红,等.减肥调脂胶囊对单纯性肥胖症(胃热湿阻证)的临床研究[J].新中医,2007,(02):28-29+8.DOI:10.13457/j.cnki.jncm.2007.02.019.	Not outcome of interest
4815	杨国伟.健脾祛湿化痰法治疗肥胖症24例[J].中国中医药现代远程教育,2010,8(07):28-29.	Not outcome of interest

4816	刘玲,陆西宛,何珂,等.七味白术散对脾虚湿困型肥胖症患者糖脂代谢及肠道菌群的影响[J].中医杂志,2020,61(23):2082-2086.DOI:10.13288/j.11-2166/r.2020.23.013.	Not outcome of interest
4817	叶丽芳,尚文斌,赵娟,等.三黄汤治疗单纯性肥胖症临床观察[J].南京中医药大学学报,2016,32(03):242-244.DOI:10.14148/j.issn.1672-0482.2016.0242.	Not outcome of interest
4818	Dai L, Xu J, Liu B, et al. Lingguizhugan Decoction, a Chinese herbal formula, improves insulin resistance in overweight/obese subjects with non-alcoholic fatty liver disease: a translational approach. Front Med. 2022;16(5):745-759. doi:10.1007/s11684-021-0880-3	Not outcome of interest
4819	Xu J, Lian F, Zhao L, et al. Structural modulation of gut microbiota during alleviation of type 2 diabetes with a Chinese herbal formula. ISME J. 2015;9(3):552-562. doi:10.1038/ismej.2014.177	Not outcome of interest
4820	马立明,吴新华,严妍.五黄养阴颗粒联合吡格列酮治疗 2 型糖尿病的临床研究[J].现代药物与临床,2020,35(01):122-126.	Not outcome of interest
4821	王遐,任建民,肖波,等.糖脂平汤颗粒冲服治疗 2 型糖尿病并脂代谢紊乱临床观察[J].山东医药,2014,54(43):86-87.	Not outcome of interest
4822	饶春燕,张祥,胡建华.降脂通脉胶囊对痰瘀互结型高脂血症患者的临床疗效观察[J].中成药,2015,37(06):1388-1390.	Not outcome of interest
4823	刘凤阁,陈静.降脂通脉胶囊联合阿托伐他汀钙治疗混合型高脂血症疗效观察[J].中国医药导报,2011,8(27):71-72.	Not outcome of interest
4824	尤秀梅.降脂通脉胶囊联合阿托伐他汀治疗高脂血症 60 例[J].河南中医,2015,35(12):3104-3106.DOI:10.16367/j.issn.1003-5028.2015.12.1335.	Not outcome of interest
4825	潘春奇,菅颖,刘善新.降脂通脉胶囊联合依折麦布片治疗高脂血症临床观察[J].新中医,2016,48(07):23-25.DOI:10.13457/j.cnki.jncm.2016.07.011.	Not outcome of interest
4826	黄洁,刘鹏, 2022，葛根芩连汤饮片治疗 2 型糖尿病的作用分析，女性健康	Not outcome of interest
4827	李华,裴启福.心舒宝胶囊联合阿托伐他汀治疗老年高血压合并高脂血症的临床研究[J].中西医结合心脑血管病杂志,2018,16(01):67-70.	Not outcome of interest
4828	陈露菲,李丽,林聪娣.葛根芩连汤辨证加减治疗在 2 型糖尿病患者血糖控制中的临床应用分析[J].糖尿病新世界,2022,25(16):185-188.DOI:10.16658/j.cnki.1672-4062.2022.16.185.	Not outcome of interest
4829	黄淑萍,张倩.葛根芩连汤辩证加减治疗对糖尿病患者血糖控制水平及临床疗效影响分析[J].糖尿病新世界,2021,24(21):35-38.DOI:10.16658/j.cnki.1672-4062.2021.21.035.	Not outcome of interest
4830	曾艺鹏,冯新格,谷成英,等.葛根芩连汤治疗对 2 型糖尿病湿热证肠道菌群影响[J].河北医学,2016,22(10):1731-1734.	Not outcome of interest
4831	李磊.葛根芩连汤对初发 2 型糖尿病患者的影响[J].光明中医,2020,35(08):1183-1184.	Not outcome of interest
4832	葛爱利.葛根芩连汤对湿热型 2 型糖尿病患者胰岛素抵抗的影响[J].北方药学,2018,15(11):43-44.	Not outcome of interest
4833	范尧夫,曹雯,胡咏新,等.葛根芩连汤对新发 2 型糖尿病胰岛素抵抗的影响研究[J].现代中西医结合杂志,2017,26(02):115-117+121.	Not outcome of interest

4834	吴利.葛根芩连汤加减治疗湿热中阻型 2 型糖尿病的临床研究[D].重庆医科大学,2021.DOI:10.27674/d.cnki.gcyku.2021.000186.	Not outcome of interest
4835	蒋磊.葛根芩连汤加减治疗胃肠湿热型老年 2 型糖尿病的疗效分析[J].糖尿病新世界,2023,26(06):72-75.DOI:10.16658/j.cnki.1672-4062.2023.06.072	Not outcome of interest
4836	倪青,倪炎炎,张美珍,等.葛根芩连汤加减治疗胃肠湿热型老年 2 型糖尿病的疗效观察[J].疑难病杂志,2021,20(06):569-573.	Not outcome of interest
4837	杨雪芹.葛根芩连汤加味联合二甲双胍治疗 2 型糖尿病湿热困脾证的临床研究[D].湖北中医药大学,2021.DOI:10.27134/d.cnki.ghbzc.2021.000196.	Not outcome of interest
4838	张慧芬,周宇清,钟钻仪.葛根芩连汤联合二甲双胍治疗湿热证 2 型糖尿病的疗效分析[J].中国社区医师,2019,35(05):128-129.	Not outcome of interest
4839	王齐有,李娜,陈玉.葛根芩连汤联合西格列汀治疗 2 型糖尿病的效果分析[J].医药前沿,2021,11(23):187-188.	Not outcome of interest
4840	张颖,蔡春沉.葛根芩连汤饮片治疗 2 型糖尿病的疗效观察[J].辽宁中医杂志,2016,43(04):783-785.DOI:10.13192/j.issn.1000-1719.2016.04.040.	Not outcome of interest
4841	张利宁,杨璐,魏志成.葛根芩连汤饮片治疗 2 型糖尿病的临床疗效评价[J].当代医学,2019,25(32):10-12.	Not outcome of interest
4842	郑金发.葛根芩连汤饮片治疗湿热证 2 型糖尿病的疗效观察[J].中医临床研究,2017,9(36):36-37.	Not outcome of interest
4843	朱亚歌.葛根芩连汤治疗 2 型糖尿病 60 例临床观察[J].中国民族民间医药,2018,27(01):114-115.	Not outcome of interest
4844	熊芹俊.葛根芩连汤治疗 2 型糖尿病的临床效果[J].临床医学研究与实践,2019,4(26):148-149.DOI:10.19347/j.cnki.2096-1413.201926063.	Not outcome of interest
4845	周霭.葛根芩连汤治疗 2 型糖尿病湿热困脾证临床研究[D].北京中医药大学,2012.	Not outcome of interest
4846	田艳.葛根芩连汤治疗糖尿病患者的临床疗效及血糖水平分析[J].中医临床研究,2020,12(36):68-69+100.	Not outcome of interest
4847	张美琴.葛根芩连汤治疗糖尿病患者的效果分析[J].中国医药指南,2019,17(34):179-180.DOI:10.15912/j.cnki.gocm.2019.34.137.	Not outcome of interest
4848	付耀华. (2016). 葛根芩连消渴方治疗湿热蕴脾型 2 型糖尿病的疗效观察 (Master's thesis, 广州中医药大学).	Not outcome of interest
4849	李宗桥.化浊益脾饮治疗痰湿内阻型 2 型糖尿病的临床研究[D].黑龙江中医药大学,2011.	Not outcome of interest
4850	王海源.黄连温胆汤辅助二甲双胍在老年 2 型糖尿病患者治疗中的应用价值[J].青海医药杂志,2022,52(07):51-53.	Not outcome of interest
4851	潘赏赏,李力,王娴.黄连温胆汤联合盐酸二甲双胍对湿热内蕴型早期 2 型糖尿病患者的临床疗效[J].中成药,2021,43(02):557-559.	Not outcome of interest
4852	陈鏷.黄连温胆汤联合盐酸二甲双胍缓释片对湿热内蕴型早期 2 型糖尿病患者的临床应用[J].智慧健康,2022,8(13):136-138.DOI:10.19335/j.cnki.2096-1219.2022.13.042.	Not outcome of interest
4853	王艳.黄连温胆汤在湿热内蕴型早期 2 型糖尿病患者中的临床应用[J].智慧健康,2022,8(07):124-127.DOI:10.19335/j.cnki.2096-1219.2022.07.039.	Not outcome of interest
4854	王明坤,程俐,赵鑫,等.黄连温胆汤治疗湿热蕴结型 2 型糖尿病临床研究[J].中西医结合研究,2021,13(06):369-372.	Not outcome of interest

4855	段公,加减抵挡汤治疗痰瘀型糖尿病的临床研究.河北省,大厂回族自治县中医院,2016-03-15.	Not outcome of interest
4856	程梦婕.加减葛根芩连汤联合二甲双胍治疗 2 型糖尿病湿热内蕴型疗效观察[D].湖南中医药大学,2018.	Not outcome of interest
4857	王磊.加味葛根芩连汤治疗新诊 2 型糖尿病痰（湿）热互结证临床研究[J].新中医,2021,53(12):16-20.DOI:10.13457/j.cnki.jncm.2021.12.004.	Not outcome of interest
4858	付朝红.加味黄连温胆汤对 2 型糖尿病痰湿互结症患者血糖及IL-6、IL-10、IL-12 水平的影响[J].现代医学与健康研究电子杂志,2021,5(04):62-64.	Not outcome of interest
4859	余臣祖,康学东,党晓娟,等.加味六君子汤对脾虚痰湿证 2 型糖尿病患者胰岛 β 细胞功能的影响[J].中国中医药信息杂志,2017,24(04):36-39.	Not outcome of interest
4860	张俪潆.清热利湿法治疗 2 型糖尿病湿热证的临床疗效观察[D].北京中医药大学,2016.	Not outcome of interest
4861	戴国令.探究采用中药葛根芩连汤治疗糖尿病患者的临床效果[J].糖尿病新世界,2022,25(11):1-4.DOI:10.16658/j.cnki.1672-4062.2022.11.001.	Not outcome of interest
4862	李爽,沈莺,赵娜.西药联合安糖饮对脾虚痰湿型 2 型糖尿病 42 例胰岛素抵抗及糖脂代谢的影响[J].中国民族民间医药,2020,29(01):97-99.	Not outcome of interest
4863	马丹.消渴健脾汤对 2 型糖尿病患者血糖及胰岛素影响临床研究[D].新疆医科大学,2016.	Not outcome of interest
4864	房露露.醒脾除陈汤治疗湿热困脾，痰气郁结型初发 2 型糖尿病的临床研究[D].山东中医药大学,2015.	Not outcome of interest
4865	孙华, 李春燕, & 薛金涛. (2019). 葛根的化学成分及药理作用研究进展. 新乡医学院学报, 36(11), 1097-1100.	Not outcome of interest
4866	刘亚平,胡梅林,张鸿婷,等.半夏白术天麻汤加减治疗中年原发性高血压疗效观察[J].中医药信息,2007,(01):27-28.	Not outcome of interest
4867	任亚国.半夏白术天麻汤治疗高血压临床效果观察[J].中西医结合心血管病电子杂志,2022,10(03):62-64.DOI:10.16282/j.cnki.cn11-9336/r.2022.03.039.	Not outcome of interest
4868	毛平,李芳.半夏白术天麻汤治疗痰湿壅盛型高血压病的疗效观察[J].内蒙古中医药,2022,41(11):20-21.DOI:10.16040/j.cnki.cn15-1101.2022.11.001.	Not outcome of interest
4869	王晨,杨威,孙芳,等.半夏白术天麻汤加减治疗原发性高血压病痰湿中阻证的临床研究[J].实用中医内科杂志,2023,37(03):91-94.DOI:10.13729/j.issn.1671-7813.Z20220415.	Not outcome of interest
4870	杨小娟.基于“脾胃升降”理论以李东垣半夏白术天麻汤加减治疗原发性高血压（痰浊上扰证）的临床疗效观察[D].天津中医药大学,2023.DOI:10.27368/d.cnki.gtzyy.2023.000318.	Not outcome of interest
4871	熊艳文.加味半夏白术天麻汤配合西药治疗痰湿壅盛型原发性高血压病 60 例的临床观察[J].中国中医药现代远程教育,2010,8(13):67-68.	Not outcome of interest
4872	黄志石,李海宁.半夏白术天麻汤联合卡托普利治疗肥胖伴高血压病的疗效观察[J].吉林医学,2014,35(28):6245-6246.	Not outcome of interest
4873	赵华云,黄嘉文,王文会,等.半夏白术天麻汤干预治疗肥胖型高血压疗效观察[J].辽宁中医药大学学报,2016,18(12):14-17.DOI:10.13194/j.issn.1673-842x.2016.12.004.	Not outcome of interest
4874	官家靓,陈晓红.半夏白术天麻汤加减对痰湿壅盛型高血压病患者血脂及同型半胱氨酸的影响[J].中药与临床,2016,7(05):42-44.	Not outcome of interest

4875	袁瑞华,吴清安.半夏白术天麻汤加味治疗高血压病 50 例[J].河南中医,2016,36(11):1919-1921.DOI:10.16367/j.issn.1003-5028.2016.11.0773.	Not outcome of interest
4876	高秋静,熊艳文,金周慧.加味四妙方治疗尿酸性肾病模型大鼠的实验研究[J].上海中医药杂志,2016,50(04):70-74.DOI:10.16305/j.1007-1334.2016.04.023.	Not outcome of interest
4877	苗灵娟,杨永枝,邢海燕,等.半夏白术天麻汤联合卡托普利治疗高血压临床疗效观察[J].内蒙古医学杂志,2017,49(05):601-604.DOI:10.16096/J.cnki.nmgyxzz.2017.49.05.041.	Not outcome of interest
4878	任敬丽.自拟化痰降脂汤治疗高脂血症 32 例疗效观察[J].云南中医中药杂志,2017,38(02):56-57.DOI:10.16254/j.cnki.53-1120/r.2017.02.023.	Not outcome of interest
4879	吴子君.半夏白术天麻汤辅助治疗原发性高血压的效果及对血清Hcy、CysC和UA水平的影响[J].慢性病学杂志,2019,20(07):1104-1106.DOI:10.16440/j.cnki.1674-8166.2019.07.052.	Not outcome of interest
4880	马惠倪,苏伟,夏晶晶,等.半夏白术天麻汤治疗原发性高血压风痰上扰证的临床研究[J].世界中西医结合杂志,2019,14(11):1579-1583.DOI:10.13935/j.cnki.sjzx.191124.	Not outcome of interest
4881	吴文妙.防风通圣丸治疗单纯性肥胖的临床疗效观察及对LP、APN的影响[D].黑龙江中医药大学,2016.	Not outcome of interest
4882	马春宇, 于洪宇, 王慧娇, 等. 苦瓜总皂苷对 2 型糖尿病大鼠降血糖作用机制的研究[J]. 天津医药, 2014, 42(4): 321-324.	Not outcome of interest
4883	阮家安,刘文洲,高峰,等.荷泽颗粒对单纯性肥胖症患者临床疗效观察[J].现代中医药,2019,39(06):80-83+86.DOI:10.13424/j.cnki.mtcm.2019.06.024.	Not outcome of interest
4884	Lenon, G. B., Li, K. X., Chang, Y. H., Yang, A. W., Da Costa, C., Li, C. G., ... & Xue, C. C. (2012). Efficacy and safety of a Chinese herbal medicine formula (RCM‐104) in the management of simple obesity: a randomized, placebo‐controlled clinical trial. Evidence‐Based Complementary and Alternative Medicine, 2012(1), 435702.	Not outcome of interest
4885	Zhao, J. V., Yeung, W. F., Chan, Y. H., Vackova, D., Leung, J. Y., Ip, D. K., ... & Schooling, C. M. (2021). Effect of berberine on cardiovascular disease risk factors: a mechanistic randomized controlled trial. Nutrients, 13(8), 2550.	Not outcome of interest
4886	Zhao, J. V., Yeung, W. F., Chan, Y. H., Vackova, D., Leung, J. Y., Ip, D. K., ... & Schooling, C. M. (2021). Effect of berberine on cardiovascular disease risk factors: a mechanistic randomized controlled trial. Nutrients, 13(8), 2550.	Not outcome of interest
4887	郑贝贝,魏敬,蒋建东,等.他汀类药物联合黄连素治疗高脂血症的疗效评估[J].南京医科大学学报(自然科学版),2009,29(11):1493-1497.	Not outcome of interest
4888	何甲均,徐学广,郑苇.辛伐他汀和盐酸小檗碱治疗高脂血症和颈动脉粥样硬化的疗效[J].天津医药,2007,(07):539-541.	Not outcome of interest
4889	苏晓叶,邢俊武,鞠娟,等.辛伐他汀联合黄连素治疗高脂血症 60 例[J].中国中医药现代远程教育,2012,10(21):41-42.	Not outcome of interest
4890	胡燕.茵陈五苓散利水渗湿治疗高脂血症[J].四川医学,2012,33(08):1456-1458.DOI:10.16252/j.cnki.issn1004-0501-2012.08.005.	Not outcome of interest

4891	陈方敏,方秋茹,李香香,等.葛根芩连汤治疗湿热型 2 型糖尿病患者服用二甲双胍后所致腹泻的疗效观察[J].内蒙古中医药,2022,41(10):20-21.DOI:10.16040/j.cnki.cn15-1101.2022.10.005.	Not outcome of interest
4892	刘云雅,叶文平,张捷.健脾活血汤治疗老年高血压病合高血脂 50 例疗效观察[J].湖南中医杂志,2016,32(08):64-66.DOI:10.16808/j.cnki.issn1003-7705.2016.08.028.	Not outcome of interest
4893	于俊英,李铮,王秋哲.小剂量辛伐他汀与黄连素联合治疗高脂血症 50 例观察[J].山东医药,2007,(22):39.	Not outcome of interest
4894	张娜.茵陈五苓散加味治疗糖尿病高脂血症[J].湖北中医杂志,2011,33(12):28.	Not outcome of interest
4895	刘亚来,于庆春,于俊英.茵陈五苓散治疗高脂血症患者 21 例疗效观察[J].山东医药,2004,(22):77.	Not outcome of interest

Table S3. The basic characteristics of individual studies

Study	Journal	Disease	Age,y, mean (sd)	Age range, y, max	Age range, y, min	No. sample size	No. Gender (Male/Female)	Diagnosis criteria	Disease course, y, mean (sd)	Disease course range, y, max	Disease course range, y, min	Disease stage
Chen DS 2012	European Journal of Integrative Medicine	Diabetes	52.1(13.19)	NR	NR	60	34/26	1999 WHO Diagnosis	4.00(1.70)	NR	NR	diabetes
Chen LF 2022	Diabetes New World	Diabetes	50.45(1.33)	65	35	162	83/79	Standardized Diagnosis and Treatment of Diabetes Mellitus Integrating Traditional Chinese and Western Medicine	5.46(1.16)	7	3	diabetes
Chen Y 2022	Smart Healthcare	Diabetes	57.23(3.42)	68	20	86	45/41	Chinese Guidelines for the Prevention and Treatment of Type 2 Diabetes Mellitus (2013)	6.03(1.48)	10	1	diabetes
Zeng YP 2006	Chinese Journal of Integrated Traditional and Western Medicine	Diabetes	54.63(9.96)	NR	NR	30	12/18	1999 WHO Diagnosis	6.85(2.43)	NR	NR	diabetes
Cheng MJ 2018	NR	Diabetes	54.68(6.08)	NR	NR	90	44/46	Chinese Guidelines for the Prevention and Treatment of Type 2 Diabetes Mellitus (2013)	NR	NR	NR	pre-diabete s
Cheng Y 2021	Chinese and Foreign Medical Research	Hypertension	46.04(2.87)	NR	NR	70	37/33	Chinese Guidelines for the Prevention and Treatment of Hypertension 2018	5.25(0.35)	13	1	stage 1, stage 2
Dai GL 2022	Diabetes New World	Diabetes	53.09(6.57)	72	45	78	45/33	Diagnostic Criteria for Type 2 Diabetes Mellitus in China by the Diabetes Branch of the Chinese Medical Association	4.65(0.69)	8	2	diabetes
Deng DQ 2018	Xinjiang Journal of Traditional Chinese Medicine	Diabetes	NR	NR	NR	64	NR/NR	Chinese Guidelines for the Prevention and Treatment of Type 2 Diabetes Mellitus (T2DM) 2013	NR	NR	NR	diabetes
Duan G 2015	Hebei Journal of Traditional Chinese Medicine	Diabetes	56.04(12.27)	69	21	156	79/77	Chinese Guidelines for the Prevention and Treatment of Type 2 Diabetes Mellitus (2010)	8.17(5.01)	17	1	diabetes
Fan YF 2017	Modern Journal of Integrated Traditional Chinese and Western Medicine	Diabetes	37.2(6.80)	NR	NR	70	40/30	1999 WHO Diagnosis	0.27(0.13)	NR	NR	diabetes
Fang LL 2015	NR	Diabetes	NR	NR	NR	60	31/29	Chinese Guidelines for the Prevention and Treatment of Type 2 Diabetes Mellitus (2013)	2.72(1.55)	NR	NR	diabetes
Fang QXX 2016	Journal of Practical Traditional Chinese Internal Medicine	Hypertension	43.00(1.84)	71	20	108	51/57	Chinese Guidelines for the Prevention and Treatment of Hypertension 2011	2.13(0.29)	4	0.333	NR
Feng XG 2016	World Journal of Integrated Traditional and Western Medicine	Diabetes	56.85(10.07)	80	27	110	66/44	1999 WHO Diagnosis	1.02(0.13)	NR	NR	diabetes

Study	Journal	Disease	Age,y, mean (sd)	Age range, y, max	Age range, y, min	No. sample size	No. Gender (Male/Female)	Diagnosis criteria	Disease course, y, mean (sd)	Disease course range, y, max	Disease course range, y, min	Disease stage
Fu YH 2017	NR	Diabetes	NR	75	35	81	43/38	Chinese Guidelines for the Prevention and Treatment of Type 2 Diabetes Mellitus (2013)	NR	NR	NR	diabetes
Fu ZH 2021	Modern Medicine and Health Research Electronic Journal	Diabetes	56.16(1.72)	71	19	120	61/59	Chinese Guidelines for the Prevention and Treatment of Type 2 Diabetes Mellitus (2013)	7.28(2.42)	11	1	diabetes
Ge AL 2018	Journal of North Pharmacy	Diabetes	50.68(7.20)	78	20	80	51/29	Chinese Guidelines for the Prevention and Treatment of Type 2 Diabetes Mellitus (2013)	1.13(0.26)	NR	NR	diabetes
George BL 2012	Evidence-Based Complementary and Alternative Medicine	Obesity	39.85(11.76)	60	18	117	20/97	BMI ≥ 30 kg/m2	NR	NR	NR	NR
Guan JL 2016	Pharmacy and Clinics of Chinese Materia Medica	Hypertension	63.20(6.60)	NR	NR	60	37/23	Chinese Guidelines for the Prevention and Treatment of Hypertension 2010	7.35(2.68)	15	2	NR
He HM 2014	Chinese Journal of Clinical Rational Drug Use	Diabetes	45.10(6.90)	NR	NR	61	27/34	1999 WHO Diagnosis	4.90(3.40)	NR	NR	diabetes
He HM 2018	Yunnan Journal of Traditional Chinese Medicine and Materia Medica	Diabetes	54.3(4.05)	NR	NR	64	35/29	Chinese Guidelines for the Prevention and Treatment of Type 2 Diabetes Mellitus (2013)	7.95(1.89)	NR	NR	diabetes
He JJ 2007	Tianjin Medical Journal	Dyslipidemia	55(9.25)	NR	NR	116	62/54	Suggestions for the Prevention and Treatment of Dyslipidemia (1997)	NR	NR	NR	NR
Hong XT 2007	Journal of clinical research	Dyslipidemia	NR	NR	NR	60	30/30	Practical Internal Medicine	NR	NR	NR	NR
Hu Y 2012	Sichuan Medical Journal	Dyslipidemia	43.35(3.79)	NR	NR	135	88/47	Chinese Guidelines for the Prevention and Treatment of Dyslipidemia in Adults (2007)	NR	25	0.42	NR
Hu YC 2015	Journal of Practical Traditional Chinese Internal Medicine	Hypertension	41.90(4.71)	75	26	80	39/41	Chinese Guidelines for the Prevention and Treatment of Hypertension 2011	0.77(0.18)	1	0.583	NR
Huang J 2022	Women's Health	Diabetes	59.67(7.26)	79	42	80	45/35	Chinese Guidelines for the Prevention and Treatment of Type 2 Diabetes Mellitus (2020)	8.20(2.33)	17	2	diabetes
Huang P 2018	Practical Clinical Journal of Integrated Traditional Chinese and Western Medicine	Hypertension	67.31(6.15)	82	60	91	52/39	Internal Medicine	7.40(3.02)	12	2	NR
Huang SP 2021	Diabetes New World	Diabetes	48.2(5.80)	NR	NR	70	37/33	Standardized Diagnosis and Treatment of Diabetes Mellitus Integrating Traditional Chinese and Western	4.95(1.36)	NR	NR	diabetes

Study	Journal	Disease	Age,y, mean (sd)	Age range, y, max	Age range, y, min	No. sample size	No. Gender (Male/Female)	Diagnosis criteria	Disease course, y, mean (sd)	Disease course range, y, max	Disease course range, y, min	Disease stage
								Medicine				
Huang YZ 2017	Hebei Journal of Traditional Chinese Medicine	Diabetes	50.04(9.05)	69	40	90	51/39	Chinese Guidelines for the Prevention and Treatment of Type 2 Diabetes Mellitus (2010)	8.83(1.99)	18.5	5	diabetes
Huang ZS 2014	Jilin Medical Journal	Hypertension	59.05(1.53)	67	35	110	58/52	Chinese Guidelines for the Prevention and Treatment of Hypertension 2004	4.95(0.62)	9.5	2	stage 1, stage 2
Jiang L 2023	Diabetes New World	Diabetes	73.53(3.67)	82	65	92	50/42	Chinese Guidelines for the Prevention and Treatment of Type 2 Diabetes Mellitus (2017)	4.03(0.65)	8	1	diabetes
Ke B 2012	Journal of Traditional Chinese Medicine	Obesity	46.10(7.37)	70	25	85	43/42	Overweight and Obesity in Chinese Adults (2003)	3.65(2.49)	NR	NR	NR
Ke ZM 2019	Journal of North Pharmacy	Diabetes	56.05(14.00)	72	37	64	35/29	1999 WHO Diagnosis	9.58(5.39)	13	4	diabetes
Kong WJ 2008	Metabolism Clinical and Experimental	Dyslipidemia	NR	NR	NR	63	NR/NR	TC＞5.2mmol/L	NR	NR	NR	NR
Liang HC 2016	Journal of Practical Traditional Chinese Internal Medicine	Diabetes	56.05(8.17)	64	45	76	39/37	Department of Noncommunicable Disease Surveillance, World Health Organization. Definition, Diagnosis, Classification of Diabetes Mellitus and Diabetic Complications	3.03(0.24)	5	2	diabetes
Li H 2018	Hunan Journal of Traditional Chinese Medicine	Diabetes	58.75(9.72)	NR	NR	96	54/42	Chinese Guidelines for the Prevention and Treatment of Type 2 Diabetes Mellitus (2013)	3.6(1.65)	NR	NR	diabetes
Li L 2020	Guangming Journal of Chinese Medicine	Diabetes	52.1(3.48)	65	40	88	45/43	Chinese Guidelines for the Prevention and Treatment of Type 2 Diabetes Mellitus (2017)	1.4(0.43)	2	1	diabetes
Li LR 2013	Inner Mongolia Journal of Traditional Chinese Medicine	Dyslipidemia	62.2(3.39)	66	43	84	61/23	Suggestions for the Prevention and Treatment of Dyslipidemia (1997)	NR	17	2	NR
Li S 2020	Chinese Journal of Ethnomedicine and Ethnopharmacy	Diabetes	41.16(3.14)	58	24	83	41/42	Chinese Guidelines for the Prevention and Treatment of Type 2 Diabetes Mellitus (2013)	2.59(0.65)	5	0.33	diabetes
Li SW 2007	New Chinese Medicine	Obesity	43.05(11.97)	NR	NR	50	21/29	Diagnostic and Efficacy Evaluation Criteria for Simple Obesity	NR	NR	NR	NR
Li X 2021	Liaoning Journal of Traditional Chinese Medicine	Diabetes	51.5(11.45)	79	32	108	57/51	Chinese Guidelines for the Prevention and Treatment of Type 2 Diabetes Mellitus (2017)	3.95(1.80)	9	0.5	diabetes
Li YF 2021	Clinical Research and Practice	Hypertension	57.17(5.19)	NR	NR	184	88/96	Chinese Guidelines for the Prevention and Treatment of Hypertension 2000	NR	NR	NR	stage 1, stage 2
Li ZQ 2011	NR	Diabetes	50.90(6.72)	NR	NR	60	36/24	1999 WHO Diagnosis	4.15(2.77)	NR	NR	diabetes
Lin XM 2017	Journal of Practical Traditional Chinese Internal Medicine	Hypertension	59.80(3.69)	79	42	90	53/37	Internal Medicine	4.05(0.26)	6	1	NR

Study	Journal	Disease	Age,y, mean (sd)	Age range, y, max	Age range, y, min	No. sample size	No. Gender (Male/Female)	Diagnosis criteria	Disease course, y, mean (sd)	Disease course range, y, max	Disease course range, y, min	Disease stage
Liu FG 2011	China Medical Herald	Dyslipidemia	52.0(9.6)	NR	NR	60	34/26	Chinese Guidelines for the Prevention and Treatment of Dyslipidemia in Adults (2007)	NR	NR	NR	NR
Liu L 2020	Journal of Traditional Chinese Medicine	Obesity	38.81(12.40)	65	18	34	14/20	Expert Consensus on the Prevention and Treatment of Obesity in Chinese Adults	10.08(8.10)	38	0.5	NR
Liu XK 2008	China Practical Medicine	Dyslipidemia	49.5/47.2	NR	NR	180	113/67	Guidelines for the Clinical Research of New Traditional Chinese Medicine for the Treatment of Hyperlipidemia	NR	NR	NR	NR
Liu XL 2016	Modern Journal of Integrated Traditional Chinese and Western Medicine	Hypertension	50.03(6.70)	72	38	86	46/40	Chinese Guidelines for the Prevention and Treatment of Hypertension 2010	9.07(0.98)	21	1	stage 1, stage 2, stage 3
Liu YP 2007	Information on Traditional Chinese Medicine	Hypertension	NR	NR	NR	80	NR/NR	1999 World Health Organization-International Society of Hypertension. Guidelines for the Treatment of Hypertension	NR	NR	NR	stage 1, stage 2
Liu Z 2014	Asia-Pacific Traditional Medicine	Hypertension	51.99(5.12)	78	33	130	71/59	Chinese Guidelines for the Prevention and Treatment of Hypertension 2005	NR	NR	NR	NR
Lu H 2017	Shanghai Medical & Pharmaceutical Journal	Dyslipidemia	58.53(9.55)	74	47	93	46/47	Chinese Guidelines for the Prevention and Treatment of Dyslipidemia in Adults (2007)	1.92(1.74)	NR	NR	NR
Lu X 2004	Shanxi Journal of Traditional Chinese Medicine	Dyslipidemia	53.5(10.69)	70	36	80	54/26	Suggestions for the Prevention and Treatment of Dyslipidemia (1997)	NR	NR	NR	NR
Lu JZ 2018	Chinese Journal of Ethnomedicine and Ethnopharmacy	Hypertension	54.75(7.11)	79	42	106	61/45	Internal Medicine	8.46(4.12)	18	0.5	NR
Luo TJ 2014	Lishizhen Medicine and Materia Medica Research	Diabetes	70.64(11.43)	75	30	124	79/45	1999 WHO Diagnosis	2.44(1.07)	4	0.33	diabetes
Ma D 2016	NR	Diabetes	44.95(6.52)	65	35	63	31/32	Chinese Guidelines for the Prevention and Treatment of Type 2 Diabetes Mellitus (2013)	3.30(1.69)	5	1	diabetes
Ma HN 2019	World Journal of Integrated Traditional and Western Medicine	Hypertension	54.19(12.56)	NR	NR	200	120/80	Chinese Guidelines for the Prevention and Treatment of Hypertension 2010	10.74(4.19)	NR	NR	stage 1, stage 2
Ma LM 2020	Drugs & Clinic	Diabetes	56.62(3.23)	71	41	96	NR/NR	Report of the WHO Diabetes Expert Committee (2005)	4.38(1.41)	9	1	diabetes
Mao P 2022	Inner Mongolia Journal of Traditional Chinese Medicine	Hypertension	51(7.50)	75	35	60	32/38	National Guidelines for the Prevention and Management of Hypertension at the Grassroots Level 2020 Edition	7.77(0.99)	NR	NR	stage 1, stage 2
Meng H 2004	Shaanxi Journal of Traditional	Dyslipidemia	49/50	73	31	157	89/68	If the lipid levels are detected twice within 2 weeks and exceed the normal	NR	19	1	NR

Study	Journal	Disease	Age,y, mean (sd)	Age range, y, max	Age range, y, min	No. sample size	No. Gender (Male/Female)	Diagnosis criteria	Disease course, y, mean (sd)	Disease course range, y, max	Disease course range, y, min	Disease stage
	Chinese Medicine							values of the clinical laboratory of our hospital (cholesterol 6.0mmol/L, triglyceride 1.7mmol/L, β-lipoprotein 4.9g/L) in 10% of the cases, the diagnosis can be made.				
Meng XR 2008	Traditional Chinese Medicine Journal	Diabetes	51.94(6.85)	NR	NR	59	33/26	1999 WHO Diagnosis	4.02(2.02)	9	0.17	diabetes
Miao LJ 2017	Inner Mongolia Medical Journal	Hypertension	56.52(6.35)	76	48	88	43/45	Chinese Guidelines for the Prevention and Treatment of Hypertension 2005	10.62(2.54)	30	3	stage 1, stage 2, stage 3
Ni Q 2021	Chinese Journal of Difficult and Complicated Cases	Diabetes	71.48(2.40)	81	60	61	29/32	1999 WHO Diagnosis	NR	NR	NR	diabetes
Pan CQ 2016	New Chinese Medicine	Dyslipidemia	64.05(8.70)	73	55	90	53/37	Chinese Guidelines for the Prevention and Treatment of Dyslipidemia in Adults (2007)	4.25(1.54)	8	1	NR
Pan SS 2021	Chinese Traditional Patent Medicine	Diabetes	50.64(5.45)	62	40	80	38/42	Chinese Guidelines for the Prevention and Treatment of Type 2 Diabetes Mellitus (2017)	4.22(0.23)	0.5	0.17	diabetes
Pang YH 2013	Journal of Liaoning University of Traditional Chinese Medicine	Hypertension	65.96(7.47)	75	45	76	58/18	Hypertension Diagnostic Criteria Set by the World Health Organization in 1999, Chinese Guidelines for the Prevention and Treatment of Hypertension 2010	7.61(2.63)	13	1	NR
Rao CY 2015	Chinese Traditional Patent Medicine	Dyslipidemia	54.5(10.93)	70	41	118	60/58	Chinese Guidelines for the Prevention and Treatment of Dyslipidemia in Adults (2007)	NR	NR	NR	NR
Ren JL 2017	Shanxi Journal of Traditional Chinese Medicine	Hypertension	59.99(6.26)	69	50	76	43/33	2003 WHO Hypertension Diagnostic Criteria	NR	NR	NR	NR
Ren YG 2022	Cardiovascular Disease Electronic Journal of Integrated Traditional Chinese and Western Medicine	Hypertension	40.68(1.76)	86	36	50	27/23	Blood pressure higher than 130/80 mmHg	NR	NR	NR	NR
Song YY 2019	Lishizhen Medicine and Materia Medica Research	Diabetes	50(10)	71	25	104	59/45	Chinese Guidelines for the Prevention and Treatment of Type 2 Diabetes Mellitus (2017)	NR	NR	NR	diabetes
Ruan JA 2019	Modern Chinese Medicine	Obesity	27.57(7.23)	52	20	117	31/76	Diagnostic and Efficacy Evaluation Criteria for Simple Obesity	NR	NR	NR	NR
Shi CZ 2019	Guangxi Journal of Traditional Chinese Medicine	Hypertension	53.34(8.24)	67	46	123	72/51	Chinese Guidelines for the Prevention and Treatment of Hypertension 2010	5.93(0.64)	7	5	stage 1, stage 2
Su XY 2012	Chinese Medicine Modern Distance	Dyslipidemia	52(10.3)	NR	NR	120	71/49	Total cholesterol (TC) > 5.72mmol/L, low-density lipoprotein cholesterol	NR	NR	NR	NR

Study	Journal	Disease	Age,y, mean (sd)	Age range, y, max	Age range, y, min	No. sample size	No. Gender (Male/Female)	Diagnosis criteria	Disease course, y, mean (sd)	Disease course range, y, max	Disease course range, y, min	Disease stage
	Education of China							(LDL-C) > 3.64mmol/L, triglyceride (TG) > 1.70mmol/L				
Sun H 2018	Diabetes New World	Diabetes	56.3(6.06)	72	35	100	54/46	WHO Diagnostic Criteria for Diabetes Mellitus	2.75(0.68)	13	1	diabetes
Sun Y 2021	Chinese Journal of Experimental Traditional Medical Formulae	Hypertension	49.30(9.00)	NR	NR	70	47/23	Chinese Guidelines for the Prevention and Treatment of Hypertension 2018	NR	NR	NR	stage 1
Tan MN 2006	Guiding Journal of Traditional Chinese Medicine and Pharmacy	Dyslipidemia	48.09(5.42)	63	20	76	45/31	Guidelines for the Clinical Research of New Traditional Chinese Medicine	4.54(1.60)	6	0.17	NR
Tian Y 2020	Clinical Journal of Chinese Medicine	Diabetes	51.02(6.99)	69	33	98	63/35	1999 WHO Diagnosis	1.80(2.27)	NR	NR	diabetes
Wang C 2023	Journal of Practical Traditional Chinese Internal Medicine	Hypertension	51.59(2.17)	75	42	120	61/59	Internal Medicine	2.16(0.27)	4	1	NR
Wang HY 2012	Clinical Journal of Chinese Medicine	Dyslipidemia	NR	74	35	172	106/66	Suggestions for the Prevention and Treatment of Dyslipidemia (1997)	NR	26	1	NR
Wang HY 2022	Qinghai Medical Journal	Diabetes	73.22(6.30)	85	60	96	55/41	Practical Guidelines for Primary Care of Type 2 Diabetes Mellitus (Practice Version 2019)	10.03(2.12)	17	3	diabetes
Wang L 2021	New Chinese Medicine	Diabetes	49.17(4.56)	65	40	100	59/41	Guidelines for the Prevention and Treatment of Diabetes Mellitus with Traditional Chinese Medicine	0.40(0.03)	1	0.83	diabetes
Wang MK 2021	Research of Integrated Traditional Chinese and Western Medicine	Diabetes	59.39(7.05)	NR	NR	60	28/32	Chinese Guidelines for the Prevention and Treatment of Type 2 Diabetes Mellitus (2020)	NR	NR	NR	diabetes
Wang QY 2021	Journal of frontiers of medicine	Diabetes	56.11(3.45)	76	35	80	45/35	Chinese Guidelines for the Prevention and Treatment of Type 2 Diabetes Mellitus (2017)	2.19(1.08)	4	1	diabetes
Wang QX 2018	China Health Standard Management	Diabetes	57.20(4.19)	75	48	265	140/125	1999 WHO Diagnosis	9.40(2.65)	19	2	diabetes
Wang Y 2022	Smart Healthcare	Diabetes	60.00(1.04)	70	50	50	31/19	① The patient's blood glucose test shows that the venous plasma 2hPG > 11.1mmol/L and FPG ≥ 7.0mmol/L; ② There are symptoms of "polyphagia, polydipsia, polyuria and weight loss"; ③ The patient has both insulin resistance or insulin deficiency.	3.50(0.32)	6	1	diabetes
Wu B 2019	Henan Traditional Chinese Medicine	Diabetes	52.45(9.90)	NR	NR	86	57/29	Chinese Guidelines for the Prevention and Treatment of Type 2 Diabetes Mellitus (2017)	0.22(0.13)	NR	NR	diabetes

Study	Journal	Disease	Age,y, mean (sd)	Age range, y, max	Age range, y, min	No. sample size	No. Gender (Male/Female)	Diagnosis criteria	Disease course, y, mean (sd)	Disease course range, y, max	Disease course range, y, min	Disease stage
Wu L 2021	NR	Diabetes	65.47(9.54)	NR	NR	56	28/28	Chinese Guidelines for the Prevention and Treatment of Type 2 Diabetes Mellitus (2017)	NR	NR	NR	diabetes
Wu TM 2020	Guangming Journal of Chinese Medicine	Hypertension	49.19(6.07)	60	41	41	21/20	Chinese Guidelines for the Prevention and Treatment of Hypertension 2011	4.64(3.16)	NR	NR	NR
Wu WM 2016	NR	Obesity	41.18(5.87)	NR	NR	72	37/35	"Standard for Determination of Adult Body Weight in the Health Industry of the People's Republic of ChinaWaist Circumference Standard for the Asia-Pacific Region Set by the World Health Organization"	11.81(3.60)	NR	NR	NR
Wu ZJ 2019	Chronic Pathematology Journal	Hypertension	52.07(5.12)	74	41	60	29/31	Chinese Guidelines for the Prevention and Treatment of Hypertension 2010	9.62(1.33)	22	1	stage 1, stage 2, stage 3
Xiao B 2014	China Modern Doctor	Dyslipidemia	68.65	79	48	80	47/33	Chinese Guidelines for the Prevention and Treatment of Dyslipidemia in Adults (2007)	NR	NR	NR	NR
Xiong QJ 2019	Clinical Research and Practice	Diabetes	53.58(7.69)	70	40	100	59/41	Chinese Guidelines for the Prevention and Treatment of Type 2 Diabetes Mellitus (2013)	4.8(1.07)	12	1	diabetes
Xiong YW 2010	Chinese Medicine Modern Distance Education of China	Hypertension	53.42(5.64)	NR	NR	60	36/24	Chinese Guidelines for the Prevention and Treatment of Hypertension 2005	6.35(3.33)	NR	NR	stage 1, stage 2, stage 3
Xu J 2015	The ISME Journal	Diabetes	53.52(8.54)	NR	NR	187	115/72	1999 WHO Diagnosis	NR	NR	NR	diabetes
Xue YF 2015	Journal of Practical Traditional Chinese Internal Medicine	Dyslipidemia	54.5(5.35)	78	39	136	80/56	Chinese Guidelines for the Prevention and Treatment of Dyslipidemia in Adults (2007)	0.35(0.11)	0.875	0.1	NR
Xu LR 2009	Traditional Chinese Medicinal Research	Diabetes	52.43(6.01)	65	35	169	81/88	1999 WHO Diagnosis	NR	22	0.5	diabetes
Yang GW 2010	Chinese Medicine Modern Distance Education of China	Obesity	36.2/35.7	53	16	48	3/45	Diagnostic and Efficacy Evaluation Criteria for Simple Obesity and Requirements for Medical Record Writing	6.55(0.15)	17	1	NR
Yan HG 2019	Chinese Journal of Ethnomedicine and Ethnopharmacy	Diabetes	54.25(8.51)	76	23	60	27/33	Chinese Guidelines for the Prevention and Treatment of Type 2 Diabetes Mellitus (2010)	8.77(6.96)	23	2	diabetes
Yang XM 2011	Hebei Journal of Traditional Chinese Medicine	Diabetes	54(5.17)	65	44	86	51/35	1999 WHO Diagnosis	9.36(6.25)	17	2	diabetes
Yang DD 2016	Journal of China Prescription Drug	Diabetes	50.72(10.55)	61	21	56	28/28	Chinese Guidelines for the Prevention and Treatment of Type 2 Diabetes Mellitus (2013)	0.34(0.09)	0.5	0.08	diabetes
Yang XJ 2023	NR	Hypertension	50.11(6.06)	NR	NR	82	34/46	Chinese Guidelines for the Prevention	4.19(2.04)	NR	NR	stage 1,

Study	Journal	Disease	Age,y, mean (sd)	Age range, y, max	Age range, y, min	No. sample size	No. Gender (Male/Female)	Diagnosis criteria	Disease course, y, mean (sd)	Disease course range, y, max	Disease course range, y, min	Disease stage
								and Treatment of Hypertension 2018				stage 2, stage 3
Yang XQ 2021	NR	Diabetes	46.05(9.05)	NR	NR	66	33/33	Chinese Guidelines for the Prevention and Treatment of Type 2 Diabetes Mellitus (2017)	6.87(2.56)	NR	NR	diabetes
Ye LF 2016	Journal of Nanjing University of Traditional Chinese Medicine	Obesity	42.62(10.14)	NR	NR	64	43/21	"Predictive Value of Body Mass Index and Waist Circumference of Chinese Adults for Abnormal Risk Factors of Related Diseases: A Study on the Optimal Cut-off Points of Body Mass Index and Waist CircumferencePrevalence and trends in obesity among US adults"	NR	NR	NR	NR
You XM 2015	Acta Chinese Medicine	Dyslipidemia	52.95(1.66)	69	35	60	35/25	Fasting serum cholesterol ≥ 6.22mmol/L and triglyceride ≥ 2.26 mmol/L	NR	NR	NR	NR
Yu CZ 2017	Chinese Journal of Information on Traditional Chinese Medicine	Diabetes	NR	NR	NR	69	34/35	1999 WHO Diagnosis	1.90(0.81)	NR	NR	diabetes
Yu HY 2016	NR	Obesity	39.5(4.47)	50	18	62	29/31	"Standard for Determination of Adult Body Weight in the Health Industry of the People's Republic of ChinaWaist Circumference Standard for the Asia-Pacific Region Set by the World Health Organization"	NR	NR	NR	NR
Yu SJ 2010	Liaoning Journal of Traditional Chinese Medicine	Hypertension	38.2(3.4)	70	30	57	57/0	Chinese Guidelines for the Prevention and Treatment of Dyslipidemia in Adults (2007)	1.28(1.03)	NR	NR	NR
Yu XL 2010	New Chinese Medicine	Dyslipidemia	52.59(5.93)	NR	NR	152	81/71	1999 WHO Diagnosis	0.52(0.21)	NR	NR	diabetes
Yuan RH 2016	Henan Traditional Chinese Medicine	Diabetes	56.05(8.34)	78	42	100	49/51	Chinese Guidelines for the Prevention and Treatment of Hypertension 2010	15.7(6.27)	NR	NR	stage 1, stage 2
Zhang HJ 2019	Heilongjiang Medicine and Pharmacy	Diabetes	53.43(7.16)	69	38	84	45/39	Practical Internal Medicine	NR	NR	NR	diabetes
Zhang HF 2019	Chinese Community Doctors	Diabetes	NR	71	35	70	NR/NR	1999 WHO Diagnosis	NR	NR	NR	diabetes
Zhang LM 2014	Chinese Journal of Information on Traditional Chinese Medicine	Diabetes	35.65	50	30	104	54/50	Chinese Guidelines for the Prevention and Treatment of Diabetes Mellitus (2007)	1.25	NR	NR	diabetes
Zhang LN 2019	Contemporary Medicine	Diabetes	48.51(10.97)	65	30	172	104/68	Chinese Guidelines for the Prevention and Treatment of Type 2 Diabetes Mellitus (2013)	NR	NR	NR	diabetes
Zhang LY 2016	NR	Diabetes	50.03(16.43)	68	28	66	43/23	1999 WHO Diagnosis	NR	NR	NR	diabetes
Zhang MQ 2019	Guide of China Medicine	Diabetes	48.7(3.48)	73	42	70	36/34	Chinese Guidelines for the Prevention and Treatment of Type 2 Diabetes Mellitus (2013)	4.95(0.61)	9	1	diabetes

Study	Journal	Disease	Age,y, mean (sd)	Age range, y, max	Age range, y, min	No. sample size	No. Gender (Male/Female)	Diagnosis criteria	Disease course, y, mean (sd)	Disease course range, y, max	Disease course range, y, min	Disease stage
Zhang Y 2016	Liaoning Journal of Traditional Chinese Medicine	Diabetes	44.15(10.88)	78	32	160	92/68	1999 WHO Diagnosis	NR	NR	NR	diabetes
Zhao HY 2016	Journal of Liaoning University of Traditional Chinese Medicine	Hypertension	63.26(8.99)	NR	NR	80	29/51	Chinese Guidelines for the Prevention and Treatment of Hypertension 2010	NR	NR	NR	stage 1, stage 2
Zhao JV 2021	Nutrients	Dyslipidemia	47.15(12.51)	NR	NR	80	80/0	Executive Summary of The Third Report of The National Cholesterol Education Program (NCEP) Expert Panel on Detection, Evaluation, And Treatment of High Blood Cholesterol In Adults (Adult Treatment Panel III)	NR	NR	NR	NR
Zheng BB 2009	Journal of Nanjing Medical University(Natura l Sciences)	Dyslipidemia	60.3(10.9)	NR	NR	99	51/48	Total cholesterol (TC) > 5.72 mmol/L, low-density lipoprotein cholesterol (LDL-C) > 3.64 mmol/L, triglyceride (TG) > 1.70 mmo/L	NR	NR	NR	NR
Zheng JF 2017	National Medical Journal of China	Diabetes	52.93(8.30)	70	41	96	55/41	WHO Diagnostic Criteria for Diabetes Mellitus	5.67(1.34)	13	1	diabetes
Zhou A 2012	NR	Diabetes	52.45(8.94)	NR	NR	110	57/53	Standardized Diagnosis and Treatment of Diabetes Mellitus Integrating Traditional Chinese and Western Medicine 2010	5.70(1.60)	13	1	diabetes
Zhou XY 2020	Tianjin Journal of Traditional Chinese Medicine	Diabetes	45.15(8.01)	NR	NR	90	48/42	Chinese Guidelines for the Prevention and Treatment of Type 2 Diabetes Mellitus (2017)	11.95(5.91)	NR	NR	diabetes
Zhu Y 2019	Henan Traditional Chinese Medicine	Hypertension	65.55(8.55)	86	40	62	24/38	Chinese Guidelines for the Prevention and Treatment of Hypertension 2018	NR	NR	NR	NR
Zhu YG 2018	Chinese Journal of Ethnomedicine and Ethnopharmacy	Diabetes	52.15(6.80)	70	44	120	67/53	Chinese Guidelines for the Prevention and Treatment of Type 2 Diabetes Mellitus (2013)	6.73(3.14)	14	1.4	diabetes

Abbreviations: NR: not report; WHO: World Health Organization; y, year. As the disease severity was not reported in any studies, thus we did not present the relevant information.

Table S4. The details of the treatment of individual studies

Study	Chinese medicine syndrome	Integrated medicine	Type of basic therapy	Intervention name	Preparation	Ingredients	Dosage, frequency of intervention	Control name	Dosage, frequency of control	Therapeutic duration (w)	Adverse events	Outcome [a]
Chen DS 2012	pattern of phlegm and dampness retain in the internal	N	lifestyle intervention	Linggui Zhugan Decoction	decoction	fuling, guizhi, baizhu, gancao, dangshen, dahuang	NR, bid	NR	NR	12	Y	2hPG, FPG
Chen LF 2022	pattern of dampness and heat accumulation and binding	Y	pharmacotherapy	Gegen Qinlian Decoction	decoction	gegen, huanglian, huangqin, gancao	100ml, bid	Metformin enteric-coated tablet	500mg, qd	8	Y	2hPG, FPG, HOMA-IR
Chen Y 2022	pattern of dampness and heat accumulation and binding	Y	pharmacotherapy	Huanglian Wendan Decoction	decoction	huanglian, baizhu, gancao, zhuru, chenpi, zhishi, shengjiang, banxia, fuling	80ml, bid	Metformin hydrochloride sustained release tablet	500mg, qd	12	NR	2hPG, FINS, FPG
Zeng YP 2006	pattern of dampness and heat accumulation and binding	Y	pharmacotherapy	Gegen Qinlian Decoction	decoction	gegen, huangqin, huanglian, gancao	100ml, bid	Isophane Suspension Recombinant Human Insulin, Insulin Regular	NR, tid<br>NR, tid	2	NR	2hPG, FPG
Cheng MJ 2018	pattern of dampness and heat accumulation and binding	N	lifestyle intervention	Gegen Qinlian Decoction	decoction	gegen, huangqin, huanglian, gancao, huangqi, shanyao, cangzhu, xuanshen, shengdi, tianhuafen, maidong, fuling	200ml, bid	Metformin hydrochloride tablet	500mg, tid	4	Y	2hPG, FPG
Cheng Y 2021	pattern of phlegm and dampness retain in the internal	Y	pharmacotherapy	Zexie Decoction Combined with Erchen Decoction	granules	zexie, baizhu, banxia, chenpi, fuling, danshen, guizhi, shengjiang, dazao, zhigancao	2 bag, NR	Amlodipine besylate tablet	5mg, qd	4	NR	DBP, SBP
Dai GL 2022	pattern of dampness and heat accumulation and binding	Y	pharmacotherapy	Gegen Qinlian Decoction	decoction	gegen, huangqin, huanglian, zhigancao	150ml, bid	Metformin	250mg, bid	8	Y	2hPG, FPG
Deng DQ 2018	pattern of spleen deficiency with dampness	N	NR	Xiaoke Jianpi Capsule	capsule	cangzhu, baizhu, tufuling, fuling, cheqianzi, yinchen,	3 pills, tid	Pioglitazone tablet	15mg, qd	12	NR	2hPG, FPG, HOMA-IR

Study	Chinese medicine syndrome	Integrated medicine	Type of basic therapy	Intervention name	Preparation	Ingredients	Dosage, frequency of intervention	Control name	Dosage, frequency of control	Therapeutic duration (w)	Adverse events	Outcome [a]
	encumbrance					chaoshanzhi, danshen						
Duan G 2015	pattern of phlegm, dampness and stasis	Y	lifestyle intervention, pharmacotherapy	Jiajian Didang Decoction	decoction	fahuanxia, shengbaizhu, shuizhi, taoren, shudahuang, fuling, chenpi	200ml, qd	30/70 Mixture Recombinant Human Insulin	0.3U/(kg·d), bid	4	NR	FPG
Fan YF 2017	pattern of dampness and heat accumulation and binding	Y	lifestyle intervention, pharmacotherapy	Gegen Qinlian Decoction	decoction	gegen, huangqin, huanglian, zhigancao	200ml, bid	Metformin hydrochloride tablet	850mg, bid	8	Y	FINS, FPG, HOMA-IR
Fang LL 2015	pattern of phlegm and dampness transforming into heat	Y	lifestyle intervention, pharmacotherapy	Xingpi Chuchen Decoction	decoction	huanglian, huangqin, gegen, shenghuangqi, chaihu, baishao, zhishi, chenpi, peilan, danshen, fuling, tianhuafen	200ml, bid	Metformin hydrochloride enteric coated tablets	500mg, tid	8	N	2hPG, FINS, FPG, HOMA-IR
Fang QXX 2016	pattern of phlegm and dampness retain in the internal	N	NR	Qushi Decoction	decoction	zhuru, zhishi, banxia, huangqin, chenpi, sanqi, mudanpi, fushen, baizhu, tianma, dihuang, shanzha, jiubaishao	200ml, bid	Hydrochlorothiazide tablet	25mg, qd	3	Y	DBP
Feng XG 2016	pattern of dampness and heat accumulation and binding	Y	lifestyle intervention, pharmacotherapy	Gegen Qinlian Decoction	decoction	gegen, huanglian, gancao, huangqin	150ml, bid	Metformin	500mg, qd	12	N	2hPG, FPG
Fu YH 2017	pattern of dampness and heat accumulation and binding	Y	lifestyle intervention, pharmacotherapy	Gegen Qinlian Xiaoke Decoction	decoction	gegen, huangqin, huanglian, zhigancao, huangqi, dangshen, baizhu, fuling, zexie, zhuling, yumixu, guizhi, danshen, ganjiang, shengdi	1 bag, qd	Metformin hydrochloride tablet	500mg, tid	8	Y	2hPG, FPG
Fu ZH 2021	pattern of phlegm and dampness	Y	lifestyle intervention,	Huanglian Wendan	decoction	fuling, huanglian, zhishi, banxia	150ml, bid	Metformin hydrochloride	850mg, tid	4	NR	2hPG, FPG

Study	Chinese medicine syndrome	Integrated medicine	Type of basic therapy	Intervention name	Preparation	Ingredients	Dosage, frequency of intervention	Control name	Dosage, frequency of control	Therapeutic duration (w)	Adverse events	Outcome [a]
	transforming into heat		pharmacotherapy	Decoction				enteric coated tablets				
Ge AL 2018	pattern of dampness and heat accumulation and binding	Y	lifestyle intervention, pharmacotherapy	Gegen Qinlian Decoction	decoction	gegen, huanglian, huangqin, gancao	1 pack decocted once, bid	Metformin	500mg, qd	8	NR	FINS, FPG, HOMA-IR
George BL 2012	pattern of phlegm and dampness retain in the internal	N	NR	RCM-104 Capsule	capsule	lücha, juemingzi, huaihua	4 pills, tid	placebo	4 pills, tid	12	Y	BMI, HC, WC, WHR
Guan JL 2016	pattern of phlegm and dampness retain in the internal	Y	lifestyle intervention, pharmacotherapy	Banxia Baizhu Tianma Decoction	decoction	tianma, jiangbanxia, gegen, baizhu, fuling, houpou, chenpi, chuanxiong, zhigancao, shanzha, shengjiang, dazao	100ml, bid	Levamlodipine benzenesulfonate	2.5mg,qd	4	N	DBP, SBP
He HM 2014	pattern of phlegm and dampness retain in the internal	Y	lifestyle intervention, pharmacotherapy	Xiaoke Jianpi Capsule	capsule	cangzhu, baizhu, tufuling, fuling, cheqianzi, yinchen, chaoshanzhi, danshen	4 pills, tid	Metformin hydrochloride tablet	500mg, tid	12	NR	2hPG, FINS, FPG, HOMA-IR
He HM 2018	pattern of spleen deficiency with dampness encumbrance	Y	lifestyle intervention, pharmacotherapy	Xiaoke Jianpi Capsule	capsule	cangzhu, baizhu, tufuling, taishen,gegen, guiban, biejia, cheqianzi, dahuang, zhizi	3 pills, tid	Metformin hydrochloride tablet	500mg, tid	12	N	2hPG, FPG
He JJ 2007	pattern of internal accumulation of dampness and heat	N	NR	Berberine	pill	berberine	0.5g, tid	simvastatin	20mg, qd	24	Y	HDL-C, LDL-C, TC, TG
Hong XT 2007	pattern of phlegm and dampness transforming into heat	N	NR	Qinghua Tanshi Decoction	decoction	renshen, shanzha, chenpi, zexie, huzhang, fahua, jiuzhi dahuang, machixian, shengshouwu	1 pack decocted once, NR	atorvastatin	10mg, qd	4	NR	HDL-C, LDL-C, TC, TG
Hu Y 2012	pattern of spleen deficiency with dampness	N	NR	Yinchen Wuling San	decoction	yinchen, zexie, zhuling, fuling, baizhu, guizhi	100ml, bid	simvastatin	20mg, qd	4	Y	HDL-C, LDL-C, TC, TG

Study	Chinese medicine syndrome	Integrated medicine	Type of basic therapy	Intervention name	Preparation	Ingredients	Dosage, frequency of intervention	Control name	Dosage, frequency of control	Therapeutic duration (w)	Adverse events	Outcome [a]
	encumbrance											
Hu YC 2015	pattern of phlegm and dampness retain in the internal	Y	lifestyle intervention, pharmacotherapy	Tanshi Neisheng Decoction	decoction	dananxing, jiangcan, zhu yu, tianma, juhong, banxia, baizhu, fuling, juemingzi, shenglonggu, shengmuli,gegen	100ml, bid	Captopril tablet	12.5mg, tid	12	Y	DBP, SBP
Huang J 2022	pattern of dampness and heat accumulation and binding	Y	lifestyle intervention, pharmacotherapy	Gegen Qinlian Decoction	decoction	gegen, huangqin, huanglian, zhigancao	100ml, bid	Metformin	500mg, bid	8	Y	2hPG, FPG, HOMA-IR
Huang P 2018	pattern of phlegm and dampness retain in the internal	Y	pharmacotherapy	Banxia Baizhu Tianma Decoction	decoction	tianma, fuling, banxia, chenpi, sharen, chuanxiong, baizhu, zhuru, dazao, danshen, shengjiang, gancao	200ml, bid	Nifedipine sustained-release tablet	10mg,bid	4	Y	DBP, SBP
Huang SP 2021	pattern of dampness and heat accumulation and binding	Y	pharmacotherapy	Gegen Qinlian Decoction	decoction	huanglian, huangling, ganjiang, gegen, shengancao	100ml, bid	Metformin	500mg, qd	8	Y	2hPG, FPG, HOMA-IR
Huang YZ 2017	pattern of qi and yin deficiency, pattern of phlegm and dampness	Y	lifestyle intervention, pharmacotherapy	Huopo Xialing Decoction	decoction	huoxiang, chuanhoupo, jiangbanxia, chifuling, xingren, shengyiyiren, baidoukou, zhuling, danshou, zexie, tongcao	150ml, bid	Metformin hydrochloride sustained release tablet	500mg, bid	4	N	2hPG, FPG
Huang ZS 2014	pattern of phlegm and dampness retain in the internal	Y	lifestyle intervention, pharmacotherapy	Banxia Baizhu Tianma Decoction	decoction	banxia, tianma, baizhu, chenpi, fuling, gancao	1 pack decocted twice, bid	Captopril tablet	12.5mg, bid	4	NR	DBP, SBP
Jiang L 2023	pattern of dampness and heat accumulation and binding	Y	lifestyle intervention, pharmacotherapy	Gegen Qinlian Decoction	decoction	gegen, huangqin, huanglian, chenpi, huangqi, gancao, ganjiang	200ml, bid	Insulin aspartate Insulin glargine	0.14IU/(kg·d)	12	Y	2hPG, FINS, FPG
Ke B	pattern of spleen	N	lifestyle	Linggui Zhugan	oral liquid	fuling, guizhi,	NR, bid	NR	NR	24	NR	BMI, WC

Study	Chinese medicine syndrome	Integrated medicine	Type of basic therapy	Intervention name	Preparation	Ingredients	Dosage, frequency of intervention	Control name	Dosage, frequency of control	Therapeutic duration (w)	Adverse events	Outcome [a]
2012	deficiency with dampness encumbrance		intervention	Decoction		baizhu, gancao, dangshen, dahuang						
Ke ZM 2019	pattern of phlegm and dampness retain in the internal	Y	pharmacotherapy	Zaoshi Huatan Huoxue Decoction	decoction	cangzhu, houpou, chenpi, jiangbanxia, fuling, shengjiang, gancao, xiangfu, chuanxiong	100ml, bid	Metformin Sitagliptin	500mg, qd 100mg, qd	4	NR	2hPG, FPG
Kong WJ 2008	pattern of internal accumulation of dampness and heat	N	NR	Berberine	pill	berberine	1g, qd	simvastatin	20mg, qd	8	N	HDL-C, LDL-C, TC, TG
Liang HC 2016	pattern of phlegm and dampness retain in the internal	Y	lifestyle intervention, pharmacotherapy	Linggui Zhugan Decoction	decoction	fuling, guizhi, baizhu, gancao, chenpi, wuzhimaotao, taishen, fahuanxia	100ml, bid	Metformin tablet	500mg, tid	12	N	2hPG, FPG
Li H 2018	pattern of dampness and heat accumulation and binding	Y	pharmacotherapy	Fufang Gegen Qinlian Decoction	decoction	gegen, huanglian, huangqin, gancao	1 pack decocted once, bid	Metformin	500mg, qd	2	N	2hPG, FPG
Li L 2020	pattern of dampness and heat accumulation and binding	Y	lifestyle intervention, pharmacotherapy	Gegen Qinlian Decoction	decoction	gegen, dangshen, zhigancao, ganjiang, baizhu, huangqin, huangqi, fuling, danshen, shengdihuang	200ml, qd	Metformin tablet	500mg, tid	12	NR	FINS, FPG, HOMA-IR
Li LR 2013	pattern of phlegm and dampness retain in the internal	Y	pharmacotherapy	Yiqi Jianpi Huatan Qushi Decoction	decoction	huangqi, baizhu, fuling, fahuanxia, shengshanzha, heye, zexie, danshen, zhi shouwu	100ml, bid	simvastatin tablet	20mg, qd	4	N	HDL-C, LDL-C, TC, TG
Li S 2020	pattern of phlegm dampness due to spleen deficiency	Y	lifestyle intervention, pharmacotherapy	Antang Decoction	decoction	baizhu, fuling, dangshen, chenpi, zhizi, shanzha, juemingzi, cangzhu, heye	200ml, bid	Metformin hydrochloride tablet	500mg, bid	12	NR	2hPG, FINS, FPG, HOMA-IR
Li SW 2007	pattern of internal accumulation of	N	lifestyle intervention	Jianfei Tiaozhi Capsule	capsule	shengdahuang, heshouwu,	2g, tid	NR	NR	8	Y	BMI, HC, WC, WHR

Study	Chinese medicine syndrome	Integrated medicine	Type of basic therapy	Intervention name	Preparation	Ingredients	Dosage, frequency of intervention	Control name	Dosage, frequency of control	Therapeutic duration (w)	Adverse events	Outcome [a]
	dampness and heat					huangqin, zexie, heye, shanzha						
Li X 2021	pattern of spleen deficiency with dampness encumbrance	Y	lifestyle intervention, pharmacotherapy	Jianpi Qushi Decoction	decoction	huangqi, shanyao, dangshen, fuling, qianshi, yiyiren, baizhu, cangzhu, huangbai, zexie, lianxu, tufuling, jinyingzi, wuzigui, gancao	150ml, bid	Metformin hydrochloride tablet	500mg, bid	4	NR	2hPG, FINS, FPG, HOMA-IR
Li YF 2021	pattern of phlegm and dampness retain in the internal	Y	pharmacotherapy	Banxia Baizhu Tianma Decoction	decoction	banxia, baizhu, tianma, chenpi, fuling, dangshen, huangqi, gancao, shengjiang, dazao	200ml, bid	Amlodipine besylate tablet	5mg, qd	4	NR	DBP, SBP
Li ZQ 2011	pattern of phlegm and dampness retain in the internal	Y	lifestyle intervention, pharmacotherapy	Huazhuo Yipi Decoction	decoction	huangqi, dangshen, chao baizhu, yiyiren, cangzhu, sharen, chenpi, fuling, fahuanxia, wuweizi, gancao	150ml, bid	Repaglinide tablet	1mg, tid	12	N	2hPG, FPG, HOMA-IR
Lin XM 2017	pattern of phlegm and dampness retain in the internal	Y	pharmacotherapy	Tanzhuo Neiyun Decoction	decoction	fuling, gouteng, chuanxiong, zexie, baizhu, shichangpu, jianqu, chenpi, tianma, banxia, dazao, shengjiang	150ml, bid	Captopril	25mg, tid	4	N	DBP, SBP
Liu FG 2011	pattern of phlegm, dampness and stasis	Y	lifestyle intervention, pharmacotherapy	Jiangzhi Tongmai Capsule	capsule	juemingzi, jianghuang, zexie, sanqi, tie xiancao	1g, tid	atorvastatin calcium tablet	20mg, qd	4	N	HDL-C, LDL-C, TC, TG
Liu L 2020	pattern of spleen deficiency with dampness encumbrance	N	lifestyle intervention	Qiwei Baizhu San	decoction	taishen, baizhu, fuling, huoxiang, muxiang,gegen, zhigancao	NR	NR	NR	12	NR	BMI
Liu XK 2008	pattern of dampness turbidity	N	NR	Lishi Jiangzhi Decoction	decoction	fuling, huangjing, zexie, yinchen, shouwu	1 pack decocted once, NR	simvastatin	20mg, qd	4	N	HDL-C, LDL-C, TC, TG
Liu XL	pattern of phlegm	Y	pharmacothera	Banxia Baizhu	decoction	banxia, tianma,	150ml, bid	Amlodipine	5mg, qd	4	NR	DBP, SBP

Study	Chinese medicine syndrome	Integrated medicine	Type of basic therapy	Intervention name	Preparation	Ingredients	Dosage, frequency of intervention	Control name	Dosage, frequency of control	Therapeutic duration (w)	Adverse events	Outcome [a]
2016	and dampness retain in the internal		py	Tianma Decoction		baizhu, fuling, juhong, gancao, shengjiang, dazao		besylate tablet				
Liu YP 2007	pattern of phlegm and dampness retain in the internal	N	NR	Banxia Baizhu Tianma Decoction	decoction	banxia, tianma, fuling, chenpi, baizhu, gancao, danggui, baishao, heye, zexie	150ml, bid	Captopril	12.5mg, bid	4	NR	DBP, SBP
Liu Z 2014	pattern of phlegm and dampness retain in the internal	Y	pharmacothera py	Banxia Baizhu Tianma Decoction	decoction	banxia, baizhu, chenpi, fuling, tianma, sangjisheng, cheqianzi, gancao	1 pack decocted once, qd	Captopril	25mg, bid	8	NR	DBP, SBP
Lu H 2017	pattern of phlegm, dampness and stasis	N	NR	Zini Jiangzhi Decoction	decoction	dangshen, chao baizhu, fuling, shengancao, banxia, chenpi, shengshanzha, danshen, chuanxiong, huainiuxi, juemingzi, chaihu, zhi heshouwu, zexie, jiaogulan	30ml, bid	NR	NR	9	NR	HDL-C, LDL-C, TC, TG
Lu X 2004	pattern of phlegm, dampness and stasis	N	NR	Gegen Qushi Decoction	decoction	gegen, dangshen, baizhu, fuling, shenqu, ba kourou, ganjiang, muxiang, sharen, qingpi, chenpi, zexie, danshen, yujin	200ml, bid	maitong capsule	1 pill, tid	8	N	HDL-C, TC, TG
Lu JZ 2018	pattern of phlegm and dampness retain in the internal	Y	pharmacothera py	Banxia Baizhu Tianma Decoction Combined with Shengjiang San	decoction	baizhu, banxia, fuling, tianma, shengjiang, juhong, gancao, dazao, chan tui, jiangcan, dahuang, jianghuang	150ml, bid<br>150ml, bid	Perindopril tablet<br>Felodipine sustained-release tablet	4mg, qd<br>5mg, qd	4	Y	DBP, SBP
Luo TJ 2014	pattern of damp-heat	N	lifestyle intervention	Simiao Granules	granules	huanglian, huangbai, cangzhu,	NR, bid	Pioglitazone hydrochloride	15mg, qd	4	NR	FINS

Study	Chinese medicine syndrome	Integrated medicine	Type of basic therapy	Intervention name	Preparation	Ingredients	Dosage, frequency of intervention	Control name	Dosage, frequency of control	Therapeutic duration (w)	Adverse events	Outcome [a]
	encumbering the spleen					yiyiren						
Ma D 2016	pattern of spleen deficiency with dampness encumbrance	Y	lifestyle intervention, pharmacotherapy	Xiaoke Jianpi Decoction	decoction	cangzhu, baizhu, fuling, tufuling, houpou, cheqianzi, yinchen, chao zhizi, peilan	200ml, bid	Pioglitazone hydrochloride tablet	15mg, qd	12	N	2hPG, FINS, FPG
Ma HN 2019	pattern of phlegm and dampness retain in the internal	Y	lifestyle intervention, pharmacotherapy	Banxia Baizhu Tianma Decoction	granules	baizhu, juhong, banxia, tianma, fuling, dazao, shengjiang, gancao	200ml, bid	Irbesartan tablet	0.15g, qd	4	N	DBP, SBP
Ma LM 2020	pattern of phlegm and dampness retain in the internal	Y	pharmacotherapy	Wuhuang Yangyin Granules	granules	huanglian, hongqi, dihuang, jianghuang, huangqin	6g, tid	Pioglitazone hydrochloride tablet	15-30mg, qd	8		2hPG, FINS, FPG, HOMA-IR
Mao P 2022	pattern of phlegm and dampness retain in the internal	Y	pharmacotherapy	Banxia Baizhu Tianma Decoction	decoction	banxia, baizhu, tianma, chenpi, fuling, zhigancao, dazao, shengjiang	100ml, tid	Enalapril Maleate	10mg,qd	4	N	DBP, SBP
Meng H 2004	pattern of phlegm, dampness and stasis	N	NR	Jiawei Erchen Decoction	decoction	chenpi, fahua, zhi nanxing, dahuang, zexie, shanzha, sanqi, fuling, gancao	1 pack decocted once, qd	dodecon capsule	3 pills, tid	8	NR	TC, TG
Meng XR 2008	pattern of phlegm and dampness retain in the internal	Y	lifestyle intervention, pharmacotherapy	Jianpi Huatan Decoction	decoction	huangqi, dangshen, cangzhu, banxia, zao ci, tianzhu huang, dananxing, shanzha, jiaogulan, heye	100ml, bid	Glipizide tablet Metformin	5mg, tid 250mg, tid	4	N	2hPG, FPG
Miao LJ 2017	pattern of phlegm and dampness retain in the internal	Y	pharmacotherapy	Banxia Baizhu Tianma Decoction	decoction	zexie, heye, baizhu, danggui, baishao, banxia, fuling, tianma, gancao	1 pack decocted twice, bid	Captopril tablet	12.5mg, bid	4	Y	24h AMBP
Ni Q 2021	pattern of dampness and heat accumulation and binding	Y	lifestyle intervention, pharmacotherapy	Gegen Qinlian Decoction	decoction	gegen, huangqin, huanglian, shudahuang, zhimu	150ml, bid	Insulin aspartate Insulin glargine	0.14IU/(kg·d)	12	NR	2hPG, FPG, HOMA-IR
Pan CQ	pattern of phlegm,	Y	pharmacotherapy	Jiangzhi Tongmai	capsule	juemingzi,	1.5g, tid	ezetimibe tablet	1 pill, qd	4	Y	HDL-C, LDL-C, TC,

Study	Chinese medicine syndrome	Integrated medicine	Type of basic therapy	Intervention name	Preparation	Ingredients	Dosage, frequency of intervention	Control name	Dosage, frequency of control	Therapeutic duration (w)	Adverse events	Outcome [a]
2016	dampness and stasis		py	Capsule		jianghuang, zexie, sanqi, tie xiancao						TG
Pan SS 2021	pattern of dampness and heat accumulation and binding	Y	pharmacotherapy	Huanglian Wendan Decoction	decoction	huanglian, zhuru, zhishi, banxia, chenpi, gancao, shengjiang, fuling, baizhu	100ml, bid	Metformin hydrochloride tablet	500mg, qd	12	NR	FINS, FPG, HOMA-IR
Pang YH 2013	pattern of phlegm and dampness retain in the internal	Y	pharmacotherapy	Banxia Baizhu Tianma Decoction	decoction	tianma, banxia, baizhu, fuling, zexie,gegen, chuanxiong, shanzha, gancao	200ml, bid	Enalapril tablet Nifedipine sustained-release tablet	5mg, bid 10mg, bid	4	NR	DBP, SBP
Rao CY 2015	pattern of phlegm, dampness and stasis	N	NR	Jiangzhi Tongmai Capsule	capsule	juemingzi, jianghuang, zexie, sanqi, tie xiancao	2g, tid	atorvastatin calcium tablet	10mg, qd	4	N	HDL-C, LDL-C, TC, TG
Ren JL 2017	pattern of phlegm and dampness retain in the internal	Y	pharmacotherapy	Banxia Baizhu Tianma Decoction	decoction	sharen, dazao, gancao, shengjiang, zhuru, zhi nanxing, fuling, chenpi, banxia, shichangpu, cheqianzi, zexie, tianma, baizhu	1 pack decocted twice, bid	Levamlodipine tablet	2.5mg,qd	4	NR	DBP, SBP
Ren YG 2022	pattern of phlegm and dampness retain in the internal	Y	lifestyle intervention, pharmacotherapy	Banxia Baizhu Tianma Decoction	decoction	fuling, shui zhi, gancao, chenpi, xiangru, baidoukou, houpou, sharen, chao baizhu, tianma, cangzhu, shengjiang, muxiang, zhuru	200ml, bid	Amlodipine besylate tablet Aspirin	5mg,qd 100mg,qd	4	NR	DBP, SBP
Song YY 2019	pattern of phlegm and dampness retain in the internal	Y	lifestyle intervention, pharmacotherapy	Huatan Jianpi Decoction	decoction	banxia, huanglian, ganjiang, rougui, renshen, cangzhu, houpou, huangqi, fuling, baizhu, shanyao, zexie	200ml, bid	Dimethylidene hydrochloride tablet Acarbose tablet	500mg, qd 50mg, tid	8	NR	2hPG, FINS, FPG, HOMA-IR
Ruan JA 2019	pattern of phlegm and dampness retain in the	N	NR	Heze Granules	granules	heye, zexie, juemingzi, zhi shouwu, chaihu,	3 bags, bid	placebo	3 bags, bid	45d	NR	BMI, HC, WC

Study	Chinese medicine syndrome	Integrated medicine	Type of basic therapy	Intervention name	Preparation	Ingredients	Dosage, frequency of intervention	Control name	Dosage, frequency of control	Therapeutic duration (w)	Adverse events	Outcome [a]
	internal					huangqin, zhike, banxia						
Shi CZ 2019	pattern of phlegm and dampness retain in the internal	Y	pharmacotherapy	Banxia Baizhu Tianma Decoction	decoction	banxia, tianma, chenpi, baizhu, fuling, gancao	1 pack decocted twice, bid	Valsartan capsule	80mg, qd	8	N	DBP, SBP
Su XY 2012	pattern of internal accumulation of dampness and heat	Y	pharmacotherapy	Berberine	pill	berberine	0.3g, tid	simvastatin	20mg, qd	8	Y	HDL-C, LDL-C, TC, TG
Sun H 2018	pattern of dampness and heat accumulation and binding	N	NR	Gegen Qinlian Decoction	decoction	zhigancao, huanglian, huangqin,gegen	1 pack decocted once, bid	Metformin hydrochloride tablet	250mg, bid	8	N	2hPG, FPG
Sun Y 2021	pattern of dampness and heat stagnation	N	lifestyle intervention	Fufang Guizhencao Granules	granules	guizhencao, yinchen, xiakucao, danshen, yumixu, dahuang	6.5g, bid	Fifang Guizhencao Kelimoniji	6.5g, bid	4	NR	24h AMBP, DBP, SBP
Tan MN 2006	pattern of phlegm, dampness and stasis	N	lifestyle intervention	Jianpi Lishi Huatan Quyu Decoction	decoction	dangshen, baizhu, fuling, zexie, fahua, shichangpu, chishao, shanzha, huzhang, taoren	150ml, bid	simvastatin	20mg, qd	8	N	HDL-C, LDL-C, TC, TG
Tian Y 2020	pattern of dampness and heat accumulation and binding	Y	pharmacotherapy	Gegen Qinlian Decoction	decoction	huanglian, ganjiang,gegen, shengancao, huangqin	100ml, bid	Metformin	250mg, tid	8	Y	2hPG, FPG
Wang C 2023	pattern of phlegm and dampness retain in the internal	Y	lifestyle intervention, pharmacotherapy	Banxia Baizhu Tianma Decoction	decoction	banxia, chenpi, fuling, tianma, baizhu, juhong, manjingzi, chuanxiong, gegen, gouteng, dazao, gancao	200ml, bid	Nifedipine Benazepril Hydrochloride	20mg,bid 10mg,qd	4	Y	DBP, SBP
Wang HY 2012	pattern of phlegm dampness due to spleen deficiency	N	NR	Banxia Baizhu Shanzha Decoction	decoction	banxia, baizhu, fuling, shanzha, danshen, dangshen, juemingzi, dilong, chaihu, honghua, yujin, yinxingye,	100ml, bid	lovastatin capsule	20mg, qd	4	Y	HDL-C, LDL-C, TC, TG

Study	Chinese medicine syndrome	Integrated medicine	Type of basic therapy	Intervention name	Preparation	Ingredients	Dosage, frequency of intervention	Control name	Dosage, frequency of control	Therapeutic duration (w)	Adverse events	Outcome [a]
						heshouwu						
Wang HY 2022	pattern of phlegm and dampness transforming into heat	Y	lifestyle intervention, pharmacotherapy	Huanglian Wendan Decoction	decoction	huanglian, fuling, zhimu, gualu, zhuru, heye, zexie, shengjiang, chenpi, zhishi, jiangbanxia, gancao	200ml, bid	Metformin hydrochloride tablet	250mg, tid	12	NR	2hPG, FPG
Wang L 2021	pattern of dampness and heat accumulation and binding	Y	pharmacotherapy	Gegen Qinlian Decoction	decoction	gegen, gualu, danshen, huangqin, peilan, zhishi, fahuanxia, huanglian, gancao	200ml, bid	Metformin hydrochloride tablet	250-500mg, bid/tid	12	NR	2hPG, FINS, FPG, HOMA-IR
Wang MK 2021	pattern of dampness and heat accumulation and binding	Y	lifestyle intervention, pharmacotherapy	Huanglian Wendan Decoction	decoction	huanglian, zhuru, zhishi, banxia, chenpi, gancao, shengjiang, fuling, baizhu	200ml, bid	Metformin hydrochloride tablet	850mg, tid	8	NR	2hPG, FINS, FPG
Wang QY 2021	pattern of dampness and heat accumulation and binding	Y	lifestyle intervention, pharmacotherapy	Gegen Qinlian Decoction	decoction	gegen, huanglian, huangqin, gancao	200ml, bid	Sitagliptin phosphate tablets	100mg, qd	12	Y	2hPG, FPG
Wang QX 2018	pattern of phlegm dampness due to spleen deficiency	Y	lifestyle intervention, pharmacotherapy	Qiwei Baizhu San Combined with Buyang Huanwu Decoction	decoction	danggui, fuling, taoren, chishao, chuanxiong, dilong, peilan, huoxiang, dangshen,gegen, baizhu, huangqi	200ml, bid	Metformin	500mg, tid	8	NR	2hPG, FINS, FPG
Wang Y 2022	pattern of dampness and heat accumulation and binding	Y	lifestyle intervention, pharmacotherapy	Huanglian Wendan Decoction	decoction	huanglian, zhuru, gancao, fuling, baizhu, zhishi, chenpi, shengjiang, banxia	150ml, bid	Metformin	500mg, qd	16	NR	2hPG, FPG
Wu B 2019	pattern of phlegm and dampness transforming into heat	Y	lifestyle intervention, pharmacotherapy	Sanhuang Decoction	granules	huangqin, dahuang, huanglian	200ml, bid	Insulin aspartate 30<br>Metformin tablet	NR, NR<br>500mg, tid	NR	Y	FINS, FPG, HOMA-IR
Wu L 2021	pattern of dampness and heat accumulation and	Y	lifestyle intervention, pharmacothera	Gegen Qinlian Decoction	decoction	gegen, huanglian, huangqin, gancao, huangqi, ganjiang,	150ml, bid	Metformin hydrochloride sustained release	500mg, bid	8	Y	2hPG, FINS, FPG, HOMA-IR

Study	Chinese medicine syndrome	Integrated medicine	Type of basic therapy	Intervention name	Preparation	Ingredients	Dosage, frequency of intervention	Control name	Dosage, frequency of control	Therapeutic duration (w)	Adverse events	Outcome [a]
	binding		py			chenpi		tablet				
Wu TM 2020	pattern of phlegm and dampness retain in the internal	Y	pharmacotherapy	Wendan Decoction	decoction	fahuanxia, zhuru, zhishi, jupi, fuling, baizhu, tianma, gancao, danshen, xiakucao, shengjiang, dazao	1 pack decocted twice, bid	Amlodipine besylate tablet	5mg, qd	4	NR	DBP, SBP
Wu WM 2016	pattern of internal accumulation of dampness and heat	N	lifestyle intervention	Fangfeng Tongsheng Pills	pill	fangfeng, chuanxiong, danggui, shao yao, dahuang, boheye, mahuang, lianqiao, mangxiao, shigao, huangqin, jiegeng, huashi, gancao, jingjiesui, chao baizhu, zhizi	6g, bid	NR	NR	12	N	BMI, WHR
Wu ZJ 2019	pattern of phlegm and dampness retain in the internal	Y	pharmacotherapy	Banxia Baizhu Tianma Decoction	decoction	dazao, banxia, fuling, baizhu, tianma, shengjiang, juhong, gancao	1 pack decocted twice, bid	Amlodipine besylate tablet	5mg, qd	4	N	DBP, SBP
Xiao B 2014	pattern of phlegm, dampness and stasis	Y	lifestyle intervention, pharmacotherapy	Jiangzhi Tongmai Capsule	capsule	juemingzi, jianghuang, zexie, sanqi, tie xiancao	1.5g, tid	atorvastatin calcium tablet	20mg, qd	8	N	HDL-C, LDL-C, TC, TG
Xiong QJ 2019	pattern of dampness and heat accumulation and binding	Y	lifestyle intervention, pharmacotherapy	Gegen Qinlian Decoction	decoction	gegen, huangqin, huanglian, shengancao, ganjiang	100ml, bid	Metformin hydrochloride tablet	250mg, tid	8	NR	2hPG, FPG
Xiong YW 2010	pattern of phlegm and dampness retain in the internal	Y	pharmacotherapy	Banxia Baizhu Tianma Decoction	decoction	banxia, baizhu, tianma, chenpi, fuling, zexie, cheqianzi, zhuru, sharen, zhi nanxing, shichangpu, shengjiang, dazao, gancao	250ml, bid	Levamlodipine tablet	2.5mg,qd	4	NR	24h-AMBP
Xu J 2015	pattern of dampness and heat	N	NR	Gegen Qinlian Decoction	decoction	gegen, huangqin, huanglian, gancao	150ml, bid	placebo	150ml, bid	12	N	2hPG, FINS, FPG, HOMA-IR

Study	Chinese medicine syndrome	Integrated medicine	Type of basic therapy	Intervention name	Preparation	Ingredients	Dosage, frequency of intervention	Control name	Dosage, frequency of control	Therapeutic duration (w)	Adverse events	Outcome [a]
	accumulation and binding											
Xue YF 2015	pattern of phlegm, dampness and stasis	Y	pharmacotherapy	Jiangzhi Decoction	decoction	heye, honghua, shanzha, chishao, chenpi, zexie, danshen, heshouwu, huangqi	100ml, bid	simvastatin	20mg, qd	4	N	HDL-C, LDL-C, TC, TG
Xu LR 2009	pattern of phlegm and dampness retain in the internal	Y	pharmacotherapy	Minshutang Capsule	capsule	huangqi, zexie, baixiezi, dahuang, zhishi, jiangcan	1.5g, tid	Glipizide tablet<br>Pioglitazone hydrochloride	5mg, tid<br>15mg, qd	16	NR	2hPG, FPG
Yang GW 2010	pattern of phlegm and dampness retain in the internal	N	lifestyle intervention	Jianpi Qushi Huatan Decoction	decoction	huangqi, shanyao, fahuanxia, zhebeimu, yiyiren, chao shanzha, danshen, fuling, baizhu, cangzhu	NR	NR	NR	12	NR	BMI
Yan HG 2019	pattern of damp-heat encumbering the spleen	N	NR	Huangqin Huashi Decoction	decoction	huangqin, huashi, zhuling, fuling, baikourou, tongcao, baizhu, yiyiren, gancao	200ml, bid	Metformin hydrochloride	500mg, tid	8	Y	2hPG, FPG
Yang XM 2011	pattern of phlegm and dampness retain in the internal	Y	pharmacotherapy	Zaoshi Huatan Decoction	decoction	banxia, chenpi, fuling, cangzhu,gegen, huanglian, shanyao, gancao	100ml, bid	Metformin enteric-coated tablet	500mg, tid	8	NR	2hPG, FINS, FPG
Yang DD 2016	pattern of spleen deficiency with dampness encumbrance	Y	pharmacotherapy	Zini Jianpi Huashi Decoction	decoction	dangshen, dihuang, chenpi, fuling, fengweicao, zhishi, huoxiang, zhuling, shengancao	1 pack decocted once, bid	Insulin Detemir	0.2U/(kg·d), qd	12	Y	2hPG, FPG
Yang XJ 2023	pattern of phlegm and dampness retain in the internal	Y	lifestyle intervention, pharmacotherapy	Banxia Baizhu Tianma Decoction	decoction	tianma, baizhu, cangzhu, fuling, zexie, huangqi, dangshen, chao qu, banxia, chenpi, huangbai, guizhi, zhigancao, gouteng,	300ml, bid	Amlodipine Besylate tablet	5mg, qd	4	Y	DBP, SBP

Study	Chinese medicine syndrome	Integrated medicine	Type of basic therapy	Intervention name	Preparation	Ingredients	Dosage, frequency of intervention	Control name	Dosage, frequency of control	Therapeutic duration (w)	Adverse events	Outcome [a]
						sharen						
Yang XQ 2021	pattern of dampness and heat accumulation and binding	Y	lifestyle intervention, pharmacotherapy	Gegen Qinlian Decoction	decoction	gegen, huangqin, huanglian, zhigancao, dangshen, baizhu, fuling, zexie	100ml, bid	Metformin hydrochloride tablet	500mg, bid	8	Y	2hPG, FPG
Ye LF 2016	pattern of phlegm and dampness transforming into heat	N	lifestyle intervention	Sanhuang Decoction	decoction	huanglian, huangqin, zhi dahuang	100ml, bid	NR	NR	12	NR	BMI, WC
You XM 2015	pattern of phlegm, dampness and stasis	Y	lifestyle intervention, pharmacotherapy	Jiangzhi Tongmai Capsule	capsule	juemingzi, jianghuang, zexie, sanqi, tie xiancao	NR	atorvastatin tablet	20mg, qd	8	N	HDL-C, LDL-C, TC, TG
Yu CZ 2017	pattern of phlegm dampness due to spleen deficiency	Y	lifestyle intervention, pharmacotherapy	Jiawei Liujunzi Decoction	decoction	renshen, baizhu, fuling, gancao, chenpi, fahuanxia, heye, peilan	200ml, tid	Metformin hydrochloride sustained release tablets	500mg, qd	12	NR	2hPG, FINS, FPG, HOMA-IR
Yu HY 2016	pattern of internal accumulation of dampness and heat	N	lifestyle intervention	Peilian Mahuang Decoction	decoction	peilan, huanglian, mahuang	150ml, bid	NR	NR	12	NR	BMI, WHR
Yu SJ 2010	pattern of phlegm, dampness and stasis	N	lifestyle intervention	Qushi Huayu Tongluo Decoction	decoction	yiyiren, tufuling, zexie, niuxi, qinpi, jixueteng	1 pack decocted once, NR	NR	NR	6	NR	TC, TG
Yu XL 2010	pattern of dampness and heat accumulation and binding	N	lifestyle intervention	Huanglian Wendan Decoction	decoction	maidong, zhuru, fuling, fahuanxia, zhike, huangqin, chenpi, huanglian, gancao	1 pack decocted 3 times, tid	NR	NR	8	Y	2hPG, FINS, FPG
Yuan RH 2016	pattern of phlegm and dampness retain in the internal	N	NR	Banxia Baizhu Tianma Decoction	decoction	qingbanxia, baizhu, gouteng, tianma, chenpi, fuling, zexie, danshen, juemingzi, shanzha, gancao, shengjiang, dazao	100ml, bid	Valsartan	80mg, qd	8	N	DBP, SBP
Zhang HJ 2019	pattern of damp-heat encumbering the	Y	lifestyle intervention, pharmacothera	Jianpi Lishi Decoction	decoction	danshen, shichangpu, fuling, peilan, chixiaodou,	200ml, bid	Liraglutide	0.6-1.8mg, qd	12	N	2hPG, FPG

Study	Chinese medicine syndrome	Integrated medicine	Type of basic therapy	Intervention name	Preparation	Ingredients	Dosage, frequency of intervention	Control name	Dosage, frequency of control	Therapeutic duration (w)	Adverse events	Outcome [a]
	spleen		py			baidou, fahuanxia, huangqin, huanglian, zhike, yujin, chenpi, zhuru						
Zhang HF 2019	pattern of dampness and heat accumulation and binding	Y	pharmacotherapy	Gegen Qinlian Decoction	decoction	gegen, huanglian, huangqin, gancao	150ml, bid	Metformin	250mg, bid	8	NR	2hPG, FPG
Zhang LM 2014	pattern of phlegm and dampness transforming into heat	Y	lifestyle intervention, pharmacotherapy	Xiaoxianxiong Decoction	decoction	huanglian, fahuanxia, gualu pi, gualu zi	100ml, tid	Metformin hydrochloride tablet	500mg, bid	12	NR	2hPG, FPG
Zhang LN 2019	pattern of dampness and heat accumulation and binding	Y	lifestyle intervention, pharmacotherapy	Gegen Qinlian Decoction	decoction	gegen, huangqin, huanglian, zhigancao	100ml, bid	Metformin	250mg, bid	8	NR	2hPG, FPG, HOMA-IR
Zhang LY 2016	pattern of dampness and heat accumulation and binding	N	lifestyle intervention	Qingre Lishi Decoction	granules	huanglian, sangbaipi, dihuang, tianhuafen, shengyiren, peilan, danshen,gegen, shanyao	200ml, bid	NR	NR	4	N	2hPG, FPG
Zhang MQ 2019	pattern of dampness and heat accumulation and binding	Y	pharmacotherapy	Gegen Qinlian Decoction	decoction	gegen, huangqin, huanglian, zhigancao	200-300ml, bid	Metformin hydrochloride sustained release tablet	500mg, qd	8	Y	2hPG, FPG
Zhang Y 2016	pattern of damp-heat encumbering the spleen	Y	lifestyle intervention, pharmacotherapy	Gegen Qinlian Decoction	decoction	gegen, huangqin, huanglian, shengancao, ganjiang	1 pack decocted once, bid	Metformin tablet	250-1000g, bid/tid	8	NR	2hPG, FPG
Zhao HY 2016	pattern of phlegm and dampness retain in the internal	Y	lifestyle intervention, pharmacotherapy	Banxia Baizhu Tianma Decoction	decoction	jiangbanxia, tianma, fuling, juhong, baizhu, gancao, shengjiang, dazao	200ml, bid	Valsartan	80mg, qd	12	NR	DBP, SBP
Zhao JV 2021	pattern of internal accumulation of dampness and heat	N	NR	Berberine	pill	berberine	0.4g, tid	NR	NR	12	Y	HDL-C, LDL-C, TC, TG
Zheng BB 2009	pattern of internal accumulation of	N	NR	Berberine	pill	berberine	0.4g, tid	simvastatin	20mg, qd	8	Y	HDL-C, LDL-C, TC, TG

Study	Chinese medicine syndrome	Integrated medicine	Type of basic therapy	Intervention name	Preparation	Ingredients	Dosage, frequency of intervention	Control name	Dosage, frequency of control	Therapeutic duration (w)	Adverse events	Outcome [a]
Zheng JF 2017	dampness and heat pattern of dampness and heat accumulation and binding	Y	pharmacotherapy	Gegen Qinlian Decoction	decoction	gegen, huanglian, huangqin, shengancao, ganjiang	1 pack decocted once, bid	Metformin hydrochloride	500mg, bid	8	NR	2hPG, FPG
Zhou A 2012	pattern of dampness and heat accumulation and binding	N	lifestyle intervention	Gegen Qinlian Decoction	decoction	gegen, huanglian, huangqin, gancao	1 pack decocted once, bid	placebo	1 pack decocted once, bid	12	Y	2hPG, FPG
Zhou XY 2020	pattern of damp-heat encumbering the spleen	Y	lifestyle intervention, pharmacotherapy	Gegen Qinlian Decoction	decoction	gegen, huangqin, huanglian, gancao	1 pack decocted twice, bid	Liraglutide	0.6-1.2mg, qd	12	Y	2hPG, FPG, HOMA-IR
Zhu Y 2019	pattern of phlegm and dampness retain in the internal	Y	pharmacotherapy	Erzhu Erchen Decoction	decoction	cangzhu, baizhu, juhong, fahuanxia, fuling, shengjiang, gancao	1 pack decocted twice, bid	Amlodipine besylate tablet	5mg, qd	4	N	DBP, SBP
Zhu YG 2018	pattern of dampness and heat accumulation and binding	N	NR	Gegen Qinlian Decoction	decoction	gegen, huanglian, huangqin, huangcha, shengancao, ganjiang	125ml, bid	Metformin	250-500mg, tid	24	Y	DBP, SBP

Abbreviations: N, no; w, week; Y, yes.

[a] 24h AMBP: 24 - hour Ambulatory Blood Pressure; 2hPG: 2 - hour Postprandial Blood Glucose; BMI: Body Mass Index; DBP: Diastolic Blood Pressure; FINS: Fasting Insulin; FPG: Fasting Plasma Glucose; HC: Hip Circumference; HDL-C: High - Density Lipoprotein Cholesterol; HOMA-IR: Homeostasis Model Assessment of Insulin Resistance; LDL-C: Low - Density Lipoprotein Cholesterol; SBP: Systolic Blood Pressure; TC: Total Cholesterol; TG: Triglyceride; WC: Waist Circumference; WHR: Waist - Hip Ratio.

[b] There are two groups of control: pharmacotherapy and Chinese herbal medicine plus pharmacotherapy.

[c] There are two groups of control: placebo of Chinese herbal medicine and no treatment.

[d] This is a study including four groups: high dose of Chinese herbal medicine, moderate dose of Chinese herbal medicine, low dose of Chinese herbal medicine and placebo of Chinese herbal medicine.

Table S5. Top frequently used Chinese herbal medicine formulae for metabolic conditions

Name of prescriptions	Frequency (No. RCT)	Ingredients in Chinese Pinyin	Ingredients in English name	Scientific names and species
Gegen Qinlian Decoction	28	1. Gegen 15g<br>2. Huangqin 9g<br>3. Huanglian 9g<br>4. Gancao 6g	1. PUERARIAE LOBATAE RADIX<br>2. SCUTELLARIAE RADIX<br>3. COPTIDIS RHIZOMA<br>4. GLYCYRRHIZAE RADIXET RHIZOMA	1. dried roots of Pueraria lobata(Willd.)Ohwi.<br>2. dried root of Astragalus membranaceus(Fisch.) Bge.var.mongholicus(Bge.)Hsiao or Astragalus membranaceus(Fisch.)Bge., n,dried root of Scutellaria baicalensis Georgi.<br>3. dried rhizome of Coptis chinensis Franch. or Coptis deltoidea C.Y.Cheng et Hsiao or Cop.<br>4. dried roots and rhizomes of Glycyrrhiza uralensis Fisch.,Glycyrrhiza inflata Bat.or Glycyrrhiza glabra L.
Banxia Baizhu Tianma Decoction	20	1. Tianma 3g<br>2. Fuling 3g<br>3. Banxia 4.5g<br>4. Chenpi 3g<br>5. Baizhu 9g<br>6. Gancao 1.5g	1. GASTRODIAE RHIZOMA<br>2. PORIA<br>3. PINELLIAE RHIZOMA<br>4. CITRI RETICULATAE PERICARPIUM<br>5. ATRACTYLODIS MACROCEPHALAE RHIZOMA<br>6. GLYCYRRHIZAE RADIXET RHIZOMA	1. dried root tuber of Gastrodia elata Bl.<br>2. dried sclerotia of Poria cocos (Schw.) Wolf.<br>3. dried tuber of Pinellia ternata (Thunb.) Breit.<br>4. dried ripe peel of Citrus reticulata Blanco.<br>5. dried rhizome of Atractylodes macrocephala Koidz.<br>6. dried roots and rhizomes of Glycyrrhiza uralensis Fisch.,Glycyrrhiza inflata Bat.or Glycyrrhiza glabra L.
Huanglian Wendan Decoction	7	1. Huanglian 6g<br>2. Zhuru 12g<br>3. Zhishi 6g<br>4. Banxia 6g<br>5. Chenpi 6g<br>6. Gancao 3g<br>7. Dazao 1piece<br>8. Fuling 10g	1. COPTIDIS RHIZOMA<br>2. BAMBUSAE CAULIS IN TAENIAS<br>3. AURANTII FRUCTUS IMMATURUS<br>4. PINELLIAE RHIZOMA<br>5. CITRI RETICULATAE PERICARPIUM<br>6. GLYCYRRHIZAE RADIXET RHIZOMA<br>7. JUJUBAE FRUCTUS<br>8. PORIA	1. dried rhizome of Coptis chinensis Franch. or Coptis deltoidea C.Y.Cheng et Hsiao or Cop.<br>2. dried middle layer of the stem of Bambusa tuldoides Munro or Sinocalamus beecheyanus (Munro)McClure var. Pubescens P.F.Li or Phyllostachys nigra(Lodd.)Munro var.henonis(Mitf.)Stapf ex Rendle.<br>3. dried young fruit of Citrus aurantium L. or Citrus sinensis Osbeck.<br>4. dried tuber of Pinellia ternata (Thunb.) Breit.<br>5. dried ripe peel of Citrus reticulata Blanco.<br>6. dried roots and rhizomes of Glycyrrhiza uralensis Fisch.,Glycyrrhiza inflata Bat.or Glycyrrhiza glabra L.<br>7. dried ripe fruit of Ziziphus jujuba Mill.<br>8. dried sclerotia of Poria cocos (Schw.) Wolf.
Jiangzhi Tongmai Capsule	5	1. Juemingzi<br>2. Jianghuang<br>3. Zexie<br>4. Sanqi	1. CASSIAE SEMEN<br>2. CURCUMAE LONGAE RHIZOMA<br>3. ALISMATIS RHIZOMA<br>4. NOTOGINSENG RADIXET RHIZOMA	1. dried ripe seed of Cassia obtusifolia L. or Cassiatora L.<br>2. dried rhizome of Curcuma Longa L.<br>3. dried tuber of Alisma orientale(Sam.)Juzep.or Alisma plantago-aquatica Linn.<br>4. dried roots and rhizomes of Panax notoginseng (Burk.) F. H. Chen.
Berberine hydrochloride	5	1. Huanglian	COPTIDIS RHIZOMA	1. dried rhizome of Coptis chinensis Franch. or Coptis deltoidea C.Y.Cheng et Hsiao or Cop.
Linggui Zhugan Decoction	3	1. Fuling 12g<br>2. Guizhi 9g<br>3. Baizhu 6g<br>4. Gancao 6g	1. PORIA<br>2. CINNAMOMI RAMULUS<br>3. ATRACTYLODIS MACROCEPHALAE RHIZOMA<br>4. GLYCYRRHIZAE RADIXET RHIZOMA	1. dried sclerotia of Poria cocos (Schw.) Wolf.<br>2. dried twigs of Cinnamomum cassia Presl.<br>3. dried rhizome of Atractylodes macrocephala Koidz.<br>4. dried roots and rhizomes of Glycyrrhiza uralensis Fisch.,Glycyrrhiza inflata Bat.or Glycyrrhiza glabra L.

Xiaoke Jianpi Capsule	3	1. Cangzhu<br>2. Baizhu<br>3. Tufuling<br>4. Fuling<br>5. Cheqianzi<br>6. Yinchen<br>7. Zhizi<br>8. Danshen	1. ATRACTYLODIS RHIZOMA<br>2. ATRACTYLODIS MACROCEPHALAE RHIZOMA<br>3. SMILACIS GLABRAE RHIZOMA<br>4. PORIA<br>5. PLANTAGINIS SEMEN<br>6. ARTEMISIAE SCOPARIAE HERBA<br>7. ARDENIAE FRUCTUS<br>8. SALVIAE MILTIORRHIZAE RADIX ET RHIZOMA	1. dried rhizome of Atractylodes lancea（Thunb.）DC.<br>2. dried rhizome of Atractylodes macrocephala Koidz.<br>3. dried rhizome of Smilax glabra Roxb.<br>4. dried sclerotia of Poria cocos (Schw.) Wolf.<br>5. dried ripe seed of Plantago asiatica L.<br>6. dried ground part of Artemisia scoparia Waldst.etKit. or Artemisia capillaris Thunb.<br>7. dried ripe flesh of Gardenia jasminoides Ellis.<br>8. dried roots and rhizomes of Salvia miltiorrhiza Bge.
Sanhuang Decoction	2	1. Huangqin 10g<br>2. Dahuang 6g<br>3. Huanglian 3g	1. SCUTELLARIAE RADIX<br>2. RHEI RADIX ET RHIZOMA<br>3. COPTIDIS RHIZOMA	1. dried root of Astragalus membranaceus (Fisch.) Bge.var.mongholicus (Bge.)Hsiao or Astragalus membranaceus(Fisch.)Bge., n,dried root of Scutellaria baicalensis Georgi.<br>2. dried roots and rhizomes of Rheum palmatum L., Rheum tanguticum Maxim. ex Bal£. or Rheum officinale Bail1.<br>3. dried rhizome of Coptis chinensis Franch. or Coptis deltoidea C.Y.Cheng et Hsiao or Cop.

Notes: Minor modifications to the herbal composition and dosage of the same CHM formula, based on symptom patterns, were observed in the included randomised controlled trials (RCTs). Only the composition of the primary CHM formulae is presented herein, with reference to: Qi, Y. S. (2017). Pharmaceutical Research on Classic Traditional Chinese Medicine Prescriptions. Beijing: China Traditional Chinese Medicine Press.

Table S6. The risk of bias assessment

Study Name	Type of disease	Type of outcomes	Randomisation sequence generation (selection bias)	Allocation concealment (selection bias)	Blinding of participants and personnel (performance bias)	Blinding of outcome assessment (detection bias)	Incomplete outcome data (attrition bias)	Selective reporting (reporting bias)
Chen DS 2012	Type 2 diabetes mellitus	Objective outcomes	Low risk	Unclear risk	Low risk	Low risk	High risk	Low risk
Chen LF 2022	Type 2 diabetes mellitus	Objective outcomes	Low risk	Unclear risk	Low risk	Low risk	Low risk	Unclear risk
Chen Y 2022	Type 2 diabetes mellitus	Objective outcomes	Unclear risk	Unclear risk	Low risk	Low risk	Low risk	Unclear risk
Cheng MJ 2018	Type 2 diabetes mellitus	Objective outcomes	Low risk	Unclear risk	Low risk	Low risk	Low risk	Unclear risk
Dai GL 2022	Type 2 diabetes mellitus	Objective outcomes	Low risk	Unclear risk	Low risk	Low risk	Low risk	Unclear risk
Deng DQ 2018	Type 2 diabetes mellitus	Objective outcomes	Unclear risk	Unclear risk	Low risk	Low risk	Unclear risk	Unclear risk
Duan GS 2015	Type 2 diabetes mellitus	Objective outcomes	Unclear risk	Unclear risk	Low risk	Low risk	Low risk	Unclear risk
Fan YF 2017	Type 2 diabetes mellitus	Objective outcomes	Unclear risk	Unclear risk	Low risk	Low risk	Low risk	Unclear risk
Fang LL 2015	Type 2 diabetes mellitus	Objective outcomes	Unclear risk	Unclear risk	Low risk	Low risk	Low risk	Unclear risk
Feng XG 2016	Type 2 diabetes mellitus	Objective outcomes	Low risk	Unclear risk	Low risk	Low risk	Low risk	Unclear risk
Fu YH 2017	Type 2 diabetes mellitus	Objective outcomes	Low risk	Low risk	Low risk	Low risk	High risk	Unclear risk
Fu ZH 2021	Type 2 diabetes mellitus	Objective outcomes	Low risk	Unclear risk	Low risk	Low risk	Low risk	Unclear risk
Ge AL 2018	Type 2 diabetes mellitus	Objective outcomes	Low risk	Unclear risk	Low risk	Low risk	Low risk	Unclear risk
He HM 2014	Type 2 diabetes mellitus	Objective outcomes	Unclear risk	Unclear risk	Low risk	Low risk	Low risk	Unclear risk
He HM 2018	Type 2 diabetes mellitus	Objective outcomes	Low risk	Unclear risk	Low risk	Low risk	Low risk	Unclear risk
Huang J 2022	Type 2 diabetes mellitus	Objective outcomes	Low risk	Unclear risk	Low risk	Low risk	Low risk	Unclear risk
Huang SP 2021	Type 2 diabetes mellitus	Objective outcomes	Unclear risk	Unclear risk	Low risk	Low risk	Low risk	Unclear risk
Huang YZ 2017	Type 2 diabetes mellitus	Objective outcomes	Unclear risk	Unclear risk	Low risk	Low risk	Low risk	Unclear risk
Jiang L 2023	Type 2 diabetes mellitus	Objective outcomes	Low risk	Unclear risk	Low risk	Low risk	Low risk	Unclear risk
Ke ZM 2019	Type 2 diabetes mellitus	Objective outcomes	Unclear risk	Unclear risk	Low risk	Low risk	Low risk	Unclear risk
Li H 2018	Type 2 diabetes mellitus	Objective outcomes	Low risk	Unclear risk	Low risk	Low risk	Low risk	Unclear risk
Li L 2020	Type 2 diabetes mellitus	Objective outcomes	Low risk	Unclear risk	Low risk	Low risk	Low risk	Unclear risk
Li S 2020	Type 2 diabetes mellitus	Objective outcomes	Low risk	Unclear risk	Low risk	Low risk	Low risk	Unclear risk
Li X 2021	Type 2 diabetes mellitus	Objective outcomes	Unclear risk	Unclear risk	Low risk	Low risk	Low risk	Unclear risk
Li ZQ 2011	Type 2 diabetes mellitus	Objective outcomes	Low risk	Unclear risk	Low risk	Low risk	Low risk	Unclear risk
Liang HC 2016	Type 2 diabetes mellitus	Objective outcomes	Low risk	Unclear risk	Low risk	Low risk	Low risk	Unclear risk
Luo TJ 2014	Type 2 diabetes mellitus	Objective outcomes	Low risk	Unclear risk	Low risk	Low risk	Unclear risk	Unclear risk
Ma D 2016	Type 2 diabetes mellitus	Objective outcomes	Low risk	Unclear risk	Low risk	Low risk	Unclear risk	Unclear risk
Ma LM 2020	Type 2 diabetes mellitus	Objective outcomes	High risk	Unclear risk	Low risk	Low risk	Low risk	Unclear risk
Meng XR 2008	Type 2 diabetes mellitus	Objective outcomes	Low risk	Unclear risk	Low risk	Low risk	Low risk	Unclear risk
Ni Q 2021	Type 2 diabetes mellitus	Objective outcomes	Low risk	Unclear risk	Low risk	Low risk	Unclear risk	Unclear risk
Pan SS 2021	Type 2 diabetes mellitus	Objective outcomes	Unclear risk	Unclear risk	Low risk	Low risk	Low risk	Unclear risk
Song YY 2019	Type 2 diabetes mellitus	Objective outcomes	Unclear risk	Unclear risk	Low risk	Low risk	Low risk	Unclear risk
Sun H 2018	Type 2 diabetes mellitus	Objective outcomes	High risk	Unclear risk	Low risk	Low risk	Low risk	Unclear risk
Tian Y 2020	Type 2 diabetes mellitus	Objective outcomes	Low risk	Unclear risk	Low risk	Low risk	Low risk	Unclear risk
Wang HY 2022	Type 2 diabetes mellitus	Objective outcomes	Unclear risk	Unclear risk	Low risk	Low risk	Low risk	Unclear risk
Wang L 2021	Type 2 diabetes mellitus	Objective outcomes	Low risk	Unclear risk	Low risk	Low risk	Low risk	Unclear risk

Wang MK 2021	Type 2 diabetes mellitus	Objective outcomes	Low risk	Unclear risk	Low risk	Low risk	Low risk	Unclear risk
Wang QX 2018	Type 2 diabetes mellitus	Objective outcomes	Unclear risk	Unclear risk	Low risk	Low risk	Low risk	Unclear risk
Wang QY 2021	Type 2 diabetes mellitus	Objective outcomes	Low risk	Unclear risk	Low risk	Low risk	Low risk	Unclear risk
Wang Y 2022	Type 2 diabetes mellitus	Objective outcomes	High risk	Unclear risk	Low risk	Low risk	Low risk	Unclear risk
Wu B 2019	Type 2 diabetes mellitus	Objective outcomes	Low risk	Unclear risk	Low risk	Low risk	Low risk	Unclear risk
Wu L 2021	Type 2 diabetes mellitus	Objective outcomes	Low risk	Unclear risk	Low risk	Low risk	High risk	Unclear risk
Xiong QJ 2019	Type 2 diabetes mellitus	Objective outcomes	Low risk	Unclear risk	Low risk	Low risk	Low risk	Unclear risk
Xu J 2015	Type 2 diabetes mellitus	Objective outcomes	Unclear risk	Unclear risk	Low risk	Low risk	Low risk	Unclear risk
Xu LR 2009	Type 2 diabetes mellitus	Objective outcomes	Low risk	Unclear risk	Low risk	Low risk	Unclear risk	Unclear risk
Yan HG 2019	Type 2 diabetes mellitus	Objective outcomes	Low risk	Unclear risk	Low risk	Low risk	Low risk	Unclear risk
Yang DD 2016	Type 2 diabetes mellitus	Objective outcomes	Low risk	Unclear risk	Low risk	Low risk	Unclear risk	Unclear risk
Yang XM 2011	Type 2 diabetes mellitus	Objective outcomes	Unclear risk	Unclear risk	Low risk	Low risk	Low risk	Unclear risk
Yang XQ 2021	Type 2 diabetes mellitus	Objective outcomes	Unclear risk	Unclear risk	Low risk	Low risk	Low risk	Unclear risk
Yu CZ 2017	Type 2 diabetes mellitus	Objective outcomes	Low risk	Unclear risk	Low risk	Low risk	Low risk	Unclear risk
Yu XL 2010	Type 2 diabetes mellitus	Objective outcomes	Unclear risk	Unclear risk	Low risk	Low risk	Low risk	Unclear risk
Zeng YP 2006	Type 2 diabetes mellitus	Objective outcomes	Unclear risk	Unclear risk	Low risk	Low risk	Low risk	Unclear risk
Zhang HF 2019	Type 2 diabetes mellitus	Objective outcomes	Unclear risk	Unclear risk	Low risk	Low risk	Low risk	Unclear risk
Zhang HJ 2019	Type 2 diabetes mellitus	Objective outcomes	Low risk	Unclear risk	Low risk	Low risk	Low risk	Unclear risk
Zhang LM 2014	Type 2 diabetes mellitus	Objective outcomes	High risk	Unclear risk	Low risk	Low risk	Low risk	Unclear risk
Zhang LN 2019	Type 2 diabetes mellitus	Objective outcomes	Low risk	Unclear risk	Low risk	Low risk	Low risk	Unclear risk
Zhang LY 2016	Type 2 diabetes mellitus	Objective outcomes	Low risk	Unclear risk	Low risk	Low risk	Low risk	Unclear risk
Zhang MQ 2019	Type 2 diabetes mellitus	Objective outcomes	Unclear risk	Unclear risk	Low risk	Low risk	Low risk	Unclear risk
Zhang Y 2016	Type 2 diabetes mellitus	Objective outcomes	Low risk	Unclear risk	Low risk	Low risk	Low risk	Unclear risk
Zheng JF 2017	Type 2 diabetes mellitus	Objective outcomes	Unclear risk	Unclear risk	Low risk	Low risk	Low risk	Unclear risk
Zhou A 2012	Type 2 diabetes mellitus	Objective outcomes	Low risk	Low risk	Low risk	Low risk	High risk	Unclear risk
Zhou XY 2020	Type 2 diabetes mellitus	Objective outcomes	Low risk	Unclear risk	Low risk	Low risk	Low risk	Unclear risk
Zhu YG 2018	Type 2 diabetes mellitus	Objective outcomes	High risk	Unclear risk	Low risk	Low risk	Low risk	Unclear risk
Wang HY 2012	Hyslipidemia	Objective outcomes	Unclear risk	Unclear risk	Low risk	Low risk	Low risk	Unclear risk
Lu X 2004	Hyslipidemia	Objective outcomes	Low risk	Unclear risk	Low risk	Low risk	Low risk	Unclear risk
Meng H 2004	Hyslipidemia	Objective outcomes	Unclear risk	Unclear risk	Low risk	Low risk	Low risk	Unclear risk
Tan MN 2006	Hyslipidemia	Objective outcomes	Unclear risk	Unclear risk	Low risk	Low risk	Low risk	Unclear risk
Xue YF 2015	Hyslipidemia	Objective outcomes	Unclear risk	Unclear risk	Low risk	Low risk	Low risk	Unclear risk
Hong XT 2007	Hyslipidemia	Objective outcomes	Unclear risk	Unclear risk	Low risk	Low risk	Low risk	Unclear risk
Lu H 2017	Hyslipidemia	Objective outcomes	Low risk	Unclear risk	Low risk	Low risk	Low risk	Unclear risk
Liu XK 2008	Hyslipidemia	Objective outcomes	Unclear risk	Unclear risk	Low risk	Low risk	Low risk	Unclear risk
Yu SJ 2010	Hyslipidemia	Objective outcomes	Unclear risk	Unclear risk	Low risk	Low risk	Low risk	Unclear risk
Li LR 2013	Hyslipidemia	Objective outcomes	Unclear risk	Unclear risk	Low risk	Low risk	Low risk	Unclear risk
Xiao B 2014	Hyslipidemia	Objective outcomes	Unclear risk	Unclear risk	Low risk	Low risk	Low risk	Unclear risk
Rao CY 2015	Hyslipidemia	Objective outcomes	Unclear risk	Unclear risk	Low risk	Low risk	Low risk	Unclear risk
Liu FG 2011	Hyslipidemia	Objective outcomes	Unclear risk	Unclear risk	Low risk	Low risk	Low risk	Unclear risk
You XM 2015	Hyslipidemia	Objective outcomes	Low risk	Unclear risk	Low risk	Low risk	Low risk	Unclear risk
Pan CQ 2016	Hyslipidemia	Objective outcomes	High risk	Unclear risk	Low risk	Low risk	Low risk	Unclear risk
Kong WJ 2008	Hyslipidemia	Objective outcomes	Unclear risk	Unclear risk	Low risk	Low risk	Low risk	Unclear risk
Zhao JV 2021	Hyslipidemia	Objective outcomes	Low risk	Low risk	Low risk	Low risk	Low risk	Low risk

Zheng BB 2009	Hyslipidemia	Objective outcomes	Unclear risk	Unclear risk	Low risk	Low risk	Low risk	Unclear risk
He JJ 2007	Hyslipidemia	Objective outcomes	Unclear risk	Unclear risk	Low risk	Low risk	Low risk	Unclear risk
Su XY 2012	Hyslipidemia	Objective outcomes	Unclear risk	Unclear risk	Low risk	Low risk	Low risk	Unclear risk
Hu Y 2012	Hyslipidemia	Objective outcomes	Low risk	Unclear risk	Low risk	Low risk	Low risk	Unclear risk
Li YF 2021	Hypertension	Clinician-reported outcomes	Unclear risk	Unclear risk	High risk	Unclear risk	Low risk	Unclear risk
Shi CZ 2019	Hypertension	Clinician-reported outcomes	Low risk	Unclear risk	High risk	Unclear risk	Low risk	Unclear risk
Huang P 2018	Hypertension	Clinician-reported outcomes	Low risk	Unclear risk	High risk	Unclear risk	Low risk	Unclear risk
Lin XM 2017	Hypertension	Clinician-reported outcomes	Low risk	Unclear risk	High risk	Unclear risk	Low risk	Unclear risk
Hu YC 2015	Hypertension	Clinician-reported outcomes	Low risk	Unclear risk	High risk	Unclear risk	Low risk	Unclear risk
Zhu Y 2019	Hypertension	Clinician-reported outcomes	Low risk	Unclear risk	High risk	Unclear risk	Low risk	Unclear risk
Sun Y 2021	Hypertension	Clinician-reported outcomes	Low risk	Low risk	Low risk	Unclear risk	Low risk	Unclear risk
Wu TM 2020	Hypertension	Clinician-reported outcomes	Unclear risk	Unclear risk	High risk	Unclear risk	Low risk	Unclear risk
Pang YH 2013	Hypertension	Clinician-reported outcomes	Unclear risk	Unclear risk	High risk	Unclear risk	Low risk	Unclear risk
Fang QXX 2016	Hypertension	Clinician-reported outcomes	Low risk	Unclear risk	High risk	Unclear risk	Low risk	Unclear risk
Cheng Y 2021	Hypertension	Clinician-reported outcomes	Unclear risk	Unclear risk	High risk	Unclear risk	Low risk	Unclear risk
Lu JZ 2018	Hypertension	Clinician-reported outcomes	Unclear risk	Unclear risk	High risk	Unclear risk	Low risk	Unclear risk
Liu Z 2014	Hypertension	Clinician-reported outcomes	Unclear risk	Unclear risk	High risk	Unclear risk	Low risk	Unclear risk
Liu YP 2007	Hypertension	Clinician-reported outcomes	Unclear risk	Unclear risk	High risk	Unclear risk	Low risk	Unclear risk
Xiong YW 2010	Hypertension	Clinician-reported outcomes	Unclear risk	Unclear risk	High risk	Unclear risk	Low risk	Unclear risk
Huang ZS 2014	Hypertension	Clinician-reported outcomes	Low risk	Unclear risk	High risk	Unclear risk	Unclear risk	Unclear risk
Zhao HY 2016	Hypertension	Clinician-reported outcomes	Unclear risk	Unclear risk	High risk	Unclear risk	Low risk	Unclear risk
Guan JL 2016	Hypertension	Clinician-reported outcomes	Unclear risk	Unclear risk	High risk	Unclear risk	Low risk	Unclear risk
Yuan RH 2016	Hypertension	Clinician-reported outcomes	Low risk	Unclear risk	High risk	Unclear risk	Low risk	Unclear risk
Liu XL 2016	Hypertension	Clinician-reported outcomes	Low risk	Unclear risk	High risk	Unclear risk	Low risk	Unclear risk
Miao LJ 2017	Hypertension	Clinician-reported outcomes	Low risk	Unclear risk	High risk	Unclear risk	Low risk	Unclear risk
Ren JL 2017	Hypertension	Clinician-reported outcomes	Low risk	Unclear risk	High risk	Unclear risk	Low risk	Unclear risk
Wu ZJ 2019	Hypertension	Clinician-reported outcomes	Low risk	Unclear risk	High risk	Unclear risk	Low risk	Unclear risk
Ma HN 2019	Hypertension	Clinician-reported outcomes	Low risk	Unclear risk	High risk	Unclear risk	Low risk	Unclear risk
Ren YG 2022	Hypertension	Clinician-reported outcomes	Unclear risk	Unclear risk	High risk	Unclear risk	Low risk	Unclear risk
Mao P 2022	Hypertension	Clinician-reported outcomes	Unclear risk	Unclear risk	High risk	Unclear risk	Low risk	Unclear risk
Wang C 2023	Hypertension	Clinician-reported outcomes	Low risk	Unclear risk	High risk	Unclear risk	Low risk	Unclear risk
Yang XJ 2023	Hypertension	Clinician-reported outcomes	Low risk	Unclear risk	High risk	Unclear risk	Low risk	Unclear risk
Li SW 2007	Obesity	Clinician-reported outcomes	Low risk	Unclear risk	High risk	Unclear risk	Low risk	Unclear risk
Yang GW 2010	Obesity	Clinician-reported outcomes	Unclear risk	Unclear risk	High risk	Unclear risk	Low risk	Unclear risk
Liu L 2020	Obesity	Clinician-reported outcomes	Low risk	Unclear risk	High risk	Unclear risk	Low risk	Unclear risk
Ye LF 2016	Obesity	Clinician-reported outcomes	Unclear risk	Unclear risk	High risk	Unclear risk	Low risk	Unclear risk
Ke B 2012	Obesity	Clinician-reported outcomes	Low risk	Unclear risk	High risk	Unclear risk	Low risk	Unclear risk
Wu WM 2016	Obesity	Clinician-reported outcomes	Low risk	Unclear risk	High risk	Unclear risk	Low risk	Unclear risk
Yu HY 2016	Obesity	Clinician-reported outcomes	Unclear risk	Unclear risk	High risk	Unclear risk	Low risk	Unclear risk
Ruan JA 2019	Obesity	Clinician-reported outcomes	Unclear risk	Unclear risk	Unclear risk	Unclear risk	Low risk	Unclear risk
George BL 2012	Obesity	Clinician-reported outcomes	Low risk	Low risk	Low risk	Unclear risk	Low risk	Low risk

Table S7. Summary of adverse events

Type 2 diabetes mellitus		
**Comparison**	**Type of adverse events**	**No. of adverse events (Intervention/Control)**
**CHM vs. Control**	Hepatobiliary disorders (e.g., hepatic function abnormal)	8/3
	Metabolism and nutrition disorders (e.g., hypoglycaemia)	6/15
	Gastrointestinal disorders (e.g., nausea, diarrhoea, abdominal pain)	3/3
	Skin and subcutaneous tissue disorders (e.g., pruritus)	1/0
	Renal and urinary disorders (e.g., renal impairment, haematuria)	0/4
	Nervous system disorders (e.g., headache, dizziness, somnolence)	0/1
**CHM+Control vs. Control**	Gastrointestinal disorders (e.g., nausea, diarrhoea, abdominal pain)	32/51
	Nervous system disorders (e.g., headache, dizziness, somnolence)	7/8
	Metabolism and nutrition disorders (e.g., hypoglycaemia)	5/25
	Renal and urinary disorders (e.g., renal impairment, haematuria)	1/1
	General disorders and administration site conditions (e.g., oedema, pyrexia, fatigue)	0/2
	Vascular disorders (e.g., hypotension, flushing)	0/1
	Without details	0/1
**Hypertension**		
**Comparison**	**Type of adverse events**	**No. of adverse events (Intervention/Control)**
**CHM vs. Control**	Gastrointestinal disorders (e.g., nausea, diarrhoea, abdominal pain)	2/3
	General disorders and administration site conditions (e.g., oedema, pyrexia, fatigue)	0/3
	Musculoskeletal and connective tissue disorders (e.g., myalgia)	0/1
	Nervous system disorders (e.g., headache, dizziness, somnolence)	0/2
**CHM+Control vs. Control**	Gastrointestinal disorders (e.g., nausea, diarrhoea, abdominal pain)	10/10
	Nervous system disorders (e.g., headache, dizziness, somnolence)	5/6
	Respiratory, thoracic and mediastinal disorders (e.g., cough, dyspnoea)	3/1
	General disorders and administration site conditions (e.g., oedema, pyrexia, fatigue)	2/1
	Vascular disorders (e.g., hypotension, flushing)	1/3
	Skin and subcutaneous tissue disorders (e.g., pruritus)	1/0
	General disorders and administration site conditions (e.g., oedema, pyrexia, fatigue)	0/1
**Dyslipidaemia**		
**Comparison**	**Type of adverse events**	**No. of adverse events (Intervention/Control)**

**CHM vs Control**	Gastrointestinal disorders (e.g., nausea, diarrhoea, abdominal pain)	11/27
	Nervous system disorders (e.g., headache, dizziness, somnolence)	3/2
	Hepatobiliary disorders (e.g., hepatic function abnormal)	0/16
**CHM+Control vs Control**	Gastrointestinal disorders (e.g., nausea, diarrhoea, abdominal pain)	3/4
	Nervous system disorders (e.g., headache, dizziness, somnolence)	3/3
	General disorders and administration site conditions (e.g., oedema, pyrexia, fatigue)	2/3
	Musculoskeletal and connective tissue disorders (e.g., myalgia)	0/1
**Obesity**		
**Comparison**	**Type of adverse events**	**No. of adverse events (Intervention/Control)**
**CHM vs Control**	Nervous system disorders (e.g., headache, dizziness, somnolence)	9/0
	Gastrointestinal disorders (e.g., nausea, diarrhoea, abdominal pain)	6/2

Supplementary Table S8.GRADE Evidence Profile

A. GRADE Evidence Profile on Chinese herbal medicine for metabolic conditions

Certainty assessment							№ of patients		Relative Effect MD (95% CI)	Certainty
№ of studies	Study design	Risk of bias	Inconsistency	Indirectness	Imprecision	Other considerations	CHM	Control		
**Effect of CHM on FPG for T2DM**										
CHM vs. Control										
12	randomised trials	serious	serious	not serious	not serious	none	535	410	MD **0.82 lower** (1.31 lower to 0.33 lower)	Low
CHM plus conventional medicine vs. control										
54	randomised trials	serious	serious	not serious	not serious	Publication bias suspected	2498	2393	MD **0.11 lower** (1.32 lower to 0.90 lower)	Low
**Effect of CHM on 2hPG for T2DM**										
CHM vs. Control										
12	randomised trials	serious	serious	not serious	not serious	none	535	410	MD **1.13 lower** (1.97 lower to 0.29 lower)	Low
CHM plus conventional medicine vs. control										
49	randomised trials	serious	serious	not serious	not serious	Publication bias suspected	2258	2153	MD **1.27 lower** (1.48 lower to 1.06 lower)	Low
**Effect of CHM on SBP for hypertension**										
CHM vs. Control										
3	randomised trials	serious	serious	not serious	serious	Unclear*	130	120	MD **8.16 higher** (7.35 lower to 23.67 higher)	Very Low
CHM plus conventional medicine vs. control										
21	randomised trials	serious	serious	not serious	not serious	Publication bias suspected	931	892	MD **10.20 lower** (13.26 lower to 7.14 lower)	Low
**Effect of CHM on DBP for hypertension**										
CHM vs. Control										
4	randomised trials	serious	serious	not serious	serious	Unclear*	184	174	MD **0.62 higher** (7.10 lower to 8.34 higher)	Very Low
CHM plus conventional medicine vs. control										
21	randomised trials	serious	serious	not serious	not serious	none	931	892	MD **6.51 lower** (8.48 lower to 4.54 lower)	Low
**Effect of CHM on TG for dyslipidemia**										
CHM vs. Control										
15	randomised trials	serious	serious	not serious	not serious	none	742	573	MD **0.25 lower** (0.33 lower to 0.16 lower)	Low
CHM plus conventional medicine vs. control										
11	randomised trials	serious	serious	not serious	not serious	none	433	366	MD **0.52 lower** (0.71 lower to 0.32 lower)	Low
**Effect of CHM on LDL-C for dyslipidemia**										
CHM vs. Control										

12	randomised trials	serious	serious	not serious	serious	none	590	431	MD **0.14 lower** (0.38 lower to 0.09 higher)	Very Low
CHM plus conventional medicine vs. control										
11	randomised trials	serious	serious	not serious	not serious	none	433	366	MD **0.25 lower** (0.49 lower to 0.02 lower)	Low
**Effect of CHM on BMI for T2DM**										
CHM vs. Control										
9	randomised trials	serious	not serious	not serious	not serious	Unclear*	320	293	MD **1.64 lower** (2.16 lower to 1.11 lower)	Moderate

B. GRADE Evidence Profile on Gegen Qinlian Decoction for T2DM

Certainty assessment							№ of patients		Relative Effect MD (95% CI)	Certainty
№ of studies	Study design	Risk of bias	Inconsistency	Indirectness	Imprecision	Other considerations	CHM	Control		
**FPG**										
CHM vs. Control										
5	randomised trials	serious	serious	not serious	not serious	Unclear*	231	188	MD **1.01 lower** (1.93 lower to 0.08 lower)	Low
CHM plus conventional medicine vs. control										
26	randomised trials	serious	serious	not serious	not serious	Publication bias suspected	1158	1155	MD **1.36 lower** (1.73 lower to 0.99 lower)	Low
**2hPG**										
CHM vs. Control										
4	randomised trials	serious	serious	not serious	serious	Unclear*	190	138	MD 0.78 **lower** (3.08 lower to 1.52 higher)	Very Low
CHM plus conventional medicine vs. control										
23	randomised trials	serious	serious	not serious	not serious	none	1039	1036	MD **1.22 lower** (1.53 lower to 0.92 lower)	Low

C. GRADE Evidence Profile on Banxia Baizhu Tianma Decoction for hypertension

Certainty assessment							№ of patients		Relative Effect MD (95% CI)	Certainty
№ of studies	Study design	Risk of bias	Inconsistency	Indirectness	Imprecision	Other considerations	CHM	Control		
**SBP**										
CHM vs. Control										
2	randomised trials	serious	serious	not serious	serious	Unclear*	95	85	MD **14.43 higher** (9.97 lower to 38.83 higher)	Very Low
CHM plus conventional medicine vs. control										
16	randomised trials	serious	serious	not serious	not serious	Publication bias suspected	759	721	MD **11.18 lower** (14.24 lower to 8.12 lower)	Low
**DBP**										
CHM vs. Control										
2	randomised trials	serious	serious	not serious	serious	Unclear*	95	85	MD **6.58 higher** (5.43 lower to 18.58 higher)	Very Low

CHM plus conventional medicine vs. control										
16	randomised trials	serious	serious	not serious	not serious	none	759	721	MD **7.35 lower** (9.61 lower to 5.10 lower)	Low

D. GRADE Evidence Profile on Jiangzhi Tongmai Capsule for dyslipidemia

Certainty assessment							№ of patients		Relative Effect MD (95% CI)	Certainty
№ of studies	Study design	Risk of bias	Inconsistency	Indirectness	Imprecision	Other considerations	CHM	Control		
**TG**										
CHM vs. Control										
1	randomised trials	serious	serious	not serious	serious	Unclear*	59	59	MD **0.41 lower** (0.83 lower to 0.01 higher)	Very Low
CHM plus conventional medicine vs. control										
4	randomised trials	serious	serious	not serious	serious	Unclear*	145	145	MD **0.66 lower** (1.23 lower to 0.09 lower)	Very Low
**LDL-C**										
CHM vs. Control										
1	randomised trials	serious	serious	not serious	serious	Unclear*	59	59	MD **0.54 lower** (0.87 lower to 0.21 lower)	Very Low
CHM plus conventional medicine vs. control										
4	randomised trials	serious	serious	not serious	serious	Unclear*	145	145	MD **0.04 lower** (0.45 lower to 0.38 higher)	Very Low

E. GRADE Evidence Profile on Berberine for dyslipidemia

Certainty assessment							№ of patients		Relative Effect MD (95% CI)	Certainty
№ of studies	Study design	Risk of bias	Inconsistency	Indirectness	Imprecision	Other considerations	CHM	Control		
**TG**										
CHM vs. Control										
4	randomised trials	serious	not serious	not serious	serious	Unclear*	135	83	MD **0.26 lower** (0.40 lower to 0.12 lower)	Low
CHM plus conventional medicine vs. control										
4	randomised trials	serious	not serious	not serious	serious	Unclear*	156	103	MD **0.20 lower** (0.23 lower to 0.17 lower)	Low
**LDL-C**										
CHM vs. Control										
4	randomised trials	serious	serious	not serious	serious	Unclear*	135	83	MD **0.12 lower** (0.73 lower to 0.50 higher)	Very Low
CHM plus conventional medicine vs. control										
4	randomised trials	serious	not serious	not serious	serious	Unclear*	156	103	MD **0.11 lower** (0.20 lower to 0.03 lower)	Low

F. GRADE Evidence Profile on Linggui Zhugan Decoction for obesity

Certainty assessment							№ of patients		Relative Effect MD (95% CI)	Certainty
№ of studies	Study design	Risk of bias	Inconsistency	Indirectness	Imprecision	Other considerations	CHM	Control		
**BMI**										

CHM vs. Control										
3	randomised trials	serious	not serious	not serious	serious	Unclear*	91	72	MD **1.77 lower** (2.72 lower to 0.82 lower)	Low

*The detection of publication bias was not applicable to the comparison as the number of RCTs for the meta-analysis was less than ten. BMI, body mass index; CHM, Chinese herbal medicine; CI: confidence interval; DBP, diastolic blood pressure; FPG, fasting plasma glucose; LDL-C, low-density lipoprotein cholesterol; MD: mean difference; SBP, Systolic blood pressure; TC, total cholesterol; T2DM, type 2 diabetes mellitus; 2 hPG, 2-hour postprandial blood glucose; TG, Triglyceride；vs., versus.
