# Supplementary material for: Efficacy and safety of Chinese herbal medicine for metabolic conditions: a systematic review and meta-analysis of randomised controlled trials
Source: Front Pharmacol. 2026 Jan 2;16:1644950. doi: 10.3389/fphar.2025.1644950 (PMC12808482; doi:10.3389/fphar.2025.1644950)
Supplement: Supplementary file 2 [file Image1.pdf]

Supplementary figures

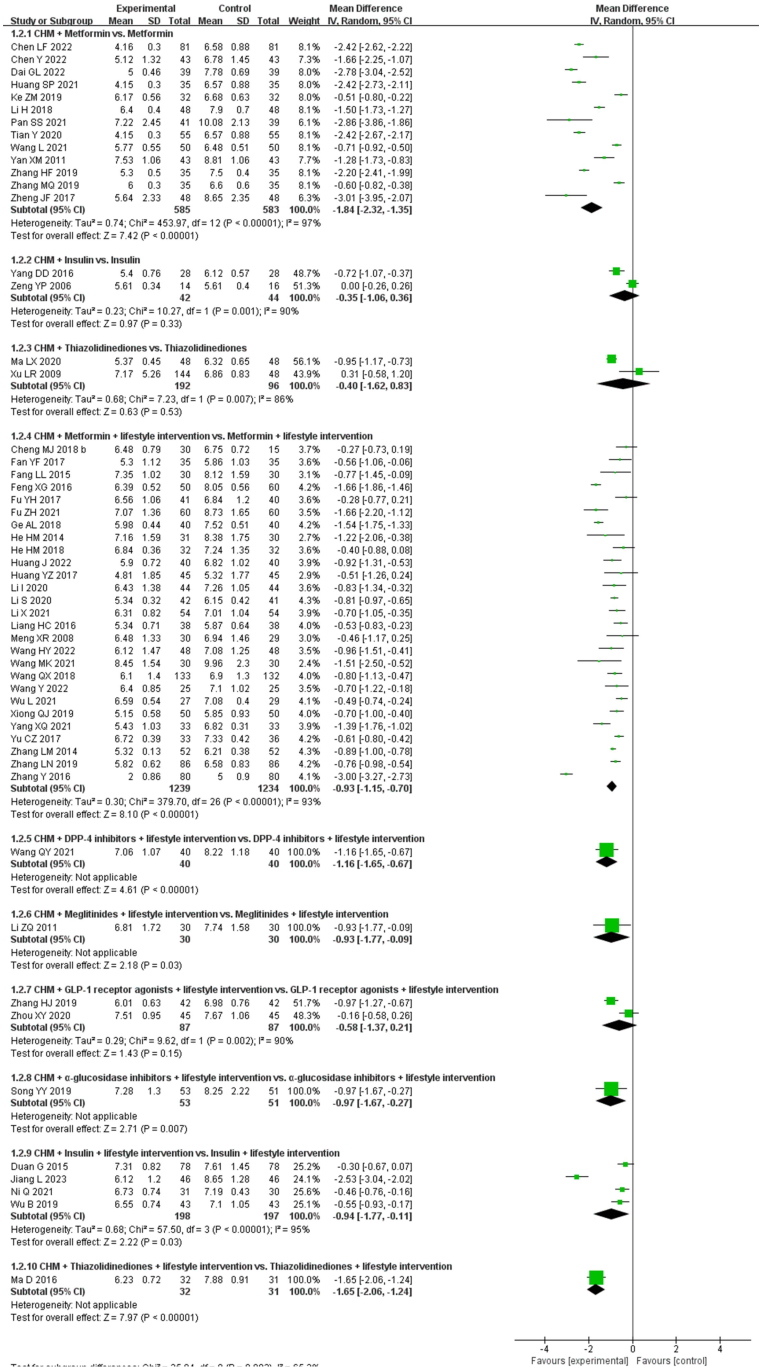

Figure S1. Meta-analysis of FPG of CHM plus conventional medicine versus control for T2DM. CHM, Chinese herbal medicine; FPG, fasting plasma glucose; T2DM, Type 2 diabetes mellitus.

Study or Subgroup	Experimental Mean	Experimental SD	Experimental Total	Control Mean	Control SD	Control Total	Weight	Mean Difference IV, Random, 95% CI
**1.4.1 CHM + Thiazolidinediones vs. Thiazolidinediones**								
Ma LX 2020	7.32	0.65	48	9.46	0.59	48	51.2%	-2.14 [-2.39, -1.89]
Xu LR 2009	9.79	1.47	144	10.06	2.02	48	48.8%	-0.27 [-0.89, 0.35]
**Subtotal (95% CI)**			**192**			**96**	**100.0%**	**-1.23 [-3.06, 0.60]**
Heterogeneity: Tau² = 1.69; Chi² = 30.13, df = 1 (P < 0.00001); I² = 97%								
Test for overall effect: Z = 1.31 (P = 0.19)								
**1.4.2 CHM + Metformin vs. Metformin**								
Chen LF 2022	7.18	0.35	81	8.73	1.16	81	9.6%	-1.55 [-1.81, -1.29]
Chen Y 2022	6.15	1.23	43	7.67	1.56	43	8.0%	-1.52 [-2.11, -0.93]
Dai GL 2022	5.85	0.66	39	6.38	0.78	39	9.4%	-0.53 [-0.85, -0.21]
Huang SP 2021	7.17	0.35	35	8.72	1.16	35	9.0%	-1.55 [-1.95, -1.15]
Ke ZM 2019	7.15	1.53	32	7.76	1.62	32	7.0%	-0.61 [-1.38, 0.16]
Li H 2018	7.2	1.4	48	10	1	48	8.6%	-2.80 [-3.29, -2.31]
Pan SS 2021	17.53	2.21	41	20.05	2.24	39	5.9%	-2.52 [-3.50, -1.54]
Tian Y 2020	7.17	0.35	55	8.72	1.16	55	9.4%	-1.55 [-1.87, -1.23]
Wang L 2021	8.94	0.79	50	10.09	0.77	50	9.5%	-1.15 [-1.46, -0.84]
Yan XM 2011	11.88	1.8	43	13.87	2.08	43	6.7%	-1.99 [-2.81, -1.17]
Zhang HF 2019	11.8	5.6	35	14.3	6.8	35	1.4%	-2.50 [-5.42, 0.42]
Zhang MQ 2019	7.6	0.5	35	8.6	0.8	35	9.4%	-1.00 [-1.31, -0.69]
Zheng JF 2017	7.56	2.55	48	10.65	2.04	48	6.1%	-3.09 [-4.01, -2.17]
**Subtotal (95% CI)**			**585**			**583**	**100.0%**	**-1.60 [-1.96, -1.23]**
Heterogeneity: Tau² = 0.34; Chi² = 96.03, df = 12 (P < 0.00001); I² = 88%								
Test for overall effect: Z = 8.59 (P < 0.00001)								
**1.4.3 CHM + Insulin vs. Insulin**								
Yang DD 2016	8.09	2.01	28	9.98	2.19	28	45.8%	-1.89 [-2.99, -0.79]
Zeng YP 2006	7.03	0.58	14	7.12	0.58	16	54.2%	-0.09 [-0.51, 0.33]
**Subtotal (95% CI)**			**42**			**44**	**100.0%**	**-0.91 [-2.67, 0.84]**
Heterogeneity: Tau² = 1.44; Chi² = 8.98, df = 1 (P = 0.003); I² = 89%								
Test for overall effect: Z = 1.02 (P = 0.31)								
**1.4.4 CHM + Metformin + lifestyle intervention vs. Metformin + lifestyle intervention**								
Cheng MJ 2018 b	8.9	1.47	30	9.52	1.62	15	3.3%	-0.62 [-1.59, 0.35]
Fang LL 2015	9.26	1.37	30	9.95	1.16	30	4.2%	-0.69 [-1.33, -0.05]
Feng XG 2016	7.35	1.23	50	9.9	1.01	60	4.8%	-2.55 [-2.98, -2.12]
Fu YH 2017	9.79	1.99	41	10.22	2.35	40	3.4%	-0.43 [-1.38, 0.52]
Fu ZH 2021	8.58	0.78	60	9.47	2.25	60	4.3%	-0.89 [-1.49, -0.29]
He HM 2014	9.44	1.64	31	10.66	2.66	30	3.0%	-1.22 [-2.33, -0.11]
He HM 2018	7.48	1.46	32	10.56	1.71	32	3.8%	-3.08 [-3.86, -2.30]
Huang J 2022	8.51	1.12	40	9.56	2.05	40	4.0%	-1.05 [-1.77, -0.33]
Huang YZ 2017	7.72	1.62	45	7.83	1.24	45	4.3%	-0.11 [-0.71, 0.49]
Li S 2020	7.24	1.26	42	8.84	1.35	41	4.4%	-1.60 [-2.16, -1.04]
Li X 2021	8.69	1.26	54	10.08	1.35	54	4.6%	-1.39 [-1.88, -0.90]
Liang HC 2016	7.2	1.3	38	8.1	1.2	38	4.4%	-0.90 [-1.46, -0.34]
Meng XR 2008	9.11	2.11	30	10.78	2.16	29	3.0%	-1.67 [-2.76, -0.58]
Wang HY 2022	6.49	1.32	48	8.23	1.27	48	4.5%	-1.74 [-2.26, -1.22]
Wang MK 2021	11.2	1.59	30	12.3	1.95	30	3.5%	-1.10 [-2.00, -0.20]
Wang QX 2018	8.1	2.5	133	10.7	2.6	132	4.3%	-2.60 [-3.21, -1.99]
Wang Y 2022	8.03	1.02	25	10.23	1.35	25	4.2%	-2.20 [-2.86, -1.54]
Wu L 2021	9.65	1.26	27	10.39	1.29	29	4.1%	-0.74 [-1.41, -0.07]
Xiong QJ 2019	6.98	1.32	50	7.99	1.55	50	4.4%	-1.01 [-1.57, -0.45]
Yang XQ 2021	8.02	0.54	33	9.32	0.58	33	5.1%	-1.30 [-1.57, -1.03]
Yu CZ 2017	7.77	0.66	33	8.33	0.79	36	4.9%	-0.56 [-0.90, -0.22]
Zhang LM 2014	6.81	0.15	52	7.73	0.68	52	5.2%	-0.92 [-1.11, -0.73]
Zhang LN 2019	8.83	4.16	86	9.84	2.16	86	3.3%	-1.01 [-2.00, -0.02]
Zhang Y 2016	7	1.32	80	7	1.52	80	4.7%	0.00 [-0.44, 0.44]
**Subtotal (95% CI)**			**1120**			**1115**	**100.0%**	**-1.22 [-1.51, -0.93]**
Heterogeneity: Tau² = 0.42; Chi² = 169.91, df = 23 (P < 0.00001); I² = 86%								
Test for overall effect: Z = 8.20 (P < 0.00001)								
**1.4.5 CHM + DPP-4 inhibitors + lifestyle intervention vs. DPP-4 inhibitors + lifestyle intervention**								
Wang QY 2021	11.48	1.21	40	12.37	1.32	40	100.0%	-0.89 [-1.44, -0.34]
**Subtotal (95% CI)**			**40**			**40**	**100.0%**	**-0.89 [-1.44, -0.34]**
Heterogeneity: Not applicable								
Test for overall effect: Z = 3.14 (P = 0.002)								
**1.4.6 CHM + Meglitinides + lifestyle intervention vs. Meglitinides + lifestyle intervention**								
Li ZQ 2011	9.26	2.27	30	10.51	2.39	30	100.0%	-1.25 [-2.43, -0.07]
**Subtotal (95% CI)**			**30**			**30**	**100.0%**	**-1.25 [-2.43, -0.07]**
Heterogeneity: Not applicable								
Test for overall effect: Z = 2.08 (P = 0.04)								
**1.4.7 CHM + GLP-1 receptor agonists + lifestyle intervention vs. GLP-1 receptor agonists + lifestyle intervention**								
Zhang HJ 2019	7.87	1.19	42	8.64	1.3	42	53.1%	-0.77 [-1.30, -0.24]
Zhou XY 2020	8.98	1.41	45	9.2	1.48	45	46.9%	-0.22 [-0.82, 0.38]
**Subtotal (95% CI)**			**87**			**87**	**100.0%**	**-0.51 [-1.05, 0.03]**
Heterogeneity: Tau² = 0.07; Chi² = 1.81, df = 1 (P = 0.18); I² = 45%								
Test for overall effect: Z = 1.87 (P = 0.06)								
**1.4.8 CHM + α-glucosidase inhibitors + lifestyle intervention vs. α-glucosidase inhibitors + lifestyle intervention**								
Song YY 2019	9.98	2.57	53	11.28	2.95	51	100.0%	-1.30 [-2.36, -0.24]
**Subtotal (95% CI)**			**53**			**51**	**100.0%**	**-1.30 [-2.36, -0.24]**
Heterogeneity: Not applicable								
Test for overall effect: Z = 2.39 (P = 0.02)								
**1.4.9 CHM + Insulin + lifestyle intervention vs. Insulin + lifestyle intervention**								
Jiang L 2023	6.76	1.12	46	9.31	2.05	46	49.3%	-2.55 [-3.23, -1.87]
Ni Q 2021	8.76	0.81	31	8.9	0.71	30	50.7%	-0.14 [-0.52, 0.24]
**Subtotal (95% CI)**			**77**			**76**	**100.0%**	**-1.33 [-3.69, 1.03]**
Heterogeneity: Tau² = 2.83; Chi² = 37.09, df = 1 (P < 0.00001); I² = 97%								
Test for overall effect: Z = 1.10 (P = 0.27)								
**1.4.10 CHM + Thiazolidinediones + lifestyle intervention vs. Thiazolidinediones + lifestyle intervention**								
Ma D 2016	9.07	1.68	32	9.88	2.03	31	100.0%	-0.81 [-1.73, 0.11]
**Subtotal (95% CI)**			**32**			**31**	**100.0%**	**-0.81 [-1.73, 0.11]**
Heterogeneity: Not applicable								
Test for overall effect: Z = 1.72 (P = 0.08)								
Test for subgroup differences: Chi² = 12.88, df = 9 (P = 0.17), I² = 30.1%								

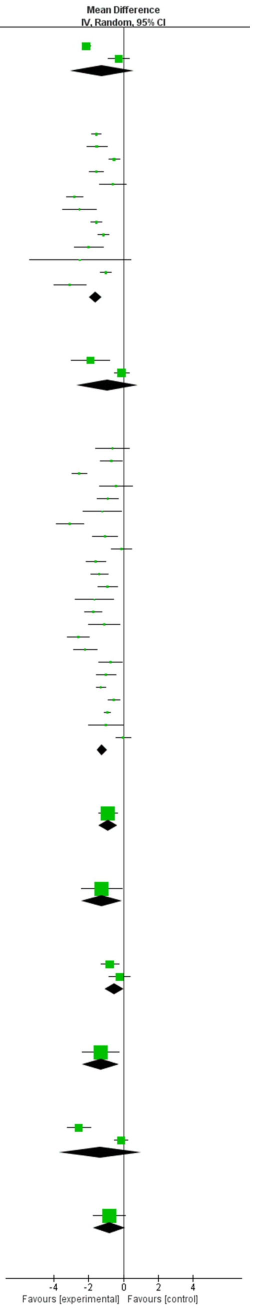

Figure S2. Meta-analysis of 2hPG of CHM plus conventional medicine versus control for T2DM. 2hPG, 2-hour postprandial blood glucose; CHM, Chinese herbal medicine; T2DM, Type 2 diabetes mellitus.

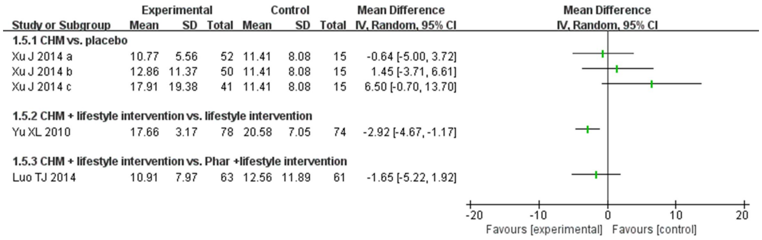

Figure S3. Meta-analysis of FINS of CHM versus control for T2DM. CHM, Chinese herbal medicine; FINS, fasting insulin; T2DM, Type 2 diabetes mellitus.

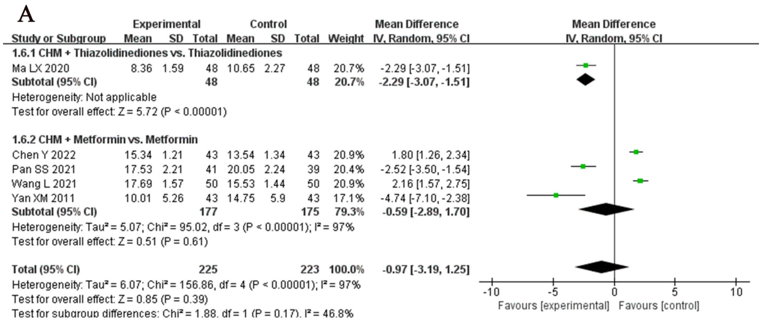

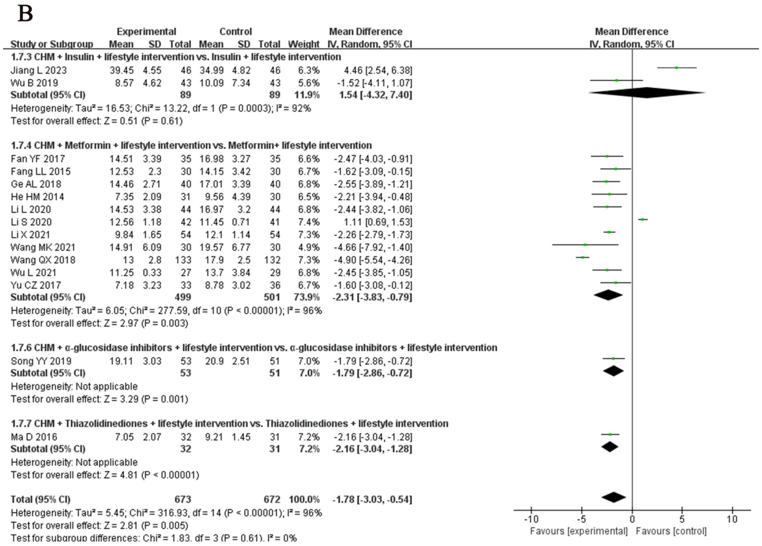

Figure S4. Meta-analysis of FINS of CHM plus conventional medicine versus control for T2DM. A.CHM plus pharmacotherapy versus pharmacotherapy; B. CHM plus pharmacotherapy plus lifestyle intervention vs pharmacotherapy plus lifestyle intervention. CHM, Chinese herbal medicine; FINS, fasting insulin; T2DM, Type 2 diabetes mellitus.

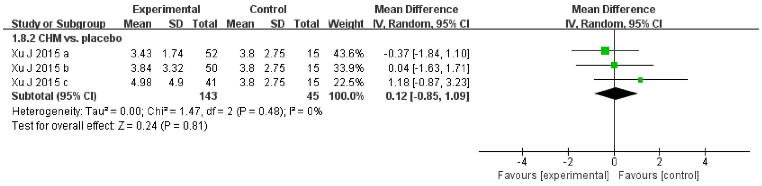

Figure S5. Meta-analysis of HOMA-IR of CHM versus control for T2DM. CHM: Chinese herbal medicine; HOMA-IR, homeostasis model assessment of insulin resistance; T2DM: Type 2 diabetes mellitus.

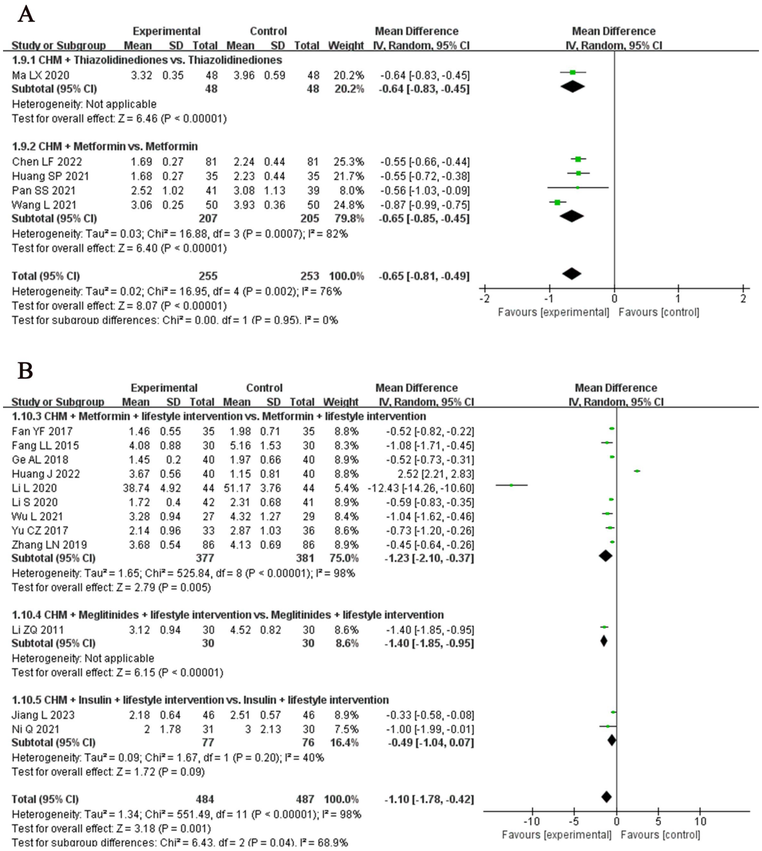

Figure S6. Meta-analysis of HOMA-IR of CHM plus conventional medicine versus control for T2DM. A.CHM plus pharmacotherapy versus pharmacotherapy; B. CHM plus pharmacotherapy plus lifestyle intervention vs pharmacotherapy plus lifestyle intervention. CHM, Chinese herbal medicine; HOMA-IR, homeostasis model assessment of insulin resistance; T2DM, Type 2 diabetes mellitus.

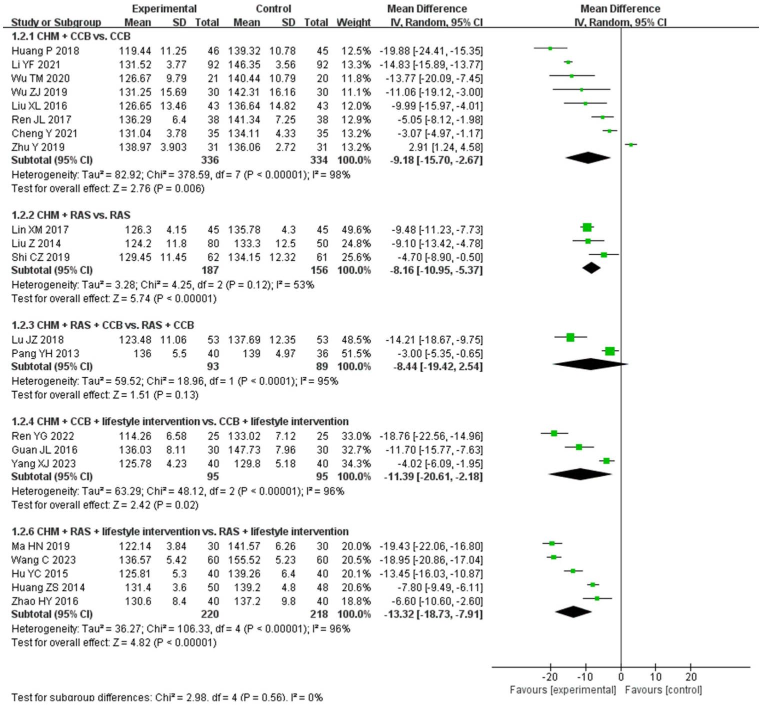

Figure S7. Meta-analysis of SBP of CHM plus conventional medicine versus control for hypertension. CCB, Calcium Channel Blockers; CHM, Chinese herbal medicine; RAS, Renin-Angiotensin System; SBP, systolic blood pressure.

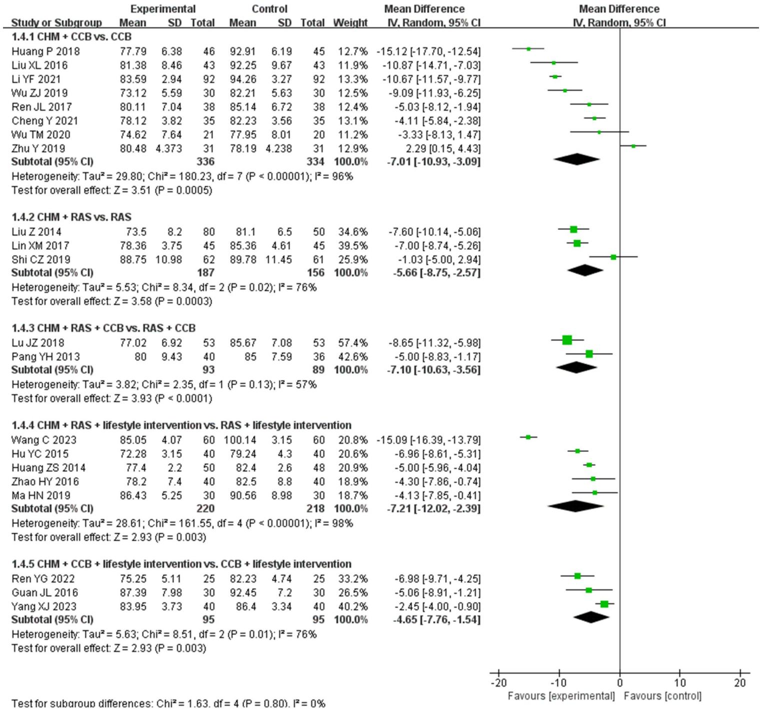

Figure S8. Meta-analysis of DBP of CHM plus conventional medicine versus control for hypertension. CCB, Calcium Channel Blockers; CHM, Chinese herbal medicine; DBP, diastolic blood Pressure; RAS, Renin-Angiotensin System.

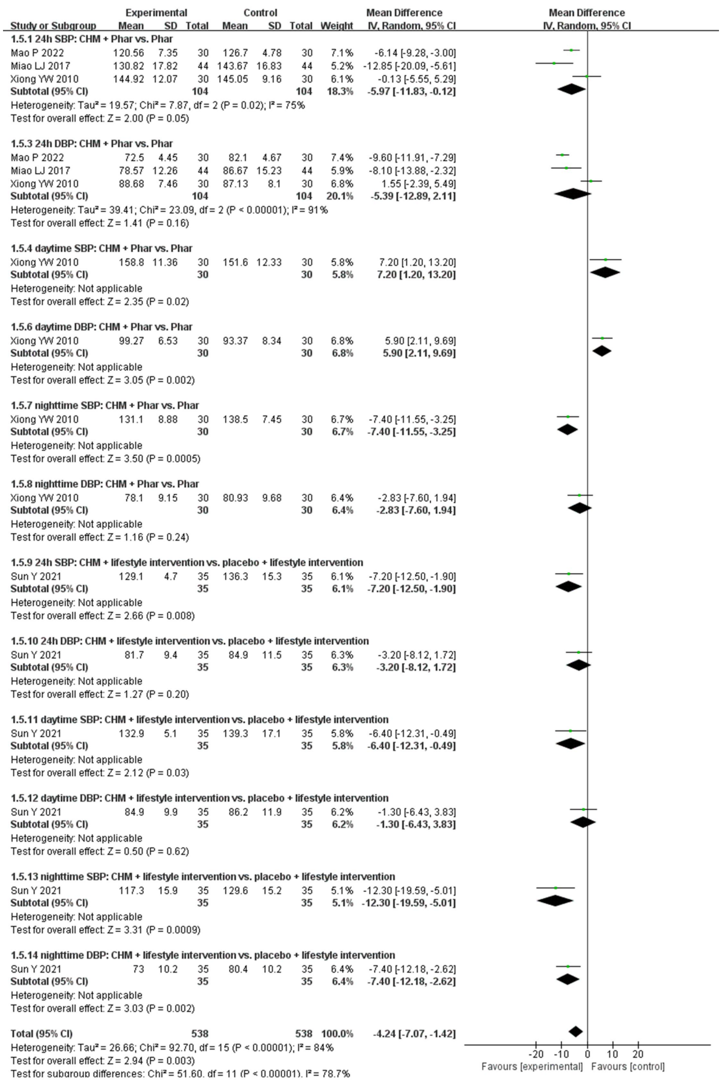

Figure S9. Meta-analysis of 24-hour ABPM for hypertension. CHM, Chinese herbal medicine; Phar, pharmacotherapy; ABPM, ambulatory blood pressure monitoring.

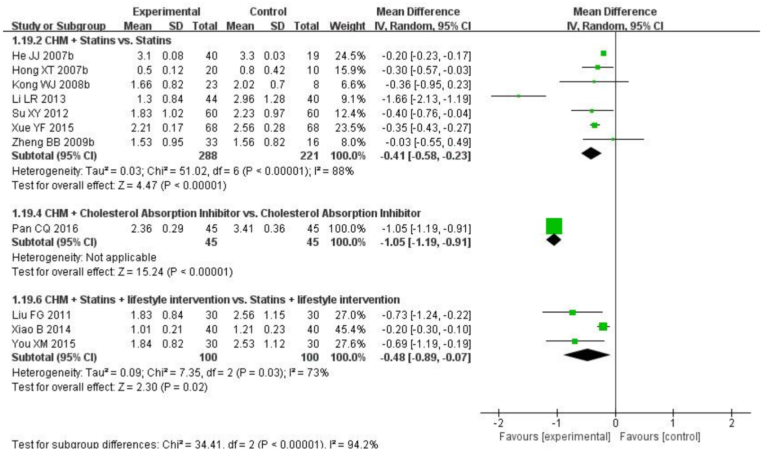

Figure S10. Meta-analysis of TG of CHM plus conventional medicine versus control for dyslipidemia. CHM, Chinese herbal medicine; TG, triglyceride.

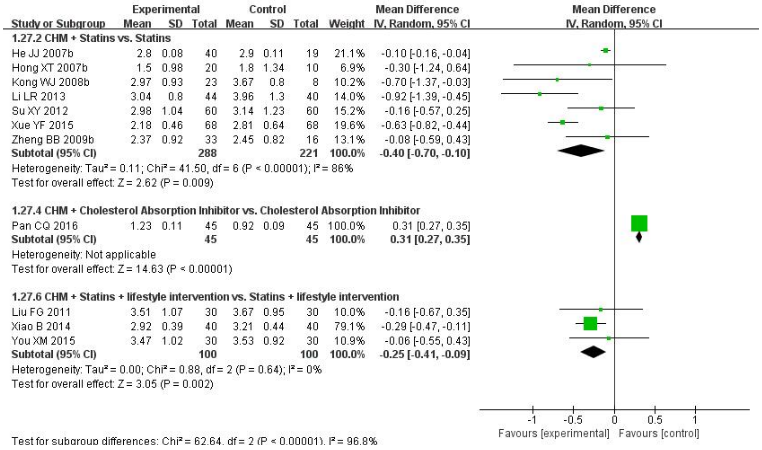

Figure S11. Meta-analysis of LDL-C of CHM plus conventional medicine versus control for dyslipidemia. CHM, Chinese herbal medicine; LDL-C, low-density lipoprotein cholesterol.

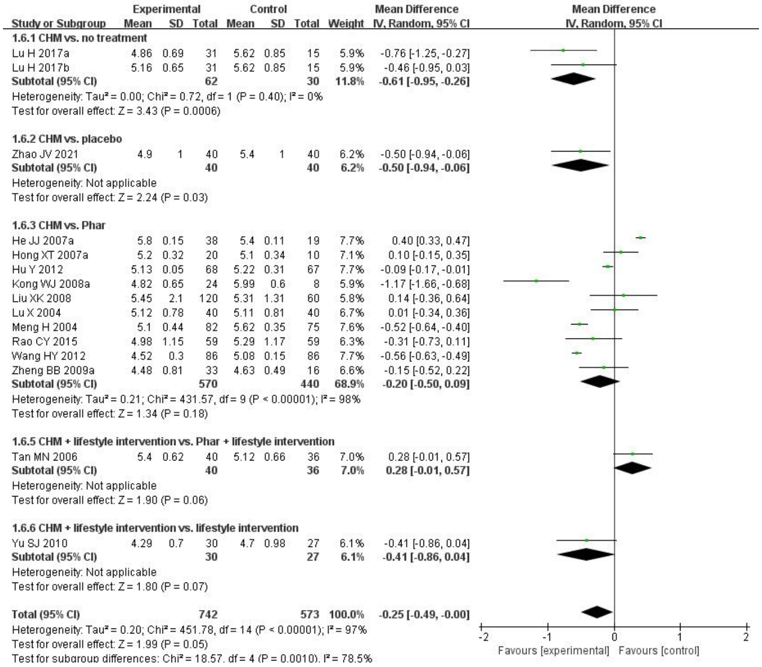

Figure S12. Meta-analysis of TC of CHM versus control for dyslipidemia. CHM, Chinese herbal medicine; Phar, pharmacotherapy; TC, total cholesterol.

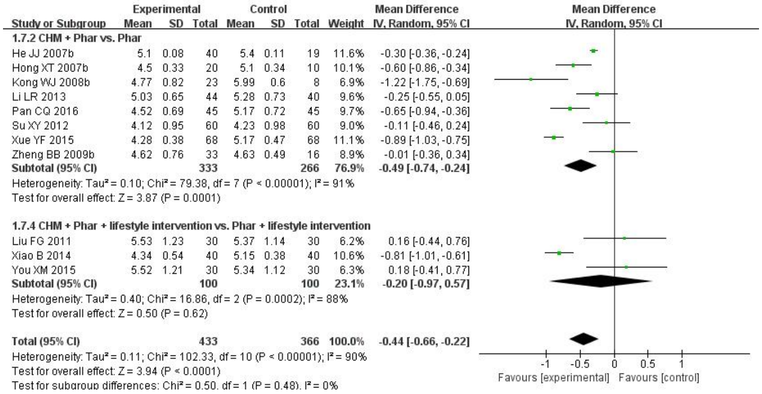

Figure S13. Meta-analysis of TC of CHM plus conventional medicine versus control for dyslipidemia. CHM, Chinese herbal medicine; TC, total cholesterol.

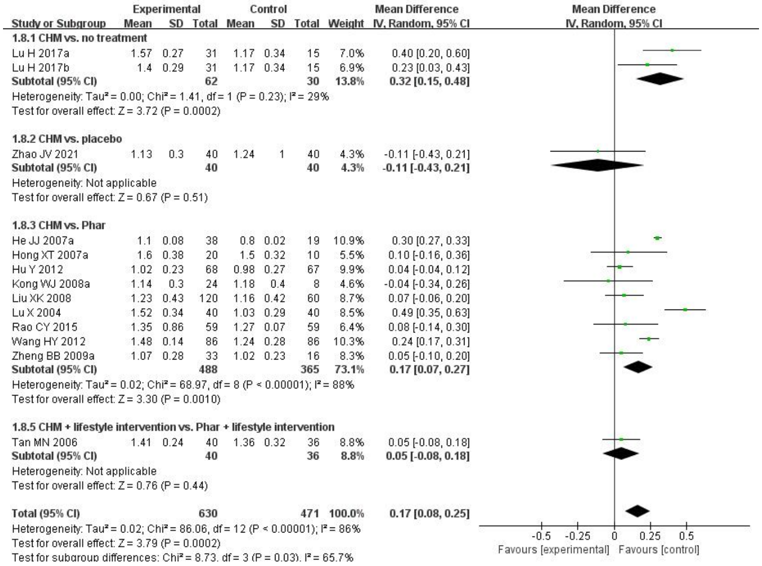

Figure S14. Meta-analysis of HDL-C of CHM versus control for dyslipidemia. CHM, Chinese herbal medicine; HDL-C, high density lipoprotein cholesterol; Phar, pharmacotherapy.

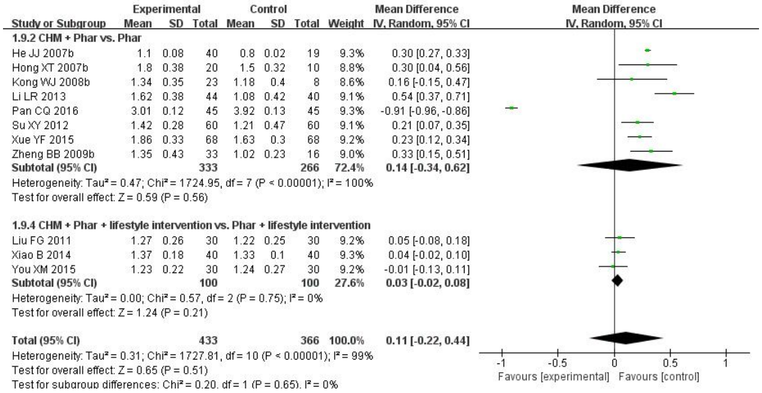

Figure S15. Meta-analysis of HDL-C of CHM plus conventional medicine versus control for dyslipidemia. CHM, Chinese herbal medicine; HDL-C, high density lipoprotein cholesterol.

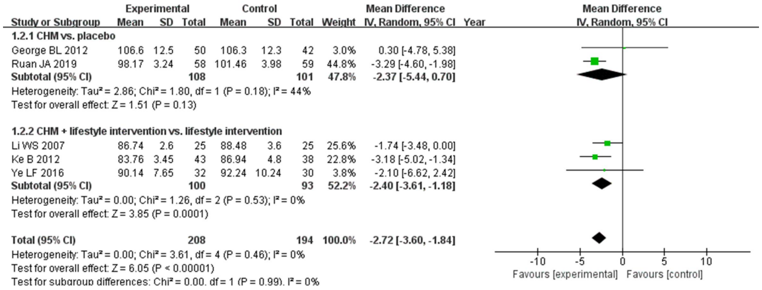

Figure S16. Meta-analysis of WC of CHM versus control for obesity. CHM, Chinese herbal medicine; WC, waist circumference.

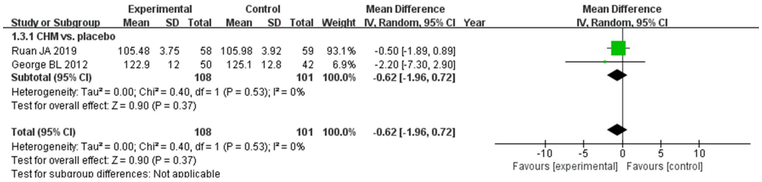

Figure S17. Meta-analysis of HC of CHM versus control for obesity. CHM, Chinese herbal medicine; HC, hip circumference.

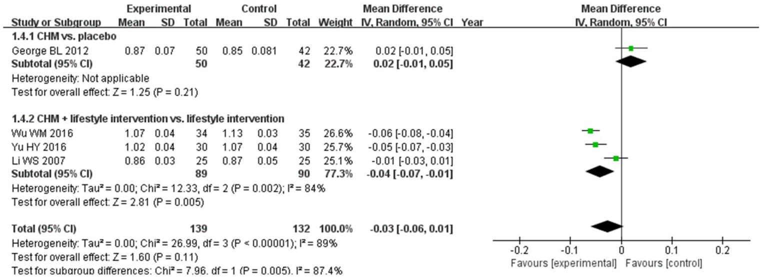

Figure S18. Meta-analysis of WHR of CHM versus control for obesity. CHM, Chinese herbal medicine; WHR, waist-to-hip ratio.
